# Assessing causal relationship between circulating cytokines and age-related neurodegenerative diseases: a bidirectional two-sample Mendelian randomization analysis

**DOI:** 10.1038/s41598-023-39520-9

**Published:** 2023-07-29

**Authors:** Zihan Yin, Jiao Chen, Manze Xia, Xinyue Zhang, Yaqin Li, Zhenghong Chen, Qiongnan Bao, Wanqi Zhong, Jin Yao, Kexin Wu, Ling Zhao, Fanrong Liang

**Affiliations:** 1 School of Acu-Mox and Tuina, Chengdu University of Traditional Chinese Medicine, 37 Shierqiao Road, Chengdu, 610075 Sichuan China; 2 Acupuncture Clinical Research Center of Sichuan Province, Chengdu, China

**Keywords:** Neuroscience, Neurogenesis, Neurological disorders

## Abstract

Numerous studies have reported that circulating cytokines (CCs) are linked to age-related neurodegenerative diseases (ANDDs); however, there is a lack of systematic investigation for the causal association. A two-sample bidirectional Mendelian Randomisation (MR) method was utilized to evaluate the causal effect. We applied genetic variants correlated with concentrations of CCs from a genome-wide association study meta-analysis (n = 8293) as instrumental variables. Summary data of three major ANDDs [Alzheimer’s disease (AD), Parkinson’s disease (PD), and Amyotrophic lateral sclerosis (ALS)] were identified from the IEU OpenGWAS platform (n = 627, 266). Inverse-variance weighted method is the main approach to analyse causal effect, and MR results are verified by several sensitivity and pleiotropy analyses. In directional MR, it suggested that several CCs were nominally correlated with the risk of ANDDs, with a causal odds ratio (OR) of Interleukin (IL)-5 of 0.909 for AD; OR of IL-2 of 1.169 for PD; and OR of Beta nerve growth factor of 1.142 for ALS). In reverse MR, there were some suggestively causal effects of ANDDs on CCs (AD on increased Basic fibroblast growth factor and IL-12 and decreased Stem cell growth factor beta; PD on decreased Monokine induced by interferon-gamma; ALS on decreased Basic fibroblast growth factor and IL-17). The findings were stable across sensitivity and pleiotropy analyses. However, after Bonferroni correction, there is no statistically significant association between CCs and ANDDs. Through the genetic epidemiological approach, our study assessed the role and presented possible causal associations between CCs and ANDDs. Further studies are warranted to verify the causal associations.

## Introduction

Age-related neurodegenerative diseases, including Alzheimer's disease (AD), Parkinson's disease (PD), and amyotrophic lateral sclerosis (ALS), are the leading causes of morbidity, disability, and mortality and impose a considerable social and economic burden worldwide^1,2^. AD affects approximately 35 million people globally, and it is estimated to triple by 2060^3,4^; PD affects approximately 1% of individuals aged over 65 years, and the incidence is predicted to quadruple by 2040^5,6^; and ALS affects approximately 4.42 per 100,000 individuals worldwide, and a rise in prevalence and incidence is associated with advancing age^7,8^. As refractory progressive nervous system diseases with various clinical features, they are underlaid by progressive loss of neuronal populations that are susceptible to damage^9–13^. Numerous studies^14–16^ have been proposed to explain the functional loss of neurons in these age-related neurodegenerative diseases, but the pathophysiologies have not been thoroughly discovered. Owing to the undiscovered pathogenesis, no curable treatments have been developed yet. Thus, there is an imperious need to seek the cause of neuron degeneration.

Nevertheless, the cause is multifactorial, and many crucial components are involved in this process^17^. Currently, the immune system has been considered a key player linked to the development of age-related neurodegeneration and specifically illuminated for AD, PD, and ALS. Meanwhile, emerging evidence supports a potential role for immunotherapy in the management of disease progression despite the precise mechanisms through which the immune system influences neuron degeneration remaining unclear^18^. Numerous studies^19–21^ have suggested that circulating cytokines, such as inflammatory-related cytokines, growth factors, and chemokines, as signalling molecules within the immune system, are associated with neuronal degeneration; for instance, overproduction/overusing of circulating pro-inflammatory cytokines [such as Interleukin-1β (IL-1β), IL-6, and Tumour necrosis factor-α]^22^, anti-inflammatory cytokines (such as IL-1RA, IL-10, and IL-12)^23^, and several growth factors [such as nerve growth factors and stem cell growth factor (SCGF)]^24^ could lead a pathophysiology progression. Meanwhile, it could modulate the immune response and may be regarded as a target site for these age-related neurodegenerative diseases prevention and treatments^19–21^. However, the associations between circulating cytokines and age-related neurodegenerative diseases were not explored in depth. Hence, understanding the precise role of circulating cytokines and the risk for age-related neurodegenerative diseases may be beneficial in developing potential prevention, prediction, and treatment targets.

Mendelian Randomization (MR)^25^, an increasingly widely applied genetic epidemiological tool for a stable and credible deduction of causal relationships, incorporates strong exposure-related genetic instrumental variations (IVs) to assess the causal associations between exposures (e.g., circulating cytokines) and outcomes (e.g., age-related neurodegenerative diseases) to identify inferences about causality for the outcome^26^. Therefore, this study aimed to analyse the causal associations between 41 circulating cytokines and three age-related neurodegenerative disease types, AD, PD and ALS, by conducting the bidirectional two-sample MR method.

## Materials and methods

### Study design

**Figure 1:**
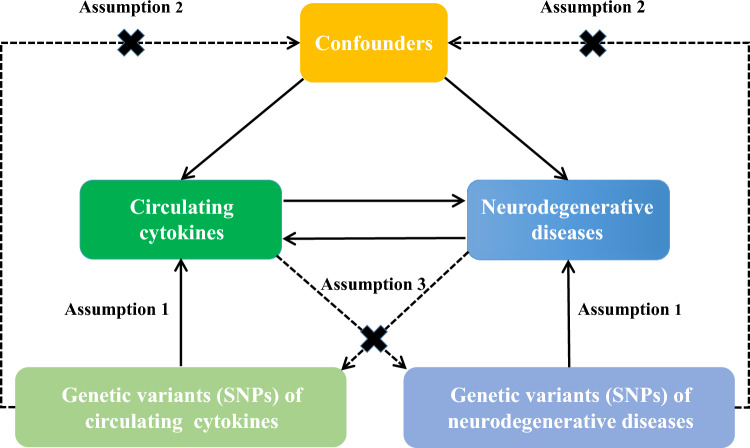
Flowchart of Mendelian randomization study revealing causality between circulating cytokines and the risk of age-related neurodegenerative diseases.

In this study, a bidirectional two-sample MR method was implemented to assess the causal effects between concentrations of circulating cytokines and age-related neurodegenerative diseases (AD, PD, and ALS) and improve informing according to Strengthening the Reporting of Observational Studies in Epidemiology Using Mendelian Randomisation (STROBE-MR)^27,28^. The MR design flow chart shown in Fig. 1. To explore the causal effects between circulating cytokines and age-related neurodegenerative diseases, MR analysis was performed to meet the three assumptions as follows: (1) the genetic instruments are strongly correlated with the exposure; (2) the genetic instruments are independent of any potential known confounders; and (3) the genetic instruments-outcome association is mediated only by the exposures. Meanwhile, the reverse MR method was conducted to explore the potential reverse causal effects. All data were retrieved from public and available large-scale genome-wide association studies (GWASs), of which each was an original study approved by the corresponding ethics committees. Informed consent was also obtained in the original studies.

### Data sources

To minimize the bias of population, the study only selected the GWAS statistics of European ancestry. The summary statistics for concentrations of 41 circulating cytokines were selected from the largest and latest available GWAS meta-analysis^29^, which covers 8293 participants from three independent cohort studies (FINRISK 1997, FINRISK 2002, and The Cardiovascular Risk in Young Finns Study). The summary statistics of three age-related neurodegenerative diseases were obtained from GWAS meta-analyses based on the IEU OpenGWAS platform (accessed on 1 October 2022). Summary statistics for AD (ID: ieu-b-2)^30^, covering 21,982 patients and 41,944 controls, were extracted from the International Genomics of Alzheimer’s Project study; summary statistics for PD (ID: ieu-b-7)^31^, covering 33,674 patients and 449,056 controls, were derived from the International Parkinson’s Disease Genomics Consortium; summary statistics for ALS (ID: ebi-a-GCST005647)^32^, covering 20,806 patients and 59,804 controls, were referred from the International Amyotrophic Lateral Sclerosis Genomics Consortium. The details of these GWAS datasets are depicted in Table 1. The details of demographics information regarding datasets about three age-related neurodegenerative diseases in Supplementary Tables 1–3.

**Table 1:** Detailed information regarding studies and datasets used in the present study.

Variable	Abbreviation	Ancestry	Numbers of subjects	Consortium
Alzheimer’s disease	AD	European	63,926	International Genomics of Alzheimer’s Project study
Parkinson’s disease	PD	European	482,730	International Parkinson’s Disease Genomics Consortium
Amyotrophic lateral sclerosis	ALS	European	80,610	International Amyotrophic Lateral Sclerosis Genomics Consortium
Beta nerve growth factor	BNGF	European	3531	FINRISK 2002, and Young Finns Study
Cutaneous T-cell attracting (CCL27)	CTACK	European	3631	FINRISK 2002, and Young Finns Study
Eotaxin (CCL11)	EOTAXIN	European	8153	FINRISK 1997, FINRISK 2002, and Young Finns Study
Basic fibroblast growth factor	bFGF	European	7565	FINRISK 1997, FINRISK 2002, and Young Finns Study
Granulocyte colony-stimulating factor	G-CSF	European	7904	FINRISK 1997, FINRISK 2002, and Young Finns Study
Growth regulated oncogene-α (CXCL1)	GROA	European	3505	FINRISK 2002, and Young Finns Study
Hepatocyte growth factor	HGF	European	8292	FINRISK 1997, FINRISK 2002, and Young Finns Study
Interferon-gamma	IFN-G	European	7701	FINRISK 1997, FINRISK 2002, and Young Finns Study
Interleukin-10	IL-10	European	7681	FINRISK 1997, FINRISK 2002, and Young Finns Study
Interleukin-12p70	IL-12	European	8270	FINRISK 1997, FINRISK 2002, and Young Finns Study
Interleukin-13	IL-13	European	3557	FINRISK 2002, and Young Finns Study
Interleukin-16	IL-16	European	3483	FINRISK 2002, and Young Finns Study
Interleukin-17	IL-17	European	7760	FINRISK 1997, FINRISK 2002, and Young Finns Study
Interleukin-18	IL-18	European	3636	FINRISK 2002, and Young Finns Study
Interleukin-1-beta	IL-1B	European	3309	FINRISK 2002, and Young Finns Study
Interleukin-1 receptor antagonist	IL-1RA	European	3638	FINRISK 2002, and Young Finns Study
Interleukin-2	IL-2	European	3475	FINRISK 2002, and Young Finns Study
Interleukin-2 receptor, alpha subunit	IL-2RA	European	3677	FINRISK 2002, and Young Finns Study
Interleukin-4	IL-4	European	8124	FINRISK 1997, FINRISK 2002, and Young Finns Study
Interleukin-5	IL-5	European	3364	FINRISK 2002, and Young Finns Study
Interleukin-6	IL-6	European	8189	FINRISK 1997, FINRISK 2002, and Young Finns Study
Interleukin-7	IL-7	European	3409	FINRISK 2002, and Young Finns Study
Interleukin-8 (CXCL8)	IL-8	European	3526	FINRISK 2002, and Young Finns Study
Interleukin-9	IL-9	European	3634	FINRISK 2002, and Young Finns Study
Interferon gamma-induced protein 10 (CXCL10)	IP-10	European	3685	FINRISK 2002, and Young Finns Study
Monocyte chemotactic protein-1 (CCL2)	MCP-1	European	8293	FINRISK 1997, FINRISK 2002, and Young Finns Study
Monocyte specific chemokine 3 (CCL7)	MCP-3	European	843	FINRISK 2002, and Young Finns Study
Macrophage colony-stimulating factor	M-CSF	European	839	FINRISK 2002, and Young Finns Study
Macrophage migration inhibitory factor (glycosylation-inhibiting factor)	MIF	European	3494	FINRISK 2002, and Young Finns Study
Monokine induced by interferon-gamma (CXCL9)	MIG	European	3685	FINRISK 2002, and Young Finns Study
Macrophage inflammatory protein-1α (CCL3)	MIP-1A	European	3522	FINRISK 2002, and Young Finns Study
Macrophage inflammatory protein-1β (CCL4)	MIP-1B	European	8243	FINRISK 1997, FINRISK 2002, and Young Finns Study
Platelet derived growth factor BB	PDGF-BB	European	8293	FINRISK 1997, FINRISK 2002, and Young Finns Study
Regulated on Activation, Normal T Cell Expressed and Secreted (CCL5)	RANTES	European	3421	FINRISK 2002, and Young Finns Study
Stem cell factor	SCF	European	8290	FINRISK 1997, FINRISK 2002, and Young Finns Study
Stem cell growth factor beta	SCGFβ	European	3682	FINRISK 2002, and Young Finns Study
Stromal cell-derived factor-1 alpha (CXCL12)	SDF-1A	European	5998	FINRISK 1997, FINRISK 2002, and Young Finns Study
Tumor necrosis factor-alpha	TNF-A	European	3454	FINRISK 2002, and Young Finns Study
Tumor necrosis factor-beta	TNF-B	European	1559	FINRISK 2002, and Young Finns Study
TNF-related apoptosis inducing ligand	TRAIL	European	8186	FINRISK 1997, FINRISK 2002, and Young Finns Study
Vascular endothelial growth factor	VEGF	European	7118	FINRISK 1997, FINRISK 2002, and Young Finns Study

### Selection of instruments

To assure the validity of the results, the MR analysis was performed following the three steps for quality control to identify instrument variables (IVs): First, single nucleotide polymorphisms (SNPs) remarkably associated with circulating cytokines / age-related neurodegenerative diseases were identified and selected as IVs. In general, the GWAS *p-value* threshold was set at 5 × 10^−8^. However, in order to maintain the genetic variance, the number of SNPs and statistical power, in the MR, we relaxed the threshold to 5 × 10^−6^, which is commonly used in numerous MR studies^33–36^ regarding age-related neurodegenerative diseases. Next, the linkage disequilibrium in the selected IVs with R^2^ threshold of < 0.001 in the distance of ≥ 1000 kilobases was clumped and eliminated using the PLINK algorithm. Third, the *F-statistic* was estimated to ensure the strength of the genetic instruments. SNPs would be eliminated from MR analysis if *F-statistics* < 10^37^. Finally, for circulating cytokine instruments, a total of 354 SNPs were identified; for age-related neurodegenerative diseases, 108 SNPs were included. Detailed summary statistics of these included SNPs are shown in Supplementary Tables 1–6.

### Statistical analysis

The inverse-variance weighted (IVW) method^38^ was considered the primary analysis with the random-effects model to evaluate the causal relationship between the circulating cytokines and age-related neurodegenerative diseases. Additional complementary MR approaches, such as MR-Egger regression and weighted median, were performed to test the robustness of the findings. In addition, Cochran’s Q test and leave-one-out analyses were applied to probe the consistency of the findings. Moreover, a funnel graph was employed to measure the horizontal pleiotropy. Moreover, MR pleiotropy residual sum and outlier (MR-PRESSO) were used to probe and correct for horizontal pleiotropic outliers. All variables were processed with a 95% confidence interval (CI). The causal effects of circulating cytokines on the risk of age-related neurodegenerative diseases were performed using odds ratios (ORs). Meanwhile, the effects of age-related neurodegenerative diseases on the circulating cytokines are displayed as beta. All statistical analyses were carried out using the *TwoSample MR* and *MR-PRESSO* packages in R version 4.1.3 software^26^. In addition, a priori statistical powers of circulating cytokines on age-related neurodegenerative diseases were calculated with type I error rate of 0.05 using https://shiny.cnsgenomics.com/mRnd/^39^, and the powers of age-related neurodegenerative diseases on circulating cytokines were calculated with significance of 0.05 level using https://sb452.shinyapps.io/power/^40^. There is suggestive evidence of potential causal effect when the *p-value* is ≤ 0.05. Moreover, statistically compelling evidence of causality was determined with a *p-value* of ≤ 0.0004 (0.05/(numbers of circulating cytokines (41) * numbers of age-related neurodegenerative diseases (3)) by multiple testing using the Bonferroni-corrected method^41,42^.

### Ethical approval

This study used the published articles or publicly available GWAS summary data. We did not collect additional raw data, and therefore approval from medical ethical committee is not required. Each study included has been approved by their institutional ethics review committees.

## Results

### Causal effect of genetically predicted circulating cytokines on age-related neurodegenerative diseases

**Figure 2:**
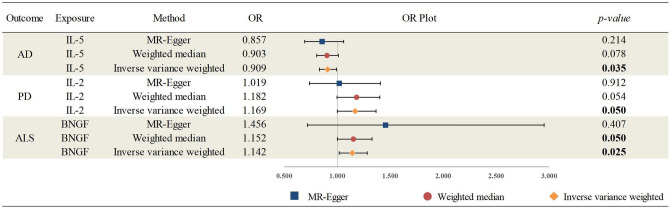
Associations between genetically predicted circulating cytokines on the risk of age-related neurodegenerative diseases; AD, Alzheimer’s disease; PD, Parkinson’s disease; ALS, Amyotrophic lateral sclerosis; IL-5, Interleukin-5; IL-2, Interleukin-2; BNGF, Beta nerve growth factor; OR, odds ratio.

MR analysis was conducted to investigate the potential causal effects of circulating cytokines on age-related neurodegenerative diseases. Based on the Bonferroni-corrected threshold, there was no statistically significant causal effect of circulating cytokines on age-related neurodegenerative diseases (all *p-values* > 0.0004). Nevertheless, the results indicated that several circulating cytokines were nominally correlated with age-related neurodegenerative diseases. Using the IVW method, the genetically predicted IL-5 was associated with a lower risk of AD (OR, 0.909; 95% CI 0.832–0.993; *p-value* = 0.035); IL-2 was associated with a higher risk of PD (OR, 1.169, 95% CI, 1.000–1.368; *p-value* = 0.05); and beta nerve growth factor (BNGF) was associated with a higher risk of ALS (OR, 1.142, 95% CI 1.017–1.283; *p-value* = 0.025). Meanwhile, there is suggestive evidence of circulating BNGF levels on ALS risk, as observed by the weighted median method. Detailed results are shown in Fig. 2 and Supplementary Materials (Table 7).

### Causal effect of genetically predicted age-related neurodegenerative diseases on circulating cytokines

**Figure 3:**
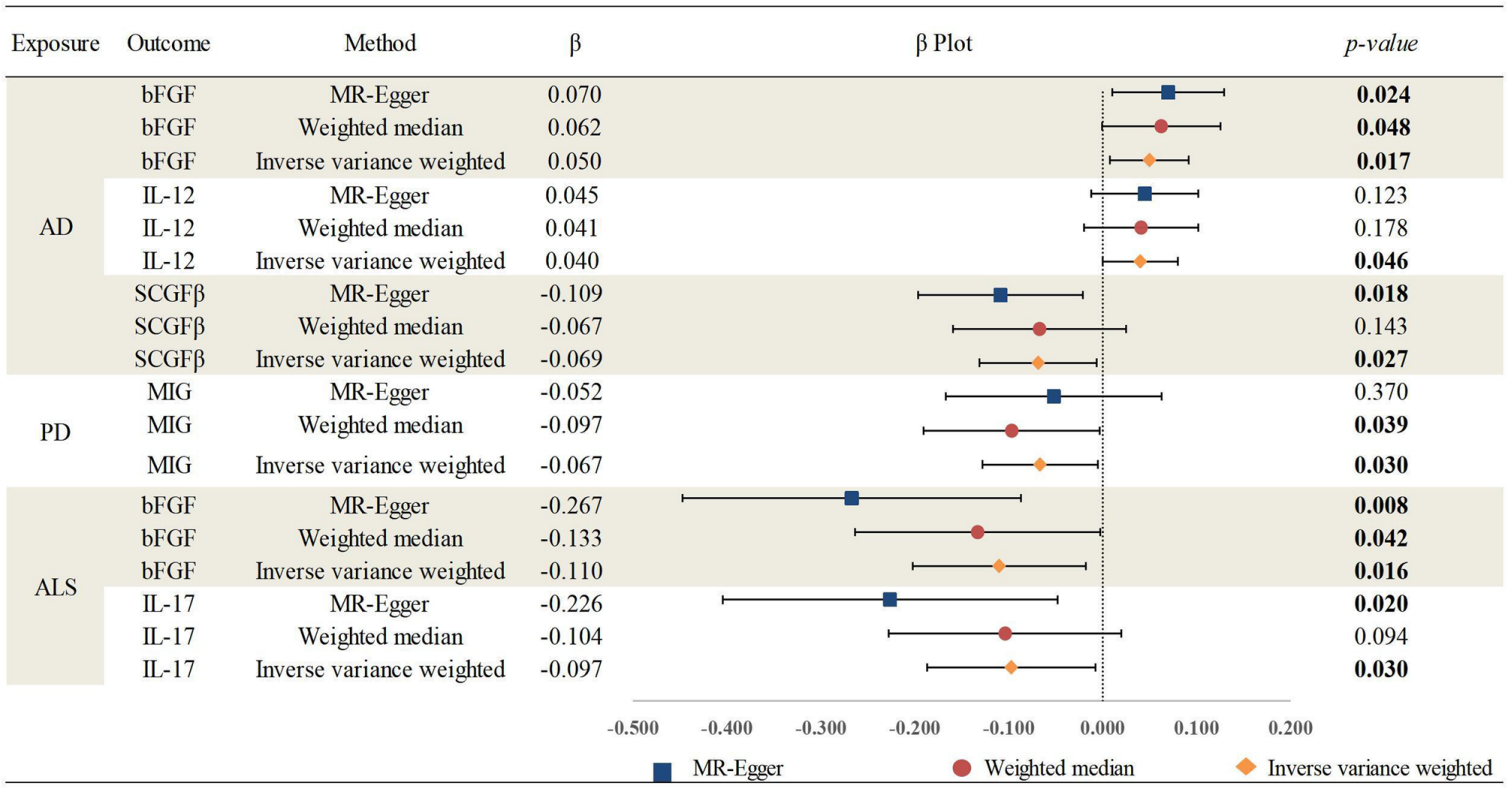
Associations between genetically predicted age-related neurodegenerative diseases on circulating cytokines; AD, Alzheimer’s disease; PD, Parkinson’s disease; ALS, Amyotrophic lateral sclerosis; bFGF, Basic fibroblast growth factor; IL-12, Interleukin-12; SCGFβ, Stem cell growth factor beta; MIG, Monokine induced by interferon-gamma; IL-17, Interleukin-17.

Possible causal effects of age-related neurodegenerative diseases on circulating cytokines were analysed using the reverse MR method. The reverse MR method revealed that age-related neurodegenerative diseases have no significant causal effect on circulating cytokines. Nevertheless, using the IVW method, genetically predicted AD demonstrated a nominally causal effect on basic fibroblast growth factor (bFGF) (β, 0.05; 95% CI 0.021–0.079; *p-value* = 0.017), in line with findings using the weighted median method and MR-Egger method; and genetically predicted AD demonstrated a nominally causal effect on IL-12 (β = 0.040; 95% CI 0.020 to 0.060; *p-value* = 0.046). Genetically predicted AD demonstrated a nominally causal effect on SCGFβ (β, − 0.069; 95% CI − 0.100 to − 0.038; *p-value* = 0.027), in line with the finding using the MR-Egger method; and genetically predicted AD demonstrated a nominally causal effect on IL-12 (β, 0.040; 95% CI 0.020–0.060; *p-value* = 0.046); genetically predicted PD showed a potential causal effect on Monokine induced by interferon-gamma (MIG: β, − 0.067; 95% CI − 0.098 to − 0.036; *p-value* = 0.03), in line with the finding using the weighted median method; genetically predicted ALS showed a potential causal effect on bFGF (β, − 0.110; 95% CI − 0.156 to − 0.064; *p-value* = 0.016), in line with findings using the weighted median method and MR-Egger method; and IL-17 (β, − 0.097; 95% CI − 0.142 to − 0.052; *p-value* = 0.03), in line with finding using the MR-Egger method. The detailed results are illustrated in Fig. 3 and Supplementary Materials (Table 8).

### Sensitivity and pleiotropy analysis

To measure the robustness of these findings, the following sensitivity and pleiotropy analyses were carried out and shown in Tables 2 and 3. For the heterogeneity analysis, little evidence was found using Cochran’s Q test and the leave-one-out method. For the pleiotropy analysis, the funnel graph showed no evidence to hold up directional pleiotropy. Meanwhile, the MR-PRESSO analysis did not show that there was pleiotropy (all *p-values* > 0.05). Detailed results are shown in Tables 2 and 3 and Supplementary Materials (Tables 7–8, eFigures 1–246).

**Table 2:** Genetically predicted circulating cytokines on the risk of age-related neurodegenerative diseases.

Exposure	Outcome	Method	OR (95% CI)	Cochran Q test		MR-Egger		MR-PRESSO
				Q	*P* value	Intercept	*P* value	*P* value
IL-5	AD	Inverse variance weighted	0.909 (0.832, 0.993)	4.443	0.617	0.012	0.577	0.339
IL-2	PD	Inverse variance weighted	1.169 (1.000, 1.368)	13.285	0.065	0.026	0.376	0.124
BNGF	ALS	Inverse variance weighted	1.142 (1.017, 1.283)	3.256	0.999	− 0.035	0.194	0.892

*AD* Alzheimer’s disease, *PD* Parkinson’s disease, *ALS* amyotrophic lateral sclerosis, *IL-5* Interleukin-5, *IL-2* Interleukin-2, *BNGF* beta nerve growth factor, *OR* odds ratio.

**Table 3:** Genetically predicted age-related neurodegenerative diseases on circulating cytokines.

Exposure	Outcome	Method	β	SE	Cochran Q test		MR-Egger		MR-PRESSO
					Q	*P* value	Intercept	*P* value	*P* value
AD	bFGF	Inverse variance weighted	0.050	0.021	31.980	0.703	− 0.005	0.348	0.766
AD	IL-12	Inverse variance weighted	0.040	0.020	35.536	0.538	− 0.001	0.812	0.618
AD	SCGFβ	Inverse variance weighted	− 0.069	0.031	39.083	0.333	0.010	0.203	0.195
PD	MIG	Inverse variance weighted	− 0.067	0.031	36.002	0.607	− 0.002	0.765	0.490
ALS	bFGF	Inverse variance weighted	− 0.110	0.046	21.441	0.372	0.021	0.059	0.758
ALS	IL-17	Inverse variance weighted	− 0.097	0.045	20.757	0.412	0.017	0.113	0.462

*AD* Alzheimer’s disease, *PD* Parkinson’s disease, *ALS* amyotrophic lateral sclerosis, *bFGF* basic fibroblast growth factor, *IL-12* Interleukin-12, *SCGFβ* stem cell growth factor beta, *MIG* monokine induced by interferon-gamma, *IL-17* Interleukin-17.

## Discussion

Numerous observational studies have illustrated the association between circulating cytokines and age-related neurodegenerative diseases; however, there are only a few MR studies in this regard. These MR studies usually focused on single age-related neurodegenerative disease^43–46^ or implemented unidirectional MR studies^47,48^. Thus, this is the first study to systematically evaluate the potential causal effects between 41 circulating cytokines and the risk of three major age-related neurodegenerative diseases using a bidirectional two-sample MR approach.

## Summary of main findings

In directional MR, findings showed that IL-5 was nominally associated with an decreased risk of AD, IL-2 was nominally associated with an increased risk of PD and BNGF was nominally associated with an increased risk of ALS. In reverse MR, the nominally causal associations of age-related neurodegenerative diseases on circulating cytokines were also detected. AD was nominally associated with increased bFGF and IL-12 and decreased SCGFβ, PD was nominally associated with decreased MIG, and ALS was nominally associated with decreased bFGF and IL-17. The results were stable in sensitivity and pleiotropy analyses.

### The associations between age-related neurodegenerative diseases and circulating cytokines

As for age-related neurodegenerative diseases, an impaired immune response was regarded as a relevant pathological factor^49^. Interleukins, as immune circulating cytokines, play a vital role in neurodegeneration.

#### The association between AD and IL-5

IL-5, a neuroprotective cytokine^50^, is produced by Th2 cells and ILC2s^51^. An observational study illustrated that AD brains showing IL-5 changes were associated with the severity of pathology^52^. Moreover, IL-5 has been shown to promote neurogenesis, reduce neuroinflammation, and protect neurons from Aβ-induced cell death in aged mice^53–55^. However, few studies have explored the effects of IL-5 as a therapeutic target in AD^56^. In our study, IL-5 was associated with a decreased risk of AD and was identified to have a nominally causal effect on AD, which is comparable to previous research and suggested it as a potential therapeutic target for AD.

#### The association between AD and IL-12

IL-12, a heterodimeric pro-inflammatory cytokine, is produced by activated monocytes, glial cells, and macrophages^57^. Evidence showed that IL-12 might be associated with AD and cognitive ageing^58^. Several observational studies^49,58,59^ and a meta-analysis^60^ found that the level of IL-12 in cerebrospinal fluid/serum was elevated in AD. Meanwhile, in a neuroimaging study, IL-12 was found to be correlated with default mode network functional connectivity of the brain^61^. In the present study, AD was nominally associated with increased IL-12, which was similar to the above studies.

#### The association between PD and IL-2

IL-2, an immunoregulatory cytokine, is produced by CD4 + helper T cells^62^. IL-2 has been considered a regulator of brain neuronal function in PD^63^. Several studies^64–66^ found that the level of IL-2 in blood was elevated in PD. In addition, it is a modulator of dopamine activity in the brain of PD^67^. In our study, IL-2 was found to be nominally associated with an increased risk of PD.

#### The association between ALS and IL-17

IL-17, a pro-inflammatory cytokine, is produced by T helper 17 cells, CD8 + T cells, innate lymphoid cells, and the like^68^. Numerous studies^69–72^ found that the level of IL-17 in cerebrospinal fluid/blood was significantly increased in ALS. In the present study, ALS was nominally associated with decreased IL-17. Thus, the level of IL-17 could act as a potential marker in ALS.

### The associations between age-related neurodegenerative diseases and circulating growth factors

Meanwhile, growth factors, chemokines and the like, as circulating cytokines, are also a crucial part of regulation of the immune system.

#### The association between ALS and BNGF

BNGF is a necessary growth factor for the survival and maintenance of neurons^73^. The abnormal level of nerve growth factor was considered a possible cause of ALS^74^. An observational study showed that the expression of plasma BNGF was associated with disease duration^75^. Moreover, a clinical study found that NGF plus riluzole treatment is a possible treatment^76^. In addition, a study found that NGF could induce the death of motor neurons^77^. In our study, BNGF was found to be associated with an increased risk of ALS and may be a potential therapeutic target for ALS.

#### The association between AD and SCGFβ

SCGFβ, a secreted sulfated glycoprotein, is produced by primitive haematopoietic progenitor cells^78^. Current evidence suggests that SCGFβ is associated with amyloid deposition in AD^79^. In addition, SCGFβ was considered a biomarker in the diagnosis and prognosis^80^. In the present study, AD was nominally associated with decreased SCGFβ, which was in accordance with the above studies.

#### The association between AD and bFGF

bFGF, a heparin-binding growth factor, is produced by bone marrow stromal cells^81^. It is characterized by neuroprotective and neurite growth activity^82^. An observational study^83^ found that the level of bFGF was increased in the brains of AD patients. Moreover, it is associated with neurotic plaques and neurofibrillary tangles^83,84^. In the present study, AD was nominally associated with increased bFGF, which was in accordance with the above studies.

#### The association between ALS and bFGF

Meanwhile, the level of these circulating cytokines was suggested as a potential biomarker. In addition, bFGF was also correlated with ALS. Some observational studies^85,86^ found that the level of bFGF was changed in the cerebrospinal fluid/blood of ALS patients. Meanwhile, a cross-sectional study^87^ illustrated that bFGF protein levels had a significant negative correlation with ALS function. In the current study, ALS was found to be nominally associated with decreased bFGF. Therefore, the level of bFGF was considered a helpful biomarker that could predict disease progression in ALS.

### The associations between age-related neurodegenerative diseases and circulating chemokine

MIG, a CXC chemokine, is positive for activated T cells^88^. Only one study^89^ found that the level of MIG in the substantia nigra was significantly changed in PD. In the present study, PD was nominally associated with decreased MIG. Thus, the level of MIG may be a potential marker in PD.

### Strengths and weaknesses

There are some strengths in the present study. First, for the MR study, the utilised statistical data were accessed from relatively up-to-date largest GWASs, which could improve the stability and accuracy of effect estimates. Second, the bidirectional MR design is aimed at reducing confounding by potential influencing elements and avoiding any reverse causality. Third, the three major age-related neurodegenerative diseases and 41 circulating cytokines were presented in the current study, which made it the most comprehensive MR study of age-related neurodegenerative diseases and circulating cytokines. Finally, discovering potential causality may influence public health policies about the diagnosis, prevention, prediction, and potential medical targets for age-related neurodegenerative diseases. The GWAS statistic included all with European ancestry, minimising the probability of bias by region and increasing the credibility and rationality of MR assumptions.

Despite the advantages of the MR design, this study has several limitations. First, we used the GWAS summary statistics in the present study with European ancestry to reduce the population bias, which may be a barrier in the application of these findings to other ethnicities. Second, to support adequate statistical power in MR, we relaxed the *p-value* threshold, which means the variance ratio introduced by the correlations between exposures and IVs might be relatively small. Even though *F-statistics* showed that weak IVs do not exist, data from more studies with large and universal samples could supply a more credible estimation of genetic impacts on exposure. In addition, the GWAS method is a significant contributor to the genetic risk factor, however, the detail was not given for the original dataset. Next, the statistical power may be deficient of circulating cytokines with a limited sample size, and therefore the MR may have overlooked potential weak associations. Finally, all *p-values* ranged as nominal levels (0.008 to 0.05), although the findings failed validation in the clinical and basic research. Thus, the potential causal associations should be interpreted cautiously and still need to be investigated for potential mechanisms.

## Conclusion

This MR research thoroughly examines, supports, and provides new findings regarding the potential causal relationship evidence between circulating cytokines and age-related neurodegenerative diseases. Nevertheless, there is no statistically compelling evidence regarding causal associations between them. Further studies are supposed to ensure the causal associations.

## Supplementary Information

Supplementary Information.

## Data Availability

All data used in the study were obtained from published articles or publicly available GWAS platform, and all data can be obtained for free.
