# Supplementary material for: Assessing causal relationship between circulating cytokines and age-related neurodegenerative diseases: a bidirectional two-sample Mendelian randomization analysis
Source: Sci Rep. 2023 Jul 29;13:12325. doi: 10.1038/s41598-023-39520-9 (PMC10387057; doi:10.1038/s41598-023-39520-9)
Supplement: Supplementary file 1 — Supplementary Information. [file 41598_2023_39520_MOESM1_ESM.pdf]

Supplementary Materials

Assessing Causal Relationship Between Circulating Cytokines and Age-Related Neurodegenerative Diseases: A Bidirectional Two-Sample Mendelian Randomization Analysis

CONTENTS

Supplementary Materials 1

Supplementary Tables 1

Supplementary Table 1. Detailed information regarding datasets about AD demographics. 1

Supplementary Table 2. Detailed information regarding datasets about PD demographics. 1

Supplementary Table 3. Detailed information regarding datasets about ALS demographics. 1

Supplementary Table 4. Detailed information for risk of AD-associated SNPs with circulating cytokines. 1

Supplementary Table 5. Detailed information for risk of PD-associated SNPs with circulating cytokines. 60

Supplementary Table 6. Detailed information for risk of ALS-associated SNPs with circulating cytokines. 124

Supplementary Table 7. Detailed information for circulating cytokines-associated SNPs with risk of Alzheimer's disease (AD). 158

Supplementary Table 8. Detailed information for circulating cytokines-associated SNPs with risk of Parkinson's disease (PD). 173

Supplementary Table 9. Detailed information for circulating cytokines-associated SNPs with risk of amyotrophic lateral sclerosis (ALS). 187

Supplementary Table 10. Detailed information for circulating cytokines on age-related neurodegenerative diseases. 202

Supplementary Table 11. Detailed information for age-related neurodegenerative diseases on circulating cytokines. 222

Supplementary Figures 242

eFigure 1. BNGF-associated SNPs with risk of Alzheimer's disease (AD) 242

eFigure 2. CTACK-associated SNPs with risk of AD 243

eFigure 3. EOTAXIN-associated SNPs with risk of AD 245

eFigure 4. bFGF-associated SNPs with risk of AD 247

eFigure 5. G-CSF-associated SNPs with risk of AD 249

eFigure 6. GROA-associated SNPs with risk of AD 251

eFigure 7. HGF-associated SNPs with risk of AD 253

eFigure 8. IFN-G-associated SNPs with risk of AD 255

eFigure 9. IL-1B-associated SNPs with risk of AD 257

eFigure 10. IL-1RA-associated SNPs with risk of AD 258

eFigure 11. IL-2-associated SNPs with risk of AD 260

eFigure 12. IL-2RA-associated SNPs with risk of AD 262

eFigure 13. IL-4-associated SNPs with risk of AD 263

eFigure 14. IL-5-associated SNPs with risk of AD 264

eFigure 15. IL-6-associated SNPs with risk of AD 266

eFigure 16. IL-7-associated SNPs with risk of AD 267

eFigure 17. IL-8-associated SNPs with risk of AD 269
eFigure 18. IL-9-associated SNPs with risk of AD 270
eFigure 19. IL-10-associated SNPs with risk of AD 272
eFigure 20. IL-12-associated SNPs with risk of AD 273
eFigure 21. IL-13-associated SNPs with risk of AD 275
eFigure 22. IL-16-associated SNPs with risk of AD 276
eFigure 23. IL-17-associated SNPs with risk of AD 278
eFigure 24. IL-18-associated SNPs with risk of AD 279
eFigure 25. IP-10-associated SNPs with risk of AD 281
eFigure 26. M-CSF-associated SNPs with risk of AD 282
eFigure 27. MCP-1-MCAF-associated SNPs with risk of AD 284
eFigure 28. MCP-3-associated SNPs with risk of AD 285
eFigure 29. MIF-associated SNPs with risk of AD 287
eFigure 30. MIG-associated SNPs with risk of AD 289
eFigure 31. MIP-1A-associated SNPs with risk of AD 290
eFigure 32. MIP-1B-associated SNPs with risk of AD 291
eFigure 33. PDGF-BB-associated SNPs with risk of AD 293
eFigure 34. RANTES-associated SNPs with risk of AD 294
eFigure 35. SCF-associated SNPs with risk of AD 296
eFigure 36. SCGFβ-associated SNPs with risk of AD 297
eFigure 37. SDF-1A-associated SNPs with risk of AD 299
eFigure 38. TNF-A-associated SNPs with risk of AD 300
eFigure 39. TNF-B-associated SNPs with risk of AD 302
eFigure 40. TRAIL-associated SNPs with risk of AD 303
eFigure 41. VEGF-associated SNPs with risk of AD 305
eFigure 42. BNGF-associated SNPs with risk of PD 306
eFigure 43. CTACK-associated SNPs with risk of PD 308
eFigure 44. EOTAXIN-associated SNPs with risk of PD 309
eFigure 45. bFGF-associated SNPs with risk of PD 311
eFigure 46. G-CSF-associated SNPs with risk of PD 312
eFigure 47. GROA-associated SNPs with risk of PD 315
eFigure 48. HGF-associated SNPs with risk of PD 316
eFigure 49. IFN-G-associated SNPs with risk of PD 317
eFigure 50. IL-1B-associated SNPs with risk of PD 319
eFigure 51. IL-1RA-associated SNPs with risk of PD 320
eFigure 52. IL-2-associated SNPs with risk of PD 322
eFigure 53. IL-2RA-associated SNPs with risk of PD 323
eFigure 54. IL-4-associated SNPs with risk of PD 325
eFigure 55. IL-5-associated SNPs with risk of PD 326
eFigure 56. IL-6-associated SNPs with risk of PD 328
eFigure 57. IL-7-associated SNPs with risk of PD 329
eFigure 58. IL-8-associated SNPs with risk of PD 331
eFigure 59. IL-9-associated SNPs with risk of PD 332
eFigure 60. IL-10-associated SNPs with risk of PD 334

eFigure 61. IL-12-associated SNPs with risk of PD 335
eFigure 62. IL-13-associated SNPs with risk of PD 337
eFigure 63. IL-16-associated SNPs with risk of PD 338
eFigure 64. IL-17-associated SNPs with risk of PD 340
eFigure 65. IL-18-associated SNPs with risk of PD 341
eFigure 66. IP-10-associated SNPs with risk of PD 343
eFigure 67. M-CSF-associated SNPs with risk of PD 344
eFigure 68. MCP-1-MCAF-associated SNPs with risk of PD 346
eFigure 69. MCP-3-associated SNPs with risk of PD 348
eFigure 70. MIF-associated SNPs with risk of PD 349
eFigure 71. MIG-associated SNPs with risk of PD 350
eFigure 72. MIP-1A-associated SNPs with risk of PD 352
eFigure 73. MIP-1B-associated SNPs with risk of PD 353
eFigure 74. PDGF-BB-associated SNPs with risk of PD 355
eFigure 75. RANTES-associated SNPs with risk of PD 356
eFigure 76. SCF-associated SNPs with risk of PD 358
eFigure 77. SCGFβ-associated SNPs with risk of PD 359
eFigure 78. SDF-1A-associated SNPs with risk of PD 361
eFigure 79. TNF-A-associated SNPs with risk of PD 362
eFigure 80. TNF-B-associated SNPs with risk of PD 364
eFigure 81. TRAIL-associated SNPs with risk of PD 365
eFigure 82. VEGF-associated SNPs with risk of PD 367
eFigure 83. BNGF-associated SNPs with risk of ALS 368
eFigure 84. CTACK-associated SNPs with risk of ALS 370
eFigure 85. EOTAXIN-associated SNPs with risk of ALS 371
eFigure 86. bFGF-associated SNPs with risk of ALS 373
eFigure 87. G-CSF-associated SNPs with risk of ALS 374
eFigure 88. GROA-associated SNPs with risk of ALS 376
eFigure 89. HGF-associated SNPs with risk of ALS 377
eFigure 90. IFN-G-associated SNPs with risk of ALS 379
eFigure 91. IL-1B-associated SNPs with risk of ALS 380
eFigure 92. IL-1RA-associated SNPs with risk of ALS 382
eFigure 93. IL-2-associated SNPs with risk of ALS 383
eFigure 94. IL-2RA-associated SNPs with risk of ALS 385
eFigure 95. IL-4-associated SNPs with risk of ALS 386
eFigure 96. IL-5-associated SNPs with risk of ALS 388
eFigure 97. IL-6-associated SNPs with risk of ALS 389
eFigure 98. IL-7-associated SNPs with risk of ALS 391
eFigure 99. IL-8-associated SNPs with risk of ALS 392
eFigure 100. IL-9-associated SNPs with risk of ALS 394
eFigure 101. IL-10-associated SNPs with risk of ALS 395
eFigure 102. IL-12-associated SNPs with risk of ALS 397
eFigure 103. IL-13-associated SNPs with risk of ALS 398
eFigure 104. IL-16-associated SNPs with risk of ALS 400

eFigure 105. IL-17-associated SNPs with risk of ALS 401
eFigure 106. IL-18-associated SNPs with risk of ALS 403
eFigure 107. IP-10-associated SNPs with risk of ALS 404
eFigure 108. M-CSF-associated SNPs with risk of ALS 406
eFigure 109. MCP-1-MCAF-associated SNPs with risk of ALS 407
eFigure 110. MCP-3-associated SNPs with risk of ALS 409
eFigure 111. MIF-associated SNPs with risk of ALS 410
eFigure 112. MIG-associated SNPs with risk of ALS 412
eFigure 113. MIP-1A-associated SNPs with risk of ALS 413
eFigure 114. MIP-1B-associated SNPs with risk of ALS 415
eFigure 115. PDGF-BB-associated SNPs with risk of ALS 416
eFigure 116. RANTES-associated SNPs with risk of ALS 418
eFigure 117. SCF-associated SNPs with risk of ALS 419
eFigure 118. SCGFβ-associated SNPs with risk of ALS 421
eFigure 119. SDF-1A-associated SNPs with risk of ALS 422
eFigure 120. TNF-A-associated SNPs with risk of ALS 424
eFigure 121. TNF-B-associated SNPs with risk of ALS 425
eFigure 122. TRAIL-associated SNPs with risk of ALS 427
eFigure 123. VEGF-associated SNPs with risk of ALS 428
eFigure 124. AD-associated SNPs with BNGF 430
eFigure 125. AD-associated SNPs with CTACK 431
eFigure 126. AD-associated SNPs with EOTAXIN 433
eFigure 127. AD-associated SNPs with bFGF 434
eFigure 128. AD-associated SNPs with G-CSF 436
eFigure 129. AD-associated SNPs with GROA 437
eFigure 130. AD-associated SNPs with HGF 439
eFigure 131. AD-associated SNPs with IFN-G 440
eFigure 132. AD-associated SNPs with IL-1B 442
eFigure 133. AD-associated SNPs with IL-1RA 443
eFigure 134. AD-associated SNPs with IL-2 445
eFigure 135. AD-associated SNPs with IL-2RA 446
eFigure 136. AD-associated SNPs with IL-4 448
eFigure 137. AD-associated SNPs with IL-5 449
eFigure 138. AD-associated SNPs with IL-6 451
eFigure 139. AD-associated SNPs with IL-7 452
eFigure 140. AD-associated SNPs with IL-8 454
eFigure 141. AD-associated SNPs with IL-9 455
eFigure 142. AD-associated SNPs with IL-10 457
eFigure 143. AD-associated SNPs with IL-12 458
eFigure 144. AD-associated SNPs with IL-13 460
eFigure 145. AD-associated SNPs with IL-16 461
eFigure 146. AD-associated SNPs with IL-17 463
eFigure 147. AD-associated SNPs with IL-18 465
eFigure 148. AD-associated SNPs with IP-10 466

eFigure 149. AD-associated SNPs with M-CSF 467
eFigure 150. AD-associated SNPs with MCP-1-MCAF 469
eFigure 151. AD-associated SNPs with MCP-3 470
eFigure 152. AD-associated SNPs with MIF 472
eFigure 153. AD-associated SNPs with MIG 473
eFigure 154. AD-associated SNPs with MIP-1A 475
eFigure 155. AD-associated SNPs with MIP-1B 476
eFigure 156. AD-associated SNPs with PDGF-BB 478
eFigure 157. AD-associated SNPs with RANTES 479
eFigure 158. AD-associated SNPs with SCF 481
eFigure 159. AD-associated SNPs with SGCF 482
eFigure 160. AD-associated SNPs with SDF-1A 484
eFigure 161. AD-associated SNPs with TNF-A 485
eFigure 162. AD-associated SNPs with TNF-B 487
eFigure 163. AD-associated SNPs with TRAIL 488
eFigure 164. AD-associated SNPs with VEGF 490
eFigure 165. PD-associated SNPs with BNGF 491
eFigure 166. PD-associated SNPs with CTACK 493
eFigure 167. PD-associated SNPs with EOTAXIN 494
eFigure 168. PD-associated SNPs with bFGF 496
eFigure 169. PD-associated SNPs with G-CSF 497
eFigure 181. PD-associated SNPs with IL-8 499
eFigure 170. PD-associated SNPs with GROA 500
eFigure 171. PD-associated SNPs with HGF 502
eFigure 172. PD-associated SNPs with IFN-G 503
eFigure 173. PD-associated SNPs with IL-1B 505
eFigure 174. PD-associated SNPs with IL-1RA 506
eFigure 175. PD-associated SNPs with IL-2 508
eFigure 176. PD-associated SNPs with IL-2RA 509
eFigure 177. PD-associated SNPs with IL-4 511
eFigure 178. PD-associated SNPs with IL-5 512
eFigure 179. PD-associated SNPs with IL-6 514
eFigure 180. PD-associated SNPs with IL-7 515
eFigure 182. PD-associated SNPs with IL-9 517
eFigure 183. PD-associated SNPs with IL-10 518
eFigure 184. PD-associated SNPs with IL-12 520
eFigure 185. PD-associated SNPs with IL-13 521
eFigure 186. PD-associated SNPs with IL-16 523
eFigure 187. PD-associated SNPs with IL-17 524
eFigure 188. PD-associated SNPs with IL-18 527
eFigure 189. PD-associated SNPs with IP-10 528
eFigure 190. PD-associated SNPs with M-CSF 529
eFigure 191. PD-associated SNPs with MCP-1-MCAF 531
eFigure 192. PD-associated SNPs with MCP-3 532

eFigure 193. PD-associated SNPs with MIF 534
eFigure 194. PD-associated SNPs with MIG 535
eFigure 195. PD-associated SNPs with MIP-1A 537
eFigure 196. PD-associated SNPs with MIP-1B 538
eFigure 197. PD-associated SNPs with PDGF-BB 540
eFigure 198. PD-associated SNPs with RANTES 541
eFigure 199. PD-associated SNPs with SCF 543
eFigure 200. PD-associated SNPs with SGCF 544
eFigure 201. PD-associated SNPs with SDF-1A 546
eFigure 202. PD-associated SNPs with TNF-A 547
eFigure 203. PD-associated SNPs with TNF-B 549
eFigure 204. PD-associated SNPs with TRAIL 550
eFigure 205. PD-associated SNPs with VEGF 552
eFigure 206. ALS-associated SNPs with BNGF 553
eFigure 207. ALS-associated SNPs with CTACK 555
eFigure 208. ALS-associated SNPs with EOTAXIN 556
eFigure 209. ALS-associated SNPs with bFGF 558
eFigure 210. ALS-associated SNPs with G-CSF 559
eFigure 211. ALS-associated SNPs with GROA 561
eFigure 212. ALS-associated SNPs with HGF 562
eFigure 213. ALS-associated SNPs with IFN-G 564
eFigure 214. ALS-associated SNPs with IL-1B 565
eFigure 215. ALS-associated SNPs with IL-1RA 567
eFigure 216. ALS-associated SNPs with IL-2 568
eFigure 217. ALS-associated SNPs with IL-2RA 570
eFigure 218. ALS-associated SNPs with IL-4 571
eFigure 219. ALS-associated SNPs with IL-5 573
eFigure 220. ALS-associated SNPs with IL-6 574
eFigure 221. ALS-associated SNPs with IL-7 576
eFigure 222. ALS-associated SNPs with IL-8 577
eFigure 223. ALS-associated SNPs with IL-9 579
eFigure 224. ALS-associated SNPs with IL-10 580
eFigure 225. ALS-associated SNPs with IL-12 582
eFigure 226. ALS-associated SNPs with IL-13 583
eFigure 227. ALS-associated SNPs with IL-16 585
eFigure 228. ALS-associated SNPs with IL-17 586
eFigure 229. ALS-associated SNPs with IL-18 588
eFigure 230. ALS-associated SNPs with IP-10 589
eFigure 231. ALS-associated SNPs with M-CSF 591
eFigure 232. ALS-associated SNPs with MCP-1-MCAF 592
eFigure 233. ALS-associated SNPs with MCP-3 594
eFigure 234. ALS-associated SNPs with MIF 595
eFigure 235. ALS-associated SNPs with MIG 597
eFigure 236. ALS-associated SNPs with MIP-1A 598

eFigure 237. ALS-associated SNPs with MIP-1B 600
eFigure 238. ALS-associated SNPs with PDGF-BB 601
eFigure 239. ALS-associated SNPs with RANTES 603
eFigure 240. ALS-associated SNPs with SCF 604
eFigure 241. ALS-associated SNPs with SGCF 606
eFigure 242. ALS-associated SNPs with SDF-1A 607
eFigure 243. ALS-associated SNPs with TNF-A 609
eFigure 244. ALS-associated SNPs with TNF-B 610
eFigure 245. ALS-associated SNPs with TRAIL 612
eFigure 246. ALS-associated SNPs with VEGF 613

Supplementary Tables

Supplementary Table 1. Detailed information regarding datasets about AD demographics.

	Alzheimer's disease cases				Controls		
	**Consortium**	***N***	**Percent female**	**Mean AAO (s.d)**	***N***	**Percent female**	**Mean AAE (s.d)**
**ieu-b-2**	ADGC	14,428	59.3	71.1 (17.3)	14,562	59.3	76.2 (9.9)
	CHARGE	2,137	67.3	82.6 (12)	13,474	55.8	76.7 (8.2)
	EADI	2,240	65	75.4 (9.1)	6,631	60.6	78.9 (7.0)
	GERAD	3,177	64	73.0 (0.2)	7,277	51.8	51.0 (0.1)
	N	21,982	61.3	72.8 (10.5)	41,944	57.1	75.51 (9.2)

Supplementary Table 2. Detailed information regarding datasets about PD demographics.

	study	Parkinson's disease cases			Controls		
		N	Percent female	Mean AAO (s.d)	*N*	Percent female	Mean AAE (s.d)
**ieu-b-7**	23andMe, post-Chang et al. 2017 enrollment	2448	39.05	61.78 (10.94)	571411	54.86	51.47 (16.79)
	Baylor College of Medicine / University of Maryland	769	33.81	64.83 (10.12)	195	69.74	65.48 (8.31)
	Finnish Parkinson's	386	45.85	55.27 (5.64)	493	78.9	92.35 (3.86)
	Harvard Biomarker Study (HBS)	527	34.35	66.31 (10.07)	472	61.65	69.9 (9.02)
	McGill Parkinson's	582	34.54	65.71 (9.79)	905	48.4	55.79 (10.69)
	Oslo Parkinson's Disease Study	476	35.71	65.32 (9.28)	462	42.21	61.85 (11.06)
	Parkinson's Disease Biomarker's Program (PDBP)	512	38.67	64.46 (9.37)	282	51.06	62.19 (10.73)
	Parkinson's Progression Markers Initiative (PPMI)	363	33.06	64.24 (9.65)	165	33.33	63.79 (10.59)
	System Genomics of Parkinson's Disease (SGPD)	1169	35.24	59.88 (10.86)*	968	53.93	66.64 (9.65)*
	Spanish Parkinson's (IPDGC)	2110	43.13	63.92 (12.54)	1333	54.39	64.03 (12.59)
	Tubingen Parkinson's Disease cohort (CouragePD)	666	36.04	59.89 (11.25)	542	57.93	67.48 (8.41)
	Vance (dbGap phs000394)	620	27.74	77.47 (8.40)	299	50.84	81.98 (12.78)
	UK PDMED (CouragePD)	1025	32.78	NA	655	72.67	NA
	UK BioBank^^	18618	57.62	58.45 (7.20)	436419	54.14	56.69 (8.05)
	IPDGC (Nalls et al. 2014 discovery phase)	13708	NA	NA	95282	NA	NA
	NeuroX - dbGaP (phs000918.v1.p1)	5851	NA	NA	5866	NA	NA
	Parkinson's Disease Web-based Study (PDWBS)	6476	NA	NA	302042	NA	NA
	N	56306	NA	NA	1417791	NA	NA

Supplementary Table 3. Detailed information regarding datasets about ALS demographics.

	Amyotrophic Lateral Sclerosis cases				Controls		
	**Ethnic**	***N***	**Percent female**	**Mean AAO (s.d)**	***N***	**Percent female**	**Mean AAE (s.d)**
**ebi-a-GCST005647**	US	3,777	40	58.1 (12.3)	33,365	72	64.2 (13.3)
	Italian	2,853	1,239 (43.4)	61.8 (11.8)	2,143	896 (41.8)	50.6 (17.4)
	UK	449	43	60.3 (12.8)	226	48.2	60.3 (12.8)
	French & Belgian	1,150	42.3	60.5 (12.6)	595	70.9	66.9 (16.8)
	N	8,229	41.7	59.8 (12.3)	36,329	69.6	63.4 (13.9)

Supplementary Table 4. Detailed information for risk of AD-associated SNPs with circulating cytokines.

Outcomes	SNP	Effect Allele	Other Allele	Beta	Se	EAF	*P-value*	*R2*	*F*	Power
BNGF	rs1081105	C	A	0.942	0.0436	0.9692	1.51E-103	0.053	466.80	
BNGF	rs111278137	A	G	-0.4735	0.0713	0.0139	3.20E-11	0.006	44.10	
BNGF	rs11168036	G	T	-0.0754	0.0143	0.5169	1.43E-07	0.003	27.80	
BNGF	rs11767557	C	T	-0.1028	0.0182	0.2177	1.56E-08	0.004	31.90	
BNGF	rs12151021	G	A	-0.1071	0.0169	0.338	2.56E-10	0.005	40.16	
BNGF	rs12590654	A	G	-0.0906	0.0157	0.3370	8.73E-09	0.004	33.30	
BNGF	rs13252043	T	C	0.114	0.0237	0.9066	1.57E-06	0.002	23.14	
BNGF	rs138727474	T	C	-0.2515	0.0545	0.9761	3.90E-06	0.003	21.30	
BNGF	rs141739979	T	G	-0.4544	0.0855	0.9861	1.07E-07	0.006	28.25	
BNGF	rs143429938	T	C	0.3535	0.0769	0.9732	4.25E-06	0.007	21.13	
BNGF	rs147711004	A	G	1.1354	0.0366	0.0288	1.00E-200	0.072	962.36	
BNGF	rs150685845	G	A	0.5561	0.0645	0.9871	6.62E-18	0.008	74.33	89.00%
BNGF	rs1582763	A	G	-0.1232	0.0149	0.6372	1.19E-16	0.007	68.37	
BNGF	rs17125924	G	A	0.1222	0.0246	0.9205	6.62E-07	0.002	24.68	
BNGF	rs2830489	T	C	-0.0837	0.0162	0.7038	2.42E-07	0.003	26.69	
BNGF	rs34665982	C	T	-0.0967	0.0166	0.4901	5.80E-09	0.005	33.93	
BNGF	rs34971488	A	G	0.094	0.0198	0.2346	2.07E-06	0.003	22.54	
BNGF	rs35695568	T	G	0.1152	0.0247	0.8986	3.20E-06	0.002	21.75	
BNGF	rs3740688	T	G	0.0935	0.0144	0.4573	9.70E-11	0.004	42.16	
BNGF	rs383902	T	C	-0.0698	0.0151	0.6402	3.81E-06	0.002	21.37	
BNGF	rs3851179	C	T	0.1198	0.0148	0.3708	5.81E-16	0.007	65.52	
BNGF	rs6014724	G	A	-0.1319	0.0259	0.9105	3.65E-07	0.003	25.94	
BNGF	rs62039712	A	G	0.1528	0.0288	0.1213	1.17E-07	0.005	28.15	
BNGF	rs6559689	T	C	0.1585	0.0335	0.9483	2.17E-06	0.002	22.39	

BNGF	rs6733839	T	C	0.1693	0.0154	0.6203	4.02E-28	0.014	120.86	
BNGF	rs679515	C	T	-0.1508	0.0183	0.828	1.55E-16	0.006	67.90	
BNGF	rs72654445	A	G	-0.5425	0.0811	0.0109	2.27E-11	0.006	44.75	
BNGF	rs72993825	T	C	-0.1763	0.036	0.9404	9.86E-07	0.003	23.98	
BNGF	rs73223431	T	C	0.0936	0.0153	0.6541	8.34E-10	0.004	37.43	
BNGF	rs7412	T	C	-0.4673	0.0305	0.9374	6.40E-53	0.026	234.74	
BNGF	rs7584040	T	C	0.0862	0.0172	0.8131	5.34E-07	0.002	25.12	
BNGF	rs7618668	A	G	-0.1297	0.0258	0.0815	4.95E-07	0.003	25.27	
BNGF	rs8111708	G	A	0.0696	0.0151	0.6541	3.95E-06	0.002	21.25	
BNGF	rs867230	A	C	0.1333	0.0158	0.5984	3.49E-17	0.009	71.18	
BNGF	rs9381563	T	C	-0.0821	0.0148	0.3221	2.93E-08	0.003	30.77	
BNGF	rs9649710	G	A	0.0676	0.0148	0.3767	4.79E-06	0.002	20.86	
BNGF	rs9787911	C	T	0.0662	0.0144	0.4274	4.39E-06	0.002	21.13	
CTACK	rs1081105	C	A	0.942	0.0436	0.9692	1.51E-103	0.053	466.80	
CTACK	rs111278137	A	G	-0.4735	0.0713	0.0139	3.20E-11	0.006	44.10	
CTACK	rs11168036	G	T	-0.0754	0.0143	0.5169	1.43E-07	0.003	27.80	
CTACK	rs117240937	A	G	-0.3122	0.0672	0.0179	3.35E-06	0.003	21.58	
CTACK	rs11767557	C	T	-0.1028	0.0182	0.2177	1.56E-08	0.004	31.90	
CTACK	rs12151021	G	A	-0.1071	0.0169	0.338	2.56E-10	0.005	40.16	
CTACK	rs12590654	A	G	-0.0906	0.0157	0.3370	8.73E-09	0.004	33.30	65.80%
CTACK	rs13252043	T	C	0.114	0.0237	0.9066	1.57E-06	0.002	23.14	
CTACK	rs138727474	T	C	-0.2515	0.0545	0.9761	3.90E-06	0.003	21.30	
CTACK	rs141739979	T	G	-0.4544	0.0855	0.9861	1.07E-07	0.006	28.25	
CTACK	rs143429938	T	C	0.3535	0.0769	0.9732	4.25E-06	0.007	21.13	
CTACK	rs147711004	A	G	1.1354	0.0366	0.0288	1.00E-200	0.072	962.36	
CTACK	rs150685845	G	A	0.5561	0.0645	0.9871	6.62E-18	0.008	74.33	

CTACK	rs1582763	A	G	-0.1232	0.0149	0.6372	1.19E-16	0.007	68.37	
CTACK	rs17125924	G	A	0.1222	0.0246	0.9205	6.62E-07	0.002	24.68	
CTACK	rs2830489	T	C	-0.0837	0.0162	0.7038	2.42E-07	0.003	26.69	
CTACK	rs34665982	C	T	-0.0967	0.0166	0.4901	5.80E-09	0.005	33.93	
CTACK	rs34971488	A	G	0.094	0.0198	0.2346	2.07E-06	0.003	22.54	
CTACK	rs35695568	T	G	0.1152	0.0247	0.8986	3.20E-06	0.002	21.75	
CTACK	rs3740688	T	G	0.0935	0.0144	0.4573	9.70E-11	0.004	42.16	
CTACK	rs383902	T	C	-0.0698	0.0151	0.6402	3.81E-06	0.002	21.37	
CTACK	rs3851179	C	T	0.1198	0.0148	0.3708	5.81E-16	0.007	65.52	
CTACK	rs6014724	G	A	-0.1319	0.0259	0.9105	3.65E-07	0.003	25.94	
CTACK	rs62039712	A	G	0.1528	0.0288	0.1213	1.17E-07	0.005	28.15	
CTACK	rs6559689	T	C	0.1585	0.0335	0.9483	2.17E-06	0.002	22.39	
CTACK	rs6733839	T	C	0.1693	0.0154	0.6203	4.02E-28	0.014	120.86	
CTACK	rs679515	C	T	-0.1508	0.0183	0.828	1.55E-16	0.006	67.90	
CTACK	rs72654445	A	G	-0.5425	0.0811	0.0109	2.27E-11	0.006	44.75	
CTACK	rs72993825	T	C	-0.1763	0.036	0.9404	9.86E-07	0.003	23.98	
CTACK	rs73223431	T	C	0.0936	0.0153	0.6541	8.34E-10	0.004	37.43	
CTACK	rs7412	T	C	-0.4673	0.0305	0.9374	6.40E-53	0.026	234.74	
CTACK	rs7584040	T	C	0.0862	0.0172	0.8131	5.34E-07	0.002	25.12	
CTACK	rs7618668	A	G	-0.1297	0.0258	0.0815	4.95E-07	0.003	25.27	
CTACK	rs8111708	G	A	0.0696	0.0151	0.6541	3.95E-06	0.002	21.25	
CTACK	rs867230	A	C	0.1333	0.0158	0.5984	3.49E-17	0.009	71.18	
CTACK	rs9381563	T	C	-0.0821	0.0148	0.3221	2.93E-08	0.003	30.77	
CTACK	rs9649710	G	A	0.0676	0.0148	0.3767	4.79E-06	0.002	20.86	
CTACK	rs9787911	C	T	0.0662	0.0144	0.4274	4.39E-06	0.002	21.13	
EOTAXIN	rs1081105	C	A	0.942	0.0436	0.9692	1.51E-103	0.053	466.80	99.10%

EOTAXIN	rs111278137	A	G	-0.4735	0.0713	0.0139	3.20E-11	0.006	44.10
EOTAXIN	rs11168036	G	T	-0.0754	0.0143	0.5169	1.43E-07	0.003	27.80
EOTAXIN	rs117240937	A	G	-0.3122	0.0672	0.0179	3.35E-06	0.003	21.58
EOTAXIN	rs11767557	C	T	-0.1028	0.0182	0.2177	1.56E-08	0.004	31.90
EOTAXIN	rs12151021	G	A	-0.1071	0.0169	0.338	2.56E-10	0.005	40.16
EOTAXIN	rs12590654	A	G	-0.0906	0.0157	0.3370	8.73E-09	0.004	33.30
EOTAXIN	rs13252043	T	C	0.114	0.0237	0.9066	1.57E-06	0.002	23.14
EOTAXIN	rs138727474	T	C	-0.2515	0.0545	0.9761	3.90E-06	0.003	21.30
EOTAXIN	rs141739979	T	G	-0.4544	0.0855	0.9861	1.07E-07	0.006	28.25
EOTAXIN	rs143429938	T	C	0.3535	0.0769	0.9732	4.25E-06	0.007	21.13
EOTAXIN	rs147711004	A	G	1.1354	0.0366	0.0288	1.00E-200	0.072	962.36
EOTAXIN	rs150685845	G	A	0.5561	0.0645	0.9871	6.62E-18	0.008	74.33
EOTAXIN	rs1582763	A	G	-0.1232	0.0149	0.6372	1.19E-16	0.007	68.37
EOTAXIN	rs17125924	G	A	0.1222	0.0246	0.9205	6.62E-07	0.002	24.68
EOTAXIN	rs2830489	T	C	-0.0837	0.0162	0.7038	2.42E-07	0.003	26.69
EOTAXIN	rs34665982	C	T	-0.0967	0.0166	0.4901	5.80E-09	0.005	33.93
EOTAXIN	rs34971488	A	G	0.094	0.0198	0.2346	2.07E-06	0.003	22.54
EOTAXIN	rs35695568	T	G	0.1152	0.0247	0.8986	3.20E-06	0.002	21.75
EOTAXIN	rs3740688	T	G	0.0935	0.0144	0.4573	9.70E-11	0.004	42.16
EOTAXIN	rs383902	T	C	-0.0698	0.0151	0.6402	3.81E-06	0.002	21.37
EOTAXIN	rs3851179	C	T	0.1198	0.0148	0.3708	5.81E-16	0.007	65.52
EOTAXIN	rs6014724	G	A	-0.1319	0.0259	0.9105	3.65E-07	0.003	25.94
EOTAXIN	rs62039712	A	G	0.1528	0.0288	0.1213	1.17E-07	0.005	28.15
EOTAXIN	rs6559689	T	C	0.1585	0.0335	0.9483	2.17E-06	0.002	22.39
EOTAXIN	rs6733839	T	C	0.1693	0.0154	0.6203	4.02E-28	0.014	120.86
EOTAXIN	rs679515	C	T	-0.1508	0.0183	0.828	1.55E-16	0.006	67.90

EOTAXIN	rs72654445	A	G	-0.5425	0.0811	0.0109	2.27E-11	0.006	44.75	
EOTAXIN	rs72993825	T	C	-0.1763	0.036	0.9404	9.86E-07	0.003	23.98	
EOTAXIN	rs73223431	T	C	0.0936	0.0153	0.6541	8.34E-10	0.004	37.43	
EOTAXIN	rs7412	T	C	-0.4673	0.0305	0.9374	6.40E-53	0.026	234.74	
EOTAXIN	rs7584040	T	C	0.0862	0.0172	0.8131	5.34E-07	0.002	25.12	
EOTAXIN	rs7618668	A	G	-0.1297	0.0258	0.0815	4.95E-07	0.003	25.27	
EOTAXIN	rs8111708	G	A	0.0696	0.0151	0.6541	3.95E-06	0.002	21.25	
EOTAXIN	rs867230	A	C	0.1333	0.0158	0.5984	3.49E-17	0.009	71.18	
EOTAXIN	rs9381563	T	C	-0.0821	0.0148	0.3221	2.93E-08	0.003	30.77	
EOTAXIN	rs9649710	G	A	0.0676	0.0148	0.3767	4.79E-06	0.002	20.86	
EOTAXIN	rs9787911	C	T	0.0662	0.0144	0.4274	4.39E-06	0.002	21.13	
FGF	rs1081105	C	A	0.942	0.0436	0.9692	1.51E-103	0.053	466.80	
FGF	rs111278137	A	G	-0.4735	0.0713	0.0139	3.20E-11	0.006	44.10	
FGF	rs11168036	G	T	-0.0754	0.0143	0.5169	1.43E-07	0.003	27.80	
FGF	rs117240937	A	G	-0.3122	0.0672	0.0179	3.35E-06	0.003	21.58	
FGF	rs11767557	C	T	-0.1028	0.0182	0.2177	1.56E-08	0.004	31.90	
FGF	rs12151021	G	A	-0.1071	0.0169	0.338	2.56E-10	0.005	40.16	
FGF	rs12590654	A	G	-0.0906	0.0157	0.3370	8.73E-09	0.004	33.30	
FGF	rs13252043	T	C	0.114	0.0237	0.9066	1.57E-06	0.002	23.14	100.00%
FGF	rs138727474	T	C	-0.2515	0.0545	0.9761	3.90E-06	0.003	21.30	
FGF	rs141739979	T	G	-0.4544	0.0855	0.9861	1.07E-07	0.006	28.25	
FGF	rs143429938	T	C	0.3535	0.0769	0.9732	4.25E-06	0.007	21.13	
FGF	rs147711004	A	G	1.1354	0.0366	0.0288	1.00E-200	0.072	962.36	
FGF	rs150685845	G	A	0.5561	0.0645	0.9871	6.62E-18	0.008	74.33	
FGF	rs1582763	A	G	-0.1232	0.0149	0.6372	1.19E-16	0.007	68.37	
FGF	rs17125924	G	A	0.1222	0.0246	0.9205	6.62E-07	0.002	24.68	

FGF	rs2830489	T	C	-0.0837	0.0162	0.7038	2.42E-07	0.003	26.69	
FGF	rs34665982	C	T	-0.0967	0.0166	0.4901	5.80E-09	0.005	33.93	
FGF	rs34971488	A	G	0.094	0.0198	0.2346	2.07E-06	0.003	22.54	
FGF	rs35695568	T	G	0.1152	0.0247	0.8986	3.20E-06	0.002	21.75	
FGF	rs3740688	T	G	0.0935	0.0144	0.4573	9.70E-11	0.004	42.16	
FGF	rs383902	T	C	-0.0698	0.0151	0.6402	3.81E-06	0.002	21.37	
FGF	rs3851179	C	T	0.1198	0.0148	0.3708	5.81E-16	0.007	65.52	
FGF	rs6014724	G	A	-0.1319	0.0259	0.9105	3.65E-07	0.003	25.94	
FGF	rs62039712	A	G	0.1528	0.0288	0.1213	1.17E-07	0.005	28.15	
FGF	rs6559689	T	C	0.1585	0.0335	0.9483	2.17E-06	0.002	22.39	
FGF	rs6733839	T	C	0.1693	0.0154	0.6203	4.02E-28	0.014	120.86	
FGF	rs679515	C	T	-0.1508	0.0183	0.828	1.55E-16	0.006	67.90	
FGF	rs72654445	A	G	-0.5425	0.0811	0.0109	2.27E-11	0.006	44.75	
FGF	rs72993825	T	C	-0.1763	0.036	0.9404	9.86E-07	0.003	23.98	
FGF	rs73223431	T	C	0.0936	0.0153	0.6541	8.34E-10	0.004	37.43	
FGF	rs7412	T	C	-0.4673	0.0305	0.9374	6.40E-53	0.026	234.74	
FGF	rs7584040	T	C	0.0862	0.0172	0.8131	5.34E-07	0.002	25.12	
FGF	rs7618668	A	G	-0.1297	0.0258	0.0815	4.95E-07	0.003	25.27	
FGF	rs8111708	G	A	0.0696	0.0151	0.6541	3.95E-06	0.002	21.25	
FGF	rs867230	A	C	0.1333	0.0158	0.5984	3.49E-17	0.009	71.18	
FGF	rs9381563	T	C	-0.0821	0.0148	0.3221	2.93E-08	0.003	30.77	
FGF	rs9649710	G	A	0.0676	0.0148	0.3767	4.79E-06	0.002	20.86	
FGF	rs9787911	C	T	0.0662	0.0144	0.4274	4.39E-06	0.002	21.13	
G-CSF	rs1081105	C	A	0.942	0.0436	0.9692	1.51E-103	0.053	466.80	
G-CSF	rs111278137	A	G	-0.4735	0.0713	0.0139	3.20E-11	0.006	44.10	100.00%
G-CSF	rs11168036	G	T	-0.0754	0.0143	0.5169	1.43E-07	0.003	27.80	

G-CSF	rs117240937	A	G	-0.3122	0.0672	0.0179	3.35E-06	0.003	21.58
G-CSF	rs11767557	C	T	-0.1028	0.0182	0.2177	1.56E-08	0.004	31.90
G-CSF	rs12151021	G	A	-0.1071	0.0169	0.338	2.56E-10	0.005	40.16
G-CSF	rs12590654	A	G	-0.0906	0.0157	0.3370	8.73E-09	0.004	33.30
G-CSF	rs13252043	T	C	0.114	0.0237	0.9066	1.57E-06	0.002	23.14
G-CSF	rs138727474	T	C	-0.2515	0.0545	0.9761	3.90E-06	0.003	21.30
G-CSF	rs141739979	T	G	-0.4544	0.0855	0.9861	1.07E-07	0.006	28.25
G-CSF	rs143429938	T	C	0.3535	0.0769	0.9732	4.25E-06	0.007	21.13
G-CSF	rs147711004	A	G	1.1354	0.0366	0.0288	1.00E-200	0.072	962.36
G-CSF	rs150685845	G	A	0.5561	0.0645	0.9871	6.62E-18	0.008	74.33
G-CSF	rs1582763	A	G	-0.1232	0.0149	0.6372	1.19E-16	0.007	68.37
G-CSF	rs17125924	G	A	0.1222	0.0246	0.9205	6.62E-07	0.002	24.68
G-CSF	rs2830489	T	C	-0.0837	0.0162	0.7038	2.42E-07	0.003	26.69
G-CSF	rs34665982	C	T	-0.0967	0.0166	0.4901	5.80E-09	0.005	33.93
G-CSF	rs34971488	A	G	0.094	0.0198	0.2346	2.07E-06	0.003	22.54
G-CSF	rs35695568	T	G	0.1152	0.0247	0.8986	3.20E-06	0.002	21.75
G-CSF	rs3740688	T	G	0.0935	0.0144	0.4573	9.70E-11	0.004	42.16
G-CSF	rs383902	T	C	-0.0698	0.0151	0.6402	3.81E-06	0.002	21.37
G-CSF	rs3851179	C	T	0.1198	0.0148	0.3708	5.81E-16	0.007	65.52
G-CSF	rs6014724	G	A	-0.1319	0.0259	0.9105	3.65E-07	0.003	25.94
G-CSF	rs62039712	A	G	0.1528	0.0288	0.1213	1.17E-07	0.005	28.15
G-CSF	rs6559689	T	C	0.1585	0.0335	0.9483	2.17E-06	0.002	22.39
G-CSF	rs6733839	T	C	0.1693	0.0154	0.6203	4.02E-28	0.014	120.86
G-CSF	rs679515	C	T	-0.1508	0.0183	0.828	1.55E-16	0.006	67.90
G-CSF	rs72654445	A	G	-0.5425	0.0811	0.0109	2.27E-11	0.006	44.75
G-CSF	rs72993825	T	C	-0.1763	0.036	0.9404	9.86E-07	0.003	23.98

G-CSF	rs73223431	T	C	0.0936	0.0153	0.6541	8.34E-10	0.004	37.43	
G-CSF	rs7412	T	C	-0.4673	0.0305	0.9374	6.40E-53	0.026	234.74	
G-CSF	rs7584040	T	C	0.0862	0.0172	0.8131	5.34E-07	0.002	25.12	
G-CSF	rs7618668	A	G	-0.1297	0.0258	0.0815	4.95E-07	0.003	25.27	
G-CSF	rs8111708	G	A	0.0696	0.0151	0.6541	3.95E-06	0.002	21.25	
G-CSF	rs867230	A	C	0.1333	0.0158	0.5984	3.49E-17	0.009	71.18	
G-CSF	rs9381563	T	C	-0.0821	0.0148	0.3221	2.93E-08	0.003	30.77	
G-CSF	rs9649710	G	A	0.0676	0.0148	0.3767	4.79E-06	0.002	20.86	
G-CSF	rs9787911	C	T	0.0662	0.0144	0.4274	4.39E-06	0.002	21.13	
GROA	rs1081105	C	A	0.942	0.0436	0.9692	1.51E-103	0.053	466.80	
GROA	rs111278137	A	G	-0.4735	0.0713	0.0139	3.20E-11	0.006	44.10	
GROA	rs11168036	G	T	-0.0754	0.0143	0.5169	1.43E-07	0.003	27.80	
GROA	rs117240937	A	G	-0.3122	0.0672	0.0179	3.35E-06	0.003	21.58	
GROA	rs11767557	C	T	-0.1028	0.0182	0.2177	1.56E-08	0.004	31.90	
GROA	rs12151021	G	A	-0.1071	0.0169	0.338	2.56E-10	0.005	40.16	
GROA	rs12590654	A	G	-0.0906	0.0157	0.3370	8.73E-09	0.004	33.30	
GROA	rs13252043	T	C	0.114	0.0237	0.9066	1.57E-06	0.002	23.14	
GROA	rs138727474	T	C	-0.2515	0.0545	0.9761	3.90E-06	0.003	21.30	28.50%
GROA	rs141739979	T	G	-0.4544	0.0855	0.9861	1.07E-07	0.006	28.25	
GROA	rs143429938	T	C	0.3535	0.0769	0.9732	4.25E-06	0.007	21.13	
GROA	rs147711004	A	G	1.1354	0.0366	0.0288	1.00E-200	0.072	962.36	
GROA	rs150685845	G	A	0.5561	0.0645	0.9871	6.62E-18	0.008	74.33	
GROA	rs1582763	A	G	-0.1232	0.0149	0.6372	1.19E-16	0.007	68.37	
GROA	rs17125924	G	A	0.1222	0.0246	0.9205	6.62E-07	0.002	24.68	
GROA	rs2830489	T	C	-0.0837	0.0162	0.7038	2.42E-07	0.003	26.69	
GROA	rs34665982	C	T	-0.0967	0.0166	0.4901	5.80E-09	0.005	33.93	

GROA	rs34971488	A	G	0.094	0.0198	0.2346	2.07E-06	0.003	22.54	
GROA	rs35695568	T	G	0.1152	0.0247	0.8986	3.20E-06	0.002	21.75	
GROA	rs3740688	T	G	0.0935	0.0144	0.4573	9.70E-11	0.004	42.16	
GROA	rs383902	T	C	-0.0698	0.0151	0.6402	3.81E-06	0.002	21.37	
GROA	rs3851179	C	T	0.1198	0.0148	0.3708	5.81E-16	0.007	65.52	
GROA	rs6014724	G	A	-0.1319	0.0259	0.9105	3.65E-07	0.003	25.94	
GROA	rs62039712	A	G	0.1528	0.0288	0.1213	1.17E-07	0.005	28.15	
GROA	rs6559689	T	C	0.1585	0.0335	0.9483	2.17E-06	0.002	22.39	
GROA	rs6733839	T	C	0.1693	0.0154	0.6203	4.02E-28	0.014	120.86	
GROA	rs679515	C	T	-0.1508	0.0183	0.828	1.55E-16	0.006	67.90	
GROA	rs72654445	A	G	-0.5425	0.0811	0.0109	2.27E-11	0.006	44.75	
GROA	rs72993825	T	C	-0.1763	0.036	0.9404	9.86E-07	0.003	23.98	
GROA	rs73223431	T	C	0.0936	0.0153	0.6541	8.34E-10	0.004	37.43	
GROA	rs7412	T	C	-0.4673	0.0305	0.9374	6.40E-53	0.026	234.74	
GROA	rs7584040	T	C	0.0862	0.0172	0.8131	5.34E-07	0.002	25.12	
GROA	rs7618668	A	G	-0.1297	0.0258	0.0815	4.95E-07	0.003	25.27	
GROA	rs8111708	G	A	0.0696	0.0151	0.6541	3.95E-06	0.002	21.25	
GROA	rs867230	A	C	0.1333	0.0158	0.5984	3.49E-17	0.009	71.18	
GROA	rs9381563	T	C	-0.0821	0.0148	0.3221	2.93E-08	0.003	30.77	
GROA	rs9649710	G	A	0.0676	0.0148	0.3767	4.79E-06	0.002	20.86	
GROA	rs9787911	C	T	0.0662	0.0144	0.4274	4.39E-06	0.002	21.13	
HGF	rs1081105	C	A	0.942	0.0436	0.9692	1.51E-103	0.053	466.80	
HGF	rs111278137	A	G	-0.4735	0.0713	0.0139	3.20E-11	0.006	44.10	
HGF	rs11168036	G	T	-0.0754	0.0143	0.5169	1.43E-07	0.003	27.80	38.50%
HGF	rs117240937	A	G	-0.3122	0.0672	0.0179	3.35E-06	0.003	21.58	
HGF	rs11767557	C	T	-0.1028	0.0182	0.2177	1.56E-08	0.004	31.90	

HGF	rs12151021	G	A	-0.1071	0.0169	0.338	2.56E-10	0.005	40.16
HGF	rs12590654	A	G	-0.0906	0.0157	0.3370	8.73E-09	0.004	33.30
HGF	rs13252043	T	C	0.114	0.0237	0.9066	1.57E-06	0.002	23.14
HGF	rs138727474	T	C	-0.2515	0.0545	0.9761	3.90E-06	0.003	21.30
HGF	rs141739979	T	G	-0.4544	0.0855	0.9861	1.07E-07	0.006	28.25
HGF	rs143429938	T	C	0.3535	0.0769	0.9732	4.25E-06	0.007	21.13
HGF	rs147711004	A	G	1.1354	0.0366	0.0288	1.00E-200	0.072	962.36
HGF	rs150685845	G	A	0.5561	0.0645	0.9871	6.62E-18	0.008	74.33
HGF	rs1582763	A	G	-0.1232	0.0149	0.6372	1.19E-16	0.007	68.37
HGF	rs17125924	G	A	0.1222	0.0246	0.9205	6.62E-07	0.002	24.68
HGF	rs2830489	T	C	-0.0837	0.0162	0.7038	2.42E-07	0.003	26.69
HGF	rs34665982	C	T	-0.0967	0.0166	0.4901	5.80E-09	0.005	33.93
HGF	rs34971488	A	G	0.094	0.0198	0.2346	2.07E-06	0.003	22.54
HGF	rs35695568	T	G	0.1152	0.0247	0.8986	3.20E-06	0.002	21.75
HGF	rs3740688	T	G	0.0935	0.0144	0.4573	9.70E-11	0.004	42.16
HGF	rs383902	T	C	-0.0698	0.0151	0.6402	3.81E-06	0.002	21.37
HGF	rs3851179	C	T	0.1198	0.0148	0.3708	5.81E-16	0.007	65.52
HGF	rs6014724	G	A	-0.1319	0.0259	0.9105	3.65E-07	0.003	25.94
HGF	rs62039712	A	G	0.1528	0.0288	0.1213	1.17E-07	0.005	28.15
HGF	rs6559689	T	C	0.1585	0.0335	0.9483	2.17E-06	0.002	22.39
HGF	rs6733839	T	C	0.1693	0.0154	0.6203	4.02E-28	0.014	120.86
HGF	rs679515	C	T	-0.1508	0.0183	0.828	1.55E-16	0.006	67.90
HGF	rs72654445	A	G	-0.5425	0.0811	0.0109	2.27E-11	0.006	44.75
HGF	rs72993825	T	C	-0.1763	0.036	0.9404	9.86E-07	0.003	23.98
HGF	rs73223431	T	C	0.0936	0.0153	0.6541	8.34E-10	0.004	37.43
HGF	rs7412	T	C	-0.4673	0.0305	0.9374	6.40E-53	0.026	234.74

HGF	rs7584040	T	C	0.0862	0.0172	0.8131	5.34E-07	0.002	25.12	
HGF	rs7618668	A	G	-0.1297	0.0258	0.0815	4.95E-07	0.003	25.27	
HGF	rs8111708	G	A	0.0696	0.0151	0.6541	3.95E-06	0.002	21.25	
HGF	rs867230	A	C	0.1333	0.0158	0.5984	3.49E-17	0.009	71.18	
HGF	rs9381563	T	C	-0.0821	0.0148	0.3221	2.93E-08	0.003	30.77	
HGF	rs9649710	G	A	0.0676	0.0148	0.3767	4.79E-06	0.002	20.86	
HGF	rs9787911	C	T	0.0662	0.0144	0.4274	4.39E-06	0.002	21.13	
IFN-G	rs1081105	C	A	0.942	0.0436	0.9692	1.51E-103	0.053	466.80	
IFN-G	rs111278137	A	G	-0.4735	0.0713	0.0139	3.20E-11	0.006	44.10	
IFN-G	rs11168036	G	T	-0.0754	0.0143	0.5169	1.43E-07	0.003	27.80	
IFN-G	rs117240937	A	G	-0.3122	0.0672	0.0179	3.35E-06	0.003	21.58	
IFN-G	rs11767557	C	T	-0.1028	0.0182	0.2177	1.56E-08	0.004	31.90	
IFN-G	rs12151021	G	A	-0.1071	0.0169	0.338	2.56E-10	0.005	40.16	
IFN-G	rs12590654	A	G	-0.0906	0.0157	0.3370	8.73E-09	0.004	33.30	
IFN-G	rs13252043	T	C	0.114	0.0237	0.9066	1.57E-06	0.002	23.14	
IFN-G	rs138727474	T	C	-0.2515	0.0545	0.9761	3.90E-06	0.003	21.30	
IFN-G	rs141739979	T	G	-0.4544	0.0855	0.9861	1.07E-07	0.006	28.25	96.40%
IFN-G	rs143429938	T	C	0.3535	0.0769	0.9732	4.25E-06	0.007	21.13	
IFN-G	rs147711004	A	G	1.1354	0.0366	0.0288	1.00E-200	0.072	962.36	
IFN-G	rs150685845	G	A	0.5561	0.0645	0.9871	6.62E-18	0.008	74.33	
IFN-G	rs1582763	A	G	-0.1232	0.0149	0.6372	1.19E-16	0.007	68.37	
IFN-G	rs17125924	G	A	0.1222	0.0246	0.9205	6.62E-07	0.002	24.68	
IFN-G	rs2830489	T	C	-0.0837	0.0162	0.7038	2.42E-07	0.003	26.69	
IFN-G	rs34665982	C	T	-0.0967	0.0166	0.4901	5.80E-09	0.005	33.93	
IFN-G	rs34971488	A	G	0.094	0.0198	0.2346	2.07E-06	0.003	22.54	
IFN-G	rs35695568	T	G	0.1152	0.0247	0.8986	3.20E-06	0.002	21.75	

IFN-G	rs3740688	T	G	0.0935	0.0144	0.4573	9.70E-11	0.004	42.16	
IFN-G	rs383902	T	C	-0.0698	0.0151	0.6402	3.81E-06	0.002	21.37	
IFN-G	rs3851179	C	T	0.1198	0.0148	0.3708	5.81E-16	0.007	65.52	
IFN-G	rs6014724	G	A	-0.1319	0.0259	0.9105	3.65E-07	0.003	25.94	
IFN-G	rs62039712	A	G	0.1528	0.0288	0.1213	1.17E-07	0.005	28.15	
IFN-G	rs6559689	T	C	0.1585	0.0335	0.9483	2.17E-06	0.002	22.39	
IFN-G	rs6733839	T	C	0.1693	0.0154	0.6203	4.02E-28	0.014	120.86	
IFN-G	rs679515	C	T	-0.1508	0.0183	0.828	1.55E-16	0.006	67.90	
IFN-G	rs72654445	A	G	-0.5425	0.0811	0.0109	2.27E-11	0.006	44.75	
IFN-G	rs72993825	T	C	-0.1763	0.036	0.9404	9.86E-07	0.003	23.98	
IFN-G	rs73223431	T	C	0.0936	0.0153	0.6541	8.34E-10	0.004	37.43	
IFN-G	rs7412	T	C	-0.4673	0.0305	0.9374	6.40E-53	0.026	234.74	
IFN-G	rs7584040	T	C	0.0862	0.0172	0.8131	5.34E-07	0.002	25.12	
IFN-G	rs7618668	A	G	-0.1297	0.0258	0.0815	4.95E-07	0.003	25.27	
IFN-G	rs8111708	G	A	0.0696	0.0151	0.6541	3.95E-06	0.002	21.25	
IFN-G	rs867230	A	C	0.1333	0.0158	0.5984	3.49E-17	0.009	71.18	
IFN-G	rs9381563	T	C	-0.0821	0.0148	0.3221	2.93E-08	0.003	30.77	
IFN-G	rs9649710	G	A	0.0676	0.0148	0.3767	4.79E-06	0.002	20.86	
IFN-G	rs9787911	C	T	0.0662	0.0144	0.4274	4.39E-06	0.002	21.13	
IL-10	rs1081105	C	A	0.942	0.0436	0.9692	1.51E-103	0.053	466.80	
IL-10	rs111278137	A	G	-0.4735	0.0713	0.0139	3.20E-11	0.006	44.10	
IL-10	rs11168036	G	T	-0.0754	0.0143	0.5169	1.43E-07	0.003	27.80	
IL-10	rs11767557	C	T	-0.1028	0.0182	0.2177	1.56E-08	0.004	31.90	100.00%
IL-10	rs12151021	G	A	-0.1071	0.0169	0.338	2.56E-10	0.005	40.16	
IL-10	rs12590654	A	G	-0.0906	0.0157	0.3370	8.73E-09	0.004	33.30	
IL-10	rs13252043	T	C	0.114	0.0237	0.9066	1.57E-06	0.002	23.14	

IL-10	rs138727474	T	C	-0.2515	0.0545	0.9761	3.90E-06	0.003	21.30
IL-10	rs141739979	T	G	-0.4544	0.0855	0.9861	1.07E-07	0.006	28.25
IL-10	rs143429938	T	C	0.3535	0.0769	0.9732	4.25E-06	0.007	21.13
IL-10	rs147711004	A	G	1.1354	0.0366	0.0288	1.00E-200	0.072	962.36
IL-10	rs150685845	G	A	0.5561	0.0645	0.9871	6.62E-18	0.008	74.33
IL-10	rs1582763	A	G	-0.1232	0.0149	0.6372	1.19E-16	0.007	68.37
IL-10	rs17125924	G	A	0.1222	0.0246	0.9205	6.62E-07	0.002	24.68
IL-10	rs2830489	T	C	-0.0837	0.0162	0.7038	2.42E-07	0.003	26.69
IL-10	rs34665982	C	T	-0.0967	0.0166	0.4901	5.80E-09	0.005	33.93
IL-10	rs34971488	A	G	0.094	0.0198	0.2346	2.07E-06	0.003	22.54
IL-10	rs35695568	T	G	0.1152	0.0247	0.8986	3.20E-06	0.002	21.75
IL-10	rs3740688	T	G	0.0935	0.0144	0.4573	9.70E-11	0.004	42.16
IL-10	rs383902	T	C	-0.0698	0.0151	0.6402	3.81E-06	0.002	21.37
IL-10	rs3851179	C	T	0.1198	0.0148	0.3708	5.81E-16	0.007	65.52
IL-10	rs6014724	G	A	-0.1319	0.0259	0.9105	3.65E-07	0.003	25.94
IL-10	rs62039712	A	G	0.1528	0.0288	0.1213	1.17E-07	0.005	28.15
IL-10	rs6559689	T	C	0.1585	0.0335	0.9483	2.17E-06	0.002	22.39
IL-10	rs6733839	T	C	0.1693	0.0154	0.6203	4.02E-28	0.014	120.86
IL-10	rs679515	C	T	-0.1508	0.0183	0.828	1.55E-16	0.006	67.90
IL-10	rs72654445	A	G	-0.5425	0.0811	0.0109	2.27E-11	0.006	44.75
IL-10	rs72993825	T	C	-0.1763	0.036	0.9404	9.86E-07	0.003	23.98
IL-10	rs73223431	T	C	0.0936	0.0153	0.6541	8.34E-10	0.004	37.43
IL-10	rs7412	T	C	-0.4673	0.0305	0.9374	6.40E-53	0.026	234.74
IL-10	rs7584040	T	C	0.0862	0.0172	0.8131	5.34E-07	0.002	25.12
IL-10	rs7618668	A	G	-0.1297	0.0258	0.0815	4.95E-07	0.003	25.27
IL-10	rs8111708	G	A	0.0696	0.0151	0.6541	3.95E-06	0.002	21.25

IL-10	rs867230	A	C	0.1333	0.0158	0.5984	3.49E-17	0.009	71.18	
IL-10	rs9381563	T	C	-0.0821	0.0148	0.3221	2.93E-08	0.003	30.77	
IL-10	rs9649710	G	A	0.0676	0.0148	0.3767	4.79E-06	0.002	20.86	
IL-10	rs9787911	C	T	0.0662	0.0144	0.4274	4.39E-06	0.002	21.13	
IL-12	rs1081105	C	A	0.942	0.0436	0.9692	1.51E-103	0.053	466.80	
IL-12	rs111278137	A	G	-0.4735	0.0713	0.0139	3.20E-11	0.006	44.10	
IL-12	rs11168036	G	T	-0.0754	0.0143	0.5169	1.43E-07	0.003	27.80	
IL-12	rs117240937	A	G	-0.3122	0.0672	0.0179	3.35E-06	0.003	21.58	
IL-12	rs11767557	C	T	-0.1028	0.0182	0.2177	1.56E-08	0.004	31.90	
IL-12	rs12151021	G	A	-0.1071	0.0169	0.338	2.56E-10	0.005	40.16	
IL-12	rs12590654	A	G	-0.0906	0.0157	0.3370	8.73E-09	0.004	33.30	
IL-12	rs13252043	T	C	0.114	0.0237	0.9066	1.57E-06	0.002	23.14	
IL-12	rs138727474	T	C	-0.2515	0.0545	0.9761	3.90E-06	0.003	21.30	
IL-12	rs141739979	T	G	-0.4544	0.0855	0.9861	1.07E-07	0.006	28.25	
IL-12	rs143429938	T	C	0.3535	0.0769	0.9732	4.25E-06	0.007	21.13	
IL-12	rs147711004	A	G	1.1354	0.0366	0.0288	1.00E-200	0.072	962.36	100%
IL-12	rs150685845	G	A	0.5561	0.0645	0.9871	6.62E-18	0.008	74.33	
IL-12	rs1582763	A	G	-0.1232	0.0149	0.6372	1.19E-16	0.007	68.37	
IL-12	rs17125924	G	A	0.1222	0.0246	0.9205	6.62E-07	0.002	24.68	
IL-12	rs2830489	T	C	-0.0837	0.0162	0.7038	2.42E-07	0.003	26.69	
IL-12	rs34665982	C	T	-0.0967	0.0166	0.4901	5.80E-09	0.005	33.93	
IL-12	rs34971488	A	G	0.094	0.0198	0.2346	2.07E-06	0.003	22.54	
IL-12	rs35695568	T	G	0.1152	0.0247	0.8986	3.20E-06	0.002	21.75	
IL-12	rs3740688	T	G	0.0935	0.0144	0.4573	9.70E-11	0.004	42.16	
IL-12	rs383902	T	C	-0.0698	0.0151	0.6402	3.81E-06	0.002	21.37	
IL-12	rs3851179	C	T	0.1198	0.0148	0.3708	5.81E-16	0.007	65.52	

IL-12	rs6014724	G	A	-0.1319	0.0259	0.9105	3.65E-07	0.003	25.94	
IL-12	rs62039712	A	G	0.1528	0.0288	0.1213	1.17E-07	0.005	28.15	
IL-12	rs6559689	T	C	0.1585	0.0335	0.9483	2.17E-06	0.002	22.39	
IL-12	rs6733839	T	C	0.1693	0.0154	0.6203	4.02E-28	0.014	120.86	
IL-12	rs679515	C	T	-0.1508	0.0183	0.828	1.55E-16	0.006	67.90	
IL-12	rs72654445	A	G	-0.5425	0.0811	0.0109	2.27E-11	0.006	44.75	
IL-12	rs72993825	T	C	-0.1763	0.036	0.9404	9.86E-07	0.003	23.98	
IL-12	rs73223431	T	C	0.0936	0.0153	0.6541	8.34E-10	0.004	37.43	
IL-12	rs7412	T	C	-0.4673	0.0305	0.9374	6.40E-53	0.026	234.74	
IL-12	rs7584040	T	C	0.0862	0.0172	0.8131	5.34E-07	0.002	25.12	
IL-12	rs7618668	A	G	-0.1297	0.0258	0.0815	4.95E-07	0.003	25.27	
IL-12	rs8111708	G	A	0.0696	0.0151	0.6541	3.95E-06	0.002	21.25	
IL-12	rs867230	A	C	0.1333	0.0158	0.5984	3.49E-17	0.009	71.18	
IL-12	rs9381563	T	C	-0.0821	0.0148	0.3221	2.93E-08	0.003	30.77	
IL-12	rs9649710	G	A	0.0676	0.0148	0.3767	4.79E-06	0.002	20.86	
IL-12	rs9787911	C	T	0.0662	0.0144	0.4274	4.39E-06	0.002	21.13	
IL-13	rs1081105	C	A	0.942	0.0436	0.9692	1.51E-103	0.053	466.80	
IL-13	rs111278137	A	G	-0.4735	0.0713	0.0139	3.20E-11	0.006	44.10	
IL-13	rs11168036	G	T	-0.0754	0.0143	0.5169	1.43E-07	0.003	27.80	
IL-13	rs11767557	C	T	-0.1028	0.0182	0.2177	1.56E-08	0.004	31.90	
IL-13	rs12151021	G	A	-0.1071	0.0169	0.338	2.56E-10	0.005	40.16	100.00%
IL-13	rs12590654	A	G	-0.0906	0.0157	0.3370	8.73E-09	0.004	33.30	
IL-13	rs13252043	T	C	0.114	0.0237	0.9066	1.57E-06	0.002	23.14	
IL-13	rs138727474	T	C	-0.2515	0.0545	0.9761	3.90E-06	0.003	21.30	
IL-13	rs141739979	T	G	-0.4544	0.0855	0.9861	1.07E-07	0.006	28.25	
IL-13	rs143429938	T	C	0.3535	0.0769	0.9732	4.25E-06	0.007	21.13	

IL-13	rs147711004	A	G	1.1354	0.0366	0.0288	1.00E-200	0.072	962.36
IL-13	rs150685845	G	A	0.5561	0.0645	0.9871	6.62E-18	0.008	74.33
IL-13	rs1582763	A	G	-0.1232	0.0149	0.6372	1.19E-16	0.007	68.37
IL-13	rs17125924	G	A	0.1222	0.0246	0.9205	6.62E-07	0.002	24.68
IL-13	rs2830489	T	C	-0.0837	0.0162	0.7038	2.42E-07	0.003	26.69
IL-13	rs34665982	C	T	-0.0967	0.0166	0.4901	5.80E-09	0.005	33.93
IL-13	rs34971488	A	G	0.094	0.0198	0.2346	2.07E-06	0.003	22.54
IL-13	rs35695568	T	G	0.1152	0.0247	0.8986	3.20E-06	0.002	21.75
IL-13	rs3740688	T	G	0.0935	0.0144	0.4573	9.70E-11	0.004	42.16
IL-13	rs383902	T	C	-0.0698	0.0151	0.6402	3.81E-06	0.002	21.37
IL-13	rs3851179	C	T	0.1198	0.0148	0.3708	5.81E-16	0.007	65.52
IL-13	rs6014724	G	A	-0.1319	0.0259	0.9105	3.65E-07	0.003	25.94
IL-13	rs62039712	A	G	0.1528	0.0288	0.1213	1.17E-07	0.005	28.15
IL-13	rs6559689	T	C	0.1585	0.0335	0.9483	2.17E-06	0.002	22.39
IL-13	rs6733839	T	C	0.1693	0.0154	0.6203	4.02E-28	0.014	120.86
IL-13	rs679515	C	T	-0.1508	0.0183	0.828	1.55E-16	0.006	67.90
IL-13	rs72993825	T	C	-0.1763	0.036	0.9404	9.86E-07	0.003	23.98
IL-13	rs73223431	T	C	0.0936	0.0153	0.6541	8.34E-10	0.004	37.43
IL-13	rs7412	T	C	-0.4673	0.0305	0.9374	6.40E-53	0.026	234.74
IL-13	rs7584040	T	C	0.0862	0.0172	0.8131	5.34E-07	0.002	25.12
IL-13	rs7618668	A	G	-0.1297	0.0258	0.0815	4.95E-07	0.003	25.27
IL-13	rs8111708	G	A	0.0696	0.0151	0.6541	3.95E-06	0.002	21.25
IL-13	rs867230	A	C	0.1333	0.0158	0.5984	3.49E-17	0.009	71.18
IL-13	rs9381563	T	C	-0.0821	0.0148	0.3221	2.93E-08	0.003	30.77
IL-13	rs9649710	G	A	0.0676	0.0148	0.3767	4.79E-06	0.002	20.86
IL-13	rs9787911	C	T	0.0662	0.0144	0.4274	4.39E-06	0.002	21.13

IL-16	rs1081105	C	A	0.942	0.0436	0.9692	1.51E-103	0.053	466.80		
IL-16	rs111278137	A	G	-0.4735	0.0713	0.0139	3.20E-11	0.006	44.10		
IL-16	rs11168036	G	T	-0.0754	0.0143	0.5169	1.43E-07	0.003	27.80		
IL-16	rs117240937	A	G	-0.3122	0.0672	0.0179	3.35E-06	0.003	21.58		
IL-16	rs11767557	C	T	-0.1028	0.0182	0.2177	1.56E-08	0.004	31.90		
IL-16	rs12151021	G	A	-0.1071	0.0169	0.338	2.56E-10	0.005	40.16		
IL-16	rs12590654	A	G	-0.0906	0.0157	0.3370	8.73E-09	0.004	33.30		
IL-16	rs13252043	T	C	0.114	0.0237	0.9066	1.57E-06	0.002	23.14		
IL-16	rs138727474	T	C	-0.2515	0.0545	0.9761	3.90E-06	0.003	21.30		
IL-16	rs141739979	T	G	-0.4544	0.0855	0.9861	1.07E-07	0.006	28.25		
IL-16	rs143429938	T	C	0.3535	0.0769	0.9732	4.25E-06	0.007	21.13		
IL-16	rs147711004	A	G	1.1354	0.0366	0.0288	1.00E-200	0.072	962.36		
IL-16	rs150685845	G	A	0.5561	0.0645	0.9871	6.62E-18	0.008	74.33	96.40%	
IL-16	rs1582763	A	G	-0.1232	0.0149	0.6372	1.19E-16	0.007	68.37		
IL-16	rs17125924	G	A	0.1222	0.0246	0.9205	6.62E-07	0.002	24.68		
IL-16	rs2830489	T	C	-0.0837	0.0162	0.7038	2.42E-07	0.003	26.69		
IL-16	rs34665982	C	T	-0.0967	0.0166	0.4901	5.80E-09	0.005	33.93		
IL-16	rs34971488	A	G	0.094	0.0198	0.2346	2.07E-06	0.003	22.54		
IL-16	rs35695568	T	G	0.1152	0.0247	0.8986	3.20E-06	0.002	21.75		
IL-16	rs3740688	T	G	0.0935	0.0144	0.4573	9.70E-11	0.004	42.16		
IL-16	rs383902	T	C	-0.0698	0.0151	0.6402	3.81E-06	0.002	21.37		
IL-16	rs3851179	C	T	0.1198	0.0148	0.3708	5.81E-16	0.007	65.52		
IL-16	rs6014724	G	A	-0.1319	0.0259	0.9105	3.65E-07	0.003	25.94		
IL-16	rs62039712	A	G	0.1528	0.0288	0.1213	1.17E-07	0.005	28.15		
IL-16	rs6559689	T	C	0.1585	0.0335	0.9483	2.17E-06	0.002	22.39		
IL-16	rs6733839	T	C	0.1693	0.0154	0.6203	4.02E-28	0.014	120.86		

IL-16	rs679515	C	T	-0.1508	0.0183	0.828	1.55E-16	0.006	67.90	
IL-16	rs72654445	A	G	-0.5425	0.0811	0.0109	2.27E-11	0.006	44.75	
IL-16	rs72993825	T	C	-0.1763	0.036	0.9404	9.86E-07	0.003	23.98	
IL-16	rs73223431	T	C	0.0936	0.0153	0.6541	8.34E-10	0.004	37.43	
IL-16	rs7412	T	C	-0.4673	0.0305	0.9374	6.40E-53	0.026	234.74	
IL-16	rs7584040	T	C	0.0862	0.0172	0.8131	5.34E-07	0.002	25.12	
IL-16	rs7618668	A	G	-0.1297	0.0258	0.0815	4.95E-07	0.003	25.27	
IL-16	rs8111708	G	A	0.0696	0.0151	0.6541	3.95E-06	0.002	21.25	
IL-16	rs867230	A	C	0.1333	0.0158	0.5984	3.49E-17	0.009	71.18	
IL-16	rs9381563	T	C	-0.0821	0.0148	0.3221	2.93E-08	0.003	30.77	
IL-16	rs9649710	G	A	0.0676	0.0148	0.3767	4.79E-06	0.002	20.86	
IL-16	rs9787911	C	T	0.0662	0.0144	0.4274	4.39E-06	0.002	21.13	
IL-17	rs1081105	C	A	0.942	0.0436	0.9692	1.51E-103	0.053	466.80	
IL-17	rs111278137	A	G	-0.4735	0.0713	0.0139	3.20E-11	0.006	44.10	
IL-17	rs11168036	G	T	-0.0754	0.0143	0.5169	1.43E-07	0.003	27.80	
IL-17	rs117240937	A	G	-0.3122	0.0672	0.0179	3.35E-06	0.003	21.58	
IL-17	rs11767557	C	T	-0.1028	0.0182	0.2177	1.56E-08	0.004	31.90	
IL-17	rs12151021	G	A	-0.1071	0.0169	0.338	2.56E-10	0.005	40.16	
IL-17	rs12590654	A	G	-0.0906	0.0157	0.3370	8.73E-09	0.004	33.30	96.40%
IL-17	rs13252043	T	C	0.114	0.0237	0.9066	1.57E-06	0.002	23.14	
IL-17	rs138727474	T	C	-0.2515	0.0545	0.9761	3.90E-06	0.003	21.30	
IL-17	rs141739979	T	G	-0.4544	0.0855	0.9861	1.07E-07	0.006	28.25	
IL-17	rs143429938	T	C	0.3535	0.0769	0.9732	4.25E-06	0.007	21.13	
IL-17	rs147711004	A	G	1.1354	0.0366	0.0288	1.00E-200	0.072	962.36	
IL-17	rs150685845	G	A	0.5561	0.0645	0.9871	6.62E-18	0.008	74.33	
IL-17	rs1582763	A	G	-0.1232	0.0149	0.6372	1.19E-16	0.007	68.37	

IL-17	rs17125924	G	A	0.1222	0.0246	0.9205	6.62E-07	0.002	24.68	
IL-17	rs2830489	T	C	-0.0837	0.0162	0.7038	2.42E-07	0.003	26.69	
IL-17	rs34665982	C	T	-0.0967	0.0166	0.4901	5.80E-09	0.005	33.93	
IL-17	rs34971488	A	G	0.094	0.0198	0.2346	2.07E-06	0.003	22.54	
IL-17	rs35695568	T	G	0.1152	0.0247	0.8986	3.20E-06	0.002	21.75	
IL-17	rs3740688	T	G	0.0935	0.0144	0.4573	9.70E-11	0.004	42.16	
IL-17	rs383902	T	C	-0.0698	0.0151	0.6402	3.81E-06	0.002	21.37	
IL-17	rs3851179	C	T	0.1198	0.0148	0.3708	5.81E-16	0.007	65.52	
IL-17	rs6014724	G	A	-0.1319	0.0259	0.9105	3.65E-07	0.003	25.94	
IL-17	rs62039712	A	G	0.1528	0.0288	0.1213	1.17E-07	0.005	28.15	
IL-17	rs6559689	T	C	0.1585	0.0335	0.9483	2.17E-06	0.002	22.39	
IL-17	rs6733839	T	C	0.1693	0.0154	0.6203	4.02E-28	0.014	120.86	
IL-17	rs679515	C	T	-0.1508	0.0183	0.828	1.55E-16	0.006	67.90	
IL-17	rs72654445	A	G	-0.5425	0.0811	0.0109	2.27E-11	0.006	44.75	
IL-17	rs72993825	T	C	-0.1763	0.036	0.9404	9.86E-07	0.003	23.98	
IL-17	rs73223431	T	C	0.0936	0.0153	0.6541	8.34E-10	0.004	37.43	
IL-17	rs7412	T	C	-0.4673	0.0305	0.9374	6.40E-53	0.026	234.74	
IL-17	rs7584040	T	C	0.0862	0.0172	0.8131	5.34E-07	0.002	25.12	
IL-17	rs7618668	A	G	-0.1297	0.0258	0.0815	4.95E-07	0.003	25.27	
IL-17	rs8111708	G	A	0.0696	0.0151	0.6541	3.95E-06	0.002	21.25	
IL-17	rs867230	A	C	0.1333	0.0158	0.5984	3.49E-17	0.009	71.18	
IL-17	rs9381563	T	C	-0.0821	0.0148	0.3221	2.93E-08	0.003	30.77	
IL-17	rs9649710	G	A	0.0676	0.0148	0.3767	4.79E-06	0.002	20.86	
IL-17	rs9787911	C	T	0.0662	0.0144	0.4274	4.39E-06	0.002	21.13	
IL-18	rs1081105	C	A	0.942	0.0436	0.9692	1.51E-103	0.053	466.80	100.00%
IL-18	rs111278137	A	G	-0.4735	0.0713	0.0139	3.20E-11	0.006	44.10	

IL-18	rs11168036	G	T	-0.0754	0.0143	0.5169	1.43E-07	0.003	27.80
IL-18	rs117240937	A	G	-0.3122	0.0672	0.0179	3.35E-06	0.003	21.58
IL-18	rs11767557	C	T	-0.1028	0.0182	0.2177	1.56E-08	0.004	31.90
IL-18	rs12151021	G	A	-0.1071	0.0169	0.338	2.56E-10	0.005	40.16
IL-18	rs12590654	A	G	-0.0906	0.0157	0.3370	8.73E-09	0.004	33.30
IL-18	rs13252043	T	C	0.114	0.0237	0.9066	1.57E-06	0.002	23.14
IL-18	rs138727474	T	C	-0.2515	0.0545	0.9761	3.90E-06	0.003	21.30
IL-18	rs141739979	T	G	-0.4544	0.0855	0.9861	1.07E-07	0.006	28.25
IL-18	rs143429938	T	C	0.3535	0.0769	0.9732	4.25E-06	0.007	21.13
IL-18	rs147711004	A	G	1.1354	0.0366	0.0288	1.00E-200	0.072	962.36
IL-18	rs150685845	G	A	0.5561	0.0645	0.9871	6.62E-18	0.008	74.33
IL-18	rs1582763	A	G	-0.1232	0.0149	0.6372	1.19E-16	0.007	68.37
IL-18	rs17125924	G	A	0.1222	0.0246	0.9205	6.62E-07	0.002	24.68
IL-18	rs2830489	T	C	-0.0837	0.0162	0.7038	2.42E-07	0.003	26.69
IL-18	rs34665982	C	T	-0.0967	0.0166	0.4901	5.80E-09	0.005	33.93
IL-18	rs34971488	A	G	0.094	0.0198	0.2346	2.07E-06	0.003	22.54
IL-18	rs35695568	T	G	0.1152	0.0247	0.8986	3.20E-06	0.002	21.75
IL-18	rs3740688	T	G	0.0935	0.0144	0.4573	9.70E-11	0.004	42.16
IL-18	rs383902	T	C	-0.0698	0.0151	0.6402	3.81E-06	0.002	21.37
IL-18	rs3851179	C	T	0.1198	0.0148	0.3708	5.81E-16	0.007	65.52
IL-18	rs6014724	G	A	-0.1319	0.0259	0.9105	3.65E-07	0.003	25.94
IL-18	rs62039712	A	G	0.1528	0.0288	0.1213	1.17E-07	0.005	28.15
IL-18	rs6559689	T	C	0.1585	0.0335	0.9483	2.17E-06	0.002	22.39
IL-18	rs6733839	T	C	0.1693	0.0154	0.6203	4.02E-28	0.014	120.86
IL-18	rs679515	C	T	-0.1508	0.0183	0.828	1.55E-16	0.006	67.90
IL-18	rs72654445	A	G	-0.5425	0.0811	0.0109	2.27E-11	0.006	44.75

IL-18	rs72993825	T	C	-0.1763	0.036	0.9404	9.86E-07	0.003	23.98	
IL-18	rs73223431	T	C	0.0936	0.0153	0.6541	8.34E-10	0.004	37.43	
IL-18	rs7412	T	C	-0.4673	0.0305	0.9374	6.40E-53	0.026	234.74	
IL-18	rs7584040	T	C	0.0862	0.0172	0.8131	5.34E-07	0.002	25.12	
IL-18	rs7618668	A	G	-0.1297	0.0258	0.0815	4.95E-07	0.003	25.27	
IL-18	rs8111708	G	A	0.0696	0.0151	0.6541	3.95E-06	0.002	21.25	
IL-18	rs867230	A	C	0.1333	0.0158	0.5984	3.49E-17	0.009	71.18	
IL-18	rs9381563	T	C	-0.0821	0.0148	0.3221	2.93E-08	0.003	30.77	
IL-18	rs9649710	G	A	0.0676	0.0148	0.3767	4.79E-06	0.002	20.86	
IL-18	rs9787911	C	T	0.0662	0.0144	0.4274	4.39E-06	0.002	21.13	
IL-1B	rs1081105	C	A	0.942	0.0436	0.9692	1.51E-103	0.053	466.80	
IL-1B	rs111278137	A	G	-0.4735	0.0713	0.0139	3.20E-11	0.006	44.10	
IL-1B	rs11168036	G	T	-0.0754	0.0143	0.5169	1.43E-07	0.003	27.80	
IL-1B	rs117240937	A	G	-0.3122	0.0672	0.0179	3.35E-06	0.003	21.58	
IL-1B	rs11767557	C	T	-0.1028	0.0182	0.2177	1.56E-08	0.004	31.90	
IL-1B	rs12151021	G	A	-0.1071	0.0169	0.338	2.56E-10	0.005	40.16	
IL-1B	rs12590654	A	G	-0.0906	0.0157	0.3370	8.73E-09	0.004	33.30	
IL-1B	rs13252043	T	C	0.114	0.0237	0.9066	1.57E-06	0.002	23.14	
IL-1B	rs138727474	T	C	-0.2515	0.0545	0.9761	3.90E-06	0.003	21.30	100.00%
IL-1B	rs141739979	T	G	-0.4544	0.0855	0.9861	1.07E-07	0.006	28.25	
IL-1B	rs143429938	T	C	0.3535	0.0769	0.9732	4.25E-06	0.007	21.13	
IL-1B	rs147711004	A	G	1.1354	0.0366	0.0288	1.00E-200	0.072	962.36	
IL-1B	rs150685845	G	A	0.5561	0.0645	0.9871	6.62E-18	0.008	74.33	
IL-1B	rs1582763	A	G	-0.1232	0.0149	0.6372	1.19E-16	0.007	68.37	
IL-1B	rs17125924	G	A	0.1222	0.0246	0.9205	6.62E-07	0.002	24.68	
IL-1B	rs2830489	T	C	-0.0837	0.0162	0.7038	2.42E-07	0.003	26.69	

IL-1B	rs34665982	C	T	-0.0967	0.0166	0.4901	5.80E-09	0.005	33.93	
IL-1B	rs34971488	A	G	0.094	0.0198	0.2346	2.07E-06	0.003	22.54	
IL-1B	rs35695568	T	G	0.1152	0.0247	0.8986	3.20E-06	0.002	21.75	
IL-1B	rs3740688	T	G	0.0935	0.0144	0.4573	9.70E-11	0.004	42.16	
IL-1B	rs383902	T	C	-0.0698	0.0151	0.6402	3.81E-06	0.002	21.37	
IL-1B	rs3851179	C	T	0.1198	0.0148	0.3708	5.81E-16	0.007	65.52	
IL-1B	rs6014724	G	A	-0.1319	0.0259	0.9105	3.65E-07	0.003	25.94	
IL-1B	rs62039712	A	G	0.1528	0.0288	0.1213	1.17E-07	0.005	28.15	
IL-1B	rs6559689	T	C	0.1585	0.0335	0.9483	2.17E-06	0.002	22.39	
IL-1B	rs6733839	T	C	0.1693	0.0154	0.6203	4.02E-28	0.014	120.86	
IL-1B	rs679515	C	T	-0.1508	0.0183	0.828	1.55E-16	0.006	67.90	
IL-1B	rs72993825	T	C	-0.1763	0.036	0.9404	9.86E-07	0.003	23.98	
IL-1B	rs73223431	T	C	0.0936	0.0153	0.6541	8.34E-10	0.004	37.43	
IL-1B	rs7412	T	C	-0.4673	0.0305	0.9374	6.40E-53	0.026	234.74	
IL-1B	rs7584040	T	C	0.0862	0.0172	0.8131	5.34E-07	0.002	25.12	
IL-1B	rs7618668	A	G	-0.1297	0.0258	0.0815	4.95E-07	0.003	25.27	
IL-1B	rs8111708	G	A	0.0696	0.0151	0.6541	3.95E-06	0.002	21.25	
IL-1B	rs867230	A	C	0.1333	0.0158	0.5984	3.49E-17	0.009	71.18	
IL-1B	rs9381563	T	C	-0.0821	0.0148	0.3221	2.93E-08	0.003	30.77	
IL-1B	rs9649710	G	A	0.0676	0.0148	0.3767	4.79E-06	0.002	20.86	
IL-1B	rs9787911	C	T	0.0662	0.0144	0.4274	4.39E-06	0.002	21.13	
IL-1RA	rs1081105	C	A	0.942	0.0436	0.9692	1.51E-103	0.053	466.80	
IL-1RA	rs111278137	A	G	-0.4735	0.0713	0.0139	3.20E-11	0.006	44.10	
IL-1RA	rs11168036	G	T	-0.0754	0.0143	0.5169	1.43E-07	0.003	27.80	97.80%
IL-1RA	rs11767557	C	T	-0.1028	0.0182	0.2177	1.56E-08	0.004	31.90	
IL-1RA	rs12151021	G	A	-0.1071	0.0169	0.338	2.56E-10	0.005	40.16	

IL-1RA	rs12590654	A	G	-0.0906	0.0157	0.3370	8.73E-09	0.004	33.30
IL-1RA	rs13252043	T	C	0.114	0.0237	0.9066	1.57E-06	0.002	23.14
IL-1RA	rs138727474	T	C	-0.2515	0.0545	0.9761	3.90E-06	0.003	21.30
IL-1RA	rs141739979	T	G	-0.4544	0.0855	0.9861	1.07E-07	0.006	28.25
IL-1RA	rs143429938	T	C	0.3535	0.0769	0.9732	4.25E-06	0.007	21.13
IL-1RA	rs147711004	A	G	1.1354	0.0366	0.0288	1.00E-200	0.072	962.36
IL-1RA	rs150685845	G	A	0.5561	0.0645	0.9871	6.62E-18	0.008	74.33
IL-1RA	rs1582763	A	G	-0.1232	0.0149	0.6372	1.19E-16	0.007	68.37
IL-1RA	rs17125924	G	A	0.1222	0.0246	0.9205	6.62E-07	0.002	24.68
IL-1RA	rs2830489	T	C	-0.0837	0.0162	0.7038	2.42E-07	0.003	26.69
IL-1RA	rs34665982	C	T	-0.0967	0.0166	0.4901	5.80E-09	0.005	33.93
IL-1RA	rs34971488	A	G	0.094	0.0198	0.2346	2.07E-06	0.003	22.54
IL-1RA	rs35695568	T	G	0.1152	0.0247	0.8986	3.20E-06	0.002	21.75
IL-1RA	rs3740688	T	G	0.0935	0.0144	0.4573	9.70E-11	0.004	42.16
IL-1RA	rs383902	T	C	-0.0698	0.0151	0.6402	3.81E-06	0.002	21.37
IL-1RA	rs3851179	C	T	0.1198	0.0148	0.3708	5.81E-16	0.007	65.52
IL-1RA	rs6014724	G	A	-0.1319	0.0259	0.9105	3.65E-07	0.003	25.94
IL-1RA	rs62039712	A	G	0.1528	0.0288	0.1213	1.17E-07	0.005	28.15
IL-1RA	rs6559689	T	C	0.1585	0.0335	0.9483	2.17E-06	0.002	22.39
IL-1RA	rs6733839	T	C	0.1693	0.0154	0.6203	4.02E-28	0.014	120.86
IL-1RA	rs679515	C	T	-0.1508	0.0183	0.828	1.55E-16	0.006	67.90
IL-1RA	rs72993825	T	C	-0.1763	0.036	0.9404	9.86E-07	0.003	23.98
IL-1RA	rs73223431	T	C	0.0936	0.0153	0.6541	8.34E-10	0.004	37.43
IL-1RA	rs7412	T	C	-0.4673	0.0305	0.9374	6.40E-53	0.026	234.74
IL-1RA	rs7584040	T	C	0.0862	0.0172	0.8131	5.34E-07	0.002	25.12
IL-1RA	rs7618668	A	G	-0.1297	0.0258	0.0815	4.95E-07	0.003	25.27

IL-1RA	rs8111708	G	A	0.0696	0.0151	0.6541	3.95E-06	0.002	21.25	
IL-1RA	rs867230	A	C	0.1333	0.0158	0.5984	3.49E-17	0.009	71.18	
IL-1RA	rs9381563	T	C	-0.0821	0.0148	0.3221	2.93E-08	0.003	30.77	
IL-1RA	rs9649710	G	A	0.0676	0.0148	0.3767	4.79E-06	0.002	20.86	
IL-1RA	rs9787911	C	T	0.0662	0.0144	0.4274	4.39E-06	0.002	21.13	
IL-2	rs1081105	C	A	0.942	0.0436	0.9692	1.51E-103	0.053	466.80	
IL-2	rs111278137	A	G	-0.4735	0.0713	0.0139	3.20E-11	0.006	44.10	
IL-2	rs11168036	G	T	-0.0754	0.0143	0.5169	1.43E-07	0.003	27.80	
IL-2	rs11767557	C	T	-0.1028	0.0182	0.2177	1.56E-08	0.004	31.90	
IL-2	rs12151021	G	A	-0.1071	0.0169	0.338	2.56E-10	0.005	40.16	
IL-2	rs12590654	A	G	-0.0906	0.0157	0.3370	8.73E-09	0.004	33.30	
IL-2	rs13252043	T	C	0.114	0.0237	0.9066	1.57E-06	0.002	23.14	
IL-2	rs138727474	T	C	-0.2515	0.0545	0.9761	3.90E-06	0.003	21.30	
IL-2	rs141739979	T	G	-0.4544	0.0855	0.9861	1.07E-07	0.006	28.25	
IL-2	rs143429938	T	C	0.3535	0.0769	0.9732	4.25E-06	0.007	21.13	
IL-2	rs147711004	A	G	1.1354	0.0366	0.0288	1.00E-200	0.072	962.36	100.00%
IL-2	rs150685845	G	A	0.5561	0.0645	0.9871	6.62E-18	0.008	74.33	
IL-2	rs1582763	A	G	-0.1232	0.0149	0.6372	1.19E-16	0.007	68.37	
IL-2	rs17125924	G	A	0.1222	0.0246	0.9205	6.62E-07	0.002	24.68	
IL-2	rs2830489	T	C	-0.0837	0.0162	0.7038	2.42E-07	0.003	26.69	
IL-2	rs34665982	C	T	-0.0967	0.0166	0.4901	5.80E-09	0.005	33.93	
IL-2	rs34971488	A	G	0.094	0.0198	0.2346	2.07E-06	0.003	22.54	
IL-2	rs35695568	T	G	0.1152	0.0247	0.8986	3.20E-06	0.002	21.75	
IL-2	rs3740688	T	G	0.0935	0.0144	0.4573	9.70E-11	0.004	42.16	
IL-2	rs383902	T	C	-0.0698	0.0151	0.6402	3.81E-06	0.002	21.37	
IL-2	rs3851179	C	T	0.1198	0.0148	0.3708	5.81E-16	0.007	65.52	

IL-2	rs6014724	G	A	-0.1319	0.0259	0.9105	3.65E-07	0.003	25.94	
IL-2	rs62039712	A	G	0.1528	0.0288	0.1213	1.17E-07	0.005	28.15	
IL-2	rs6559689	T	C	0.1585	0.0335	0.9483	2.17E-06	0.002	22.39	
IL-2	rs6733839	T	C	0.1693	0.0154	0.6203	4.02E-28	0.014	120.86	
IL-2	rs679515	C	T	-0.1508	0.0183	0.828	1.55E-16	0.006	67.90	
IL-2	rs72993825	T	C	-0.1763	0.036	0.9404	9.86E-07	0.003	23.98	
IL-2	rs73223431	T	C	0.0936	0.0153	0.6541	8.34E-10	0.004	37.43	
IL-2	rs7412	T	C	-0.4673	0.0305	0.9374	6.40E-53	0.026	234.74	
IL-2	rs7584040	T	C	0.0862	0.0172	0.8131	5.34E-07	0.002	25.12	
IL-2	rs7618668	A	G	-0.1297	0.0258	0.0815	4.95E-07	0.003	25.27	
IL-2	rs8111708	G	A	0.0696	0.0151	0.6541	3.95E-06	0.002	21.25	
IL-2	rs867230	A	C	0.1333	0.0158	0.5984	3.49E-17	0.009	71.18	
IL-2	rs9381563	T	C	-0.0821	0.0148	0.3221	2.93E-08	0.003	30.77	
IL-2	rs9649710	G	A	0.0676	0.0148	0.3767	4.79E-06	0.002	20.86	
IL-2	rs9787911	C	T	0.0662	0.0144	0.4274	4.39E-06	0.002	21.13	
IL-2RA	rs1081105	C	A	0.942	0.0436	0.9692	1.51E-103	0.053	466.80	
IL-2RA	rs111278137	A	G	-0.4735	0.0713	0.0139	3.20E-11	0.006	44.10	
IL-2RA	rs11168036	G	T	-0.0754	0.0143	0.5169	1.43E-07	0.003	27.80	
IL-2RA	rs11767557	C	T	-0.1028	0.0182	0.2177	1.56E-08	0.004	31.90	
IL-2RA	rs12151021	G	A	-0.1071	0.0169	0.338	2.56E-10	0.005	40.16	
IL-2RA	rs12590654	A	G	-0.0906	0.0157	0.3370	8.73E-09	0.004	33.30	98.40%
IL-2RA	rs13252043	T	C	0.114	0.0237	0.9066	1.57E-06	0.002	23.14	
IL-2RA	rs138727474	T	C	-0.2515	0.0545	0.9761	3.90E-06	0.003	21.30	
IL-2RA	rs141739979	T	G	-0.4544	0.0855	0.9861	1.07E-07	0.006	28.25	
IL-2RA	rs143429938	T	C	0.3535	0.0769	0.9732	4.25E-06	0.007	21.13	
IL-2RA	rs147711004	A	G	1.1354	0.0366	0.0288	1.00E-200	0.072	962.36	

IL-2RA	rs150685845	G	A	0.5561	0.0645	0.9871	6.62E-18	0.008	74.33	
IL-2RA	rs1582763	A	G	-0.1232	0.0149	0.6372	1.19E-16	0.007	68.37	
IL-2RA	rs17125924	G	A	0.1222	0.0246	0.9205	6.62E-07	0.002	24.68	
IL-2RA	rs2830489	T	C	-0.0837	0.0162	0.7038	2.42E-07	0.003	26.69	
IL-2RA	rs34665982	C	T	-0.0967	0.0166	0.4901	5.80E-09	0.005	33.93	
IL-2RA	rs34971488	A	G	0.094	0.0198	0.2346	2.07E-06	0.003	22.54	
IL-2RA	rs35695568	T	G	0.1152	0.0247	0.8986	3.20E-06	0.002	21.75	
IL-2RA	rs3740688	T	G	0.0935	0.0144	0.4573	9.70E-11	0.004	42.16	
IL-2RA	rs383902	T	C	-0.0698	0.0151	0.6402	3.81E-06	0.002	21.37	
IL-2RA	rs3851179	C	T	0.1198	0.0148	0.3708	5.81E-16	0.007	65.52	
IL-2RA	rs6014724	G	A	-0.1319	0.0259	0.9105	3.65E-07	0.003	25.94	
IL-2RA	rs62039712	A	G	0.1528	0.0288	0.1213	1.17E-07	0.005	28.15	
IL-2RA	rs6559689	T	C	0.1585	0.0335	0.9483	2.17E-06	0.002	22.39	
IL-2RA	rs6733839	T	C	0.1693	0.0154	0.6203	4.02E-28	0.014	120.86	
IL-2RA	rs679515	C	T	-0.1508	0.0183	0.828	1.55E-16	0.006	67.90	
IL-2RA	rs72993825	T	C	-0.1763	0.036	0.9404	9.86E-07	0.003	23.98	
IL-2RA	rs73223431	T	C	0.0936	0.0153	0.6541	8.34E-10	0.004	37.43	
IL-2RA	rs7412	T	C	-0.4673	0.0305	0.9374	6.40E-53	0.026	234.74	
IL-2RA	rs7584040	T	C	0.0862	0.0172	0.8131	5.34E-07	0.002	25.12	
IL-2RA	rs7618668	A	G	-0.1297	0.0258	0.0815	4.95E-07	0.003	25.27	
IL-2RA	rs8111708	G	A	0.0696	0.0151	0.6541	3.95E-06	0.002	21.25	
IL-2RA	rs867230	A	C	0.1333	0.0158	0.5984	3.49E-17	0.009	71.18	
IL-2RA	rs9381563	T	C	-0.0821	0.0148	0.3221	2.93E-08	0.003	30.77	
IL-2RA	rs9649710	G	A	0.0676	0.0148	0.3767	4.79E-06	0.002	20.86	
IL-2RA	rs9787911	C	T	0.0662	0.0144	0.4274	4.39E-06	0.002	21.13	
IL-4	rs1081105	C	A	0.942	0.0436	0.9692	1.51E-103	0.053	466.80	83.20%

IL-4	rs111278137	A	G	-0.4735	0.0713	0.0139	3.20E-11	0.006	44.10
IL-4	rs11168036	G	T	-0.0754	0.0143	0.5169	1.43E-07	0.003	27.80
IL-4	rs117240937	A	G	-0.3122	0.0672	0.0179	3.35E-06	0.003	21.58
IL-4	rs11767557	C	T	-0.1028	0.0182	0.2177	1.56E-08	0.004	31.90
IL-4	rs12151021	G	A	-0.1071	0.0169	0.338	2.56E-10	0.005	40.16
IL-4	rs12590654	A	G	-0.0906	0.0157	0.3370	8.73E-09	0.004	33.30
IL-4	rs13252043	T	C	0.114	0.0237	0.9066	1.57E-06	0.002	23.14
IL-4	rs138727474	T	C	-0.2515	0.0545	0.9761	3.90E-06	0.003	21.30
IL-4	rs141739979	T	G	-0.4544	0.0855	0.9861	1.07E-07	0.006	28.25
IL-4	rs143429938	T	C	0.3535	0.0769	0.9732	4.25E-06	0.007	21.13
IL-4	rs147711004	A	G	1.1354	0.0366	0.0288	1.00E-200	0.072	962.36
IL-4	rs150685845	G	A	0.5561	0.0645	0.9871	6.62E-18	0.008	74.33
IL-4	rs1582763	A	G	-0.1232	0.0149	0.6372	1.19E-16	0.007	68.37
IL-4	rs17125924	G	A	0.1222	0.0246	0.9205	6.62E-07	0.002	24.68
IL-4	rs2830489	T	C	-0.0837	0.0162	0.7038	2.42E-07	0.003	26.69
IL-4	rs34665982	C	T	-0.0967	0.0166	0.4901	5.80E-09	0.005	33.93
IL-4	rs34971488	A	G	0.094	0.0198	0.2346	2.07E-06	0.003	22.54
IL-4	rs35695568	T	G	0.1152	0.0247	0.8986	3.20E-06	0.002	21.75
IL-4	rs3740688	T	G	0.0935	0.0144	0.4573	9.70E-11	0.004	42.16
IL-4	rs383902	T	C	-0.0698	0.0151	0.6402	3.81E-06	0.002	21.37
IL-4	rs3851179	C	T	0.1198	0.0148	0.3708	5.81E-16	0.007	65.52
IL-4	rs6014724	G	A	-0.1319	0.0259	0.9105	3.65E-07	0.003	25.94
IL-4	rs62039712	A	G	0.1528	0.0288	0.1213	1.17E-07	0.005	28.15
IL-4	rs6559689	T	C	0.1585	0.0335	0.9483	2.17E-06	0.002	22.39
IL-4	rs6733839	T	C	0.1693	0.0154	0.6203	4.02E-28	0.014	120.86
IL-4	rs679515	C	T	-0.1508	0.0183	0.828	1.55E-16	0.006	67.90

IL-4	rs72654445	A	G	-0.5425	0.0811	0.0109	2.27E-11	0.006	44.75	
IL-4	rs72993825	T	C	-0.1763	0.036	0.9404	9.86E-07	0.003	23.98	
IL-4	rs73223431	T	C	0.0936	0.0153	0.6541	8.34E-10	0.004	37.43	
IL-4	rs7412	T	C	-0.4673	0.0305	0.9374	6.40E-53	0.026	234.74	
IL-4	rs7584040	T	C	0.0862	0.0172	0.8131	5.34E-07	0.002	25.12	
IL-4	rs7618668	A	G	-0.1297	0.0258	0.0815	4.95E-07	0.003	25.27	
IL-4	rs8111708	G	A	0.0696	0.0151	0.6541	3.95E-06	0.002	21.25	
IL-4	rs867230	A	C	0.1333	0.0158	0.5984	3.49E-17	0.009	71.18	
IL-4	rs9381563	T	C	-0.0821	0.0148	0.3221	2.93E-08	0.003	30.77	
IL-4	rs9649710	G	A	0.0676	0.0148	0.3767	4.79E-06	0.002	20.86	
IL-4	rs9787911	C	T	0.0662	0.0144	0.4274	4.39E-06	0.002	21.13	
IL-5	rs1081105	C	A	0.942	0.0436	0.9692	1.51E-103	0.053	466.80	
IL-5	rs111278137	A	G	-0.4735	0.0713	0.0139	3.20E-11	0.006	44.10	
IL-5	rs11168036	G	T	-0.0754	0.0143	0.5169	1.43E-07	0.003	27.80	
IL-5	rs117240937	A	G	-0.3122	0.0672	0.0179	3.35E-06	0.003	21.58	
IL-5	rs11767557	C	T	-0.1028	0.0182	0.2177	1.56E-08	0.004	31.90	
IL-5	rs12151021	G	A	-0.1071	0.0169	0.338	2.56E-10	0.005	40.16	
IL-5	rs12590654	A	G	-0.0906	0.0157	0.3370	8.73E-09	0.004	33.30	
IL-5	rs13252043	T	C	0.114	0.0237	0.9066	1.57E-06	0.002	23.14	16.00%
IL-5	rs138727474	T	C	-0.2515	0.0545	0.9761	3.90E-06	0.003	21.30	
IL-5	rs141739979	T	G	-0.4544	0.0855	0.9861	1.07E-07	0.006	28.25	
IL-5	rs143429938	T	C	0.3535	0.0769	0.9732	4.25E-06	0.007	21.13	
IL-5	rs147711004	A	G	1.1354	0.0366	0.0288	1.00E-200	0.072	962.36	
IL-5	rs150685845	G	A	0.5561	0.0645	0.9871	6.62E-18	0.008	74.33	
IL-5	rs1582763	A	G	-0.1232	0.0149	0.6372	1.19E-16	0.007	68.37	
IL-5	rs17125924	G	A	0.1222	0.0246	0.9205	6.62E-07	0.002	24.68	

IL-5	rs2830489	T	C	-0.0837	0.0162	0.7038	2.42E-07	0.003	26.69	
IL-5	rs34665982	C	T	-0.0967	0.0166	0.4901	5.80E-09	0.005	33.93	
IL-5	rs34971488	A	G	0.094	0.0198	0.2346	2.07E-06	0.003	22.54	
IL-5	rs35695568	T	G	0.1152	0.0247	0.8986	3.20E-06	0.002	21.75	
IL-5	rs3740688	T	G	0.0935	0.0144	0.4573	9.70E-11	0.004	42.16	
IL-5	rs383902	T	C	-0.0698	0.0151	0.6402	3.81E-06	0.002	21.37	
IL-5	rs3851179	C	T	0.1198	0.0148	0.3708	5.81E-16	0.007	65.52	
IL-5	rs6014724	G	A	-0.1319	0.0259	0.9105	3.65E-07	0.003	25.94	
IL-5	rs62039712	A	G	0.1528	0.0288	0.1213	1.17E-07	0.005	28.15	
IL-5	rs6559689	T	C	0.1585	0.0335	0.9483	2.17E-06	0.002	22.39	
IL-5	rs6733839	T	C	0.1693	0.0154	0.6203	4.02E-28	0.014	120.86	
IL-5	rs679515	C	T	-0.1508	0.0183	0.828	1.55E-16	0.006	67.90	
IL-5	rs72993825	T	C	-0.1763	0.036	0.9404	9.86E-07	0.003	23.98	
IL-5	rs73223431	T	C	0.0936	0.0153	0.6541	8.34E-10	0.004	37.43	
IL-5	rs7412	T	C	-0.4673	0.0305	0.9374	6.40E-53	0.026	234.74	
IL-5	rs7584040	T	C	0.0862	0.0172	0.8131	5.34E-07	0.002	25.12	
IL-5	rs7618668	A	G	-0.1297	0.0258	0.0815	4.95E-07	0.003	25.27	
IL-5	rs8111708	G	A	0.0696	0.0151	0.6541	3.95E-06	0.002	21.25	
IL-5	rs867230	A	C	0.1333	0.0158	0.5984	3.49E-17	0.009	71.18	
IL-5	rs9381563	T	C	-0.0821	0.0148	0.3221	2.93E-08	0.003	30.77	
IL-5	rs9649710	G	A	0.0676	0.0148	0.3767	4.79E-06	0.002	20.86	
IL-5	rs9787911	C	T	0.0662	0.0144	0.4274	4.39E-06	0.002	21.13	
IL-6	rs1081105	C	A	0.942	0.0436	0.9692	1.51E-103	0.053	466.80	
IL-6	rs111278137	A	G	-0.4735	0.0713	0.0139	3.20E-11	0.006	44.10	44.00%
IL-6	rs11168036	G	T	-0.0754	0.0143	0.5169	1.43E-07	0.003	27.80	
IL-6	rs117240937	A	G	-0.3122	0.0672	0.0179	3.35E-06	0.003	21.58	

IL-6	rs11767557	C	T	-0.1028	0.0182	0.2177	1.56E-08	0.004	31.90
IL-6	rs12151021	G	A	-0.1071	0.0169	0.338	2.56E-10	0.005	40.16
IL-6	rs12590654	A	G	-0.0906	0.0157	0.3370	8.73E-09	0.004	33.30
IL-6	rs13252043	T	C	0.114	0.0237	0.9066	1.57E-06	0.002	23.14
IL-6	rs138727474	T	C	-0.2515	0.0545	0.9761	3.90E-06	0.003	21.30
IL-6	rs141739979	T	G	-0.4544	0.0855	0.9861	1.07E-07	0.006	28.25
IL-6	rs143429938	T	C	0.3535	0.0769	0.9732	4.25E-06	0.007	21.13
IL-6	rs147711004	A	G	1.1354	0.0366	0.0288	1.00E-200	0.072	962.36
IL-6	rs150685845	G	A	0.5561	0.0645	0.9871	6.62E-18	0.008	74.33
IL-6	rs1582763	A	G	-0.1232	0.0149	0.6372	1.19E-16	0.007	68.37
IL-6	rs17125924	G	A	0.1222	0.0246	0.9205	6.62E-07	0.002	24.68
IL-6	rs2830489	T	C	-0.0837	0.0162	0.7038	2.42E-07	0.003	26.69
IL-6	rs34665982	C	T	-0.0967	0.0166	0.4901	5.80E-09	0.005	33.93
IL-6	rs34971488	A	G	0.094	0.0198	0.2346	2.07E-06	0.003	22.54
IL-6	rs35695568	T	G	0.1152	0.0247	0.8986	3.20E-06	0.002	21.75
IL-6	rs3740688	T	G	0.0935	0.0144	0.4573	9.70E-11	0.004	42.16
IL-6	rs383902	T	C	-0.0698	0.0151	0.6402	3.81E-06	0.002	21.37
IL-6	rs3851179	C	T	0.1198	0.0148	0.3708	5.81E-16	0.007	65.52
IL-6	rs6014724	G	A	-0.1319	0.0259	0.9105	3.65E-07	0.003	25.94
IL-6	rs62039712	A	G	0.1528	0.0288	0.1213	1.17E-07	0.005	28.15
IL-6	rs6559689	T	C	0.1585	0.0335	0.9483	2.17E-06	0.002	22.39
IL-6	rs6733839	T	C	0.1693	0.0154	0.6203	4.02E-28	0.014	120.86
IL-6	rs679515	C	T	-0.1508	0.0183	0.828	1.55E-16	0.006	67.90
IL-6	rs72654445	A	G	-0.5425	0.0811	0.0109	2.27E-11	0.006	44.75
IL-6	rs72993825	T	C	-0.1763	0.036	0.9404	9.86E-07	0.003	23.98
IL-6	rs73223431	T	C	0.0936	0.0153	0.6541	8.34E-10	0.004	37.43

IL-6	rs7412	T	C	-0.4673	0.0305	0.9374	6.40E-53	0.026	234.74	
IL-6	rs7584040	T	C	0.0862	0.0172	0.8131	5.34E-07	0.002	25.12	
IL-6	rs7618668	A	G	-0.1297	0.0258	0.0815	4.95E-07	0.003	25.27	
IL-6	rs8111708	G	A	0.0696	0.0151	0.6541	3.95E-06	0.002	21.25	
IL-6	rs867230	A	C	0.1333	0.0158	0.5984	3.49E-17	0.009	71.18	
IL-6	rs9381563	T	C	-0.0821	0.0148	0.3221	2.93E-08	0.003	30.77	
IL-6	rs9649710	G	A	0.0676	0.0148	0.3767	4.79E-06	0.002	20.86	
IL-6	rs9787911	C	T	0.0662	0.0144	0.4274	4.39E-06	0.002	21.13	
IL-7	rs1081105	C	A	0.942	0.0436	0.9692	1.51E-103	0.053	466.80	
IL-7	rs111278137	A	G	-0.4735	0.0713	0.0139	3.20E-11	0.006	44.10	
IL-7	rs11168036	G	T	-0.0754	0.0143	0.5169	1.43E-07	0.003	27.80	
IL-7	rs11767557	C	T	-0.1028	0.0182	0.2177	1.56E-08	0.004	31.90	
IL-7	rs12151021	G	A	-0.1071	0.0169	0.338	2.56E-10	0.005	40.16	
IL-7	rs12590654	A	G	-0.0906	0.0157	0.3370	8.73E-09	0.004	33.30	
IL-7	rs13252043	T	C	0.114	0.0237	0.9066	1.57E-06	0.002	23.14	
IL-7	rs138727474	T	C	-0.2515	0.0545	0.9761	3.90E-06	0.003	21.30	
IL-7	rs141739979	T	G	-0.4544	0.0855	0.9861	1.07E-07	0.006	28.25	
IL-7	rs143429938	T	C	0.3535	0.0769	0.9732	4.25E-06	0.007	21.13	100.00%
IL-7	rs147711004	A	G	1.1354	0.0366	0.0288	1.00E-200	0.072	962.36	
IL-7	rs150685845	G	A	0.5561	0.0645	0.9871	6.62E-18	0.008	74.33	
IL-7	rs1582763	A	G	-0.1232	0.0149	0.6372	1.19E-16	0.007	68.37	
IL-7	rs17125924	G	A	0.1222	0.0246	0.9205	6.62E-07	0.002	24.68	
IL-7	rs2830489	T	C	-0.0837	0.0162	0.7038	2.42E-07	0.003	26.69	
IL-7	rs34665982	C	T	-0.0967	0.0166	0.4901	5.80E-09	0.005	33.93	
IL-7	rs34971488	A	G	0.094	0.0198	0.2346	2.07E-06	0.003	22.54	
IL-7	rs35695568	T	G	0.1152	0.0247	0.8986	3.20E-06	0.002	21.75	

IL-7	rs3740688	T	G	0.0935	0.0144	0.4573	9.70E-11	0.004	42.16	
IL-7	rs383902	T	C	-0.0698	0.0151	0.6402	3.81E-06	0.002	21.37	
IL-7	rs3851179	C	T	0.1198	0.0148	0.3708	5.81E-16	0.007	65.52	
IL-7	rs6014724	G	A	-0.1319	0.0259	0.9105	3.65E-07	0.003	25.94	
IL-7	rs62039712	A	G	0.1528	0.0288	0.1213	1.17E-07	0.005	28.15	
IL-7	rs6559689	T	C	0.1585	0.0335	0.9483	2.17E-06	0.002	22.39	
IL-7	rs6733839	T	C	0.1693	0.0154	0.6203	4.02E-28	0.014	120.86	
IL-7	rs679515	C	T	-0.1508	0.0183	0.828	1.55E-16	0.006	67.90	
IL-7	rs72993825	T	C	-0.1763	0.036	0.9404	9.86E-07	0.003	23.98	
IL-7	rs73223431	T	C	0.0936	0.0153	0.6541	8.34E-10	0.004	37.43	
IL-7	rs7412	T	C	-0.4673	0.0305	0.9374	6.40E-53	0.026	234.74	
IL-7	rs7584040	T	C	0.0862	0.0172	0.8131	5.34E-07	0.002	25.12	
IL-7	rs7618668	A	G	-0.1297	0.0258	0.0815	4.95E-07	0.003	25.27	
IL-7	rs8111708	G	A	0.0696	0.0151	0.6541	3.95E-06	0.002	21.25	
IL-7	rs867230	A	C	0.1333	0.0158	0.5984	3.49E-17	0.009	71.18	
IL-7	rs9381563	T	C	-0.0821	0.0148	0.3221	2.93E-08	0.003	30.77	
IL-7	rs9649710	G	A	0.0676	0.0148	0.3767	4.79E-06	0.002	20.86	
IL-7	rs9787911	C	T	0.0662	0.0144	0.4274	4.39E-06	0.002	21.13	
IL-8	rs1081105	C	A	0.942	0.0436	0.9692	1.51E-103	0.053	466.80	
IL-8	rs111278137	A	G	-0.4735	0.0713	0.0139	3.20E-11	0.006	44.10	
IL-8	rs11168036	G	T	-0.0754	0.0143	0.5169	1.43E-07	0.003	27.80	
IL-8	rs117240937	A	G	-0.3122	0.0672	0.0179	3.35E-06	0.003	21.58	82.50%
IL-8	rs11767557	C	T	-0.1028	0.0182	0.2177	1.56E-08	0.004	31.90	
IL-8	rs12151021	G	A	-0.1071	0.0169	0.338	2.56E-10	0.005	40.16	
IL-8	rs12590654	A	G	-0.0906	0.0157	0.3370	8.73E-09	0.004	33.30	
IL-8	rs13252043	T	C	0.114	0.0237	0.9066	1.57E-06	0.002	23.14	

IL-8	rs138727474	T	C	-0.2515	0.0545	0.9761	3.90E-06	0.003	21.30
IL-8	rs141739979	T	G	-0.4544	0.0855	0.9861	1.07E-07	0.006	28.25
IL-8	rs143429938	T	C	0.3535	0.0769	0.9732	4.25E-06	0.007	21.13
IL-8	rs147711004	A	G	1.1354	0.0366	0.0288	1.00E-200	0.072	962.36
IL-8	rs150685845	G	A	0.5561	0.0645	0.9871	6.62E-18	0.008	74.33
IL-8	rs1582763	A	G	-0.1232	0.0149	0.6372	1.19E-16	0.007	68.37
IL-8	rs17125924	G	A	0.1222	0.0246	0.9205	6.62E-07	0.002	24.68
IL-8	rs2830489	T	C	-0.0837	0.0162	0.7038	2.42E-07	0.003	26.69
IL-8	rs34665982	C	T	-0.0967	0.0166	0.4901	5.80E-09	0.005	33.93
IL-8	rs34971488	A	G	0.094	0.0198	0.2346	2.07E-06	0.003	22.54
IL-8	rs35695568	T	G	0.1152	0.0247	0.8986	3.20E-06	0.002	21.75
IL-8	rs3740688	T	G	0.0935	0.0144	0.4573	9.70E-11	0.004	42.16
IL-8	rs383902	T	C	-0.0698	0.0151	0.6402	3.81E-06	0.002	21.37
IL-8	rs3851179	C	T	0.1198	0.0148	0.3708	5.81E-16	0.007	65.52
IL-8	rs6014724	G	A	-0.1319	0.0259	0.9105	3.65E-07	0.003	25.94
IL-8	rs62039712	A	G	0.1528	0.0288	0.1213	1.17E-07	0.005	28.15
IL-8	rs6559689	T	C	0.1585	0.0335	0.9483	2.17E-06	0.002	22.39
IL-8	rs6733839	T	C	0.1693	0.0154	0.6203	4.02E-28	0.014	120.86
IL-8	rs679515	C	T	-0.1508	0.0183	0.828	1.55E-16	0.006	67.90
IL-8	rs72993825	T	C	-0.1763	0.036	0.9404	9.86E-07	0.003	23.98
IL-8	rs73223431	T	C	0.0936	0.0153	0.6541	8.34E-10	0.004	37.43
IL-8	rs7412	T	C	-0.4673	0.0305	0.9374	6.40E-53	0.026	234.74
IL-8	rs7584040	T	C	0.0862	0.0172	0.8131	5.34E-07	0.002	25.12
IL-8	rs7618668	A	G	-0.1297	0.0258	0.0815	4.95E-07	0.003	25.27
IL-8	rs8111708	G	A	0.0696	0.0151	0.6541	3.95E-06	0.002	21.25
IL-8	rs867230	A	C	0.1333	0.0158	0.5984	3.49E-17	0.009	71.18

IL-8	rs9381563	T	C	-0.0821	0.0148	0.3221	2.93E-08	0.003	30.77	
IL-8	rs9649710	G	A	0.0676	0.0148	0.3767	4.79E-06	0.002	20.86	
IL-8	rs9787911	C	T	0.0662	0.0144	0.4274	4.39E-06	0.002	21.13	
IL-9	rs1081105	C	A	0.942	0.0436	0.9692	1.51E-103	0.053	466.80	
IL-9	rs111278137	A	G	-0.4735	0.0713	0.0139	3.20E-11	0.006	44.10	
IL-9	rs11168036	G	T	-0.0754	0.0143	0.5169	1.43E-07	0.003	27.80	
IL-9	rs11767557	C	T	-0.1028	0.0182	0.2177	1.56E-08	0.004	31.90	
IL-9	rs12151021	G	A	-0.1071	0.0169	0.338	2.56E-10	0.005	40.16	
IL-9	rs12590654	A	G	-0.0906	0.0157	0.3370	8.73E-09	0.004	33.30	
IL-9	rs13252043	T	C	0.114	0.0237	0.9066	1.57E-06	0.002	23.14	
IL-9	rs138727474	T	C	-0.2515	0.0545	0.9761	3.90E-06	0.003	21.30	
IL-9	rs141739979	T	G	-0.4544	0.0855	0.9861	1.07E-07	0.006	28.25	
IL-9	rs143429938	T	C	0.3535	0.0769	0.9732	4.25E-06	0.007	21.13	
IL-9	rs147711004	A	G	1.1354	0.0366	0.0288	1.00E-200	0.072	962.36	
IL-9	rs150685845	G	A	0.5561	0.0645	0.9871	6.62E-18	0.008	74.33	69.30%
IL-9	rs1582763	A	G	-0.1232	0.0149	0.6372	1.19E-16	0.007	68.37	
IL-9	rs17125924	G	A	0.1222	0.0246	0.9205	6.62E-07	0.002	24.68	
IL-9	rs2830489	T	C	-0.0837	0.0162	0.7038	2.42E-07	0.003	26.69	
IL-9	rs34665982	C	T	-0.0967	0.0166	0.4901	5.80E-09	0.005	33.93	
IL-9	rs34971488	A	G	0.094	0.0198	0.2346	2.07E-06	0.003	22.54	
IL-9	rs35695568	T	G	0.1152	0.0247	0.8986	3.20E-06	0.002	21.75	
IL-9	rs3740688	T	G	0.0935	0.0144	0.4573	9.70E-11	0.004	42.16	
IL-9	rs383902	T	C	-0.0698	0.0151	0.6402	3.81E-06	0.002	21.37	
IL-9	rs3851179	C	T	0.1198	0.0148	0.3708	5.81E-16	0.007	65.52	
IL-9	rs6014724	G	A	-0.1319	0.0259	0.9105	3.65E-07	0.003	25.94	
IL-9	rs62039712	A	G	0.1528	0.0288	0.1213	1.17E-07	0.005	28.15	

IL-9	rs6559689	T	C	0.1585	0.0335	0.9483	2.17E-06	0.002	22.39		
IL-9	rs6733839	T	C	0.1693	0.0154	0.6203	4.02E-28	0.014	120.86		
IL-9	rs679515	C	T	-0.1508	0.0183	0.828	1.55E-16	0.006	67.90		
IL-9	rs72993825	T	C	-0.1763	0.036	0.9404	9.86E-07	0.003	23.98		
IL-9	rs73223431	T	C	0.0936	0.0153	0.6541	8.34E-10	0.004	37.43		
IL-9	rs7412	T	C	-0.4673	0.0305	0.9374	6.40E-53	0.026	234.74		
IL-9	rs7584040	T	C	0.0862	0.0172	0.8131	5.34E-07	0.002	25.12		
IL-9	rs7618668	A	G	-0.1297	0.0258	0.0815	4.95E-07	0.003	25.27		
IL-9	rs8111708	G	A	0.0696	0.0151	0.6541	3.95E-06	0.002	21.25		
IL-9	rs867230	A	C	0.1333	0.0158	0.5984	3.49E-17	0.009	71.18		
IL-9	rs9381563	T	C	-0.0821	0.0148	0.3221	2.93E-08	0.003	30.77		
IL-9	rs9649710	G	A	0.0676	0.0148	0.3767	4.79E-06	0.002	20.86		
IL-9	rs9787911	C	T	0.0662	0.0144	0.4274	4.39E-06	0.002	21.13		
IP-10	rs1081105	C	A	0.942	0.0436	0.9692	1.51E-103	0.053	466.80		
IP-10	rs111278137	A	G	-0.4735	0.0713	0.0139	3.20E-11	0.006	44.10		
IP-10	rs11168036	G	T	-0.0754	0.0143	0.5169	1.43E-07	0.003	27.80		
IP-10	rs11767557	C	T	-0.1028	0.0182	0.2177	1.56E-08	0.004	31.90		
IP-10	rs12151021	G	A	-0.1071	0.0169	0.338	2.56E-10	0.005	40.16		
IP-10	rs12590654	A	G	-0.0906	0.0157	0.3370	8.73E-09	0.004	33.30		
IP-10	rs13252043	T	C	0.114	0.0237	0.9066	1.57E-06	0.002	23.14	100.00%	
IP-10	rs138727474	T	C	-0.2515	0.0545	0.9761	3.90E-06	0.003	21.30		
IP-10	rs141739979	T	G	-0.4544	0.0855	0.9861	1.07E-07	0.006	28.25		
IP-10	rs143429938	T	C	0.3535	0.0769	0.9732	4.25E-06	0.007	21.13		
IP-10	rs147711004	A	G	1.1354	0.0366	0.0288	1.00E-200	0.072	962.36		
IP-10	rs150685845	G	A	0.5561	0.0645	0.9871	6.62E-18	0.008	74.33		
IP-10	rs1582763	A	G	-0.1232	0.0149	0.6372	1.19E-16	0.007	68.37		

IP-10	rs17125924	G	A	0.1222	0.0246	0.9205	6.62E-07	0.002	24.68	
IP-10	rs2830489	T	C	-0.0837	0.0162	0.7038	2.42E-07	0.003	26.69	
IP-10	rs34665982	C	T	-0.0967	0.0166	0.4901	5.80E-09	0.005	33.93	
IP-10	rs34971488	A	G	0.094	0.0198	0.2346	2.07E-06	0.003	22.54	
IP-10	rs35695568	T	G	0.1152	0.0247	0.8986	3.20E-06	0.002	21.75	
IP-10	rs3740688	T	G	0.0935	0.0144	0.4573	9.70E-11	0.004	42.16	
IP-10	rs383902	T	C	-0.0698	0.0151	0.6402	3.81E-06	0.002	21.37	
IP-10	rs3851179	C	T	0.1198	0.0148	0.3708	5.81E-16	0.007	65.52	
IP-10	rs6014724	G	A	-0.1319	0.0259	0.9105	3.65E-07	0.003	25.94	
IP-10	rs62039712	A	G	0.1528	0.0288	0.1213	1.17E-07	0.005	28.15	
IP-10	rs6559689	T	C	0.1585	0.0335	0.9483	2.17E-06	0.002	22.39	
IP-10	rs6733839	T	C	0.1693	0.0154	0.6203	4.02E-28	0.014	120.86	
IP-10	rs679515	C	T	-0.1508	0.0183	0.828	1.55E-16	0.006	67.90	
IP-10	rs72654445	A	G	-0.5425	0.0811	0.0109	2.27E-11	0.006	44.75	
IP-10	rs72993825	T	C	-0.1763	0.036	0.9404	9.86E-07	0.003	23.98	
IP-10	rs73223431	T	C	0.0936	0.0153	0.6541	8.34E-10	0.004	37.43	
IP-10	rs7412	T	C	-0.4673	0.0305	0.9374	6.40E-53	0.026	234.74	
IP-10	rs7584040	T	C	0.0862	0.0172	0.8131	5.34E-07	0.002	25.12	
IP-10	rs7618668	A	G	-0.1297	0.0258	0.0815	4.95E-07	0.003	25.27	
IP-10	rs8111708	G	A	0.0696	0.0151	0.6541	3.95E-06	0.002	21.25	
IP-10	rs867230	A	C	0.1333	0.0158	0.5984	3.49E-17	0.009	71.18	
IP-10	rs9381563	T	C	-0.0821	0.0148	0.3221	2.93E-08	0.003	30.77	
IP-10	rs9649710	G	A	0.0676	0.0148	0.3767	4.79E-06	0.002	20.86	
IP-10	rs9787911	C	T	0.0662	0.0144	0.4274	4.39E-06	0.002	21.13	
MCP-1-MCAF	rs1081105	C	A	0.942	0.0436	0.9692	1.51E-103	0.053	466.80	49.50%
MCP-1-MCAF	rs111278137	A	G	-0.4735	0.0713	0.0139	3.20E-11	0.006	44.10	

MCP-1-MCAF	rs11168036	G	T	-0.0754	0.0143	0.5169	1.43E-07	0.003	27.80
MCP-1-MCAF	rs117240937	A	G	-0.3122	0.0672	0.0179	3.35E-06	0.003	21.58
MCP-1-MCAF	rs11767557	C	T	-0.1028	0.0182	0.2177	1.56E-08	0.004	31.90
MCP-1-MCAF	rs12151021	G	A	-0.1071	0.0169	0.338	2.56E-10	0.005	40.16
MCP-1-MCAF	rs12590654	A	G	-0.0906	0.0157	0.3370	8.73E-09	0.004	33.30
MCP-1-MCAF	rs13252043	T	C	0.114	0.0237	0.9066	1.57E-06	0.002	23.14
MCP-1-MCAF	rs138727474	T	C	-0.2515	0.0545	0.9761	3.90E-06	0.003	21.30
MCP-1-MCAF	rs141739979	T	G	-0.4544	0.0855	0.9861	1.07E-07	0.006	28.25
MCP-1-MCAF	rs143429938	T	C	0.3535	0.0769	0.9732	4.25E-06	0.007	21.13
MCP-1-MCAF	rs147711004	A	G	1.1354	0.0366	0.0288	1.00E-200	0.072	962.36
MCP-1-MCAF	rs150685845	G	A	0.5561	0.0645	0.9871	6.62E-18	0.008	74.33
MCP-1-MCAF	rs1582763	A	G	-0.1232	0.0149	0.6372	1.19E-16	0.007	68.37
MCP-1-MCAF	rs17125924	G	A	0.1222	0.0246	0.9205	6.62E-07	0.002	24.68
MCP-1-MCAF	rs2830489	T	C	-0.0837	0.0162	0.7038	2.42E-07	0.003	26.69
MCP-1-MCAF	rs34665982	C	T	-0.0967	0.0166	0.4901	5.80E-09	0.005	33.93
MCP-1-MCAF	rs34971488	A	G	0.094	0.0198	0.2346	2.07E-06	0.003	22.54
MCP-1-MCAF	rs35695568	T	G	0.1152	0.0247	0.8986	3.20E-06	0.002	21.75
MCP-1-MCAF	rs3740688	T	G	0.0935	0.0144	0.4573	9.70E-11	0.004	42.16
MCP-1-MCAF	rs383902	T	C	-0.0698	0.0151	0.6402	3.81E-06	0.002	21.37
MCP-1-MCAF	rs3851179	C	T	0.1198	0.0148	0.3708	5.81E-16	0.007	65.52
MCP-1-MCAF	rs6014724	G	A	-0.1319	0.0259	0.9105	3.65E-07	0.003	25.94
MCP-1-MCAF	rs62039712	A	G	0.1528	0.0288	0.1213	1.17E-07	0.005	28.15
MCP-1-MCAF	rs6559689	T	C	0.1585	0.0335	0.9483	2.17E-06	0.002	22.39
MCP-1-MCAF	rs6733839	T	C	0.1693	0.0154	0.6203	4.02E-28	0.014	120.86
MCP-1-MCAF	rs679515	C	T	-0.1508	0.0183	0.828	1.55E-16	0.006	67.90
MCP-1-MCAF	rs72654445	A	G	-0.5425	0.0811	0.0109	2.27E-11	0.006	44.75

MCP-1-MCAF	rs72993825	T	C	-0.1763	0.036	0.9404	9.86E-07	0.003	23.98	
MCP-1-MCAF	rs73223431	T	C	0.0936	0.0153	0.6541	8.34E-10	0.004	37.43	
MCP-1-MCAF	rs7412	T	C	-0.4673	0.0305	0.9374	6.40E-53	0.026	234.74	
MCP-1-MCAF	rs7584040	T	C	0.0862	0.0172	0.8131	5.34E-07	0.002	25.12	
MCP-1-MCAF	rs7618668	A	G	-0.1297	0.0258	0.0815	4.95E-07	0.003	25.27	
MCP-1-MCAF	rs8111708	G	A	0.0696	0.0151	0.6541	3.95E-06	0.002	21.25	
MCP-1-MCAF	rs867230	A	C	0.1333	0.0158	0.5984	3.49E-17	0.009	71.18	
MCP-1-MCAF	rs9381563	T	C	-0.0821	0.0148	0.3221	2.93E-08	0.003	30.77	
MCP-1-MCAF	rs9649710	G	A	0.0676	0.0148	0.3767	4.79E-06	0.002	20.86	
MCP-1-MCAF	rs9787911	C	T	0.0662	0.0144	0.4274	4.39E-06	0.002	21.13	
MCP-3	rs11168036	G	T	-0.0754	0.0143	0.5169	1.43E-07	0.003	27.80	
MCP-3	rs11767557	C	T	-0.1028	0.0182	0.2177	1.56E-08	0.004	31.90	
MCP-3	rs12151021	G	A	-0.1071	0.0169	0.338	2.56E-10	0.005	40.16	
MCP-3	rs12590654	A	G	-0.0906	0.0157	0.3370	8.73E-09	0.004	33.30	
MCP-3	rs13252043	T	C	0.114	0.0237	0.9066	1.57E-06	0.002	23.14	
MCP-3	rs138727474	T	C	-0.2515	0.0545	0.9761	3.90E-06	0.003	21.30	
MCP-3	rs143429938	T	C	0.3535	0.0769	0.9732	4.25E-06	0.007	21.13	
MCP-3	rs147711004	A	G	1.1354	0.0366	0.0288	1.00E-200	0.072	962.36	100.00%
MCP-3	rs1582763	A	G	-0.1232	0.0149	0.6372	1.19E-16	0.007	68.37	
MCP-3	rs17125924	G	A	0.1222	0.0246	0.9205	6.62E-07	0.002	24.68	
MCP-3	rs2830489	T	C	-0.0837	0.0162	0.7038	2.42E-07	0.003	26.69	
MCP-3	rs34665982	C	T	-0.0967	0.0166	0.4901	5.80E-09	0.005	33.93	
MCP-3	rs34971488	A	G	0.094	0.0198	0.2346	2.07E-06	0.003	22.54	
MCP-3	rs35695568	T	G	0.1152	0.0247	0.8986	3.20E-06	0.002	21.75	
MCP-3	rs3740688	T	G	0.0935	0.0144	0.4573	9.70E-11	0.004	42.16	
MCP-3	rs383902	T	C	-0.0698	0.0151	0.6402	3.81E-06	0.002	21.37	

MCP-3	rs3851179	C	T	0.1198	0.0148	0.3708	5.81E-16	0.007	65.52	
MCP-3	rs6014724	G	A	-0.1319	0.0259	0.9105	3.65E-07	0.003	25.94	
MCP-3	rs62039712	A	G	0.1528	0.0288	0.1213	1.17E-07	0.005	28.15	
MCP-3	rs6559689	T	C	0.1585	0.0335	0.9483	2.17E-06	0.002	22.39	
MCP-3	rs6733839	T	C	0.1693	0.0154	0.6203	4.02E-28	0.014	120.86	
MCP-3	rs679515	C	T	-0.1508	0.0183	0.828	1.55E-16	0.006	67.90	
MCP-3	rs72993825	T	C	-0.1763	0.036	0.9404	9.86E-07	0.003	23.98	
MCP-3	rs73223431	T	C	0.0936	0.0153	0.6541	8.34E-10	0.004	37.43	
MCP-3	rs7412	T	C	-0.4673	0.0305	0.9374	6.40E-53	0.026	234.74	
MCP-3	rs7584040	T	C	0.0862	0.0172	0.8131	5.34E-07	0.002	25.12	
MCP-3	rs7618668	A	G	-0.1297	0.0258	0.0815	4.95E-07	0.003	25.27	
MCP-3	rs8111708	G	A	0.0696	0.0151	0.6541	3.95E-06	0.002	21.25	
MCP-3	rs867230	A	C	0.1333	0.0158	0.5984	3.49E-17	0.009	71.18	
MCP-3	rs9381563	T	C	-0.0821	0.0148	0.3221	2.93E-08	0.003	30.77	
MCP-3	rs9649710	G	A	0.0676	0.0148	0.3767	4.79E-06	0.002	20.86	
MCP-3	rs9787911	C	T	0.0662	0.0144	0.4274	4.39E-06	0.002	21.13	
M-CSF	rs1081105	C	A	0.942	0.0436	0.9692	1.51E-103	0.053	466.80	
M-CSF	rs111278137	A	G	-0.4735	0.0713	0.0139	3.20E-11	0.006	44.10	
M-CSF	rs11168036	G	T	-0.0754	0.0143	0.5169	1.43E-07	0.003	27.80	
M-CSF	rs117240937	A	G	-0.3122	0.0672	0.0179	3.35E-06	0.003	21.58	
M-CSF	rs11767557	C	T	-0.1028	0.0182	0.2177	1.56E-08	0.004	31.90	100.00%
M-CSF	rs12151021	G	A	-0.1071	0.0169	0.338	2.56E-10	0.005	40.16	
M-CSF	rs12590654	A	G	-0.0906	0.0157	0.3370	8.73E-09	0.004	33.30	
M-CSF	rs13252043	T	C	0.114	0.0237	0.9066	1.57E-06	0.002	23.14	
M-CSF	rs138727474	T	C	-0.2515	0.0545	0.9761	3.90E-06	0.003	21.30	
M-CSF	rs141739979	T	G	-0.4544	0.0855	0.9861	1.07E-07	0.006	28.25	

M-CSF	rs143429938	T	C	0.3535	0.0769	0.9732	4.25E-06	0.007	21.13
M-CSF	rs147711004	A	G	1.1354	0.0366	0.0288	1.00E-200	0.072	962.36
M-CSF	rs150685845	G	A	0.5561	0.0645	0.9871	6.62E-18	0.008	74.33
M-CSF	rs1582763	A	G	-0.1232	0.0149	0.6372	1.19E-16	0.007	68.37
M-CSF	rs17125924	G	A	0.1222	0.0246	0.9205	6.62E-07	0.002	24.68
M-CSF	rs2830489	T	C	-0.0837	0.0162	0.7038	2.42E-07	0.003	26.69
M-CSF	rs34665982	C	T	-0.0967	0.0166	0.4901	5.80E-09	0.005	33.93
M-CSF	rs34971488	A	G	0.094	0.0198	0.2346	2.07E-06	0.003	22.54
M-CSF	rs35695568	T	G	0.1152	0.0247	0.8986	3.20E-06	0.002	21.75
M-CSF	rs3740688	T	G	0.0935	0.0144	0.4573	9.70E-11	0.004	42.16
M-CSF	rs383902	T	C	-0.0698	0.0151	0.6402	3.81E-06	0.002	21.37
M-CSF	rs3851179	C	T	0.1198	0.0148	0.3708	5.81E-16	0.007	65.52
M-CSF	rs6014724	G	A	-0.1319	0.0259	0.9105	3.65E-07	0.003	25.94
M-CSF	rs62039712	A	G	0.1528	0.0288	0.1213	1.17E-07	0.005	28.15
M-CSF	rs6559689	T	C	0.1585	0.0335	0.9483	2.17E-06	0.002	22.39
M-CSF	rs6733839	T	C	0.1693	0.0154	0.6203	4.02E-28	0.014	120.86
M-CSF	rs679515	C	T	-0.1508	0.0183	0.828	1.55E-16	0.006	67.90
M-CSF	rs72654445	A	G	-0.5425	0.0811	0.0109	2.27E-11	0.006	44.75
M-CSF	rs72993825	T	C	-0.1763	0.036	0.9404	9.86E-07	0.003	23.98
M-CSF	rs73223431	T	C	0.0936	0.0153	0.6541	8.34E-10	0.004	37.43
M-CSF	rs7412	T	C	-0.4673	0.0305	0.9374	6.40E-53	0.026	234.74
M-CSF	rs7584040	T	C	0.0862	0.0172	0.8131	5.34E-07	0.002	25.12
M-CSF	rs7618668	A	G	-0.1297	0.0258	0.0815	4.95E-07	0.003	25.27
M-CSF	rs8111708	G	A	0.0696	0.0151	0.6541	3.95E-06	0.002	21.25
M-CSF	rs867230	A	C	0.1333	0.0158	0.5984	3.49E-17	0.009	71.18
M-CSF	rs9381563	T	C	-0.0821	0.0148	0.3221	2.93E-08	0.003	30.77

M-CSF	rs9649710	G	A	0.0676	0.0148	0.3767	4.79E-06	0.002	20.86	
M-CSF	rs9787911	C	T	0.0662	0.0144	0.4274	4.39E-06	0.002	21.13	
MIF	rs1081105	C	A	0.942	0.0436	0.9692	1.51E-103	0.053	466.80	
MIF	rs11168036	G	T	-0.0754	0.0143	0.5169	1.43E-07	0.003	27.80	
MIF	rs11767557	C	T	-0.1028	0.0182	0.2177	1.56E-08	0.004	31.90	
MIF	rs12151021	G	A	-0.1071	0.0169	0.338	2.56E-10	0.005	40.16	
MIF	rs12590654	A	G	-0.0906	0.0157	0.3370	8.73E-09	0.004	33.30	
MIF	rs13252043	T	C	0.114	0.0237	0.9066	1.57E-06	0.002	23.14	
MIF	rs138727474	T	C	-0.2515	0.0545	0.9761	3.90E-06	0.003	21.30	
MIF	rs141739979	T	G	-0.4544	0.0855	0.9861	1.07E-07	0.006	28.25	
MIF	rs143429938	T	C	0.3535	0.0769	0.9732	4.25E-06	0.007	21.13	
MIF	rs147711004	A	G	1.1354	0.0366	0.0288	1.00E-200	0.072	962.36	
MIF	rs150685845	G	A	0.5561	0.0645	0.9871	6.62E-18	0.008	74.33	
MIF	rs1582763	A	G	-0.1232	0.0149	0.6372	1.19E-16	0.007	68.37	99.80%
MIF	rs17125924	G	A	0.1222	0.0246	0.9205	6.62E-07	0.002	24.68	
MIF	rs2830489	T	C	-0.0837	0.0162	0.7038	2.42E-07	0.003	26.69	
MIF	rs34665982	C	T	-0.0967	0.0166	0.4901	5.80E-09	0.005	33.93	
MIF	rs34971488	A	G	0.094	0.0198	0.2346	2.07E-06	0.003	22.54	
MIF	rs35695568	T	G	0.1152	0.0247	0.8986	3.20E-06	0.002	21.75	
MIF	rs3740688	T	G	0.0935	0.0144	0.4573	9.70E-11	0.004	42.16	
MIF	rs383902	T	C	-0.0698	0.0151	0.6402	3.81E-06	0.002	21.37	
MIF	rs3851179	C	T	0.1198	0.0148	0.3708	5.81E-16	0.007	65.52	
MIF	rs6014724	G	A	-0.1319	0.0259	0.9105	3.65E-07	0.003	25.94	
MIF	rs62039712	A	G	0.1528	0.0288	0.1213	1.17E-07	0.005	28.15	
MIF	rs6559689	T	C	0.1585	0.0335	0.9483	2.17E-06	0.002	22.39	
MIF	rs6733839	T	C	0.1693	0.0154	0.6203	4.02E-28	0.014	120.86	

MIF	rs679515	C	T	-0.1508	0.0183	0.828	1.55E-16	0.006	67.90	
MIF	rs72993825	T	C	-0.1763	0.036	0.9404	9.86E-07	0.003	23.98	
MIF	rs73223431	T	C	0.0936	0.0153	0.6541	8.34E-10	0.004	37.43	
MIF	rs7412	T	C	-0.4673	0.0305	0.9374	6.40E-53	0.026	234.74	
MIF	rs7584040	T	C	0.0862	0.0172	0.8131	5.34E-07	0.002	25.12	
MIF	rs7618668	A	G	-0.1297	0.0258	0.0815	4.95E-07	0.003	25.27	
MIF	rs8111708	G	A	0.0696	0.0151	0.6541	3.95E-06	0.002	21.25	
MIF	rs867230	A	C	0.1333	0.0158	0.5984	3.49E-17	0.009	71.18	
MIF	rs9381563	T	C	-0.0821	0.0148	0.3221	2.93E-08	0.003	30.77	
MIF	rs9649710	G	A	0.0676	0.0148	0.3767	4.79E-06	0.002	20.86	
MIF	rs9787911	C	T	0.0662	0.0144	0.4274	4.39E-06	0.002	21.13	
MIG	rs1081105	C	A	0.942	0.0436	0.9692	1.51E-103	0.053	466.80	
MIG	rs111278137	A	G	-0.4735	0.0713	0.0139	3.20E-11	0.006	44.10	
MIG	rs11168036	G	T	-0.0754	0.0143	0.5169	1.43E-07	0.003	27.80	
MIG	rs11767557	C	T	-0.1028	0.0182	0.2177	1.56E-08	0.004	31.90	
MIG	rs12151021	G	A	-0.1071	0.0169	0.338	2.56E-10	0.005	40.16	
MIG	rs12590654	A	G	-0.0906	0.0157	0.3370	8.73E-09	0.004	33.30	
MIG	rs13252043	T	C	0.114	0.0237	0.9066	1.57E-06	0.002	23.14	
MIG	rs138727474	T	C	-0.2515	0.0545	0.9761	3.90E-06	0.003	21.30	98.00%
MIG	rs141739979	T	G	-0.4544	0.0855	0.9861	1.07E-07	0.006	28.25	
MIG	rs143429938	T	C	0.3535	0.0769	0.9732	4.25E-06	0.007	21.13	
MIG	rs147711004	A	G	1.1354	0.0366	0.0288	1.00E-200	0.072	962.36	
MIG	rs150685845	G	A	0.5561	0.0645	0.9871	6.62E-18	0.008	74.33	
MIG	rs1582763	A	G	-0.1232	0.0149	0.6372	1.19E-16	0.007	68.37	
MIG	rs17125924	G	A	0.1222	0.0246	0.9205	6.62E-07	0.002	24.68	
MIG	rs2830489	T	C	-0.0837	0.0162	0.7038	2.42E-07	0.003	26.69	

MIG	rs34665982	C	T	-0.0967	0.0166	0.4901	5.80E-09	0.005	33.93	
MIG	rs34971488	A	G	0.094	0.0198	0.2346	2.07E-06	0.003	22.54	
MIG	rs35695568	T	G	0.1152	0.0247	0.8986	3.20E-06	0.002	21.75	
MIG	rs3740688	T	G	0.0935	0.0144	0.4573	9.70E-11	0.004	42.16	
MIG	rs383902	T	C	-0.0698	0.0151	0.6402	3.81E-06	0.002	21.37	
MIG	rs3851179	C	T	0.1198	0.0148	0.3708	5.81E-16	0.007	65.52	
MIG	rs6014724	G	A	-0.1319	0.0259	0.9105	3.65E-07	0.003	25.94	
MIG	rs62039712	A	G	0.1528	0.0288	0.1213	1.17E-07	0.005	28.15	
MIG	rs6559689	T	C	0.1585	0.0335	0.9483	2.17E-06	0.002	22.39	
MIG	rs6733839	T	C	0.1693	0.0154	0.6203	4.02E-28	0.014	120.86	
MIG	rs679515	C	T	-0.1508	0.0183	0.828	1.55E-16	0.006	67.90	
MIG	rs72654445	A	G	-0.5425	0.0811	0.0109	2.27E-11	0.006	44.75	
MIG	rs72993825	T	C	-0.1763	0.036	0.9404	9.86E-07	0.003	23.98	
MIG	rs73223431	T	C	0.0936	0.0153	0.6541	8.34E-10	0.004	37.43	
MIG	rs7412	T	C	-0.4673	0.0305	0.9374	6.40E-53	0.026	234.74	
MIG	rs7584040	T	C	0.0862	0.0172	0.8131	5.34E-07	0.002	25.12	
MIG	rs7618668	A	G	-0.1297	0.0258	0.0815	4.95E-07	0.003	25.27	
MIG	rs8111708	G	A	0.0696	0.0151	0.6541	3.95E-06	0.002	21.25	
MIG	rs867230	A	C	0.1333	0.0158	0.5984	3.49E-17	0.009	71.18	
MIG	rs9381563	T	C	-0.0821	0.0148	0.3221	2.93E-08	0.003	30.77	
MIG	rs9649710	G	A	0.0676	0.0148	0.3767	4.79E-06	0.002	20.86	
MIG	rs9787911	C	T	0.0662	0.0144	0.4274	4.39E-06	0.002	21.13	
MIP-1A	rs1081105	C	A	0.942	0.0436	0.9692	1.51E-103	0.053	466.80	
MIP-1A	rs111278137	A	G	-0.4735	0.0713	0.0139	3.20E-11	0.006	44.10	
MIP-1A	rs11168036	G	T	-0.0754	0.0143	0.5169	1.43E-07	0.003	27.80	100.00%
MIP-1A	rs117240937	A	G	-0.3122	0.0672	0.0179	3.35E-06	0.003	21.58	

MIP-1A	rs11767557	C	T	-0.1028	0.0182	0.2177	1.56E-08	0.004	31.90
MIP-1A	rs12151021	G	A	-0.1071	0.0169	0.338	2.56E-10	0.005	40.16
MIP-1A	rs12590654	A	G	-0.0906	0.0157	0.3370	8.73E-09	0.004	33.30
MIP-1A	rs13252043	T	C	0.114	0.0237	0.9066	1.57E-06	0.002	23.14
MIP-1A	rs138727474	T	C	-0.2515	0.0545	0.9761	3.90E-06	0.003	21.30
MIP-1A	rs141739979	T	G	-0.4544	0.0855	0.9861	1.07E-07	0.006	28.25
MIP-1A	rs143429938	T	C	0.3535	0.0769	0.9732	4.25E-06	0.007	21.13
MIP-1A	rs147711004	A	G	1.1354	0.0366	0.0288	1.00E-200	0.072	962.36
MIP-1A	rs150685845	G	A	0.5561	0.0645	0.9871	6.62E-18	0.008	74.33
MIP-1A	rs1582763	A	G	-0.1232	0.0149	0.6372	1.19E-16	0.007	68.37
MIP-1A	rs17125924	G	A	0.1222	0.0246	0.9205	6.62E-07	0.002	24.68
MIP-1A	rs2830489	T	C	-0.0837	0.0162	0.7038	2.42E-07	0.003	26.69
MIP-1A	rs34665982	C	T	-0.0967	0.0166	0.4901	5.80E-09	0.005	33.93
MIP-1A	rs34971488	A	G	0.094	0.0198	0.2346	2.07E-06	0.003	22.54
MIP-1A	rs35695568	T	G	0.1152	0.0247	0.8986	3.20E-06	0.002	21.75
MIP-1A	rs3740688	T	G	0.0935	0.0144	0.4573	9.70E-11	0.004	42.16
MIP-1A	rs383902	T	C	-0.0698	0.0151	0.6402	3.81E-06	0.002	21.37
MIP-1A	rs3851179	C	T	0.1198	0.0148	0.3708	5.81E-16	0.007	65.52
MIP-1A	rs6014724	G	A	-0.1319	0.0259	0.9105	3.65E-07	0.003	25.94
MIP-1A	rs62039712	A	G	0.1528	0.0288	0.1213	1.17E-07	0.005	28.15
MIP-1A	rs6559689	T	C	0.1585	0.0335	0.9483	2.17E-06	0.002	22.39
MIP-1A	rs6733839	T	C	0.1693	0.0154	0.6203	4.02E-28	0.014	120.86
MIP-1A	rs679515	C	T	-0.1508	0.0183	0.828	1.55E-16	0.006	67.90
MIP-1A	rs72654445	A	G	-0.5425	0.0811	0.0109	2.27E-11	0.006	44.75
MIP-1A	rs72993825	T	C	-0.1763	0.036	0.9404	9.86E-07	0.003	23.98
MIP-1A	rs73223431	T	C	0.0936	0.0153	0.6541	8.34E-10	0.004	37.43

MIP-1A	rs7412	T	C	-0.4673	0.0305	0.9374	6.40E-53	0.026	234.74	
MIP-1A	rs7584040	T	C	0.0862	0.0172	0.8131	5.34E-07	0.002	25.12	
MIP-1A	rs7618668	A	G	-0.1297	0.0258	0.0815	4.95E-07	0.003	25.27	
MIP-1A	rs8111708	G	A	0.0696	0.0151	0.6541	3.95E-06	0.002	21.25	
MIP-1A	rs867230	A	C	0.1333	0.0158	0.5984	3.49E-17	0.009	71.18	
MIP-1A	rs9381563	T	C	-0.0821	0.0148	0.3221	2.93E-08	0.003	30.77	
MIP-1A	rs9649710	G	A	0.0676	0.0148	0.3767	4.79E-06	0.002	20.86	
MIP-1A	rs9787911	C	T	0.0662	0.0144	0.4274	4.39E-06	0.002	21.13	
MIP-1B	rs1081105	C	A	0.942	0.0436	0.9692	1.51E-103	0.053	466.80	
MIP-1B	rs111278137	A	G	-0.4735	0.0713	0.0139	3.20E-11	0.006	44.10	
MIP-1B	rs11168036	G	T	-0.0754	0.0143	0.5169	1.43E-07	0.003	27.80	
MIP-1B	rs117240937	A	G	-0.3122	0.0672	0.0179	3.35E-06	0.003	21.58	
MIP-1B	rs11767557	C	T	-0.1028	0.0182	0.2177	1.56E-08	0.004	31.90	
MIP-1B	rs12151021	G	A	-0.1071	0.0169	0.338	2.56E-10	0.005	40.16	
MIP-1B	rs12590654	A	G	-0.0906	0.0157	0.3370	8.73E-09	0.004	33.30	
MIP-1B	rs13252043	T	C	0.114	0.0237	0.9066	1.57E-06	0.002	23.14	
MIP-1B	rs138727474	T	C	-0.2515	0.0545	0.9761	3.90E-06	0.003	21.30	
MIP-1B	rs141739979	T	G	-0.4544	0.0855	0.9861	1.07E-07	0.006	28.25	8.00%
MIP-1B	rs143429938	T	C	0.3535	0.0769	0.9732	4.25E-06	0.007	21.13	
MIP-1B	rs147711004	A	G	1.1354	0.0366	0.0288	1.00E-200	0.072	962.36	
MIP-1B	rs150685845	G	A	0.5561	0.0645	0.9871	6.62E-18	0.008	74.33	
MIP-1B	rs1582763	A	G	-0.1232	0.0149	0.6372	1.19E-16	0.007	68.37	
MIP-1B	rs17125924	G	A	0.1222	0.0246	0.9205	6.62E-07	0.002	24.68	
MIP-1B	rs2830489	T	C	-0.0837	0.0162	0.7038	2.42E-07	0.003	26.69	
MIP-1B	rs34665982	C	T	-0.0967	0.0166	0.4901	5.80E-09	0.005	33.93	
MIP-1B	rs34971488	A	G	0.094	0.0198	0.2346	2.07E-06	0.003	22.54	

MIP-1B	rs35695568	T	G	0.1152	0.0247	0.8986	3.20E-06	0.002	21.75	
MIP-1B	rs3740688	T	G	0.0935	0.0144	0.4573	9.70E-11	0.004	42.16	
MIP-1B	rs383902	T	C	-0.0698	0.0151	0.6402	3.81E-06	0.002	21.37	
MIP-1B	rs3851179	C	T	0.1198	0.0148	0.3708	5.81E-16	0.007	65.52	
MIP-1B	rs6014724	G	A	-0.1319	0.0259	0.9105	3.65E-07	0.003	25.94	
MIP-1B	rs62039712	A	G	0.1528	0.0288	0.1213	1.17E-07	0.005	28.15	
MIP-1B	rs6559689	T	C	0.1585	0.0335	0.9483	2.17E-06	0.002	22.39	
MIP-1B	rs6733839	T	C	0.1693	0.0154	0.6203	4.02E-28	0.014	120.86	
MIP-1B	rs679515	C	T	-0.1508	0.0183	0.828	1.55E-16	0.006	67.90	
MIP-1B	rs72654445	A	G	-0.5425	0.0811	0.0109	2.27E-11	0.006	44.75	
MIP-1B	rs72993825	T	C	-0.1763	0.036	0.9404	9.86E-07	0.003	23.98	
MIP-1B	rs73223431	T	C	0.0936	0.0153	0.6541	8.34E-10	0.004	37.43	
MIP-1B	rs7412	T	C	-0.4673	0.0305	0.9374	6.40E-53	0.026	234.74	
MIP-1B	rs7584040	T	C	0.0862	0.0172	0.8131	5.34E-07	0.002	25.12	
MIP-1B	rs7618668	A	G	-0.1297	0.0258	0.0815	4.95E-07	0.003	25.27	
MIP-1B	rs8111708	G	A	0.0696	0.0151	0.6541	3.95E-06	0.002	21.25	
MIP-1B	rs867230	A	C	0.1333	0.0158	0.5984	3.49E-17	0.009	71.18	
MIP-1B	rs9381563	T	C	-0.0821	0.0148	0.3221	2.93E-08	0.003	30.77	
MIP-1B	rs9649710	G	A	0.0676	0.0148	0.3767	4.79E-06	0.002	20.86	
MIP-1B	rs9787911	C	T	0.0662	0.0144	0.4274	4.39E-06	0.002	21.13	
PDGF-BB	rs1081105	C	A	0.942	0.0436	0.9692	1.51E-103	0.053	466.80	
PDGF-BB	rs111278137	A	G	-0.4735	0.0713	0.0139	3.20E-11	0.006	44.10	
PDGF-BB	rs11168036	G	T	-0.0754	0.0143	0.5169	1.43E-07	0.003	27.80	49.50%
PDGF-BB	rs117240937	A	G	-0.3122	0.0672	0.0179	3.35E-06	0.003	21.58	
PDGF-BB	rs11767557	C	T	-0.1028	0.0182	0.2177	1.56E-08	0.004	31.90	
PDGF-BB	rs12151021	G	A	-0.1071	0.0169	0.338	2.56E-10	0.005	40.16	

PDGF-BB	rs12590654	A	G	-0.0906	0.0157	0.3370	8.73E-09	0.004	33.30
PDGF-BB	rs13252043	T	C	0.114	0.0237	0.9066	1.57E-06	0.002	23.14
PDGF-BB	rs138727474	T	C	-0.2515	0.0545	0.9761	3.90E-06	0.003	21.30
PDGF-BB	rs141739979	T	G	-0.4544	0.0855	0.9861	1.07E-07	0.006	28.25
PDGF-BB	rs143429938	T	C	0.3535	0.0769	0.9732	4.25E-06	0.007	21.13
PDGF-BB	rs147711004	A	G	1.1354	0.0366	0.0288	1.00E-200	0.072	962.36
PDGF-BB	rs150685845	G	A	0.5561	0.0645	0.9871	6.62E-18	0.008	74.33
PDGF-BB	rs1582763	A	G	-0.1232	0.0149	0.6372	1.19E-16	0.007	68.37
PDGF-BB	rs17125924	G	A	0.1222	0.0246	0.9205	6.62E-07	0.002	24.68
PDGF-BB	rs2830489	T	C	-0.0837	0.0162	0.7038	2.42E-07	0.003	26.69
PDGF-BB	rs34665982	C	T	-0.0967	0.0166	0.4901	5.80E-09	0.005	33.93
PDGF-BB	rs34971488	A	G	0.094	0.0198	0.2346	2.07E-06	0.003	22.54
PDGF-BB	rs35695568	T	G	0.1152	0.0247	0.8986	3.20E-06	0.002	21.75
PDGF-BB	rs3740688	T	G	0.0935	0.0144	0.4573	9.70E-11	0.004	42.16
PDGF-BB	rs383902	T	C	-0.0698	0.0151	0.6402	3.81E-06	0.002	21.37
PDGF-BB	rs3851179	C	T	0.1198	0.0148	0.3708	5.81E-16	0.007	65.52
PDGF-BB	rs6014724	G	A	-0.1319	0.0259	0.9105	3.65E-07	0.003	25.94
PDGF-BB	rs62039712	A	G	0.1528	0.0288	0.1213	1.17E-07	0.005	28.15
PDGF-BB	rs6559689	T	C	0.1585	0.0335	0.9483	2.17E-06	0.002	22.39
PDGF-BB	rs6733839	T	C	0.1693	0.0154	0.6203	4.02E-28	0.014	120.86
PDGF-BB	rs679515	C	T	-0.1508	0.0183	0.828	1.55E-16	0.006	67.90
PDGF-BB	rs72654445	A	G	-0.5425	0.0811	0.0109	2.27E-11	0.006	44.75
PDGF-BB	rs72993825	T	C	-0.1763	0.036	0.9404	9.86E-07	0.003	23.98
PDGF-BB	rs73223431	T	C	0.0936	0.0153	0.6541	8.34E-10	0.004	37.43
PDGF-BB	rs7412	T	C	-0.4673	0.0305	0.9374	6.40E-53	0.026	234.74
PDGF-BB	rs7584040	T	C	0.0862	0.0172	0.8131	5.34E-07	0.002	25.12

PDGF-BB	rs7618668	A	G	-0.1297	0.0258	0.0815	4.95E-07	0.003	25.27		
PDGF-BB	rs8111708	G	A	0.0696	0.0151	0.6541	3.95E-06	0.002	21.25		
PDGF-BB	rs867230	A	C	0.1333	0.0158	0.5984	3.49E-17	0.009	71.18		
PDGF-BB	rs9381563	T	C	-0.0821	0.0148	0.3221	2.93E-08	0.003	30.77		
PDGF-BB	rs9649710	G	A	0.0676	0.0148	0.3767	4.79E-06	0.002	20.86		
PDGF-BB	rs9787911	C	T	0.0662	0.0144	0.4274	4.39E-06	0.002	21.13		
RANTES	rs1081105	C	A	0.942	0.0436	0.9692	1.51E-103	0.053	466.80		
RANTES	rs111278137	A	G	-0.4735	0.0713	0.0139	3.20E-11	0.006	44.10		
RANTES	rs11168036	G	T	-0.0754	0.0143	0.5169	1.43E-07	0.003	27.80		
RANTES	rs11767557	C	T	-0.1028	0.0182	0.2177	1.56E-08	0.004	31.90		
RANTES	rs12151021	G	A	-0.1071	0.0169	0.338	2.56E-10	0.005	40.16		
RANTES	rs12590654	A	G	-0.0906	0.0157	0.3370	8.73E-09	0.004	33.30		
RANTES	rs13252043	T	C	0.114	0.0237	0.9066	1.57E-06	0.002	23.14		
RANTES	rs138727474	T	C	-0.2515	0.0545	0.9761	3.90E-06	0.003	21.30		
RANTES	rs141739979	T	G	-0.4544	0.0855	0.9861	1.07E-07	0.006	28.25		
RANTES	rs143429938	T	C	0.3535	0.0769	0.9732	4.25E-06	0.007	21.13		
RANTES	rs147711004	A	G	1.1354	0.0366	0.0288	1.00E-200	0.072	962.36	4.60%	
RANTES	rs150685845	G	A	0.5561	0.0645	0.9871	6.62E-18	0.008	74.33		
RANTES	rs1582763	A	G	-0.1232	0.0149	0.6372	1.19E-16	0.007	68.37		
RANTES	rs17125924	G	A	0.1222	0.0246	0.9205	6.62E-07	0.002	24.68		
RANTES	rs2830489	T	C	-0.0837	0.0162	0.7038	2.42E-07	0.003	26.69		
RANTES	rs34665982	C	T	-0.0967	0.0166	0.4901	5.80E-09	0.005	33.93		
RANTES	rs34971488	A	G	0.094	0.0198	0.2346	2.07E-06	0.003	22.54		
RANTES	rs35695568	T	G	0.1152	0.0247	0.8986	3.20E-06	0.002	21.75		
RANTES	rs3740688	T	G	0.0935	0.0144	0.4573	9.70E-11	0.004	42.16		
RANTES	rs383902	T	C	-0.0698	0.0151	0.6402	3.81E-06	0.002	21.37		

RANTES	rs3851179	C	T	0.1198	0.0148	0.3708	5.81E-16	0.007	65.52	
RANTES	rs6014724	G	A	-0.1319	0.0259	0.9105	3.65E-07	0.003	25.94	
RANTES	rs62039712	A	G	0.1528	0.0288	0.1213	1.17E-07	0.005	28.15	
RANTES	rs6559689	T	C	0.1585	0.0335	0.9483	2.17E-06	0.002	22.39	
RANTES	rs6733839	T	C	0.1693	0.0154	0.6203	4.02E-28	0.014	120.86	
RANTES	rs679515	C	T	-0.1508	0.0183	0.828	1.55E-16	0.006	67.90	
RANTES	rs72654445	A	G	-0.5425	0.0811	0.0109	2.27E-11	0.006	44.75	
RANTES	rs72993825	T	C	-0.1763	0.036	0.9404	9.86E-07	0.003	23.98	
RANTES	rs73223431	T	C	0.0936	0.0153	0.6541	8.34E-10	0.004	37.43	
RANTES	rs7412	T	C	-0.4673	0.0305	0.9374	6.40E-53	0.026	234.74	
RANTES	rs7584040	T	C	0.0862	0.0172	0.8131	5.34E-07	0.002	25.12	
RANTES	rs7618668	A	G	-0.1297	0.0258	0.0815	4.95E-07	0.003	25.27	
RANTES	rs8111708	G	A	0.0696	0.0151	0.6541	3.95E-06	0.002	21.25	
RANTES	rs867230	A	C	0.1333	0.0158	0.5984	3.49E-17	0.009	71.18	
RANTES	rs9381563	T	C	-0.0821	0.0148	0.3221	2.93E-08	0.003	30.77	
RANTES	rs9649710	G	A	0.0676	0.0148	0.3767	4.79E-06	0.002	20.86	
RANTES	rs9787911	C	T	0.0662	0.0144	0.4274	4.39E-06	0.002	21.13	
SCF	rs1081105	C	A	0.942	0.0436	0.9692	1.51E-103	0.053	466.80	
SCF	rs111278137	A	G	-0.4735	0.0713	0.0139	3.20E-11	0.006	44.10	
SCF	rs11168036	G	T	-0.0754	0.0143	0.5169	1.43E-07	0.003	27.80	
SCF	rs117240937	A	G	-0.3122	0.0672	0.0179	3.35E-06	0.003	21.58	
SCF	rs11767557	C	T	-0.1028	0.0182	0.2177	1.56E-08	0.004	31.90	6.10%
SCF	rs12151021	G	A	-0.1071	0.0169	0.338	2.56E-10	0.005	40.16	
SCF	rs12590654	A	G	-0.0906	0.0157	0.3370	8.73E-09	0.004	33.30	
SCF	rs13252043	T	C	0.114	0.0237	0.9066	1.57E-06	0.002	23.14	
SCF	rs138727474	T	C	-0.2515	0.0545	0.9761	3.90E-06	0.003	21.30	

SCF	rs141739979	T	G	-0.4544	0.0855	0.9861	1.07E-07	0.006	28.25
SCF	rs143429938	T	C	0.3535	0.0769	0.9732	4.25E-06	0.007	21.13
SCF	rs147711004	A	G	1.1354	0.0366	0.0288	1.00E-200	0.072	962.36
SCF	rs150685845	G	A	0.5561	0.0645	0.9871	6.62E-18	0.008	74.33
SCF	rs1582763	A	G	-0.1232	0.0149	0.6372	1.19E-16	0.007	68.37
SCF	rs17125924	G	A	0.1222	0.0246	0.9205	6.62E-07	0.002	24.68
SCF	rs2830489	T	C	-0.0837	0.0162	0.7038	2.42E-07	0.003	26.69
SCF	rs34665982	C	T	-0.0967	0.0166	0.4901	5.80E-09	0.005	33.93
SCF	rs34971488	A	G	0.094	0.0198	0.2346	2.07E-06	0.003	22.54
SCF	rs35695568	T	G	0.1152	0.0247	0.8986	3.20E-06	0.002	21.75
SCF	rs3740688	T	G	0.0935	0.0144	0.4573	9.70E-11	0.004	42.16
SCF	rs383902	T	C	-0.0698	0.0151	0.6402	3.81E-06	0.002	21.37
SCF	rs3851179	C	T	0.1198	0.0148	0.3708	5.81E-16	0.007	65.52
SCF	rs6014724	G	A	-0.1319	0.0259	0.9105	3.65E-07	0.003	25.94
SCF	rs62039712	A	G	0.1528	0.0288	0.1213	1.17E-07	0.005	28.15
SCF	rs6559689	T	C	0.1585	0.0335	0.9483	2.17E-06	0.002	22.39
SCF	rs6733839	T	C	0.1693	0.0154	0.6203	4.02E-28	0.014	120.86
SCF	rs679515	C	T	-0.1508	0.0183	0.828	1.55E-16	0.006	67.90
SCF	rs72654445	A	G	-0.5425	0.0811	0.0109	2.27E-11	0.006	44.75
SCF	rs72993825	T	C	-0.1763	0.036	0.9404	9.86E-07	0.003	23.98
SCF	rs73223431	T	C	0.0936	0.0153	0.6541	8.34E-10	0.004	37.43
SCF	rs7412	T	C	-0.4673	0.0305	0.9374	6.40E-53	0.026	234.74
SCF	rs7584040	T	C	0.0862	0.0172	0.8131	5.34E-07	0.002	25.12
SCF	rs7618668	A	G	-0.1297	0.0258	0.0815	4.95E-07	0.003	25.27
SCF	rs8111708	G	A	0.0696	0.0151	0.6541	3.95E-06	0.002	21.25
SCF	rs867230	A	C	0.1333	0.0158	0.5984	3.49E-17	0.009	71.18

SCF	rs9381563	T	C	-0.0821	0.0148	0.3221	2.93E-08	0.003	30.77	
SCF	rs9649710	G	A	0.0676	0.0148	0.3767	4.79E-06	0.002	20.86	
SCF	rs9787911	C	T	0.0662	0.0144	0.4274	4.39E-06	0.002	21.13	
SCGF	rs1081105	C	A	0.942	0.0436	0.9692	1.51E-103	0.053	466.80	
SCGF	rs111278137	A	G	-0.4735	0.0713	0.0139	3.20E-11	0.006	44.10	
SCGF	rs11168036	G	T	-0.0754	0.0143	0.5169	1.43E-07	0.003	27.80	
SCGF	rs11767557	C	T	-0.1028	0.0182	0.2177	1.56E-08	0.004	31.90	
SCGF	rs12151021	G	A	-0.1071	0.0169	0.338	2.56E-10	0.005	40.16	
SCGF	rs12590654	A	G	-0.0906	0.0157	0.3370	8.73E-09	0.004	33.30	
SCGF	rs13252043	T	C	0.114	0.0237	0.9066	1.57E-06	0.002	23.14	
SCGF	rs138727474	T	C	-0.2515	0.0545	0.9761	3.90E-06	0.003	21.30	
SCGF	rs141739979	T	G	-0.4544	0.0855	0.9861	1.07E-07	0.006	28.25	
SCGF	rs143429938	T	C	0.3535	0.0769	0.9732	4.25E-06	0.007	21.13	
SCGF	rs147711004	A	G	1.1354	0.0366	0.0288	1.00E-200	0.072	962.36	
SCGF	rs150685845	G	A	0.5561	0.0645	0.9871	6.62E-18	0.008	74.33	100.00%
SCGF	rs1582763	A	G	-0.1232	0.0149	0.6372	1.19E-16	0.007	68.37	
SCGF	rs17125924	G	A	0.1222	0.0246	0.9205	6.62E-07	0.002	24.68	
SCGF	rs2830489	T	C	-0.0837	0.0162	0.7038	2.42E-07	0.003	26.69	
SCGF	rs34665982	C	T	-0.0967	0.0166	0.4901	5.80E-09	0.005	33.93	
SCGF	rs34971488	A	G	0.094	0.0198	0.2346	2.07E-06	0.003	22.54	
SCGF	rs35695568	T	G	0.1152	0.0247	0.8986	3.20E-06	0.002	21.75	
SCGF	rs3740688	T	G	0.0935	0.0144	0.4573	9.70E-11	0.004	42.16	
SCGF	rs383902	T	C	-0.0698	0.0151	0.6402	3.81E-06	0.002	21.37	
SCGF	rs3851179	C	T	0.1198	0.0148	0.3708	5.81E-16	0.007	65.52	
SCGF	rs6014724	G	A	-0.1319	0.0259	0.9105	3.65E-07	0.003	25.94	
SCGF	rs62039712	A	G	0.1528	0.0288	0.1213	1.17E-07	0.005	28.15	

SCGF	rs6559689	T	C	0.1585	0.0335	0.9483	2.17E-06	0.002	22.39	
SCGF	rs6733839	T	C	0.1693	0.0154	0.6203	4.02E-28	0.014	120.86	
SCGF	rs679515	C	T	-0.1508	0.0183	0.828	1.55E-16	0.006	67.90	
SCGF	rs72654445	A	G	-0.5425	0.0811	0.0109	2.27E-11	0.006	44.75	
SCGF	rs72993825	T	C	-0.1763	0.036	0.9404	9.86E-07	0.003	23.98	
SCGF	rs73223431	T	C	0.0936	0.0153	0.6541	8.34E-10	0.004	37.43	
SCGF	rs7412	T	C	-0.4673	0.0305	0.9374	6.40E-53	0.026	234.74	
SCGF	rs7584040	T	C	0.0862	0.0172	0.8131	5.34E-07	0.002	25.12	
SCGF	rs7618668	A	G	-0.1297	0.0258	0.0815	4.95E-07	0.003	25.27	
SCGF	rs8111708	G	A	0.0696	0.0151	0.6541	3.95E-06	0.002	21.25	
SCGF	rs867230	A	C	0.1333	0.0158	0.5984	3.49E-17	0.009	71.18	
SCGF	rs9381563	T	C	-0.0821	0.0148	0.3221	2.93E-08	0.003	30.77	
SCGF	rs9649710	G	A	0.0676	0.0148	0.3767	4.79E-06	0.002	20.86	
SCGF	rs9787911	C	T	0.0662	0.0144	0.4274	4.39E-06	0.002	21.13	
SDF-1A	rs1081105	C	A	0.942	0.0436	0.9692	1.51E-103	0.053	466.80	
SDF-1A	rs111278137	A	G	-0.4735	0.0713	0.0139	3.20E-11	0.006	44.10	
SDF-1A	rs11168036	G	T	-0.0754	0.0143	0.5169	1.43E-07	0.003	27.80	
SDF-1A	rs117240937	A	G	-0.3122	0.0672	0.0179	3.35E-06	0.003	21.58	
SDF-1A	rs11767557	C	T	-0.1028	0.0182	0.2177	1.56E-08	0.004	31.90	
SDF-1A	rs12151021	G	A	-0.1071	0.0169	0.338	2.56E-10	0.005	40.16	70.70%
SDF-1A	rs12590654	A	G	-0.0906	0.0157	0.3370	8.73E-09	0.004	33.30	
SDF-1A	rs13252043	T	C	0.114	0.0237	0.9066	1.57E-06	0.002	23.14	
SDF-1A	rs138727474	T	C	-0.2515	0.0545	0.9761	3.90E-06	0.003	21.30	
SDF-1A	rs141739979	T	G	-0.4544	0.0855	0.9861	1.07E-07	0.006	28.25	
SDF-1A	rs143429938	T	C	0.3535	0.0769	0.9732	4.25E-06	0.007	21.13	
SDF-1A	rs147711004	A	G	1.1354	0.0366	0.0288	1.00E-200	0.072	962.36	

SDF-1A	rs150685845	G	A	0.5561	0.0645	0.9871	6.62E-18	0.008	74.33
SDF-1A	rs1582763	A	G	-0.1232	0.0149	0.6372	1.19E-16	0.007	68.37
SDF-1A	rs17125924	G	A	0.1222	0.0246	0.9205	6.62E-07	0.002	24.68
SDF-1A	rs2830489	T	C	-0.0837	0.0162	0.7038	2.42E-07	0.003	26.69
SDF-1A	rs34665982	C	T	-0.0967	0.0166	0.4901	5.80E-09	0.005	33.93
SDF-1A	rs34971488	A	G	0.094	0.0198	0.2346	2.07E-06	0.003	22.54
SDF-1A	rs35695568	T	G	0.1152	0.0247	0.8986	3.20E-06	0.002	21.75
SDF-1A	rs3740688	T	G	0.0935	0.0144	0.4573	9.70E-11	0.004	42.16
SDF-1A	rs383902	T	C	-0.0698	0.0151	0.6402	3.81E-06	0.002	21.37
SDF-1A	rs3851179	C	T	0.1198	0.0148	0.3708	5.81E-16	0.007	65.52
SDF-1A	rs6014724	G	A	-0.1319	0.0259	0.9105	3.65E-07	0.003	25.94
SDF-1A	rs62039712	A	G	0.1528	0.0288	0.1213	1.17E-07	0.005	28.15
SDF-1A	rs6559689	T	C	0.1585	0.0335	0.9483	2.17E-06	0.002	22.39
SDF-1A	rs6733839	T	C	0.1693	0.0154	0.6203	4.02E-28	0.014	120.86
SDF-1A	rs679515	C	T	-0.1508	0.0183	0.828	1.55E-16	0.006	67.90
SDF-1A	rs72654445	A	G	-0.5425	0.0811	0.0109	2.27E-11	0.006	44.75
SDF-1A	rs72993825	T	C	-0.1763	0.036	0.9404	9.86E-07	0.003	23.98
SDF-1A	rs73223431	T	C	0.0936	0.0153	0.6541	8.34E-10	0.004	37.43
SDF-1A	rs7412	T	C	-0.4673	0.0305	0.9374	6.40E-53	0.026	234.74
SDF-1A	rs7584040	T	C	0.0862	0.0172	0.8131	5.34E-07	0.002	25.12
SDF-1A	rs7618668	A	G	-0.1297	0.0258	0.0815	4.95E-07	0.003	25.27
SDF-1A	rs8111708	G	A	0.0696	0.0151	0.6541	3.95E-06	0.002	21.25
SDF-1A	rs867230	A	C	0.1333	0.0158	0.5984	3.49E-17	0.009	71.18
SDF-1A	rs9381563	T	C	-0.0821	0.0148	0.3221	2.93E-08	0.003	30.77
SDF-1A	rs9649710	G	A	0.0676	0.0148	0.3767	4.79E-06	0.002	20.86
SDF-1A	rs9787911	C	T	0.0662	0.0144	0.4274	4.39E-06	0.002	21.13

TNF-A	rs1081105	C	A	0.942	0.0436	0.9692	1.51E-103	0.053	466.80	
TNF-A	rs111278137	A	G	-0.4735	0.0713	0.0139	3.20E-11	0.006	44.10	
TNF-A	rs11168036	G	T	-0.0754	0.0143	0.5169	1.43E-07	0.003	27.80	
TNF-A	rs11767557	C	T	-0.1028	0.0182	0.2177	1.56E-08	0.004	31.90	
TNF-A	rs12151021	G	A	-0.1071	0.0169	0.338	2.56E-10	0.005	40.16	
TNF-A	rs12590654	A	G	-0.0906	0.0157	0.3370	8.73E-09	0.004	33.30	
TNF-A	rs13252043	T	C	0.114	0.0237	0.9066	1.57E-06	0.002	23.14	
TNF-A	rs138727474	T	C	-0.2515	0.0545	0.9761	3.90E-06	0.003	21.30	
TNF-A	rs141739979	T	G	-0.4544	0.0855	0.9861	1.07E-07	0.006	28.25	
TNF-A	rs143429938	T	C	0.3535	0.0769	0.9732	4.25E-06	0.007	21.13	
TNF-A	rs147711004	A	G	1.1354	0.0366	0.0288	1.00E-200	0.072	962.36	
TNF-A	rs150685845	G	A	0.5561	0.0645	0.9871	6.62E-18	0.008	74.33	
TNF-A	rs1582763	A	G	-0.1232	0.0149	0.6372	1.19E-16	0.007	68.37	95.00%
TNF-A	rs17125924	G	A	0.1222	0.0246	0.9205	6.62E-07	0.002	24.68	
TNF-A	rs2830489	T	C	-0.0837	0.0162	0.7038	2.42E-07	0.003	26.69	
TNF-A	rs34665982	C	T	-0.0967	0.0166	0.4901	5.80E-09	0.005	33.93	
TNF-A	rs34971488	A	G	0.094	0.0198	0.2346	2.07E-06	0.003	22.54	
TNF-A	rs35695568	T	G	0.1152	0.0247	0.8986	3.20E-06	0.002	21.75	
TNF-A	rs3740688	T	G	0.0935	0.0144	0.4573	9.70E-11	0.004	42.16	
TNF-A	rs383902	T	C	-0.0698	0.0151	0.6402	3.81E-06	0.002	21.37	
TNF-A	rs3851179	C	T	0.1198	0.0148	0.3708	5.81E-16	0.007	65.52	
TNF-A	rs6014724	G	A	-0.1319	0.0259	0.9105	3.65E-07	0.003	25.94	
TNF-A	rs62039712	A	G	0.1528	0.0288	0.1213	1.17E-07	0.005	28.15	
TNF-A	rs6559689	T	C	0.1585	0.0335	0.9483	2.17E-06	0.002	22.39	
TNF-A	rs6733839	T	C	0.1693	0.0154	0.6203	4.02E-28	0.014	120.86	
TNF-A	rs679515	C	T	-0.1508	0.0183	0.828	1.55E-16	0.006	67.90	

TNF-A	rs72654445	A	G	-0.5425	0.0811	0.0109	2.27E-11	0.006	44.75	
TNF-A	rs72993825	T	C	-0.1763	0.036	0.9404	9.86E-07	0.003	23.98	
TNF-A	rs73223431	T	C	0.0936	0.0153	0.6541	8.34E-10	0.004	37.43	
TNF-A	rs7412	T	C	-0.4673	0.0305	0.9374	6.40E-53	0.026	234.74	
TNF-A	rs7584040	T	C	0.0862	0.0172	0.8131	5.34E-07	0.002	25.12	
TNF-A	rs7618668	A	G	-0.1297	0.0258	0.0815	4.95E-07	0.003	25.27	
TNF-A	rs8111708	G	A	0.0696	0.0151	0.6541	3.95E-06	0.002	21.25	
TNF-A	rs867230	A	C	0.1333	0.0158	0.5984	3.49E-17	0.009	71.18	
TNF-A	rs9381563	T	C	-0.0821	0.0148	0.3221	2.93E-08	0.003	30.77	
TNF-A	rs9649710	G	A	0.0676	0.0148	0.3767	4.79E-06	0.002	20.86	
TNF-A	rs9787911	C	T	0.0662	0.0144	0.4274	4.39E-06	0.002	21.13	
TNF-B	rs11168036	G	T	-0.0754	0.0143	0.5169	1.43E-07	0.003	27.80	
TNF-B	rs11767557	C	T	-0.1028	0.0182	0.2177	1.56E-08	0.004	31.90	
TNF-B	rs12151021	G	A	-0.1071	0.0169	0.338	2.56E-10	0.005	40.16	
TNF-B	rs12590654	A	G	-0.0906	0.0157	0.3370	8.73E-09	0.004	33.30	
TNF-B	rs13252043	T	C	0.114	0.0237	0.9066	1.57E-06	0.002	23.14	
TNF-B	rs147711004	A	G	1.1354	0.0366	0.0288	1.00E-200	0.072	962.36	
TNF-B	rs1582763	A	G	-0.1232	0.0149	0.6372	1.19E-16	0.007	68.37	
TNF-B	rs17125924	G	A	0.1222	0.0246	0.9205	6.62E-07	0.002	24.68	98.70%
TNF-B	rs2830489	T	C	-0.0837	0.0162	0.7038	2.42E-07	0.003	26.69	
TNF-B	rs34665982	C	T	-0.0967	0.0166	0.4901	5.80E-09	0.005	33.93	
TNF-B	rs34971488	A	G	0.094	0.0198	0.2346	2.07E-06	0.003	22.54	
TNF-B	rs35695568	T	G	0.1152	0.0247	0.8986	3.20E-06	0.002	21.75	
TNF-B	rs3740688	T	G	0.0935	0.0144	0.4573	9.70E-11	0.004	42.16	
TNF-B	rs383902	T	C	-0.0698	0.0151	0.6402	3.81E-06	0.002	21.37	
TNF-B	rs3851179	C	T	0.1198	0.0148	0.3708	5.81E-16	0.007	65.52	

TNF-B	rs6014724	G	A	-0.1319	0.0259	0.9105	3.65E-07	0.003	25.94	
TNF-B	rs62039712	A	G	0.1528	0.0288	0.1213	1.17E-07	0.005	28.15	
TNF-B	rs6559689	T	C	0.1585	0.0335	0.9483	2.17E-06	0.002	22.39	
TNF-B	rs6733839	T	C	0.1693	0.0154	0.6203	4.02E-28	0.014	120.86	
TNF-B	rs679515	C	T	-0.1508	0.0183	0.828	1.55E-16	0.006	67.90	
TNF-B	rs72993825	T	C	-0.1763	0.036	0.9404	9.86E-07	0.003	23.98	
TNF-B	rs73223431	T	C	0.0936	0.0153	0.6541	8.34E-10	0.004	37.43	
TNF-B	rs7412	T	C	-0.4673	0.0305	0.9374	6.40E-53	0.026	234.74	
TNF-B	rs7584040	T	C	0.0862	0.0172	0.8131	5.34E-07	0.002	25.12	
TNF-B	rs7618668	A	G	-0.1297	0.0258	0.0815	4.95E-07	0.003	25.27	
TNF-B	rs8111708	G	A	0.0696	0.0151	0.6541	3.95E-06	0.002	21.25	
TNF-B	rs867230	A	C	0.1333	0.0158	0.5984	3.49E-17	0.009	71.18	
TNF-B	rs9381563	T	C	-0.0821	0.0148	0.3221	2.93E-08	0.003	30.77	
TNF-B	rs9649710	G	A	0.0676	0.0148	0.3767	4.79E-06	0.002	20.86	
TNF-B	rs9787911	C	T	0.0662	0.0144	0.4274	4.39E-06	0.002	21.13	
TRAIL	rs1081105	C	A	0.942	0.0436	0.9692	1.51E-103	0.053	466.80	
TRAIL	rs111278137	A	G	-0.4735	0.0713	0.0139	3.20E-11	0.006	44.10	
TRAIL	rs11168036	G	T	-0.0754	0.0143	0.5169	1.43E-07	0.003	27.80	
TRAIL	rs117240937	A	G	-0.3122	0.0672	0.0179	3.35E-06	0.003	21.58	
TRAIL	rs11767557	C	T	-0.1028	0.0182	0.2177	1.56E-08	0.004	31.90	
TRAIL	rs12151021	G	A	-0.1071	0.0169	0.338	2.56E-10	0.005	40.16	70.70%
TRAIL	rs12590654	A	G	-0.0906	0.0157	0.3370	8.73E-09	0.004	33.30	
TRAIL	rs13252043	T	C	0.114	0.0237	0.9066	1.57E-06	0.002	23.14	
TRAIL	rs138727474	T	C	-0.2515	0.0545	0.9761	3.90E-06	0.003	21.30	
TRAIL	rs141739979	T	G	-0.4544	0.0855	0.9861	1.07E-07	0.006	28.25	
TRAIL	rs143429938	T	C	0.3535	0.0769	0.9732	4.25E-06	0.007	21.13	

TRAIL	rs147711004	A	G	1.1354	0.0366	0.0288	1.00E-200	0.072	962.36
TRAIL	rs150685845	G	A	0.5561	0.0645	0.9871	6.62E-18	0.008	74.33
TRAIL	rs1582763	A	G	-0.1232	0.0149	0.6372	1.19E-16	0.007	68.37
TRAIL	rs17125924	G	A	0.1222	0.0246	0.9205	6.62E-07	0.002	24.68
TRAIL	rs2830489	T	C	-0.0837	0.0162	0.7038	2.42E-07	0.003	26.69
TRAIL	rs34665982	C	T	-0.0967	0.0166	0.4901	5.80E-09	0.005	33.93
TRAIL	rs34971488	A	G	0.094	0.0198	0.2346	2.07E-06	0.003	22.54
TRAIL	rs35695568	T	G	0.1152	0.0247	0.8986	3.20E-06	0.002	21.75
TRAIL	rs3740688	T	G	0.0935	0.0144	0.4573	9.70E-11	0.004	42.16
TRAIL	rs383902	T	C	-0.0698	0.0151	0.6402	3.81E-06	0.002	21.37
TRAIL	rs3851179	C	T	0.1198	0.0148	0.3708	5.81E-16	0.007	65.52
TRAIL	rs6014724	G	A	-0.1319	0.0259	0.9105	3.65E-07	0.003	25.94
TRAIL	rs62039712	A	G	0.1528	0.0288	0.1213	1.17E-07	0.005	28.15
TRAIL	rs6559689	T	C	0.1585	0.0335	0.9483	2.17E-06	0.002	22.39
TRAIL	rs6733839	T	C	0.1693	0.0154	0.6203	4.02E-28	0.014	120.86
TRAIL	rs679515	C	T	-0.1508	0.0183	0.828	1.55E-16	0.006	67.90
TRAIL	rs72654445	A	G	-0.5425	0.0811	0.0109	2.27E-11	0.006	44.75
TRAIL	rs72993825	T	C	-0.1763	0.036	0.9404	9.86E-07	0.003	23.98
TRAIL	rs73223431	T	C	0.0936	0.0153	0.6541	8.34E-10	0.004	37.43
TRAIL	rs7412	T	C	-0.4673	0.0305	0.9374	6.40E-53	0.026	234.74
TRAIL	rs7584040	T	C	0.0862	0.0172	0.8131	5.34E-07	0.002	25.12
TRAIL	rs7618668	A	G	-0.1297	0.0258	0.0815	4.95E-07	0.003	25.27
TRAIL	rs8111708	G	A	0.0696	0.0151	0.6541	3.95E-06	0.002	21.25
TRAIL	rs867230	A	C	0.1333	0.0158	0.5984	3.49E-17	0.009	71.18
TRAIL	rs9381563	T	C	-0.0821	0.0148	0.3221	2.93E-08	0.003	30.77
TRAIL	rs9649710	G	A	0.0676	0.0148	0.3767	4.79E-06	0.002	20.86

TRAIL	rs9787911	C	T	0.0662	0.0144	0.4274	4.39E-06	0.002	21.13	
VEGF	rs1081105	C	A	0.942	0.0436	0.9692	1.51E-103	0.053	466.80	
VEGF	rs111278137	A	G	-0.4735	0.0713	0.0139	3.20E-11	0.006	44.10	
VEGF	rs11168036	G	T	-0.0754	0.0143	0.5169	1.43E-07	0.003	27.80	
VEGF	rs117240937	A	G	-0.3122	0.0672	0.0179	3.35E-06	0.003	21.58	
VEGF	rs11767557	C	T	-0.1028	0.0182	0.2177	1.56E-08	0.004	31.90	
VEGF	rs12151021	G	A	-0.1071	0.0169	0.338	2.56E-10	0.005	40.16	
VEGF	rs12590654	A	G	-0.0906	0.0157	0.3370	8.73E-09	0.004	33.30	
VEGF	rs13252043	T	C	0.114	0.0237	0.9066	1.57E-06	0.002	23.14	
VEGF	rs138727474	T	C	-0.2515	0.0545	0.9761	3.90E-06	0.003	21.30	
VEGF	rs141739979	T	G	-0.4544	0.0855	0.9861	1.07E-07	0.006	28.25	
VEGF	rs143429938	T	C	0.3535	0.0769	0.9732	4.25E-06	0.007	21.13	
VEGF	rs147711004	A	G	1.1354	0.0366	0.0288	1.00E-200	0.072	962.36	86.50%
VEGF	rs150685845	G	A	0.5561	0.0645	0.9871	6.62E-18	0.008	74.33	
VEGF	rs1582763	A	G	-0.1232	0.0149	0.6372	1.19E-16	0.007	68.37	
VEGF	rs17125924	G	A	0.1222	0.0246	0.9205	6.62E-07	0.002	24.68	
VEGF	rs2830489	T	C	-0.0837	0.0162	0.7038	2.42E-07	0.003	26.69	
VEGF	rs34665982	C	T	-0.0967	0.0166	0.4901	5.80E-09	0.005	33.93	
VEGF	rs34971488	A	G	0.094	0.0198	0.2346	2.07E-06	0.003	22.54	
VEGF	rs35695568	T	G	0.1152	0.0247	0.8986	3.20E-06	0.002	21.75	
VEGF	rs3740688	T	G	0.0935	0.0144	0.4573	9.70E-11	0.004	42.16	
VEGF	rs383902	T	C	-0.0698	0.0151	0.6402	3.81E-06	0.002	21.37	
VEGF	rs3851179	C	T	0.1198	0.0148	0.3708	5.81E-16	0.007	65.52	
VEGF	rs6014724	G	A	-0.1319	0.0259	0.9105	3.65E-07	0.003	25.94	
VEGF	rs62039712	A	G	0.1528	0.0288	0.1213	1.17E-07	0.005	28.15	

VEGF	rs6559689	T	C	0.1585	0.0335	0.9483	2.17E-06	0.002	22.39
VEGF	rs6733839	T	C	0.1693	0.0154	0.6203	4.02E-28	0.014	120.86
VEGF	rs679515	C	T	-0.1508	0.0183	0.828	1.55E-16	0.006	67.90
VEGF	rs72654445	A	G	-0.5425	0.0811	0.0109	2.27E-11	0.006	44.75
VEGF	rs72993825	T	C	-0.1763	0.036	0.9404	9.86E-07	0.003	23.98
VEGF	rs73223431	T	C	0.0936	0.0153	0.6541	8.34E-10	0.004	37.43
VEGF	rs7412	T	C	-0.4673	0.0305	0.9374	6.40E-53	0.026	234.74
VEGF	rs7584040	T	C	0.0862	0.0172	0.8131	5.34E-07	0.002	25.12
VEGF	rs7618668	A	G	-0.1297	0.0258	0.0815	4.95E-07	0.003	25.27
VEGF	rs8111708	G	A	0.0696	0.0151	0.6541	3.95E-06	0.002	21.25
VEGF	rs867230	A	C	0.1333	0.0158	0.5984	3.49E-17	0.009	71.18
VEGF	rs9381563	T	C	-0.0821	0.0148	0.3221	2.93E-08	0.003	30.77
VEGF	rs9649710	G	A	0.0676	0.0148	0.3767	4.79E-06	0.002	20.86
VEGF	rs9787911	C	T	0.0662	0.0144	0.4274	4.39E-06	0.002	21.13

Supplementary Table 5. Detailed information for risk of PD-associated SNPs with circulating cytokines.

Outcomes	SNP	Effect Allele	Other Allele	Beta	Se	EAF	*P-value*	*R2*	*F*	Power
BNGF	rs10513789	G	T	-0.1596	0.0219	0.1978	3.18E-13	0.008	53.11	88.90%
BNGF	rs10756905	T	C	0.1011	0.0196	0.7654	2.46E-07	0.004	26.61	
BNGF	rs10766301	T	C	0.0907	0.0192	0.6153	2.21E-06	0.004	22.32	
BNGF	rs10847864	T	G	0.1274	0.0179	0.3390	9.81E-13	0.007	50.66	
BNGF	rs111972941	G	A	0.2482	0.0514	0.9453	1.37E-06	0.006	23.32	
BNGF	rs112413063	C	T	-0.1813	0.0396	0.0557	4.62E-06	0.003	20.96	
BNGF	rs12929797	T	C	0.082	0.017	0.9771	1.32E-06	0.000	23.27	
BNGF	rs142660239	T	G	-0.3772	0.0779	0.0179	1.28E-06	0.005	23.45	
BNGF	rs144814361	T	C	0.4411	0.068	0.9871	9.07E-11	0.005	42.08	
BNGF	rs2208485	A	G	-0.0936	0.0182	0.3926	2.65E-07	0.004	26.45	
BNGF	rs2248244	A	G	0.1001	0.0211	0.2604	2.00E-06	0.004	22.51	
BNGF	rs2295547	C	A	-0.0894	0.0182	0.6630	8.77E-07	0.004	24.13	
BNGF	rs28370649	G	A	0.2836	0.0547	0.9831	2.20E-07	0.003	26.88	
BNGF	rs2949760	A	G	0.0915	0.0193	0.3519	2.00E-06	0.004	22.48	
BNGF	rs34311866	C	T	0.2272	0.0231	0.8131	7.97E-23	0.016	96.74	
BNGF	rs34679758	G	A	-0.1275	0.0259	0.8728	8.72E-07	0.004	24.23	
BNGF	rs356203	T	C	-0.2398	0.0178	0.6133	3.01E-41	0.027	181.49	
BNGF	rs35749011	A	G	0.7508	0.0659	0.0219	5.02E-30	0.024	129.80	
BNGF	rs41286192	G	A	0.3164	0.067	0.9642	2.30E-06	0.007	22.30	
BNGF	rs4488803	A	G	-0.1136	0.0199	0.6272	1.08E-08	0.006	32.59	
BNGF	rs4588066	A	G	0.1046	0.0178	0.3390	4.45E-09	0.005	34.53	
BNGF	rs4698412	A	G	0.1258	0.0168	0.5596	7.05E-14	0.008	56.07	
BNGF	rs4774417	A	G	0.1052	0.0192	0.7137	4.63E-08	0.005	30.02	
BNGF	rs4810687	T	G	0.0932	0.0187	0.4135	6.27E-07	0.004	24.84	

BNGF	rs4836108	A	G	0.1094	0.0225	0.5070	1.20E-06	0.006	23.64	
BNGF	rs4851487	T	C	0.0808	0.0174	0.5755	3.25E-06	0.003	21.56	
BNGF	rs61835654	C	T	0.1032	0.0216	0.2535	1.85E-06	0.004	22.83	
BNGF	rs620490	G	T	-0.1174	0.019	0.2992	6.46E-10	0.006	38.18	
BNGF	rs6741007	G	T	-0.1233	0.0175	0.4970	2.09E-12	0.008	49.64	
BNGF	rs6808178	C	T	-0.0864	0.0174	0.6292	7.20E-07	0.003	24.66	
BNGF	rs73032517	G	A	0.2706	0.0558	0.9742	1.27E-06	0.004	23.52	
BNGF	rs75505347	T	C	0.3917	0.0674	0.9791	6.12E-09	0.006	33.77	
BNGF	rs75646569	G	T	0.1916	0.0266	0.1252	5.62E-13	0.008	51.88	
BNGF	rs7695720	C	A	-0.1255	0.0208	0.7644	1.53E-09	0.006	36.40	
BNGF	rs7818035	A	G	-0.693	0.1429	0.0209	1.24E-06	0.020	23.52	
BNGF	rs79436216	G	A	0.2816	0.0603	0.9583	3.02E-06	0.006	21.81	
BNGF	rs858295	G	A	-0.1039	0.0176	0.3708	3.83E-09	0.005	34.85	
BNGF	rs9840232	T	C	0.1157	0.0233	0.8121	6.56E-07	0.004	24.66	
BNGF	rs9845968	G	A	0.0842	0.0175	0.4483	1.42E-06	0.004	23.15	
CTACK	rs10513789	G	T	-0.1596	0.0219	0.1978	3.18E-13	0.008	53.11	
CTACK	rs10756905	T	C	0.1011	0.0196	0.7654	2.46E-07	0.004	26.61	
CTACK	rs10766301	T	C	0.0907	0.0192	0.6153	2.21E-06	0.004	22.32	
CTACK	rs10847864	T	G	0.1274	0.0179	0.3390	9.81E-13	0.007	50.66	
CTACK	rs111972941	G	A	0.2482	0.0514	0.9453	1.37E-06	0.006	23.32	
CTACK	rs112413063	C	T	-0.1813	0.0396	0.0557	4.62E-06	0.003	20.96	10.80%
CTACK	rs12929797	T	C	0.082	0.017	0.9771	1.32E-06	0.000	23.27	
CTACK	rs142660239	T	G	-0.3772	0.0779	0.0179	1.28E-06	0.005	23.45	
CTACK	rs144814361	T	C	0.4411	0.068	0.9871	9.07E-11	0.005	42.08	
CTACK	rs182621729	T	C	0.3083	0.0633	0.9662	1.11E-06	0.006	23.72	
CTACK	rs2208485	A	G	-0.0936	0.0182	0.3926	2.65E-07	0.004	26.45	

CTACK	rs2248244	A	G	0.1001	0.0211	0.2604	2.00E-06	0.004	22.51
CTACK	rs2295547	C	A	-0.0894	0.0182	0.6630	8.77E-07	0.004	24.13
CTACK	rs28370649	G	A	0.2836	0.0547	0.9831	2.20E-07	0.003	26.88
CTACK	rs2949760	A	G	0.0915	0.0193	0.3519	2.00E-06	0.004	22.48
CTACK	rs34311866	C	T	0.2272	0.0231	0.8131	7.97E-23	0.016	96.74
CTACK	rs34679758	G	A	-0.1275	0.0259	0.8728	8.72E-07	0.004	24.23
CTACK	rs356203	T	C	-0.2398	0.0178	0.6133	3.01E-41	0.027	181.49
CTACK	rs35749011	A	G	0.7508	0.0659	0.0219	5.02E-30	0.024	129.80
CTACK	rs41286192	G	A	0.3164	0.067	0.9642	2.30E-06	0.007	22.30
CTACK	rs4488803	A	G	-0.1136	0.0199	0.6272	1.08E-08	0.006	32.59
CTACK	rs4588066	A	G	0.1046	0.0178	0.3390	4.45E-09	0.005	34.53
CTACK	rs4698412	A	G	0.1258	0.0168	0.5596	7.05E-14	0.008	56.07
CTACK	rs4774417	A	G	0.1052	0.0192	0.7137	4.63E-08	0.005	30.02
CTACK	rs4810687	T	G	0.0932	0.0187	0.4135	6.27E-07	0.004	24.84
CTACK	rs4836108	A	G	0.1094	0.0225	0.5070	1.20E-06	0.006	23.64
CTACK	rs4851487	T	C	0.0808	0.0174	0.5755	3.25E-06	0.003	21.56
CTACK	rs61835654	C	T	0.1032	0.0216	0.2535	1.85E-06	0.004	22.83
CTACK	rs620490	G	T	-0.1174	0.019	0.2992	6.46E-10	0.006	38.18
CTACK	rs6715875	C	T	0.2902	0.0599	0.0249	1.25E-06	0.004	23.47
CTACK	rs6741007	G	T	-0.1233	0.0175	0.4970	2.09E-12	0.008	49.64
CTACK	rs6808178	C	T	-0.0864	0.0174	0.6292	7.20E-07	0.003	24.66
CTACK	rs73032517	G	A	0.2706	0.0558	0.9742	1.27E-06	0.004	23.52
CTACK	rs75505347	T	C	0.3917	0.0674	0.9791	6.12E-09	0.006	33.77
CTACK	rs75646569	G	T	0.1916	0.0266	0.1252	5.62E-13	0.008	51.88
CTACK	rs7695720	C	A	-0.1255	0.0208	0.7644	1.53E-09	0.006	36.40
CTACK	rs7818035	A	G	-0.693	0.1429	0.0209	1.24E-06	0.020	23.52

CTACK	rs79436216	G	A	0.2816	0.0603	0.9583	3.02E-06	0.006	21.81	
CTACK	rs858295	G	A	-0.1039	0.0176	0.3708	3.83E-09	0.005	34.85	
CTACK	rs9840232	T	C	0.1157	0.0233	0.8121	6.56E-07	0.004	24.66	
CTACK	rs9845968	G	A	0.0842	0.0175	0.4483	1.42E-06	0.004	23.15	
EOTAXIN	rs10513789	G	T	-0.1596	0.0219	0.1978	3.18E-13	0.008	53.11	
EOTAXIN	rs10756905	T	C	0.1011	0.0196	0.7654	2.46E-07	0.004	26.61	
EOTAXIN	rs10766301	T	C	0.0907	0.0192	0.6153	2.21E-06	0.004	22.32	
EOTAXIN	rs10847864	T	G	0.1274	0.0179	0.3390	9.81E-13	0.007	50.66	
EOTAXIN	rs111972941	G	A	0.2482	0.0514	0.9453	1.37E-06	0.006	23.32	
EOTAXIN	rs112413063	C	T	-0.1813	0.0396	0.0557	4.62E-06	0.003	20.96	
EOTAXIN	rs114797774	T	C	0.3901	0.0796	0.9771	9.43E-07	0.007	24.02	
EOTAXIN	rs12929797	T	C	0.082	0.017	0.9771	1.32E-06	0.000	23.27	
EOTAXIN	rs142660239	T	G	-0.3772	0.0779	0.0179	1.28E-06	0.005	23.45	
EOTAXIN	rs144814361	T	C	0.4411	0.068	0.9871	9.07E-11	0.005	42.08	
EOTAXIN	rs182621729	T	C	0.3083	0.0633	0.9662	1.11E-06	0.006	23.72	100.00%
EOTAXIN	rs2208485	A	G	-0.0936	0.0182	0.3926	2.65E-07	0.004	26.45	
EOTAXIN	rs2248244	A	G	0.1001	0.0211	0.2604	2.00E-06	0.004	22.51	
EOTAXIN	rs2295547	C	A	-0.0894	0.0182	0.6630	8.77E-07	0.004	24.13	
EOTAXIN	rs28370649	G	A	0.2836	0.0547	0.9831	2.20E-07	0.003	26.88	
EOTAXIN	rs2949760	A	G	0.0915	0.0193	0.3519	2.00E-06	0.004	22.48	
EOTAXIN	rs34311866	C	T	0.2272	0.0231	0.8131	7.97E-23	0.016	96.74	
EOTAXIN	rs34679758	G	A	-0.1275	0.0259	0.8728	8.72E-07	0.004	24.23	
EOTAXIN	rs356203	T	C	-0.2398	0.0178	0.6133	3.01E-41	0.027	181.49	
EOTAXIN	rs35749011	A	G	0.7508	0.0659	0.0219	5.02E-30	0.024	129.80	
EOTAXIN	rs41286192	G	A	0.3164	0.067	0.9642	2.30E-06	0.007	22.30	
EOTAXIN	rs4488803	A	G	-0.1136	0.0199	0.6272	1.08E-08	0.006	32.59	

EOTAXIN	rs4588066	A	G	0.1046	0.0178	0.3390	4.45E-09	0.005	34.53	
EOTAXIN	rs4698412	A	G	0.1258	0.0168	0.5596	7.05E-14	0.008	56.07	
EOTAXIN	rs4774417	A	G	0.1052	0.0192	0.7137	4.63E-08	0.005	30.02	
EOTAXIN	rs4810687	T	G	0.0932	0.0187	0.4135	6.27E-07	0.004	24.84	
EOTAXIN	rs4836108	A	G	0.1094	0.0225	0.5070	1.20E-06	0.006	23.64	
EOTAXIN	rs4851487	T	C	0.0808	0.0174	0.5755	3.25E-06	0.003	21.56	
EOTAXIN	rs61835654	C	T	0.1032	0.0216	0.2535	1.85E-06	0.004	22.83	
EOTAXIN	rs620490	G	T	-0.1174	0.019	0.2992	6.46E-10	0.006	38.18	
EOTAXIN	rs6741007	G	T	-0.1233	0.0175	0.4970	2.09E-12	0.008	49.64	
EOTAXIN	rs6808178	C	T	-0.0864	0.0174	0.6292	7.20E-07	0.003	24.66	
EOTAXIN	rs73032517	G	A	0.2706	0.0558	0.9742	1.27E-06	0.004	23.52	
EOTAXIN	rs75505347	T	C	0.3917	0.0674	0.9791	6.12E-09	0.006	33.77	
EOTAXIN	rs75646569	G	T	0.1916	0.0266	0.1252	5.62E-13	0.008	51.88	
EOTAXIN	rs7695720	C	A	-0.1255	0.0208	0.7644	1.53E-09	0.006	36.40	
EOTAXIN	rs7818035	A	G	-0.693	0.1429	0.0209	1.24E-06	0.020	23.52	
EOTAXIN	rs79436216	G	A	0.2816	0.0603	0.9583	3.02E-06	0.006	21.81	
EOTAXIN	rs858295	G	A	-0.1039	0.0176	0.3708	3.83E-09	0.005	34.85	
EOTAXIN	rs9840232	T	C	0.1157	0.0233	0.8121	6.56E-07	0.004	24.66	
EOTAXIN	rs9845968	G	A	0.0842	0.0175	0.4483	1.42E-06	0.004	23.15	
FGF	rs10513789	G	T	-0.1596	0.0219	0.1978	3.18E-13	0.008	53.11	
FGF	rs10756905	T	C	0.1011	0.0196	0.7654	2.46E-07	0.004	26.61	
FGF	rs10766301	T	C	0.0907	0.0192	0.6153	2.21E-06	0.004	22.32	
FGF	rs10847864	T	G	0.1274	0.0179	0.3390	9.81E-13	0.007	50.66	5.50%
FGF	rs111972941	G	A	0.2482	0.0514	0.9453	1.37E-06	0.006	23.32	
FGF	rs112413063	C	T	-0.1813	0.0396	0.0557	4.62E-06	0.003	20.96	
FGF	rs12929797	T	C	0.082	0.017	0.9771	1.32E-06	0.000	23.27	

FGF	rs142660239	T	G	-0.3772	0.0779	0.0179	1.28E-06	0.005	23.45
FGF	rs144814361	T	C	0.4411	0.068	0.9871	9.07E-11	0.005	42.08
FGF	rs182621729	T	C	0.3083	0.0633	0.9662	1.11E-06	0.006	23.72
FGF	rs2208485	A	G	-0.0936	0.0182	0.3926	2.65E-07	0.004	26.45
FGF	rs2248244	A	G	0.1001	0.0211	0.2604	2.00E-06	0.004	22.51
FGF	rs2295547	C	A	-0.0894	0.0182	0.6630	8.77E-07	0.004	24.13
FGF	rs28370649	G	A	0.2836	0.0547	0.9831	2.20E-07	0.003	26.88
FGF	rs2949760	A	G	0.0915	0.0193	0.3519	2.00E-06	0.004	22.48
FGF	rs34311866	C	T	0.2272	0.0231	0.8131	7.97E-23	0.016	96.74
FGF	rs34679758	G	A	-0.1275	0.0259	0.8728	8.72E-07	0.004	24.23
FGF	rs356203	T	C	-0.2398	0.0178	0.6133	3.01E-41	0.027	181.49
FGF	rs35749011	A	G	0.7508	0.0659	0.0219	5.02E-30	0.024	129.80
FGF	rs41286192	G	A	0.3164	0.067	0.9642	2.30E-06	0.007	22.30
FGF	rs4488803	A	G	-0.1136	0.0199	0.6272	1.08E-08	0.006	32.59
FGF	rs4588066	A	G	0.1046	0.0178	0.3390	4.45E-09	0.005	34.53
FGF	rs4698412	A	G	0.1258	0.0168	0.5596	7.05E-14	0.008	56.07
FGF	rs4774417	A	G	0.1052	0.0192	0.7137	4.63E-08	0.005	30.02
FGF	rs4810687	T	G	0.0932	0.0187	0.4135	6.27E-07	0.004	24.84
FGF	rs4836108	A	G	0.1094	0.0225	0.5070	1.20E-06	0.006	23.64
FGF	rs4851487	T	C	0.0808	0.0174	0.5755	3.25E-06	0.003	21.56
FGF	rs61835654	C	T	0.1032	0.0216	0.2535	1.85E-06	0.004	22.83
FGF	rs620490	G	T	-0.1174	0.019	0.2992	6.46E-10	0.006	38.18
FGF	rs6741007	G	T	-0.1233	0.0175	0.4970	2.09E-12	0.008	49.64
FGF	rs6808178	C	T	-0.0864	0.0174	0.6292	7.20E-07	0.003	24.66
FGF	rs73032517	G	A	0.2706	0.0558	0.9742	1.27E-06	0.004	23.52
FGF	rs75505347	T	C	0.3917	0.0674	0.9791	6.12E-09	0.006	33.77

FGF	rs75646569	G	T	0.1916	0.0266	0.1252	5.62E-13	0.008	51.88	
FGF	rs7695720	C	A	-0.1255	0.0208	0.7644	1.53E-09	0.006	36.40	
FGF	rs7818035	A	G	-0.693	0.1429	0.0209	1.24E-06	0.020	23.52	
FGF	rs79436216	G	A	0.2816	0.0603	0.9583	3.02E-06	0.006	21.81	
FGF	rs858295	G	A	-0.1039	0.0176	0.3708	3.83E-09	0.005	34.85	
FGF	rs9840232	T	C	0.1157	0.0233	0.8121	6.56E-07	0.004	24.66	
FGF	rs9845968	G	A	0.0842	0.0175	0.4483	1.42E-06	0.004	23.15	
G-CSF	rs10513789	G	T	-0.1596	0.0219	0.1978	3.18E-13	0.008	53.11	
G-CSF	rs10756905	T	C	0.1011	0.0196	0.7654	2.46E-07	0.004	26.61	
G-CSF	rs10766301	T	C	0.0907	0.0192	0.6153	2.21E-06	0.004	22.32	
G-CSF	rs10847864	T	G	0.1274	0.0179	0.3390	9.81E-13	0.007	50.66	
G-CSF	rs111972941	G	A	0.2482	0.0514	0.9453	1.37E-06	0.006	23.32	
G-CSF	rs112413063	C	T	-0.1813	0.0396	0.0557	4.62E-06	0.003	20.96	
G-CSF	rs12929797	T	C	0.082	0.017	0.9771	1.32E-06	0.000	23.27	
G-CSF	rs142660239	T	G	-0.3772	0.0779	0.0179	1.28E-06	0.005	23.45	
G-CSF	rs144814361	T	C	0.4411	0.068	0.9871	9.07E-11	0.005	42.08	
G-CSF	rs182621729	T	C	0.3083	0.0633	0.9662	1.11E-06	0.006	23.72	100.00%
G-CSF	rs2208485	A	G	-0.0936	0.0182	0.3926	2.65E-07	0.004	26.45	
G-CSF	rs2248244	A	G	0.1001	0.0211	0.2604	2.00E-06	0.004	22.51	
G-CSF	rs2295547	C	A	-0.0894	0.0182	0.6630	8.77E-07	0.004	24.13	
G-CSF	rs28370649	G	A	0.2836	0.0547	0.9831	2.20E-07	0.003	26.88	
G-CSF	rs2949760	A	G	0.0915	0.0193	0.3519	2.00E-06	0.004	22.48	
G-CSF	rs34311866	C	T	0.2272	0.0231	0.8131	7.97E-23	0.016	96.74	
G-CSF	rs34679758	G	A	-0.1275	0.0259	0.8728	8.72E-07	0.004	24.23	
G-CSF	rs356203	T	C	-0.2398	0.0178	0.6133	3.01E-41	0.027	181.49	
G-CSF	rs35749011	A	G	0.7508	0.0659	0.0219	5.02E-30	0.024	129.80	

G-CSF	rs41286192	G	A	0.3164	0.067	0.9642	2.30E-06	0.007	22.30	
G-CSF	rs4488803	A	G	-0.1136	0.0199	0.6272	1.08E-08	0.006	32.59	
G-CSF	rs4588066	A	G	0.1046	0.0178	0.3390	4.45E-09	0.005	34.53	
G-CSF	rs4698412	A	G	0.1258	0.0168	0.5596	7.05E-14	0.008	56.07	
G-CSF	rs4774417	A	G	0.1052	0.0192	0.7137	4.63E-08	0.005	30.02	
G-CSF	rs4810687	T	G	0.0932	0.0187	0.4135	6.27E-07	0.004	24.84	
G-CSF	rs4836108	A	G	0.1094	0.0225	0.5070	1.20E-06	0.006	23.64	
G-CSF	rs4851487	T	C	0.0808	0.0174	0.5755	3.25E-06	0.003	21.56	
G-CSF	rs61835654	C	T	0.1032	0.0216	0.2535	1.85E-06	0.004	22.83	
G-CSF	rs620490	G	T	-0.1174	0.019	0.2992	6.46E-10	0.006	38.18	
G-CSF	rs6741007	G	T	-0.1233	0.0175	0.4970	2.09E-12	0.008	49.64	
G-CSF	rs6808178	C	T	-0.0864	0.0174	0.6292	7.20E-07	0.003	24.66	
G-CSF	rs73032517	G	A	0.2706	0.0558	0.9742	1.27E-06	0.004	23.52	
G-CSF	rs75505347	T	C	0.3917	0.0674	0.9791	6.12E-09	0.006	33.77	
G-CSF	rs75646569	G	T	0.1916	0.0266	0.1252	5.62E-13	0.008	51.88	
G-CSF	rs7695720	C	A	-0.1255	0.0208	0.7644	1.53E-09	0.006	36.40	
G-CSF	rs7818035	A	G	-0.693	0.1429	0.0209	1.24E-06	0.020	23.52	
G-CSF	rs79436216	G	A	0.2816	0.0603	0.9583	3.02E-06	0.006	21.81	
G-CSF	rs858295	G	A	-0.1039	0.0176	0.3708	3.83E-09	0.005	34.85	
G-CSF	rs9840232	T	C	0.1157	0.0233	0.8121	6.56E-07	0.004	24.66	
G-CSF	rs9845968	G	A	0.0842	0.0175	0.4483	1.42E-06	0.004	23.15	
GROA	rs10513789	G	T	-0.1596	0.0219	0.1978	3.18E-13	0.008	53.11	
GROA	rs10756905	T	C	0.1011	0.0196	0.7654	2.46E-07	0.004	26.61	
GROA	rs10766301	T	C	0.0907	0.0192	0.6153	2.21E-06	0.004	22.32	100.00%
GROA	rs10847864	T	G	0.1274	0.0179	0.3390	9.81E-13	0.007	50.66	
GROA	rs111972941	G	A	0.2482	0.0514	0.9453	1.37E-06	0.006	23.32	

GROA	rs112413063	C	T	-0.1813	0.0396	0.0557	4.62E-06	0.003	20.96
GROA	rs12929797	T	C	0.082	0.017	0.9771	1.32E-06	0.000	23.27
GROA	rs142660239	T	G	-0.3772	0.0779	0.0179	1.28E-06	0.005	23.45
GROA	rs144814361	T	C	0.4411	0.068	0.9871	9.07E-11	0.005	42.08
GROA	rs182621729	T	C	0.3083	0.0633	0.9662	1.11E-06	0.006	23.72
GROA	rs2208485	A	G	-0.0936	0.0182	0.3926	2.65E-07	0.004	26.45
GROA	rs2248244	A	G	0.1001	0.0211	0.2604	2.00E-06	0.004	22.51
GROA	rs2295547	C	A	-0.0894	0.0182	0.6630	8.77E-07	0.004	24.13
GROA	rs28370649	G	A	0.2836	0.0547	0.9831	2.20E-07	0.003	26.88
GROA	rs2949760	A	G	0.0915	0.0193	0.3519	2.00E-06	0.004	22.48
GROA	rs34311866	C	T	0.2272	0.0231	0.8131	7.97E-23	0.016	96.74
GROA	rs34679758	G	A	-0.1275	0.0259	0.8728	8.72E-07	0.004	24.23
GROA	rs356203	T	C	-0.2398	0.0178	0.6133	3.01E-41	0.027	181.49
GROA	rs35749011	A	G	0.7508	0.0659	0.0219	5.02E-30	0.024	129.80
GROA	rs41286192	G	A	0.3164	0.067	0.9642	2.30E-06	0.007	22.30
GROA	rs4488803	A	G	-0.1136	0.0199	0.6272	1.08E-08	0.006	32.59
GROA	rs4588066	A	G	0.1046	0.0178	0.3390	4.45E-09	0.005	34.53
GROA	rs4698412	A	G	0.1258	0.0168	0.5596	7.05E-14	0.008	56.07
GROA	rs4774417	A	G	0.1052	0.0192	0.7137	4.63E-08	0.005	30.02
GROA	rs4810687	T	G	0.0932	0.0187	0.4135	6.27E-07	0.004	24.84
GROA	rs4836108	A	G	0.1094	0.0225	0.5070	1.20E-06	0.006	23.64
GROA	rs4851487	T	C	0.0808	0.0174	0.5755	3.25E-06	0.003	21.56
GROA	rs61835654	C	T	0.1032	0.0216	0.2535	1.85E-06	0.004	22.83
GROA	rs620490	G	T	-0.1174	0.019	0.2992	6.46E-10	0.006	38.18
GROA	rs6741007	G	T	-0.1233	0.0175	0.4970	2.09E-12	0.008	49.64
GROA	rs6808178	C	T	-0.0864	0.0174	0.6292	7.20E-07	0.003	24.66

GROA	rs73032517	G	A	0.2706	0.0558	0.9742	1.27E-06	0.004	23.52	
GROA	rs75505347	T	C	0.3917	0.0674	0.9791	6.12E-09	0.006	33.77	
GROA	rs75646569	G	T	0.1916	0.0266	0.1252	5.62E-13	0.008	51.88	
GROA	rs7695720	C	A	-0.1255	0.0208	0.7644	1.53E-09	0.006	36.40	
GROA	rs7818035	A	G	-0.693	0.1429	0.0209	1.24E-06	0.020	23.52	
GROA	rs79436216	G	A	0.2816	0.0603	0.9583	3.02E-06	0.006	21.81	
GROA	rs858295	G	A	-0.1039	0.0176	0.3708	3.83E-09	0.005	34.85	
GROA	rs9840232	T	C	0.1157	0.0233	0.8121	6.56E-07	0.004	24.66	
GROA	rs9845968	G	A	0.0842	0.0175	0.4483	1.42E-06	0.004	23.15	
HGF	rs10513789	G	T	-0.1596	0.0219	0.1978	3.18E-13	0.008	53.11	
HGF	rs10756905	T	C	0.1011	0.0196	0.7654	2.46E-07	0.004	26.61	
HGF	rs10766301	T	C	0.0907	0.0192	0.6153	2.21E-06	0.004	22.32	
HGF	rs10847864	T	G	0.1274	0.0179	0.3390	9.81E-13	0.007	50.66	
HGF	rs111972941	G	A	0.2482	0.0514	0.9453	1.37E-06	0.006	23.32	
HGF	rs112413063	C	T	-0.1813	0.0396	0.0557	4.62E-06	0.003	20.96	
HGF	rs12929797	T	C	0.082	0.017	0.9771	1.32E-06	0.000	23.27	
HGF	rs142660239	T	G	-0.3772	0.0779	0.0179	1.28E-06	0.005	23.45	
HGF	rs144814361	T	C	0.4411	0.068	0.9871	9.07E-11	0.005	42.08	99.60%
HGF	rs182621729	T	C	0.3083	0.0633	0.9662	1.11E-06	0.006	23.72	
HGF	rs2208485	A	G	-0.0936	0.0182	0.3926	2.65E-07	0.004	26.45	
HGF	rs2248244	A	G	0.1001	0.0211	0.2604	2.00E-06	0.004	22.51	
HGF	rs2295547	C	A	-0.0894	0.0182	0.6630	8.77E-07	0.004	24.13	
HGF	rs28370649	G	A	0.2836	0.0547	0.9831	2.20E-07	0.003	26.88	
HGF	rs2949760	A	G	0.0915	0.0193	0.3519	2.00E-06	0.004	22.48	
HGF	rs34311866	C	T	0.2272	0.0231	0.8131	7.97E-23	0.016	96.74	
HGF	rs34679758	G	A	-0.1275	0.0259	0.8728	8.72E-07	0.004	24.23	

HGF	rs356203	T	C	-0.2398	0.0178	0.6133	3.01E-41	0.027	181.49	
HGF	rs35749011	A	G	0.7508	0.0659	0.0219	5.02E-30	0.024	129.80	
HGF	rs41286192	G	A	0.3164	0.067	0.9642	2.30E-06	0.007	22.30	
HGF	rs4488803	A	G	-0.1136	0.0199	0.6272	1.08E-08	0.006	32.59	
HGF	rs4588066	A	G	0.1046	0.0178	0.3390	4.45E-09	0.005	34.53	
HGF	rs4698412	A	G	0.1258	0.0168	0.5596	7.05E-14	0.008	56.07	
HGF	rs4774417	A	G	0.1052	0.0192	0.7137	4.63E-08	0.005	30.02	
HGF	rs4810687	T	G	0.0932	0.0187	0.4135	6.27E-07	0.004	24.84	
HGF	rs4836108	A	G	0.1094	0.0225	0.5070	1.20E-06	0.006	23.64	
HGF	rs4851487	T	C	0.0808	0.0174	0.5755	3.25E-06	0.003	21.56	
HGF	rs61835654	C	T	0.1032	0.0216	0.2535	1.85E-06	0.004	22.83	
HGF	rs620490	G	T	-0.1174	0.019	0.2992	6.46E-10	0.006	38.18	
HGF	rs6741007	G	T	-0.1233	0.0175	0.4970	2.09E-12	0.008	49.64	
HGF	rs6808178	C	T	-0.0864	0.0174	0.6292	7.20E-07	0.003	24.66	
HGF	rs73032517	G	A	0.2706	0.0558	0.9742	1.27E-06	0.004	23.52	
HGF	rs75505347	T	C	0.3917	0.0674	0.9791	6.12E-09	0.006	33.77	
HGF	rs75646569	G	T	0.1916	0.0266	0.1252	5.62E-13	0.008	51.88	
HGF	rs7695720	C	A	-0.1255	0.0208	0.7644	1.53E-09	0.006	36.40	
HGF	rs7818035	A	G	-0.693	0.1429	0.0209	1.24E-06	0.020	23.52	
HGF	rs79436216	G	A	0.2816	0.0603	0.9583	3.02E-06	0.006	21.81	
HGF	rs858295	G	A	-0.1039	0.0176	0.3708	3.83E-09	0.005	34.85	
HGF	rs9840232	T	C	0.1157	0.0233	0.8121	6.56E-07	0.004	24.66	
HGF	rs9845968	G	A	0.0842	0.0175	0.4483	1.42E-06	0.004	23.15	
IFN-G	rs10513789	G	T	-0.1596	0.0219	0.1978	3.18E-13	0.008	53.11	
IFN-G	rs10756905	T	C	0.1011	0.0196	0.7654	2.46E-07	0.004	26.61	99.70%
IFN-G	rs10766301	T	C	0.0907	0.0192	0.6153	2.21E-06	0.004	22.32	

IFN-G	rs10847864	T	G	0.1274	0.0179	0.3390	9.81E-13	0.007	50.66
IFN-G	rs111972941	G	A	0.2482	0.0514	0.9453	1.37E-06	0.006	23.32
IFN-G	rs112413063	C	T	-0.1813	0.0396	0.0557	4.62E-06	0.003	20.96
IFN-G	rs12929797	T	C	0.082	0.017	0.9771	1.32E-06	0.000	23.27
IFN-G	rs142660239	T	G	-0.3772	0.0779	0.0179	1.28E-06	0.005	23.45
IFN-G	rs144814361	T	C	0.4411	0.068	0.9871	9.07E-11	0.005	42.08
IFN-G	rs182621729	T	C	0.3083	0.0633	0.9662	1.11E-06	0.006	23.72
IFN-G	rs2208485	A	G	-0.0936	0.0182	0.3926	2.65E-07	0.004	26.45
IFN-G	rs2248244	A	G	0.1001	0.0211	0.2604	2.00E-06	0.004	22.51
IFN-G	rs2295547	C	A	-0.0894	0.0182	0.6630	8.77E-07	0.004	24.13
IFN-G	rs28370649	G	A	0.2836	0.0547	0.9831	2.20E-07	0.003	26.88
IFN-G	rs2949760	A	G	0.0915	0.0193	0.3519	2.00E-06	0.004	22.48
IFN-G	rs34311866	C	T	0.2272	0.0231	0.8131	7.97E-23	0.016	96.74
IFN-G	rs34679758	G	A	-0.1275	0.0259	0.8728	8.72E-07	0.004	24.23
IFN-G	rs356203	T	C	-0.2398	0.0178	0.6133	3.01E-41	0.027	181.49
IFN-G	rs35749011	A	G	0.7508	0.0659	0.0219	5.02E-30	0.024	129.80
IFN-G	rs41286192	G	A	0.3164	0.067	0.9642	2.30E-06	0.007	22.30
IFN-G	rs4488803	A	G	-0.1136	0.0199	0.6272	1.08E-08	0.006	32.59
IFN-G	rs4588066	A	G	0.1046	0.0178	0.3390	4.45E-09	0.005	34.53
IFN-G	rs4698412	A	G	0.1258	0.0168	0.5596	7.05E-14	0.008	56.07
IFN-G	rs4774417	A	G	0.1052	0.0192	0.7137	4.63E-08	0.005	30.02
IFN-G	rs4810687	T	G	0.0932	0.0187	0.4135	6.27E-07	0.004	24.84
IFN-G	rs4836108	A	G	0.1094	0.0225	0.5070	1.20E-06	0.006	23.64
IFN-G	rs4851487	T	C	0.0808	0.0174	0.5755	3.25E-06	0.003	21.56
IFN-G	rs61835654	C	T	0.1032	0.0216	0.2535	1.85E-06	0.004	22.83
IFN-G	rs620490	G	T	-0.1174	0.019	0.2992	6.46E-10	0.006	38.18

IFN-G	rs6715875	C	T	0.2902	0.0599	0.0249	1.25E-06	0.004	23.47	
IFN-G	rs6741007	G	T	-0.1233	0.0175	0.4970	2.09E-12	0.008	49.64	
IFN-G	rs6808178	C	T	-0.0864	0.0174	0.6292	7.20E-07	0.003	24.66	
IFN-G	rs73032517	G	A	0.2706	0.0558	0.9742	1.27E-06	0.004	23.52	
IFN-G	rs75505347	T	C	0.3917	0.0674	0.9791	6.12E-09	0.006	33.77	
IFN-G	rs75646569	G	T	0.1916	0.0266	0.1252	5.62E-13	0.008	51.88	
IFN-G	rs7695720	C	A	-0.1255	0.0208	0.7644	1.53E-09	0.006	36.40	
IFN-G	rs7818035	A	G	-0.693	0.1429	0.0209	1.24E-06	0.020	23.52	
IFN-G	rs79436216	G	A	0.2816	0.0603	0.9583	3.02E-06	0.006	21.81	
IFN-G	rs858295	G	A	-0.1039	0.0176	0.3708	3.83E-09	0.005	34.85	
IFN-G	rs9840232	T	C	0.1157	0.0233	0.8121	6.56E-07	0.004	24.66	
IFN-G	rs9845968	G	A	0.0842	0.0175	0.4483	1.42E-06	0.004	23.15	
IL-10	rs10513789	G	T	-0.1596	0.0219	0.1978	3.18E-13	0.008	53.11	
IL-10	rs10756905	T	C	0.1011	0.0196	0.7654	2.46E-07	0.004	26.61	
IL-10	rs10766301	T	C	0.0907	0.0192	0.6153	2.21E-06	0.004	22.32	
IL-10	rs10847864	T	G	0.1274	0.0179	0.3390	9.81E-13	0.007	50.66	
IL-10	rs111972941	G	A	0.2482	0.0514	0.9453	1.37E-06	0.006	23.32	
IL-10	rs112413063	C	T	-0.1813	0.0396	0.0557	4.62E-06	0.003	20.96	
IL-10	rs12929797	T	C	0.082	0.017	0.9771	1.32E-06	0.000	23.27	100.00%
IL-10	rs142660239	T	G	-0.3772	0.0779	0.0179	1.28E-06	0.005	23.45	
IL-10	rs144814361	T	C	0.4411	0.068	0.9871	9.07E-11	0.005	42.08	
IL-10	rs182621729	T	C	0.3083	0.0633	0.9662	1.11E-06	0.006	23.72	
IL-10	rs2208485	A	G	-0.0936	0.0182	0.3926	2.65E-07	0.004	26.45	
IL-10	rs2248244	A	G	0.1001	0.0211	0.2604	2.00E-06	0.004	22.51	
IL-10	rs2295547	C	A	-0.0894	0.0182	0.6630	8.77E-07	0.004	24.13	
IL-10	rs28370649	G	A	0.2836	0.0547	0.9831	2.20E-07	0.003	26.88	

IL-10	rs2949760	A	G	0.0915	0.0193	0.3519	2.00E-06	0.004	22.48
IL-10	rs34311866	C	T	0.2272	0.0231	0.8131	7.97E-23	0.016	96.74
IL-10	rs34679758	G	A	-0.1275	0.0259	0.8728	8.72E-07	0.004	24.23
IL-10	rs356203	T	C	-0.2398	0.0178	0.6133	3.01E-41	0.027	181.49
IL-10	rs35749011	A	G	0.7508	0.0659	0.0219	5.02E-30	0.024	129.80
IL-10	rs41286192	G	A	0.3164	0.067	0.9642	2.30E-06	0.007	22.30
IL-10	rs4488803	A	G	-0.1136	0.0199	0.6272	1.08E-08	0.006	32.59
IL-10	rs4588066	A	G	0.1046	0.0178	0.3390	4.45E-09	0.005	34.53
IL-10	rs4698412	A	G	0.1258	0.0168	0.5596	7.05E-14	0.008	56.07
IL-10	rs4774417	A	G	0.1052	0.0192	0.7137	4.63E-08	0.005	30.02
IL-10	rs4810687	T	G	0.0932	0.0187	0.4135	6.27E-07	0.004	24.84
IL-10	rs4836108	A	G	0.1094	0.0225	0.5070	1.20E-06	0.006	23.64
IL-10	rs4851487	T	C	0.0808	0.0174	0.5755	3.25E-06	0.003	21.56
IL-10	rs61835654	C	T	0.1032	0.0216	0.2535	1.85E-06	0.004	22.83
IL-10	rs620490	G	T	-0.1174	0.019	0.2992	6.46E-10	0.006	38.18
IL-10	rs6715875	C	T	0.2902	0.0599	0.0249	1.25E-06	0.004	23.47
IL-10	rs6741007	G	T	-0.1233	0.0175	0.4970	2.09E-12	0.008	49.64
IL-10	rs6808178	C	T	-0.0864	0.0174	0.6292	7.20E-07	0.003	24.66
IL-10	rs73032517	G	A	0.2706	0.0558	0.9742	1.27E-06	0.004	23.52
IL-10	rs75505347	T	C	0.3917	0.0674	0.9791	6.12E-09	0.006	33.77
IL-10	rs75646569	G	T	0.1916	0.0266	0.1252	5.62E-13	0.008	51.88
IL-10	rs7695720	C	A	-0.1255	0.0208	0.7644	1.53E-09	0.006	36.40
IL-10	rs7818035	A	G	-0.693	0.1429	0.0209	1.24E-06	0.020	23.52
IL-10	rs79436216	G	A	0.2816	0.0603	0.9583	3.02E-06	0.006	21.81
IL-10	rs858295	G	A	-0.1039	0.0176	0.3708	3.83E-09	0.005	34.85
IL-10	rs9840232	T	C	0.1157	0.0233	0.8121	6.56E-07	0.004	24.66

IL-10	rs9845968	G	A	0.0842	0.0175	0.4483	1.42E-06	0.004	23.15	
IL-12	rs10513789	G	T	-0.1596	0.0219	0.1978	3.18E-13	0.008	53.11	
IL-12	rs10756905	T	C	0.1011	0.0196	0.7654	2.46E-07	0.004	26.61	
IL-12	rs10766301	T	C	0.0907	0.0192	0.6153	2.21E-06	0.004	22.32	
IL-12	rs10847864	T	G	0.1274	0.0179	0.3390	9.81E-13	0.007	50.66	
IL-12	rs111972941	G	A	0.2482	0.0514	0.9453	1.37E-06	0.006	23.32	
IL-12	rs112413063	C	T	-0.1813	0.0396	0.0557	4.62E-06	0.003	20.96	
IL-12	rs12929797	T	C	0.082	0.017	0.9771	1.32E-06	0.000	23.27	
IL-12	rs142660239	T	G	-0.3772	0.0779	0.0179	1.28E-06	0.005	23.45	
IL-12	rs144814361	T	C	0.4411	0.068	0.9871	9.07E-11	0.005	42.08	
IL-12	rs182621729	T	C	0.3083	0.0633	0.9662	1.11E-06	0.006	23.72	
IL-12	rs2208485	A	G	-0.0936	0.0182	0.3926	2.65E-07	0.004	26.45	
IL-12	rs2248244	A	G	0.1001	0.0211	0.2604	2.00E-06	0.004	22.51	
IL-12	rs2295547	C	A	-0.0894	0.0182	0.6630	8.77E-07	0.004	24.13	97.70%
IL-12	rs28370649	G	A	0.2836	0.0547	0.9831	2.20E-07	0.003	26.88	
IL-12	rs2949760	A	G	0.0915	0.0193	0.3519	2.00E-06	0.004	22.48	
IL-12	rs34311866	C	T	0.2272	0.0231	0.8131	7.97E-23	0.016	96.74	
IL-12	rs34679758	G	A	-0.1275	0.0259	0.8728	8.72E-07	0.004	24.23	
IL-12	rs356203	T	C	-0.2398	0.0178	0.6133	3.01E-41	0.027	181.49	
IL-12	rs35749011	A	G	0.7508	0.0659	0.0219	5.02E-30	0.024	129.80	
IL-12	rs41286192	G	A	0.3164	0.067	0.9642	2.30E-06	0.007	22.30	
IL-12	rs4488803	A	G	-0.1136	0.0199	0.6272	1.08E-08	0.006	32.59	
IL-12	rs4588066	A	G	0.1046	0.0178	0.3390	4.45E-09	0.005	34.53	
IL-12	rs4698412	A	G	0.1258	0.0168	0.5596	7.05E-14	0.008	56.07	
IL-12	rs4774417	A	G	0.1052	0.0192	0.7137	4.63E-08	0.005	30.02	
IL-12	rs4810687	T	G	0.0932	0.0187	0.4135	6.27E-07	0.004	24.84	

IL-12	rs4836108	A	G	0.1094	0.0225	0.5070	1.20E-06	0.006	23.64	
IL-12	rs4851487	T	C	0.0808	0.0174	0.5755	3.25E-06	0.003	21.56	
IL-12	rs61835654	C	T	0.1032	0.0216	0.2535	1.85E-06	0.004	22.83	
IL-12	rs620490	G	T	-0.1174	0.019	0.2992	6.46E-10	0.006	38.18	
IL-12	rs6715875	C	T	0.2902	0.0599	0.0249	1.25E-06	0.004	23.47	
IL-12	rs6741007	G	T	-0.1233	0.0175	0.4970	2.09E-12	0.008	49.64	
IL-12	rs6808178	C	T	-0.0864	0.0174	0.6292	7.20E-07	0.003	24.66	
IL-12	rs73032517	G	A	0.2706	0.0558	0.9742	1.27E-06	0.004	23.52	
IL-12	rs75505347	T	C	0.3917	0.0674	0.9791	6.12E-09	0.006	33.77	
IL-12	rs75646569	G	T	0.1916	0.0266	0.1252	5.62E-13	0.008	51.88	
IL-12	rs7695720	C	A	-0.1255	0.0208	0.7644	1.53E-09	0.006	36.40	
IL-12	rs7818035	A	G	-0.693	0.1429	0.0209	1.24E-06	0.020	23.52	
IL-12	rs79436216	G	A	0.2816	0.0603	0.9583	3.02E-06	0.006	21.81	
IL-12	rs858295	G	A	-0.1039	0.0176	0.3708	3.83E-09	0.005	34.85	
IL-12	rs9840232	T	C	0.1157	0.0233	0.8121	6.56E-07	0.004	24.66	
IL-12	rs9845968	G	A	0.0842	0.0175	0.4483	1.42E-06	0.004	23.15	
IL-13	rs10513789	G	T	-0.1596	0.0219	0.1978	3.18E-13	0.008	53.11	
IL-13	rs10756905	T	C	0.1011	0.0196	0.7654	2.46E-07	0.004	26.61	
IL-13	rs10766301	T	C	0.0907	0.0192	0.6153	2.21E-06	0.004	22.32	
IL-13	rs10847864	T	G	0.1274	0.0179	0.3390	9.81E-13	0.007	50.66	
IL-13	rs111972941	G	A	0.2482	0.0514	0.9453	1.37E-06	0.006	23.32	18.40%
IL-13	rs112413063	C	T	-0.1813	0.0396	0.0557	4.62E-06	0.003	20.96	
IL-13	rs12929797	T	C	0.082	0.017	0.9771	1.32E-06	0.000	23.27	
IL-13	rs142660239	T	G	-0.3772	0.0779	0.0179	1.28E-06	0.005	23.45	
IL-13	rs144814361	T	C	0.4411	0.068	0.9871	9.07E-11	0.005	42.08	
IL-13	rs2208485	A	G	-0.0936	0.0182	0.3926	2.65E-07	0.004	26.45	

IL-13	rs2248244	A	G	0.1001	0.0211	0.2604	2.00E-06	0.004	22.51
IL-13	rs2295547	C	A	-0.0894	0.0182	0.6630	8.77E-07	0.004	24.13
IL-13	rs28370649	G	A	0.2836	0.0547	0.9831	2.20E-07	0.003	26.88
IL-13	rs2949760	A	G	0.0915	0.0193	0.3519	2.00E-06	0.004	22.48
IL-13	rs34311866	C	T	0.2272	0.0231	0.8131	7.97E-23	0.016	96.74
IL-13	rs34679758	G	A	-0.1275	0.0259	0.8728	8.72E-07	0.004	24.23
IL-13	rs356203	T	C	-0.2398	0.0178	0.6133	3.01E-41	0.027	181.49
IL-13	rs35749011	A	G	0.7508	0.0659	0.0219	5.02E-30	0.024	129.80
IL-13	rs41286192	G	A	0.3164	0.067	0.9642	2.30E-06	0.007	22.30
IL-13	rs4488803	A	G	-0.1136	0.0199	0.6272	1.08E-08	0.006	32.59
IL-13	rs4588066	A	G	0.1046	0.0178	0.3390	4.45E-09	0.005	34.53
IL-13	rs4698412	A	G	0.1258	0.0168	0.5596	7.05E-14	0.008	56.07
IL-13	rs4774417	A	G	0.1052	0.0192	0.7137	4.63E-08	0.005	30.02
IL-13	rs4810687	T	G	0.0932	0.0187	0.4135	6.27E-07	0.004	24.84
IL-13	rs4836108	A	G	0.1094	0.0225	0.5070	1.20E-06	0.006	23.64
IL-13	rs4851487	T	C	0.0808	0.0174	0.5755	3.25E-06	0.003	21.56
IL-13	rs61835654	C	T	0.1032	0.0216	0.2535	1.85E-06	0.004	22.83
IL-13	rs620490	G	T	-0.1174	0.019	0.2992	6.46E-10	0.006	38.18
IL-13	rs6741007	G	T	-0.1233	0.0175	0.4970	2.09E-12	0.008	49.64
IL-13	rs6808178	C	T	-0.0864	0.0174	0.6292	7.20E-07	0.003	24.66
IL-13	rs73032517	G	A	0.2706	0.0558	0.9742	1.27E-06	0.004	23.52
IL-13	rs75505347	T	C	0.3917	0.0674	0.9791	6.12E-09	0.006	33.77
IL-13	rs75646569	G	T	0.1916	0.0266	0.1252	5.62E-13	0.008	51.88
IL-13	rs7695720	C	A	-0.1255	0.0208	0.7644	1.53E-09	0.006	36.40
IL-13	rs7818035	A	G	-0.693	0.1429	0.0209	1.24E-06	0.020	23.52
IL-13	rs79436216	G	A	0.2816	0.0603	0.9583	3.02E-06	0.006	21.81

IL-13	rs858295	G	A	-0.1039	0.0176	0.3708	3.83E-09	0.005	34.85	
IL-13	rs9840232	T	C	0.1157	0.0233	0.8121	6.56E-07	0.004	24.66	
IL-13	rs9845968	G	A	0.0842	0.0175	0.4483	1.42E-06	0.004	23.15	
IL-16	rs10513789	G	T	-0.1596	0.0219	0.1978	3.18E-13	0.008	53.11	
IL-16	rs10756905	T	C	0.1011	0.0196	0.7654	2.46E-07	0.004	26.61	
IL-16	rs10766301	T	C	0.0907	0.0192	0.6153	2.21E-06	0.004	22.32	
IL-16	rs10847864	T	G	0.1274	0.0179	0.3390	9.81E-13	0.007	50.66	
IL-16	rs111972941	G	A	0.2482	0.0514	0.9453	1.37E-06	0.006	23.32	
IL-16	rs112413063	C	T	-0.1813	0.0396	0.0557	4.62E-06	0.003	20.96	
IL-16	rs12929797	T	C	0.082	0.017	0.9771	1.32E-06	0.000	23.27	
IL-16	rs142660239	T	G	-0.3772	0.0779	0.0179	1.28E-06	0.005	23.45	
IL-16	rs144814361	T	C	0.4411	0.068	0.9871	9.07E-11	0.005	42.08	
IL-16	rs182621729	T	C	0.3083	0.0633	0.9662	1.11E-06	0.006	23.72	
IL-16	rs2208485	A	G	-0.0936	0.0182	0.3926	2.65E-07	0.004	26.45	
IL-16	rs2248244	A	G	0.1001	0.0211	0.2604	2.00E-06	0.004	22.51	10.70%
IL-16	rs2295547	C	A	-0.0894	0.0182	0.6630	8.77E-07	0.004	24.13	
IL-16	rs28370649	G	A	0.2836	0.0547	0.9831	2.20E-07	0.003	26.88	
IL-16	rs2949760	A	G	0.0915	0.0193	0.3519	2.00E-06	0.004	22.48	
IL-16	rs34311866	C	T	0.2272	0.0231	0.8131	7.97E-23	0.016	96.74	
IL-16	rs34679758	G	A	-0.1275	0.0259	0.8728	8.72E-07	0.004	24.23	
IL-16	rs356203	T	C	-0.2398	0.0178	0.6133	3.01E-41	0.027	181.49	
IL-16	rs35749011	A	G	0.7508	0.0659	0.0219	5.02E-30	0.024	129.80	
IL-16	rs41286192	G	A	0.3164	0.067	0.9642	2.30E-06	0.007	22.30	
IL-16	rs4488803	A	G	-0.1136	0.0199	0.6272	1.08E-08	0.006	32.59	
IL-16	rs4588066	A	G	0.1046	0.0178	0.3390	4.45E-09	0.005	34.53	
IL-16	rs4698412	A	G	0.1258	0.0168	0.5596	7.05E-14	0.008	56.07	

IL-16	rs4774417	A	G	0.1052	0.0192	0.7137	4.63E-08	0.005	30.02	
IL-16	rs4810687	T	G	0.0932	0.0187	0.4135	6.27E-07	0.004	24.84	
IL-16	rs4836108	A	G	0.1094	0.0225	0.5070	1.20E-06	0.006	23.64	
IL-16	rs4851487	T	C	0.0808	0.0174	0.5755	3.25E-06	0.003	21.56	
IL-16	rs61835654	C	T	0.1032	0.0216	0.2535	1.85E-06	0.004	22.83	
IL-16	rs620490	G	T	-0.1174	0.019	0.2992	6.46E-10	0.006	38.18	
IL-16	rs6741007	G	T	-0.1233	0.0175	0.4970	2.09E-12	0.008	49.64	
IL-16	rs6808178	C	T	-0.0864	0.0174	0.6292	7.20E-07	0.003	24.66	
IL-16	rs73032517	G	A	0.2706	0.0558	0.9742	1.27E-06	0.004	23.52	
IL-16	rs75505347	T	C	0.3917	0.0674	0.9791	6.12E-09	0.006	33.77	
IL-16	rs75646569	G	T	0.1916	0.0266	0.1252	5.62E-13	0.008	51.88	
IL-16	rs7695720	C	A	-0.1255	0.0208	0.7644	1.53E-09	0.006	36.40	
IL-16	rs7818035	A	G	-0.693	0.1429	0.0209	1.24E-06	0.020	23.52	
IL-16	rs79436216	G	A	0.2816	0.0603	0.9583	3.02E-06	0.006	21.81	
IL-16	rs858295	G	A	-0.1039	0.0176	0.3708	3.83E-09	0.005	34.85	
IL-16	rs9840232	T	C	0.1157	0.0233	0.8121	6.56E-07	0.004	24.66	
IL-16	rs9845968	G	A	0.0842	0.0175	0.4483	1.42E-06	0.004	23.15	
IL-17	rs10513789	G	T	-0.1596	0.0219	0.1978	3.18E-13	0.008	53.11	
IL-17	rs10756905	T	C	0.1011	0.0196	0.7654	2.46E-07	0.004	26.61	
IL-17	rs10766301	T	C	0.0907	0.0192	0.6153	2.21E-06	0.004	22.32	
IL-17	rs10847864	T	G	0.1274	0.0179	0.3390	9.81E-13	0.007	50.66	
IL-17	rs111972941	G	A	0.2482	0.0514	0.9453	1.37E-06	0.006	23.32	81.60%
IL-17	rs112413063	C	T	-0.1813	0.0396	0.0557	4.62E-06	0.003	20.96	
IL-17	rs12929797	T	C	0.082	0.017	0.9771	1.32E-06	0.000	23.27	
IL-17	rs142660239	T	G	-0.3772	0.0779	0.0179	1.28E-06	0.005	23.45	
IL-17	rs144814361	T	C	0.4411	0.068	0.9871	9.07E-11	0.005	42.08	

IL-17	rs182621729	T	C	0.3083	0.0633	0.9662	1.11E-06	0.006	23.72
IL-17	rs2208485	A	G	-0.0936	0.0182	0.3926	2.65E-07	0.004	26.45
IL-17	rs2248244	A	G	0.1001	0.0211	0.2604	2.00E-06	0.004	22.51
IL-17	rs2295547	C	A	-0.0894	0.0182	0.6630	8.77E-07	0.004	24.13
IL-17	rs28370649	G	A	0.2836	0.0547	0.9831	2.20E-07	0.003	26.88
IL-17	rs2949760	A	G	0.0915	0.0193	0.3519	2.00E-06	0.004	22.48
IL-17	rs34311866	C	T	0.2272	0.0231	0.8131	7.97E-23	0.016	96.74
IL-17	rs34679758	G	A	-0.1275	0.0259	0.8728	8.72E-07	0.004	24.23
IL-17	rs356203	T	C	-0.2398	0.0178	0.6133	3.01E-41	0.027	181.49
IL-17	rs35749011	A	G	0.7508	0.0659	0.0219	5.02E-30	0.024	129.80
IL-17	rs41286192	G	A	0.3164	0.067	0.9642	2.30E-06	0.007	22.30
IL-17	rs4488803	A	G	-0.1136	0.0199	0.6272	1.08E-08	0.006	32.59
IL-17	rs4588066	A	G	0.1046	0.0178	0.3390	4.45E-09	0.005	34.53
IL-17	rs4698412	A	G	0.1258	0.0168	0.5596	7.05E-14	0.008	56.07
IL-17	rs4774417	A	G	0.1052	0.0192	0.7137	4.63E-08	0.005	30.02
IL-17	rs4810687	T	G	0.0932	0.0187	0.4135	6.27E-07	0.004	24.84
IL-17	rs4836108	A	G	0.1094	0.0225	0.5070	1.20E-06	0.006	23.64
IL-17	rs4851487	T	C	0.0808	0.0174	0.5755	3.25E-06	0.003	21.56
IL-17	rs61835654	C	T	0.1032	0.0216	0.2535	1.85E-06	0.004	22.83
IL-17	rs620490	G	T	-0.1174	0.019	0.2992	6.46E-10	0.006	38.18
IL-17	rs6741007	G	T	-0.1233	0.0175	0.4970	2.09E-12	0.008	49.64
IL-17	rs6808178	C	T	-0.0864	0.0174	0.6292	7.20E-07	0.003	24.66
IL-17	rs73032517	G	A	0.2706	0.0558	0.9742	1.27E-06	0.004	23.52
IL-17	rs75505347	T	C	0.3917	0.0674	0.9791	6.12E-09	0.006	33.77
IL-17	rs75646569	G	T	0.1916	0.0266	0.1252	5.62E-13	0.008	51.88
IL-17	rs7695720	C	A	-0.1255	0.0208	0.7644	1.53E-09	0.006	36.40

IL-17	rs7818035	A	G	-0.693	0.1429	0.0209	1.24E-06	0.020	23.52		
IL-17	rs79436216	G	A	0.2816	0.0603	0.9583	3.02E-06	0.006	21.81		
IL-17	rs858295	G	A	-0.1039	0.0176	0.3708	3.83E-09	0.005	34.85		
IL-17	rs9840232	T	C	0.1157	0.0233	0.8121	6.56E-07	0.004	24.66		
IL-17	rs9845968	G	A	0.0842	0.0175	0.4483	1.42E-06	0.004	23.15		
IL-18	rs10513789	G	T	-0.1596	0.0219	0.1978	3.18E-13	0.008	53.11		
IL-18	rs10756905	T	C	0.1011	0.0196	0.7654	2.46E-07	0.004	26.61		
IL-18	rs10766301	T	C	0.0907	0.0192	0.6153	2.21E-06	0.004	22.32		
IL-18	rs10847864	T	G	0.1274	0.0179	0.3390	9.81E-13	0.007	50.66		
IL-18	rs111972941	G	A	0.2482	0.0514	0.9453	1.37E-06	0.006	23.32		
IL-18	rs112413063	C	T	-0.1813	0.0396	0.0557	4.62E-06	0.003	20.96		
IL-18	rs12929797	T	C	0.082	0.017	0.9771	1.32E-06	0.000	23.27		
IL-18	rs142660239	T	G	-0.3772	0.0779	0.0179	1.28E-06	0.005	23.45		
IL-18	rs144814361	T	C	0.4411	0.068	0.9871	9.07E-11	0.005	42.08		
IL-18	rs182621729	T	C	0.3083	0.0633	0.9662	1.11E-06	0.006	23.72		
IL-18	rs2208485	A	G	-0.0936	0.0182	0.3926	2.65E-07	0.004	26.45	94.70%	
IL-18	rs2248244	A	G	0.1001	0.0211	0.2604	2.00E-06	0.004	22.51		
IL-18	rs2295547	C	A	-0.0894	0.0182	0.6630	8.77E-07	0.004	24.13		
IL-18	rs28370649	G	A	0.2836	0.0547	0.9831	2.20E-07	0.003	26.88		
IL-18	rs2949760	A	G	0.0915	0.0193	0.3519	2.00E-06	0.004	22.48		
IL-18	rs34311866	C	T	0.2272	0.0231	0.8131	7.97E-23	0.016	96.74		
IL-18	rs34679758	G	A	-0.1275	0.0259	0.8728	8.72E-07	0.004	24.23		
IL-18	rs356203	T	C	-0.2398	0.0178	0.6133	3.01E-41	0.027	181.49		
IL-18	rs35749011	A	G	0.7508	0.0659	0.0219	5.02E-30	0.024	129.80		
IL-18	rs41286192	G	A	0.3164	0.067	0.9642	2.30E-06	0.007	22.30		
IL-18	rs4488803	A	G	-0.1136	0.0199	0.6272	1.08E-08	0.006	32.59		

IL-18	rs4588066	A	G	0.1046	0.0178	0.3390	4.45E-09	0.005	34.53	
IL-18	rs4698412	A	G	0.1258	0.0168	0.5596	7.05E-14	0.008	56.07	
IL-18	rs4774417	A	G	0.1052	0.0192	0.7137	4.63E-08	0.005	30.02	
IL-18	rs4810687	T	G	0.0932	0.0187	0.4135	6.27E-07	0.004	24.84	
IL-18	rs4836108	A	G	0.1094	0.0225	0.5070	1.20E-06	0.006	23.64	
IL-18	rs4851487	T	C	0.0808	0.0174	0.5755	3.25E-06	0.003	21.56	
IL-18	rs61835654	C	T	0.1032	0.0216	0.2535	1.85E-06	0.004	22.83	
IL-18	rs620490	G	T	-0.1174	0.019	0.2992	6.46E-10	0.006	38.18	
IL-18	rs6741007	G	T	-0.1233	0.0175	0.4970	2.09E-12	0.008	49.64	
IL-18	rs6808178	C	T	-0.0864	0.0174	0.6292	7.20E-07	0.003	24.66	
IL-18	rs73032517	G	A	0.2706	0.0558	0.9742	1.27E-06	0.004	23.52	
IL-18	rs75505347	T	C	0.3917	0.0674	0.9791	6.12E-09	0.006	33.77	
IL-18	rs75646569	G	T	0.1916	0.0266	0.1252	5.62E-13	0.008	51.88	
IL-18	rs7695720	C	A	-0.1255	0.0208	0.7644	1.53E-09	0.006	36.40	
IL-18	rs7818035	A	G	-0.693	0.1429	0.0209	1.24E-06	0.020	23.52	
IL-18	rs79436216	G	A	0.2816	0.0603	0.9583	3.02E-06	0.006	21.81	
IL-18	rs858295	G	A	-0.1039	0.0176	0.3708	3.83E-09	0.005	34.85	
IL-18	rs9840232	T	C	0.1157	0.0233	0.8121	6.56E-07	0.004	24.66	
IL-18	rs9845968	G	A	0.0842	0.0175	0.4483	1.42E-06	0.004	23.15	
IL-1B	rs10513789	G	T	-0.1596	0.0219	0.1978	3.18E-13	0.008	53.11	
IL-1B	rs10756905	T	C	0.1011	0.0196	0.7654	2.46E-07	0.004	26.61	
IL-1B	rs10766301	T	C	0.0907	0.0192	0.6153	2.21E-06	0.004	22.32	
IL-1B	rs10847864	T	G	0.1274	0.0179	0.3390	9.81E-13	0.007	50.66	100.00%
IL-1B	rs111972941	G	A	0.2482	0.0514	0.9453	1.37E-06	0.006	23.32	
IL-1B	rs112413063	C	T	-0.1813	0.0396	0.0557	4.62E-06	0.003	20.96	
IL-1B	rs12929797	T	C	0.082	0.017	0.9771	1.32E-06	0.000	23.27	

IL-1B	rs142660239	T	G	-0.3772	0.0779	0.0179	1.28E-06	0.005	23.45
IL-1B	rs144814361	T	C	0.4411	0.068	0.9871	9.07E-11	0.005	42.08
IL-1B	rs182621729	T	C	0.3083	0.0633	0.9662	1.11E-06	0.006	23.72
IL-1B	rs2208485	A	G	-0.0936	0.0182	0.3926	2.65E-07	0.004	26.45
IL-1B	rs2248244	A	G	0.1001	0.0211	0.2604	2.00E-06	0.004	22.51
IL-1B	rs2295547	C	A	-0.0894	0.0182	0.6630	8.77E-07	0.004	24.13
IL-1B	rs28370649	G	A	0.2836	0.0547	0.9831	2.20E-07	0.003	26.88
IL-1B	rs2949760	A	G	0.0915	0.0193	0.3519	2.00E-06	0.004	22.48
IL-1B	rs34311866	C	T	0.2272	0.0231	0.8131	7.97E-23	0.016	96.74
IL-1B	rs34679758	G	A	-0.1275	0.0259	0.8728	8.72E-07	0.004	24.23
IL-1B	rs356203	T	C	-0.2398	0.0178	0.6133	3.01E-41	0.027	181.49
IL-1B	rs35749011	A	G	0.7508	0.0659	0.0219	5.02E-30	0.024	129.80
IL-1B	rs41286192	G	A	0.3164	0.067	0.9642	2.30E-06	0.007	22.30
IL-1B	rs4488803	A	G	-0.1136	0.0199	0.6272	1.08E-08	0.006	32.59
IL-1B	rs4588066	A	G	0.1046	0.0178	0.3390	4.45E-09	0.005	34.53
IL-1B	rs4698412	A	G	0.1258	0.0168	0.5596	7.05E-14	0.008	56.07
IL-1B	rs4774417	A	G	0.1052	0.0192	0.7137	4.63E-08	0.005	30.02
IL-1B	rs4810687	T	G	0.0932	0.0187	0.4135	6.27E-07	0.004	24.84
IL-1B	rs4836108	A	G	0.1094	0.0225	0.5070	1.20E-06	0.006	23.64
IL-1B	rs4851487	T	C	0.0808	0.0174	0.5755	3.25E-06	0.003	21.56
IL-1B	rs61835654	C	T	0.1032	0.0216	0.2535	1.85E-06	0.004	22.83
IL-1B	rs620490	G	T	-0.1174	0.019	0.2992	6.46E-10	0.006	38.18
IL-1B	rs6741007	G	T	-0.1233	0.0175	0.4970	2.09E-12	0.008	49.64
IL-1B	rs6808178	C	T	-0.0864	0.0174	0.6292	7.20E-07	0.003	24.66
IL-1B	rs73032517	G	A	0.2706	0.0558	0.9742	1.27E-06	0.004	23.52
IL-1B	rs75505347	T	C	0.3917	0.0674	0.9791	6.12E-09	0.006	33.77

IL-1B	rs75646569	G	T	0.1916	0.0266	0.1252	5.62E-13	0.008	51.88	
IL-1B	rs7695720	C	A	-0.1255	0.0208	0.7644	1.53E-09	0.006	36.40	
IL-1B	rs7818035	A	G	-0.693	0.1429	0.0209	1.24E-06	0.020	23.52	
IL-1B	rs79436216	G	A	0.2816	0.0603	0.9583	3.02E-06	0.006	21.81	
IL-1B	rs858295	G	A	-0.1039	0.0176	0.3708	3.83E-09	0.005	34.85	
IL-1B	rs9840232	T	C	0.1157	0.0233	0.8121	6.56E-07	0.004	24.66	
IL-1B	rs9845968	G	A	0.0842	0.0175	0.4483	1.42E-06	0.004	23.15	
IL-1RA	rs10513789	G	T	-0.1596	0.0219	0.1978	3.18E-13	0.008	53.11	
IL-1RA	rs10756905	T	C	0.1011	0.0196	0.7654	2.46E-07	0.004	26.61	
IL-1RA	rs10766301	T	C	0.0907	0.0192	0.6153	2.21E-06	0.004	22.32	
IL-1RA	rs10847864	T	G	0.1274	0.0179	0.3390	9.81E-13	0.007	50.66	
IL-1RA	rs111972941	G	A	0.2482	0.0514	0.9453	1.37E-06	0.006	23.32	
IL-1RA	rs112413063	C	T	-0.1813	0.0396	0.0557	4.62E-06	0.003	20.96	
IL-1RA	rs12929797	T	C	0.082	0.017	0.9771	1.32E-06	0.000	23.27	
IL-1RA	rs142660239	T	G	-0.3772	0.0779	0.0179	1.28E-06	0.005	23.45	
IL-1RA	rs144814361	T	C	0.4411	0.068	0.9871	9.07E-11	0.005	42.08	
IL-1RA	rs2208485	A	G	-0.0936	0.0182	0.3926	2.65E-07	0.004	26.45	80.80%
IL-1RA	rs2248244	A	G	0.1001	0.0211	0.2604	2.00E-06	0.004	22.51	
IL-1RA	rs2295547	C	A	-0.0894	0.0182	0.6630	8.77E-07	0.004	24.13	
IL-1RA	rs28370649	G	A	0.2836	0.0547	0.9831	2.20E-07	0.003	26.88	
IL-1RA	rs2949760	A	G	0.0915	0.0193	0.3519	2.00E-06	0.004	22.48	
IL-1RA	rs34311866	C	T	0.2272	0.0231	0.8131	7.97E-23	0.016	96.74	
IL-1RA	rs34679758	G	A	-0.1275	0.0259	0.8728	8.72E-07	0.004	24.23	
IL-1RA	rs356203	T	C	-0.2398	0.0178	0.6133	3.01E-41	0.027	181.49	
IL-1RA	rs35749011	A	G	0.7508	0.0659	0.0219	5.02E-30	0.024	129.80	
IL-1RA	rs41286192	G	A	0.3164	0.067	0.9642	2.30E-06	0.007	22.30	

IL-1RA	rs4488803	A	G	-0.1136	0.0199	0.6272	1.08E-08	0.006	32.59	
IL-1RA	rs4588066	A	G	0.1046	0.0178	0.3390	4.45E-09	0.005	34.53	
IL-1RA	rs4698412	A	G	0.1258	0.0168	0.5596	7.05E-14	0.008	56.07	
IL-1RA	rs4774417	A	G	0.1052	0.0192	0.7137	4.63E-08	0.005	30.02	
IL-1RA	rs4810687	T	G	0.0932	0.0187	0.4135	6.27E-07	0.004	24.84	
IL-1RA	rs4836108	A	G	0.1094	0.0225	0.5070	1.20E-06	0.006	23.64	
IL-1RA	rs4851487	T	C	0.0808	0.0174	0.5755	3.25E-06	0.003	21.56	
IL-1RA	rs61835654	C	T	0.1032	0.0216	0.2535	1.85E-06	0.004	22.83	
IL-1RA	rs620490	G	T	-0.1174	0.019	0.2992	6.46E-10	0.006	38.18	
IL-1RA	rs6741007	G	T	-0.1233	0.0175	0.4970	2.09E-12	0.008	49.64	
IL-1RA	rs6808178	C	T	-0.0864	0.0174	0.6292	7.20E-07	0.003	24.66	
IL-1RA	rs73032517	G	A	0.2706	0.0558	0.9742	1.27E-06	0.004	23.52	
IL-1RA	rs75505347	T	C	0.3917	0.0674	0.9791	6.12E-09	0.006	33.77	
IL-1RA	rs75646569	G	T	0.1916	0.0266	0.1252	5.62E-13	0.008	51.88	
IL-1RA	rs7695720	C	A	-0.1255	0.0208	0.7644	1.53E-09	0.006	36.40	
IL-1RA	rs7818035	A	G	-0.693	0.1429	0.0209	1.24E-06	0.020	23.52	
IL-1RA	rs79436216	G	A	0.2816	0.0603	0.9583	3.02E-06	0.006	21.81	
IL-1RA	rs858295	G	A	-0.1039	0.0176	0.3708	3.83E-09	0.005	34.85	
IL-1RA	rs9840232	T	C	0.1157	0.0233	0.8121	6.56E-07	0.004	24.66	
IL-1RA	rs9845968	G	A	0.0842	0.0175	0.4483	1.42E-06	0.004	23.15	
IL-2	rs10513789	G	T	-0.1596	0.0219	0.1978	3.18E-13	0.008	53.11	
IL-2	rs10756905	T	C	0.1011	0.0196	0.7654	2.46E-07	0.004	26.61	
IL-2	rs10766301	T	C	0.0907	0.0192	0.6153	2.21E-06	0.004	22.32	100.00%
IL-2	rs10847864	T	G	0.1274	0.0179	0.3390	9.81E-13	0.007	50.66	
IL-2	rs111972941	G	A	0.2482	0.0514	0.9453	1.37E-06	0.006	23.32	
IL-2	rs112413063	C	T	-0.1813	0.0396	0.0557	4.62E-06	0.003	20.96	

IL-2	rs12929797	T	C	0.082	0.017	0.9771	1.32E-06	0.000	23.27
IL-2	rs142660239	T	G	-0.3772	0.0779	0.0179	1.28E-06	0.005	23.45
IL-2	rs144814361	T	C	0.4411	0.068	0.9871	9.07E-11	0.005	42.08
IL-2	rs182621729	T	C	0.3083	0.0633	0.9662	1.11E-06	0.006	23.72
IL-2	rs2208485	A	G	-0.0936	0.0182	0.3926	2.65E-07	0.004	26.45
IL-2	rs2248244	A	G	0.1001	0.0211	0.2604	2.00E-06	0.004	22.51
IL-2	rs2295547	C	A	-0.0894	0.0182	0.6630	8.77E-07	0.004	24.13
IL-2	rs28370649	G	A	0.2836	0.0547	0.9831	2.20E-07	0.003	26.88
IL-2	rs2949760	A	G	0.0915	0.0193	0.3519	2.00E-06	0.004	22.48
IL-2	rs34311866	C	T	0.2272	0.0231	0.8131	7.97E-23	0.016	96.74
IL-2	rs34679758	G	A	-0.1275	0.0259	0.8728	8.72E-07	0.004	24.23
IL-2	rs356203	T	C	-0.2398	0.0178	0.6133	3.01E-41	0.027	181.49
IL-2	rs35749011	A	G	0.7508	0.0659	0.0219	5.02E-30	0.024	129.80
IL-2	rs41286192	G	A	0.3164	0.067	0.9642	2.30E-06	0.007	22.30
IL-2	rs4488803	A	G	-0.1136	0.0199	0.6272	1.08E-08	0.006	32.59
IL-2	rs4588066	A	G	0.1046	0.0178	0.3390	4.45E-09	0.005	34.53
IL-2	rs4698412	A	G	0.1258	0.0168	0.5596	7.05E-14	0.008	56.07
IL-2	rs4774417	A	G	0.1052	0.0192	0.7137	4.63E-08	0.005	30.02
IL-2	rs4810687	T	G	0.0932	0.0187	0.4135	6.27E-07	0.004	24.84
IL-2	rs4836108	A	G	0.1094	0.0225	0.5070	1.20E-06	0.006	23.64
IL-2	rs4851487	T	C	0.0808	0.0174	0.5755	3.25E-06	0.003	21.56
IL-2	rs61835654	C	T	0.1032	0.0216	0.2535	1.85E-06	0.004	22.83
IL-2	rs620490	G	T	-0.1174	0.019	0.2992	6.46E-10	0.006	38.18
IL-2	rs6741007	G	T	-0.1233	0.0175	0.4970	2.09E-12	0.008	49.64
IL-2	rs6808178	C	T	-0.0864	0.0174	0.6292	7.20E-07	0.003	24.66
IL-2	rs73032517	G	A	0.2706	0.0558	0.9742	1.27E-06	0.004	23.52

IL-2	rs75505347	T	C	0.3917	0.0674	0.9791	6.12E-09	0.006	33.77	
IL-2	rs75646569	G	T	0.1916	0.0266	0.1252	5.62E-13	0.008	51.88	
IL-2	rs7695720	C	A	-0.1255	0.0208	0.7644	1.53E-09	0.006	36.40	
IL-2	rs7818035	A	G	-0.693	0.1429	0.0209	1.24E-06	0.020	23.52	
IL-2	rs79436216	G	A	0.2816	0.0603	0.9583	3.02E-06	0.006	21.81	
IL-2	rs858295	G	A	-0.1039	0.0176	0.3708	3.83E-09	0.005	34.85	
IL-2	rs9840232	T	C	0.1157	0.0233	0.8121	6.56E-07	0.004	24.66	
IL-2	rs9845968	G	A	0.0842	0.0175	0.4483	1.42E-06	0.004	23.15	
IL-2RA	rs10513789	G	T	-0.1596	0.0219	0.1978	3.18E-13	0.008	53.11	
IL-2RA	rs10756905	T	C	0.1011	0.0196	0.7654	2.46E-07	0.004	26.61	
IL-2RA	rs10766301	T	C	0.0907	0.0192	0.6153	2.21E-06	0.004	22.32	
IL-2RA	rs10847864	T	G	0.1274	0.0179	0.3390	9.81E-13	0.007	50.66	
IL-2RA	rs111972941	G	A	0.2482	0.0514	0.9453	1.37E-06	0.006	23.32	
IL-2RA	rs112413063	C	T	-0.1813	0.0396	0.0557	4.62E-06	0.003	20.96	
IL-2RA	rs12929797	T	C	0.082	0.017	0.9771	1.32E-06	0.000	23.27	
IL-2RA	rs142660239	T	G	-0.3772	0.0779	0.0179	1.28E-06	0.005	23.45	
IL-2RA	rs144814361	T	C	0.4411	0.068	0.9871	9.07E-11	0.005	42.08	
IL-2RA	rs182621729	T	C	0.3083	0.0633	0.9662	1.11E-06	0.006	23.72	100.00%
IL-2RA	rs2208485	A	G	-0.0936	0.0182	0.3926	2.65E-07	0.004	26.45	
IL-2RA	rs2248244	A	G	0.1001	0.0211	0.2604	2.00E-06	0.004	22.51	
IL-2RA	rs2295547	C	A	-0.0894	0.0182	0.6630	8.77E-07	0.004	24.13	
IL-2RA	rs28370649	G	A	0.2836	0.0547	0.9831	2.20E-07	0.003	26.88	
IL-2RA	rs2949760	A	G	0.0915	0.0193	0.3519	2.00E-06	0.004	22.48	
IL-2RA	rs34311866	C	T	0.2272	0.0231	0.8131	7.97E-23	0.016	96.74	
IL-2RA	rs34679758	G	A	-0.1275	0.0259	0.8728	8.72E-07	0.004	24.23	
IL-2RA	rs356203	T	C	-0.2398	0.0178	0.6133	3.01E-41	0.027	181.49	

IL-2RA	rs35749011	A	G	0.7508	0.0659	0.0219	5.02E-30	0.024	129.80	
IL-2RA	rs41286192	G	A	0.3164	0.067	0.9642	2.30E-06	0.007	22.30	
IL-2RA	rs4488803	A	G	-0.1136	0.0199	0.6272	1.08E-08	0.006	32.59	
IL-2RA	rs4588066	A	G	0.1046	0.0178	0.3390	4.45E-09	0.005	34.53	
IL-2RA	rs4698412	A	G	0.1258	0.0168	0.5596	7.05E-14	0.008	56.07	
IL-2RA	rs4774417	A	G	0.1052	0.0192	0.7137	4.63E-08	0.005	30.02	
IL-2RA	rs4810687	T	G	0.0932	0.0187	0.4135	6.27E-07	0.004	24.84	
IL-2RA	rs4836108	A	G	0.1094	0.0225	0.5070	1.20E-06	0.006	23.64	
IL-2RA	rs4851487	T	C	0.0808	0.0174	0.5755	3.25E-06	0.003	21.56	
IL-2RA	rs61835654	C	T	0.1032	0.0216	0.2535	1.85E-06	0.004	22.83	
IL-2RA	rs620490	G	T	-0.1174	0.019	0.2992	6.46E-10	0.006	38.18	
IL-2RA	rs6715875	C	T	0.2902	0.0599	0.0249	1.25E-06	0.004	23.47	
IL-2RA	rs6741007	G	T	-0.1233	0.0175	0.4970	2.09E-12	0.008	49.64	
IL-2RA	rs6808178	C	T	-0.0864	0.0174	0.6292	7.20E-07	0.003	24.66	
IL-2RA	rs73032517	G	A	0.2706	0.0558	0.9742	1.27E-06	0.004	23.52	
IL-2RA	rs75505347	T	C	0.3917	0.0674	0.9791	6.12E-09	0.006	33.77	
IL-2RA	rs75646569	G	T	0.1916	0.0266	0.1252	5.62E-13	0.008	51.88	
IL-2RA	rs7695720	C	A	-0.1255	0.0208	0.7644	1.53E-09	0.006	36.40	
IL-2RA	rs7818035	A	G	-0.693	0.1429	0.0209	1.24E-06	0.020	23.52	
IL-2RA	rs79436216	G	A	0.2816	0.0603	0.9583	3.02E-06	0.006	21.81	
IL-2RA	rs858295	G	A	-0.1039	0.0176	0.3708	3.83E-09	0.005	34.85	
IL-2RA	rs9840232	T	C	0.1157	0.0233	0.8121	6.56E-07	0.004	24.66	
IL-2RA	rs9845968	G	A	0.0842	0.0175	0.4483	1.42E-06	0.004	23.15	
IL-4	rs10513789	G	T	-0.1596	0.0219	0.1978	3.18E-13	0.008	53.11	
IL-4	rs10756905	T	C	0.1011	0.0196	0.7654	2.46E-07	0.004	26.61	81.60%
IL-4	rs10766301	T	C	0.0907	0.0192	0.6153	2.21E-06	0.004	22.32	

IL-4	rs10847864	T	G	0.1274	0.0179	0.3390	9.81E-13	0.007	50.66
IL-4	rs111972941	G	A	0.2482	0.0514	0.9453	1.37E-06	0.006	23.32
IL-4	rs112413063	C	T	-0.1813	0.0396	0.0557	4.62E-06	0.003	20.96
IL-4	rs12929797	T	C	0.082	0.017	0.9771	1.32E-06	0.000	23.27
IL-4	rs142660239	T	G	-0.3772	0.0779	0.0179	1.28E-06	0.005	23.45
IL-4	rs144814361	T	C	0.4411	0.068	0.9871	9.07E-11	0.005	42.08
IL-4	rs182621729	T	C	0.3083	0.0633	0.9662	1.11E-06	0.006	23.72
IL-4	rs2208485	A	G	-0.0936	0.0182	0.3926	2.65E-07	0.004	26.45
IL-4	rs2248244	A	G	0.1001	0.0211	0.2604	2.00E-06	0.004	22.51
IL-4	rs2295547	C	A	-0.0894	0.0182	0.6630	8.77E-07	0.004	24.13
IL-4	rs28370649	G	A	0.2836	0.0547	0.9831	2.20E-07	0.003	26.88
IL-4	rs2949760	A	G	0.0915	0.0193	0.3519	2.00E-06	0.004	22.48
IL-4	rs34311866	C	T	0.2272	0.0231	0.8131	7.97E-23	0.016	96.74
IL-4	rs34679758	G	A	-0.1275	0.0259	0.8728	8.72E-07	0.004	24.23
IL-4	rs356203	T	C	-0.2398	0.0178	0.6133	3.01E-41	0.027	181.49
IL-4	rs35749011	A	G	0.7508	0.0659	0.0219	5.02E-30	0.024	129.80
IL-4	rs41286192	G	A	0.3164	0.067	0.9642	2.30E-06	0.007	22.30
IL-4	rs4488803	A	G	-0.1136	0.0199	0.6272	1.08E-08	0.006	32.59
IL-4	rs4588066	A	G	0.1046	0.0178	0.3390	4.45E-09	0.005	34.53
IL-4	rs4698412	A	G	0.1258	0.0168	0.5596	7.05E-14	0.008	56.07
IL-4	rs4774417	A	G	0.1052	0.0192	0.7137	4.63E-08	0.005	30.02
IL-4	rs4810687	T	G	0.0932	0.0187	0.4135	6.27E-07	0.004	24.84
IL-4	rs4836108	A	G	0.1094	0.0225	0.5070	1.20E-06	0.006	23.64
IL-4	rs4851487	T	C	0.0808	0.0174	0.5755	3.25E-06	0.003	21.56
IL-4	rs61835654	C	T	0.1032	0.0216	0.2535	1.85E-06	0.004	22.83
IL-4	rs620490	G	T	-0.1174	0.019	0.2992	6.46E-10	0.006	38.18

IL-4	rs6741007	G	T	-0.1233	0.0175	0.4970	2.09E-12	0.008	49.64	
IL-4	rs6808178	C	T	-0.0864	0.0174	0.6292	7.20E-07	0.003	24.66	
IL-4	rs73032517	G	A	0.2706	0.0558	0.9742	1.27E-06	0.004	23.52	
IL-4	rs75505347	T	C	0.3917	0.0674	0.9791	6.12E-09	0.006	33.77	
IL-4	rs75646569	G	T	0.1916	0.0266	0.1252	5.62E-13	0.008	51.88	
IL-4	rs7695720	C	A	-0.1255	0.0208	0.7644	1.53E-09	0.006	36.40	
IL-4	rs7818035	A	G	-0.693	0.1429	0.0209	1.24E-06	0.020	23.52	
IL-4	rs79436216	G	A	0.2816	0.0603	0.9583	3.02E-06	0.006	21.81	
IL-4	rs858295	G	A	-0.1039	0.0176	0.3708	3.83E-09	0.005	34.85	
IL-4	rs9840232	T	C	0.1157	0.0233	0.8121	6.56E-07	0.004	24.66	
IL-4	rs9845968	G	A	0.0842	0.0175	0.4483	1.42E-06	0.004	23.15	
IL-5	rs10513789	G	T	-0.1596	0.0219	0.1978	3.18E-13	0.008	53.11	
IL-5	rs10756905	T	C	0.1011	0.0196	0.7654	2.46E-07	0.004	26.61	
IL-5	rs10766301	T	C	0.0907	0.0192	0.6153	2.21E-06	0.004	22.32	
IL-5	rs10847864	T	G	0.1274	0.0179	0.3390	9.81E-13	0.007	50.66	
IL-5	rs111972941	G	A	0.2482	0.0514	0.9453	1.37E-06	0.006	23.32	
IL-5	rs112413063	C	T	-0.1813	0.0396	0.0557	4.62E-06	0.003	20.96	
IL-5	rs12929797	T	C	0.082	0.017	0.9771	1.32E-06	0.000	23.27	
IL-5	rs142660239	T	G	-0.3772	0.0779	0.0179	1.28E-06	0.005	23.45	100.00%
IL-5	rs144814361	T	C	0.4411	0.068	0.9871	9.07E-11	0.005	42.08	
IL-5	rs182621729	T	C	0.3083	0.0633	0.9662	1.11E-06	0.006	23.72	
IL-5	rs2208485	A	G	-0.0936	0.0182	0.3926	2.65E-07	0.004	26.45	
IL-5	rs2248244	A	G	0.1001	0.0211	0.2604	2.00E-06	0.004	22.51	
IL-5	rs2295547	C	A	-0.0894	0.0182	0.6630	8.77E-07	0.004	24.13	
IL-5	rs28370649	G	A	0.2836	0.0547	0.9831	2.20E-07	0.003	26.88	
IL-5	rs2949760	A	G	0.0915	0.0193	0.3519	2.00E-06	0.004	22.48	

IL-5	rs34311866	C	T	0.2272	0.0231	0.8131	7.97E-23	0.016	96.74	
IL-5	rs34679758	G	A	-0.1275	0.0259	0.8728	8.72E-07	0.004	24.23	
IL-5	rs356203	T	C	-0.2398	0.0178	0.6133	3.01E-41	0.027	181.49	
IL-5	rs35749011	A	G	0.7508	0.0659	0.0219	5.02E-30	0.024	129.80	
IL-5	rs41286192	G	A	0.3164	0.067	0.9642	2.30E-06	0.007	22.30	
IL-5	rs4488803	A	G	-0.1136	0.0199	0.6272	1.08E-08	0.006	32.59	
IL-5	rs4588066	A	G	0.1046	0.0178	0.3390	4.45E-09	0.005	34.53	
IL-5	rs4698412	A	G	0.1258	0.0168	0.5596	7.05E-14	0.008	56.07	
IL-5	rs4774417	A	G	0.1052	0.0192	0.7137	4.63E-08	0.005	30.02	
IL-5	rs4810687	T	G	0.0932	0.0187	0.4135	6.27E-07	0.004	24.84	
IL-5	rs4836108	A	G	0.1094	0.0225	0.5070	1.20E-06	0.006	23.64	
IL-5	rs4851487	T	C	0.0808	0.0174	0.5755	3.25E-06	0.003	21.56	
IL-5	rs61835654	C	T	0.1032	0.0216	0.2535	1.85E-06	0.004	22.83	
IL-5	rs620490	G	T	-0.1174	0.019	0.2992	6.46E-10	0.006	38.18	
IL-5	rs6741007	G	T	-0.1233	0.0175	0.4970	2.09E-12	0.008	49.64	
IL-5	rs6808178	C	T	-0.0864	0.0174	0.6292	7.20E-07	0.003	24.66	
IL-5	rs73032517	G	A	0.2706	0.0558	0.9742	1.27E-06	0.004	23.52	
IL-5	rs75505347	T	C	0.3917	0.0674	0.9791	6.12E-09	0.006	33.77	
IL-5	rs75646569	G	T	0.1916	0.0266	0.1252	5.62E-13	0.008	51.88	
IL-5	rs7695720	C	A	-0.1255	0.0208	0.7644	1.53E-09	0.006	36.40	
IL-5	rs7818035	A	G	-0.693	0.1429	0.0209	1.24E-06	0.020	23.52	
IL-5	rs79436216	G	A	0.2816	0.0603	0.9583	3.02E-06	0.006	21.81	
IL-5	rs858295	G	A	-0.1039	0.0176	0.3708	3.83E-09	0.005	34.85	
IL-5	rs9840232	T	C	0.1157	0.0233	0.8121	6.56E-07	0.004	24.66	
IL-5	rs9845968	G	A	0.0842	0.0175	0.4483	1.42E-06	0.004	23.15	
IL-6	rs10513789	G	T	-0.1596	0.0219	0.1978	3.18E-13	0.008	53.11	94.70%

IL-6	rs10756905	T	C	0.1011	0.0196	0.7654	2.46E-07	0.004	26.61
IL-6	rs10766301	T	C	0.0907	0.0192	0.6153	2.21E-06	0.004	22.32
IL-6	rs10847864	T	G	0.1274	0.0179	0.3390	9.81E-13	0.007	50.66
IL-6	rs111972941	G	A	0.2482	0.0514	0.9453	1.37E-06	0.006	23.32
IL-6	rs112413063	C	T	-0.1813	0.0396	0.0557	4.62E-06	0.003	20.96
IL-6	rs12929797	T	C	0.082	0.017	0.9771	1.32E-06	0.000	23.27
IL-6	rs142660239	T	G	-0.3772	0.0779	0.0179	1.28E-06	0.005	23.45
IL-6	rs144814361	T	C	0.4411	0.068	0.9871	9.07E-11	0.005	42.08
IL-6	rs182621729	T	C	0.3083	0.0633	0.9662	1.11E-06	0.006	23.72
IL-6	rs2208485	A	G	-0.0936	0.0182	0.3926	2.65E-07	0.004	26.45
IL-6	rs2248244	A	G	0.1001	0.0211	0.2604	2.00E-06	0.004	22.51
IL-6	rs2295547	C	A	-0.0894	0.0182	0.6630	8.77E-07	0.004	24.13
IL-6	rs28370649	G	A	0.2836	0.0547	0.9831	2.20E-07	0.003	26.88
IL-6	rs2949760	A	G	0.0915	0.0193	0.3519	2.00E-06	0.004	22.48
IL-6	rs34311866	C	T	0.2272	0.0231	0.8131	7.97E-23	0.016	96.74
IL-6	rs34679758	G	A	-0.1275	0.0259	0.8728	8.72E-07	0.004	24.23
IL-6	rs356203	T	C	-0.2398	0.0178	0.6133	3.01E-41	0.027	181.49
IL-6	rs35749011	A	G	0.7508	0.0659	0.0219	5.02E-30	0.024	129.80
IL-6	rs41286192	G	A	0.3164	0.067	0.9642	2.30E-06	0.007	22.30
IL-6	rs4488803	A	G	-0.1136	0.0199	0.6272	1.08E-08	0.006	32.59
IL-6	rs4588066	A	G	0.1046	0.0178	0.3390	4.45E-09	0.005	34.53
IL-6	rs4698412	A	G	0.1258	0.0168	0.5596	7.05E-14	0.008	56.07
IL-6	rs4774417	A	G	0.1052	0.0192	0.7137	4.63E-08	0.005	30.02
IL-6	rs4810687	T	G	0.0932	0.0187	0.4135	6.27E-07	0.004	24.84
IL-6	rs4836108	A	G	0.1094	0.0225	0.5070	1.20E-06	0.006	23.64
IL-6	rs4851487	T	C	0.0808	0.0174	0.5755	3.25E-06	0.003	21.56

IL-6	rs61835654	C	T	0.1032	0.0216	0.2535	1.85E-06	0.004	22.83	
IL-6	rs620490	G	T	-0.1174	0.019	0.2992	6.46E-10	0.006	38.18	
IL-6	rs6741007	G	T	-0.1233	0.0175	0.4970	2.09E-12	0.008	49.64	
IL-6	rs6808178	C	T	-0.0864	0.0174	0.6292	7.20E-07	0.003	24.66	
IL-6	rs73032517	G	A	0.2706	0.0558	0.9742	1.27E-06	0.004	23.52	
IL-6	rs75505347	T	C	0.3917	0.0674	0.9791	6.12E-09	0.006	33.77	
IL-6	rs75646569	G	T	0.1916	0.0266	0.1252	5.62E-13	0.008	51.88	
IL-6	rs7695720	C	A	-0.1255	0.0208	0.7644	1.53E-09	0.006	36.40	
IL-6	rs7818035	A	G	-0.693	0.1429	0.0209	1.24E-06	0.020	23.52	
IL-6	rs79436216	G	A	0.2816	0.0603	0.9583	3.02E-06	0.006	21.81	
IL-6	rs858295	G	A	-0.1039	0.0176	0.3708	3.83E-09	0.005	34.85	
IL-6	rs9840232	T	C	0.1157	0.0233	0.8121	6.56E-07	0.004	24.66	
IL-6	rs9845968	G	A	0.0842	0.0175	0.4483	1.42E-06	0.004	23.15	
IL-7	rs10513789	G	T	-0.1596	0.0219	0.1978	3.18E-13	0.008	53.11	
IL-7	rs10756905	T	C	0.1011	0.0196	0.7654	2.46E-07	0.004	26.61	
IL-7	rs10766301	T	C	0.0907	0.0192	0.6153	2.21E-06	0.004	22.32	
IL-7	rs10847864	T	G	0.1274	0.0179	0.3390	9.81E-13	0.007	50.66	
IL-7	rs111972941	G	A	0.2482	0.0514	0.9453	1.37E-06	0.006	23.32	
IL-7	rs112413063	C	T	-0.1813	0.0396	0.0557	4.62E-06	0.003	20.96	
IL-7	rs12929797	T	C	0.082	0.017	0.9771	1.32E-06	0.000	23.27	97.60%
IL-7	rs142660239	T	G	-0.3772	0.0779	0.0179	1.28E-06	0.005	23.45	
IL-7	rs144814361	T	C	0.4411	0.068	0.9871	9.07E-11	0.005	42.08	
IL-7	rs182621729	T	C	0.3083	0.0633	0.9662	1.11E-06	0.006	23.72	
IL-7	rs2208485	A	G	-0.0936	0.0182	0.3926	2.65E-07	0.004	26.45	
IL-7	rs2248244	A	G	0.1001	0.0211	0.2604	2.00E-06	0.004	22.51	
IL-7	rs2295547	C	A	-0.0894	0.0182	0.6630	8.77E-07	0.004	24.13	

IL-7	rs28370649	G	A	0.2836	0.0547	0.9831	2.20E-07	0.003	26.88
IL-7	rs2949760	A	G	0.0915	0.0193	0.3519	2.00E-06	0.004	22.48
IL-7	rs34311866	C	T	0.2272	0.0231	0.8131	7.97E-23	0.016	96.74
IL-7	rs34679758	G	A	-0.1275	0.0259	0.8728	8.72E-07	0.004	24.23
IL-7	rs356203	T	C	-0.2398	0.0178	0.6133	3.01E-41	0.027	181.49
IL-7	rs35749011	A	G	0.7508	0.0659	0.0219	5.02E-30	0.024	129.80
IL-7	rs41286192	G	A	0.3164	0.067	0.9642	2.30E-06	0.007	22.30
IL-7	rs4488803	A	G	-0.1136	0.0199	0.6272	1.08E-08	0.006	32.59
IL-7	rs4588066	A	G	0.1046	0.0178	0.3390	4.45E-09	0.005	34.53
IL-7	rs4698412	A	G	0.1258	0.0168	0.5596	7.05E-14	0.008	56.07
IL-7	rs4774417	A	G	0.1052	0.0192	0.7137	4.63E-08	0.005	30.02
IL-7	rs4810687	T	G	0.0932	0.0187	0.4135	6.27E-07	0.004	24.84
IL-7	rs4836108	A	G	0.1094	0.0225	0.5070	1.20E-06	0.006	23.64
IL-7	rs4851487	T	C	0.0808	0.0174	0.5755	3.25E-06	0.003	21.56
IL-7	rs61835654	C	T	0.1032	0.0216	0.2535	1.85E-06	0.004	22.83
IL-7	rs620490	G	T	-0.1174	0.019	0.2992	6.46E-10	0.006	38.18
IL-7	rs6741007	G	T	-0.1233	0.0175	0.4970	2.09E-12	0.008	49.64
IL-7	rs6808178	C	T	-0.0864	0.0174	0.6292	7.20E-07	0.003	24.66
IL-7	rs73032517	G	A	0.2706	0.0558	0.9742	1.27E-06	0.004	23.52
IL-7	rs75505347	T	C	0.3917	0.0674	0.9791	6.12E-09	0.006	33.77
IL-7	rs75646569	G	T	0.1916	0.0266	0.1252	5.62E-13	0.008	51.88
IL-7	rs7695720	C	A	-0.1255	0.0208	0.7644	1.53E-09	0.006	36.40
IL-7	rs7818035	A	G	-0.693	0.1429	0.0209	1.24E-06	0.020	23.52
IL-7	rs79436216	G	A	0.2816	0.0603	0.9583	3.02E-06	0.006	21.81
IL-7	rs858295	G	A	-0.1039	0.0176	0.3708	3.83E-09	0.005	34.85
IL-7	rs9840232	T	C	0.1157	0.0233	0.8121	6.56E-07	0.004	24.66

IL-7	rs9845968	G	A	0.0842	0.0175	0.4483	1.42E-06	0.004	23.15	
IL-8	rs10513789	G	T	-0.1596	0.0219	0.1978	3.18E-13	0.008	53.11	
IL-8	rs10756905	T	C	0.1011	0.0196	0.7654	2.46E-07	0.004	26.61	
IL-8	rs10766301	T	C	0.0907	0.0192	0.6153	2.21E-06	0.004	22.32	
IL-8	rs10847864	T	G	0.1274	0.0179	0.3390	9.81E-13	0.007	50.66	
IL-8	rs111972941	G	A	0.2482	0.0514	0.9453	1.37E-06	0.006	23.32	
IL-8	rs112413063	C	T	-0.1813	0.0396	0.0557	4.62E-06	0.003	20.96	
IL-8	rs12929797	T	C	0.082	0.017	0.9771	1.32E-06	0.000	23.27	
IL-8	rs142660239	T	G	-0.3772	0.0779	0.0179	1.28E-06	0.005	23.45	
IL-8	rs144814361	T	C	0.4411	0.068	0.9871	9.07E-11	0.005	42.08	
IL-8	rs182621729	T	C	0.3083	0.0633	0.9662	1.11E-06	0.006	23.72	
IL-8	rs2208485	A	G	-0.0936	0.0182	0.3926	2.65E-07	0.004	26.45	
IL-8	rs2248244	A	G	0.1001	0.0211	0.2604	2.00E-06	0.004	22.51	
IL-8	rs2295547	C	A	-0.0894	0.0182	0.6630	8.77E-07	0.004	24.13	100.00%
IL-8	rs28370649	G	A	0.2836	0.0547	0.9831	2.20E-07	0.003	26.88	
IL-8	rs2949760	A	G	0.0915	0.0193	0.3519	2.00E-06	0.004	22.48	
IL-8	rs34311866	C	T	0.2272	0.0231	0.8131	7.97E-23	0.016	96.74	
IL-8	rs34679758	G	A	-0.1275	0.0259	0.8728	8.72E-07	0.004	24.23	
IL-8	rs356203	T	C	-0.2398	0.0178	0.6133	3.01E-41	0.027	181.49	
IL-8	rs35749011	A	G	0.7508	0.0659	0.0219	5.02E-30	0.024	129.80	
IL-8	rs41286192	G	A	0.3164	0.067	0.9642	2.30E-06	0.007	22.30	
IL-8	rs4488803	A	G	-0.1136	0.0199	0.6272	1.08E-08	0.006	32.59	
IL-8	rs4588066	A	G	0.1046	0.0178	0.3390	4.45E-09	0.005	34.53	
IL-8	rs4698412	A	G	0.1258	0.0168	0.5596	7.05E-14	0.008	56.07	
IL-8	rs4774417	A	G	0.1052	0.0192	0.7137	4.63E-08	0.005	30.02	
IL-8	rs4810687	T	G	0.0932	0.0187	0.4135	6.27E-07	0.004	24.84	

IL-8	rs4836108	A	G	0.1094	0.0225	0.5070	1.20E-06	0.006	23.64	
IL-8	rs4851487	T	C	0.0808	0.0174	0.5755	3.25E-06	0.003	21.56	
IL-8	rs61835654	C	T	0.1032	0.0216	0.2535	1.85E-06	0.004	22.83	
IL-8	rs620490	G	T	-0.1174	0.019	0.2992	6.46E-10	0.006	38.18	
IL-8	rs6741007	G	T	-0.1233	0.0175	0.4970	2.09E-12	0.008	49.64	
IL-8	rs6808178	C	T	-0.0864	0.0174	0.6292	7.20E-07	0.003	24.66	
IL-8	rs73032517	G	A	0.2706	0.0558	0.9742	1.27E-06	0.004	23.52	
IL-8	rs75505347	T	C	0.3917	0.0674	0.9791	6.12E-09	0.006	33.77	
IL-8	rs75646569	G	T	0.1916	0.0266	0.1252	5.62E-13	0.008	51.88	
IL-8	rs7695720	C	A	-0.1255	0.0208	0.7644	1.53E-09	0.006	36.40	
IL-8	rs7818035	A	G	-0.693	0.1429	0.0209	1.24E-06	0.020	23.52	
IL-8	rs79436216	G	A	0.2816	0.0603	0.9583	3.02E-06	0.006	21.81	
IL-8	rs858295	G	A	-0.1039	0.0176	0.3708	3.83E-09	0.005	34.85	
IL-8	rs9840232	T	C	0.1157	0.0233	0.8121	6.56E-07	0.004	24.66	
IL-8	rs9845968	G	A	0.0842	0.0175	0.4483	1.42E-06	0.004	23.15	
IL-9	rs10513789	G	T	-0.1596	0.0219	0.1978	3.18E-13	0.008	53.11	
IL-9	rs10756905	T	C	0.1011	0.0196	0.7654	2.46E-07	0.004	26.61	
IL-9	rs10766301	T	C	0.0907	0.0192	0.6153	2.21E-06	0.004	22.32	
IL-9	rs10847864	T	G	0.1274	0.0179	0.3390	9.81E-13	0.007	50.66	
IL-9	rs111972941	G	A	0.2482	0.0514	0.9453	1.37E-06	0.006	23.32	
IL-9	rs112413063	C	T	-0.1813	0.0396	0.0557	4.62E-06	0.003	20.96	94.70%
IL-9	rs12929797	T	C	0.082	0.017	0.9771	1.32E-06	0.000	23.27	
IL-9	rs142660239	T	G	-0.3772	0.0779	0.0179	1.28E-06	0.005	23.45	
IL-9	rs144814361	T	C	0.4411	0.068	0.9871	9.07E-11	0.005	42.08	
IL-9	rs182621729	T	C	0.3083	0.0633	0.9662	1.11E-06	0.006	23.72	
IL-9	rs2208485	A	G	-0.0936	0.0182	0.3926	2.65E-07	0.004	26.45	

IL-9	rs2248244	A	G	0.1001	0.0211	0.2604	2.00E-06	0.004	22.51
IL-9	rs2295547	C	A	-0.0894	0.0182	0.6630	8.77E-07	0.004	24.13
IL-9	rs28370649	G	A	0.2836	0.0547	0.9831	2.20E-07	0.003	26.88
IL-9	rs2949760	A	G	0.0915	0.0193	0.3519	2.00E-06	0.004	22.48
IL-9	rs34311866	C	T	0.2272	0.0231	0.8131	7.97E-23	0.016	96.74
IL-9	rs34679758	G	A	-0.1275	0.0259	0.8728	8.72E-07	0.004	24.23
IL-9	rs356203	T	C	-0.2398	0.0178	0.6133	3.01E-41	0.027	181.49
IL-9	rs35749011	A	G	0.7508	0.0659	0.0219	5.02E-30	0.024	129.80
IL-9	rs41286192	G	A	0.3164	0.067	0.9642	2.30E-06	0.007	22.30
IL-9	rs4488803	A	G	-0.1136	0.0199	0.6272	1.08E-08	0.006	32.59
IL-9	rs4588066	A	G	0.1046	0.0178	0.3390	4.45E-09	0.005	34.53
IL-9	rs4698412	A	G	0.1258	0.0168	0.5596	7.05E-14	0.008	56.07
IL-9	rs4774417	A	G	0.1052	0.0192	0.7137	4.63E-08	0.005	30.02
IL-9	rs4810687	T	G	0.0932	0.0187	0.4135	6.27E-07	0.004	24.84
IL-9	rs4836108	A	G	0.1094	0.0225	0.5070	1.20E-06	0.006	23.64
IL-9	rs4851487	T	C	0.0808	0.0174	0.5755	3.25E-06	0.003	21.56
IL-9	rs61835654	C	T	0.1032	0.0216	0.2535	1.85E-06	0.004	22.83
IL-9	rs620490	G	T	-0.1174	0.019	0.2992	6.46E-10	0.006	38.18
IL-9	rs6741007	G	T	-0.1233	0.0175	0.4970	2.09E-12	0.008	49.64
IL-9	rs6808178	C	T	-0.0864	0.0174	0.6292	7.20E-07	0.003	24.66
IL-9	rs73032517	G	A	0.2706	0.0558	0.9742	1.27E-06	0.004	23.52
IL-9	rs75505347	T	C	0.3917	0.0674	0.9791	6.12E-09	0.006	33.77
IL-9	rs75646569	G	T	0.1916	0.0266	0.1252	5.62E-13	0.008	51.88
IL-9	rs7695720	C	A	-0.1255	0.0208	0.7644	1.53E-09	0.006	36.40
IL-9	rs7818035	A	G	-0.693	0.1429	0.0209	1.24E-06	0.020	23.52
IL-9	rs79436216	G	A	0.2816	0.0603	0.9583	3.02E-06	0.006	21.81

IL-9	rs858295	G	A	-0.1039	0.0176	0.3708	3.83E-09	0.005	34.85	
IL-9	rs9840232	T	C	0.1157	0.0233	0.8121	6.56E-07	0.004	24.66	
IL-9	rs9845968	G	A	0.0842	0.0175	0.4483	1.42E-06	0.004	23.15	
IP-10	rs10513789	G	T	-0.1596	0.0219	0.1978	3.18E-13	0.008	53.11	
IP-10	rs10756905	T	C	0.1011	0.0196	0.7654	2.46E-07	0.004	26.61	
IP-10	rs10766301	T	C	0.0907	0.0192	0.6153	2.21E-06	0.004	22.32	
IP-10	rs10847864	T	G	0.1274	0.0179	0.3390	9.81E-13	0.007	50.66	
IP-10	rs111972941	G	A	0.2482	0.0514	0.9453	1.37E-06	0.006	23.32	
IP-10	rs112413063	C	T	-0.1813	0.0396	0.0557	4.62E-06	0.003	20.96	
IP-10	rs12929797	T	C	0.082	0.017	0.9771	1.32E-06	0.000	23.27	
IP-10	rs142660239	T	G	-0.3772	0.0779	0.0179	1.28E-06	0.005	23.45	
IP-10	rs144814361	T	C	0.4411	0.068	0.9871	9.07E-11	0.005	42.08	
IP-10	rs182621729	T	C	0.3083	0.0633	0.9662	1.11E-06	0.006	23.72	
IP-10	rs2208485	A	G	-0.0936	0.0182	0.3926	2.65E-07	0.004	26.45	
IP-10	rs2248244	A	G	0.1001	0.0211	0.2604	2.00E-06	0.004	22.51	100.00%
IP-10	rs2295547	C	A	-0.0894	0.0182	0.6630	8.77E-07	0.004	24.13	
IP-10	rs28370649	G	A	0.2836	0.0547	0.9831	2.20E-07	0.003	26.88	
IP-10	rs2949760	A	G	0.0915	0.0193	0.3519	2.00E-06	0.004	22.48	
IP-10	rs34311866	C	T	0.2272	0.0231	0.8131	7.97E-23	0.016	96.74	
IP-10	rs34679758	G	A	-0.1275	0.0259	0.8728	8.72E-07	0.004	24.23	
IP-10	rs356203	T	C	-0.2398	0.0178	0.6133	3.01E-41	0.027	181.49	
IP-10	rs35749011	A	G	0.7508	0.0659	0.0219	5.02E-30	0.024	129.80	
IP-10	rs41286192	G	A	0.3164	0.067	0.9642	2.30E-06	0.007	22.30	
IP-10	rs4488803	A	G	-0.1136	0.0199	0.6272	1.08E-08	0.006	32.59	
IP-10	rs4588066	A	G	0.1046	0.0178	0.3390	4.45E-09	0.005	34.53	
IP-10	rs4698412	A	G	0.1258	0.0168	0.5596	7.05E-14	0.008	56.07	

IP-10	rs4774417	A	G	0.1052	0.0192	0.7137	4.63E-08	0.005	30.02	
IP-10	rs4810687	T	G	0.0932	0.0187	0.4135	6.27E-07	0.004	24.84	
IP-10	rs4836108	A	G	0.1094	0.0225	0.5070	1.20E-06	0.006	23.64	
IP-10	rs4851487	T	C	0.0808	0.0174	0.5755	3.25E-06	0.003	21.56	
IP-10	rs61835654	C	T	0.1032	0.0216	0.2535	1.85E-06	0.004	22.83	
IP-10	rs620490	G	T	-0.1174	0.019	0.2992	6.46E-10	0.006	38.18	
IP-10	rs6741007	G	T	-0.1233	0.0175	0.4970	2.09E-12	0.008	49.64	
IP-10	rs6808178	C	T	-0.0864	0.0174	0.6292	7.20E-07	0.003	24.66	
IP-10	rs73032517	G	A	0.2706	0.0558	0.9742	1.27E-06	0.004	23.52	
IP-10	rs75505347	T	C	0.3917	0.0674	0.9791	6.12E-09	0.006	33.77	
IP-10	rs75646569	G	T	0.1916	0.0266	0.1252	5.62E-13	0.008	51.88	
IP-10	rs7695720	C	A	-0.1255	0.0208	0.7644	1.53E-09	0.006	36.40	
IP-10	rs7818035	A	G	-0.693	0.1429	0.0209	1.24E-06	0.020	23.52	
IP-10	rs79436216	G	A	0.2816	0.0603	0.9583	3.02E-06	0.006	21.81	
IP-10	rs858295	G	A	-0.1039	0.0176	0.3708	3.83E-09	0.005	34.85	
IP-10	rs9840232	T	C	0.1157	0.0233	0.8121	6.56E-07	0.004	24.66	
IP-10	rs9845968	G	A	0.0842	0.0175	0.4483	1.42E-06	0.004	23.15	
MCP-1-MCAF	rs10513789	G	T	-0.1596	0.0219	0.1978	3.18E-13	0.008	53.11	
MCP-1-MCAF	rs10756905	T	C	0.1011	0.0196	0.7654	2.46E-07	0.004	26.61	
MCP-1-MCAF	rs10766301	T	C	0.0907	0.0192	0.6153	2.21E-06	0.004	22.32	
MCP-1-MCAF	rs10847864	T	G	0.1274	0.0179	0.3390	9.81E-13	0.007	50.66	
MCP-1-MCAF	rs111972941	G	A	0.2482	0.0514	0.9453	1.37E-06	0.006	23.32	10.80%
MCP-1-MCAF	rs112413063	C	T	-0.1813	0.0396	0.0557	4.62E-06	0.003	20.96	
MCP-1-MCAF	rs114797774	T	C	0.3901	0.0796	0.9771	9.43E-07	0.007	24.02	
MCP-1-MCAF	rs12929797	T	C	0.082	0.017	0.9771	1.32E-06	0.000	23.27	
MCP-1-MCAF	rs142660239	T	G	-0.3772	0.0779	0.0179	1.28E-06	0.005	23.45	

MCP-1-MCAF	rs144814361	T	C	0.4411	0.068	0.9871	9.07E-11	0.005	42.08
MCP-1-MCAF	rs182621729	T	C	0.3083	0.0633	0.9662	1.11E-06	0.006	23.72
MCP-1-MCAF	rs2208485	A	G	-0.0936	0.0182	0.3926	2.65E-07	0.004	26.45
MCP-1-MCAF	rs2248244	A	G	0.1001	0.0211	0.2604	2.00E-06	0.004	22.51
MCP-1-MCAF	rs2295547	C	A	-0.0894	0.0182	0.6630	8.77E-07	0.004	24.13
MCP-1-MCAF	rs28370649	G	A	0.2836	0.0547	0.9831	2.20E-07	0.003	26.88
MCP-1-MCAF	rs2949760	A	G	0.0915	0.0193	0.3519	2.00E-06	0.004	22.48
MCP-1-MCAF	rs34311866	C	T	0.2272	0.0231	0.8131	7.97E-23	0.016	96.74
MCP-1-MCAF	rs34679758	G	A	-0.1275	0.0259	0.8728	8.72E-07	0.004	24.23
MCP-1-MCAF	rs356203	T	C	-0.2398	0.0178	0.6133	3.01E-41	0.027	181.49
MCP-1-MCAF	rs35749011	A	G	0.7508	0.0659	0.0219	5.02E-30	0.024	129.80
MCP-1-MCAF	rs41286192	G	A	0.3164	0.067	0.9642	2.30E-06	0.007	22.30
MCP-1-MCAF	rs4488803	A	G	-0.1136	0.0199	0.6272	1.08E-08	0.006	32.59
MCP-1-MCAF	rs4588066	A	G	0.1046	0.0178	0.3390	4.45E-09	0.005	34.53
MCP-1-MCAF	rs4698412	A	G	0.1258	0.0168	0.5596	7.05E-14	0.008	56.07
MCP-1-MCAF	rs4774417	A	G	0.1052	0.0192	0.7137	4.63E-08	0.005	30.02
MCP-1-MCAF	rs4810687	T	G	0.0932	0.0187	0.4135	6.27E-07	0.004	24.84
MCP-1-MCAF	rs4836108	A	G	0.1094	0.0225	0.5070	1.20E-06	0.006	23.64
MCP-1-MCAF	rs4851487	T	C	0.0808	0.0174	0.5755	3.25E-06	0.003	21.56
MCP-1-MCAF	rs61835654	C	T	0.1032	0.0216	0.2535	1.85E-06	0.004	22.83
MCP-1-MCAF	rs620490	G	T	-0.1174	0.019	0.2992	6.46E-10	0.006	38.18
MCP-1-MCAF	rs6741007	G	T	-0.1233	0.0175	0.4970	2.09E-12	0.008	49.64
MCP-1-MCAF	rs6808178	C	T	-0.0864	0.0174	0.6292	7.20E-07	0.003	24.66
MCP-1-MCAF	rs73032517	G	A	0.2706	0.0558	0.9742	1.27E-06	0.004	23.52
MCP-1-MCAF	rs75505347	T	C	0.3917	0.0674	0.9791	6.12E-09	0.006	33.77
MCP-1-MCAF	rs75646569	G	T	0.1916	0.0266	0.1252	5.62E-13	0.008	51.88

MCP-1-MCAF	rs7695720	C	A	-0.1255	0.0208	0.7644	1.53E-09	0.006	36.40	
MCP-1-MCAF	rs7818035	A	G	-0.693	0.1429	0.0209	1.24E-06	0.020	23.52	
MCP-1-MCAF	rs79436216	G	A	0.2816	0.0603	0.9583	3.02E-06	0.006	21.81	
MCP-1-MCAF	rs858295	G	A	-0.1039	0.0176	0.3708	3.83E-09	0.005	34.85	
MCP-1-MCAF	rs9840232	T	C	0.1157	0.0233	0.8121	6.56E-07	0.004	24.66	
MCP-1-MCAF	rs9845968	G	A	0.0842	0.0175	0.4483	1.42E-06	0.004	23.15	
MCP-3	rs10513789	G	T	-0.1596	0.0219	0.1978	3.18E-13	0.008	53.11	
MCP-3	rs10756905	T	C	0.1011	0.0196	0.7654	2.46E-07	0.004	26.61	
MCP-3	rs10766301	T	C	0.0907	0.0192	0.6153	2.21E-06	0.004	22.32	
MCP-3	rs10847864	T	G	0.1274	0.0179	0.3390	9.81E-13	0.007	50.66	
MCP-3	rs111972941	G	A	0.2482	0.0514	0.9453	1.37E-06	0.006	23.32	
MCP-3	rs112413063	C	T	-0.1813	0.0396	0.0557	4.62E-06	0.003	20.96	
MCP-3	rs12929797	T	C	0.082	0.017	0.9771	1.32E-06	0.000	23.27	
MCP-3	rs142660239	T	G	-0.3772	0.0779	0.0179	1.28E-06	0.005	23.45	
MCP-3	rs2208485	A	G	-0.0936	0.0182	0.3926	2.65E-07	0.004	26.45	
MCP-3	rs2248244	A	G	0.1001	0.0211	0.2604	2.00E-06	0.004	22.51	67.30%
MCP-3	rs2295547	C	A	-0.0894	0.0182	0.6630	8.77E-07	0.004	24.13	
MCP-3	rs2949760	A	G	0.0915	0.0193	0.3519	2.00E-06	0.004	22.48	
MCP-3	rs34311866	C	T	0.2272	0.0231	0.8131	7.97E-23	0.016	96.74	
MCP-3	rs34679758	G	A	-0.1275	0.0259	0.8728	8.72E-07	0.004	24.23	
MCP-3	rs356203	T	C	-0.2398	0.0178	0.6133	3.01E-41	0.027	181.49	
MCP-3	rs35749011	A	G	0.7508	0.0659	0.0219	5.02E-30	0.024	129.80	
MCP-3	rs41286192	G	A	0.3164	0.067	0.9642	2.30E-06	0.007	22.30	
MCP-3	rs4488803	A	G	-0.1136	0.0199	0.6272	1.08E-08	0.006	32.59	
MCP-3	rs4588066	A	G	0.1046	0.0178	0.3390	4.45E-09	0.005	34.53	
MCP-3	rs4698412	A	G	0.1258	0.0168	0.5596	7.05E-14	0.008	56.07	

MCP-3	rs4774417	A	G	0.1052	0.0192	0.7137	4.63E-08	0.005	30.02
MCP-3	rs4810687	T	G	0.0932	0.0187	0.4135	6.27E-07	0.004	24.84
MCP-3	rs4836108	A	G	0.1094	0.0225	0.5070	1.20E-06	0.006	23.64
MCP-3	rs4851487	T	C	0.0808	0.0174	0.5755	3.25E-06	0.003	21.56
MCP-3	rs61835654	C	T	0.1032	0.0216	0.2535	1.85E-06	0.004	22.83
MCP-3	rs620490	G	T	-0.1174	0.019	0.2992	6.46E-10	0.006	38.18
MCP-3	rs6741007	G	T	-0.1233	0.0175	0.4970	2.09E-12	0.008	49.64
MCP-3	rs6808178	C	T	-0.0864	0.0174	0.6292	7.20E-07	0.003	24.66
MCP-3	rs73032517	G	A	0.2706	0.0558	0.9742	1.27E-06	0.004	23.52
MCP-3	rs75646569	G	T	0.1916	0.0266	0.1252	5.62E-13	0.008	51.88
MCP-3	rs7695720	C	A	-0.1255	0.0208	0.7644	1.53E-09	0.006	36.40
MCP-3	rs7818035	A	G	-0.693	0.1429	0.0209	1.24E-06	0.020	23.52
MCP-3	rs79436216	G	A	0.2816	0.0603	0.9583	3.02E-06	0.006	21.81
MCP-3	rs858295	G	A	-0.1039	0.0176	0.3708	3.83E-09	0.005	34.85
MCP-3	rs9840232	T	C	0.1157	0.0233	0.8121	6.56E-07	0.004	24.66
MCP-3	rs9845968	G	A	0.0842	0.0175	0.4483	1.42E-06	0.004	23.15
M-CSF	rs10513789	G	T	-0.1596	0.0219	0.1978	3.18E-13	0.008	53.11
M-CSF	rs10756905	T	C	0.1011	0.0196	0.7654	2.46E-07	0.004	26.61
M-CSF	rs10766301	T	C	0.0907	0.0192	0.6153	2.21E-06	0.004	22.32
M-CSF	rs10847864	T	G	0.1274	0.0179	0.3390	9.81E-13	0.007	50.66
M-CSF	rs111972941	G	A	0.2482	0.0514	0.9453	1.37E-06	0.006	23.32
M-CSF	rs112413063	C	T	-0.1813	0.0396	0.0557	4.62E-06	0.003	20.96
M-CSF	rs12929797	T	C	0.082	0.017	0.9771	1.32E-06	0.000	23.27
M-CSF	rs142660239	T	G	-0.3772	0.0779	0.0179	1.28E-06	0.005	23.45
M-CSF	rs144814361	T	C	0.4411	0.068	0.9871	9.07E-11	0.005	42.08
M-CSF	rs182621729	T	C	0.3083	0.0633	0.9662	1.11E-06	0.006	23.72

100.00%

M-CSF	rs2208485	A	G	-0.0936	0.0182	0.3926	2.65E-07	0.004	26.45
M-CSF	rs2248244	A	G	0.1001	0.0211	0.2604	2.00E-06	0.004	22.51
M-CSF	rs2295547	C	A	-0.0894	0.0182	0.6630	8.77E-07	0.004	24.13
M-CSF	rs28370649	G	A	0.2836	0.0547	0.9831	2.20E-07	0.003	26.88
M-CSF	rs2949760	A	G	0.0915	0.0193	0.3519	2.00E-06	0.004	22.48
M-CSF	rs34311866	C	T	0.2272	0.0231	0.8131	7.97E-23	0.016	96.74
M-CSF	rs34679758	G	A	-0.1275	0.0259	0.8728	8.72E-07	0.004	24.23
M-CSF	rs356203	T	C	-0.2398	0.0178	0.6133	3.01E-41	0.027	181.49
M-CSF	rs35749011	A	G	0.7508	0.0659	0.0219	5.02E-30	0.024	129.80
M-CSF	rs41286192	G	A	0.3164	0.067	0.9642	2.30E-06	0.007	22.30
M-CSF	rs4488803	A	G	-0.1136	0.0199	0.6272	1.08E-08	0.006	32.59
M-CSF	rs4588066	A	G	0.1046	0.0178	0.3390	4.45E-09	0.005	34.53
M-CSF	rs4698412	A	G	0.1258	0.0168	0.5596	7.05E-14	0.008	56.07
M-CSF	rs4774417	A	G	0.1052	0.0192	0.7137	4.63E-08	0.005	30.02
M-CSF	rs4810687	T	G	0.0932	0.0187	0.4135	6.27E-07	0.004	24.84
M-CSF	rs4836108	A	G	0.1094	0.0225	0.5070	1.20E-06	0.006	23.64
M-CSF	rs4851487	T	C	0.0808	0.0174	0.5755	3.25E-06	0.003	21.56
M-CSF	rs61835654	C	T	0.1032	0.0216	0.2535	1.85E-06	0.004	22.83
M-CSF	rs620490	G	T	-0.1174	0.019	0.2992	6.46E-10	0.006	38.18
M-CSF	rs6741007	G	T	-0.1233	0.0175	0.4970	2.09E-12	0.008	49.64
M-CSF	rs6808178	C	T	-0.0864	0.0174	0.6292	7.20E-07	0.003	24.66
M-CSF	rs73032517	G	A	0.2706	0.0558	0.9742	1.27E-06	0.004	23.52
M-CSF	rs75505347	T	C	0.3917	0.0674	0.9791	6.12E-09	0.006	33.77
M-CSF	rs75646569	G	T	0.1916	0.0266	0.1252	5.62E-13	0.008	51.88
M-CSF	rs7695720	C	A	-0.1255	0.0208	0.7644	1.53E-09	0.006	36.40
M-CSF	rs7818035	A	G	-0.693	0.1429	0.0209	1.24E-06	0.020	23.52

M-CSF	rs79436216	G	A	0.2816	0.0603	0.9583	3.02E-06	0.006	21.81	
M-CSF	rs858295	G	A	-0.1039	0.0176	0.3708	3.83E-09	0.005	34.85	
M-CSF	rs9840232	T	C	0.1157	0.0233	0.8121	6.56E-07	0.004	24.66	
M-CSF	rs9845968	G	A	0.0842	0.0175	0.4483	1.42E-06	0.004	23.15	
MIF	rs10513789	G	T	-0.1596	0.0219	0.1978	3.18E-13	0.008	53.11	
MIF	rs10756905	T	C	0.1011	0.0196	0.7654	2.46E-07	0.004	26.61	
MIF	rs10766301	T	C	0.0907	0.0192	0.6153	2.21E-06	0.004	22.32	
MIF	rs10847864	T	G	0.1274	0.0179	0.3390	9.81E-13	0.007	50.66	
MIF	rs111972941	G	A	0.2482	0.0514	0.9453	1.37E-06	0.006	23.32	
MIF	rs112413063	C	T	-0.1813	0.0396	0.0557	4.62E-06	0.003	20.96	
MIF	rs12929797	T	C	0.082	0.017	0.9771	1.32E-06	0.000	23.27	
MIF	rs142660239	T	G	-0.3772	0.0779	0.0179	1.28E-06	0.005	23.45	
MIF	rs144814361	T	C	0.4411	0.068	0.9871	9.07E-11	0.005	42.08	
MIF	rs182621729	T	C	0.3083	0.0633	0.9662	1.11E-06	0.006	23.72	
MIF	rs2208485	A	G	-0.0936	0.0182	0.3926	2.65E-07	0.004	26.45	99.90%
MIF	rs2248244	A	G	0.1001	0.0211	0.2604	2.00E-06	0.004	22.51	
MIF	rs2295547	C	A	-0.0894	0.0182	0.6630	8.77E-07	0.004	24.13	
MIF	rs28370649	G	A	0.2836	0.0547	0.9831	2.20E-07	0.003	26.88	
MIF	rs2949760	A	G	0.0915	0.0193	0.3519	2.00E-06	0.004	22.48	
MIF	rs34311866	C	T	0.2272	0.0231	0.8131	7.97E-23	0.016	96.74	
MIF	rs34679758	G	A	-0.1275	0.0259	0.8728	8.72E-07	0.004	24.23	
MIF	rs356203	T	C	-0.2398	0.0178	0.6133	3.01E-41	0.027	181.49	
MIF	rs35749011	A	G	0.7508	0.0659	0.0219	5.02E-30	0.024	129.80	
MIF	rs41286192	G	A	0.3164	0.067	0.9642	2.30E-06	0.007	22.30	
MIF	rs4488803	A	G	-0.1136	0.0199	0.6272	1.08E-08	0.006	32.59	
MIF	rs4588066	A	G	0.1046	0.0178	0.3390	4.45E-09	0.005	34.53	

MIF	rs4698412	A	G	0.1258	0.0168	0.5596	7.05E-14	0.008	56.07
MIF	rs4774417	A	G	0.1052	0.0192	0.7137	4.63E-08	0.005	30.02
MIF	rs4810687	T	G	0.0932	0.0187	0.4135	6.27E-07	0.004	24.84
MIF	rs4836108	A	G	0.1094	0.0225	0.5070	1.20E-06	0.006	23.64
MIF	rs4851487	T	C	0.0808	0.0174	0.5755	3.25E-06	0.003	21.56
MIF	rs61835654	C	T	0.1032	0.0216	0.2535	1.85E-06	0.004	22.83
MIF	rs620490	G	T	-0.1174	0.019	0.2992	6.46E-10	0.006	38.18
MIF	rs6741007	G	T	-0.1233	0.0175	0.4970	2.09E-12	0.008	49.64
MIF	rs6808178	C	T	-0.0864	0.0174	0.6292	7.20E-07	0.003	24.66
MIF	rs73032517	G	A	0.2706	0.0558	0.9742	1.27E-06	0.004	23.52
MIF	rs75505347	T	C	0.3917	0.0674	0.9791	6.12E-09	0.006	33.77
MIF	rs75646569	G	T	0.1916	0.0266	0.1252	5.62E-13	0.008	51.88
MIF	rs7695720	C	A	-0.1255	0.0208	0.7644	1.53E-09	0.006	36.40
MIF	rs7818035	A	G	-0.693	0.1429	0.0209	1.24E-06	0.020	23.52
MIF	rs79436216	G	A	0.2816	0.0603	0.9583	3.02E-06	0.006	21.81
MIF	rs858295	G	A	-0.1039	0.0176	0.3708	3.83E-09	0.005	34.85
MIF	rs9840232	T	C	0.1157	0.0233	0.8121	6.56E-07	0.004	24.66
MIF	rs9845968	G	A	0.0842	0.0175	0.4483	1.42E-06	0.004	23.15
MIG	rs10513789	G	T	-0.1596	0.0219	0.1978	3.18E-13	0.008	53.11
MIG	rs10756905	T	C	0.1011	0.0196	0.7654	2.46E-07	0.004	26.61
MIG	rs10766301	T	C	0.0907	0.0192	0.6153	2.21E-06	0.004	22.32
MIG	rs10847864	T	G	0.1274	0.0179	0.3390	9.81E-13	0.007	50.66
MIG	rs111972941	G	A	0.2482	0.0514	0.9453	1.37E-06	0.006	23.32
MIG	rs112413063	C	T	-0.1813	0.0396	0.0557	4.62E-06	0.003	20.96
MIG	rs12929797	T	C	0.082	0.017	0.9771	1.32E-06	0.000	23.27
MIG	rs142660239	T	G	-0.3772	0.0779	0.0179	1.28E-06	0.005	23.45

100.00%

MIG	rs144814361	T	C	0.4411	0.068	0.9871	9.07E-11	0.005	42.08
MIG	rs182621729	T	C	0.3083	0.0633	0.9662	1.11E-06	0.006	23.72
MIG	rs2208485	A	G	-0.0936	0.0182	0.3926	2.65E-07	0.004	26.45
MIG	rs2248244	A	G	0.1001	0.0211	0.2604	2.00E-06	0.004	22.51
MIG	rs2295547	C	A	-0.0894	0.0182	0.6630	8.77E-07	0.004	24.13
MIG	rs28370649	G	A	0.2836	0.0547	0.9831	2.20E-07	0.003	26.88
MIG	rs2949760	A	G	0.0915	0.0193	0.3519	2.00E-06	0.004	22.48
MIG	rs34311866	C	T	0.2272	0.0231	0.8131	7.97E-23	0.016	96.74
MIG	rs34679758	G	A	-0.1275	0.0259	0.8728	8.72E-07	0.004	24.23
MIG	rs356203	T	C	-0.2398	0.0178	0.6133	3.01E-41	0.027	181.49
MIG	rs35749011	A	G	0.7508	0.0659	0.0219	5.02E-30	0.024	129.80
MIG	rs41286192	G	A	0.3164	0.067	0.9642	2.30E-06	0.007	22.30
MIG	rs4488803	A	G	-0.1136	0.0199	0.6272	1.08E-08	0.006	32.59
MIG	rs4588066	A	G	0.1046	0.0178	0.3390	4.45E-09	0.005	34.53
MIG	rs4698412	A	G	0.1258	0.0168	0.5596	7.05E-14	0.008	56.07
MIG	rs4774417	A	G	0.1052	0.0192	0.7137	4.63E-08	0.005	30.02
MIG	rs4810687	T	G	0.0932	0.0187	0.4135	6.27E-07	0.004	24.84
MIG	rs4836108	A	G	0.1094	0.0225	0.5070	1.20E-06	0.006	23.64
MIG	rs4851487	T	C	0.0808	0.0174	0.5755	3.25E-06	0.003	21.56
MIG	rs61835654	C	T	0.1032	0.0216	0.2535	1.85E-06	0.004	22.83
MIG	rs620490	G	T	-0.1174	0.019	0.2992	6.46E-10	0.006	38.18
MIG	rs6741007	G	T	-0.1233	0.0175	0.4970	2.09E-12	0.008	49.64
MIG	rs6808178	C	T	-0.0864	0.0174	0.6292	7.20E-07	0.003	24.66
MIG	rs73032517	G	A	0.2706	0.0558	0.9742	1.27E-06	0.004	23.52
MIG	rs75505347	T	C	0.3917	0.0674	0.9791	6.12E-09	0.006	33.77
MIG	rs75646569	G	T	0.1916	0.0266	0.1252	5.62E-13	0.008	51.88

MIG	rs7695720	C	A	-0.1255	0.0208	0.7644	1.53E-09	0.006	36.40	
MIG	rs7818035	A	G	-0.693	0.1429	0.0209	1.24E-06	0.020	23.52	
MIG	rs79436216	G	A	0.2816	0.0603	0.9583	3.02E-06	0.006	21.81	
MIG	rs858295	G	A	-0.1039	0.0176	0.3708	3.83E-09	0.005	34.85	
MIG	rs9840232	T	C	0.1157	0.0233	0.8121	6.56E-07	0.004	24.66	
MIG	rs9845968	G	A	0.0842	0.0175	0.4483	1.42E-06	0.004	23.15	
MIP-1A	rs10513789	G	T	-0.1596	0.0219	0.1978	3.18E-13	0.008	53.11	
MIP-1A	rs10756905	T	C	0.1011	0.0196	0.7654	2.46E-07	0.004	26.61	
MIP-1A	rs10766301	T	C	0.0907	0.0192	0.6153	2.21E-06	0.004	22.32	
MIP-1A	rs10847864	T	G	0.1274	0.0179	0.3390	9.81E-13	0.007	50.66	
MIP-1A	rs111972941	G	A	0.2482	0.0514	0.9453	1.37E-06	0.006	23.32	
MIP-1A	rs112413063	C	T	-0.1813	0.0396	0.0557	4.62E-06	0.003	20.96	
MIP-1A	rs12929797	T	C	0.082	0.017	0.9771	1.32E-06	0.000	23.27	
MIP-1A	rs142660239	T	G	-0.3772	0.0779	0.0179	1.28E-06	0.005	23.45	
MIP-1A	rs144814361	T	C	0.4411	0.068	0.9871	9.07E-11	0.005	42.08	
MIP-1A	rs182621729	T	C	0.3083	0.0633	0.9662	1.11E-06	0.006	23.72	
MIP-1A	rs2208485	A	G	-0.0936	0.0182	0.3926	2.65E-07	0.004	26.45	5.50%
MIP-1A	rs2248244	A	G	0.1001	0.0211	0.2604	2.00E-06	0.004	22.51	
MIP-1A	rs2295547	C	A	-0.0894	0.0182	0.6630	8.77E-07	0.004	24.13	
MIP-1A	rs28370649	G	A	0.2836	0.0547	0.9831	2.20E-07	0.003	26.88	
MIP-1A	rs2949760	A	G	0.0915	0.0193	0.3519	2.00E-06	0.004	22.48	
MIP-1A	rs34311866	C	T	0.2272	0.0231	0.8131	7.97E-23	0.016	96.74	
MIP-1A	rs34679758	G	A	-0.1275	0.0259	0.8728	8.72E-07	0.004	24.23	
MIP-1A	rs356203	T	C	-0.2398	0.0178	0.6133	3.01E-41	0.027	181.49	
MIP-1A	rs35749011	A	G	0.7508	0.0659	0.0219	5.02E-30	0.024	129.80	
MIP-1A	rs41286192	G	A	0.3164	0.067	0.9642	2.30E-06	0.007	22.30	

MIP-1A	rs4488803	A	G	-0.1136	0.0199	0.6272	1.08E-08	0.006	32.59
MIP-1A	rs4588066	A	G	0.1046	0.0178	0.3390	4.45E-09	0.005	34.53
MIP-1A	rs4698412	A	G	0.1258	0.0168	0.5596	7.05E-14	0.008	56.07
MIP-1A	rs4774417	A	G	0.1052	0.0192	0.7137	4.63E-08	0.005	30.02
MIP-1A	rs4810687	T	G	0.0932	0.0187	0.4135	6.27E-07	0.004	24.84
MIP-1A	rs4836108	A	G	0.1094	0.0225	0.5070	1.20E-06	0.006	23.64
MIP-1A	rs4851487	T	C	0.0808	0.0174	0.5755	3.25E-06	0.003	21.56
MIP-1A	rs61835654	C	T	0.1032	0.0216	0.2535	1.85E-06	0.004	22.83
MIP-1A	rs620490	G	T	-0.1174	0.019	0.2992	6.46E-10	0.006	38.18
MIP-1A	rs6741007	G	T	-0.1233	0.0175	0.4970	2.09E-12	0.008	49.64
MIP-1A	rs6808178	C	T	-0.0864	0.0174	0.6292	7.20E-07	0.003	24.66
MIP-1A	rs73032517	G	A	0.2706	0.0558	0.9742	1.27E-06	0.004	23.52
MIP-1A	rs75505347	T	C	0.3917	0.0674	0.9791	6.12E-09	0.006	33.77
MIP-1A	rs75646569	G	T	0.1916	0.0266	0.1252	5.62E-13	0.008	51.88
MIP-1A	rs7695720	C	A	-0.1255	0.0208	0.7644	1.53E-09	0.006	36.40
MIP-1A	rs7818035	A	G	-0.693	0.1429	0.0209	1.24E-06	0.020	23.52
MIP-1A	rs79436216	G	A	0.2816	0.0603	0.9583	3.02E-06	0.006	21.81
MIP-1A	rs858295	G	A	-0.1039	0.0176	0.3708	3.83E-09	0.005	34.85
MIP-1A	rs9840232	T	C	0.1157	0.0233	0.8121	6.56E-07	0.004	24.66
MIP-1A	rs9845968	G	A	0.0842	0.0175	0.4483	1.42E-06	0.004	23.15
MIP-1B	rs10513789	G	T	-0.1596	0.0219	0.1978	3.18E-13	0.008	53.11
MIP-1B	rs10756905	T	C	0.1011	0.0196	0.7654	2.46E-07	0.004	26.61
MIP-1B	rs10766301	T	C	0.0907	0.0192	0.6153	2.21E-06	0.004	22.32
MIP-1B	rs10847864	T	G	0.1274	0.0179	0.3390	9.81E-13	0.007	50.66
MIP-1B	rs111972941	G	A	0.2482	0.0514	0.9453	1.37E-06	0.006	23.32
MIP-1B	rs112413063	C	T	-0.1813	0.0396	0.0557	4.62E-06	0.003	20.96

100.00%

MIP-1B	rs114797774	T	C	0.3901	0.0796	0.9771	9.43E-07	0.007	24.02
MIP-1B	rs12929797	T	C	0.082	0.017	0.9771	1.32E-06	0.000	23.27
MIP-1B	rs142660239	T	G	-0.3772	0.0779	0.0179	1.28E-06	0.005	23.45
MIP-1B	rs144814361	T	C	0.4411	0.068	0.9871	9.07E-11	0.005	42.08
MIP-1B	rs182621729	T	C	0.3083	0.0633	0.9662	1.11E-06	0.006	23.72
MIP-1B	rs2208485	A	G	-0.0936	0.0182	0.3926	2.65E-07	0.004	26.45
MIP-1B	rs2248244	A	G	0.1001	0.0211	0.2604	2.00E-06	0.004	22.51
MIP-1B	rs2295547	C	A	-0.0894	0.0182	0.6630	8.77E-07	0.004	24.13
MIP-1B	rs28370649	G	A	0.2836	0.0547	0.9831	2.20E-07	0.003	26.88
MIP-1B	rs2949760	A	G	0.0915	0.0193	0.3519	2.00E-06	0.004	22.48
MIP-1B	rs34311866	C	T	0.2272	0.0231	0.8131	7.97E-23	0.016	96.74
MIP-1B	rs34679758	G	A	-0.1275	0.0259	0.8728	8.72E-07	0.004	24.23
MIP-1B	rs356203	T	C	-0.2398	0.0178	0.6133	3.01E-41	0.027	181.49
MIP-1B	rs35749011	A	G	0.7508	0.0659	0.0219	5.02E-30	0.024	129.80
MIP-1B	rs41286192	G	A	0.3164	0.067	0.9642	2.30E-06	0.007	22.30
MIP-1B	rs4488803	A	G	-0.1136	0.0199	0.6272	1.08E-08	0.006	32.59
MIP-1B	rs4588066	A	G	0.1046	0.0178	0.3390	4.45E-09	0.005	34.53
MIP-1B	rs4698412	A	G	0.1258	0.0168	0.5596	7.05E-14	0.008	56.07
MIP-1B	rs4774417	A	G	0.1052	0.0192	0.7137	4.63E-08	0.005	30.02
MIP-1B	rs4810687	T	G	0.0932	0.0187	0.4135	6.27E-07	0.004	24.84
MIP-1B	rs4836108	A	G	0.1094	0.0225	0.5070	1.20E-06	0.006	23.64
MIP-1B	rs4851487	T	C	0.0808	0.0174	0.5755	3.25E-06	0.003	21.56
MIP-1B	rs61835654	C	T	0.1032	0.0216	0.2535	1.85E-06	0.004	22.83
MIP-1B	rs620490	G	T	-0.1174	0.019	0.2992	6.46E-10	0.006	38.18
MIP-1B	rs6741007	G	T	-0.1233	0.0175	0.4970	2.09E-12	0.008	49.64
MIP-1B	rs6808178	C	T	-0.0864	0.0174	0.6292	7.20E-07	0.003	24.66

MIP-1B	rs73032517	G	A	0.2706	0.0558	0.9742	1.27E-06	0.004	23.52	
MIP-1B	rs75505347	T	C	0.3917	0.0674	0.9791	6.12E-09	0.006	33.77	
MIP-1B	rs75646569	G	T	0.1916	0.0266	0.1252	5.62E-13	0.008	51.88	
MIP-1B	rs7695720	C	A	-0.1255	0.0208	0.7644	1.53E-09	0.006	36.40	
MIP-1B	rs7818035	A	G	-0.693	0.1429	0.0209	1.24E-06	0.020	23.52	
MIP-1B	rs79436216	G	A	0.2816	0.0603	0.9583	3.02E-06	0.006	21.81	
MIP-1B	rs858295	G	A	-0.1039	0.0176	0.3708	3.83E-09	0.005	34.85	
MIP-1B	rs9840232	T	C	0.1157	0.0233	0.8121	6.56E-07	0.004	24.66	
MIP-1B	rs9845968	G	A	0.0842	0.0175	0.4483	1.42E-06	0.004	23.15	
PDGF-BB	rs10513789	G	T	-0.1596	0.0219	0.1978	3.18E-13	0.008	53.11	
PDGF-BB	rs10756905	T	C	0.1011	0.0196	0.7654	2.46E-07	0.004	26.61	
PDGF-BB	rs10766301	T	C	0.0907	0.0192	0.6153	2.21E-06	0.004	22.32	
PDGF-BB	rs10847864	T	G	0.1274	0.0179	0.3390	9.81E-13	0.007	50.66	
PDGF-BB	rs111972941	G	A	0.2482	0.0514	0.9453	1.37E-06	0.006	23.32	
PDGF-BB	rs112413063	C	T	-0.1813	0.0396	0.0557	4.62E-06	0.003	20.96	
PDGF-BB	rs12929797	T	C	0.082	0.017	0.9771	1.32E-06	0.000	23.27	
PDGF-BB	rs142660239	T	G	-0.3772	0.0779	0.0179	1.28E-06	0.005	23.45	
PDGF-BB	rs144814361	T	C	0.4411	0.068	0.9871	9.07E-11	0.005	42.08	57.40%
PDGF-BB	rs182621729	T	C	0.3083	0.0633	0.9662	1.11E-06	0.006	23.72	
PDGF-BB	rs2208485	A	G	-0.0936	0.0182	0.3926	2.65E-07	0.004	26.45	
PDGF-BB	rs2248244	A	G	0.1001	0.0211	0.2604	2.00E-06	0.004	22.51	
PDGF-BB	rs2295547	C	A	-0.0894	0.0182	0.6630	8.77E-07	0.004	24.13	
PDGF-BB	rs28370649	G	A	0.2836	0.0547	0.9831	2.20E-07	0.003	26.88	
PDGF-BB	rs2949760	A	G	0.0915	0.0193	0.3519	2.00E-06	0.004	22.48	
PDGF-BB	rs34311866	C	T	0.2272	0.0231	0.8131	7.97E-23	0.016	96.74	
PDGF-BB	rs34679758	G	A	-0.1275	0.0259	0.8728	8.72E-07	0.004	24.23	

PDGF-BB	rs356203	T	C	-0.2398	0.0178	0.6133	3.01E-41	0.027	181.49	
PDGF-BB	rs35749011	A	G	0.7508	0.0659	0.0219	5.02E-30	0.024	129.80	
PDGF-BB	rs41286192	G	A	0.3164	0.067	0.9642	2.30E-06	0.007	22.30	
PDGF-BB	rs4488803	A	G	-0.1136	0.0199	0.6272	1.08E-08	0.006	32.59	
PDGF-BB	rs4588066	A	G	0.1046	0.0178	0.3390	4.45E-09	0.005	34.53	
PDGF-BB	rs4698412	A	G	0.1258	0.0168	0.5596	7.05E-14	0.008	56.07	
PDGF-BB	rs4774417	A	G	0.1052	0.0192	0.7137	4.63E-08	0.005	30.02	
PDGF-BB	rs4810687	T	G	0.0932	0.0187	0.4135	6.27E-07	0.004	24.84	
PDGF-BB	rs4836108	A	G	0.1094	0.0225	0.5070	1.20E-06	0.006	23.64	
PDGF-BB	rs4851487	T	C	0.0808	0.0174	0.5755	3.25E-06	0.003	21.56	
PDGF-BB	rs61835654	C	T	0.1032	0.0216	0.2535	1.85E-06	0.004	22.83	
PDGF-BB	rs620490	G	T	-0.1174	0.019	0.2992	6.46E-10	0.006	38.18	
PDGF-BB	rs6741007	G	T	-0.1233	0.0175	0.4970	2.09E-12	0.008	49.64	
PDGF-BB	rs6808178	C	T	-0.0864	0.0174	0.6292	7.20E-07	0.003	24.66	
PDGF-BB	rs73032517	G	A	0.2706	0.0558	0.9742	1.27E-06	0.004	23.52	
PDGF-BB	rs75505347	T	C	0.3917	0.0674	0.9791	6.12E-09	0.006	33.77	
PDGF-BB	rs75646569	G	T	0.1916	0.0266	0.1252	5.62E-13	0.008	51.88	
PDGF-BB	rs7695720	C	A	-0.1255	0.0208	0.7644	1.53E-09	0.006	36.40	
PDGF-BB	rs7818035	A	G	-0.693	0.1429	0.0209	1.24E-06	0.020	23.52	
PDGF-BB	rs79436216	G	A	0.2816	0.0603	0.9583	3.02E-06	0.006	21.81	
PDGF-BB	rs858295	G	A	-0.1039	0.0176	0.3708	3.83E-09	0.005	34.85	
PDGF-BB	rs9840232	T	C	0.1157	0.0233	0.8121	6.56E-07	0.004	24.66	
PDGF-BB	rs9845968	G	A	0.0842	0.0175	0.4483	1.42E-06	0.004	23.15	
RANTES	rs10513789	G	T	-0.1596	0.0219	0.1978	3.18E-13	0.008	53.11	
RANTES	rs10756905	T	C	0.1011	0.0196	0.7654	2.46E-07	0.004	26.61	94.70%
RANTES	rs10766301	T	C	0.0907	0.0192	0.6153	2.21E-06	0.004	22.32	

RANTES	rs10847864	T	G	0.1274	0.0179	0.3390	9.81E-13	0.007	50.66
RANTES	rs111972941	G	A	0.2482	0.0514	0.9453	1.37E-06	0.006	23.32
RANTES	rs112413063	C	T	-0.1813	0.0396	0.0557	4.62E-06	0.003	20.96
RANTES	rs12929797	T	C	0.082	0.017	0.9771	1.32E-06	0.000	23.27
RANTES	rs142660239	T	G	-0.3772	0.0779	0.0179	1.28E-06	0.005	23.45
RANTES	rs144814361	T	C	0.4411	0.068	0.9871	9.07E-11	0.005	42.08
RANTES	rs182621729	T	C	0.3083	0.0633	0.9662	1.11E-06	0.006	23.72
RANTES	rs2208485	A	G	-0.0936	0.0182	0.3926	2.65E-07	0.004	26.45
RANTES	rs2248244	A	G	0.1001	0.0211	0.2604	2.00E-06	0.004	22.51
RANTES	rs2295547	C	A	-0.0894	0.0182	0.6630	8.77E-07	0.004	24.13
RANTES	rs28370649	G	A	0.2836	0.0547	0.9831	2.20E-07	0.003	26.88
RANTES	rs2949760	A	G	0.0915	0.0193	0.3519	2.00E-06	0.004	22.48
RANTES	rs34311866	C	T	0.2272	0.0231	0.8131	7.97E-23	0.016	96.74
RANTES	rs34679758	G	A	-0.1275	0.0259	0.8728	8.72E-07	0.004	24.23
RANTES	rs356203	T	C	-0.2398	0.0178	0.6133	3.01E-41	0.027	181.49
RANTES	rs35749011	A	G	0.7508	0.0659	0.0219	5.02E-30	0.024	129.80
RANTES	rs41286192	G	A	0.3164	0.067	0.9642	2.30E-06	0.007	22.30
RANTES	rs4488803	A	G	-0.1136	0.0199	0.6272	1.08E-08	0.006	32.59
RANTES	rs4588066	A	G	0.1046	0.0178	0.3390	4.45E-09	0.005	34.53
RANTES	rs4698412	A	G	0.1258	0.0168	0.5596	7.05E-14	0.008	56.07
RANTES	rs4774417	A	G	0.1052	0.0192	0.7137	4.63E-08	0.005	30.02
RANTES	rs4810687	T	G	0.0932	0.0187	0.4135	6.27E-07	0.004	24.84
RANTES	rs4836108	A	G	0.1094	0.0225	0.5070	1.20E-06	0.006	23.64
RANTES	rs4851487	T	C	0.0808	0.0174	0.5755	3.25E-06	0.003	21.56
RANTES	rs61835654	C	T	0.1032	0.0216	0.2535	1.85E-06	0.004	22.83
RANTES	rs620490	G	T	-0.1174	0.019	0.2992	6.46E-10	0.006	38.18

RANTES	rs6741007	G	T	-0.1233	0.0175	0.4970	2.09E-12	0.008	49.64	
RANTES	rs6808178	C	T	-0.0864	0.0174	0.6292	7.20E-07	0.003	24.66	
RANTES	rs73032517	G	A	0.2706	0.0558	0.9742	1.27E-06	0.004	23.52	
RANTES	rs75505347	T	C	0.3917	0.0674	0.9791	6.12E-09	0.006	33.77	
RANTES	rs75646569	G	T	0.1916	0.0266	0.1252	5.62E-13	0.008	51.88	
RANTES	rs7695720	C	A	-0.1255	0.0208	0.7644	1.53E-09	0.006	36.40	
RANTES	rs7818035	A	G	-0.693	0.1429	0.0209	1.24E-06	0.020	23.52	
RANTES	rs79436216	G	A	0.2816	0.0603	0.9583	3.02E-06	0.006	21.81	
RANTES	rs858295	G	A	-0.1039	0.0176	0.3708	3.83E-09	0.005	34.85	
RANTES	rs9840232	T	C	0.1157	0.0233	0.8121	6.56E-07	0.004	24.66	
RANTES	rs9845968	G	A	0.0842	0.0175	0.4483	1.42E-06	0.004	23.15	
SCF	rs10513789	G	T	-0.1596	0.0219	0.1978	3.18E-13	0.008	53.11	
SCF	rs10756905	T	C	0.1011	0.0196	0.7654	2.46E-07	0.004	26.61	
SCF	rs10766301	T	C	0.0907	0.0192	0.6153	2.21E-06	0.004	22.32	
SCF	rs10847864	T	G	0.1274	0.0179	0.3390	9.81E-13	0.007	50.66	
SCF	rs111972941	G	A	0.2482	0.0514	0.9453	1.37E-06	0.006	23.32	
SCF	rs112413063	C	T	-0.1813	0.0396	0.0557	4.62E-06	0.003	20.96	
SCF	rs12929797	T	C	0.082	0.017	0.9771	1.32E-06	0.000	23.27	
SCF	rs142660239	T	G	-0.3772	0.0779	0.0179	1.28E-06	0.005	23.45	70.70%
SCF	rs144814361	T	C	0.4411	0.068	0.9871	9.07E-11	0.005	42.08	
SCF	rs182621729	T	C	0.3083	0.0633	0.9662	1.11E-06	0.006	23.72	
SCF	rs2208485	A	G	-0.0936	0.0182	0.3926	2.65E-07	0.004	26.45	
SCF	rs2248244	A	G	0.1001	0.0211	0.2604	2.00E-06	0.004	22.51	
SCF	rs2295547	C	A	-0.0894	0.0182	0.6630	8.77E-07	0.004	24.13	
SCF	rs28370649	G	A	0.2836	0.0547	0.9831	2.20E-07	0.003	26.88	
SCF	rs2949760	A	G	0.0915	0.0193	0.3519	2.00E-06	0.004	22.48	

SCF	rs34311866	C	T	0.2272	0.0231	0.8131	7.97E-23	0.016	96.74	
SCF	rs34679758	G	A	-0.1275	0.0259	0.8728	8.72E-07	0.004	24.23	
SCF	rs356203	T	C	-0.2398	0.0178	0.6133	3.01E-41	0.027	181.49	
SCF	rs35749011	A	G	0.7508	0.0659	0.0219	5.02E-30	0.024	129.80	
SCF	rs41286192	G	A	0.3164	0.067	0.9642	2.30E-06	0.007	22.30	
SCF	rs4488803	A	G	-0.1136	0.0199	0.6272	1.08E-08	0.006	32.59	
SCF	rs4588066	A	G	0.1046	0.0178	0.3390	4.45E-09	0.005	34.53	
SCF	rs4698412	A	G	0.1258	0.0168	0.5596	7.05E-14	0.008	56.07	
SCF	rs4774417	A	G	0.1052	0.0192	0.7137	4.63E-08	0.005	30.02	
SCF	rs4810687	T	G	0.0932	0.0187	0.4135	6.27E-07	0.004	24.84	
SCF	rs4836108	A	G	0.1094	0.0225	0.5070	1.20E-06	0.006	23.64	
SCF	rs4851487	T	C	0.0808	0.0174	0.5755	3.25E-06	0.003	21.56	
SCF	rs61835654	C	T	0.1032	0.0216	0.2535	1.85E-06	0.004	22.83	
SCF	rs620490	G	T	-0.1174	0.019	0.2992	6.46E-10	0.006	38.18	
SCF	rs6741007	G	T	-0.1233	0.0175	0.4970	2.09E-12	0.008	49.64	
SCF	rs6808178	C	T	-0.0864	0.0174	0.6292	7.20E-07	0.003	24.66	
SCF	rs73032517	G	A	0.2706	0.0558	0.9742	1.27E-06	0.004	23.52	
SCF	rs75505347	T	C	0.3917	0.0674	0.9791	6.12E-09	0.006	33.77	
SCF	rs75646569	G	T	0.1916	0.0266	0.1252	5.62E-13	0.008	51.88	
SCF	rs7695720	C	A	-0.1255	0.0208	0.7644	1.53E-09	0.006	36.40	
SCF	rs7818035	A	G	-0.693	0.1429	0.0209	1.24E-06	0.020	23.52	
SCF	rs79436216	G	A	0.2816	0.0603	0.9583	3.02E-06	0.006	21.81	
SCF	rs858295	G	A	-0.1039	0.0176	0.3708	3.83E-09	0.005	34.85	
SCF	rs9840232	T	C	0.1157	0.0233	0.8121	6.56E-07	0.004	24.66	
SCF	rs9845968	G	A	0.0842	0.0175	0.4483	1.42E-06	0.004	23.15	
SCGF	rs10513789	G	T	-0.1596	0.0219	0.1978	3.18E-13	0.008	53.11	100.00%

SCGF	rs10756905	T	C	0.1011	0.0196	0.7654	2.46E-07	0.004	26.61
SCGF	rs10766301	T	C	0.0907	0.0192	0.6153	2.21E-06	0.004	22.32
SCGF	rs10847864	T	G	0.1274	0.0179	0.3390	9.81E-13	0.007	50.66
SCGF	rs111972941	G	A	0.2482	0.0514	0.9453	1.37E-06	0.006	23.32
SCGF	rs112413063	C	T	-0.1813	0.0396	0.0557	4.62E-06	0.003	20.96
SCGF	rs12929797	T	C	0.082	0.017	0.9771	1.32E-06	0.000	23.27
SCGF	rs142660239	T	G	-0.3772	0.0779	0.0179	1.28E-06	0.005	23.45
SCGF	rs144814361	T	C	0.4411	0.068	0.9871	9.07E-11	0.005	42.08
SCGF	rs2208485	A	G	-0.0936	0.0182	0.3926	2.65E-07	0.004	26.45
SCGF	rs2248244	A	G	0.1001	0.0211	0.2604	2.00E-06	0.004	22.51
SCGF	rs2295547	C	A	-0.0894	0.0182	0.6630	8.77E-07	0.004	24.13
SCGF	rs28370649	G	A	0.2836	0.0547	0.9831	2.20E-07	0.003	26.88
SCGF	rs2949760	A	G	0.0915	0.0193	0.3519	2.00E-06	0.004	22.48
SCGF	rs34311866	C	T	0.2272	0.0231	0.8131	7.97E-23	0.016	96.74
SCGF	rs34679758	G	A	-0.1275	0.0259	0.8728	8.72E-07	0.004	24.23
SCGF	rs356203	T	C	-0.2398	0.0178	0.6133	3.01E-41	0.027	181.49
SCGF	rs35749011	A	G	0.7508	0.0659	0.0219	5.02E-30	0.024	129.80
SCGF	rs41286192	G	A	0.3164	0.067	0.9642	2.30E-06	0.007	22.30
SCGF	rs4488803	A	G	-0.1136	0.0199	0.6272	1.08E-08	0.006	32.59
SCGF	rs4588066	A	G	0.1046	0.0178	0.3390	4.45E-09	0.005	34.53
SCGF	rs4698412	A	G	0.1258	0.0168	0.5596	7.05E-14	0.008	56.07
SCGF	rs4774417	A	G	0.1052	0.0192	0.7137	4.63E-08	0.005	30.02
SCGF	rs4810687	T	G	0.0932	0.0187	0.4135	6.27E-07	0.004	24.84
SCGF	rs4836108	A	G	0.1094	0.0225	0.5070	1.20E-06	0.006	23.64
SCGF	rs4851487	T	C	0.0808	0.0174	0.5755	3.25E-06	0.003	21.56
SCGF	rs61835654	C	T	0.1032	0.0216	0.2535	1.85E-06	0.004	22.83

SCGF	rs620490	G	T	-0.1174	0.019	0.2992	6.46E-10	0.006	38.18	
SCGF	rs6741007	G	T	-0.1233	0.0175	0.4970	2.09E-12	0.008	49.64	
SCGF	rs6808178	C	T	-0.0864	0.0174	0.6292	7.20E-07	0.003	24.66	
SCGF	rs73032517	G	A	0.2706	0.0558	0.9742	1.27E-06	0.004	23.52	
SCGF	rs75505347	T	C	0.3917	0.0674	0.9791	6.12E-09	0.006	33.77	
SCGF	rs75646569	G	T	0.1916	0.0266	0.1252	5.62E-13	0.008	51.88	
SCGF	rs7695720	C	A	-0.1255	0.0208	0.7644	1.53E-09	0.006	36.40	
SCGF	rs7818035	A	G	-0.693	0.1429	0.0209	1.24E-06	0.020	23.52	
SCGF	rs79436216	G	A	0.2816	0.0603	0.9583	3.02E-06	0.006	21.81	
SCGF	rs858295	G	A	-0.1039	0.0176	0.3708	3.83E-09	0.005	34.85	
SCGF	rs9840232	T	C	0.1157	0.0233	0.8121	6.56E-07	0.004	24.66	
SCGF	rs9845968	G	A	0.0842	0.0175	0.4483	1.42E-06	0.004	23.15	
SDF-1A	rs10513789	G	T	-0.1596	0.0219	0.1978	3.18E-13	0.008	53.11	
SDF-1A	rs10756905	T	C	0.1011	0.0196	0.7654	2.46E-07	0.004	26.61	
SDF-1A	rs10766301	T	C	0.0907	0.0192	0.6153	2.21E-06	0.004	22.32	
SDF-1A	rs10847864	T	G	0.1274	0.0179	0.3390	9.81E-13	0.007	50.66	
SDF-1A	rs111972941	G	A	0.2482	0.0514	0.9453	1.37E-06	0.006	23.32	
SDF-1A	rs112413063	C	T	-0.1813	0.0396	0.0557	4.62E-06	0.003	20.96	
SDF-1A	rs12929797	T	C	0.082	0.017	0.9771	1.32E-06	0.000	23.27	95.00%
SDF-1A	rs142660239	T	G	-0.3772	0.0779	0.0179	1.28E-06	0.005	23.45	
SDF-1A	rs144814361	T	C	0.4411	0.068	0.9871	9.07E-11	0.005	42.08	
SDF-1A	rs182621729	T	C	0.3083	0.0633	0.9662	1.11E-06	0.006	23.72	
SDF-1A	rs2208485	A	G	-0.0936	0.0182	0.3926	2.65E-07	0.004	26.45	
SDF-1A	rs2248244	A	G	0.1001	0.0211	0.2604	2.00E-06	0.004	22.51	
SDF-1A	rs2295547	C	A	-0.0894	0.0182	0.6630	8.77E-07	0.004	24.13	
SDF-1A	rs28370649	G	A	0.2836	0.0547	0.9831	2.20E-07	0.003	26.88	

SDF-1A	rs2949760	A	G	0.0915	0.0193	0.3519	2.00E-06	0.004	22.48
SDF-1A	rs34311866	C	T	0.2272	0.0231	0.8131	7.97E-23	0.016	96.74
SDF-1A	rs34679758	G	A	-0.1275	0.0259	0.8728	8.72E-07	0.004	24.23
SDF-1A	rs356203	T	C	-0.2398	0.0178	0.6133	3.01E-41	0.027	181.49
SDF-1A	rs35749011	A	G	0.7508	0.0659	0.0219	5.02E-30	0.024	129.80
SDF-1A	rs41286192	G	A	0.3164	0.067	0.9642	2.30E-06	0.007	22.30
SDF-1A	rs4488803	A	G	-0.1136	0.0199	0.6272	1.08E-08	0.006	32.59
SDF-1A	rs4588066	A	G	0.1046	0.0178	0.3390	4.45E-09	0.005	34.53
SDF-1A	rs4698412	A	G	0.1258	0.0168	0.5596	7.05E-14	0.008	56.07
SDF-1A	rs4774417	A	G	0.1052	0.0192	0.7137	4.63E-08	0.005	30.02
SDF-1A	rs4810687	T	G	0.0932	0.0187	0.4135	6.27E-07	0.004	24.84
SDF-1A	rs4836108	A	G	0.1094	0.0225	0.5070	1.20E-06	0.006	23.64
SDF-1A	rs4851487	T	C	0.0808	0.0174	0.5755	3.25E-06	0.003	21.56
SDF-1A	rs61835654	C	T	0.1032	0.0216	0.2535	1.85E-06	0.004	22.83
SDF-1A	rs620490	G	T	-0.1174	0.019	0.2992	6.46E-10	0.006	38.18
SDF-1A	rs6715875	C	T	0.2902	0.0599	0.0249	1.25E-06	0.004	23.47
SDF-1A	rs6741007	G	T	-0.1233	0.0175	0.4970	2.09E-12	0.008	49.64
SDF-1A	rs6808178	C	T	-0.0864	0.0174	0.6292	7.20E-07	0.003	24.66
SDF-1A	rs73032517	G	A	0.2706	0.0558	0.9742	1.27E-06	0.004	23.52
SDF-1A	rs75505347	T	C	0.3917	0.0674	0.9791	6.12E-09	0.006	33.77
SDF-1A	rs75646569	G	T	0.1916	0.0266	0.1252	5.62E-13	0.008	51.88
SDF-1A	rs7695720	C	A	-0.1255	0.0208	0.7644	1.53E-09	0.006	36.40
SDF-1A	rs7818035	A	G	-0.693	0.1429	0.0209	1.24E-06	0.020	23.52
SDF-1A	rs79436216	G	A	0.2816	0.0603	0.9583	3.02E-06	0.006	21.81
SDF-1A	rs858295	G	A	-0.1039	0.0176	0.3708	3.83E-09	0.005	34.85
SDF-1A	rs9840232	T	C	0.1157	0.0233	0.8121	6.56E-07	0.004	24.66

SDF-1A	rs9845968	G	A	0.0842	0.0175	0.4483	1.42E-06	0.004	23.15		
TNF-A	rs10513789	G	T	-0.1596	0.0219	0.1978	3.18E-13	0.008	53.11		
TNF-A	rs10756905	T	C	0.1011	0.0196	0.7654	2.46E-07	0.004	26.61		
TNF-A	rs10766301	T	C	0.0907	0.0192	0.6153	2.21E-06	0.004	22.32		
TNF-A	rs10847864	T	G	0.1274	0.0179	0.3390	9.81E-13	0.007	50.66		
TNF-A	rs111972941	G	A	0.2482	0.0514	0.9453	1.37E-06	0.006	23.32		
TNF-A	rs112413063	C	T	-0.1813	0.0396	0.0557	4.62E-06	0.003	20.96		
TNF-A	rs12929797	T	C	0.082	0.017	0.9771	1.32E-06	0.000	23.27		
TNF-A	rs142660239	T	G	-0.3772	0.0779	0.0179	1.28E-06	0.005	23.45		
TNF-A	rs144814361	T	C	0.4411	0.068	0.9871	9.07E-11	0.005	42.08		
TNF-A	rs182621729	T	C	0.3083	0.0633	0.9662	1.11E-06	0.006	23.72		
TNF-A	rs2208485	A	G	-0.0936	0.0182	0.3926	2.65E-07	0.004	26.45		
TNF-A	rs2248244	A	G	0.1001	0.0211	0.2604	2.00E-06	0.004	22.51		
TNF-A	rs2295547	C	A	-0.0894	0.0182	0.6630	8.77E-07	0.004	24.13	100.00%	
TNF-A	rs28370649	G	A	0.2836	0.0547	0.9831	2.20E-07	0.003	26.88		
TNF-A	rs2949760	A	G	0.0915	0.0193	0.3519	2.00E-06	0.004	22.48		
TNF-A	rs34311866	C	T	0.2272	0.0231	0.8131	7.97E-23	0.016	96.74		
TNF-A	rs34679758	G	A	-0.1275	0.0259	0.8728	8.72E-07	0.004	24.23		
TNF-A	rs356203	T	C	-0.2398	0.0178	0.6133	3.01E-41	0.027	181.49		
TNF-A	rs35749011	A	G	0.7508	0.0659	0.0219	5.02E-30	0.024	129.80		
TNF-A	rs41286192	G	A	0.3164	0.067	0.9642	2.30E-06	0.007	22.30		
TNF-A	rs4488803	A	G	-0.1136	0.0199	0.6272	1.08E-08	0.006	32.59		
TNF-A	rs4588066	A	G	0.1046	0.0178	0.3390	4.45E-09	0.005	34.53		
TNF-A	rs4698412	A	G	0.1258	0.0168	0.5596	7.05E-14	0.008	56.07		
TNF-A	rs4774417	A	G	0.1052	0.0192	0.7137	4.63E-08	0.005	30.02		
TNF-A	rs4810687	T	G	0.0932	0.0187	0.4135	6.27E-07	0.004	24.84		

TNF-A	rs4836108	A	G	0.1094	0.0225	0.5070	1.20E-06	0.006	23.64	
TNF-A	rs4851487	T	C	0.0808	0.0174	0.5755	3.25E-06	0.003	21.56	
TNF-A	rs61835654	C	T	0.1032	0.0216	0.2535	1.85E-06	0.004	22.83	
TNF-A	rs620490	G	T	-0.1174	0.019	0.2992	6.46E-10	0.006	38.18	
TNF-A	rs6741007	G	T	-0.1233	0.0175	0.4970	2.09E-12	0.008	49.64	
TNF-A	rs6808178	C	T	-0.0864	0.0174	0.6292	7.20E-07	0.003	24.66	
TNF-A	rs73032517	G	A	0.2706	0.0558	0.9742	1.27E-06	0.004	23.52	
TNF-A	rs75505347	T	C	0.3917	0.0674	0.9791	6.12E-09	0.006	33.77	
TNF-A	rs75646569	G	T	0.1916	0.0266	0.1252	5.62E-13	0.008	51.88	
TNF-A	rs7695720	C	A	-0.1255	0.0208	0.7644	1.53E-09	0.006	36.40	
TNF-A	rs7818035	A	G	-0.693	0.1429	0.0209	1.24E-06	0.020	23.52	
TNF-A	rs79436216	G	A	0.2816	0.0603	0.9583	3.02E-06	0.006	21.81	
TNF-A	rs858295	G	A	-0.1039	0.0176	0.3708	3.83E-09	0.005	34.85	
TNF-A	rs9840232	T	C	0.1157	0.0233	0.8121	6.56E-07	0.004	24.66	
TNF-A	rs9845968	G	A	0.0842	0.0175	0.4483	1.42E-06	0.004	23.15	
TNF-B	rs10513789	G	T	-0.1596	0.0219	0.1978	3.18E-13	0.008	53.11	
TNF-B	rs10756905	T	C	0.1011	0.0196	0.7654	2.46E-07	0.004	26.61	
TNF-B	rs10766301	T	C	0.0907	0.0192	0.6153	2.21E-06	0.004	22.32	
TNF-B	rs10847864	T	G	0.1274	0.0179	0.3390	9.81E-13	0.007	50.66	
TNF-B	rs111972941	G	A	0.2482	0.0514	0.9453	1.37E-06	0.006	23.32	
TNF-B	rs112413063	C	T	-0.1813	0.0396	0.0557	4.62E-06	0.003	20.96	100.00%
TNF-B	rs12929797	T	C	0.082	0.017	0.9771	1.32E-06	0.000	23.27	
TNF-B	rs2208485	A	G	-0.0936	0.0182	0.3926	2.65E-07	0.004	26.45	
TNF-B	rs2248244	A	G	0.1001	0.0211	0.2604	2.00E-06	0.004	22.51	
TNF-B	rs2295547	C	A	-0.0894	0.0182	0.6630	8.77E-07	0.004	24.13	
TNF-B	rs2949760	A	G	0.0915	0.0193	0.3519	2.00E-06	0.004	22.48	

TNF-B	rs34311866	C	T	0.2272	0.0231	0.8131	7.97E-23	0.016	96.74	
TNF-B	rs34679758	G	A	-0.1275	0.0259	0.8728	8.72E-07	0.004	24.23	
TNF-B	rs356203	T	C	-0.2398	0.0178	0.6133	3.01E-41	0.027	181.49	
TNF-B	rs35749011	A	G	0.7508	0.0659	0.0219	5.02E-30	0.024	129.80	
TNF-B	rs41286192	G	A	0.3164	0.067	0.9642	2.30E-06	0.007	22.30	
TNF-B	rs4488803	A	G	-0.1136	0.0199	0.6272	1.08E-08	0.006	32.59	
TNF-B	rs4588066	A	G	0.1046	0.0178	0.3390	4.45E-09	0.005	34.53	
TNF-B	rs4698412	A	G	0.1258	0.0168	0.5596	7.05E-14	0.008	56.07	
TNF-B	rs4774417	A	G	0.1052	0.0192	0.7137	4.63E-08	0.005	30.02	
TNF-B	rs4810687	T	G	0.0932	0.0187	0.4135	6.27E-07	0.004	24.84	
TNF-B	rs4836108	A	G	0.1094	0.0225	0.5070	1.20E-06	0.006	23.64	
TNF-B	rs4851487	T	C	0.0808	0.0174	0.5755	3.25E-06	0.003	21.56	
TNF-B	rs61835654	C	T	0.1032	0.0216	0.2535	1.85E-06	0.004	22.83	
TNF-B	rs620490	G	T	-0.1174	0.019	0.2992	6.46E-10	0.006	38.18	
TNF-B	rs6741007	G	T	-0.1233	0.0175	0.4970	2.09E-12	0.008	49.64	
TNF-B	rs6808178	C	T	-0.0864	0.0174	0.6292	7.20E-07	0.003	24.66	
TNF-B	rs73032517	G	A	0.2706	0.0558	0.9742	1.27E-06	0.004	23.52	
TNF-B	rs75646569	G	T	0.1916	0.0266	0.1252	5.62E-13	0.008	51.88	
TNF-B	rs7695720	C	A	-0.1255	0.0208	0.7644	1.53E-09	0.006	36.40	
TNF-B	rs79436216	G	A	0.2816	0.0603	0.9583	3.02E-06	0.006	21.81	
TNF-B	rs858295	G	A	-0.1039	0.0176	0.3708	3.83E-09	0.005	34.85	
TNF-B	rs9840232	T	C	0.1157	0.0233	0.8121	6.56E-07	0.004	24.66	
TNF-B	rs9845968	G	A	0.0842	0.0175	0.4483	1.42E-06	0.004	23.15	
TRAIL	rs10513789	G	T	-0.1596	0.0219	0.1978	3.18E-13	0.008	53.11	
TRAIL	rs10756905	T	C	0.1011	0.0196	0.7654	2.46E-07	0.004	26.61	71.30%
TRAIL	rs10766301	T	C	0.0907	0.0192	0.6153	2.21E-06	0.004	22.32	

TRAIL	rs10847864	T	G	0.1274	0.0179	0.3390	9.81E-13	0.007	50.66
TRAIL	rs111972941	G	A	0.2482	0.0514	0.9453	1.37E-06	0.006	23.32
TRAIL	rs112413063	C	T	-0.1813	0.0396	0.0557	4.62E-06	0.003	20.96
TRAIL	rs12929797	T	C	0.082	0.017	0.9771	1.32E-06	0.000	23.27
TRAIL	rs142660239	T	G	-0.3772	0.0779	0.0179	1.28E-06	0.005	23.45
TRAIL	rs144814361	T	C	0.4411	0.068	0.9871	9.07E-11	0.005	42.08
TRAIL	rs182621729	T	C	0.3083	0.0633	0.9662	1.11E-06	0.006	23.72
TRAIL	rs2208485	A	G	-0.0936	0.0182	0.3926	2.65E-07	0.004	26.45
TRAIL	rs2248244	A	G	0.1001	0.0211	0.2604	2.00E-06	0.004	22.51
TRAIL	rs2295547	C	A	-0.0894	0.0182	0.6630	8.77E-07	0.004	24.13
TRAIL	rs28370649	G	A	0.2836	0.0547	0.9831	2.20E-07	0.003	26.88
TRAIL	rs2949760	A	G	0.0915	0.0193	0.3519	2.00E-06	0.004	22.48
TRAIL	rs34311866	C	T	0.2272	0.0231	0.8131	7.97E-23	0.016	96.74
TRAIL	rs34679758	G	A	-0.1275	0.0259	0.8728	8.72E-07	0.004	24.23
TRAIL	rs356203	T	C	-0.2398	0.0178	0.6133	3.01E-41	0.027	181.49
TRAIL	rs35749011	A	G	0.7508	0.0659	0.0219	5.02E-30	0.024	129.80
TRAIL	rs41286192	G	A	0.3164	0.067	0.9642	2.30E-06	0.007	22.30
TRAIL	rs4488803	A	G	-0.1136	0.0199	0.6272	1.08E-08	0.006	32.59
TRAIL	rs4588066	A	G	0.1046	0.0178	0.3390	4.45E-09	0.005	34.53
TRAIL	rs4698412	A	G	0.1258	0.0168	0.5596	7.05E-14	0.008	56.07
TRAIL	rs4774417	A	G	0.1052	0.0192	0.7137	4.63E-08	0.005	30.02
TRAIL	rs4810687	T	G	0.0932	0.0187	0.4135	6.27E-07	0.004	24.84
TRAIL	rs4836108	A	G	0.1094	0.0225	0.5070	1.20E-06	0.006	23.64
TRAIL	rs4851487	T	C	0.0808	0.0174	0.5755	3.25E-06	0.003	21.56
TRAIL	rs61835654	C	T	0.1032	0.0216	0.2535	1.85E-06	0.004	22.83
TRAIL	rs620490	G	T	-0.1174	0.019	0.2992	6.46E-10	0.006	38.18

TRAIL	rs6715875	C	T	0.2902	0.0599	0.0249	1.25E-06	0.004	23.47
TRAIL	rs6741007	G	T	-0.1233	0.0175	0.4970	2.09E-12	0.008	49.64
TRAIL	rs6808178	C	T	-0.0864	0.0174	0.6292	7.20E-07	0.003	24.66
TRAIL	rs73032517	G	A	0.2706	0.0558	0.9742	1.27E-06	0.004	23.52
TRAIL	rs75505347	T	C	0.3917	0.0674	0.9791	6.12E-09	0.006	33.77
TRAIL	rs75646569	G	T	0.1916	0.0266	0.1252	5.62E-13	0.008	51.88
TRAIL	rs7695720	C	A	-0.1255	0.0208	0.7644	1.53E-09	0.006	36.40
TRAIL	rs7818035	A	G	-0.693	0.1429	0.0209	1.24E-06	0.020	23.52
TRAIL	rs79436216	G	A	0.2816	0.0603	0.9583	3.02E-06	0.006	21.81
TRAIL	rs858295	G	A	-0.1039	0.0176	0.3708	3.83E-09	0.005	34.85
TRAIL	rs9840232	T	C	0.1157	0.0233	0.8121	6.56E-07	0.004	24.66
TRAIL	rs9845968	G	A	0.0842	0.0175	0.4483	1.42E-06	0.004	23.15
VEGF	rs10513789	G	T	-0.1596	0.0219	0.1978	3.18E-13	0.008	53.11
VEGF	rs10756905	T	C	0.1011	0.0196	0.7654	2.46E-07	0.004	26.61
VEGF	rs10766301	T	C	0.0907	0.0192	0.6153	2.21E-06	0.004	22.32
VEGF	rs10847864	T	G	0.1274	0.0179	0.3390	9.81E-13	0.007	50.66
VEGF	rs111972941	G	A	0.2482	0.0514	0.9453	1.37E-06	0.006	23.32
VEGF	rs112413063	C	T	-0.1813	0.0396	0.0557	4.62E-06	0.003	20.96
VEGF	rs12929797	T	C	0.082	0.017	0.9771	1.32E-06	0.000	23.27
VEGF	rs142660239	T	G	-0.3772	0.0779	0.0179	1.28E-06	0.005	23.45
VEGF	rs144814361	T	C	0.4411	0.068	0.9871	9.07E-11	0.005	42.08
VEGF	rs182621729	T	C	0.3083	0.0633	0.9662	1.11E-06	0.006	23.72
VEGF	rs2208485	A	G	-0.0936	0.0182	0.3926	2.65E-07	0.004	26.45
VEGF	rs2248244	A	G	0.1001	0.0211	0.2604	2.00E-06	0.004	22.51
VEGF	rs2295547	C	A	-0.0894	0.0182	0.6630	8.77E-07	0.004	24.13
VEGF	rs28370649	G	A	0.2836	0.0547	0.9831	2.20E-07	0.003	26.88

100.00%

VEGF	rs2949760	A	G	0.0915	0.0193	0.3519	2.00E-06	0.004	22.48
VEGF	rs34311866	C	T	0.2272	0.0231	0.8131	7.97E-23	0.016	96.74
VEGF	rs34679758	G	A	-0.1275	0.0259	0.8728	8.72E-07	0.004	24.23
VEGF	rs356203	T	C	-0.2398	0.0178	0.6133	3.01E-41	0.027	181.49
VEGF	rs35749011	A	G	0.7508	0.0659	0.0219	5.02E-30	0.024	129.80
VEGF	rs41286192	G	A	0.3164	0.067	0.9642	2.30E-06	0.007	22.30
VEGF	rs4488803	A	G	-0.1136	0.0199	0.6272	1.08E-08	0.006	32.59
VEGF	rs4588066	A	G	0.1046	0.0178	0.3390	4.45E-09	0.005	34.53
VEGF	rs4698412	A	G	0.1258	0.0168	0.5596	7.05E-14	0.008	56.07
VEGF	rs4774417	A	G	0.1052	0.0192	0.7137	4.63E-08	0.005	30.02
VEGF	rs4810687	T	G	0.0932	0.0187	0.4135	6.27E-07	0.004	24.84
VEGF	rs4836108	A	G	0.1094	0.0225	0.5070	1.20E-06	0.006	23.64
VEGF	rs4851487	T	C	0.0808	0.0174	0.5755	3.25E-06	0.003	21.56
VEGF	rs61835654	C	T	0.1032	0.0216	0.2535	1.85E-06	0.004	22.83
VEGF	rs620490	G	T	-0.1174	0.019	0.2992	6.46E-10	0.006	38.18
VEGF	rs6715875	C	T	0.2902	0.0599	0.0249	1.25E-06	0.004	23.47
VEGF	rs6741007	G	T	-0.1233	0.0175	0.4970	2.09E-12	0.008	49.64
VEGF	rs6808178	C	T	-0.0864	0.0174	0.6292	7.20E-07	0.003	24.66
VEGF	rs73032517	G	A	0.2706	0.0558	0.9742	1.27E-06	0.004	23.52
VEGF	rs75505347	T	C	0.3917	0.0674	0.9791	6.12E-09	0.006	33.77
VEGF	rs75646569	G	T	0.1916	0.0266	0.1252	5.62E-13	0.008	51.88
VEGF	rs7695720	C	A	-0.1255	0.0208	0.7644	1.53E-09	0.006	36.40
VEGF	rs7818035	A	G	-0.693	0.1429	0.0209	1.24E-06	0.020	23.52
VEGF	rs79436216	G	A	0.2816	0.0603	0.9583	3.02E-06	0.006	21.81
VEGF	rs858295	G	A	-0.1039	0.0176	0.3708	3.83E-09	0.005	34.85
VEGF	rs9840232	T	C	0.1157	0.0233	0.8121	6.56E-07	0.004	24.66

VEGF	rs9845968	G	A	0.0842	0.0175	0.4483	1.42E-06	0.004	23.15

Supplementary Table 6. Detailed information for risk of ALS-associated SNPs with circulating cytokines.

Outcomes	SNP	Effect Allele	Other Allele	Beta	Se	EAF	*P-value*	*R2*	*F*	Power
BNGF	rs10139154	T	C	0.0767	0.0146	0.659	1.43999E-07	0.003	27.60	
BNGF	rs10463311	T	C	-0.0854	0.0156	0.2714	3.99899E-08	0.003	29.97	
BNGF	rs11195948	T	C	-0.0707	0.0144	0.6282	9.13692E-07	0.002	24.11	
BNGF	rs11786739	G	A	0.1371	0.029	0.9364	2.20298E-06	0.002	22.35	
BNGF	rs118049474	C	T	-0.1335	0.0284	0.0577	2.65601E-06	0.002	22.10	
BNGF	rs11842416	C	T	0.0814	0.0172	0.2416	2.35999E-06	0.002	22.40	
BNGF	rs146107389	C	T	0.3509	0.0764	0.0159	4.38995E-06	0.004	21.10	
BNGF	rs2013478	T	C	0.0656	0.0144	0.6590	4.93799E-06	0.002	20.75	
BNGF	rs34182166	G	A	0.1372	0.03	0.9463	4.70002E-06	0.002	20.92	
BNGF	rs34517613	T	C	-0.1057	0.0223	0.8718	2.17601E-06	0.002	22.47	
BNGF	rs3849943	T	C	-0.1764	0.0155	0.7674	3.76964E-30	0.011	129.52	22.40%
BNGF	rs538622	G	A	-0.0693	0.0139	0.3877	6.68806E-07	0.002	24.86	
BNGF	rs61527579	C	T	-0.0906	0.0193	0.1789	2.66698E-06	0.002	22.04	
BNGF	rs61880881	C	A	0.1317	0.0281	0.9423	2.72898E-06	0.002	21.97	
BNGF	rs72714928	C	T	0.0869	0.0183	0.1670	2.11602E-06	0.002	22.55	
BNGF	rs74654358	A	G	0.1976	0.0337	0.0318	4.65801E-09	0.002	34.38	
BNGF	rs75087725	A	C	0.5145	0.0672	0.0119	1.84799E-14	0.006	58.62	
BNGF	rs7552104	C	T	-0.089	0.0182	0.1779	1.06001E-06	0.002	23.91	
BNGF	rs79068040	T	C	0.2058	0.0444	0.9781	3.62502E-06	0.002	21.48	
BNGF	rs8125607	A	G	0.0688	0.0151	0.3221	4.91904E-06	0.002	20.76	
BNGF	rs9901522	T	C	0.1462	0.0273	0.9423	8.58697E-08	0.002	28.68	
CTACK	rs10139154	T	C	0.0767	0.0146	0.659	1.43999E-07	0.003	27.60	
CTACK	rs10463311	T	C	-0.0854	0.0156	0.2714	3.99899E-08	0.003	29.97	29.30%
CTACK	rs11195948	T	C	-0.0707	0.0144	0.6282	9.13692E-07	0.002	24.11	

CTACK	rs11786739	G	A	0.1371	0.029	0.9364	2.20298E-06	0.002	22.35	
CTACK	rs118049474	C	T	-0.1335	0.0284	0.0577	2.65601E-06	0.002	22.10	
CTACK	rs11842416	C	T	0.0814	0.0172	0.2416	2.35999E-06	0.002	22.40	
CTACK	rs146107389	C	T	0.3509	0.0764	0.0159	4.38995E-06	0.004	21.10	
CTACK	rs2013478	T	C	0.0656	0.0144	0.6590	4.93799E-06	0.002	20.75	
CTACK	rs34182166	G	A	0.1372	0.03	0.9463	4.70002E-06	0.002	20.92	
CTACK	rs34517613	T	C	-0.1057	0.0223	0.8718	2.17601E-06	0.002	22.47	
CTACK	rs3849943	T	C	-0.1764	0.0155	0.7674	3.76964E-30	0.011	129.52	
CTACK	rs538622	G	A	-0.0693	0.0139	0.3877	6.68806E-07	0.002	24.86	
CTACK	rs61527579	C	T	-0.0906	0.0193	0.1789	2.66698E-06	0.002	22.04	
CTACK	rs61880881	C	A	0.1317	0.0281	0.9423	2.72898E-06	0.002	21.97	
CTACK	rs72714928	C	T	0.0869	0.0183	0.1670	2.11602E-06	0.002	22.55	
CTACK	rs74654358	A	G	0.1976	0.0337	0.0318	4.65801E-09	0.002	34.38	
CTACK	rs75087725	A	C	0.5145	0.0672	0.0119	1.84799E-14	0.006	58.62	
CTACK	rs7552104	C	T	-0.089	0.0182	0.1779	1.06001E-06	0.002	23.91	
CTACK	rs79068040	T	C	0.2058	0.0444	0.9781	3.62502E-06	0.002	21.48	
CTACK	rs8125607	A	G	0.0688	0.0151	0.3221	4.91904E-06	0.002	20.76	
CTACK	rs9901522	T	C	0.1462	0.0273	0.9423	8.58697E-08	0.002	28.68	
EOTAXIN	rs10139154	T	C	0.0767	0.0146	0.659	1.44E-07	0.003	27.60	
EOTAXIN	rs10463311	T	C	-0.0854	0.0156	0.2714	4.00E-08	0.003	29.97	
EOTAXIN	rs11195948	T	C	-0.0707	0.0144	0.6282	9.14E-07	0.002	24.11	
EOTAXIN	rs11786739	G	A	0.1371	0.029	0.9364	2.20E-06	0.002	22.35	36.90%
EOTAXIN	rs118049474	C	T	-0.1335	0.0284	0.0577	2.66E-06	0.002	22.10	
EOTAXIN	rs11842416	C	T	0.0814	0.0172	0.2416	2.36E-06	0.002	22.40	
EOTAXIN	rs146107389	C	T	0.3509	0.0764	0.0159	4.39E-06	0.004	21.10	
EOTAXIN	rs2013478	T	C	0.0656	0.0144	0.6590	4.94E-06	0.002	20.75	

EOTAXIN	rs34182166	G	A	0.1372	0.03	0.9463	4.70E-06	0.002	20.92	
EOTAXIN	rs34517613	T	C	-0.1057	0.0223	0.8718	2.18E-06	0.002	22.47	
EOTAXIN	rs3849943	T	C	-0.1764	0.0155	0.7674	3.77E-30	0.011	129.52	
EOTAXIN	rs538622	G	A	-0.0693	0.0139	0.3877	6.69E-07	0.002	24.86	
EOTAXIN	rs61527579	C	T	-0.0906	0.0193	0.1789	2.67E-06	0.002	22.04	
EOTAXIN	rs61880881	C	A	0.1317	0.0281	0.9423	2.73E-06	0.002	21.97	
EOTAXIN	rs72714928	C	T	0.0869	0.0183	0.1670	2.12E-06	0.002	22.55	
EOTAXIN	rs74654358	A	G	0.1976	0.0337	0.0318	4.66E-09	0.002	34.38	
EOTAXIN	rs75087725	A	C	0.5145	0.0672	0.0119	1.85E-14	0.006	58.62	
EOTAXIN	rs7552104	C	T	-0.089	0.0182	0.1779	1.06E-06	0.002	23.91	
EOTAXIN	rs79068040	T	C	0.2058	0.0444	0.9781	3.63E-06	0.002	21.48	
EOTAXIN	rs8125607	A	G	0.0688	0.0151	0.3221	4.92E-06	0.002	20.76	
EOTAXIN	rs9901522	T	C	0.1462	0.0273	0.9423	8.59E-08	0.002	28.68	
FGF	rs10139154	T	C	0.0767	0.0146	0.659	1.44E-07	0.003	27.60	
FGF	rs10463311	T	C	-0.0854	0.0156	0.2714	4.00E-08	0.003	29.97	
FGF	rs11195948	T	C	-0.0707	0.0144	0.6282	9.14E-07	0.002	24.11	
FGF	rs11786739	G	A	0.1371	0.029	0.9364	2.20E-06	0.002	22.35	
FGF	rs118049474	C	T	-0.1335	0.0284	0.0577	2.66E-06	0.002	22.10	
FGF	rs11842416	C	T	0.0814	0.0172	0.2416	2.36E-06	0.002	22.40	
FGF	rs146107389	C	T	0.3509	0.0764	0.0159	4.39E-06	0.004	21.10	100.00%
FGF	rs2013478	T	C	0.0656	0.0144	0.6590	4.94E-06	0.002	20.75	
FGF	rs34182166	G	A	0.1372	0.03	0.9463	4.70E-06	0.002	20.92	
FGF	rs34517613	T	C	-0.1057	0.0223	0.8718	2.18E-06	0.002	22.47	
FGF	rs3849943	T	C	-0.1764	0.0155	0.7674	3.77E-30	0.011	129.52	
FGF	rs538622	G	A	-0.0693	0.0139	0.3877	6.69E-07	0.002	24.86	
FGF	rs61527579	C	T	-0.0906	0.0193	0.1789	2.67E-06	0.002	22.04	

FGF	rs61880881	C	A	0.1317	0.0281	0.9423	2.73E-06	0.002	21.97	
FGF	rs72714928	C	T	0.0869	0.0183	0.1670	2.12E-06	0.002	22.55	
FGF	rs74654358	A	G	0.1976	0.0337	0.0318	4.66E-09	0.002	34.38	
FGF	rs75087725	A	C	0.5145	0.0672	0.0119	1.85E-14	0.006	58.62	
FGF	rs7552104	C	T	-0.089	0.0182	0.1779	1.06E-06	0.002	23.91	
FGF	rs79068040	T	C	0.2058	0.0444	0.9781	3.63E-06	0.002	21.48	
FGF	rs8125607	A	G	0.0688	0.0151	0.3221	4.92E-06	0.002	20.76	
FGF	rs9901522	T	C	0.1462	0.0273	0.9423	8.59E-08	0.002	28.68	
G-CSF	rs10139154	T	C	0.0767	0.0146	0.659	1.44E-07	0.003	27.60	
G-CSF	rs10463311	T	C	-0.0854	0.0156	0.2714	4.00E-08	0.003	29.97	
G-CSF	rs11195948	T	C	-0.0707	0.0144	0.6282	9.14E-07	0.002	24.11	
G-CSF	rs11786739	G	A	0.1371	0.029	0.9364	2.20E-06	0.002	22.35	
G-CSF	rs118049474	C	T	-0.1335	0.0284	0.0577	2.66E-06	0.002	22.10	
G-CSF	rs11842416	C	T	0.0814	0.0172	0.2416	2.36E-06	0.002	22.40	
G-CSF	rs146107389	C	T	0.3509	0.0764	0.0159	4.39E-06	0.004	21.10	
G-CSF	rs2013478	T	C	0.0656	0.0144	0.6590	4.94E-06	0.002	20.75	
G-CSF	rs34182166	G	A	0.1372	0.03	0.9463	4.70E-06	0.002	20.92	
G-CSF	rs34517613	T	C	-0.1057	0.0223	0.8718	2.18E-06	0.002	22.47	9.30%
G-CSF	rs3849943	T	C	-0.1764	0.0155	0.7674	3.77E-30	0.011	129.52	
G-CSF	rs538622	G	A	-0.0693	0.0139	0.3877	6.69E-07	0.002	24.86	
G-CSF	rs61527579	C	T	-0.0906	0.0193	0.1789	2.67E-06	0.002	22.04	
G-CSF	rs61880881	C	A	0.1317	0.0281	0.9423	2.73E-06	0.002	21.97	
G-CSF	rs72714928	C	T	0.0869	0.0183	0.1670	2.12E-06	0.002	22.55	
G-CSF	rs74654358	A	G	0.1976	0.0337	0.0318	4.66E-09	0.002	34.38	
G-CSF	rs75087725	A	C	0.5145	0.0672	0.0119	1.85E-14	0.006	58.62	
G-CSF	rs7552104	C	T	-0.089	0.0182	0.1779	1.06E-06	0.002	23.91	

G-CSF	rs79068040	T	C	0.2058	0.0444	0.9781	3.63E-06	0.002	21.48	
G-CSF	rs8125607	A	G	0.0688	0.0151	0.3221	4.92E-06	0.002	20.76	
G-CSF	rs9901522	T	C	0.1462	0.0273	0.9423	8.59E-08	0.002	28.68	
GROA	rs10139154	T	C	0.0767	0.0146	0.659	1.44E-07	0.003	27.60	
GROA	rs10463311	T	C	-0.0854	0.0156	0.2714	4.00E-08	0.003	29.97	
GROA	rs11195948	T	C	-0.0707	0.0144	0.6282	9.14E-07	0.002	24.11	
GROA	rs11786739	G	A	0.1371	0.029	0.9364	2.20E-06	0.002	22.35	
GROA	rs118049474	C	T	-0.1335	0.0284	0.0577	2.66E-06	0.002	22.10	
GROA	rs11842416	C	T	0.0814	0.0172	0.2416	2.36E-06	0.002	22.40	
GROA	rs146107389	C	T	0.3509	0.0764	0.0159	4.39E-06	0.004	21.10	
GROA	rs2013478	T	C	0.0656	0.0144	0.6590	4.94E-06	0.002	20.75	
GROA	rs34182166	G	A	0.1372	0.03	0.9463	4.70E-06	0.002	20.92	
GROA	rs34517613	T	C	-0.1057	0.0223	0.8718	2.18E-06	0.002	22.47	
GROA	rs3849943	T	C	-0.1764	0.0155	0.7674	3.77E-30	0.011	129.52	72.10%
GROA	rs538622	G	A	-0.0693	0.0139	0.3877	6.69E-07	0.002	24.86	
GROA	rs61527579	C	T	-0.0906	0.0193	0.1789	2.67E-06	0.002	22.04	
GROA	rs61880881	C	A	0.1317	0.0281	0.9423	2.73E-06	0.002	21.97	
GROA	rs72714928	C	T	0.0869	0.0183	0.1670	2.12E-06	0.002	22.55	
GROA	rs74654358	A	G	0.1976	0.0337	0.0318	4.66E-09	0.002	34.38	
GROA	rs75087725	A	C	0.5145	0.0672	0.0119	1.85E-14	0.006	58.62	
GROA	rs7552104	C	T	-0.089	0.0182	0.1779	1.06E-06	0.002	23.91	
GROA	rs79068040	T	C	0.2058	0.0444	0.9781	3.63E-06	0.002	21.48	
GROA	rs8125607	A	G	0.0688	0.0151	0.3221	4.92E-06	0.002	20.76	
GROA	rs9901522	T	C	0.1462	0.0273	0.9423	8.59E-08	0.002	28.68	
HGF	rs10139154	T	C	0.0767	0.0146	0.659	1.44E-07	0.003	27.60	84.40%
HGF	rs10463311	T	C	-0.0854	0.0156	0.2714	4.00E-08	0.003	29.97	

HGF	rs11195948	T	C	-0.0707	0.0144	0.6282	9.14E-07	0.002	24.11	
HGF	rs11786739	G	A	0.1371	0.029	0.9364	2.20E-06	0.002	22.35	
HGF	rs118049474	C	T	-0.1335	0.0284	0.0577	2.66E-06	0.002	22.10	
HGF	rs11842416	C	T	0.0814	0.0172	0.2416	2.36E-06	0.002	22.40	
HGF	rs146107389	C	T	0.3509	0.0764	0.0159	4.39E-06	0.004	21.10	
HGF	rs2013478	T	C	0.0656	0.0144	0.6590	4.94E-06	0.002	20.75	
HGF	rs34182166	G	A	0.1372	0.03	0.9463	4.70E-06	0.002	20.92	
HGF	rs34517613	T	C	-0.1057	0.0223	0.8718	2.18E-06	0.002	22.47	
HGF	rs3849943	T	C	-0.1764	0.0155	0.7674	3.77E-30	0.011	129.52	
HGF	rs538622	G	A	-0.0693	0.0139	0.3877	6.69E-07	0.002	24.86	
HGF	rs61527579	C	T	-0.0906	0.0193	0.1789	2.67E-06	0.002	22.04	
HGF	rs61880881	C	A	0.1317	0.0281	0.9423	2.73E-06	0.002	21.97	
HGF	rs72714928	C	T	0.0869	0.0183	0.1670	2.12E-06	0.002	22.55	
HGF	rs74654358	A	G	0.1976	0.0337	0.0318	4.66E-09	0.002	34.38	
HGF	rs75087725	A	C	0.5145	0.0672	0.0119	1.85E-14	0.006	58.62	
HGF	rs7552104	C	T	-0.089	0.0182	0.1779	1.06E-06	0.002	23.91	
HGF	rs79068040	T	C	0.2058	0.0444	0.9781	3.63E-06	0.002	21.48	
HGF	rs8125607	A	G	0.0688	0.0151	0.3221	4.92E-06	0.002	20.76	
HGF	rs9901522	T	C	0.1462	0.0273	0.9423	8.59E-08	0.002	28.68	
IFN-G	rs12973192	T	C	0.0767	0.0146	0.6581	1.44E-07	0.003	27.60	
IFN-G	rs142321490	T	C	-0.0854	0.0156	0.0169	4.00E-08	0.000	29.97	
IFN-G	rs146107389	T	C	-0.0707	0.0144	0.0159	9.14E-07	0.000	24.11	
IFN-G	rs17070492	G	A	0.1371	0.029	0.9046	2.20E-06	0.003	22.35	97.10%
IFN-G	rs17148125	C	T	-0.1335	0.0284	0.1412	2.66E-06	0.004	22.10	
IFN-G	rs2013478	C	T	0.0814	0.0172	0.6590	2.36E-06	0.003	22.40	
IFN-G	rs2285642	C	T	0.3509	0.0764	0.4483	4.39E-06	0.061	21.10	

IFN-G	rs34182166	T	C	0.0656	0.0144	0.9463	4.94E-06	0.000	20.75		
IFN-G	rs34517613	G	A	0.1372	0.03	0.8718	4.70E-06	0.004	20.92		
IFN-G	rs3849943	T	C	-0.1057	0.0223	0.7674	2.18E-06	0.004	22.47		
IFN-G	rs538622	T	C	-0.1764	0.0155	0.3877	3.77E-30	0.015	129.52		
IFN-G	rs61527579	G	A	-0.0693	0.0139	0.1789	6.69E-07	0.001	24.86		
IFN-G	rs61880881	C	T	-0.0906	0.0193	0.9423	2.67E-06	0.001	22.04		
IFN-G	rs67710834	C	A	0.1317	0.0281	0.3907	2.73E-06	0.008	21.97		
IFN-G	rs72714928	C	T	0.0869	0.0183	0.1670	2.12E-06	0.002	22.55		
IFN-G	rs74654358	A	G	0.1976	0.0337	0.0318	4.66E-09	0.002	34.38		
IFN-G	rs75087725	A	C	0.5145	0.0672	0.0119	1.85E-14	0.006	58.62		
IFN-G	rs7552104	C	T	-0.089	0.0182	0.1779	1.06E-06	0.002	23.91		
IFN-G	rs79068040	T	C	0.2058	0.0444	0.9781	3.63E-06	0.002	21.48		
IFN-G	rs8125607	A	G	0.0688	0.0151	0.3221	4.92E-06	0.002	20.76		
IFN-G	rs9901522	T	C	0.1462	0.0273	0.9423	8.59E-08	0.002	28.68		
IL-10	rs10139154	T	C	0.0767	0.0146	0.659	1.44E-07	0.003	27.60		
IL-10	rs10463311	T	C	-0.0854	0.0156	0.2714	4.00E-08	0.003	29.97		
IL-10	rs11195948	T	C	-0.0707	0.0144	0.6282	9.14E-07	0.002	24.11		
IL-10	rs11786739	G	A	0.1371	0.029	0.9364	2.20E-06	0.002	22.35		
IL-10	rs118049474	C	T	-0.1335	0.0284	0.0577	2.66E-06	0.002	22.10		
IL-10	rs11842416	C	T	0.0814	0.0172	0.2416	2.36E-06	0.002	22.40	61.90%	
IL-10	rs146107389	C	T	0.3509	0.0764	0.0159	4.39E-06	0.004	21.10		
IL-10	rs2013478	T	C	0.0656	0.0144	0.6590	4.94E-06	0.002	20.75		
IL-10	rs34182166	G	A	0.1372	0.03	0.9463	4.70E-06	0.002	20.92		
IL-10	rs34517613	T	C	-0.1057	0.0223	0.8718	2.18E-06	0.002	22.47		
IL-10	rs3849943	T	C	-0.1764	0.0155	0.7674	3.77E-30	0.011	129.52		
IL-10	rs538622	G	A	-0.0693	0.0139	0.3877	6.69E-07	0.002	24.86		

IL-10	rs61527579	C	T	-0.0906	0.0193	0.1789	2.67E-06	0.002	22.04	
IL-10	rs61880881	C	A	0.1317	0.0281	0.9423	2.73E-06	0.002	21.97	
IL-10	rs72714928	C	T	0.0869	0.0183	0.1670	2.12E-06	0.002	22.55	
IL-10	rs74654358	A	G	0.1976	0.0337	0.0318	4.66E-09	0.002	34.38	
IL-10	rs75087725	A	C	0.5145	0.0672	0.0119	1.85E-14	0.006	58.62	
IL-10	rs7552104	C	T	-0.089	0.0182	0.1779	1.06E-06	0.002	23.91	
IL-10	rs79068040	T	C	0.2058	0.0444	0.9781	3.63E-06	0.002	21.48	
IL-10	rs8125607	A	G	0.0688	0.0151	0.3221	4.92E-06	0.002	20.76	
IL-10	rs9901522	T	C	0.1462	0.0273	0.9423	8.59E-08	0.002	28.68	
IL-12	rs10139154	T	C	0.0767	0.0146	0.659	1.44E-07	0.003	27.60	
IL-12	rs10463311	T	C	-0.0854	0.0156	0.2714	4.00E-08	0.003	29.97	
IL-12	rs11195948	T	C	-0.0707	0.0144	0.6282	9.14E-07	0.002	24.11	
IL-12	rs11786739	G	A	0.1371	0.029	0.9364	2.20E-06	0.002	22.35	
IL-12	rs118049474	C	T	-0.1335	0.0284	0.0577	2.66E-06	0.002	22.10	
IL-12	rs11842416	C	T	0.0814	0.0172	0.2416	2.36E-06	0.002	22.40	
IL-12	rs146107389	C	T	0.3509	0.0764	0.0159	4.39E-06	0.004	21.10	
IL-12	rs2013478	T	C	0.0656	0.0144	0.6590	4.94E-06	0.002	20.75	
IL-12	rs34182166	G	A	0.1372	0.03	0.9463	4.70E-06	0.002	20.92	72.10%
IL-12	rs34517613	T	C	-0.1057	0.0223	0.8718	2.18E-06	0.002	22.47	
IL-12	rs3849943	T	C	-0.1764	0.0155	0.7674	3.77E-30	0.011	129.52	
IL-12	rs538622	G	A	-0.0693	0.0139	0.3877	6.69E-07	0.002	24.86	
IL-12	rs61527579	C	T	-0.0906	0.0193	0.1789	2.67E-06	0.002	22.04	
IL-12	rs61880881	C	A	0.1317	0.0281	0.9423	2.73E-06	0.002	21.97	
IL-12	rs72714928	C	T	0.0869	0.0183	0.1670	2.12E-06	0.002	22.55	
IL-12	rs74654358	A	G	0.1976	0.0337	0.0318	4.66E-09	0.002	34.38	
IL-12	rs75087725	A	C	0.5145	0.0672	0.0119	1.85E-14	0.006	58.62	

IL-12	rs7552104	C	T	-0.089	0.0182	0.1779	1.06E-06	0.002	23.91	
IL-12	rs79068040	T	C	0.2058	0.0444	0.9781	3.63E-06	0.002	21.48	
IL-12	rs8125607	A	G	0.0688	0.0151	0.3221	4.92E-06	0.002	20.76	
IL-12	rs9901522	T	C	0.1462	0.0273	0.9423	8.59E-08	0.002	28.68	
IL-13	rs10139154	T	C	0.0767	0.0146	0.659	1.44E-07	0.003	27.60	
IL-13	rs10463311	T	C	-0.0854	0.0156	0.2714	4.00E-08	0.003	29.97	
IL-13	rs11195948	T	C	-0.0707	0.0144	0.6282	9.14E-07	0.002	24.11	
IL-13	rs11786739	G	A	0.1371	0.029	0.9364	2.20E-06	0.002	22.35	
IL-13	rs118049474	C	T	-0.1335	0.0284	0.0577	2.66E-06	0.002	22.10	
IL-13	rs11842416	C	T	0.0814	0.0172	0.2416	2.36E-06	0.002	22.40	
IL-13	rs146107389	C	T	0.3509	0.0764	0.0159	4.39E-06	0.004	21.10	
IL-13	rs2013478	T	C	0.0656	0.0144	0.6590	4.94E-06	0.002	20.75	
IL-13	rs34182166	G	A	0.1372	0.03	0.9463	4.70E-06	0.002	20.92	
IL-13	rs34517613	T	C	-0.1057	0.0223	0.8718	2.18E-06	0.002	22.47	
IL-13	rs3849943	T	C	-0.1764	0.0155	0.7674	3.77E-30	0.011	129.52	13.30%
IL-13	rs538622	G	A	-0.0693	0.0139	0.3877	6.69E-07	0.002	24.86	
IL-13	rs61527579	C	T	-0.0906	0.0193	0.1789	2.67E-06	0.002	22.04	
IL-13	rs61880881	C	A	0.1317	0.0281	0.9423	2.73E-06	0.002	21.97	
IL-13	rs72714928	C	T	0.0869	0.0183	0.1670	2.12E-06	0.002	22.55	
IL-13	rs74654358	A	G	0.1976	0.0337	0.0318	4.66E-09	0.002	34.38	
IL-13	rs75087725	A	C	0.5145	0.0672	0.0119	1.85E-14	0.006	58.62	
IL-13	rs7552104	C	T	-0.089	0.0182	0.1779	1.06E-06	0.002	23.91	
IL-13	rs79068040	T	C	0.2058	0.0444	0.9781	3.63E-06	0.002	21.48	
IL-13	rs8125607	A	G	0.0688	0.0151	0.3221	4.92E-06	0.002	20.76	
IL-13	rs9901522	T	C	0.1462	0.0273	0.9423	8.59E-08	0.002	28.68	
IL-16	rs10139154	T	C	0.0767	0.0146	0.659	1.44E-07	0.003	27.60	97.30%

IL-16	rs10463311	T	C	-0.0854	0.0156	0.2714	4.00E-08	0.003	29.97	
IL-16	rs11195948	T	C	-0.0707	0.0144	0.6282	9.14E-07	0.002	24.11	
IL-16	rs11786739	G	A	0.1371	0.029	0.9364	2.20E-06	0.002	22.35	
IL-16	rs118049474	C	T	-0.1335	0.0284	0.0577	2.66E-06	0.002	22.10	
IL-16	rs11842416	C	T	0.0814	0.0172	0.2416	2.36E-06	0.002	22.40	
IL-16	rs146107389	C	T	0.3509	0.0764	0.0159	4.39E-06	0.004	21.10	
IL-16	rs2013478	T	C	0.0656	0.0144	0.6590	4.94E-06	0.002	20.75	
IL-16	rs34182166	G	A	0.1372	0.03	0.9463	4.70E-06	0.002	20.92	
IL-16	rs34517613	T	C	-0.1057	0.0223	0.8718	2.18E-06	0.002	22.47	
IL-16	rs3849943	T	C	-0.1764	0.0155	0.7674	3.77E-30	0.011	129.52	
IL-16	rs538622	G	A	-0.0693	0.0139	0.3877	6.69E-07	0.002	24.86	
IL-16	rs61527579	C	T	-0.0906	0.0193	0.1789	2.67E-06	0.002	22.04	
IL-16	rs61880881	C	A	0.1317	0.0281	0.9423	2.73E-06	0.002	21.97	
IL-16	rs72714928	C	T	0.0869	0.0183	0.1670	2.12E-06	0.002	22.55	
IL-16	rs74654358	A	G	0.1976	0.0337	0.0318	4.66E-09	0.002	34.38	
IL-16	rs75087725	A	C	0.5145	0.0672	0.0119	1.85E-14	0.006	58.62	
IL-16	rs7552104	C	T	-0.089	0.0182	0.1779	1.06E-06	0.002	23.91	
IL-16	rs79068040	T	C	0.2058	0.0444	0.9781	3.63E-06	0.002	21.48	
IL-16	rs8125607	A	G	0.0688	0.0151	0.3221	4.92E-06	0.002	20.76	
IL-16	rs9901522	T	C	0.1462	0.0273	0.9423	8.59E-08	0.002	28.68	
IL-17	rs10139154	T	C	0.0767	0.0146	0.659	1.44E-07	0.003	27.60	
IL-17	rs10463311	T	C	-0.0854	0.0156	0.2714	4.00E-08	0.003	29.97	
IL-17	rs11195948	T	C	-0.0707	0.0144	0.6282	9.14E-07	0.002	24.11	
IL-17	rs11786739	G	A	0.1371	0.029	0.9364	2.20E-06	0.002	22.35	100.00%
IL-17	rs118049474	C	T	-0.1335	0.0284	0.0577	2.66E-06	0.002	22.10	
IL-17	rs11842416	C	T	0.0814	0.0172	0.2416	2.36E-06	0.002	22.40	

IL-17	rs146107389	C	T	0.3509	0.0764	0.0159	4.39E-06	0.004	21.10	
IL-17	rs2013478	T	C	0.0656	0.0144	0.6590	4.94E-06	0.002	20.75	
IL-17	rs34182166	G	A	0.1372	0.03	0.9463	4.70E-06	0.002	20.92	
IL-17	rs34517613	T	C	-0.1057	0.0223	0.8718	2.18E-06	0.002	22.47	
IL-17	rs3849943	T	C	-0.1764	0.0155	0.7674	3.77E-30	0.011	129.52	
IL-17	rs538622	G	A	-0.0693	0.0139	0.3877	6.69E-07	0.002	24.86	
IL-17	rs61527579	C	T	-0.0906	0.0193	0.1789	2.67E-06	0.002	22.04	
IL-17	rs61880881	C	A	0.1317	0.0281	0.9423	2.73E-06	0.002	21.97	
IL-17	rs72714928	C	T	0.0869	0.0183	0.1670	2.12E-06	0.002	22.55	
IL-17	rs74654358	A	G	0.1976	0.0337	0.0318	4.66E-09	0.002	34.38	
IL-17	rs75087725	A	C	0.5145	0.0672	0.0119	1.85E-14	0.006	58.62	
IL-17	rs7552104	C	T	-0.089	0.0182	0.1779	1.06E-06	0.002	23.91	
IL-17	rs79068040	T	C	0.2058	0.0444	0.9781	3.63E-06	0.002	21.48	
IL-17	rs8125607	A	G	0.0688	0.0151	0.3221	4.92E-06	0.002	20.76	
IL-17	rs9901522	T	C	0.1462	0.0273	0.9423	8.59E-08	0.002	28.68	
IL-18	rs10139154	T	C	0.0767	0.0146	0.659	1.44E-07	0.003	27.60	
IL-18	rs10463311	T	C	-0.0854	0.0156	0.2714	4.00E-08	0.003	29.97	
IL-18	rs11195948	T	C	-0.0707	0.0144	0.6282	9.14E-07	0.002	24.11	
IL-18	rs11786739	G	A	0.1371	0.029	0.9364	2.20E-06	0.002	22.35	
IL-18	rs118049474	C	T	-0.1335	0.0284	0.0577	2.66E-06	0.002	22.10	
IL-18	rs11842416	C	T	0.0814	0.0172	0.2416	2.36E-06	0.002	22.40	100.00%
IL-18	rs146107389	C	T	0.3509	0.0764	0.0159	4.39E-06	0.004	21.10	
IL-18	rs2013478	T	C	0.0656	0.0144	0.6590	4.94E-06	0.002	20.75	
IL-18	rs34182166	G	A	0.1372	0.03	0.9463	4.70E-06	0.002	20.92	
IL-18	rs34517613	T	C	-0.1057	0.0223	0.8718	2.18E-06	0.002	22.47	
IL-18	rs3849943	T	C	-0.1764	0.0155	0.7674	3.77E-30	0.011	129.52	

IL-18	rs538622	G	A	-0.0693	0.0139	0.3877	6.69E-07	0.002	24.86	
IL-18	rs61527579	C	T	-0.0906	0.0193	0.1789	2.67E-06	0.002	22.04	
IL-18	rs61880881	C	A	0.1317	0.0281	0.9423	2.73E-06	0.002	21.97	
IL-18	rs72714928	C	T	0.0869	0.0183	0.1670	2.12E-06	0.002	22.55	
IL-18	rs74654358	A	G	0.1976	0.0337	0.0318	4.66E-09	0.002	34.38	
IL-18	rs75087725	A	C	0.5145	0.0672	0.0119	1.85E-14	0.006	58.62	
IL-18	rs7552104	C	T	-0.089	0.0182	0.1779	1.06E-06	0.002	23.91	
IL-18	rs79068040	T	C	0.2058	0.0444	0.9781	3.63E-06	0.002	21.48	
IL-18	rs8125607	A	G	0.0688	0.0151	0.3221	4.92E-06	0.002	20.76	
IL-18	rs9901522	T	C	0.1462	0.0273	0.9423	8.59E-08	0.002	28.68	
IL-1B	rs10139154	T	C	0.0767	0.0146	0.659	1.44E-07	0.003	27.60	
IL-1B	rs10143310	T	C	-0.0854	0.0156	0.2237	4.00E-08	0.003	29.97	
IL-1B	rs10463311	T	C	-0.0707	0.0144	0.2714	9.14E-07	0.002	24.11	
IL-1B	rs11195948	G	A	0.1371	0.029	0.6282	2.20E-06	0.009	22.35	
IL-1B	rs11786739	C	T	-0.1335	0.0284	0.9364	2.66E-06	0.002	22.10	
IL-1B	rs118049474	C	T	0.0814	0.0172	0.0577	2.36E-06	0.001	22.40	
IL-1B	rs11842416	C	T	0.3509	0.0764	0.2416	4.39E-06	0.045	21.10	
IL-1B	rs12973192	T	C	0.0656	0.0144	0.6581	4.94E-06	0.002	20.75	100.00%
IL-1B	rs142321490	G	A	0.1372	0.03	0.0169	4.70E-06	0.001	20.92	
IL-1B	rs146107389	T	C	-0.1057	0.0223	0.0159	2.18E-06	0.000	22.47	
IL-1B	rs17070492	T	C	-0.1764	0.0155	0.9046	3.77E-30	0.005	129.52	
IL-1B	rs17148125	G	A	-0.0693	0.0139	0.1412	6.69E-07	0.001	24.86	
IL-1B	rs2013478	C	T	-0.0906	0.0193	0.6590	2.67E-06	0.004	22.04	
IL-1B	rs2285642	C	A	0.1317	0.0281	0.4483	2.73E-06	0.009	21.97	
IL-1B	rs34182166	C	T	0.0869	0.0183	0.9463	2.12E-06	0.001	22.55	
IL-1B	rs34517613	A	G	0.1976	0.0337	0.8718	4.66E-09	0.009	34.38	

IL-1B	rs3849943	A	C	0.5145	0.0672	0.7674	1.85E-14	0.095	58.62	
IL-1B	rs538622	C	T	-0.089	0.0182	0.3877	1.06E-06	0.004	23.91	
IL-1B	rs61527579	T	C	0.2058	0.0444	0.1789	3.63E-06	0.012	21.48	
IL-1B	rs61880881	A	G	0.0688	0.0151	0.9423	4.92E-06	0.001	20.76	
IL-1B	rs67710834	T	C	0.1462	0.0273	0.3907	8.59E-08	0.010	28.68	
IL-1RA	rs10139154	T	C	0.0767	0.0146	0.659	1.44E-07	0.003	27.60	
IL-1RA	rs10463311	T	C	-0.0854	0.0156	0.2714	4.00E-08	0.003	29.97	
IL-1RA	rs11195948	T	C	-0.0707	0.0144	0.6282	9.14E-07	0.002	24.11	
IL-1RA	rs11786739	G	A	0.1371	0.029	0.9364	2.20E-06	0.002	22.35	
IL-1RA	rs118049474	C	T	-0.1335	0.0284	0.0577	2.66E-06	0.002	22.10	
IL-1RA	rs11842416	C	T	0.0814	0.0172	0.2416	2.36E-06	0.002	22.40	
IL-1RA	rs146107389	C	T	0.3509	0.0764	0.0159	4.39E-06	0.004	21.10	
IL-1RA	rs2013478	T	C	0.0656	0.0144	0.6590	4.94E-06	0.002	20.75	
IL-1RA	rs34182166	G	A	0.1372	0.03	0.9463	4.70E-06	0.002	20.92	
IL-1RA	rs34517613	T	C	-0.1057	0.0223	0.8718	2.18E-06	0.002	22.47	
IL-1RA	rs3849943	T	C	-0.1764	0.0155	0.7674	3.77E-30	0.011	129.52	99.70%
IL-1RA	rs538622	G	A	-0.0693	0.0139	0.3877	6.69E-07	0.002	24.86	
IL-1RA	rs61527579	C	T	-0.0906	0.0193	0.1789	2.67E-06	0.002	22.04	
IL-1RA	rs61880881	C	A	0.1317	0.0281	0.9423	2.73E-06	0.002	21.97	
IL-1RA	rs72714928	C	T	0.0869	0.0183	0.1670	2.12E-06	0.002	22.55	
IL-1RA	rs74654358	A	G	0.1976	0.0337	0.0318	4.66E-09	0.002	34.38	
IL-1RA	rs75087725	A	C	0.5145	0.0672	0.0119	1.85E-14	0.006	58.62	
IL-1RA	rs7552104	C	T	-0.089	0.0182	0.1779	1.06E-06	0.002	23.91	
IL-1RA	rs79068040	T	C	0.2058	0.0444	0.9781	3.63E-06	0.002	21.48	
IL-1RA	rs8125607	A	G	0.0688	0.0151	0.3221	4.92E-06	0.002	20.76	
IL-1RA	rs9901522	T	C	0.1462	0.0273	0.9423	8.59E-08	0.002	28.68	

IL-2	rs10139154	T	C	0.0767	0.0146	0.659	1.44E-07	0.003	27.60	
IL-2	rs10463311	T	C	-0.0854	0.0156	0.2714	4.00E-08	0.003	29.97	
IL-2	rs11195948	T	C	-0.0707	0.0144	0.6282	9.14E-07	0.002	24.11	
IL-2	rs11786739	G	A	0.1371	0.029	0.9364	2.20E-06	0.002	22.35	
IL-2	rs118049474	C	T	-0.1335	0.0284	0.0577	2.66E-06	0.002	22.10	
IL-2	rs11842416	C	T	0.0814	0.0172	0.2416	2.36E-06	0.002	22.40	
IL-2	rs146107389	C	T	0.3509	0.0764	0.0159	4.39E-06	0.004	21.10	
IL-2	rs2013478	T	C	0.0656	0.0144	0.6590	4.94E-06	0.002	20.75	
IL-2	rs34182166	G	A	0.1372	0.03	0.9463	4.70E-06	0.002	20.92	
IL-2	rs34517613	T	C	-0.1057	0.0223	0.8718	2.18E-06	0.002	22.47	
IL-2	rs3849943	T	C	-0.1764	0.0155	0.7674	3.77E-30	0.011	129.52	100.00%
IL-2	rs538622	G	A	-0.0693	0.0139	0.3877	6.69E-07	0.002	24.86	
IL-2	rs61527579	C	T	-0.0906	0.0193	0.1789	2.67E-06	0.002	22.04	
IL-2	rs61880881	C	A	0.1317	0.0281	0.9423	2.73E-06	0.002	21.97	
IL-2	rs72714928	C	T	0.0869	0.0183	0.1670	2.12E-06	0.002	22.55	
IL-2	rs74654358	A	G	0.1976	0.0337	0.0318	4.66E-09	0.002	34.38	
IL-2	rs75087725	A	C	0.5145	0.0672	0.0119	1.85E-14	0.006	58.62	
IL-2	rs7552104	C	T	-0.089	0.0182	0.1779	1.06E-06	0.002	23.91	
IL-2	rs79068040	T	C	0.2058	0.0444	0.9781	3.63E-06	0.002	21.48	
IL-2	rs8125607	A	G	0.0688	0.0151	0.3221	4.92E-06	0.002	20.76	
IL-2	rs9901522	T	C	0.1462	0.0273	0.9423	8.59E-08	0.002	28.68	
IL-2RA	rs10139154	T	C	0.0767	0.0146	0.659	1.44E-07	0.003	27.60	
IL-2RA	rs10463311	T	C	-0.0854	0.0156	0.2714	4.00E-08	0.003	29.97	
IL-2RA	rs11195948	T	C	-0.0707	0.0144	0.6282	9.14E-07	0.002	24.11	100.00%
IL-2RA	rs11786739	G	A	0.1371	0.029	0.9364	2.20E-06	0.002	22.35	
IL-2RA	rs118049474	C	T	-0.1335	0.0284	0.0577	2.66E-06	0.002	22.10	

IL-2RA	rs11842416	C	T	0.0814	0.0172	0.2416	2.36E-06	0.002	22.40		
IL-2RA	rs146107389	C	T	0.3509	0.0764	0.0159	4.39E-06	0.004	21.10		
IL-2RA	rs2013478	T	C	0.0656	0.0144	0.6590	4.94E-06	0.002	20.75		
IL-2RA	rs34182166	G	A	0.1372	0.03	0.9463	4.70E-06	0.002	20.92		
IL-2RA	rs34517613	T	C	-0.1057	0.0223	0.8718	2.18E-06	0.002	22.47		
IL-2RA	rs3849943	T	C	-0.1764	0.0155	0.7674	3.77E-30	0.011	129.52		
IL-2RA	rs538622	G	A	-0.0693	0.0139	0.3877	6.69E-07	0.002	24.86		
IL-2RA	rs61527579	C	T	-0.0906	0.0193	0.1789	2.67E-06	0.002	22.04		
IL-2RA	rs61880881	C	A	0.1317	0.0281	0.9423	2.73E-06	0.002	21.97		
IL-2RA	rs72714928	C	T	0.0869	0.0183	0.1670	2.12E-06	0.002	22.55		
IL-2RA	rs74654358	A	G	0.1976	0.0337	0.0318	4.66E-09	0.002	34.38		
IL-2RA	rs75087725	A	C	0.5145	0.0672	0.0119	1.85E-14	0.006	58.62		
IL-2RA	rs7552104	C	T	-0.089	0.0182	0.1779	1.06E-06	0.002	23.91		
IL-2RA	rs79068040	T	C	0.2058	0.0444	0.9781	3.63E-06	0.002	21.48		
IL-2RA	rs8125607	A	G	0.0688	0.0151	0.3221	4.92E-06	0.002	20.76		
IL-2RA	rs9901522	T	C	0.1462	0.0273	0.9423	8.59E-08	0.002	28.68		
IL-4	rs10139154	T	C	0.0767	0.0146	0.659	1.44E-07	0.003	27.60		
IL-4	rs10463311	T	C	-0.0854	0.0156	0.2714	4.00E-08	0.003	29.97		
IL-4	rs11195948	T	C	-0.0707	0.0144	0.6282	9.14E-07	0.002	24.11		
IL-4	rs11786739	G	A	0.1371	0.029	0.9364	2.20E-06	0.002	22.35		
IL-4	rs118049474	C	T	-0.1335	0.0284	0.0577	2.66E-06	0.002	22.10	42.40%
IL-4	rs11842416	C	T	0.0814	0.0172	0.2416	2.36E-06	0.002	22.40	
IL-4	rs146107389	C	T	0.3509	0.0764	0.0159	4.39E-06	0.004	21.10	
IL-4	rs2013478	T	C	0.0656	0.0144	0.6590	4.94E-06	0.002	20.75	
IL-4	rs34182166	G	A	0.1372	0.03	0.9463	4.70E-06	0.002	20.92	
IL-4	rs34517613	T	C	-0.1057	0.0223	0.8718	2.18E-06	0.002	22.47	

IL-4	rs3849943	T	C	-0.1764	0.0155	0.7674	3.77E-30	0.011	129.52	
IL-4	rs538622	G	A	-0.0693	0.0139	0.3877	6.69E-07	0.002	24.86	
IL-4	rs61527579	C	T	-0.0906	0.0193	0.1789	2.67E-06	0.002	22.04	
IL-4	rs61880881	C	A	0.1317	0.0281	0.9423	2.73E-06	0.002	21.97	
IL-4	rs72714928	C	T	0.0869	0.0183	0.1670	2.12E-06	0.002	22.55	
IL-4	rs74654358	A	G	0.1976	0.0337	0.0318	4.66E-09	0.002	34.38	
IL-4	rs75087725	A	C	0.5145	0.0672	0.0119	1.85E-14	0.006	58.62	
IL-4	rs7552104	C	T	-0.089	0.0182	0.1779	1.06E-06	0.002	23.91	
IL-4	rs79068040	T	C	0.2058	0.0444	0.9781	3.63E-06	0.002	21.48	
IL-4	rs8125607	A	G	0.0688	0.0151	0.3221	4.92E-06	0.002	20.76	
IL-4	rs9901522	T	C	0.1462	0.0273	0.9423	8.59E-08	0.002	28.68	
IL-5	rs10139154	T	C	0.0767	0.0146	0.659	1.44E-07	0.003	27.60	
IL-5	rs10463311	T	C	-0.0854	0.0156	0.2714	4.00E-08	0.003	29.97	
IL-5	rs11195948	T	C	-0.0707	0.0144	0.6282	9.14E-07	0.002	24.11	
IL-5	rs11786739	G	A	0.1371	0.029	0.9364	2.20E-06	0.002	22.35	
IL-5	rs118049474	C	T	-0.1335	0.0284	0.0577	2.66E-06	0.002	22.10	
IL-5	rs11842416	C	T	0.0814	0.0172	0.2416	2.36E-06	0.002	22.40	
IL-5	rs146107389	C	T	0.3509	0.0764	0.0159	4.39E-06	0.004	21.10	
IL-5	rs2013478	T	C	0.0656	0.0144	0.6590	4.94E-06	0.002	20.75	99.90%
IL-5	rs34182166	G	A	0.1372	0.03	0.9463	4.70E-06	0.002	20.92	
IL-5	rs34517613	T	C	-0.1057	0.0223	0.8718	2.18E-06	0.002	22.47	
IL-5	rs3849943	T	C	-0.1764	0.0155	0.7674	3.77E-30	0.011	129.52	
IL-5	rs538622	G	A	-0.0693	0.0139	0.3877	6.69E-07	0.002	24.86	
IL-5	rs61527579	C	T	-0.0906	0.0193	0.1789	2.67E-06	0.002	22.04	
IL-5	rs61880881	C	A	0.1317	0.0281	0.9423	2.73E-06	0.002	21.97	
IL-5	rs72714928	C	T	0.0869	0.0183	0.1670	2.12E-06	0.002	22.55	

IL-5	rs74654358	A	G	0.1976	0.0337	0.0318	4.66E-09	0.002	34.38	
IL-5	rs75087725	A	C	0.5145	0.0672	0.0119	1.85E-14	0.006	58.62	
IL-5	rs7552104	C	T	-0.089	0.0182	0.1779	1.06E-06	0.002	23.91	
IL-5	rs79068040	T	C	0.2058	0.0444	0.9781	3.63E-06	0.002	21.48	
IL-5	rs8125607	A	G	0.0688	0.0151	0.3221	4.92E-06	0.002	20.76	
IL-5	rs9901522	T	C	0.1462	0.0273	0.9423	8.59E-08	0.002	28.68	
IL-6	rs10139154	T	C	0.0767	0.0146	0.659	1.44E-07	0.003	27.60	
IL-6	rs10143310	T	C	-0.0854	0.0156	0.2237	4.00E-08	0.003	29.97	
IL-6	rs10463311	T	C	-0.0707	0.0144	0.2714	9.14E-07	0.002	24.11	
IL-6	rs11195948	G	A	0.1371	0.029	0.6282	2.20E-06	0.009	22.35	
IL-6	rs11786739	C	T	-0.1335	0.0284	0.9364	2.66E-06	0.002	22.10	
IL-6	rs118049474	C	T	0.0814	0.0172	0.0577	2.36E-06	0.001	22.40	
IL-6	rs11842416	C	T	0.3509	0.0764	0.2416	4.39E-06	0.045	21.10	
IL-6	rs12973192	T	C	0.0656	0.0144	0.6581	4.94E-06	0.002	20.75	
IL-6	rs142321490	G	A	0.1372	0.03	0.0169	4.70E-06	0.001	20.92	
IL-6	rs146107389	T	C	-0.1057	0.0223	0.0159	2.18E-06	0.000	22.47	
IL-6	rs17070492	T	C	-0.1764	0.0155	0.9046	3.77E-30	0.005	129.52	100.00%
IL-6	rs17148125	G	A	-0.0693	0.0139	0.1412	6.69E-07	0.001	24.86	
IL-6	rs2013478	C	T	-0.0906	0.0193	0.6590	2.67E-06	0.004	22.04	
IL-6	rs2285642	C	A	0.1317	0.0281	0.4483	2.73E-06	0.009	21.97	
IL-6	rs34182166	C	T	0.0869	0.0183	0.9463	2.12E-06	0.001	22.55	
IL-6	rs34517613	A	G	0.1976	0.0337	0.8718	4.66E-09	0.009	34.38	
IL-6	rs3849943	A	C	0.5145	0.0672	0.7674	1.85E-14	0.095	58.62	
IL-6	rs538622	C	T	-0.089	0.0182	0.3877	1.06E-06	0.004	23.91	
IL-6	rs61527579	T	C	0.2058	0.0444	0.1789	3.63E-06	0.012	21.48	
IL-6	rs61880881	A	G	0.0688	0.0151	0.9423	4.92E-06	0.001	20.76	

IL-6	rs67710834	T	C	0.1462	0.0273	0.3907	8.59E-08	0.010	28.68		
IL-7	rs10139154	T	C	0.0767	0.0146	0.659	1.44E-07	0.003	27.60		
IL-7	rs10463311	T	C	-0.0854	0.0156	0.2714	4.00E-08	0.003	29.97		
IL-7	rs11195948	T	C	-0.0707	0.0144	0.6282	9.14E-07	0.002	24.11		
IL-7	rs11786739	G	A	0.1371	0.029	0.9364	2.20E-06	0.002	22.35		
IL-7	rs118049474	C	T	-0.1335	0.0284	0.0577	2.66E-06	0.002	22.10		
IL-7	rs11842416	C	T	0.0814	0.0172	0.2416	2.36E-06	0.002	22.40		
IL-7	rs146107389	C	T	0.3509	0.0764	0.0159	4.39E-06	0.004	21.10		
IL-7	rs2013478	T	C	0.0656	0.0144	0.6590	4.94E-06	0.002	20.75		
IL-7	rs34182166	G	A	0.1372	0.03	0.9463	4.70E-06	0.002	20.92		
IL-7	rs34517613	T	C	-0.1057	0.0223	0.8718	2.18E-06	0.002	22.47		
IL-7	rs3849943	T	C	-0.1764	0.0155	0.7674	3.77E-30	0.011	129.52	99.90%	
IL-7	rs538622	G	A	-0.0693	0.0139	0.3877	6.69E-07	0.002	24.86		
IL-7	rs61527579	C	T	-0.0906	0.0193	0.1789	2.67E-06	0.002	22.04		
IL-7	rs61880881	C	A	0.1317	0.0281	0.9423	2.73E-06	0.002	21.97		
IL-7	rs72714928	C	T	0.0869	0.0183	0.1670	2.12E-06	0.002	22.55		
IL-7	rs74654358	A	G	0.1976	0.0337	0.0318	4.66E-09	0.002	34.38		
IL-7	rs75087725	A	C	0.5145	0.0672	0.0119	1.85E-14	0.006	58.62		
IL-7	rs7552104	C	T	-0.089	0.0182	0.1779	1.06E-06	0.002	23.91		
IL-7	rs79068040	T	C	0.2058	0.0444	0.9781	3.63E-06	0.002	21.48		
IL-7	rs8125607	A	G	0.0688	0.0151	0.3221	4.92E-06	0.002	20.76		
IL-7	rs9901522	T	C	0.1462	0.0273	0.9423	8.59E-08	0.002	28.68		
IL-8	rs10139154	T	C	0.0767	0.0146	0.659	1.44E-07	0.003	27.60		
IL-8	rs10143310	T	C	-0.0854	0.0156	0.2237	4.00E-08	0.003	29.97	99.20%	
IL-8	rs10463311	T	C	-0.0707	0.0144	0.2714	9.14E-07	0.002	24.11		
IL-8	rs11195948	G	A	0.1371	0.029	0.6282	2.20E-06	0.009	22.35		

IL-8	rs11786739	C	T	-0.1335	0.0284	0.9364	2.66E-06	0.002	22.10	
IL-8	rs118049474	C	T	0.0814	0.0172	0.0577	2.36E-06	0.001	22.40	
IL-8	rs11842416	C	T	0.3509	0.0764	0.2416	4.39E-06	0.045	21.10	
IL-8	rs12973192	T	C	0.0656	0.0144	0.6581	4.94E-06	0.002	20.75	
IL-8	rs142321490	G	A	0.1372	0.03	0.0169	4.70E-06	0.001	20.92	
IL-8	rs146107389	T	C	-0.1057	0.0223	0.0159	2.18E-06	0.000	22.47	
IL-8	rs17070492	T	C	-0.1764	0.0155	0.9046	3.77E-30	0.005	129.52	
IL-8	rs17148125	G	A	-0.0693	0.0139	0.1412	6.69E-07	0.001	24.86	
IL-8	rs2013478	C	T	-0.0906	0.0193	0.6590	2.67E-06	0.004	22.04	
IL-8	rs2285642	C	A	0.1317	0.0281	0.4483	2.73E-06	0.009	21.97	
IL-8	rs34182166	C	T	0.0869	0.0183	0.9463	2.12E-06	0.001	22.55	
IL-8	rs34517613	A	G	0.1976	0.0337	0.8718	4.66E-09	0.009	34.38	
IL-8	rs3849943	A	C	0.5145	0.0672	0.7674	1.85E-14	0.095	58.62	
IL-8	rs538622	C	T	-0.089	0.0182	0.3877	1.06E-06	0.004	23.91	
IL-8	rs61527579	T	C	0.2058	0.0444	0.1789	3.63E-06	0.012	21.48	
IL-8	rs61880881	A	G	0.0688	0.0151	0.9423	4.92E-06	0.001	20.76	
IL-8	rs67710834	T	C	0.1462	0.0273	0.3907	8.59E-08	0.010	28.68	
IL-9	rs10139154	T	C	0.0767	0.0146	0.659	1.44E-07	0.003	27.60	
IL-9	rs10463311	T	C	-0.0854	0.0156	0.2714	4.00E-08	0.003	29.97	
IL-9	rs11195948	T	C	-0.0707	0.0144	0.6282	9.14E-07	0.002	24.11	
IL-9	rs11786739	G	A	0.1371	0.029	0.9364	2.20E-06	0.002	22.35	
IL-9	rs118049474	C	T	-0.1335	0.0284	0.0577	2.66E-06	0.002	22.10	76.60%
IL-9	rs11842416	C	T	0.0814	0.0172	0.2416	2.36E-06	0.002	22.40	
IL-9	rs146107389	C	T	0.3509	0.0764	0.0159	4.39E-06	0.004	21.10	
IL-9	rs2013478	T	C	0.0656	0.0144	0.6590	4.94E-06	0.002	20.75	
IL-9	rs34182166	G	A	0.1372	0.03	0.9463	4.70E-06	0.002	20.92	

IL-9	rs34517613	T	C	-0.1057	0.0223	0.8718	2.18E-06	0.002	22.47	
IL-9	rs3849943	T	C	-0.1764	0.0155	0.7674	3.77E-30	0.011	129.52	
IL-9	rs538622	G	A	-0.0693	0.0139	0.3877	6.69E-07	0.002	24.86	
IL-9	rs61527579	C	T	-0.0906	0.0193	0.1789	2.67E-06	0.002	22.04	
IL-9	rs61880881	C	A	0.1317	0.0281	0.9423	2.73E-06	0.002	21.97	
IL-9	rs72714928	C	T	0.0869	0.0183	0.1670	2.12E-06	0.002	22.55	
IL-9	rs74654358	A	G	0.1976	0.0337	0.0318	4.66E-09	0.002	34.38	
IL-9	rs75087725	A	C	0.5145	0.0672	0.0119	1.85E-14	0.006	58.62	
IL-9	rs7552104	C	T	-0.089	0.0182	0.1779	1.06E-06	0.002	23.91	
IL-9	rs79068040	T	C	0.2058	0.0444	0.9781	3.63E-06	0.002	21.48	
IL-9	rs8125607	A	G	0.0688	0.0151	0.3221	4.92E-06	0.002	20.76	
IL-9	rs9901522	T	C	0.1462	0.0273	0.9423	8.59E-08	0.002	28.68	
IP-10	rs10139154	T	C	0.0767	0.0146	0.659	1.44E-07	0.003	27.60	
IP-10	rs10463311	T	C	-0.0854	0.0156	0.2714	4.00E-08	0.003	29.97	
IP-10	rs11195948	T	C	-0.0707	0.0144	0.6282	9.14E-07	0.002	24.11	
IP-10	rs11786739	G	A	0.1371	0.029	0.9364	2.20E-06	0.002	22.35	
IP-10	rs118049474	C	T	-0.1335	0.0284	0.0577	2.66E-06	0.002	22.10	
IP-10	rs11842416	C	T	0.0814	0.0172	0.2416	2.36E-06	0.002	22.40	
IP-10	rs146107389	C	T	0.3509	0.0764	0.0159	4.39E-06	0.004	21.10	
IP-10	rs2013478	T	C	0.0656	0.0144	0.6590	4.94E-06	0.002	20.75	100.00%
IP-10	rs34182166	G	A	0.1372	0.03	0.9463	4.70E-06	0.002	20.92	
IP-10	rs34517613	T	C	-0.1057	0.0223	0.8718	2.18E-06	0.002	22.47	
IP-10	rs3849943	T	C	-0.1764	0.0155	0.7674	3.77E-30	0.011	129.52	
IP-10	rs538622	G	A	-0.0693	0.0139	0.3877	6.69E-07	0.002	24.86	
IP-10	rs61527579	C	T	-0.0906	0.0193	0.1789	2.67E-06	0.002	22.04	
IP-10	rs61880881	C	A	0.1317	0.0281	0.9423	2.73E-06	0.002	21.97	

IP-10	rs72714928	C	T	0.0869	0.0183	0.1670	2.12E-06	0.002	22.55		
IP-10	rs74654358	A	G	0.1976	0.0337	0.0318	4.66E-09	0.002	34.38		
IP-10	rs75087725	A	C	0.5145	0.0672	0.0119	1.85E-14	0.006	58.62		
IP-10	rs7552104	C	T	-0.089	0.0182	0.1779	1.06E-06	0.002	23.91		
IP-10	rs79068040	T	C	0.2058	0.0444	0.9781	3.63E-06	0.002	21.48		
IP-10	rs8125607	A	G	0.0688	0.0151	0.3221	4.92E-06	0.002	20.76		
IP-10	rs9901522	T	C	0.1462	0.0273	0.9423	8.59E-08	0.002	28.68		
MCP-1-MCAF	rs10139154	T	C	0.0767	0.0146	0.659	1.44E-07	0.003	27.60		
MCP-1-MCAF	rs10463311	T	C	-0.0854	0.0156	0.2714	4.00E-08	0.003	29.97		
MCP-1-MCAF	rs11195948	T	C	-0.0707	0.0144	0.6282	9.14E-07	0.002	24.11		
MCP-1-MCAF	rs11786739	G	A	0.1371	0.029	0.9364	2.20E-06	0.002	22.35		
MCP-1-MCAF	rs118049474	C	T	-0.1335	0.0284	0.0577	2.66E-06	0.002	22.10		
MCP-1-MCAF	rs11842416	C	T	0.0814	0.0172	0.2416	2.36E-06	0.002	22.40		
MCP-1-MCAF	rs146107389	C	T	0.3509	0.0764	0.0159	4.39E-06	0.004	21.10		
MCP-1-MCAF	rs2013478	T	C	0.0656	0.0144	0.6590	4.94E-06	0.002	20.75		
MCP-1-MCAF	rs34182166	G	A	0.1372	0.03	0.9463	4.70E-06	0.002	20.92		
MCP-1-MCAF	rs34517613	T	C	-0.1057	0.0223	0.8718	2.18E-06	0.002	22.47	78.70%	
MCP-1-MCAF	rs3849943	T	C	-0.1764	0.0155	0.7674	3.77E-30	0.011	129.52		
MCP-1-MCAF	rs538622	G	A	-0.0693	0.0139	0.3877	6.69E-07	0.002	24.86		
MCP-1-MCAF	rs61527579	C	T	-0.0906	0.0193	0.1789	2.67E-06	0.002	22.04		
MCP-1-MCAF	rs61880881	C	A	0.1317	0.0281	0.9423	2.73E-06	0.002	21.97		
MCP-1-MCAF	rs72714928	C	T	0.0869	0.0183	0.1670	2.12E-06	0.002	22.55		
MCP-1-MCAF	rs74654358	A	G	0.1976	0.0337	0.0318	4.66E-09	0.002	34.38		
MCP-1-MCAF	rs75087725	A	C	0.5145	0.0672	0.0119	1.85E-14	0.006	58.62		
MCP-1-MCAF	rs7552104	C	T	-0.089	0.0182	0.1779	1.06E-06	0.002	23.91		
MCP-1-MCAF	rs79068040	T	C	0.2058	0.0444	0.9781	3.63E-06	0.002	21.48		

MCP-1-MCAF	rs8125607	A	G	0.0688	0.0151	0.3221	4.92E-06	0.002	20.76	
MCP-1-MCAF	rs9901522	T	C	0.1462	0.0273	0.9423	8.59E-08	0.002	28.68	
MCP-3	rs10139154	T	C	0.0767	0.0146	0.659	1.44E-07	0.003	27.60	
MCP-3	rs10143310	T	C	-0.0854	0.0156	0.2237	4.00E-08	0.003	29.97	
MCP-3	rs10463311	T	C	-0.0707	0.0144	0.2714	9.14E-07	0.002	24.11	
MCP-3	rs11195948	G	A	0.1371	0.029	0.6282	2.20E-06	0.009	22.35	
MCP-3	rs11786739	C	T	-0.1335	0.0284	0.9364	2.66E-06	0.002	22.10	
MCP-3	rs118049474	C	T	0.0814	0.0172	0.0577	2.36E-06	0.001	22.40	
MCP-3	rs11842416	C	T	0.3509	0.0764	0.2416	4.39E-06	0.045	21.10	
MCP-3	rs12973192	T	C	0.0656	0.0144	0.6581	4.94E-06	0.002	20.75	
MCP-3	rs142321490	G	A	0.1372	0.03	0.0169	4.70E-06	0.001	20.92	
MCP-3	rs146107389	T	C	-0.1057	0.0223	0.0159	2.18E-06	0.000	22.47	100.00%
MCP-3	rs17070492	T	C	-0.1764	0.0155	0.9046	3.77E-30	0.005	129.52	
MCP-3	rs17148125	G	A	-0.0693	0.0139	0.1412	6.69E-07	0.001	24.86	
MCP-3	rs2013478	C	T	-0.0906	0.0193	0.6590	2.67E-06	0.004	22.04	
MCP-3	rs2285642	C	A	0.1317	0.0281	0.4483	2.73E-06	0.009	21.97	
MCP-3	rs34182166	C	T	0.0869	0.0183	0.9463	2.12E-06	0.001	22.55	
MCP-3	rs34517613	A	G	0.1976	0.0337	0.8718	4.66E-09	0.009	34.38	
MCP-3	rs3849943	C	T	-0.089	0.0182	0.7674	1.06E-06	0.003	23.91	
MCP-3	rs538622	T	C	0.2058	0.0444	0.3877	3.63E-06	0.020	21.48	
MCP-3	rs61527579	A	G	0.0688	0.0151	0.1789	4.92E-06	0.001	20.76	
MCP-3	rs61880881	T	C	0.1462	0.0273	0.9423	8.59E-08	0.002	28.68	
M-CSF	rs10139154	T	C	0.0767	0.0146	0.659	1.44E-07	0.003	27.60	
M-CSF	rs10463311	T	C	-0.0854	0.0156	0.2714	4.00E-08	0.003	29.97	6.20%
M-CSF	rs11195948	T	C	-0.0707	0.0144	0.6282	9.14E-07	0.002	24.11	
M-CSF	rs11786739	G	A	0.1371	0.029	0.9364	2.20E-06	0.002	22.35	

M-CSF	rs118049474	C	T	-0.1335	0.0284	0.0577	2.66E-06	0.002	22.10	
M-CSF	rs11842416	C	T	0.0814	0.0172	0.2416	2.36E-06	0.002	22.40	
M-CSF	rs146107389	C	T	0.3509	0.0764	0.0159	4.39E-06	0.004	21.10	
M-CSF	rs2013478	T	C	0.0656	0.0144	0.6590	4.94E-06	0.002	20.75	
M-CSF	rs34182166	G	A	0.1372	0.03	0.9463	4.70E-06	0.002	20.92	
M-CSF	rs34517613	T	C	-0.1057	0.0223	0.8718	2.18E-06	0.002	22.47	
M-CSF	rs3849943	T	C	-0.1764	0.0155	0.7674	3.77E-30	0.011	129.52	
M-CSF	rs538622	G	A	-0.0693	0.0139	0.3877	6.69E-07	0.002	24.86	
M-CSF	rs61527579	C	T	-0.0906	0.0193	0.1789	2.67E-06	0.002	22.04	
M-CSF	rs61880881	C	A	0.1317	0.0281	0.9423	2.73E-06	0.002	21.97	
M-CSF	rs72714928	C	T	0.0869	0.0183	0.1670	2.12E-06	0.002	22.55	
M-CSF	rs74654358	A	G	0.1976	0.0337	0.0318	4.66E-09	0.002	34.38	
M-CSF	rs75087725	A	C	0.5145	0.0672	0.0119	1.85E-14	0.006	58.62	
M-CSF	rs7552104	C	T	-0.089	0.0182	0.1779	1.06E-06	0.002	23.91	
M-CSF	rs79068040	T	C	0.2058	0.0444	0.9781	3.63E-06	0.002	21.48	
M-CSF	rs8125607	A	G	0.0688	0.0151	0.3221	4.92E-06	0.002	20.76	
M-CSF	rs9901522	T	C	0.1462	0.0273	0.9423	8.59E-08	0.002	28.68	
MIF	rs10139154	T	C	0.0767	0.0146	0.659	1.44E-07	0.003	27.60	
MIF	rs10463311	T	C	-0.0854	0.0156	0.2714	4.00E-08	0.003	29.97	
MIF	rs11195948	T	C	-0.0707	0.0144	0.6282	9.14E-07	0.002	24.11	
MIF	rs11786739	G	A	0.1371	0.029	0.9364	2.20E-06	0.002	22.35	
MIF	rs118049474	C	T	-0.1335	0.0284	0.0577	2.66E-06	0.002	22.10	100.00%
MIF	rs11842416	C	T	0.0814	0.0172	0.2416	2.36E-06	0.002	22.40	
MIF	rs146107389	C	T	0.3509	0.0764	0.0159	4.39E-06	0.004	21.10	
MIF	rs2013478	T	C	0.0656	0.0144	0.6590	4.94E-06	0.002	20.75	
MIF	rs34182166	G	A	0.1372	0.03	0.9463	4.70E-06	0.002	20.92	

MIF	rs34517613	T	C	-0.1057	0.0223	0.8718	2.18E-06	0.002	22.47	
MIF	rs3849943	T	C	-0.1764	0.0155	0.7674	3.77E-30	0.011	129.52	
MIF	rs538622	G	A	-0.0693	0.0139	0.3877	6.69E-07	0.002	24.86	
MIF	rs61527579	C	T	-0.0906	0.0193	0.1789	2.67E-06	0.002	22.04	
MIF	rs61880881	C	A	0.1317	0.0281	0.9423	2.73E-06	0.002	21.97	
MIF	rs72714928	C	T	0.0869	0.0183	0.1670	2.12E-06	0.002	22.55	
MIF	rs74654358	A	G	0.1976	0.0337	0.0318	4.66E-09	0.002	34.38	
MIF	rs75087725	A	C	0.5145	0.0672	0.0119	1.85E-14	0.006	58.62	
MIF	rs7552104	C	T	-0.089	0.0182	0.1779	1.06E-06	0.002	23.91	
MIF	rs79068040	T	C	0.2058	0.0444	0.9781	3.63E-06	0.002	21.48	
MIF	rs8125607	A	G	0.0688	0.0151	0.3221	4.92E-06	0.002	20.76	
MIF	rs9901522	T	C	0.1462	0.0273	0.9423	8.59E-08	0.002	28.68	
MIG	rs10139154	T	C	0.0767	0.0146	0.659	1.44E-07	0.003	27.60	
MIG	rs10143310	T	C	-0.0854	0.0156	0.2237	4.00E-08	0.003	29.97	
MIG	rs10463311	T	C	-0.0707	0.0144	0.2714	9.14E-07	0.002	24.11	
MIG	rs11195948	G	A	0.1371	0.029	0.6282	2.20E-06	0.009	22.35	
MIG	rs11786739	C	T	-0.1335	0.0284	0.9364	2.66E-06	0.002	22.10	
MIG	rs118049474	C	T	0.0814	0.0172	0.0577	2.36E-06	0.001	22.40	
MIG	rs11842416	C	T	0.3509	0.0764	0.2416	4.39E-06	0.045	21.10	91.10%
MIG	rs12973192	T	C	0.0656	0.0144	0.6581	4.94E-06	0.002	20.75	
MIG	rs142321490	G	A	0.1372	0.03	0.0169	4.70E-06	0.001	20.92	
MIG	rs146107389	T	C	-0.1057	0.0223	0.0159	2.18E-06	0.000	22.47	
MIG	rs17070492	T	C	-0.1764	0.0155	0.9046	3.77E-30	0.005	129.52	
MIG	rs17148125	G	A	-0.0693	0.0139	0.1412	6.69E-07	0.001	24.86	
MIG	rs2013478	C	T	-0.0906	0.0193	0.6590	2.67E-06	0.004	22.04	
MIG	rs2285642	C	A	0.1317	0.0281	0.4483	2.73E-06	0.009	21.97	

MIG	rs34182166	C	T	0.0869	0.0183	0.9463	2.12E-06	0.001	22.55	
MIG	rs34517613	A	G	0.1976	0.0337	0.8718	4.66E-09	0.009	34.38	
MIG	rs3849943	A	C	0.5145	0.0672	0.7674	1.85E-14	0.095	58.62	
MIG	rs538622	C	T	-0.089	0.0182	0.3877	1.06E-06	0.004	23.91	
MIG	rs61527579	T	C	0.2058	0.0444	0.1789	3.63E-06	0.012	21.48	
MIG	rs61880881	A	G	0.0688	0.0151	0.9423	4.92E-06	0.001	20.76	
MIG	rs67710834	T	C	0.1462	0.0273	0.3907	8.59E-08	0.010	28.68	
MIP-1A	rs10139154	T	C	0.0767	0.0146	0.659	1.44E-07	0.003	27.60	
MIP-1A	rs10463311	T	C	-0.0854	0.0156	0.2714	4.00E-08	0.003	29.97	
MIP-1A	rs11195948	T	C	-0.0707	0.0144	0.6282	9.14E-07	0.002	24.11	
MIP-1A	rs11786739	G	A	0.1371	0.029	0.9364	2.20E-06	0.002	22.35	
MIP-1A	rs118049474	C	T	-0.1335	0.0284	0.0577	2.66E-06	0.002	22.10	
MIP-1A	rs11842416	C	T	0.0814	0.0172	0.2416	2.36E-06	0.002	22.40	
MIP-1A	rs146107389	C	T	0.3509	0.0764	0.0159	4.39E-06	0.004	21.10	
MIP-1A	rs2013478	T	C	0.0656	0.0144	0.6590	4.94E-06	0.002	20.75	
MIP-1A	rs34182166	G	A	0.1372	0.03	0.9463	4.70E-06	0.002	20.92	
MIP-1A	rs34517613	T	C	-0.1057	0.0223	0.8718	2.18E-06	0.002	22.47	100.00%
MIP-1A	rs3849943	T	C	-0.1764	0.0155	0.7674	3.77E-30	0.011	129.52	
MIP-1A	rs538622	G	A	-0.0693	0.0139	0.3877	6.69E-07	0.002	24.86	
MIP-1A	rs61527579	C	T	-0.0906	0.0193	0.1789	2.67E-06	0.002	22.04	
MIP-1A	rs61880881	C	A	0.1317	0.0281	0.9423	2.73E-06	0.002	21.97	
MIP-1A	rs72714928	C	T	0.0869	0.0183	0.1670	2.12E-06	0.002	22.55	
MIP-1A	rs74654358	A	G	0.1976	0.0337	0.0318	4.66E-09	0.002	34.38	
MIP-1A	rs75087725	A	C	0.5145	0.0672	0.0119	1.85E-14	0.006	58.62	
MIP-1A	rs7552104	C	T	-0.089	0.0182	0.1779	1.06E-06	0.002	23.91	
MIP-1A	rs79068040	T	C	0.2058	0.0444	0.9781	3.63E-06	0.002	21.48	

MIP-1A	rs8125607	A	G	0.0688	0.0151	0.3221	4.92E-06	0.002	20.76	
MIP-1A	rs9901522	T	C	0.1462	0.0273	0.9423	8.59E-08	0.002	28.68	
MIP-1B	rs10139154	T	C	0.0767	0.0146	0.659	1.44E-07	0.003	27.60	
MIP-1B	rs10463311	T	C	-0.0854	0.0156	0.2714	4.00E-08	0.003	29.97	
MIP-1B	rs11195948	T	C	-0.0707	0.0144	0.6282	9.14E-07	0.002	24.11	
MIP-1B	rs11786739	G	A	0.1371	0.029	0.9364	2.20E-06	0.002	22.35	
MIP-1B	rs118049474	C	T	-0.1335	0.0284	0.0577	2.66E-06	0.002	22.10	
MIP-1B	rs11842416	C	T	0.0814	0.0172	0.2416	2.36E-06	0.002	22.40	
MIP-1B	rs146107389	C	T	0.3509	0.0764	0.0159	4.39E-06	0.004	21.10	
MIP-1B	rs2013478	T	C	0.0656	0.0144	0.6590	4.94E-06	0.002	20.75	
MIP-1B	rs34182166	G	A	0.1372	0.03	0.9463	4.70E-06	0.002	20.92	
MIP-1B	rs34517613	T	C	-0.1057	0.0223	0.8718	2.18E-06	0.002	22.47	
MIP-1B	rs3849943	T	C	-0.1764	0.0155	0.7674	3.77E-30	0.011	129.52	8.20%
MIP-1B	rs538622	G	A	-0.0693	0.0139	0.3877	6.69E-07	0.002	24.86	
MIP-1B	rs61527579	C	T	-0.0906	0.0193	0.1789	2.67E-06	0.002	22.04	
MIP-1B	rs61880881	C	A	0.1317	0.0281	0.9423	2.73E-06	0.002	21.97	
MIP-1B	rs72714928	C	T	0.0869	0.0183	0.1670	2.12E-06	0.002	22.55	
MIP-1B	rs74654358	A	G	0.1976	0.0337	0.0318	4.66E-09	0.002	34.38	
MIP-1B	rs75087725	A	C	0.5145	0.0672	0.0119	1.85E-14	0.006	58.62	
MIP-1B	rs7552104	C	T	-0.089	0.0182	0.1779	1.06E-06	0.002	23.91	
MIP-1B	rs79068040	T	C	0.2058	0.0444	0.9781	3.63E-06	0.002	21.48	
MIP-1B	rs8125607	A	G	0.0688	0.0151	0.3221	4.92E-06	0.002	20.76	
MIP-1B	rs9901522	T	C	0.1462	0.0273	0.9423	8.59E-08	0.002	28.68	
PDGF-BB	rs10139154	T	C	0.0767	0.0146	0.659	1.44E-07	0.003	27.60	
PDGF-BB	rs10143310	C	G	0.0821	0.0161	0.2237	3.23E-07	0.002	26.00	81.70%
PDGF-BB	rs10463311	T	C	-0.0854	0.0156	0.2714	4.00E-08	0.003	29.97	

PDGF-BB	rs11195948	T	C	-0.0707	0.0144	0.6282	9.14E-07	0.002	24.11	
PDGF-BB	rs11786739	G	A	0.1371	0.029	0.9364	2.20E-06	0.002	22.35	
PDGF-BB	rs118049474	C	T	-0.1335	0.0284	0.0577	2.66E-06	0.002	22.10	
PDGF-BB	rs11842416	C	T	0.0814	0.0172	0.2416	2.36E-06	0.002	22.40	
PDGF-BB	rs12973192	G	C	0.1205	0.0153	0.6581	3.92E-15	0.007	62.03	
PDGF-BB	rs142321490	C	G	0.3172	0.0513	0.0169	6.15E-10	0.003	38.23	
PDGF-BB	rs146107389	C	T	0.3509	0.0764	0.0159	4.39E-06	0.004	21.10	
PDGF-BB	rs17070492	G	C	-0.1242	0.0234	0.9046	1.04E-07	0.003	28.17	
PDGF-BB	rs17148125	C	G	0.0851	0.0181	0.1412	2.66E-06	0.002	22.11	
PDGF-BB	rs2013478	T	C	0.0656	0.0144	0.6590	4.94E-06	0.002	20.75	
PDGF-BB	rs2285642	C	G	0.0674	0.0141	0.4483	1.80E-06	0.002	22.85	
PDGF-BB	rs34182166	G	A	0.1372	0.03	0.9463	4.70E-06	0.002	20.92	
PDGF-BB	rs34517613	T	C	-0.1057	0.0223	0.8718	2.18E-06	0.002	22.47	
PDGF-BB	rs3849943	T	C	-0.1764	0.0155	0.7674	3.77E-30	0.011	129.52	
PDGF-BB	rs538622	G	A	-0.0693	0.0139	0.3877	6.69E-07	0.002	24.86	
PDGF-BB	rs61527579	C	T	-0.0906	0.0193	0.1789	2.67E-06	0.002	22.04	
PDGF-BB	rs61880881	C	A	0.1317	0.0281	0.9423	2.73E-06	0.002	21.97	
PDGF-BB	rs67710834	C	G	0.0698	0.015	0.3907	3.31E-06	0.002	21.65	
PDGF-BB	rs72714928	C	T	0.0869	0.0183	0.1670	2.12E-06	0.002	22.55	
PDGF-BB	rs74654358	A	G	0.1976	0.0337	0.0318	4.66E-09	0.002	34.38	
PDGF-BB	rs75087725	A	C	0.5145	0.0672	0.0119	1.85E-14	0.006	58.62	
PDGF-BB	rs7552104	C	T	-0.089	0.0182	0.1779	1.06E-06	0.002	23.91	
PDGF-BB	rs79068040	T	C	0.2058	0.0444	0.9781	3.63E-06	0.002	21.48	
PDGF-BB	rs8125607	A	G	0.0688	0.0151	0.3221	4.92E-06	0.002	20.76	
PDGF-BB	rs9901522	T	C	0.1462	0.0273	0.9423	8.59E-08	0.002	28.68	
RANTES	rs10139154	T	C	0.0767	0.0146	0.659	1.44E-07	0.003	27.60	79.30%

RANTES	rs10143310	T	C	-0.0854	0.0156	0.2237	4.00E-08	0.003	29.97	
RANTES	rs10463311	T	C	-0.0707	0.0144	0.2714	9.14E-07	0.002	24.11	
RANTES	rs11195948	G	A	0.1371	0.029	0.6282	2.20E-06	0.009	22.35	
RANTES	rs11786739	C	T	-0.1335	0.0284	0.9364	2.66E-06	0.002	22.10	
RANTES	rs118049474	C	T	0.0814	0.0172	0.0577	2.36E-06	0.001	22.40	
RANTES	rs11842416	C	T	0.3509	0.0764	0.2416	4.39E-06	0.045	21.10	
RANTES	rs12973192	T	C	0.0656	0.0144	0.6581	4.94E-06	0.002	20.75	
RANTES	rs142321490	G	A	0.1372	0.03	0.0169	4.70E-06	0.001	20.92	
RANTES	rs146107389	T	C	-0.1057	0.0223	0.0159	2.18E-06	0.000	22.47	
RANTES	rs17070492	T	C	-0.1764	0.0155	0.9046	3.77E-30	0.005	129.52	
RANTES	rs17148125	G	A	-0.0693	0.0139	0.1412	6.69E-07	0.001	24.86	
RANTES	rs2013478	C	T	-0.0906	0.0193	0.6590	2.67E-06	0.004	22.04	
RANTES	rs2285642	C	A	0.1317	0.0281	0.4483	2.73E-06	0.009	21.97	
RANTES	rs34182166	C	T	0.0869	0.0183	0.9463	2.12E-06	0.001	22.55	
RANTES	rs34517613	A	G	0.1976	0.0337	0.8718	4.66E-09	0.009	34.38	
RANTES	rs3849943	A	C	0.5145	0.0672	0.7674	1.85E-14	0.095	58.62	
RANTES	rs538622	C	T	-0.089	0.0182	0.3877	1.06E-06	0.004	23.91	
RANTES	rs61527579	T	C	0.2058	0.0444	0.1789	3.63E-06	0.012	21.48	
RANTES	rs61880881	A	G	0.0688	0.0151	0.9423	4.92E-06	0.001	20.76	
RANTES	rs67710834	T	C	0.1462	0.0273	0.3907	8.59E-08	0.010	28.68	
SCF	rs10139154	T	C	0.0767	0.0146	0.659	1.44E-07	0.003	27.60	
SCF	rs10143310	T	C	-0.0854	0.0156	0.2237	4.00E-08	0.003	29.97	
SCF	rs10463311	T	C	-0.0707	0.0144	0.2714	9.14E-07	0.002	24.11	
SCF	rs11195948	G	A	0.1371	0.029	0.6282	2.20E-06	0.009	22.35	99.80%
SCF	rs11786739	C	T	-0.1335	0.0284	0.9364	2.66E-06	0.002	22.10	
SCF	rs118049474	C	T	0.0814	0.0172	0.0577	2.36E-06	0.001	22.40	

SCF	rs11842416	C	T	0.3509	0.0764	0.2416	4.39E-06	0.045	21.10	
SCF	rs12973192	T	C	0.0656	0.0144	0.6581	4.94E-06	0.002	20.75	
SCF	rs142321490	G	A	0.1372	0.03	0.0169	4.70E-06	0.001	20.92	
SCF	rs146107389	T	C	-0.1057	0.0223	0.0159	2.18E-06	0.000	22.47	
SCF	rs17070492	T	C	-0.1764	0.0155	0.9046	3.77E-30	0.005	129.52	
SCF	rs17148125	G	A	-0.0693	0.0139	0.1412	6.69E-07	0.001	24.86	
SCF	rs2013478	C	T	-0.0906	0.0193	0.6590	2.67E-06	0.004	22.04	
SCF	rs2285642	C	A	0.1317	0.0281	0.4483	2.73E-06	0.009	21.97	
SCF	rs34182166	C	T	0.0869	0.0183	0.9463	2.12E-06	0.001	22.55	
SCF	rs34517613	A	G	0.1976	0.0337	0.8718	4.66E-09	0.009	34.38	
SCF	rs3849943	A	C	0.5145	0.0672	0.7674	1.85E-14	0.095	58.62	
SCF	rs538622	C	T	-0.089	0.0182	0.3877	1.06E-06	0.004	23.91	
SCF	rs61527579	T	C	0.2058	0.0444	0.1789	3.63E-06	0.012	21.48	
SCF	rs61880881	A	G	0.0688	0.0151	0.9423	4.92E-06	0.001	20.76	
SCF	rs67710834	T	C	0.1462	0.0273	0.3907	8.59E-08	0.010	28.68	
SCGF	rs10139154	T	C	0.0767	0.0146	0.659	1.44E-07	0.003	27.60	
SCGF	rs10463311	T	C	-0.0854	0.0156	0.2714	4.00E-08	0.003	29.97	
SCGF	rs11195948	T	C	-0.0707	0.0144	0.6282	9.14E-07	0.002	24.11	
SCGF	rs11786739	G	A	0.1371	0.029	0.9364	2.20E-06	0.002	22.35	
SCGF	rs118049474	C	T	-0.1335	0.0284	0.0577	2.66E-06	0.002	22.10	
SCGF	rs11842416	C	T	0.0814	0.0172	0.2416	2.36E-06	0.002	22.40	100.00%
SCGF	rs146107389	C	T	0.3509	0.0764	0.0159	4.39E-06	0.004	21.10	
SCGF	rs2013478	T	C	0.0656	0.0144	0.6590	4.94E-06	0.002	20.75	
SCGF	rs34182166	G	A	0.1372	0.03	0.9463	4.70E-06	0.002	20.92	
SCGF	rs34517613	T	C	-0.1057	0.0223	0.8718	2.18E-06	0.002	22.47	
SCGF	rs3849943	T	C	-0.1764	0.0155	0.7674	3.77E-30	0.011	129.52	

SCGF	rs538622	G	A	-0.0693	0.0139	0.3877	6.69E-07	0.002	24.86		
SCGF	rs61527579	C	T	-0.0906	0.0193	0.1789	2.67E-06	0.002	22.04		
SCGF	rs61880881	C	A	0.1317	0.0281	0.9423	2.73E-06	0.002	21.97		
SCGF	rs72714928	C	T	0.0869	0.0183	0.1670	2.12E-06	0.002	22.55		
SCGF	rs74654358	A	G	0.1976	0.0337	0.0318	4.66E-09	0.002	34.38		
SCGF	rs75087725	A	C	0.5145	0.0672	0.0119	1.85E-14	0.006	58.62		
SCGF	rs7552104	C	T	-0.089	0.0182	0.1779	1.06E-06	0.002	23.91		
SCGF	rs79068040	T	C	0.2058	0.0444	0.9781	3.63E-06	0.002	21.48		
SCGF	rs8125607	A	G	0.0688	0.0151	0.3221	4.92E-06	0.002	20.76		
SCGF	rs9901522	T	C	0.1462	0.0273	0.9423	8.59E-08	0.002	28.68		
SDF-1A	rs10139154	T	C	0.0767	0.0146	0.659	1.44E-07	0.003	27.60		
SDF-1A	rs10143310	T	C	-0.0854	0.0156	0.2237	4.00E-08	0.003	29.97		
SDF-1A	rs10463311	T	C	-0.0707	0.0144	0.2714	9.14E-07	0.002	24.11		
SDF-1A	rs11195948	G	A	0.1371	0.029	0.6282	2.20E-06	0.009	22.35		
SDF-1A	rs11786739	C	T	-0.1335	0.0284	0.9364	2.66E-06	0.002	22.10		
SDF-1A	rs118049474	C	T	0.0814	0.0172	0.0577	2.36E-06	0.001	22.40		
SDF-1A	rs11842416	C	T	0.3509	0.0764	0.2416	4.39E-06	0.045	21.10		
SDF-1A	rs12973192	T	C	0.0656	0.0144	0.6581	4.94E-06	0.002	20.75		
SDF-1A	rs142321490	G	A	0.1372	0.03	0.0169	4.70E-06	0.001	20.92	99.40%	
SDF-1A	rs146107389	T	C	-0.1057	0.0223	0.0159	2.18E-06	0.000	22.47		
SDF-1A	rs17070492	T	C	-0.1764	0.0155	0.9046	3.77E-30	0.005	129.52		
SDF-1A	rs17148125	G	A	-0.0693	0.0139	0.1412	6.69E-07	0.001	24.86		
SDF-1A	rs2013478	C	T	-0.0906	0.0193	0.6590	2.67E-06	0.004	22.04		
SDF-1A	rs2285642	C	A	0.1317	0.0281	0.4483	2.73E-06	0.009	21.97		
SDF-1A	rs34182166	C	T	0.0869	0.0183	0.9463	2.12E-06	0.001	22.55		
SDF-1A	rs34517613	A	G	0.1976	0.0337	0.8718	4.66E-09	0.009	34.38		

SDF-1A	rs3849943	A	C	0.5145	0.0672	0.7674	1.85E-14	0.095	58.62	
SDF-1A	rs538622	C	T	-0.089	0.0182	0.3877	1.06E-06	0.004	23.91	
SDF-1A	rs61527579	T	C	0.2058	0.0444	0.1789	3.63E-06	0.012	21.48	
SDF-1A	rs61880881	A	G	0.0688	0.0151	0.9423	4.92E-06	0.001	20.76	
SDF-1A	rs67710834	T	C	0.1462	0.0273	0.3907	8.59E-08	0.010	28.68	
TNF-A	rs10139154	T	C	0.0767	0.0146	0.659	1.44E-07	0.003	27.60	
TNF-A	rs10143310	C	G	0.0821	0.0161	0.2237	3.23E-07	0.002	26.00	
TNF-A	rs10463311	T	C	-0.0854	0.0156	0.2714	4.00E-08	0.003	29.97	
TNF-A	rs11195948	T	C	-0.0707	0.0144	0.6282	9.14E-07	0.002	24.11	
TNF-A	rs11786739	G	A	0.1371	0.029	0.9364	2.20E-06	0.002	22.35	
TNF-A	rs118049474	C	T	-0.1335	0.0284	0.0577	2.66E-06	0.002	22.10	
TNF-A	rs11842416	C	T	0.0814	0.0172	0.2416	2.36E-06	0.002	22.40	
TNF-A	rs12973192	G	C	0.1205	0.0153	0.6581	3.92E-15	0.007	62.03	
TNF-A	rs142321490	C	G	0.3172	0.0513	0.0169	6.15E-10	0.003	38.23	
TNF-A	rs146107389	C	T	0.3509	0.0764	0.0159	4.39E-06	0.004	21.10	
TNF-A	rs17070492	G	C	-0.1242	0.0234	0.9046	1.04E-07	0.003	28.17	99.80%
TNF-A	rs17148125	C	G	0.0851	0.0181	0.1412	2.66E-06	0.002	22.11	
TNF-A	rs2013478	T	C	0.0656	0.0144	0.6590	4.94E-06	0.002	20.75	
TNF-A	rs2285642	C	G	0.0674	0.0141	0.4483	1.80E-06	0.002	22.85	
TNF-A	rs34182166	G	A	0.1372	0.03	0.9463	4.70E-06	0.002	20.92	
TNF-A	rs34517613	T	C	-0.1057	0.0223	0.8718	2.18E-06	0.002	22.47	
TNF-A	rs3849943	T	C	-0.1764	0.0155	0.7674	3.77E-30	0.011	129.52	
TNF-A	rs538622	G	A	-0.0693	0.0139	0.3877	6.69E-07	0.002	24.86	
TNF-A	rs61527579	C	T	-0.0906	0.0193	0.1789	2.67E-06	0.002	22.04	
TNF-A	rs61880881	C	A	0.1317	0.0281	0.9423	2.73E-06	0.002	21.97	
TNF-A	rs67710834	C	G	0.0698	0.015	0.3907	3.31E-06	0.002	21.65	

TNF-A	rs72714928	C	T	0.0869	0.0183	0.1670	2.12E-06	0.002	22.55	
TNF-A	rs74654358	A	G	0.1976	0.0337	0.0318	4.66E-09	0.002	34.38	
TNF-A	rs75087725	A	C	0.5145	0.0672	0.0119	1.85E-14	0.006	58.62	
TNF-A	rs7552104	C	T	-0.089	0.0182	0.1779	1.06E-06	0.002	23.91	
TNF-A	rs79068040	T	C	0.2058	0.0444	0.9781	3.63E-06	0.002	21.48	
TNF-A	rs8125607	A	G	0.0688	0.0151	0.3221	4.92E-06	0.002	20.76	
TNF-A	rs9901522	T	C	0.1462	0.0273	0.9423	8.59E-08	0.002	28.68	
TNF-B	rs10139154	T	C	0.0767	0.0146	0.659	1.44E-07	0.003	27.60	
TNF-B	rs10463311	T	C	-0.0854	0.0156	0.2714	4.00E-08	0.003	29.97	
TNF-B	rs11195948	T	C	-0.0707	0.0144	0.6282	9.14E-07	0.002	24.11	
TNF-B	rs11786739	G	A	0.1371	0.029	0.9364	2.20E-06	0.002	22.35	
TNF-B	rs118049474	C	T	-0.1335	0.0284	0.0577	2.66E-06	0.002	22.10	
TNF-B	rs11842416	C	T	0.0814	0.0172	0.2416	2.36E-06	0.002	22.40	
TNF-B	rs2013478	T	C	0.0656	0.0144	0.6590	4.94E-06	0.002	20.75	
TNF-B	rs34517613	T	C	-0.1057	0.0223	0.8718	2.18E-06	0.002	22.47	
TNF-B	rs3849943	T	C	-0.1764	0.0155	0.7674	3.77E-30	0.011	129.52	100.00%
TNF-B	rs538622	G	A	-0.0693	0.0139	0.3877	6.69E-07	0.002	24.86	
TNF-B	rs61527579	C	T	-0.0906	0.0193	0.1789	2.67E-06	0.002	22.04	
TNF-B	rs61880881	C	A	0.1317	0.0281	0.9423	2.73E-06	0.002	21.97	
TNF-B	rs72714928	C	T	0.0869	0.0183	0.1670	2.12E-06	0.002	22.55	
TNF-B	rs7552104	C	T	-0.089	0.0182	0.1779	1.06E-06	0.002	23.91	
TNF-B	rs8125607	A	G	0.0688	0.0151	0.3221	4.92E-06	0.002	20.76	
TNF-B	rs9901522	T	C	0.1462	0.0273	0.9423	8.59E-08	0.002	28.68	
TRAIL	rs10139154	T	C	0.0767	0.0146	0.659	1.44E-07	0.003	27.60	
TRAIL	rs10463311	T	C	-0.0854	0.0156	0.2714	4.00E-08	0.003	29.97	72.10%
TRAIL	rs11195948	T	C	-0.0707	0.0144	0.6282	9.14E-07	0.002	24.11	

TRAIL	rs11786739	G	A	0.1371	0.029	0.9364	2.20E-06	0.002	22.35	
TRAIL	rs118049474	C	T	-0.1335	0.0284	0.0577	2.66E-06	0.002	22.10	
TRAIL	rs11842416	C	T	0.0814	0.0172	0.2416	2.36E-06	0.002	22.40	
TRAIL	rs146107389	C	T	0.3509	0.0764	0.0159	4.39E-06	0.004	21.10	
TRAIL	rs2013478	T	C	0.0656	0.0144	0.6590	4.94E-06	0.002	20.75	
TRAIL	rs34182166	G	A	0.1372	0.03	0.9463	4.70E-06	0.002	20.92	
TRAIL	rs34517613	T	C	-0.1057	0.0223	0.8718	2.18E-06	0.002	22.47	
TRAIL	rs3849943	T	C	-0.1764	0.0155	0.7674	3.77E-30	0.011	129.52	
TRAIL	rs538622	G	A	-0.0693	0.0139	0.3877	6.69E-07	0.002	24.86	
TRAIL	rs61527579	C	T	-0.0906	0.0193	0.1789	2.67E-06	0.002	22.04	
TRAIL	rs61880881	C	A	0.1317	0.0281	0.9423	2.73E-06	0.002	21.97	
TRAIL	rs72714928	C	T	0.0869	0.0183	0.1670	2.12E-06	0.002	22.55	
TRAIL	rs74654358	A	G	0.1976	0.0337	0.0318	4.66E-09	0.002	34.38	
TRAIL	rs75087725	A	C	0.5145	0.0672	0.0119	1.85E-14	0.006	58.62	
TRAIL	rs7552104	C	T	-0.089	0.0182	0.1779	1.06E-06	0.002	23.91	
TRAIL	rs79068040	T	C	0.2058	0.0444	0.9781	3.63E-06	0.002	21.48	
TRAIL	rs8125607	A	G	0.0688	0.0151	0.3221	4.92E-06	0.002	20.76	
TRAIL	rs9901522	T	C	0.1462	0.0273	0.9423	8.59E-08	0.002	28.68	
VEGF	rs10139154	T	C	0.0767	0.0146	0.659	1.44E-07	0.003	27.60	
VEGF	rs10463311	T	C	-0.0854	0.0156	0.2714	4.00E-08	0.003	29.97	
VEGF	rs11195948	T	C	-0.0707	0.0144	0.6282	9.14E-07	0.002	24.11	
VEGF	rs11786739	G	A	0.1371	0.029	0.9364	2.20E-06	0.002	22.35	
VEGF	rs118049474	C	T	-0.1335	0.0284	0.0577	2.66E-06	0.002	22.10	99.70%
VEGF	rs11842416	C	T	0.0814	0.0172	0.2416	2.36E-06	0.002	22.40	
VEGF	rs146107389	C	T	0.3509	0.0764	0.0159	4.39E-06	0.004	21.10	
VEGF	rs2013478	T	C	0.0656	0.0144	0.6590	4.94E-06	0.002	20.75	

VEGF	rs34182166	G	A	0.1372	0.03	0.9463	4.70E-06	0.002	20.92
VEGF	rs34517613	T	C	-0.1057	0.0223	0.8718	2.18E-06	0.002	22.47
VEGF	rs3849943	T	C	-0.1764	0.0155	0.7674	3.77E-30	0.011	129.52
VEGF	rs538622	G	A	-0.0693	0.0139	0.3877	6.69E-07	0.002	24.86
VEGF	rs61527579	C	T	-0.0906	0.0193	0.1789	2.67E-06	0.002	22.04
VEGF	rs61880881	C	A	0.1317	0.0281	0.9423	2.73E-06	0.002	21.97
VEGF	rs72714928	C	T	0.0869	0.0183	0.1670	2.12E-06	0.002	22.55
VEGF	rs74654358	A	G	0.1976	0.0337	0.0318	4.66E-09	0.002	34.38
VEGF	rs75087725	A	C	0.5145	0.0672	0.0119	1.85E-14	0.006	58.62
VEGF	rs7552104	C	T	-0.089	0.0182	0.1779	1.06E-06	0.002	23.91
VEGF	rs79068040	T	C	0.2058	0.0444	0.9781	3.63E-06	0.002	21.48
VEGF	rs8125607	A	G	0.0688	0.0151	0.3221	4.92E-06	0.002	20.76
VEGF	rs9901522	T	C	0.1462	0.0273	0.9423	8.59E-08	0.002	28.68

Supplementary Table 7. Detailed information for circulating cytokines-associated SNPs with risk of Alzheimer's disease (AD).

Exposure	SNP	Effect Allele	Other Allele	Beta	Se	EAF	*P-value*	*R2*	*F*	Power
BNGF	rs7970581	T	G	0.1358	0.028	0.7644	1.23E-06	0.007	23.52	5.00%
BNGF	rs73472576	T	C	-0.1146	0.0251	0.6044	4.98E-06	0.006	20.85	
BNGF	rs71641308	T	C	0.1969	0.0429	0.9463	4.44E-06	0.004	21.07	
BNGF	rs28637706	T	G	-0.1554	0.0261	0.6829	2.62E-09	0.010	35.45	
CTACK	rs76395525	A	G	0.5193	0.1081	0.0149	1.56E-06	0.008	23.08	5.00%
CTACK	rs7333764	T	C	0.2811	0.0591	0.9732	1.97E-06	0.004	22.62	
CTACK	rs72729450	T	C	-0.5123	0.1094	0.9861	2.83E-06	0.007	21.93	
CTACK	rs60247384	T	C	0.1128	0.0245	0.7058	4.14E-06	0.005	21.20	
CTACK	rs57789542	T	C	-0.7687	0.1659	0.0109	3.60E-06	0.013	21.47	
CTACK	rs57338032	A	G	0.1443	0.0316	0.8201	4.96E-06	0.006	20.85	
CTACK	rs55764737	T	C	0.5424	0.0967	0.0229	2.03E-08	0.013	31.46	
CTACK	rs2070074	A	G	0.4401	0.0372	0.8976	2.71E-32	0.036	139.96	
CTACK	rs135567	A	G	0.1664	0.0265	0.2614	3.40E-10	0.011	39.43	
CTACK	rs118084576	A	G	0.5675	0.1226	0.0139	3.68E-06	0.009	21.43	
CTACK	rs117385454	A	G	-0.1968	0.0413	0.9443	1.89E-06	0.004	22.71	
CTACK	rs116303454	A	G	0.3754	0.081	0.0288	3.58E-06	0.008	21.48	
EOTAXIN	rs9317045	A	C	0.1172	0.0236	0.8539	6.83E-07	0.003	24.66	5.00%
EOTAXIN	rs80341932	A	G	0.101	0.0204	0.7078	7.38E-07	0.004	24.51	
EOTAXIN	rs79722574	T	C	-0.1092	0.0227	0.84	1.50E-06	0.003	23.14	
EOTAXIN	rs75426604	A	C	-0.1371	0.0291	0.1312	2.46E-06	0.004	22.20	
EOTAXIN	rs745331	A	G	-0.0821	0.0176	0.3091	3.09E-06	0.003	21.76	
EOTAXIN	rs59808887	T	C	-0.1698	0.0356	0.9225	1.85E-06	0.004	22.75	
EOTAXIN	rs5754733	A	C	-0.105	0.0213	0.7883	8.24E-07	0.004	24.30	
EOTAXIN	rs2228467	T	C	-0.4154	0.0291	0.0696	3.14E-46	0.022	203.77	

EOTAXIN	rs2211994	T	C	0.0876	0.0177	0.7495	7.45E-07	0.003	24.49	
EOTAXIN	rs2027855	T	C	0.0743	0.0162	0.329	4.51E-06	0.002	21.04	
EOTAXIN	rs2024050	A	G	0.164	0.0302	0.8996	5.62E-08	0.005	29.49	
EOTAXIN	rs147287945	A	G	-0.1512	0.0313	0.0726	1.36E-06	0.003	23.34	
EOTAXIN	rs12075	A	G	0.1692	0.0155	0.6024	9.65E-28	0.014	119.16	
EOTAXIN	rs112347425	T	C	0.1595	0.0276	0.8926	7.52E-09	0.005	33.40	
EOTAXIN	rs11087905	A	C	0.0954	0.0188	0.3419	3.89E-07	0.004	25.75	
FGF	rs9903590	T	C	0.1281	0.0267	0.0974	1.60E-06	0.003	23.02	
FGF	rs78873483	A	G	0.1286	0.0282	0.0944	5.11E-06	0.003	20.80	
FGF	rs75168112	T	C	-0.1024	0.0214	0.1779	1.71E-06	0.003	22.90	5.00%
FGF	rs145577605	A	G	0.2043	0.0427	0.0109	1.71E-06	0.001	22.89	
FGF	rs13412535	A	G	-0.1129	0.0224	0.2256	4.65E-07	0.004	25.40	
FGF	rs116745220	A	G	-0.6176	0.1324	0.9801	3.09E-06	0.015	21.76	
G-CSF	rs77318030	T	C	-0.2031	0.0427	0.0517	1.97E-06	0.004	22.62	
G-CSF	rs76287671	T	C	0.0894	0.0189	0.8091	2.24E-06	0.002	22.37	
G-CSF	rs74148555	T	C	-0.3771	0.0753	0.9473	5.50E-07	0.014	25.08	
G-CSF	rs586313	T	C	-0.0883	0.0187	0.2286	2.34E-06	0.003	22.30	5.00%
G-CSF	rs2671444	A	G	-0.0776	0.0166	0.3648	2.94E-06	0.003	21.85	
G-CSF	rs11903143	A	G	0.0889	0.0175	0.7425	3.77E-07	0.003	25.81	
G-CSF	rs117261691	T	C	0.1318	0.0288	0.9652	4.73E-06	0.001	20.94	
G-CSF	rs116745220	A	G	-0.6789	0.1359	0.9801	5.87E-07	0.018	24.96	
GROA	rs78653452	T	G	-0.7395	0.1559	0.9881	2.10E-06	0.013	22.50	
GROA	rs62024303	A	G	-0.3013	0.066	0.9543	4.99E-06	0.008	20.84	
GROA	rs493091	T	C	0.384	0.0279	0.2346	4.23E-43	0.053	189.43	5.00%
GROA	rs188345231	T	C	0.6177	0.1322	0.9881	2.98E-06	0.009	21.83	
GROA	rs140734053	A	G	0.7333	0.1545	0.0249	2.07E-06	0.026	22.53	

GROA	rs12075	A	G	0.3724	0.0236	0.6024	4.29E-56	0.066	249.00	
GROA	rs118158560	A	G	0.2761	0.0592	0.0626	3.10E-06	0.009	21.75	
GROA	rs114991247	T	C	-0.2202	0.0463	0.0596	1.98E-06	0.005	22.62	
GROA	rs1113500	T	G	0.1162	0.0243	0.3698	1.74E-06	0.006	22.87	
HGF	rs5745687	T	C	-0.3008	0.0404	0.0567	9.65E-14	0.010	55.44	
HGF	rs57146176	A	G	-0.0987	0.0208	0.9404	2.08E-06	0.001	22.52	
HGF	rs4245058	T	C	-0.1552	0.0331	0.9066	2.75E-06	0.004	21.99	
HGF	rs3748034	T	G	0.1529	0.0233	0.1362	5.30E-11	0.006	43.06	5.00%
HGF	rs2003620	T	C	0.2277	0.0487	0.0606	2.93E-06	0.006	21.86	
HGF	rs13412535	A	G	-0.1043	0.0213	0.2256	9.75E-07	0.004	23.98	
HGF	rs11060254	A	G	-0.0765	0.0166	0.3429	4.06E-06	0.003	21.24	
IFN-G	rs78296352	T	G	0.3419	0.065	0.9712	1.44E-07	0.007	27.67	
IFN-G	rs74148555	T	C	-0.3771	0.077	0.9473	9.71E-07	0.014	23.98	
IFN-G	rs60059008	A	G	0.0852	0.0176	0.6670	1.29E-06	0.003	23.43	
IFN-G	rs2073438	A	G	0.092	0.0188	0.2714	9.90E-07	0.003	23.95	
IFN-G	rs12420286	T	C	0.2357	0.05	0.0288	2.43E-06	0.003	22.22	7.00%
IFN-G	rs11843756	T	G	0.1812	0.0391	0.0288	3.58E-06	0.002	21.48	
IFN-G	rs115729819	A	G	0.2511	0.0514	0.9791	1.03E-06	0.003	23.87	
IFN-G	rs113600793	A	C	0.1871	0.0371	0.0398	4.58E-07	0.003	25.43	
IFN-G	rs10760686	T	C	0.0795	0.0168	0.6879	2.22E-06	0.003	22.39	
IL-10	rs7088799	T	G	-0.0815	0.0166	0.4344	9.12E-07	0.003	24.10	
IL-10	rs6921438	A	G	-0.2876	0.0166	0.4672	3.03E-67	0.041	300.17	
IL-10	rs6799107	T	C	-0.095	0.0206	0.2137	3.99E-06	0.003	21.27	7.00%
IL-10	rs6117725	T	C	0.0979	0.0202	0.2634	1.26E-06	0.004	23.49	
IL-10	rs41282660	A	G	-0.1169	0.0254	0.8678	4.18E-06	0.003	21.18	
IL-10	rs383684	A	G	0.092	0.0197	0.1183	3.01E-06	0.002	21.81	

IL-10	rs339203	T	C	0.0954	0.0203	0.9602	2.61E-06	0.001	22.09	
IL-10	rs3025021	T	C	0.0913	0.0194	0.6322	2.52E-06	0.004	22.15	
IL-10	rs282258	T	C	0.0993	0.0162	0.5746	8.81E-10	0.005	37.57	
IL-10	rs2086656	T	C	-0.08	0.017	0.3052	2.53E-06	0.003	22.15	
IL-10	rs1530455	T	C	0.082	0.0174	0.5915	2.45E-06	0.003	22.21	
IL-10	rs10493718	A	C	-0.1081	0.0222	0.2913	1.12E-06	0.005	23.71	
IL-10	rs10457128	A	G	-0.0854	0.0172	0.6392	6.87E-07	0.003	24.65	
IL-12	rs9472183	A	G	-0.1006	0.0157	0.5109	1.48E-10	0.005	41.06	
IL-12	rs782111	A	C	-0.0765	0.0156	0.5547	9.40E-07	0.003	24.05	
IL-12	rs72831623	A	G	0.1929	0.0367	0.0527	1.47E-07	0.004	27.63	
IL-12	rs71361173	T	G	0.1105	0.0238	0.1133	3.44E-06	0.002	21.56	
IL-12	rs6532374	T	C	-0.1033	0.0226	0.1819	4.86E-06	0.003	20.89	
IL-12	rs41282644	A	G	0.1401	0.0303	0.0636	3.77E-06	0.002	21.38	5.00%
IL-12	rs34291323	T	C	0.0954	0.0198	0.3787	1.45E-06	0.004	23.21	
IL-12	rs282258	T	C	0.0726	0.0156	0.5746	3.26E-06	0.003	21.66	
IL-12	rs273702	A	G	-0.127	0.027	0.1233	2.55E-06	0.003	22.12	
IL-12	rs2123852	T	C	0.0942	0.0204	0.8241	3.88E-06	0.003	21.32	
IL-12	rs13209117	A	G	0.0981	0.0186	0.2843	1.33E-07	0.004	27.82	
IL-12	rs13206436	A	G	-0.3739	0.0158	0.4493	8.36E-124	0.069	560.01	
IL-13	rs9472168	A	G	0.4211	0.0246	0.5507	1.09E-65	0.088	293.02	
IL-13	rs77955971	A	C	0.4408	0.0868	0.0239	3.81E-07	0.009	25.79	
IL-13	rs73192842	A	G	0.1472	0.0299	0.2107	8.52E-07	0.007	24.24	
IL-13	rs27949	T	C	-0.1144	0.025	0.6312	4.74E-06	0.006	20.94	7.00%
IL-13	rs139083458	T	C	0.9995	0.211	0.9841	2.17E-06	0.031	22.44	
IL-13	rs138854806	A	G	-0.4204	0.0839	0.0119	5.42E-07	0.004	25.11	
IL-13	rs12623722	A	G	-0.1189	0.0257	0.2823	3.72E-06	0.006	21.40	

IL-13	rs117795020	A	G	-0.3584	0.0716	0.0179	5.57E-07	0.005	25.06	
IL-13	rs10995615	T	C	-0.1591	0.0341	0.1521	3.08E-06	0.007	21.77	
IL-16	rs9706053	T	C	0.4412	0.0928	0.9841	1.99E-06	0.006	22.60	
IL-16	rs4778636	A	G	-0.7286	0.063	0.0746	6.19E-31	0.073	133.75	
IL-16	rs4253283	T	C	0.1506	0.026	0.6998	6.94E-09	0.010	33.55	
IL-16	rs1801020	A	G	0.1678	0.0271	0.7753	5.95E-10	0.010	38.34	
IL-16	rs144691581	A	G	0.4929	0.0958	0.0149	2.67E-07	0.007	26.47	6.00%
IL-16	rs1255143	T	C	0.1387	0.0241	0.4344	8.65E-09	0.009	33.12	
IL-16	rs117916513	A	G	-0.4713	0.0982	0.0209	1.59E-06	0.009	23.03	
IL-16	rs117217798	T	C	-0.2064	0.044	0.9314	2.72E-06	0.005	22.00	
IL-16	rs116135478	A	G	0.8296	0.1637	0.9821	4.02E-07	0.024	25.68	
IL-17	rs78296352	T	G	0.2949	0.0645	0.9712	4.83E-06	0.005	20.90	
IL-17	rs184080173	T	C	0.236	0.0471	0.0626	5.43E-07	0.007	25.11	
IL-17	rs17282552	T	C	-0.2026	0.0403	0.0268	4.97E-07	0.002	25.27	
IL-17	rs1530455	T	C	0.1088	0.0173	0.5915	3.19E-10	0.006	39.55	5.00%
IL-17	rs12735700	T	G	-0.0943	0.0206	0.7515	4.70E-06	0.003	20.96	
IL-17	rs117556572	T	C	-0.5256	0.1097	0.9891	1.66E-06	0.006	22.96	
IL-17	rs113098509	A	G	-0.1553	0.0337	0.9235	4.06E-06	0.003	21.24	
IL-18	rs78716465	A	G	0.3173	0.0679	0.0378	2.97E-06	0.007	21.84	
IL-18	rs78623212	T	C	0.8322	0.1676	0.9761	6.86E-07	0.032	24.66	
IL-18	rs71478720	T	C	-0.26	0.0273	0.7823	1.67E-21	0.023	90.70	
IL-18	rs610473	A	G	0.1274	0.0242	0.6501	1.41E-07	0.007	27.71	5.00%
IL-18	rs4482818	A	G	0.1233	0.0243	0.6412	3.89E-07	0.007	25.75	
IL-18	rs385076	T	C	-0.2472	0.0247	0.6471	1.40E-23	0.028	100.16	
IL-18	rs1979967	T	C	0.14	0.0285	0.7406	9.00E-07	0.008	24.13	
IL-18	rs17229943	A	C	-0.3076	0.0463	0.9503	3.06E-11	0.009	44.14	

IL-18	rs117266781	T	C	0.7051	0.1436	0.9891	9.10E-07	0.011	24.11	
IL-18	rs116383510	A	C	-0.5412	0.1052	0.9801	2.68E-07	0.011	26.47	
IL-18	rs10414552	T	C	-0.1817	0.0347	0.8787	1.64E-07	0.007	27.42	
IL-1B	rs62015704	A	G	0.1786	0.0372	0.8787	1.58E-06	0.007	23.05	
IL-1B	rs61335305	A	C	0.4333	0.0928	0.0109	3.02E-06	0.004	21.80	
IL-1B	rs143319329	T	C	0.4357	0.093	0.9761	2.80E-06	0.009	21.95	6.00%
IL-1B	rs115242021	A	C	0.2795	0.0553	0.0815	4.32E-07	0.012	25.55	
IL-1RA	rs6699436	A	G	-0.1858	0.0404	0.1431	4.25E-06	0.008	21.15	
IL-1RA	rs61335305	A	C	0.4315	0.0904	0.0109	1.81E-06	0.004	22.78	
IL-1RA	rs56134659	A	G	-0.1109	0.0236	0.5258	2.61E-06	0.006	22.08	
IL-1RA	rs4441609	T	C	0.1056	0.0231	0.6302	4.84E-06	0.005	20.90	
IL-1RA	rs3876037	A	G	0.1234	0.027	0.4463	4.87E-06	0.008	20.89	5.00%
IL-1RA	rs187166731	T	C	-0.2424	0.0504	0.9881	1.51E-06	0.001	23.13	
IL-1RA	rs11627423	A	C	0.1178	0.0246	0.6103	1.68E-06	0.007	22.93	
IL-1RA	rs1054402	T	C	0.1325	0.0269	0.7336	8.41E-07	0.007	24.26	
IL-2	rs7615304	A	G	-0.1139	0.024	0.3489	2.08E-06	0.006	22.52	
IL-2	rs62124990	T	G	-0.7013	0.149	0.9841	2.52E-06	0.015	22.15	
IL-2	rs61335305	A	C	0.4439	0.0913	0.0109	1.16E-06	0.004	23.64	
IL-2	rs4634519	A	G	-0.1249	0.0268	0.6879	3.16E-06	0.007	21.72	
IL-2	rs2690020	A	G	0.1158	0.0245	0.5547	2.28E-06	0.007	22.34	11.00%
IL-2	rs170117	T	C	-0.1637	0.0347	0.8797	2.39E-06	0.006	22.26	
IL-2	rs16836080	A	G	0.1158	0.0253	0.2922	4.72E-06	0.006	20.95	
IL-2	rs13412535	A	G	0.174	0.0331	0.2256	1.47E-07	0.011	27.63	
IL-2RA	rs56213152	T	C	0.1269	0.0271	0.2604	2.83E-06	0.006	21.93	
IL-2RA	rs4733117	A	C	0.1439	0.0291	0.8360	7.61E-07	0.006	24.45	5.00%
IL-2RA	rs185231391	T	C	0.8568	0.1803	0.0149	2.01E-06	0.022	22.58	

IL-2RA	rs12799226	T	C	-0.1285	0.0277	0.8091	3.50E-06	0.005	21.52	
IL-2RA	rs12722497	A	C	0.6287	0.0482	0.8559	6.91E-39	0.097	170.13	
IL-2RA	rs117244812	A	G	-0.7187	0.1493	0.0139	1.48E-06	0.014	23.17	
IL-2RA	rs11241559	T	G	-0.124	0.0264	0.7813	2.64E-06	0.005	22.06	
IL-4	rs9941733	A	G	0.1156	0.0229	0.825	4.46E-07	0.004	25.48	
IL-4	rs9508291	T	C	-0.168	0.0358	0.0646	2.70E-06	0.003	22.02	
IL-4	rs79597994	T	C	-0.5855	0.1271	0.9722	4.09E-06	0.019	21.22	
IL-4	rs7613691	A	G	0.1787	0.0382	0.9473	2.90E-06	0.003	21.88	
IL-4	rs73023729	A	G	-0.1796	0.0365	0.0229	8.63E-07	0.001	24.21	
IL-4	rs6765768	A	G	0.0796	0.0167	0.6083	1.87E-06	0.003	22.72	5.00%
IL-4	rs2708550	T	C	-0.0764	0.0165	0.7803	3.65E-06	0.002	21.44	
IL-4	rs2073438	A	G	0.0847	0.0183	0.2714	3.68E-06	0.003	21.42	
IL-4	rs17713451	A	G	0.1255	0.0252	0.1650	6.35E-07	0.004	24.80	
IL-4	rs12238729	T	C	0.5271	0.1096	0.9881	1.51E-06	0.007	23.13	
IL-4	rs116705532	T	G	-0.4675	0.0978	0.0169	1.75E-06	0.007	22.85	
IL-4	rs10512267	T	C	-0.0824	0.016	0.3300	2.60E-07	0.003	26.52	
IL-5	rs74811276	A	G	0.217	0.0471	0.0686	4.08E-06	0.006	21.23	
IL-5	rs72831687	A	G	-0.5337	0.1104	0.0169	1.34E-06	0.009	23.37	
IL-5	rs6737109	T	C	0.1135	0.0246	0.4254	3.95E-06	0.006	21.29	
IL-5	rs4320361	T	G	-0.1553	0.025	0.5477	5.23E-10	0.012	38.59	18.00%
IL-5	rs28793375	T	C	0.1697	0.0362	0.8330	2.76E-06	0.008	21.98	
IL-5	rs148634917	A	G	-0.517	0.1087	0.9811	1.97E-06	0.010	22.62	
IL-5	rs11680908	A	G	0.2593	0.0552	0.9453	2.63E-06	0.007	22.07	
IL-6	rs76856708	T	C	0.336	0.0697	0.0378	1.43E-06	0.008	23.24	
IL-6	rs73273528	T	C	0.268	0.0553	0.9662	1.26E-06	0.005	23.49	5.00%
IL-6	rs72831623	A	G	0.197	0.0369	0.0527	9.36E-08	0.004	28.50	

IL-6	rs4684700	T	C	-0.0747	0.0162	0.5139	4.01E-06	0.003	21.26	
IL-6	rs2404476	A	G	0.0734	0.0156	0.4722	2.54E-06	0.003	22.14	
IL-6	rs13412535	A	G	-0.1186	0.0214	0.2256	2.99E-08	0.005	30.71	
IL-6	rs1333040	T	C	0.0747	0.0157	0.4264	1.96E-06	0.003	22.64	
IL-6	rs113098456	A	G	-0.1553	0.0339	0.0765	4.62E-06	0.003	20.99	
IL-6	rs10817609	T	C	0.0845	0.0175	0.2157	1.38E-06	0.002	23.32	
IL-7	rs78346957	A	G	0.4632	0.1008	0.0229	4.32E-06	0.010	21.12	
IL-7	rs77981494	T	C	-0.5201	0.1055	0.0159	8.23E-07	0.008	24.30	
IL-7	rs6921438	A	G	-0.3204	0.0246	0.4672	8.89E-39	0.051	169.63	
IL-7	rs62006410	T	C	-0.1492	0.0302	0.7853	7.80E-07	0.008	24.41	
IL-7	rs28793375	T	C	0.1644	0.036	0.8330	4.96E-06	0.008	20.85	
IL-7	rs1958987	T	C	0.1261	0.0263	0.6998	1.63E-06	0.007	22.99	5.00%
IL-7	rs17091524	T	C	0.5092	0.1015	0.0427	5.26E-07	0.021	25.17	
IL-7	rs141425475	T	C	-0.4801	0.1018	0.0288	2.40E-06	0.013	22.24	
IL-7	rs117509142	T	C	-0.3213	0.0684	0.0567	2.64E-06	0.011	22.07	
IL-7	rs115215018	T	C	0.5985	0.1308	0.9881	4.75E-06	0.008	20.94	
IL-7	rs10196226	A	G	0.1538	0.0327	0.1471	2.56E-06	0.006	22.12	
IL-8	rs75840288	A	C	0.5125	0.1121	0.9702	4.84E-06	0.015	20.90	
IL-8	rs3786107	A	G	0.2463	0.0517	0.0596	1.90E-06	0.007	22.70	
IL-8	rs2673604	A	C	-0.118	0.0254	0.2992	3.39E-06	0.006	21.58	
IL-8	rs183628733	T	C	0.6547	0.1417	0.0219	3.83E-06	0.018	21.35	6.00%
IL-8	rs141926526	A	C	-0.6221	0.1308	0.9523	1.97E-06	0.035	22.62	
IL-8	rs12438669	A	C	-0.1182	0.0252	0.6571	2.73E-06	0.006	22.00	
IL-8	rs12075	A	G	0.1148	0.0235	0.6024	1.03E-06	0.006	23.86	
IL-8	rs116726256	T	C	-0.2247	0.0489	0.0318	4.33E-06	0.003	21.11	
IL-9	rs76963786	T	C	-0.2856	0.0556	0.9135	2.80E-07	0.013	26.39	9.00%

IL-9	rs73443903	A	C	0.2162	0.046	0.1004	2.60E-06	0.008	22.09	
IL-9	rs4880409	T	C	-0.3552	0.0716	0.0278	7.02E-07	0.007	24.61	
IL-9	rs41294750	T	C	0.3442	0.0736	0.9722	2.92E-06	0.006	21.87	
IP-10	rs9450351	T	C	-0.2651	0.0488	0.0616	5.56E-08	0.008	29.51	
IP-10	rs7645625	T	G	-0.1116	0.0236	0.4324	2.26E-06	0.006	22.36	
IP-10	rs75970138	A	G	-0.4845	0.1037	0.0129	2.98E-06	0.006	21.83	
IP-10	rs6707974	A	G	0.1574	0.0337	0.8380	3.00E-06	0.007	21.81	
IP-10	rs4862110	T	C	-0.1453	0.0318	0.1869	4.90E-06	0.006	20.88	8.00%
IP-10	rs397816	T	C	0.1211	0.0248	0.5736	1.04E-06	0.007	23.84	
IP-10	rs34383175	T	C	-0.3196	0.0653	0.9732	9.86E-07	0.005	23.95	
IP-10	rs143799975	A	G	-0.7551	0.1638	0.9871	4.03E-06	0.015	21.25	
IP-10	rs113831257	A	G	0.3639	0.0641	0.0417	1.37E-08	0.011	32.23	
MCP-1-MCAF	rs9317045	A	C	0.1157	0.0235	0.8539	8.51E-07	0.003	24.24	
MCP-1-MCAF	rs7632755	A	G	0.2984	0.0315	0.0805	2.72E-21	0.013	89.74	
MCP-1-MCAF	rs7517040	A	G	-0.097	0.019	0.2823	3.30E-07	0.004	26.06	
MCP-1-MCAF	rs7197349	A	G	0.0971	0.0206	0.8628	2.43E-06	0.002	22.22	
MCP-1-MCAF	rs7019112	T	G	-0.2206	0.0468	0.0119	2.43E-06	0.001	22.22	
MCP-1-MCAF	rs56212190	T	C	0.1799	0.0372	0.9453	1.32E-06	0.003	23.39	
MCP-1-MCAF	rs2288370	T	C	-0.1036	0.0162	0.3956	1.60E-10	0.005	40.90	
MCP-1-MCAF	rs2036297	A	G	0.1182	0.016	0.3429	1.50E-13	0.006	54.58	5.00%
MCP-1-MCAF	rs146522229	T	C	-0.5942	0.1161	0.9761	3.09E-07	0.016	26.19	
MCP-1-MCAF	rs143815843	A	G	-0.2049	0.0447	0.0109	4.56E-06	0.001	21.01	
MCP-1-MCAF	rs12075	A	G	0.2186	0.0154	0.6024	9.87E-46	0.023	201.49	
MCP-1-MCAF	rs111995966	T	G	0.1428	0.0309	0.0258	3.81E-06	0.001	21.36	
MCP-1-MCAF	rs11118557	A	G	0.148	0.0319	0.9264	3.49E-06	0.003	21.52	
MCP-1-MCAF	rs10744620	T	C	0.0783	0.0161	0.6392	1.15E-06	0.003	23.65	

MCP-3	rs62492260	T	G	-0.2802	0.0578	0.8509	1.25E-06	0.020	23.50	
MCP-3	rs3129806	T	C	-0.1975	0.0433	0.6322	5.09E-06	0.018	20.80	
MCP-3	rs2838065	A	G	-0.221	0.0479	0.1968	3.95E-06	0.015	21.29	7.00%
MCP-3	rs117286643	A	G	0.6934	0.1474	0.0109	2.55E-06	0.010	22.13	
MCP-3	rs10892381	T	C	0.2432	0.0473	0.3002	2.72E-07	0.025	26.44	
M-CSF	rs9387100	T	C	-0.135	0.029	0.6730	3.24E-06	0.008	21.67	
M-CSF	rs78296352	T	G	0.522	0.111	0.9712	2.57E-06	0.015	22.12	
M-CSF	rs72723242	T	G	-0.4969	0.1083	0.9374	4.47E-06	0.029	21.05	
M-CSF	rs62294910	A	G	0.3472	0.0687	0.0527	4.33E-07	0.012	25.54	
M-CSF	rs56367447	T	C	-0.4878	0.0876	0.9662	2.57E-08	0.016	31.01	5.00%
M-CSF	rs34089869	T	C	0.2194	0.0462	0.8976	2.05E-06	0.009	22.55	
M-CSF	rs12962919	T	C	0.3025	0.0659	0.9066	4.43E-06	0.015	21.07	
M-CSF	rs116274860	T	G	0.8262	0.1739	0.0159	2.02E-06	0.021	22.57	
MIF	rs78098071	T	C	-0.4583	0.0915	0.0189	5.48E-07	0.008	25.09	
MIF	rs3814097	A	G	-0.1163	0.0251	0.4751	3.60E-06	0.007	21.47	
MIF	rs35890933	T	G	0.1676	0.0365	0.2008	4.39E-06	0.009	21.08	
MIF	rs13142904	T	C	-0.2232	0.0425	0.9314	1.51E-07	0.006	27.58	6.00%
MIF	rs12594190	A	G	0.1321	0.0266	0.7535	6.83E-07	0.006	24.66	
MIF	rs113218956	A	G	-0.8789	0.1876	0.0109	2.80E-06	0.017	21.95	
MIG	rs816960	T	C	-0.1179	0.0242	0.2594	1.11E-06	0.005	23.74	
MIG	rs77086208	T	C	0.327	0.0694	0.9811	2.46E-06	0.004	22.20	
MIG	rs6679677	A	C	0.1628	0.0327	0.0915	6.40E-07	0.004	24.79	
MIG	rs1796086	T	C	-0.2172	0.04	0.0944	5.64E-08	0.008	29.48	5.00%
MIG	rs139010077	T	C	0.4337	0.0943	0.9891	4.24E-06	0.004	21.15	
MIG	rs117831247	T	C	-0.8819	0.173	0.9811	3.44E-07	0.029	25.99	
MIG	rs112861654	A	G	-0.2682	0.0527	0.9135	3.60E-07	0.011	25.90	

MIG	rs11177248	A	G	0.3157	0.0667	0.0606	2.21E-06	0.011	22.40	
MIG	rs111607343	A	G	-0.5235	0.1119	0.0378	2.89E-06	0.020	21.89	
MIP-1A	rs60198979	A	G	-0.2154	0.0455	0.0865	2.20E-06	0.007	22.41	
MIP-1A	rs57786342	A	G	0.139	0.0283	0.2157	9.03E-07	0.007	24.12	9.00%
MIP-1A	rs184154340	A	G	0.3251	0.0689	0.0408	2.38E-06	0.008	22.26	
MIP-1A	rs12690897	A	G	0.1215	0.026	0.2813	2.97E-06	0.006	21.84	
MIP-1B	rs9793308	A	G	0.0842	0.0177	0.6282	1.96E-06	0.003	22.63	
MIP-1B	rs77668982	A	G	0.3274	0.0389	0.0179	3.88E-17	0.004	70.84	
MIP-1B	rs76776296	A	G	0.313	0.0598	0.9632	1.66E-07	0.007	27.40	
MIP-1B	rs76582507	A	G	0.3259	0.0676	0.0268	1.43E-06	0.006	23.24	
MIP-1B	rs72799710	T	C	-0.1037	0.0217	0.8340	1.76E-06	0.003	22.84	
MIP-1B	rs72791296	T	C	0.2364	0.0466	0.9414	3.92E-07	0.006	25.73	
MIP-1B	rs6908843	A	G	0.0997	0.0209	0.1809	1.84E-06	0.003	22.76	
MIP-1B	rs5743614	T	C	0.1115	0.0232	0.2744	1.54E-06	0.005	23.10	5.00%
MIP-1B	rs281749	T	C	0.0795	0.0171	0.2913	3.33E-06	0.003	21.61	
MIP-1B	rs17138331	A	G	-0.1434	0.0295	0.9056	1.17E-06	0.004	23.63	
MIP-1B	rs1564708	T	C	-0.1697	0.0187	0.7296	1.14E-19	0.011	82.35	
MIP-1B	rs117657747	A	G	0.2089	0.0453	0.0656	4.00E-06	0.005	21.27	
MIP-1B	rs117453826	A	G	-0.5907	0.0591	0.9841	1.60E-23	0.011	99.90	
MIP-1B	rs116237296	A	G	0.5284	0.1115	0.0159	2.15E-06	0.009	22.46	
MIP-1B	rs113877493	T	C	-0.607	0.0217	0.8777	3.53E-172	0.079	782.45	
MIP-1B	rs113010081	T	C	-0.5799	0.0236	0.1083	2.52E-133	0.065	603.78	
PDGF-BB	rs9941733	A	G	0.1165	0.0227	0.825	2.86E-07	0.004	26.34	
PDGF-BB	rs73162807	A	C	-0.2313	0.0499	0.0129	3.56E-06	0.001	21.49	5.00%
PDGF-BB	rs72777070	T	G	-0.1048	0.02	0.1998	1.61E-07	0.004	27.46	
PDGF-BB	rs55680718	T	C	-0.1359	0.0245	0.8628	2.91E-08	0.004	30.77	

PDGF-BB	rs4965869	T	C	0.1843	0.0181	0.7237	2.38E-24	0.014	103.68	
PDGF-BB	rs35859699	A	G	-0.3854	0.0838	0.0229	4.24E-06	0.007	21.15	
PDGF-BB	rs2324229	T	C	0.0884	0.0161	0.3936	4.00E-08	0.004	30.15	
PDGF-BB	rs13412535	A	G	0.3317	0.0214	0.2256	3.47E-54	0.038	240.25	
PDGF-BB	rs12289510	A	G	-0.0772	0.0158	0.4672	1.03E-06	0.003	23.87	
PDGF-BB	rs11766649	A	G	0.0902	0.0196	0.7386	4.18E-06	0.003	21.18	
PDGF-BB	rs116445074	T	G	0.2869	0.0587	0.9841	1.02E-06	0.003	23.89	
RANTES	rs9675798	T	G	-0.2583	0.0552	0.0378	2.88E-06	0.005	21.90	
RANTES	rs74472919	T	C	0.3547	0.06	0.9811	3.39E-09	0.005	34.95	
RANTES	rs72793342	A	G	-0.1505	0.0307	0.2078	9.47E-07	0.007	24.03	
RANTES	rs2731672	T	C	-0.1242	0.0272	0.2266	4.97E-06	0.005	20.85	5.00%
RANTES	rs2251660	A	C	0.1831	0.0356	0.1451	2.70E-07	0.008	26.45	
RANTES	rs147509526	T	C	-0.3558	0.0715	0.9871	6.48E-07	0.003	24.76	
RANTES	rs112072646	A	G	0.4209	0.0859	0.0308	9.59E-07	0.011	24.01	
RANTES	rs10505135	T	C	0.1315	0.0252	0.6759	1.81E-07	0.008	27.23	
SCF	rs80271436	A	G	-0.2393	0.0484	0.0497	7.65E-07	0.005	24.45	
SCF	rs78666213	T	G	-0.2845	0.0574	0.0258	7.18E-07	0.004	24.57	
SCF	rs78369473	T	C	-0.2256	0.0484	0.9712	3.14E-06	0.003	21.73	
SCF	rs7535409	A	G	-0.1173	0.0169	0.6849	3.90E-12	0.006	48.18	
SCF	rs7037688	A	G	-0.0786	0.0167	0.2982	2.52E-06	0.003	22.15	6.00%
SCF	rs507666	A	G	-0.1035	0.0191	0.1859	6.00E-08	0.003	29.36	
SCF	rs4841895	A	G	0.1004	0.0178	0.3231	1.70E-08	0.004	31.81	
SCF	rs1568119	T	C	-0.5946	0.1129	0.9871	1.39E-07	0.009	27.74	
SCF	rs13412535	A	G	-0.1065	0.0213	0.2256	5.73E-07	0.004	25.00	
SCF	rs113127926	A	C	0.1974	0.0418	0.0626	2.33E-06	0.005	22.30	
SCGF	rs78217154	T	C	0.3942	0.0861	0.0229	4.69E-06	0.007	20.96	7.00%

SCGF	rs7815967	T	C	0.1325	0.0288	0.8807	4.21E-06	0.004	21.17	
SCGF	rs77954165	T	C	0.2631	0.0562	0.0755	2.85E-06	0.010	21.92	
SCGF	rs4656185	A	G	0.2103	0.0254	0.3151	1.24E-16	0.019	68.55	
SCGF	rs34911860	A	G	-0.3674	0.0787	0.0119	3.04E-06	0.003	21.79	
SCGF	rs264157	A	G	0.1079	0.0233	0.4702	3.64E-06	0.006	21.45	
SCGF	rs17876031	A	G	-0.1496	0.0254	0.6889	3.87E-09	0.010	34.69	
SCGF	rs151194174	A	G	0.4536	0.0941	0.0209	1.43E-06	0.008	23.24	
SCGF	rs150733161	T	C	-0.5255	0.112	0.9791	2.71E-06	0.011	22.01	
SCGF	rs149009264	A	G	0.4551	0.0985	0.9811	3.83E-06	0.008	21.35	
SCGF	rs143829871	T	C	-0.1866	0.0399	0.0596	2.92E-06	0.004	21.87	
SCGF	rs139413256	A	G	-0.5174	0.1076	0.0239	1.52E-06	0.012	23.12	
SCGF	rs12480722	T	C	0.1654	0.0353	0.0974	2.79E-06	0.005	21.95	
SCGF	rs117716477	A	C	0.8242	0.084	0.0209	1.00E-22	0.028	96.27	
SCGF	rs116924815	T	C	0.6046	0.0737	0.9642	2.33E-16	0.025	67.30	
SCGF	rs1149926	T	C	-0.3458	0.0749	0.9672	3.90E-06	0.008	21.32	
SCGF	rs112346514	T	C	-0.3261	0.0703	0.9761	3.51E-06	0.005	21.52	
SDF-1A	rs78037609	A	G	-0.6261	0.1334	0.0129	2.69E-06	0.010	22.03	
SDF-1A	rs3988298	T	C	-0.1263	0.0266	0.8499	2.05E-06	0.004	22.54	
SDF-1A	rs1600396	A	G	-0.0933	0.0204	0.7197	4.80E-06	0.004	20.92	14.00%
SDF-1A	rs149893336	A	G	-0.494	0.1082	0.9801	4.98E-06	0.010	20.84	
SDF-1A	rs12141941	T	C	-0.0881	0.0186	0.2654	2.17E-06	0.003	22.43	
SDF-1A	rs10474392	A	G	0.0934	0.0177	0.2694	1.31E-07	0.003	27.84	
TNF-A	rs79105320	A	G	0.5573	0.1177	0.0209	2.19E-06	0.013	22.42	
TNF-A	rs7256693	T	C	-0.1841	0.04	0.1113	4.17E-06	0.007	21.18	6.00%
TNF-A	rs115669577	A	G	0.981	0.1994	0.0119	8.67E-07	0.023	24.20	
TNF-A	rs111332265	A	G	-0.3678	0.0745	0.9284	7.94E-07	0.018	24.37	

TNF-A	rs10834996	A	G	0.123	0.0256	0.3598	1.55E-06	0.007	23.09	
TNF-B	rs78296352	T	G	1.2028	0.1366	0.9712	1.31E-18	0.081	77.53	
TNF-B	rs7629875	A	G	0.3841	0.0774	0.9423	6.96E-07	0.016	24.63	7.00%
TNF-B	rs2080926	T	C	0.1725	0.037	0.4642	3.13E-06	0.015	21.74	
TNF-B	rs10925040	T	C	0.1738	0.0372	0.6113	2.98E-06	0.014	21.83	
TRAIL	rs79287178	A	G	-0.4304	0.042	0.0258	1.21E-24	0.009	105.01	
TRAIL	rs75928541	A	G	0.2784	0.0591	0.0169	2.47E-06	0.003	22.19	
TRAIL	rs74778900	T	C	0.5791	0.0531	0.9861	1.08E-27	0.009	118.94	
TRAIL	rs747324	T	C	-0.0826	0.0178	0.6412	3.48E-06	0.003	21.53	
TRAIL	rs73039026	A	C	-0.3098	0.0634	0.9841	1.03E-06	0.003	23.88	
TRAIL	rs72899452	T	C	0.1223	0.0264	0.9235	3.61E-06	0.002	21.46	5.00%
TRAIL	rs62093514	T	C	1.0459	0.0549	0.9742	6.45E-81	0.055	362.94	
TRAIL	rs57396456	T	C	-0.5641	0.0516	0.0338	8.09E-28	0.021	119.51	
TRAIL	rs550057	T	C	-0.0783	0.0169	0.7177	3.60E-06	0.002	21.47	
TRAIL	rs193112415	T	C	-1.0456	0.062	0.0139	8.21E-64	0.030	284.41	
TRAIL	rs17434886	T	C	-0.0918	0.0199	0.8231	3.97E-06	0.002	21.28	
VEGF	rs9472183	A	G	-0.1264	0.017	0.5109	1.04E-13	0.008	55.28	
VEGF	rs8045833	A	G	0.103	0.0211	0.2634	1.05E-06	0.004	23.83	
VEGF	rs73872715	T	C	-0.6079	0.1299	0.9801	2.87E-06	0.014	21.90	
VEGF	rs73418461	A	G	-0.2498	0.0521	0.0606	1.63E-06	0.007	22.99	
VEGF	rs6921438	A	G	-0.4866	0.0174	0.4672	4.27E-172	0.118	782.07	5.00%
VEGF	rs4082730	A	G	0.2455	0.0533	0.9543	4.10E-06	0.005	21.22	
VEGF	rs3108686	A	C	-0.7967	0.1702	0.9831	2.86E-06	0.021	21.91	
VEGF	rs143479231	A	G	-0.2628	0.0489	0.0219	7.69E-08	0.003	28.88	
VEGF	rs13209117	A	G	0.1253	0.02	0.2843	3.73E-10	0.006	39.25	
VEGF	rs12456390	T	C	-0.0818	0.0179	0.3171	4.88E-06	0.003	20.88	

VEGF	rs10967186	T	C	0.0899	0.0169	0.4324	1.04E-07	0.004	28.30

Supplementary Table 8. Detailed information for circulating cytokines-associated SNPs with risk of Parkinson's disease (PD).

Exposure	SNP	Effect Allele	Other Allele	Beta	Se	EAF	*P-value*	*R2*	*F*	Power
BNGF	rs7970581	T	G	0.1358	0.028	0.7644	1.23E-06	0.007	23.52	10.00%
BNGF	rs73472576	T	C	-0.1146	0.0251	0.6044	4.98E-06	0.006	20.85	
BNGF	rs71641308	T	C	0.1969	0.0429	0.9463	4.44E-06	0.004	21.07	
BNGF	rs28637706	T	G	-0.1554	0.0261	0.6829	2.62E-09	0.010	35.45	
CTACK	rs76395525	A	G	0.5193	0.1081	0.0149	1.56E-06	0.008	23.08	21.00%
CTACK	rs7333764	T	C	0.2811	0.0591	0.9732	1.97E-06	0.004	22.62	
CTACK	rs72729450	T	C	-0.5123	0.1094	0.9861	2.83E-06	0.007	21.93	
CTACK	rs60247384	T	C	0.1128	0.0245	0.7058	4.14E-06	0.005	21.20	
CTACK	rs57789542	T	C	-0.7687	0.1659	0.0109	3.60E-06	0.013	21.47	
CTACK	rs57338032	A	G	0.1443	0.0316	0.8201	4.96E-06	0.006	20.85	
CTACK	rs55764737	T	C	0.5424	0.0967	0.0229	2.03E-08	0.013	31.46	
CTACK	rs2070074	A	G	0.4401	0.0372	0.8976	2.71E-32	0.036	139.96	
CTACK	rs135567	A	G	0.1664	0.0265	0.2614	3.40E-10	0.011	39.43	
CTACK	rs118084576	A	G	0.5675	0.1226	0.0139	3.68E-06	0.009	21.43	
CTACK	rs117385454	A	G	-0.1968	0.0413	0.9443	1.89E-06	0.004	22.71	
CTACK	rs116303454	A	G	0.3754	0.081	0.0288	3.58E-06	0.008	21.48	
EOTAXIN	rs9317045	A	C	0.1172	0.0236	0.8539	6.83E-07	0.003	24.66	5.00%
EOTAXIN	rs80341932	A	G	0.101	0.0204	0.7078	7.38E-07	0.004	24.51	
EOTAXIN	rs79722574	T	C	-0.1092	0.0227	0.84	1.50E-06	0.003	23.14	
EOTAXIN	rs75426604	A	C	-0.1371	0.0291	0.1312	2.46E-06	0.004	22.20	
EOTAXIN	rs745331	A	G	-0.0821	0.0176	0.3091	3.09E-06	0.003	21.76	
EOTAXIN	rs59808887	T	C	-0.1698	0.0356	0.9225	1.85E-06	0.004	22.75	
EOTAXIN	rs5754733	A	C	-0.105	0.0213	0.7883	8.24E-07	0.004	24.30	
EOTAXIN	rs2228467	T	C	-0.4154	0.0291	0.0696	3.14E-46	0.022	203.77	

EOTAXIN	rs2211994	T	C	0.0876	0.0177	0.7495	7.45E-07	0.003	24.49	
EOTAXIN	rs2027855	T	C	0.0743	0.0162	0.329	4.51E-06	0.002	21.04	
EOTAXIN	rs2024050	A	G	0.164	0.0302	0.8996	5.62E-08	0.005	29.49	
EOTAXIN	rs147287945	A	G	-0.1512	0.0313	0.0726	1.36E-06	0.003	23.34	
EOTAXIN	rs12075	A	G	0.1692	0.0155	0.6024	9.65E-28	0.014	119.16	
EOTAXIN	rs112347425	T	C	0.1595	0.0276	0.8926	7.52E-09	0.005	33.40	
EOTAXIN	rs11087905	A	C	0.0954	0.0188	0.3419	3.89E-07	0.004	25.75	
FGF	rs9903590	T	C	0.1281	0.0267	0.0974	1.60E-06	0.003	23.02	
FGF	rs78873483	A	G	0.1286	0.0282	0.0944	5.11E-06	0.003	20.80	
FGF	rs75168112	T	C	-0.1024	0.0214	0.1779	1.71E-06	0.003	22.90	
FGF	rs145577605	A	G	0.2043	0.0427	0.0109	1.71E-06	0.001	22.89	8.00%
FGF	rs13412535	A	G	-0.1129	0.0224	0.2256	4.65E-07	0.004	25.40	
FGF	rs116745220	A	G	-0.6176	0.1324	0.9801	3.09E-06	0.015	21.76	
G-CSF	rs77318030	T	C	-0.2031	0.0427	0.0517	1.97E-06	0.004	22.62	
G-CSF	rs76287671	T	C	0.0894	0.0189	0.8091	2.24E-06	0.002	22.37	
G-CSF	rs74148555	T	C	-0.3771	0.0753	0.9473	5.50E-07	0.014	25.08	
G-CSF	rs586313	T	C	-0.0883	0.0187	0.2286	2.34E-06	0.003	22.30	
G-CSF	rs2671444	A	G	-0.0776	0.0166	0.3648	2.94E-06	0.003	21.85	5.00%
G-CSF	rs11903143	A	G	0.0889	0.0175	0.7425	3.77E-07	0.003	25.81	
G-CSF	rs117261691	T	C	0.1318	0.0288	0.9652	4.73E-06	0.001	20.94	
G-CSF	rs116745220	A	G	-0.6789	0.1359	0.9801	5.87E-07	0.018	24.96	
GROA	rs78653452	T	G	-0.7395	0.1559	0.9881	2.10E-06	0.013	22.50	
GROA	rs62024303	A	G	-0.3013	0.066	0.9543	4.99E-06	0.008	20.84	
GROA	rs493091	T	C	0.384	0.0279	0.2346	4.23E-43	0.053	189.43	41.00%
GROA	rs188345231	T	C	0.6177	0.1322	0.9881	2.98E-06	0.009	21.83	
GROA	rs140734053	A	G	0.7333	0.1545	0.0249	2.07E-06	0.026	22.53	

GROA	rs12075	A	G	0.3724	0.0236	0.6024	4.29E-56	0.066	249.00	
GROA	rs118158560	A	G	0.2761	0.0592	0.0626	3.10E-06	0.009	21.75	
GROA	rs114991247	T	C	-0.2202	0.0463	0.0596	1.98E-06	0.005	22.62	
GROA	rs1113500	T	G	0.1162	0.0243	0.3698	1.74E-06	0.006	22.87	
HGF	rs5745687	T	C	-0.3008	0.0404	0.0567	9.65E-14	0.010	55.44	
HGF	rs57146176	A	G	-0.0987	0.0208	0.9404	2.08E-06	0.001	22.52	
HGF	rs4245058	T	C	-0.1552	0.0331	0.9066	2.75E-06	0.004	21.99	
HGF	rs3748034	T	G	0.1529	0.0233	0.1362	5.30E-11	0.006	43.06	5.00%
HGF	rs2003620	T	C	0.2277	0.0487	0.0606	2.93E-06	0.006	21.86	
HGF	rs13412535	A	G	-0.1043	0.0213	0.2256	9.75E-07	0.004	23.98	
HGF	rs11060254	A	G	-0.0765	0.0166	0.3429	4.06E-06	0.003	21.24	
IFN-G	rs78296352	T	G	0.3419	0.065	0.9712	1.44E-07	0.007	27.67	
IFN-G	rs74148555	T	C	-0.3771	0.077	0.9473	9.71E-07	0.014	23.98	
IFN-G	rs60059008	A	G	0.0852	0.0176	0.6670	1.29E-06	0.003	23.43	
IFN-G	rs2073438	A	G	0.092	0.0188	0.2714	9.90E-07	0.003	23.95	
IFN-G	rs12420286	T	C	0.2357	0.05	0.0288	2.43E-06	0.003	22.22	5.00%
IFN-G	rs11843756	T	G	0.1812	0.0391	0.0288	3.58E-06	0.002	21.48	
IFN-G	rs115729819	A	G	0.2511	0.0514	0.9791	1.03E-06	0.003	23.87	
IFN-G	rs113600793	A	C	0.1871	0.0371	0.0398	4.58E-07	0.003	25.43	
IFN-G	rs10760686	T	C	0.0795	0.0168	0.6879	2.22E-06	0.003	22.39	
IL-10	rs7088799	T	G	-0.0815	0.0166	0.4344	9.12E-07	0.003	24.10	
IL-10	rs6921438	A	G	-0.2876	0.0166	0.4672	3.03E-67	0.041	300.17	
IL-10	rs6799107	T	C	-0.095	0.0206	0.2137	3.99E-06	0.003	21.27	6.00%
IL-10	rs6117725	T	C	0.0979	0.0202	0.2634	1.26E-06	0.004	23.49	
IL-10	rs41282660	A	G	-0.1169	0.0254	0.8678	4.18E-06	0.003	21.18	
IL-10	rs383684	A	G	0.092	0.0197	0.1183	3.01E-06	0.002	21.81	

IL-10	rs339203	T	C	0.0954	0.0203	0.9602	2.61E-06	0.001	22.09	
IL-10	rs3025021	T	C	0.0913	0.0194	0.6322	2.52E-06	0.004	22.15	
IL-10	rs282258	T	C	0.0993	0.0162	0.5746	8.81E-10	0.005	37.57	
IL-10	rs2086656	T	C	-0.08	0.017	0.8241	2.53E-06	0.002	22.15	
IL-10	rs1530455	T	C	0.082	0.0174	0.5915	2.45E-06	0.003	22.21	
IL-10	rs10493718	A	C	-0.1081	0.0222	0.2913	1.12E-06	0.005	23.71	
IL-10	rs10457128	A	G	-0.0854	0.0172	0.6392	6.87E-07	0.003	24.65	
IL-12	rs7088799	T	G	-0.0815	0.0166	0.4344	9.12E-07	0.003	24.10	
IL-12	rs6921438	A	G	-0.2876	0.0166	0.4672	3.03E-67	0.041	300.17	
IL-12	rs6799107	T	C	-0.095	0.0206	0.2137	3.99E-06	0.003	21.27	
IL-12	rs6117725	T	C	0.0979	0.0202	0.2634	1.26E-06	0.004	23.49	
IL-12	rs41282660	A	G	-0.1169	0.0254	0.0636	4.18E-06	0.002	21.18	
IL-12	rs383684	A	G	0.092	0.0197	0.1183	3.01E-06	0.002	21.81	
IL-12	rs339203	T	C	0.0954	0.0203	0.9602	2.61E-06	0.001	22.09	5.00%
IL-12	rs3025021	T	C	0.0913	0.0194	0.6322	2.52E-06	0.004	22.15	
IL-12	rs282258	T	C	0.0993	0.0162	0.5746	8.81E-10	0.005	37.57	
IL-12	rs2086656	T	C	-0.08	0.017	0.3052	2.53E-06	0.003	22.15	
IL-12	rs1530455	T	C	0.082	0.0174	0.5915	2.45E-06	0.003	22.21	
IL-12	rs10493718	A	C	-0.1081	0.0222	0.2913	1.12E-06	0.005	23.71	
IL-12	rs10457128	A	G	-0.0854	0.0172	0.6392	6.87E-07	0.003	24.65	
IL-13	rs9472168	A	G	0.4211	0.0246	0.5507	1.09E-65	0.088	293.02	
IL-13	rs77955971	A	C	0.4408	0.0868	0.0239	3.81E-07	0.009	25.79	
IL-13	rs73192842	A	G	0.1472	0.0299	0.2107	8.52E-07	0.007	24.24	6.00%
IL-13	rs27949	T	C	-0.1144	0.025	0.6312	4.74E-06	0.006	20.94	
IL-13	rs139083458	T	C	0.9995	0.211	0.9841	2.17E-06	0.031	22.44	
IL-13	rs138854806	A	G	-0.4204	0.0839	0.0119	5.42E-07	0.004	25.11	

IL-13	rs12623722	A	G	-0.1189	0.0257	0.2823	3.72E-06	0.006	21.40	
IL-13	rs117795020	A	G	-0.3584	0.0716	0.0179	5.57E-07	0.005	25.06	
IL-13	rs10995615	T	C	-0.1591	0.0341	0.1521	3.08E-06	0.007	21.77	
IL-16	rs9706053	T	C	0.4412	0.0928	0.9841	1.99E-06	0.006	22.60	
IL-16	rs4778636	A	G	-0.7286	0.063	0.0746	6.19E-31	0.073	133.75	
IL-16	rs4253283	T	C	0.1506	0.026	0.6998	6.94E-09	0.010	33.55	
IL-16	rs1801020	A	G	0.1678	0.0271	0.7753	5.95E-10	0.010	38.34	
IL-16	rs144691581	A	G	0.4929	0.0958	0.0149	2.67E-07	0.007	26.47	10.00%
IL-16	rs1255143	T	C	0.1387	0.0241	0.4344	8.65E-09	0.009	33.12	
IL-16	rs117916513	A	G	-0.4713	0.0982	0.0209	1.59E-06	0.009	23.03	
IL-16	rs117217798	T	C	-0.2064	0.044	0.9314	2.72E-06	0.005	22.00	
IL-16	rs116135478	A	G	0.8296	0.1637	0.9821	4.02E-07	0.024	25.68	
IL-17	rs78296352	T	G	0.2949	0.0645	0.9712	4.83E-06	0.005	20.90	
IL-17	rs184080173	T	C	0.236	0.0471	0.0626	5.43E-07	0.007	25.11	
IL-17	rs17282552	T	C	-0.2026	0.0403	0.0268	4.97E-07	0.002	25.27	
IL-17	rs1530455	T	C	0.1088	0.0173	0.5915	3.19E-10	0.006	39.55	5.00%
IL-17	rs12735700	T	G	-0.0943	0.0206	0.7515	4.70E-06	0.003	20.96	
IL-17	rs117556572	T	C	-0.5256	0.1097	0.9891	1.66E-06	0.006	22.96	
IL-17	rs113098509	A	G	-0.1553	0.0337	0.9235	4.06E-06	0.003	21.24	
IL-18	rs78716465	A	G	0.3173	0.0679	0.0378	2.97E-06	0.007	21.84	
IL-18	rs78623212	T	C	0.8322	0.1676	0.9761	6.86E-07	0.032	24.66	
IL-18	rs71478720	T	C	-0.26	0.0273	0.7823	1.67E-21	0.023	90.70	
IL-18	rs610473	A	G	0.1274	0.0242	0.6501	1.41E-07	0.007	27.71	17.00%
IL-18	rs4482818	A	G	0.1233	0.0243	0.6412	3.89E-07	0.007	25.75	
IL-18	rs385076	T	C	-0.2472	0.0247	0.6471	1.40E-23	0.028	100.16	
IL-18	rs1979967	T	C	0.14	0.0285	0.7406	9.00E-07	0.008	24.13	

IL-18	rs17229943	A	C	-0.3076	0.0463	0.9503	3.06E-11	0.009	44.14	
IL-18	rs117266781	T	C	0.7051	0.1436	0.9891	9.10E-07	0.011	24.11	
IL-18	rs116383510	A	C	-0.5412	0.1052	0.9801	2.68E-07	0.011	26.47	
IL-18	rs10414552	T	C	-0.1817	0.0347	0.8787	1.64E-07	0.007	27.42	
IL-1B	rs62015704	A	G	0.1786	0.0372	0.8787	1.58E-06	0.007	23.05	
IL-1B	rs61335305	A	C	0.4333	0.0928	0.0109	3.02E-06	0.004	21.80	5.00%
IL-1B	rs143319329	T	C	0.4357	0.093	0.9761	2.80E-06	0.009	21.95	
IL-1B	rs115242021	A	C	0.2795	0.0553	0.0815	4.32E-07	0.012	25.55	
IL-1RA	rs6699436	A	G	-0.1858	0.0404	0.1431	4.25E-06	0.008	21.15	
IL-1RA	rs61335305	A	C	0.4315	0.0904	0.0109	1.81E-06	0.004	22.78	
IL-1RA	rs56134659	A	G	-0.1109	0.0236	0.5258	2.61E-06	0.006	22.08	
IL-1RA	rs4441609	T	C	0.1056	0.0231	0.6302	4.84E-06	0.005	20.90	9.00%
IL-1RA	rs187166731	T	C	-0.2424	0.0504	0.9881	1.51E-06	0.001	23.13	
IL-1RA	rs11627423	A	C	0.1178	0.0246	0.6103	1.68E-06	0.007	22.93	
IL-1RA	rs1054402	T	C	0.1325	0.0269	0.7336	8.41E-07	0.007	24.26	
IL-2	rs7615304	A	G	-0.1139	0.024	0.3489	2.08E-06	0.006	22.52	
IL-2	rs62124990	T	G	-0.7013	0.149	0.9742	2.52E-06	0.025	22.15	
IL-2	rs61335305	A	C	0.4439	0.0913	0.0109	1.16E-06	0.004	23.64	
IL-2	rs4634519	A	G	-0.1249	0.0268	0.6879	3.16E-06	0.007	21.72	47.00%
IL-2	rs2690020	A	G	0.1158	0.0245	0.5547	2.28E-06	0.007	22.34	
IL-2	rs170117	T	C	-0.1637	0.0347	0.8797	2.39E-06	0.006	22.26	
IL-2	rs16836080	A	G	0.1158	0.0253	0.2922	4.72E-06	0.006	20.95	
IL-2	rs13412535	A	G	0.174	0.0331	0.2256	1.47E-07	0.011	27.63	
IL-2RA	rs56213152	T	C	0.1269	0.0271	0.2604	2.83E-06	0.006	21.93	
IL-2RA	rs4733117	A	C	0.1439	0.0291	0.8360	7.61E-07	0.006	24.45	5.00%
IL-2RA	rs185231391	T	C	0.8568	0.1803	0.0149	2.01E-06	0.022	22.58	

IL-2RA	rs12799226	T	C	-0.1285	0.0277	0.8091	3.50E-06	0.005	21.52	
IL-2RA	rs12722497	A	C	0.6287	0.0482	0.8559	6.91E-39	0.097	170.13	
IL-2RA	rs117244812	A	G	-0.7187	0.1493	0.0139	1.48E-06	0.014	23.17	
IL-2RA	rs11241559	T	G	-0.124	0.0264	0.7813	2.64E-06	0.005	22.06	
IL-4	rs9941733	A	G	0.1156	0.0229	0.825	4.46E-07	0.004	25.48	
IL-4	rs9508291	T	C	-0.168	0.0358	0.0646	2.70E-06	0.003	22.02	
IL-4	rs79597994	T	C	-0.5855	0.1271	0.9722	4.09E-06	0.019	21.22	
IL-4	rs7613691	A	G	0.1787	0.0382	0.9473	2.90E-06	0.003	21.88	
IL-4	rs73023729	A	G	-0.1796	0.0365	0.0229	8.63E-07	0.001	24.21	
IL-4	rs6765768	A	G	0.0796	0.0167	0.6083	1.87E-06	0.003	22.72	
IL-4	rs2708550	T	C	-0.0764	0.0165	0.7803	3.65E-06	0.002	21.44	5.00%
IL-4	rs2073438	A	G	0.0847	0.0183	0.2714	3.68E-06	0.003	21.42	
IL-4	rs17713451	A	G	0.1255	0.0252	0.1650	6.35E-07	0.004	24.80	
IL-4	rs12238729	T	C	0.5271	0.1096	0.9881	1.51E-06	0.007	23.13	
IL-4	rs117146485	T	C	-0.2856	0.0625	0.0149	4.89E-06	0.002	20.88	
IL-4	rs116705532	T	G	-0.4675	0.0978	0.0169	1.75E-06	0.007	22.85	
IL-4	rs10512267	T	C	-0.0824	0.016	0.3300	2.60E-07	0.003	26.52	
IL-5	rs74811276	A	G	0.217	0.0471	0.0686	4.08E-06	0.006	21.23	
IL-5	rs72831687	A	G	-0.5337	0.1104	0.0169	1.34E-06	0.009	23.37	
IL-5	rs6737109	T	C	0.1135	0.0246	0.4254	3.95E-06	0.006	21.29	
IL-5	rs4320361	T	G	-0.1553	0.025	0.5477	5.23E-10	0.012	38.59	6.00%
IL-5	rs28793375	T	C	0.1697	0.0362	0.8330	2.76E-06	0.008	21.98	
IL-5	rs148634917	A	G	-0.517	0.1087	0.9811	1.97E-06	0.010	22.62	
IL-5	rs11680908	A	G	0.2593	0.0552	0.9453	2.63E-06	0.007	22.07	
IL-6	rs76856708	T	C	0.336	0.0697	0.0378	1.43E-06	0.008	23.24	6.00%
IL-6	rs73273528	T	C	0.268	0.0553	0.9662	1.26E-06	0.005	23.49	

IL-6	rs72831623	A	G	0.197	0.0369	0.0527	9.36E-08	0.004	28.50		
IL-6	rs4684700	T	C	-0.0747	0.0162	0.5139	4.01E-06	0.003	21.26		
IL-6	rs2404476	A	G	0.0734	0.0156	0.4722	2.54E-06	0.003	22.14		
IL-6	rs13412535	A	G	-0.1186	0.0214	0.2256	2.99E-08	0.005	30.71		
IL-6	rs1333040	T	C	0.0747	0.0157	0.4264	1.96E-06	0.003	22.64		
IL-6	rs113098456	A	G	-0.1553	0.0339	0.0765	4.62E-06	0.003	20.99		
IL-6	rs10817609	T	C	0.0845	0.0175	0.2157	1.38E-06	0.002	23.32		
IL-7	rs77981494	T	C	-0.5201	0.1055	0.0159	8.23E-07	0.008	24.30		
IL-7	rs6921438	A	G	-0.3204	0.0246	0.4672	8.89E-39	0.051	169.63		
IL-7	rs62006410	T	C	-0.1492	0.0302	0.7853	7.80E-07	0.008	24.41		
IL-7	rs28793375	T	C	0.1644	0.036	0.8330	4.96E-06	0.008	20.85		
IL-7	rs1958987	T	C	0.1261	0.0263	0.6998	1.63E-06	0.007	22.99	25.00%	
IL-7	rs17091524	T	C	0.5092	0.1015	0.0427	5.26E-07	0.021	25.17		
IL-7	rs141425475	T	C	-0.4801	0.1018	0.0288	2.40E-06	0.013	22.24		
IL-7	rs117509142	T	C	-0.3213	0.0684	0.0567	2.64E-06	0.011	22.07		
IL-7	rs115215018	T	C	0.5985	0.1308	0.9881	4.75E-06	0.008	20.94		
IL-7	rs10196226	A	G	0.1538	0.0327	0.1471	2.56E-06	0.006	22.12		
IL-8	rs75840288	A	C	0.5125	0.1121	0.9702	4.84E-06	0.015	20.90		
IL-8	rs3786107	A	G	0.2463	0.0517	0.0596	1.90E-06	0.007	22.70		
IL-8	rs2673604	A	C	-0.118	0.0254	0.2992	3.39E-06	0.006	21.58		
IL-8	rs183628733	T	C	0.6547	0.1417	0.0219	3.83E-06	0.018	21.35	5.00%	
IL-8	rs141926526	A	C	-0.6221	0.1308	0.9523	1.97E-06	0.035	22.62		
IL-8	rs12438669	A	C	-0.1182	0.0252	0.6571	2.73E-06	0.006	22.00		
IL-8	rs12075	A	G	0.1148	0.0235	0.6024	1.03E-06	0.006	23.86		
IL-8	rs116726256	T	C	-0.2247	0.0489	0.0318	4.33E-06	0.003	21.11		
IL-9	rs76963786	T	C	-0.2856	0.0556	0.9135	2.80E-07	0.013	26.39	10.00%	

IL-9	rs73443903	A	C	0.2162	0.046	0.1004	2.60E-06	0.008	22.09	
IL-9	rs4880409	T	C	-0.3552	0.0716	0.0278	7.02E-07	0.007	24.61	
IL-9	rs41294750	T	C	0.3442	0.0736	0.9722	2.92E-06	0.006	21.87	
IP-10	rs9450351	T	C	-0.2651	0.0488	0.0616	5.56E-08	0.008	29.51	
IP-10	rs7645625	T	G	-0.1116	0.0236	0.4324	2.26E-06	0.006	22.36	
IP-10	rs75970138	A	G	-0.4845	0.1037	0.0129	2.98E-06	0.006	21.83	
IP-10	rs6707974	A	G	0.1574	0.0337	0.8380	3.00E-06	0.007	21.81	5.00%
IP-10	rs4862110	T	C	-0.1453	0.0318	0.1869	4.90E-06	0.006	20.88	
IP-10	rs34383175	T	C	-0.3196	0.0653	0.9732	9.86E-07	0.005	23.95	
IP-10	rs143799975	A	G	-0.7551	0.1638	0.9871	4.03E-06	0.015	21.25	
IP-10	rs113831257	A	G	0.3639	0.0641	0.0417	1.37E-08	0.011	32.23	
MCP-1-MCAF	rs9317045	A	C	0.1157	0.0235	0.8539	8.51E-07	0.003	24.24	
MCP-1-MCAF	rs7632755	A	G	0.2984	0.0315	0.0805	2.72E-21	0.013	89.74	
MCP-1-MCAF	rs7517040	A	G	-0.097	0.019	0.2823	3.30E-07	0.004	26.06	
MCP-1-MCAF	rs7197349	A	G	0.0971	0.0206	0.8628	2.43E-06	0.002	22.22	
MCP-1-MCAF	rs7019112	T	G	-0.2206	0.0468	0.0119	2.43E-06	0.001	22.22	
MCP-1-MCAF	rs56212190	T	C	0.1799	0.0372	0.9453	1.32E-06	0.003	23.39	
MCP-1-MCAF	rs2288370	T	C	-0.1036	0.0162	0.3956	1.60E-10	0.005	40.90	5.00%
MCP-1-MCAF	rs2036297	A	G	0.1182	0.016	0.3429	1.50E-13	0.006	54.58	
MCP-1-MCAF	rs146522229	T	C	-0.5942	0.1161	0.9761	3.09E-07	0.016	26.19	
MCP-1-MCAF	rs143815843	A	G	-0.2049	0.0447	0.0109	4.56E-06	0.001	21.01	
MCP-1-MCAF	rs12075	A	G	0.2186	0.0154	0.6024	9.87E-46	0.023	201.49	
MCP-1-MCAF	rs111995966	T	G	0.1428	0.0309	0.0258	3.81E-06	0.001	21.36	
MCP-1-MCAF	rs11118557	A	G	0.148	0.0319	0.9264	3.49E-06	0.003	21.52	
MCP-1-MCAF	rs10744620	T	C	0.0783	0.0161	0.6392	1.15E-06	0.003	23.65	
MCP-3	rs62492260	T	G	-0.2802	0.0578	0.8509	1.25E-06	0.020	23.50	6.00%

MCP-3	rs3129806	T	C	-0.1975	0.0433	0.6322	5.09E-06	0.018	20.80	
MCP-3	rs2838065	A	G	-0.221	0.0479	0.1968	3.95E-06	0.015	21.29	
MCP-3	rs117286643	A	G	0.6934	0.1474	0.0109	2.55E-06	0.010	22.13	
MCP-3	rs10892381	T	C	0.2432	0.0473	0.3002	2.72E-07	0.025	26.44	
M-CSF	rs9387100	T	C	-0.135	0.029	0.6730	3.24E-06	0.008	21.67	
M-CSF	rs78296352	T	G	0.522	0.111	0.9712	2.57E-06	0.015	22.12	
M-CSF	rs72723242	T	G	-0.4969	0.1083	0.9374	4.47E-06	0.029	21.05	
M-CSF	rs62294910	A	G	0.3472	0.0687	0.0527	4.33E-07	0.012	25.54	
M-CSF	rs56367447	T	C	-0.4878	0.0876	0.9662	2.57E-08	0.016	31.01	5.00%
M-CSF	rs34089869	T	C	0.2194	0.0462	0.8976	2.05E-06	0.009	22.55	
M-CSF	rs12962919	T	C	0.3025	0.0659	0.9066	4.43E-06	0.015	21.07	
M-CSF	rs117867915	T	C	0.5224	0.1096	0.0139	1.88E-06	0.007	22.72	
M-CSF	rs116274860	T	G	0.8262	0.1739	0.0159	2.02E-06	0.021	22.57	
MIF	rs78098071	T	C	-0.4583	0.0915	0.0189	5.48E-07	0.008	25.09	
MIF	rs3814097	A	G	-0.1163	0.0251	0.4751	3.60E-06	0.007	21.47	
MIF	rs35890933	T	G	0.1676	0.0365	0.2008	4.39E-06	0.009	21.08	
MIF	rs141009259	T	C	-0.6194	0.1285	0.0129	1.43E-06	0.010	23.23	6.00%
MIF	rs13142904	T	C	-0.2232	0.0425	0.9314	1.51E-07	0.006	27.58	
MIF	rs12594190	A	G	0.1321	0.0266	0.7535	6.83E-07	0.006	24.66	
MIF	rs113218956	A	G	-0.8789	0.1876	0.0109	2.80E-06	0.017	21.95	
MIG	rs816960	T	C	-0.1179	0.0242	0.2594	1.11E-06	0.005	23.74	
MIG	rs77086208	T	C	0.327	0.0694	0.9811	2.46E-06	0.004	22.20	
MIG	rs6679677	A	C	0.1628	0.0327	0.0915	6.40E-07	0.004	24.79	21.00%
MIG	rs1796086	T	C	-0.2172	0.04	0.0944	5.64E-08	0.008	29.48	
MIG	rs139010077	T	C	0.4337	0.0943	0.9891	4.24E-06	0.004	21.15	
MIG	rs117831247	T	C	-0.8819	0.173	0.9811	3.44E-07	0.029	25.99	

MIG	rs112861654	A	G	-0.2682	0.0527	0.9135	3.60E-07	0.011	25.90	
MIG	rs11177248	A	G	0.3157	0.0667	0.0606	2.21E-06	0.011	22.40	
MIG	rs111607343	A	G	-0.5235	0.1119	0.0378	2.89E-06	0.020	21.89	
MIP-1A	rs60198979	A	G	-0.2154	0.0455	0.0865	2.20E-06	0.007	22.41	
MIP-1A	rs57786342	A	G	0.139	0.0283	0.2157	9.03E-07	0.007	24.12	15.00%
MIP-1A	rs184154340	A	G	0.3251	0.0689	0.0408	2.38E-06	0.008	22.26	
MIP-1A	rs12690897	A	G	0.1215	0.026	0.2813	2.97E-06	0.006	21.84	
MIP-1B	rs77668982	A	G	0.3274	0.0389	0.0179	3.88E-17	0.004	70.84	
MIP-1B	rs76776296	A	G	0.313	0.0598	0.9632	1.66E-07	0.007	27.40	
MIP-1B	rs76582507	A	G	0.3259	0.0676	0.0268	1.43E-06	0.006	23.24	
MIP-1B	rs72799710	T	C	-0.1037	0.0217	0.8340	1.76E-06	0.003	22.84	
MIP-1B	rs72791296	T	C	0.2364	0.0466	0.9414	3.92E-07	0.006	25.73	
MIP-1B	rs6908843	A	G	0.0997	0.0209	0.1809	1.84E-06	0.003	22.76	
MIP-1B	rs5743614	T	C	0.1115	0.0232	0.2744	1.54E-06	0.005	23.10	5.00%
MIP-1B	rs281749	T	C	0.0795	0.0171	0.2913	3.33E-06	0.003	21.61	
MIP-1B	rs17138331	A	G	-0.1434	0.0295	0.9056	1.17E-06	0.004	23.63	
MIP-1B	rs117657747	A	G	0.2089	0.0453	0.0656	4.00E-06	0.005	21.27	
MIP-1B	rs117453826	A	G	-0.5907	0.0591	0.9841	1.60E-23	0.011	99.90	
MIP-1B	rs116237296	A	G	0.5284	0.1115	0.0159	2.15E-06	0.009	22.46	
MIP-1B	rs113877493	T	C	-0.607	0.0217	0.8777	3.53E-172	0.079	782.45	
MIP-1B	rs113010081	T	C	-0.5799	0.0236	0.1083	2.52E-133	0.065	603.78	
PDGF-BB	rs9941733	A	G	0.1165	0.0227	0.825	2.86E-07	0.004	26.34	
PDGF-BB	rs73162807	A	C	-0.2313	0.0499	0.0129	3.56E-06	0.001	21.49	
PDGF-BB	rs72777070	T	G	-0.1048	0.02	0.1998	1.61E-07	0.004	27.46	5.00%
PDGF-BB	rs55680718	T	C	-0.1359	0.0245	0.8628	2.91E-08	0.004	30.77	
PDGF-BB	rs4965869	T	C	0.1843	0.0181	0.7237	2.38E-24	0.014	103.68	

PDGF-BB	rs35859699	A	G	-0.3854	0.0838	0.0229	4.24E-06	0.007	21.15	
PDGF-BB	rs2324229	T	C	0.0884	0.0161	0.3936	4.00E-08	0.004	30.15	
PDGF-BB	rs13412535	A	G	0.3317	0.0214	0.2256	3.47E-54	0.038	240.25	
PDGF-BB	rs12289510	A	G	-0.0772	0.0158	0.4672	1.03E-06	0.003	23.87	
PDGF-BB	rs11766649	A	G	0.0902	0.0196	0.7386	4.18E-06	0.003	21.18	
PDGF-BB	rs116445074	T	G	0.2869	0.0587	0.9841	1.02E-06	0.003	23.89	
RANTES	rs9675798	T	G	-0.2583	0.0552	0.0378	2.88E-06	0.005	21.90	
RANTES	rs74472919	T	C	0.3547	0.06	0.9811	3.39E-09	0.005	34.95	
RANTES	rs72793342	A	G	-0.1505	0.0307	0.2078	9.47E-07	0.007	24.03	
RANTES	rs2731672	T	C	-0.1242	0.0272	0.2266	4.97E-06	0.005	20.85	
RANTES	rs2251660	A	C	0.1831	0.0356	0.1451	2.70E-07	0.008	26.45	5.00%
RANTES	rs147509526	T	C	-0.3558	0.0715	0.9871	6.48E-07	0.003	24.76	
RANTES	rs112072646	A	G	0.4209	0.0859	0.0308	9.59E-07	0.011	24.01	
RANTES	rs10505135	T	C	0.1315	0.0252	0.6759	1.81E-07	0.008	27.23	
SCF	rs80271436	A	G	-0.2393	0.0484	0.0497	7.65E-07	0.005	24.45	
SCF	rs78666213	T	G	-0.2845	0.0574	0.0258	7.18E-07	0.004	24.57	
SCF	rs78369473	T	C	-0.2256	0.0484	0.9712	3.14E-06	0.003	21.73	
SCF	rs7535409	A	G	-0.1173	0.0169	0.6849	3.90E-12	0.006	48.18	
SCF	rs7037688	A	G	-0.0786	0.0167	0.2982	2.52E-06	0.003	22.15	5.00%
SCF	rs4841895	A	G	0.1004	0.0178	0.3231	1.70E-08	0.004	31.81	
SCF	rs1568119	T	C	-0.5946	0.1129	0.9871	1.39E-07	0.009	27.74	
SCF	rs13412535	A	G	-0.1065	0.0213	0.2256	5.73E-07	0.004	25.00	
SCF	rs113127926	A	C	0.1974	0.0418	0.0626	2.33E-06	0.005	22.30	
SCGF	rs78217154	T	C	0.3942	0.0861	0.0229	4.69E-06	0.007	20.96	
SCGF	rs7815967	T	C	0.1325	0.0288	0.8807	4.21E-06	0.004	21.17	5.00%
SCGF	rs77954165	T	C	0.2631	0.0562	0.0755	2.85E-06	0.010	21.92	

SCGF	rs4656185	A	G	0.2103	0.0254	0.3151	1.24E-16	0.019	68.55	
SCGF	rs34911860	A	G	-0.3674	0.0787	0.0119	3.04E-06	0.003	21.79	
SCGF	rs264157	A	G	0.1079	0.0233	0.4702	3.64E-06	0.006	21.45	
SCGF	rs17876031	A	G	-0.1496	0.0254	0.6889	3.87E-09	0.010	34.69	
SCGF	rs151194174	A	G	0.4536	0.0941	0.0209	1.43E-06	0.008	23.24	
SCGF	rs150733161	T	C	-0.5255	0.112	0.9791	2.71E-06	0.011	22.01	
SCGF	rs149009264	A	G	0.4551	0.0985	0.9811	3.83E-06	0.008	21.35	
SCGF	rs143829871	T	C	-0.1866	0.0399	0.0596	2.92E-06	0.004	21.87	
SCGF	rs139413256	A	G	-0.5174	0.1076	0.0239	1.52E-06	0.012	23.12	
SCGF	rs12480722	T	C	0.1654	0.0353	0.0974	2.79E-06	0.005	21.95	
SCGF	rs117716477	A	C	0.8242	0.084	0.0209	1.00E-22	0.028	96.27	
SCGF	rs116924815	T	C	0.6046	0.0737	0.9642	2.33E-16	0.025	67.30	
SCGF	rs1149926	T	C	-0.3458	0.0749	0.9672	3.90E-06	0.008	21.32	
SCGF	rs112346514	T	C	-0.3261	0.0703	0.9761	3.51E-06	0.005	21.52	
SDF-1A	rs78037609	A	G	-0.6261	0.1334	0.0129	2.69E-06	0.010	22.03	
SDF-1A	rs62194947	T	C	-0.0852	0.0185	0.9841	4.12E-06	0.000	21.21	
SDF-1A	rs3988298	T	C	-0.1263	0.0266	0.8499	2.05E-06	0.004	22.54	5.00%
SDF-1A	rs1600396	A	G	-0.0933	0.0204	0.7197	4.80E-06	0.004	20.92	
SDF-1A	rs149893336	A	G	-0.494	0.1082	0.9801	4.98E-06	0.010	20.84	
TNF-A	rs79105320	A	G	0.5573	0.1177	0.0209	2.19E-06	0.013	22.42	
TNF-A	rs115669577	A	G	0.981	0.1994	0.0119	8.67E-07	0.023	24.20	5.00%
TNF-A	rs111332265	A	G	-0.3678	0.0745	0.9284	7.94E-07	0.018	24.37	
TNF-A	rs10834996	A	G	0.123	0.0256	0.3598	1.55E-06	0.007	23.09	
TNF-B	rs78296352	T	G	1.2028	0.1366	0.9712	1.31E-18	0.081	77.53	
TNF-B	rs7629875	A	G	0.3841	0.0774	0.9423	6.96E-07	0.016	24.63	5.00%
TNF-B	rs2080926	T	C	0.1725	0.037	0.4642	3.13E-06	0.015	21.74	

TNF-B	rs10925040	T	C	0.1738	0.0372	0.6113	2.98E-06	0.014	21.83	
TRAIL	rs78296352	T	G	0.3419	0.065	0.9712	1.44E-07	0.007	27.67	
TRAIL	rs74148555	T	C	-0.3771	0.077	0.9473	9.71E-07	0.014	23.98	
TRAIL	rs60059008	A	G	0.0852	0.0176	0.6670	1.29E-06	0.003	23.43	
TRAIL	rs2073438	A	G	0.092	0.0188	0.2714	9.90E-07	0.003	23.95	
TRAIL	rs12420286	T	C	0.2357	0.05	0.0288	2.43E-06	0.003	22.22	5.00%
TRAIL	rs11843756	T	G	0.1812	0.0391	0.0288	3.58E-06	0.002	21.48	
TRAIL	rs115729819	A	G	0.2511	0.0514	0.9791	1.03E-06	0.003	23.87	
TRAIL	rs113600793	A	C	0.1871	0.0371	0.0398	4.58E-07	0.003	25.43	
TRAIL	rs10760686	T	C	0.0795	0.0168	0.6879	2.22E-06	0.003	22.39	
VEGF	rs9472183	A	G	-0.1264	0.017	0.5109	1.04E-13	0.008	55.28	
VEGF	rs8045833	A	G	0.103	0.0211	0.2634	1.05E-06	0.004	23.83	
VEGF	rs73872715	T	C	-0.6079	0.1299	0.9801	2.87E-06	0.014	21.90	
VEGF	rs73418461	A	G	-0.2498	0.0521	0.0606	1.63E-06	0.007	22.99	
VEGF	rs6921438	A	G	-0.4866	0.0174	0.4672	4.27E-172	0.118	782.07	
VEGF	rs4082730	A	G	0.2455	0.0533	0.9543	4.10E-06	0.005	21.22	5.00%
VEGF	rs3108686	A	C	-0.7967	0.1702	0.6322	2.86E-06	0.295	21.91	
VEGF	rs143479231	A	G	-0.2628	0.0489	0.0219	7.69E-08	0.003	28.88	
VEGF	rs13209117	A	G	0.1253	0.02	0.2843	3.73E-10	0.006	39.25	
VEGF	rs12456390	T	C	-0.0818	0.0179	0.3171	4.88E-06	0.003	20.88	
VEGF	rs10967186	T	C	0.0899	0.0169	0.4324	1.04E-07	0.004	28.30	

Supplementary Table 9. Detailed information for circulating cytokines-associated SNPs with risk of amyotrophic lateral sclerosis (ALS).

Exposure	SNP	Effect Allele	Other Allele	Beta	Se	EAF	*P-value*	*R2*	*F*	Power
BNGF	rs7970581	A	G	0.1358	0.028	0.7644	1.23E-06	0.007	23.52	17.00%
BNGF	rs73472576	T	C	-0.1146	0.0251	0.6044	4.98E-06	0.006	20.85	
BNGF	rs71641308	T	C	0.1969	0.0429	0.9463	4.44E-06	0.004	21.07	
BNGF	rs28637706	A	G	-0.1554	0.0261	0.6829	2.62E-09	0.010	35.45	
CTACK	rs76395525	A	G	0.5193	0.1081	0.0149	1.56E-06	0.008	23.08	8.00%
CTACK	rs7333764	T	C	0.2811	0.0591	0.9732	1.97E-06	0.004	22.62	
CTACK	rs72729450	T	C	-0.5123	0.1094	0.9861	2.83E-06	0.007	21.93	
CTACK	rs60247384	T	C	0.1128	0.0245	0.7058	4.14E-06	0.005	21.20	
CTACK	rs57789542	T	C	-0.7687	0.1659	0.0109	3.60E-06	0.013	21.47	
CTACK	rs57338032	A	G	0.1443	0.0316	0.8201	4.96E-06	0.006	20.85	
CTACK	rs55764737	T	C	0.5424	0.0967	0.0229	2.03E-08	0.013	31.46	
CTACK	rs2070074	A	G	0.4401	0.0372	0.8976	2.71E-32	0.036	139.96	
CTACK	rs135567	A	G	0.1664	0.0265	0.2614	3.40E-10	0.011	39.43	
CTACK	rs118084576	A	G	0.5675	0.1226	0.0139	3.68E-06	0.009	21.43	
CTACK	rs116303454	A	G	0.3754	0.081	0.0288	3.58E-06	0.008	21.48	
EOTAXIN	rs9793308	A	G	0.0842	0.0177	0.6282	1.96E-06	0.003	22.63	5.00%
EOTAXIN	rs77668982	A	G	0.3274	0.0389	0.0179	3.88E-17	0.004	70.84	
EOTAXIN	rs76776296	A	G	0.313	0.0598	0.9632	1.66E-07	0.007	27.40	
EOTAXIN	rs76582507	A	G	0.3259	0.0676	0.0268	1.43E-06	0.006	23.24	
EOTAXIN	rs72799710	T	C	-0.1037	0.0217	0.0527	1.76E-06	0.001	22.84	
EOTAXIN	rs72791296	T	C	0.2364	0.0466	0.9414	3.92E-07	0.006	25.73	
EOTAXIN	rs6908843	A	G	0.0997	0.0209	0.1809	1.84E-06	0.003	22.76	
EOTAXIN	rs5743614	T	C	0.1115	0.0232	0.0567	1.54E-06	0.001	23.10	
EOTAXIN	rs281749	T	C	0.0795	0.0171	0.2913	3.33E-06	0.003	21.61	

EOTAXIN	rs17138331	A	G	-0.1434	0.0295	0.9056	1.17E-06	0.004	23.63	
EOTAXIN	rs1564708	T	C	-0.1697	0.0187	0.7296	1.14E-19	0.011	82.35	
EOTAXIN	rs117657747	A	G	0.2089	0.0453	0.0656	4.00E-06	0.005	21.27	
EOTAXIN	rs117453826	A	G	-0.5907	0.0591	0.9841	1.60E-23	0.011	99.90	
EOTAXIN	rs116237296	A	G	0.5284	0.1115	0.0159	2.15E-06	0.009	22.46	
EOTAXIN	rs113877493	T	C	-0.607	0.0217	0.8777	3.53E-172	0.079	782.45	
EOTAXIN	rs113010081	T	C	-0.5799	0.0236	0.1083	2.52E-133	0.065	603.78	
FGF	rs9903590	T	C	0.1281	0.0267	0.0974	1.60E-06	0.003	23.02	
FGF	rs78873483	A	G	0.1286	0.0282	0.0944	5.11E-06	0.003	20.80	
FGF	rs75168112	T	C	-0.1024	0.0214	0.1779	1.71E-06	0.003	22.90	5.00%
FGF	rs13412535	A	G	-0.1129	0.0224	0.2256	4.65E-07	0.004	25.40	
FGF	rs116745220	A	G	-0.6176	0.1324	0.9801	3.09E-06	0.015	21.76	
G-CSF	rs77318030	T	C	-0.2031	0.0427	0.0517	1.97E-06	0.004	22.62	
G-CSF	rs76287671	T	C	0.0894	0.0189	0.8091	2.24E-06	0.002	22.37	
G-CSF	rs74148555	T	C	-0.3771	0.0753	0.9473	5.50E-07	0.014	25.08	
G-CSF	rs586313	T	C	-0.0883	0.0187	0.2286	2.34E-06	0.003	22.30	6.00%
G-CSF	rs2671444	A	G	-0.0776	0.0166	0.3648	2.94E-06	0.003	21.85	
G-CSF	rs11903143	A	G	0.0889	0.0175	0.7425	3.77E-07	0.003	25.81	
G-CSF	rs117261691	T	C	0.1318	0.0288	0.9652	4.73E-06	0.001	20.94	
G-CSF	rs116745220	A	G	-0.6789	0.1359	0.9801	5.87E-07	0.018	24.96	
GROA	rs78653452	T	G	-0.7395	0.1559	0.9881	2.10E-06	0.013	22.50	
GROA	rs62024303	A	G	-0.3013	0.066	0.9543	4.99E-06	0.008	20.84	
GROA	rs493091	T	C	0.384	0.0279	0.2346	4.23E-43	0.053	189.43	7.00%
GROA	rs188345231	T	C	0.6177	0.1322	0.9881	2.98E-06	0.009	21.83	
GROA	rs140734053	A	G	0.7333	0.1545	0.0249	2.07E-06	0.026	22.53	
GROA	rs12075	A	G	0.3724	0.0236	0.6024	4.29E-56	0.066	249.00	

GROA	rs118158560	A	G	0.2761	0.0592	0.0626	3.10E-06	0.009	21.75	
GROA	rs114991247	T	C	-0.2202	0.0463	0.0596	1.98E-06	0.005	22.62	
GROA	rs1113500	T	G	0.1162	0.0243	0.3698	1.74E-06	0.006	22.87	
HGF	rs5745687	T	C	-0.3008	0.0404	0.7883	9.65E-14	0.030	55.44	
HGF	rs57146176	A	G	-0.0987	0.0208	0.9404	2.08E-06	0.001	22.52	
HGF	rs4245058	T	C	-0.1552	0.0331	0.9066	2.75E-06	0.004	21.99	
HGF	rs3748034	T	G	0.1529	0.0233	0.1362	5.30E-11	0.006	43.06	5.00%
HGF	rs2003620	T	C	0.2277	0.0487	0.0606	2.93E-06	0.006	21.86	
HGF	rs13412535	A	G	-0.1043	0.0213	0.2256	9.75E-07	0.004	23.98	
HGF	rs11060254	A	G	-0.0765	0.0166	0.3429	4.06E-06	0.003	21.24	
IFN-G	rs78296352	T	G	0.3419	0.065	0.9712	1.44E-07	0.007	27.67	
IFN-G	rs74148555	T	C	-0.3771	0.077	0.9473	9.71E-07	0.014	23.98	
IFN-G	rs60059008	A	G	0.0852	0.0176	0.6670	1.29E-06	0.003	23.43	
IFN-G	rs2073438	A	G	0.092	0.0188	0.2714	9.90E-07	0.003	23.95	
IFN-G	rs12420286	T	C	0.2357	0.05	0.0288	2.43E-06	0.003	22.22	5.00%
IFN-G	rs11843756	T	G	0.1812	0.0391	0.0288	3.58E-06	0.002	21.48	
IFN-G	rs115729819	A	G	0.2511	0.0514	0.9791	1.03E-06	0.003	23.87	
IFN-G	rs113600793	A	C	0.1871	0.0371	0.0398	4.58E-07	0.003	25.43	
IFN-G	rs10760686	T	C	0.0795	0.0168	0.6879	2.22E-06	0.003	22.39	
IL-10	rs7088799	T	G	-0.0815	0.0166	0.4344	9.12E-07	0.003	24.10	
IL-10	rs6921438	A	G	-0.2876	0.0166	0.4672	3.03E-67	0.041	300.17	
IL-10	rs6799107	T	C	-0.095	0.0206	0.2137	3.99E-06	0.003	21.27	
IL-10	rs6117725	T	C	0.0979	0.0202	0.2634	1.26E-06	0.004	23.49	5.00%
IL-10	rs41282660	A	G	-0.1169	0.0254	0.8678	4.18E-06	0.003	21.18	
IL-10	rs383684	A	G	0.092	0.0197	0.1183	3.01E-06	0.002	21.81	
IL-10	rs339203	T	C	0.0954	0.0203	0.9602	2.61E-06	0.001	22.09	

IL-10	rs3025021	T	C	0.0913	0.0194	0.6322	2.52E-06	0.004	22.15	
IL-10	rs282258	T	C	0.0993	0.0162	0.5746	8.81E-10	0.005	37.57	
IL-10	rs2086656	T	C	-0.08	0.017	0.3052	2.53E-06	0.003	22.15	
IL-10	rs1530455	T	C	0.082	0.0174	0.5915	2.45E-06	0.003	22.21	
IL-10	rs10493718	A	C	-0.1081	0.0222	0.2913	1.12E-06	0.005	23.71	
IL-10	rs10457128	A	G	-0.0854	0.0172	0.6392	6.87E-07	0.003	24.65	
IL-12	rs7088799	T	G	-0.0815	0.0166	0.4344	9.12E-07	0.003	24.10	
IL-12	rs6921438	A	G	-0.2876	0.0166	0.4672	3.03E-67	0.041	300.17	
IL-12	rs6799107	T	C	-0.095	0.0206	0.2137	3.99E-06	0.003	21.27	
IL-12	rs6117725	T	C	0.0979	0.0202	0.2634	1.26E-06	0.004	23.49	
IL-12	rs41282660	A	G	-0.1169	0.0254	0.8678	4.18E-06	0.003	21.18	
IL-12	rs383684	A	G	0.092	0.0197	0.1183	3.01E-06	0.002	21.81	
IL-12	rs339203	T	C	0.0954	0.0203	0.9602	2.61E-06	0.001	22.09	5.00%
IL-12	rs3025021	T	C	0.0913	0.0194	0.6322	2.52E-06	0.004	22.15	
IL-12	rs282258	T	C	0.0993	0.0162	0.5746	8.81E-10	0.005	37.57	
IL-12	rs2086656	T	C	-0.08	0.017	0.3052	2.53E-06	0.003	22.15	
IL-12	rs1530455	T	C	0.082	0.0174	0.5915	2.45E-06	0.003	22.21	
IL-12	rs10493718	A	C	-0.1081	0.0222	0.2913	1.12E-06	0.005	23.71	
IL-12	rs10457128	A	G	-0.0854	0.0172	0.6392	6.87E-07	0.003	24.65	
IL-13	rs9472168	A	G	0.4211	0.0246	0.5507	1.09E-65	0.088	293.02	
IL-13	rs77955971	A	C	0.4408	0.0868	0.0239	3.81E-07	0.009	25.79	
IL-13	rs73192842	A	G	0.1472	0.0299	0.2107	8.52E-07	0.007	24.24	
IL-13	rs7073807	T	C	0.1618	0.0354	0.8827	4.86E-06	0.005	20.89	6.00%
IL-13	rs27949	T	C	-0.1144	0.025	0.6312	4.74E-06	0.006	20.94	
IL-13	rs139083458	T	C	0.9995	0.211	0.9841	2.17E-06	0.031	22.44	
IL-13	rs138854806	A	G	-0.4204	0.0839	0.0119	5.42E-07	0.004	25.11	

IL-13	rs12623722	A	G	-0.1189	0.0257	0.2823	3.72E-06	0.006	21.40	
IL-13	rs117795020	A	G	-0.3584	0.0716	0.0179	5.57E-07	0.005	25.06	
IL-13	rs10995615	T	C	-0.1591	0.0341	0.1521	3.08E-06	0.007	21.77	
IL-16	rs9706053	T	C	0.4412	0.0928	0.0378	1.99E-06	0.014	22.60	
IL-16	rs4778636	A	G	-0.7286	0.063	0.0746	6.19E-31	0.073	133.75	
IL-16	rs4253283	T	C	0.1506	0.026	0.6998	6.94E-09	0.010	33.55	
IL-16	rs1801020	A	G	0.1678	0.0271	0.7753	5.95E-10	0.010	38.34	
IL-16	rs144691581	A	G	0.4929	0.0958	0.0149	2.67E-07	0.007	26.47	6.00%
IL-16	rs1255143	T	C	0.1387	0.0241	0.4344	8.65E-09	0.009	33.12	
IL-16	rs117916513	A	G	-0.4713	0.0982	0.0209	1.59E-06	0.009	23.03	
IL-16	rs117217798	T	C	-0.2064	0.044	0.9314	2.72E-06	0.005	22.00	
IL-16	rs116135478	A	G	0.8296	0.1637	0.9821	4.02E-07	0.024	25.68	
IL-17	rs78296352	T	G	0.2949	0.0645	0.9712	4.83E-06	0.005	20.90	
IL-17	rs184080173	T	C	0.236	0.0471	0.0626	5.43E-07	0.007	25.11	
IL-17	rs17282552	T	C	-0.2026	0.0403	0.0268	4.97E-07	0.002	25.27	
IL-17	rs1530455	T	C	0.1088	0.0173	0.5915	3.19E-10	0.006	39.55	6.00%
IL-17	rs12735700	T	G	-0.0943	0.0206	0.7515	4.70E-06	0.003	20.96	
IL-17	rs117556572	T	C	-0.5256	0.1097	0.9891	1.66E-06	0.006	22.96	
IL-17	rs113098509	A	G	-0.1553	0.0337	0.9235	4.06E-06	0.003	21.24	
IL-18	rs78716465	A	G	0.3173	0.0679	0.0378	2.97E-06	0.007	21.84	
IL-18	rs78623212	T	C	0.8322	0.1676	0.9761	6.86E-07	0.032	24.66	
IL-18	rs7444013	A	G	-0.5318	0.0955	0.9811	2.57E-08	0.010	31.01	
IL-18	rs71478720	T	C	-0.26	0.0273	0.7823	1.67E-21	0.023	90.70	5.00%
IL-18	rs610473	A	G	0.1274	0.0242	0.6501	1.41E-07	0.007	27.71	
IL-18	rs4482818	A	G	0.1233	0.0243	0.6412	3.89E-07	0.007	25.75	
IL-18	rs385076	T	C	-0.2472	0.0247	0.6471	1.40E-23	0.028	100.16	

IL-18	rs1979967	T	C	0.14	0.0285	0.7406	9.00E-07	0.008	24.13	
IL-18	rs17229943	A	C	-0.3076	0.0463	0.9503	3.06E-11	0.009	44.14	
IL-18	rs117266781	T	C	0.7051	0.1436	0.9891	9.10E-07	0.011	24.11	
IL-18	rs116383510	A	C	-0.5412	0.1052	0.9801	2.68E-07	0.011	26.47	
IL-18	rs10414552	T	C	-0.1817	0.0347	0.8787	1.64E-07	0.007	27.42	
IL-1B	rs62015704	A	G	0.1786	0.0372	0.8787	1.58E-06	0.007	23.05	
IL-1B	rs61335305	A	C	0.4333	0.0928	0.0109	3.02E-06	0.004	21.80	6.00%
IL-1B	rs143319329	T	C	0.4357	0.093	0.9761	2.80E-06	0.009	21.95	
IL-1RA	rs6699436	A	G	-0.1858	0.0404	0.1431	4.25E-06	0.008	21.15	
IL-1RA	rs61335305	A	C	0.4315	0.0904	0.0109	1.81E-06	0.004	22.78	
IL-1RA	rs56134659	A	G	-0.1109	0.0236	0.5258	2.61E-06	0.006	22.08	19.00%
IL-1RA	rs4441609	T	C	0.1056	0.0231	0.6302	4.84E-06	0.005	20.90	
IL-1RA	rs11627423	A	C	0.1178	0.0246	0.6103	1.68E-06	0.007	22.93	
IL-1RA	rs1054402	T	C	0.1325	0.0269	0.7336	8.41E-07	0.007	24.26	
IL-2	rs7615304	A	G	-0.1139	0.024	0.3489	2.08E-06	0.006	22.52	
IL-2	rs62124990	T	G	-0.7013	0.149	0.9841	2.52E-06	0.015	22.15	
IL-2	rs61335305	A	C	0.4439	0.0913	0.0109	1.16E-06	0.004	23.64	
IL-2	rs4634519	A	G	-0.1249	0.0268	0.6879	3.16E-06	0.007	21.72	5.00%
IL-2	rs2690020	A	G	0.1158	0.0245	0.5547	2.28E-06	0.007	22.34	
IL-2	rs170117	T	C	-0.1637	0.0347	0.8797	2.39E-06	0.006	22.26	
IL-2	rs16836080	A	G	0.1158	0.0253	0.2922	4.72E-06	0.006	20.95	
IL-2	rs13412535	A	G	0.174	0.0331	0.2256	1.47E-07	0.011	27.63	
IL-2RA	rs56213152	T	C	0.1269	0.0271	0.2604	2.83E-06	0.006	21.93	
IL-2RA	rs4733117	A	C	0.1439	0.0291	0.8360	7.61E-07	0.006	24.45	7.00%
IL-2RA	rs185231391	T	C	0.8568	0.1803	0.0149	2.01E-06	0.022	22.58	
IL-2RA	rs12799226	T	C	-0.1285	0.0277	0.8091	3.50E-06	0.005	21.52	

IL-2RA	rs12722497	A	C	0.6287	0.0482	0.8559	6.91E-39	0.097	170.13	
IL-2RA	rs115360066	A	G	0.1776	0.0377	0.8897	2.47E-06	0.006	22.19	
IL-2RA	rs11241559	T	G	-0.124	0.0264	0.7813	2.64E-06	0.005	22.06	
IL-4	rs9941733	A	G	0.1156	0.0229	0.825	4.46E-07	0.004	25.48	
IL-4	rs9508291	T	C	-0.168	0.0358	0.5109	2.70E-06	0.014	22.02	
IL-4	rs79597994	T	C	-0.5855	0.1271	0.9722	4.09E-06	0.019	21.22	
IL-4	rs7613691	A	G	0.1787	0.0382	0.9473	2.90E-06	0.003	21.88	
IL-4	rs73023729	A	G	-0.1796	0.0365	0.0229	8.63E-07	0.001	24.21	
IL-4	rs6765768	A	G	0.0796	0.0167	0.6083	1.87E-06	0.003	22.72	5.00%
IL-4	rs2708550	T	C	-0.0764	0.0165	0.7803	3.65E-06	0.002	21.44	
IL-4	rs2073438	A	G	0.0847	0.0183	0.2714	3.68E-06	0.003	21.42	
IL-4	rs17713451	A	G	0.1255	0.0252	0.1650	6.35E-07	0.004	24.80	
IL-4	rs12238729	T	C	0.5271	0.1096	0.9881	1.51E-06	0.007	23.13	
IL-4	rs116705532	T	G	-0.4675	0.0978	0.0169	1.75E-06	0.007	22.85	
IL-4	rs10512267	T	C	-0.0824	0.016	0.3300	2.60E-07	0.003	26.52	
IL-5	rs74811276	A	G	0.217	0.0471	0.0686	4.08E-06	0.006	21.23	
IL-5	rs73040130	T	C	0.2745	0.0525	0.0646	1.71E-07	0.009	27.34	
IL-5	rs72831687	A	G	-0.5337	0.1104	0.0169	1.34E-06	0.009	23.37	
IL-5	rs6737109	T	C	0.1135	0.0246	0.4254	3.95E-06	0.006	21.29	5.00%
IL-5	rs4320361	T	G	-0.1553	0.025	0.5477	5.23E-10	0.012	38.59	
IL-5	rs28793375	T	C	0.1697	0.0362	0.8330	2.76E-06	0.008	21.98	
IL-5	rs148634917	A	G	-0.517	0.1087	0.9811	1.97E-06	0.010	22.62	
IL-5	rs11680908	A	G	0.2593	0.0552	0.9453	2.63E-06	0.007	22.07	
IL-6	rs76856708	T	C	0.336	0.0697	0.0378	1.43E-06	0.008	23.24	
IL-6	rs73273528	T	C	0.268	0.0553	0.9662	1.26E-06	0.005	23.49	5.00%
IL-6	rs72831623	A	G	0.197	0.0369	0.0527	9.36E-08	0.004	28.50	

IL-6	rs2404476	A	G	0.0734	0.0156	0.4722	2.54E-06	0.003	22.14	
IL-6	rs13412535	A	G	-0.1186	0.0214	0.2256	2.99E-08	0.005	30.71	
IL-6	rs1333040	T	C	0.0747	0.0157	0.4264	1.96E-06	0.003	22.64	
IL-6	rs113098456	A	G	-0.1553	0.0339	0.0765	4.62E-06	0.003	20.99	
IL-6	rs10817609	T	C	0.0845	0.0175	0.2157	1.38E-06	0.002	23.32	
IL-7	rs78346957	A	G	0.4632	0.1008	0.0229	4.32E-06	0.010	21.12	
IL-7	rs77981494	T	C	-0.5201	0.1055	0.0159	8.23E-07	0.008	24.30	
IL-7	rs6921438	A	G	-0.3204	0.0246	0.4672	8.89E-39	0.051	169.63	
IL-7	rs62006410	T	C	-0.1492	0.0302	0.7853	7.80E-07	0.008	24.41	
IL-7	rs28793375	T	C	0.1644	0.036	0.8330	4.96E-06	0.008	20.85	
IL-7	rs1958987	T	C	0.1261	0.0263	0.6998	1.63E-06	0.007	22.99	6.00%
IL-7	rs17091524	T	C	0.5092	0.1015	0.0427	5.26E-07	0.021	25.17	
IL-7	rs141425475	T	C	-0.4801	0.1018	0.0288	2.40E-06	0.013	22.24	
IL-7	rs117509142	T	C	-0.3213	0.0684	0.0567	2.64E-06	0.011	22.07	
IL-7	rs115215018	T	C	0.5985	0.1308	0.9881	4.75E-06	0.008	20.94	
IL-7	rs10196226	A	G	0.1538	0.0327	0.1471	2.56E-06	0.006	22.12	
IL-8	rs75840288	A	C	0.5125	0.1121	0.9702	4.84E-06	0.015	20.90	
IL-8	rs3786107	A	G	0.2463	0.0517	0.0596	1.90E-06	0.007	22.70	
IL-8	rs2673604	A	C	-0.118	0.0254	0.2992	3.39E-06	0.006	21.58	
IL-8	rs183628733	T	C	0.6547	0.1417	0.0219	3.83E-06	0.018	21.35	9.00%
IL-8	rs141926526	A	C	-0.6221	0.1308	0.9523	1.97E-06	0.035	22.62	
IL-8	rs12438669	A	C	-0.1182	0.0252	0.6571	2.73E-06	0.006	22.00	
IL-8	rs12075	A	G	0.1148	0.0235	0.6024	1.03E-06	0.006	23.86	
IL-8	rs116726256	T	C	-0.2247	0.0489	0.0318	4.33E-06	0.003	21.11	
IL-9	rs76963786	T	C	-0.2856	0.0556	0.9135	2.80E-07	0.013	26.39	5.00%
IL-9	rs73443903	A	C	0.2162	0.046	0.1004	2.60E-06	0.008	22.09	

IL-9	rs4880409	T	C	-0.3552	0.0716	0.0278	7.02E-07	0.007	24.61	
IL-9	rs41294750	T	C	0.3442	0.0736	0.9722	2.92E-06	0.006	21.87	
IP-10	rs9450351	T	C	-0.2651	0.0488	0.0616	5.56E-08	0.008	29.51	
IP-10	rs8112909	A	G	-0.139	0.0297	0.2594	2.87E-06	0.007	21.90	
IP-10	rs79848609	A	C	0.2514	0.0535	0.84	2.61E-06	0.017	22.08	
IP-10	rs7645625	T	G	-0.1116	0.0236	0.4324	2.26E-06	0.006	22.36	6.00%
IP-10	rs6707974	A	G	0.1574	0.0337	0.8380	3.00E-06	0.007	21.81	
IP-10	rs4862110	T	C	-0.1453	0.0318	0.1869	4.90E-06	0.006	20.88	
IP-10	rs34383175	T	C	-0.3196	0.0653	0.9732	9.86E-07	0.005	23.95	
IP-10	rs113831257	A	G	0.3639	0.0641	0.0417	1.37E-08	0.011	32.23	
MCP-1-MCAF	rs9317045	A	C	0.1157	0.0235	0.8539	8.51E-07	0.003	24.24	
MCP-1-MCAF	rs7632755	A	G	0.2984	0.0315	0.0805	2.72E-21	0.013	89.74	
MCP-1-MCAF	rs7517040	A	G	-0.097	0.019	0.2823	3.30E-07	0.004	26.06	
MCP-1-MCAF	rs7197349	A	G	0.0971	0.0206	0.8628	2.43E-06	0.002	22.22	
MCP-1-MCAF	rs7019112	T	G	-0.2206	0.0468	0.0119	2.43E-06	0.001	22.22	
MCP-1-MCAF	rs56212190	T	C	0.1799	0.0372	0.9453	1.32E-06	0.003	23.39	
MCP-1-MCAF	rs2288370	T	C	-0.1036	0.0162	0.3956	1.60E-10	0.005	40.90	5.00%
MCP-1-MCAF	rs2036297	A	G	0.1182	0.016	0.3429	1.50E-13	0.006	54.58	
MCP-1-MCAF	rs146522229	T	C	-0.5942	0.1161	0.9761	3.09E-07	0.016	26.19	
MCP-1-MCAF	rs12075	A	G	0.2186	0.0154	0.6024	9.87E-46	0.023	201.49	
MCP-1-MCAF	rs111995966	T	G	0.1428	0.0309	0.0258	3.81E-06	0.001	21.36	
MCP-1-MCAF	rs11118557	A	G	0.148	0.0319	0.9264	3.49E-06	0.003	21.52	
MCP-1-MCAF	rs10744620	T	C	0.0783	0.0161	0.6392	1.15E-06	0.003	23.65	
MCP-3	rs62492260	T	G	-0.2802	0.0578	0.8509	1.25E-06	0.020	23.50	
MCP-3	rs3129806	T	C	-0.1975	0.0433	0.6322	5.09E-06	0.018	20.80	5.00%
MCP-3	rs2838065	A	G	-0.221	0.0479	0.1968	3.95E-06	0.015	21.29	

MCP-3	rs117286643	A	G	0.6934	0.1474	0.0109	2.55E-06	0.010	22.13	
MCP-3	rs10892381	T	C	0.2432	0.0473	0.3002	2.72E-07	0.025	26.44	
M-CSF	rs9387100	T	C	-0.135	0.029	0.6730	3.24E-06	0.008	21.67	
M-CSF	rs78296352	T	G	0.522	0.111	0.9712	2.57E-06	0.015	22.12	
M-CSF	rs72723242	T	G	-0.4969	0.1083	0.9374	4.47E-06	0.029	21.05	
M-CSF	rs62294910	A	G	0.3472	0.0687	0.0527	4.33E-07	0.012	25.54	
M-CSF	rs56367447	T	C	-0.4878	0.0876	0.9662	2.57E-08	0.016	31.01	5.00%
M-CSF	rs34089869	T	C	0.2194	0.0462	0.8976	2.05E-06	0.009	22.55	
M-CSF	rs12962919	T	C	0.3025	0.0659	0.9066	4.43E-06	0.015	21.07	
M-CSF	rs117867915	T	C	0.5224	0.1096	0.0139	1.88E-06	0.007	22.72	
M-CSF	rs116274860	T	G	0.8262	0.1739	0.0159	2.02E-06	0.021	22.57	
MIF	rs78098071	T	C	-0.4583	0.0915	0.0189	5.48E-07	0.008	25.09	
MIF	rs3814097	A	G	-0.1163	0.0251	0.4751	3.60E-06	0.007	21.47	
MIF	rs35890933	T	G	0.1676	0.0365	0.2008	4.39E-06	0.009	21.08	6.00%
MIF	rs13142904	T	C	-0.2232	0.0425	0.9314	1.51E-07	0.006	27.58	
MIF	rs12594190	A	G	0.1321	0.0266	0.7535	6.83E-07	0.006	24.66	
MIG	rs816960	T	C	-0.1179	0.0242	0.8539	1.11E-06	0.003	23.74	
MIG	rs77086208	T	C	0.327	0.0694	0.9811	2.46E-06	0.004	22.20	
MIG	rs6679677	A	C	0.1628	0.0327	0.0915	6.40E-07	0.004	24.79	
MIG	rs62562991	A	G	0.6239	0.1259	0.0199	7.21E-07	0.015	24.56	
MIG	rs1796086	T	C	-0.2172	0.04	0.0944	5.64E-08	0.008	29.48	8.00%
MIG	rs139010077	T	C	0.4337	0.0943	0.9891	4.24E-06	0.004	21.15	
MIG	rs117831247	T	C	-0.8819	0.173	0.9811	3.44E-07	0.029	25.99	
MIG	rs112861654	A	G	-0.2682	0.0527	0.9135	3.60E-07	0.011	25.90	
MIG	rs11177248	A	G	0.3157	0.0667	0.0606	2.21E-06	0.011	22.40	
MIG	rs111607343	A	G	-0.5235	0.1119	0.0378	2.89E-06	0.020	21.89	

MIP-1A	rs6900267	A	C	-0.2472	0.0515	0.9254	1.59E-06	0.008	23.04	
MIP-1A	rs60198979	A	G	-0.2154	0.0455	0.0865	2.20E-06	0.007	22.41	
MIP-1A	rs57786342	A	G	0.139	0.0283	0.2157	9.03E-07	0.007	24.12	5.00%
MIP-1A	rs184154340	A	G	0.3251	0.0689	0.0408	2.38E-06	0.008	22.26	
MIP-1A	rs12690897	A	G	0.1215	0.026	0.2813	2.97E-06	0.006	21.84	
MIP-1A	rs116615337	A	G	0.1286	0.0278	0.7087	3.73E-06	0.007	21.40	
MIP-1B	rs9793308	A	G	0.0842	0.0177	0.9841	1.96E-06	0.000	22.63	
MIP-1B	rs77668982	A	G	0.3274	0.0389	0.0179	3.88E-17	0.004	70.84	
MIP-1B	rs76776296	A	G	0.313	0.0598	0.9632	1.66E-07	0.007	27.40	
MIP-1B	rs76582507	A	G	0.3259	0.0676	0.0268	1.43E-06	0.006	23.24	
MIP-1B	rs72799710	T	C	-0.1037	0.0217	0.8340	1.76E-06	0.003	22.84	
MIP-1B	rs72791296	T	C	0.2364	0.0466	0.9414	3.92E-07	0.006	25.73	
MIP-1B	rs6908843	A	G	0.0997	0.0209	0.1809	1.84E-06	0.003	22.76	
MIP-1B	rs5743614	T	C	0.1115	0.0232	0.2744	1.54E-06	0.005	23.10	5.00%
MIP-1B	rs281749	T	C	0.0795	0.0171	0.6312	3.33E-06	0.003	21.61	
MIP-1B	rs17138331	A	G	-0.1434	0.0295	0.9056	1.17E-06	0.004	23.63	
MIP-1B	rs117657747	A	G	0.2089	0.0453	0.0656	4.00E-06	0.005	21.27	
MIP-1B	rs117453826	A	G	-0.5907	0.0591	0.9443	1.60E-23	0.037	99.90	
MIP-1B	rs116237296	A	G	0.5284	0.1115	0.0159	2.15E-06	0.009	22.46	
MIP-1B	rs113877493	T	C	-0.607	0.0217	0.8777	3.53E-172	0.079	782.45	
MIP-1B	rs113010081	T	C	-0.5799	0.0236	0.1083	2.52E-133	0.065	603.78	
PDGF-BB	rs9941733	A	G	0.1165	0.0227	0.825	2.86E-07	0.004	26.34	
PDGF-BB	rs73162807	A	C	-0.2313	0.0499	0.0129	3.56E-06	0.001	21.49	
PDGF-BB	rs72777070	T	G	-0.1048	0.02	0.1998	1.61E-07	0.004	27.46	5.00%
PDGF-BB	rs55680718	T	C	-0.1359	0.0245	0.8628	2.91E-08	0.004	30.77	
PDGF-BB	rs4965869	T	C	0.1843	0.0181	0.7237	2.38E-24	0.014	103.68	

PDGF-BB	rs35859699	A	G	-0.3854	0.0838	0.0229	4.24E-06	0.007	21.15	
PDGF-BB	rs2324229	T	C	0.0884	0.0161	0.3936	4.00E-08	0.004	30.15	
PDGF-BB	rs13412535	A	G	0.3317	0.0214	0.2256	3.47E-54	0.038	240.25	
PDGF-BB	rs12289510	A	G	-0.0772	0.0158	0.4672	1.03E-06	0.003	23.87	
PDGF-BB	rs11766649	A	G	0.0902	0.0196	0.7386	4.18E-06	0.003	21.18	
PDGF-BB	rs116445074	T	G	0.2869	0.0587	0.9841	1.02E-06	0.003	23.89	
RANTES	rs9675798	T	G	-0.2583	0.0552	0.0646	2.88E-06	0.008	21.90	
RANTES	rs74472919	T	C	0.3547	0.06	0.3091	3.39E-09	0.054	34.95	
RANTES	rs72793342	A	G	-0.1505	0.0307	0.2078	9.47E-07	0.007	24.03	
RANTES	rs62438851	A	G	-0.1904	0.0413	0.8648	4.02E-06	0.008	21.25	
RANTES	rs2731672	T	C	-0.1242	0.0272	0.2266	4.97E-06	0.005	20.85	8.00%
RANTES	rs2251660	A	C	0.1831	0.0356	0.1451	2.70E-07	0.008	26.45	
RANTES	rs147509526	T	C	-0.3558	0.0715	0.9871	6.48E-07	0.003	24.76	
RANTES	rs112072646	A	G	0.4209	0.0859	0.0308	9.59E-07	0.011	24.01	
RANTES	rs10505135	T	C	0.1315	0.0252	0.6759	1.81E-07	0.008	27.23	
SCF	rs80271436	A	G	-0.2393	0.0484	0.0497	7.65E-07	0.005	24.45	
SCF	rs78666213	T	G	-0.2845	0.0574	0.0258	7.18E-07	0.004	24.57	
SCF	rs78369473	T	C	-0.2256	0.0484	0.9712	3.14E-06	0.003	21.73	
SCF	rs7535409	A	G	-0.1173	0.0169	0.6849	3.90E-12	0.006	48.18	
SCF	rs7037688	A	G	-0.0786	0.0167	0.2982	2.52E-06	0.003	22.15	
SCF	rs507666	A	G	-0.1035	0.0191	0.1859	6.00E-08	0.003	29.36	5.00%
SCF	rs4841895	A	G	0.1004	0.0178	0.3231	1.70E-08	0.004	31.81	
SCF	rs1568119	T	C	-0.5946	0.1129	0.9871	1.39E-07	0.009	27.74	
SCF	rs13412535	A	G	-0.1065	0.0213	0.2256	5.73E-07	0.004	25.00	
SCF	rs113127926	A	C	0.1974	0.0418	0.0626	2.33E-06	0.005	22.30	
SCGF	rs78217154	T	C	0.3942	0.0861	0.0229	4.69E-06	0.007	20.96	5.00%

SCGF	rs7815967	T	C	0.1325	0.0288	0.8807	4.21E-06	0.004	21.17	
SCGF	rs77954165	T	C	0.2631	0.0562	0.0755	2.85E-06	0.010	21.92	
SCGF	rs4656185	A	G	0.2103	0.0254	0.3151	1.24E-16	0.019	68.55	
SCGF	rs34911860	A	G	-0.3674	0.0787	0.0119	3.04E-06	0.003	21.79	
SCGF	rs264157	A	G	0.1079	0.0233	0.4702	3.64E-06	0.006	21.45	
SCGF	rs17876031	A	G	-0.1496	0.0254	0.6889	3.87E-09	0.010	34.69	
SCGF	rs151194174	A	G	0.4536	0.0941	0.0209	1.43E-06	0.008	23.24	
SCGF	rs150733161	T	C	-0.5255	0.112	0.9791	2.71E-06	0.011	22.01	
SCGF	rs149009264	A	G	0.4551	0.0985	0.9811	3.83E-06	0.008	21.35	
SCGF	rs143829871	T	C	-0.1866	0.0399	0.0596	2.92E-06	0.004	21.87	
SCGF	rs139413256	A	G	-0.5174	0.1076	0.0239	1.52E-06	0.012	23.12	
SCGF	rs12480722	T	C	0.1654	0.0353	0.0974	2.79E-06	0.005	21.95	
SCGF	rs117716477	A	C	0.8242	0.084	0.0209	1.00E-22	0.028	96.27	
SCGF	rs116924815	T	C	0.6046	0.0737	0.9642	2.33E-16	0.025	67.30	
SCGF	rs1149926	T	C	-0.3458	0.0749	0.9672	3.90E-06	0.008	21.32	
SCGF	rs112346514	T	C	-0.3261	0.0703	0.9761	3.51E-06	0.005	21.52	
SDF-1A	rs62194947	T	C	-0.0852	0.0185	0.7376	4.12E-06	0.003	21.21	
SDF-1A	rs3988298	T	C	-0.1263	0.0266	0.8499	2.05E-06	0.004	22.54	
SDF-1A	rs1600396	A	G	-0.0933	0.0204	0.7197	4.80E-06	0.004	20.92	5.00%
SDF-1A	rs149893336	A	G	-0.494	0.1082	0.9801	4.98E-06	0.010	20.84	
SDF-1A	rs12141941	T	C	-0.0881	0.0186	0.2654	2.17E-06	0.003	22.43	
SDF-1A	rs10474392	A	G	0.0934	0.0177	0.2694	1.31E-07	0.003	27.84	
TNF-A	rs79105320	A	G	0.5573	0.1177	0.0209	2.19E-06	0.013	22.42	
TNF-A	rs111332265	A	G	-0.3678	0.0745	0.9284	7.94E-07	0.018	24.37	7.00%
TNF-A	rs10834996	A	G	0.123	0.0256	0.3598	1.55E-06	0.007	23.09	
TNF-B	rs78296352	T	G	1.2028	0.1366	0.9712	1.31E-18	0.081	77.53	5.00%

TNF-B	rs7629875	A	G	0.3841	0.0774	0.9423	6.96E-07	0.016	24.63	
TNF-B	rs2080926	T	C	0.1725	0.037	0.4642	3.13E-06	0.015	21.74	
TNF-B	rs10925040	T	C	0.1738	0.0372	0.6113	2.98E-06	0.014	21.83	
TRAIL	rs79287178	A	G	-0.4304	0.042	0.0258	1.21E-24	0.009	105.01	
TRAIL	rs75928541	A	G	0.2784	0.0591	0.0169	2.47E-06	0.003	22.19	
TRAIL	rs74778900	T	C	0.5791	0.0531	0.9861	1.08E-27	0.009	118.94	
TRAIL	rs747324	T	C	-0.0826	0.0178	0.6412	3.48E-06	0.003	21.53	
TRAIL	rs73039026	A	C	-0.3098	0.0634	0.9841	1.03E-06	0.003	23.88	
TRAIL	rs72899452	T	C	0.1223	0.0264	0.9235	3.61E-06	0.002	21.46	5.00%
TRAIL	rs62093514	T	C	1.0459	0.0549	0.9742	6.45E-81	0.055	362.94	
TRAIL	rs57396456	T	C	-0.5641	0.0516	0.0338	8.09E-28	0.021	119.51	
TRAIL	rs193112415	T	C	-1.0456	0.062	0.0139	8.21E-64	0.030	284.41	
TRAIL	rs17434886	T	C	-0.0918	0.0199	0.8231	3.97E-06	0.002	21.28	
TRAIL	rs148051545	T	C	-0.4211	0.0843	0.9871	5.88E-07	0.005	24.95	
TRAIL	rs13278062	T	G	0.08	0.0157	0.497	3.48E-07	0.003	25.96	
VEGF	rs9472183	A	G	-0.1264	0.017	0.5109	1.04E-13	0.008	55.28	
VEGF	rs8045833	A	G	0.103	0.0211	0.2634	1.05E-06	0.004	23.83	
VEGF	rs73872715	T	C	-0.6079	0.1299	0.9801	2.87E-06	0.014	21.90	
VEGF	rs73418461	A	G	-0.2498	0.0521	0.0606	1.63E-06	0.007	22.99	
VEGF	rs6921438	A	G	-0.4866	0.0174	0.4672	4.27E-172	0.118	782.07	
VEGF	rs4082730	A	G	0.2455	0.0533	0.9543	4.10E-06	0.005	21.22	5.00%
VEGF	rs3108686	A	C	-0.7967	0.1702	0.9831	2.86E-06	0.021	21.91	
VEGF	rs143479231	A	G	-0.2628	0.0489	0.0219	7.69E-08	0.003	28.88	
VEGF	rs13209117	A	G	0.1253	0.02	0.2843	3.73E-10	0.006	39.25	
VEGF	rs12456390	T	C	-0.0818	0.0179	0.3171	4.88E-06	0.003	20.88	
VEGF	rs10967186	T	C	0.0899	0.0169	0.4324	1.04E-07	0.004	28.30	

VEGF	rs10934631	T	C	-0.1132	0.0244	0.1799	3.50E-06	0.004	21.52

Supplementary Table 10. Detailed information for circulating cytokines on age-related neurodegenerative diseases.

Exposure	Outcome	N SNPs	Method	OR	OR-LCI	OR-UCI	*P* value
BNGF	AD	4	MR-Egger	1.045	0.971	1.125	0.258
BNGF	AD	4	Weighted median	1.011	0.875	1.168	0.883
BNGF	AD	4	Inverse variance weighted	1.005	0.889	1.137	0.936
BNGF	AD	4	MR-PRESSO	/	/	/	0.932
CTACK	AD	12	MR-Egger	1.016	0.918	1.123	0.771
CTACK	AD	12	Weighted median	1.013	0.936	1.097	0.746
CTACK	AD	12	Inverse variance weighted	1.034	0.978	1.092	0.243
CTACK	AD	12	MR-PRESSO	/	/	/	0.508
EOTAXIN	AD	15	MR-Egger	1.024	0.873	1.202	0.771
EOTAXIN	AD	15	Weighted median	0.992	0.901	1.091	0.863
EOTAXIN	AD	15	Inverse variance weighted	1.012	0.943	1.086	0.74
EOTAXIN	AD	15	MR-PRESSO	/	/	/	0.851
bFGF	AD	6	MR-Egger	1.172	0.883	1.555	0.334
bFGF	AD	6	Weighted median	1.08	0.905	1.29	0.393
bFGF	AD	6	Inverse variance weighted	1.012	0.861	1.191	0.881
bFGF	AD	6	MR-PRESSO	/	/	/	0.947
G-CSF	AD	8	MR-Egger	1.014	0.831	1.238	0.892
G-CSF	AD	8	Weighted median	1.058	0.906	1.235	0.479
G-CSF	AD	8	Inverse variance weighted	1.03	0.918	1.155	0.619
G-CSF	AD	8	MR-PRESSO	/	/	/	0.897
GROA	AD	9	MR-Egger	1.03	0.922	1.151	0.618
GROA	AD	9	Weighted median	1.024	0.965	1.088	0.432
GROA	AD	9	Inverse variance weighted	0.999	0.953	1.047	0.952

GROA	AD	9	MR-PRESSO	/	/	/	0.718
HGF	AD	7	MR-Egger	0.974	0.761	1.246	0.841
HGF	AD	7	Weighted median	0.946	0.83	1.078	0.406
HGF	AD	7	Inverse variance weighted	0.934	0.836	1.043	0.225
HGF	AD	7	MR-PRESSO	/	/	/	0.348
IFN-G	AD	9	MR-Egger	0.97	0.679	1.386	0.872
IFN-G	AD	9	Weighted median	0.901	0.765	1.063	0.216
IFN-G	AD	9	Inverse variance weighted	0.91	0.762	1.086	0.294
IFN-G	AD	9	MR-PRESSO	/	/	/	0.215
IL-1B	AD	4	MR-Egger	1.096	0.792	1.516	0.635
IL-1B	AD	4	Weighted median	0.946	0.823	1.089	0.441
IL-1B	AD	4	Inverse variance weighted	0.956	0.852	1.072	0.443
IL-1B	AD	4	MR-PRESSO	/	/	/	0.319
IL-1RA	AD	8	MR-Egger	0.985	0.532	1.824	0.964
IL-1RA	AD	8	Weighted median	1.066	0.934	1.218	0.344
IL-1RA	AD	8	Inverse variance weighted	1.014	0.85	1.209	0.876
IL-1RA	AD	8	MR-PRESSO	/	/	/	0.913
IL-2	AD	8	MR-Egger	1.132	0.97	1.32	0.167
IL-2	AD	8	Weighted median	1.088	0.975	1.213	0.132
IL-2	AD	8	Inverse variance weighted	1.069	0.988	1.157	0.097
IL-2	AD	8	MR-PRESSO	/	/	/	0.07
IL-2RA	AD	7	MR-Egger	0.914	0.833	1.003	0.117
IL-2RA	AD	7	Weighted median	0.966	0.904	1.033	0.313
IL-2RA	AD	7	Inverse variance weighted	0.993	0.915	1.078	0.862
IL-2RA	AD	7	MR-PRESSO	/	/	/	0.672
IL-4	AD	12	MR-Egger	0.982	0.845	1.141	0.814

IL-4	AD	12	Weighted median	0.987	0.886	1.099	0.809
IL-4	AD	12	Inverse variance weighted	0.972	0.884	1.068	0.548
IL-4	AD	12	MR-PRESSO	/	/	/	0.522
IL-5	AD	7	MR-Egger	0.857	0.693	1.06	0.214
IL-5	AD	7	Weighted median	0.903	0.806	1.012	0.078
IL-5	AD	7	Inverse variance weighted	0.909	0.832	0.993	**0.035**
IL-5	AD	7	MR-PRESSO	/	/	/	0.339
IL-6	AD	9	MR-Egger	0.797	0.559	1.136	0.25
IL-6	AD	9	Weighted median	0.894	0.756	1.058	0.193
IL-6	AD	9	Inverse variance weighted	0.954	0.797	1.141	0.604
IL-6	AD	9	MR-PRESSO	/	/	/	0.448
IL-7	AD	11	MR-Egger	1.066	0.921	1.233	0.416
IL-7	AD	11	Weighted median	0.956	0.884	1.033	0.251
IL-7	AD	11	Inverse variance weighted	1.003	0.939	1.071	0.936
IL-7	AD	11	MR-PRESSO	/	/	/	0.937
IL-8	AD	8	MR-Egger	0.943	0.837	1.063	0.373
IL-8	AD	8	Weighted median	1.008	0.914	1.111	0.875
IL-8	AD	8	Inverse variance weighted	1.018	0.933	1.111	0.684
IL-8	AD	8	MR-PRESSO	/	/	/	0.697
IL-9	AD	4	MR-Egger	1.485	0.71	3.107	0.403
IL-9	AD	4	Weighted median	1.039	0.9	1.199	0.604
IL-9	AD	4	Inverse variance weighted	1.079	0.947	1.229	0.252
IL-9	AD	4	MR-PRESSO	/	/	/	0.546
IL-10	AD	13	MR-Egger	0.943	0.81	1.097	0.463
IL-10	AD	13	Weighted median	0.941	0.86	1.03	0.189
IL-10	AD	13	Inverse variance weighted	0.946	0.88	1.017	0.135

IL-10	AD	13	MR-PRESSO	/	/	/	0.125
IL-12	AD	12	MR-Egger	0.957	0.855	1.07	0.457
IL-12	AD	12	Weighted median	0.964	0.897	1.035	0.311
IL-12	AD	12	Inverse variance weighted	0.972	0.913	1.036	0.387
IL-12	AD	12	MR-PRESSO	/	/	/	0.18
IL-13	AD	9	MR-Egger	0.965	0.884	1.052	0.446
IL-13	AD	9	Weighted median	0.961	0.906	1.02	0.195
IL-13	AD	9	Inverse variance weighted	0.977	0.928	1.028	0.365
IL-13	AD	9	MR-PRESSO	/	/	/	0.497
IL-16	AD	9	MR-Egger	0.963	0.89	1.041	0.369
IL-16	AD	9	Weighted median	0.984	0.925	1.048	0.625
IL-16	AD	9	Inverse variance weighted	0.983	0.936	1.034	0.51
IL-16	AD	9	MR-PRESSO	/	/	/	0.347
IL-17	AD	7	MR-Egger	0.902	0.683	1.191	0.5
IL-17	AD	7	Weighted median	0.901	0.769	1.055	0.196
IL-17	AD	7	Inverse variance weighted	0.95	0.843	1.07	0.398
IL-17	AD	7	MR-PRESSO	/	/	/	0.207
IL-18	AD	11	MR-Egger	1.1	0.948	1.278	0.241
IL-18	AD	11	Weighted median	1.034	0.953	1.122	0.425
IL-18	AD	11	Inverse variance weighted	1.001	0.926	1.082	0.976
IL-18	AD	11	MR-PRESSO	/	/	/	0.799
IP-10	AD	9	MR-Egger	0.865	0.728	1.027	0.141
IP-10	AD	9	Weighted median	0.941	0.844	1.049	0.271
IP-10	AD	9	Inverse variance weighted	0.957	0.881	1.04	0.302
IP-10	AD	9	MR-PRESSO	/	/	/	0.213
M-CSF	AD	8	MR-Egger	0.981	0.867	1.109	0.767

M-CSF	AD	8	Weighted median	0.98	0.906	1.059	0.606
M-CSF	AD	8	Inverse variance weighted	0.988	0.929	1.05	0.689
M-CSF	AD	8	MR-PRESSO	/	/	/	0.359
MCP-1-MCAF	AD	14	MR-Egger	1.042	0.858	1.265	0.688
MCP-1-MCAF	AD	14	Weighted median	1.043	0.935	1.164	0.452
MCP-1-MCAF	AD	14	Inverse variance weighted	1.054	0.967	1.148	0.234
MCP-1-MCAF	AD	14	MR-PRESSO	/	/	/	0.255
MCP-3	AD	5	MR-Egger	1.016	0.75	1.376	0.924
MCP-3	AD	5	Weighted median	0.992	0.907	1.086	0.868
MCP-3	AD	5	Inverse variance weighted	1	0.926	1.08	0.998
MCP-3	AD	5	MR-PRESSO	/	/	/	0.755
MIF	AD	6	MR-Egger	0.831	0.651	1.061	0.212
MIF	AD	6	Weighted median	0.945	0.82	1.089	0.435
MIF	AD	6	Inverse variance weighted	0.971	0.85	1.108	0.659
MIF	AD	6	MR-PRESSO	/	/	/	0.569
MIG	AD	9	MR-Egger	1.049	0.896	1.229	0.569
MIG	AD	9	Weighted median	0.942	0.853	1.042	0.245
MIG	AD	9	Inverse variance weighted	0.995	0.915	1.083	0.913
MIG	AD	9	MR-PRESSO	/	/	/	0.626
MIP-1A	AD	4	MR-Egger	0.754	0.535	1.062	0.248
MIP-1A	AD	4	Weighted median	1.052	0.902	1.227	0.517
MIP-1A	AD	4	Inverse variance weighted	1.074	0.901	1.28	0.424
MIP-1A	AD	4	MR-PRESSO	/	/	/	0.482
MIP-1B	AD	16	MR-Egger	1.045	0.971	1.125	0.258
MIP-1B	AD	16	Weighted median	1.036	0.974	1.103	0.264
MIP-1B	AD	16	Inverse variance weighted	1.022	0.974	1.073	0.372

MIP-1B	AD	16	MR-PRESSO	/	/	/	0.241
PDGF-BB	AD	11	MR-Egger	1.133	0.896	1.434	0.324
PDGF-BB	AD	11	Weighted median	1.009	0.908	1.121	0.87
PDGF-BB	AD	11	Inverse variance weighted	0.985	0.873	1.113	0.814
PDGF-BB	AD	11	MR-PRESSO	/	/	/	0.85
RANTES	AD	8	MR-Egger	0.936	0.754	1.161	0.569
RANTES	AD	8	Weighted median	1.003	0.902	1.115	0.962
RANTES	AD	8	Inverse variance weighted	0.992	0.91	1.081	0.851
RANTES	AD	8	MR-PRESSO	/	/	/	0.824
SCF	AD	10	MR-Egger	1.014	0.787	1.308	0.915
SCF	AD	10	Weighted median	0.943	0.824	1.08	0.398
SCF	AD	10	Inverse variance weighted	0.979	0.872	1.098	0.714
SCF	AD	10	MR-PRESSO	/	/	/	0.551
SCGFβ	AD	17	MR-Egger	1.005	0.906	1.116	0.92
SCGFβ	AD	17	Weighted median	1.007	0.937	1.083	0.846
SCGFβ	AD	17	Inverse variance weighted	1.024	0.97	1.081	0.386
SCGFβ	AD	17	MR-PRESSO	/	/	/	0.33
SDF-1A	AD	6	MR-Egger	0.836	0.638	1.097	0.266
SDF-1A	AD	6	Weighted median	0.921	0.774	1.096	0.353
SDF-1A	AD	6	Inverse variance weighted	0.887	0.774	1.017	0.086
SDF-1A	AD	6	MR-PRESSO	/	/	/	0.154
TNF-A	AD	5	MR-Egger	0.956	0.819	1.116	0.609
TNF-A	AD	5	Weighted median	0.978	0.877	1.091	0.694
TNF-A	AD	5	Inverse variance weighted	0.98	0.895	1.074	0.668
TNF-A	AD	5	MR-PRESSO	/	/	/	0.53
TNF-B	AD	4	MR-Egger	0.929	0.849	1.017	0.251

TNF-B	AD	4	Weighted median	1.001	0.931	1.075	0.988
TNF-B	AD	4	Inverse variance weighted	1.029	0.923	1.146	0.608
TNF-B	AD	4	MR-PRESSO	/	/	/	0.571
TRAIL	AD	11	MR-Egger	1.028	0.956	1.105	0.474
TRAIL	AD	11	Weighted median	1.06	0.986	1.14	0.116
TRAIL	AD	11	Inverse variance weighted	1.035	0.977	1.096	0.237
TRAIL	AD	11	MR-PRESSO	/	/	/	0.972
VEGF	AD	11	MR-Egger	0.978	0.909	1.053	0.572
VEGF	AD	11	Weighted median	0.97	0.919	1.024	0.265
VEGF	AD	11	Inverse variance weighted	1	0.954	1.049	0.998
VEGF	AD	11	MR-PRESSO	/	/	/	0.89
BNGF	PD	4	MR-Egger	0.466	0.172	1.262	0.272
BNGF	PD	4	Weighted median	1.081	0.866	1.349	0.492
BNGF	PD	4	Inverse variance weighted	1.090	0.870	1.367	0.453
BNGF	PD	4	MR-PRESSO	/	/	/	0.507
CTACK	PD	12	MR-Egger	1.131	0.980	1.305	0.122
CTACK	PD	12	Weighted median	1.076	0.966	1.198	0.185
CTACK	PD	12	Inverse variance weighted	1.077	0.994	1.167	0.071
CTACK	PD	12	MR-PRESSO	/	/	/	0.098
EOTAXIN	PD	15	MR-Egger	1.017	0.836	1.236	0.872
EOTAXIN	PD	15	Weighted median	1.003	0.890	1.130	0.957
EOTAXIN	PD	15	Inverse variance weighted	1.014	0.927	1.110	0.756
EOTAXIN	PD	15	MR-PRESSO	/	/	/	0.812
bFGF	PD	6	MR-Egger	1.029	0.653	1.621	0.909
bFGF	PD	6	Weighted median	0.933	0.713	1.222	0.615
bFGF	PD	6	Inverse variance weighted	0.891	0.716	1.109	0.302

bFGF	PD	6	MR-PRESSO	/	/	/	0.219
G-CSF	PD	8	MR-Egger	1.035	0.780	1.374	0.818
G-CSF	PD	8	Weighted median	0.975	0.792	1.202	0.815
G-CSF	PD	8	Inverse variance weighted	1.001	0.851	1.177	0.990
G-CSF	PD	8	MR-PRESSO	/	/	/	0.785
GROA	PD	9	MR-Egger	0.947	0.743	1.206	0.671
GROA	PD	9	Weighted median	0.989	0.913	1.072	0.796
GROA	PD	9	Inverse variance weighted	1.023	0.935	1.119	0.616
GROA	PD	9	MR-PRESSO	/	/	/	0.745
HGF	PD	7	MR-Egger	0.914	0.577	1.447	0.717
HGF	PD	7	Weighted median	0.893	0.744	1.072	0.226
HGF	PD	7	Inverse variance weighted	0.867	0.722	1.040	0.125
HGF	PD	7	MR-PRESSO	/	/	/	0.089
IFN-G	PD	9	MR-Egger	1.013	0.760	1.349	0.934
IFN-G	PD	9	Weighted median	1.050	0.856	1.288	0.640
IFN-G	PD	9	Inverse variance weighted	1.047	0.897	1.222	0.560
IFN-G	PD	9	MR-PRESSO	/	/	/	0.607
IL-1B	PD	4	MR-Egger	0.639	0.365	1.118	0.257
IL-1B	PD	4	Weighted median	0.961	0.734	1.258	0.771
IL-1B	PD	4	Inverse variance weighted	0.933	0.725	1.201	0.590
IL-1B	PD	4	MR-PRESSO	/	/	/	0.627
IL-1RA	PD	7	MR-Egger	0.661	0.346	1.264	0.266
IL-1RA	PD	7	Weighted median	0.924	0.758	1.127	0.436
IL-1RA	PD	7	Inverse variance weighted	0.933	0.767	1.134	0.485
IL-1RA	PD	7	MR-PRESSO	/	/	/	0.574
IL-2	PD	8	MR-Egger	1.019	0.738	1.408	0.912

IL-2	PD	8	Weighted median	1.182	0.997	1.401	0.054
IL-2	PD	8	Inverse variance weighted	1.169	1.000	1.368	**0.050**
IL-2	PD	8	MR-PRESSO	/	/	/	0.124
IL-2RA	PD	7	MR-Egger	1.028	0.916	1.155	0.657
IL-2RA	PD	7	Weighted median	1.003	0.919	1.094	0.949
IL-2RA	PD	7	Inverse variance weighted	1.007	0.932	1.089	0.855
IL-2RA	PD	7	MR-PRESSO	/	/	/	0.917
IL-4	PD	13	MR-Egger	0.943	0.771	1.152	0.576
IL-4	PD	13	Weighted median	0.988	0.837	1.167	0.889
IL-4	PD	13	Inverse variance weighted	1.057	0.923	1.210	0.425
IL-4	PD	13	MR-PRESSO	/	/	/	0.384
IL-5	PD	7	MR-Egger	1.091	0.805	1.478	0.599
IL-5	PD	7	Weighted median	1.055	0.888	1.254	0.543
IL-5	PD	7	Inverse variance weighted	1.031	0.905	1.176	0.644
IL-5	PD	7	MR-PRESSO	/	/	/	0.570
IL-6	PD	9	MR-Egger	0.897	0.552	1.457	0.673
IL-6	PD	9	Weighted median	0.821	0.644	1.046	0.111
IL-6	PD	9	Inverse variance weighted	0.850	0.686	1.053	0.137
IL-6	PD	9	MR-PRESSO	/	/	/	0.158
IL-7	PD	10	MR-Egger	1.047	0.873	1.256	0.632
IL-7	PD	10	Weighted median	0.931	0.835	1.040	0.205
IL-7	PD	10	Inverse variance weighted	0.926	0.850	1.010	0.081
IL-7	PD	10	MR-PRESSO	/	/	/	0.079
IL-8	PD	8	MR-Egger	0.948	0.815	1.102	0.510
IL-8	PD	8	Weighted median	1.008	0.891	1.140	0.898
IL-8	PD	8	Inverse variance weighted	0.999	0.906	1.101	0.982

IL-8	PD	8	MR-PRESSO	/	/	/	0.975
IL-9	PD	4	MR-Egger	0.758	0.173	3.320	0.749
IL-9	PD	4	Weighted median	1.143	0.915	1.427	0.240
IL-9	PD	4	Inverse variance weighted	1.078	0.850	1.367	0.534
IL-9	PD	4	MR-PRESSO	/	/	/	0.583
IL-10	PD	13	MR-Egger	0.902	0.680	1.197	0.490
IL-10	PD	13	Weighted median	0.940	0.818	1.081	0.389
IL-10	PD	13	Inverse variance weighted	0.962	0.848	1.091	0.544
IL-10	PD	13	MR-PRESSO	/	/	/	0.451
IL-12	PD	13	MR-Egger	0.902	0.680	1.197	0.490
IL-12	PD	13	Weighted median	0.940	0.821	1.078	0.377
IL-12	PD	13	Inverse variance weighted	0.962	0.848	1.091	0.544
IL-12	PD	13	MR-PRESSO	/	/	/	0.451
IL-13	PD	9	MR-Egger	0.899	0.778	1.039	0.194
IL-13	PD	9	Weighted median	0.962	0.867	1.067	0.466
IL-13	PD	9	Inverse variance weighted	0.985	0.904	1.073	0.730
IL-13	PD	9	MR-PRESSO	/	/	/	0.743
IL-16	PD	9	MR-Egger	1.008	0.887	1.144	0.911
IL-16	PD	9	Weighted median	1.060	0.981	1.146	0.139
IL-16	PD	9	Inverse variance weighted	1.036	0.959	1.119	0.365
IL-16	PD	9	MR-PRESSO	/	/	/	0.391
IL-17	PD	7	MR-Egger	0.889	0.632	1.252	0.532
IL-17	PD	7	Weighted median	1.011	0.816	1.254	0.918
IL-17	PD	7	Inverse variance weighted	1.016	0.862	1.198	0.847
IL-17	PD	7	MR-PRESSO	/	/	/	0.920
IL-18	PD	11	MR-Egger	0.952	0.817	1.109	0.542

IL-18	PD	11	Weighted median	1.069	0.966	1.183	0.198
IL-18	PD	11	Inverse variance weighted	1.058	0.984	1.137	0.129
IL-18	PD	11	MR-PRESSO	/	/	/	0.241
IP-10	PD	8	MR-Egger	0.865	0.581	1.287	0.500
IP-10	PD	8	Weighted median	0.911	0.769	1.080	0.282
IP-10	PD	8	Inverse variance weighted	1.003	0.819	1.229	0.975
IP-10	PD	8	MR-PRESSO	/	/	/	0.976
M-CSF	PD	9	MR-Egger	0.939	0.747	1.181	0.607
M-CSF	PD	9	Weighted median	1.026	0.909	1.158	0.677
M-CSF	PD	9	Inverse variance weighted	0.992	0.887	1.109	0.881
M-CSF	PD	9	MR-PRESSO	/	/	/	0.589
MCP-1-MCAF	PD	14	MR-Egger	1.031	0.850	1.249	0.764
MCP-1-MCAF	PD	14	Weighted median	1.016	0.903	1.144	0.789
MCP-1-MCAF	PD	14	Inverse variance weighted	1.011	0.923	1.106	0.817
MCP-1-MCAF	PD	14	MR-PRESSO	/	/	/	0.796
MCP-3	PD	5	MR-Egger	0.799	0.553	1.154	0.317
MCP-3	PD	5	Weighted median	0.976	0.864	1.102	0.693
MCP-3	PD	5	Inverse variance weighted	0.964	0.876	1.060	0.447
MCP-3	PD	5	MR-PRESSO	/	/	/	0.418
MIF	PD	7	MR-Egger	1.045	0.725	1.507	0.822
MIF	PD	7	Weighted median	1.067	0.860	1.323	0.555
MIF	PD	7	Inverse variance weighted	1.020	0.864	1.205	0.815
MIF	PD	7	MR-PRESSO	/	/	/	0.829
MIG	PD	9	MR-Egger	1.020	0.830	1.253	0.855
MIG	PD	9	Weighted median	1.008	0.874	1.163	0.915
MIG	PD	9	Inverse variance weighted	0.933	0.842	1.032	0.179

MIG	PD	9	MR-PRESSO	/	/	/	0.209
MIP-1A	PD	4	MR-Egger	0.923	0.561	1.517	0.782
MIP-1A	PD	4	Weighted median	0.890	0.726	1.092	0.265
MIP-1A	PD	4	Inverse variance weighted	0.884	0.747	1.048	0.155
MIP-1A	PD	4	MR-PRESSO	/	/	/	0.123
MIP-1B	PD	14	MR-Egger	1.129	1.024	1.244	**0.031**
MIP-1B	PD	14	Weighted median	1.085	1.003	1.173	**0.041**
MIP-1B	PD	14	Inverse variance weighted	1.064	0.998	1.133	0.056
MIP-1B	PD	14	MR-PRESSO	/	/	/	0.608
PDGF-BB	PD	11	MR-Egger	1.175	0.864	1.599	0.331
PDGF-BB	PD	11	Weighted median	1.039	0.891	1.212	0.623
PDGF-BB	PD	11	Inverse variance weighted	1.029	0.880	1.205	0.717
PDGF-BB	PD	11	MR-PRESSO	/	/	/	0.575
RANTES	PD	8	MR-Egger	0.941	0.527	1.682	0.845
RANTES	PD	8	Weighted median	0.908	0.755	1.093	0.308
RANTES	PD	8	Inverse variance weighted	0.994	0.819	1.207	0.952
RANTES	PD	8	MR-PRESSO	/	/	/	0.981
SCF	PD	9	MR-Egger	0.794	0.507	1.244	0.348
SCF	PD	9	Weighted median	1.066	0.859	1.323	0.564
SCF	PD	9	Inverse variance weighted	1.006	0.827	1.225	0.949
SCF	PD	9	MR-PRESSO	/	/	/	0.644
SCGFβ	PD	17	MR-Egger	0.946	0.817	1.096	0.474
SCGFβ	PD	17	Weighted median	1.021	0.923	1.128	0.689
SCGFβ	PD	17	Inverse variance weighted	1.011	0.939	1.089	0.764
SCGFβ	PD	17	MR-PRESSO	/	/	/	0.593
SDF-1A	PD	4	MR-Egger	0.767	0.442	1.329	0.413

SDF-1A	PD	4	Weighted median	0.774	0.587	1.020	0.069
SDF-1A	PD	4	Inverse variance weighted	0.853	0.644	1.131	0.269
SDF-1A	PD	4	MR-PRESSO	/	/	/	0.893
TNF-A	PD	4	MR-Egger	0.882	0.700	1.112	0.401
TNF-A	PD	4	Weighted median	0.999	0.840	1.188	0.992
TNF-A	PD	4	Inverse variance weighted	1.002	0.871	1.152	0.982
TNF-A	PD	4	MR-PRESSO	/	/	/	0.982
TNF-B	PD	4	MR-Egger	1.051	0.948	1.165	0.446
TNF-B	PD	4	Weighted median	0.998	0.923	1.080	0.966
TNF-B	PD	4	Inverse variance weighted	1.000	0.930	1.074	0.997
TNF-B	PD	4	MR-PRESSO	/	/	/	0.836
TRAIL	PD	9	MR-Egger	1.013	0.760	1.349	0.934
TRAIL	PD	9	Weighted median	1.050	0.858	1.284	0.635
TRAIL	PD	9	Inverse variance weighted	1.047	0.897	1.222	0.560
TRAIL	PD	9	MR-PRESSO	/	/	/	0.607
VEGF	PD	11	MR-Egger	0.943	0.844	1.055	0.332
VEGF	PD	11	Weighted median	0.953	0.877	1.035	0.252
VEGF	PD	11	Inverse variance weighted	0.955	0.889	1.026	0.211
VEGF	PD	11	MR-PRESSO	/	/	/	0.241
BNGF	ALS	4	MR-Egger	1.456	0.718	2.952	0.407
BNGF	ALS	4	Weighted median	1.152	1.000	1.328	**0.050**
BNGF	ALS	4	Inverse variance weighted	1.142	1.017	1.283	**0.025**
BNGF	ALS	4	MR-PRESSO	/	/	/	0.892
CTACK	ALS	11	MR-Egger	1.047	0.948	1.157	0.386
CTACK	ALS	11	Weighted median	1.030	0.959	1.108	0.418
CTACK	ALS	11	Inverse variance weighted	1.032	0.977	1.090	0.253

CTACK	ALS	11	MR-PRESSO	/	/	/	0.543
EOTAXIN	ALS	16	MR-Egger	1.045	0.971	1.125	0.258
EOTAXIN	ALS	16	Weighted median	1.036	0.974	1.102	0.259
EOTAXIN	ALS	16	Inverse variance weighted	1.022	0.974	1.073	0.372
EOTAXIN	ALS	16	MR-PRESSO	/	/	/	0.499
bFGF	ALS	5	MR-Egger	1.104	0.846	1.442	0.519
bFGF	ALS	5	Weighted median	1.067	0.902	1.262	0.451
bFGF	ALS	5	Inverse variance weighted	1.006	0.872	1.161	0.936
bFGF	ALS	5	MR-PRESSO	/	/	/	0.471
G-CSF	ALS	8	MR-Egger	1.034	0.873	1.224	0.712
G-CSF	ALS	8	Weighted median	1.046	0.917	1.192	0.503
G-CSF	ALS	8	Inverse variance weighted	1.076	0.971	1.193	0.164
G-CSF	ALS	8	MR-PRESSO	/	/	/	0.570
GROA	ALS	9	MR-Egger	0.977	0.883	1.081	0.664
GROA	ALS	9	Weighted median	0.987	0.935	1.042	0.641
GROA	ALS	9	Inverse variance weighted	0.979	0.937	1.022	0.335
GROA	ALS	9	MR-PRESSO	/	/	/	0.828
HGF	ALS	7	MR-Egger	1.160	0.913	1.474	0.278
HGF	ALS	7	Weighted median	1.057	0.927	1.205	0.406
HGF	ALS	7	Inverse variance weighted	1.037	0.933	1.153	0.497
HGF	ALS	7	MR-PRESSO	/	/	/	0.759
IFN-G	ALS	9	MR-Egger	0.899	0.735	1.099	0.333
IFN-G	ALS	9	Weighted median	1.022	0.885	1.180	0.771
IFN-G	ALS	9	Inverse variance weighted	1.001	0.898	1.115	0.990
IFN-G	ALS	9	MR-PRESSO	/	/	/	0.783
IL-1B	ALS	3	MR-Egger	1.402	0.922	2.132	0.359

IL-1B	ALS	3	Weighted median	1.037	0.868	1.239	0.687
IL-1B	ALS	3	Inverse variance weighted	1.048	0.839	1.310	0.678
IL-1B	ALS	3	MR-PRESSO	/	/	/	NA
IL-1RA	ALS	6	MR-Egger	1.315	0.850	2.036	0.286
IL-1RA	ALS	6	Weighted median	1.117	0.972	1.284	0.118
IL-1RA	ALS	6	Inverse variance weighted	1.131	0.990	1.292	0.069
IL-1RA	ALS	6	MR-PRESSO	/	/	/	0.208
IL-2	ALS	8	MR-Egger	0.968	0.786	1.193	0.772
IL-2	ALS	8	Weighted median	0.947	0.856	1.048	0.292
IL-2	ALS	8	Inverse variance weighted	0.984	0.889	1.089	0.754
IL-2	ALS	8	MR-PRESSO	/	/	/	0.079
IL-2RA	ALS	7	MR-Egger	0.994	0.904	1.092	0.898
IL-2RA	ALS	7	Weighted median	0.976	0.911	1.046	0.495
IL-2RA	ALS	7	Inverse variance weighted	0.974	0.917	1.034	0.384
IL-2RA	ALS	7	MR-PRESSO	/	/	/	0.583
IL-4	ALS	12	MR-Egger	1.016	0.893	1.157	0.810
IL-4	ALS	12	Weighted median	1.024	0.917	1.142	0.675
IL-4	ALS	12	Inverse variance weighted	1.022	0.942	1.109	0.604
IL-4	ALS	12	MR-PRESSO	/	/	/	0.848
IL-5	ALS	8	MR-Egger	1.024	0.793	1.322	0.862
IL-5	ALS	8	Weighted median	1.019	0.911	1.139	0.747
IL-5	ALS	8	Inverse variance weighted	0.986	0.897	1.084	0.773
IL-5	ALS	8	MR-PRESSO	/	/	/	0.247
IL-6	ALS	8	MR-Egger	1.056	0.797	1.400	0.717
IL-6	ALS	8	Weighted median	1.043	0.900	1.209	0.577
IL-6	ALS	8	Inverse variance weighted	1.073	0.942	1.222	0.292

IL-6	ALS	8	MR-PRESSO	/	/	/	0.150
IL-7	ALS	11	MR-Egger	1.053	0.899	1.233	0.540
IL-7	ALS	11	Weighted median	1.000	0.930	1.076	0.995
IL-7	ALS	11	Inverse variance weighted	0.980	0.912	1.052	0.572
IL-7	ALS	11	MR-PRESSO	/	/	/	0.060
IL-8	ALS	8	MR-Egger	1.051	0.910	1.212	0.525
IL-8	ALS	8	Weighted median	1.079	0.980	1.188	0.121
IL-8	ALS	8	Inverse variance weighted	1.044	0.959	1.136	0.324
IL-8	ALS	8	MR-PRESSO	/	/	/	0.160
IL-9	ALS	4	MR-Egger	0.500	0.277	0.903	0.148
IL-9	ALS	4	Weighted median	1.025	0.878	1.197	0.751
IL-9	ALS	4	Inverse variance weighted	1.072	0.893	1.285	0.457
IL-9	ALS	4	MR-PRESSO	/	/	/	0.125
IL-10	ALS	13	MR-Egger	1.012	0.874	1.172	0.876
IL-10	ALS	13	Weighted median	0.999	0.916	1.090	0.982
IL-10	ALS	13	Inverse variance weighted	1.004	0.936	1.076	0.913
IL-10	ALS	13	MR-PRESSO	/	/	/	0.601
IL-12	ALS	13	MR-Egger	1.012	0.874	1.172	0.876
IL-12	ALS	13	Weighted median	0.999	0.915	1.091	0.045
IL-12	ALS	13	Inverse variance weighted	1.004	0.9364396	1.076	0.913
IL-12	ALS	13	MR-PRESSO	/	/	/	0.594
IL-13	ALS	10	MR-Egger	1.060	0.976	1.151	0.205
IL-13	ALS	10	Weighted median	1.009	0.950	1.073	0.765
IL-13	ALS	10	Inverse variance weighted	1.019	0.972	1.070	0.430
IL-13	ALS	10	MR-PRESSO	/	/	/	0.732
IL-16	ALS	9	MR-Egger	1.035	0.959	1.116	0.408

IL-16	ALS	9	Weighted median	1.023	0.964	1.086	0.455
IL-16	ALS	9	Inverse variance weighted	1.013	0.965	1.063	0.603
IL-16	ALS	9	MR-PRESSO	/	/	/	0.671
IL-17	ALS	7	MR-Egger	0.921	0.738	1.149	0.497
IL-17	ALS	7	Weighted median	0.898	0.785	1.027	0.117
IL-17	ALS	7	Inverse variance weighted	0.942	0.849	1.046	0.263
IL-17	ALS	7	MR-PRESSO	/	/	/	0.559
IL-18	ALS	12	MR-Egger	0.942	0.850	1.043	0.276
IL-18	ALS	12	Weighted median	0.968	0.904	1.038	0.365
IL-18	ALS	12	Inverse variance weighted	0.989	0.940	1.040	0.660
IL-18	ALS	12	MR-PRESSO	/	/	/	0.417
IP-10	ALS	8	MR-Egger	0.954	0.763	1.194	0.696
IP-10	ALS	8	Weighted median	1.065	0.946	1.198	0.299
IP-10	ALS	8	Inverse variance weighted	1.030	0.946	1.122	0.498
IP-10	ALS	8	MR-PRESSO	/	/	/	0.504
M-CSF	ALS	9	MR-Egger	0.987	0.878	1.110	0.833
M-CSF	ALS	9	Weighted median	1.019	0.945	1.098	0.625
M-CSF	ALS	9	Inverse variance weighted	1.012	0.955	1.073	0.682
M-CSF	ALS	9	MR-PRESSO	/	/	/	0.504
MCP-1-MCAF	ALS	13	MR-Egger	0.948	0.789	1.138	0.576
MCP-1-MCAF	ALS	13	Weighted median	0.936	0.847	1.034	0.191
MCP-1-MCAF	ALS	13	Inverse variance weighted	0.937	0.870	1.010	0.088
MCP-1-MCAF	ALS	13	MR-PRESSO	/	/	/	0.427
MCP-3	ALS	5	MR-Egger	0.977	0.781	1.221	0.849
MCP-3	ALS	5	Weighted median	1.020	0.938	1.109	0.646
MCP-3	ALS	5	Inverse variance weighted	0.992	0.929	1.059	0.812

MCP-3	ALS	5	MR-PRESSO	/	/	/	0.583
MIF	ALS	5	MR-Egger	0.809	0.478	1.372	0.489
MIF	ALS	5	Weighted median	0.933	0.799	1.090	0.383
MIF	ALS	5	Inverse variance weighted	0.959	0.834	1.102	0.552
MIF	ALS	5	MR-PRESSO	/	/	/	0.481
MIG	ALS	10	MR-Egger	0.841	0.743	0.953	**0.026**
MIG	ALS	10	Weighted median	0.918	0.837	1.006	0.067
MIG	ALS	10	Inverse variance weighted	0.965	0.885	1.053	0.428
MIG	ALS	10	MR-PRESSO	/	/	/	0.095
MIP-1A	ALS	6	MR-Egger	1.064	0.643	1.761	0.820
MIP-1A	ALS	6	Weighted median	0.988	0.856	1.140	0.867
MIP-1A	ALS	6	Inverse variance weighted	0.990	0.849	1.154	0.895
MIP-1A	ALS	6	MR-PRESSO	/	/	/	0.627
MIP-1B	ALS	15	MR-Egger	0.973	0.907	1.044	0.456
MIP-1B	ALS	15	Weighted median	1.013	0.952	1.077	0.684
MIP-1B	ALS	15	Inverse variance weighted	1.006	0.959	1.055	0.802
MIP-1B	ALS	15	MR-PRESSO	/	/	/	0.427
PDGF-BB	ALS	11	MR-Egger	0.930	0.807	1.072	0.344
PDGF-BB	ALS	11	Weighted median	0.964	0.876	1.062	0.459
PDGF-BB	ALS	11	Inverse variance weighted	0.946	0.883	1.015	0.122
PDGF-BB	ALS	11	MR-PRESSO	/	/	/	0.198
RANTES	ALS	9	MR-Egger	1.048	0.830	1.324	0.703
RANTES	ALS	9	Weighted median	0.949	0.856	1.053	0.324
RANTES	ALS	9	Inverse variance weighted	0.967	0.890	1.050	0.426
RANTES	ALS	9	MR-PRESSO	/	/	/	0.340
SCF	ALS	10	MR-Egger	1.034	0.826	1.295	0.778

SCF	ALS	10	Weighted median	1.056	0.928	1.201	0.409
SCF	ALS	10	Inverse variance weighted	1.034	0.938	1.141	0.498
SCF	ALS	10	MR-PRESSO	/	/	/	0.725
SCGFβ	ALS	17	MR-Egger	0.996	0.894	1.109	0.936
SCGFβ	ALS	17	Weighted median	1.005	0.934	1.082	0.892
SCGFβ	ALS	17	Inverse variance weighted	1.006	0.954	1.062	0.813
SCGFβ	ALS	17	MR-PRESSO	/	/	/	0.812
SDF-1A	ALS	6	MR-Egger	0.906	0.677	1.212	0.543
SDF-1A	ALS	6	Weighted median	0.968	0.821	1.143	0.705
SDF-1A	ALS	6	Inverse variance weighted	1.000	0.880	1.137	0.995
SDF-1A	ALS	6	MR-PRESSO	/	/	/	0.845
TNF-A	ALS	3	MR-Egger	1.024	0.845	1.241	0.847
TNF-A	ALS	3	Weighted median	0.971	0.858	1.099	0.641
TNF-A	ALS	3	Inverse variance weighted	0.954	0.858	1.061	0.385
TNF-A	ALS	3	MR-PRESSO	/	/	/	NA
TNF-B	ALS	4	MR-Egger	1.003	0.925	1.086	0.956
TNF-B	ALS	4	Weighted median	1.015	0.954	1.080	0.639
TNF-B	ALS	4	Inverse variance weighted	1.016	0.962	1.073	0.558
TNF-B	ALS	4	MR-PRESSO	/	/	/	0.729
TRAIL	ALS	12	MR-Egger	0.984	0.922	1.050	0.641
TRAIL	ALS	12	Weighted median	0.975	0.916	1.037	0.417
TRAIL	ALS	12	Inverse variance weighted	0.977	0.929	1.029	0.384
TRAIL	ALS	12	MR-PRESSO	/	/	/	0.916
VEGF	ALS	12	MR-Egger	1.018	0.948	1.094	0.626
VEGF	ALS	12	Weighted median	0.997	0.946	1.050	0.906
VEGF	ALS	12	Inverse variance weighted	0.991	0.946	1.037	0.687

VEGF	ALS	12	MR-PRESSO	/	/	/	0.603

Supplementary Table 11. Detailed information for age-related neurodegenerative diseases on circulating cytokines.

Exposure	Outcome	N SNPs	Method	BETA	SE	*P* value
AD	BNGF	37	MR-Egger	-0.031	0.044	0.491
AD	BNGF	37	Weighted median	-0.074	0.044	0.097
AD	BNGF	37	Inverse variance weighted	-0.023	0.031	0.451
AD	BNGF	37	MR-PRESSO	/	/	0.591
AD	CTACK	38	MR-Egger	0.017	0.042	0.688
AD	CTACK	38	Weighted median	0.029	0.045	0.519
AD	CTACK	38	Inverse variance weighted	0.017	0.030	0.581
AD	CTACK	38	MR-PRESSO	/	/	0.987
AD	EOTAXIN	38	MR-Egger	0.056	0.032	0.089
AD	EOTAXIN	38	Weighted median	0.046	0.031	0.140
AD	EOTAXIN	38	Inverse variance weighted	0.031	0.022	0.162
AD	EOTAXIN	38	MR-PRESSO	/	/	0.073
AD	bFGF	38	MR-Egger	0.070	0.030	**0.024**
AD	bFGF	38	Weighted median	0.062	0.031	**0.048**
AD	bFGF	38	Inverse variance weighted	0.050	0.021	**0.017**
AD	bFGF	38	MR-PRESSO	/	/	0.814
AD	G-CSF	38	MR-Egger	0.046	0.029	0.120
AD	G-CSF	38	Weighted median	0.037	0.032	0.242
AD	G-CSF	38	Inverse variance weighted	0.039	0.020	0.055
AD	G-CSF	38	MR-PRESSO	/	/	0.547
AD	GROA	38	MR-Egger	-0.025	0.053	0.639
AD	GROA	38	Weighted median	0.003	0.048	0.953
AD	GROA	38	Inverse variance weighted	-0.010	0.037	0.781
AD	GROA	38	MR-PRESSO	/	/	0.065

AD	HGF	38	MR-Egger	0.026	0.028	0.357
AD	HGF	38	Weighted median	0.005	0.031	0.867
AD	HGF	38	Inverse variance weighted	0.012	0.020	0.554
AD	HGF	38	MR-PRESSO	/	/	0.859
AD	IFN-G	38	MR-Egger	0.017	0.030	0.574
AD	IFN-G	38	Weighted median	0.024	0.033	0.466
AD	IFN-G	38	Inverse variance weighted	0.027	0.021	0.188
AD	IFN-G	38	MR-PRESSO	/	/	0.772
AD	IL-1B	37	MR-Egger	0.079	0.045	0.088
AD	IL-1B	37	Weighted median	0.088	0.047	0.063
AD	IL-1B	37	Inverse variance weighted	0.043	0.032	0.180
AD	IL-1B	37	MR-PRESSO	/	/	0.727
AD	IL-1RA	36	MR-Egger	0.038	0.043	0.390
AD	IL-1RA	36	Weighted median	0.061	0.047	0.195
AD	IL-1RA	36	Inverse variance weighted	0.029	0.030	0.332
AD	IL-1RA	36	MR-PRESSO	/	/	0.834
AD	IL-2	36	MR-Egger	0.062	0.044	0.172
AD	IL-2	36	Weighted median	0.102	0.047	**0.029**
AD	IL-2	36	Inverse variance weighted	0.045	0.031	0.145
AD	IL-2	36	MR-PRESSO	/	/	0.610
AD	IL-2RA	36	MR-Egger	-0.062	0.046	0.186
AD	IL-2RA	36	Weighted median	-0.028	0.046	0.538
AD	IL-2RA	36	Inverse variance weighted	-0.030	0.033	0.362
AD	IL-2RA	36	MR-PRESSO	/	/	0.295
AD	IL-4	38	MR-Egger	0.027	0.032	0.406
AD	IL-4	38	Weighted median	0.034	0.029	0.249

AD	IL-4	38	Inverse variance weighted	0.021	0.022	0.353
AD	IL-4	38	MR-PRESSO	/	/	0.233
AD	IL-5	37	MR-Egger	0.061	0.045	0.182
AD	IL-5	37	Weighted median	0.044	0.044	0.323
AD	IL-5	37	Inverse variance weighted	0.007	0.032	0.827
AD	IL-5	37	MR-PRESSO	/	/	0.761
AD	IL-6	38	MR-Egger	0.039	0.029	0.178
AD	IL-6	38	Weighted median	0.031	0.030	0.314
AD	IL-6	38	Inverse variance weighted	0.013	0.020	0.512
AD	IL-6	38	MR-PRESSO	/	/	0.697
AD	IL-7	36	MR-Egger	0.071	0.045	0.124
AD	IL-7	36	Weighted median	0.081	0.048	0.088
AD	IL-7	36	Inverse variance weighted	0.039	0.031	0.213
AD	IL-7	36	MR-PRESSO	/	/	0.729
AD	IL-8	37	MR-Egger	0.087	0.044	0.056
AD	IL-8	37	Weighted median	0.075	0.047	0.109
AD	IL-8	37	Inverse variance weighted	0.021	0.032	0.500
AD	IL-8	37	MR-PRESSO	/	/	0.230
AD	IL-9	36	MR-Egger	0.021	0.043	0.631
AD	IL-9	36	Weighted median	0.023	0.046	0.614
AD	IL-9	36	Inverse variance weighted	0.018	0.030	0.542
AD	IL-9	36	MR-PRESSO	/	/	0.749
AD	IL-10	37	MR-Egger	0.061	0.032	0.069
AD	IL-10	37	Weighted median	0.046	0.032	0.156
AD	IL-10	37	Inverse variance weighted	0.041	0.023	0.069
AD	IL-10	37	MR-PRESSO	/	/	0.332

AD	IL-12	38	MR-Egger	0.045	0.028	0.123
AD	IL-12	38	Weighted median	0.041	0.030	0.178
AD	IL-12	38	Inverse variance weighted	0.040	0.020	**0.046**
AD	IL-12	38	MR-PRESSO	/	/	0.570
AD	IL-13	36	MR-Egger	0.094	0.044	**0.040**
AD	IL-13	36	Weighted median	0.099	0.045	**0.028**
AD	IL-13	36	Inverse variance weighted	0.043	0.031	0.163
AD	IL-13	36	MR-PRESSO	/	/	0.958
AD	IL-16	38	MR-Egger	-0.049	0.054	0.367
AD	IL-16	38	Weighted median	-0.031	0.048	0.515
AD	IL-16	38	Inverse variance weighted	-0.027	0.037	0.470
AD	IL-16	38	MR-PRESSO	/	/	0.086
AD	IL-17	38	MR-Egger	0.043	0.029	0.153
AD	IL-17	38	Weighted median	0.038	0.031	0.213
AD	IL-17	38	Inverse variance weighted	0.027	0.021	0.191
AD	IL-17	38	MR-PRESSO	/	/	0.748
AD	IL-18	38	MR-Egger	-0.042	0.043	0.337
AD	IL-18	38	Weighted median	-0.040	0.045	0.376
AD	IL-18	38	Inverse variance weighted	-0.042	0.030	0.164
AD	IL-18	38	MR-PRESSO	/	/	0.706
AD	IP-10	37	MR-Egger	0.002	0.048	0.973
AD	IP-10	37	Weighted median	0.020	0.043	0.640
AD	IP-10	37	Inverse variance weighted	0.012	0.033	0.710
AD	IP-10	37	MR-PRESSO	/	/	0.291
AD	M-CSF	37	MR-Egger	-0.011	0.056	0.841
AD	M-CSF	37	Weighted median	-0.009	0.054	0.862

AD	M-CSF	37	Inverse variance weighted	-0.067	0.040	0.098
AD	M-CSF	37	MR-PRESSO	/	/	0.304
AD	MCP-1-MCAF	38	MR-Egger	0.017	0.034	0.615
AD	MCP-1-MCAF	38	Weighted median	0.005	0.032	0.871
AD	MCP-1-MCAF	38	Inverse variance weighted	-0.014	0.024	0.548
AD	MCP-1-MCAF	38	MR-PRESSO	/	/	0.093
AD	MCP-3	32	MR-Egger	0.039	0.096	0.687
AD	MCP-3	32	Weighted median	-0.048	0.084	0.567
AD	MCP-3	32	Inverse variance weighted	-0.062	0.067	0.354
AD	MCP-3	32	MR-PRESSO	/	/	0.246
AD	MIF	35	MR-Egger	-0.022	0.048	0.653
AD	MIF	35	Weighted median	-0.051	0.046	0.274
AD	MIF	35	Inverse variance weighted	-0.036	0.033	0.282
AD	MIF	35	MR-PRESSO	/	/	0.249
AD	MIG	37	MR-Egger	0.041	0.042	0.345
AD	MIG	37	Weighted median	0.036	0.045	0.424
AD	MIG	37	Inverse variance weighted	0.029	0.030	0.324
AD	MIG	37	MR-PRESSO	/	/	0.775
AD	MIP-1A	38	MR-Egger	0.066	0.044	0.140
AD	MIP-1A	38	Weighted median	0.101	0.046	**0.028**
AD	MIP-1A	38	Inverse variance weighted	0.052	0.031	0.089
AD	MIP-1A	38	MR-PRESSO	/	/	0.958
AD	MIP-1B	38	MR-Egger	0.020	0.028	0.486
AD	MIP-1B	38	Weighted median	0.019	0.030	0.533
AD	MIP-1B	38	Inverse variance weighted	0.004	0.020	0.824
AD	MIP-1B	38	MR-PRESSO	/	/	0.672

AD	PDGF-BB	38	MR-Egger	0.003	0.028	0.918
AD	PDGF-BB	38	Weighted median	0.005	0.030	0.877
AD	PDGF-BB	38	Inverse variance weighted	0.014	0.020	0.477
AD	PDGF-BB	38	MR-PRESSO	/	/	0.596
AD	RANTES	37	MR-Egger	-0.006	0.044	0.889
AD	RANTES	37	Weighted median	-0.032	0.046	0.491
AD	RANTES	37	Inverse variance weighted	0.002	0.031	0.942
AD	RANTES	37	MR-PRESSO	/	/	0.593
AD	SCF	38	MR-Egger	-0.008	0.030	0.783
AD	SCF	38	Weighted median	-0.016	0.029	0.587
AD	SCF	38	Inverse variance weighted	0.003	0.021	0.886
AD	SCF	38	MR-PRESSO	/	/	0.359
AD	SCGFβ	37	MR-Egger	-0.109	0.044	**0.018**
AD	SCGFβ	37	Weighted median	-0.067	0.046	0.143
AD	SCGFβ	37	Inverse variance weighted	-0.069	0.031	**0.027**
AD	SCGFβ	37	MR-PRESSO	/	/	0.195
AD	SDF-1A	38	MR-Egger	0.039	0.030	0.206
AD	SDF-1A	38	Weighted median	-0.004	0.032	0.894
AD	SDF-1A	38	Inverse variance weighted	0.018	0.021	0.404
AD	SDF-1A	38	MR-PRESSO	/	/	0.475
AD	TNF-A	37	MR-Egger	0.028	0.044	0.531
AD	TNF-A	37	Weighted median	0.024	0.047	0.611
AD	TNF-A	37	Inverse variance weighted	0.026	0.031	0.410
AD	TNF-A	37	MR-PRESSO	/	/	0.982
AD	TNF-B	30	MR-Egger	0.022	0.089	0.805
AD	TNF-B	30	Weighted median	-0.021	0.074	0.773

AD	TNF-B	30	Inverse variance weighted	0.036	0.058	0.534
AD	TNF-B	30	MR-PRESSO	/	/	0.420
AD	TRAIL	38	MR-Egger	0.022	0.028	0.446
AD	TRAIL	38	Weighted median	0.016	0.030	0.595
AD	TRAIL	38	Inverse variance weighted	0.018	0.020	0.380
AD	TRAIL	38	MR-PRESSO	/	/	0.974
AD	VEGF	38	MR-Egger	0.040	0.031	0.200
AD	VEGF	38	Weighted median	0.019	0.033	0.575
AD	VEGF	38	Inverse variance weighted	0.022	0.022	0.303
AD	VEGF	38	MR-PRESSO	/	/	0.641
PD	BNGF	39	MR-Egger	0.011	0.060	0.860
PD	BNGF	39	Weighted median	-0.022	0.046	0.637
PD	BNGF	39	Inverse variance weighted	0.009	0.032	0.770
PD	BNGF	39	MR-PRESSO	/	/	0.399
PD	CTACK	41	MR-Egger	0.018	0.058	0.762
PD	CTACK	41	Weighted median	0.013	0.048	0.794
PD	CTACK	41	Inverse variance weighted	0.002	0.031	0.938
PD	CTACK	41	MR-PRESSO	/	/	0.708
PD	EOTAXIN	41	MR-Egger	0.001	0.038	0.986
PD	EOTAXIN	41	Weighted median	-0.027	0.031	0.390
PD	EOTAXIN	41	Inverse variance weighted	-0.037	0.021	0.075
PD	EOTAXIN	41	MR-PRESSO	/	/	0.696
PD	bFGF	40	MR-Egger	0.014	0.041	0.740
PD	bFGF	40	Weighted median	0.021	0.032	0.519
PD	bFGF	40	Inverse variance weighted	0.000	0.022	0.997
PD	bFGF	40	MR-PRESSO	/	/	0.242

PD	G-CSF	40	MR-Egger	0.029	0.039	0.470
PD	G-CSF	40	Weighted median	0.034	0.031	0.273
PD	G-CSF	40	Inverse variance weighted	0.025	0.021	0.226
PD	G-CSF	40	MR-PRESSO	/	/	0.566
PD	GROA	40	MR-Egger	0.039	0.059	0.509
PD	GROA	40	Weighted median	0.004	0.048	0.929
PD	GROA	40	Inverse variance weighted	0.017	0.032	0.598
PD	GROA	40	MR-PRESSO	/	/	0.917
PD	HGF	40	MR-Egger	-0.021	0.044	0.638
PD	HGF	40	Weighted median	-0.027	0.032	0.406
PD	HGF	40	Inverse variance weighted	-0.013	0.023	0.565
PD	HGF	40	MR-PRESSO	/	/	0.070
PD	IFN-G	41	MR-Egger	-0.054	0.039	0.180
PD	IFN-G	41	Weighted median	-0.050	0.033	0.136
PD	IFN-G	41	Inverse variance weighted	-0.013	0.021	0.551
PD	IFN-G	41	MR-PRESSO	/	/	0.387
PD	IL-1B	40	MR-Egger	-0.109	0.061	0.080
PD	IL-1B	40	Weighted median	-0.062	0.048	0.193
PD	IL-1B	40	Inverse variance weighted	-0.018	0.033	0.588
PD	IL-1B	40	MR-PRESSO	/	/	0.767
PD	IL-1RA	39	MR-Egger	-0.018	0.058	0.762
PD	IL-1RA	39	Weighted median	-0.024	0.045	0.587
PD	IL-1RA	39	Inverse variance weighted	-0.008	0.031	0.799
PD	IL-1RA	39	MR-PRESSO	/	/	0.605
PD	IL-2	41	MR-Egger	-0.085	0.059	0.155
PD	IL-2	41	Weighted median	-0.069	0.049	0.163

PD	IL-2	41	Inverse variance weighted	-0.050	0.031	0.109
PD	IL-2	41	MR-PRESSO	/	/	0.691
PD	IL-2RA	41	MR-Egger	0.061	0.057	0.297
PD	IL-2RA	41	Weighted median	0.007	0.046	0.881
PD	IL-2RA	41	Inverse variance weighted	0.036	0.031	0.247
PD	IL-2RA	41	MR-PRESSO	/	/	0.755
PD	IL-4	40	MR-Egger	0.005	0.039	0.897
PD	IL-4	40	Weighted median	0.020	0.030	0.511
PD	IL-4	40	Inverse variance weighted	0.008	0.021	0.690
PD	IL-4	40	MR-PRESSO	/	/	0.709
PD	IL-5	40	MR-Egger	0.014	0.060	0.818
PD	IL-5	40	Weighted median	0.072	0.052	0.169
PD	IL-5	40	Inverse variance weighted	0.035	0.032	0.271
PD	IL-5	40	MR-PRESSO	/	/	0.386
PD	IL-6	40	MR-Egger	-0.005	0.039	0.890
PD	IL-6	40	Weighted median	0.006	0.030	0.839
PD	IL-6	40	Inverse variance weighted	0.010	0.021	0.631
PD	IL-6	40	MR-PRESSO	/	/	0.239
PD	IL-7	40	MR-Egger	-0.057	0.060	0.347
PD	IL-7	40	Weighted median	-0.042	0.052	0.418
PD	IL-7	40	Inverse variance weighted	0.011	0.032	0.728
PD	IL-7	40	MR-PRESSO	/	/	0.596
PD	IL-8	40	MR-Egger	-0.057	0.059	0.342
PD	IL-8	40	Weighted median	-0.097	0.048	0.044
PD	IL-8	40	Inverse variance weighted	-0.046	0.031	0.141
PD	IL-8	40	MR-PRESSO	/	/	0.820

PD	IL-9	40	MR-Egger	-0.043	0.058	0.469
PD	IL-9	40	Weighted median	-0.069	0.045	0.127
PD	IL-9	40	Inverse variance weighted	-0.010	0.031	0.753
PD	IL-9	40	MR-PRESSO	/	/	0.501
PD	IL-10	41	MR-Egger	-0.013	0.040	0.739
PD	IL-10	41	Weighted median	-0.016	0.030	0.600
PD	IL-10	41	Inverse variance weighted	0.015	0.022	0.481
PD	IL-10	41	MR-PRESSO	/	/	0.362
PD	IL-12	41	MR-Egger	0.010	0.038	0.789
PD	IL-12	41	Weighted median	-0.006	0.031	0.839
PD	IL-12	41	Inverse variance weighted	0.011	0.021	0.581
PD	IL-12	41	MR-PRESSO	/	/	0.341
PD	IL-13	39	MR-Egger	-0.006	0.059	0.923
PD	IL-13	39	Weighted median	0.002	0.051	0.967
PD	IL-13	39	Inverse variance weighted	0.003	0.031	0.919
PD	IL-13	39	MR-PRESSO	/	/	0.698
PD	IL-16	40	MR-Egger	0.080	0.059	0.184
PD	IL-16	40	Weighted median	0.044	0.048	0.351
PD	IL-16	40	Inverse variance weighted	-0.002	0.032	0.944
PD	IL-16	40	MR-PRESSO	/	/	0.919
PD	IL-17	40	MR-Egger	-0.033	0.042	0.440
PD	IL-17	40	Weighted median	-0.038	0.032	0.226
PD	IL-17	40	Inverse variance weighted	-0.008	0.022	0.726
PD	IL-17	40	MR-PRESSO	/	/	**0.049**
PD	IL-18	40	MR-Egger	0.004	0.062	0.950
PD	IL-18	40	Weighted median	-0.023	0.048	0.632

PD	IL-18	40	Inverse variance weighted	0.010	0.032	0.768
PD	IL-18	40	MR-PRESSO	/	/	0.108
PD	IP-10	40	MR-Egger	-0.018	0.058	0.758
PD	IP-10	40	Weighted median	0.010	0.048	0.830
PD	IP-10	40	Inverse variance weighted	-0.001	0.031	0.986
PD	IP-10	40	MR-PRESSO	/	/	0.513
PD	M-CSF	40	MR-Egger	0.054	0.070	0.447
PD	M-CSF	40	Weighted median	0.027	0.058	0.642
PD	M-CSF	40	Inverse variance weighted	0.015	0.038	0.693
PD	M-CSF	40	MR-PRESSO	/	/	0.312
PD	MCP-1-MCAF	41	MR-Egger	0.028	0.039	0.468
PD	MCP-1-MCAF	41	Weighted median	-0.012	0.031	0.701
PD	MCP-1-MCAF	41	Inverse variance weighted	-0.002	0.021	0.924
PD	MCP-1-MCAF	41	MR-PRESSO	/	/	0.593
PD	MCP-3	36	MR-Egger	-0.002	0.108	0.988
PD	MCP-3	36	Weighted median	0.022	0.087	0.801
PD	MCP-3	36	Inverse variance weighted	0.007	0.057	0.906
PD	MCP-3	36	MR-PRESSO	/	/	0.458
PD	MIF	40	MR-Egger	0.052	0.059	0.385
PD	MIF	40	Weighted median	0.035	0.047	0.455
PD	MIF	40	Inverse variance weighted	0.014	0.032	0.669
PD	MIF	40	MR-PRESSO	/	/	0.790
PD	MIG	40	MR-Egger	-0.052	0.058	0.370
PD	MIG	40	Weighted median	-0.097	0.047	**0.039**
PD	MIG	40	Inverse variance weighted	-0.067	0.031	**0.030**
PD	MIG	40	MR-PRESSO	/	/	0.490

PD	MIP-1A	40	MR-Egger	-0.025	0.063	0.690
PD	MIP-1A	40	Weighted median	-0.034	0.048	0.486
PD	MIP-1A	40	Inverse variance weighted	-0.001	0.033	0.966
PD	MIP-1A	40	MR-PRESSO	/	/	0.206
PD	MIP-1B	41	MR-Egger	-0.039	0.054	0.472
PD	MIP-1B	41	Weighted median	0.009	0.030	0.766
PD	MIP-1B	41	Inverse variance weighted	0.025	0.029	0.386
PD	MIP-1B	41	MR-PRESSO	/	/	0.213
PD	PDGF-BB	40	MR-Egger	-0.013	0.038	0.728
PD	PDGF-BB	40	Weighted median	-0.005	0.032	0.880
PD	PDGF-BB	40	Inverse variance weighted	-0.006	0.021	0.781
PD	PDGF-BB	40	MR-PRESSO	/	/	0.712
PD	RANTES	40	MR-Egger	-0.102	0.060	0.096
PD	RANTES	40	Weighted median	-0.050	0.048	0.297
PD	RANTES	40	Inverse variance weighted	-0.010	0.032	0.749
PD	RANTES	40	MR-PRESSO	/	/	0.599
PD	SCF	40	MR-Egger	-0.003	0.045	0.951
PD	SCF	40	Weighted median	-0.055	0.032	0.084
PD	SCF	40	Inverse variance weighted	-0.007	0.024	0.784
PD	SCF	40	MR-PRESSO	/	/	0.103
PD	SCGFβ	39	MR-Egger	0.031	0.064	0.635
PD	SCGFβ	39	Weighted median	0.024	0.048	0.612
PD	SCGFβ	39	Inverse variance weighted	0.028	0.034	0.406
PD	SCGFβ	39	MR-PRESSO	/	/	0.120
PD	SDF-1A	41	MR-Egger	0.048	0.043	0.273
PD	SDF-1A	41	Weighted median	0.034	0.035	0.331

PD	SDF-1A	41	Inverse variance weighted	0.010	0.023	0.671
PD	SDF-1A	41	MR-PRESSO	/	/	0.172
PD	TNF-A	40	MR-Egger	-0.002	0.062	0.971
PD	TNF-A	40	Weighted median	0.000	0.050	0.998
PD	TNF-A	40	Inverse variance weighted	0.018	0.033	0.593
PD	TNF-A	40	MR-PRESSO	/	/	0.200
PD	TNF-B	34	MR-Egger	-0.064	0.107	0.554
PD	TNF-B	34	Weighted median	-0.025	0.075	0.744
PD	TNF-B	34	Inverse variance weighted	0.045	0.051	0.377
PD	TNF-B	34	MR-PRESSO	/	/	0.704
PD	TRAIL	41	MR-Egger	0.037	0.038	0.348
PD	TRAIL	41	Weighted median	-0.009	0.029	0.746
PD	TRAIL	41	Inverse variance weighted	0.007	0.021	0.746
PD	TRAIL	41	MR-PRESSO	/	/	0.817
PD	VEGF	41	MR-Egger	-0.028	0.041	0.505
PD	VEGF	41	Weighted median	-0.004	0.032	0.902
PD	VEGF	41	Inverse variance weighted	0.017	0.022	0.435
PD	VEGF	41	MR-PRESSO	/	/	0.256
ALS	BNGF	21	MR-Egger	0.009	0.134	0.946
ALS	BNGF	21	Weighted median	-0.004	0.089	0.964
ALS	BNGF	21	Inverse variance weighted	-0.017	0.066	0.790
ALS	BNGF	21	MR-PRESSO	/	/	0.556
ALS	CTACK	21	MR-Egger	0.147	0.140	0.309
ALS	CTACK	21	Weighted median	0.133	0.093	0.153
ALS	CTACK	21	Inverse variance weighted	0.020	0.069	0.768
ALS	CTACK	21	MR-PRESSO	/	/	0.561

ALS	EOTAXIN	21	MR-Egger	-0.108	0.101	0.296
ALS	EOTAXIN	21	Weighted median	-0.055	0.065	0.391
ALS	EOTAXIN	21	Inverse variance weighted	-0.023	0.050	0.648
ALS	EOTAXIN	21	MR-PRESSO	/	/	0.417
ALS	bFGF	21	MR-Egger	-0.267	0.090	**0.008**
ALS	bFGF	21	Weighted median	-0.133	0.065	**0.042**
ALS	bFGF	21	Inverse variance weighted	-0.110	0.046	**0.016**
ALS	bFGF	21	MR-PRESSO	/	/	0.758
ALS	G-CSF	21	MR-Egger	-0.117	0.096	0.240
ALS	G-CSF	21	Weighted median	-0.019	0.061	0.754
ALS	G-CSF	21	Inverse variance weighted	-0.009	0.048	0.847
ALS	G-CSF	21	MR-PRESSO	/	/	0.120
ALS	GROA	21	MR-Egger	0.163	0.135	0.240
ALS	GROA	21	Weighted median	0.075	0.095	0.427
ALS	GROA	21	Inverse variance weighted	0.036	0.066	0.589
ALS	GROA	21	MR-PRESSO	/	/	0.307
ALS	HGF	21	MR-Egger	-0.149	0.086	0.099
ALS	HGF	21	Weighted median	-0.098	0.064	0.125
ALS	HGF	21	Inverse variance weighted	-0.042	0.043	0.320
ALS	HGF	21	MR-PRESSO	/	/	0.277
ALS	IFN-G	21	MR-Egger	-0.197	0.090	**0.040**
ALS	IFN-G	21	Weighted median	-0.070	0.062	0.263
ALS	IFN-G	21	Inverse variance weighted	-0.038	0.044	0.387
ALS	IFN-G	21	MR-PRESSO	/	/	0.891
ALS	IL-1B	21	MR-Egger	-0.101	0.140	0.478
ALS	IL-1B	21	Weighted median	-0.012	0.101	0.903

ALS	IL-1B	21	Inverse variance weighted	-0.061	0.068	0.367
ALS	IL-1B	21	MR-PRESSO	/	/	0.172
ALS	IL-1RA	21	MR-Egger	0.053	0.142	0.712
ALS	IL-1RA	21	Weighted median	0.012	0.095	0.901
ALS	IL-1RA	21	Inverse variance weighted	-0.067	0.069	0.334
ALS	IL-1RA	21	MR-PRESSO	/	/	0.129
ALS	IL-2	21	MR-Egger	0.031	0.147	0.835
ALS	IL-2	21	Weighted median	0.025	0.095	0.796
ALS	IL-2	21	Inverse variance weighted	-0.117	0.072	0.104
ALS	IL-2	21	MR-PRESSO	/	/	0.305
ALS	IL-2RA	21	MR-Egger	-0.072	0.131	0.592
ALS	IL-2RA	21	Weighted median	-0.104	0.089	0.243
ALS	IL-2RA	21	Inverse variance weighted	-0.107	0.064	0.097
ALS	IL-2RA	21	MR-PRESSO	/	/	0.913
ALS	IL-4	21	MR-Egger	-0.134	0.103	0.209
ALS	IL-4	21	Weighted median	-0.056	0.067	0.399
ALS	IL-4	21	Inverse variance weighted	-0.025	0.051	0.625
ALS	IL-4	21	MR-PRESSO	/	/	0.180
ALS	IL-5	21	MR-Egger	-0.125	0.150	0.413
ALS	IL-5	21	Weighted median	-0.102	0.102	0.318
ALS	IL-5	21	Inverse variance weighted	-0.071	0.072	0.321
ALS	IL-5	21	MR-PRESSO	/	/	0.104
ALS	IL-6	21	MR-Egger	-0.107	0.087	0.233
ALS	IL-6	21	Weighted median	-0.061	0.062	0.319
ALS	IL-6	21	Inverse variance weighted	-0.049	0.043	0.251
ALS	IL-6	21	MR-PRESSO	/	/	0.664

ALS	IL-7	21	MR-Egger	-0.040	0.137	0.774
ALS	IL-7	21	Weighted median	0.100	0.092	0.274
ALS	IL-7	21	Inverse variance weighted	0.071	0.067	0.289
ALS	IL-7	21	MR-PRESSO	/	/	0.690
ALS	IL-8	21	MR-Egger	0.050	0.134	0.711
ALS	IL-8	21	Weighted median	0.020	0.093	0.826
ALS	IL-8	21	Inverse variance weighted	-0.033	0.066	0.617
ALS	IL-8	21	MR-PRESSO	/	/	0.540
ALS	IL-9	21	MR-Egger	-0.112	0.136	0.419
ALS	IL-9	21	Weighted median	-0.066	0.093	0.475
ALS	IL-9	21	Inverse variance weighted	-0.038	0.065	0.555
ALS	IL-9	21	MR-PRESSO	/	/	0.164
ALS	IL-10	21	MR-Egger	-0.046	0.090	0.616
ALS	IL-10	21	Weighted median	-0.067	0.066	0.307
ALS	IL-10	21	Inverse variance weighted	-0.032	0.044	0.468
ALS	IL-10	21	MR-PRESSO	/	/	0.856
ALS	IL-12	21	MR-Egger	-0.148	0.086	0.103
ALS	IL-12	21	Weighted median	-0.021	0.062	0.735
ALS	IL-12	21	Inverse variance weighted	-0.036	0.043	0.396
ALS	IL-12	21	MR-PRESSO	/	/	0.951
ALS	IL-13	21	MR-Egger	-0.005	0.135	0.971
ALS	IL-13	21	Weighted median	0.038	0.089	0.667
ALS	IL-13	21	Inverse variance weighted	0.012	0.065	0.856
ALS	IL-13	21	MR-PRESSO	/	/	0.981
ALS	IL-16	21	MR-Egger	-0.126	0.135	0.363
ALS	IL-16	21	Weighted median	-0.078	0.097	0.422

ALS	IL-16	21	Inverse variance weighted	-0.055	0.066	0.405
ALS	IL-16	21	MR-PRESSO	/	/	0.777
ALS	IL-17	21	MR-Egger	-0.226	0.089	**0.020**
ALS	IL-17	21	Weighted median	-0.104	0.062	0.094
ALS	IL-17	21	Inverse variance weighted	-0.097	0.045	**0.030**
ALS	IL-17	21	MR-PRESSO	/	/	0.462
ALS	IL-18	21	MR-Egger	0.101	0.133	0.459
ALS	IL-18	21	Weighted median	0.039	0.091	0.670
ALS	IL-18	21	Inverse variance weighted	0.117	0.065	0.069
ALS	IL-18	21	MR-PRESSO	/	/	0.591
ALS	IP-10	21	MR-Egger	0.220	0.148	0.153
ALS	IP-10	21	Weighted median	0.151	0.099	0.125
ALS	IP-10	21	Inverse variance weighted	0.104	0.072	0.146
ALS	IP-10	21	MR-PRESSO	/	/	0.162
ALS	M-CSF	21	MR-Egger	-0.089	0.162	0.591
ALS	M-CSF	21	Weighted median	-0.057	0.108	0.596
ALS	M-CSF	21	Inverse variance weighted	0.006	0.079	0.937
ALS	M-CSF	21	MR-PRESSO	/	/	0.974
ALS	MCP-1-MCAF	21	MR-Egger	-0.037	0.086	0.672
ALS	MCP-1-MCAF	21	Weighted median	-0.045	0.060	0.455
ALS	MCP-1-MCAF	21	Inverse variance weighted	-0.039	0.043	0.359
ALS	MCP-1-MCAF	21	MR-PRESSO	/	/	0.946
ALS	MCP-3	20	MR-Egger	0.143	0.362	0.698
ALS	MCP-3	20	Weighted median	0.120	0.183	0.513
ALS	MCP-3	20	Inverse variance weighted	0.086	0.148	0.559
ALS	MCP-3	20	MR-PRESSO	/	/	0.179

ALS	MIF	21	MR-Egger	0.034	0.135	0.806
ALS	MIF	21	Weighted median	-0.107	0.093	0.251
ALS	MIF	21	Inverse variance weighted	-0.101	0.066	0.125
ALS	MIF	21	MR-PRESSO	/	/	0.224
ALS	MIG	21	MR-Egger	0.110	0.132	0.414
ALS	MIG	21	Weighted median	-0.032	0.092	0.729
ALS	MIG	21	Inverse variance weighted	0.025	0.064	0.702
ALS	MIG	21	MR-PRESSO	/	/	0.716
ALS	MIP-1A	21	MR-Egger	-0.061	0.134	0.653
ALS	MIP-1A	21	Weighted median	-0.039	0.091	0.665
ALS	MIP-1A	21	Inverse variance weighted	-0.088	0.066	0.178
ALS	MIP-1A	21	MR-PRESSO	/	/	0.523
ALS	MIP-1B	21	MR-Egger	-0.097	0.086	0.272
ALS	MIP-1B	21	Weighted median	-0.025	0.059	0.671
ALS	MIP-1B	21	Inverse variance weighted	-0.008	0.043	0.854
ALS	MIP-1B	21	MR-PRESSO	/	/	0.064
ALS	PDGF-BB	21	MR-Egger	-0.028	0.086	0.748
ALS	PDGF-BB	21	Weighted median	0.010	0.060	0.860
ALS	PDGF-BB	21	Inverse variance weighted	0.035	0.043	0.411
ALS	PDGF-BB	21	MR-PRESSO	/	/	0.199
ALS	RANTES	21	MR-Egger	-0.111	0.138	0.429
ALS	RANTES	21	Weighted median	-0.075	0.096	0.438
ALS	RANTES	21	Inverse variance weighted	0.021	0.067	0.751
ALS	RANTES	21	MR-PRESSO	/	/	0.891
ALS	SCF	21	MR-Egger	0.025	0.086	0.777
ALS	SCF	21	Weighted median	0.018	0.060	0.765

ALS	SCF	21	Inverse variance weighted	-0.037	0.043	0.385
ALS	SCF	21	MR-PRESSO	/	/	0.375
ALS	SCGFβ	21	MR-Egger	-0.203	0.131	0.138
ALS	SCGFβ	21	Weighted median	-0.118	0.089	0.187
ALS	SCGFβ	21	Inverse variance weighted	-0.079	0.064	0.221
ALS	SCGFβ	21	MR-PRESSO	/	/	0.809
ALS	SDF-1A	21	MR-Egger	0.011	0.089	0.904
ALS	SDF-1A	21	Weighted median	0.002	0.059	0.976
ALS	SDF-1A	21	Inverse variance weighted	0.034	0.044	0.434
ALS	SDF-1A	21	MR-PRESSO	/	/	0.961
ALS	TNF-A	21	MR-Egger	-0.020	0.156	0.900
ALS	TNF-A	21	Weighted median	-0.103	0.094	0.273
ALS	TNF-A	21	Inverse variance weighted	-0.060	0.075	0.421
ALS	TNF-A	21	MR-PRESSO	/	/	0.145
ALS	TNF-B	16	MR-Egger	0.384	0.401	0.354
ALS	TNF-B	16	Weighted median	0.107	0.179	0.550
ALS	TNF-B	16	Inverse variance weighted	0.097	0.124	0.433
ALS	TNF-B	16	MR-PRESSO	/	/	0.590
ALS	TRAIL	21	MR-Egger	-0.121	0.116	0.309
ALS	TRAIL	21	Weighted median	-0.026	0.066	0.692
ALS	TRAIL	21	Inverse variance weighted	-0.036	0.057	0.525
ALS	TRAIL	21	MR-PRESSO	/	/	0.306
ALS	VEGF	21	MR-Egger	-0.088	0.093	0.357
ALS	VEGF	21	Weighted median	-0.099	0.065	0.127
ALS	VEGF	21	Inverse variance weighted	-0.067	0.046	0.144
ALS	VEGF	21	MR-PRESSO	/	/	0.911

Supplementary Figures

eFigure 1. BNGF-associated SNPs with risk of Alzheimer's disease (AD)

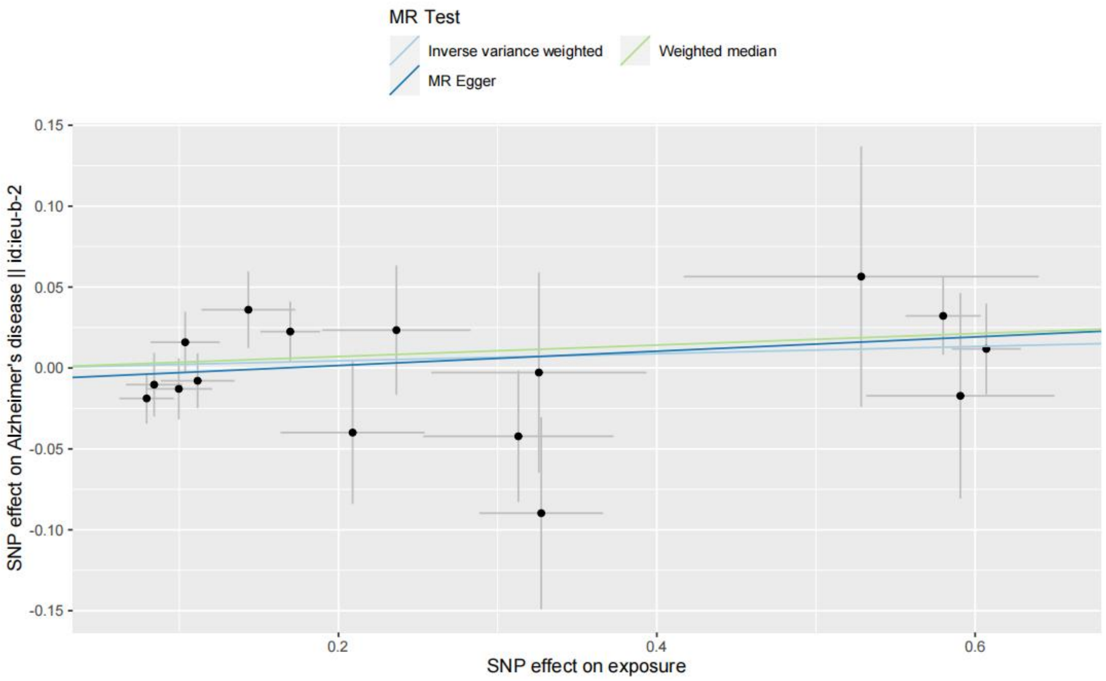

A. Scatter plot of BNGF with risk of AD

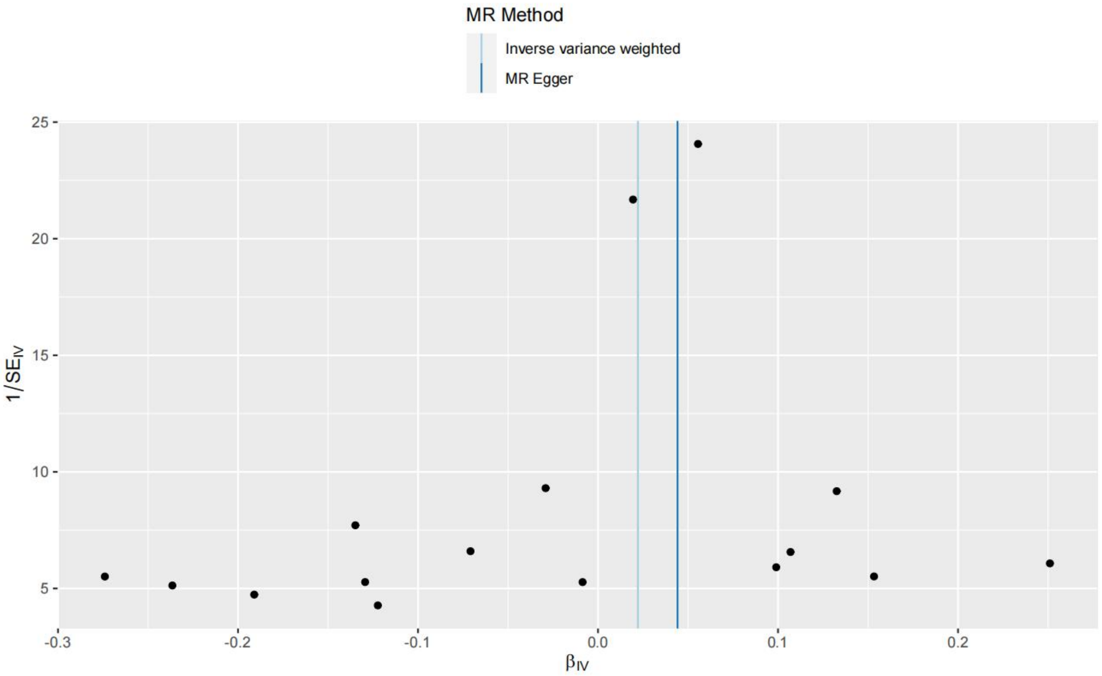

B. Funnel plot of BNGF instruments strength on AD

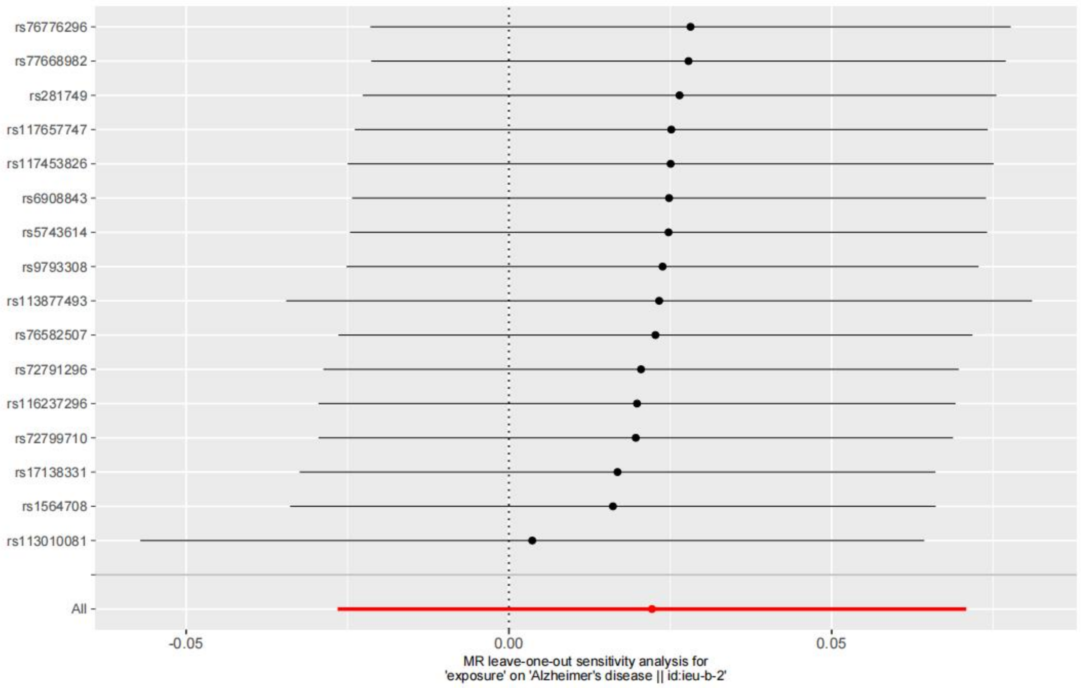

C. MR leave-one-out sensitivity analysis for BNGF on AD

eFigure 2. CTACK-associated SNPs with risk of AD

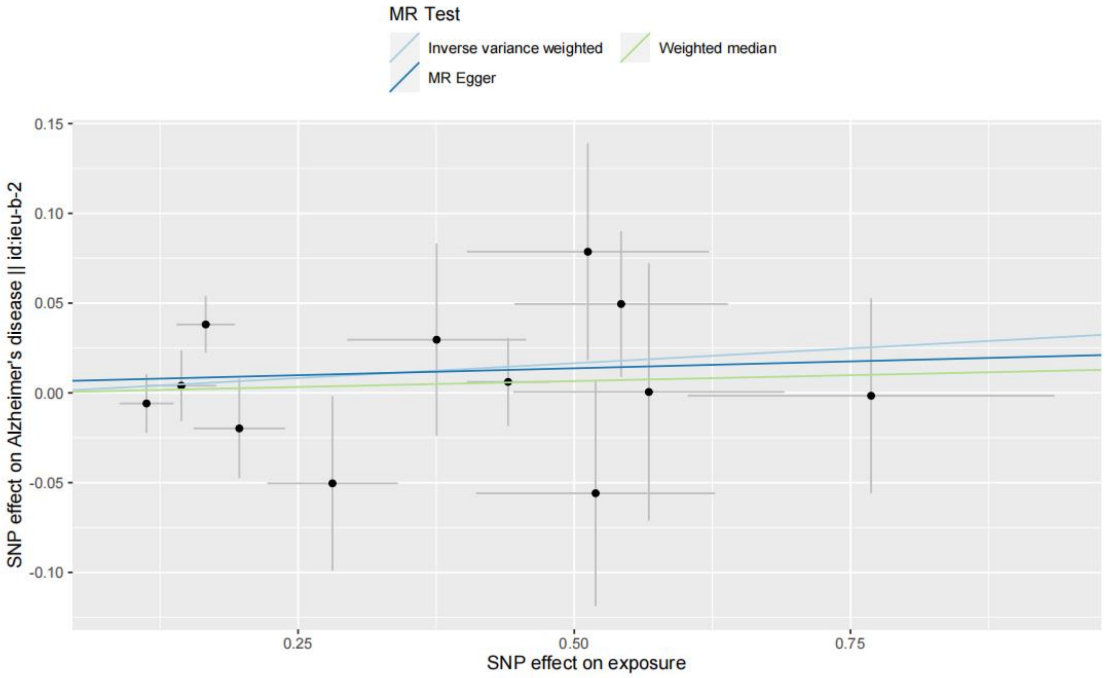

A. Scatter plot of CTACK with risk of AD

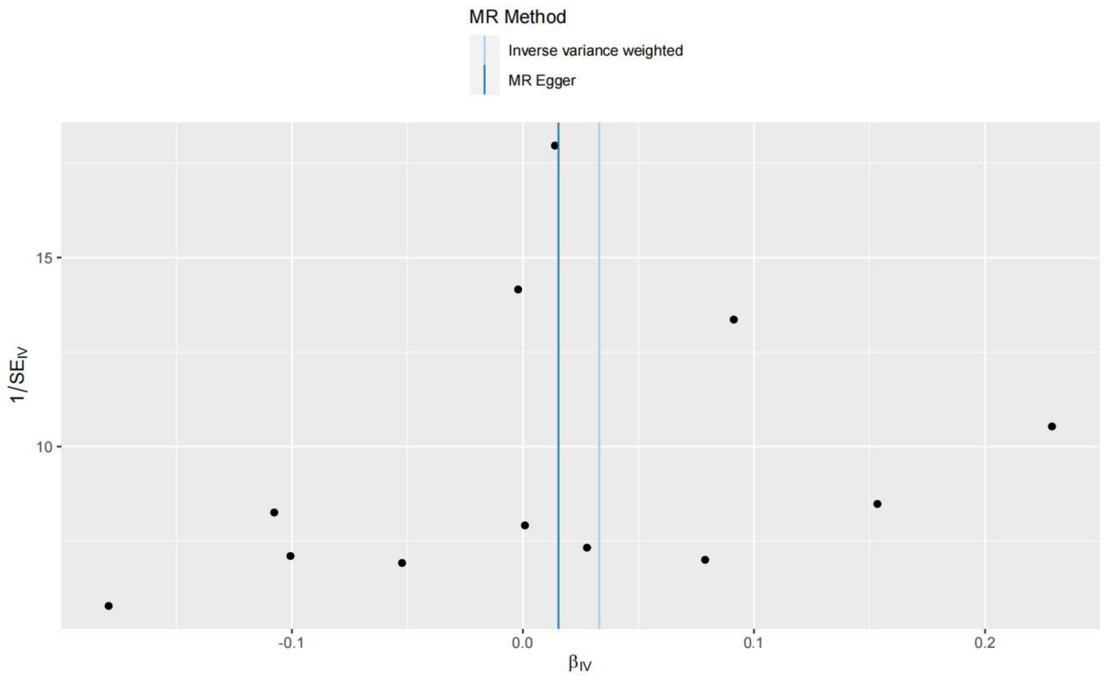

B. Funnel plot of CTACK instruments strength on AD

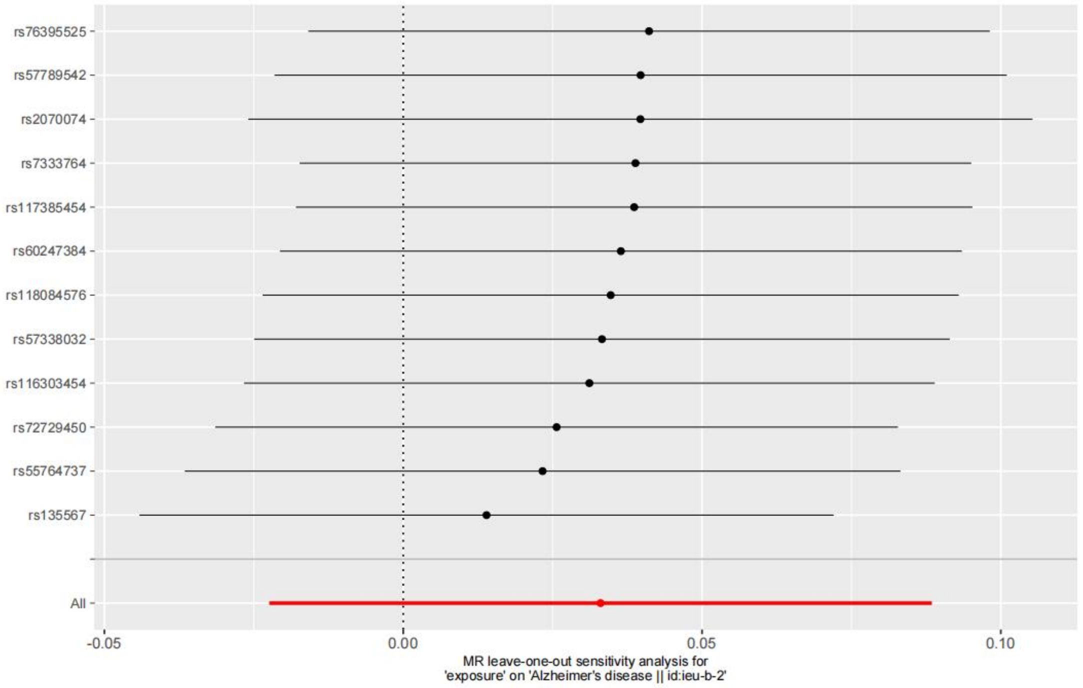

C. MR leave-one-out sensitivity analysis for CTACK-transferase on AD

eFigure 3. EOTAXIN-associated SNPs with risk of AD

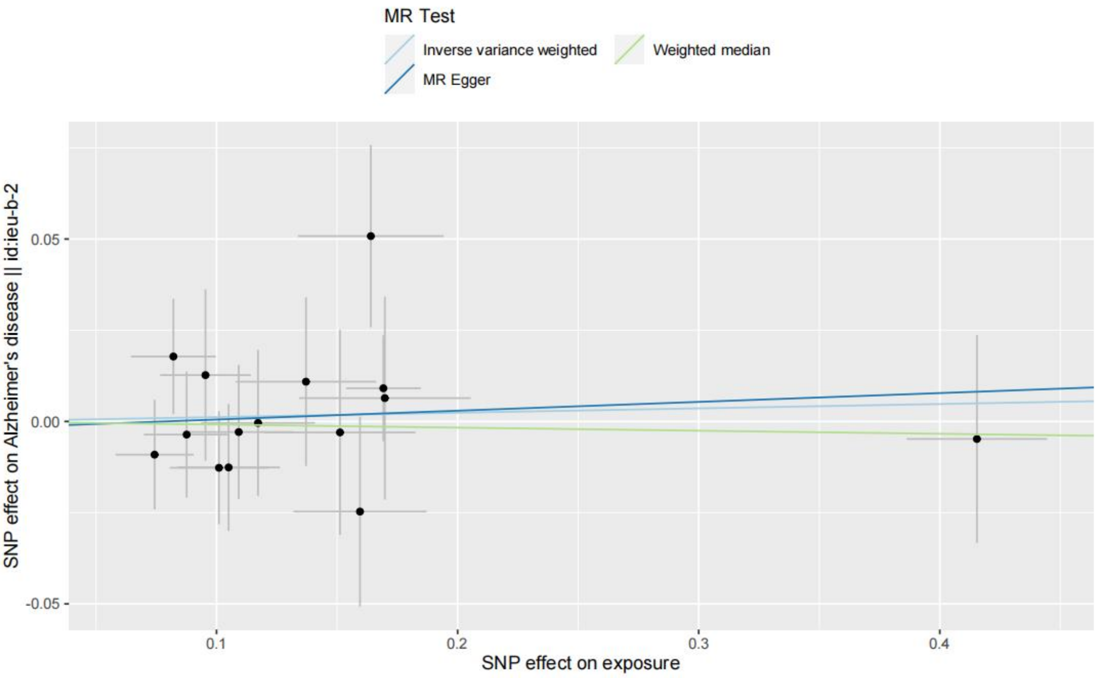

A. Scatter plot of EOTAXIN with risk of AD

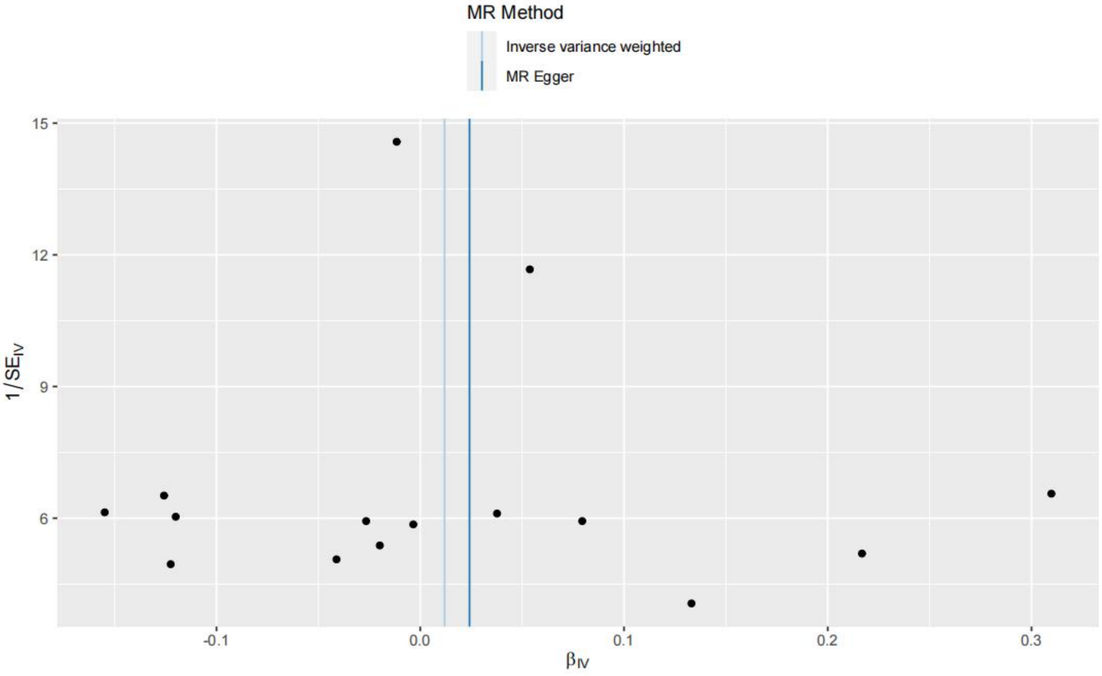

B. Funnel plot of EOTAXIN instruments strength on AD

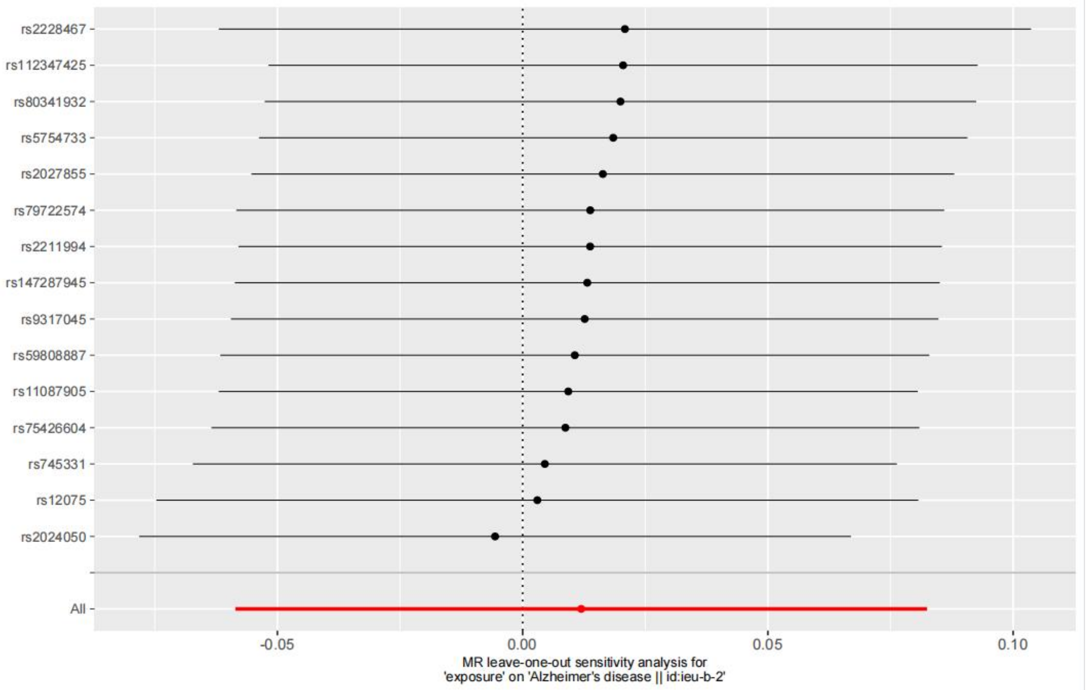

C. MR leave-one-out sensitivity analysis for EOTAXIN on AD

eFigure 4. bFGF-associated SNPs with risk of AD

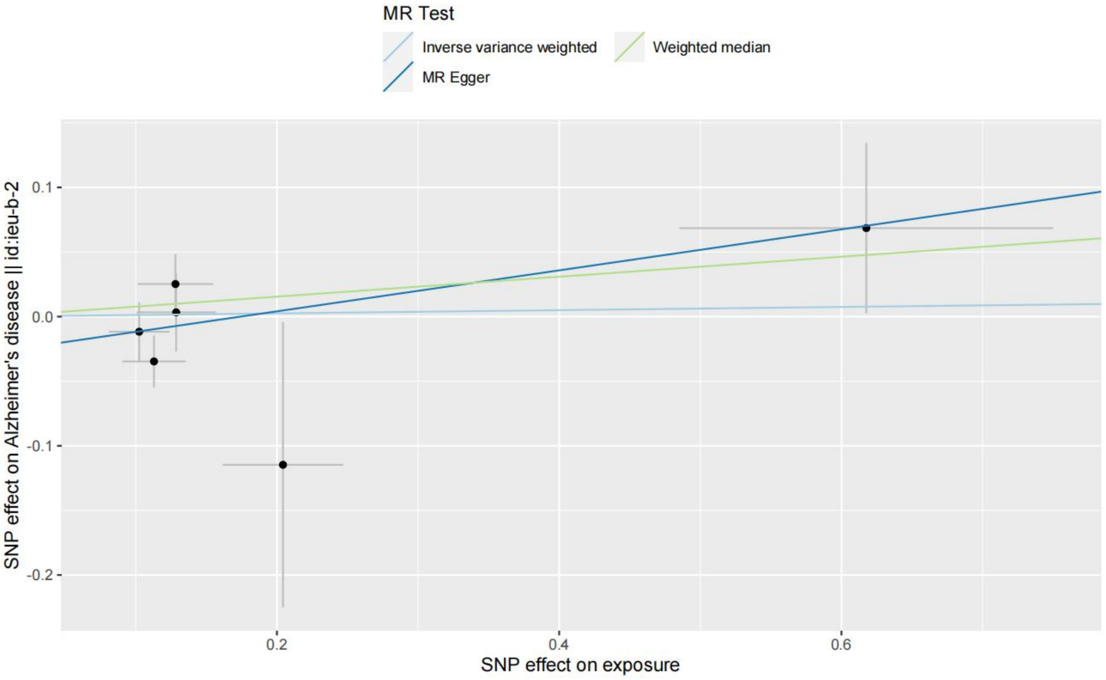

A. Scatter plot of bFGF with risk of AD

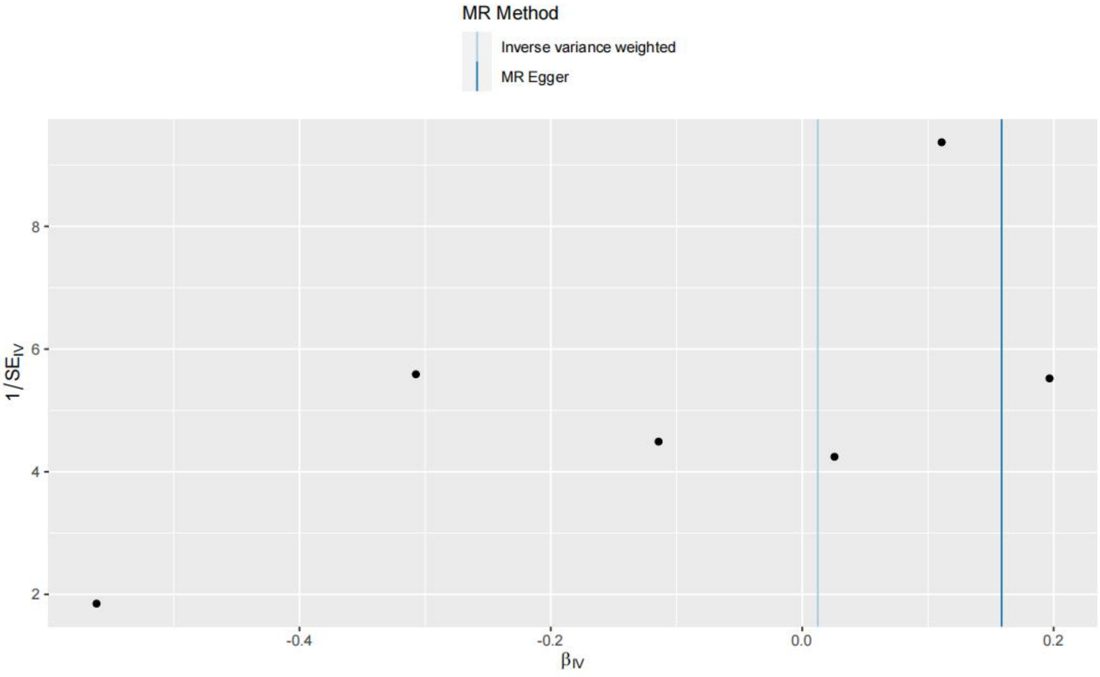

B. Funnel plot of bFGF instruments strength on AD

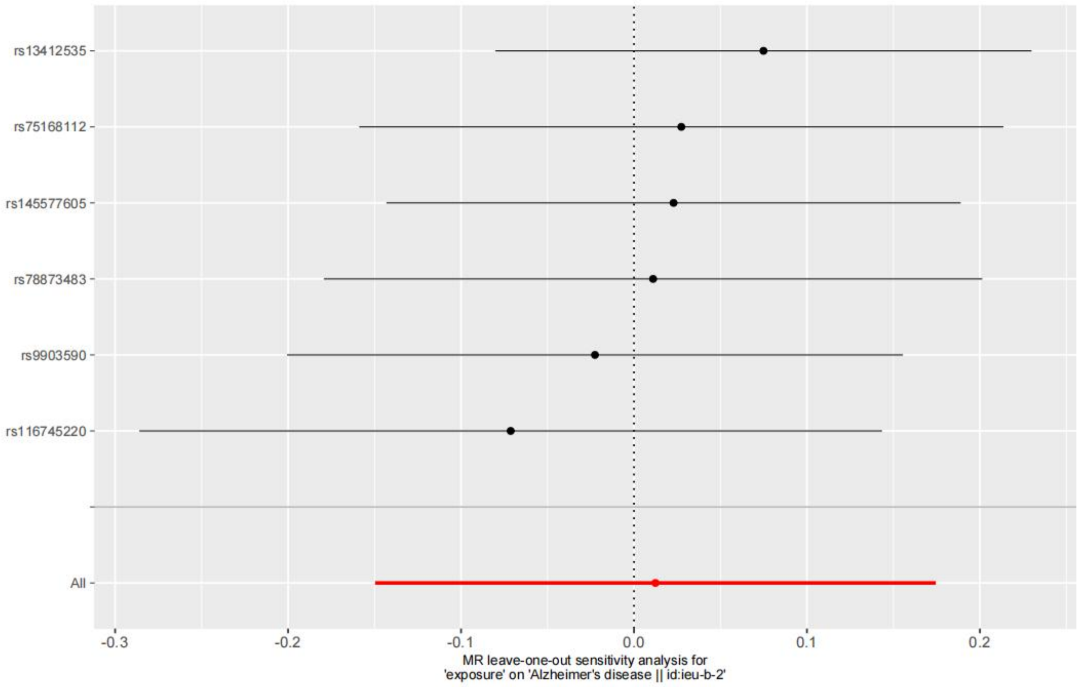

C. MR leave-one-out sensitivity analysis for bFGF on AD

eFigure 5. G-CSF-associated SNPs with risk of AD

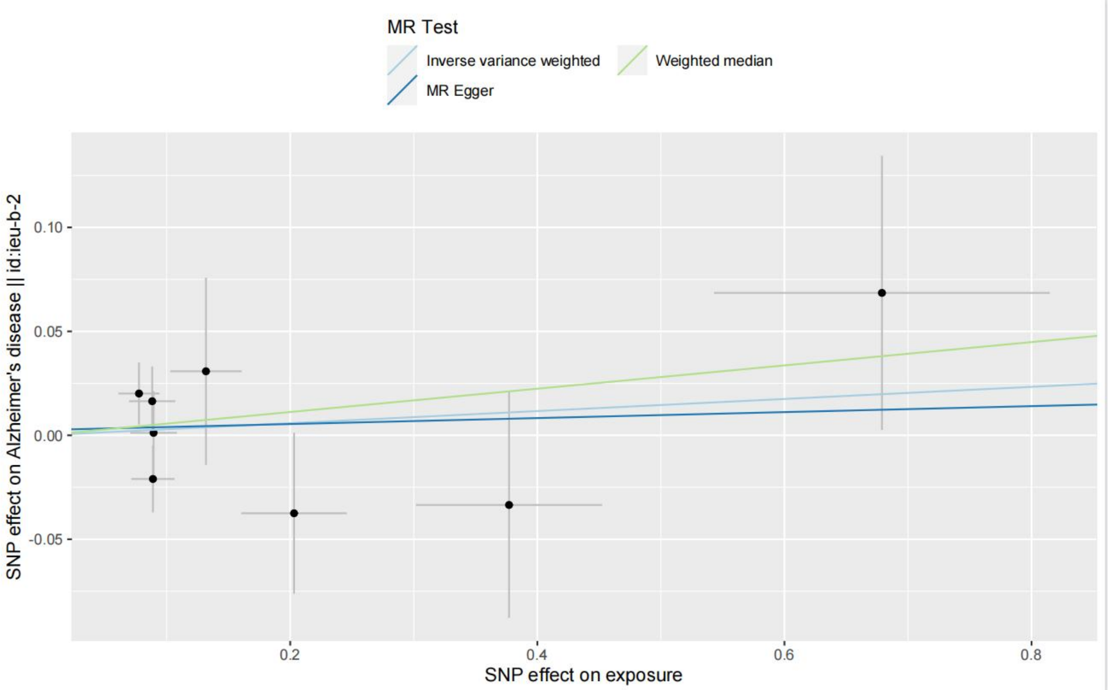

A. Scatter plot of G-CSF with risk of AD

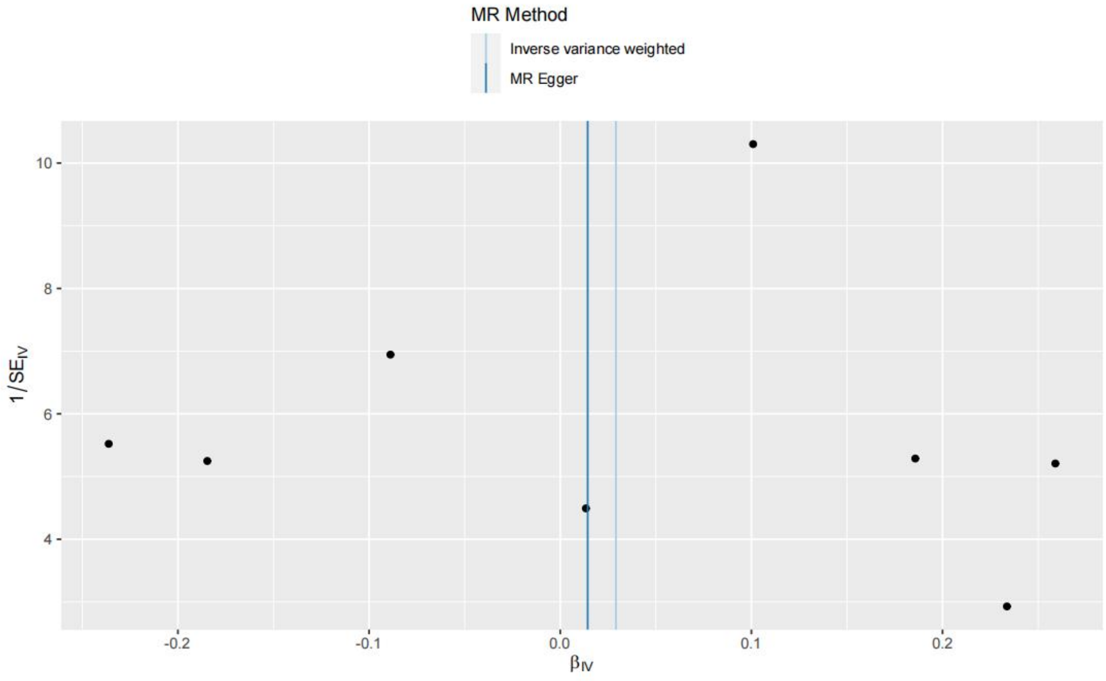

B. Funnel plot of G-CSF instruments strength on AD

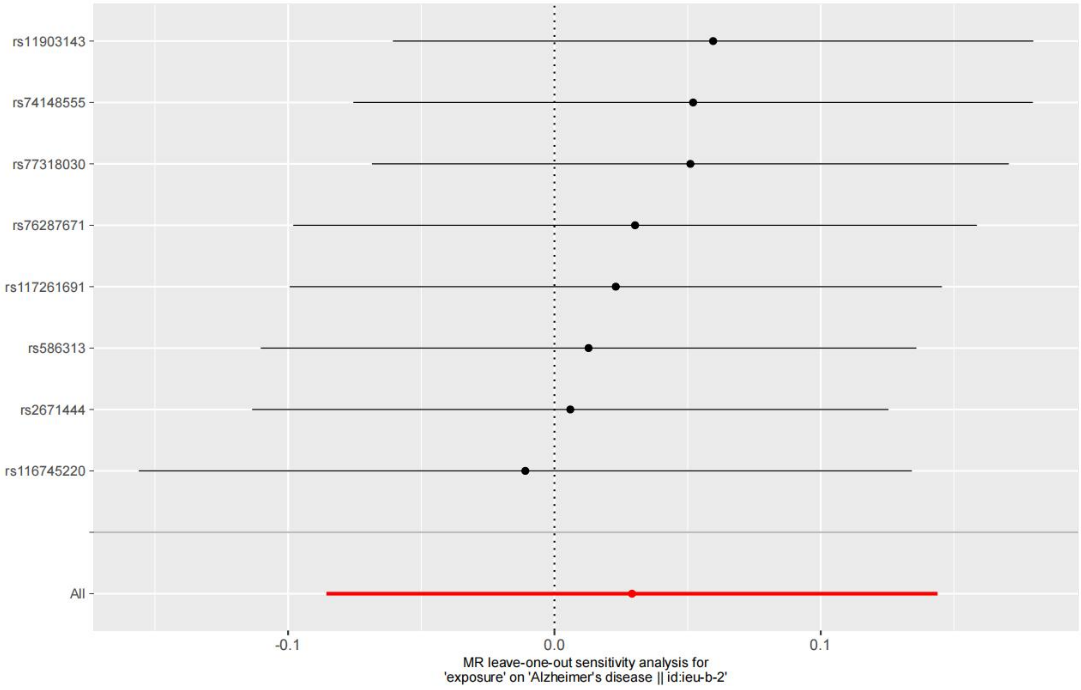

C. MR leave-one-out sensitivity analysis for G-CSF on AD

eFigure 6. GROA-associated SNPs with risk of AD

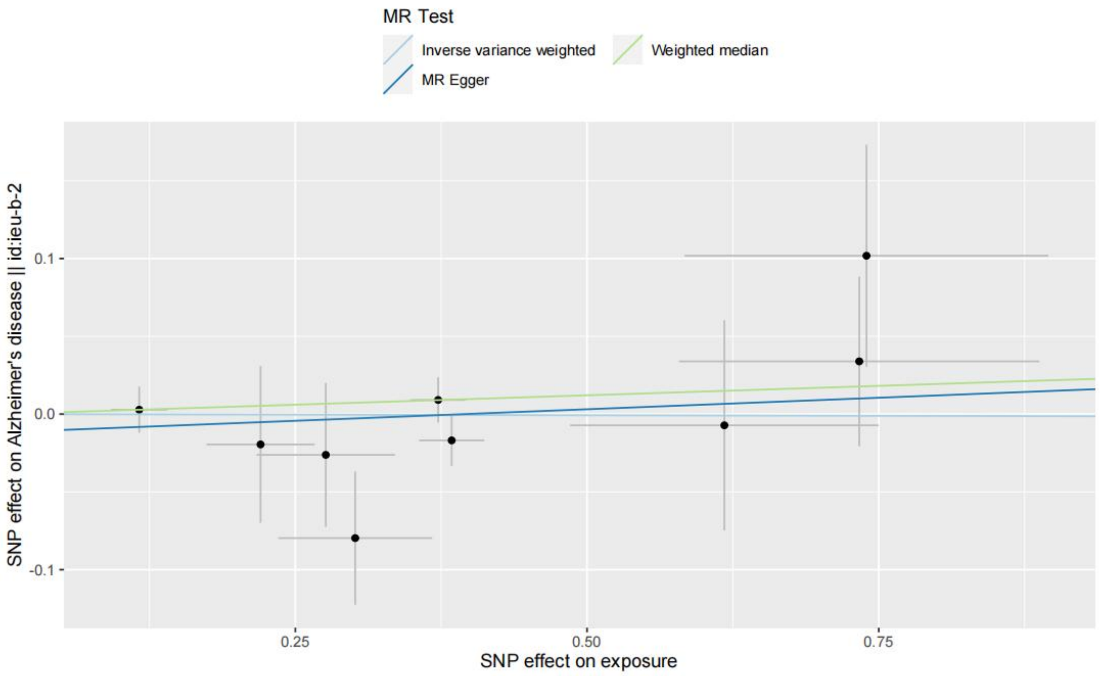

A. Scatter plot of GROA with risk of AD

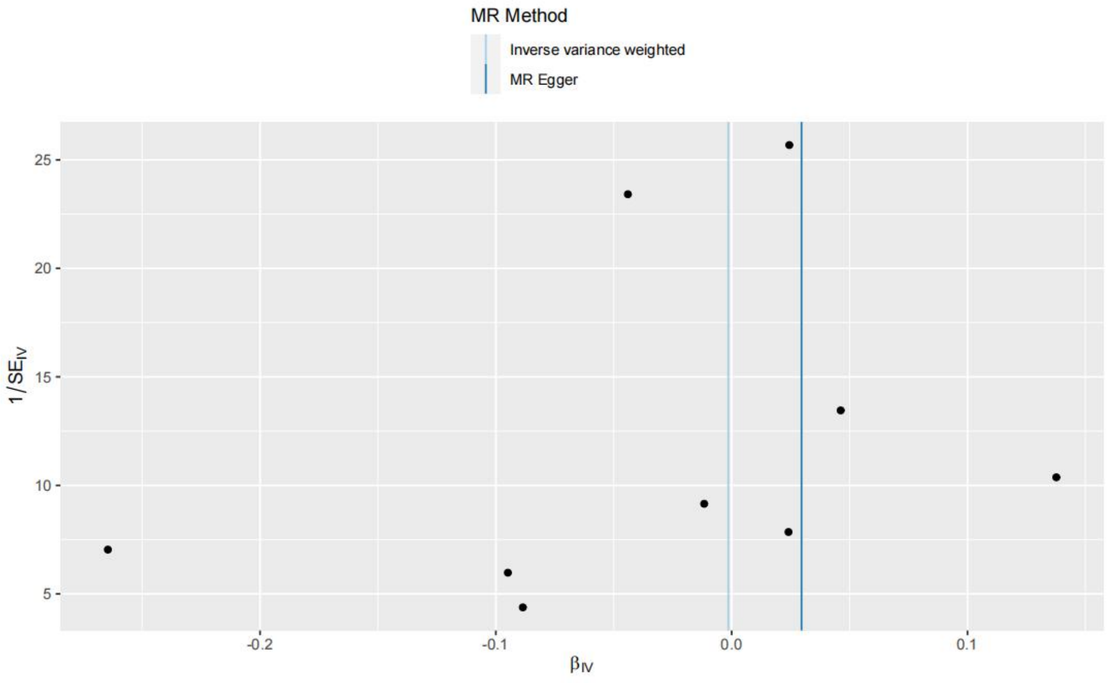

B. Funnel plot of GROA instruments strength on AD

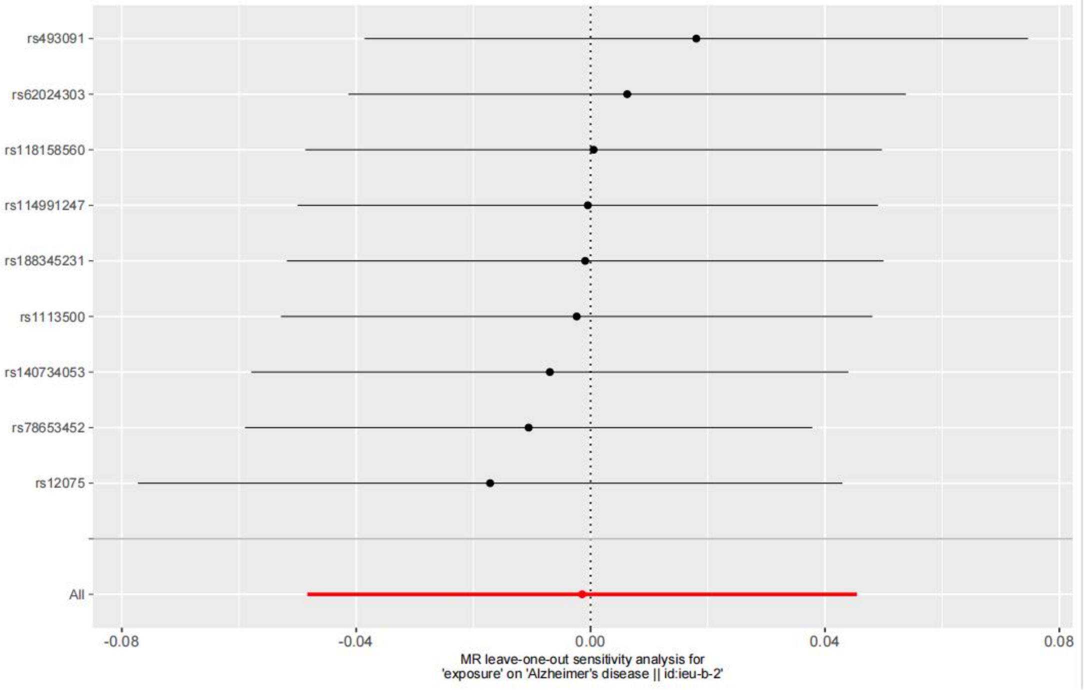

C. MR leave-one-out sensitivity analysis for GROA on AD

eFigure 7. HGF-associated SNPs with risk of AD

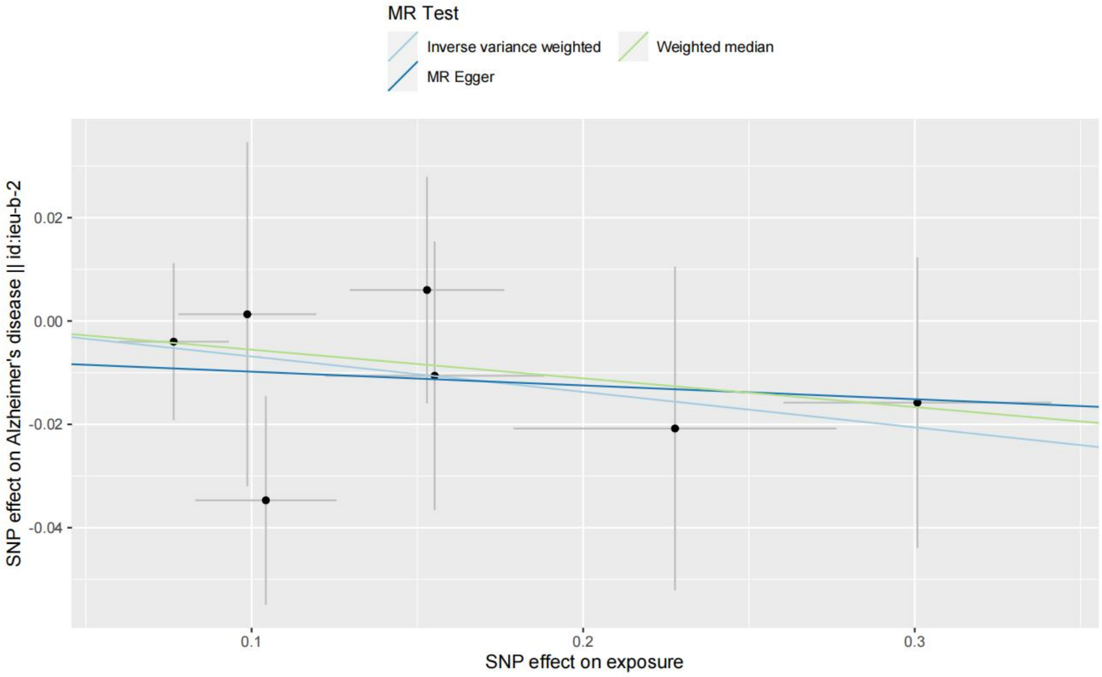

A. Scatter plot of HGF with risk of AD

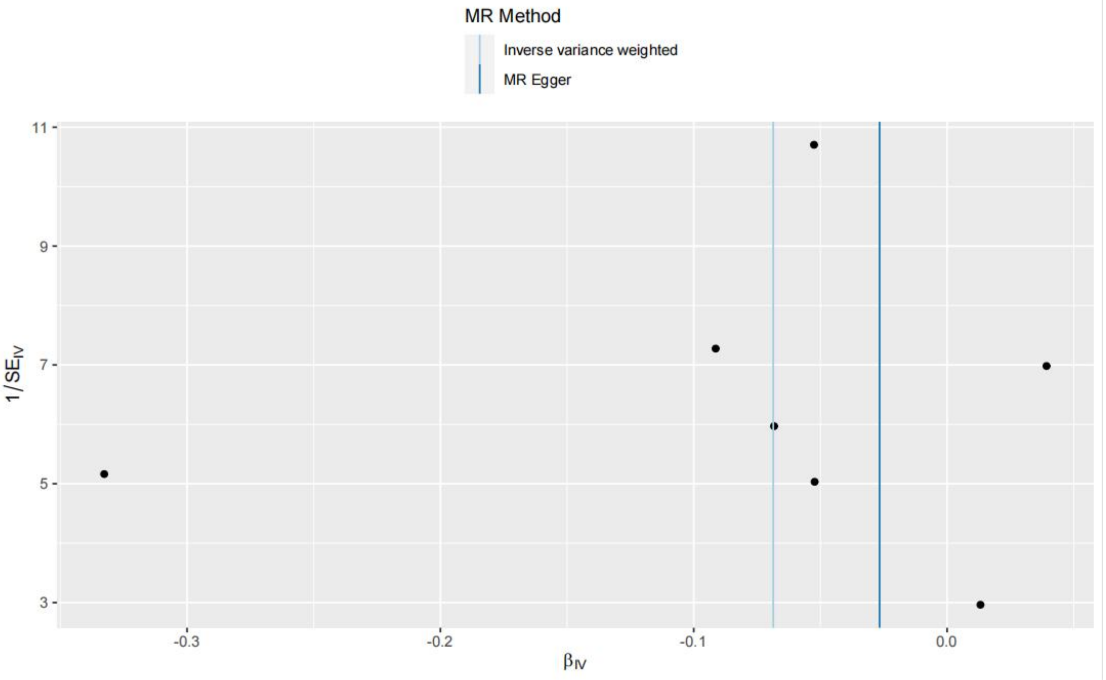

B. Funnel plot of HGF instruments strength on AD

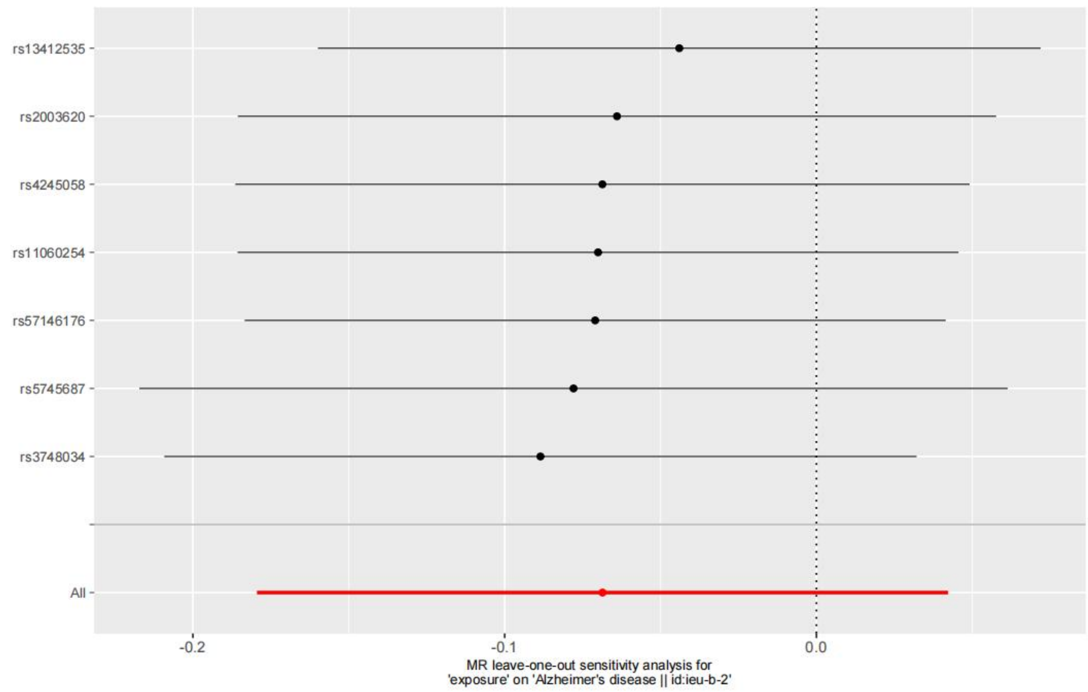

C. MR leave-one-out sensitivity analysis for HGF on AD

eFigure 8. IFN-G-associated SNPs with risk of AD

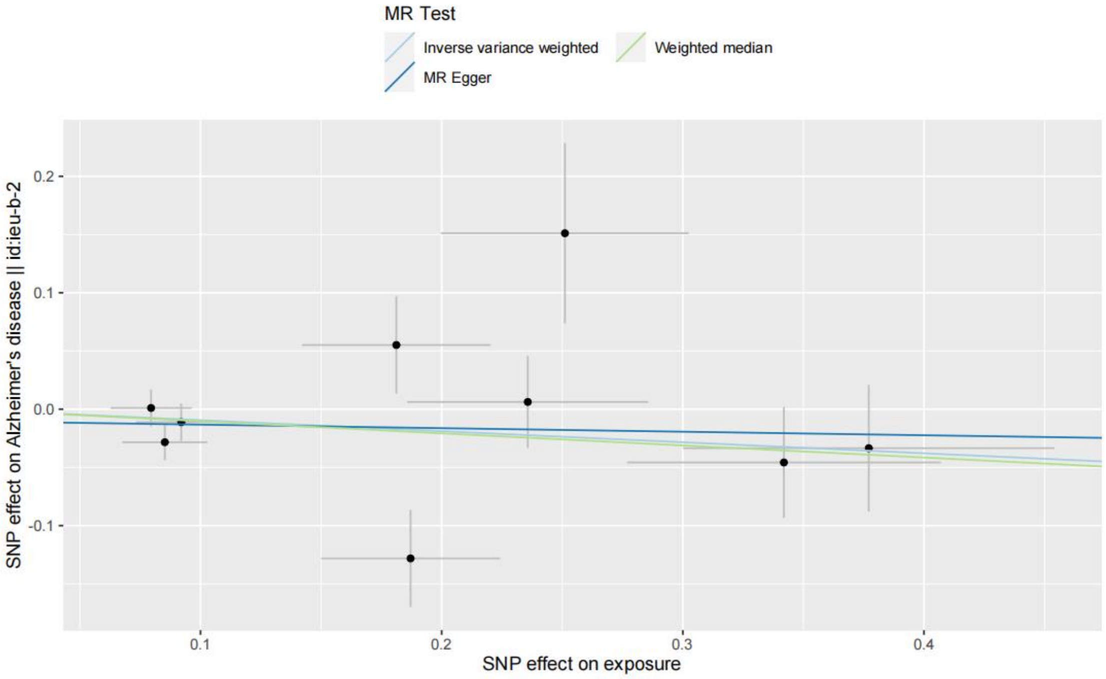

A. Scatter plot of IFN-G with risk of AD

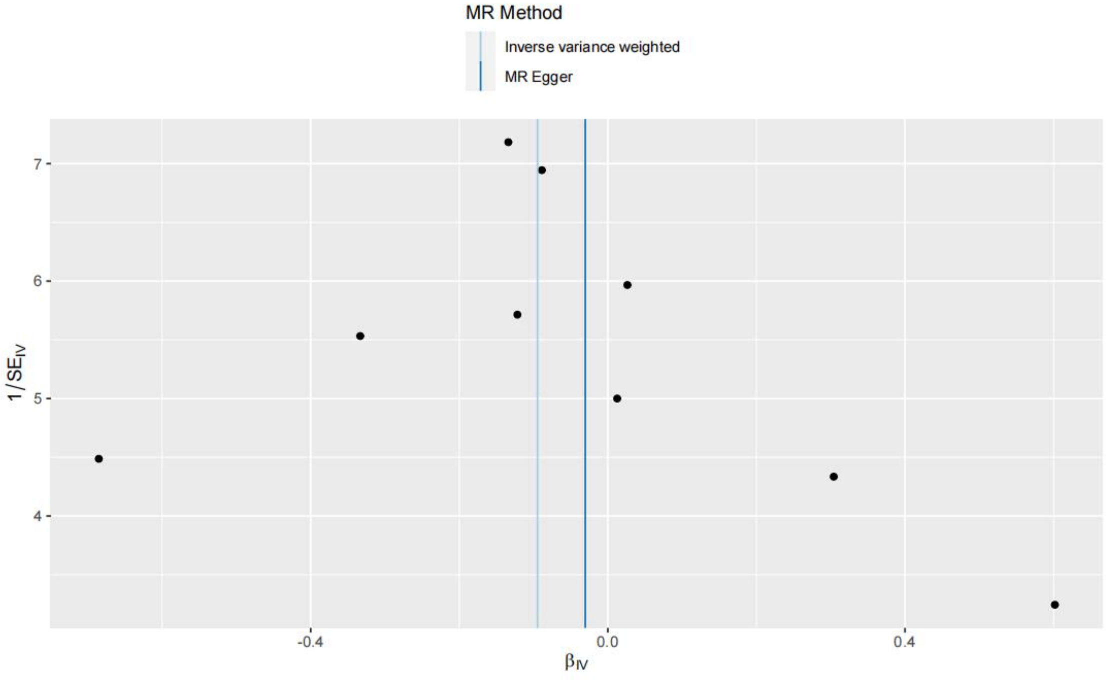

B. Funnel plot of IFN-G instruments strength on AD

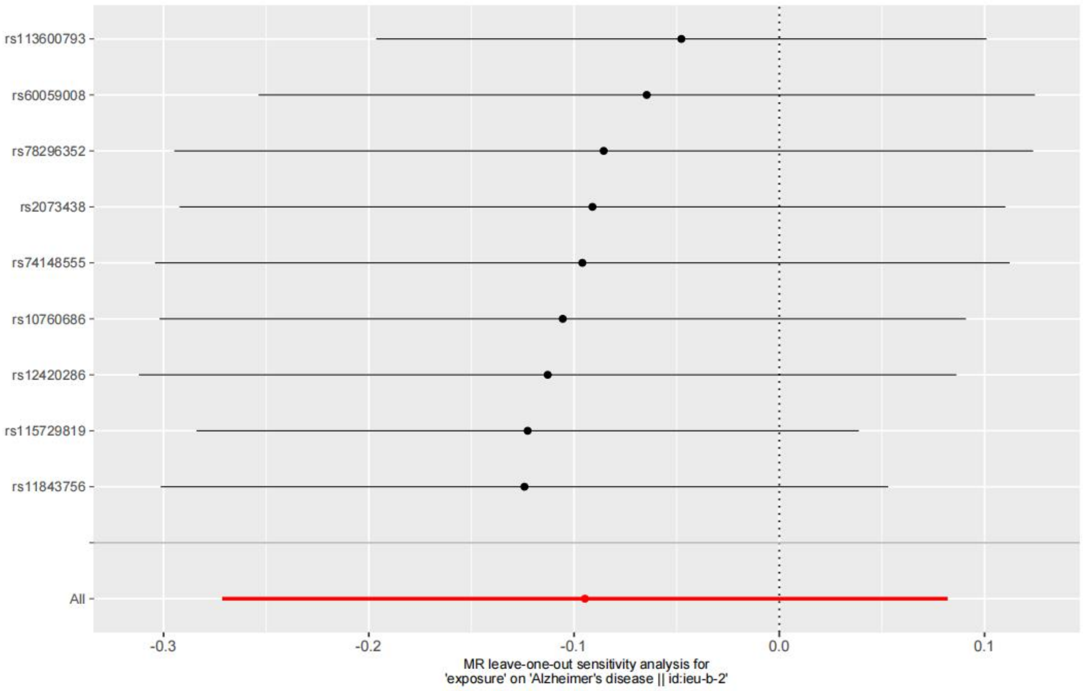

C. MR leave-one-out sensitivity analysis for IFN-G on AD

eFigure 9. IL-1B-associated SNPs with risk of AD

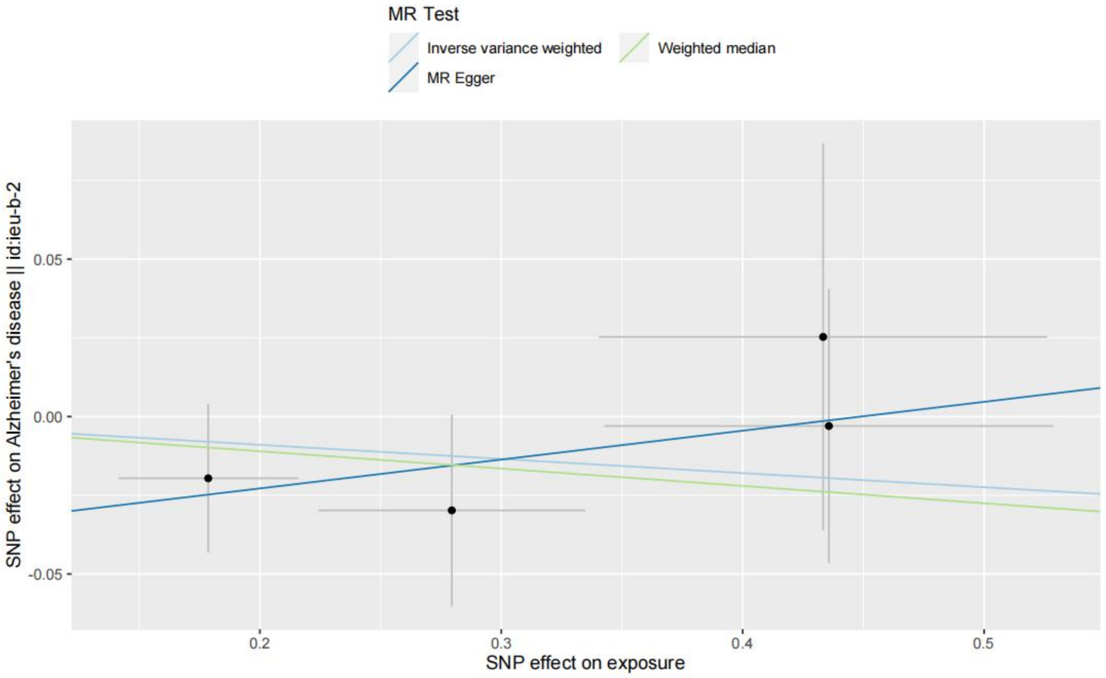

A. Scatter plot of IL-1B with risk of AD

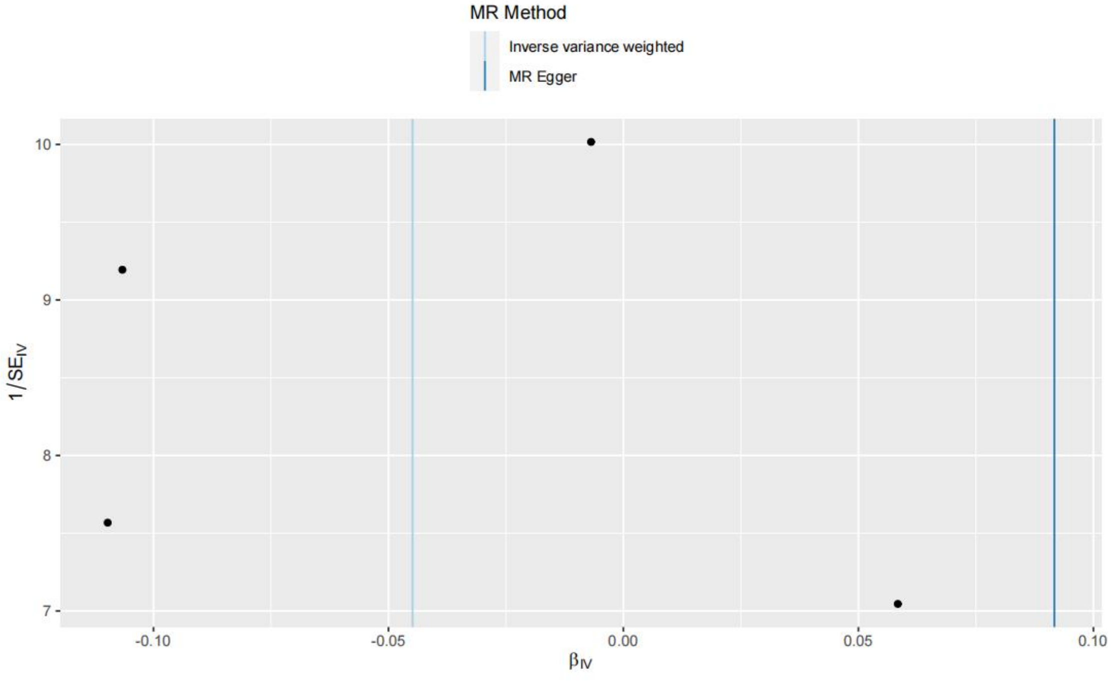

B. Funnel plot of IL-1B instruments strength on AD

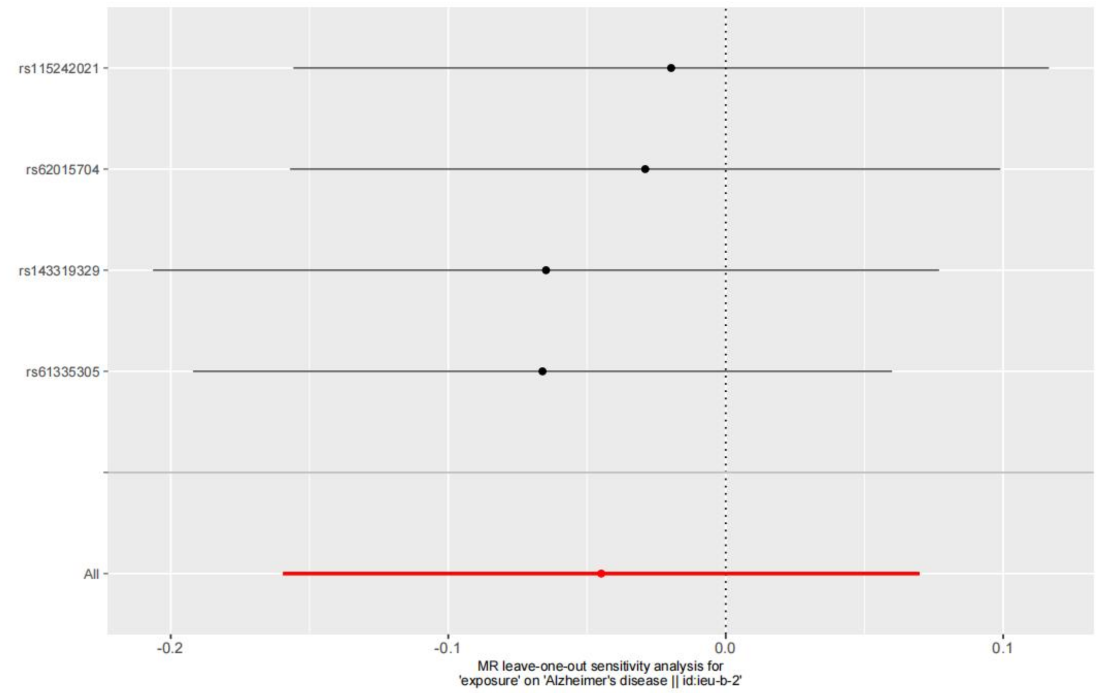

C. MR leave-one-out sensitivity analysis for IL-1B on AD

eFigure 10. IL-1RA-associated SNPs with risk of AD

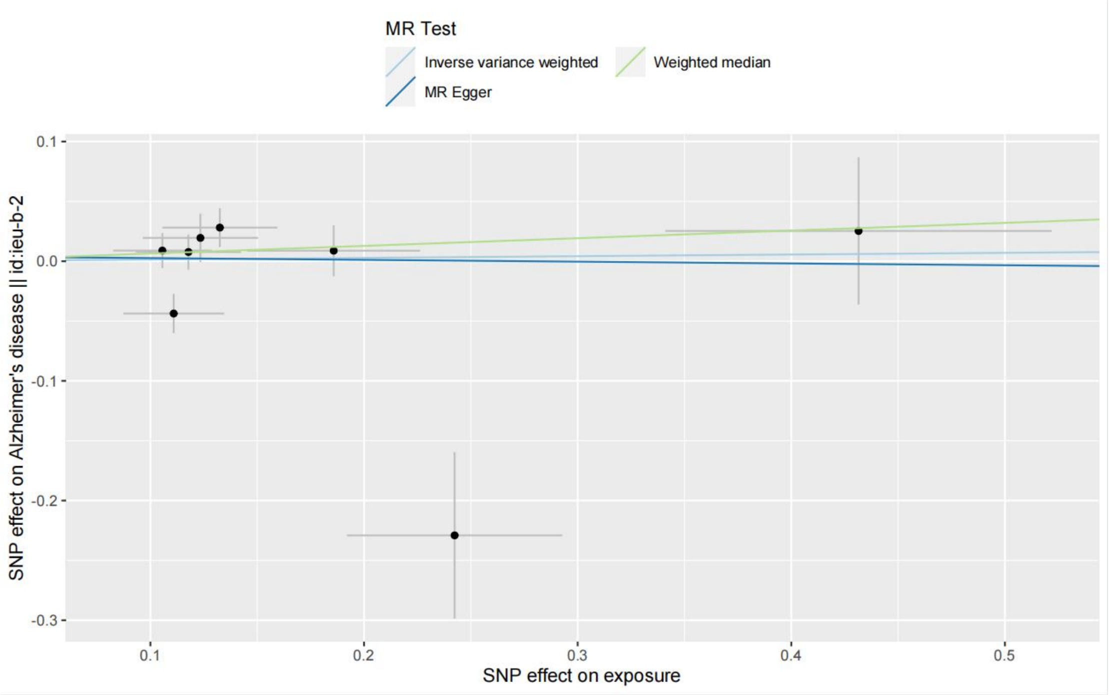

A. Scatter plot of IL-1RA with risk of AD

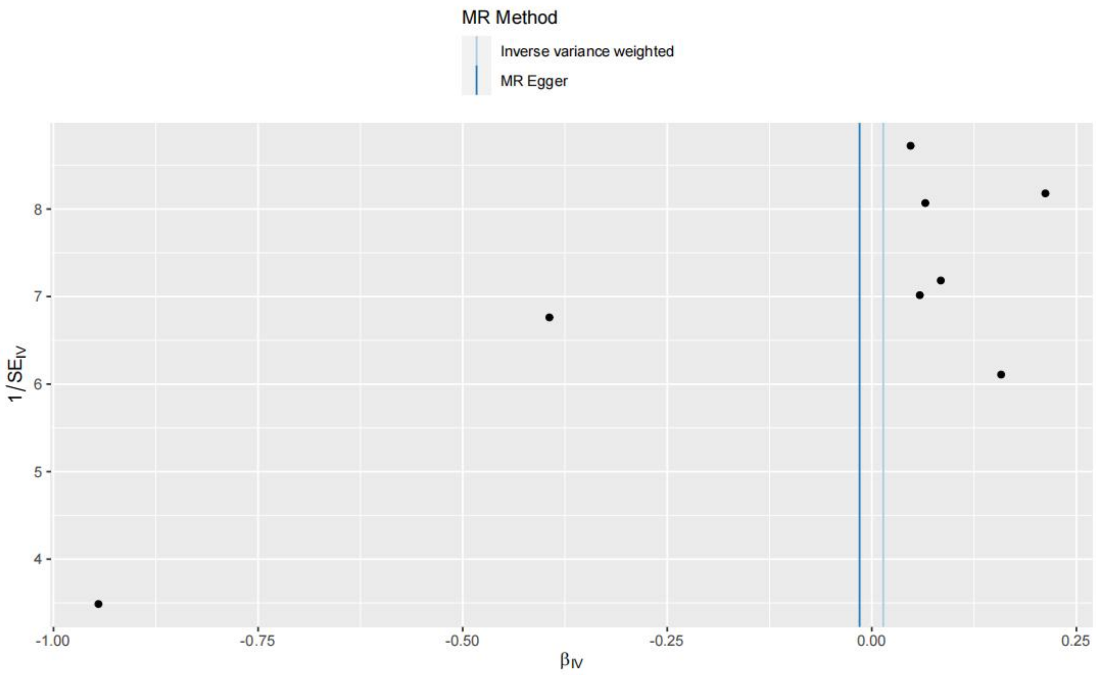

B. Funnel plot of IL-1RA instruments strength on AD

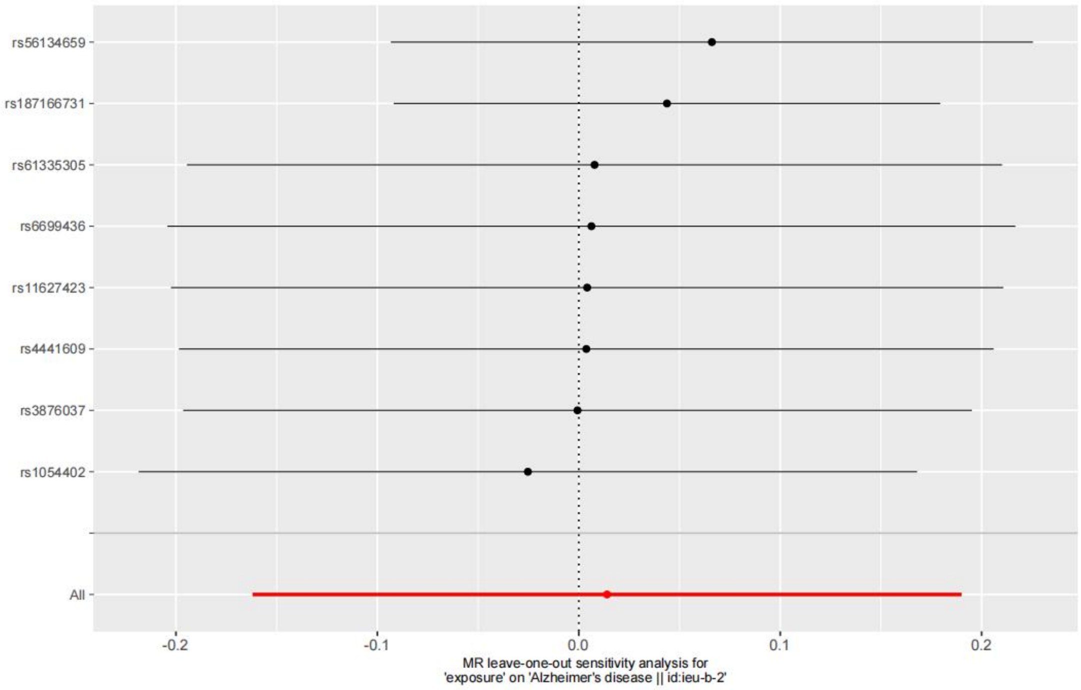

C. MR leave-one-out sensitivity analysis for IL-1RA on AD

eFigure 11. IL-2-associated SNPs with risk of AD

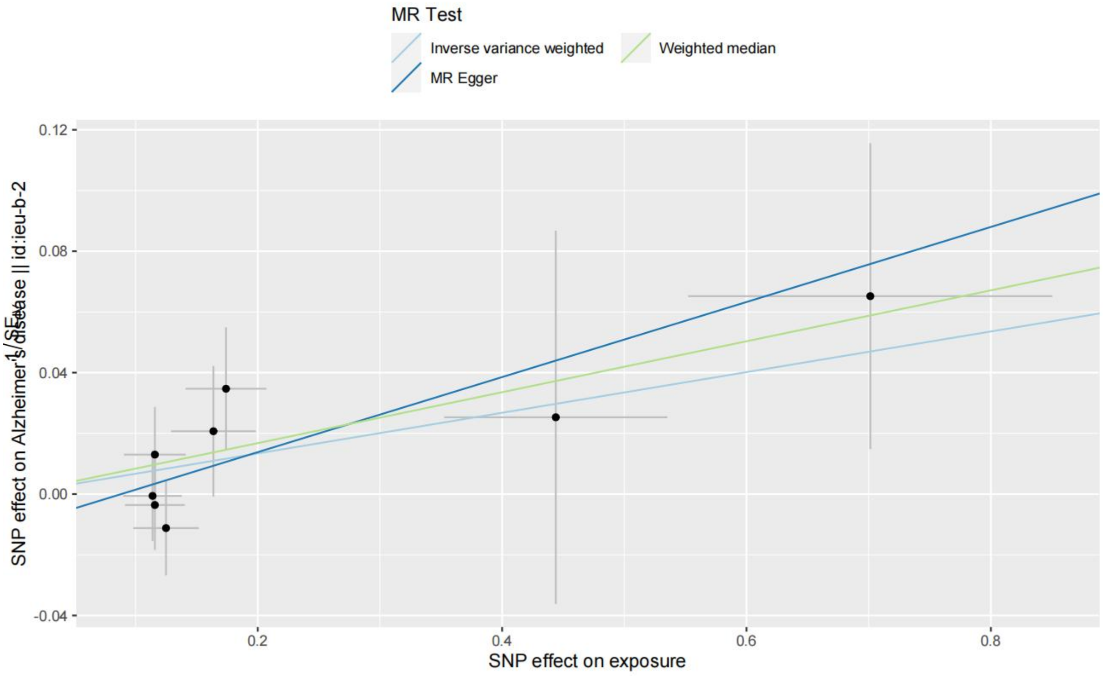

A. Scatter plot of IL-2 with risk of AD

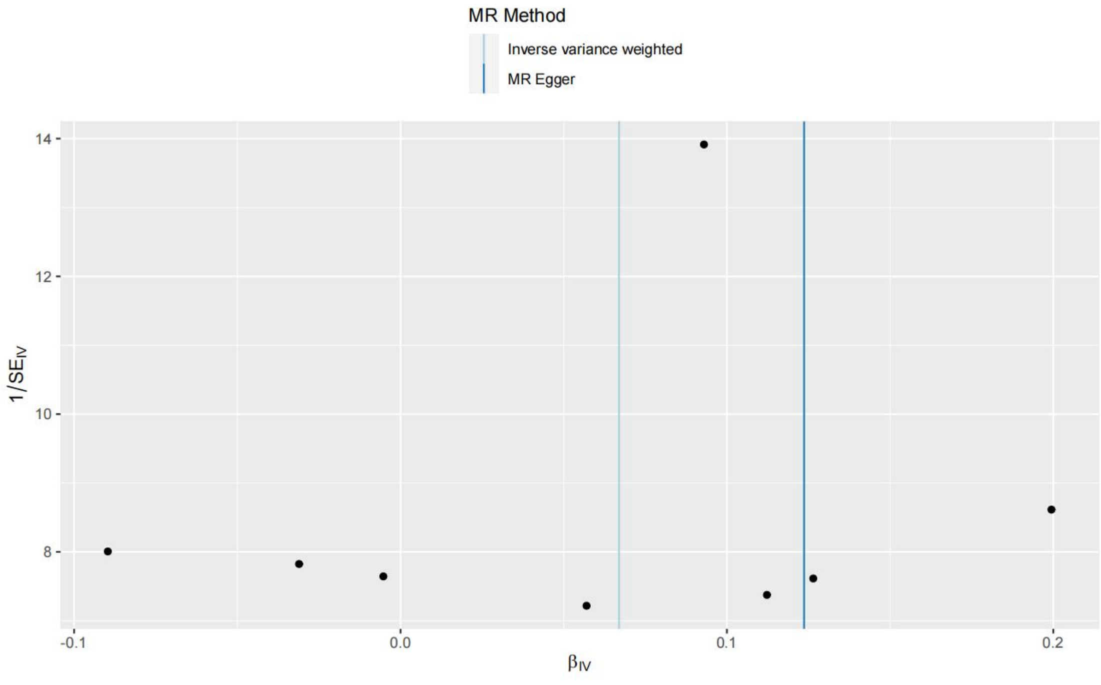

B. Funnel plot of IL-2 instruments strength on AD

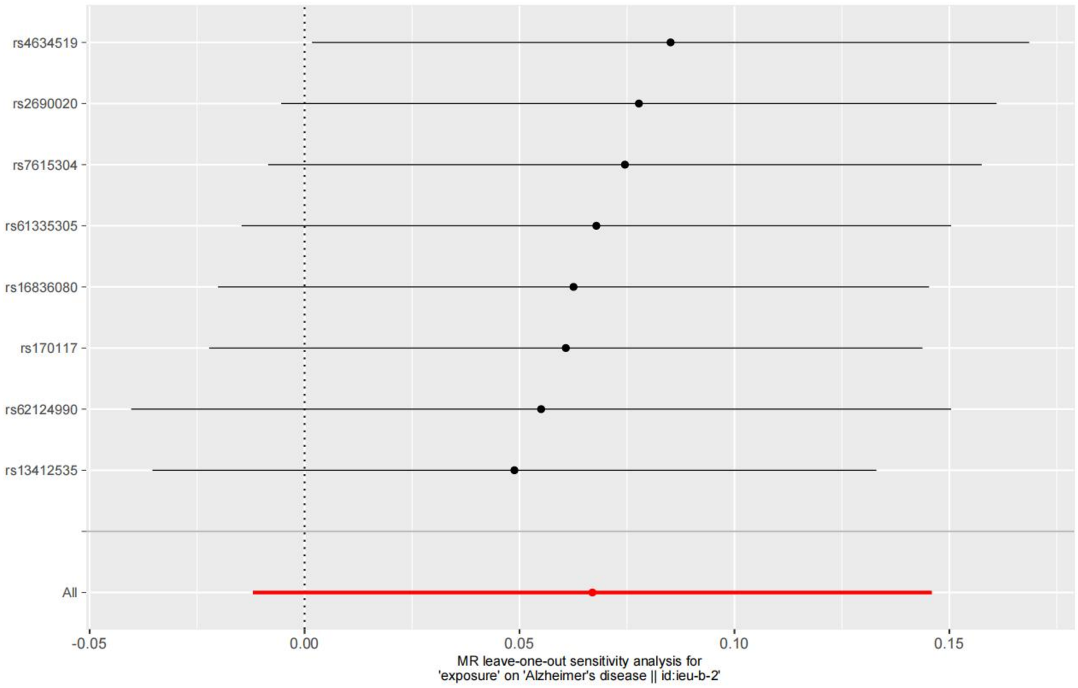

C. MR leave-one-out sensitivity analysis for IL-2 on AD

eFigure 12. IL-2RA-associated SNPs with risk of AD

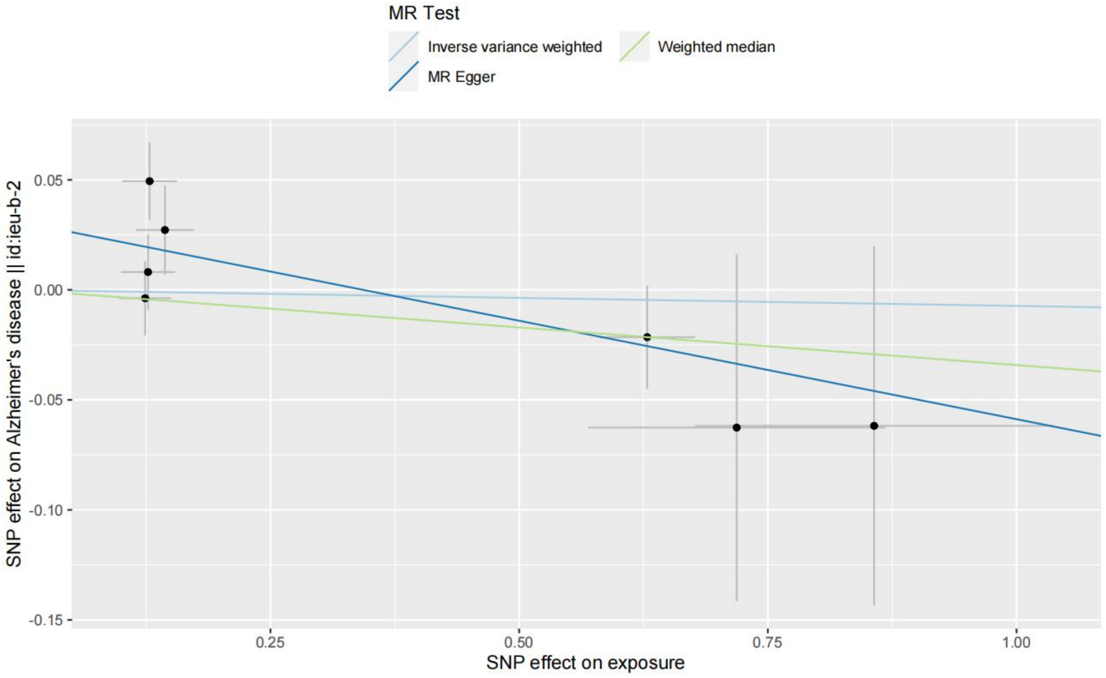

A. Scatter plot of IL-2RA with risk of AD

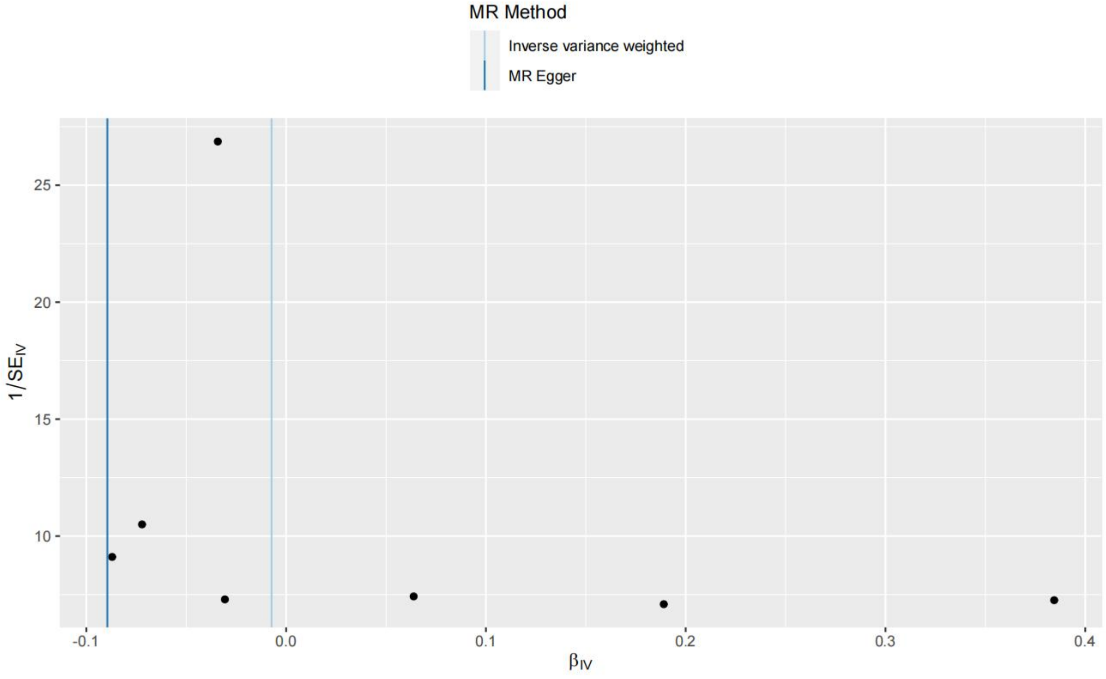

B. Funnel plot of IL-2RA instruments strength on AD

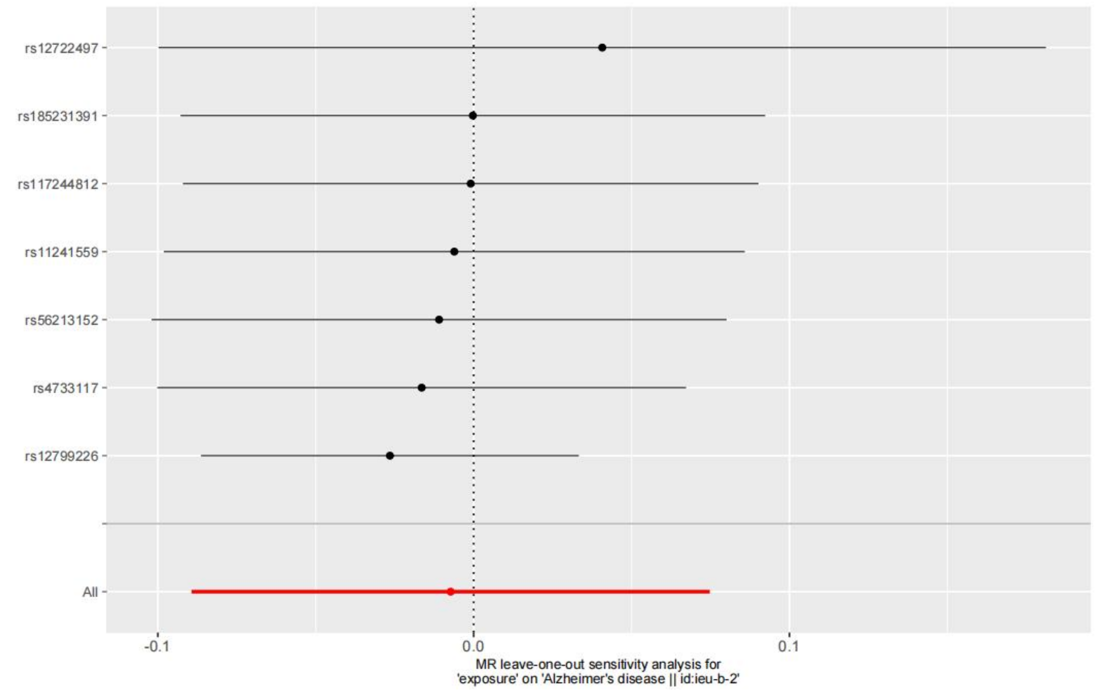

C. MR leave-one-out sensitivity analysis for IL-2RA on AD

eFigure 13. IL-4-associated SNPs with risk of AD

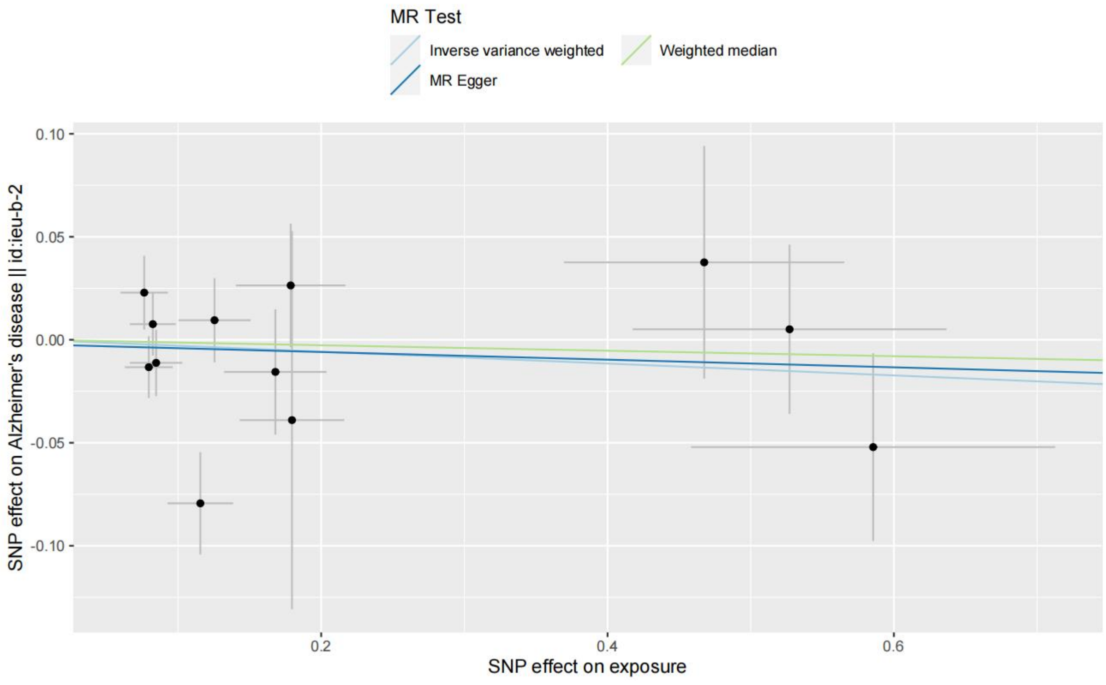

A. Scatter plot of IL-4 with risk of AD

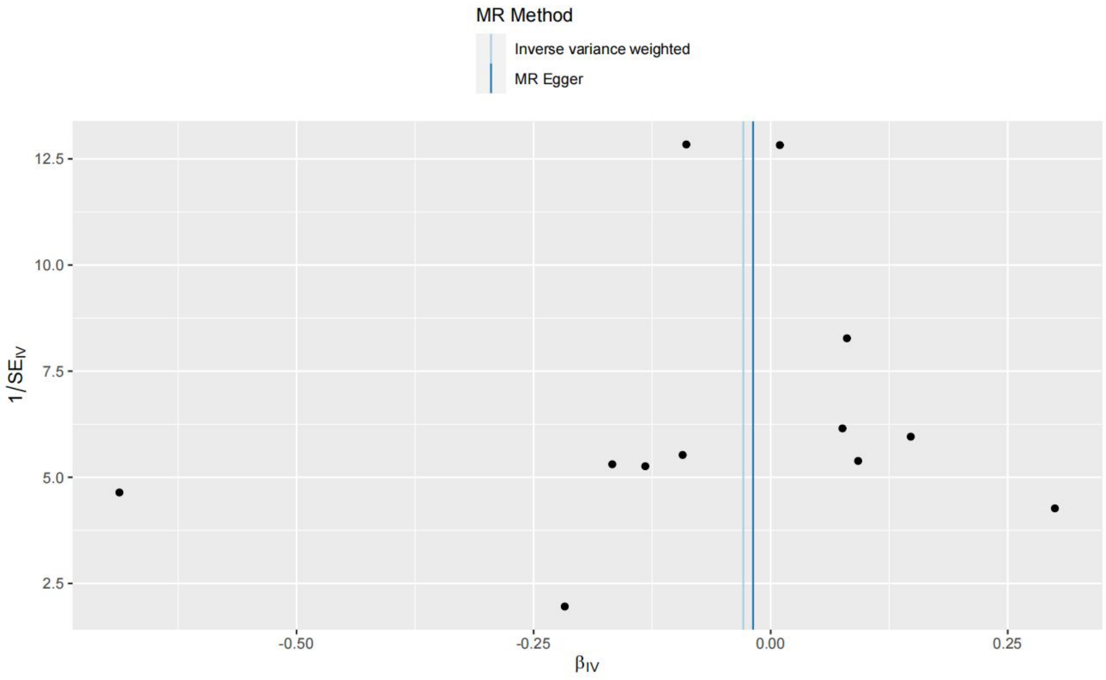

B. Funnel plot of IL-4 instruments strength on AD

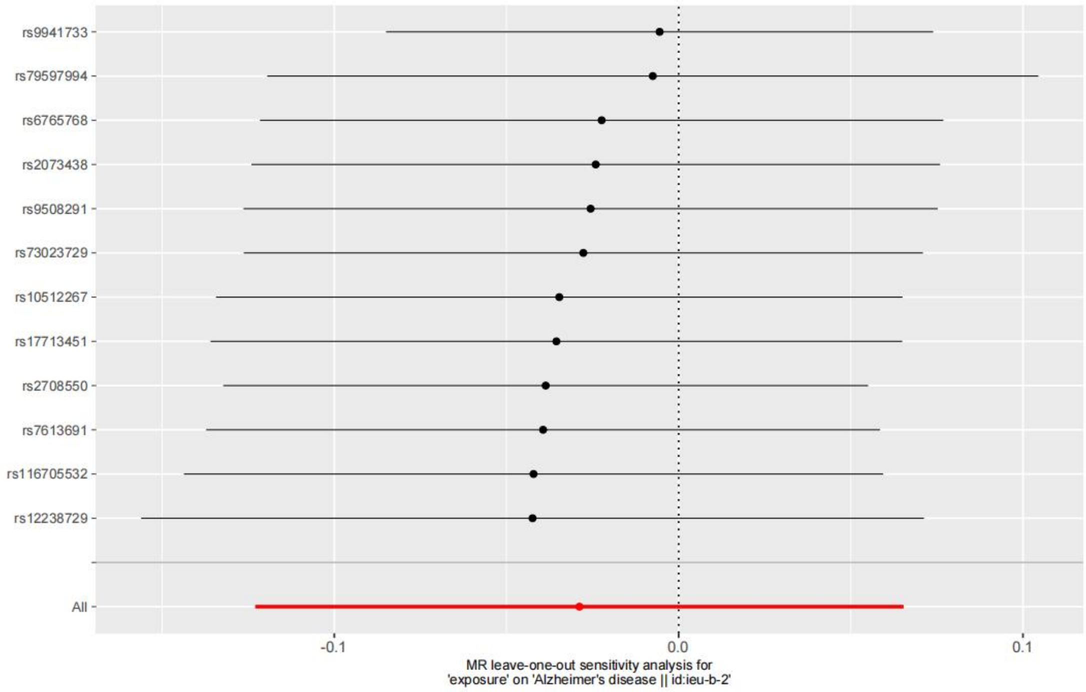

C. MR leave-one-out sensitivity analysis for IL-4 on AD

eFigure 14. IL-5-associated SNPs with risk of AD

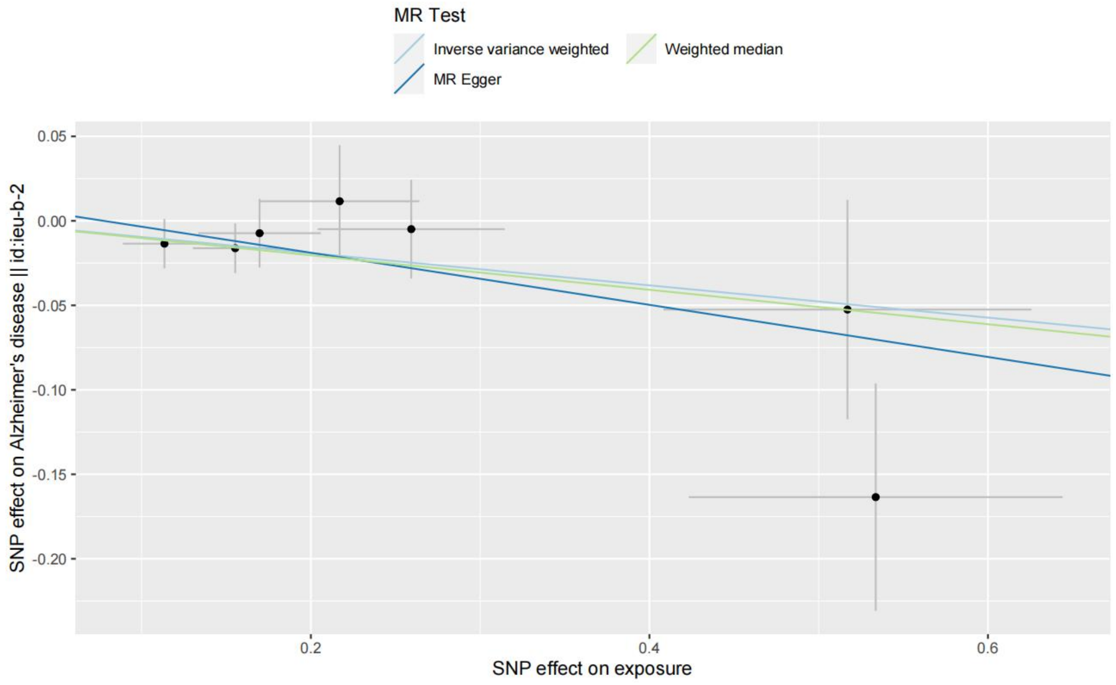

A. Scatter plot of IL-5 with risk of AD

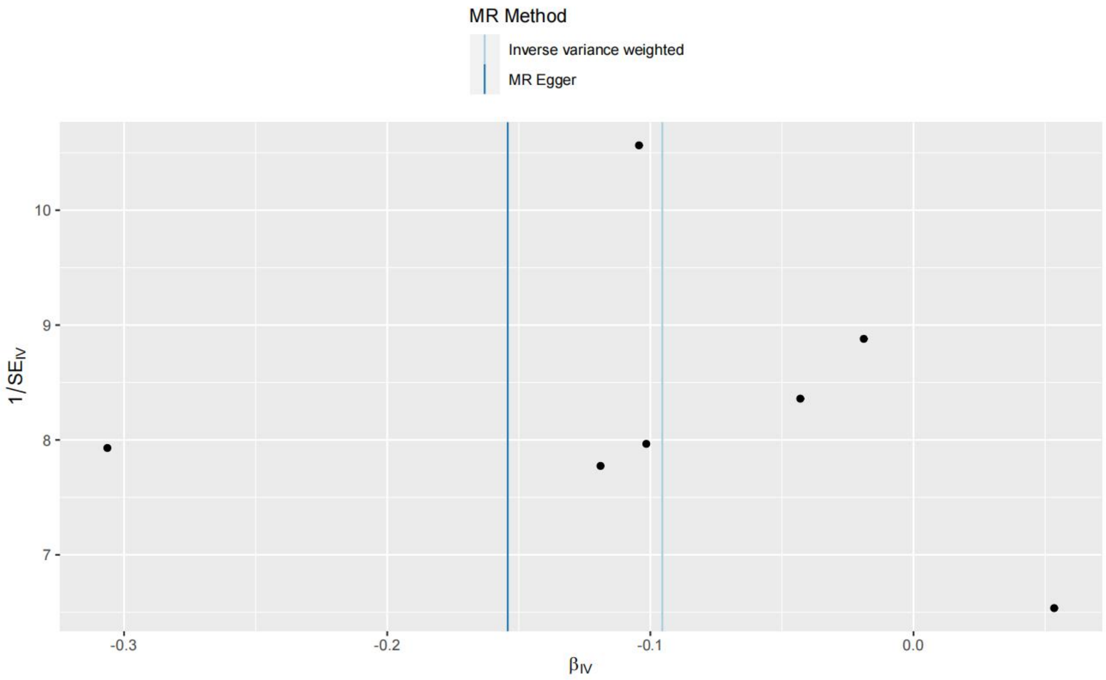

B. Funnel plot of IL-5 instruments strength on AD

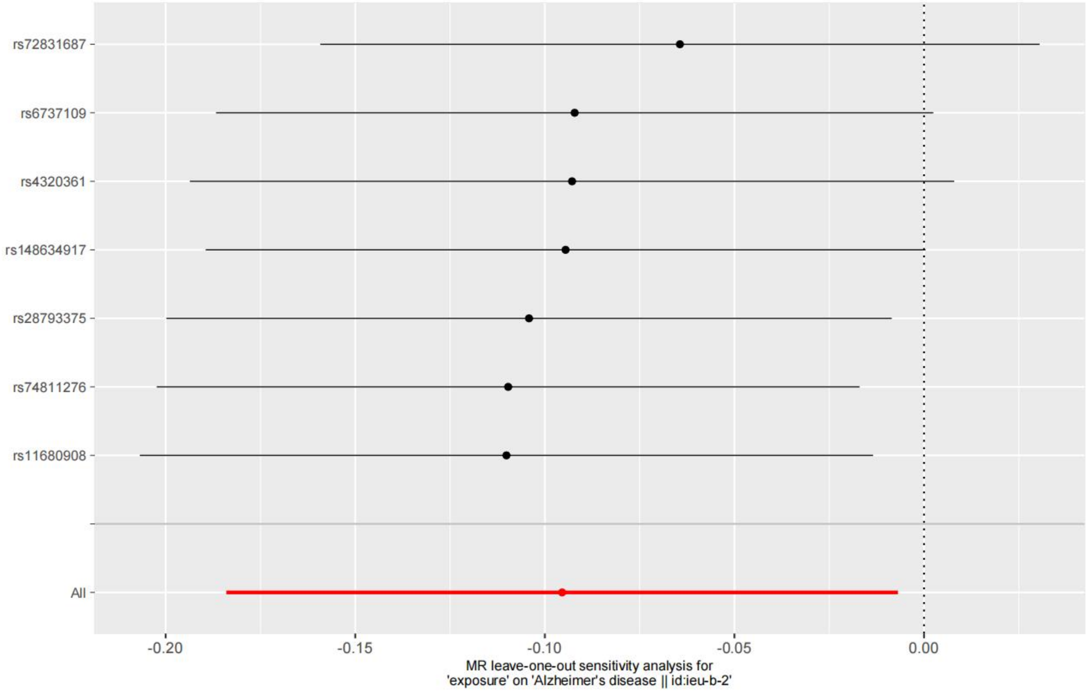

C. MR leave-one-out sensitivity analysis for IL-5 on AD

eFigure 15. IL-6-associated SNPs with risk of AD

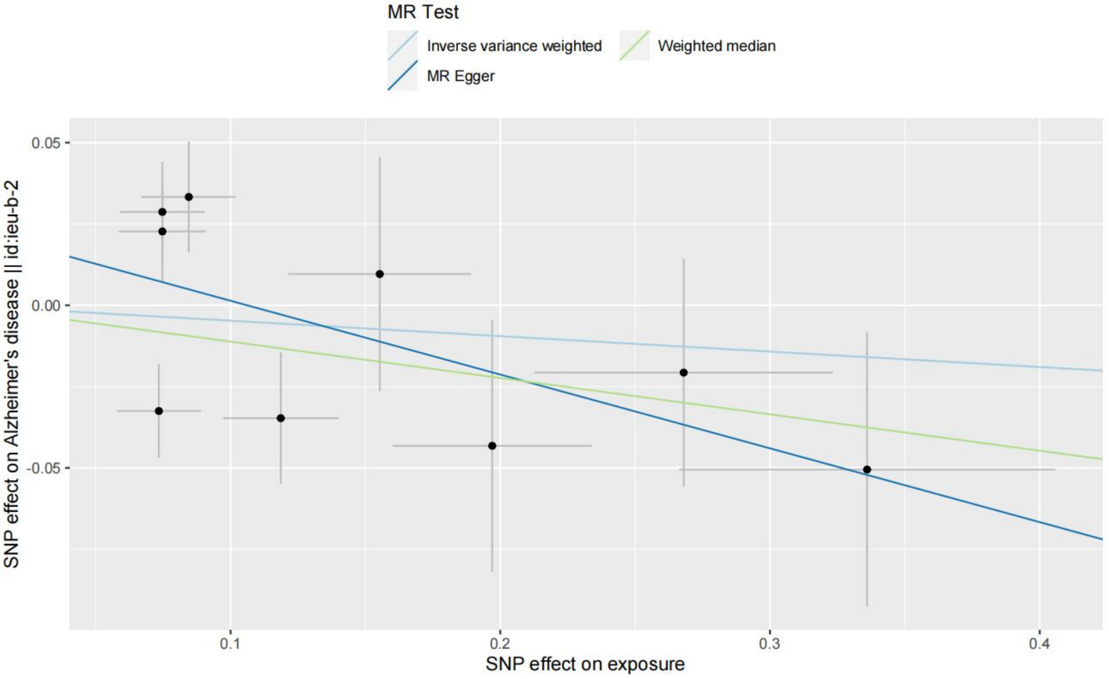

A. Scatter plot of IL-6 with risk of AD

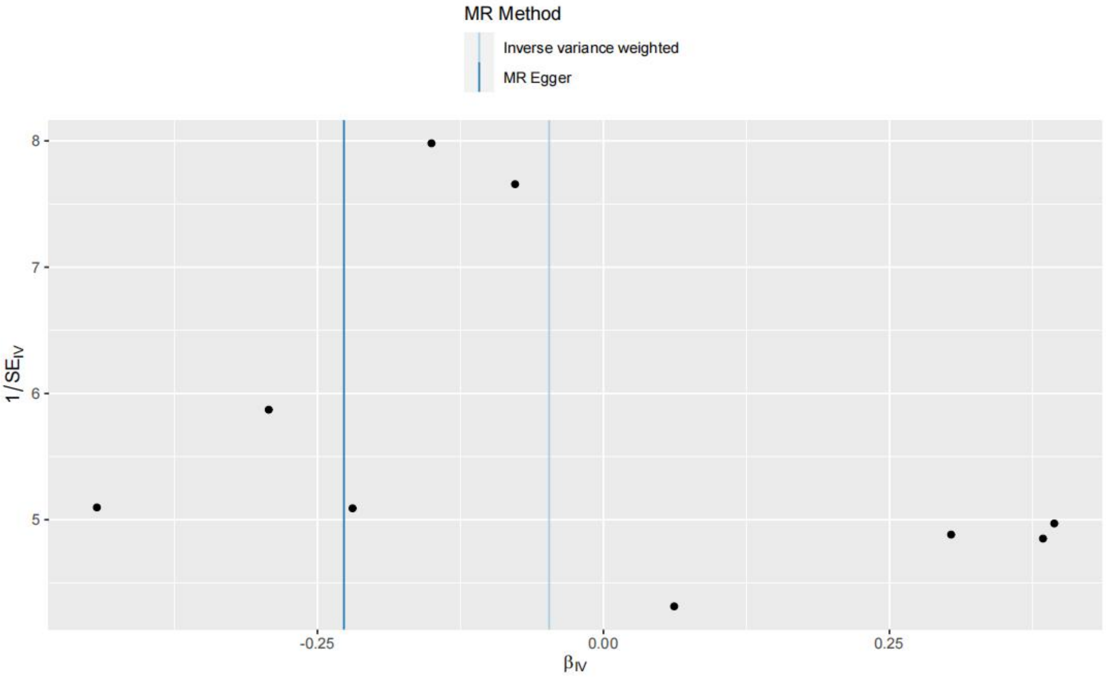

B. Funnel plot of IL-6 instruments strength on AD

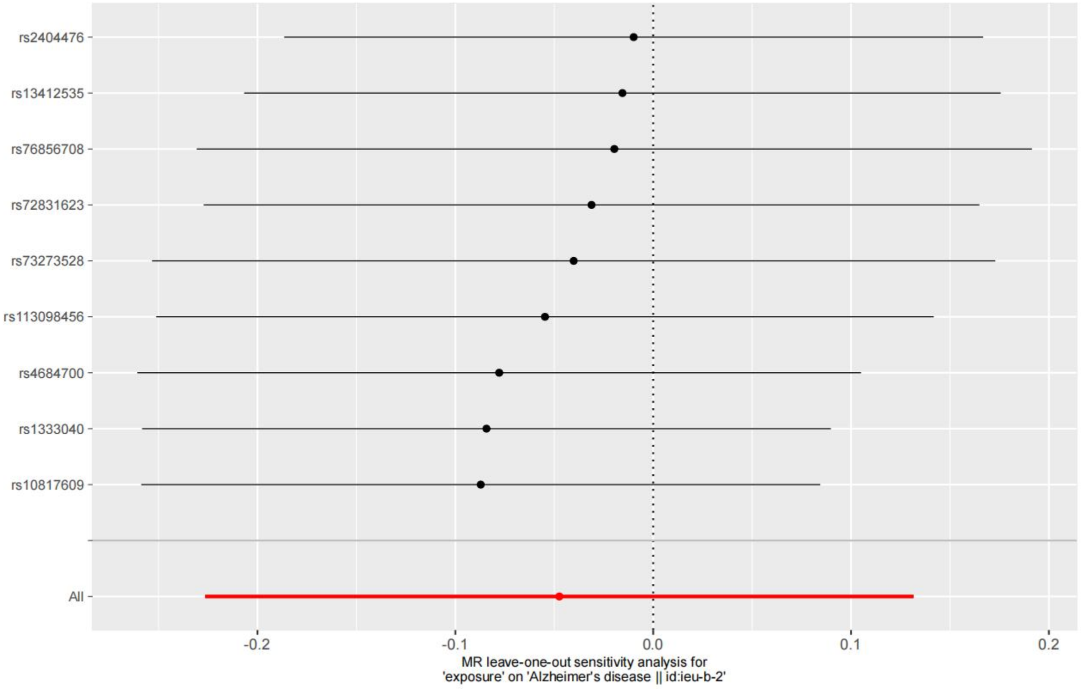

C. MR leave-one-out sensitivity analysis for IL-6 on AD

eFigure 16. IL-7-associated SNPs with risk of AD

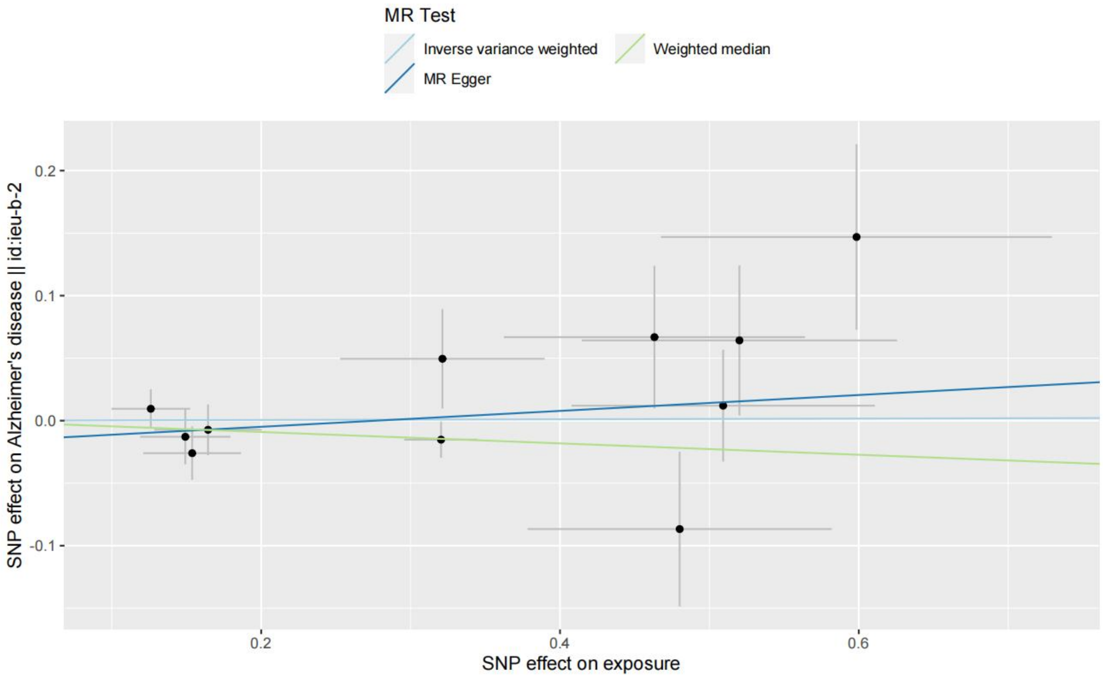

A. Scatter plot of IL-7 with risk of AD

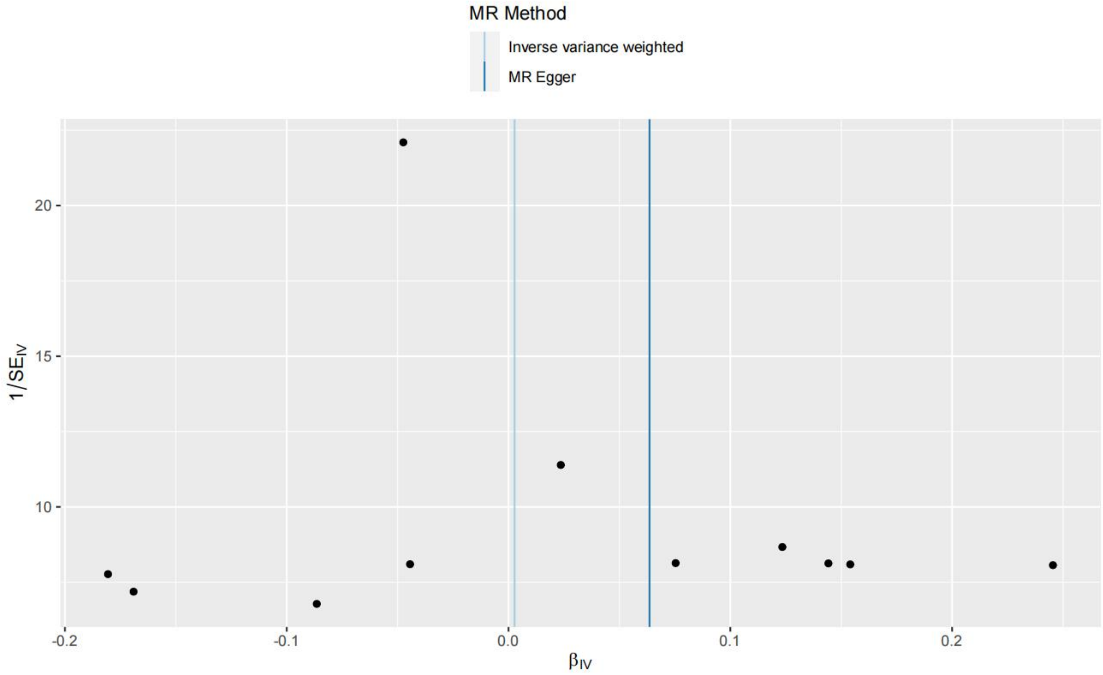

B. Funnel plot of IL-7 instruments strength on AD

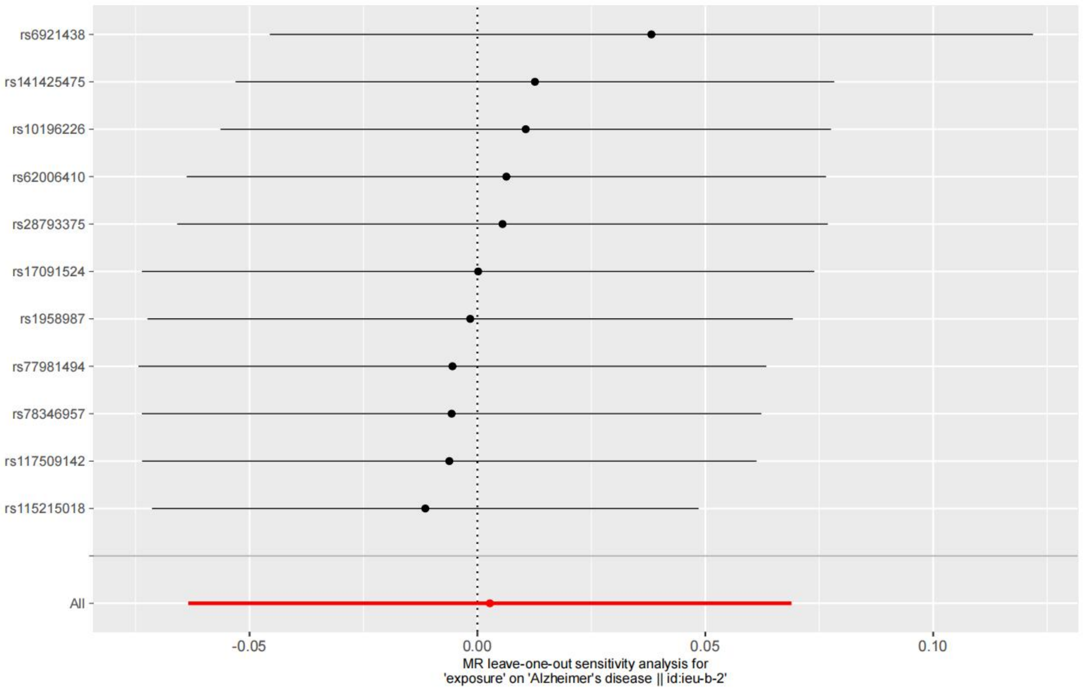

C. MR leave-one-out sensitivity analysis for IL-7 on AD

eFigure 17. IL-8-associated SNPs with risk of AD

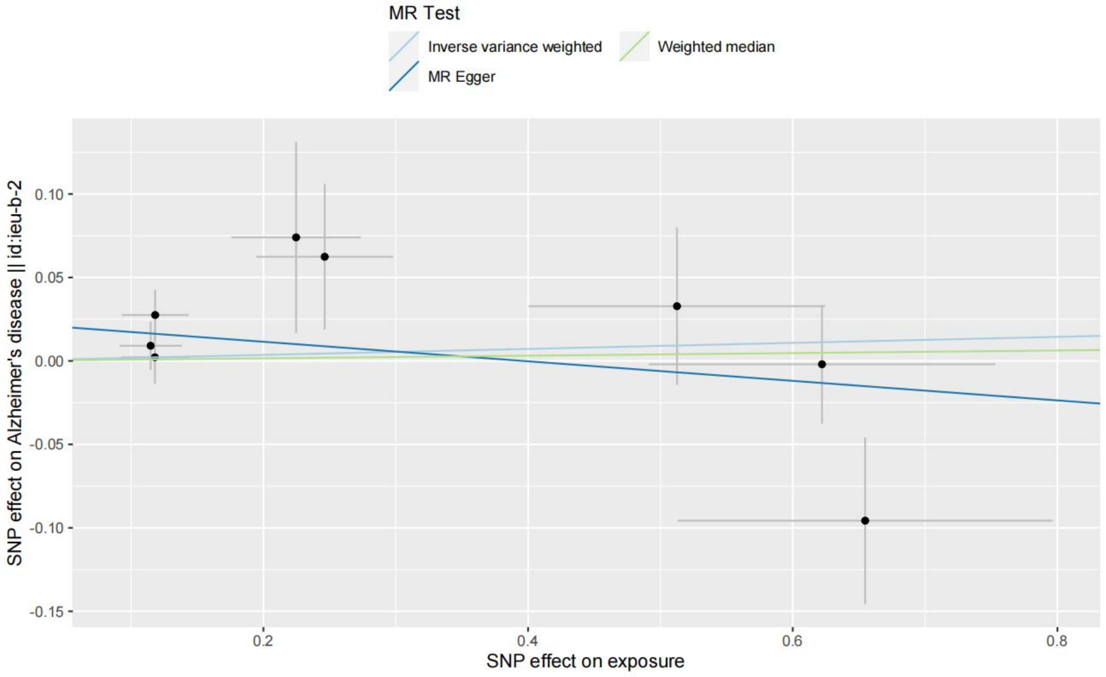

A. Scatter plot of IL-8 with risk of AD

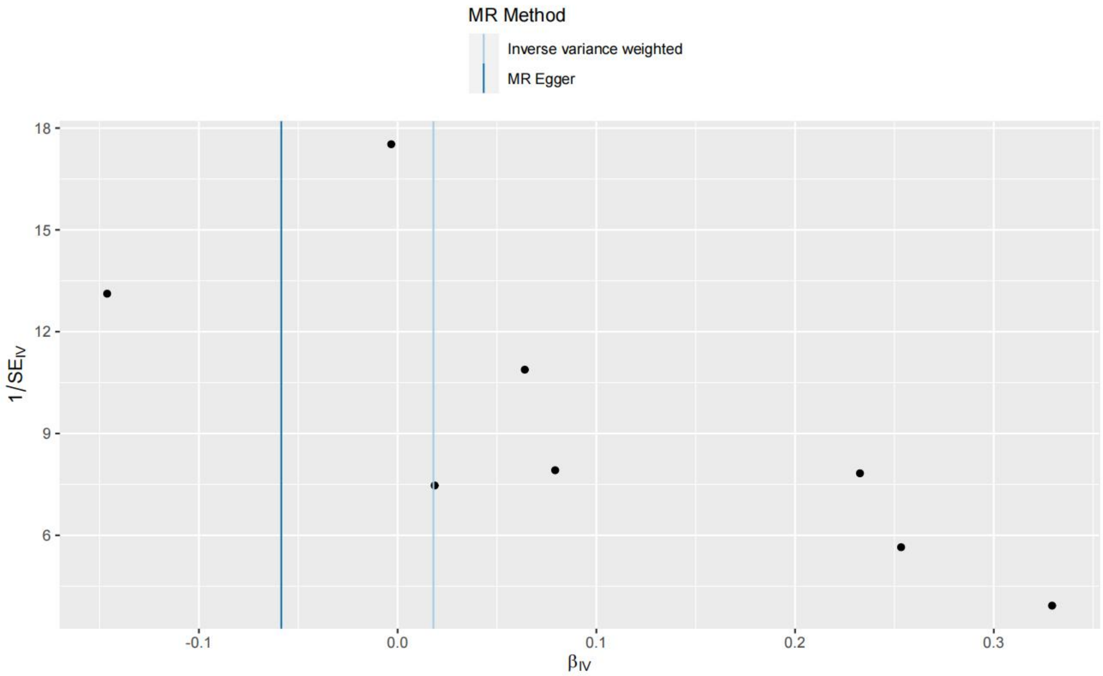

B. Funnel plot of IL-8 instruments strength on AD

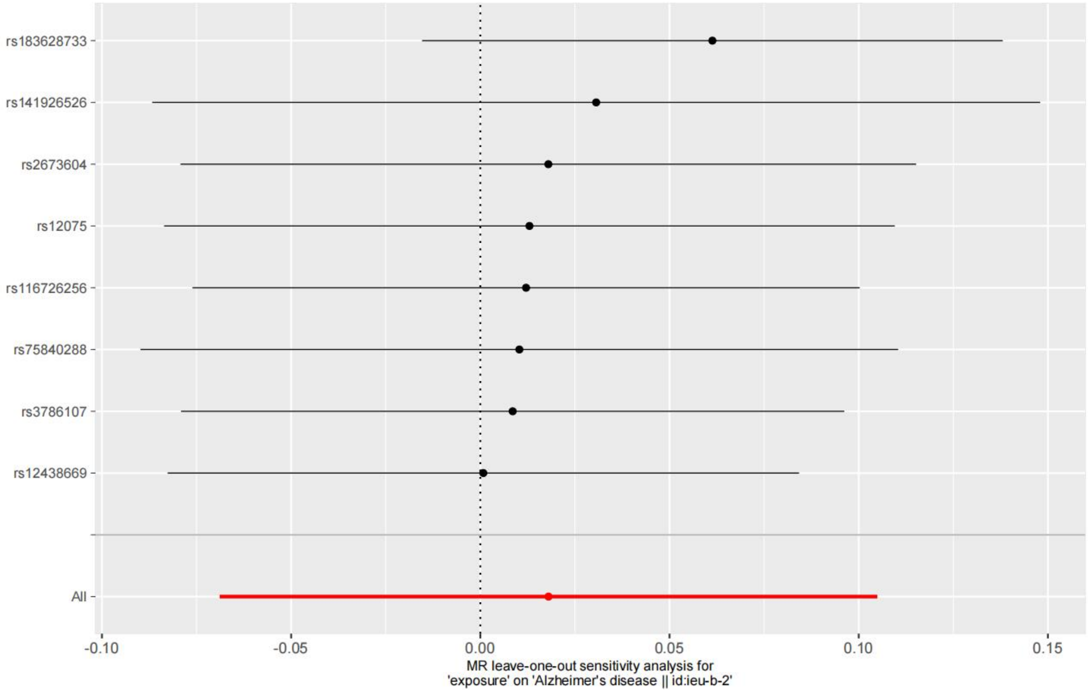

C. MR leave-one-out sensitivity analysis for IL-8 on AD

eFigure 18. IL-9-associated SNPs with risk of AD

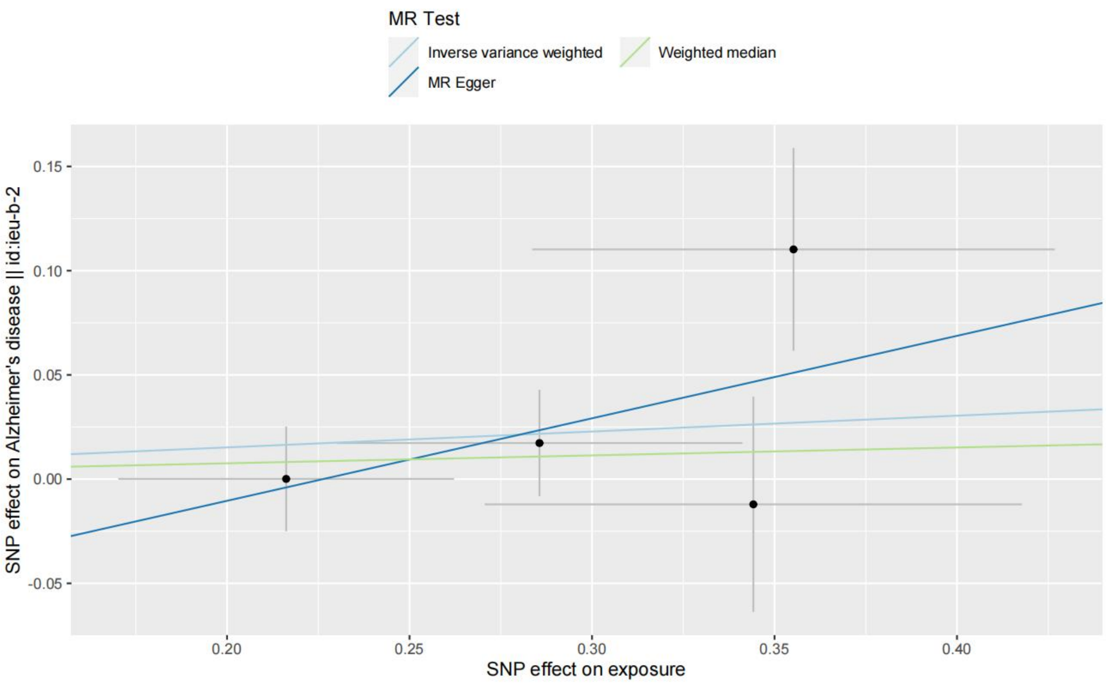

A. Scatter plot of IL-9 with risk of AD

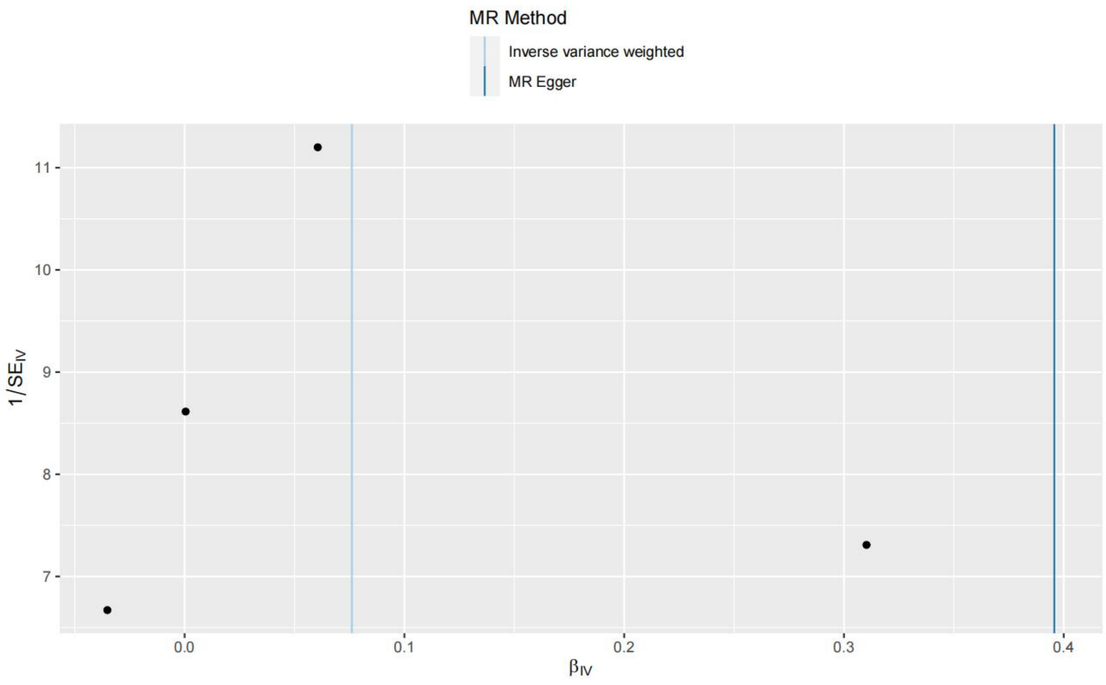

B. Funnel plot of IL-9 instruments strength on AD

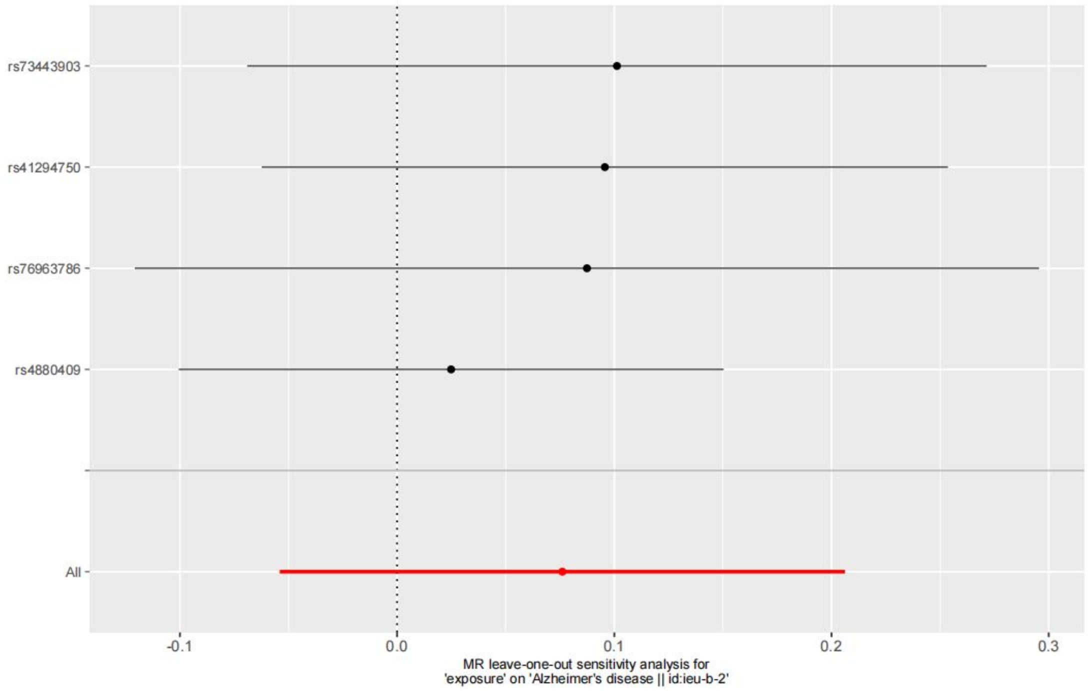

C. MR leave-one-out sensitivity analysis for IL-9 on AD

eFigure 19. IL-10-associated SNPs with risk of AD

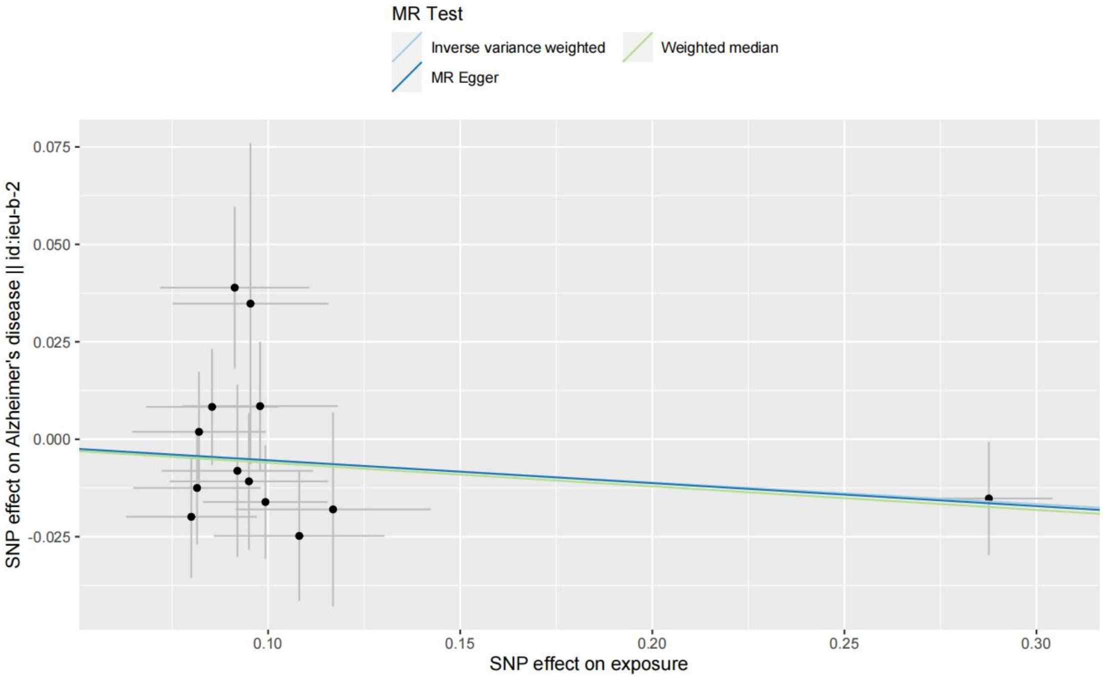

A. Scatter plot of IL-10 with risk of AD

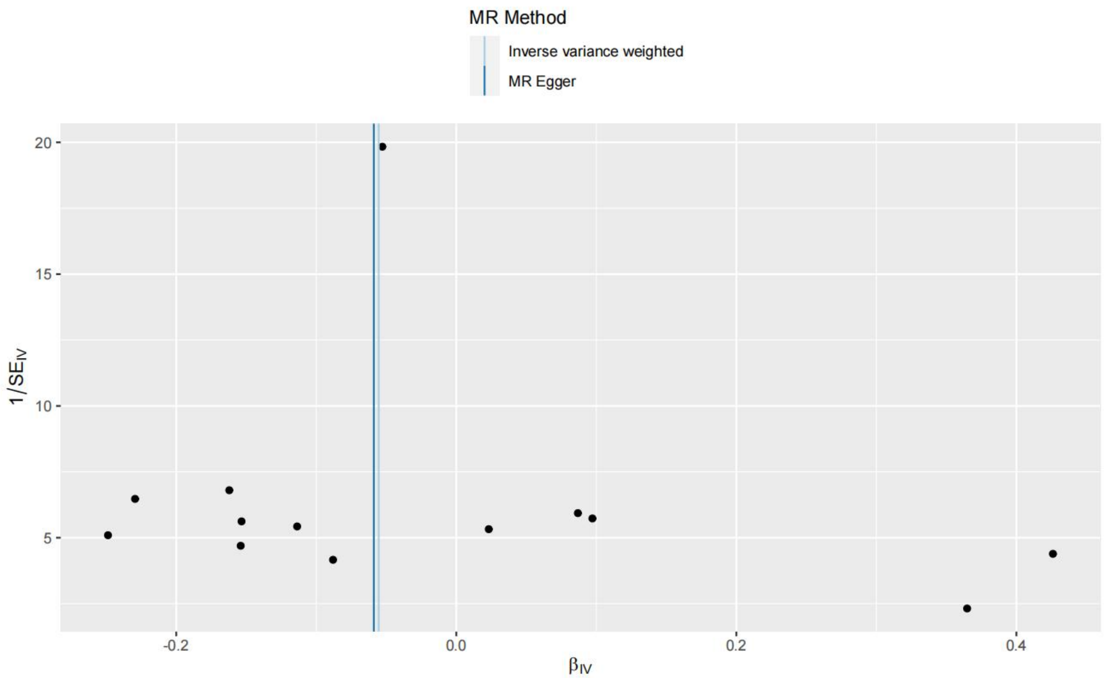

B. Funnel plot of IL-10 instruments strength on AD

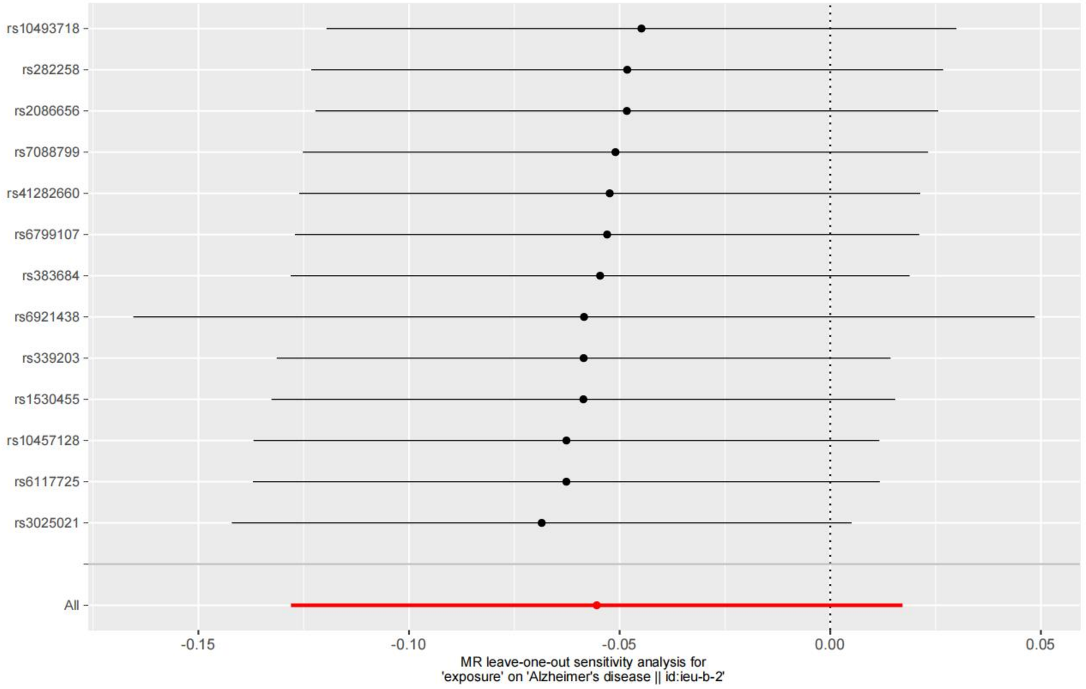

C. MR leave-one-out sensitivity analysis for IL-10 on AD

eFigure 20. IL-12-associated SNPs with risk of AD

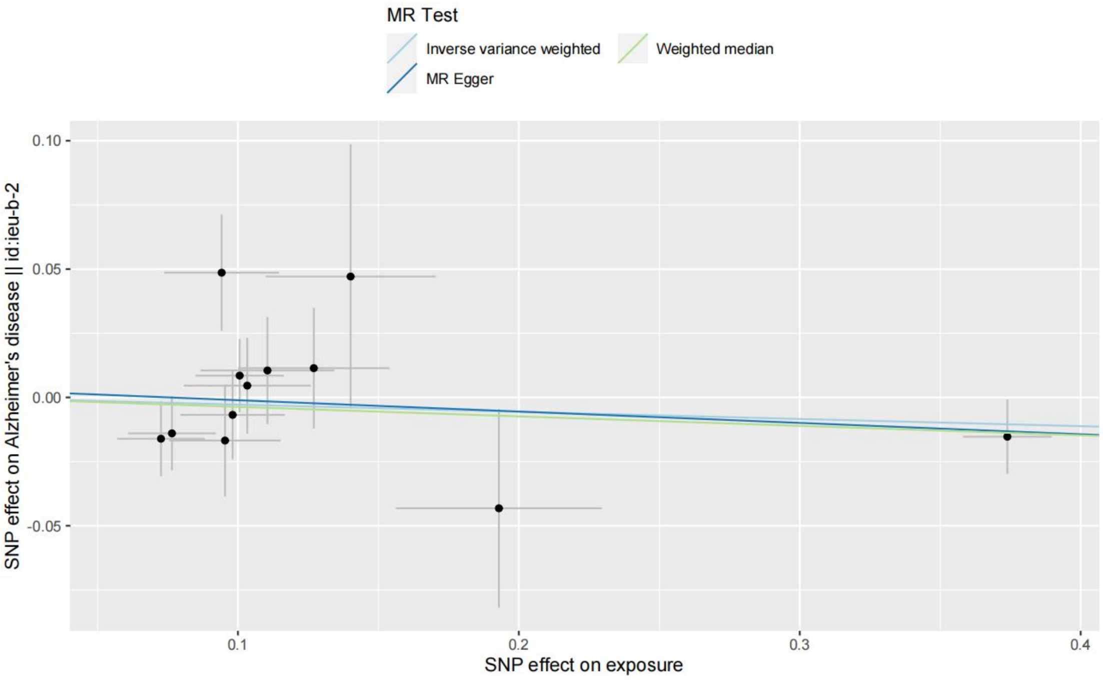

A. Scatter plot of IL-12 with risk of AD

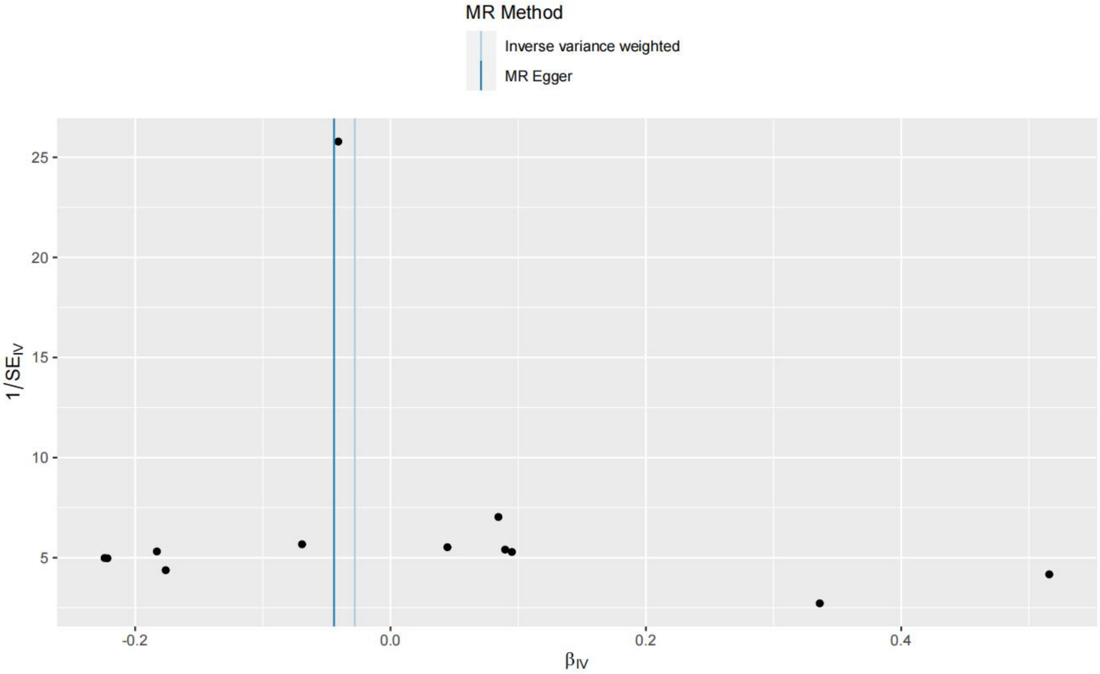

B. Funnel plot of IL-12 instruments strength on AD

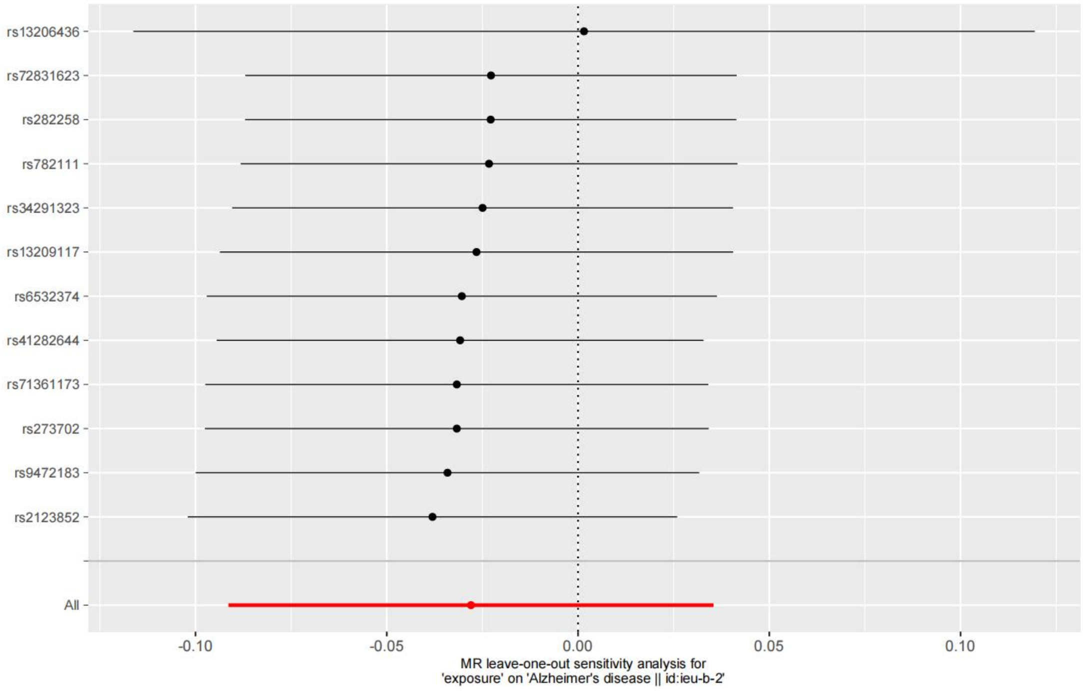

C. MR leave-one-out sensitivity analysis for IL-12 on AD

eFigure 21. IL-13-associated SNPs with risk of AD

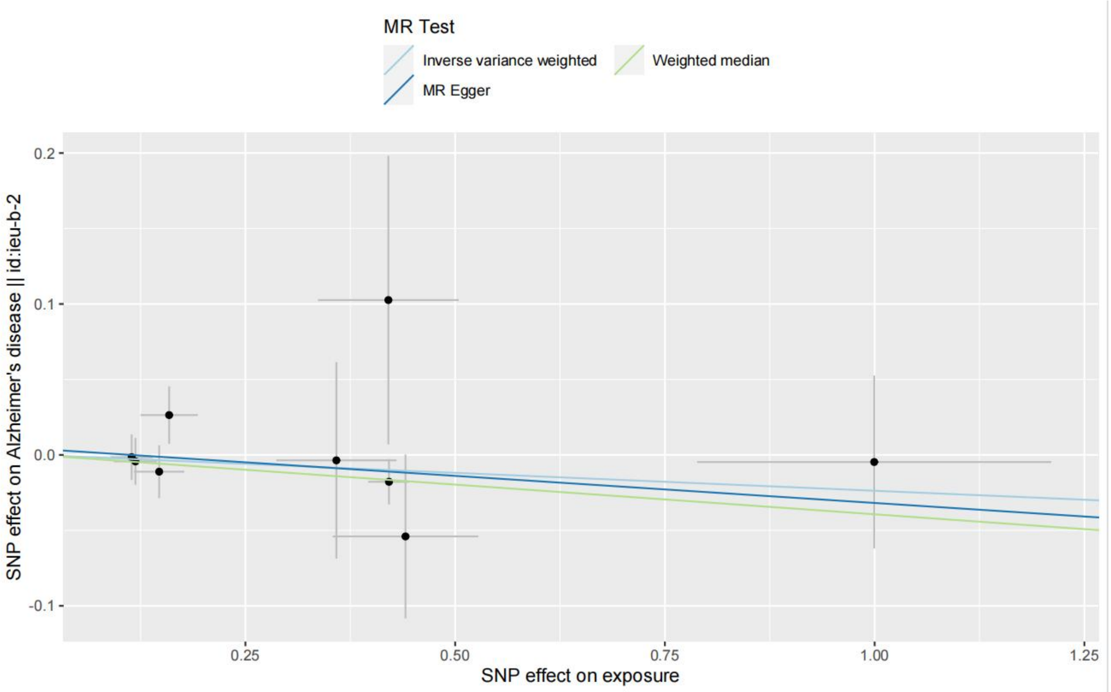

A. Scatter plot of IL-13 with risk of AD

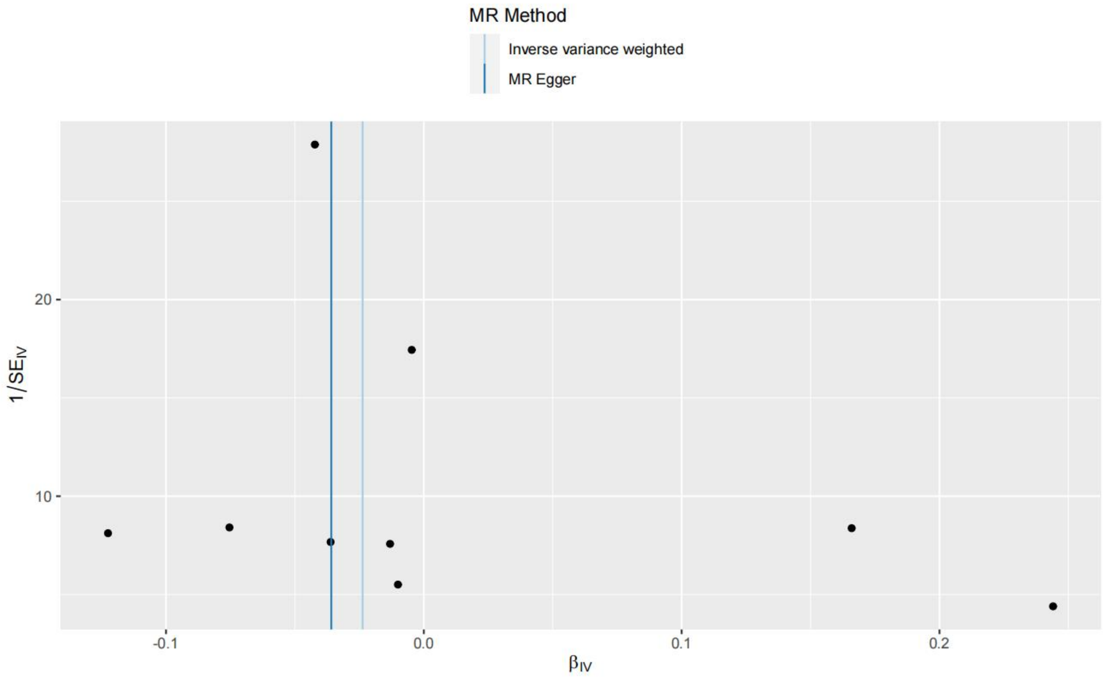

B. Funnel plot of IL-13 instruments strength on AD

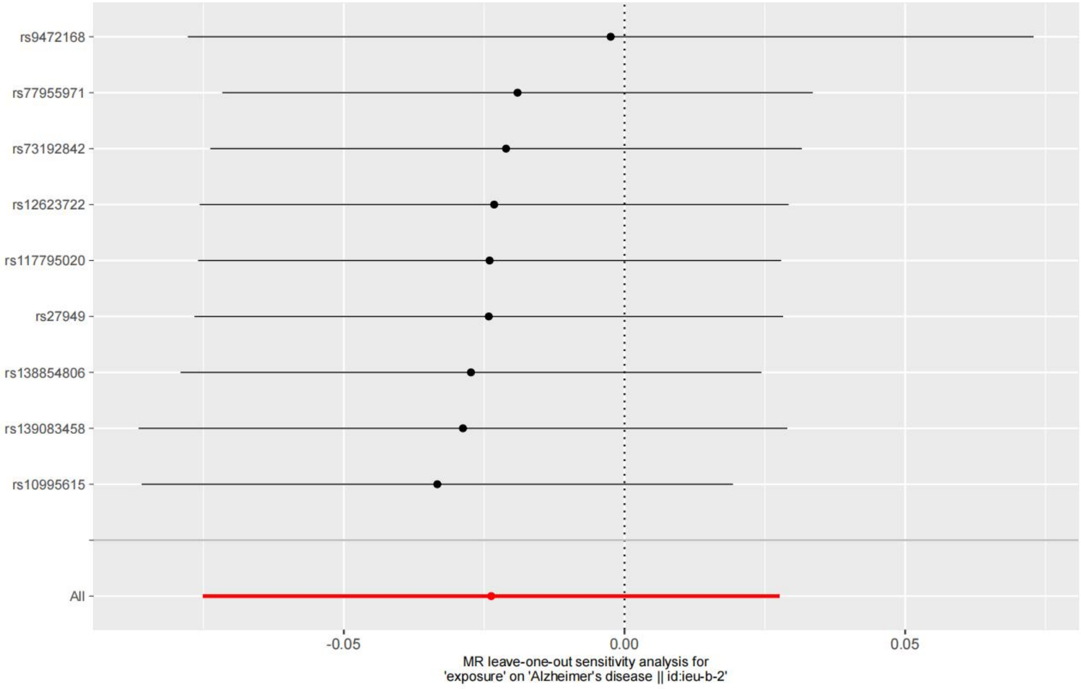

C. MR leave-one-out sensitivity analysis for IL-13 on AD

eFigure 22. IL-16-associated SNPs with risk of AD

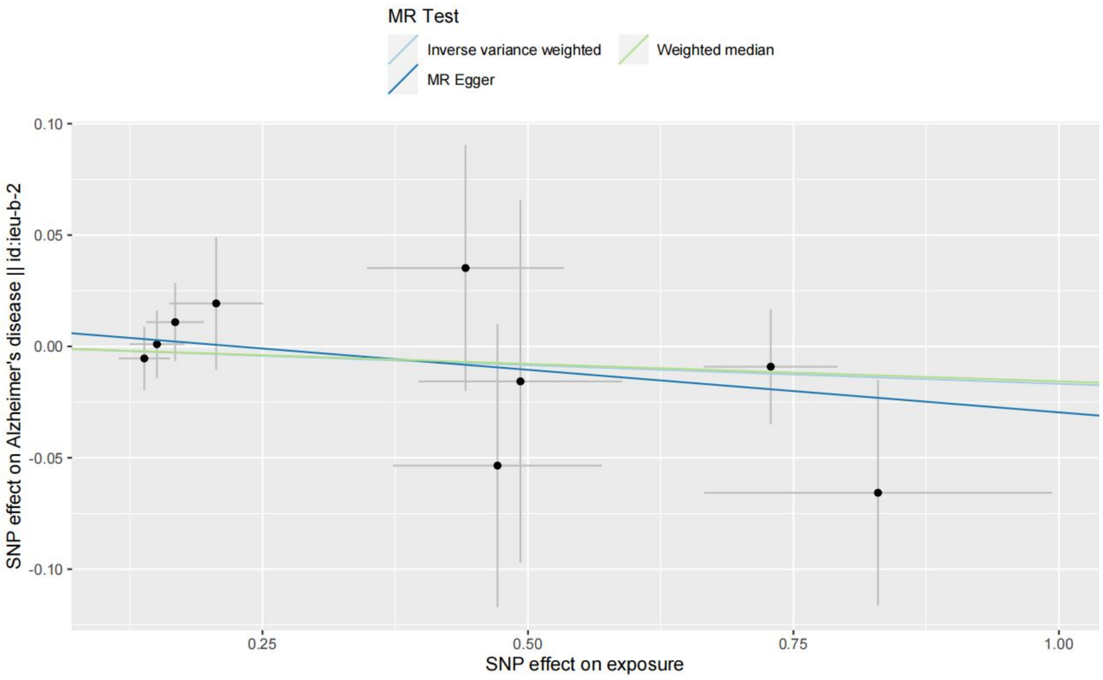

A. Scatter plot of IL-16 with risk of AD

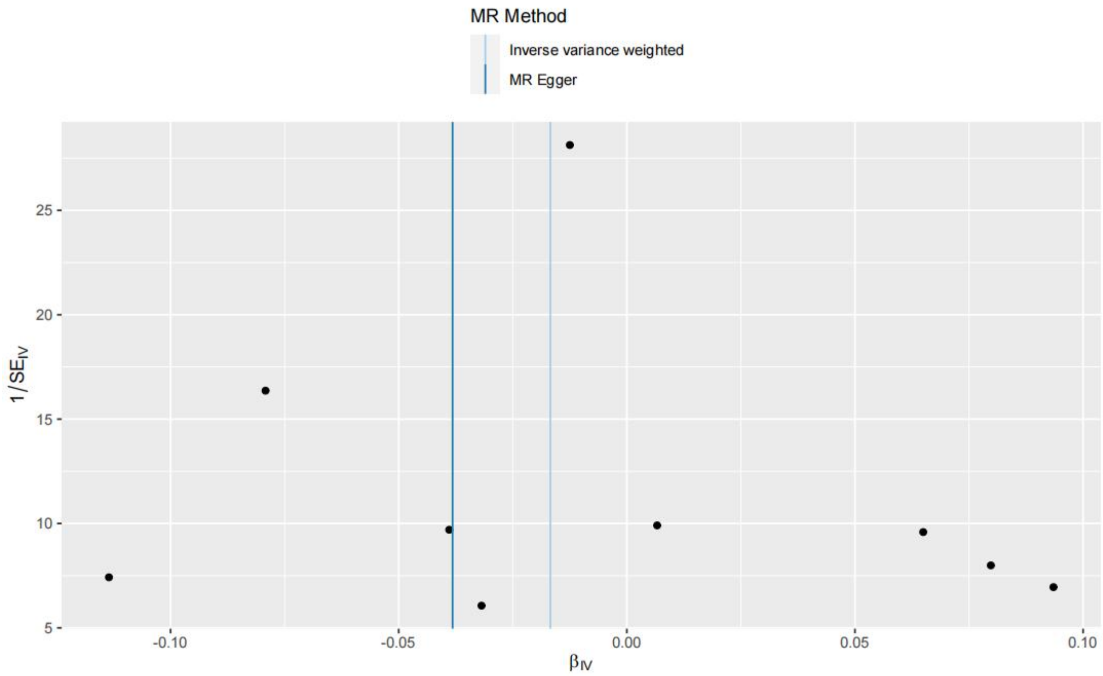

B. Funnel plot of IL-16 instruments strength on AD

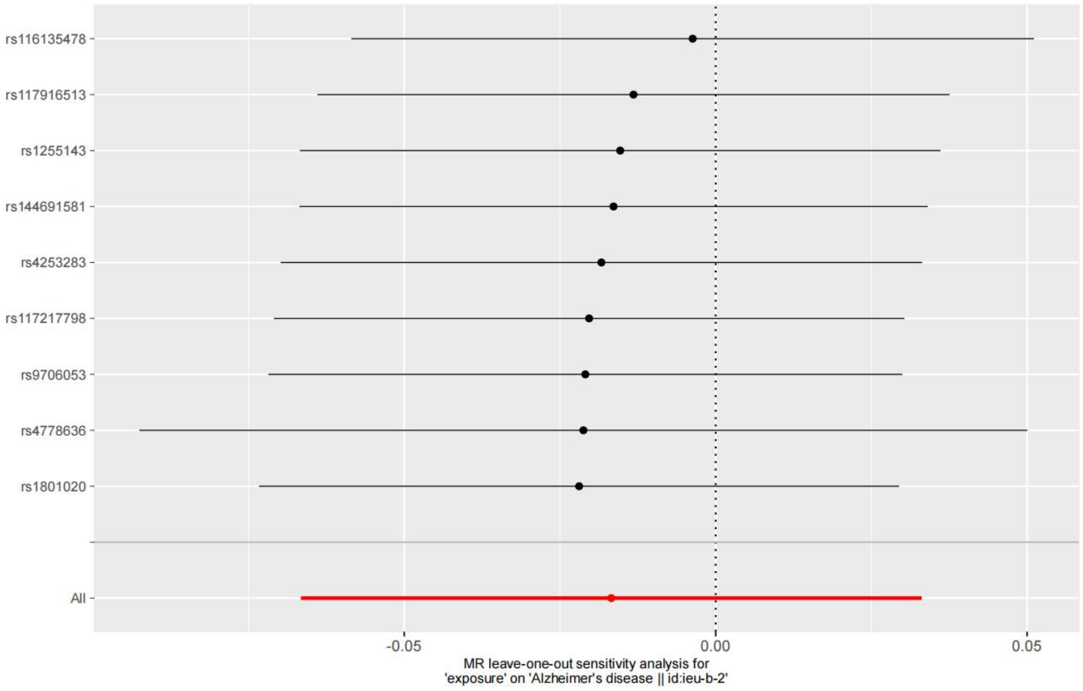

C. MR leave-one-out sensitivity analysis for IL-16 on AD

eFigure 23. IL-17-associated SNPs with risk of AD

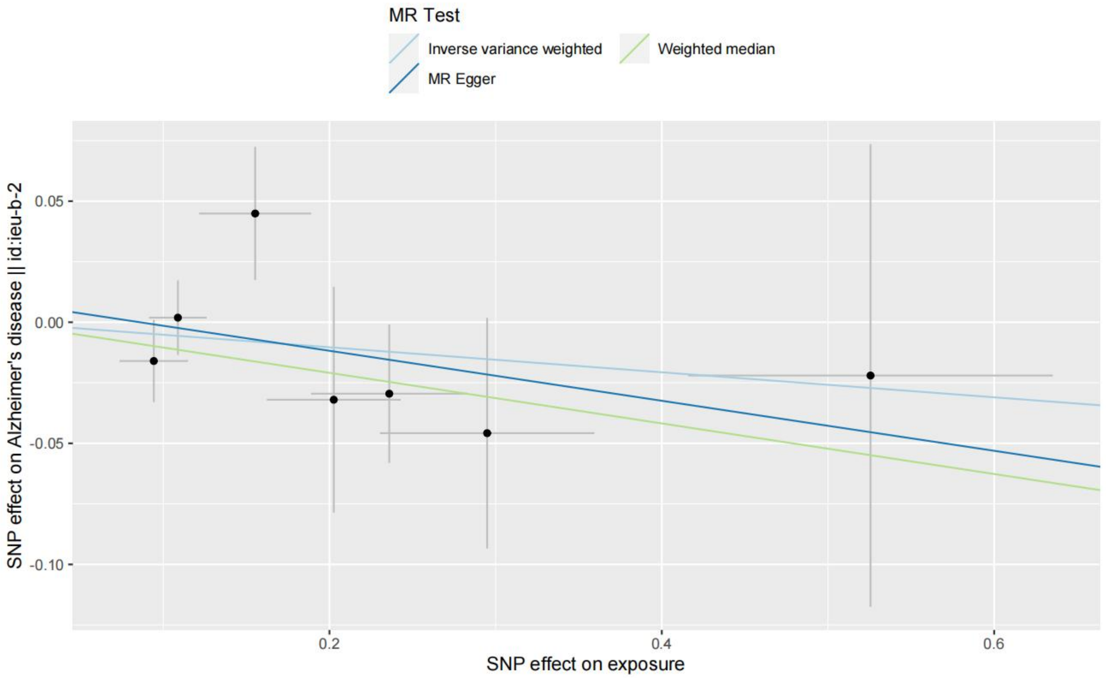

A. Scatter plot of IL-17 with risk of AD

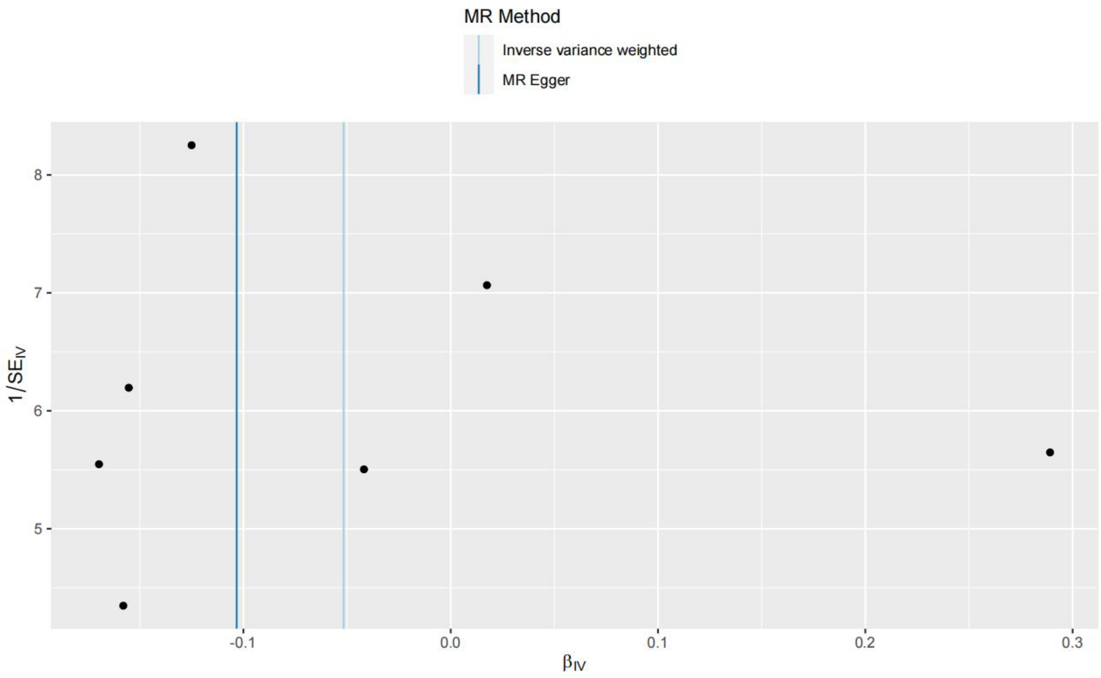

B. Funnel plot of IL-17 instruments strength on AD

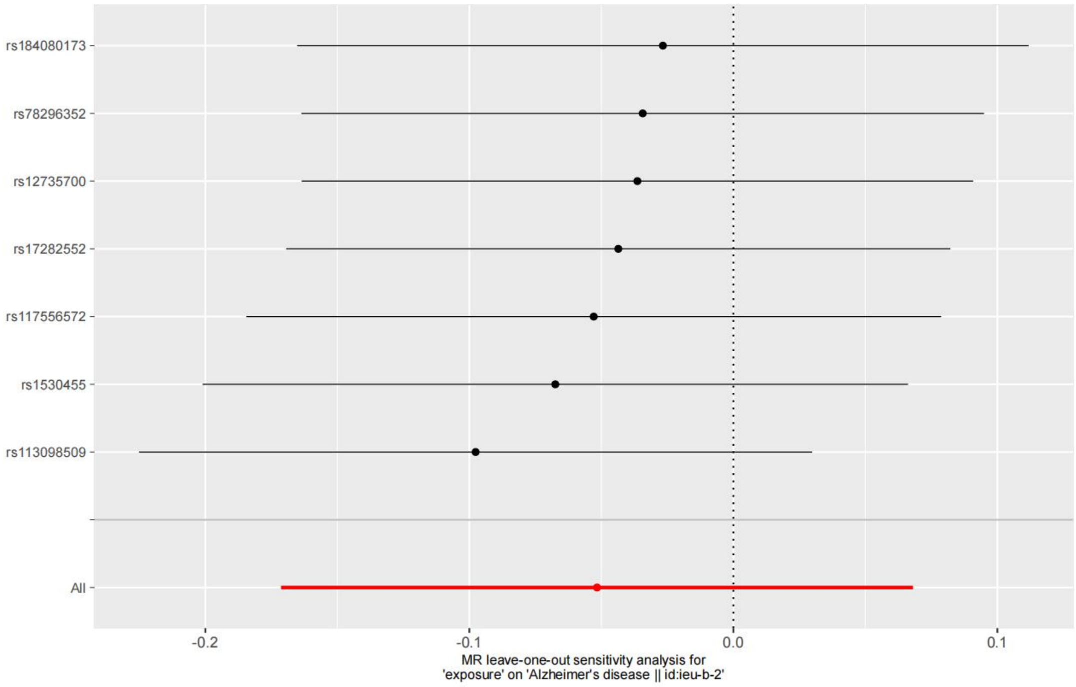

C. MR leave-one-out sensitivity analysis for IL-17 on AD

eFigure 24. IL-18-associated SNPs with risk of AD

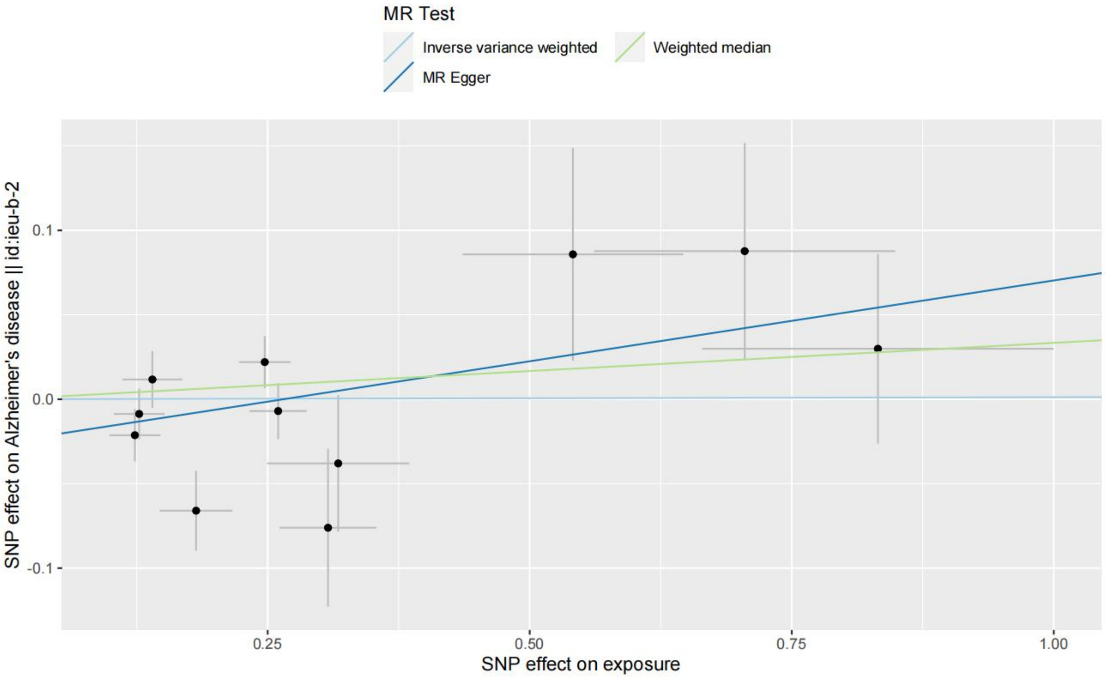

A. Scatter plot of IL-18 with risk of AD

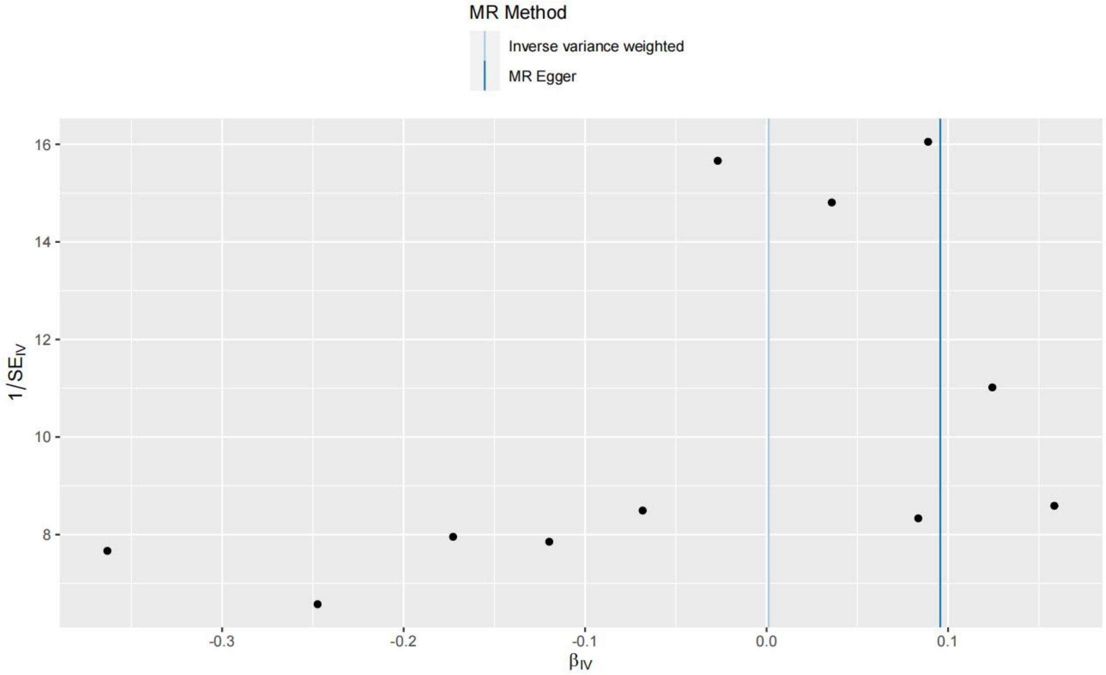

B. Funnel plot of IL-18 instruments strength on AD

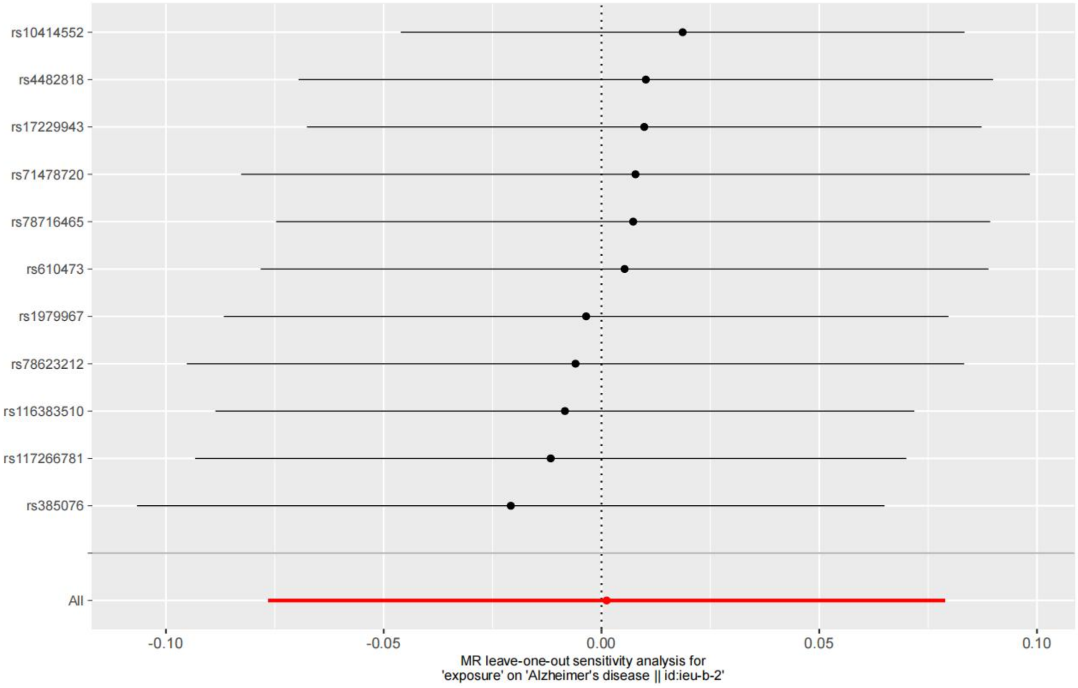

C. MR leave-one-out sensitivity analysis for IL-18 on AD

eFigure 25. IP-10-associated SNPs with risk of AD

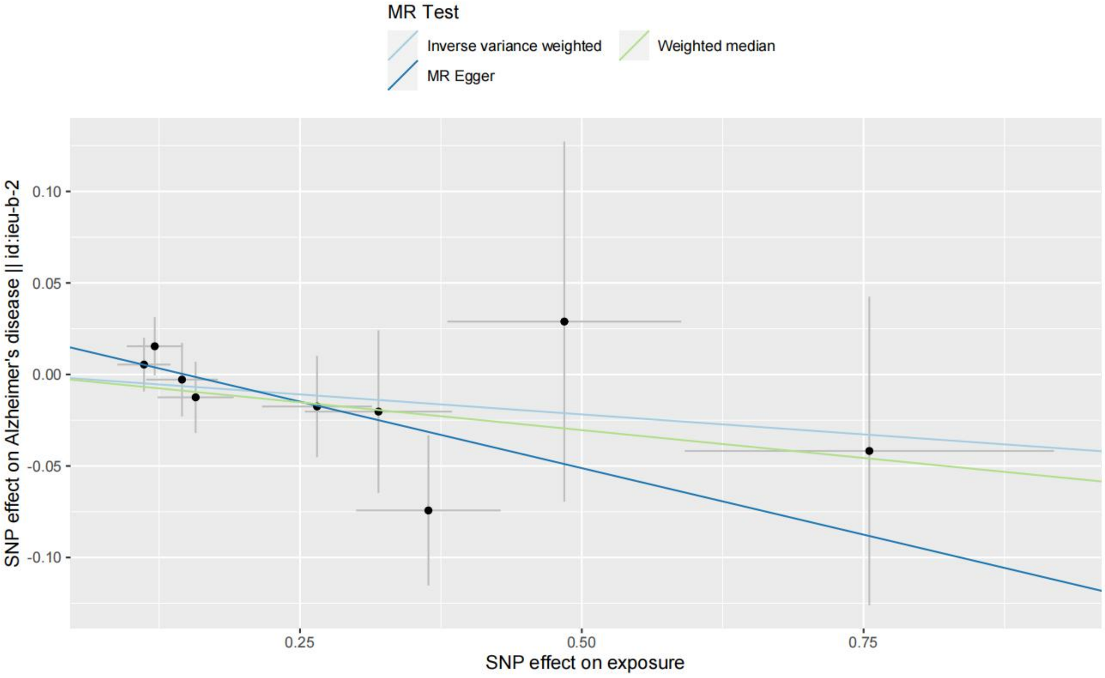

A. Scatter plot of IP-10 with risk of AD

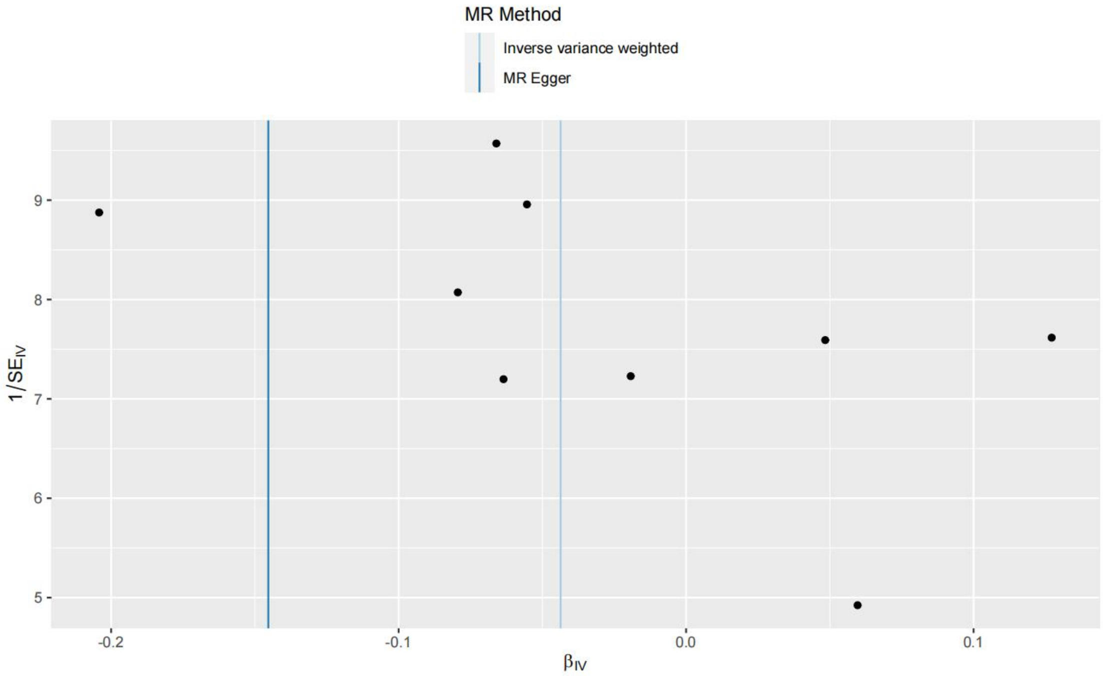

B. Funnel plot of IP-10 instruments strength on AD

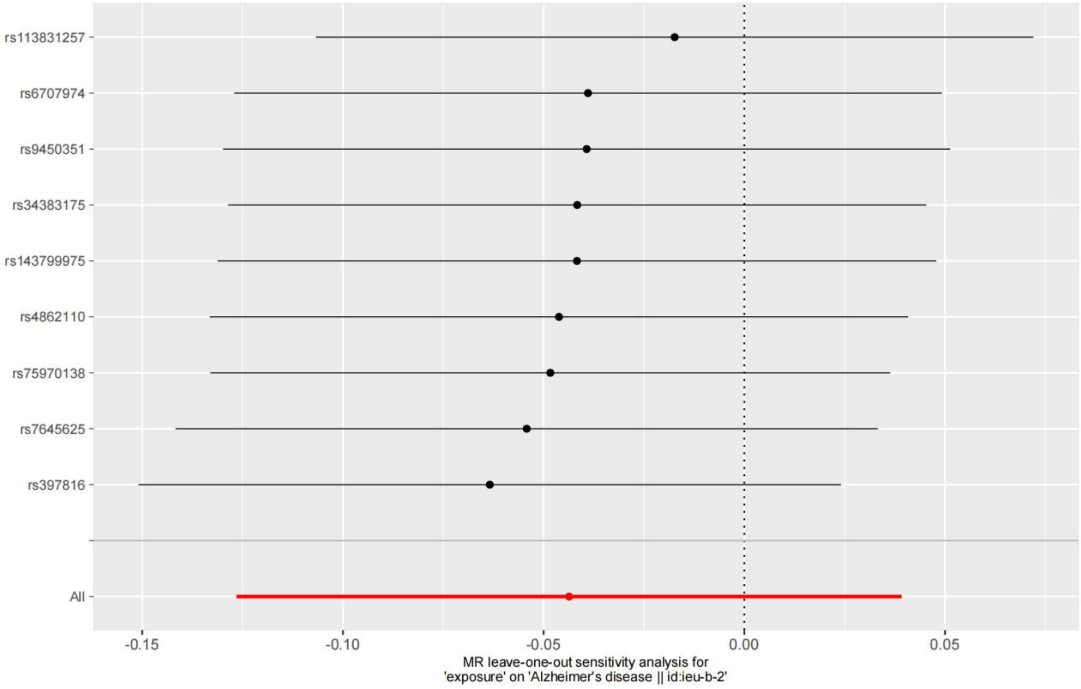

C. MR leave-one-out sensitivity analysis for IP-10 on AD

eFigure 26. M-CSF-associated SNPs with risk of AD

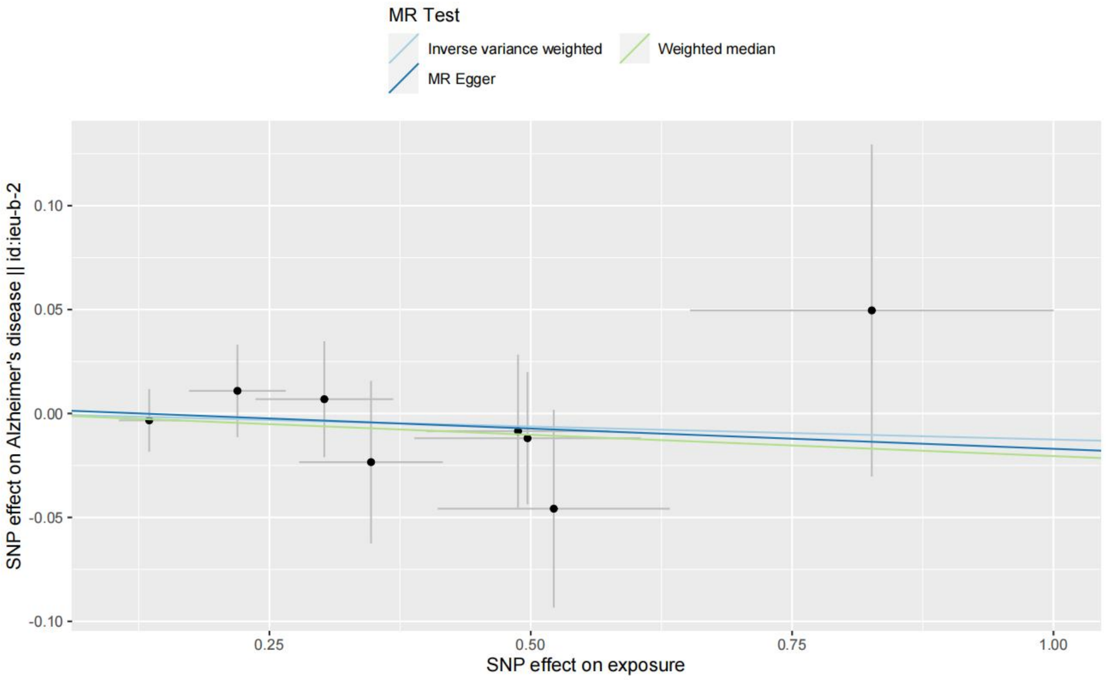

A. Scatter plot of M-CSF with risk of AD

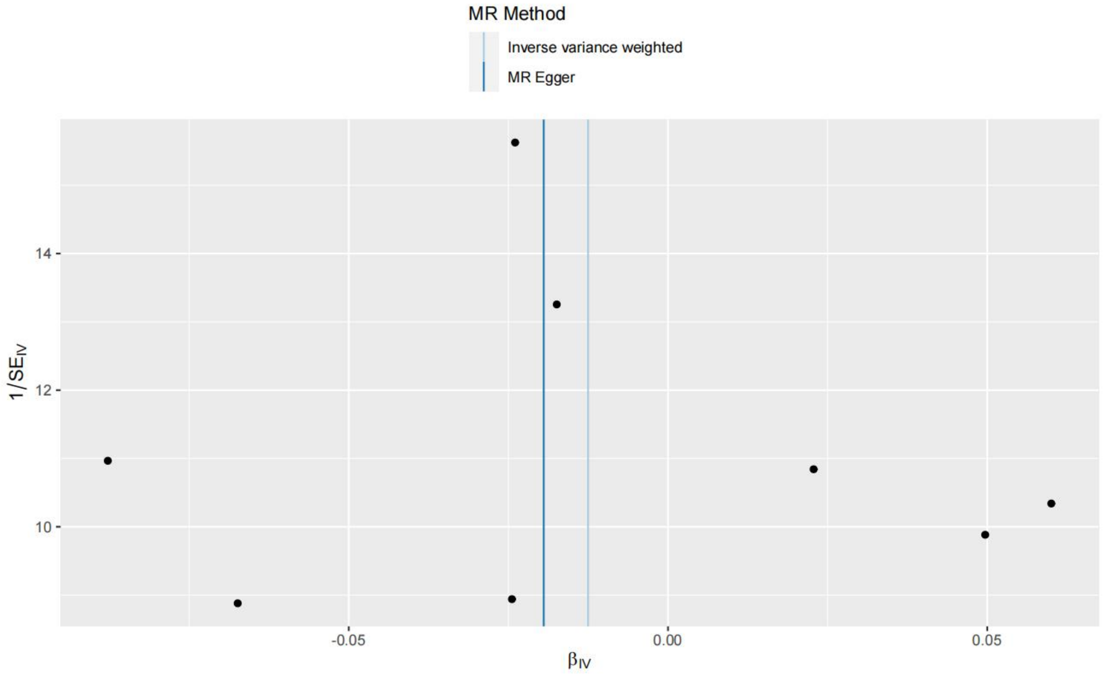

B. Funnel plot of M-CSF instruments strength on AD

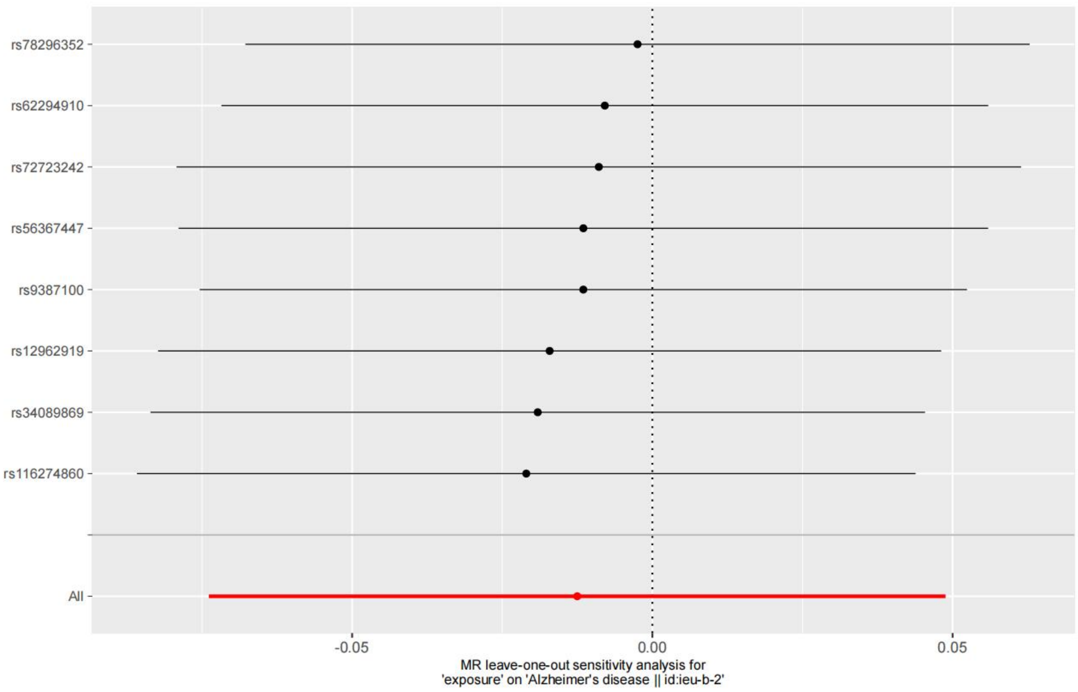

C. MR leave-one-out sensitivity analysis for M-CSF on AD

eFigure 27. MCP-1-MCAF-associated SNPs with risk of AD

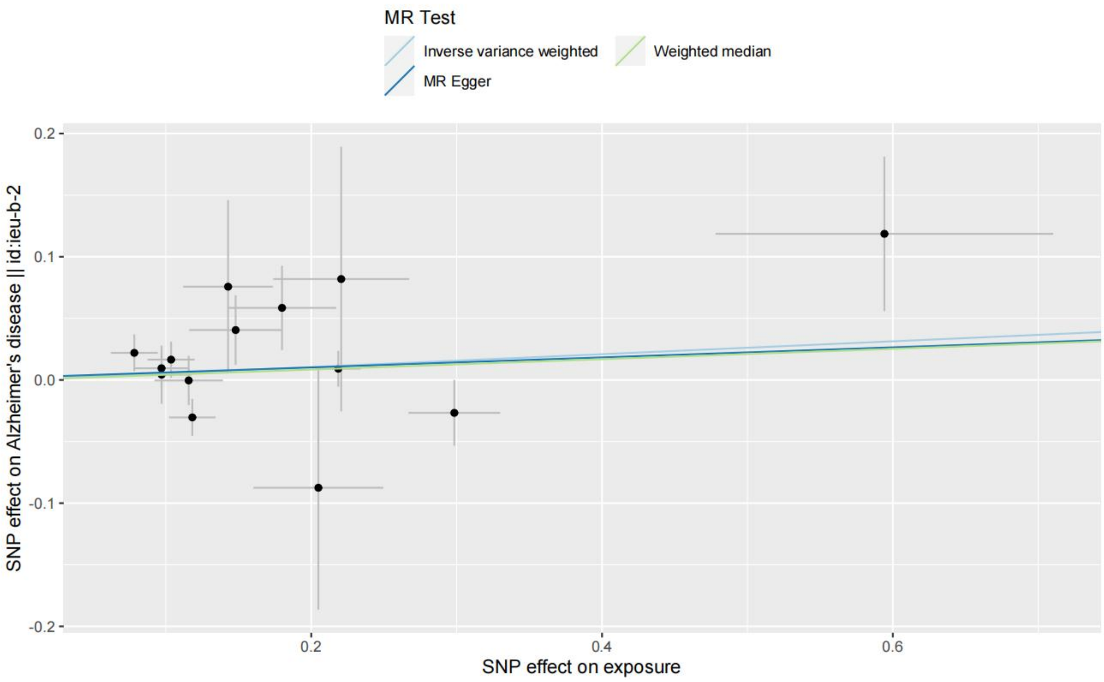

A. Scatter plot of MCP-1-MCAF with risk of AD

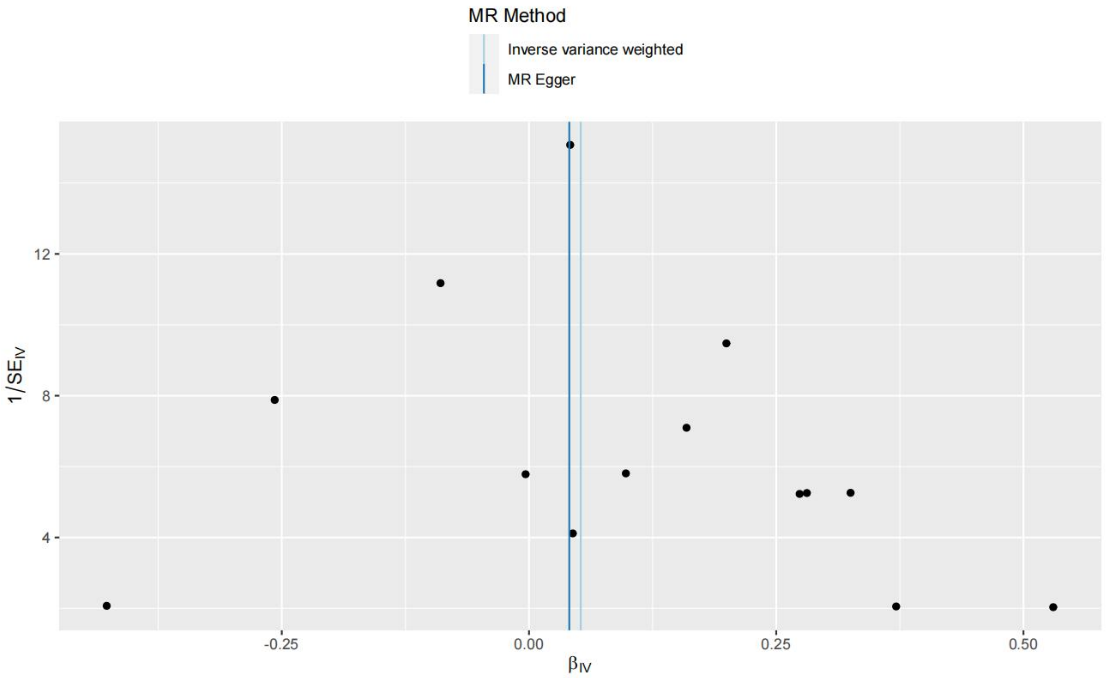

B. Funnel plot of MCP-1-MCAF instruments strength on AD

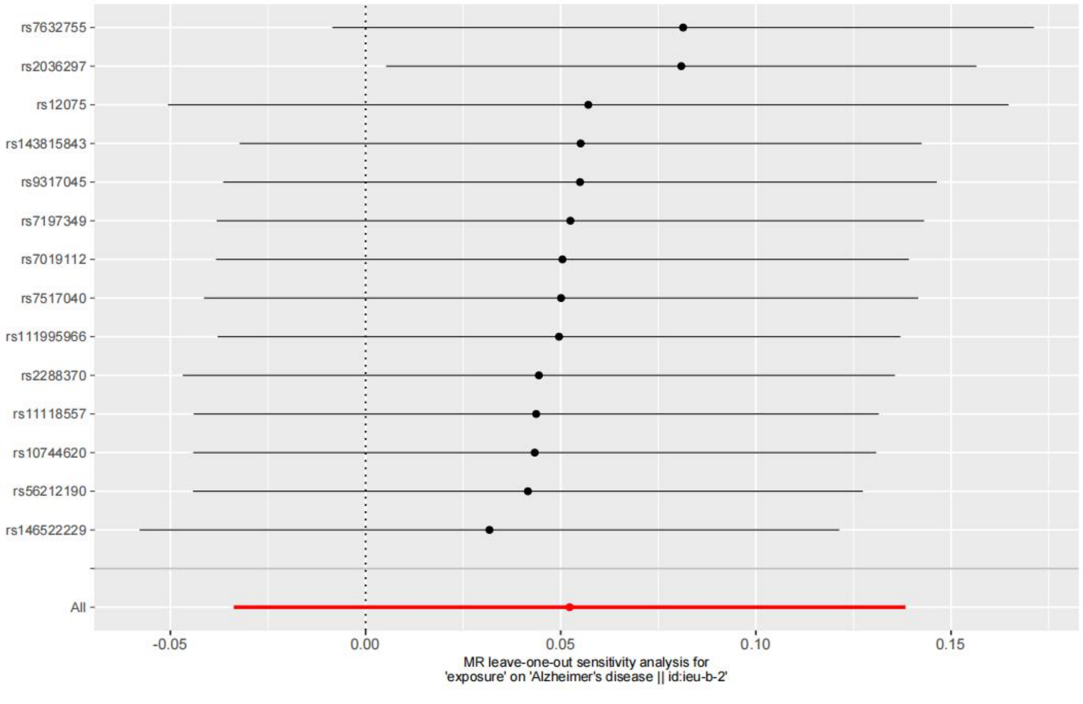

C. MR leave-one-out sensitivity analysis for MCP-1-MCAF on AD

eFigure 28. MCP-3-associated SNPs with risk of AD

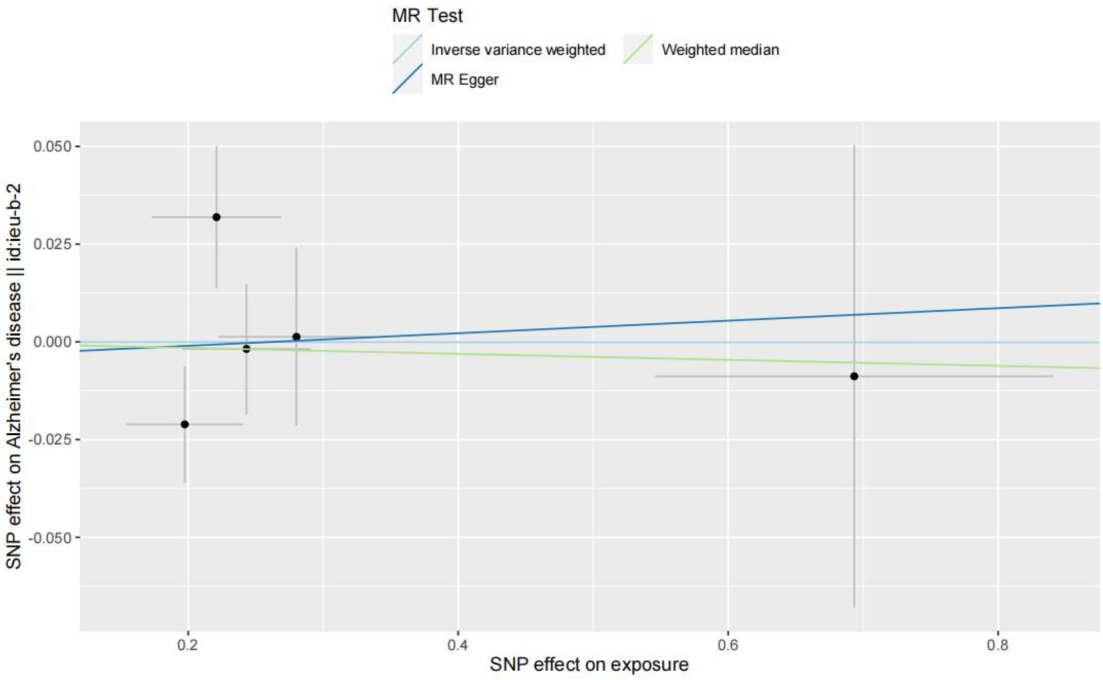

A. Scatter plot of MCP-3 with risk of AD

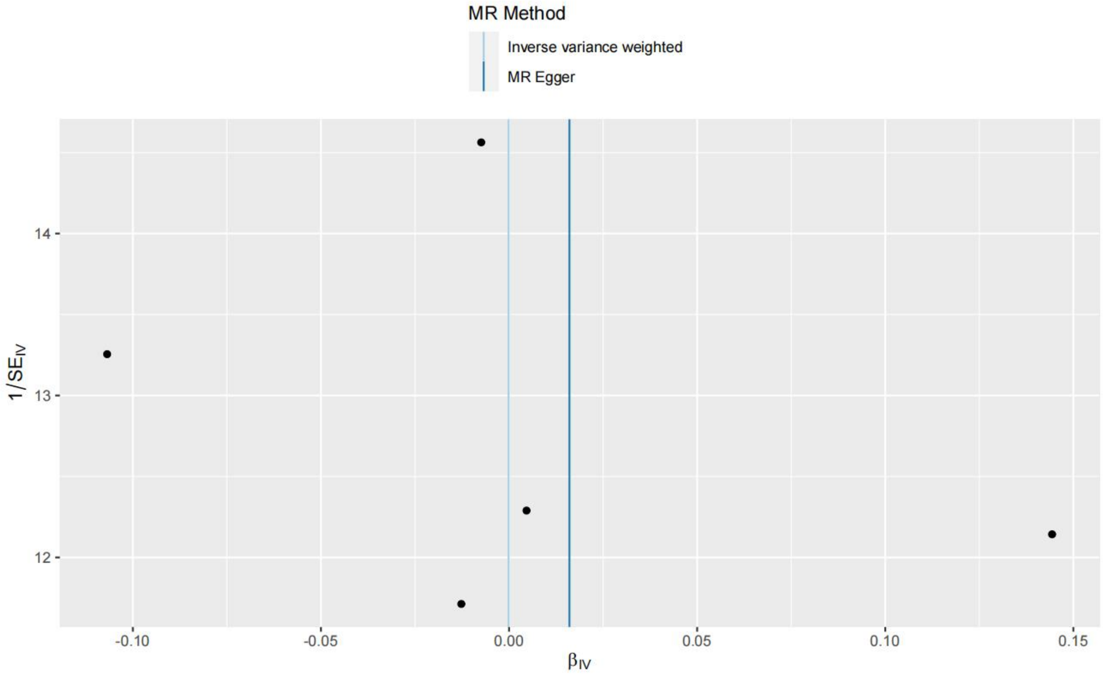

B. Funnel plot of MCP-3 instruments strength on AD

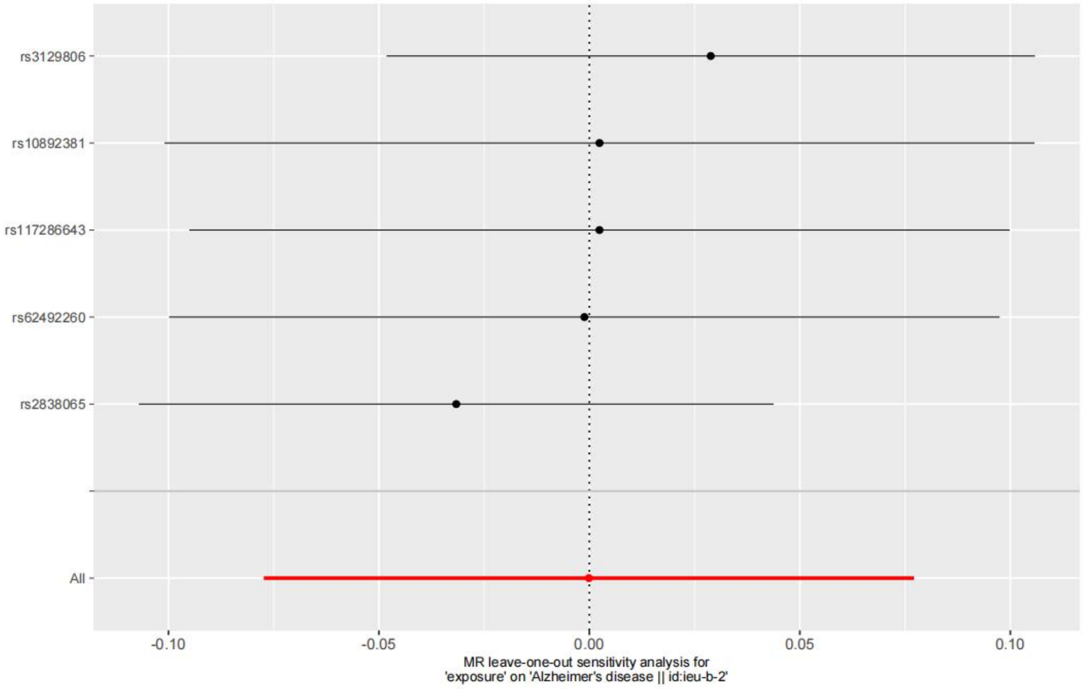

C. MR leave-one-out sensitivity analysis for MCP-3 on AD

eFigure 29. MIF-associated SNPs with risk of AD

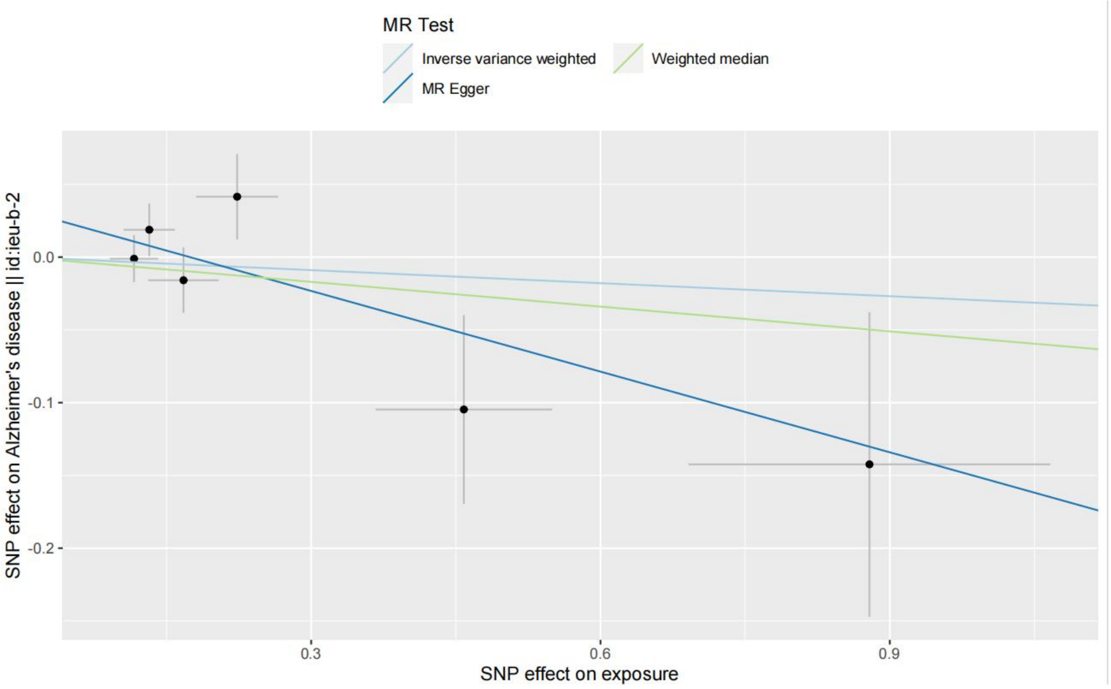

A. Scatter plot of MIF with risk of AD

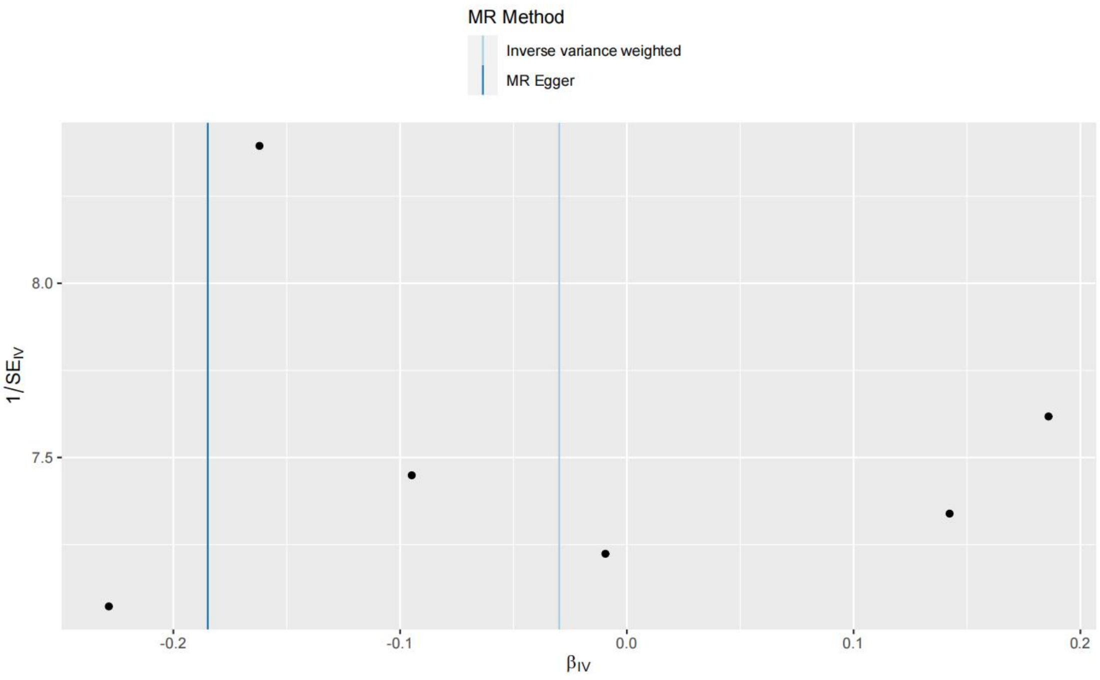

B. Funnel plot of MIF instruments strength on AD

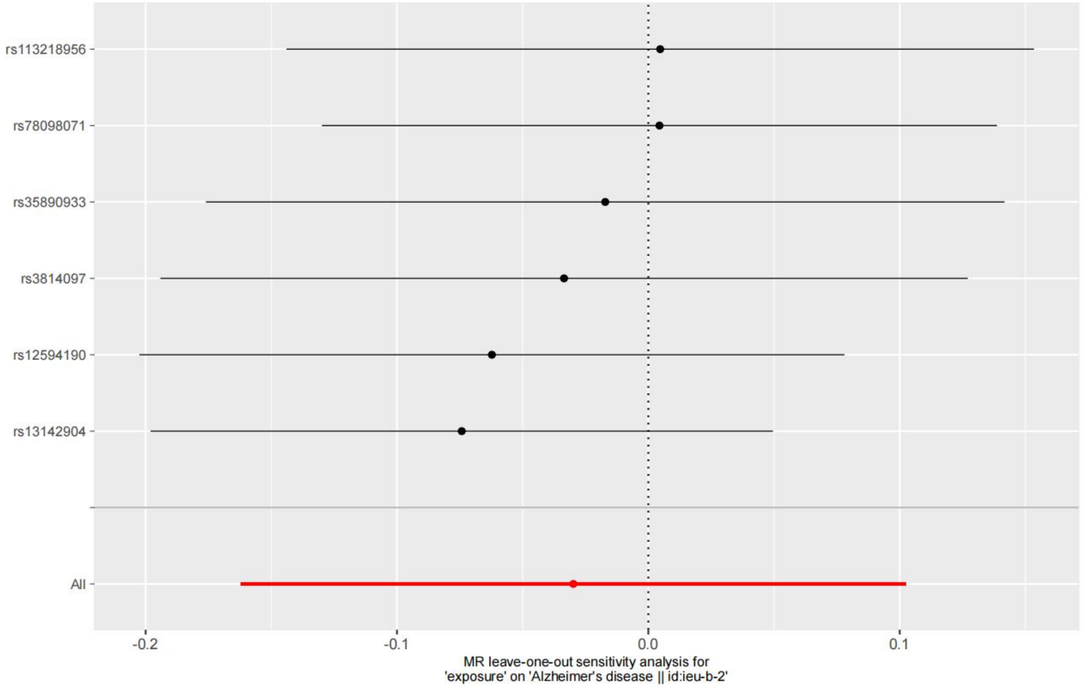

C. MR leave-one-out sensitivity analysis for MIF on AD

eFigure 30. MIG-associated SNPs with risk of AD

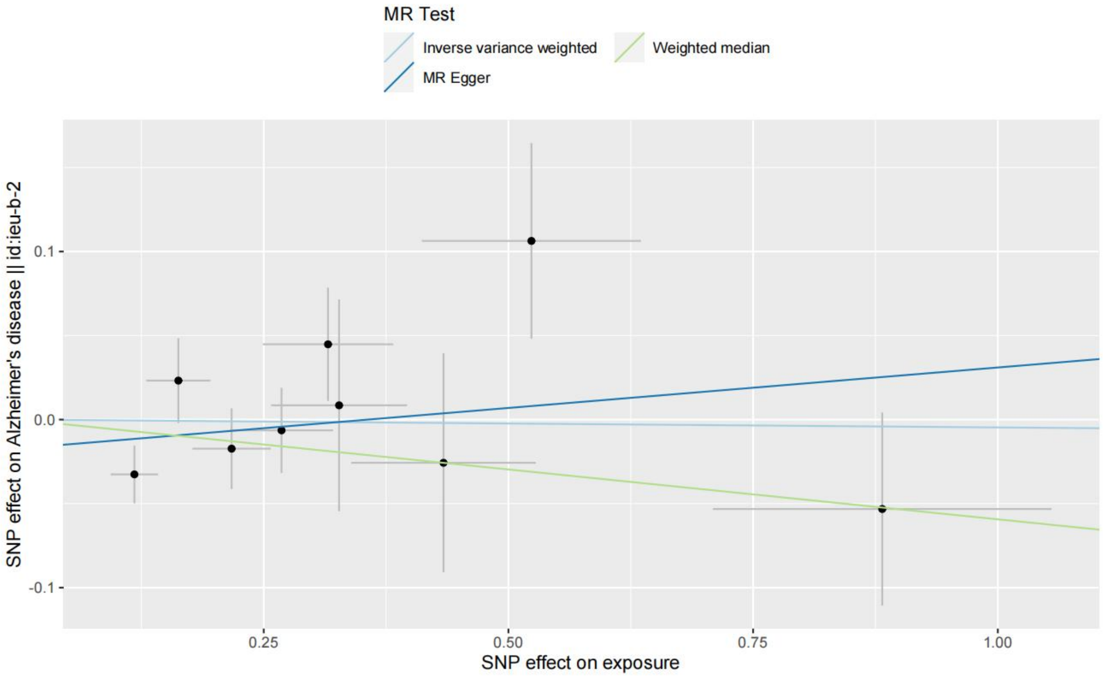

A. Scatter plot of MIG with risk of AD

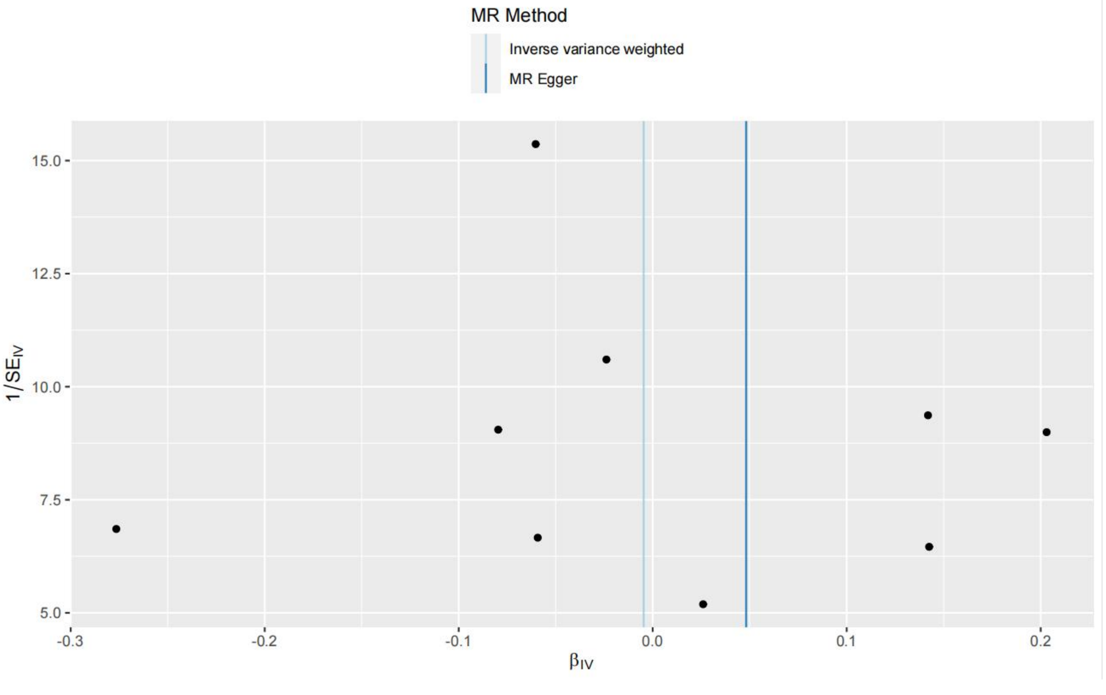

B. Funnel plot of MIG instruments strength on AD

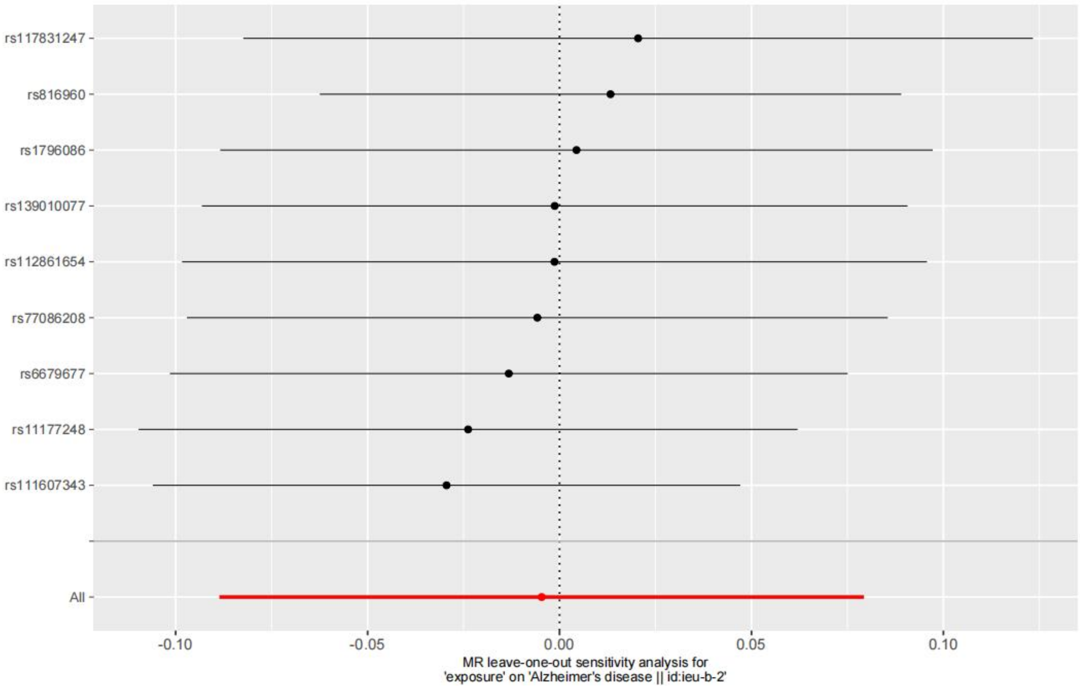

C. MR leave-one-out sensitivity analysis for MIG on AD

eFigure 31. MIP-1A-associated SNPs with risk of AD

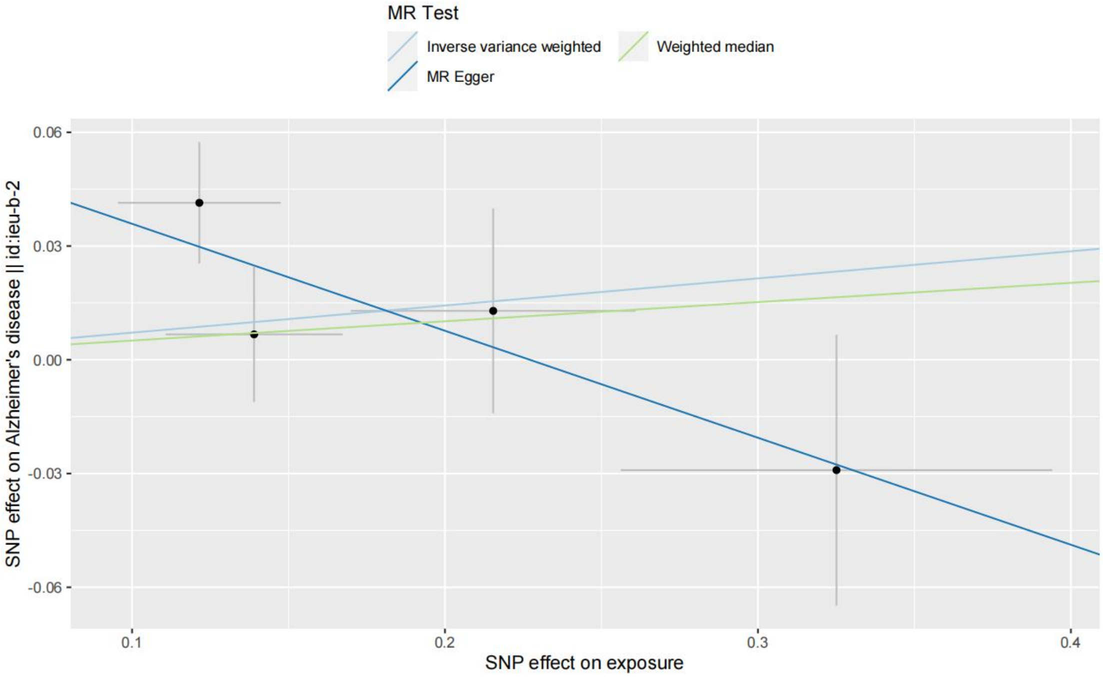

A. Scatter plot of MIP-1A with risk of AD

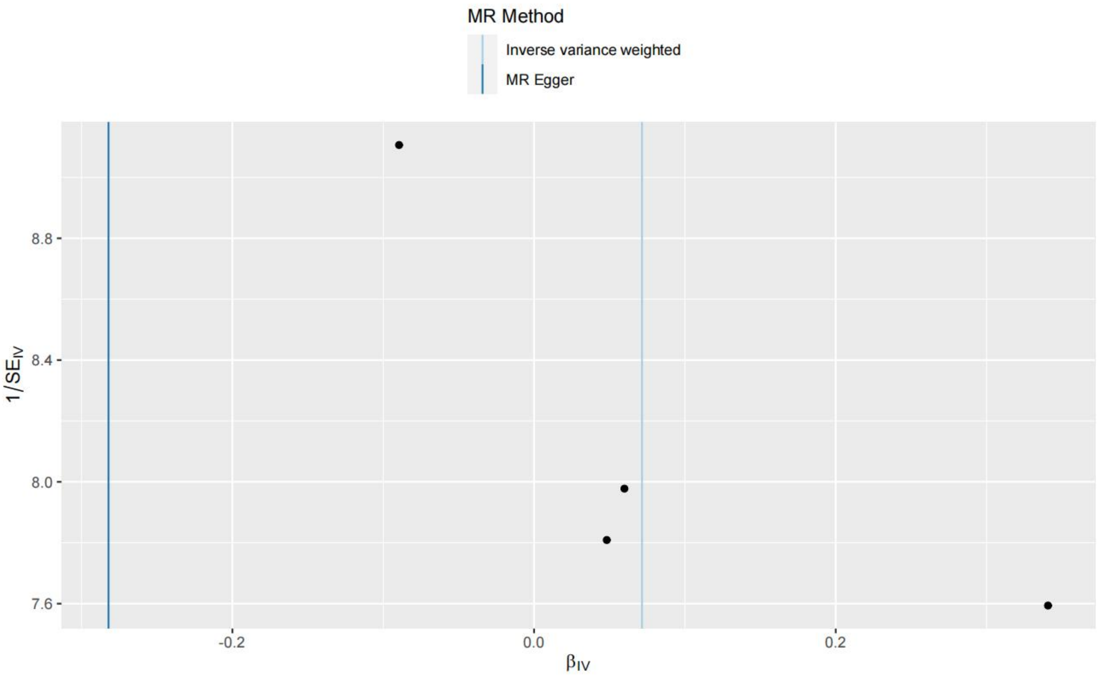

B. Funnel plot of MIP-1A instruments strength on AD

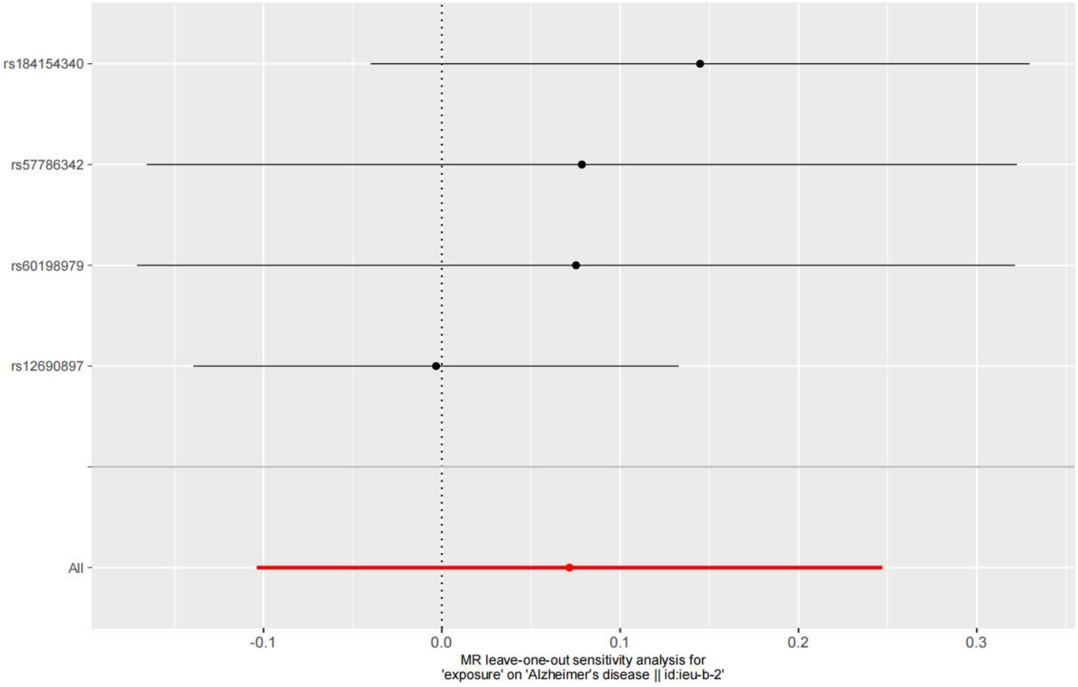

C. MR leave-one-out sensitivity analysis for MIP-1A on AD

eFigure 32. MIP-1B-associated SNPs with risk of AD

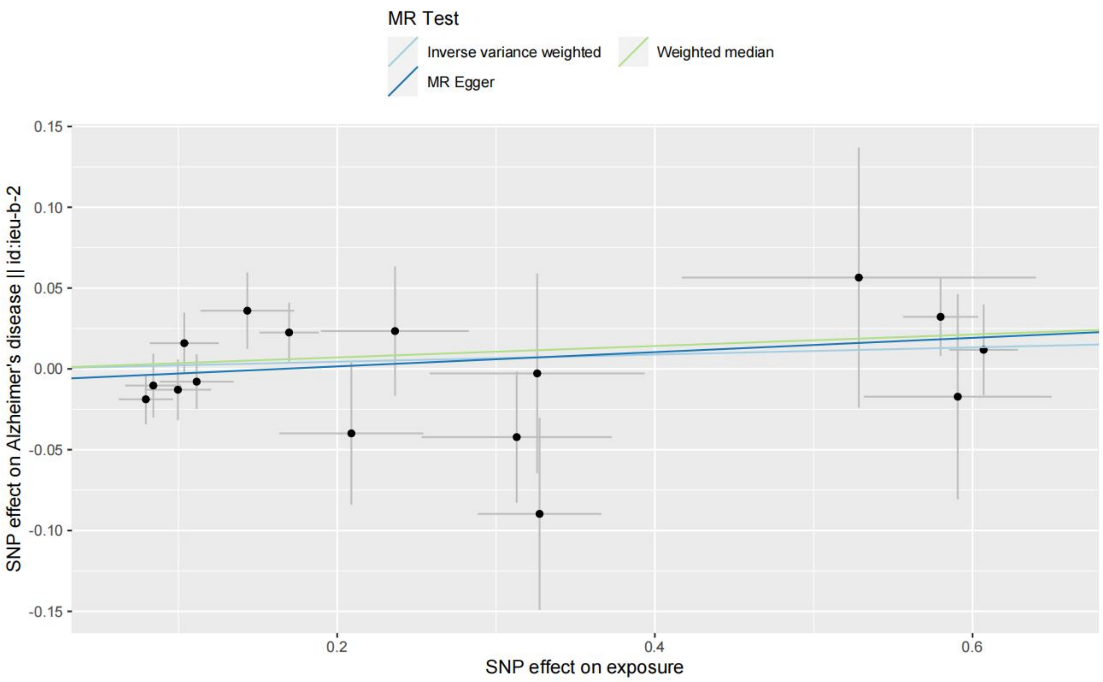

A. Scatter plot of MIP-1B with risk of AD

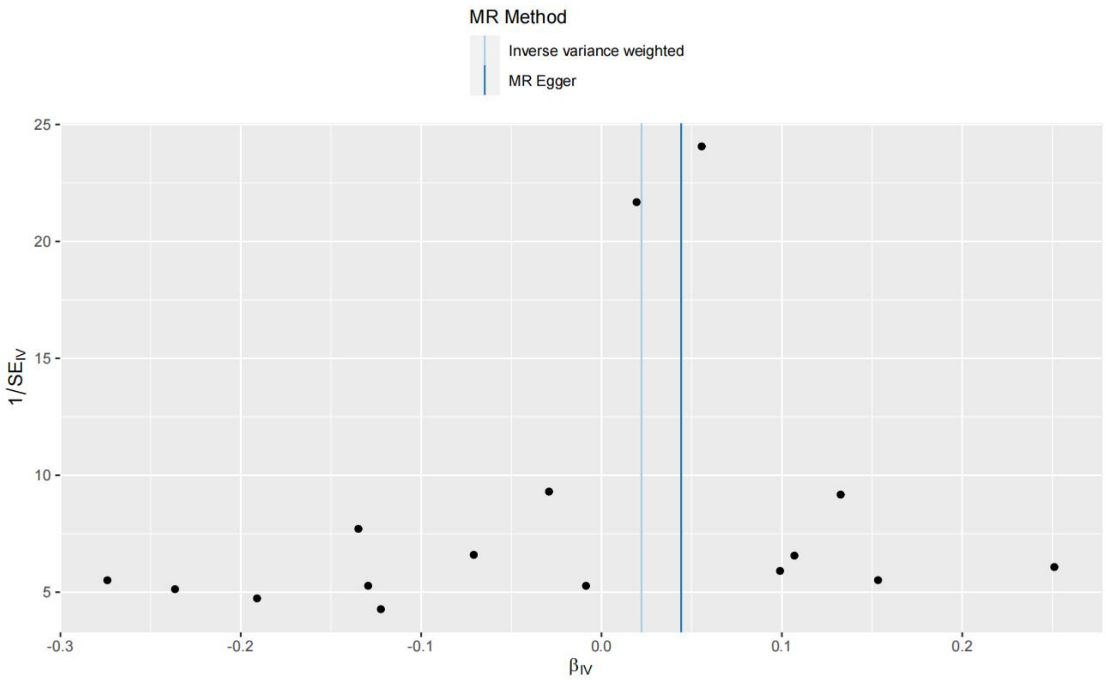

B. Funnel plot of MIP-1B instruments strength on AD

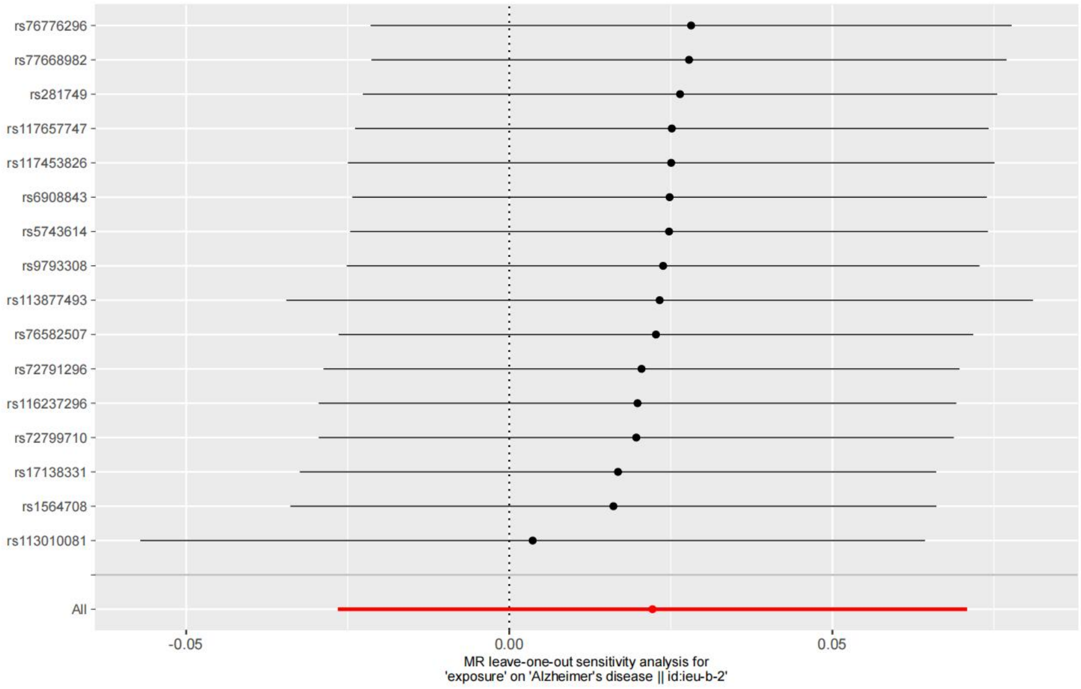

C. MR leave-one-out sensitivity analysis for MIP-1B on AD

eFigure 33. PDGF-BB-associated SNPs with risk of AD

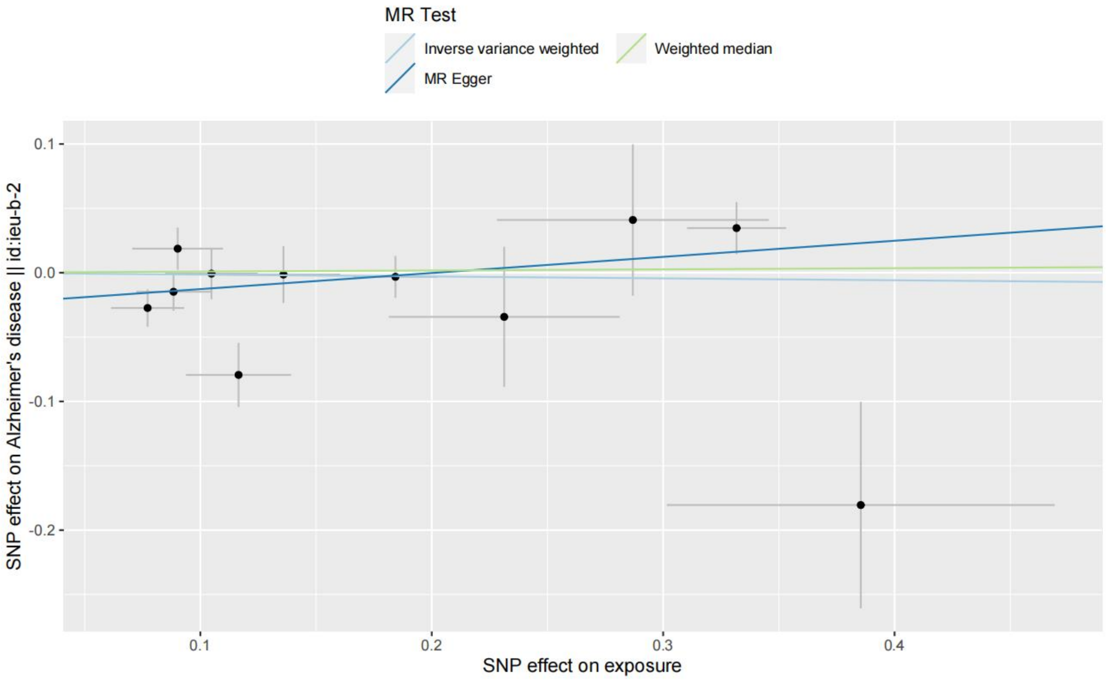

A. Scatter plot of PDGF-BB with risk of AD

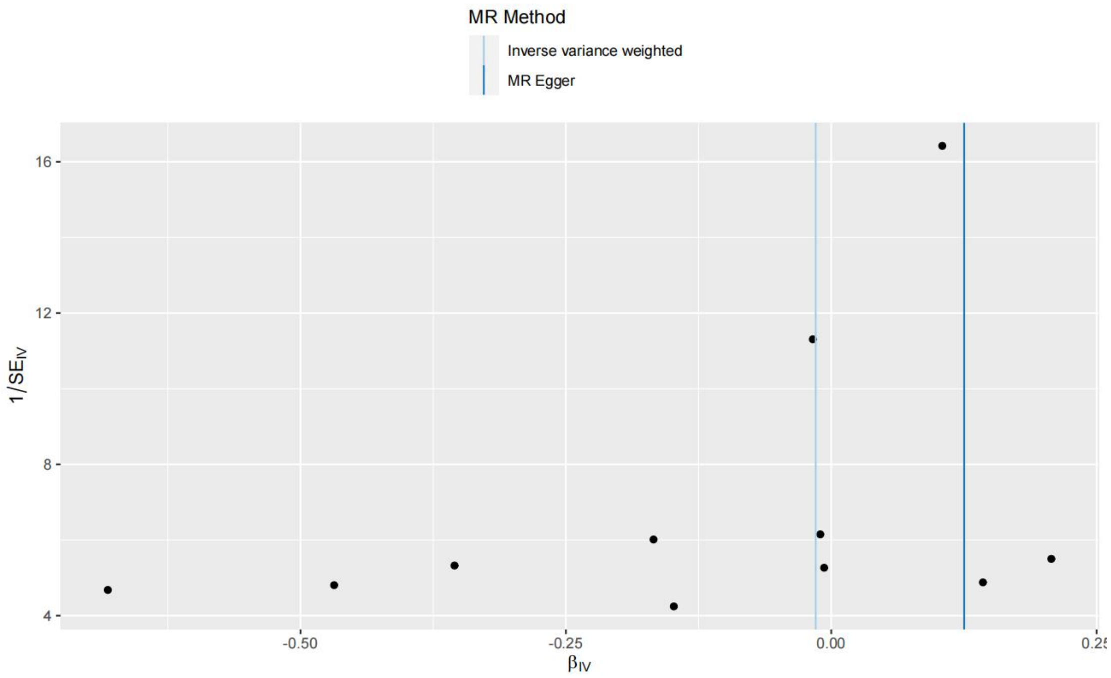

B. Funnel plot of PDGF-BB instruments strength on AD

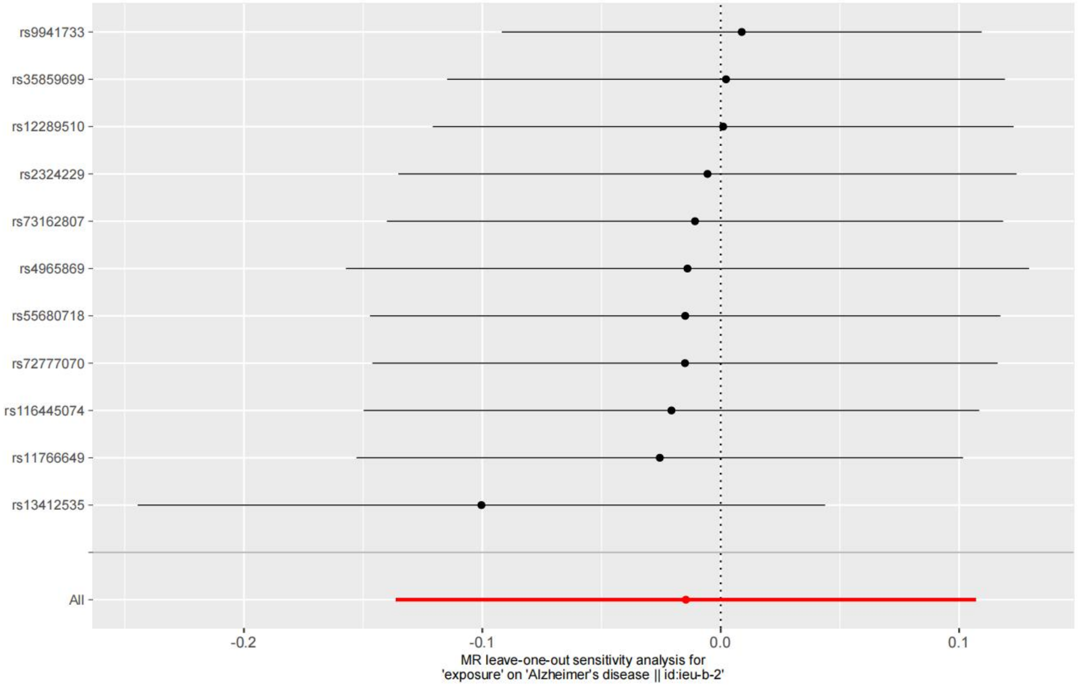

C. MR leave-one-out sensitivity analysis for PDGF-BB on AD

eFigure 34. RANTES-associated SNPs with risk of AD

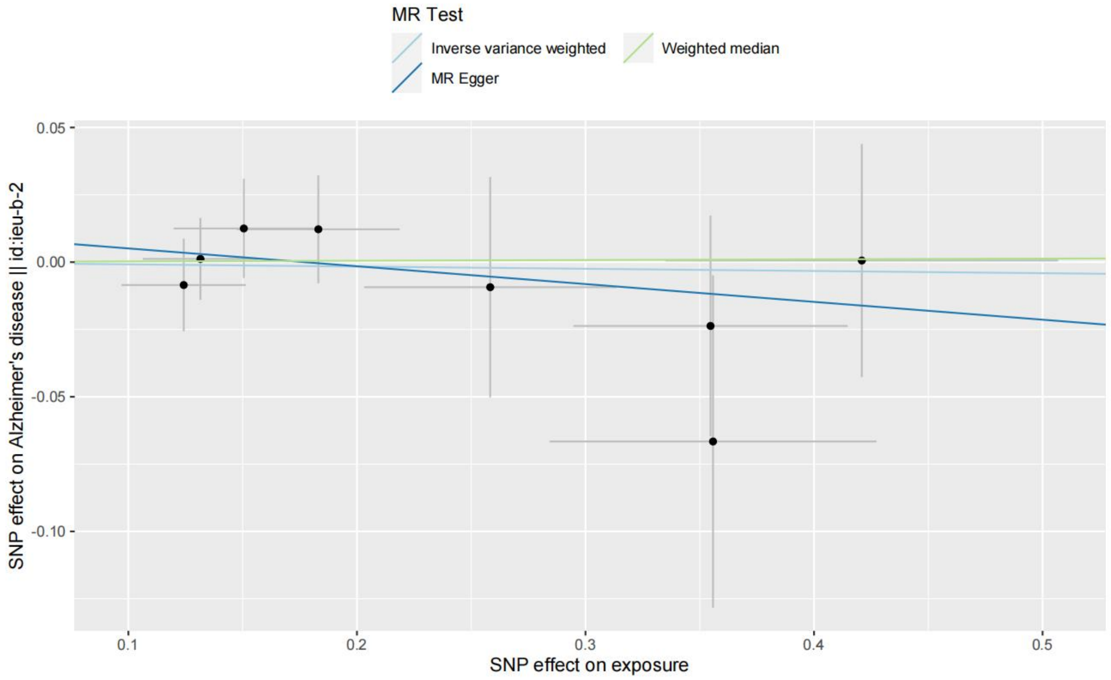

A. Scatter plot of RANTES with risk of AD

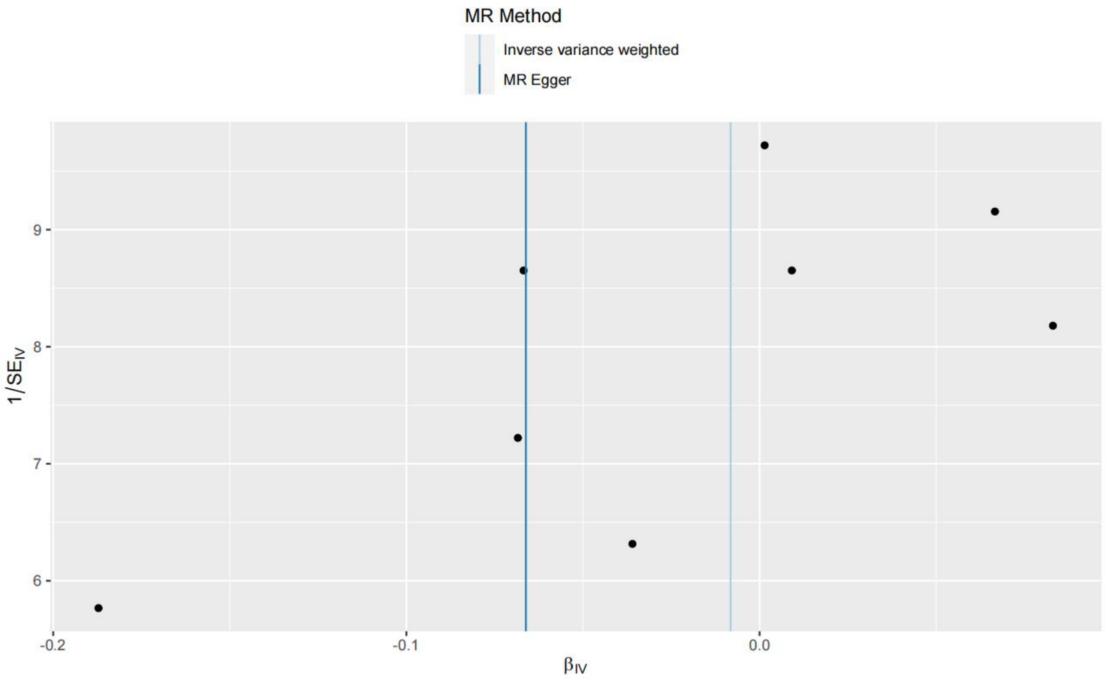

B. Funnel plot of RANTES instruments strength on AD

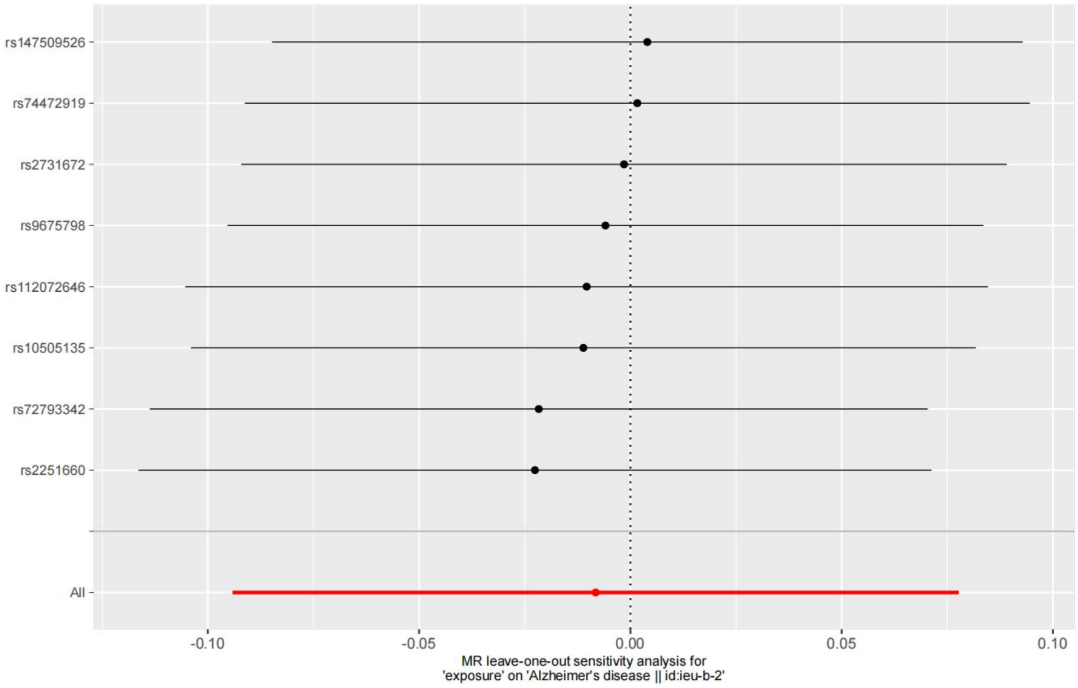

C. MR leave-one-out sensitivity analysis for RANTES on AD

eFigure 35. SCF-associated SNPs with risk of AD

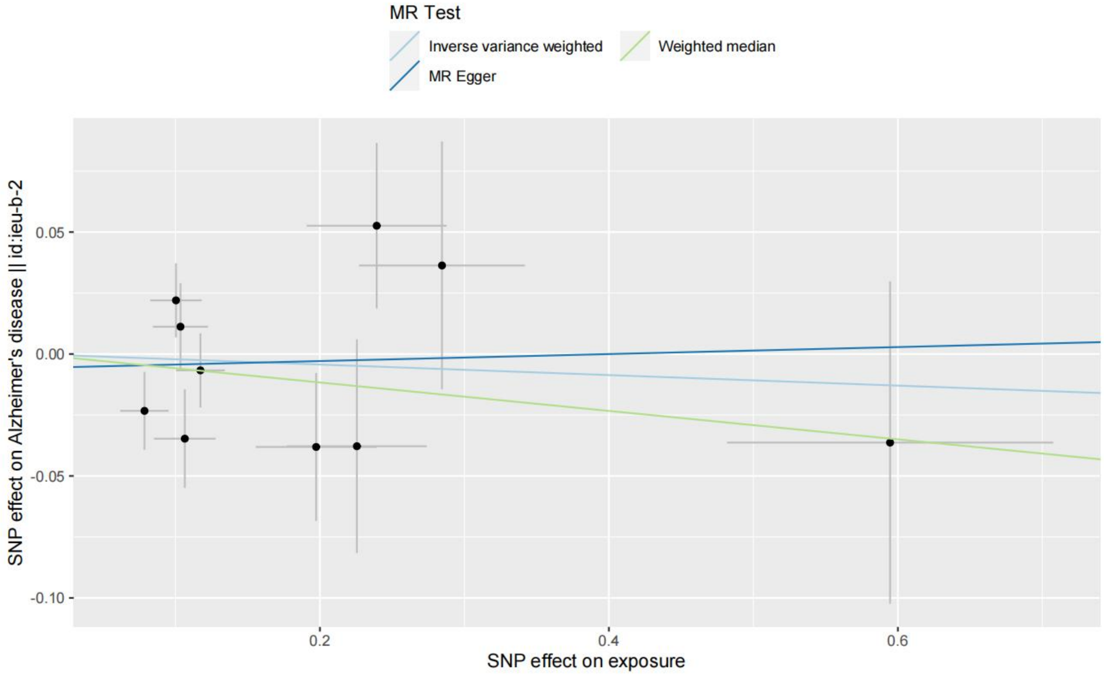

A. Scatter plot of SCF with risk of AD

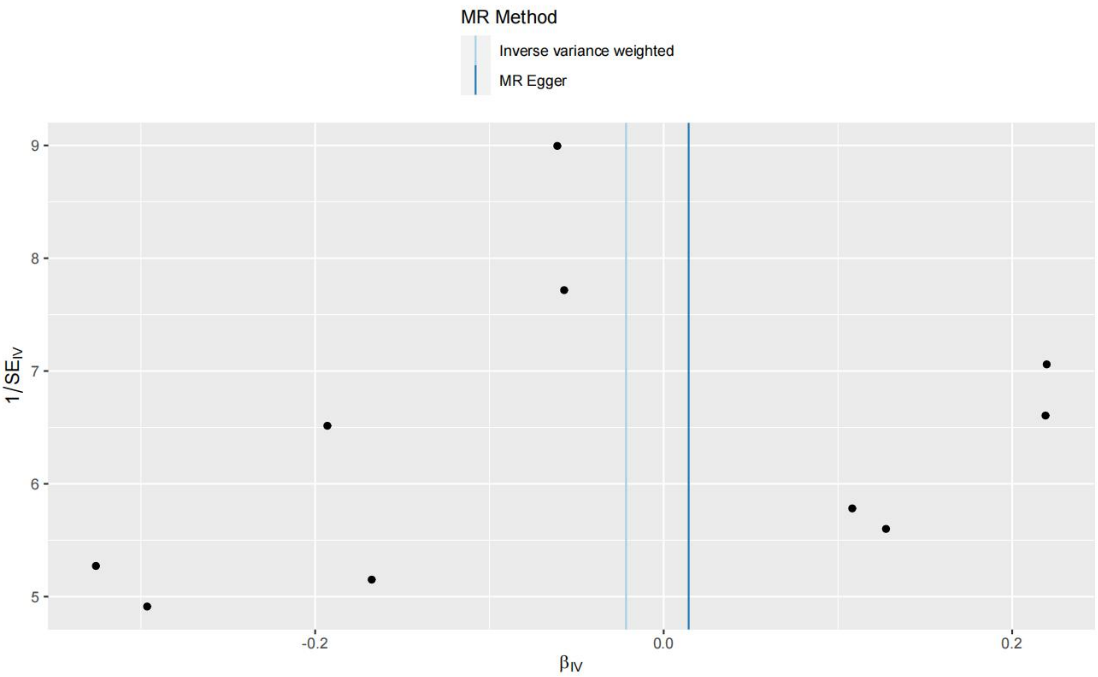

B. Funnel plot of SCF instruments strength on AD

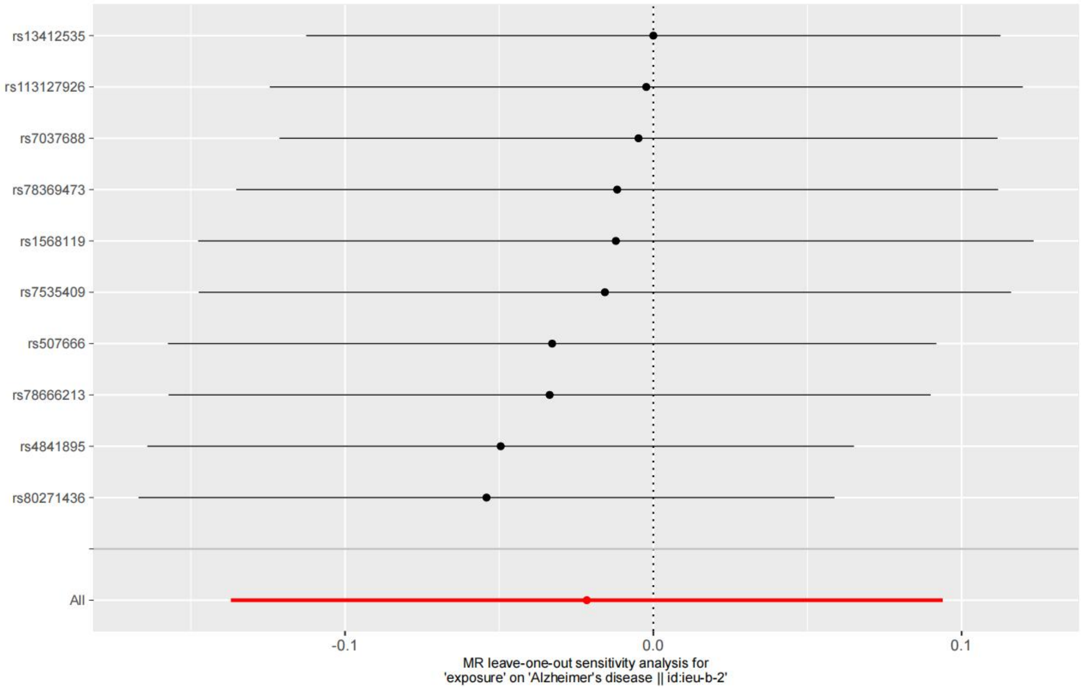

C. MR leave-one-out sensitivity analysis for SCF on AD

eFigure 36. SCGFβ-associated SNPs with risk of AD

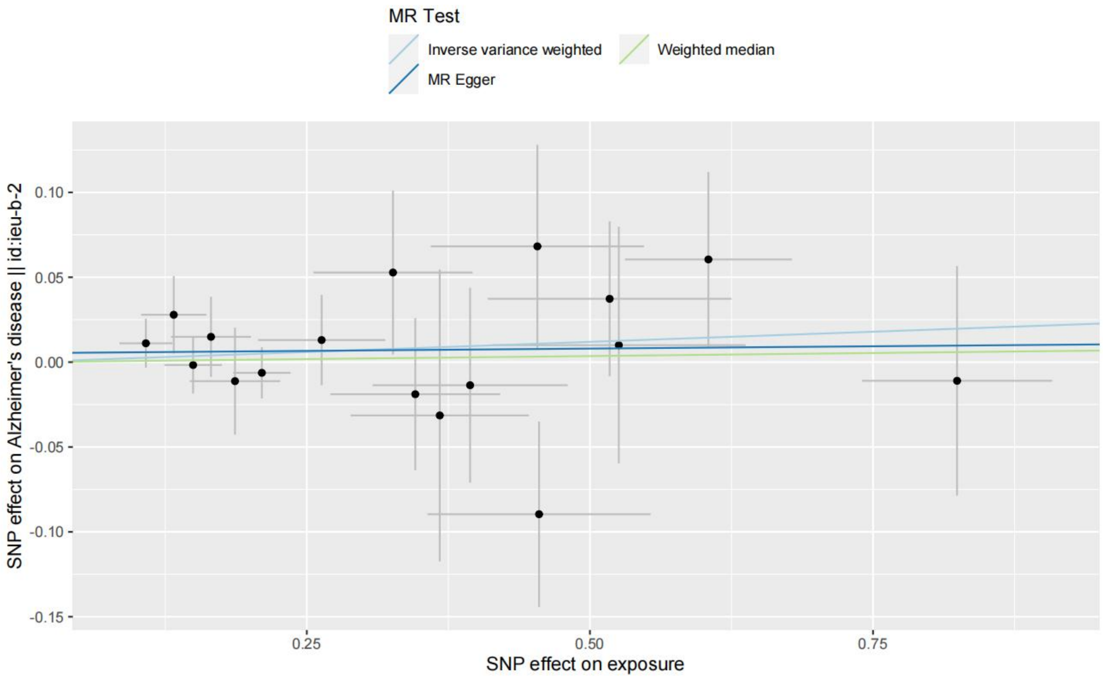

A. Scatter plot of SCGFβ with risk of AD

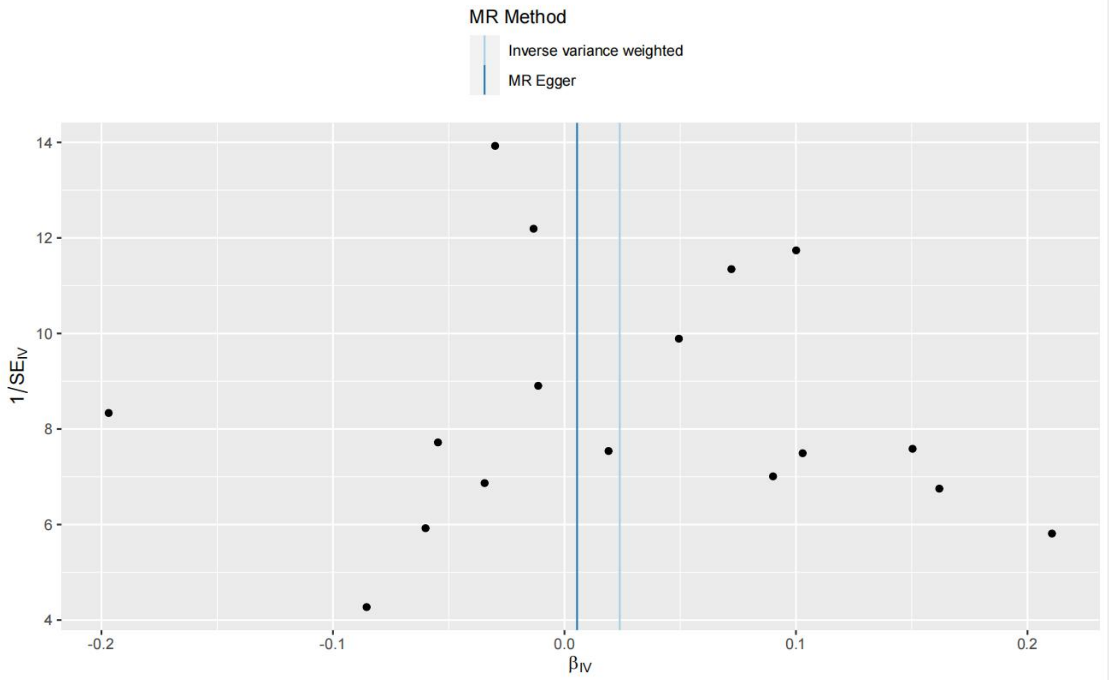

B. Funnel plot of SCGFβ instruments strength on AD

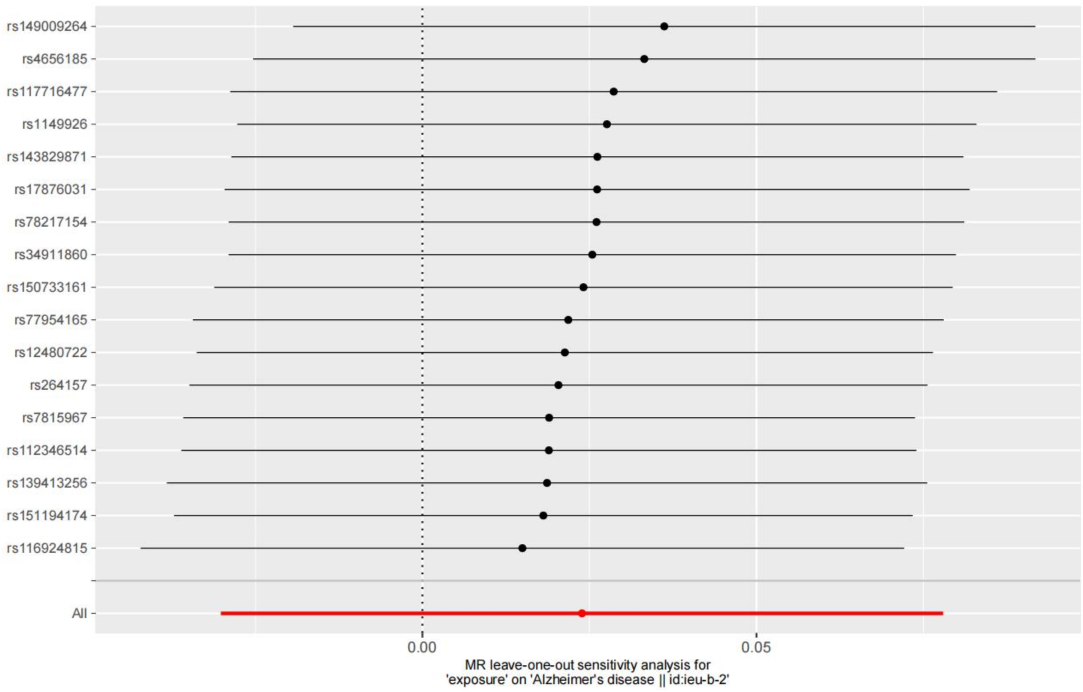

C. MR leave-one-out sensitivity analysis for SCGFβ on AD

eFigure 37. SDF-1A-associated SNPs with risk of AD

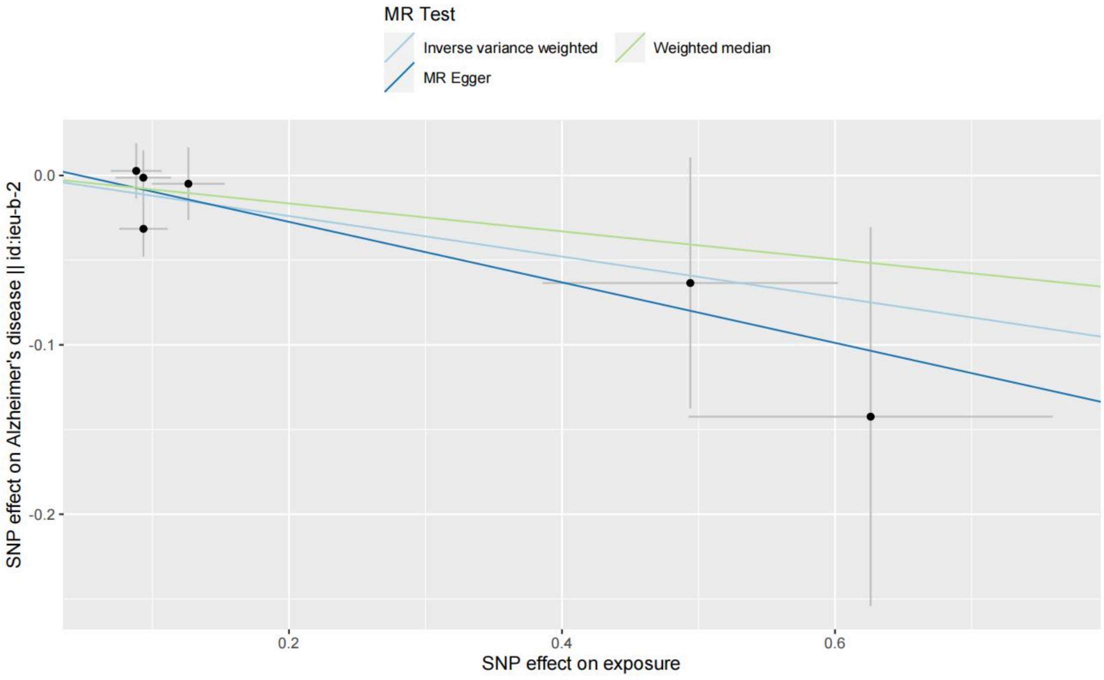

A. Scatter plot of SDF-1A with risk of AD

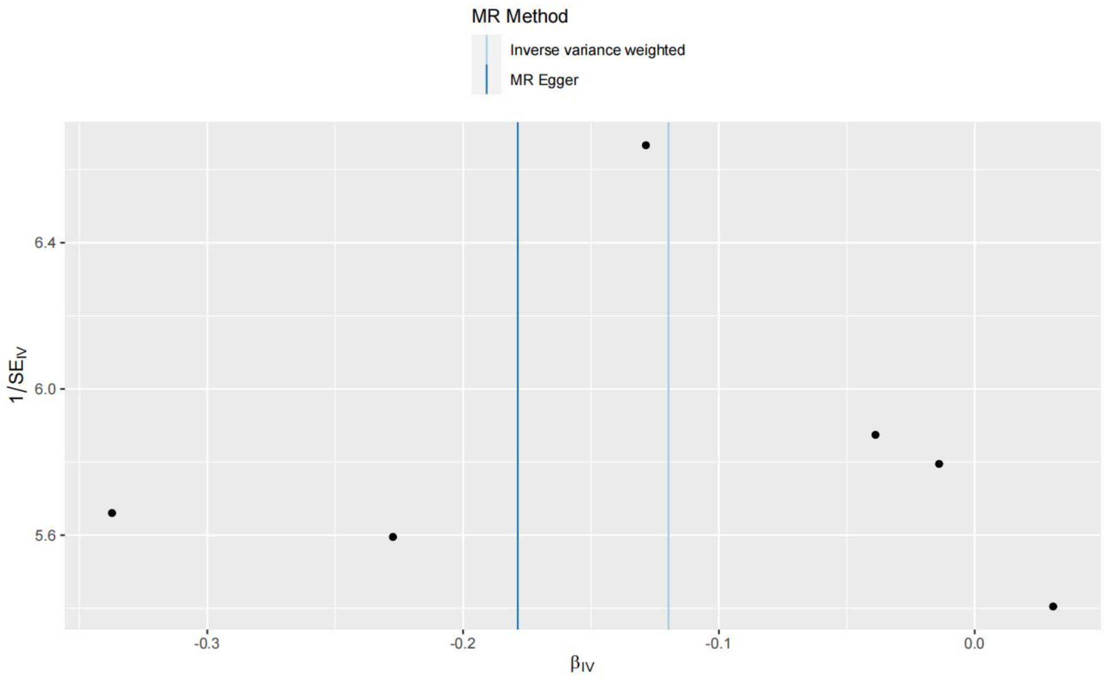

B. Funnel plot of SDF-1A instruments strength on AD

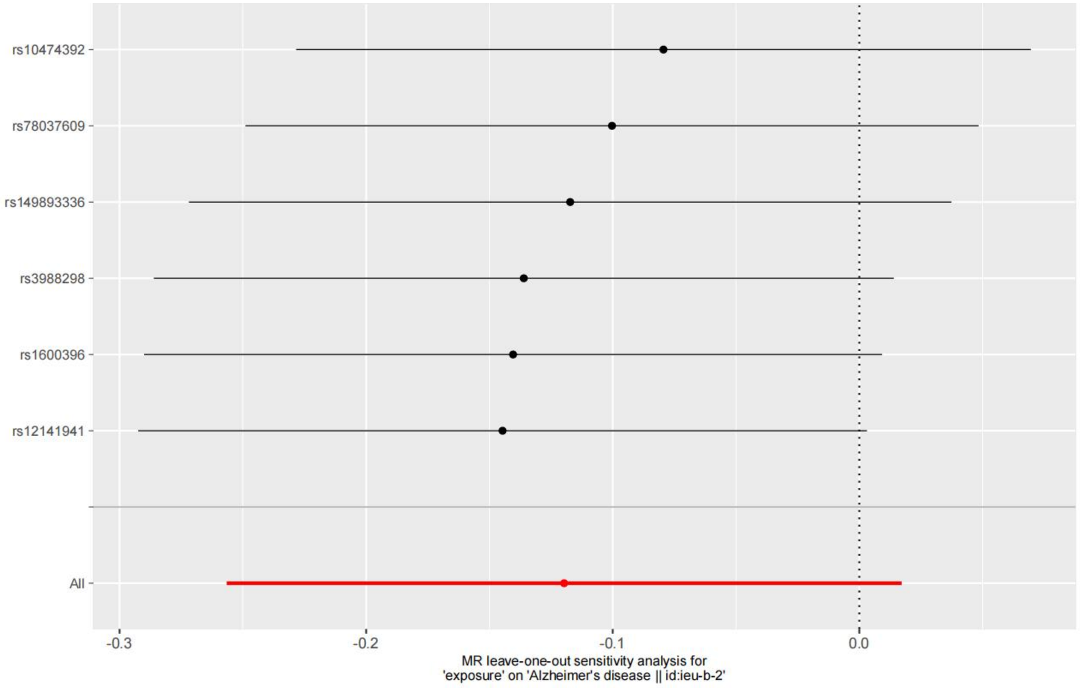

C. MR leave-one-out sensitivity analysis for SDF-1A on AD

eFigure 38. TNF-A-associated SNPs with risk of AD

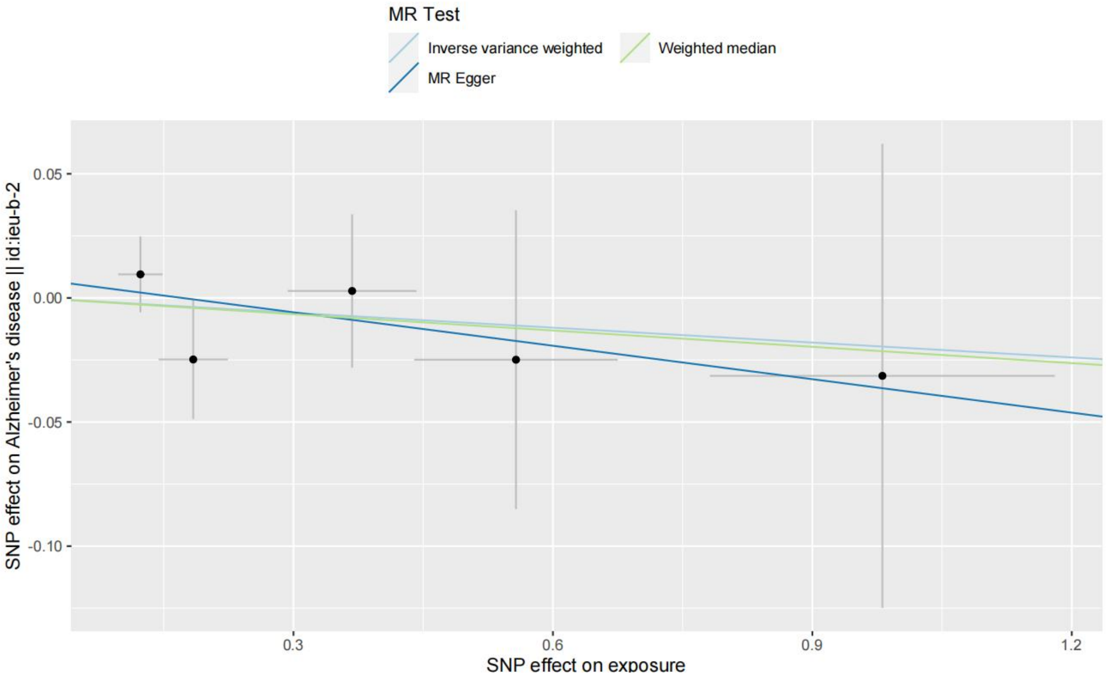

A. Scatter plot of TNF-A with risk of AD

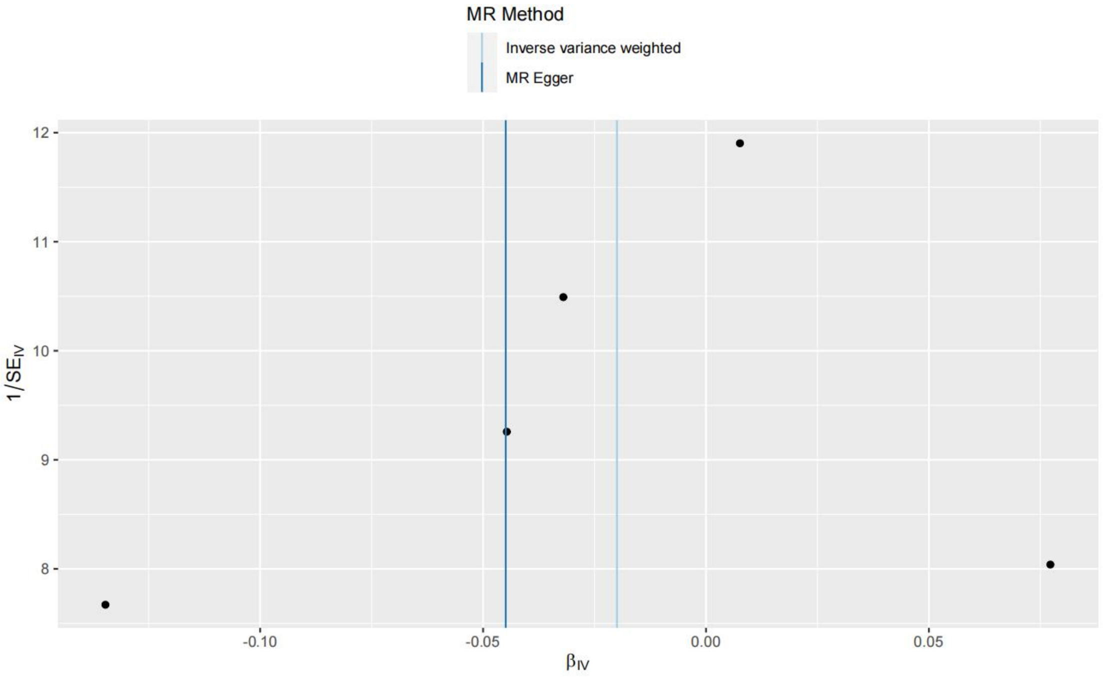

B. Funnel plot of TNF-A instruments strength on AD

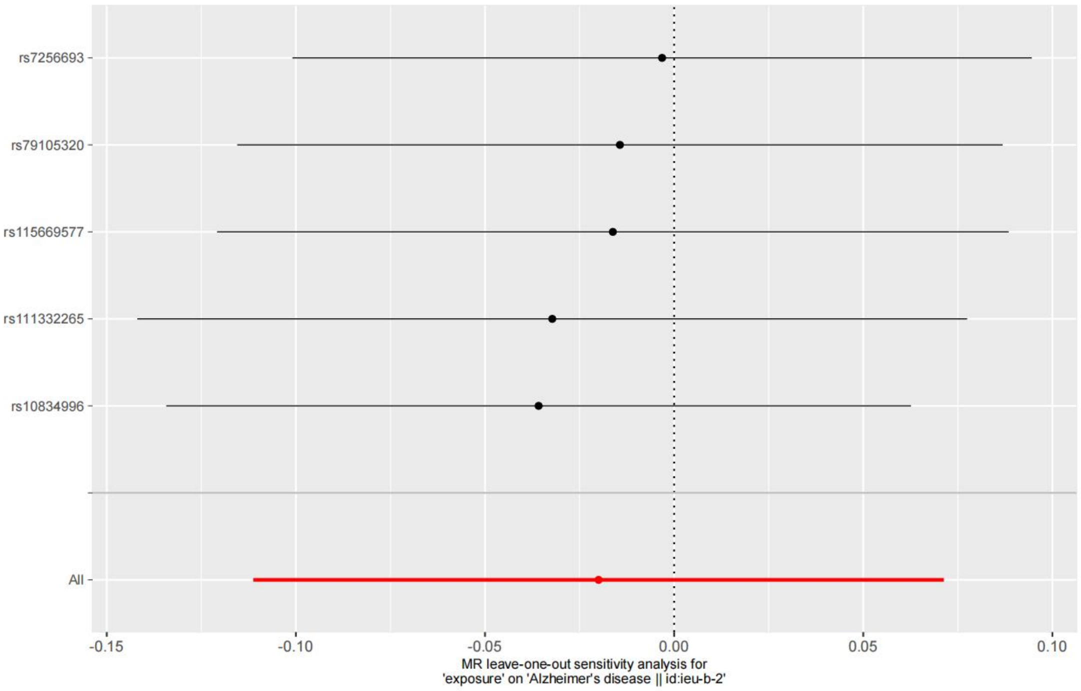

C. MR leave-one-out sensitivity analysis for TNF-A on AD

eFigure 39. TNF-B-associated SNPs with risk of AD

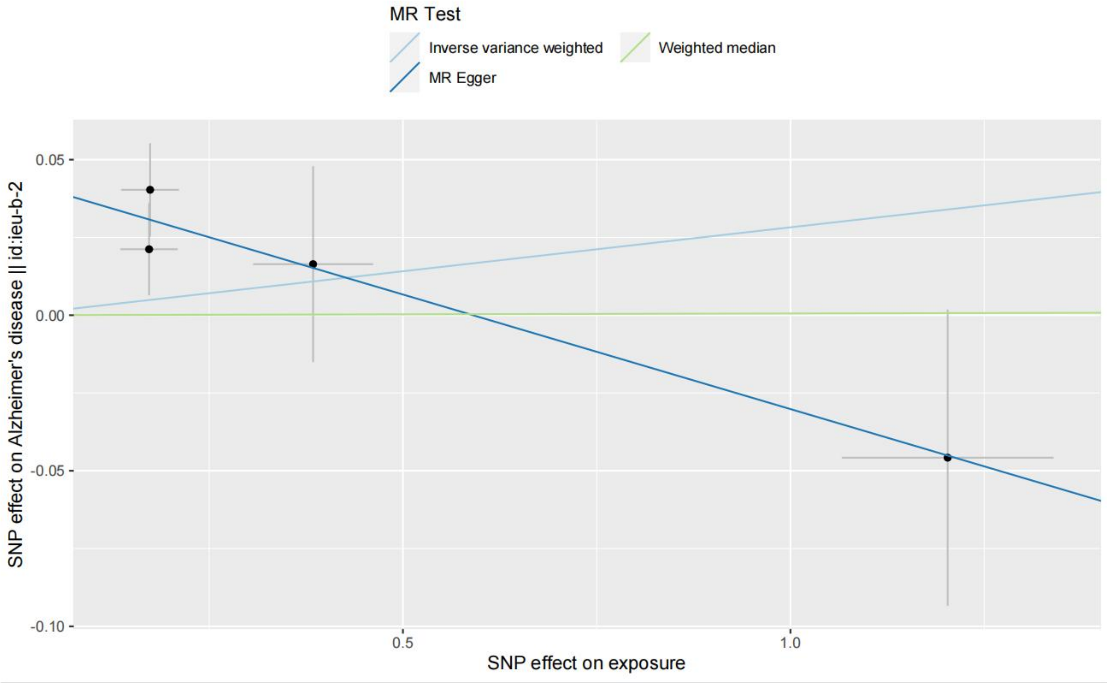

A. Scatter plot of TNF-B with risk of AD

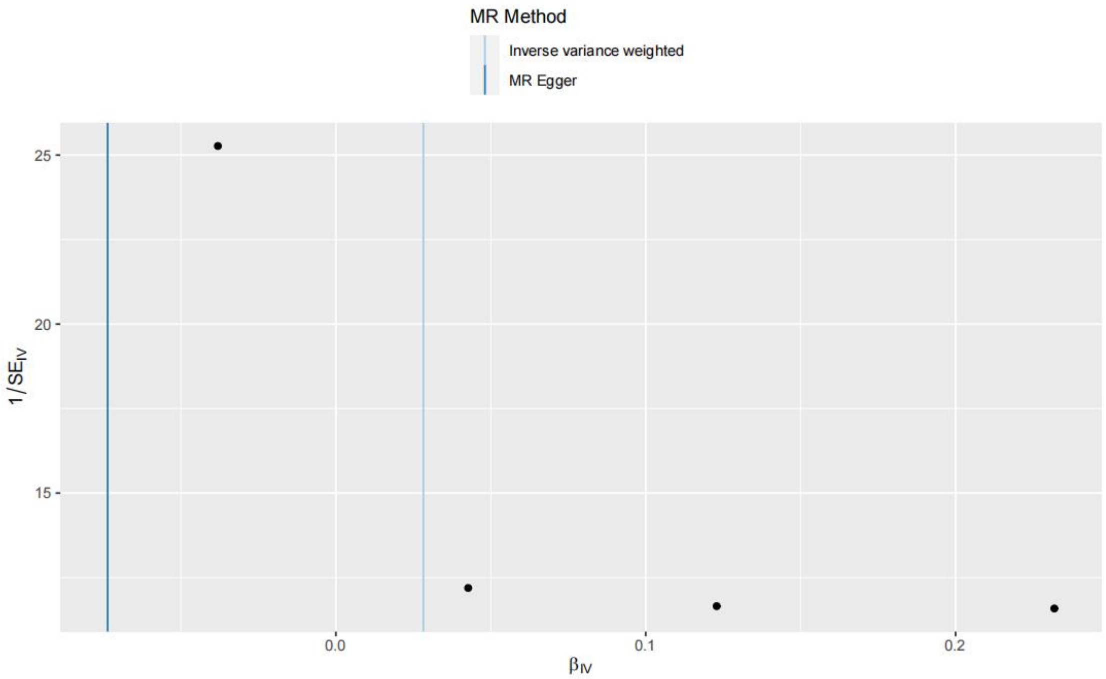

B. Funnel plot of TNF-B instruments strength on AD

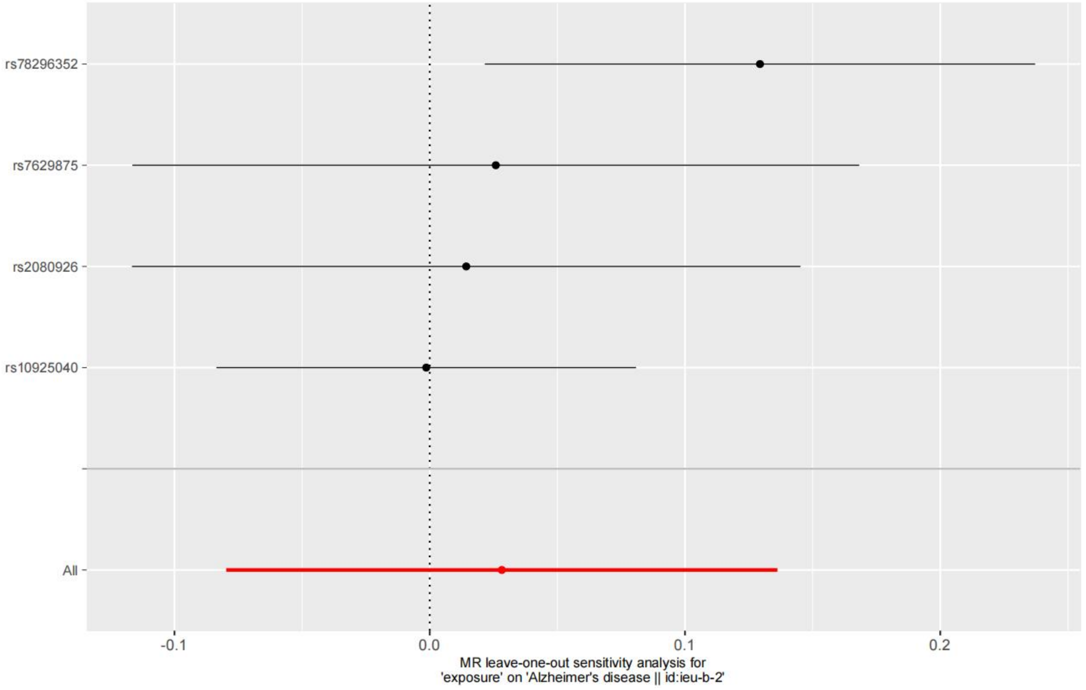

C. MR leave-one-out sensitivity analysis for TNF-B on AD

eFigure 40. TRAIL-associated SNPs with risk of AD

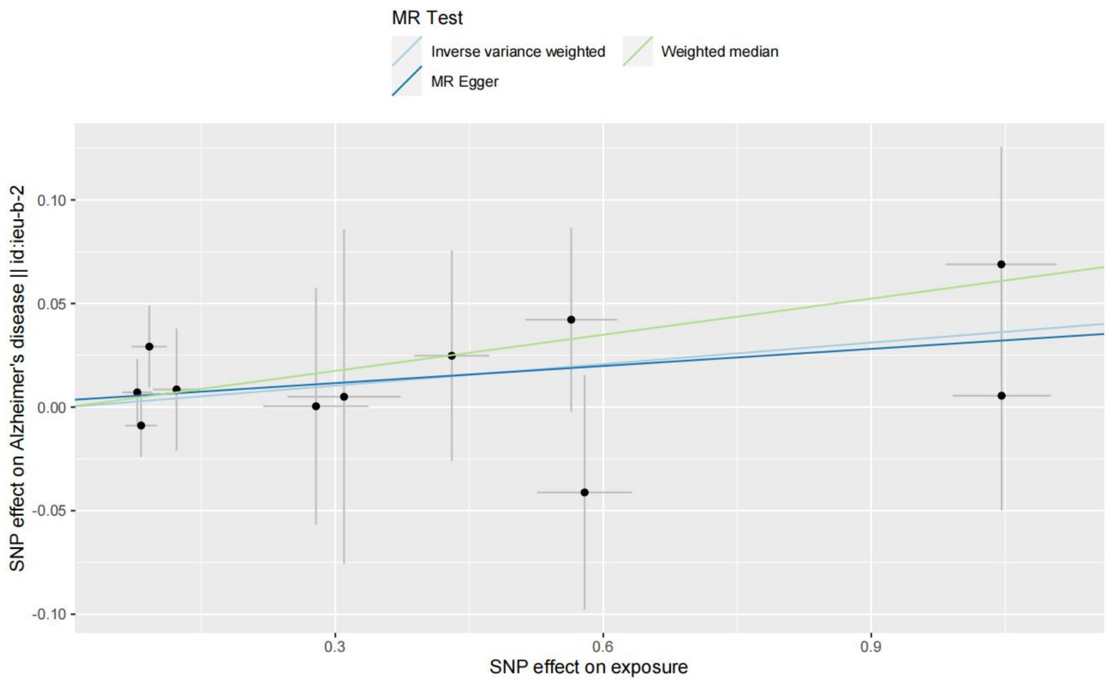

A. Scatter plot of TRAIL with risk of AD

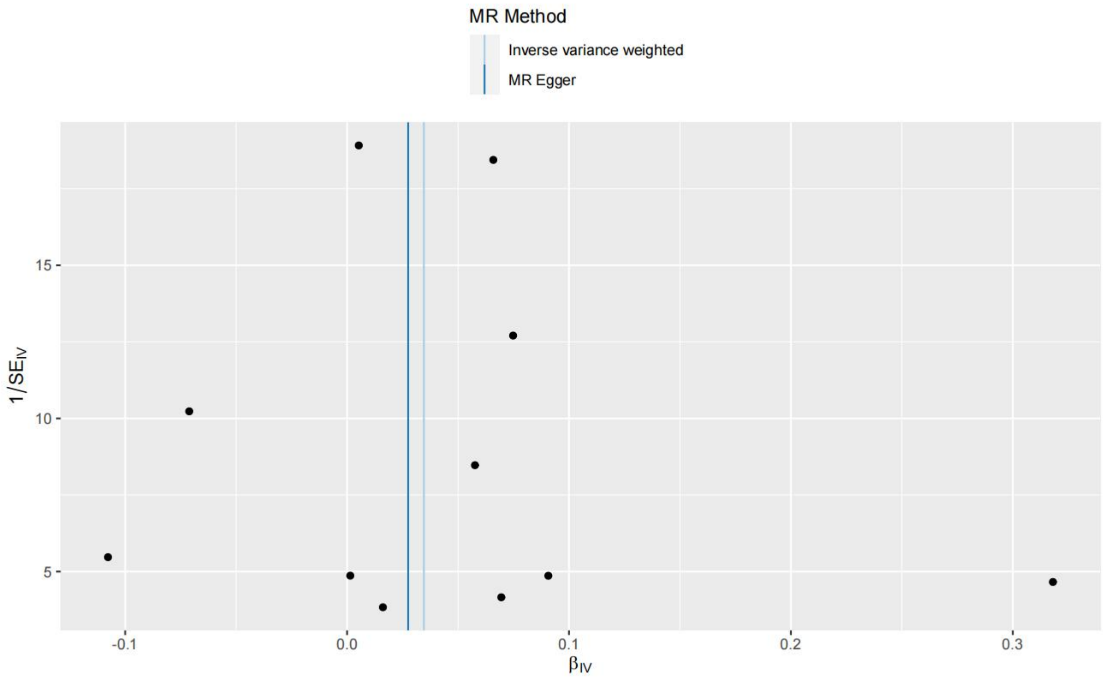

B. Funnel plot of TRAIL instruments strength on AD

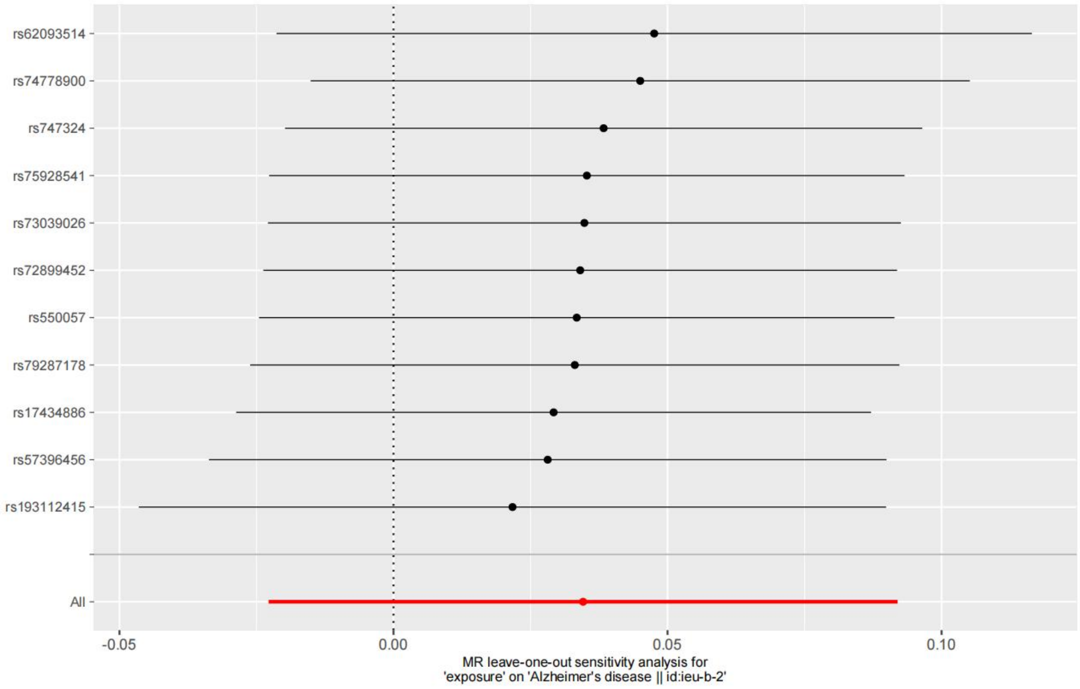

C. MR leave-one-out sensitivity analysis for TRAIL on AD

eFigure 41. VEGF-associated SNPs with risk of AD

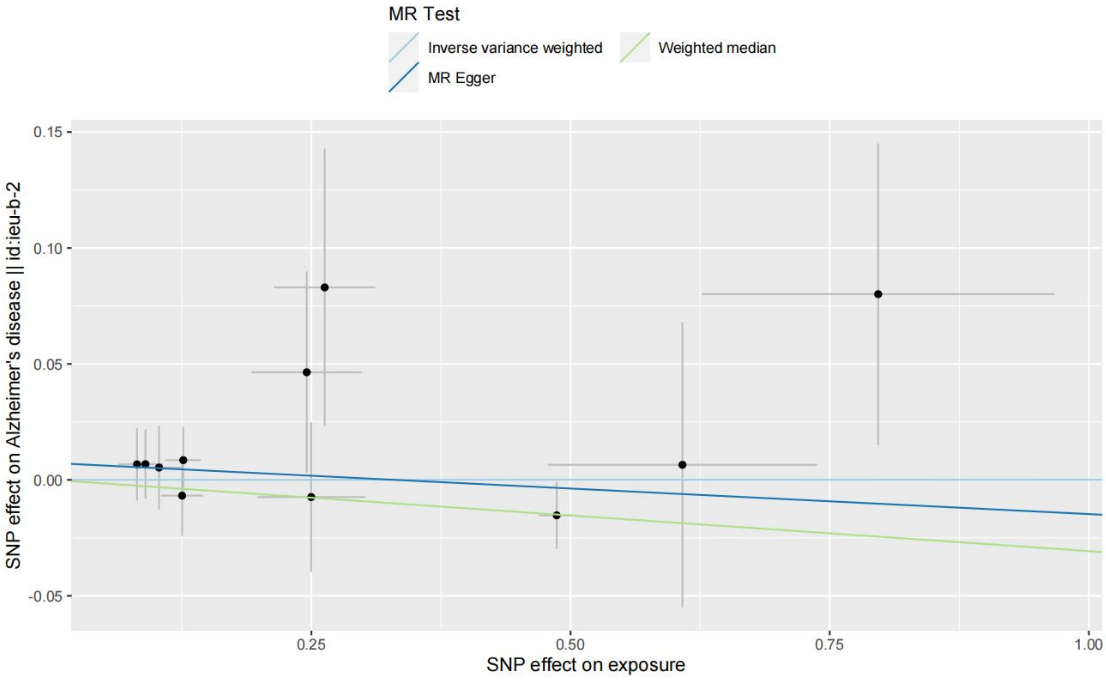

A. Scatter plot of VEGF with risk of AD

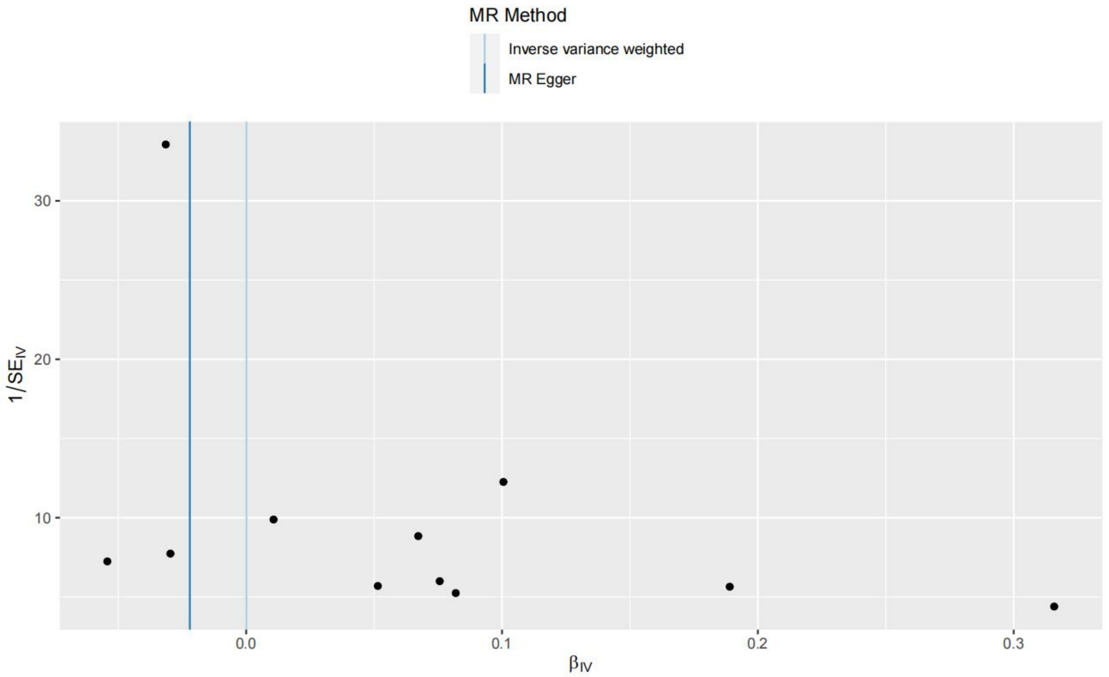

B. Funnel plot of VEGF instruments strength on AD

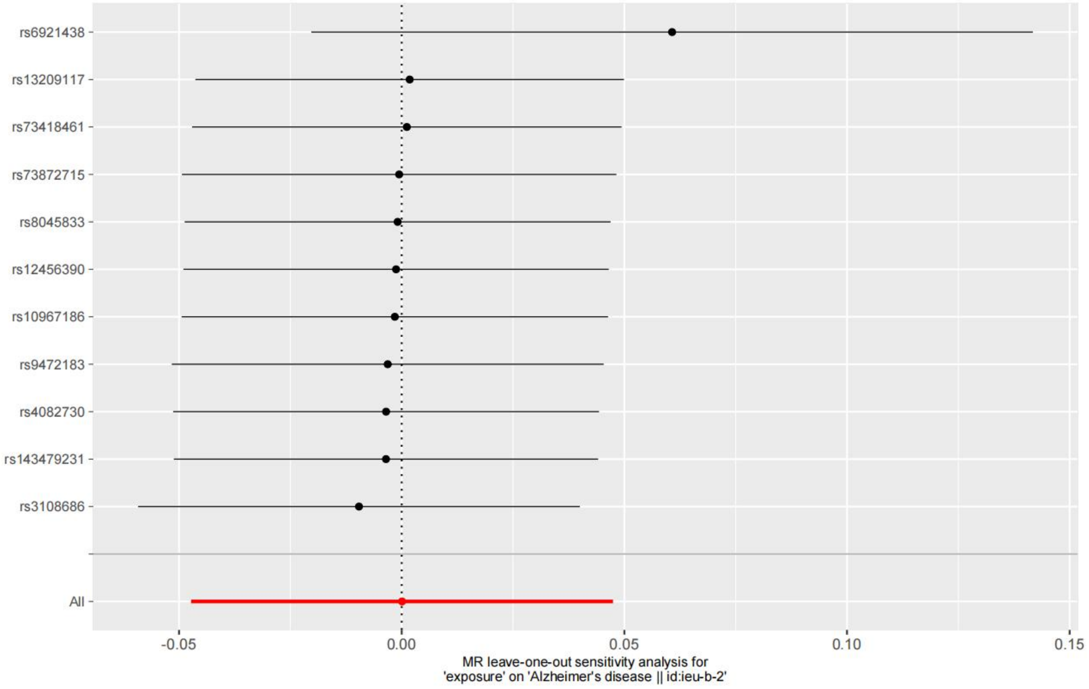

C. MR leave-one-out sensitivity analysis for VEGF on AD

eFigure 42. BNGF-associated SNPs with risk of PD

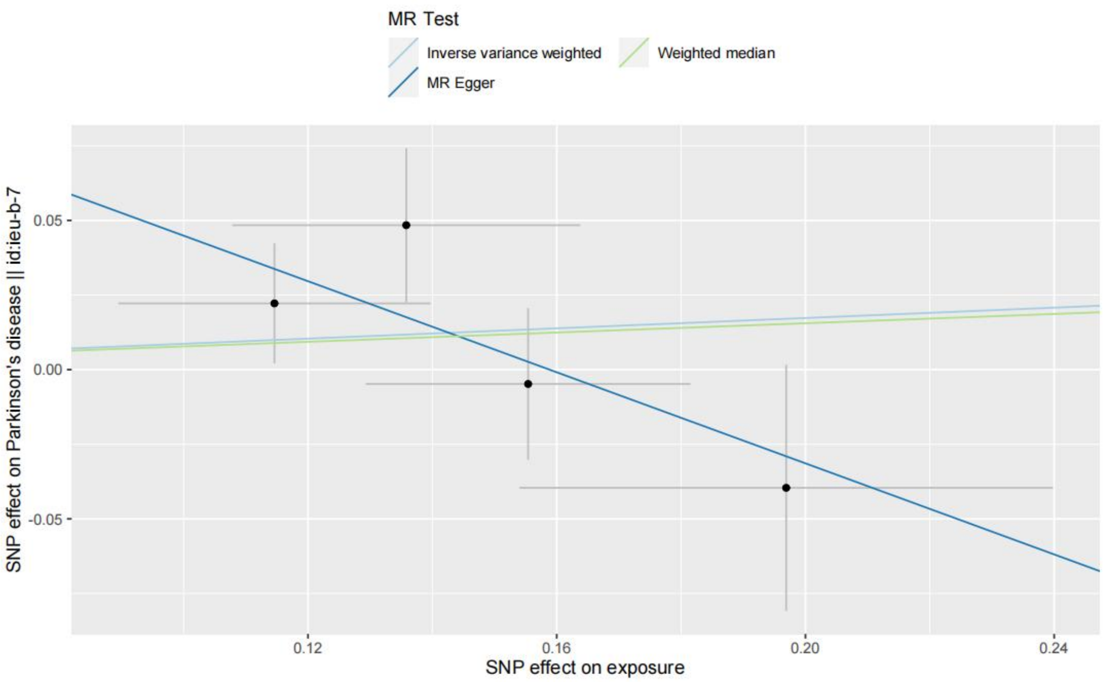

A. Scatter plot of BNGF with risk of PD

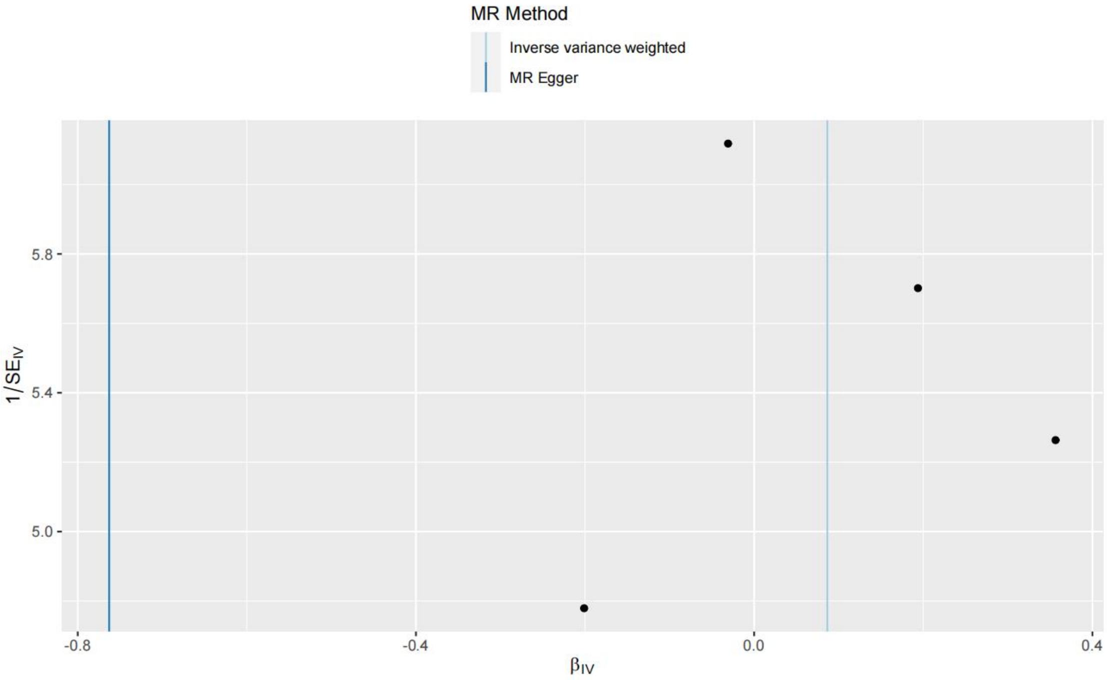

B. Funnel plot of BNGF instruments strength on PD

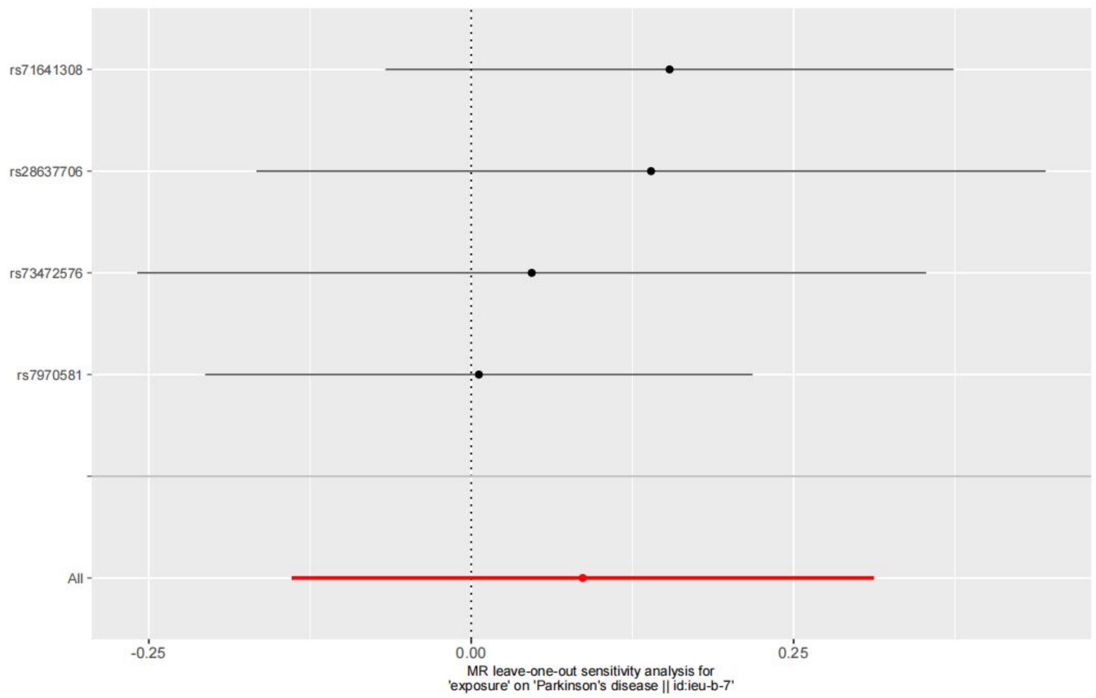

C. MR leave-one-out sensitivity analysis for BNGF on PD

eFigure 43. CTACK-associated SNPs with risk of PD

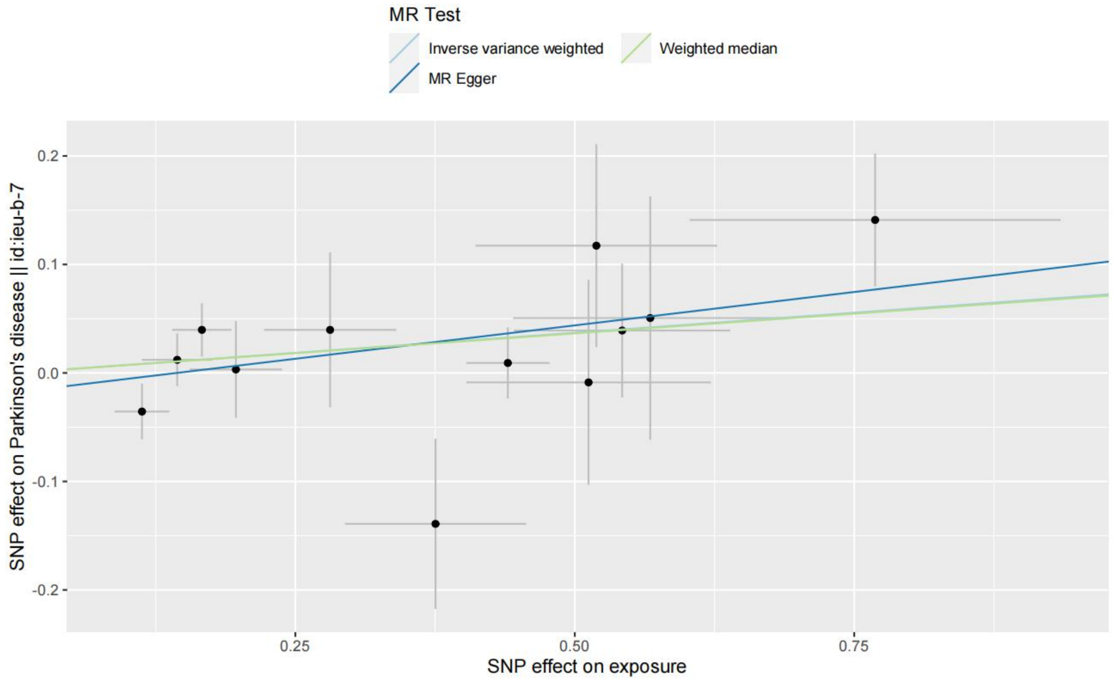

A. Scatter plot of CTACK with risk of PD

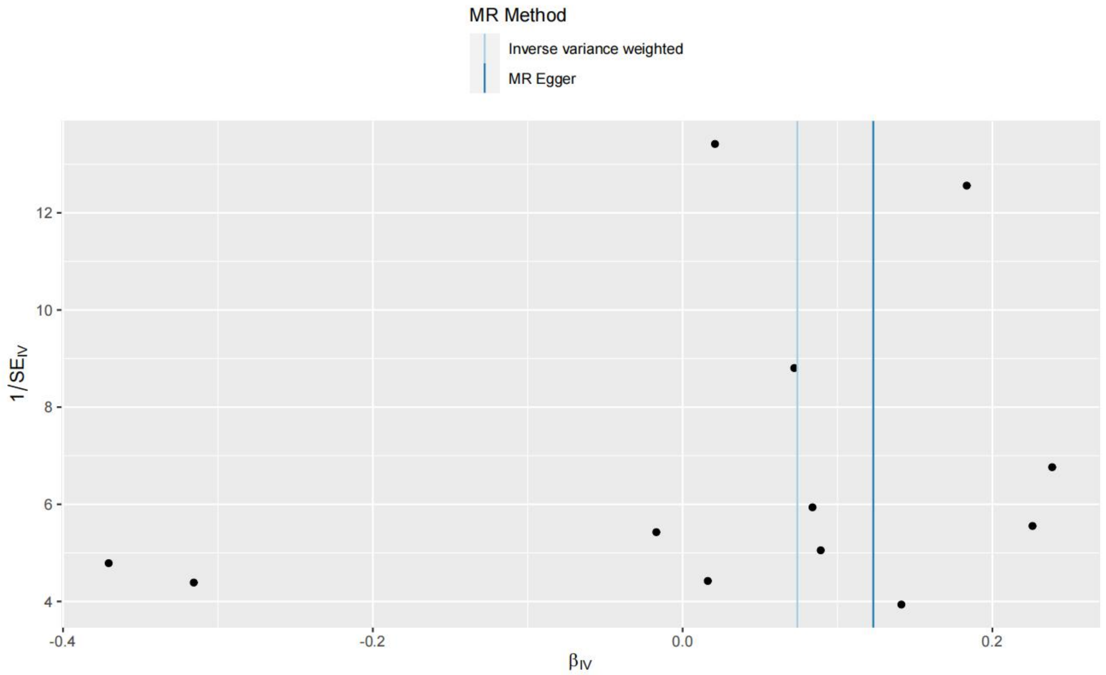

B. Funnel plot of CTACK instruments strength on PD

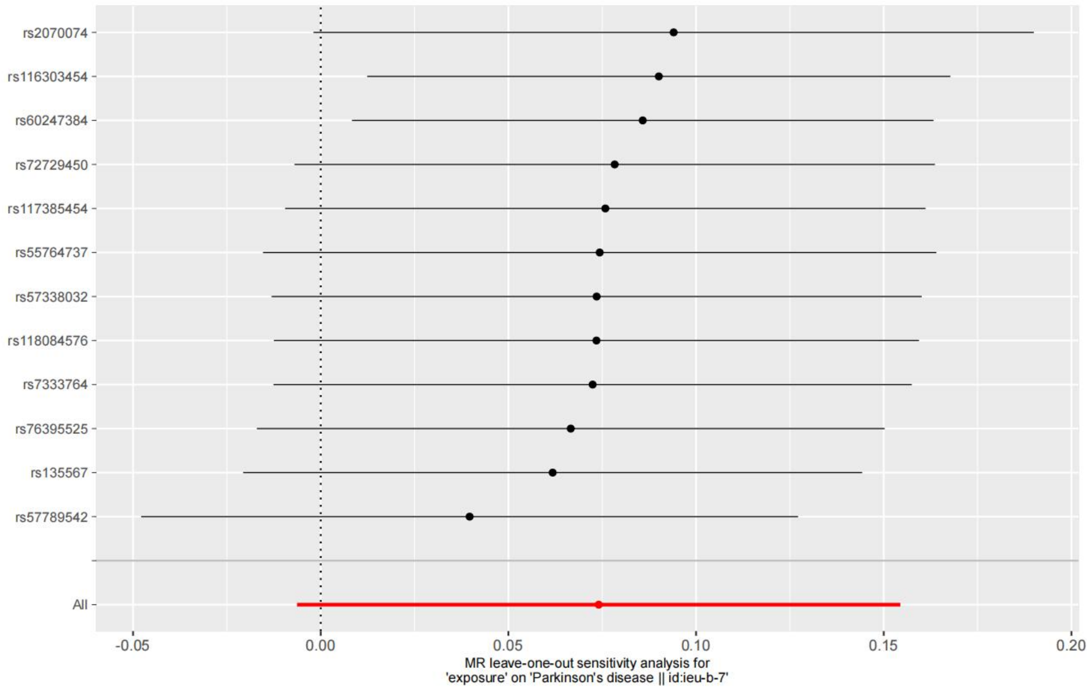

C. MR leave-one-out sensitivity analysis for CTACK-transferase on PD

eFigure 44. EOTAXIN-associated SNPs with risk of PD

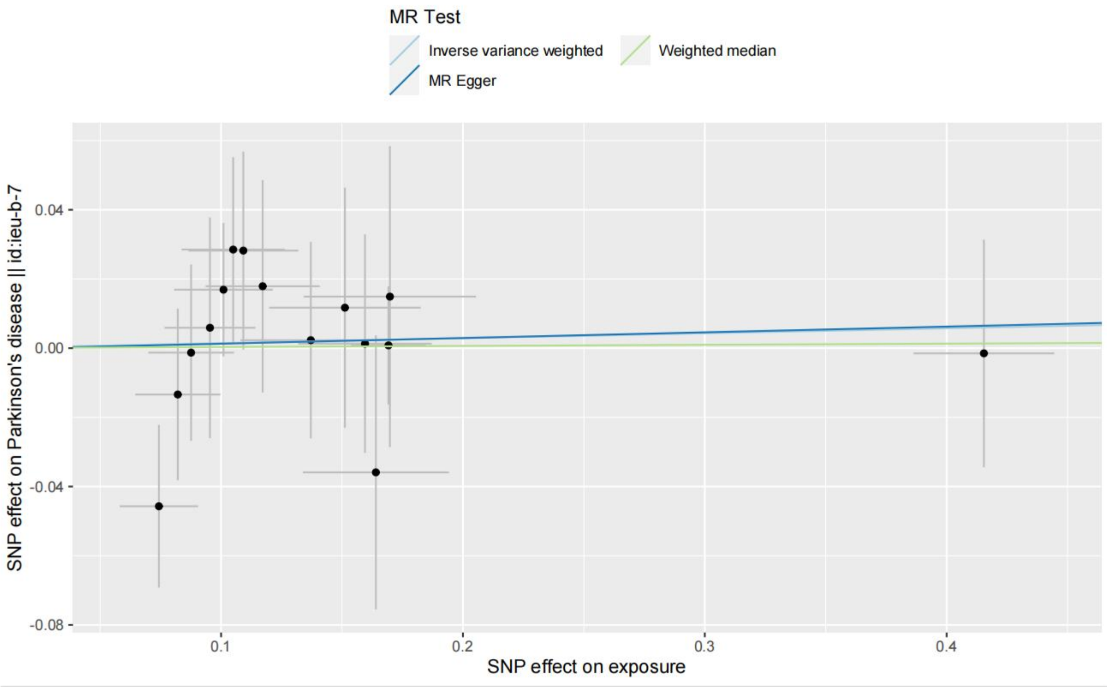

A. Scatter plot of EOTAXIN with risk of PD

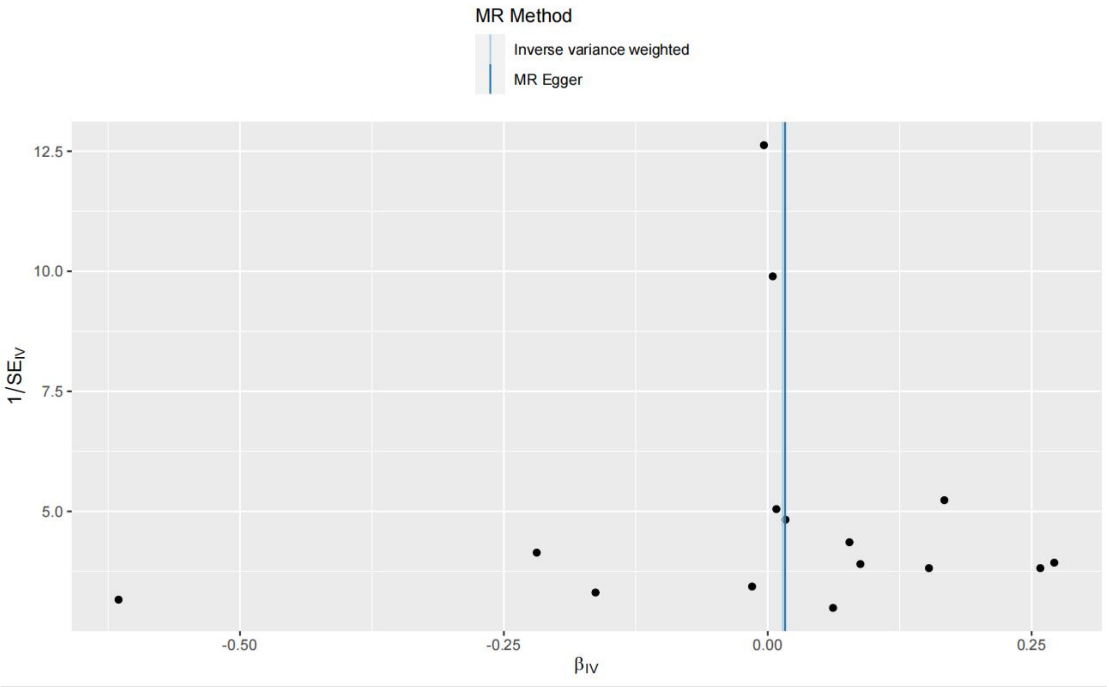

B. Funnel plot of EOTAXIN instruments strength on PD

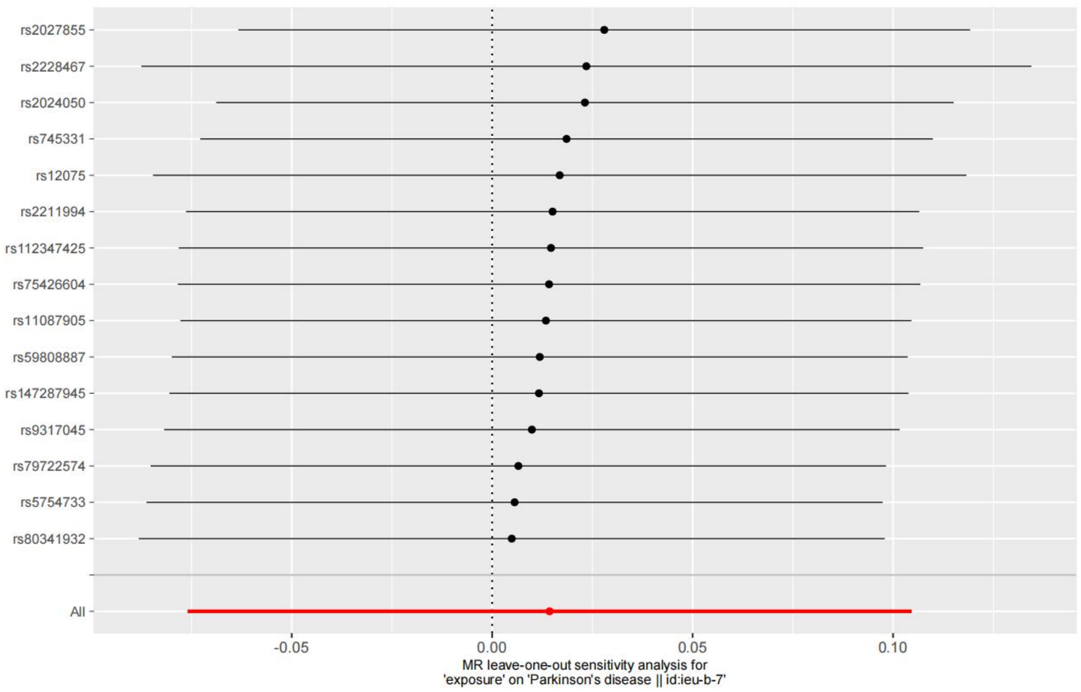

C. MR leave-one-out sensitivity analysis for EOTAXIN on PD

eFigure 45. bFGF-associated SNPs with risk of PD

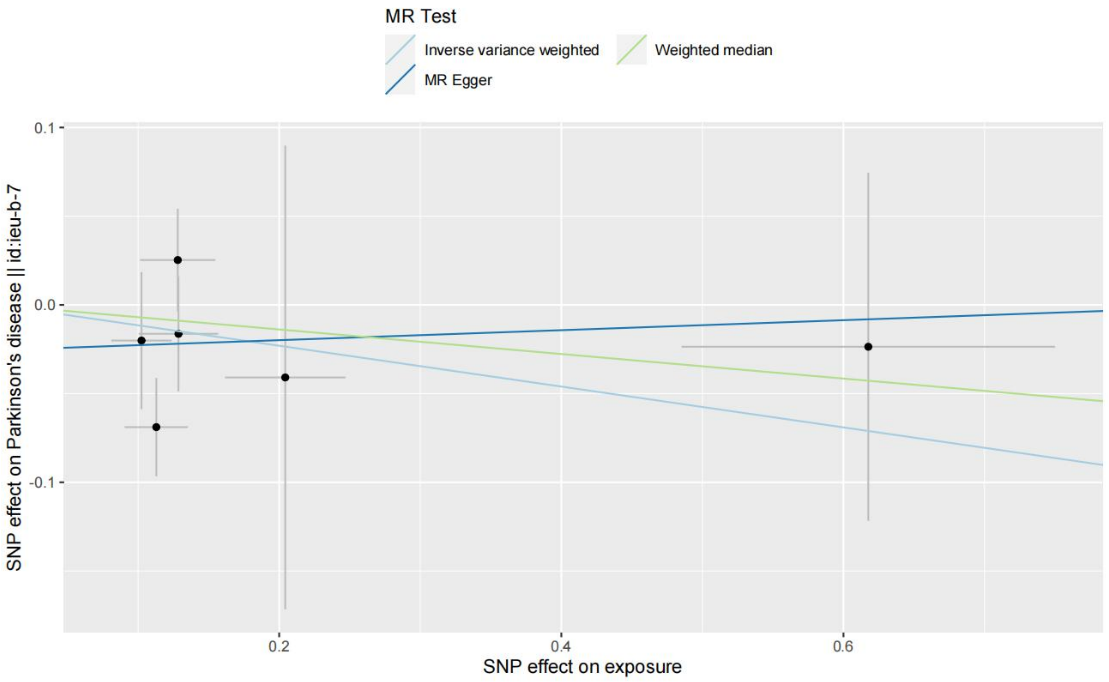

A. Scatter plot of bFGF with risk of PD

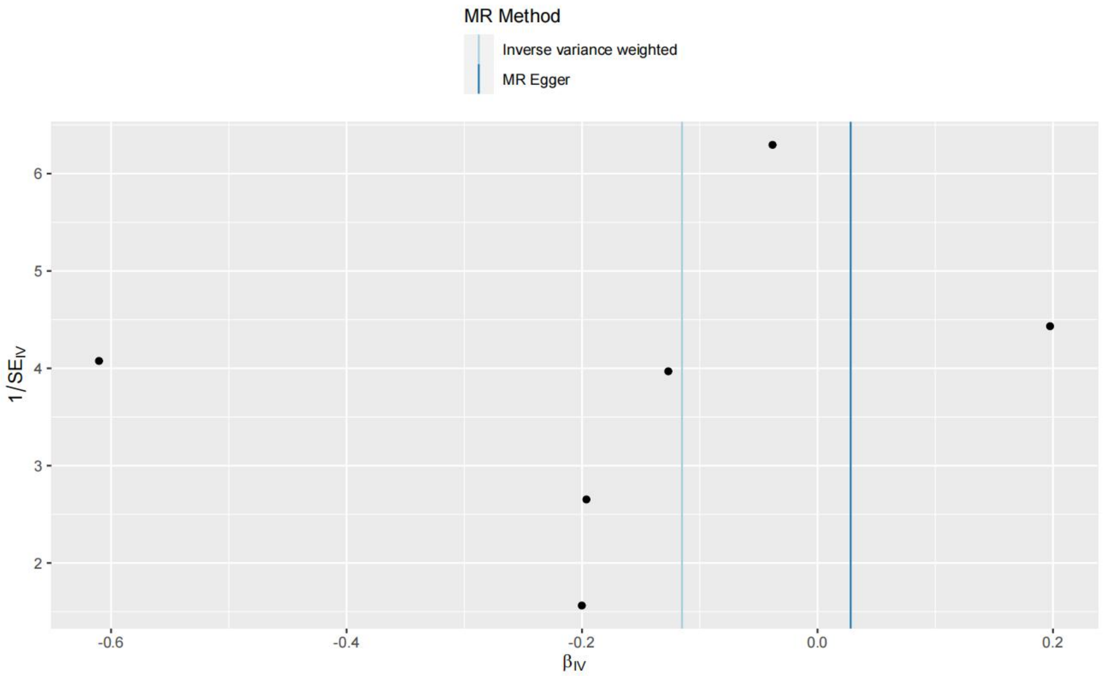

B. Funnel plot of bFGF instruments strength on PD

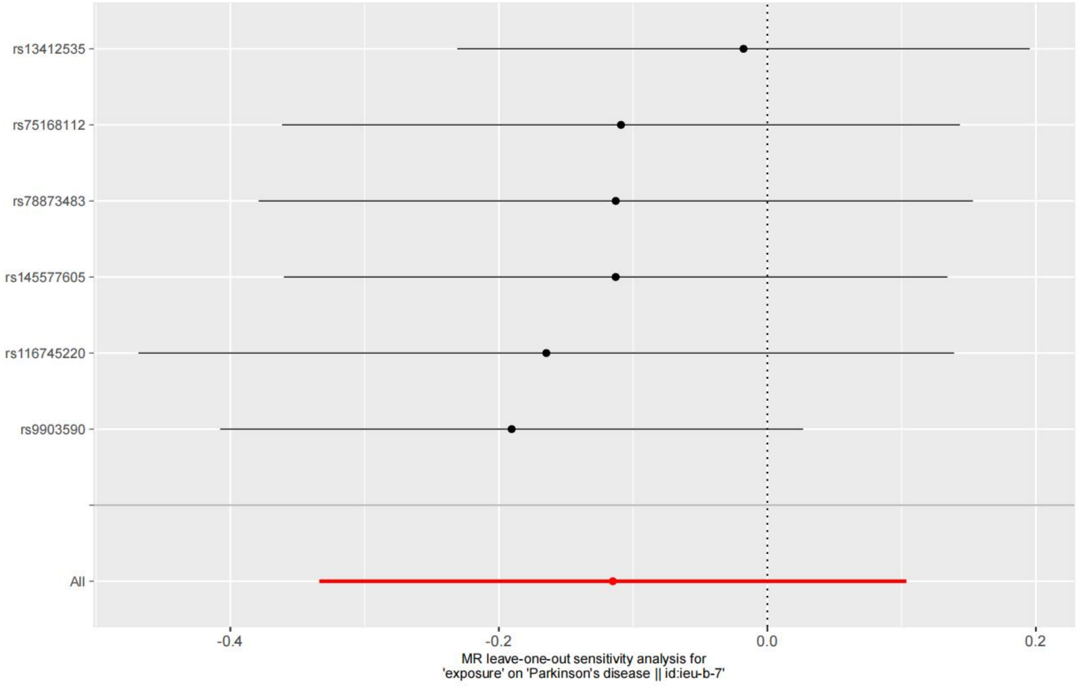

C. MR leave-one-out sensitivity analysis for bFGF on PD

eFigure 46. G-CSF-associated SNPs with risk of PD

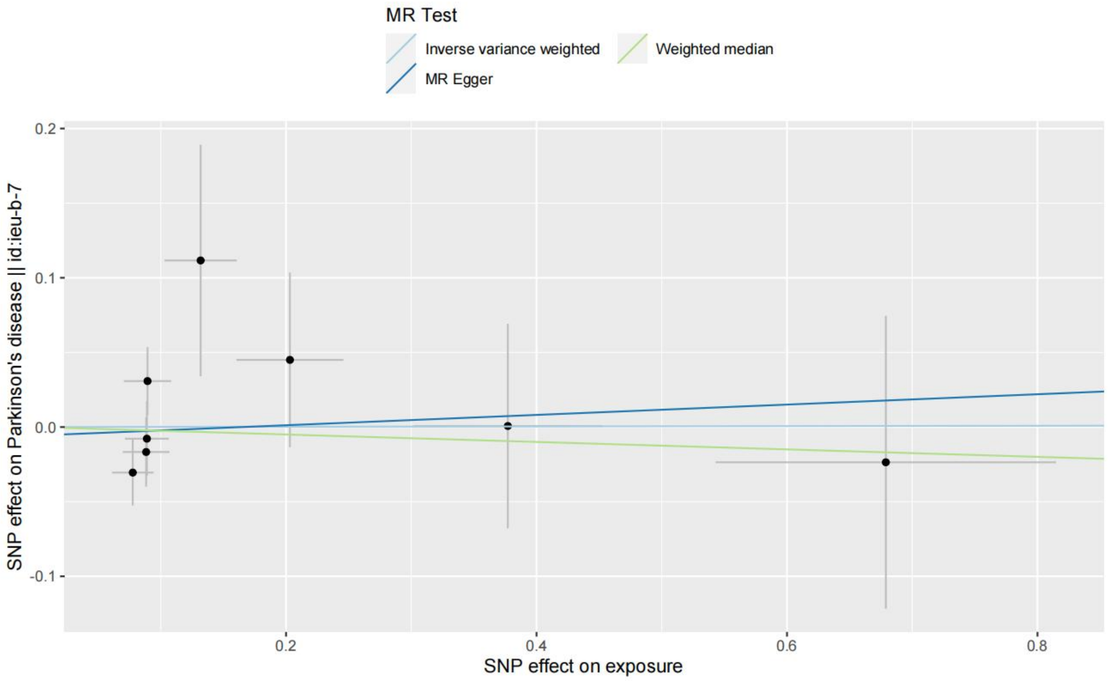

A. Scatter plot of G-CSF with risk of PD

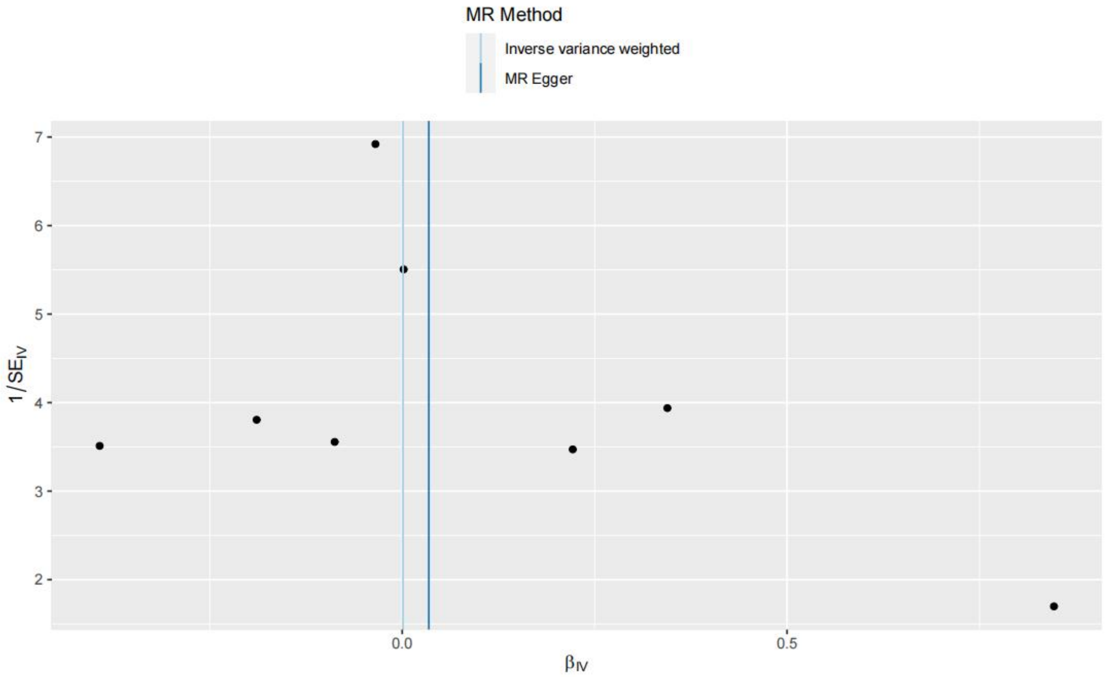

B. Funnel plot of G-CSF instruments strength on PD

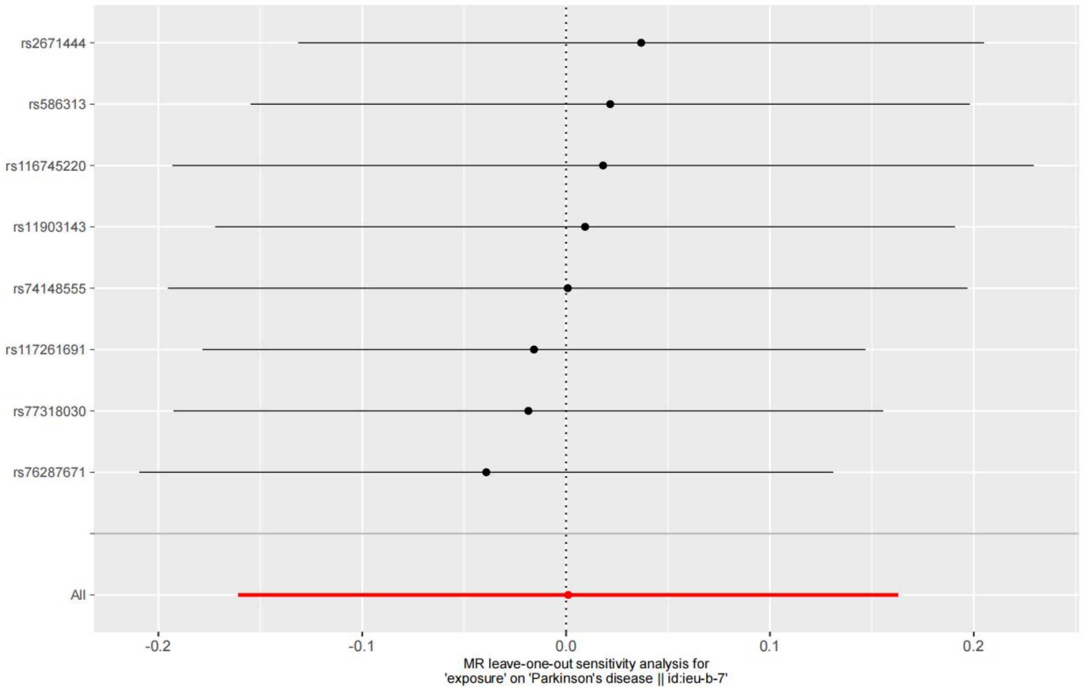

C. MR leave-one-out sensitivity analysis for G-CSF on PD

eFigure 47. GROA-associated SNPs with risk of PD

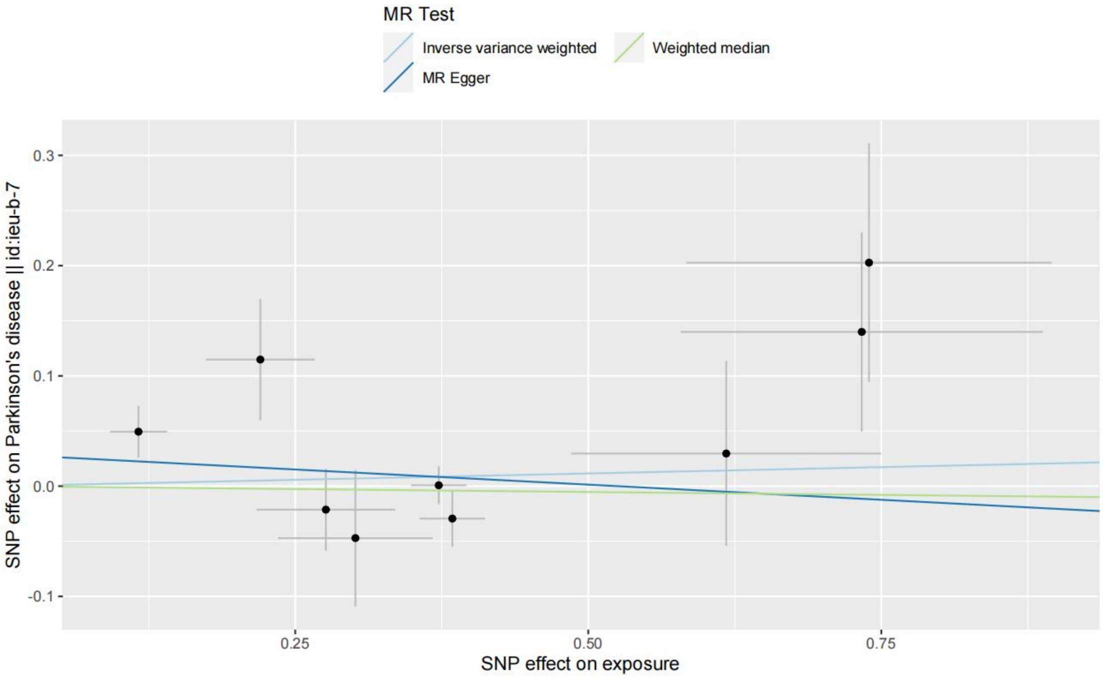

A. Scatter plot of GROA with risk of PD

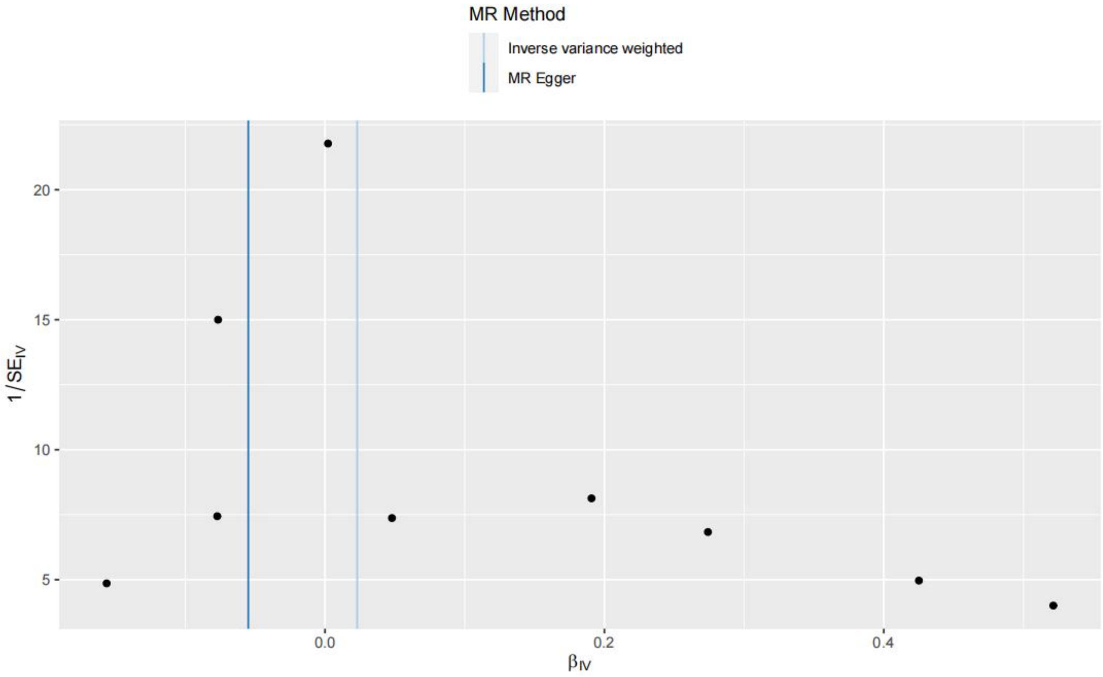

B. Funnel plot of GROA instruments strength on PD

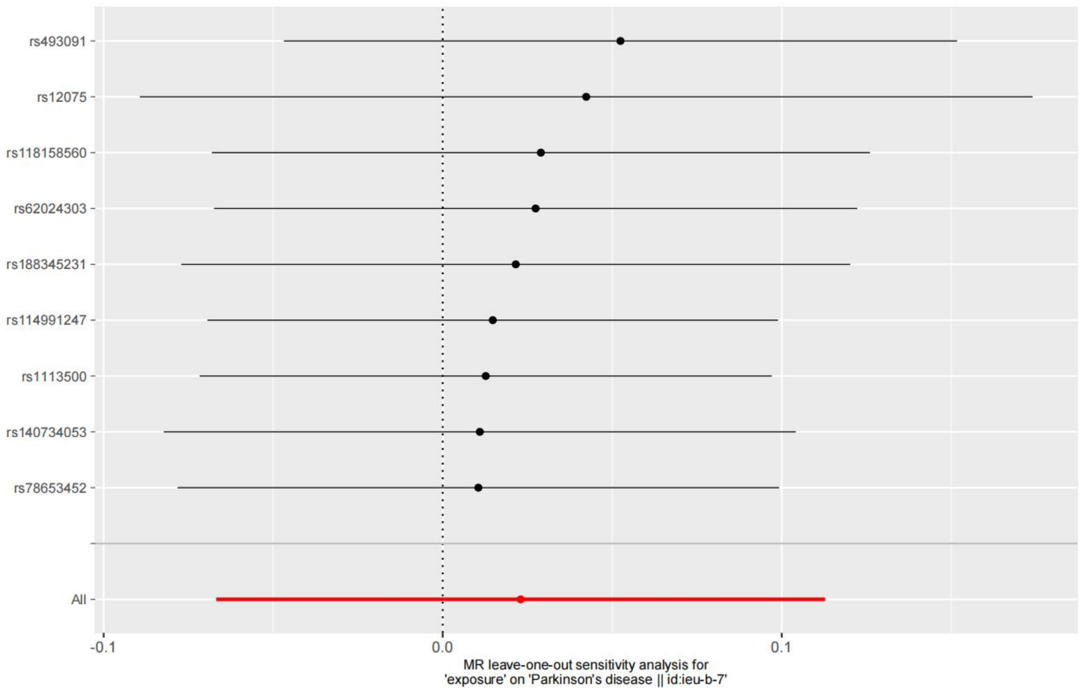

C. MR leave-one-out sensitivity analysis for GROA on PD

eFigure 48. HGF-associated SNPs with risk of PD

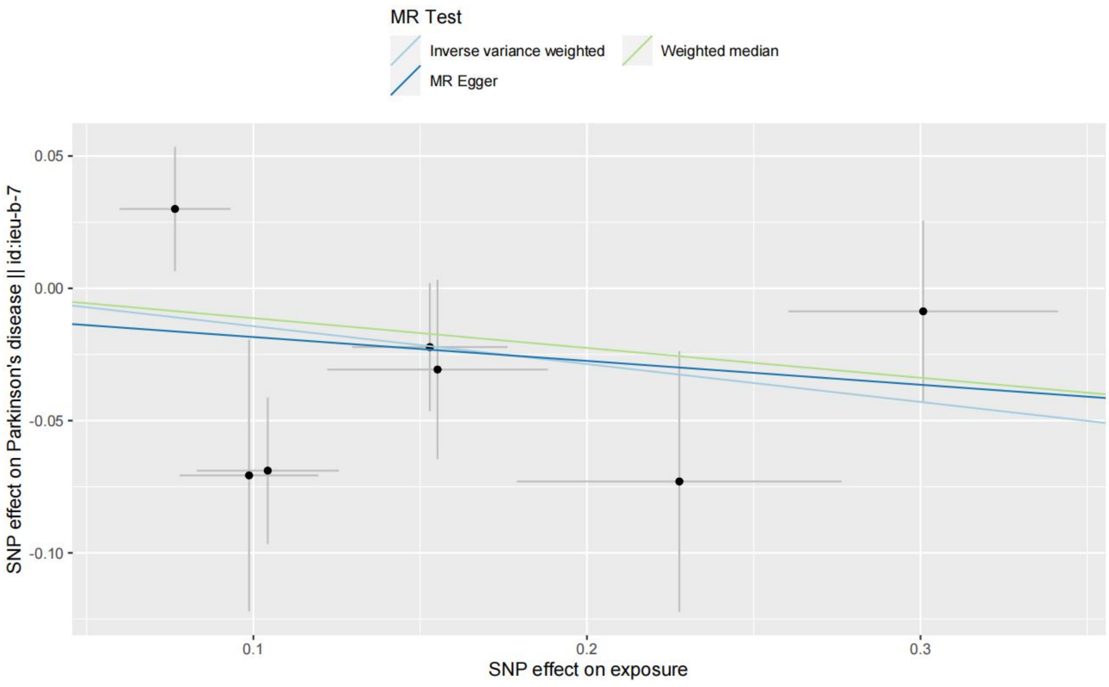

A. Scatter plot of HGF with risk of PD

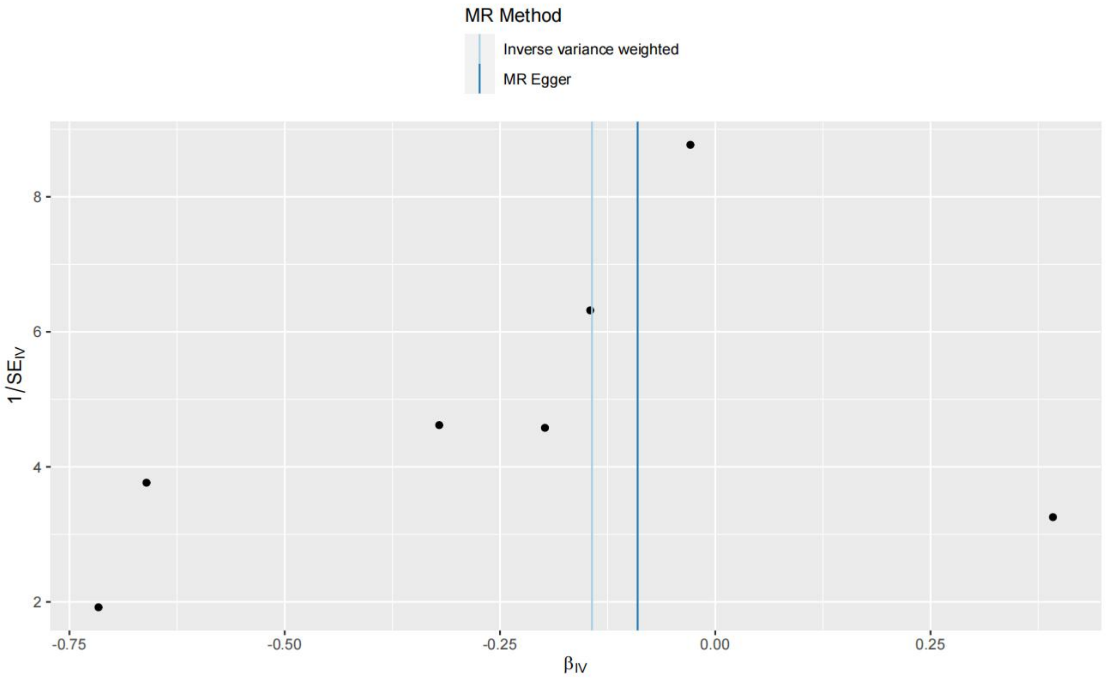

B. Funnel plot of HGF instruments strength on PD

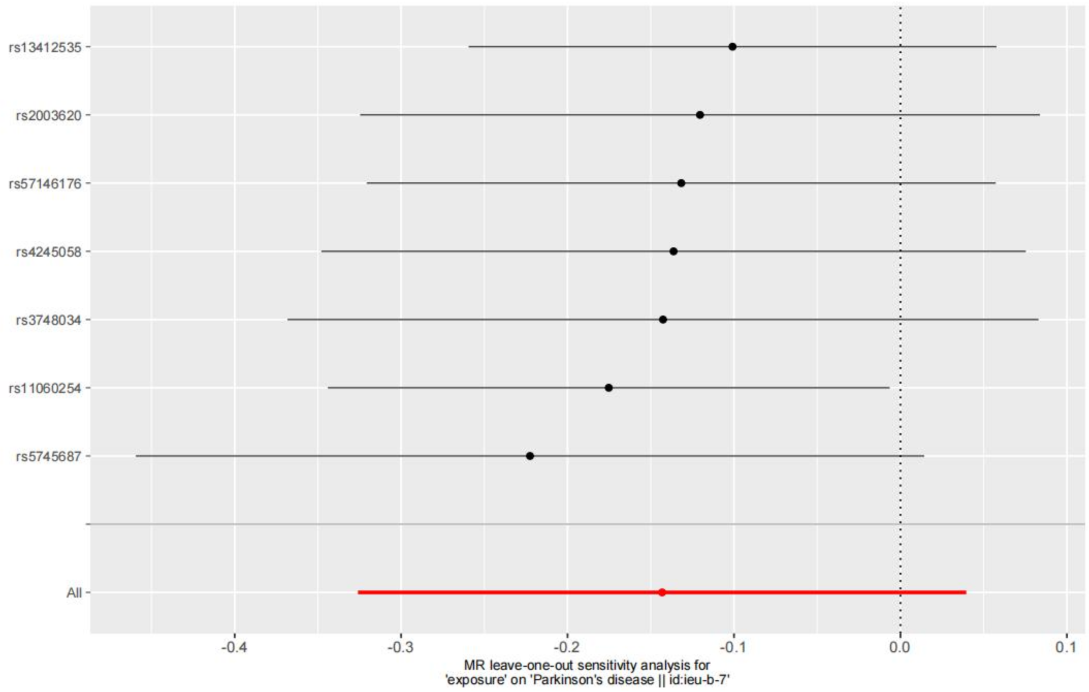

C. MR leave-one-out sensitivity analysis for HGF on PD

eFigure 49. IFN-G-associated SNPs with risk of PD

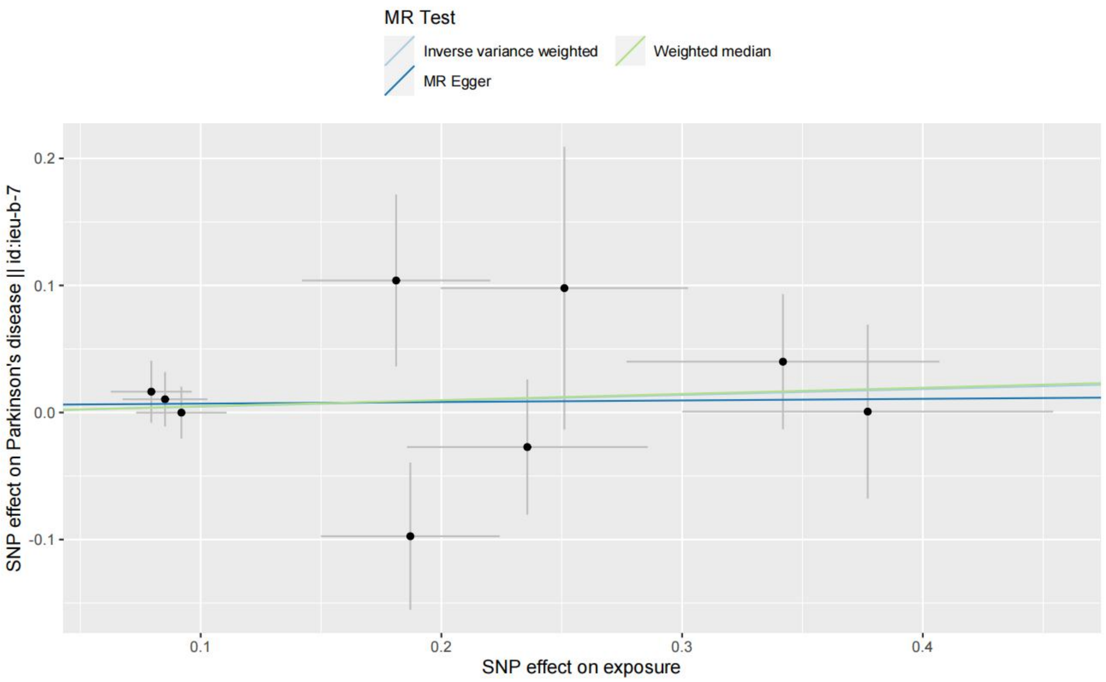

A. Scatter plot of IFN-G with risk of PD

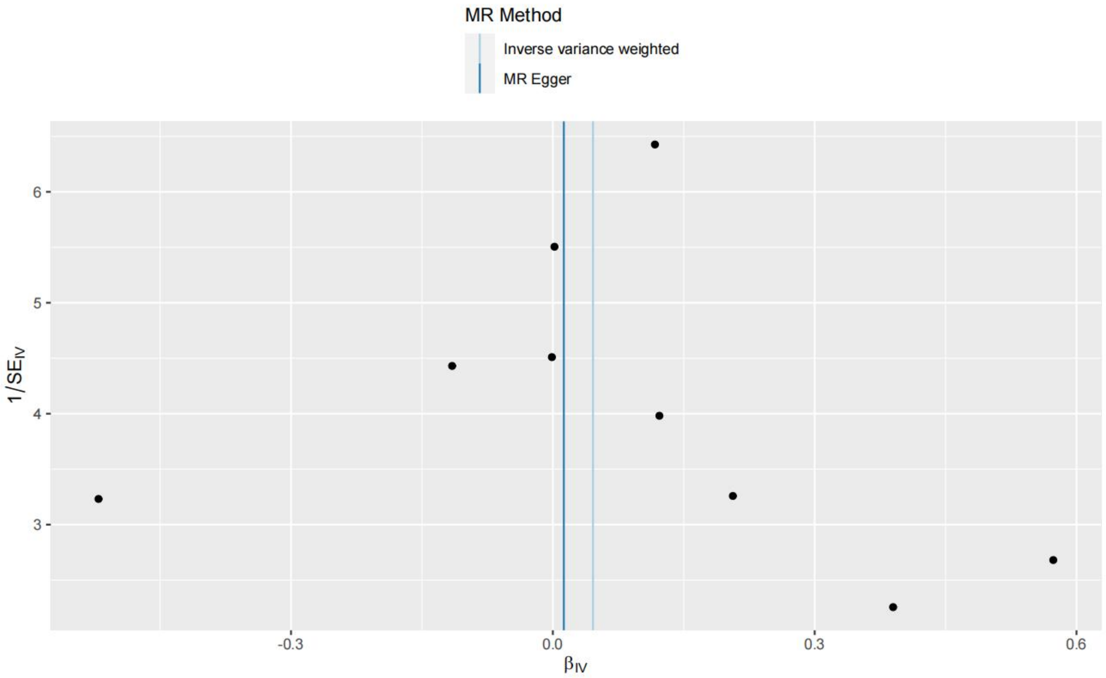

B. Funnel plot of IFN-G instruments strength on PD

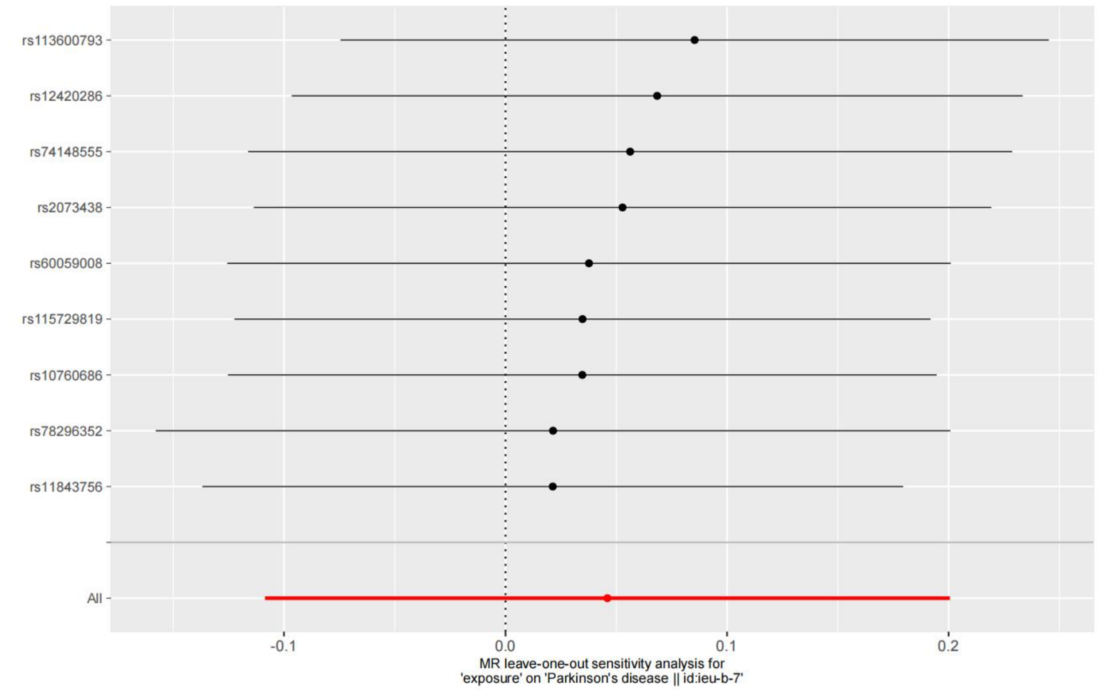

C. MR leave-one-out sensitivity analysis for IFN-G on PD

eFigure 50. IL-1B-associated SNPs with risk of PD

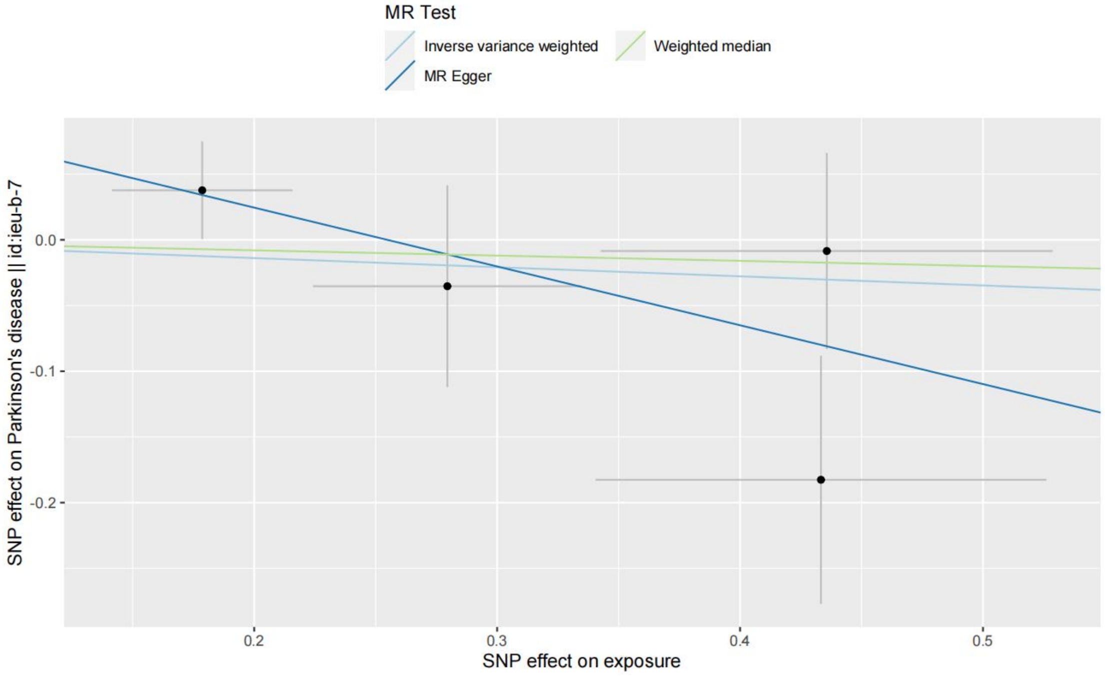

A. Scatter plot of IL-1B with risk of PD

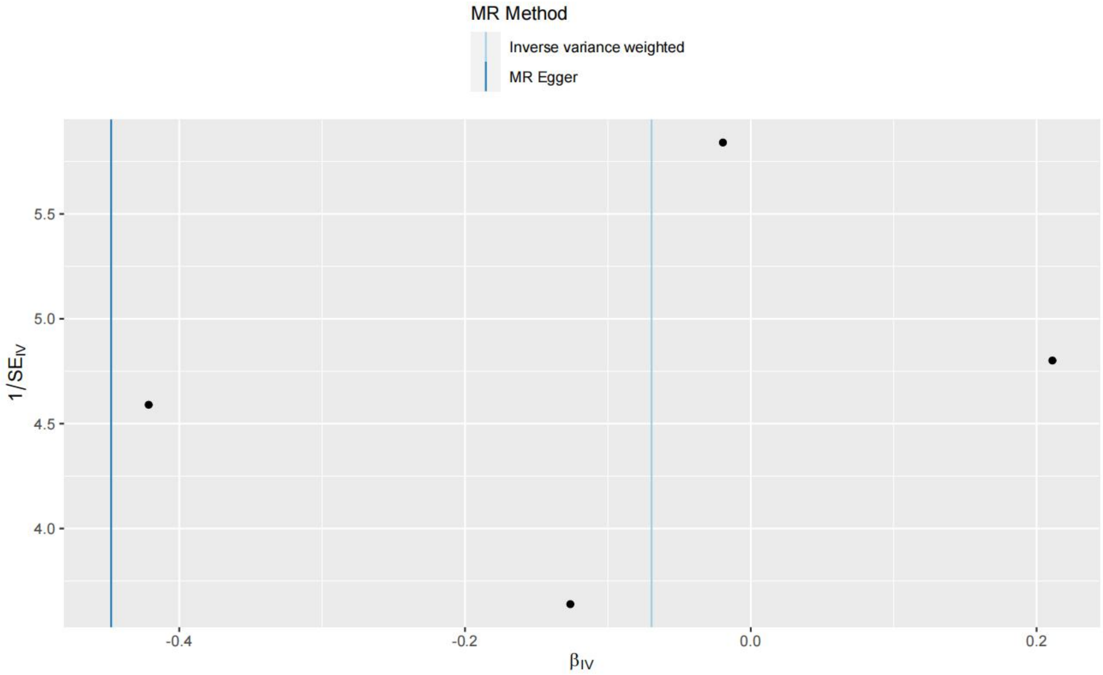

B. Funnel plot of IL-1B instruments strength on PD

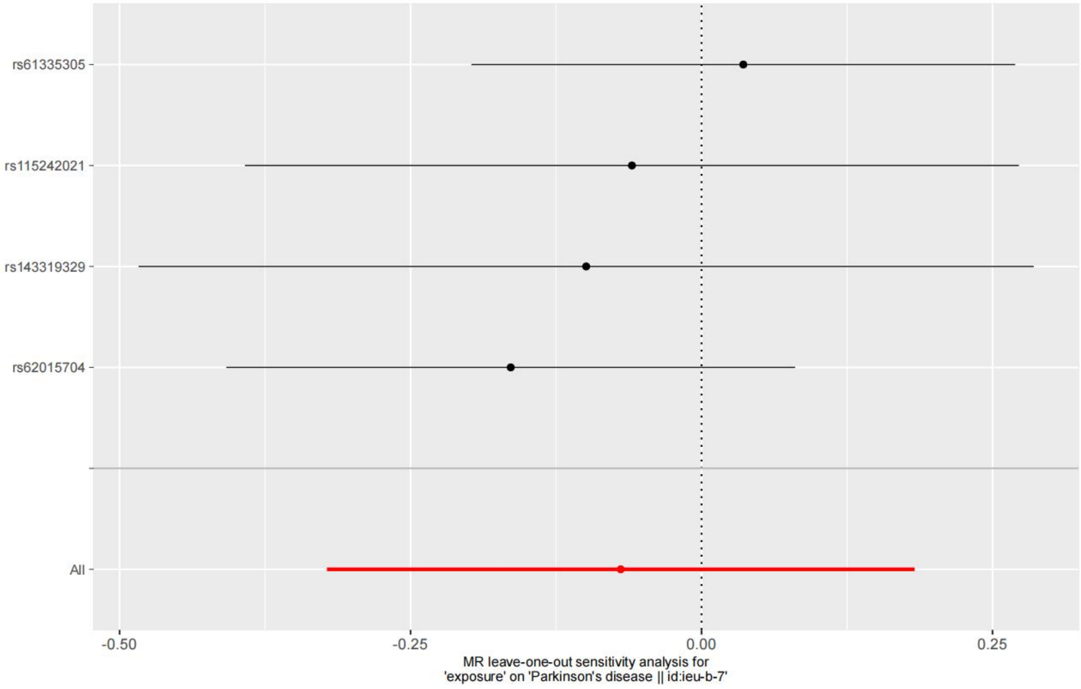

C. MR leave-one-out sensitivity analysis for IL-1B on PD

eFigure 51. IL-1RA-associated SNPs with risk of PD

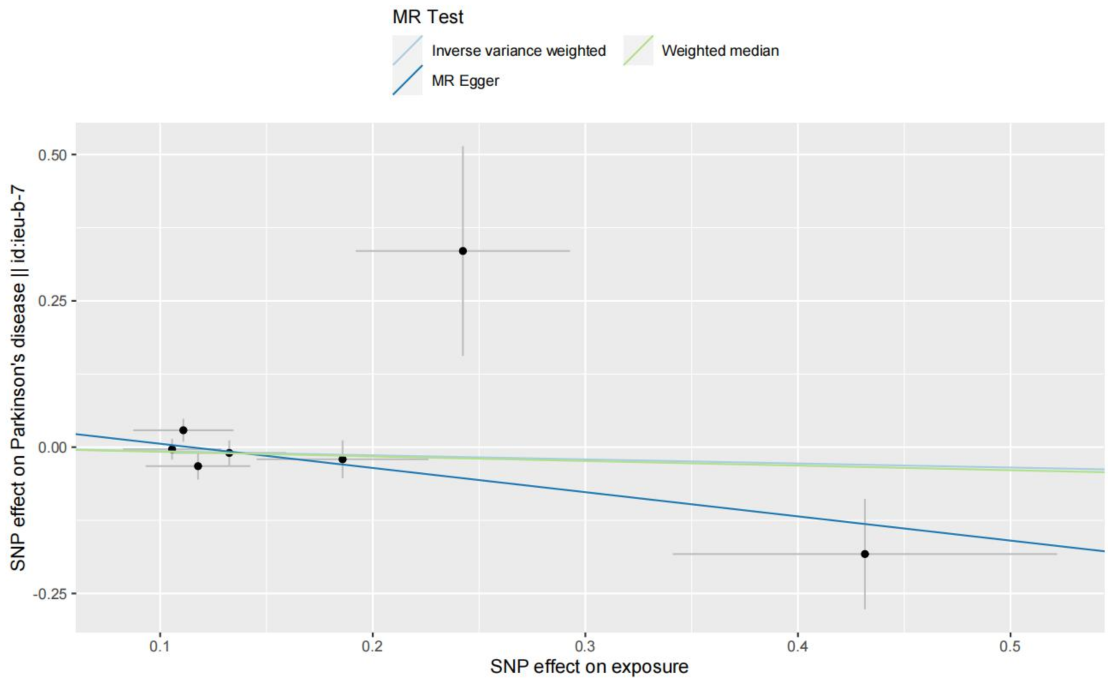

A. Scatter plot of IL-1RA with risk of PD

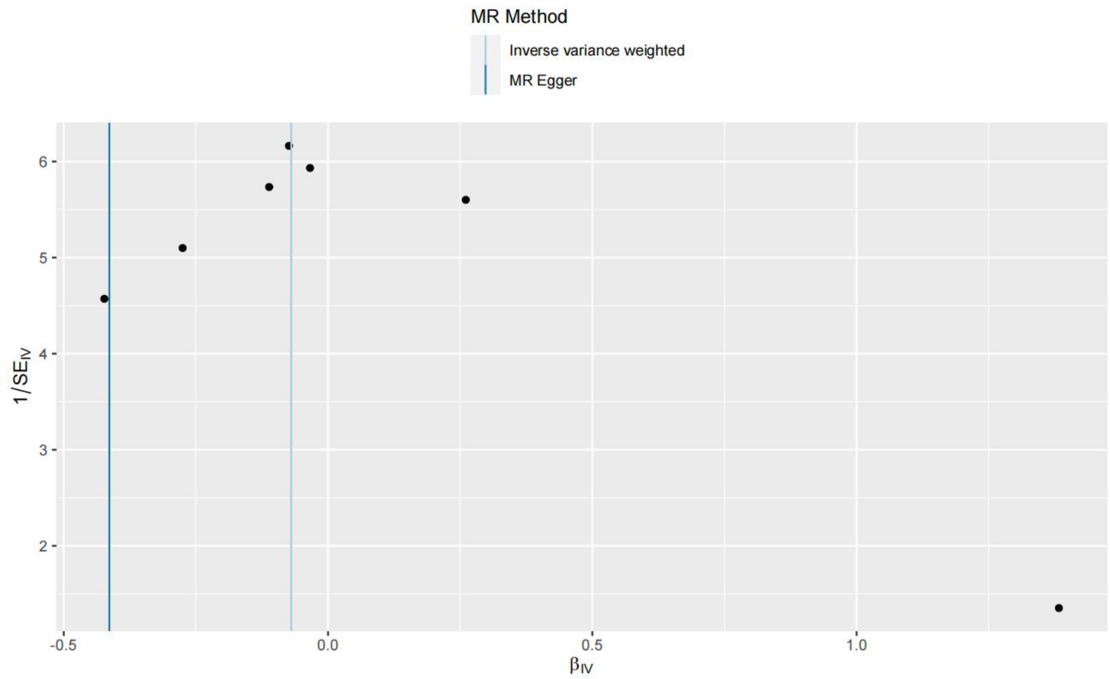

B. Funnel plot of IL-1RA instruments strength on PD

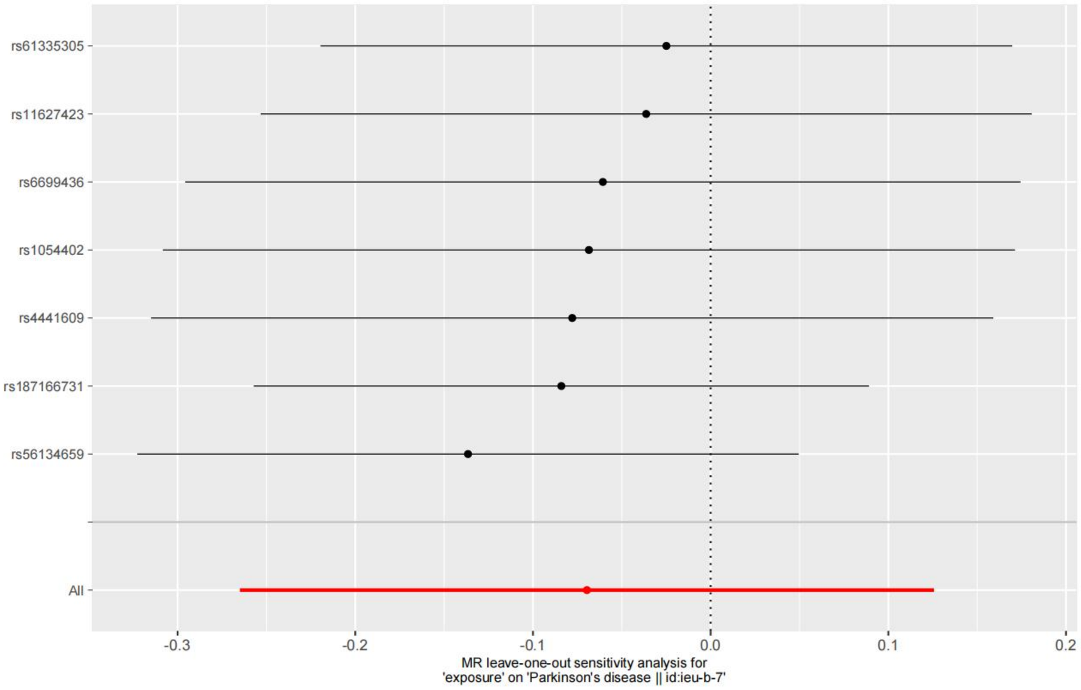

C. MR leave-one-out sensitivity analysis for IL-1RA on PD

eFigure 52. IL-2-associated SNPs with risk of PD

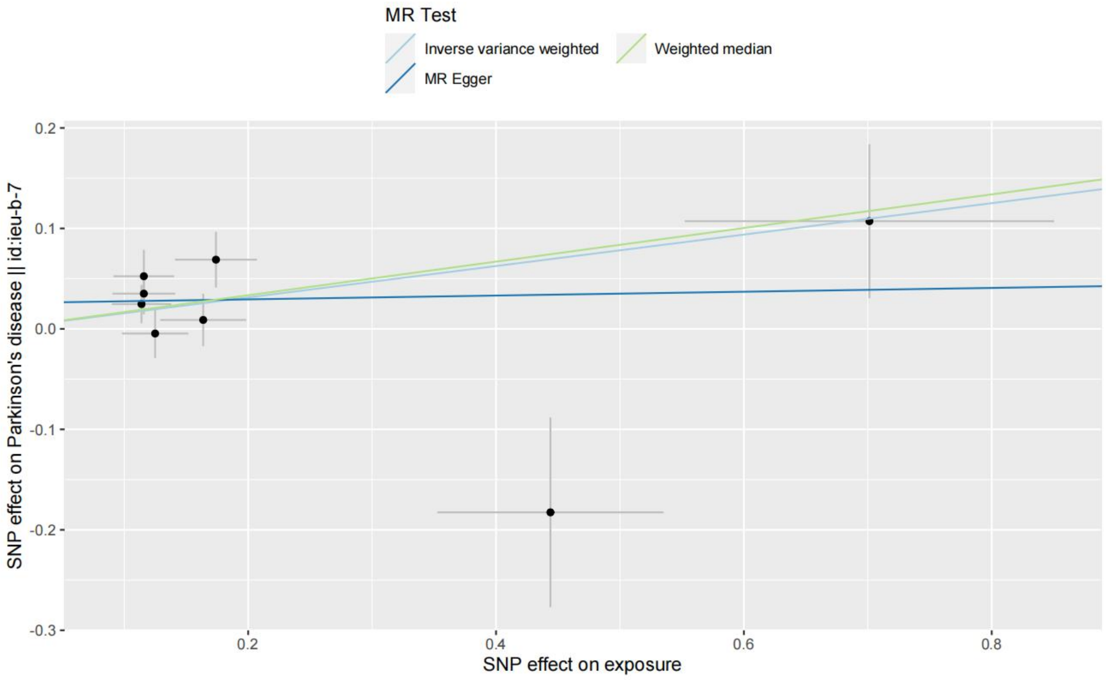

A. Scatter plot of IL-2 with risk of PD

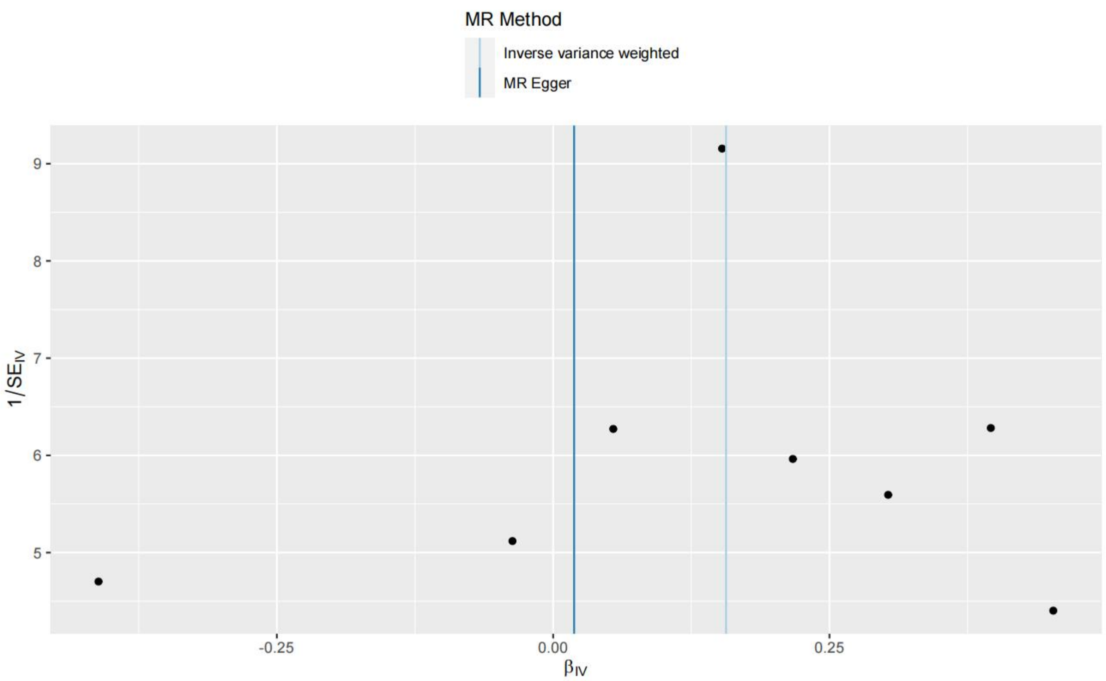

B. Funnel plot of IL-2 instruments strength on PD

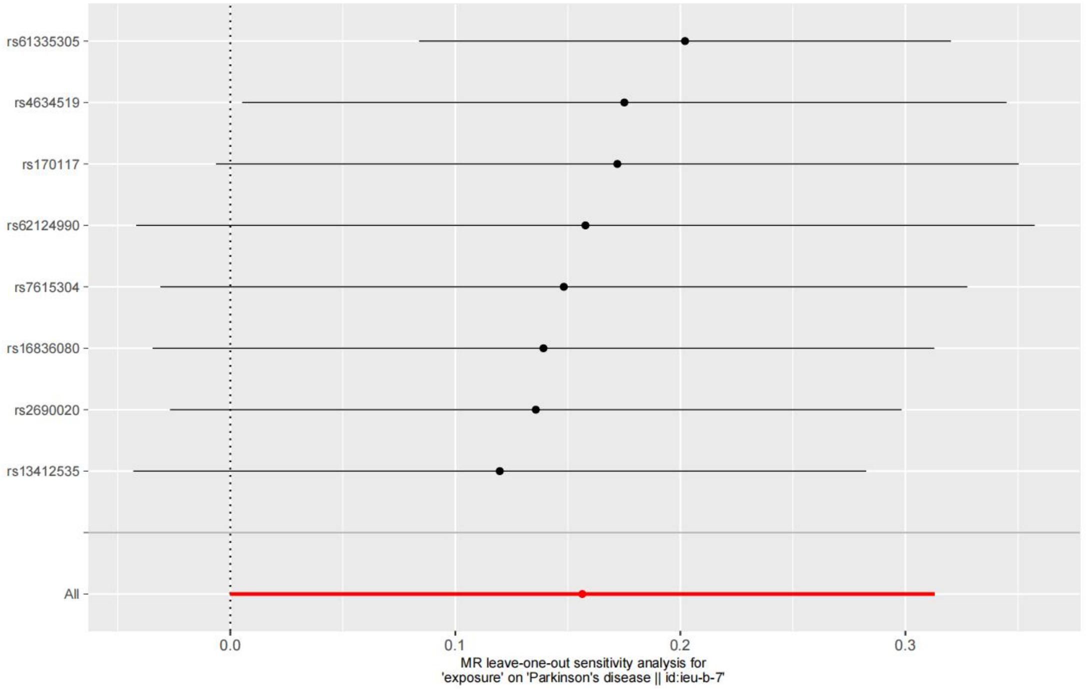

C. MR leave-one-out sensitivity analysis for IL-2 on PD

eFigure 53. IL-2RA-associated SNPs with risk of PD

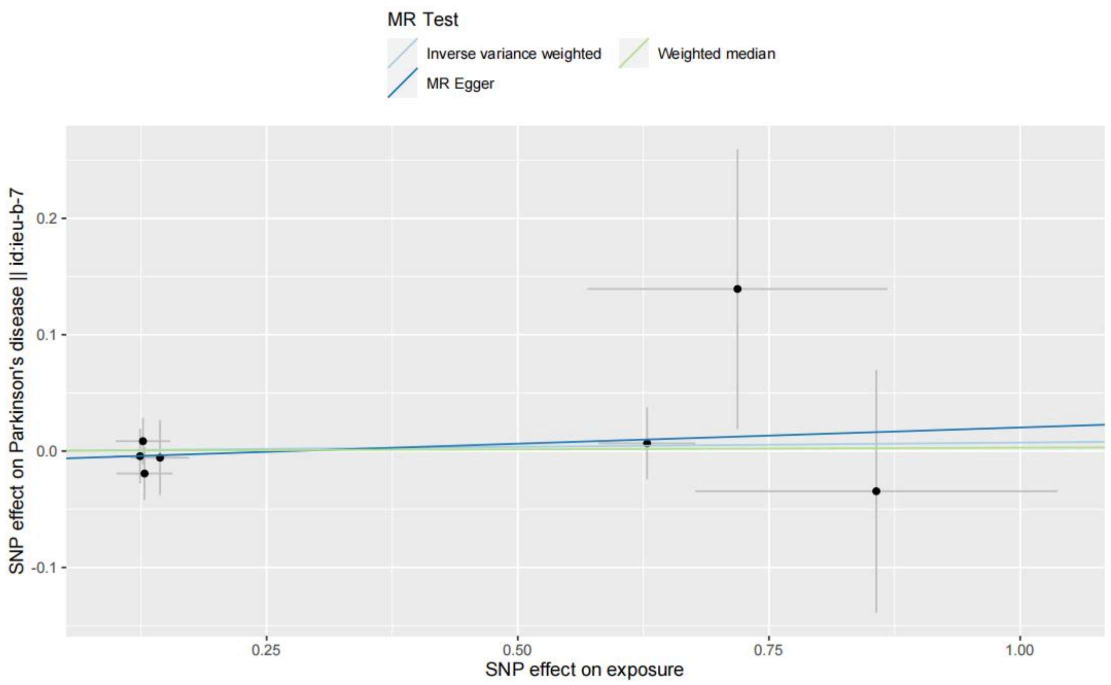

A. Scatter plot of IL-2RA with risk of PD

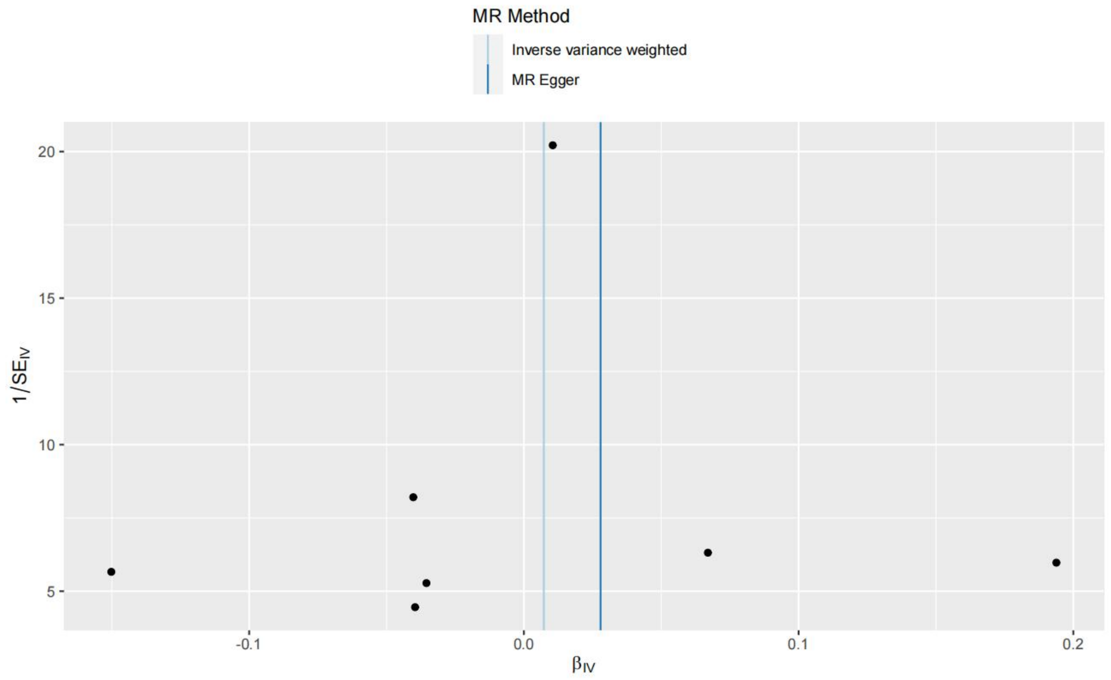

B. Funnel plot of IL-2RA instruments strength on PD

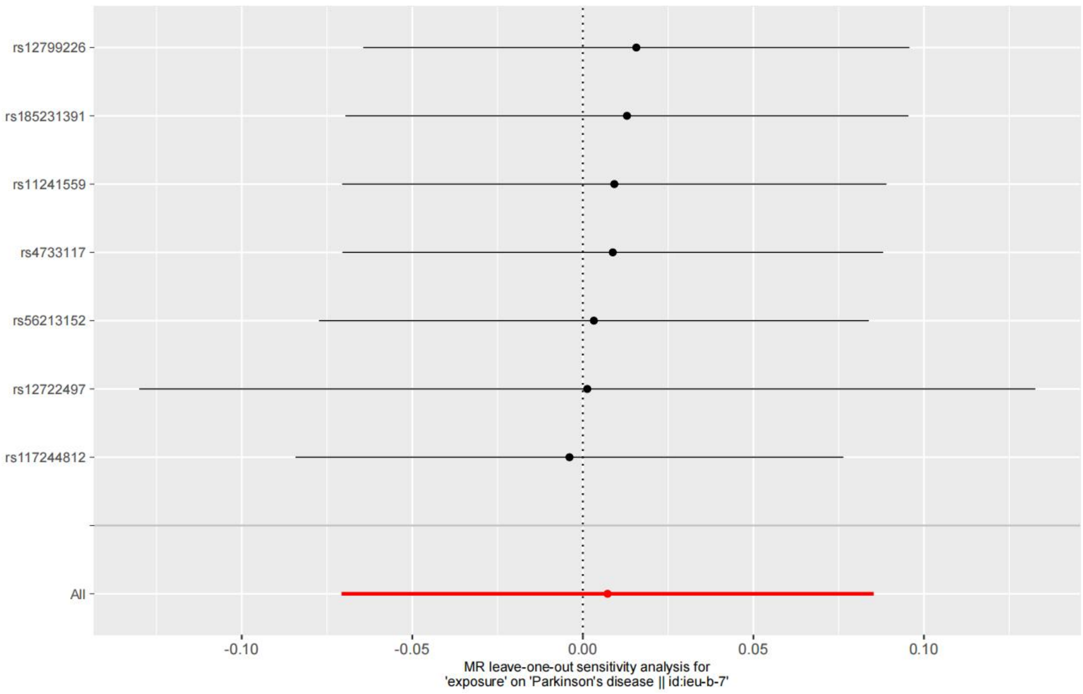

C. MR leave-one-out sensitivity analysis for IL-2RA on PD

eFigure 54. IL-4-associated SNPs with risk of PD

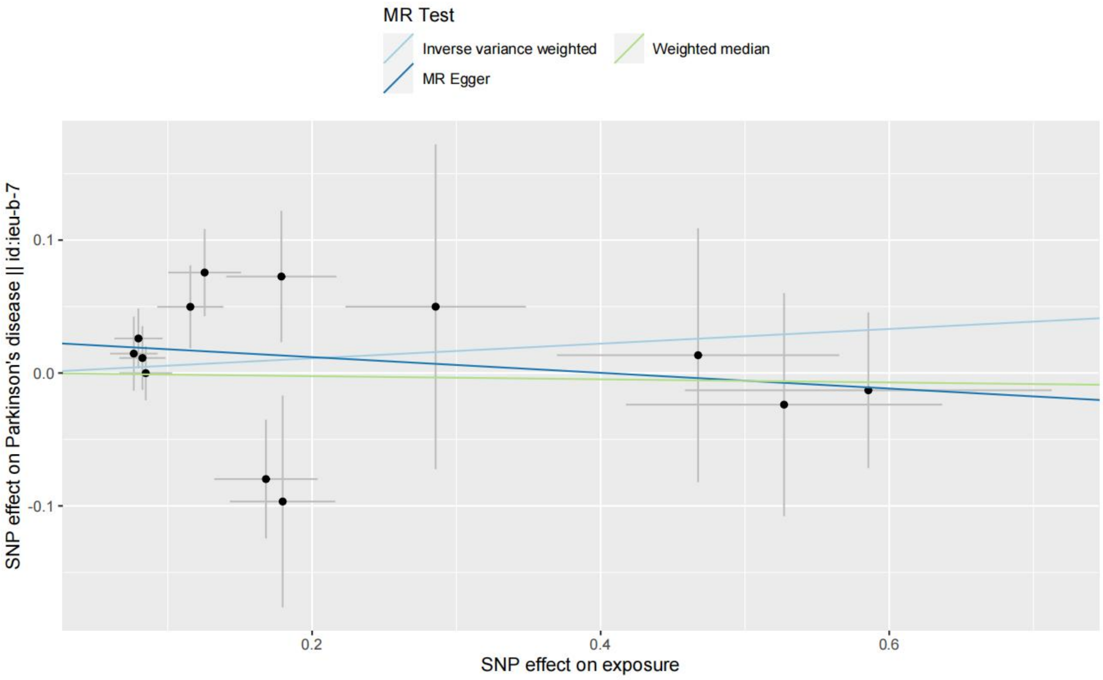

A. Scatter plot of IL-4 with risk of PD

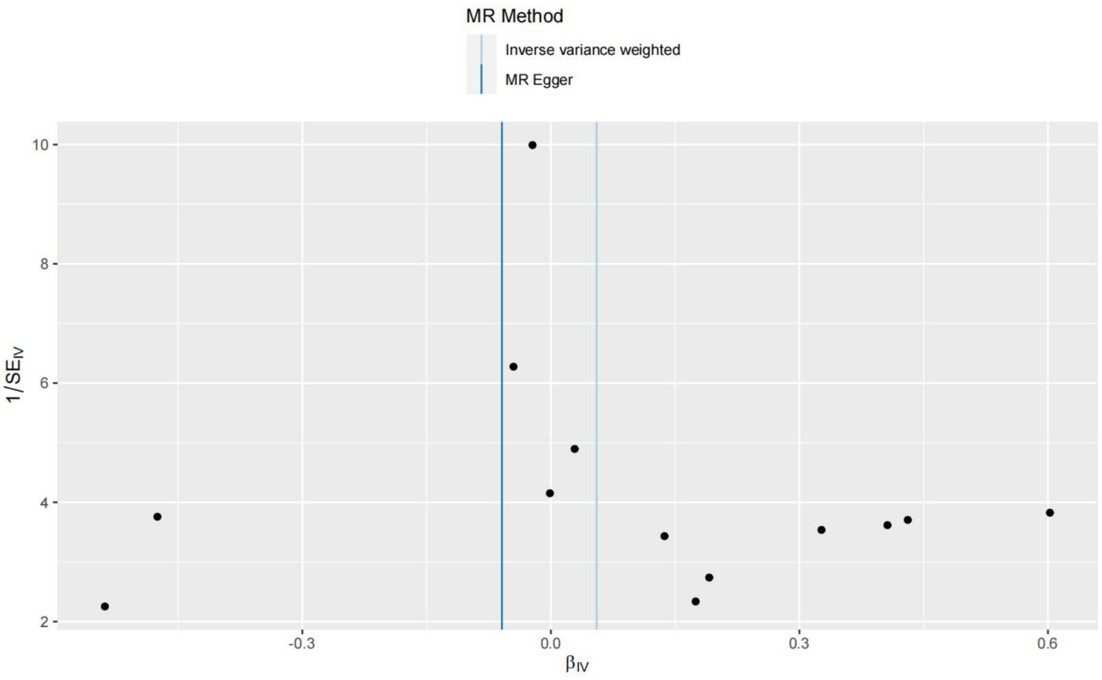

B. Funnel plot of IL-4 instruments strength on PD

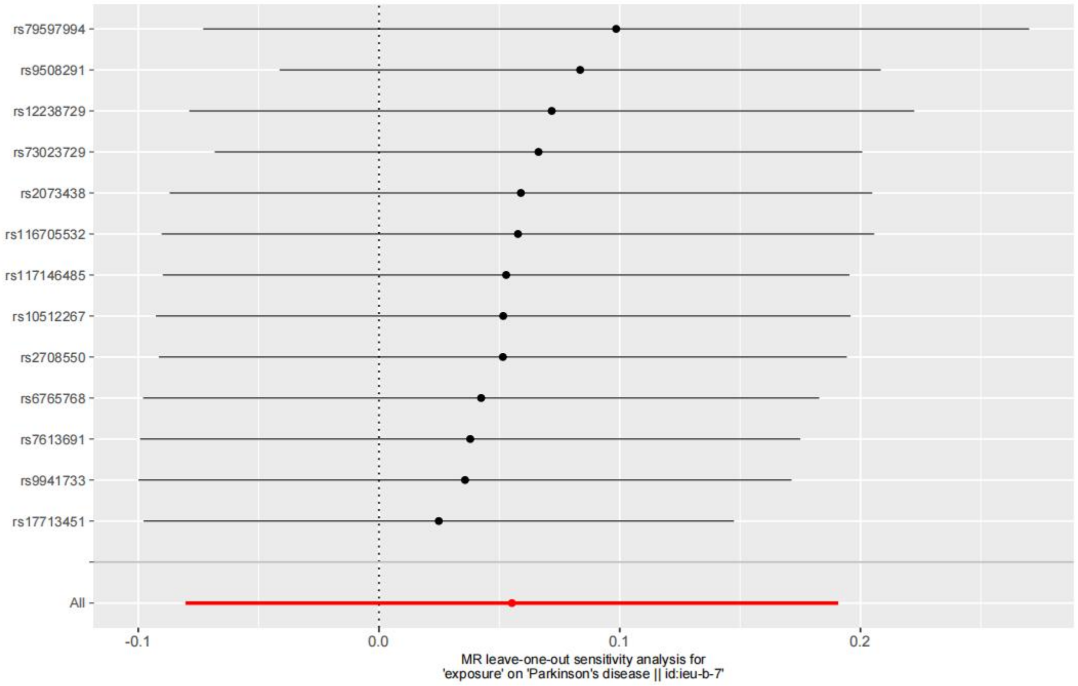

C. MR leave-one-out sensitivity analysis for IL-4 on PD

eFigure 55. IL-5-associated SNPs with risk of PD

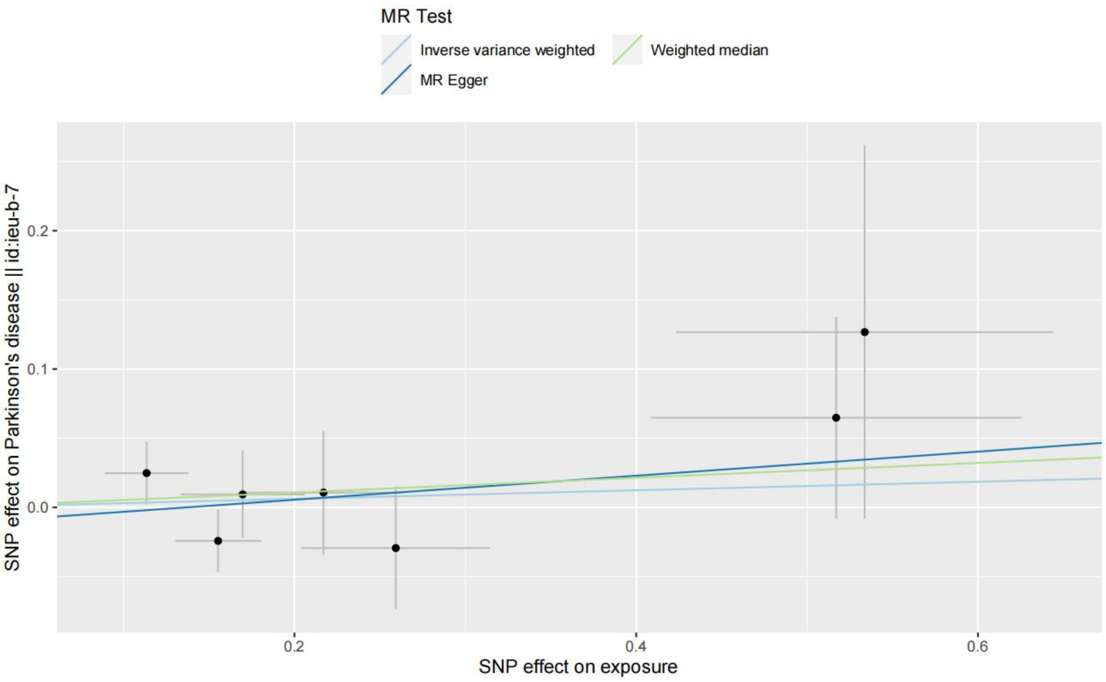

A. Scatter plot of IL-5 with risk of PD

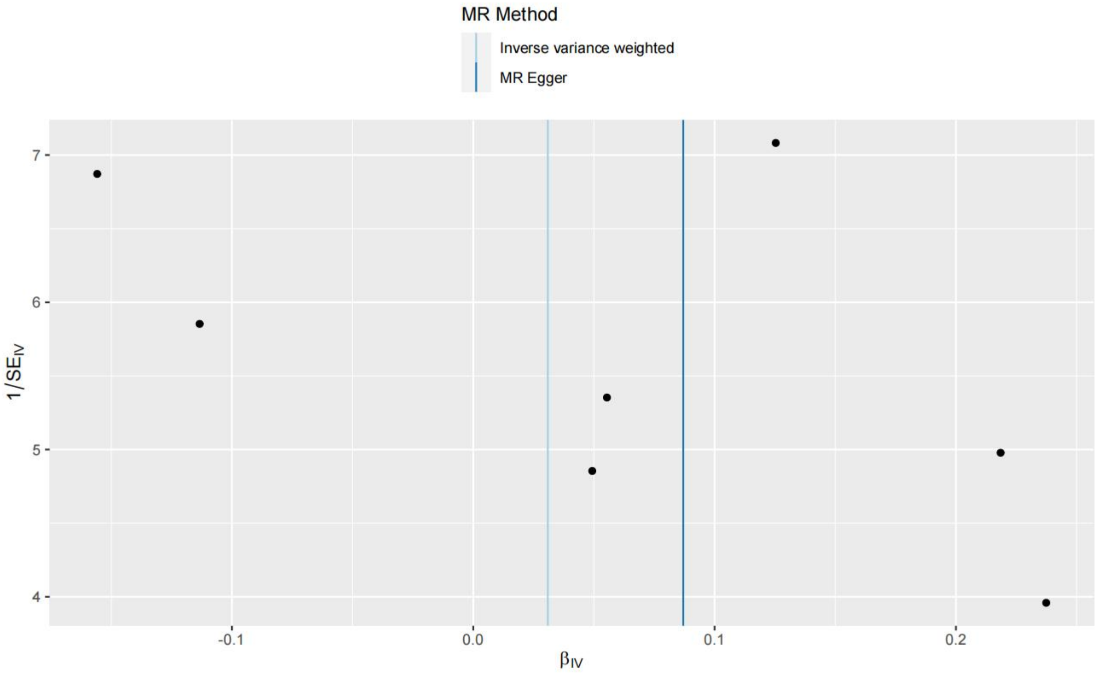

B. Funnel plot of IL-5 instruments strength on PD

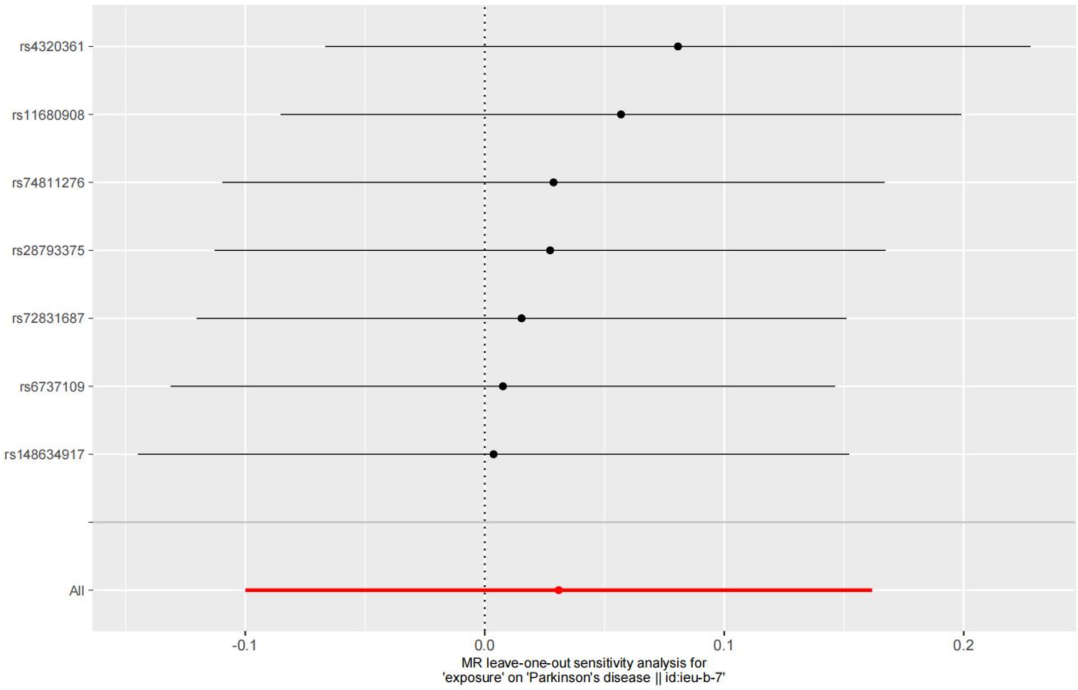

C. MR leave-one-out sensitivity analysis for IL-5 on PD

eFigure 56. IL-6-associated SNPs with risk of PD

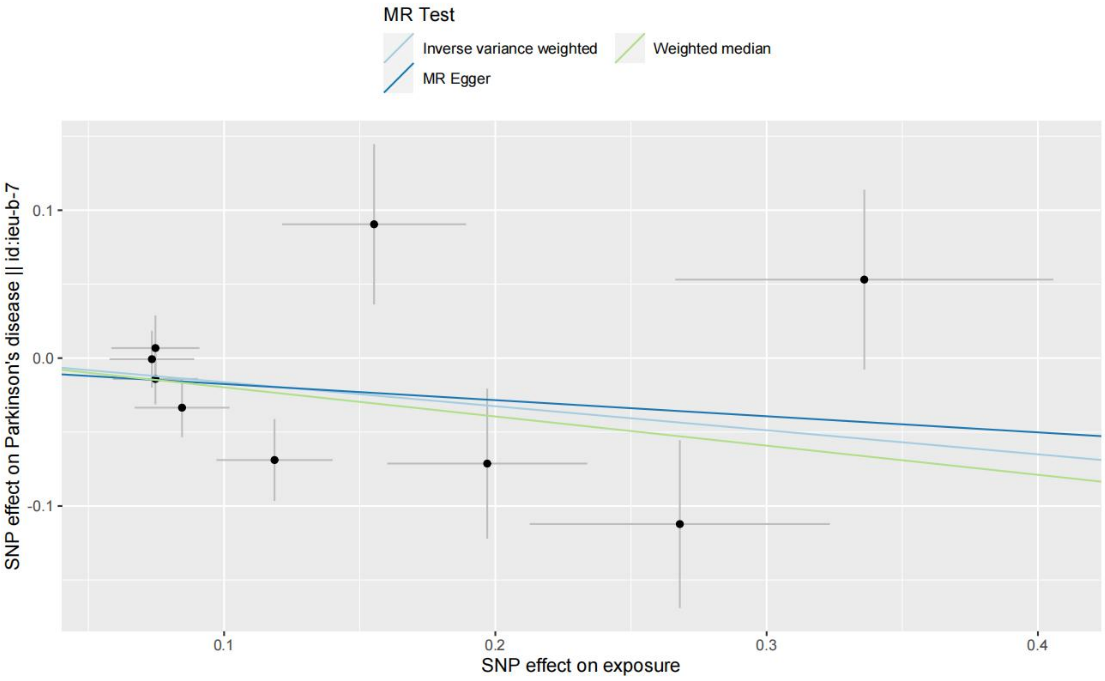

A. Scatter plot of IL-6 with risk of PD

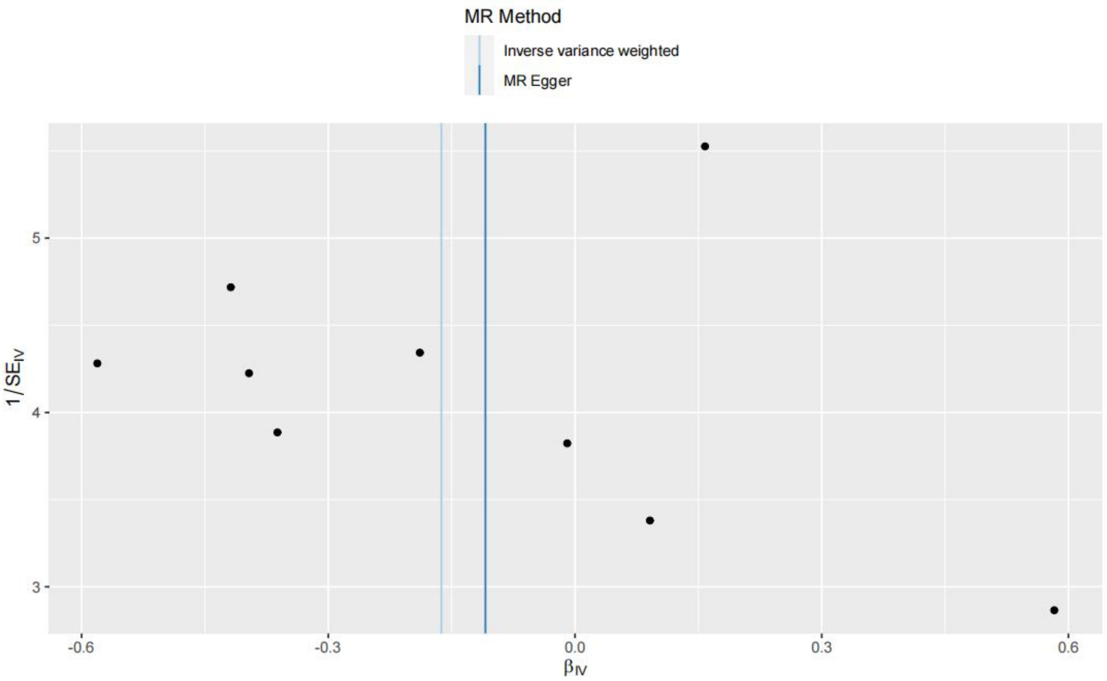

B. Funnel plot of IL-6 instruments strength on PD

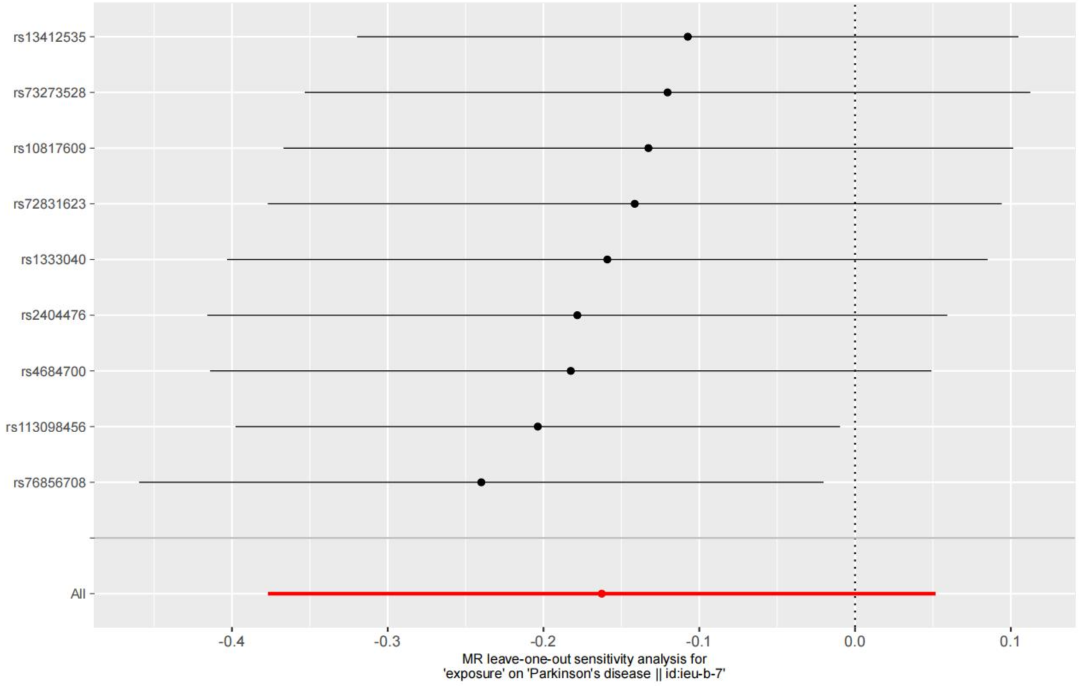

C. MR leave-one-out sensitivity analysis for IL-6 on PD

eFigure 57. IL-7-associated SNPs with risk of PD

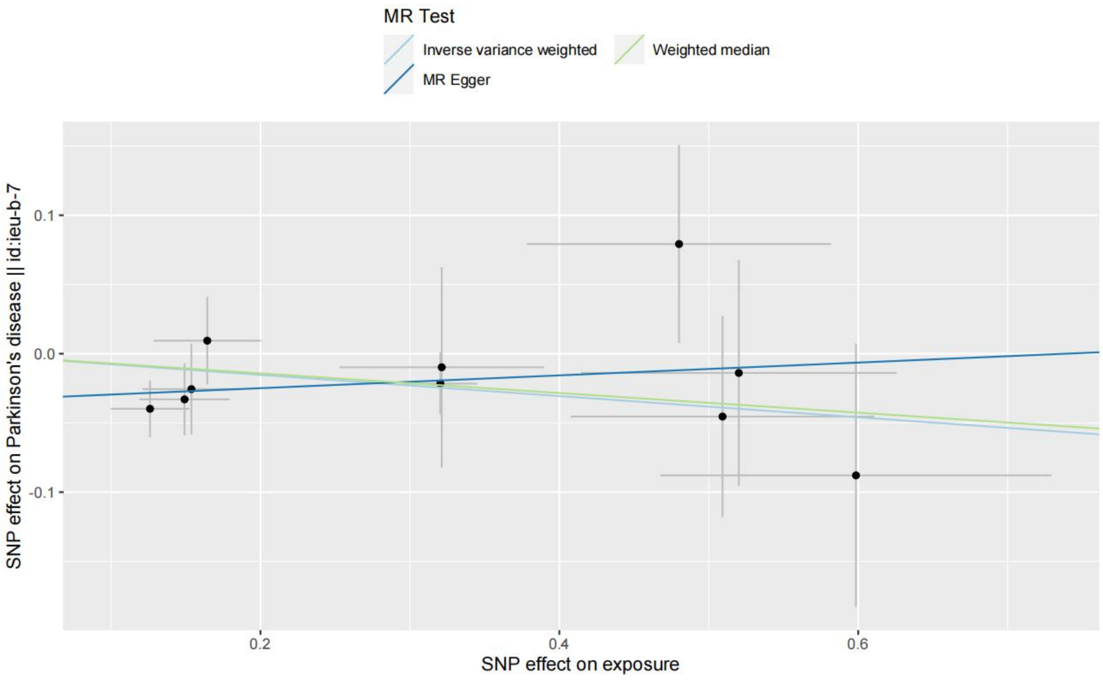

A. Scatter plot of IL-7 with risk of PD

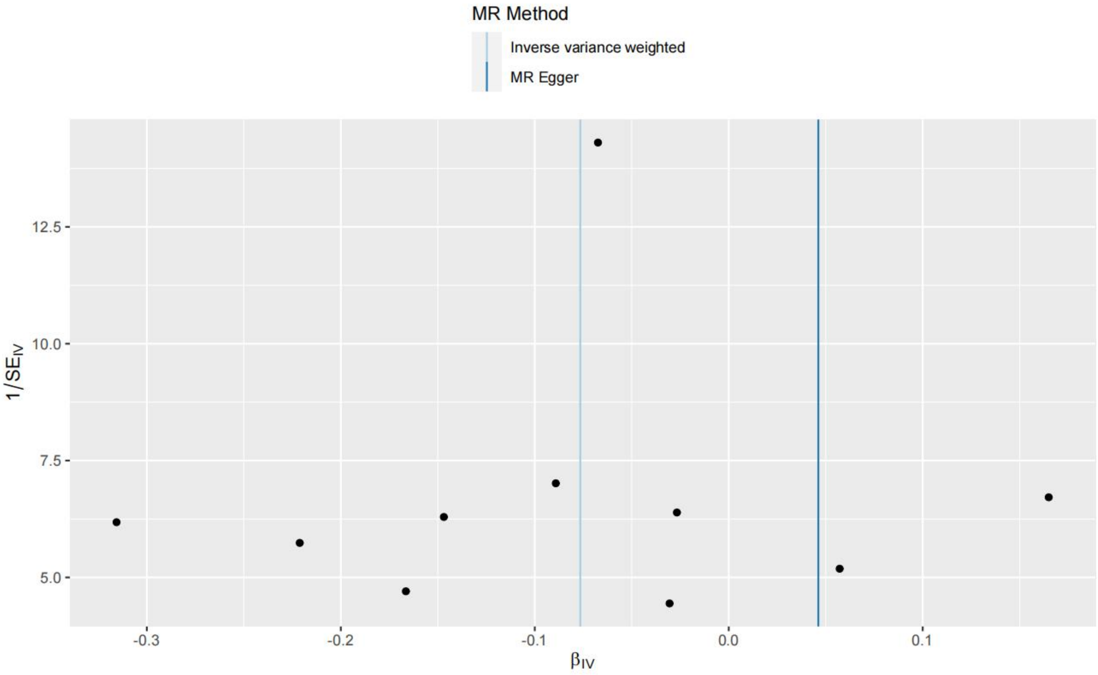

B. Funnel plot of IL-7 instruments strength on PD

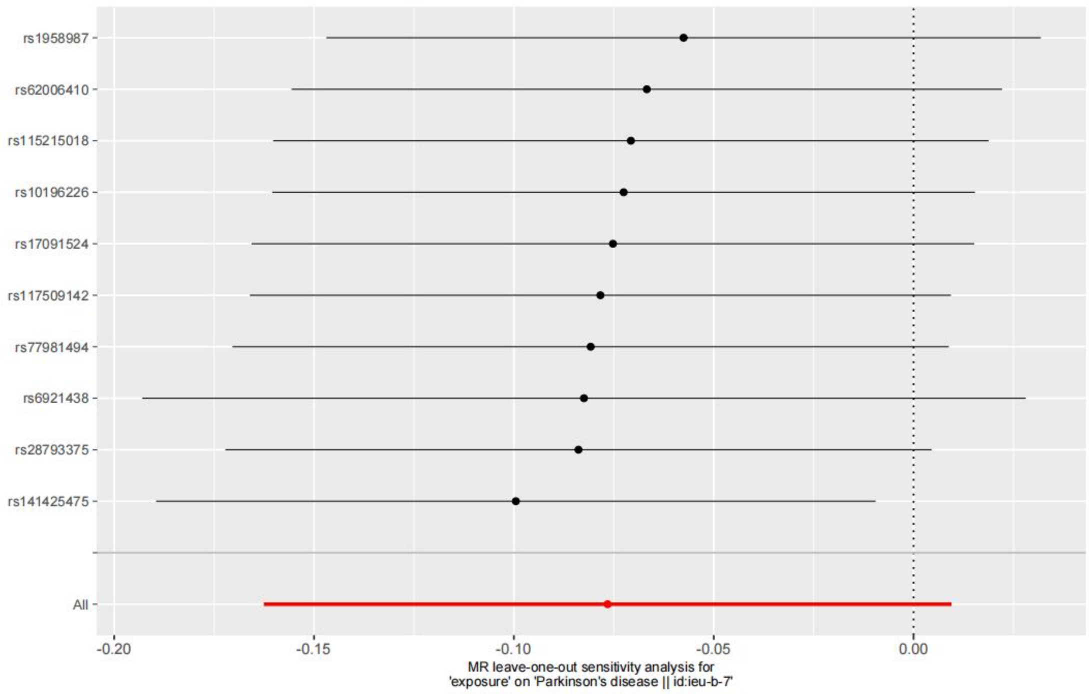

C. MR leave-one-out sensitivity analysis for IL-7 on PD

eFigure 58. IL-8-associated SNPs with risk of PD

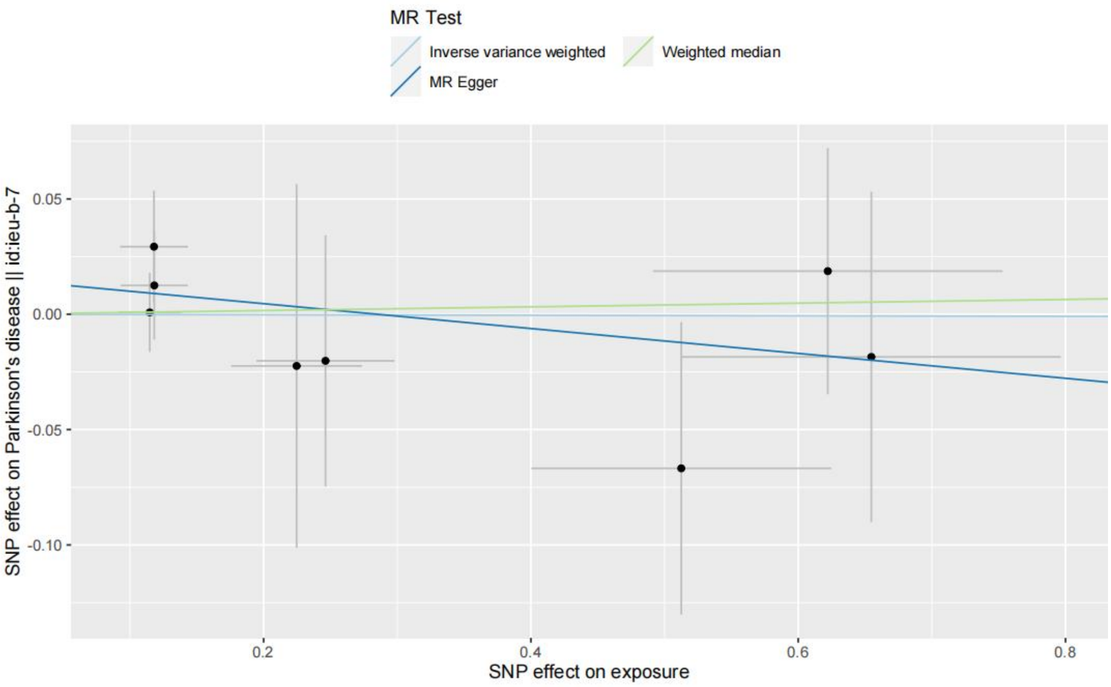

A. Scatter plot of IL-8 with risk of PD

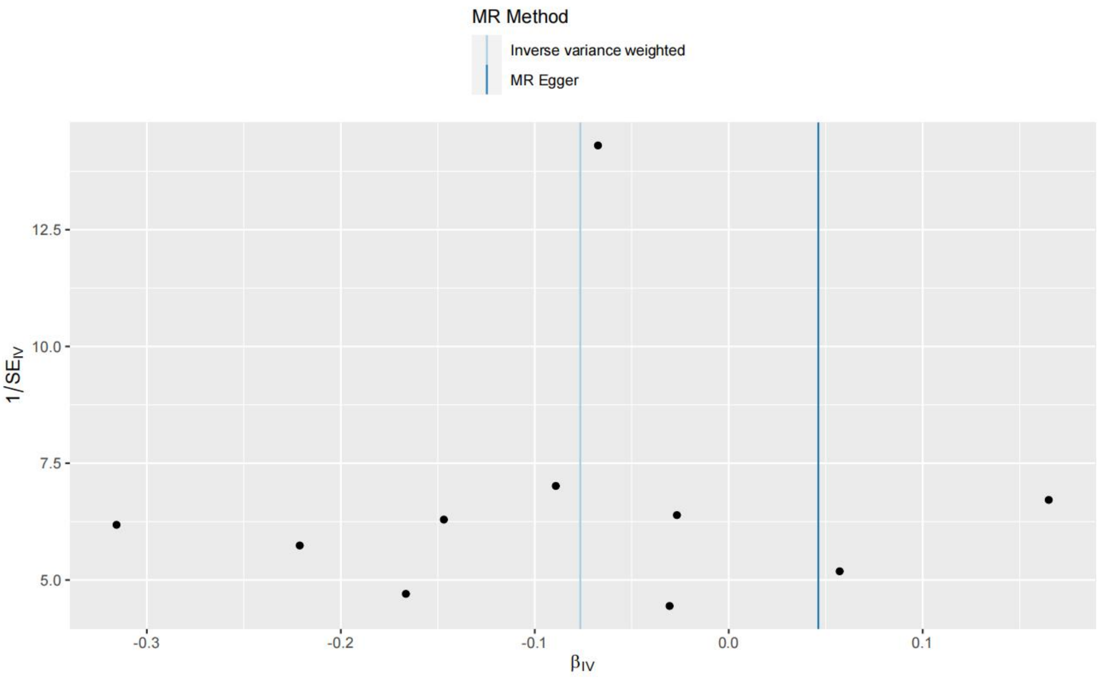

B. Funnel plot of IL-8 instruments strength on PD

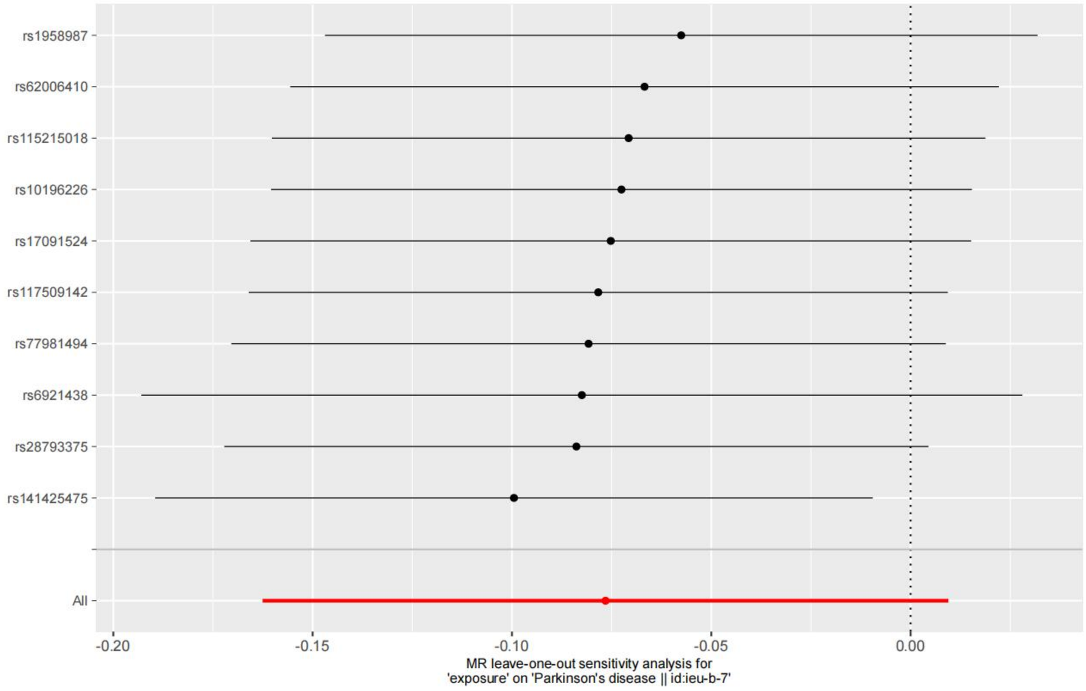

C. MR leave-one-out sensitivity analysis for IL-8 on PD

eFigure 59. IL-9-associated SNPs with risk of PD

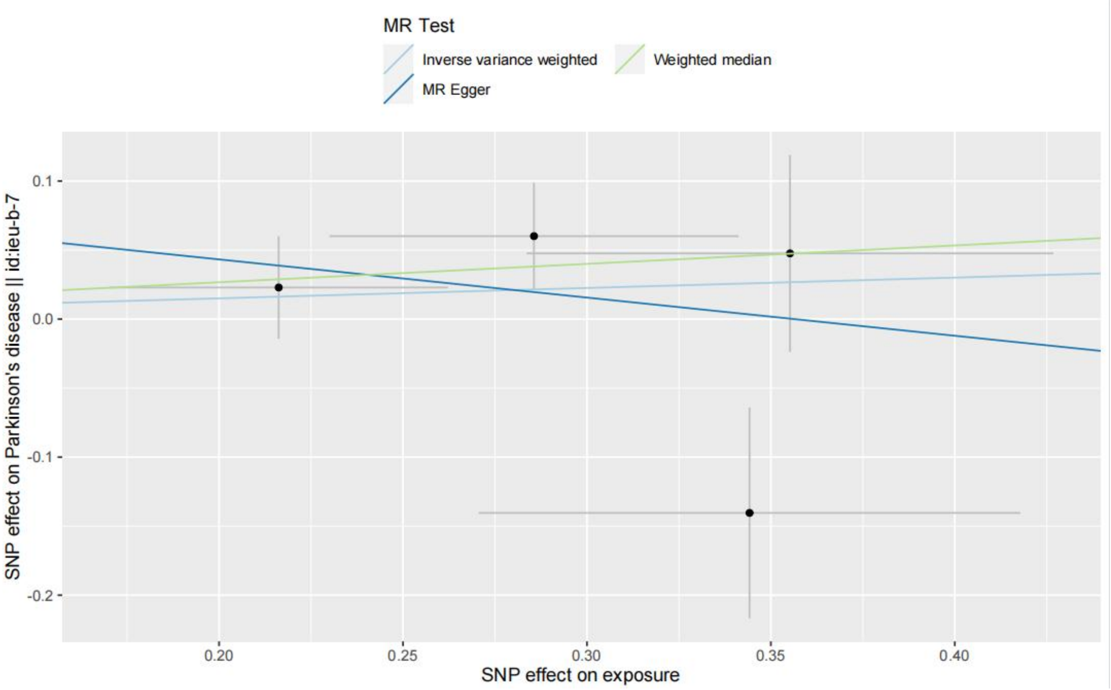

A. Scatter plot of IL-9 with risk of PD

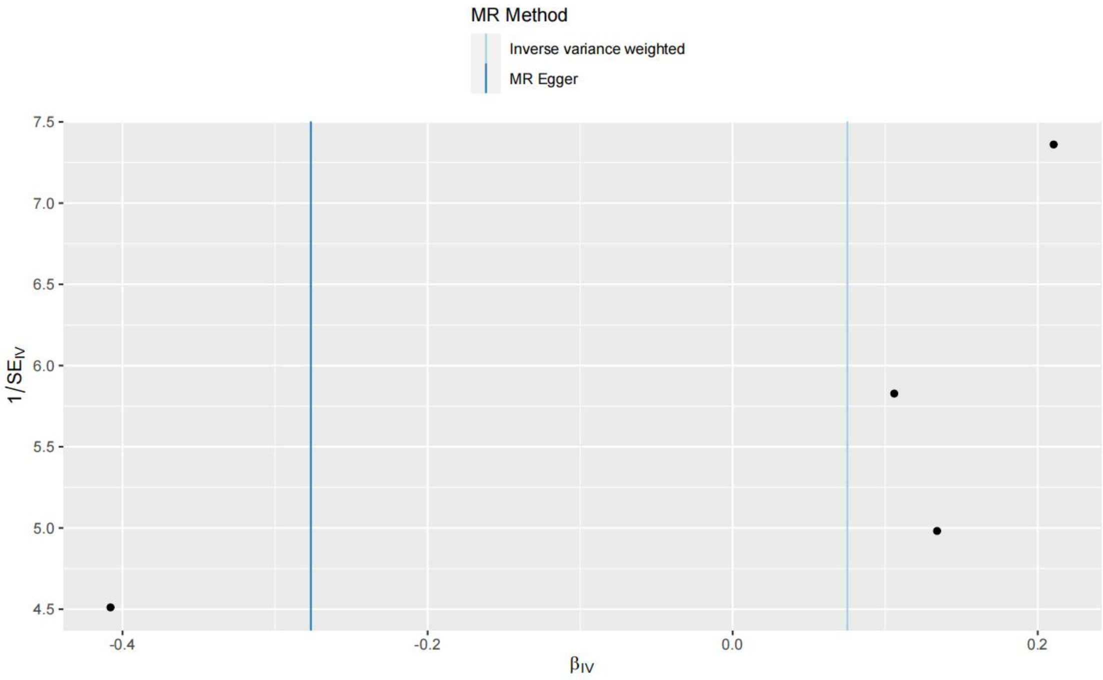

B. Funnel plot of IL-9 instruments strength on PD

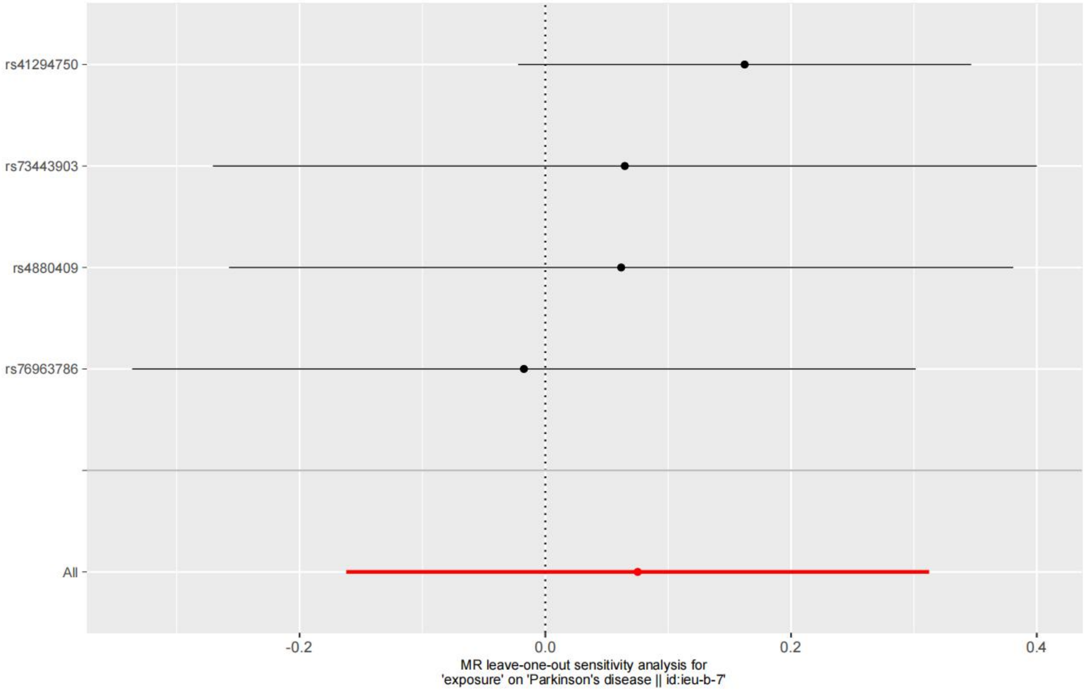

C. MR leave-one-out sensitivity analysis for IL-9 on PD

eFigure 60. IL-10-associated SNPs with risk of PD

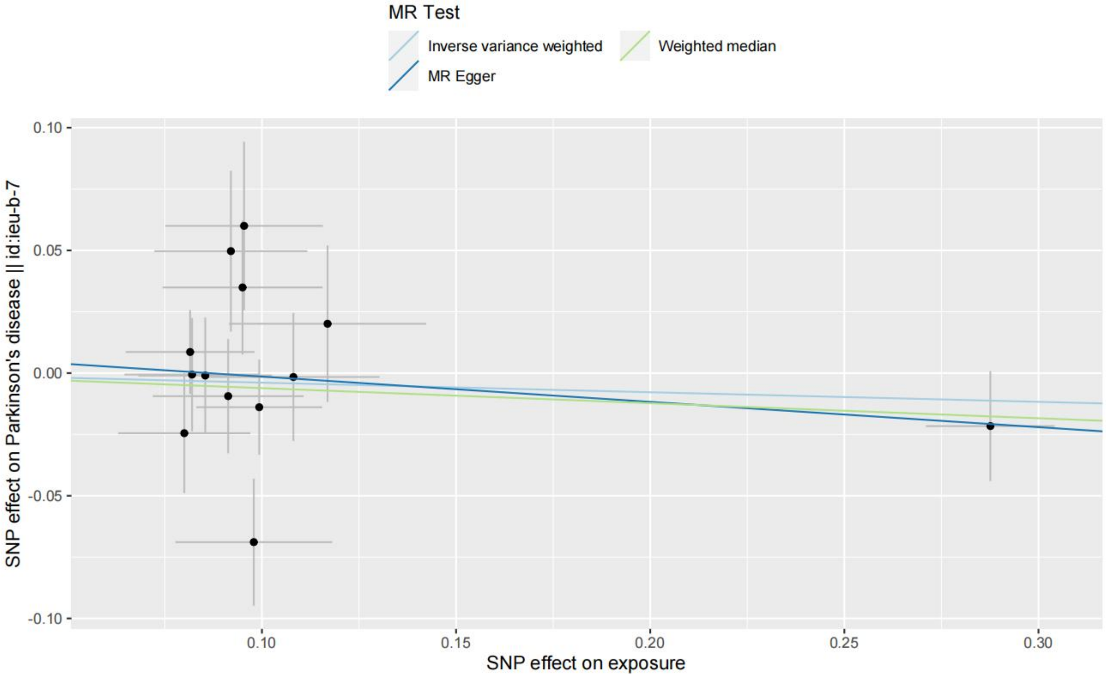

A. Scatter plot of IL-10 with risk of PD

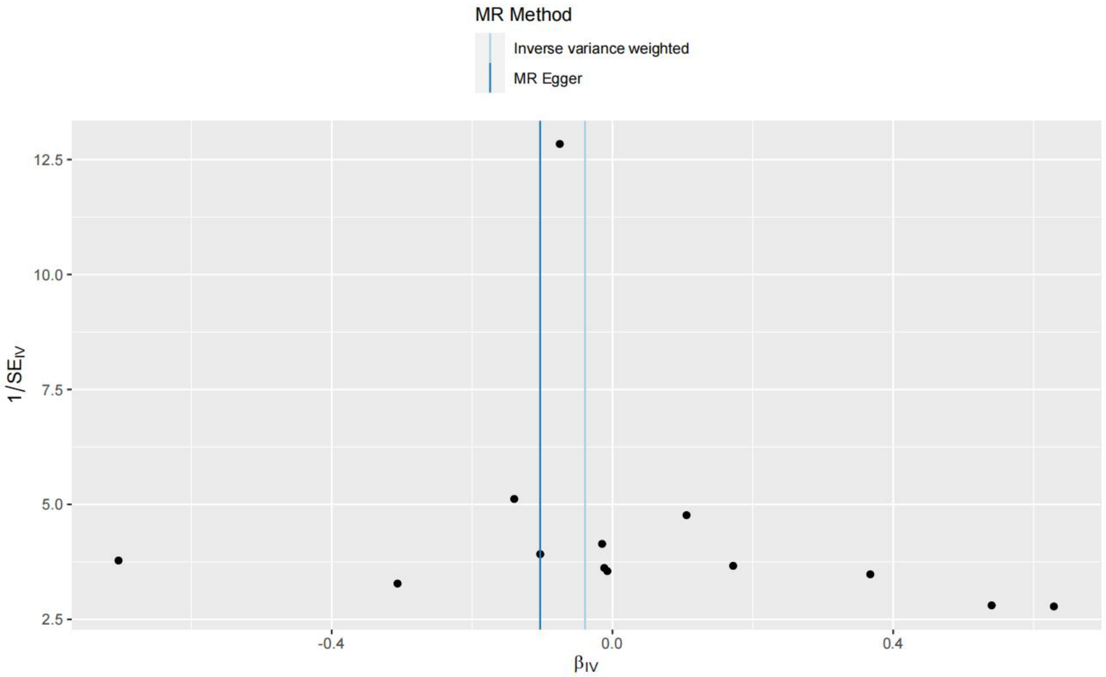

B. Funnel plot of IL-10 instruments strength on PD

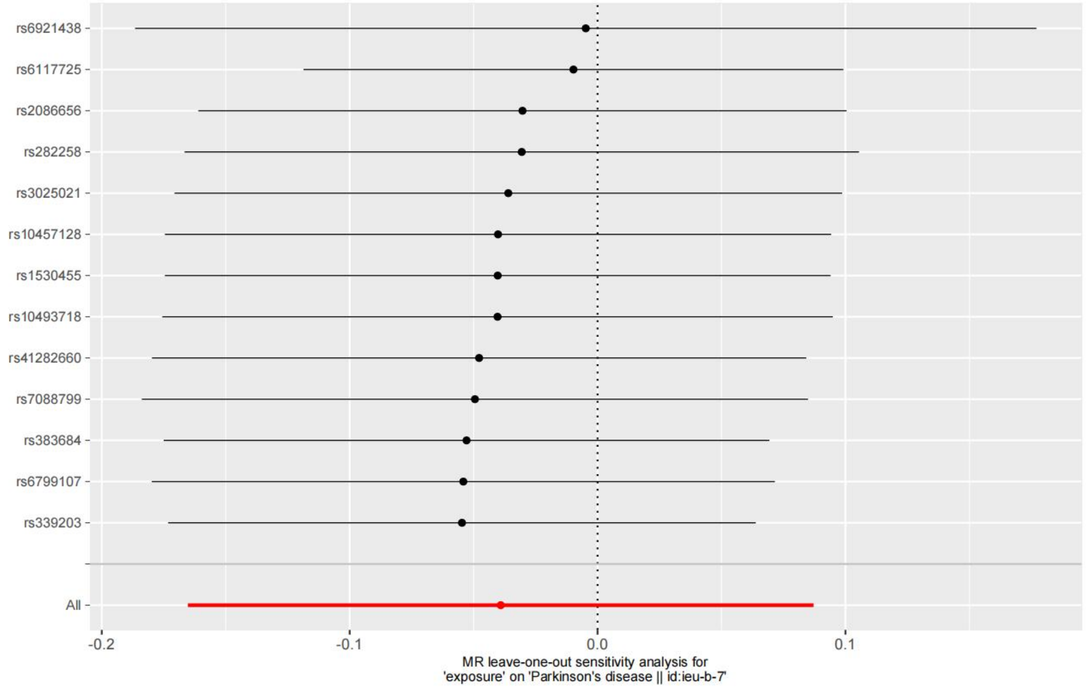

C. MR leave-one-out sensitivity analysis for IL-10 on PD

eFigure 61. IL-12-associated SNPs with risk of PD

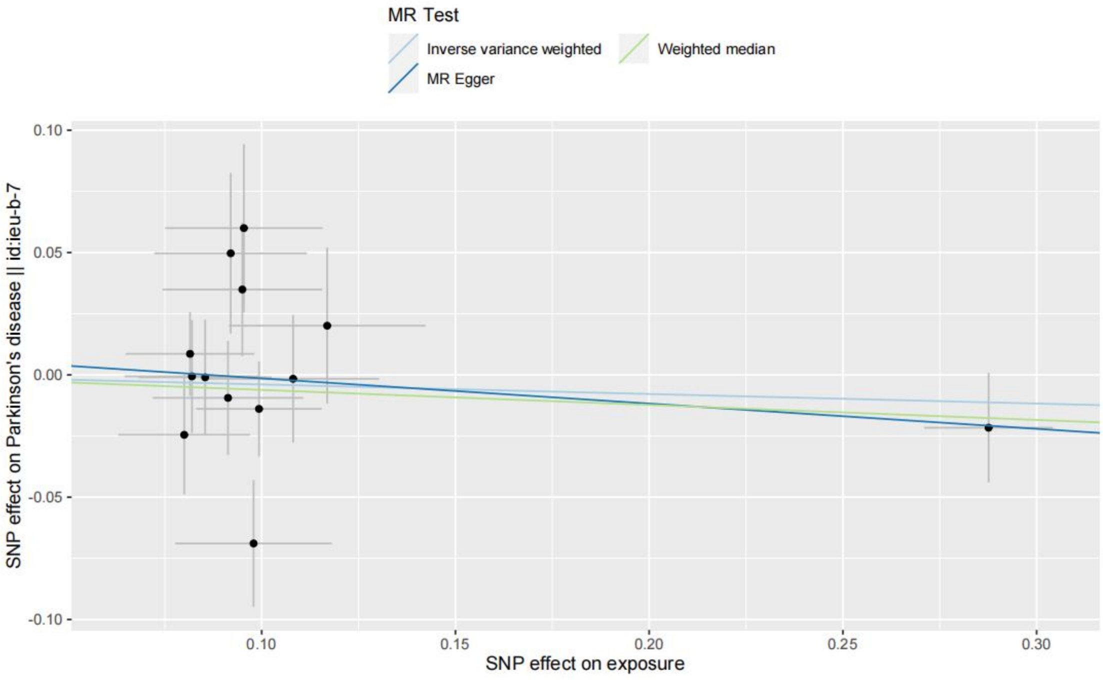

A. Scatter plot of IL-12 with risk of PD

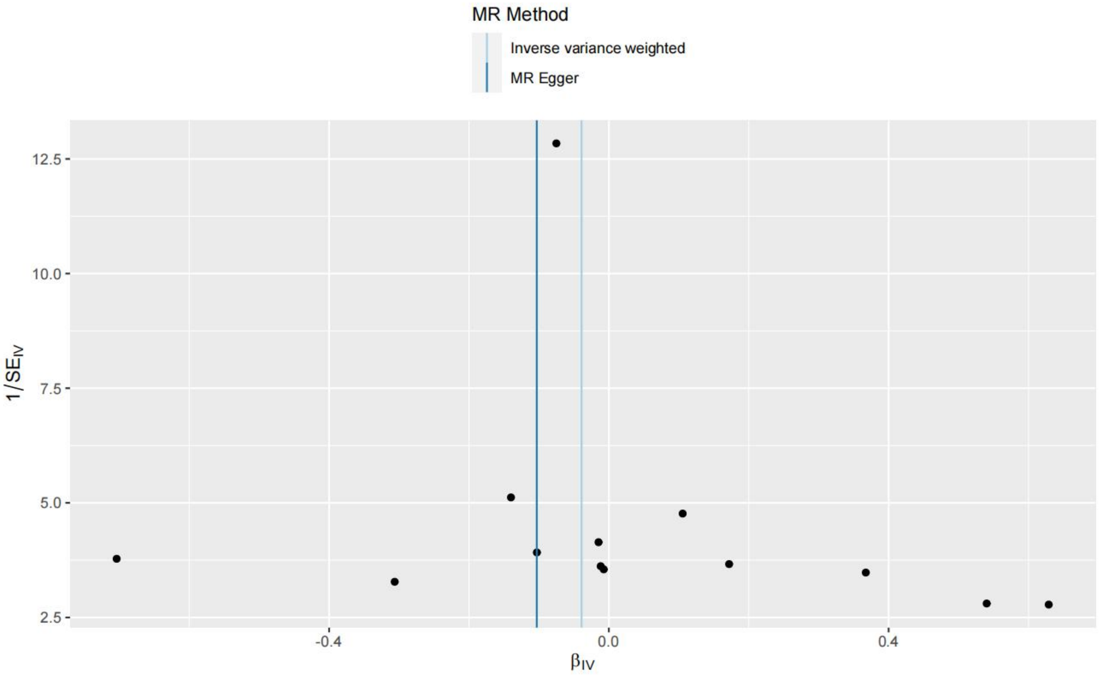

B. Funnel plot of IL-12 instruments strength on PD

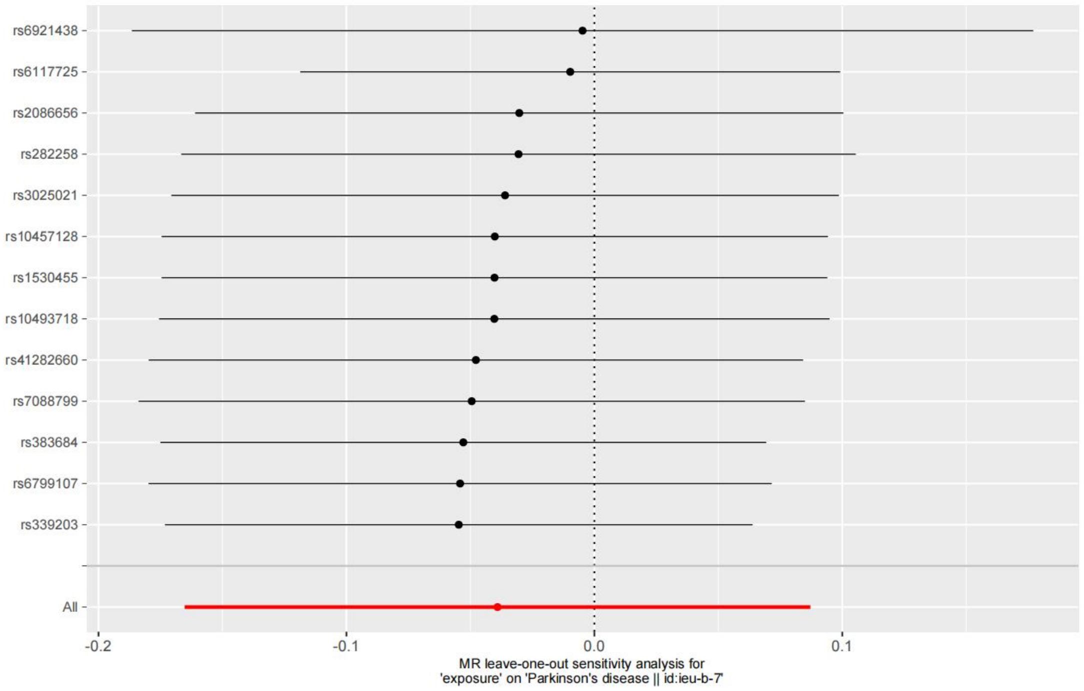

C. MR leave-one-out sensitivity analysis for IL-12 on PD

eFigure 62. IL-13-associated SNPs with risk of PD

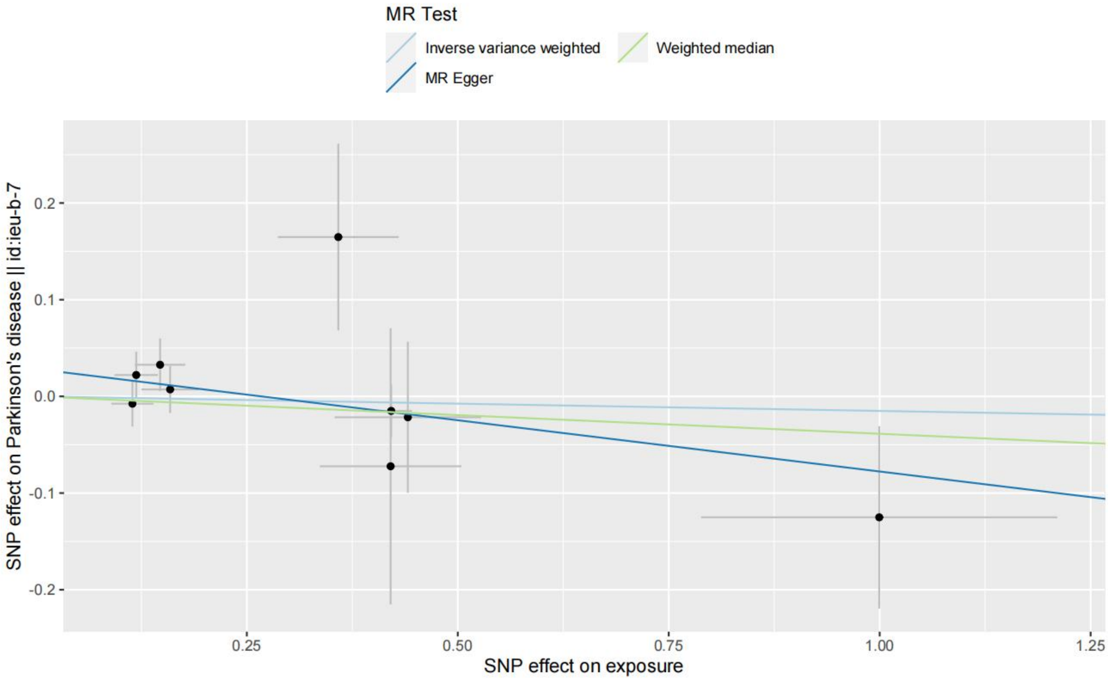

A. Scatter plot of IL-13 with risk of PD

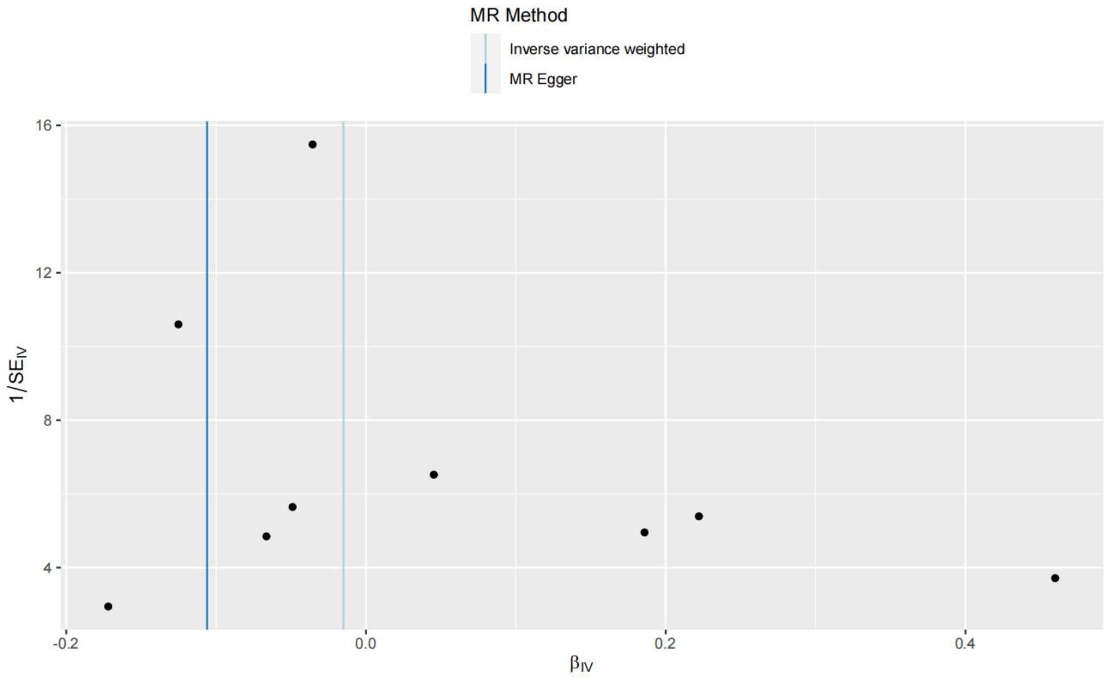

B. Funnel plot of IL-13 instruments strength on PD

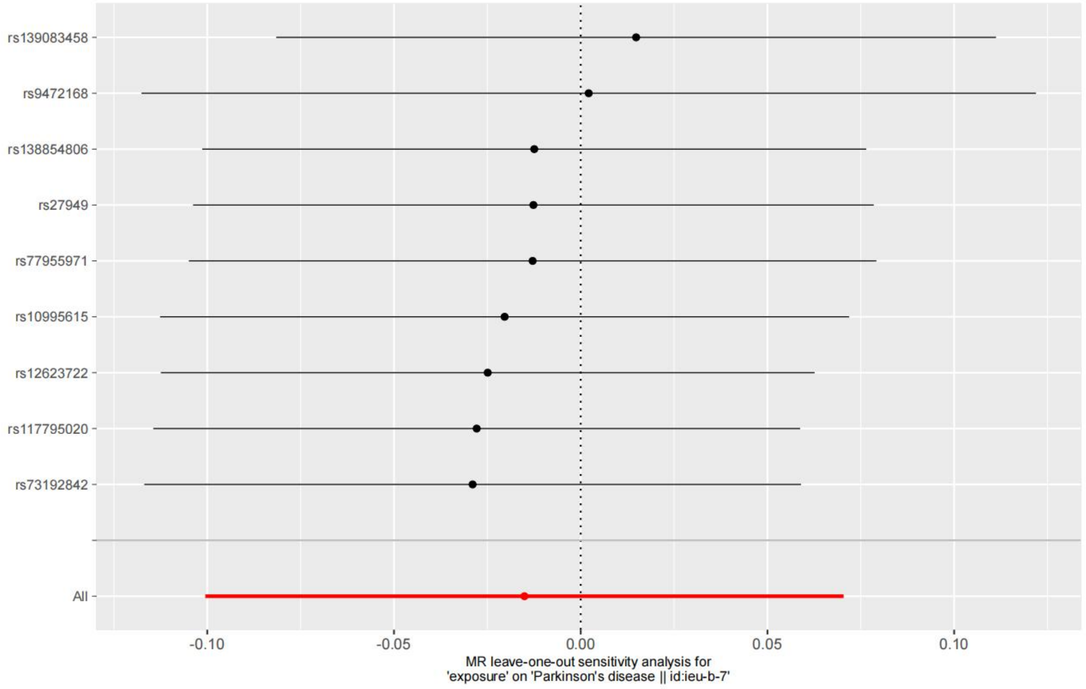

C. MR leave-one-out sensitivity analysis for IL-13 on PD

eFigure 63. IL-16-associated SNPs with risk of PD

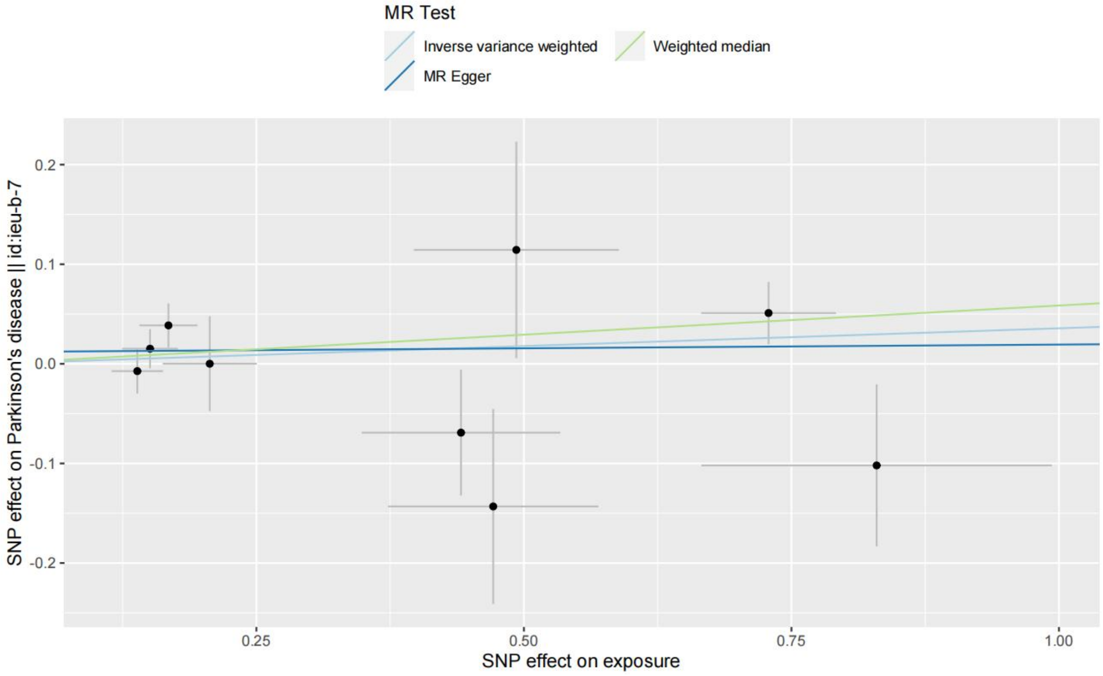

A. Scatter plot of IL-16 with risk of PD

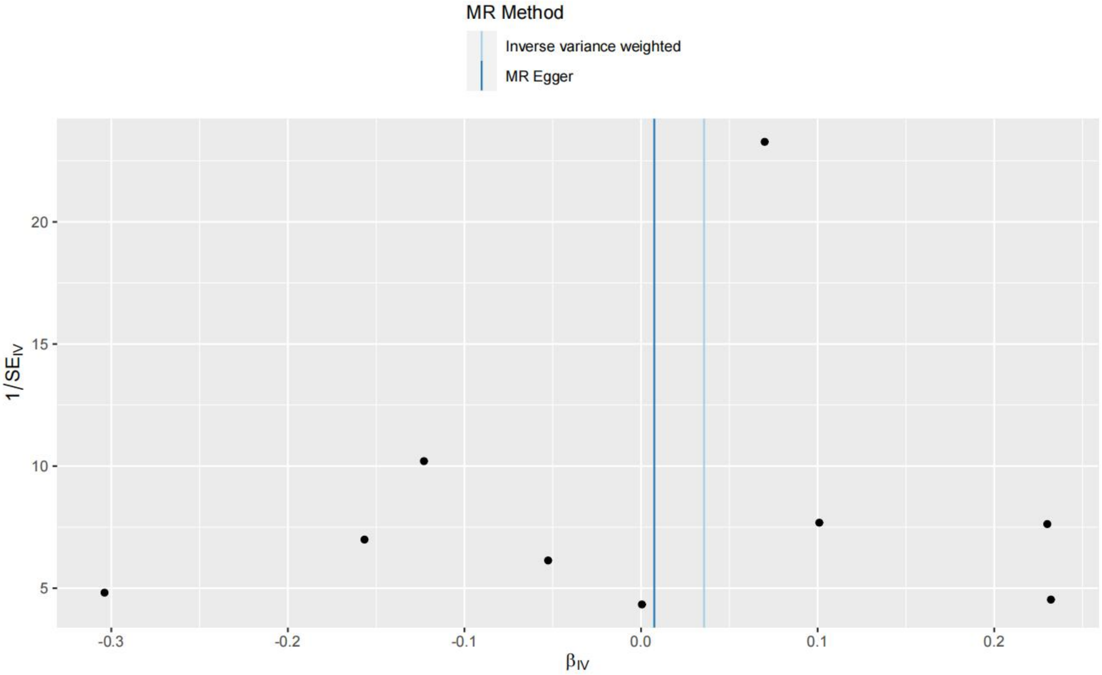

B. Funnel plot of IL-16 instruments strength on PD

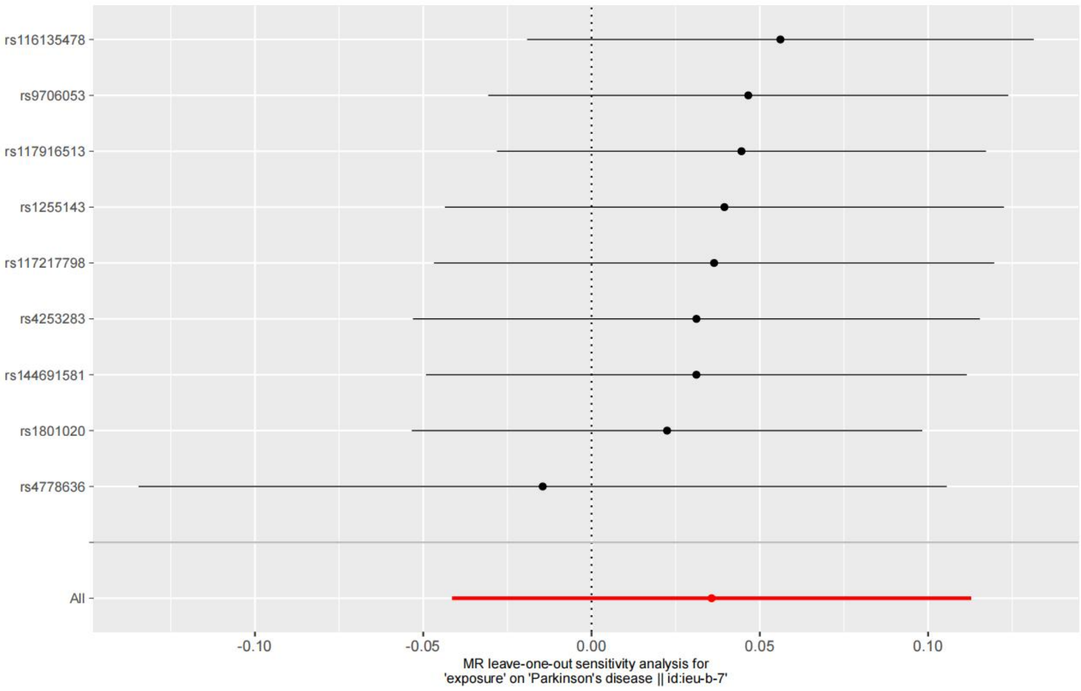

C. MR leave-one-out sensitivity analysis for IL-16 on PD

eFigure 64. IL-17-associated SNPs with risk of PD

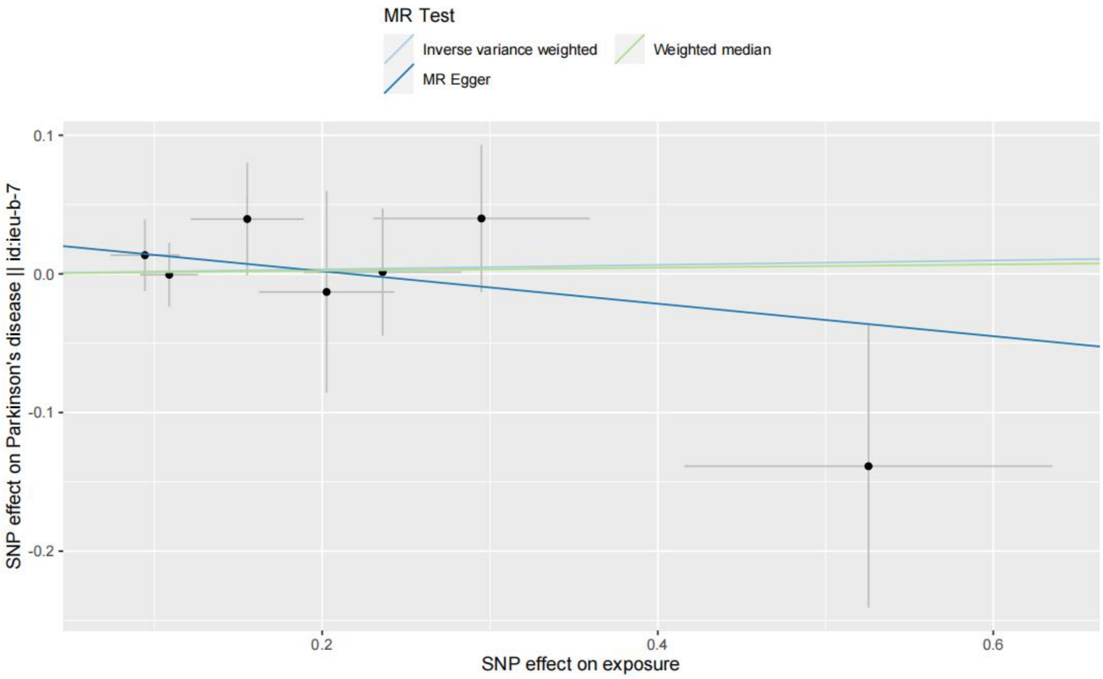

A. Scatter plot of IL-17 with risk of PD

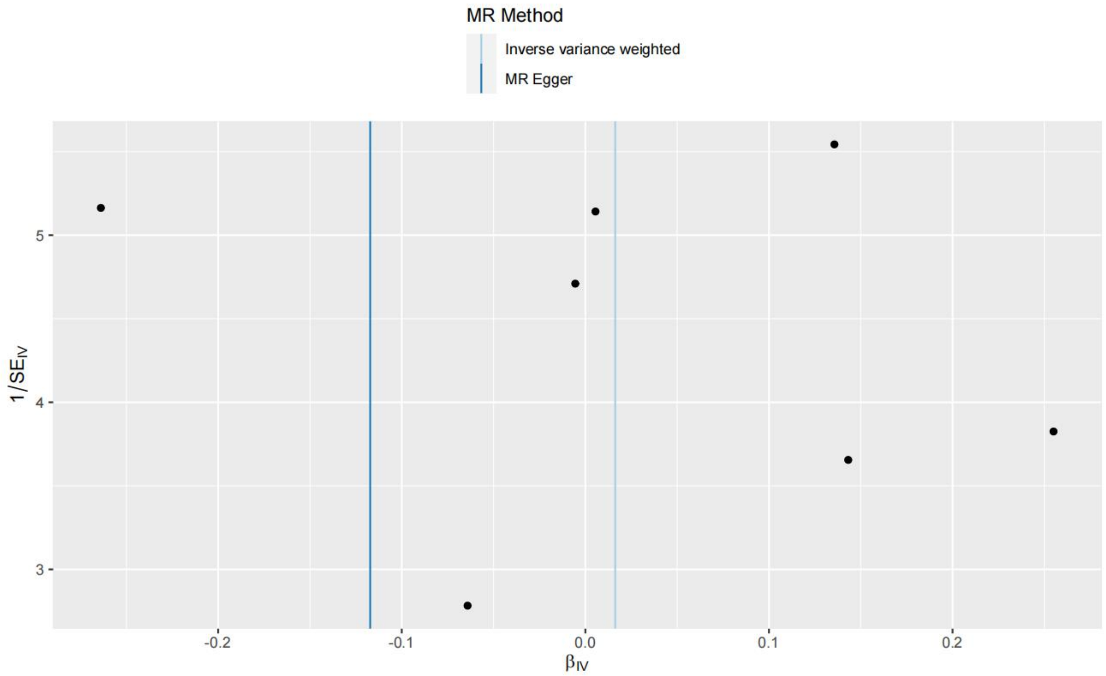

B. Funnel plot of IL-17 instruments strength on PD

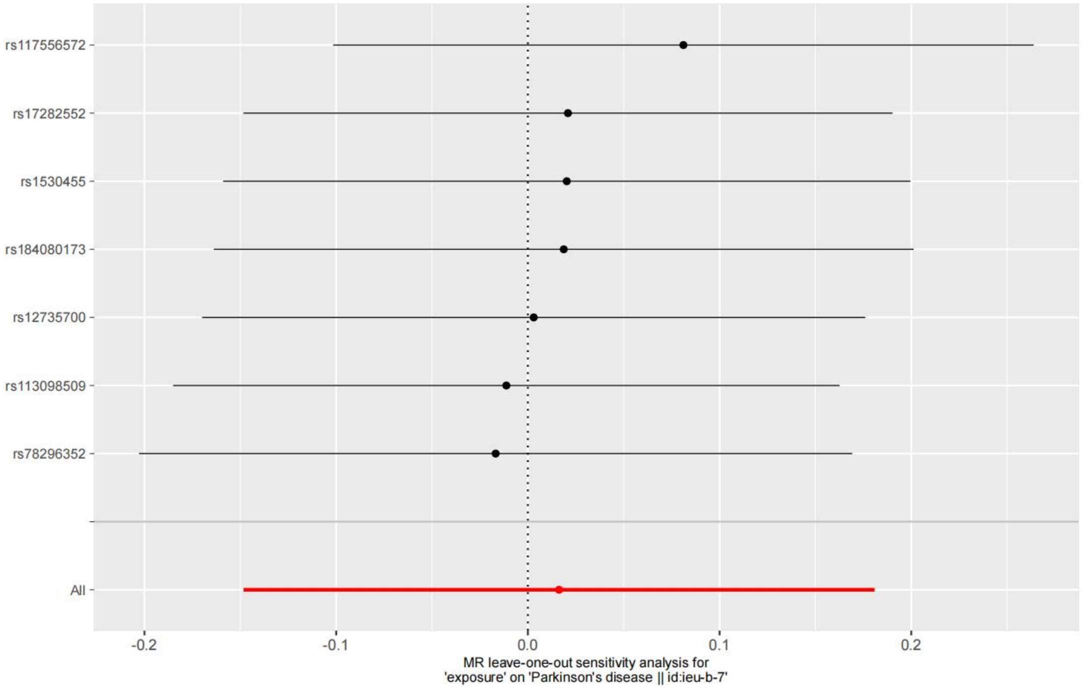

C. MR leave-one-out sensitivity analysis for IL-17 on PD

eFigure 65. IL-18-associated SNPs with risk of PD

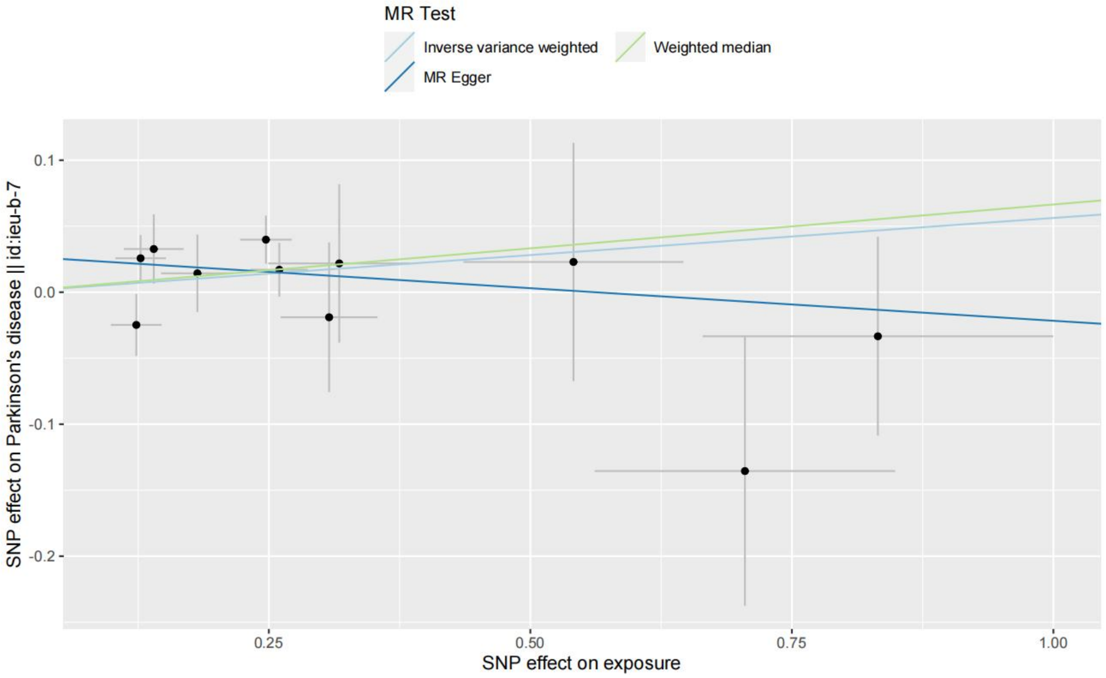

A. Scatter plot of IL-18 with risk of PD

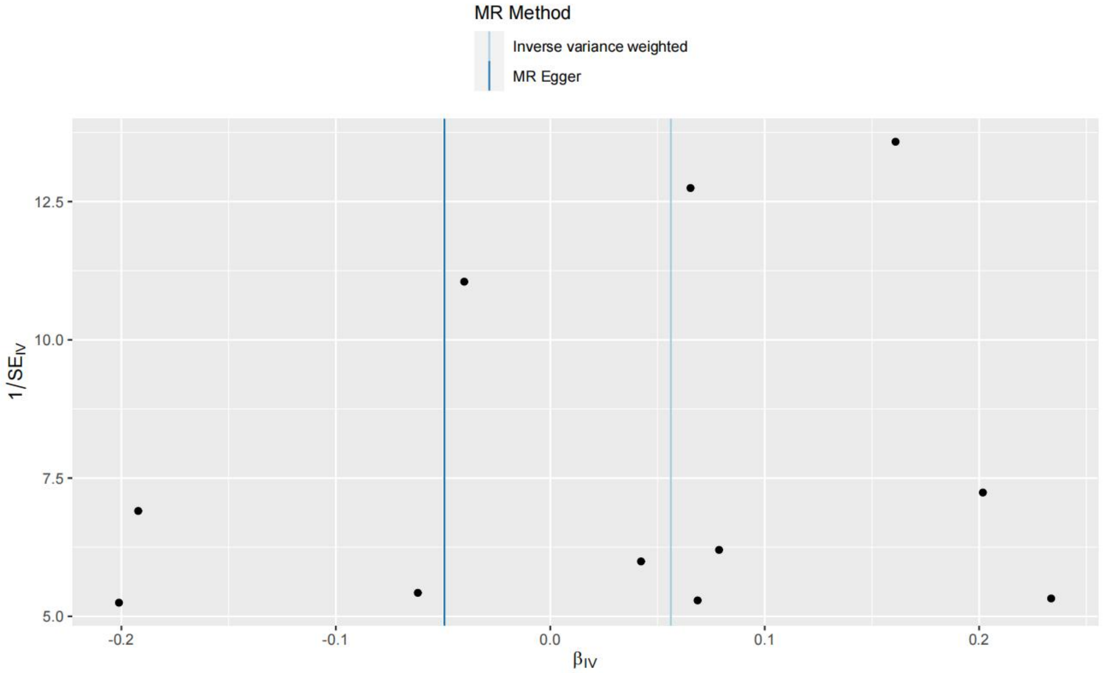

B. Funnel plot of IL-18 instruments strength on PD

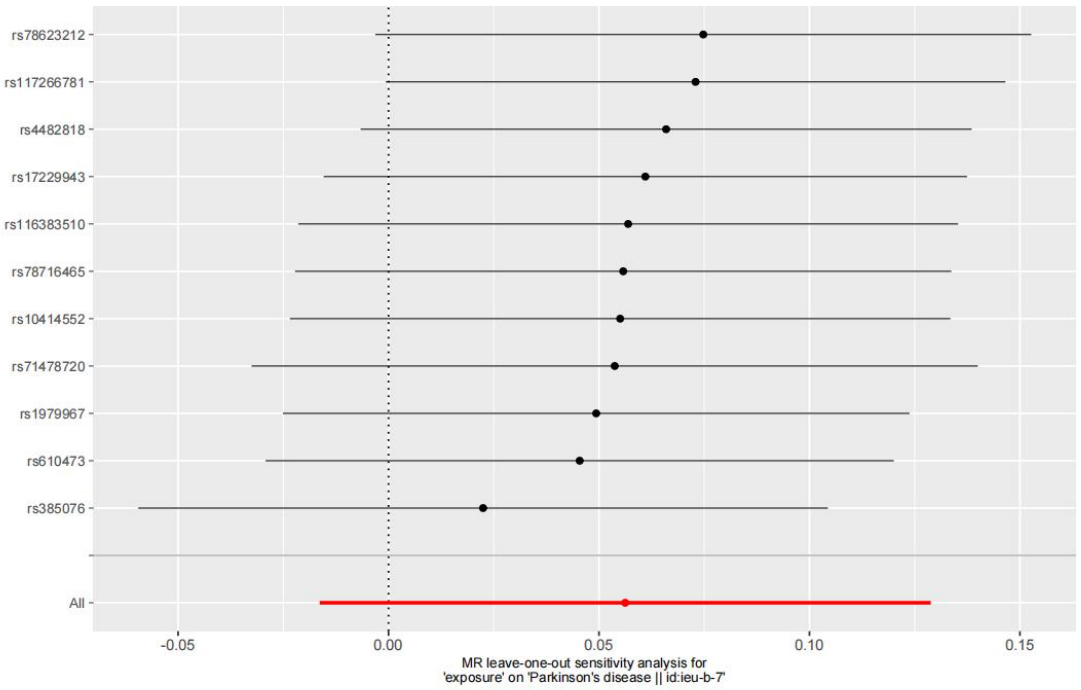

C. MR leave-one-out sensitivity analysis for IL-18 on PD

eFigure 66. IP-10-associated SNPs with risk of PD

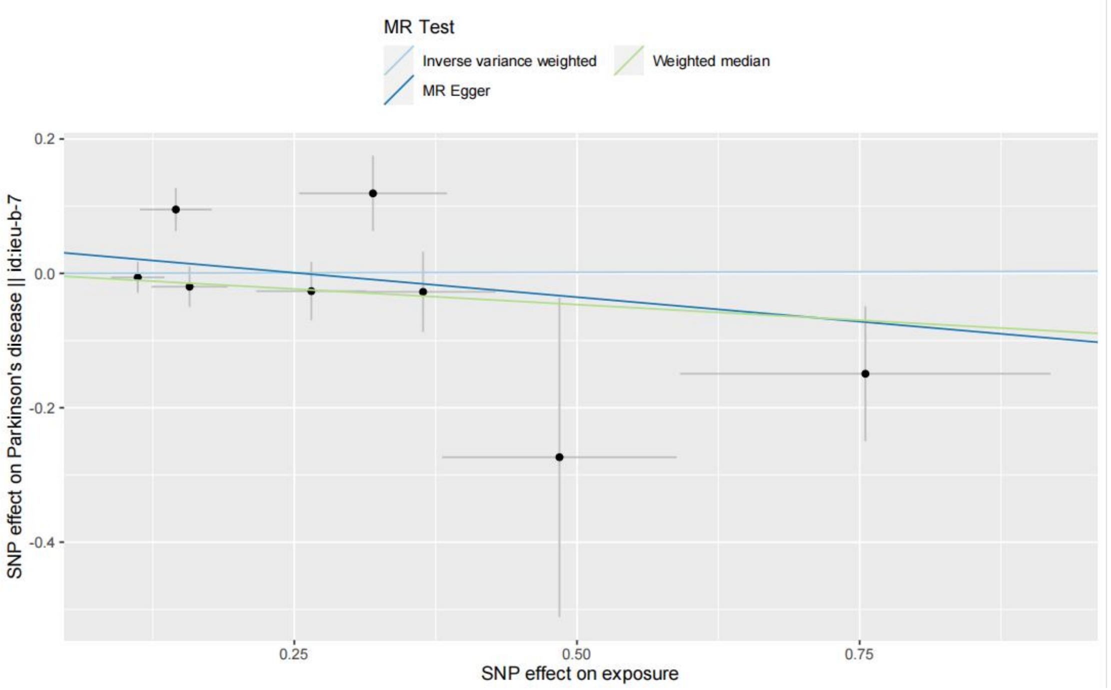

A. Scatter plot of IP-10 with risk of PD

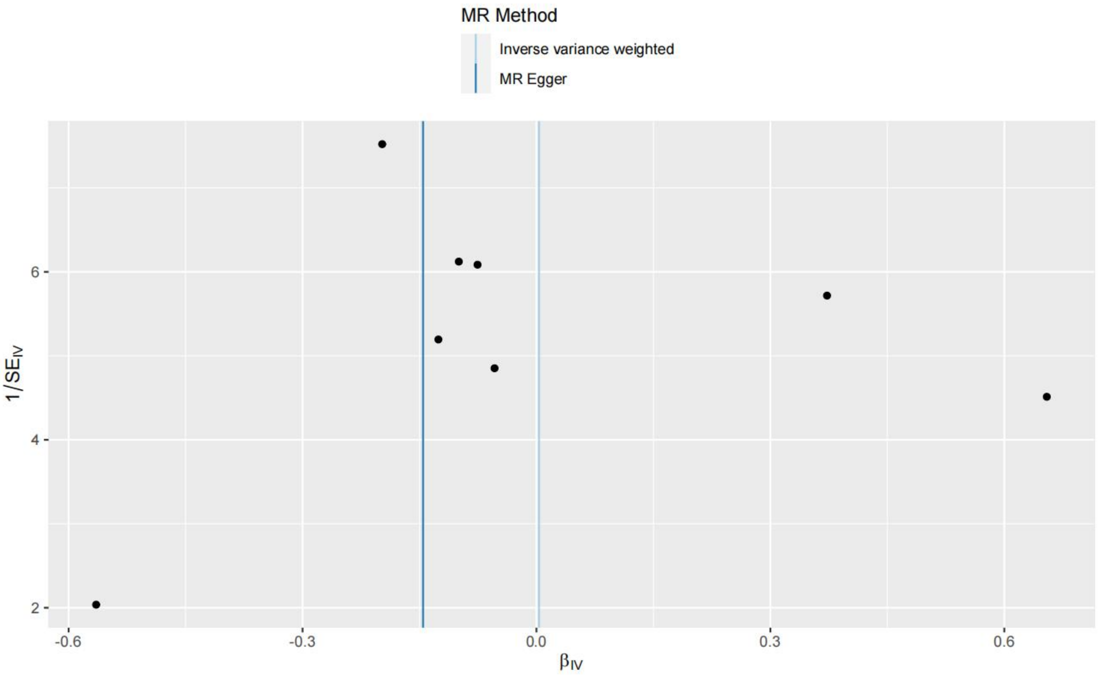

B. Funnel plot of IP-10 instruments strength on PD

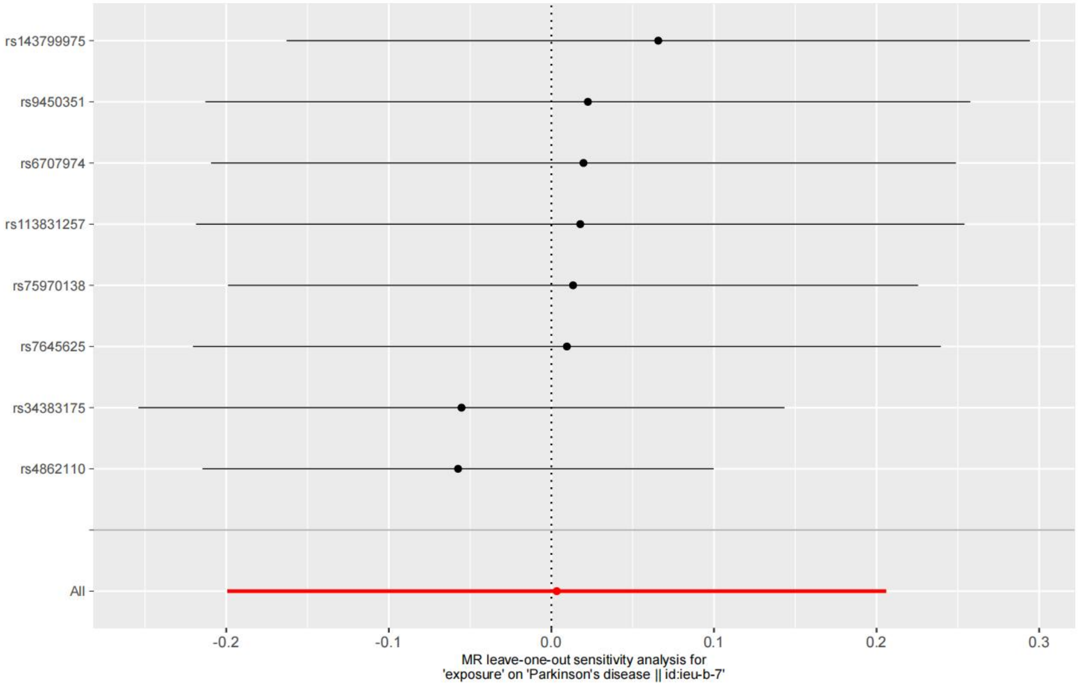

C. MR leave-one-out sensitivity analysis for IP-10 on PD

eFigure 67. M-CSF-associated SNPs with risk of PD

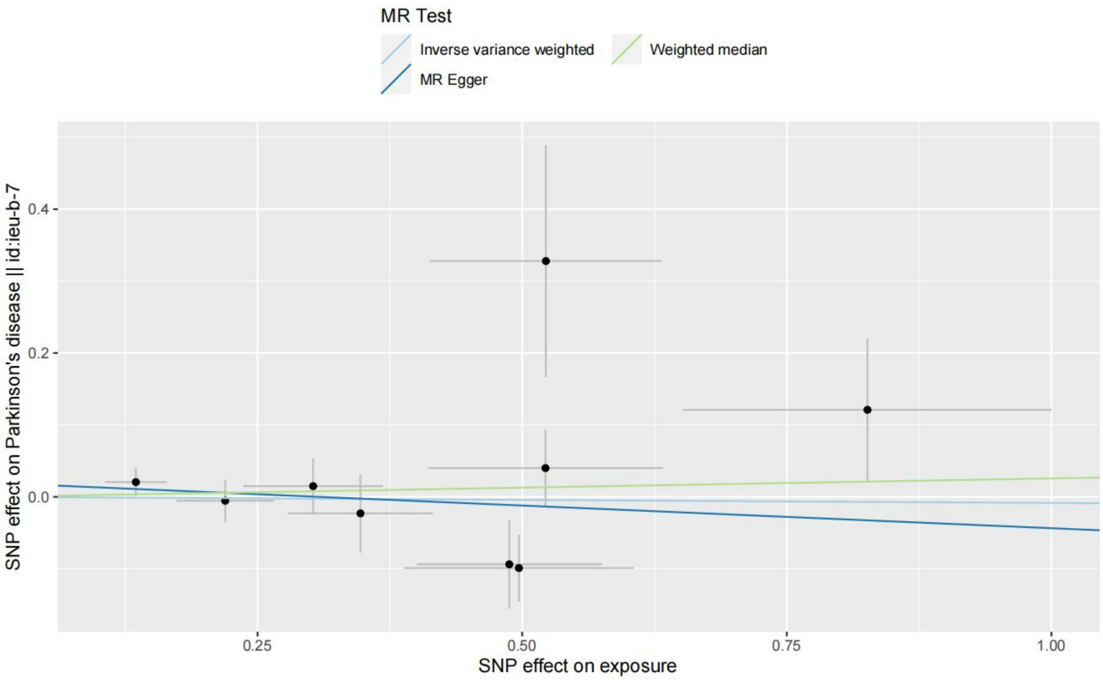

A. Scatter plot of M-CSF with risk of PD

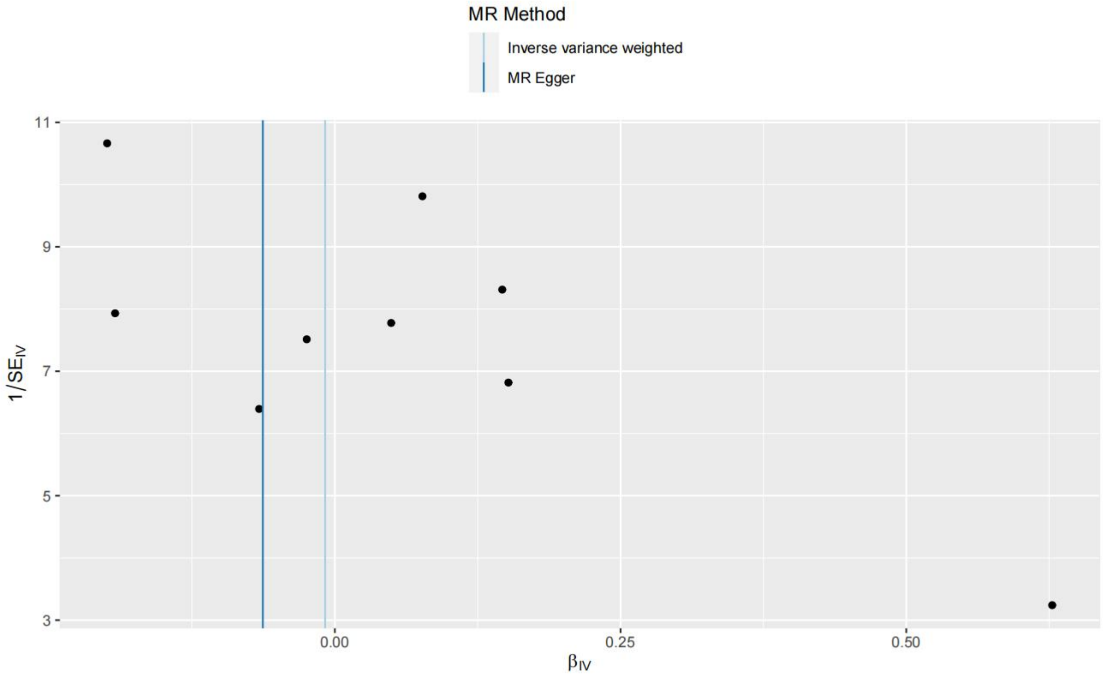

B. Funnel plot of M-CSF instruments strength on PD

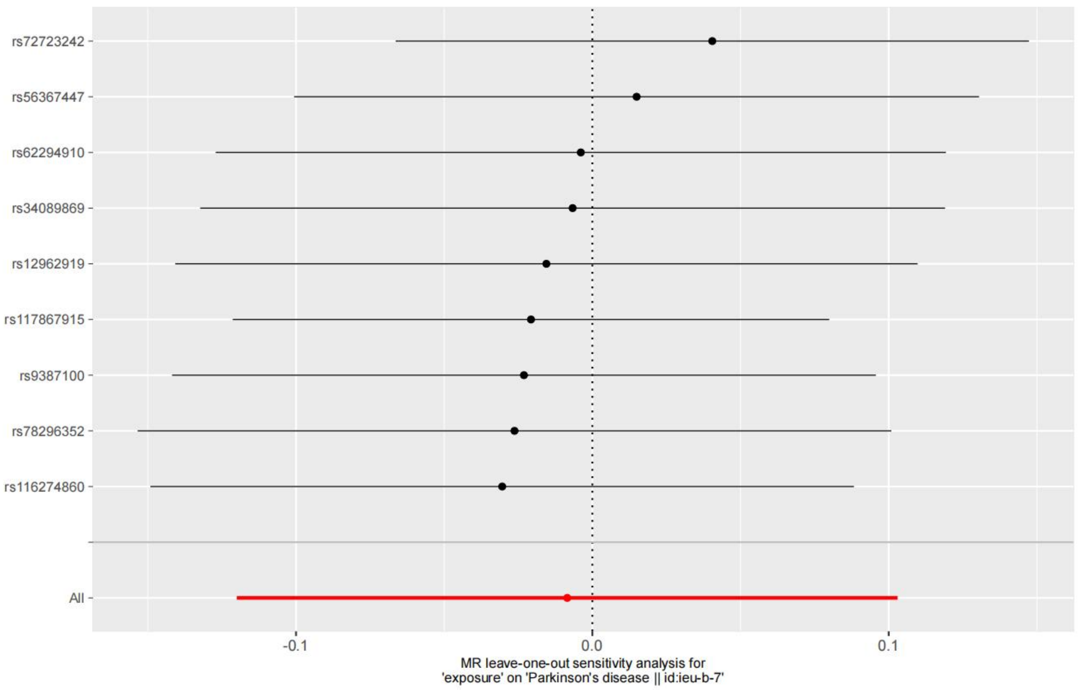

C. MR leave-one-out sensitivity analysis for M-CSF on PD

eFigure 68. MCP-1-MCAF-associated SNPs with risk of PD

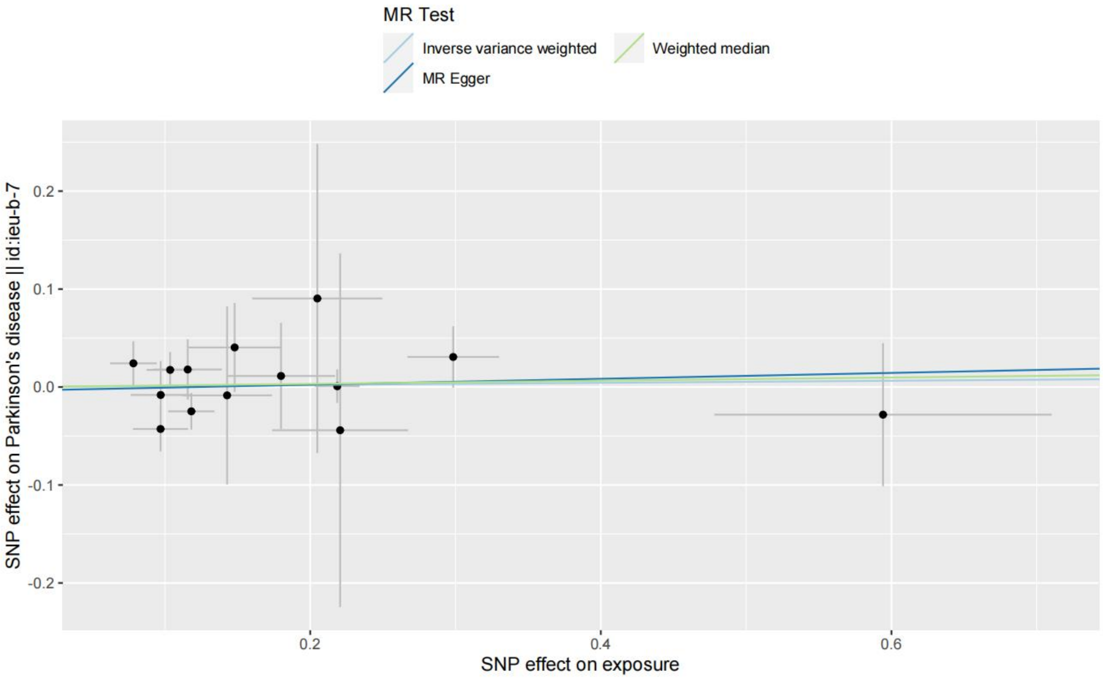

A. Scatter plot of MCP-1-MCAF with risk of PD

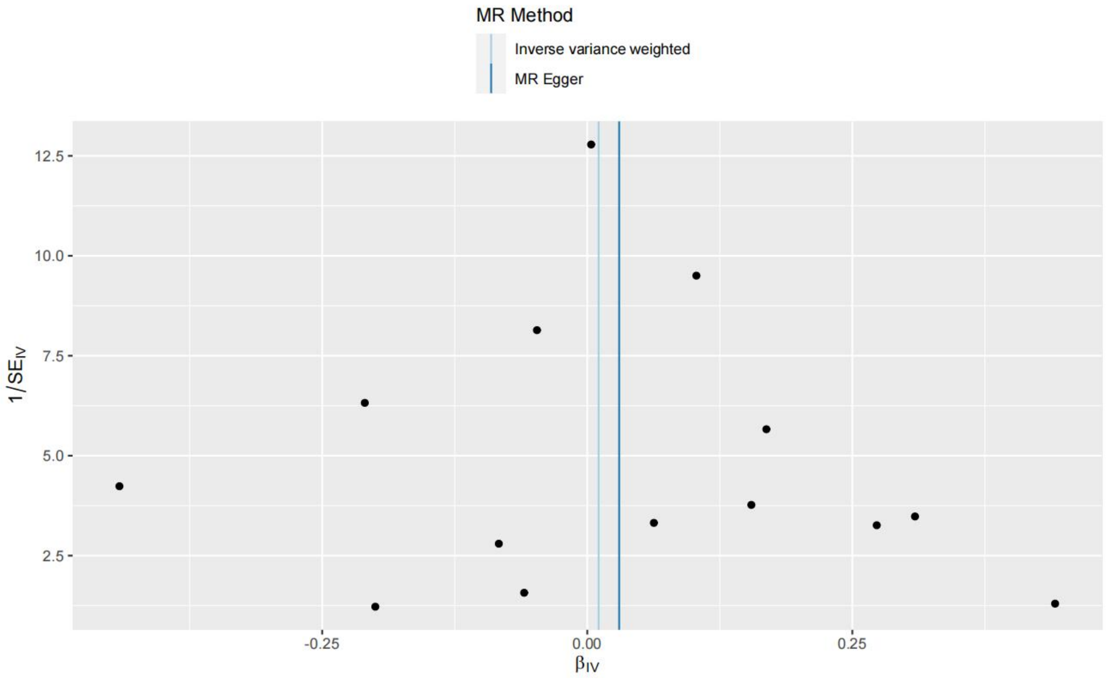

B. Funnel plot of MCP-1-MCAF instruments strength on PD

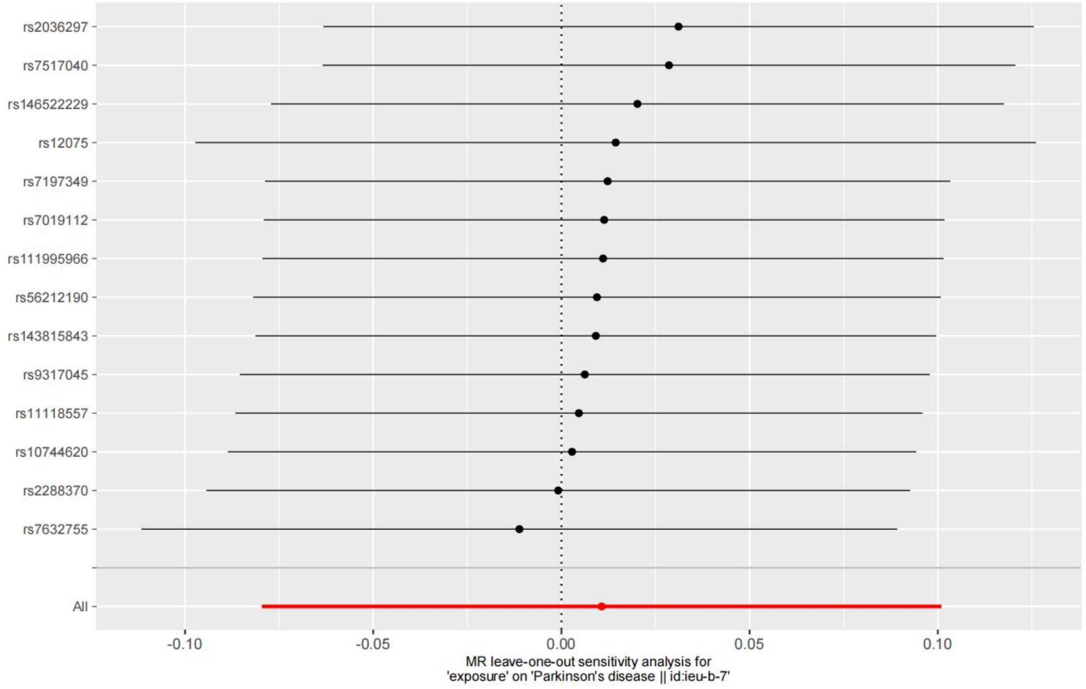

C. MR leave-one-out sensitivity analysis for MCP-1-MCAF on PD

eFigure 69. MCP-3-associated SNPs with risk of PD

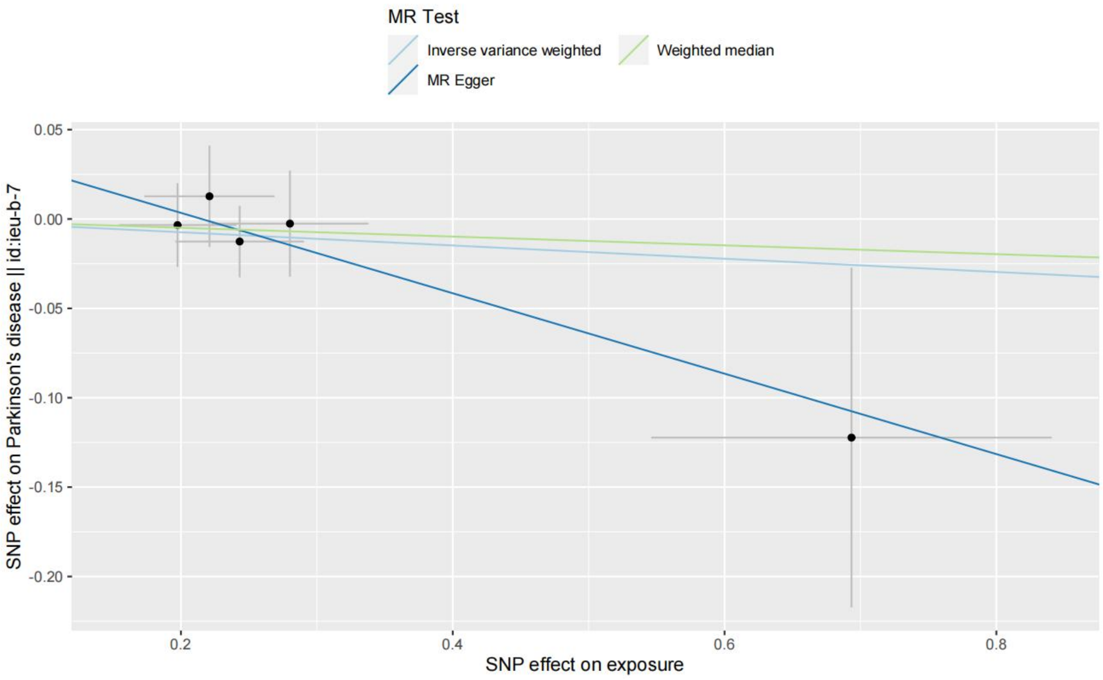

A. Scatter plot of MCP-3 with risk of PD

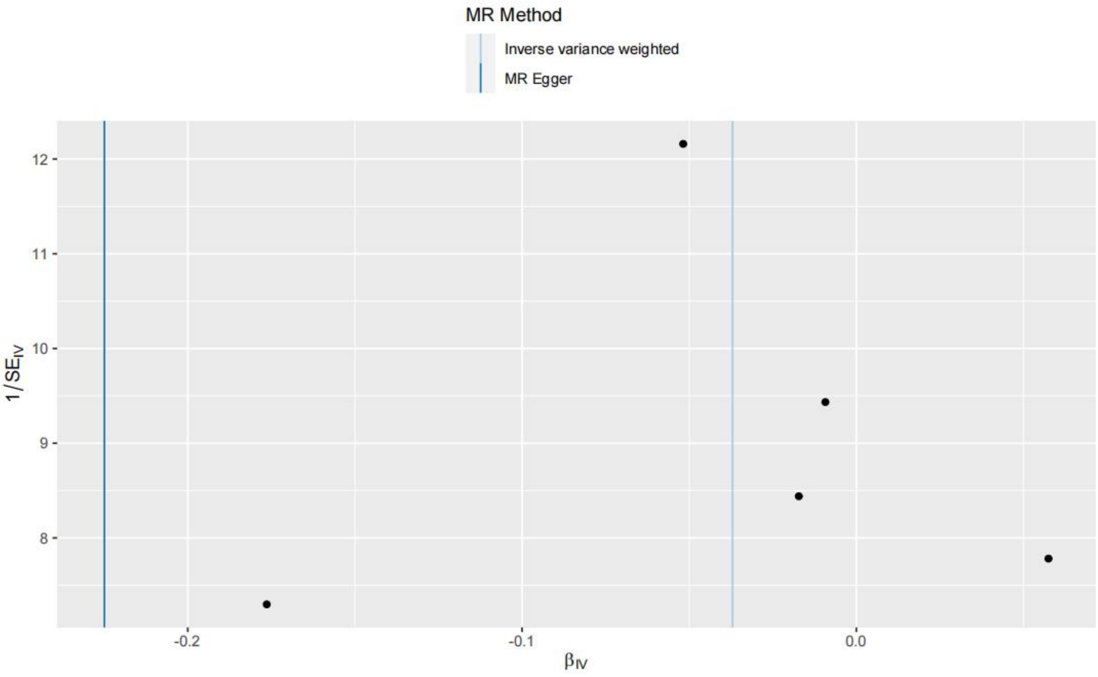

B. Funnel plot of MCP-3 instruments strength on PD

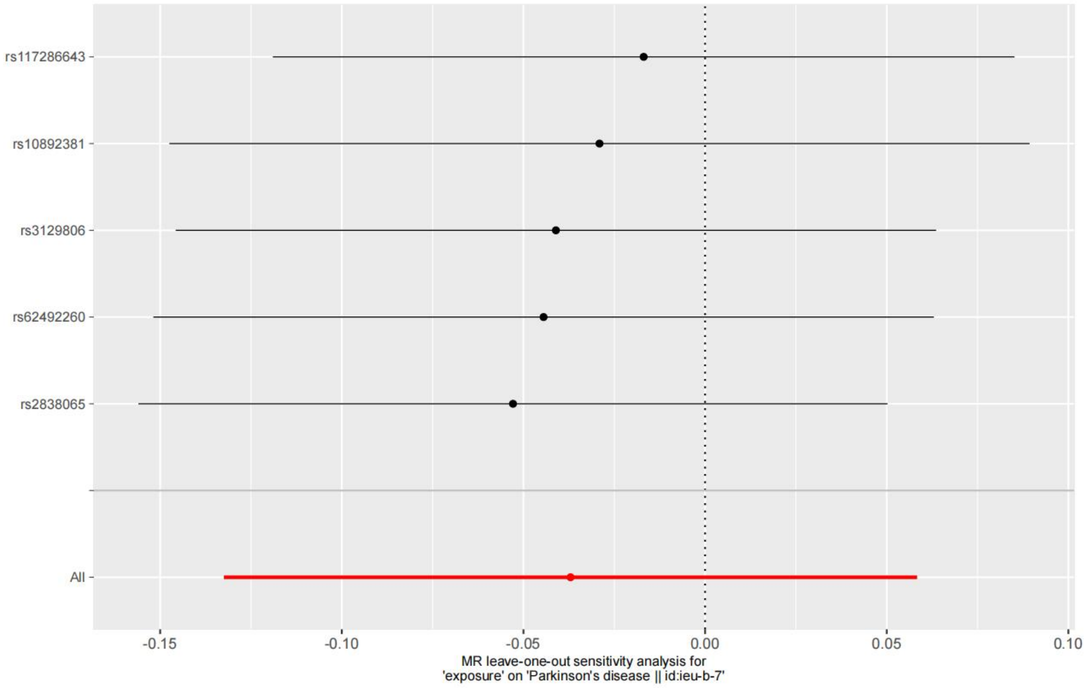

C. MR leave-one-out sensitivity analysis for MCP-3 on PD

eFigure 70. MIF-associated SNPs with risk of PD

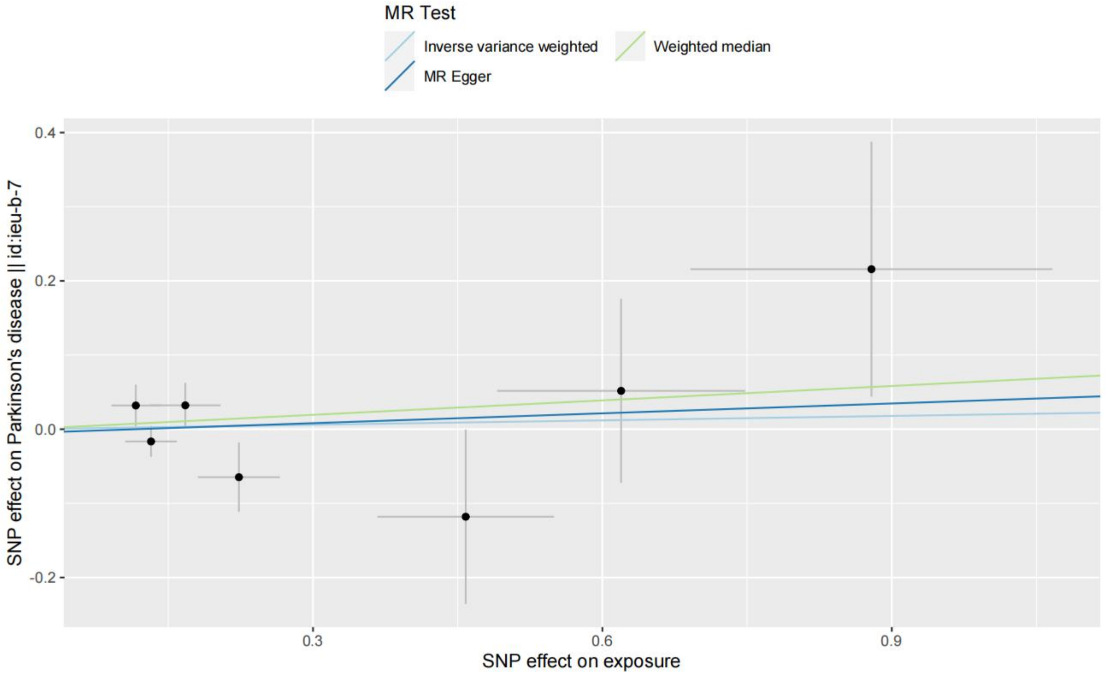

A. Scatter plot of MIF with risk of PD

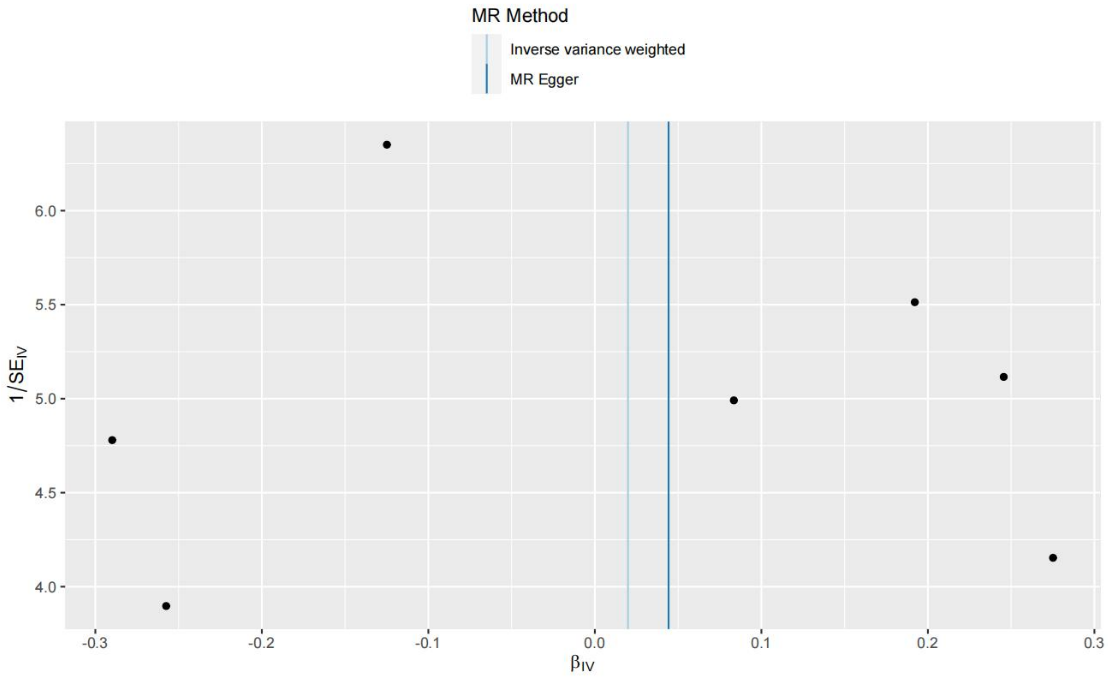

B. Funnel plot of MIF instruments strength on PD

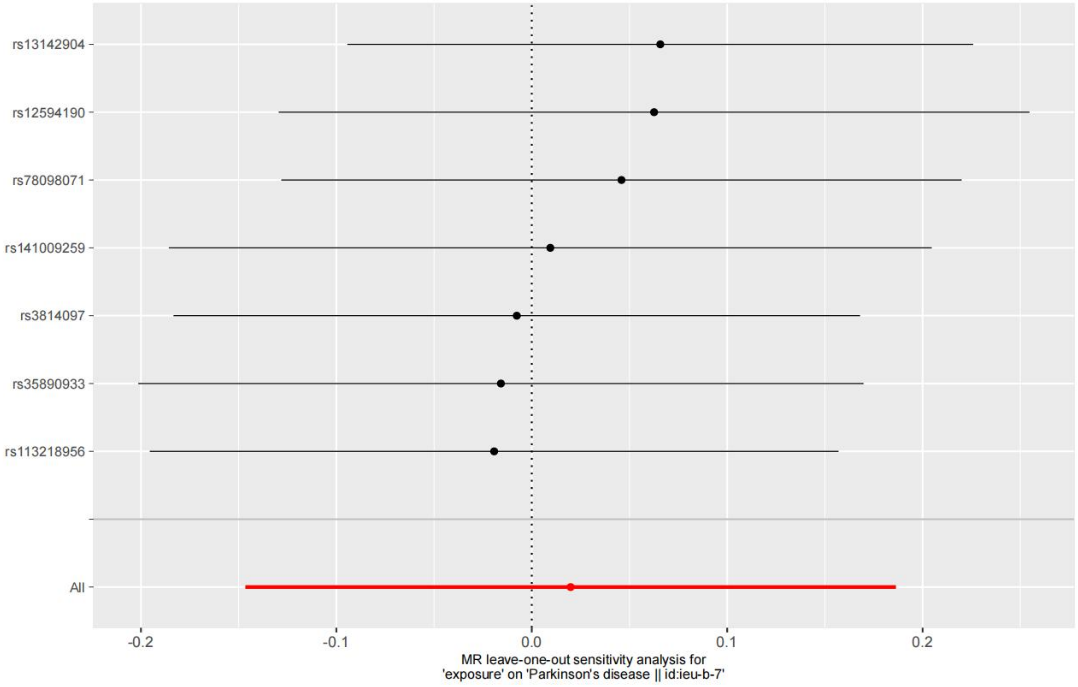

C. MR leave-one-out sensitivity analysis for MIF on PD

eFigure 71. MIG-associated SNPs with risk of PD

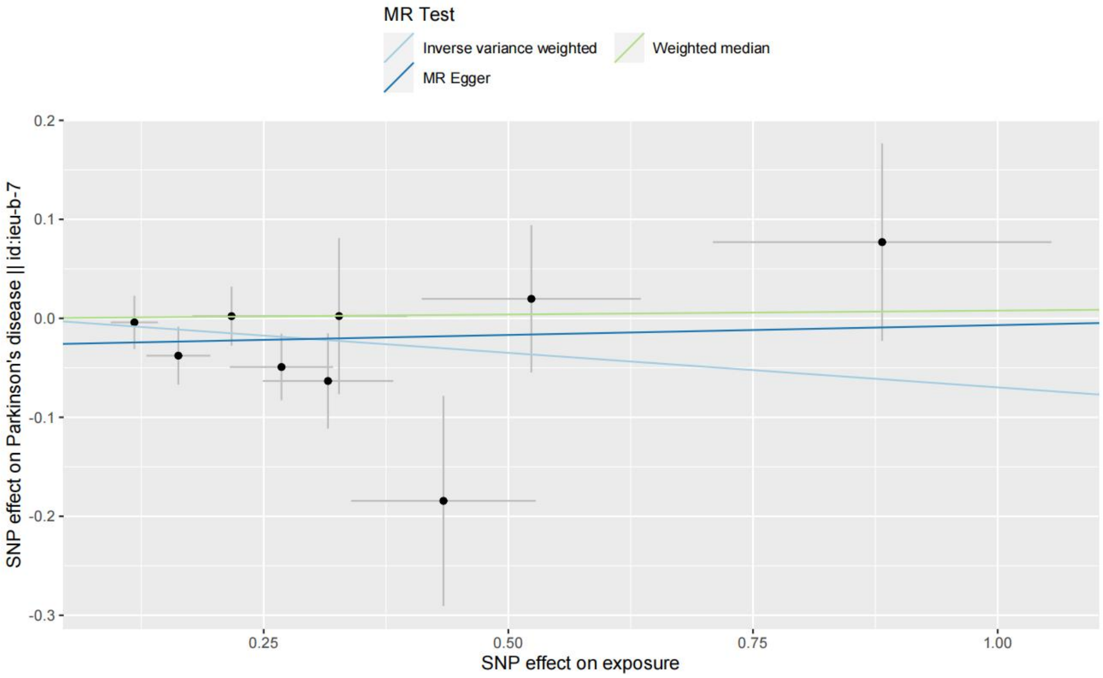

A. Scatter plot of MIG with risk of PD

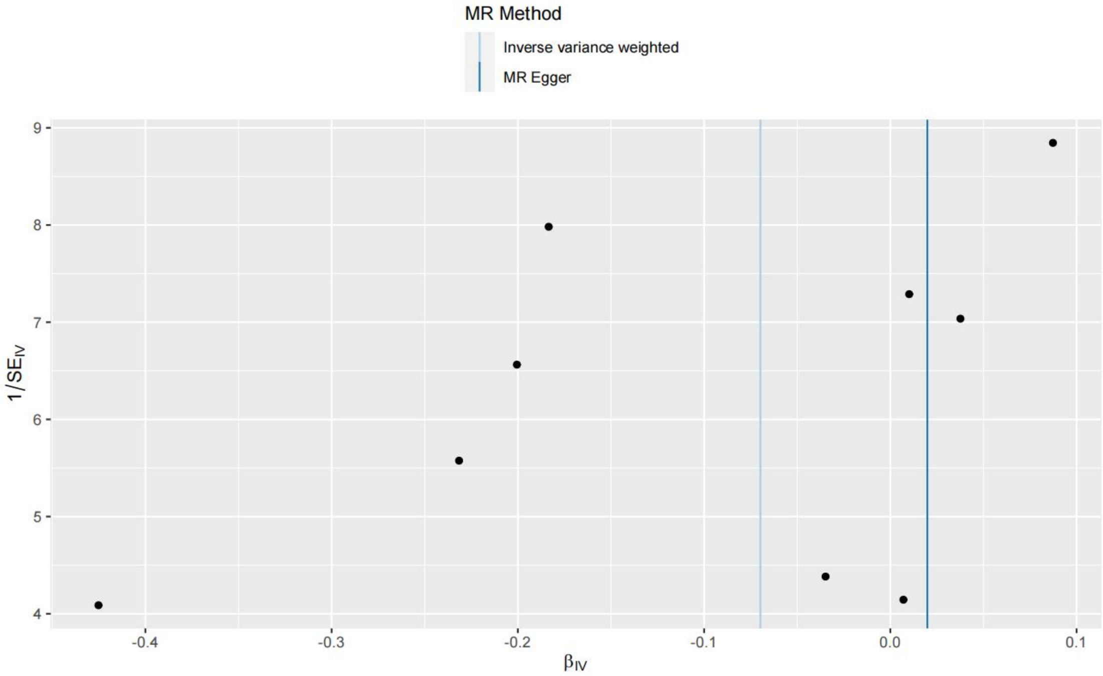

B. Funnel plot of MIG instruments strength on PD

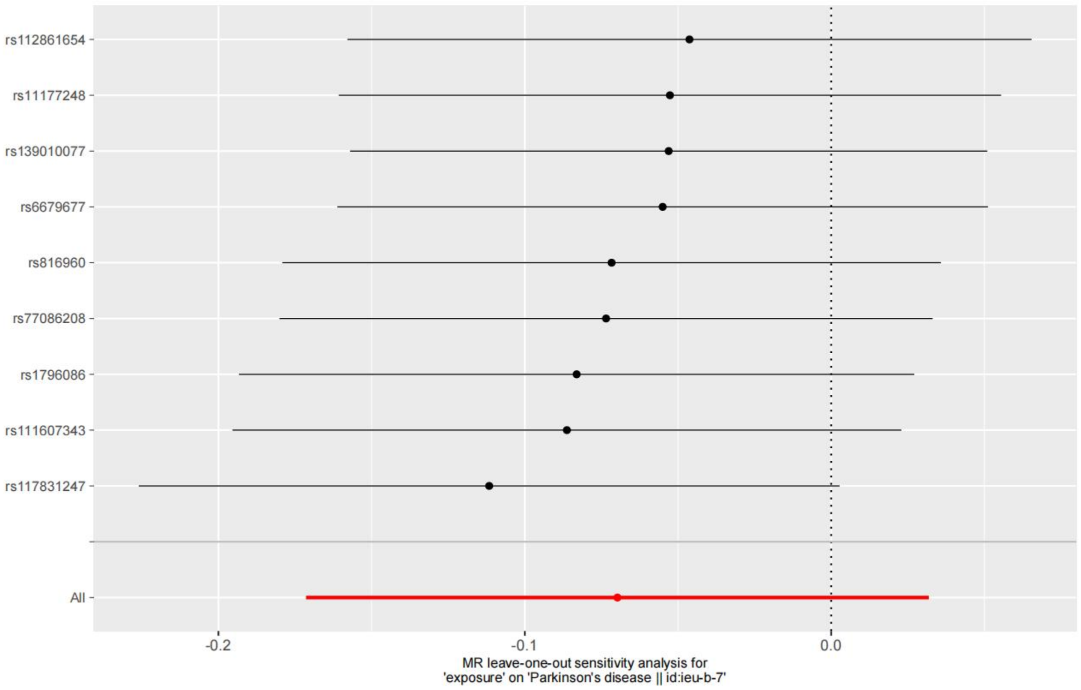

C. MR leave-one-out sensitivity analysis for MIG on PD

eFigure 72. MIP-1A-associated SNPs with risk of PD

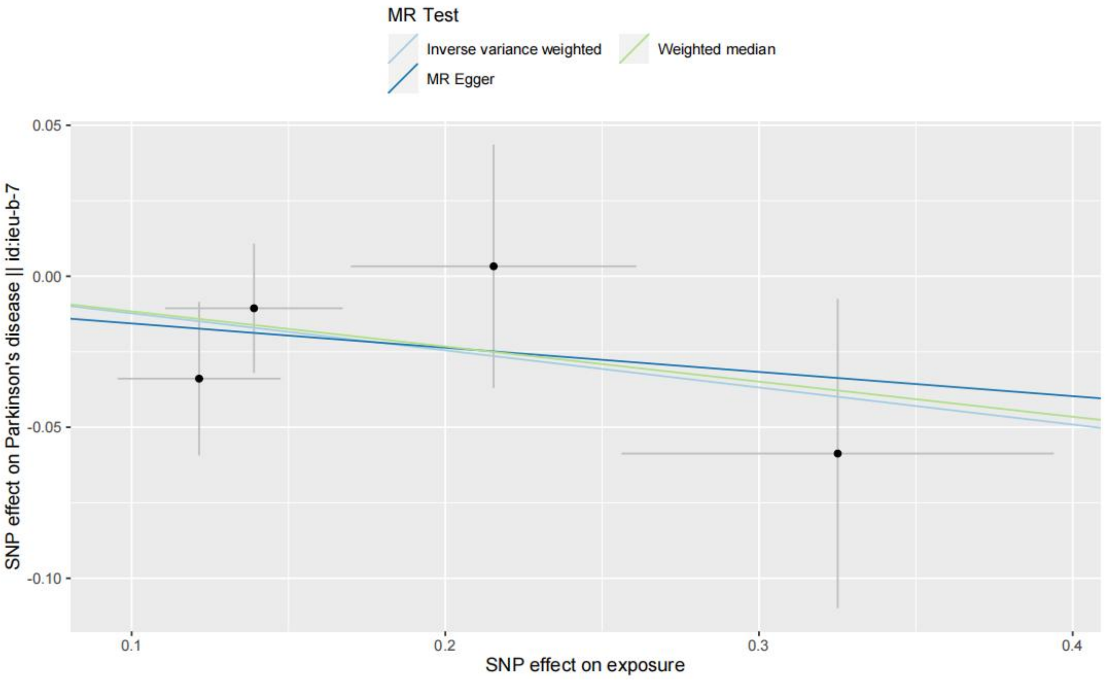

A. Scatter plot of MIP-1A with risk of PD

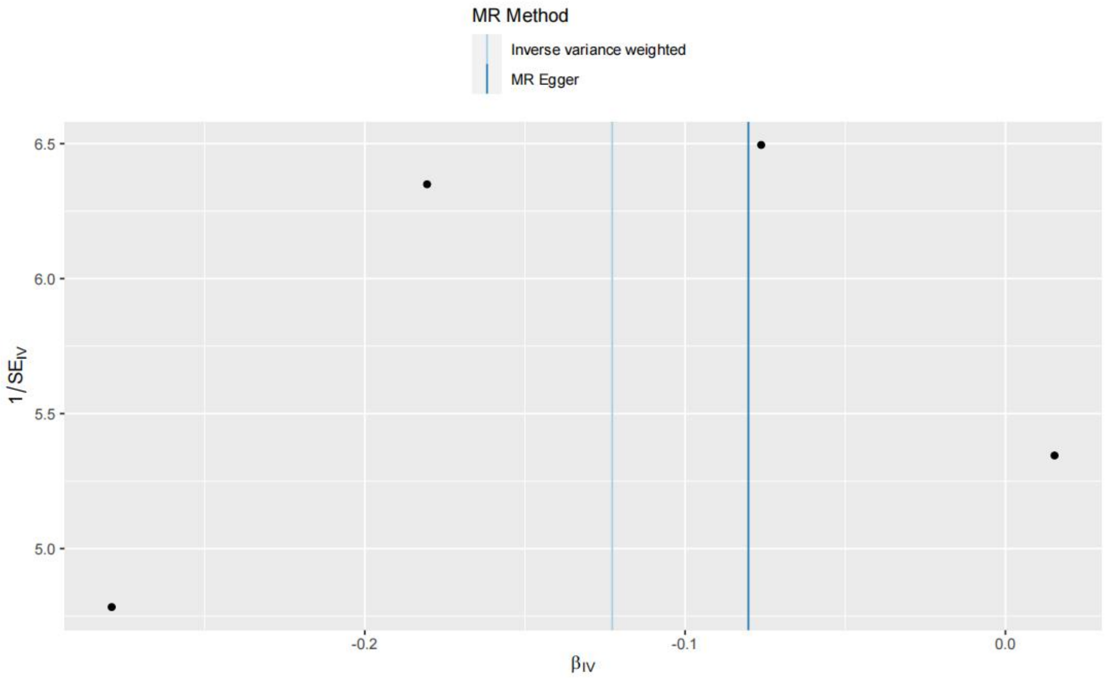

B. Funnel plot of MIP-1A instruments strength on PD

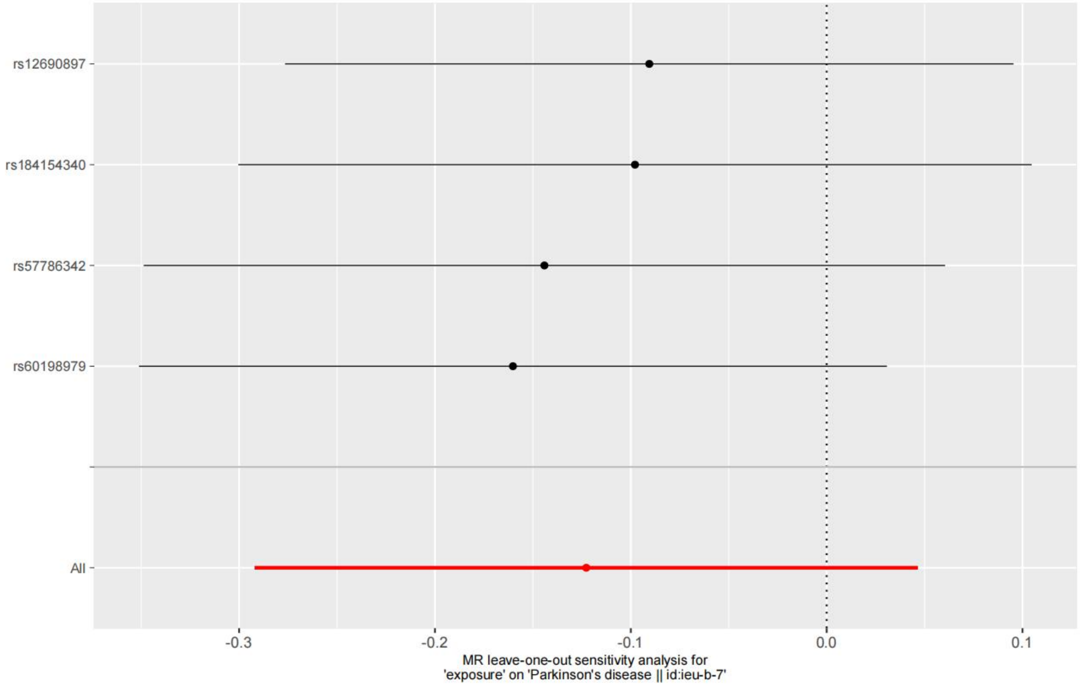

C. MR leave-one-out sensitivity analysis for MIP-1A on PD

eFigure 73. MIP-1B-associated SNPs with risk of PD

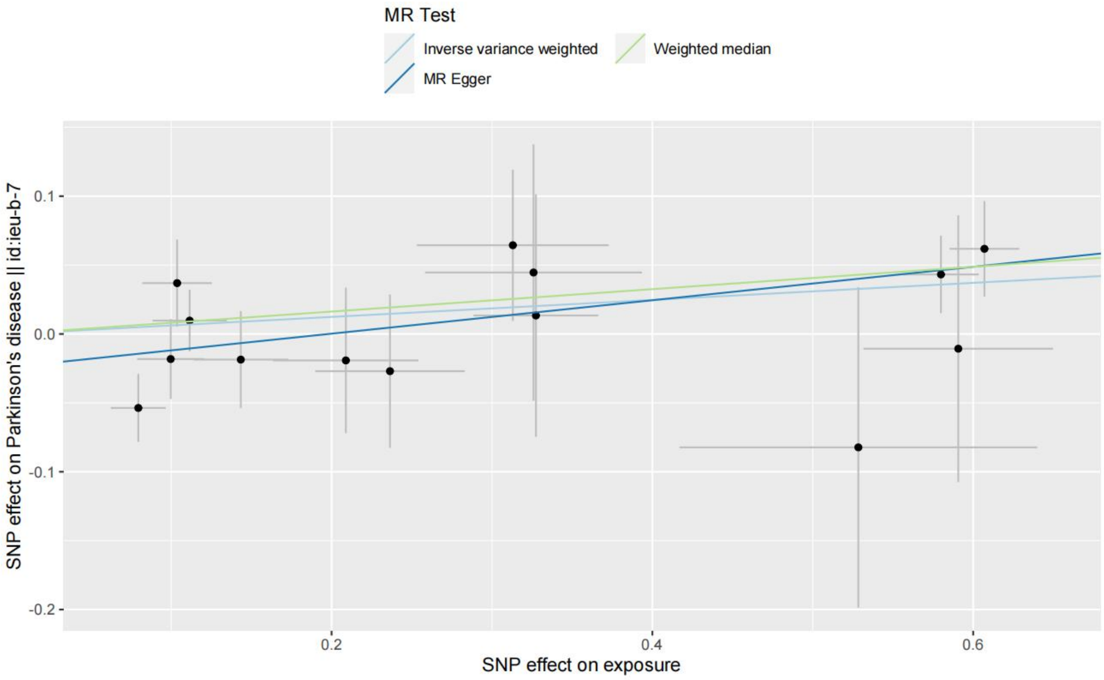

A. Scatter plot of MIP-1B with risk of PD

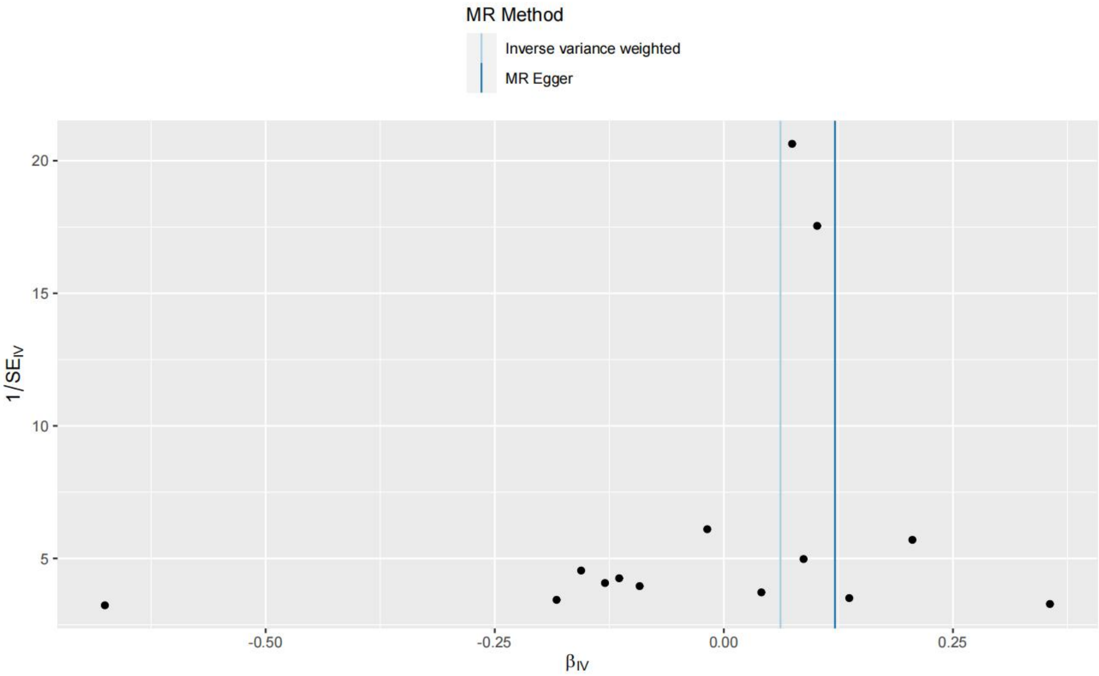

B. Funnel plot of MIP-1B instruments strength on PD

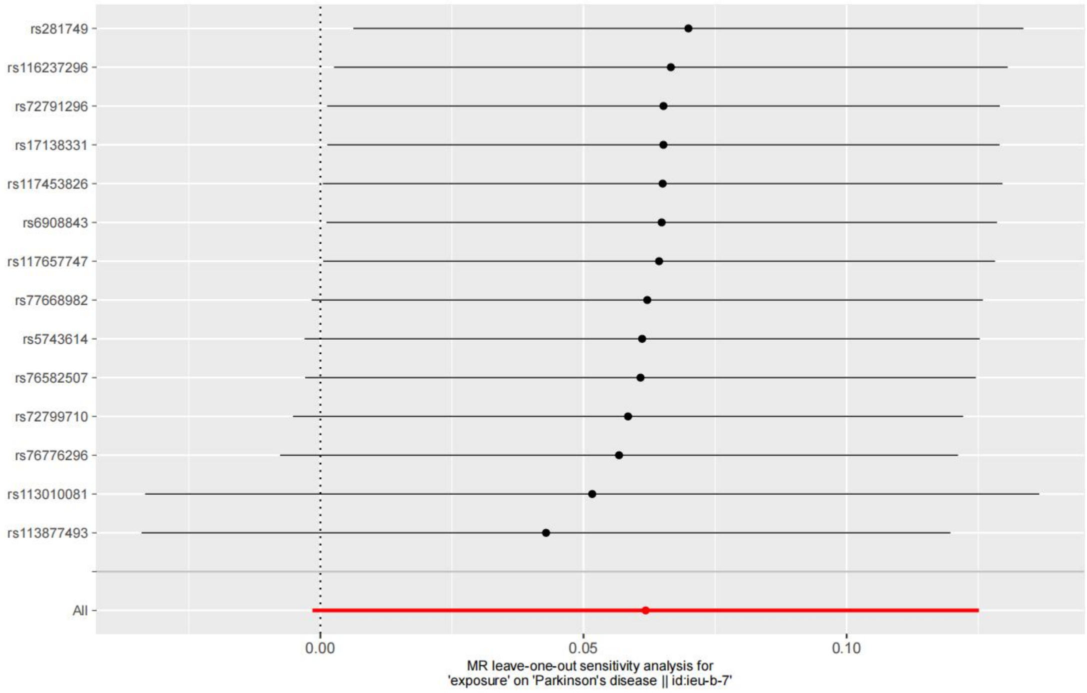

C. MR leave-one-out sensitivity analysis for MIP-1B on PD

eFigure 74. PDGF-BB-associated SNPs with risk of PD

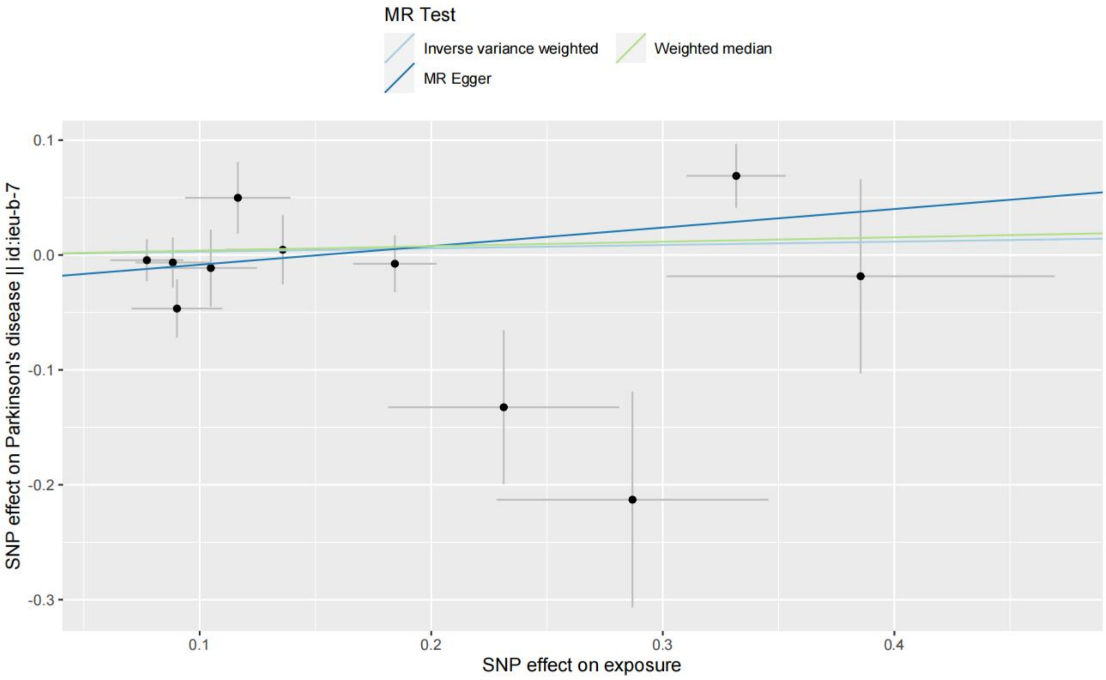

A. Scatter plot of PDGF-BB with risk of PD

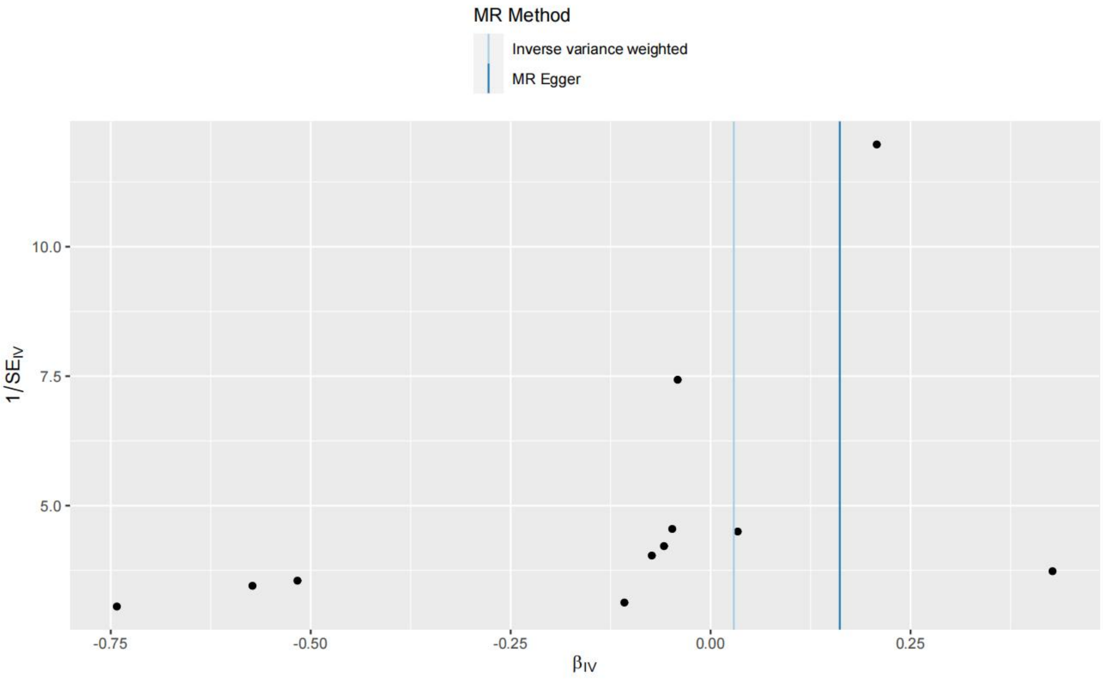

B. Funnel plot of PDGF-BB instruments strength on PD

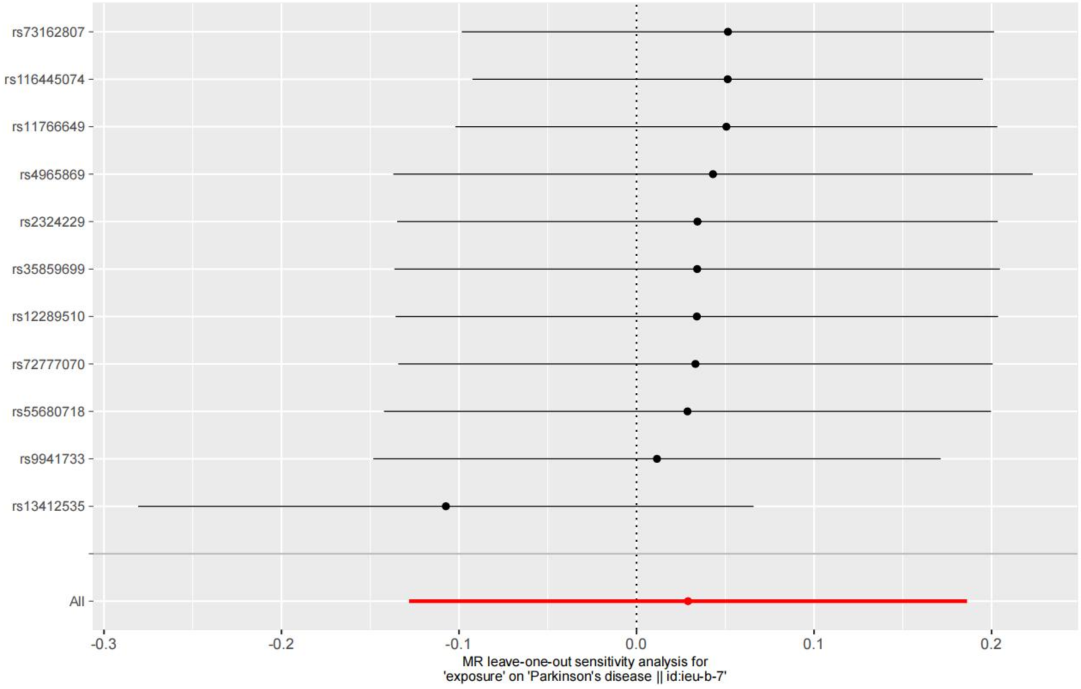

C. MR leave-one-out sensitivity analysis for PDGF-BB on PD

eFigure 75. RANTES-associated SNPs with risk of PD

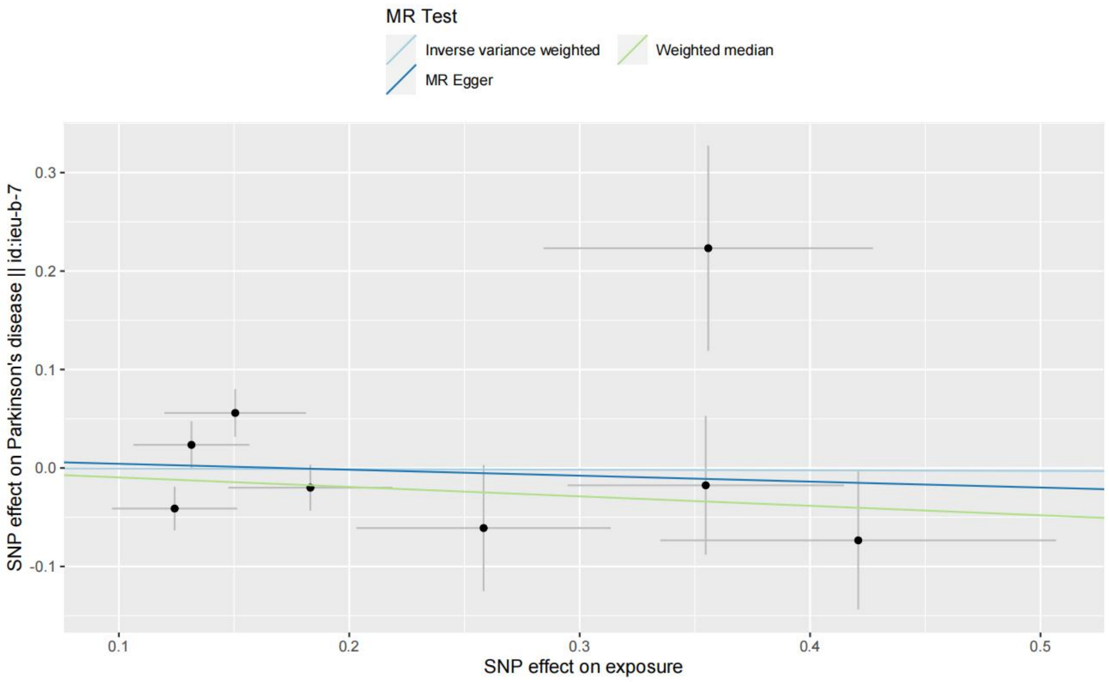

A. Scatter plot of RANTES with risk of PD

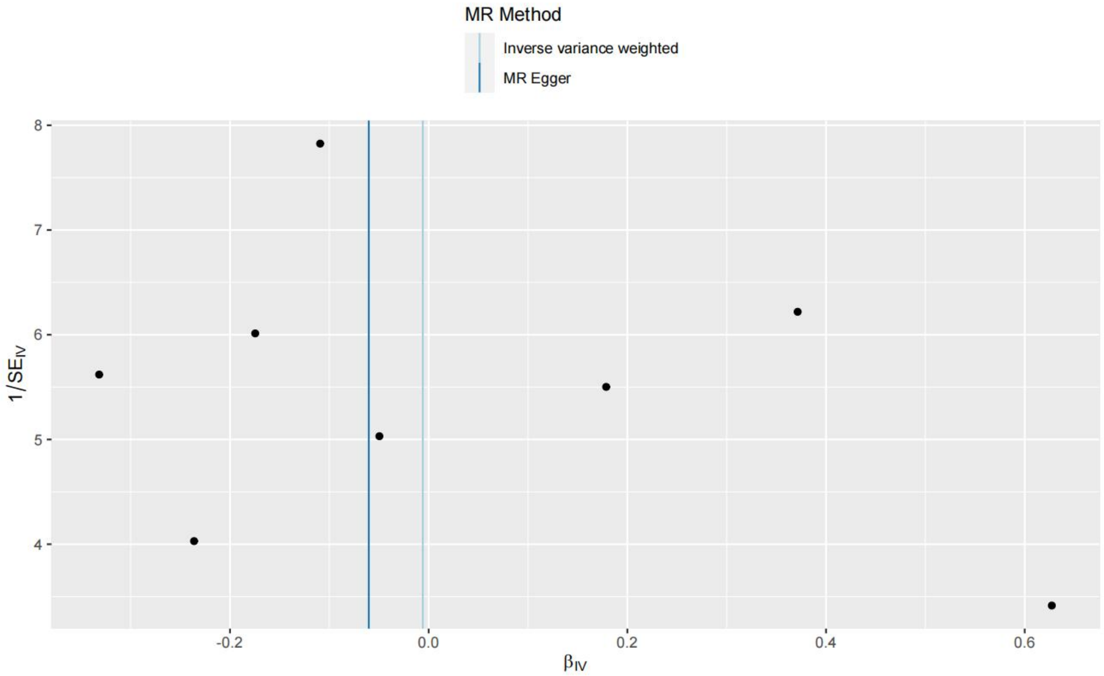

B. Funnel plot of RANTES instruments strength on PD

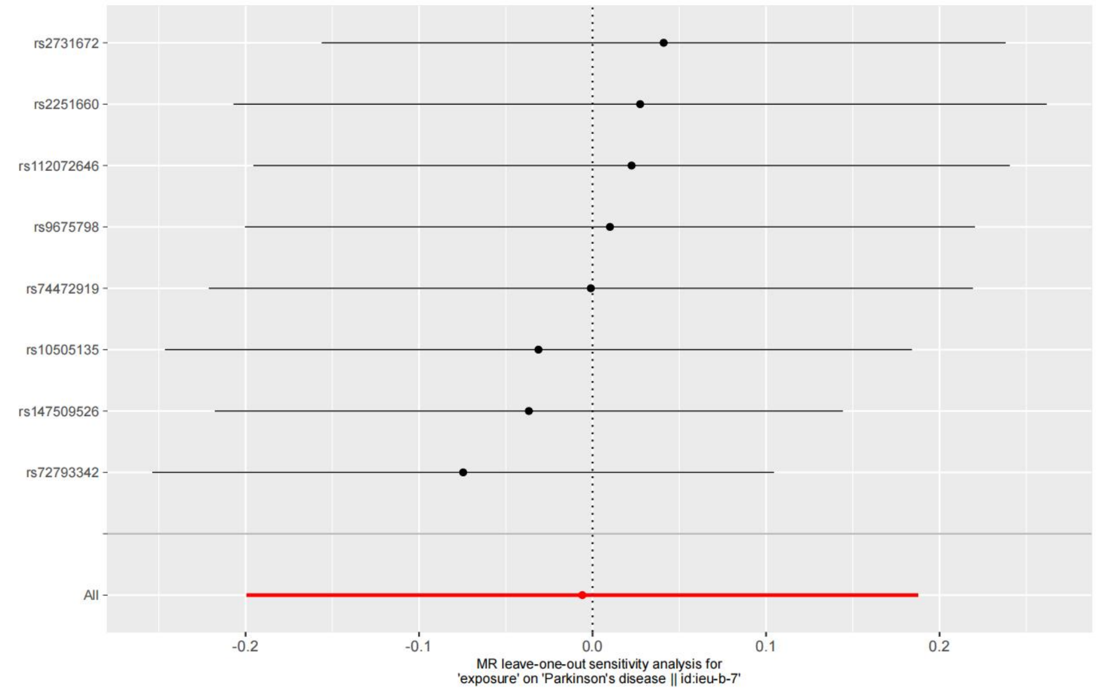

C. MR leave-one-out sensitivity analysis for RANTES on PD

eFigure 76. SCF-associated SNPs with risk of PD

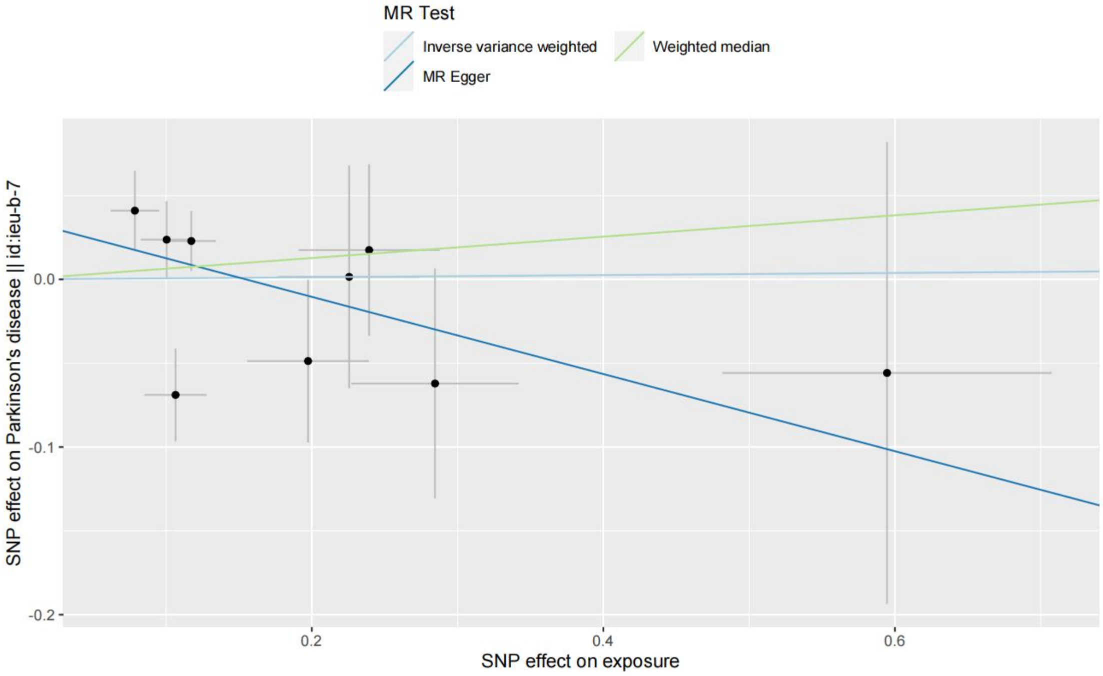

A. Scatter plot of SCF with risk of PD

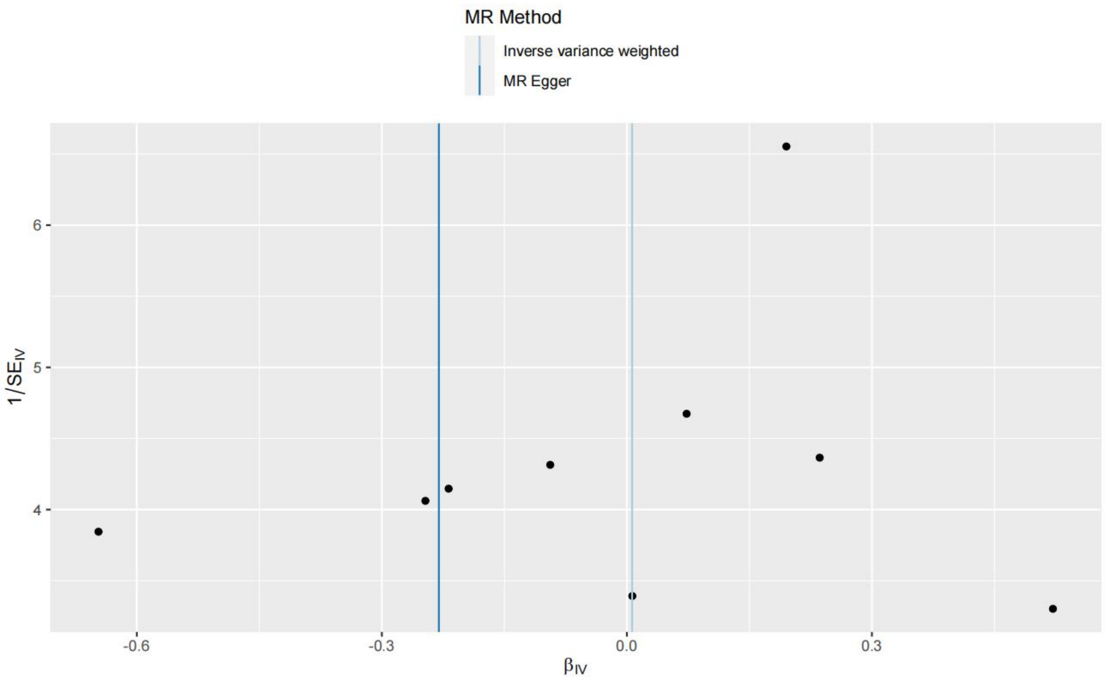

B. Funnel plot of SCF instruments strength on PD

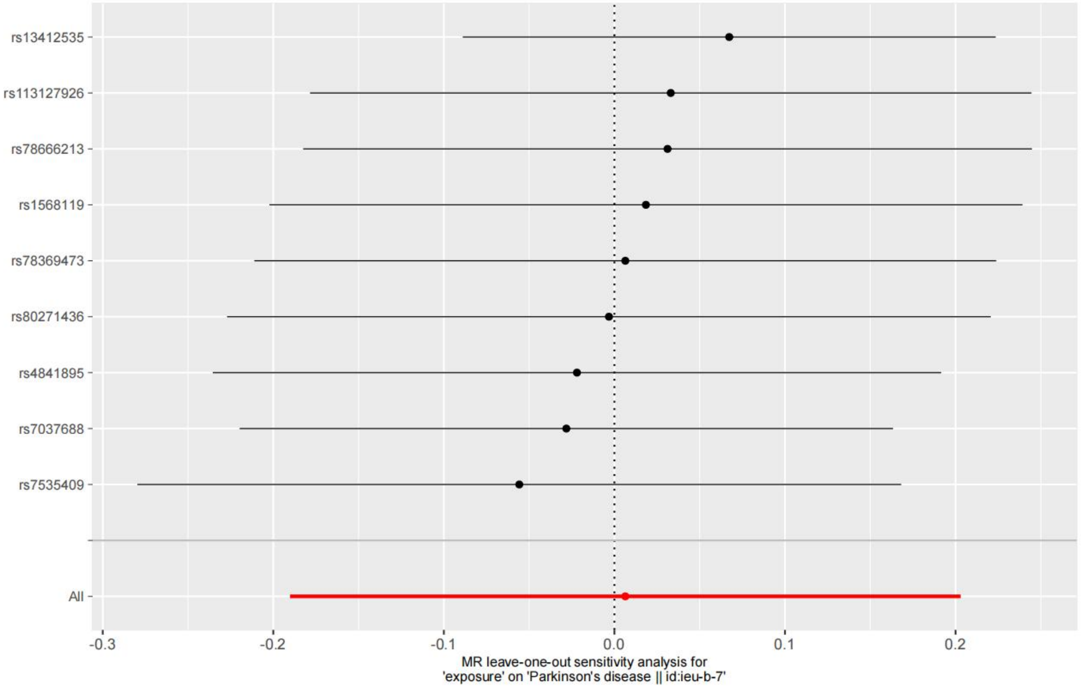

C. MR leave-one-out sensitivity analysis for SCF on PD

eFigure 77. SCGFβ-associated SNPs with risk of PD

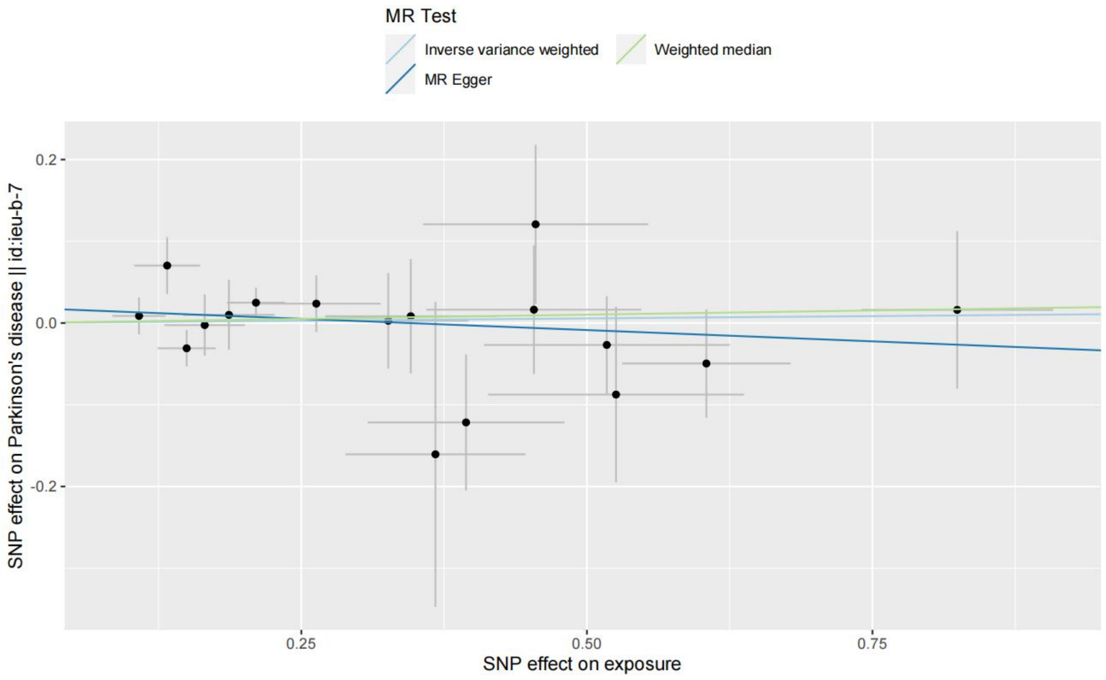

A. Scatter plot of SCGFβ with risk of PD

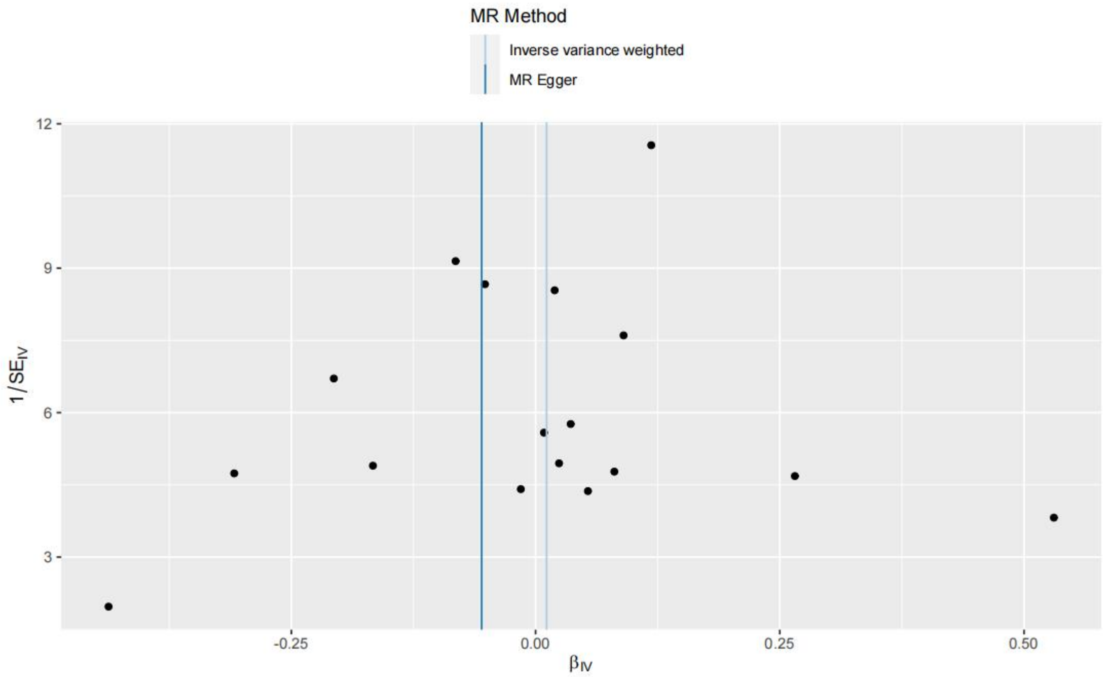

B. Funnel plot of SCGFβ instruments strength on PD

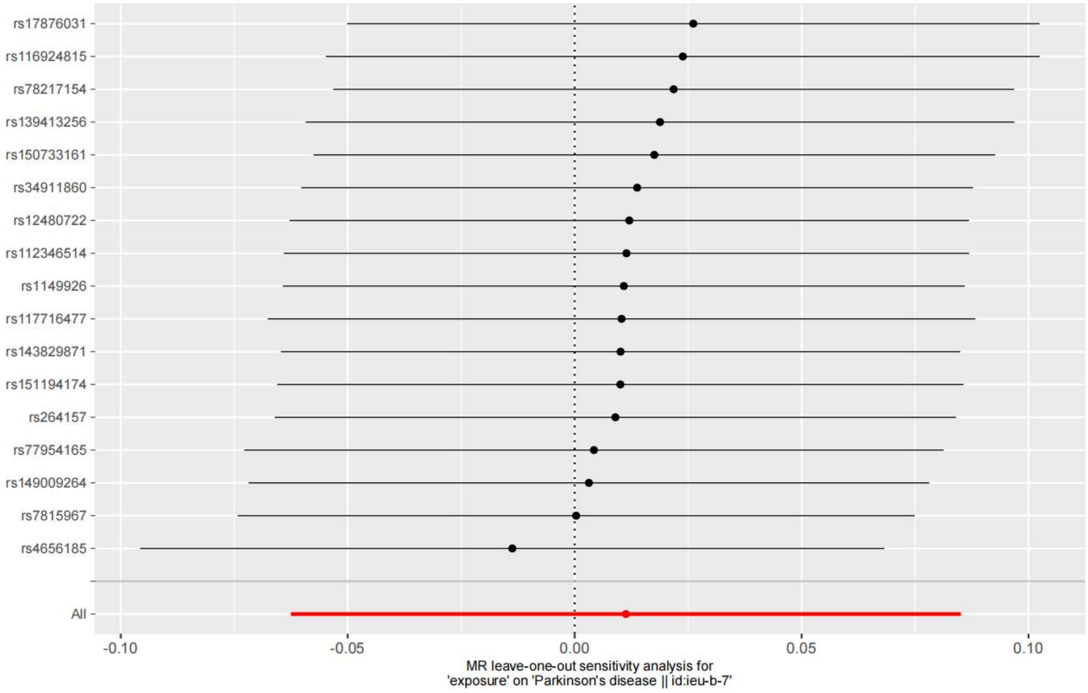

C. MR leave-one-out sensitivity analysis for SCGFβ on PD

eFigure 78. SDF-1A-associated SNPs with risk of PD

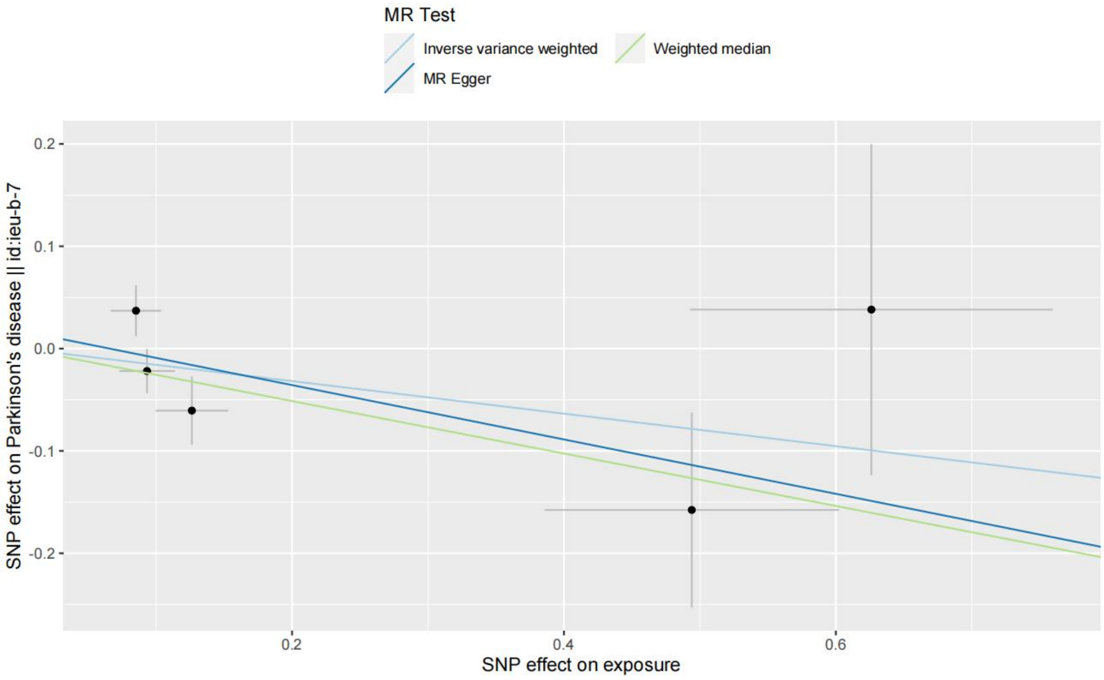

A. Scatter plot of SDF-1A with risk of PD

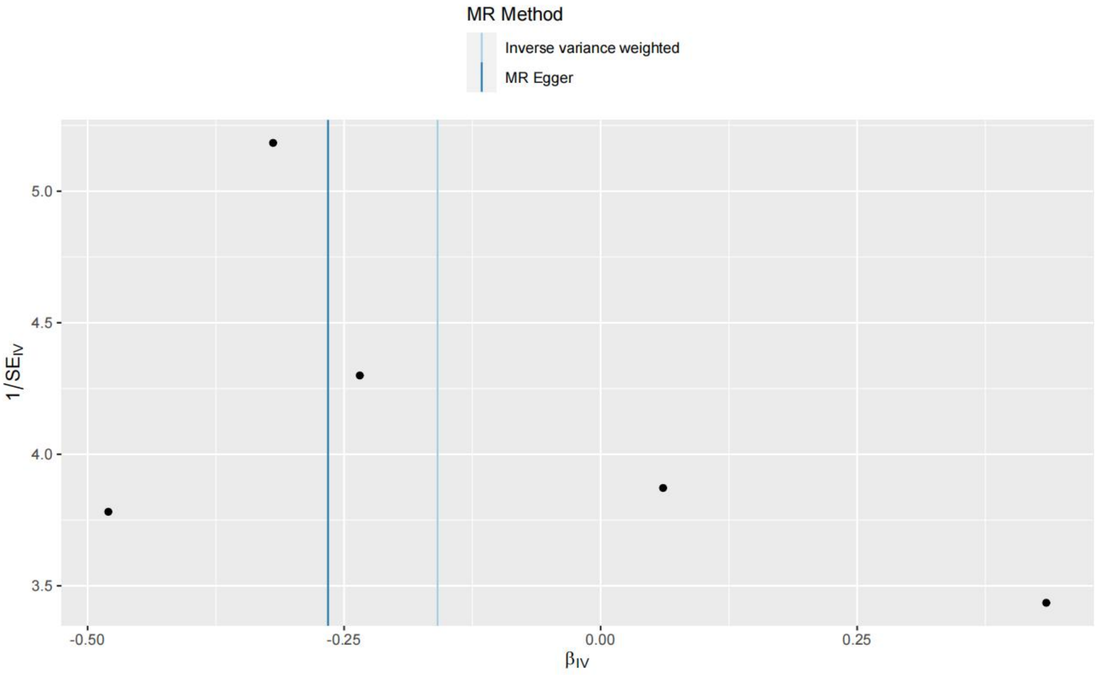

B. Funnel plot of SDF-1A instruments strength on PD

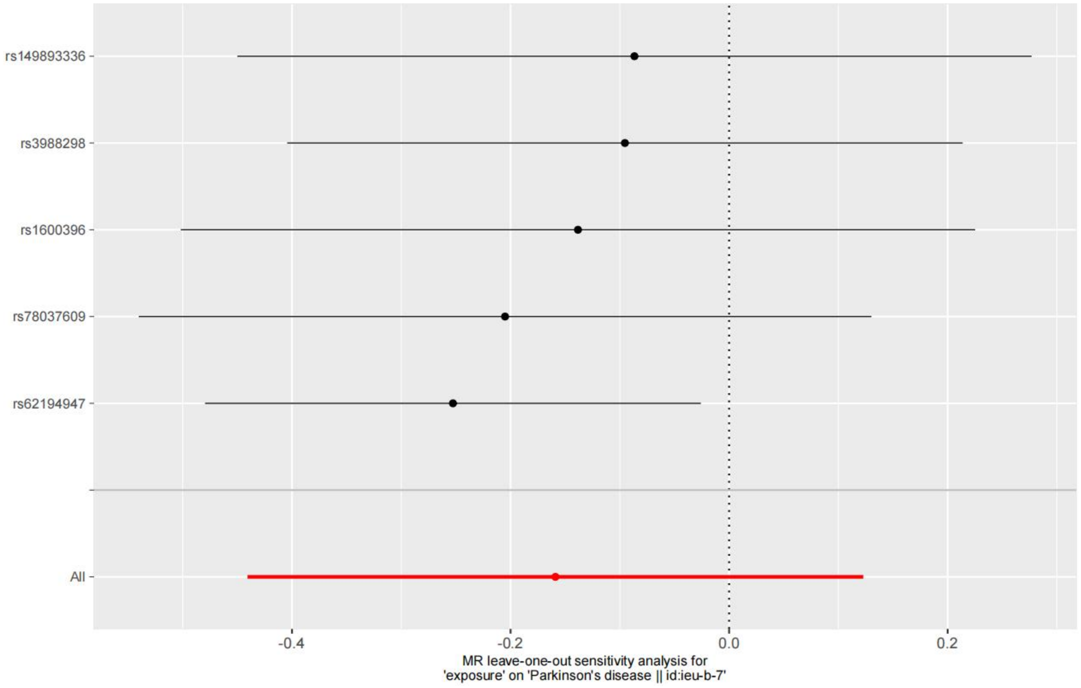

C. MR leave-one-out sensitivity analysis for SDF-1A on PD

eFigure 79. TNF-A-associated SNPs with risk of PD

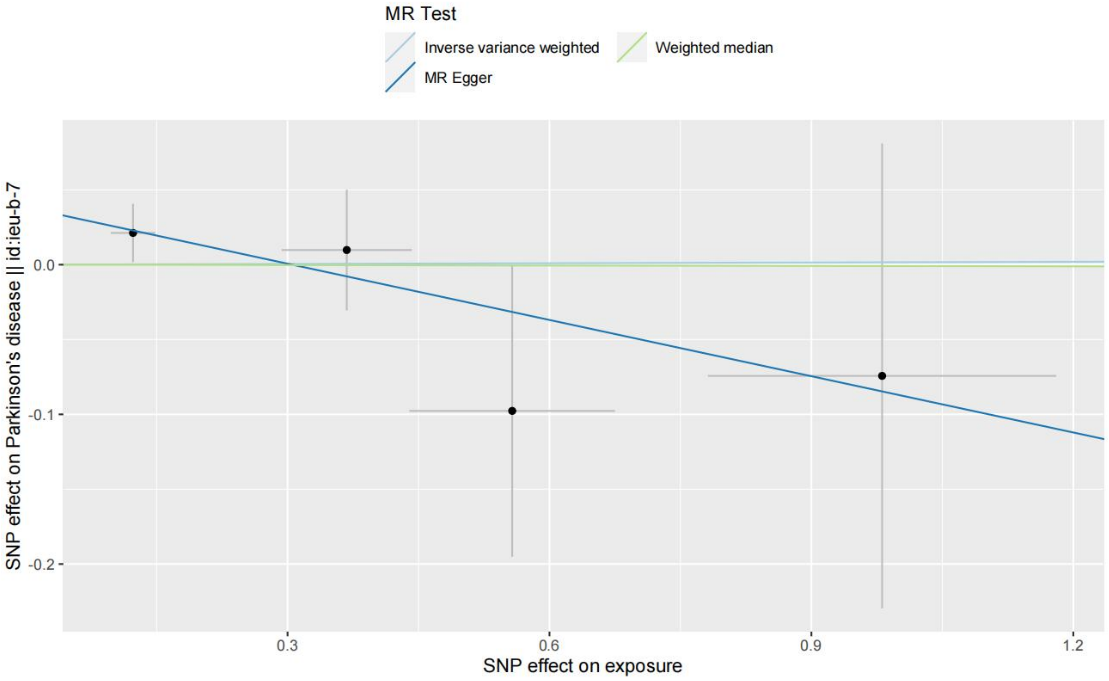

A. Scatter plot of TNF-A with risk of PD

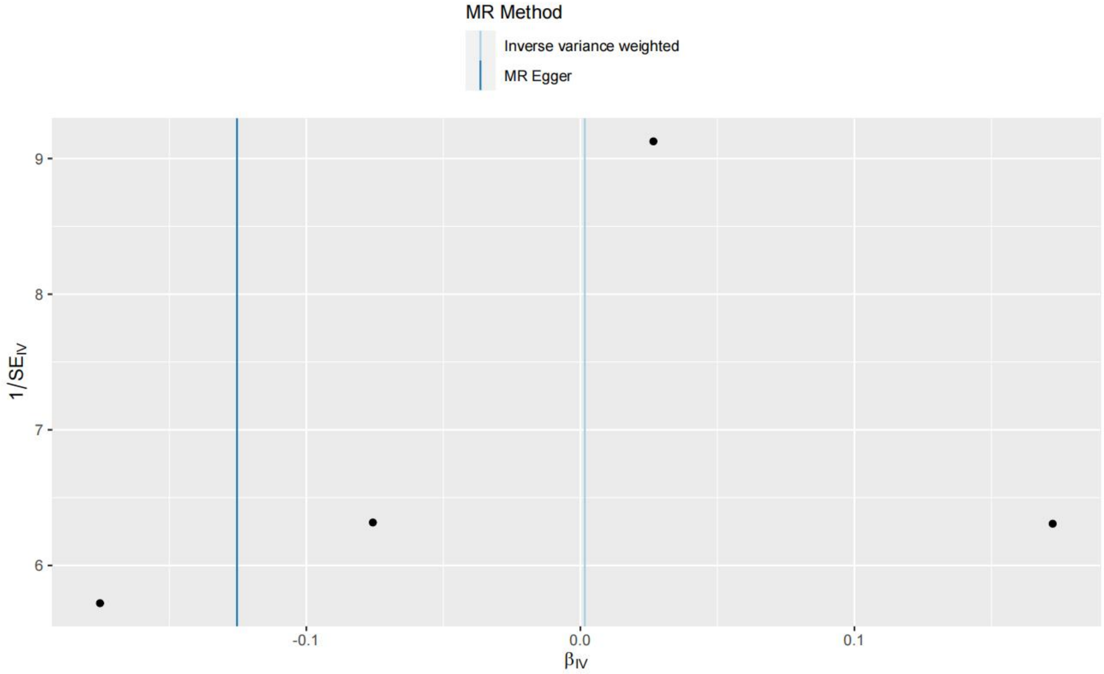

B. Funnel plot of TNF-A instruments strength on PD

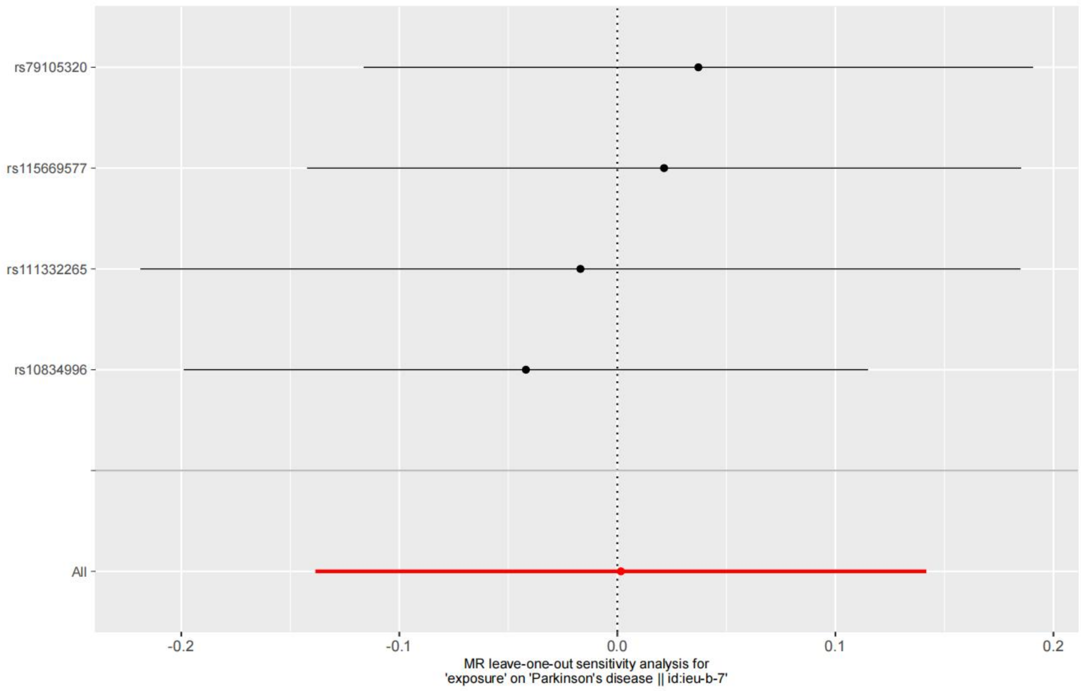

C. MR leave-one-out sensitivity analysis for TNF-A on PD

eFigure 80. TNF-B-associated SNPs with risk of PD

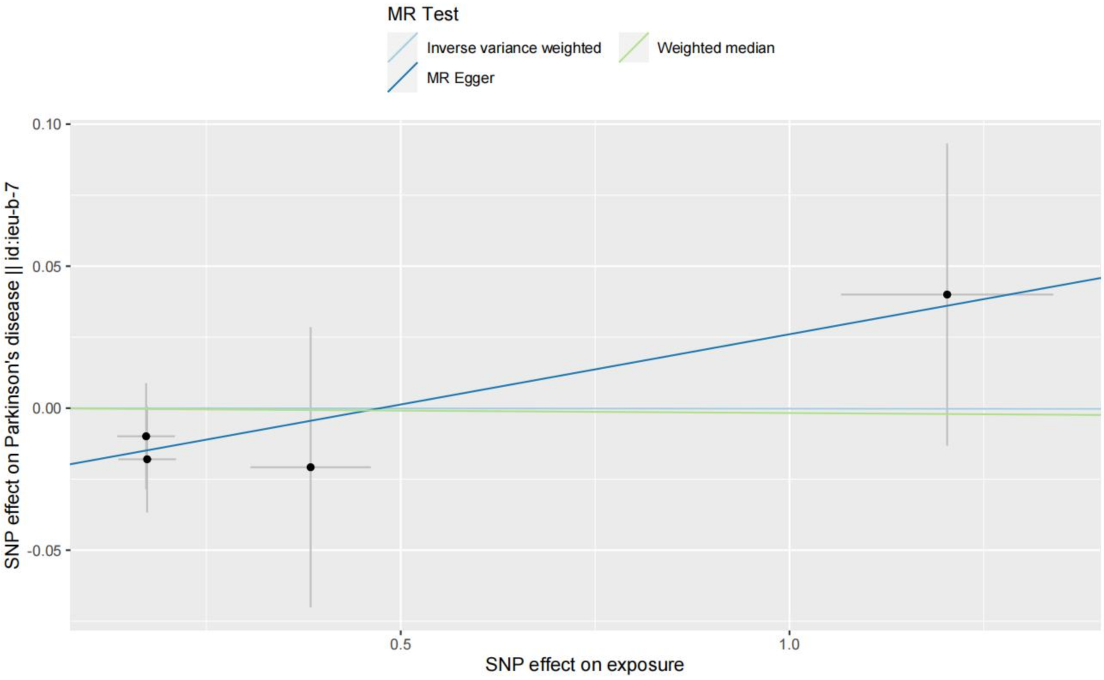

A. Scatter plot of TNF-B with risk of PD

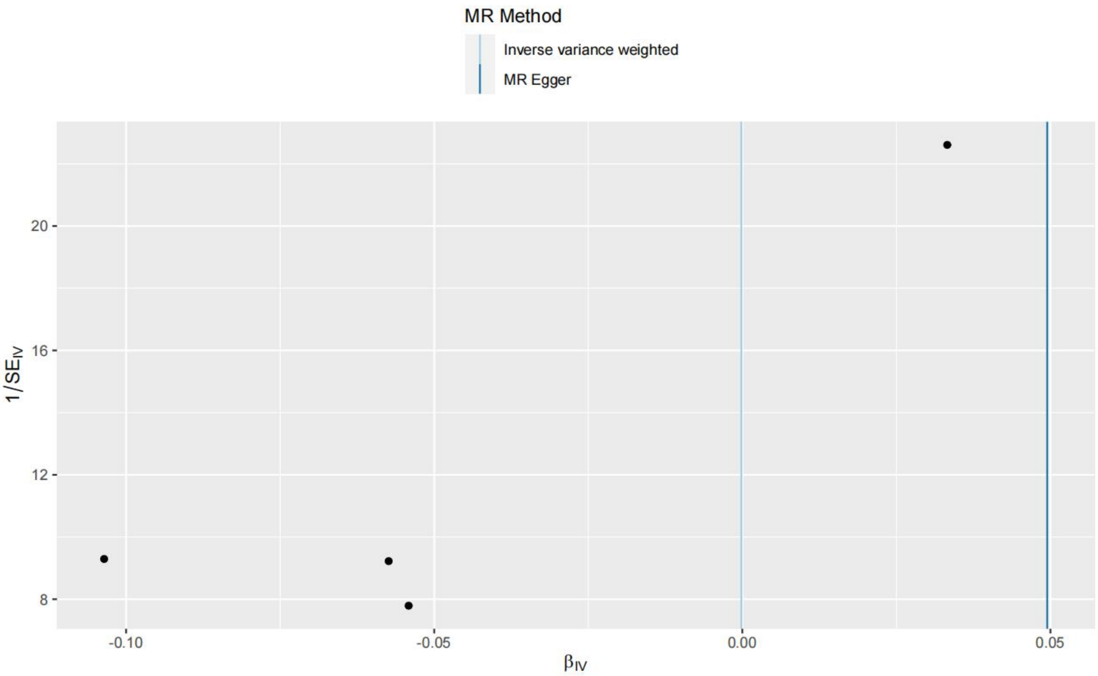

B. Funnel plot of TNF-B instruments strength on PD

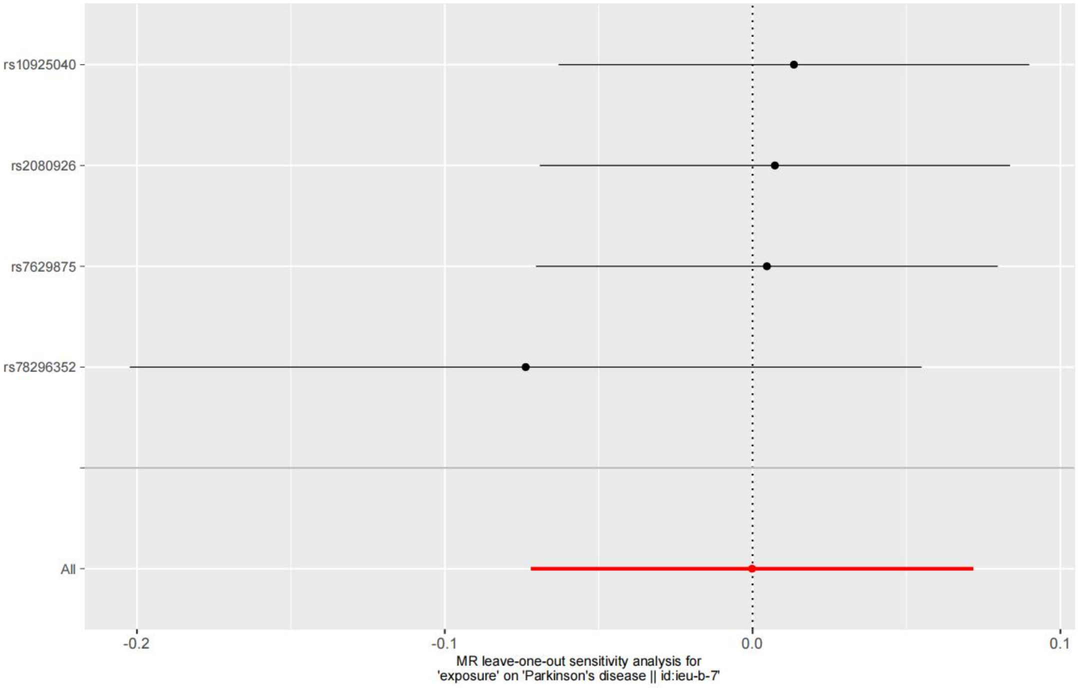

C. MR leave-one-out sensitivity analysis for TNF-B on PD

eFigure 81. TRAIL-associated SNPs with risk of PD

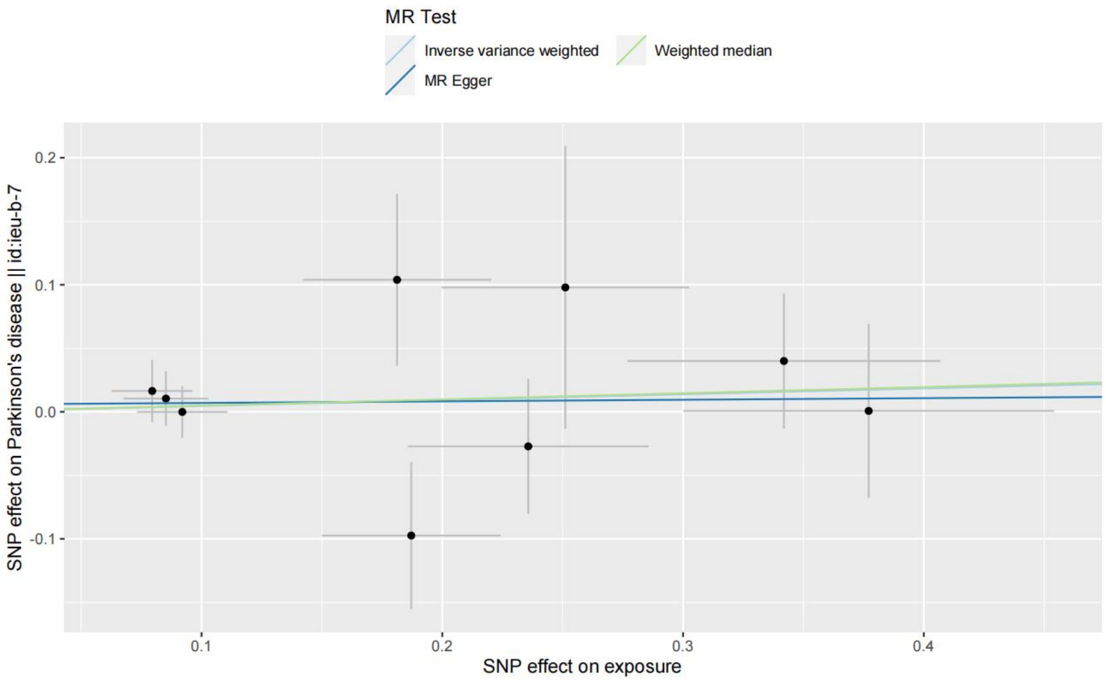

A. Scatter plot of TRAIL with risk of PD

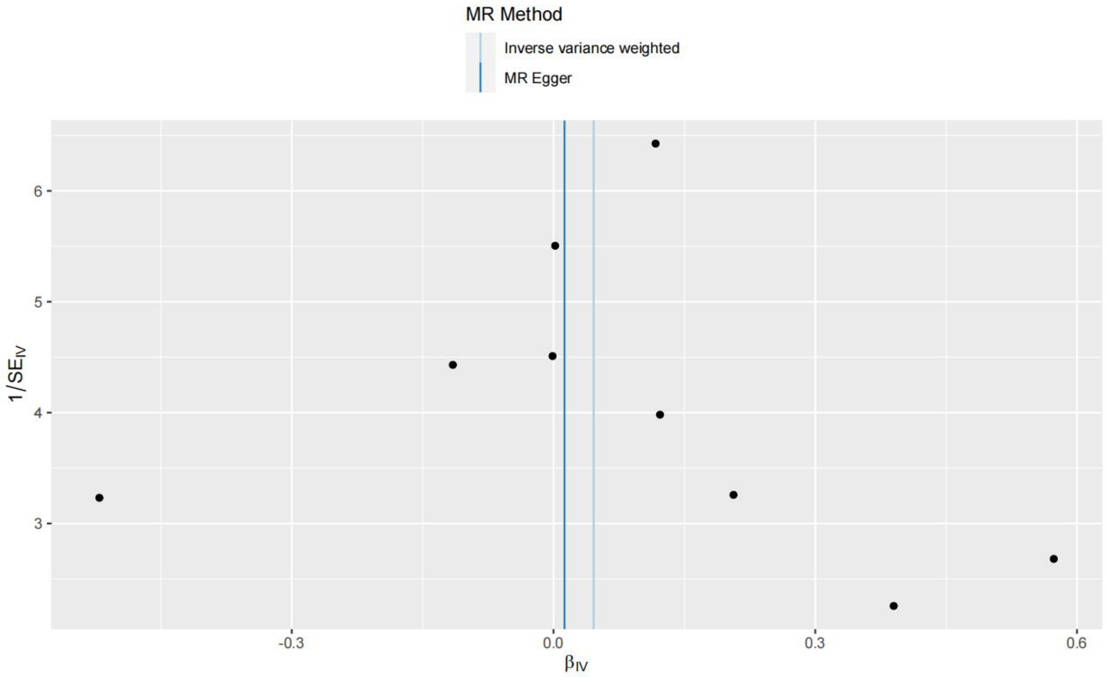

B. Funnel plot of TRAIL instruments strength on PD

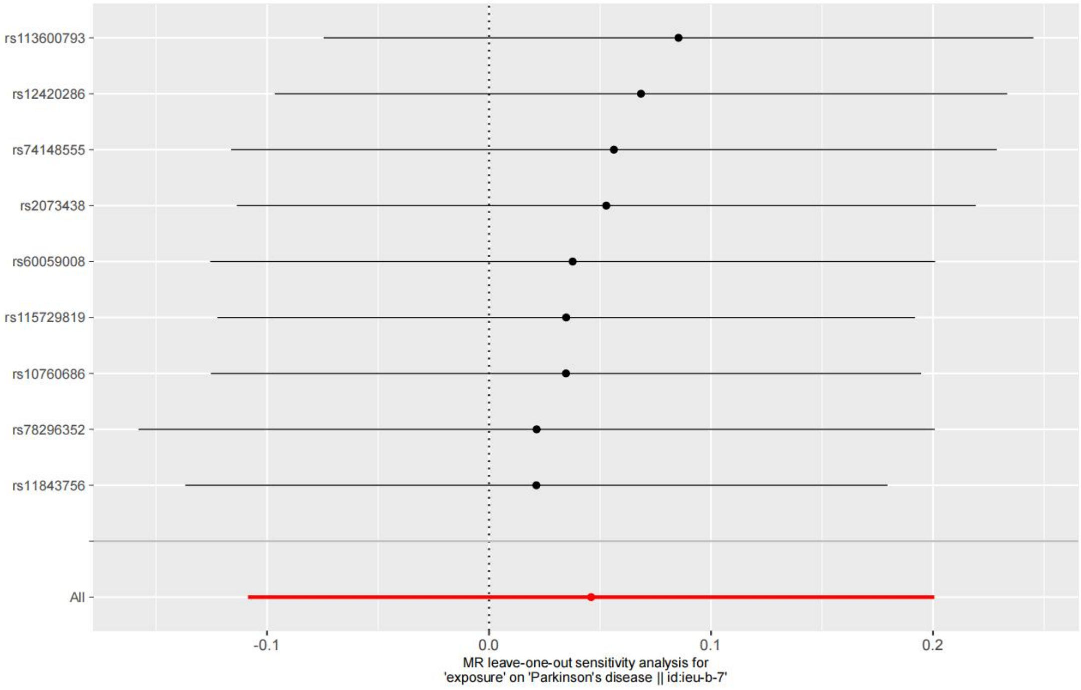

C. MR leave-one-out sensitivity analysis for TRAIL on PD

eFigure 82. VEGF-associated SNPs with risk of PD

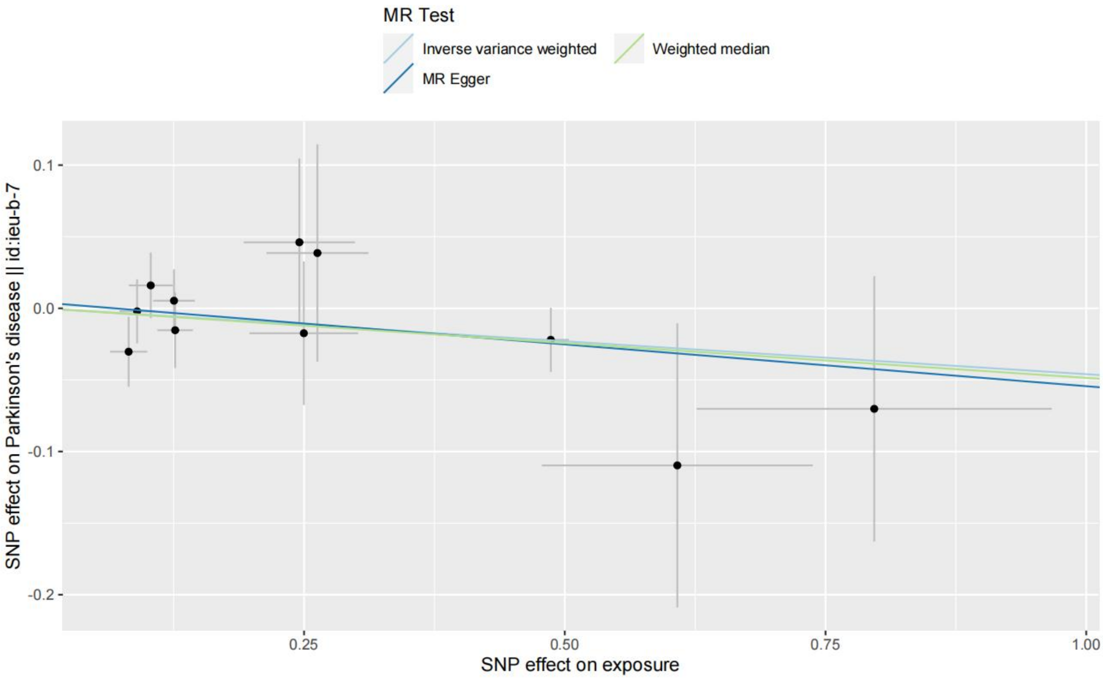

A. Scatter plot of VEGF with risk of PD

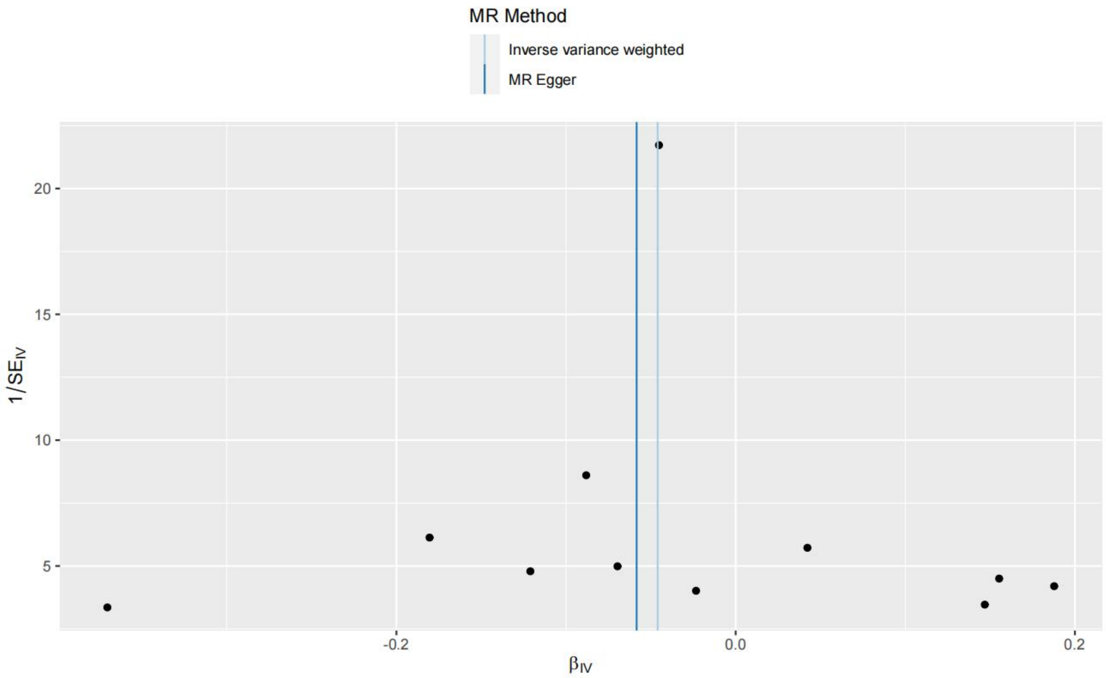

B. Funnel plot of VEGF instruments strength on PD

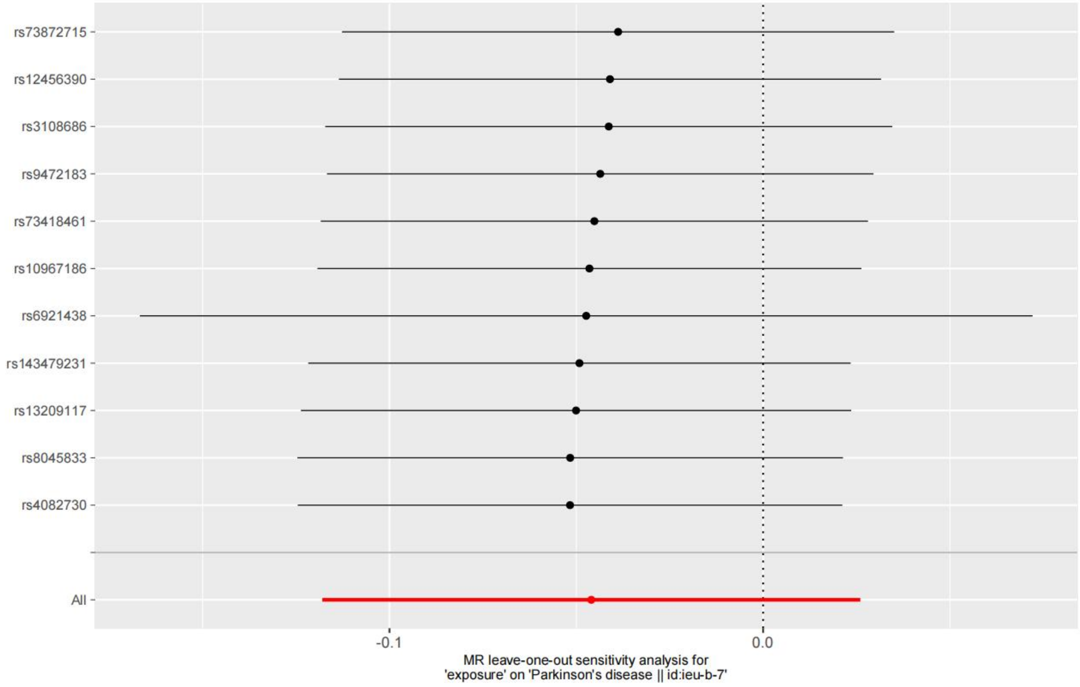

C. MR leave-one-out sensitivity analysis for VEGF on PD

eFigure 83. BNGF-associated SNPs with risk of ALS

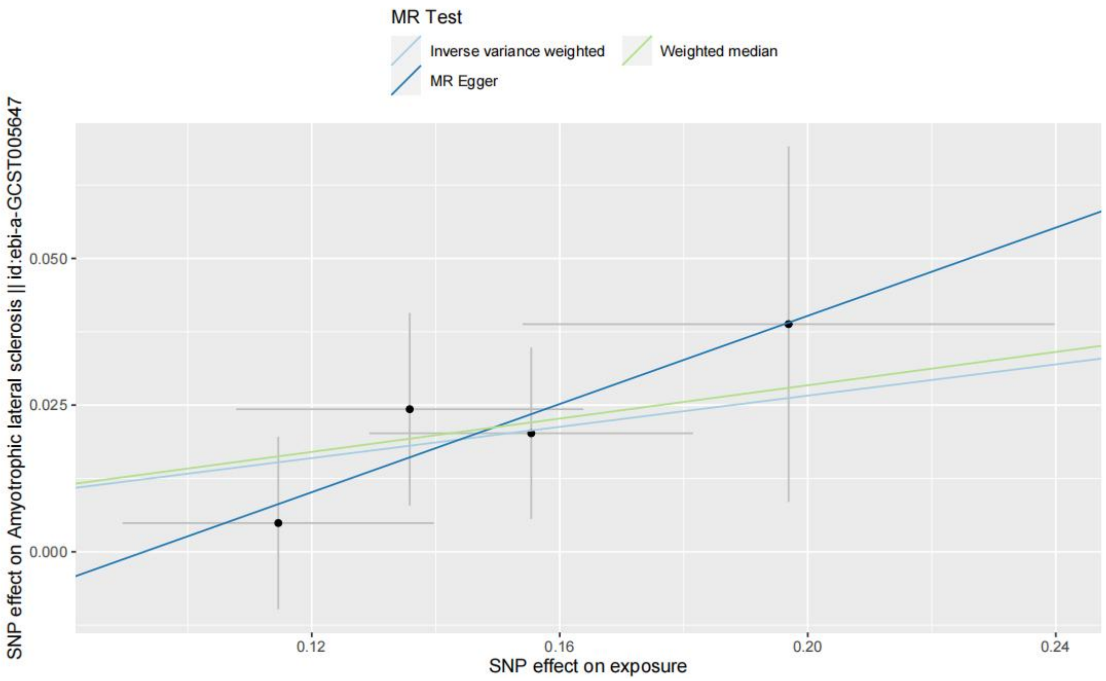

A. Scatter plot of BNGF with risk of ALS

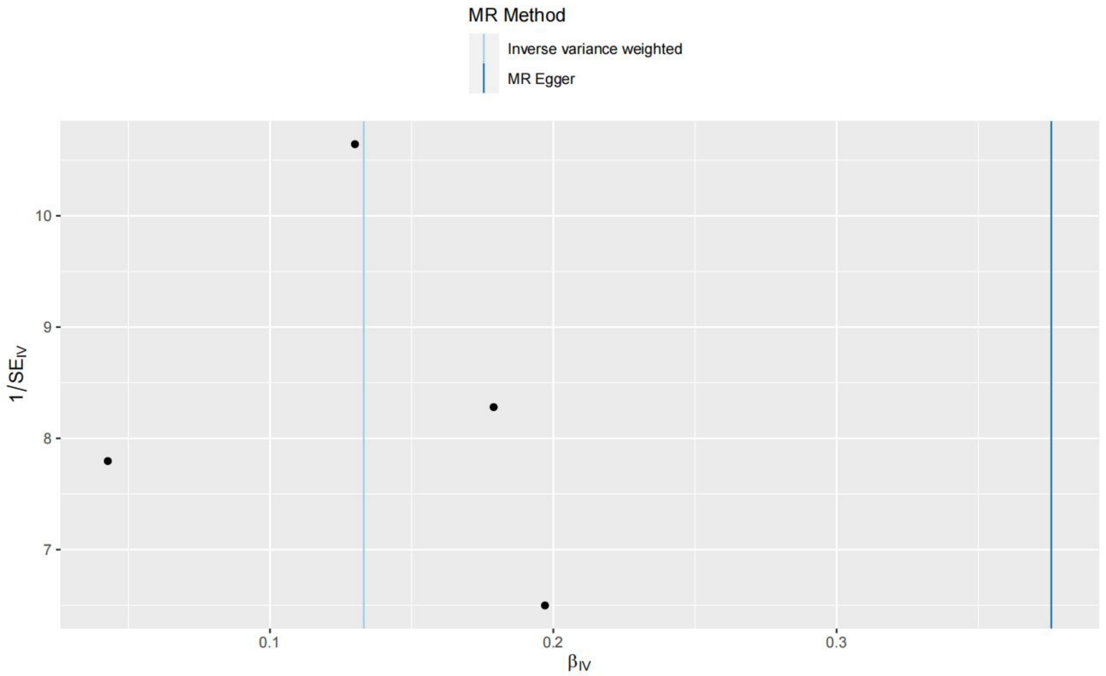

B. Funnel plot of BNGF instruments strength on ALS

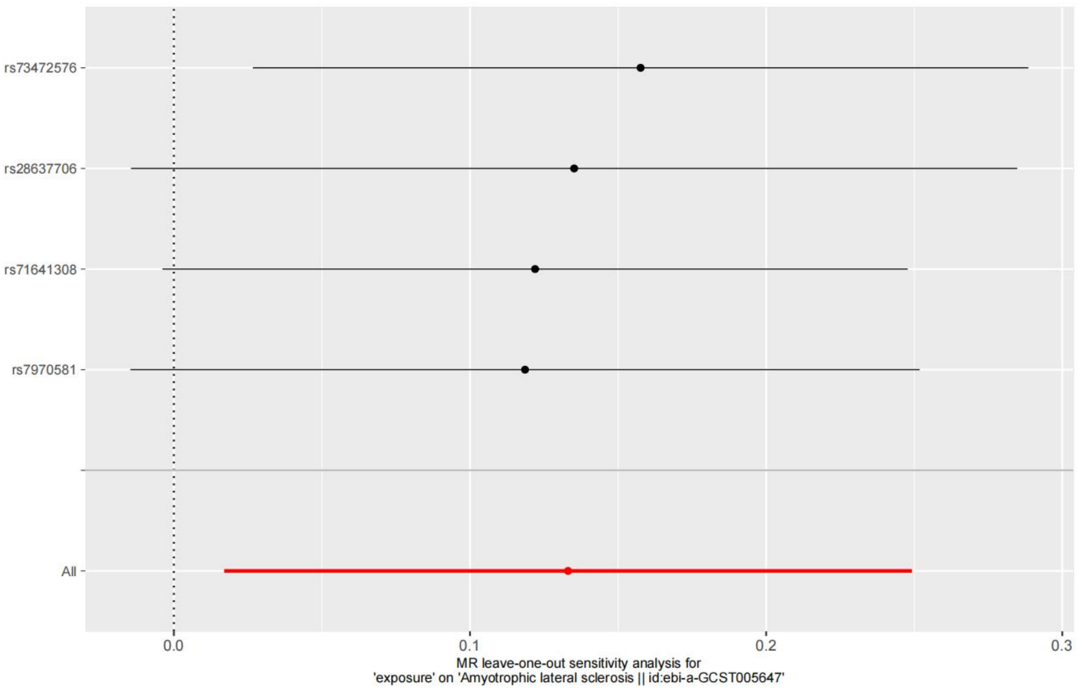

C. MR leave-one-out sensitivity analysis for BNGF on ALS

eFigure 84. CTACK-associated SNPs with risk of ALS

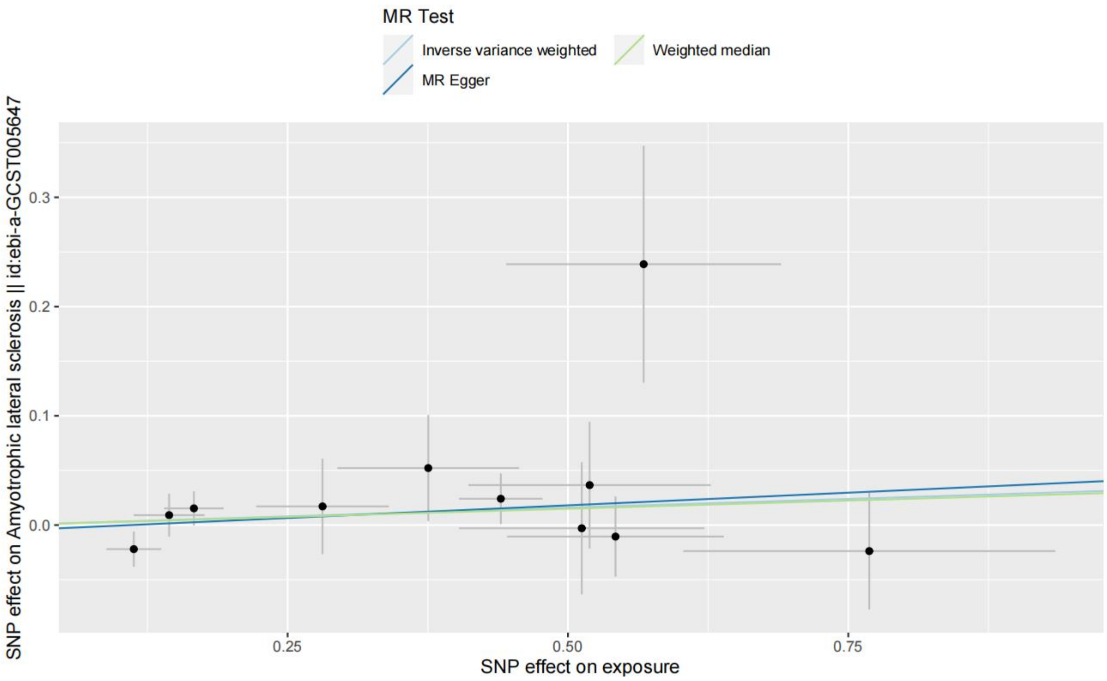

A. Scatter plot of CTACK with risk of ALS

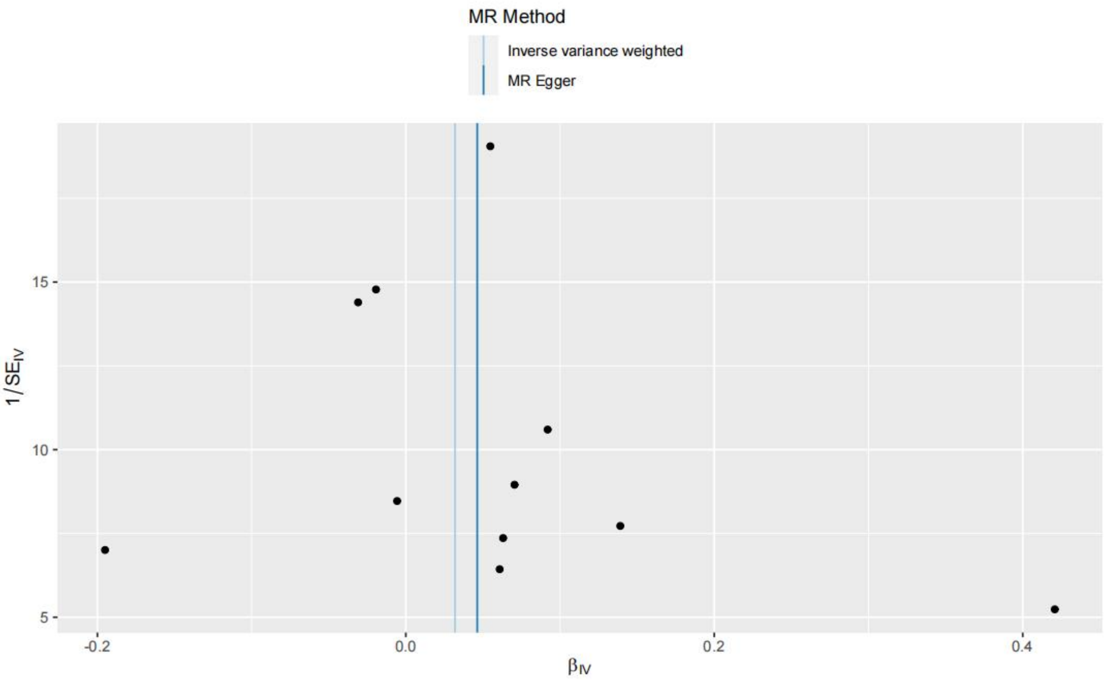

B. Funnel plot of CTACK instruments strength on ALS

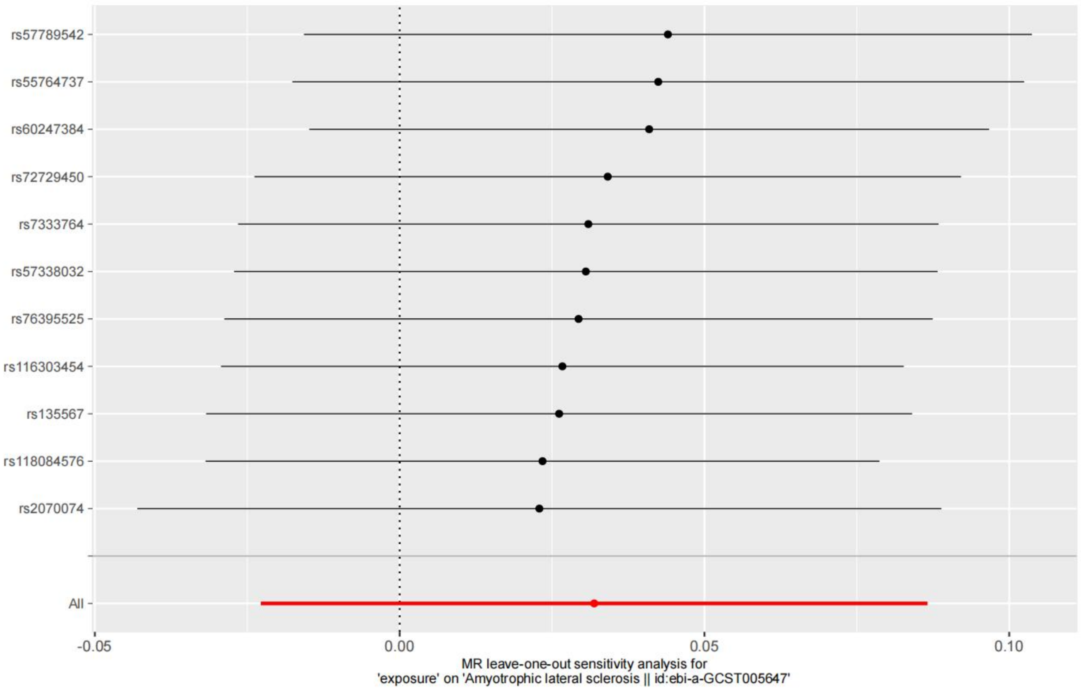

C. MR leave-one-out sensitivity analysis for CTACK-transferase on ALS

eFigure 85. EOTAXIN-associated SNPs with risk of ALS

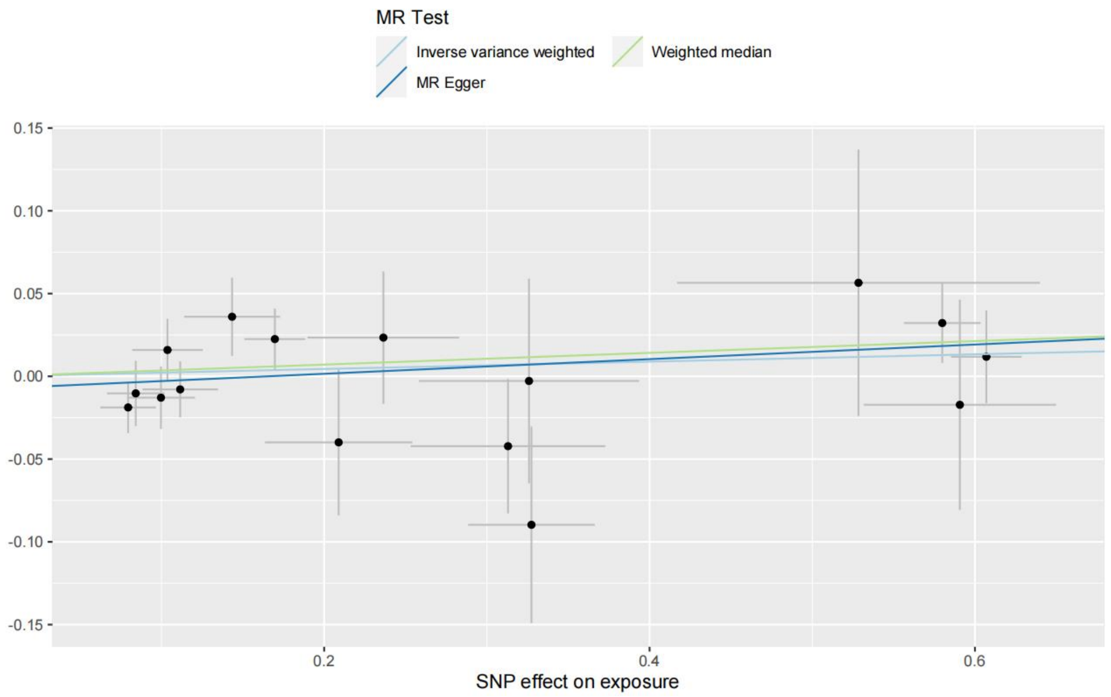

A. Scatter plot of EOTAXIN with risk of ALS

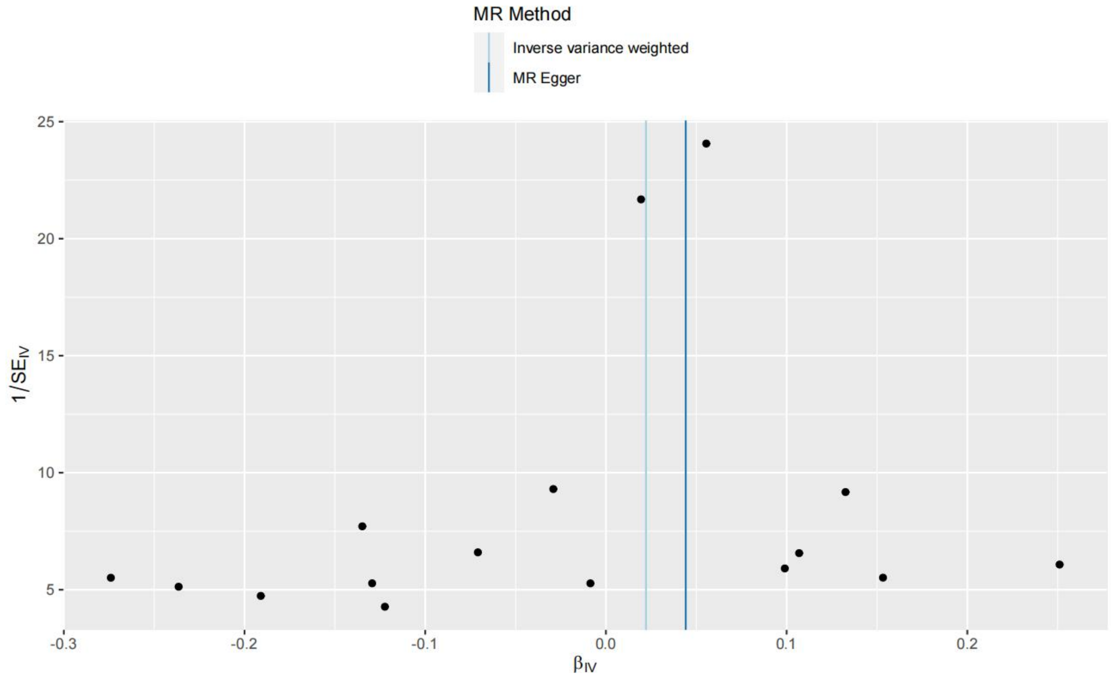

B. Funnel plot of EOTAXIN instruments strength on ALS

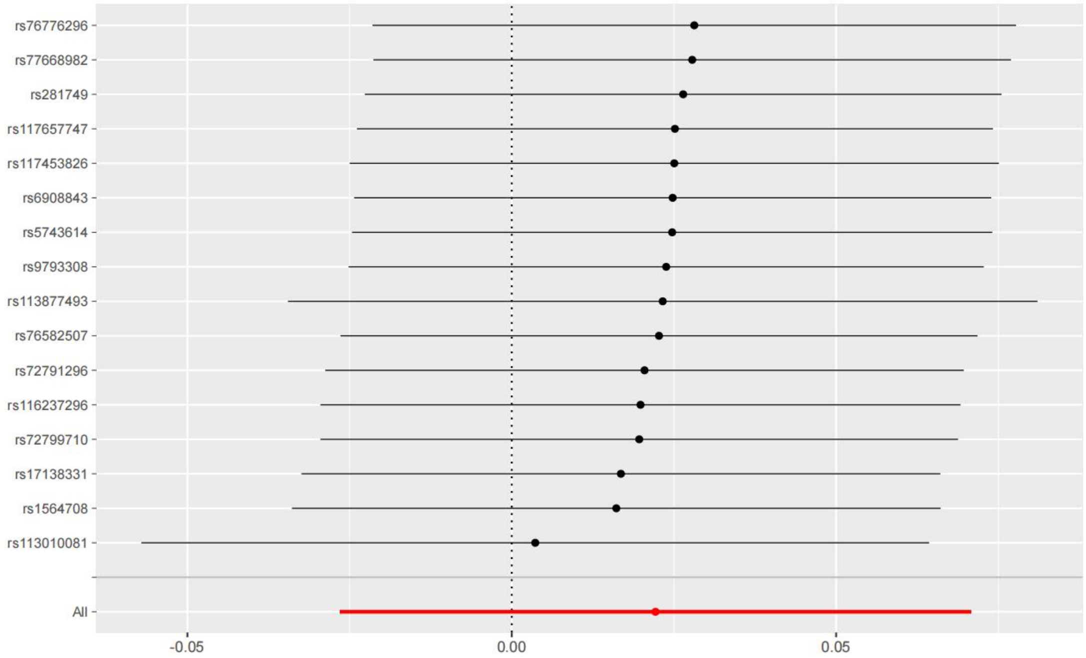

C. MR leave-one-out sensitivity analysis for EOTAXIN on ALS

eFigure 86. bFGF-associated SNPs with risk of ALS

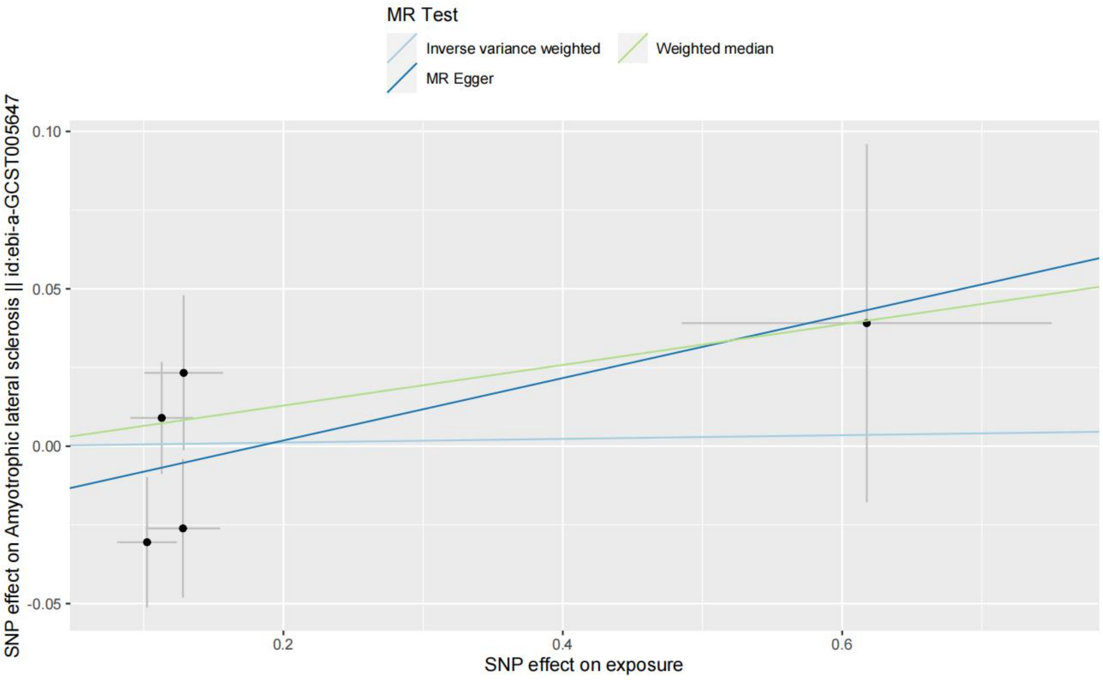

A. Scatter plot of bFGF with risk of ALS

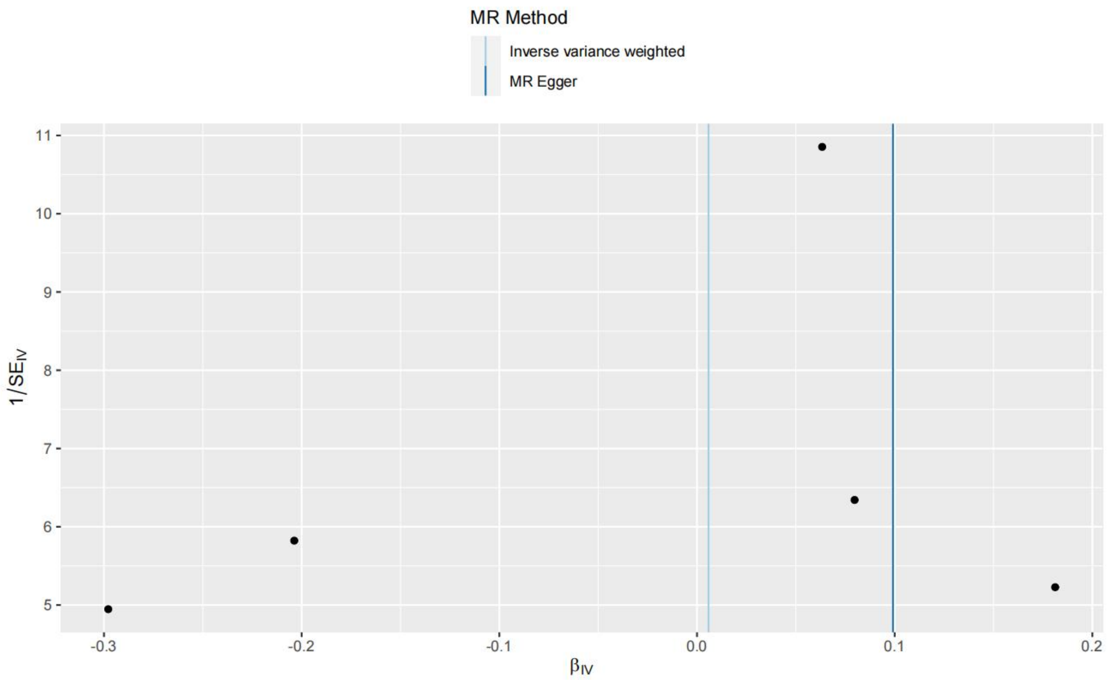

B. Funnel plot of bFGF instruments strength on ALS

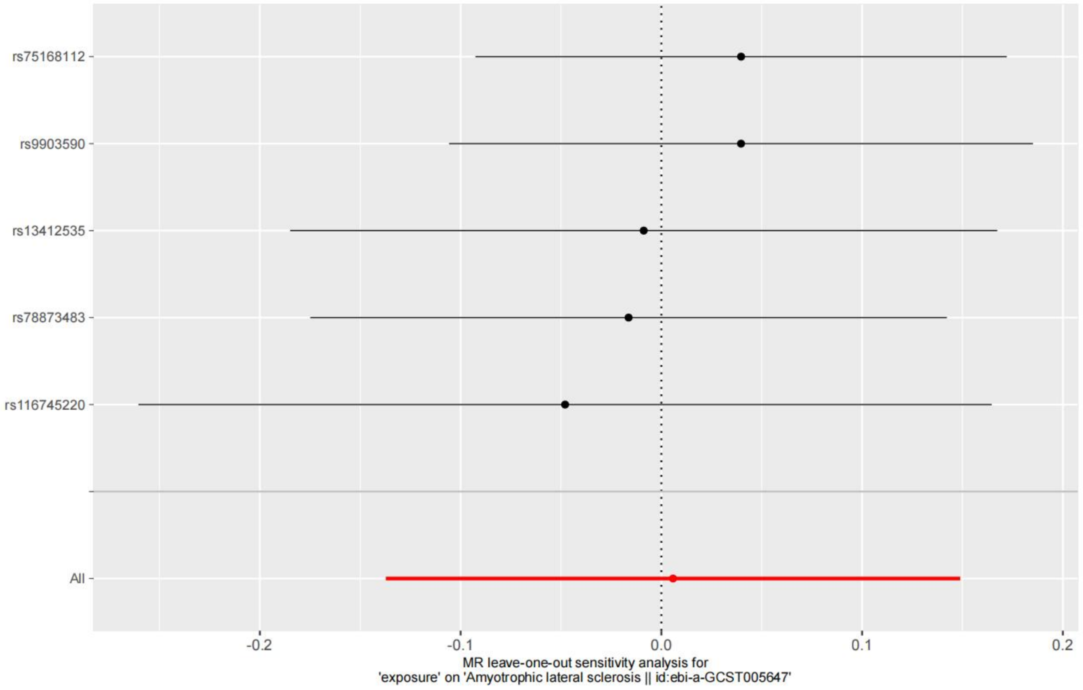

C. MR leave-one-out sensitivity analysis for bFGF on ALS

eFigure 87. G-CSF-associated SNPs with risk of ALS

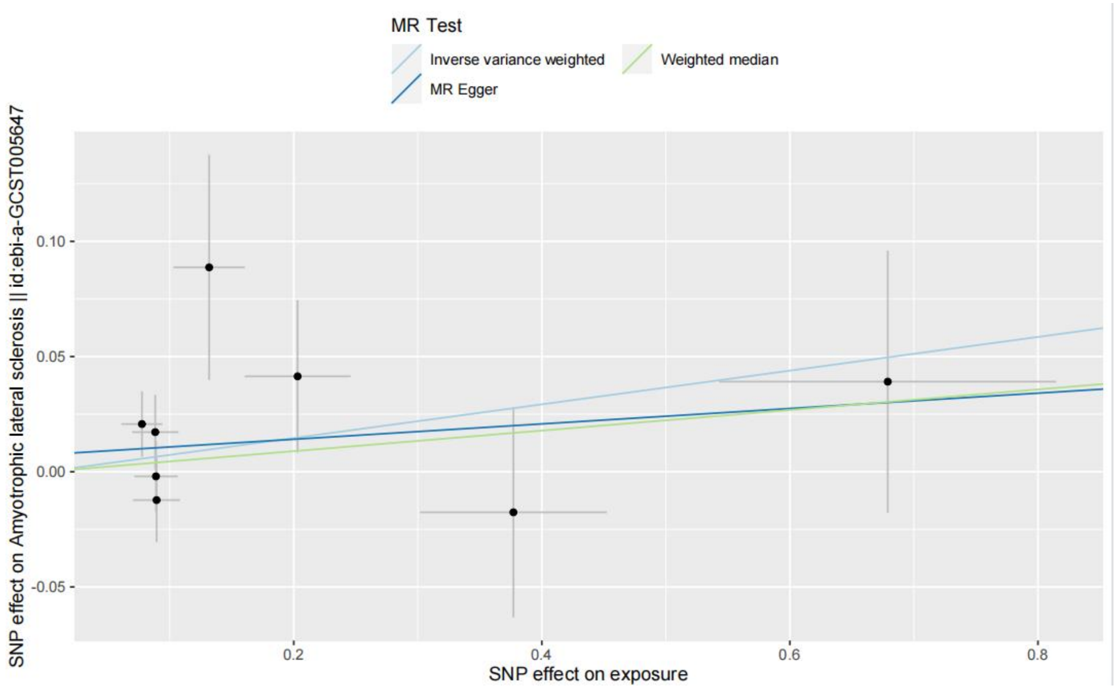

A. Scatter plot of G-CSF with risk of ALS

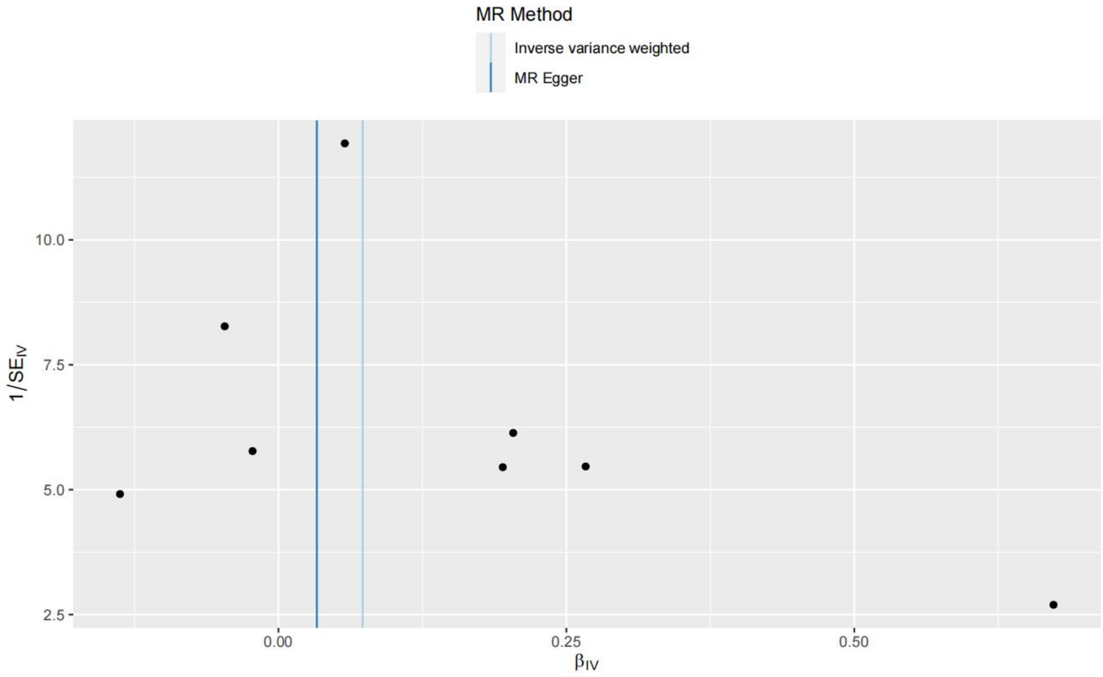

B. Funnel plot of G-CSF instruments strength on ALS

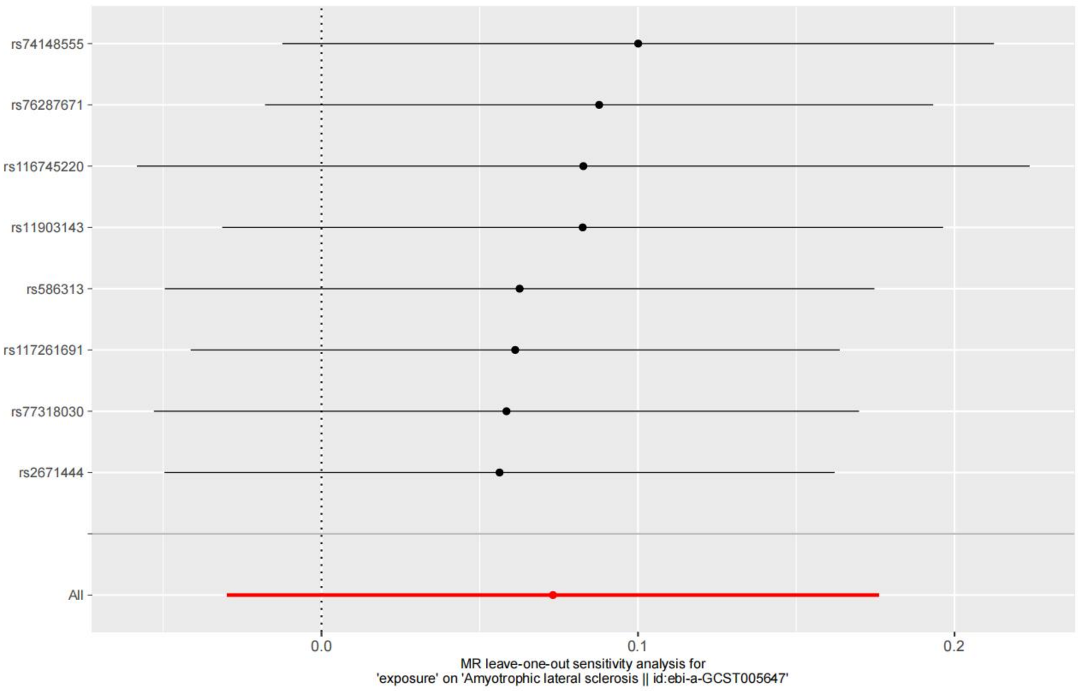

C. MR leave-one-out sensitivity analysis for G-CSF on ALS

eFigure 88. GROA-associated SNPs with risk of ALS

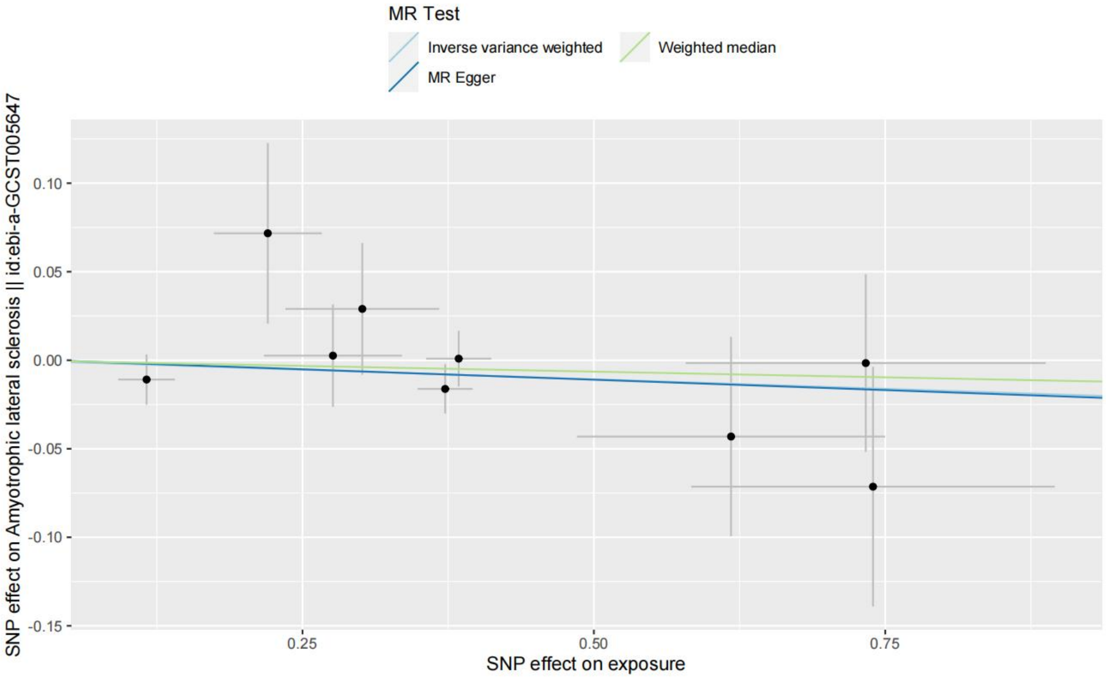

A. Scatter plot of GROA with risk of ALS

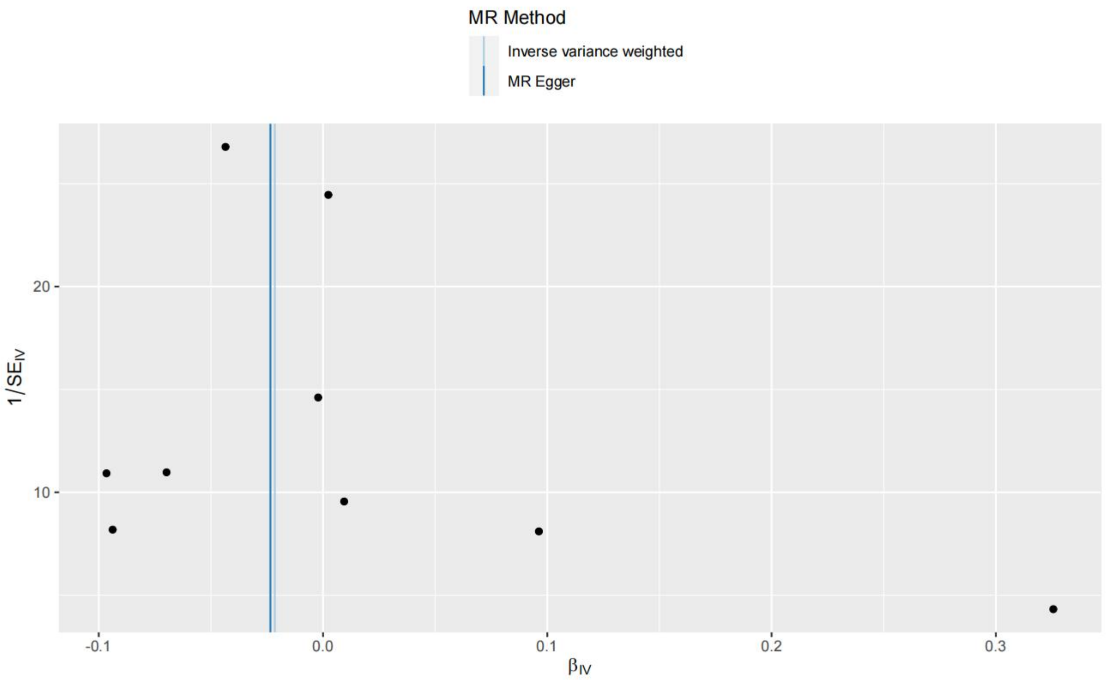

B. Funnel plot of GROA instruments strength on ALS

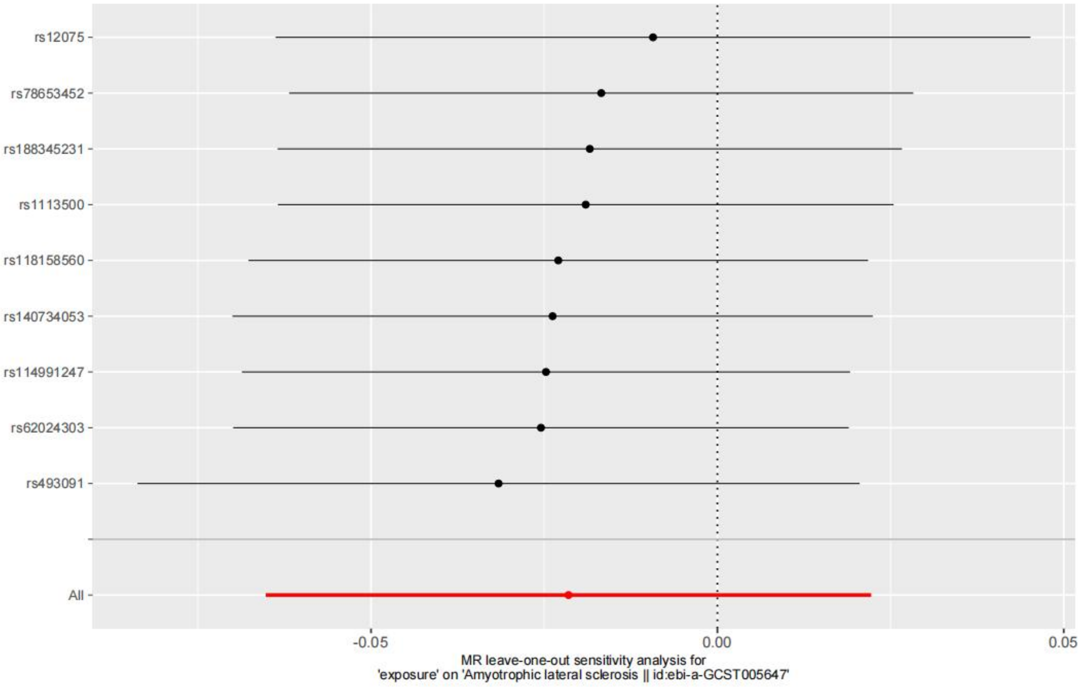

C. MR leave-one-out sensitivity analysis for GROA on ALS

eFigure 89. HGF-associated SNPs with risk of ALS

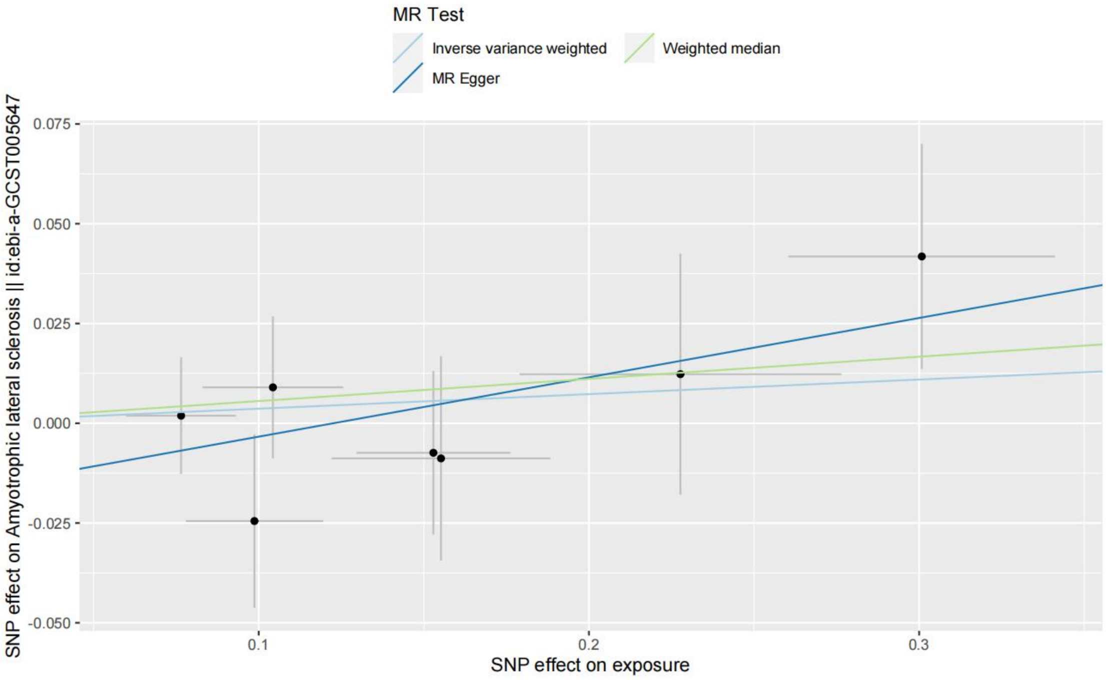

A. Scatter plot of HGF with risk of ALS

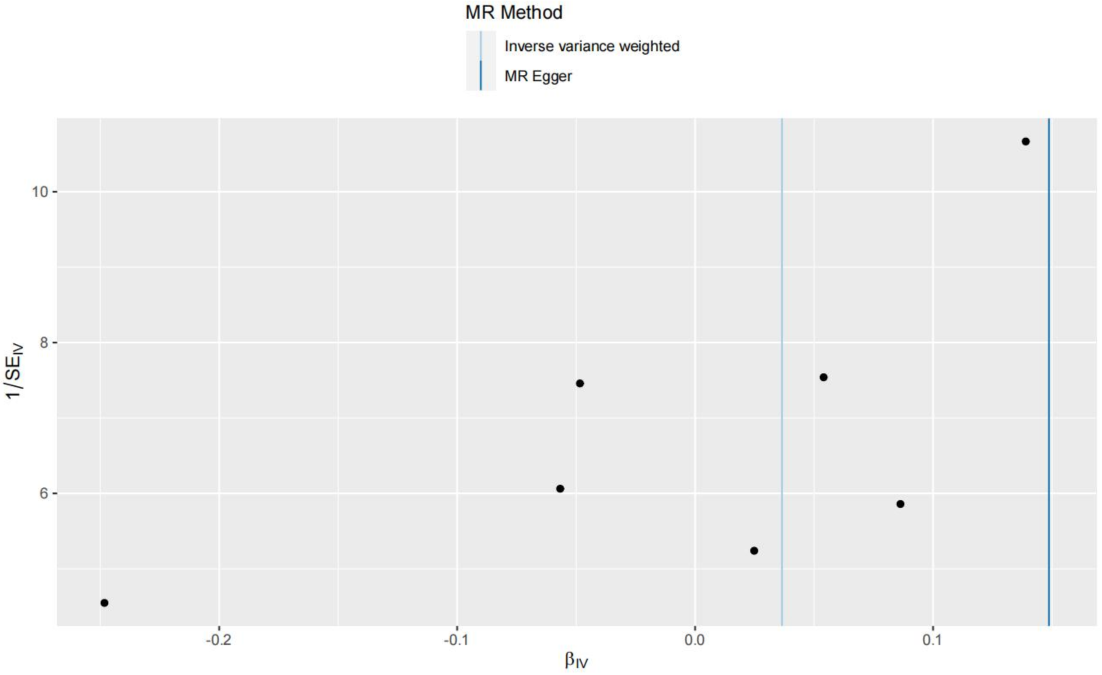

B. Funnel plot of HGF instruments strength on ALS

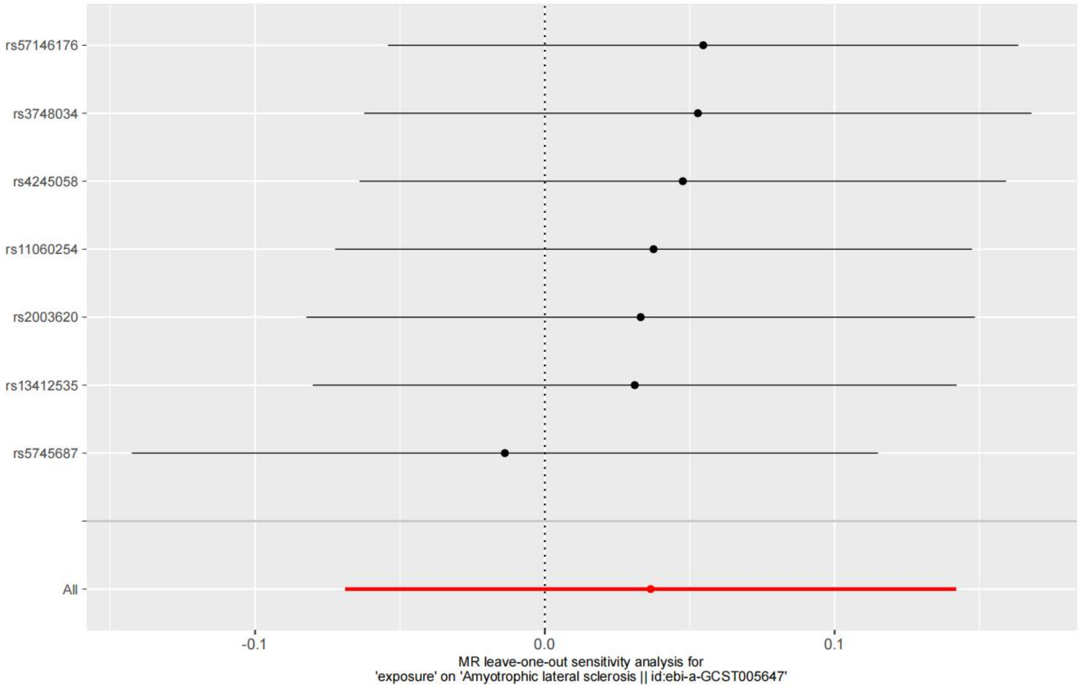

C. MR leave-one-out sensitivity analysis for HGF on ALS

eFigure 90. IFN-G-associated SNPs with risk of ALS

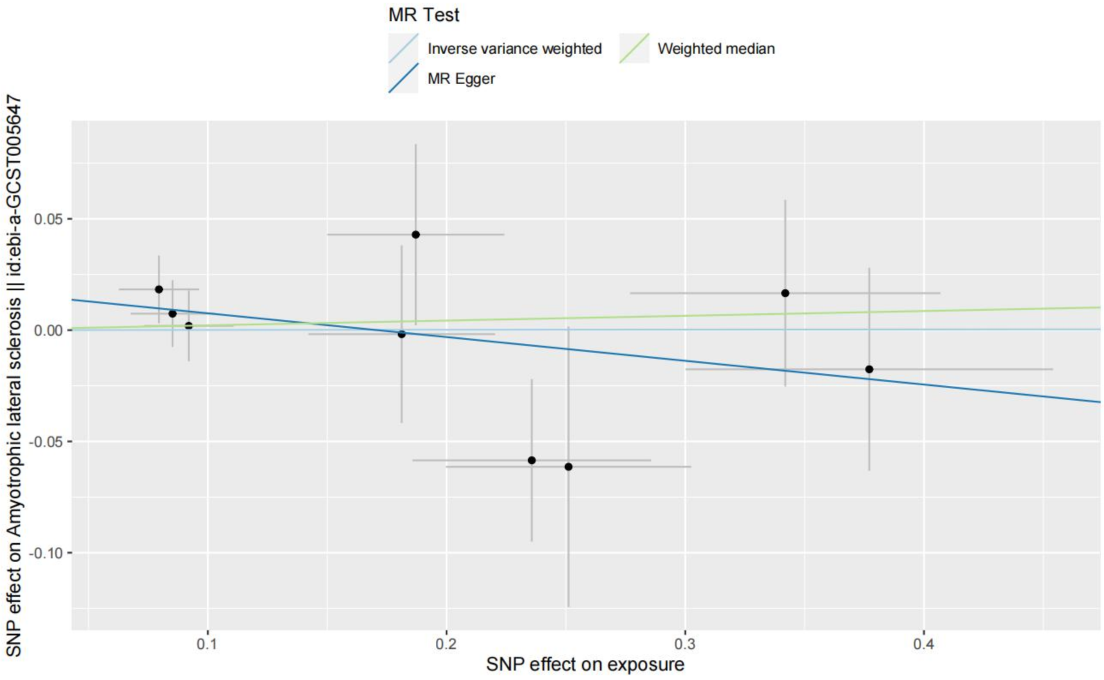

A. Scatter plot of IFN-G with risk of ALS

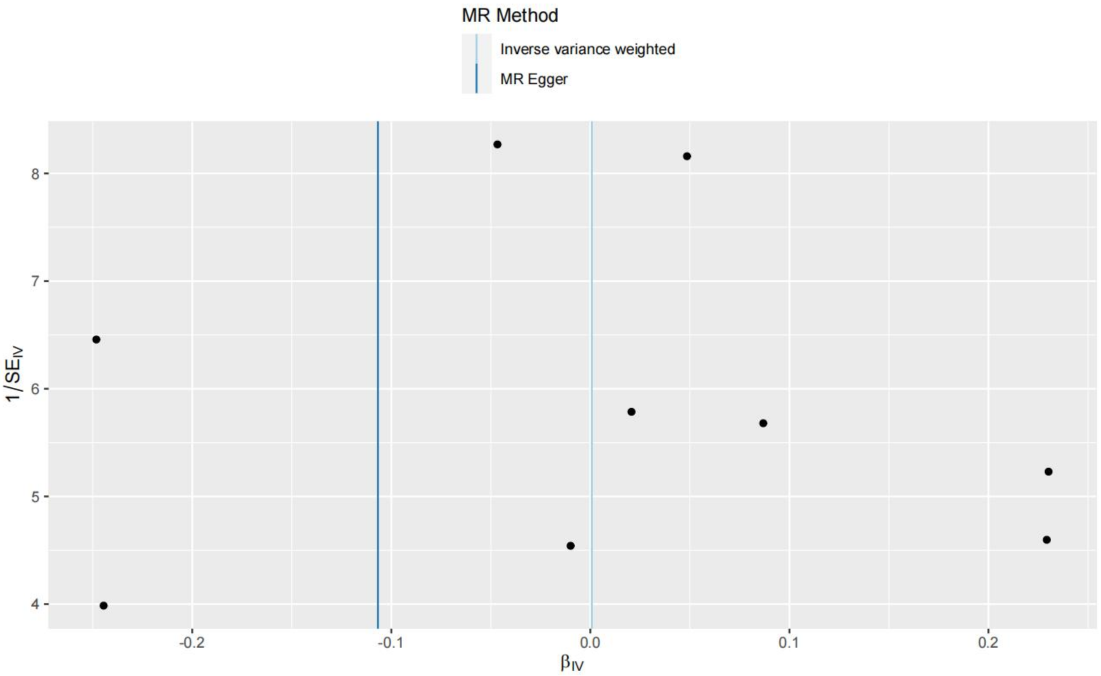

B. Funnel plot of IFN-G instruments strength on ALS

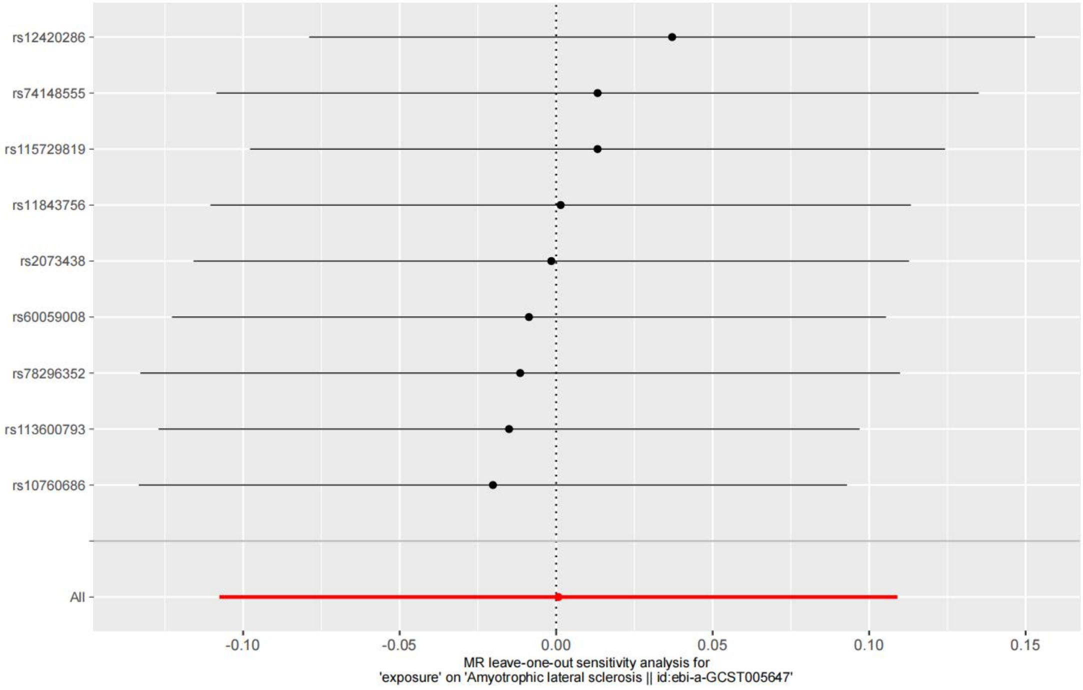

C. MR leave-one-out sensitivity analysis for IFN-G on ALS

eFigure 91. IL-1B-associated SNPs with risk of ALS

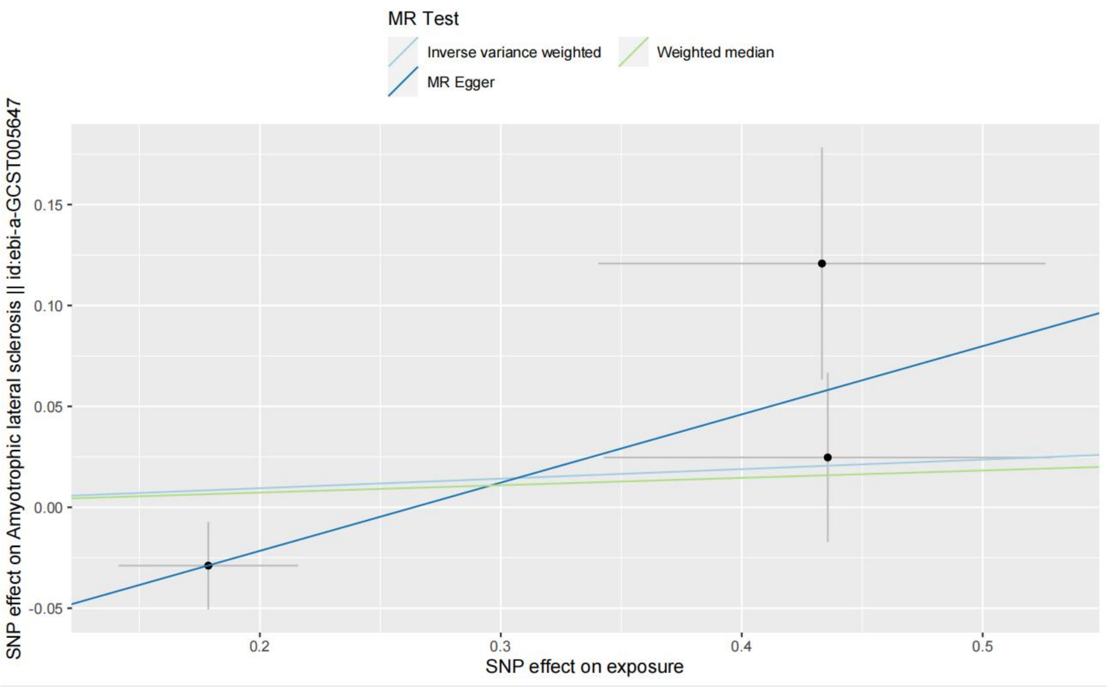

A. Scatter plot of IL-1B with risk of ALS

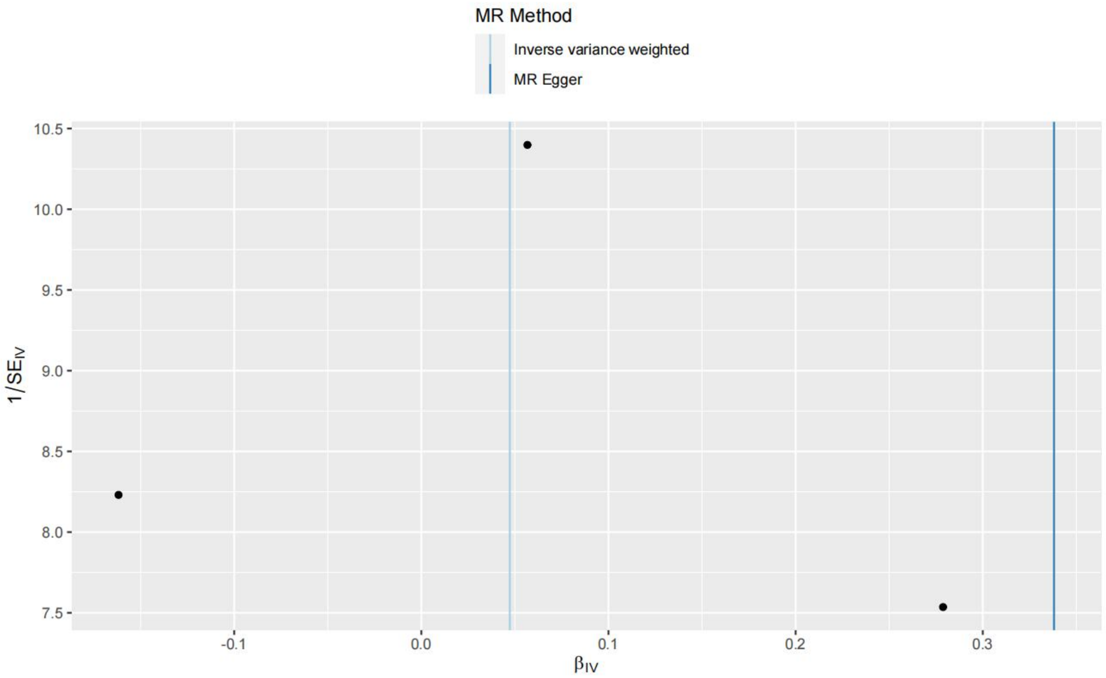

B. Funnel plot of IL-1B instruments strength on ALS

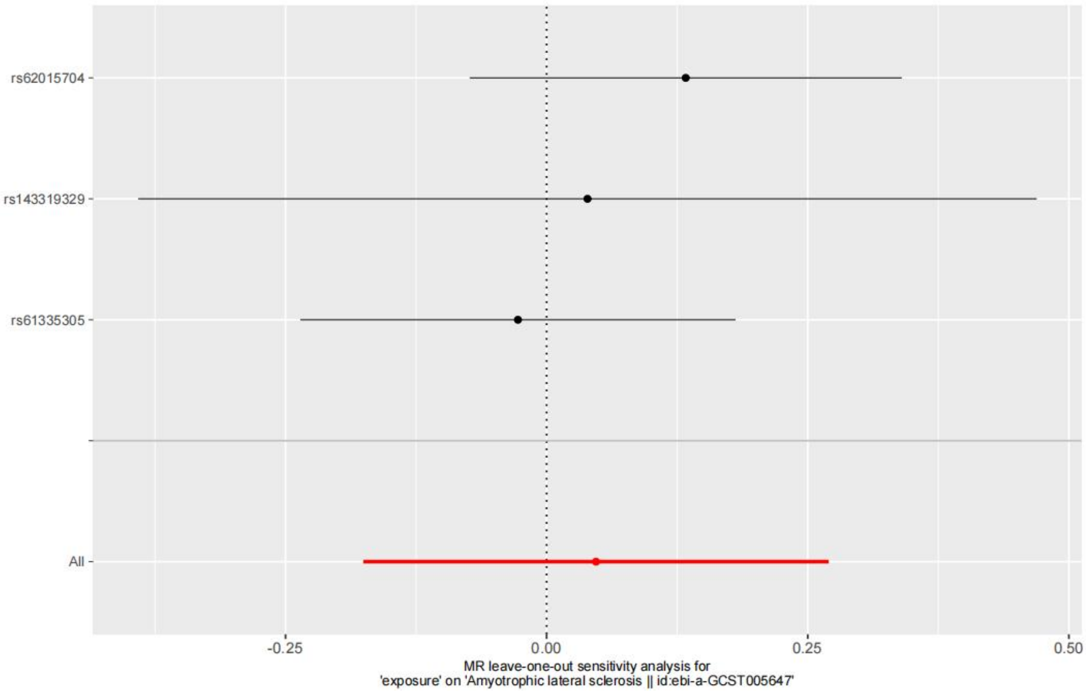

C. MR leave-one-out sensitivity analysis for IL-1B on ALS

eFigure 92. IL-1RA-associated SNPs with risk of ALS

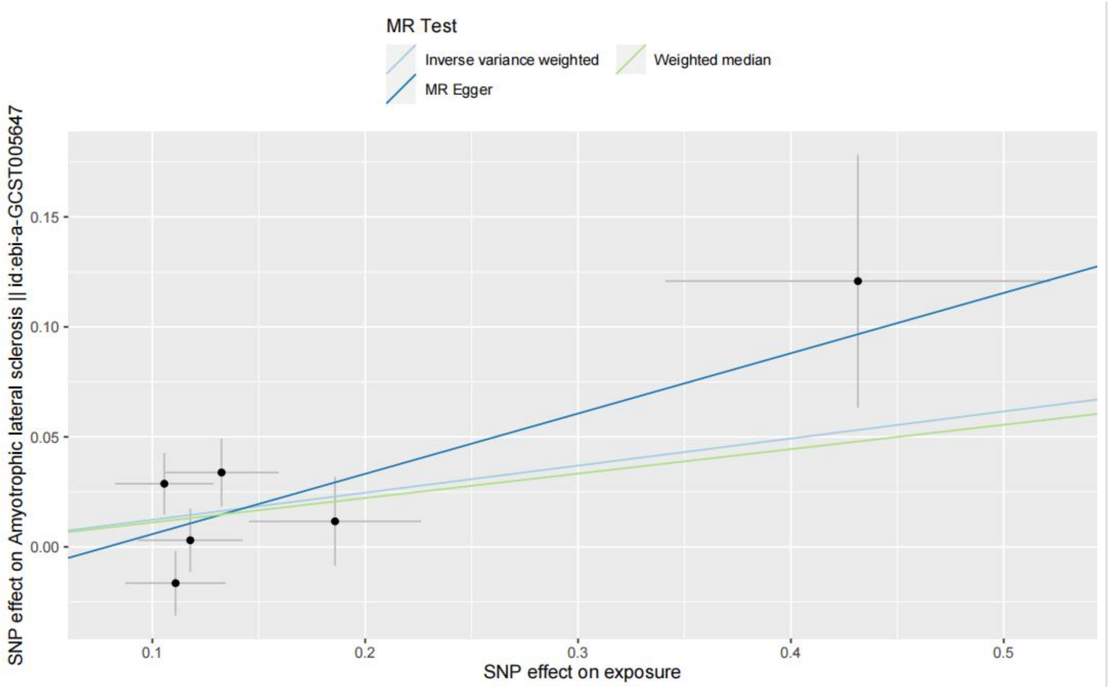

A. Scatter plot of IL-1RA with risk of ALS

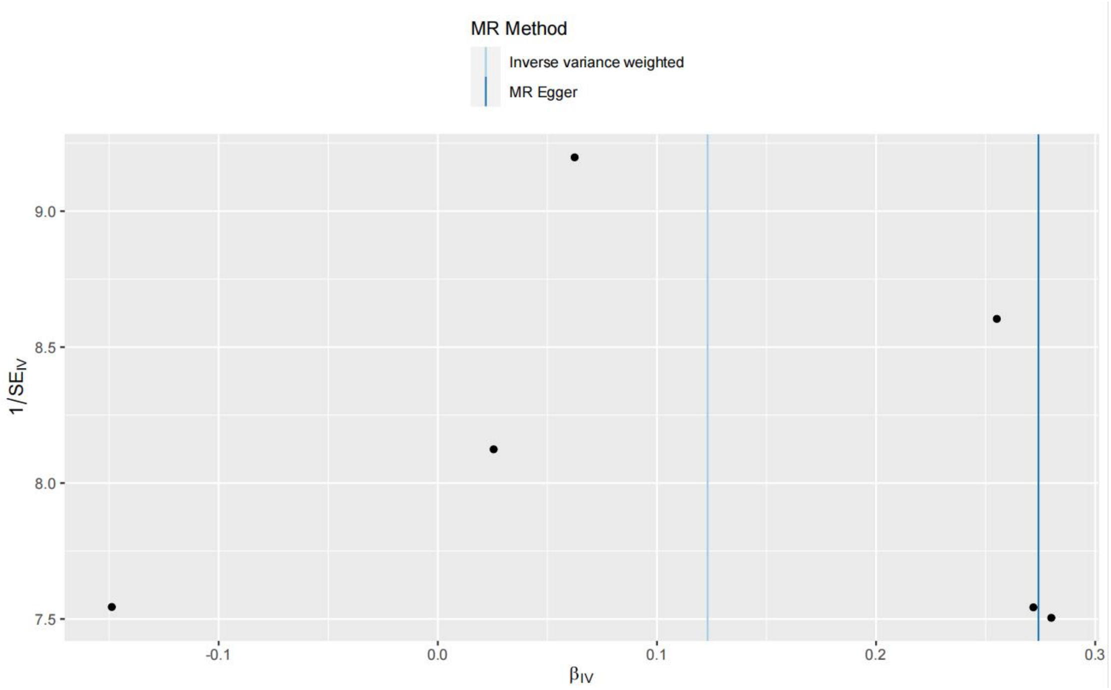

B. Funnel plot of IL-1RA instruments strength on ALS

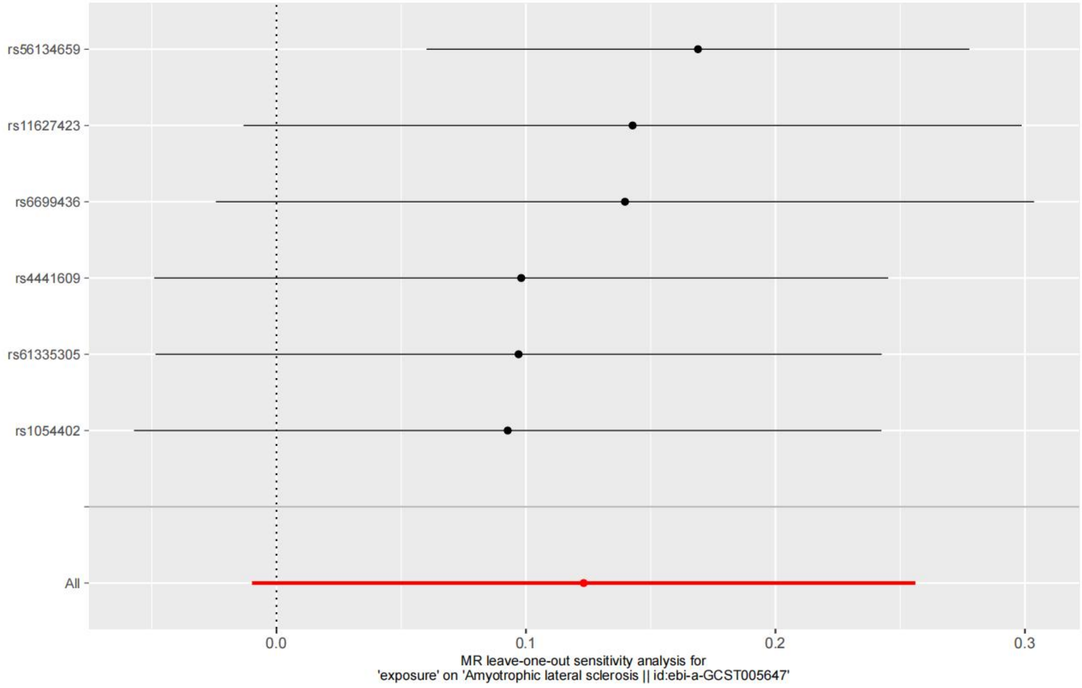

C. MR leave-one-out sensitivity analysis for IL-1RA on ALS

eFigure 93. IL-2-associated SNPs with risk of ALS

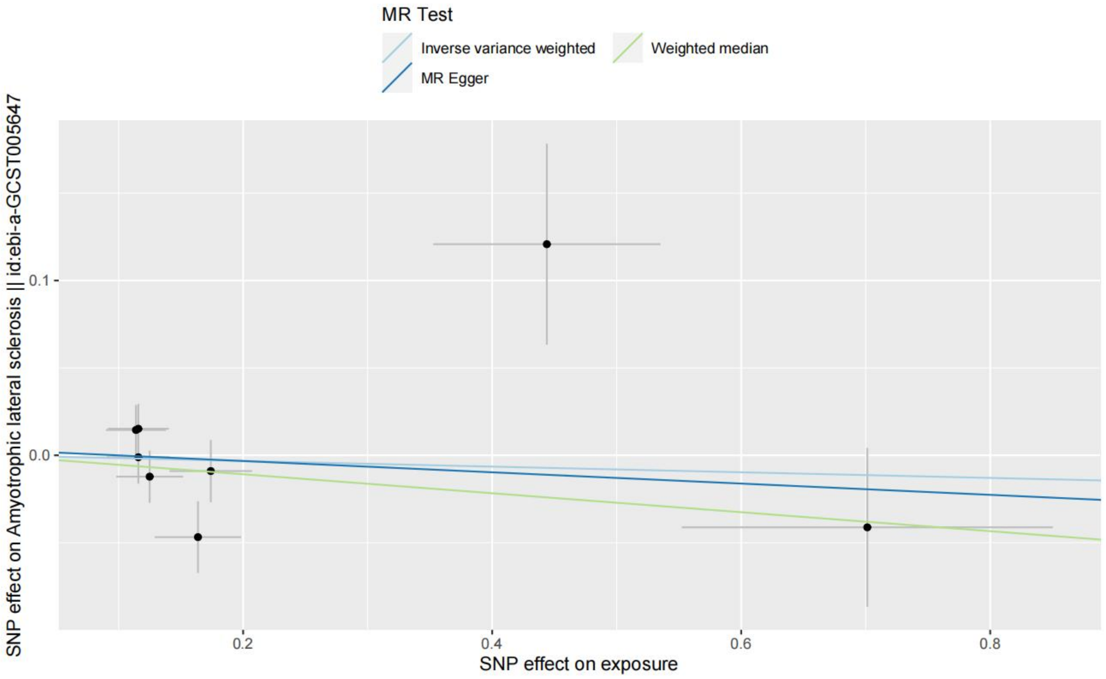

A. Scatter plot of IL-2 with risk of ALS

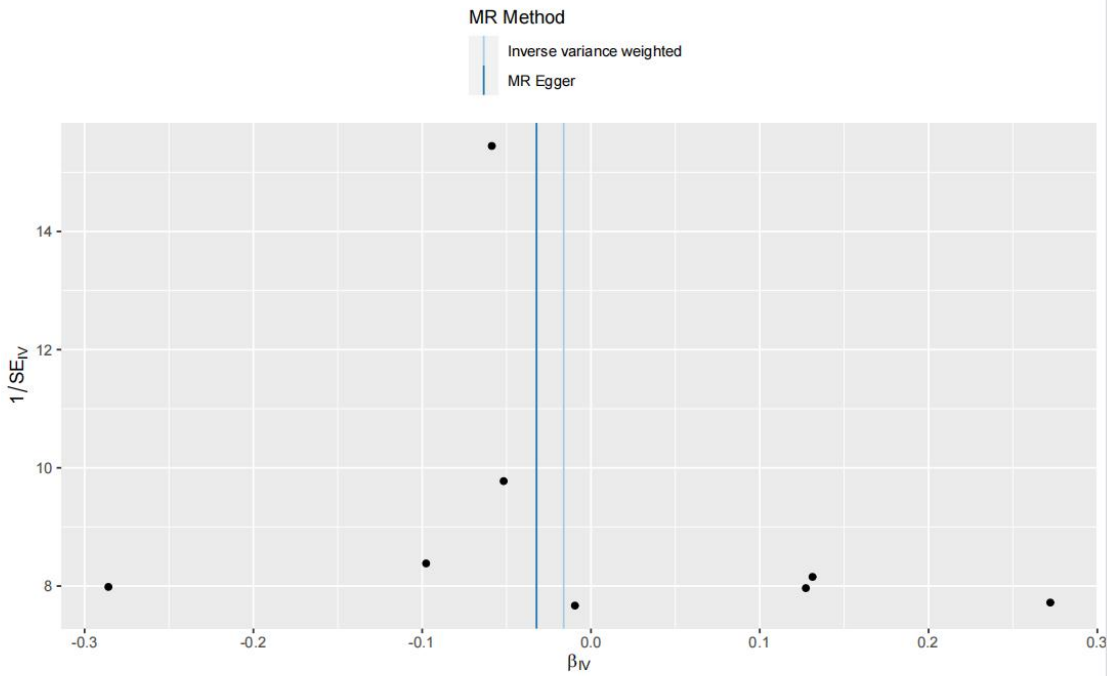

B. Funnel plot of IL-2 instruments strength on ALS

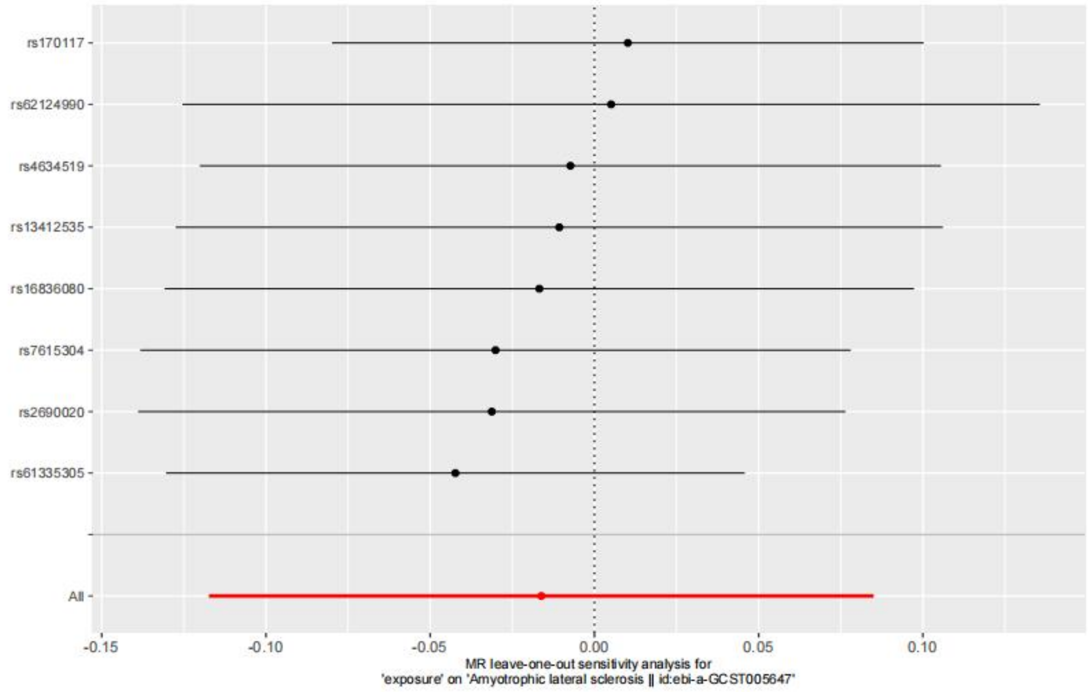

C. MR leave-one-out sensitivity analysis for IL-2 on ALS

eFigure 94. IL-2RA-associated SNPs with risk of ALS

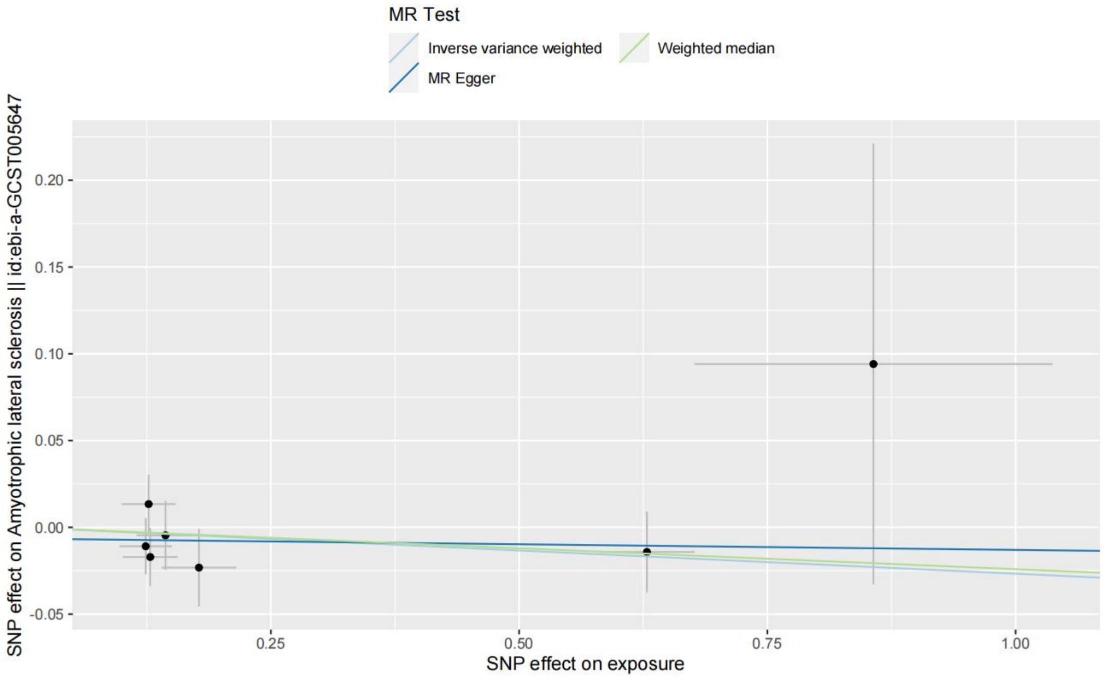

A. Scatter plot of IL-2RA with risk of ALS

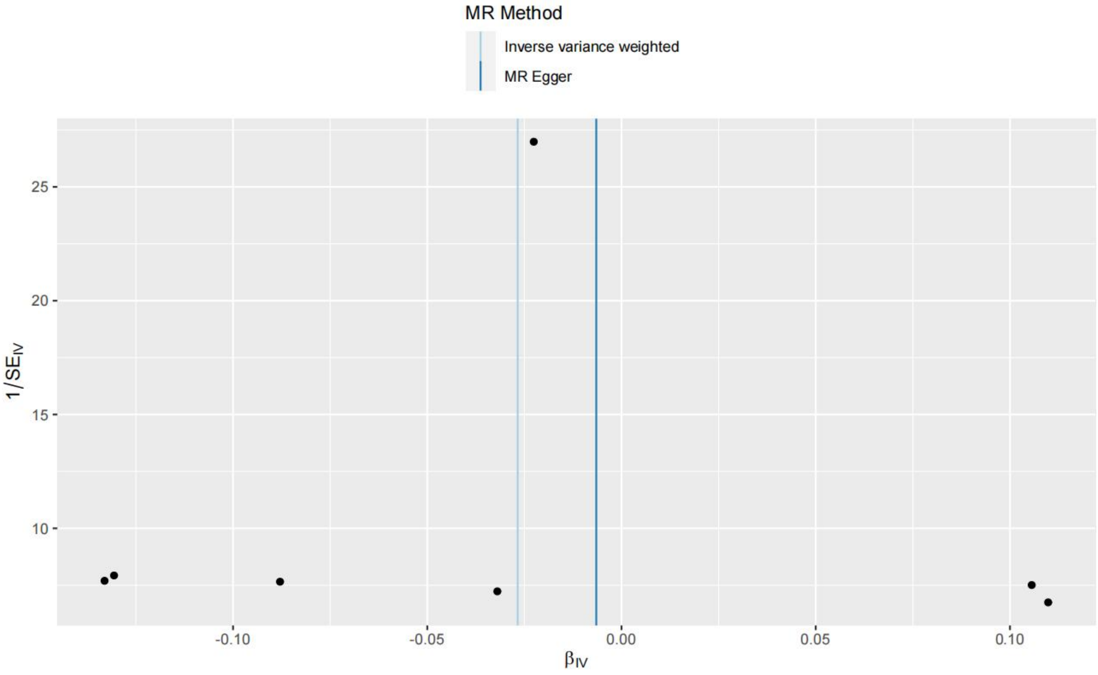

B. Funnel plot of IL-2RA instruments strength on ALS

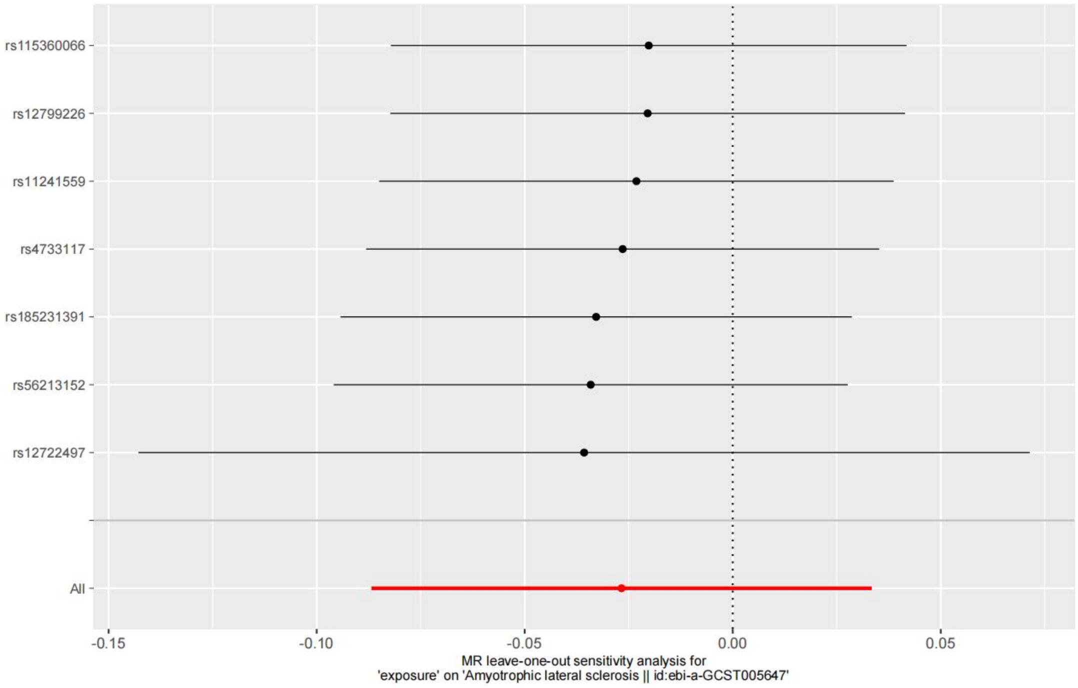

C. MR leave-one-out sensitivity analysis for IL-2RA on ALS

eFigure 95. IL-4-associated SNPs with risk of ALS

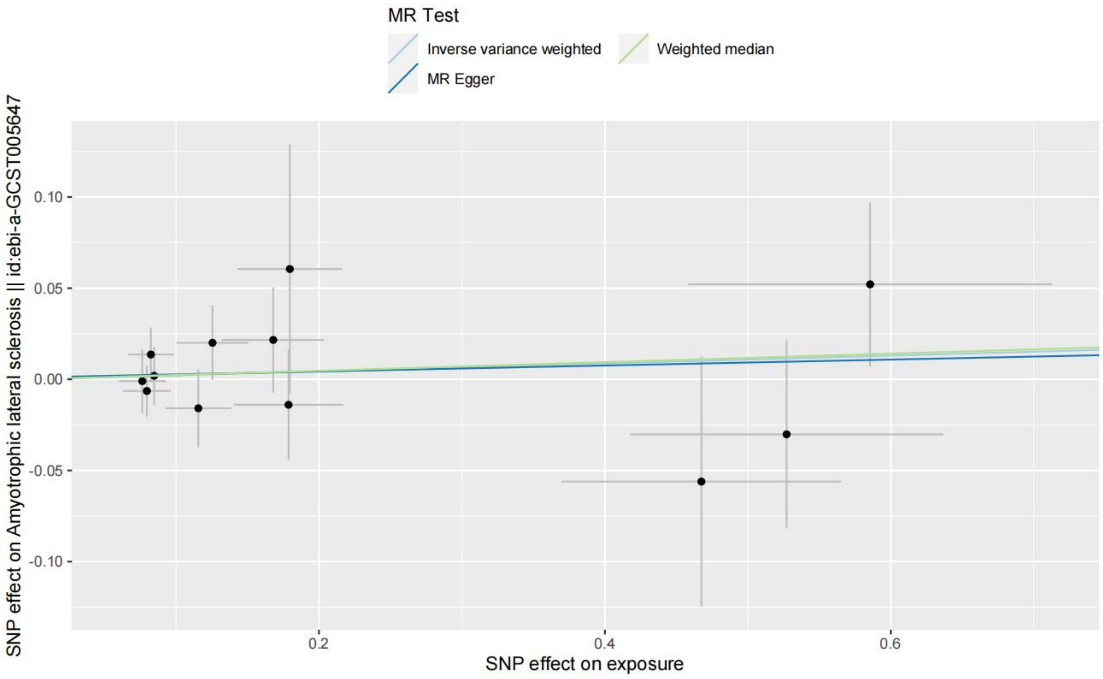

A. Scatter plot of IL-4 with risk of ALS

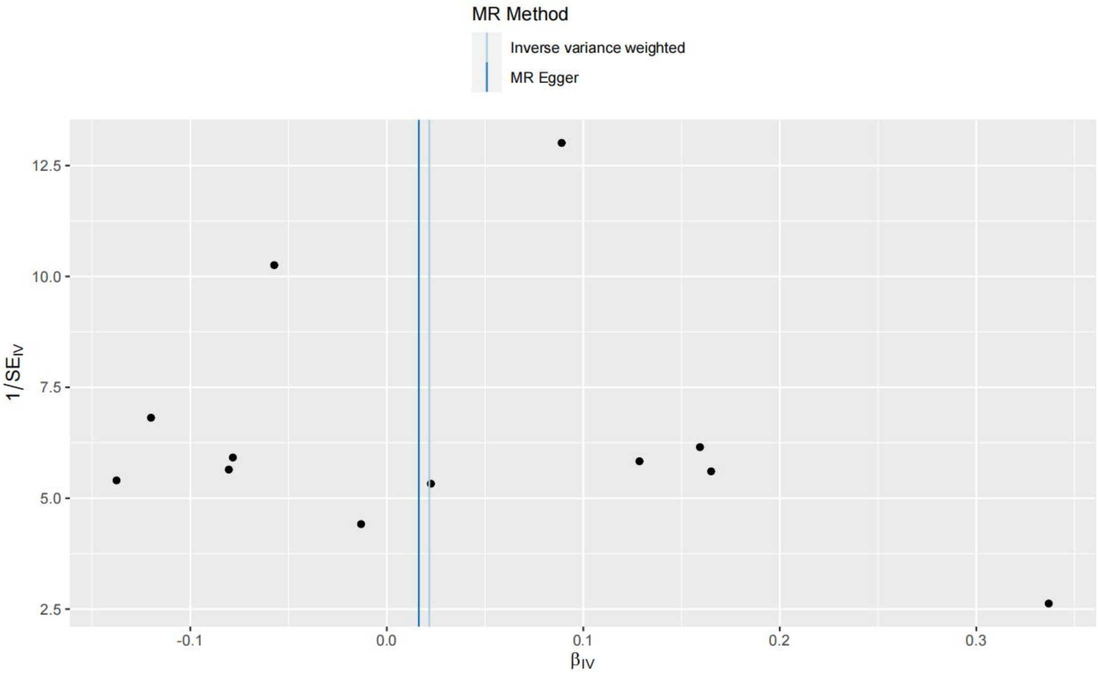

B. Funnel plot of IL-4 instruments strength on ALS

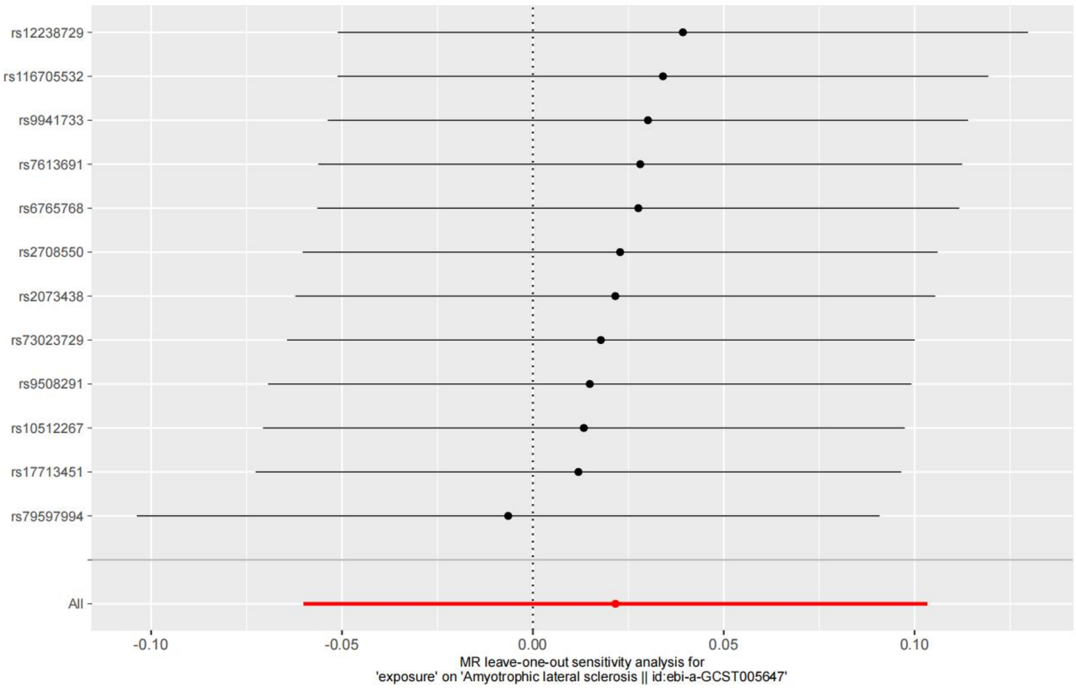

C. MR leave-one-out sensitivity analysis for IL-4 on ALS

eFigure 96. IL-5-associated SNPs with risk of ALS

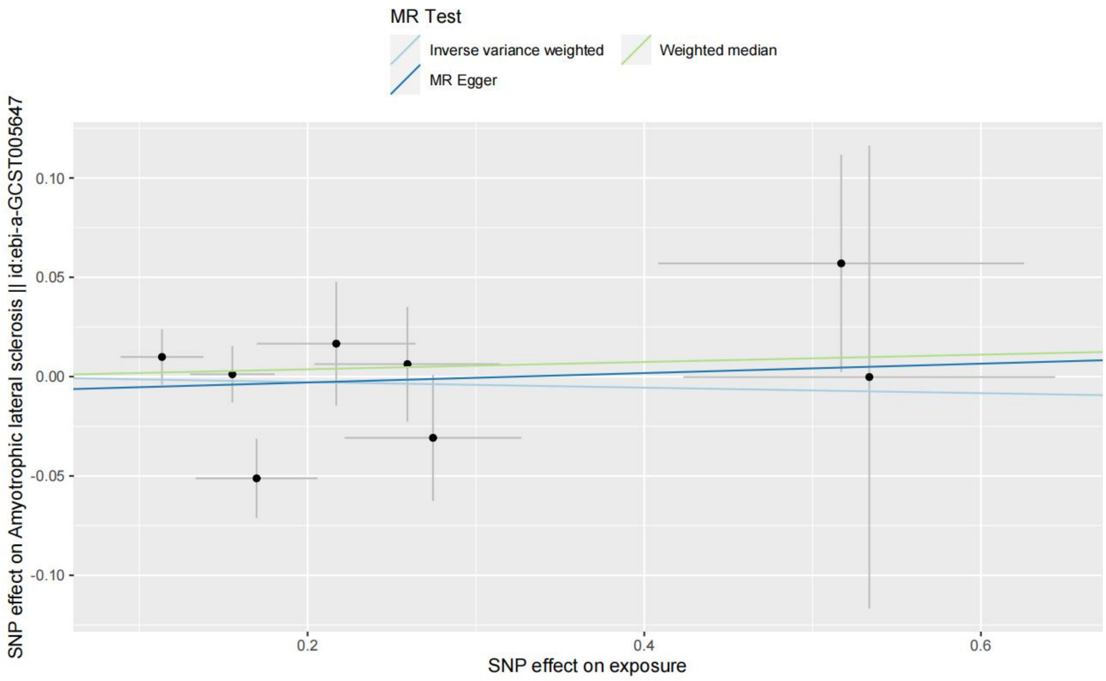

A. Scatter plot of IL-5 with risk of ALS

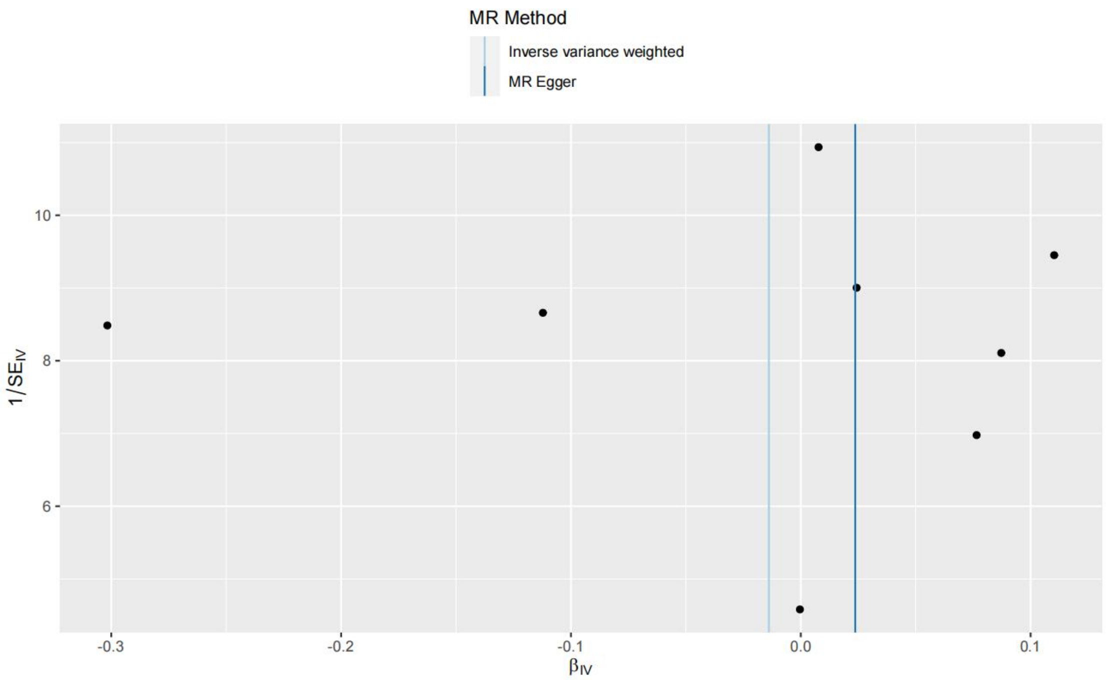

B. Funnel plot of IL-5 instruments strength on ALS

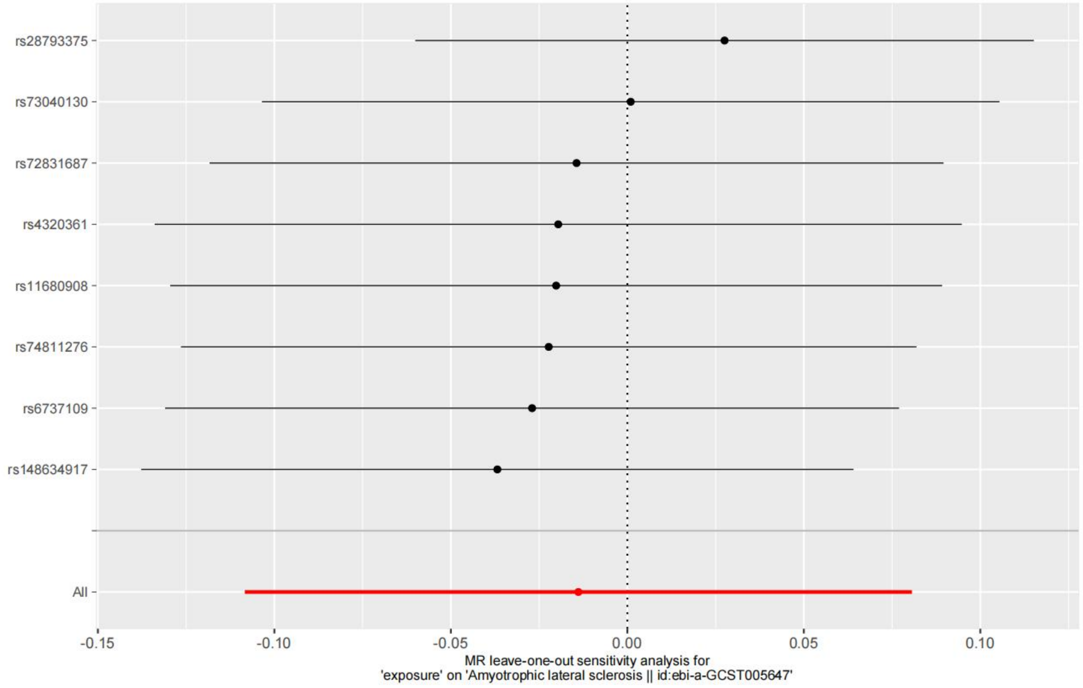

C. MR leave-one-out sensitivity analysis for IL-5 on ALS

eFigure 97. IL-6-associated SNPs with risk of ALS

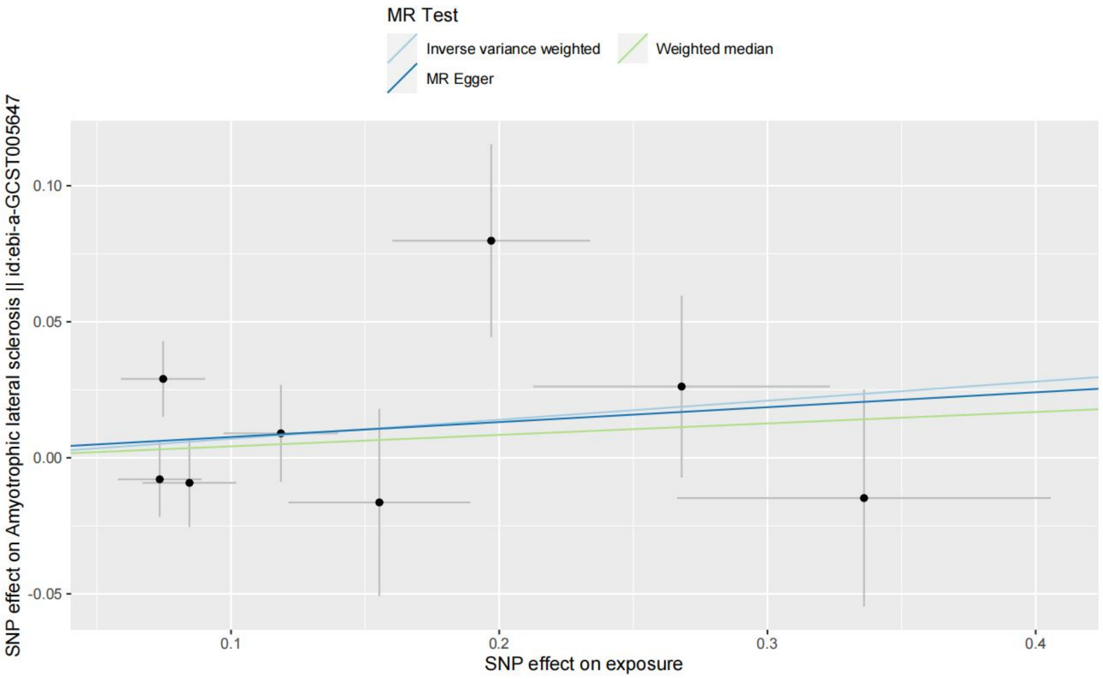

A. Scatter plot of IL-6 with risk of ALS

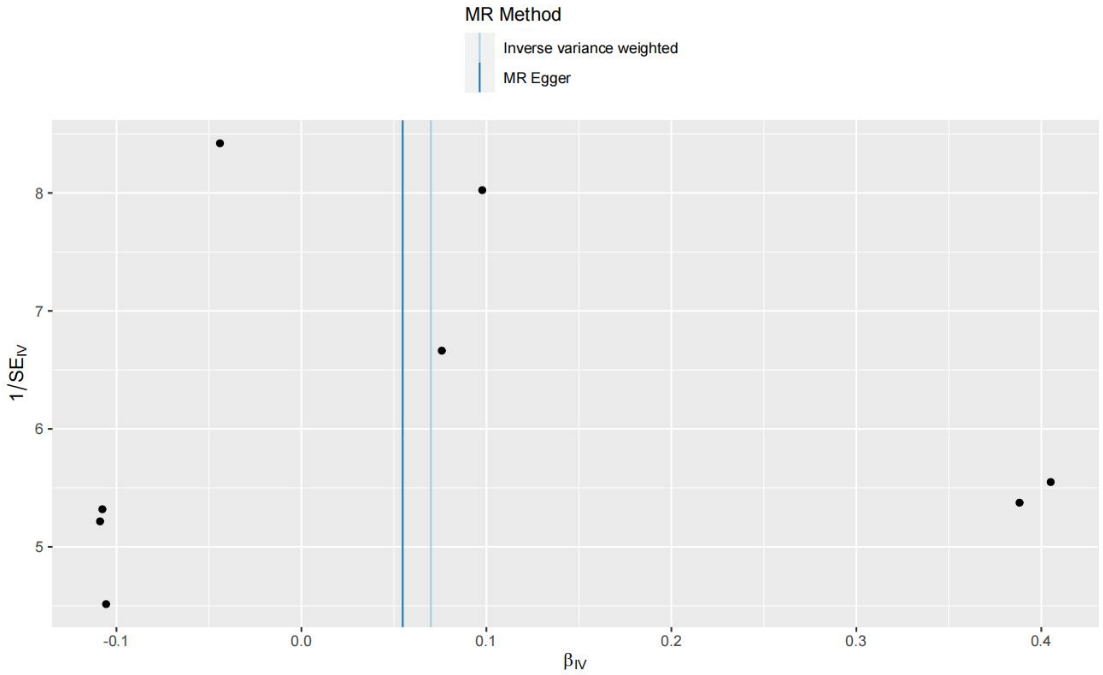

B. Funnel plot of IL-6 instruments strength on ALS

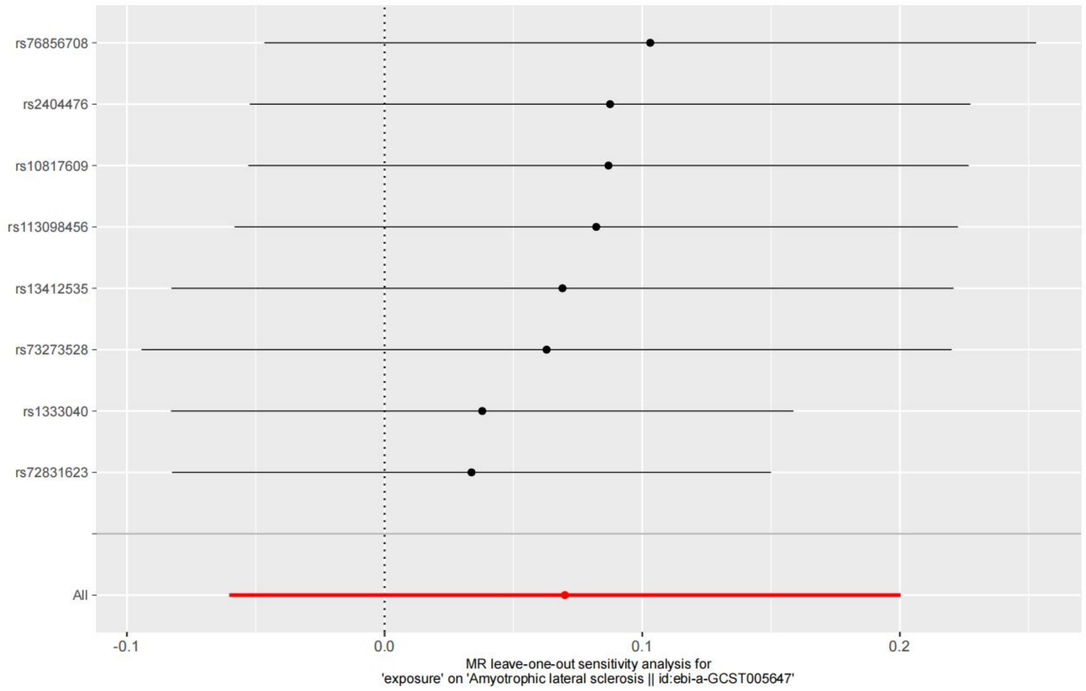

C. MR leave-one-out sensitivity analysis for IL-6 on ALS

eFigure 98. IL-7-associated SNPs with risk of ALS

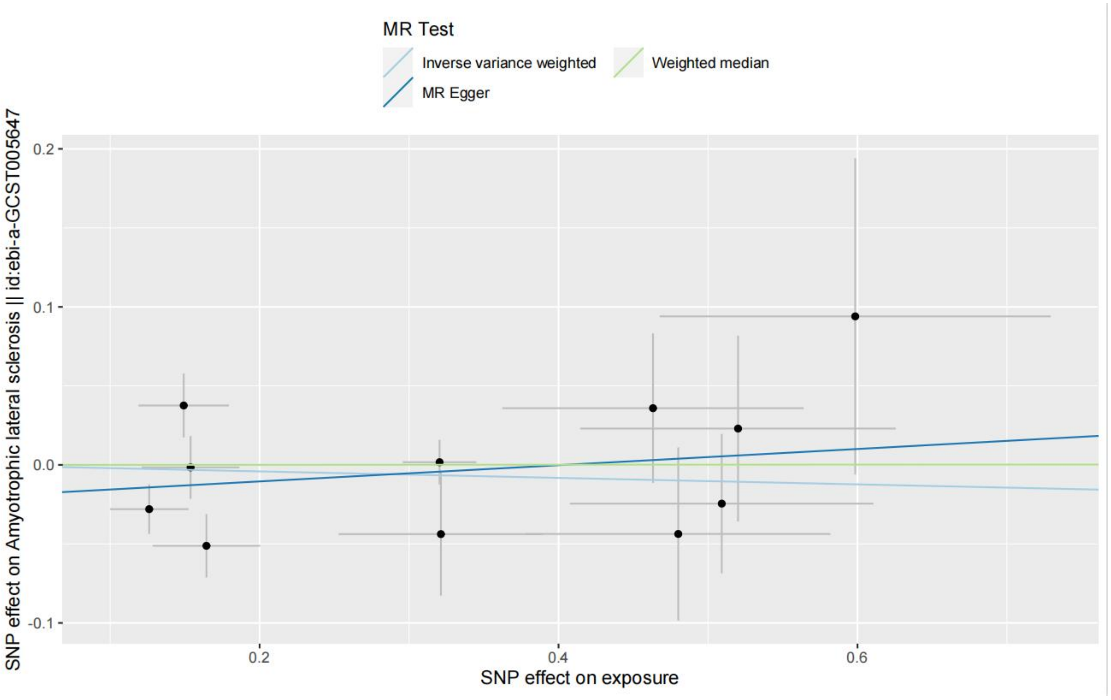

A. Scatter plot of IL-7 with risk of ALS

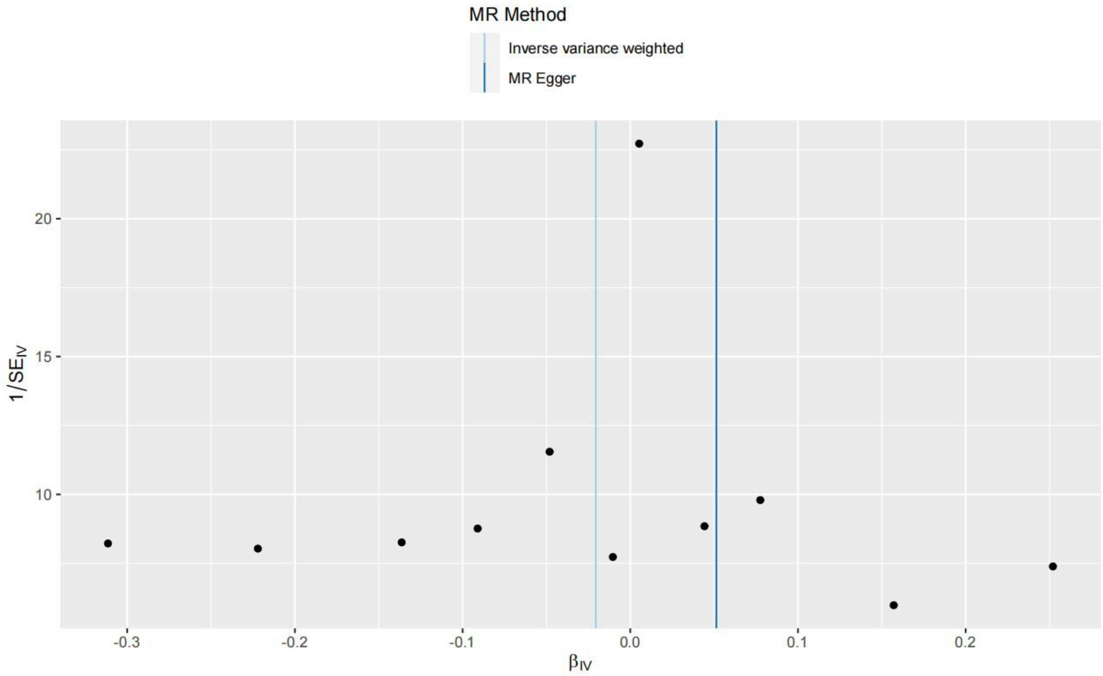

B. Funnel plot of IL-7 instruments strength on ALS

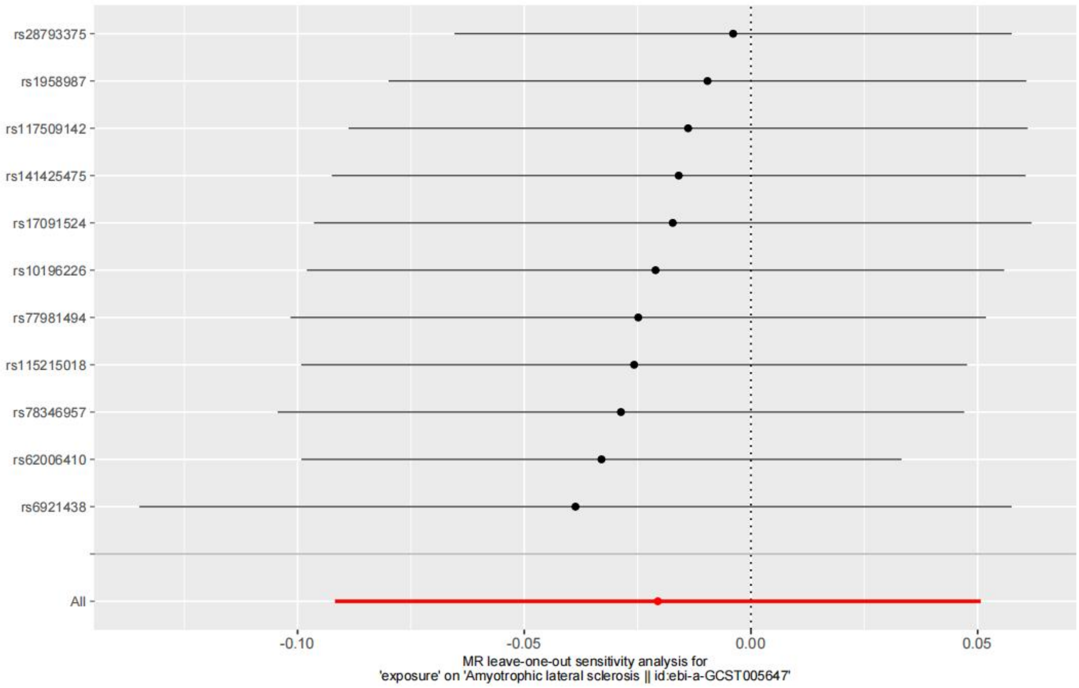

C. MR leave-one-out sensitivity analysis for IL-7 on ALS

eFigure 99. IL-8-associated SNPs with risk of ALS

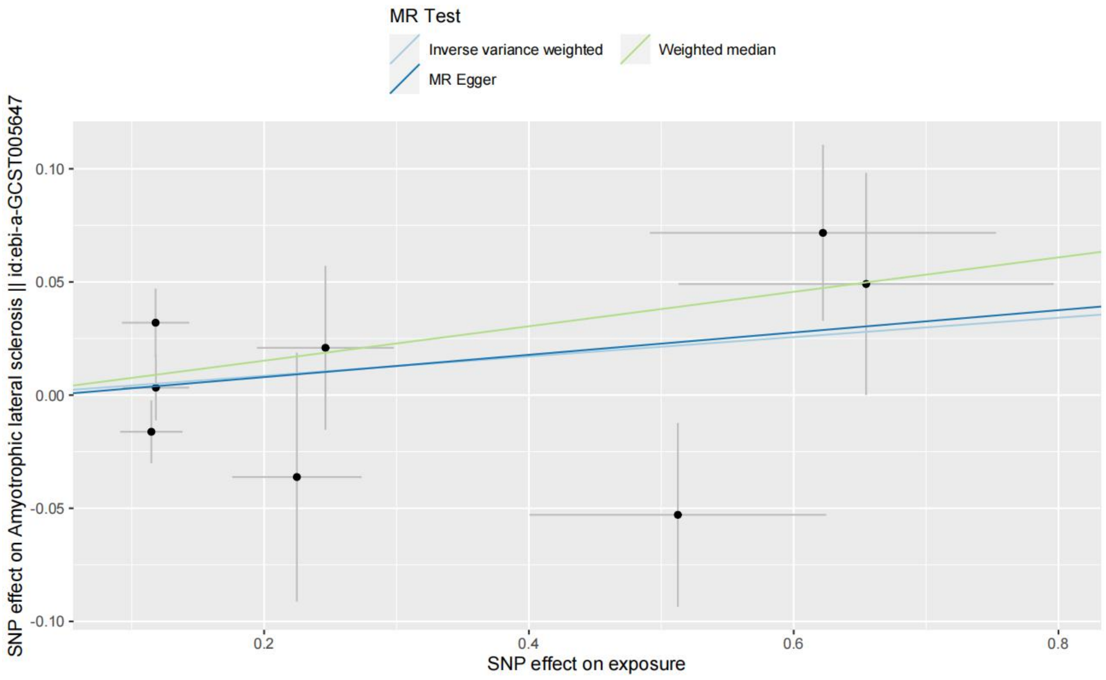

A. Scatter plot of IL-8 with risk of ALS

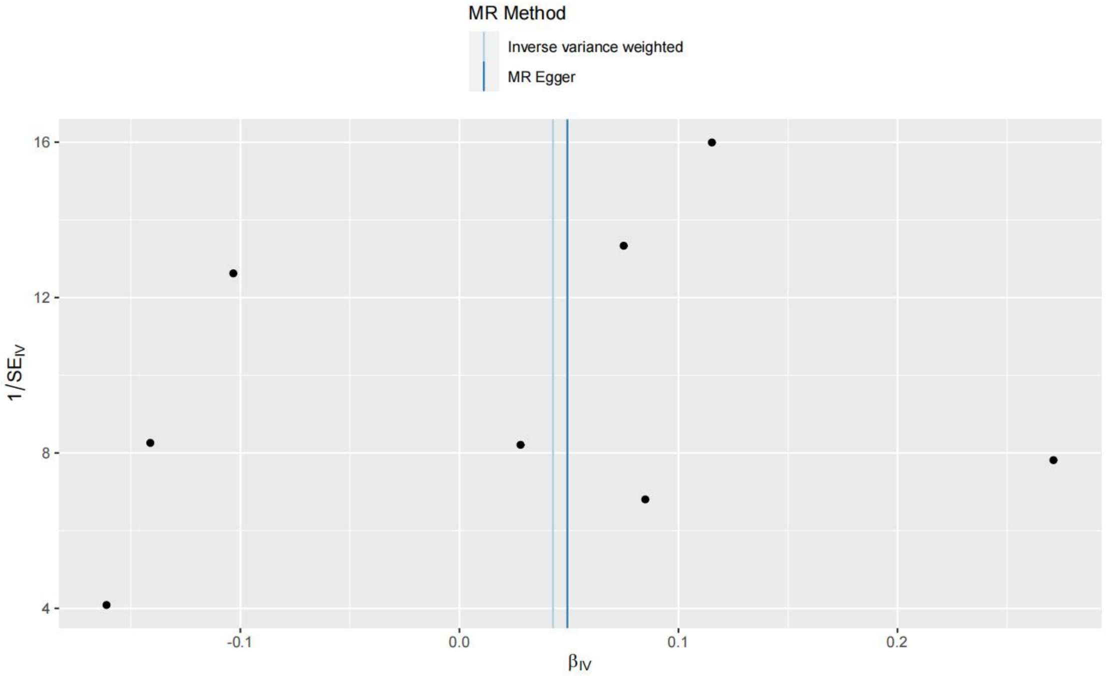

B. Funnel plot of IL-8 instruments strength on ALS

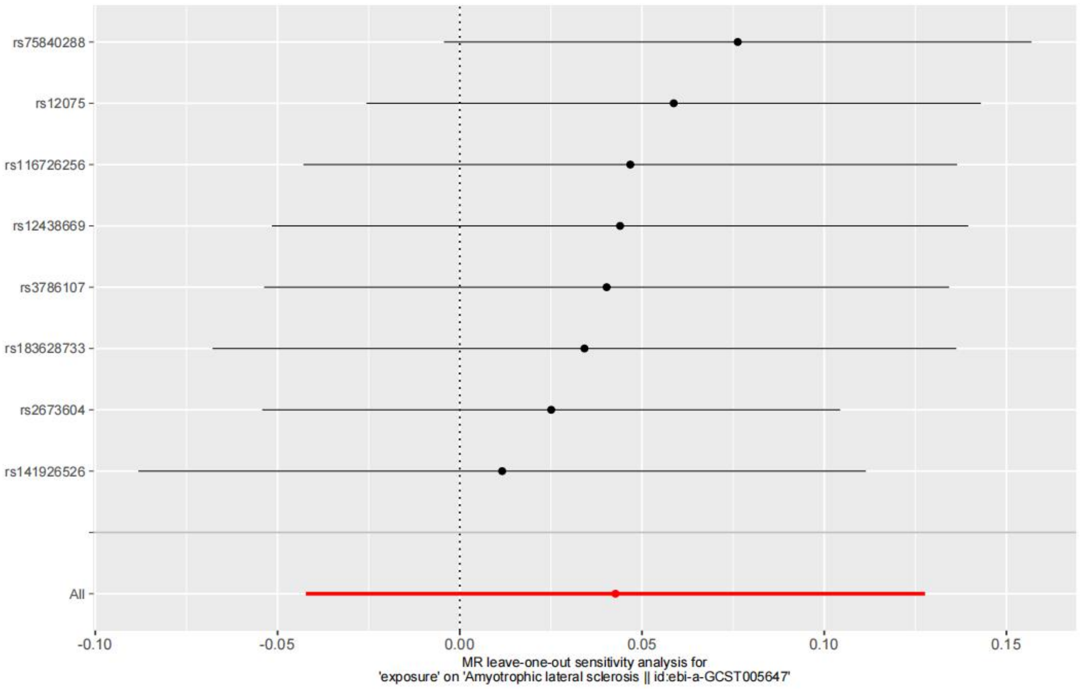

C. MR leave-one-out sensitivity analysis for IL-8 on ALS

eFigure 100. IL-9-associated SNPs with risk of ALS

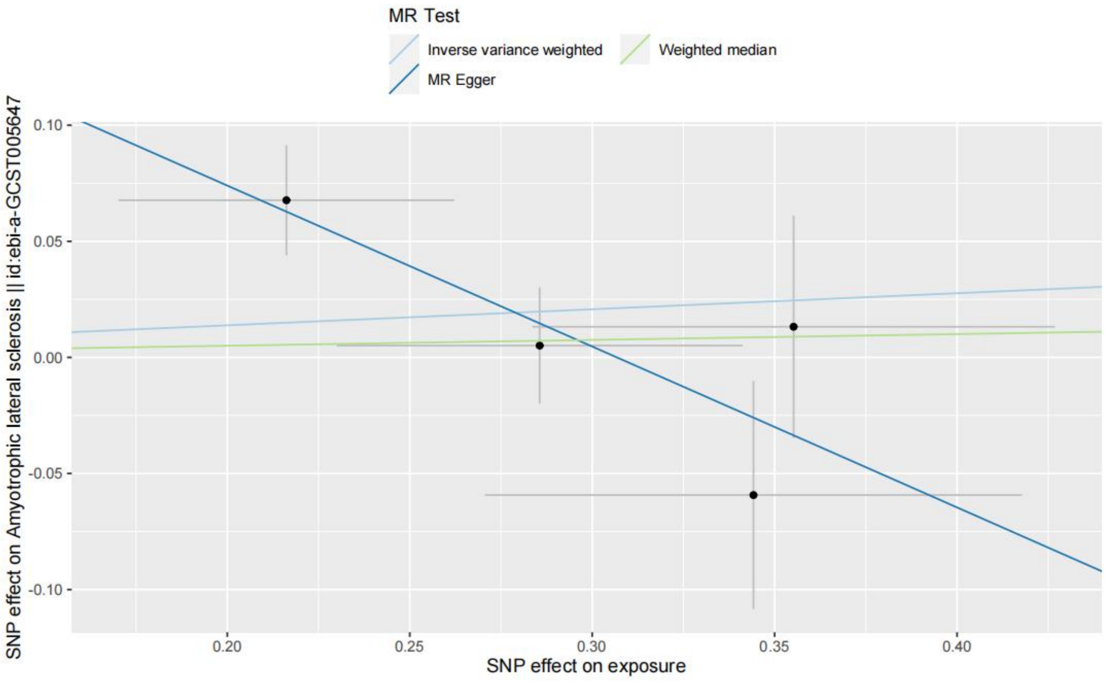

A. Scatter plot of IL-9 with risk of ALS

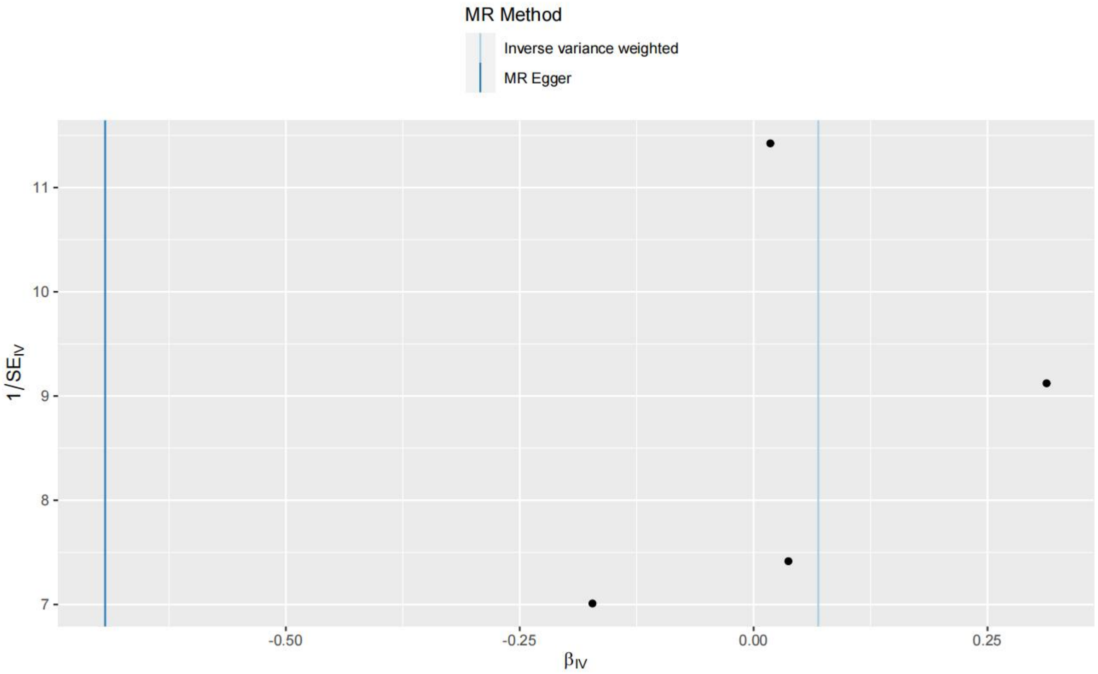

B. Funnel plot of IL-9 instruments strength on ALS

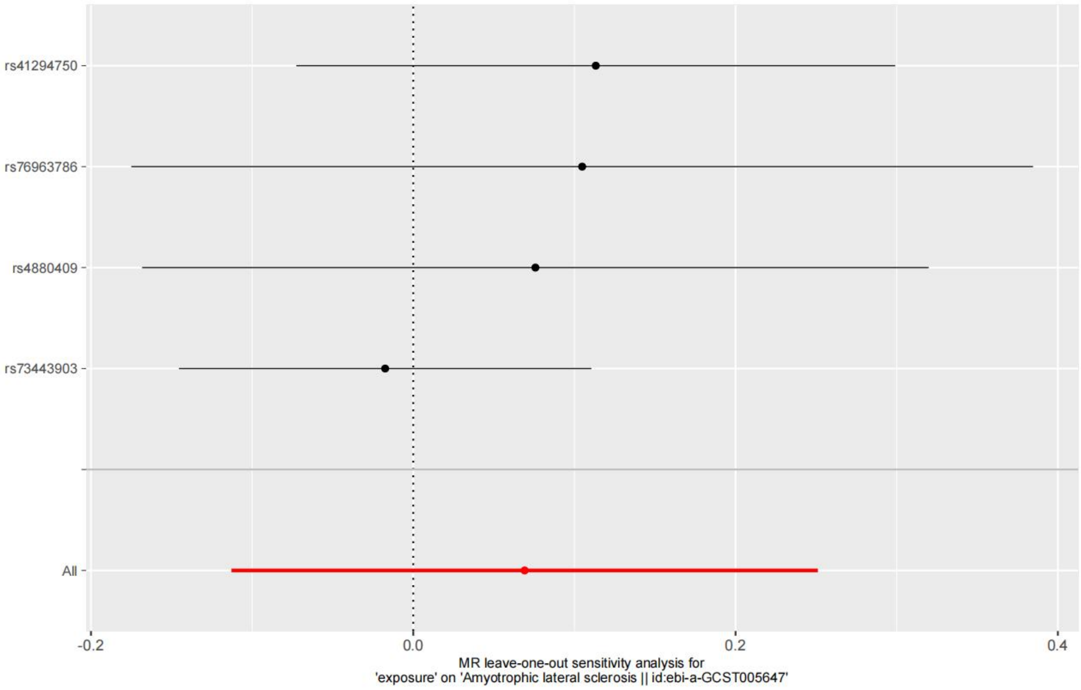

C. MR leave-one-out sensitivity analysis for IL-9 on ALS

eFigure 101. IL-10-associated SNPs with risk of ALS

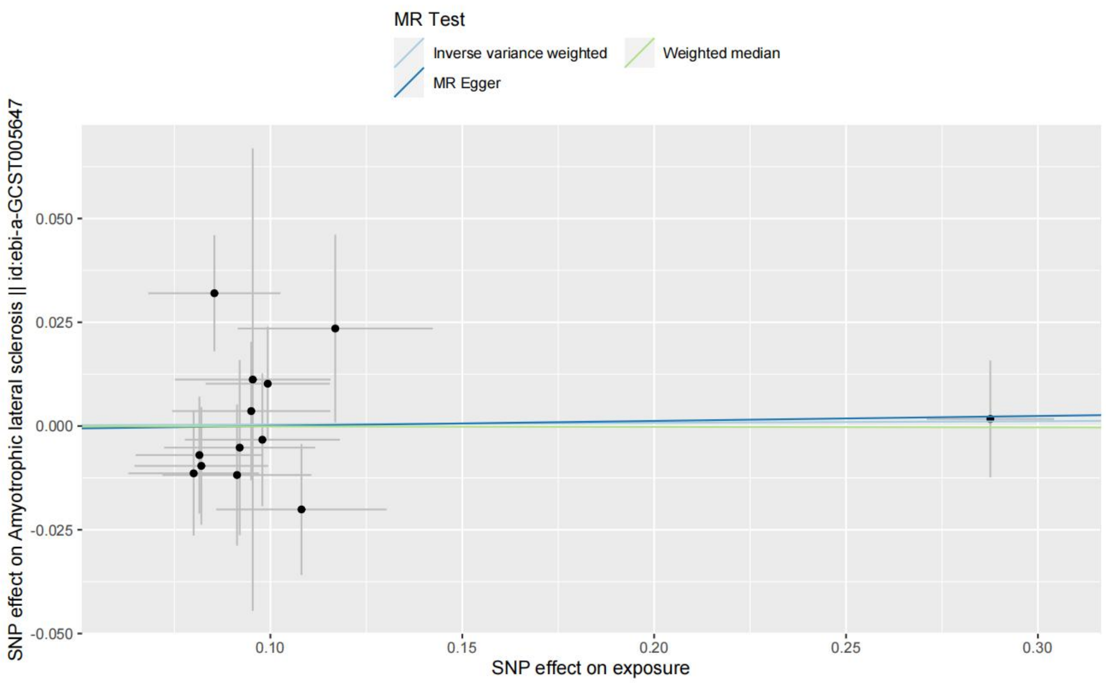

A. Scatter plot of IL-10 with risk of ALS

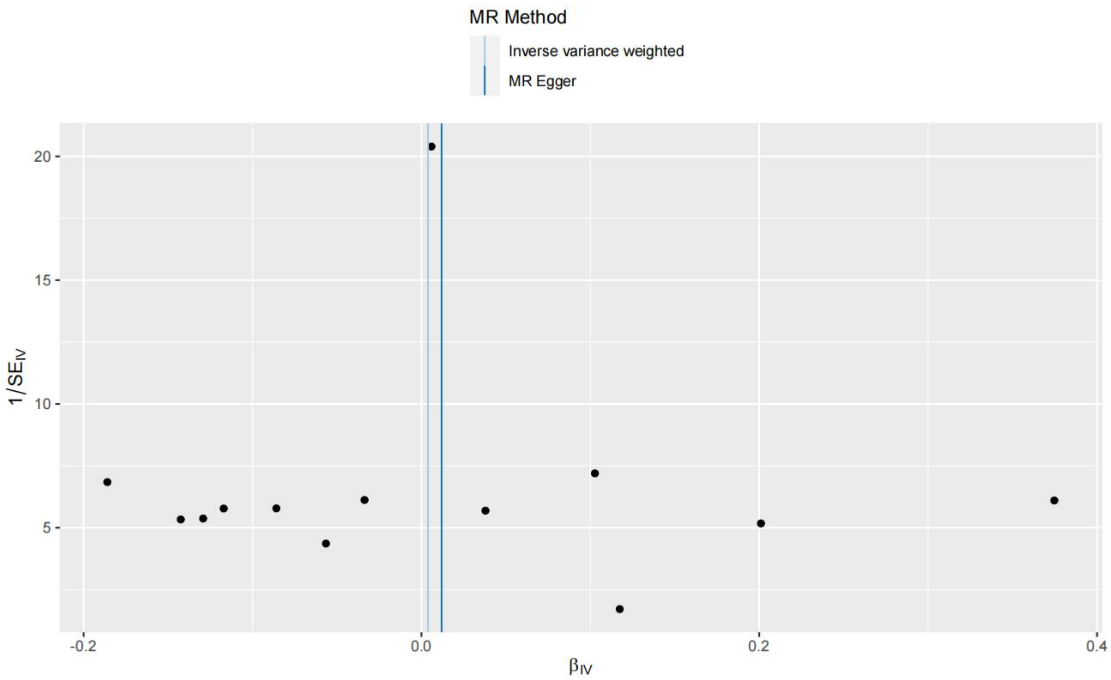

B. Funnel plot of IL-10 instruments strength on ALS

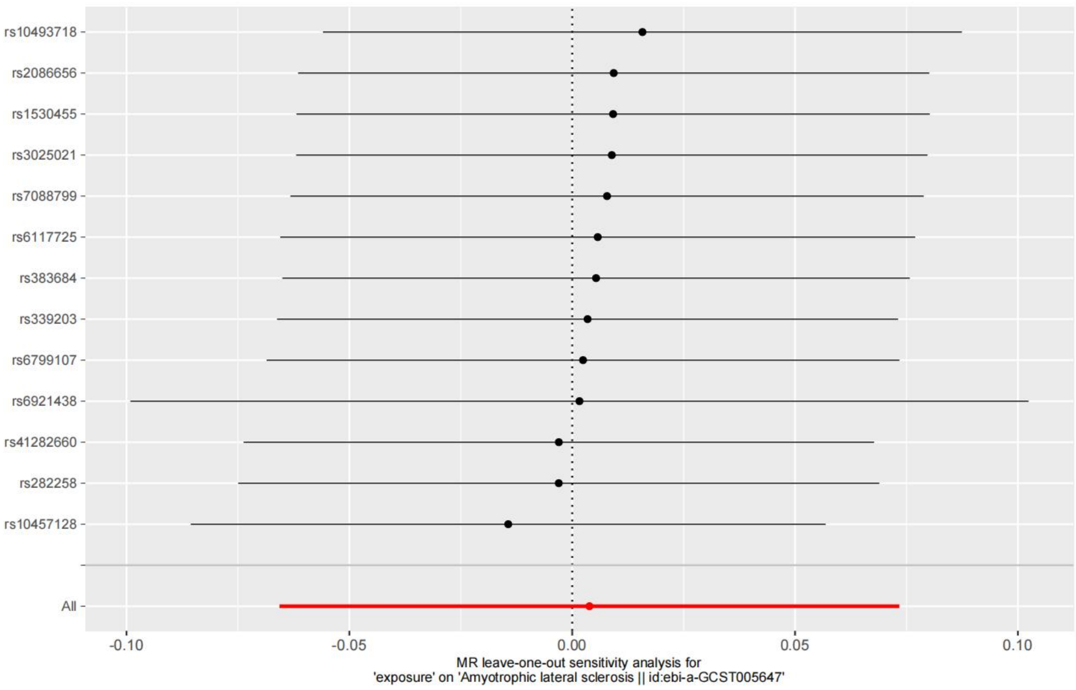

C. MR leave-one-out sensitivity analysis for IL-10 on ALS

eFigure 102. IL-12-associated SNPs with risk of ALS

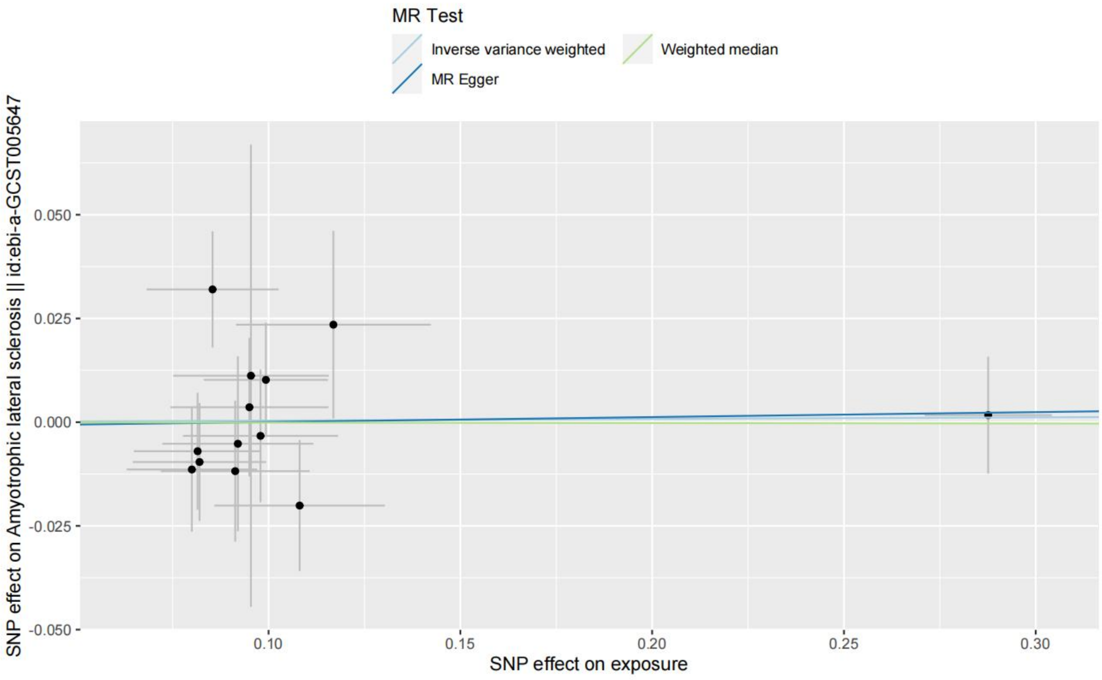

A. Scatter plot of IL-12 with risk of ALS

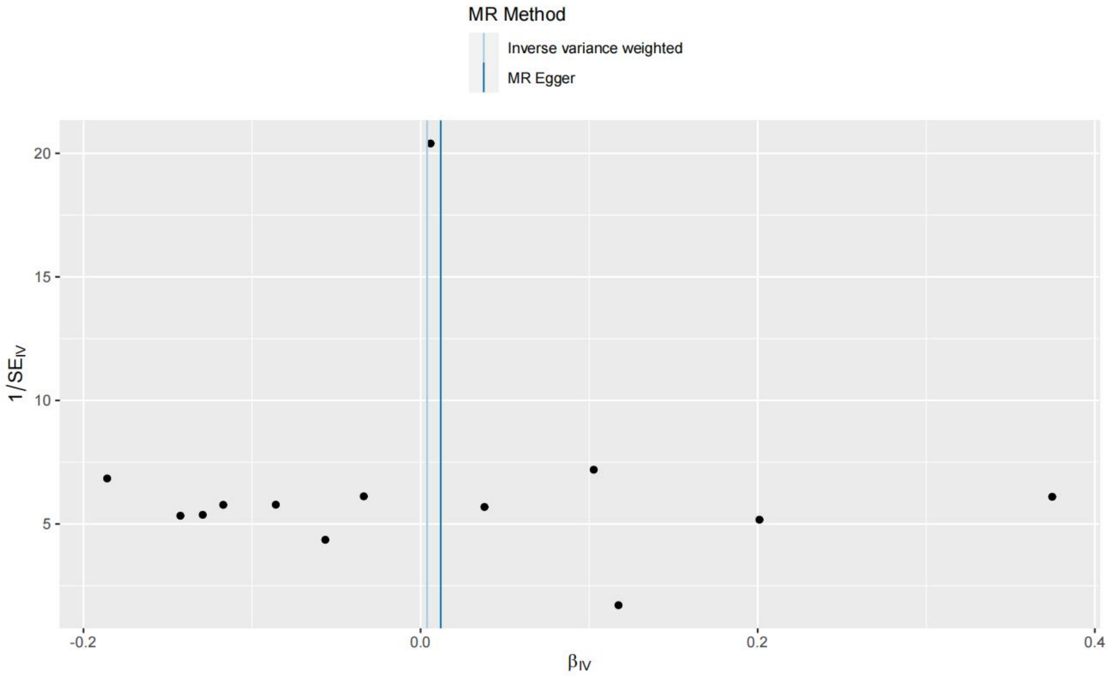

B. Funnel plot of IL-12 instruments strength on ALS

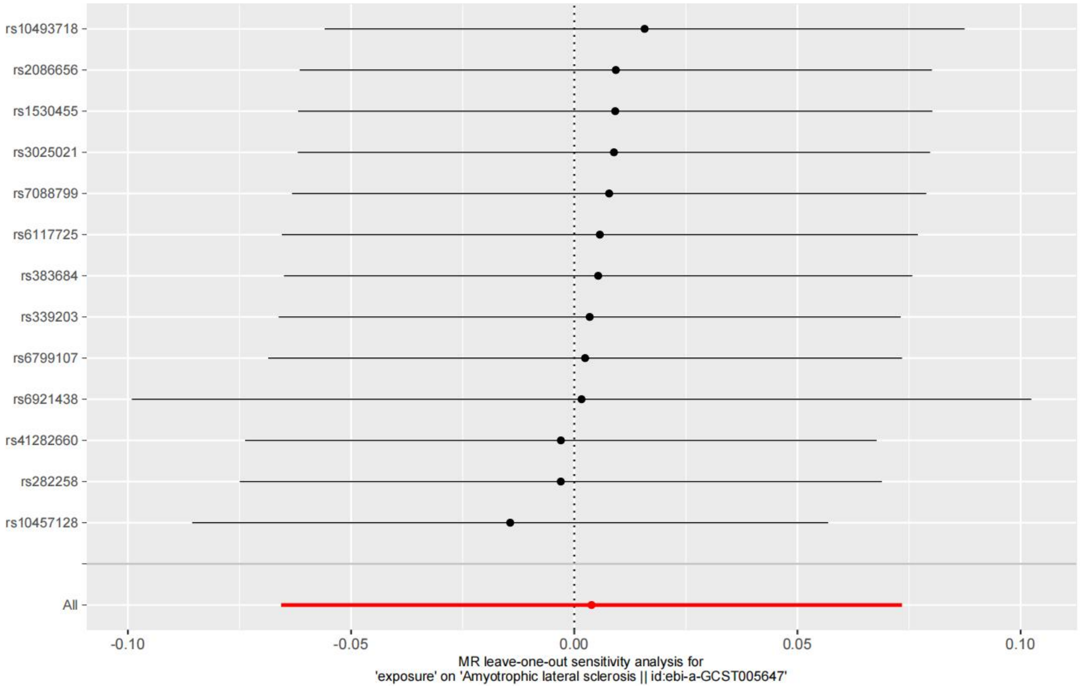

C. MR leave-one-out sensitivity analysis for IL-12 on ALS

eFigure 103. IL-13-associated SNPs with risk of ALS

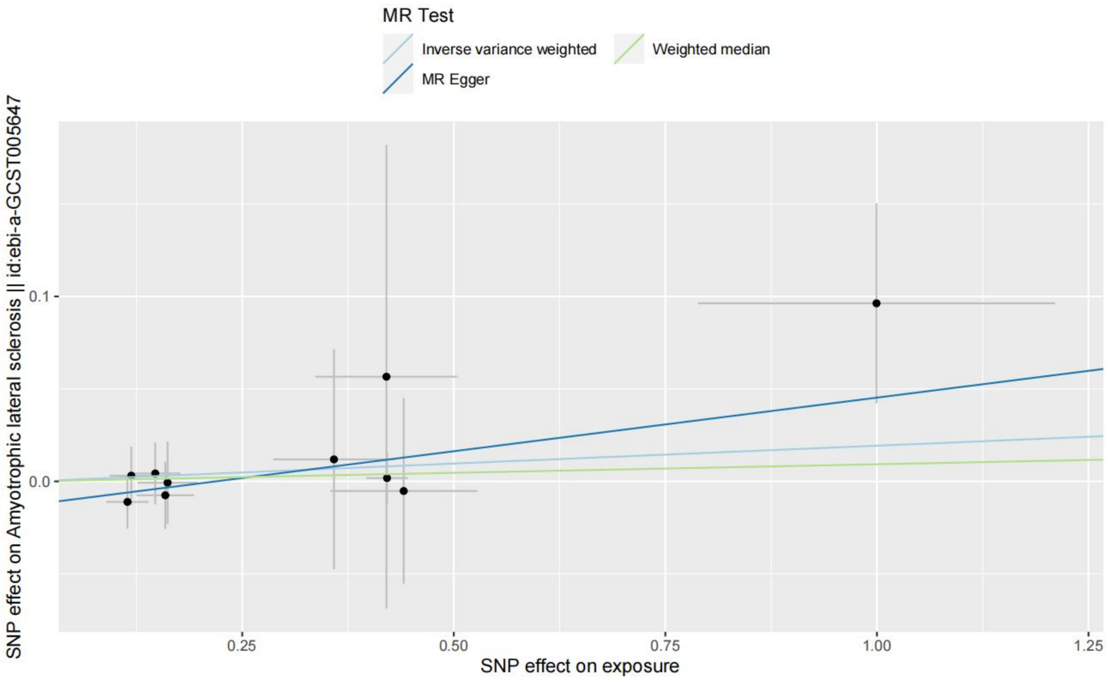

A. Scatter plot of IL-13 with risk of ALS

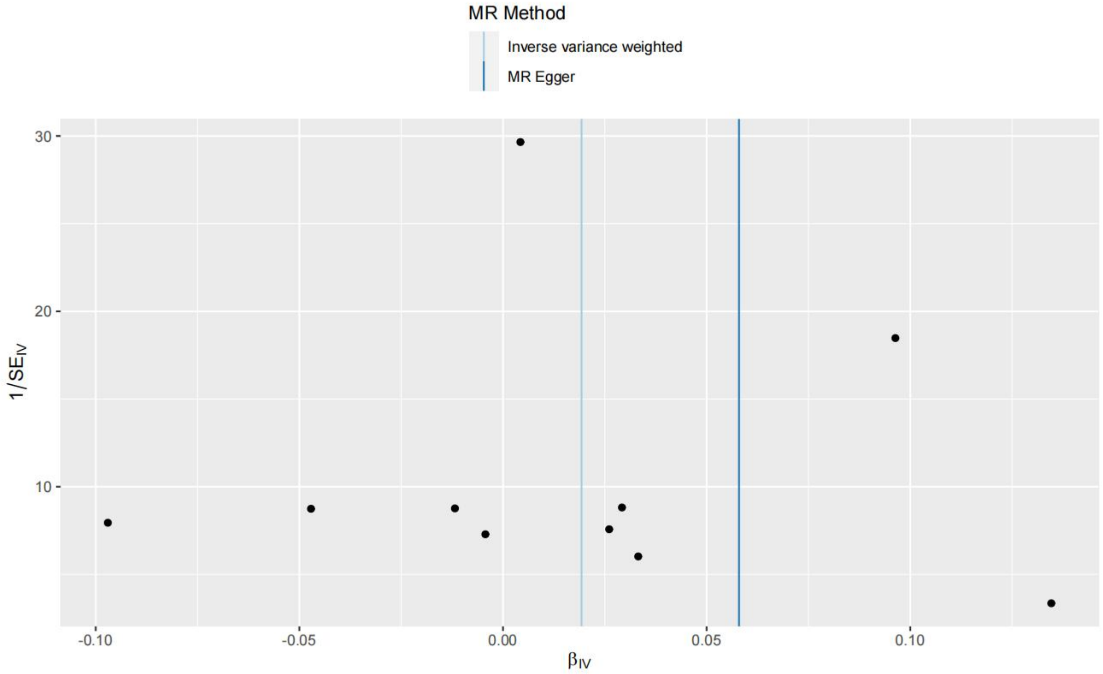

B. Funnel plot of IL-13 instruments strength on ALS

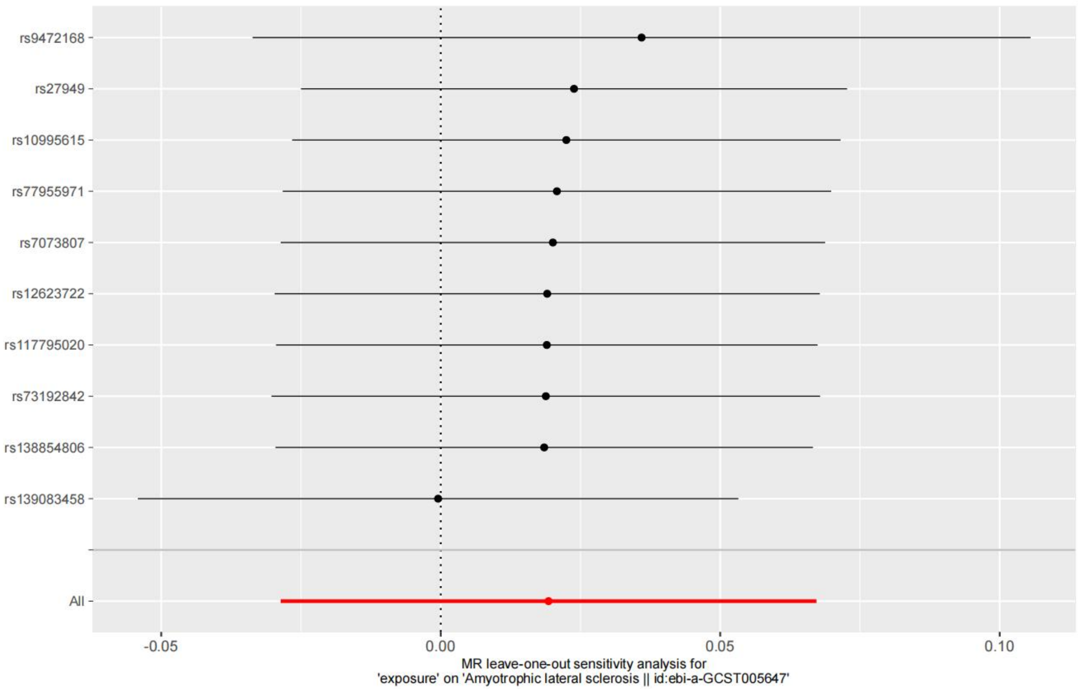

C. MR leave-one-out sensitivity analysis for IL-13 on ALS

eFigure 104. IL-16-associated SNPs with risk of ALS

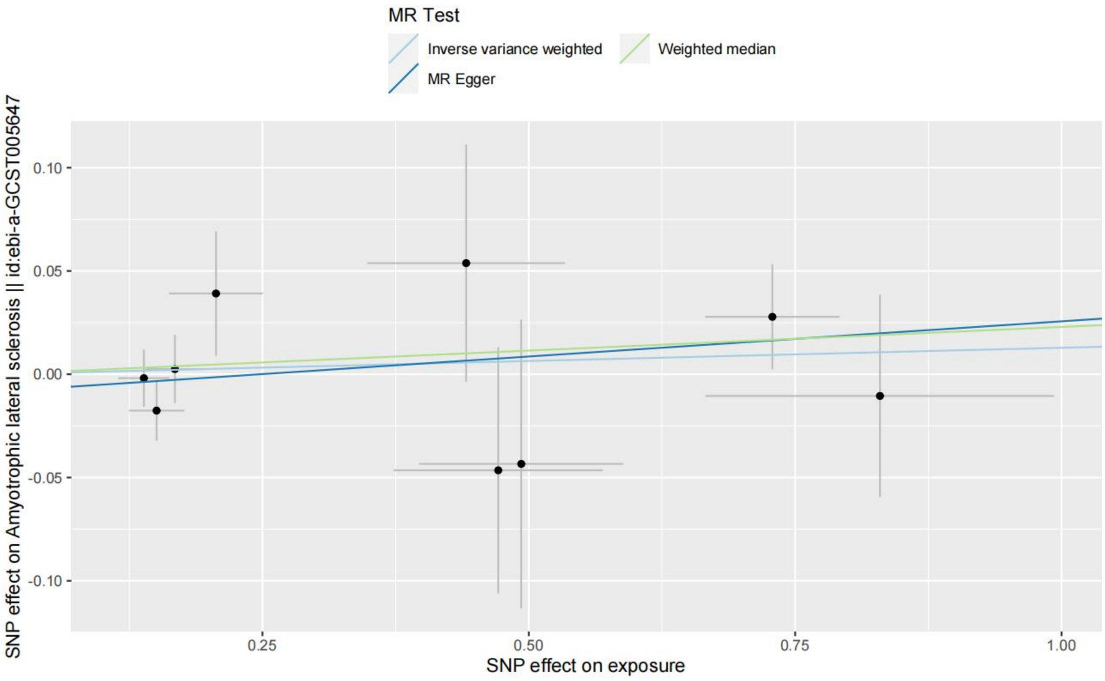

A. Scatter plot of IL-16 with risk of ALS

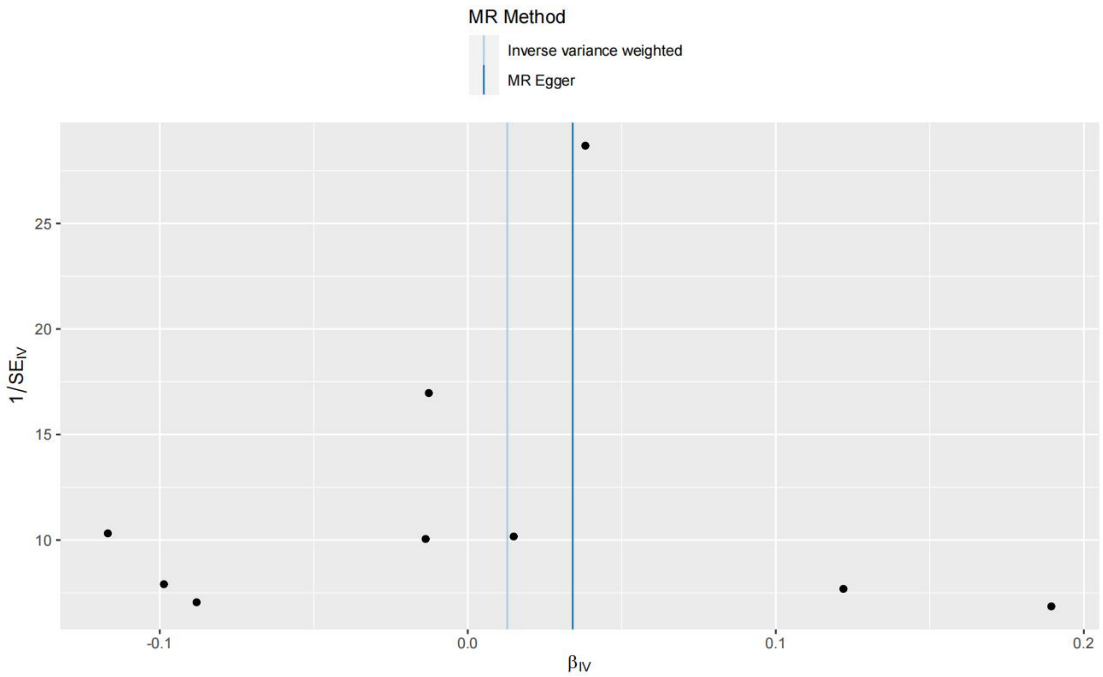

B. Funnel plot of IL-16 instruments strength on ALS

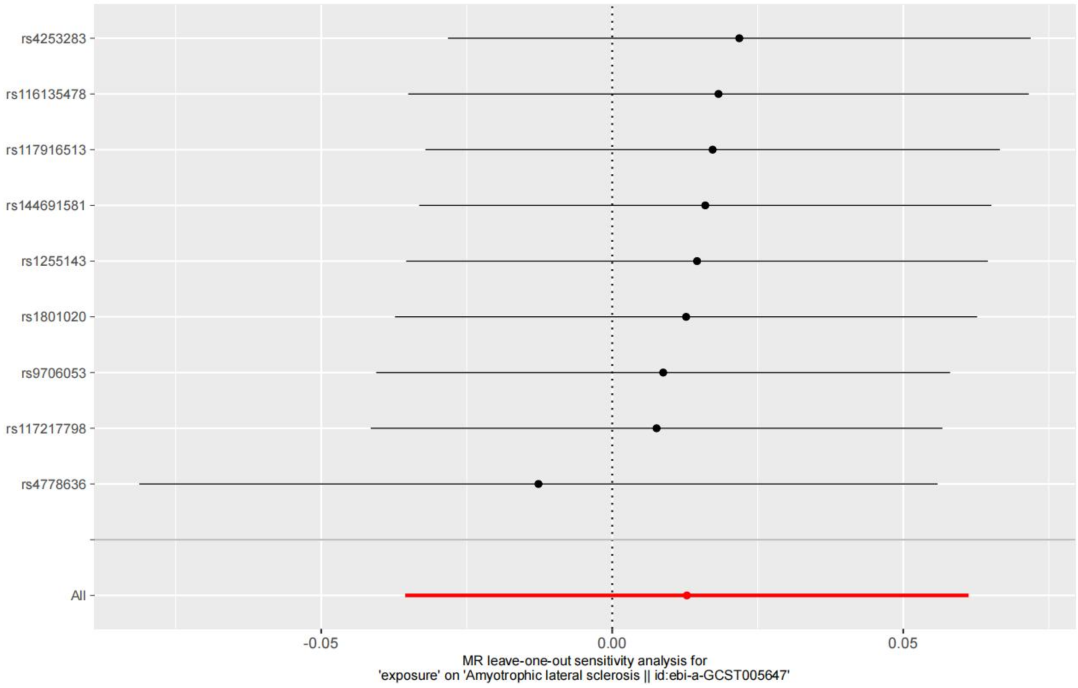

C. MR leave-one-out sensitivity analysis for IL-16 on ALS

eFigure 105. IL-17-associated SNPs with risk of ALS

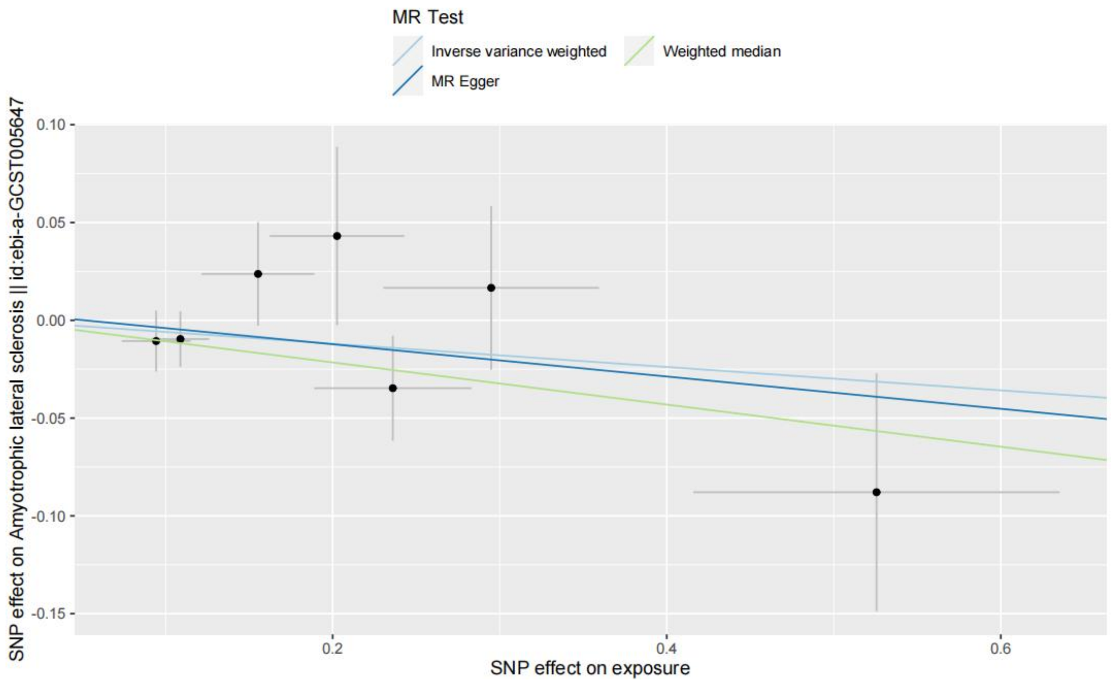

A. Scatter plot of IL-17 with risk of ALS

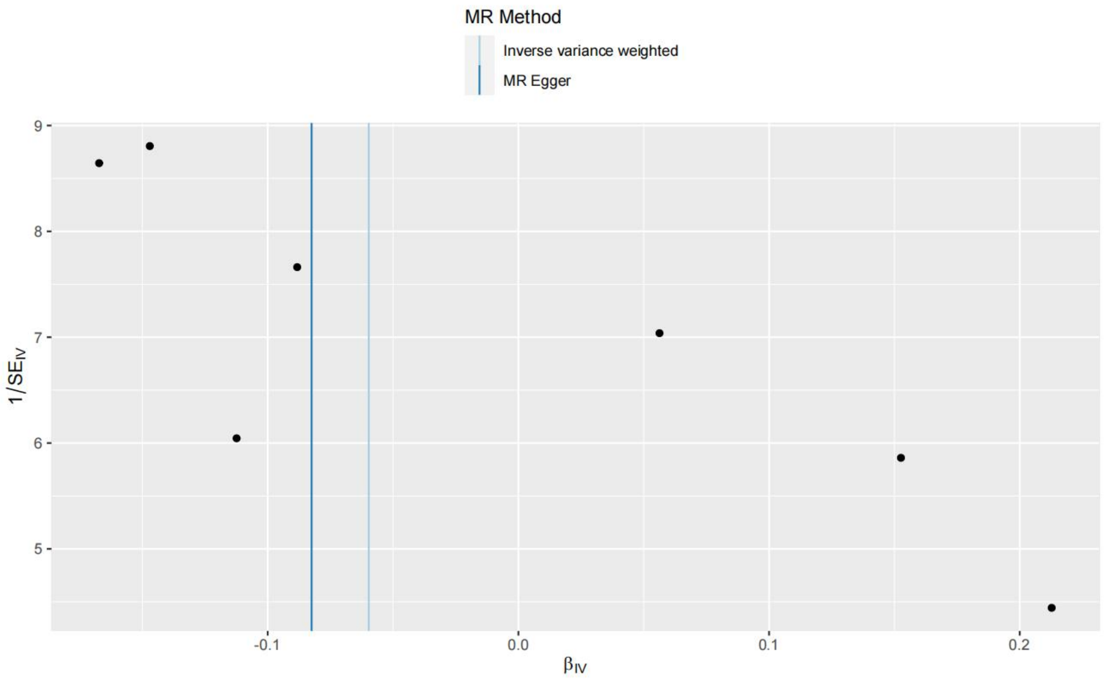

B. Funnel plot of IL-17 instruments strength on ALS

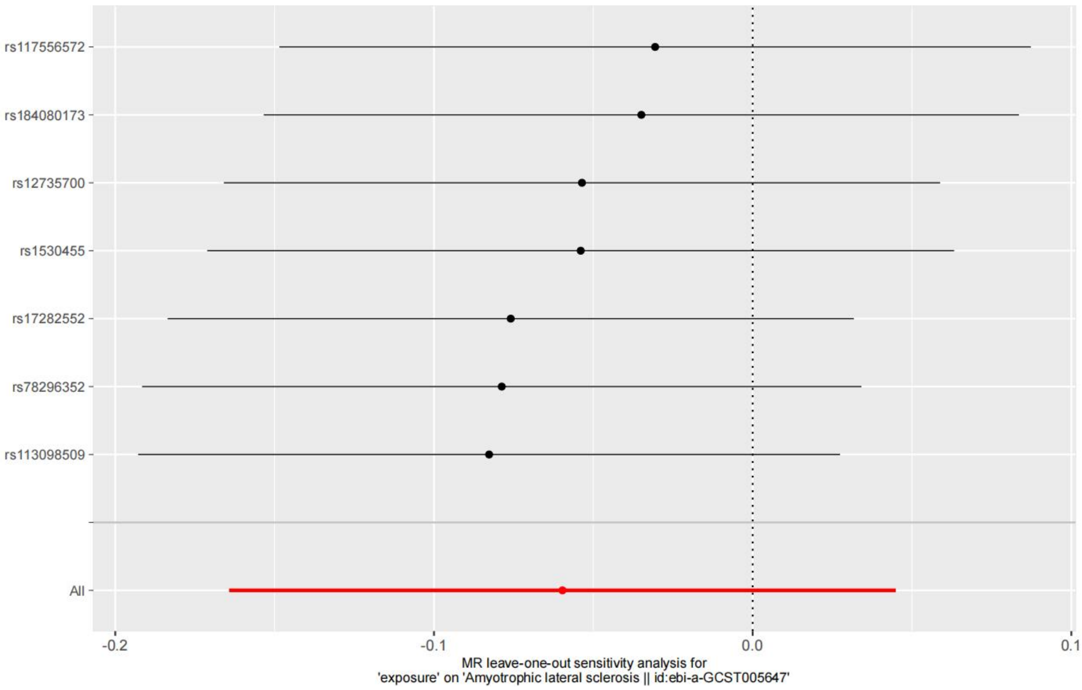

C. MR leave-one-out sensitivity analysis for IL-17 on ALS

eFigure 106. IL-18-associated SNPs with risk of ALS

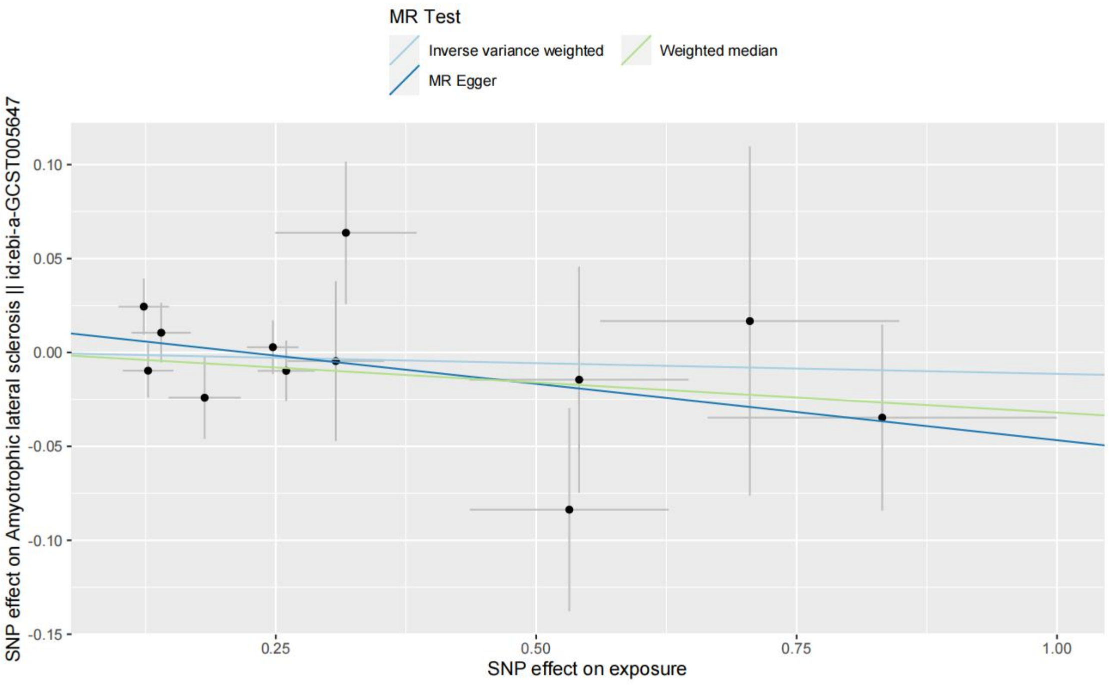

A. Scatter plot of IL-18 with risk of ALS

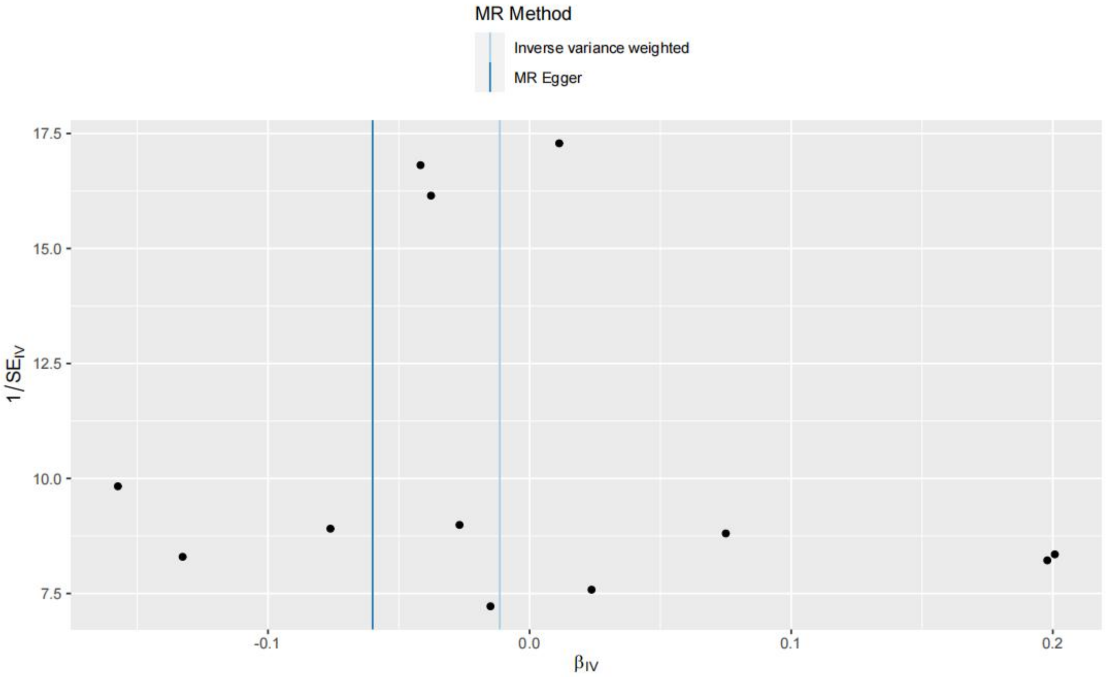

B. Funnel plot of IL-18 instruments strength on ALS

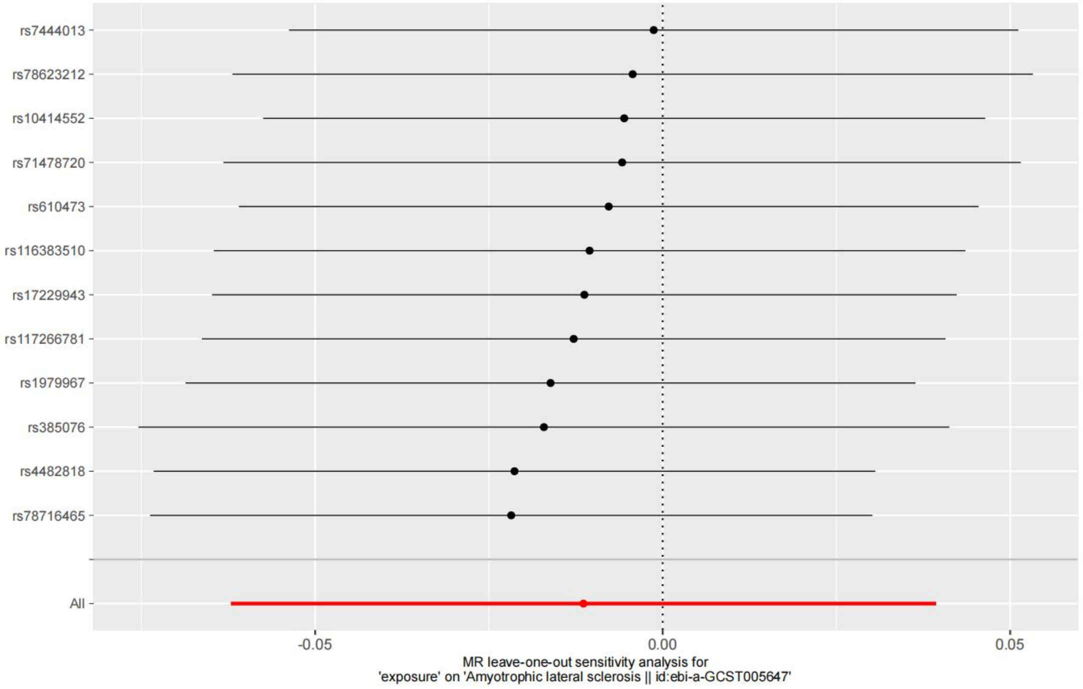

C. MR leave-one-out sensitivity analysis for IL-18 on ALS

eFigure 107. IP-10-associated SNPs with risk of ALS

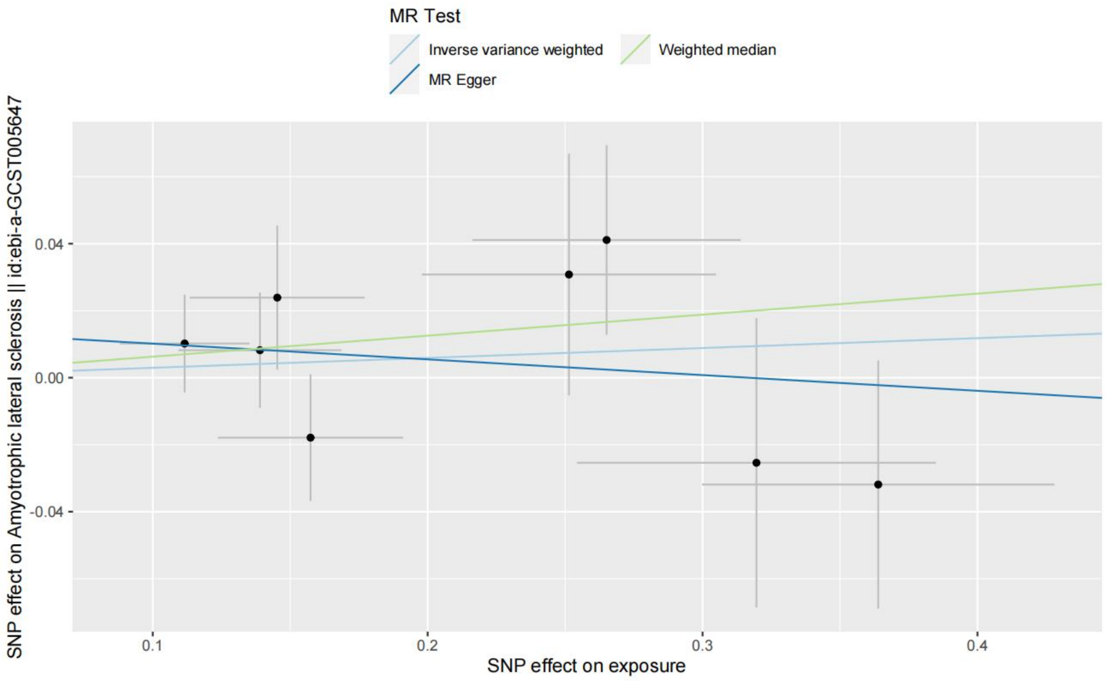

A. Scatter plot of IP-10 with risk of ALS

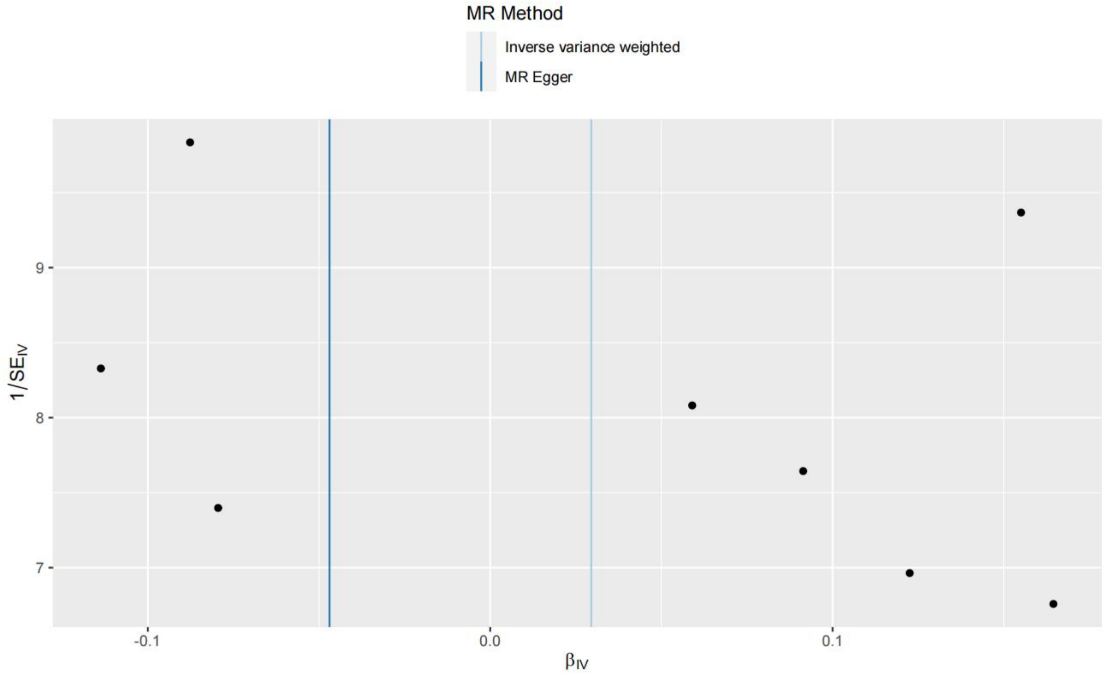

B. Funnel plot of IP-10 instruments strength on ALS

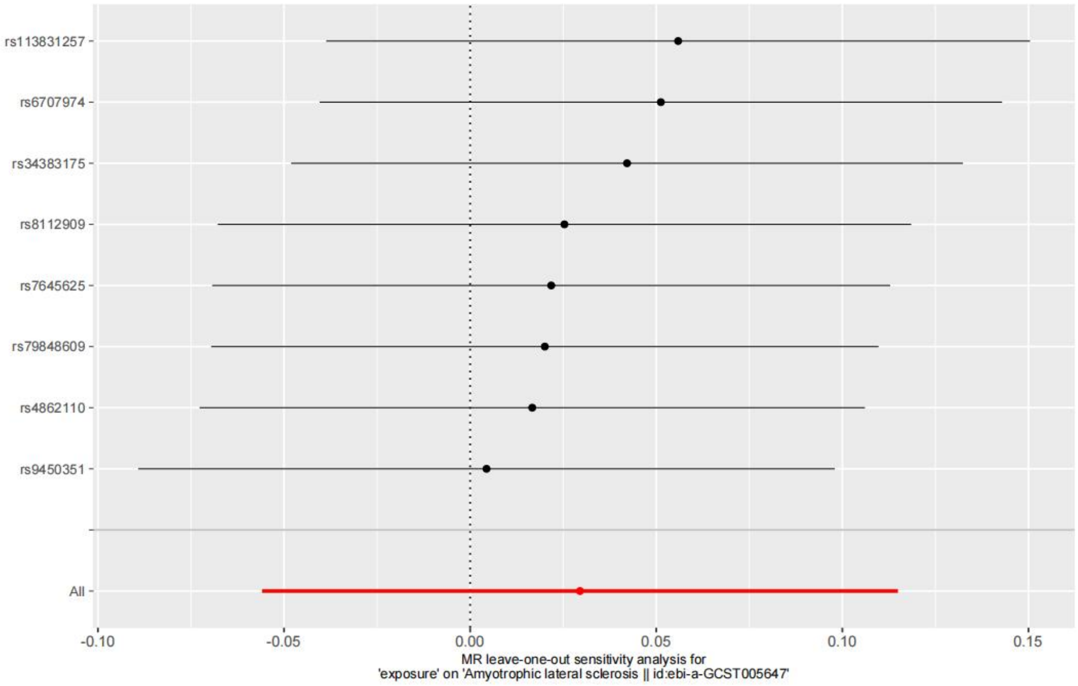

C. MR leave-one-out sensitivity analysis for IP-10 on ALS

eFigure 108. M-CSF-associated SNPs with risk of ALS

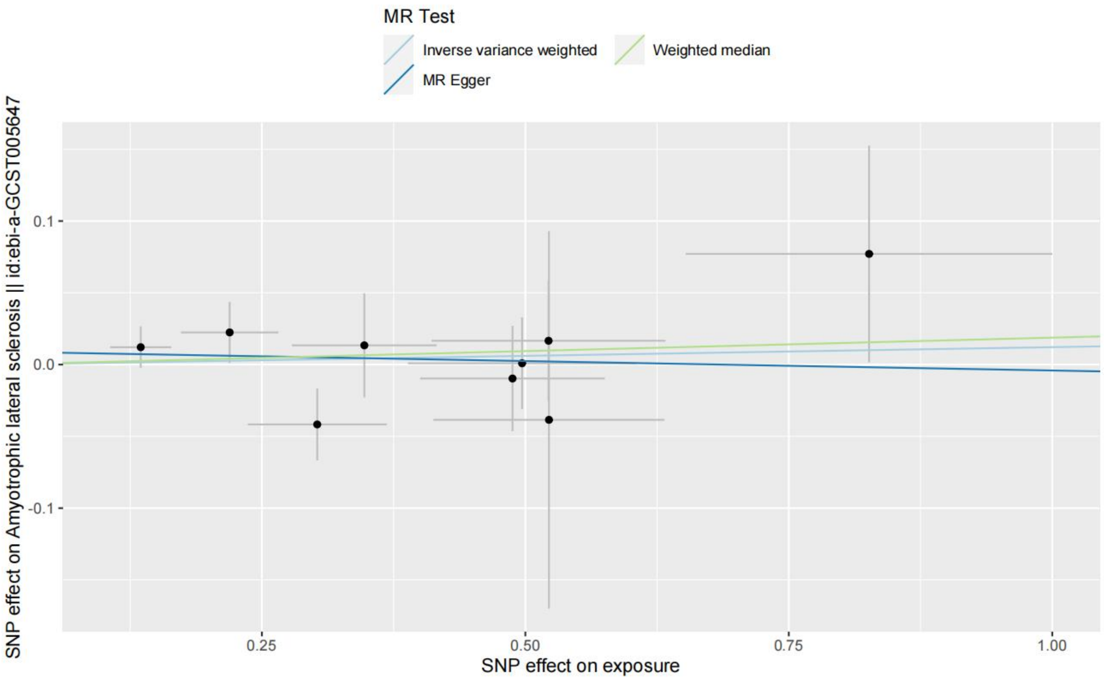

A. Scatter plot of M-CSF with risk of ALS

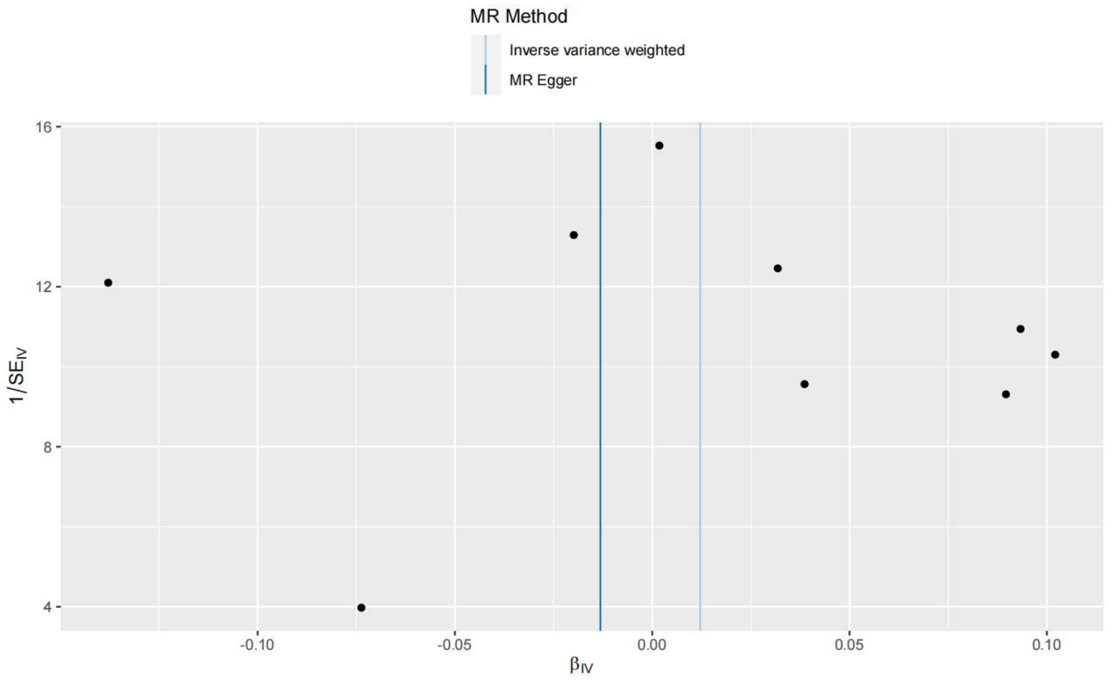

B. Funnel plot of M-CSF instruments strength on ALS

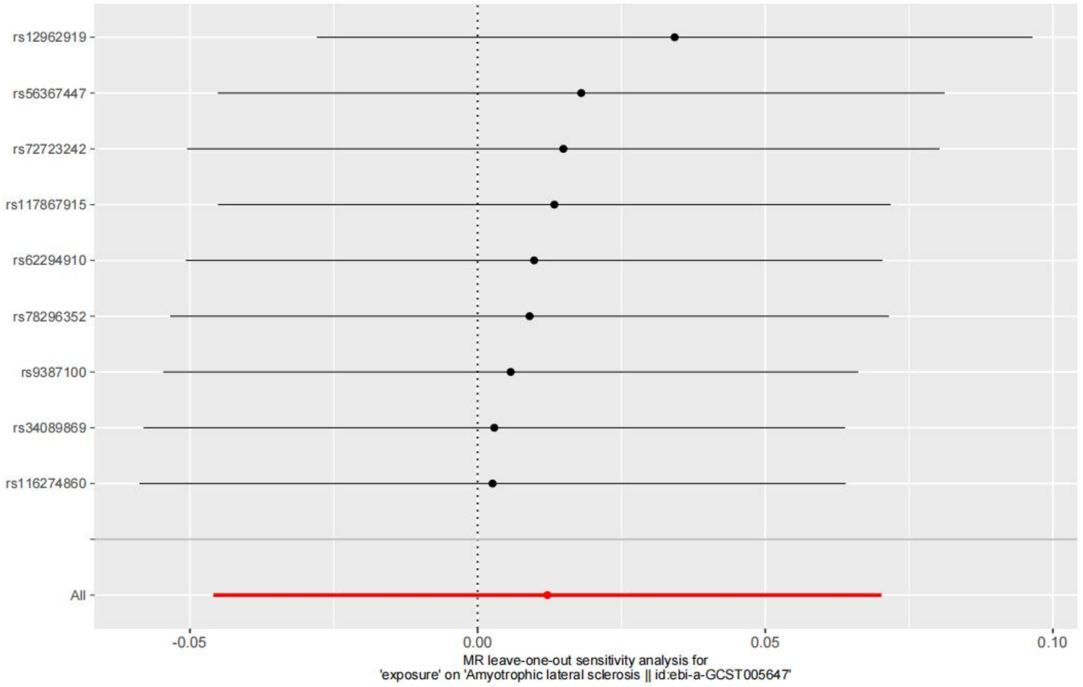

C. MR leave-one-out sensitivity analysis for M-CSF on ALS

eFigure 109. MCP-1-MCAF-associated SNPs with risk of ALS

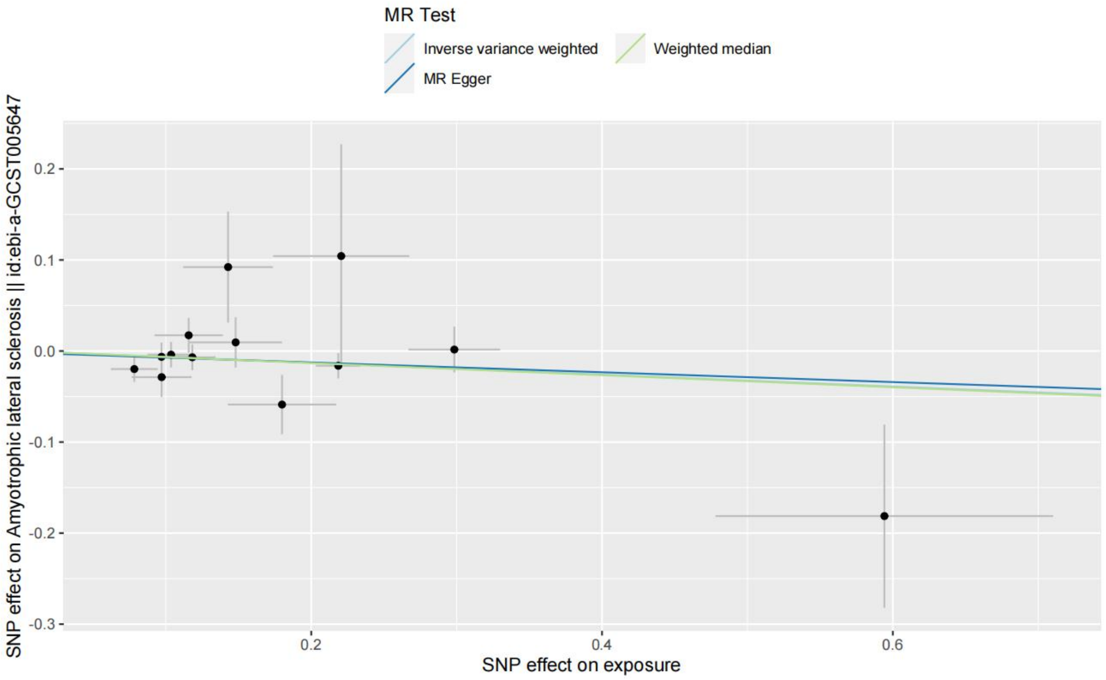

A. Scatter plot of MCP-1-MCAF with risk of ALS

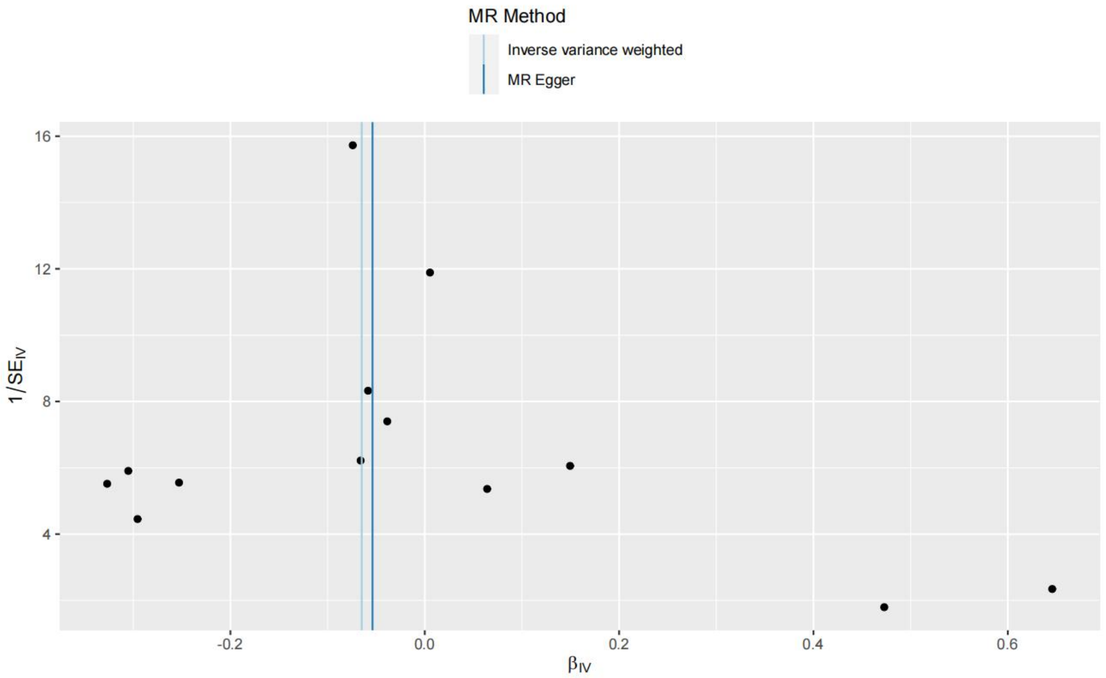

B. Funnel plot of MCP-1-MCAF instruments strength on ALS

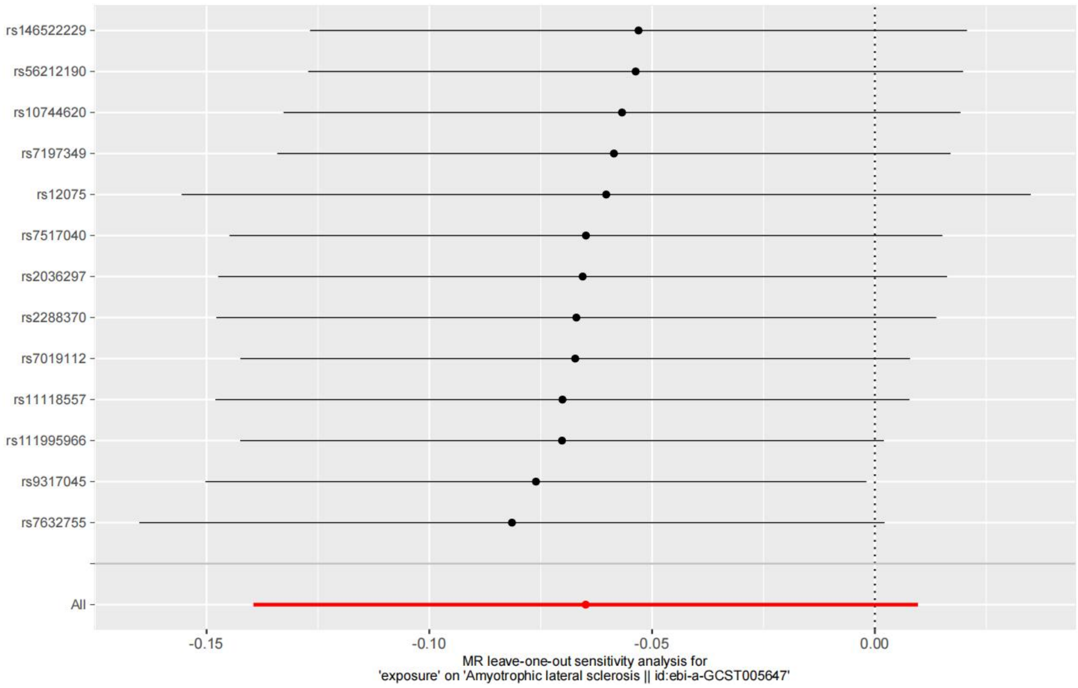

C. MR leave-one-out sensitivity analysis for MCP-1-MCAF on ALS

eFigure 110. MCP-3-associated SNPs with risk of ALS

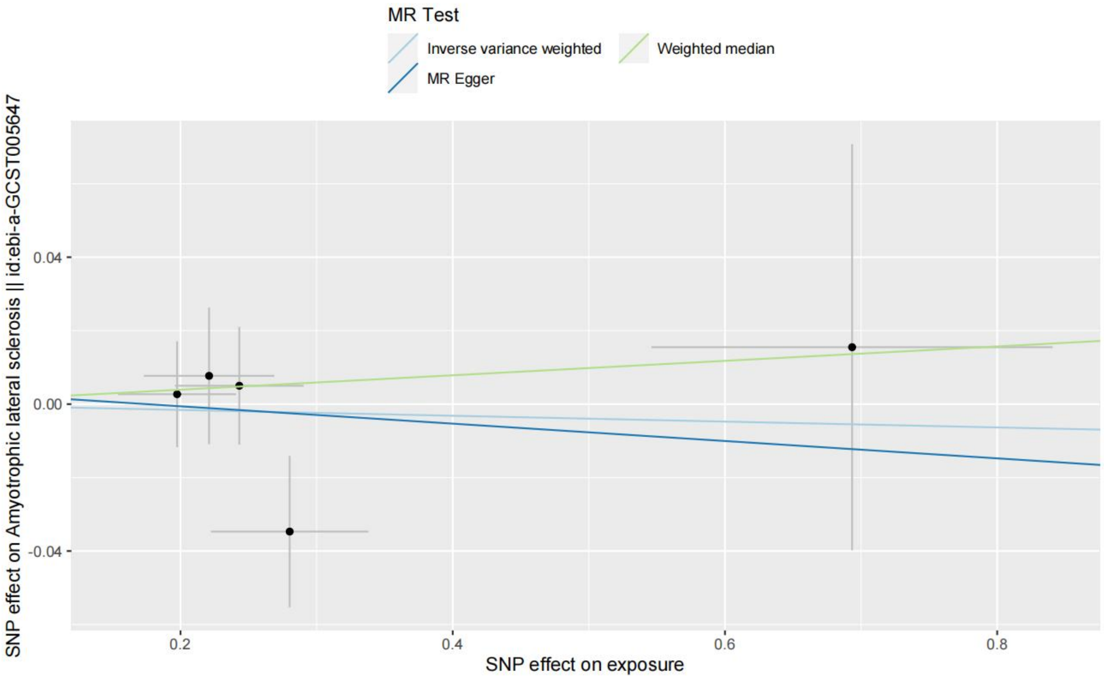

A. Scatter plot of MCP-3 with risk of ALS

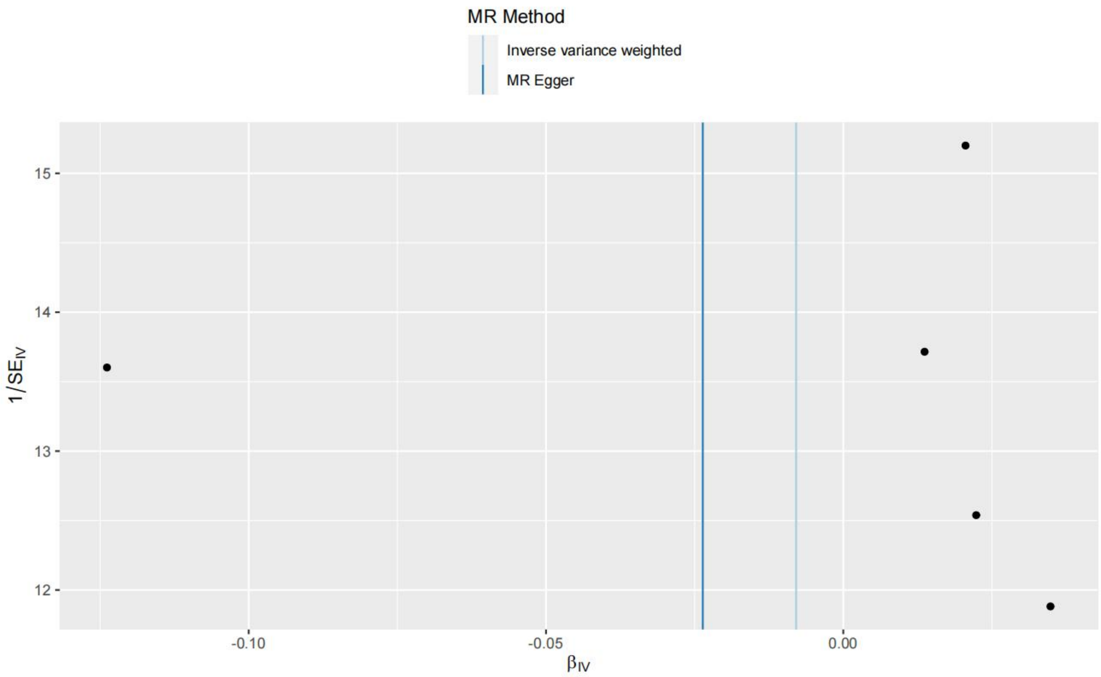

B. Funnel plot of MCP-3 instruments strength on ALS

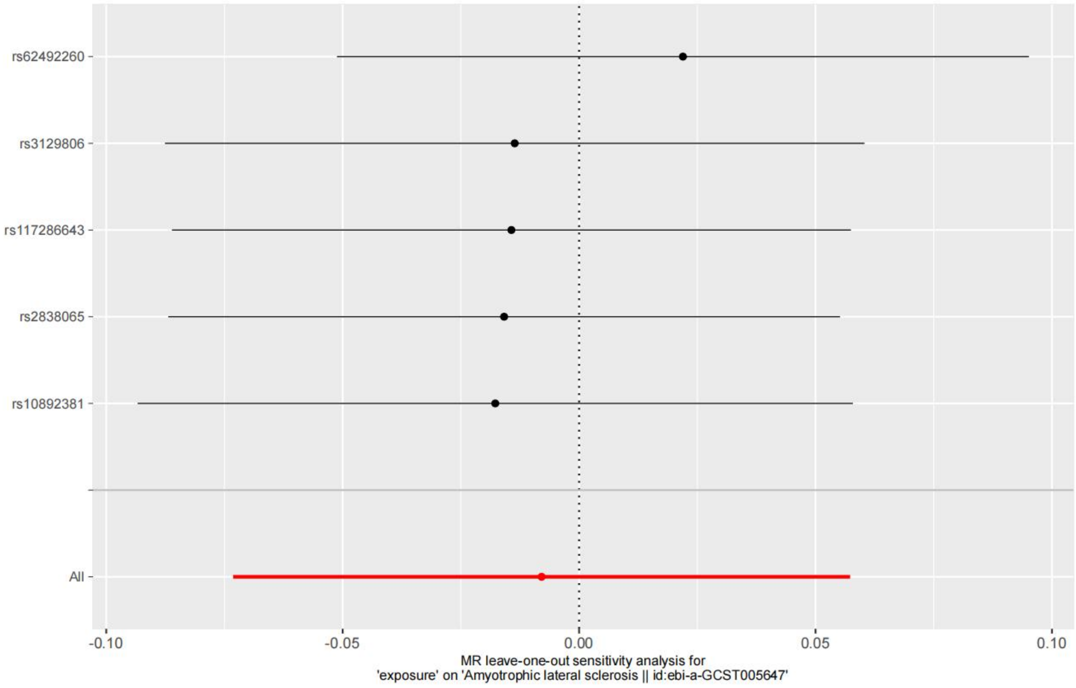

C. MR leave-one-out sensitivity analysis for MCP-3 on ALS

eFigure 111. MIF-associated SNPs with risk of ALS

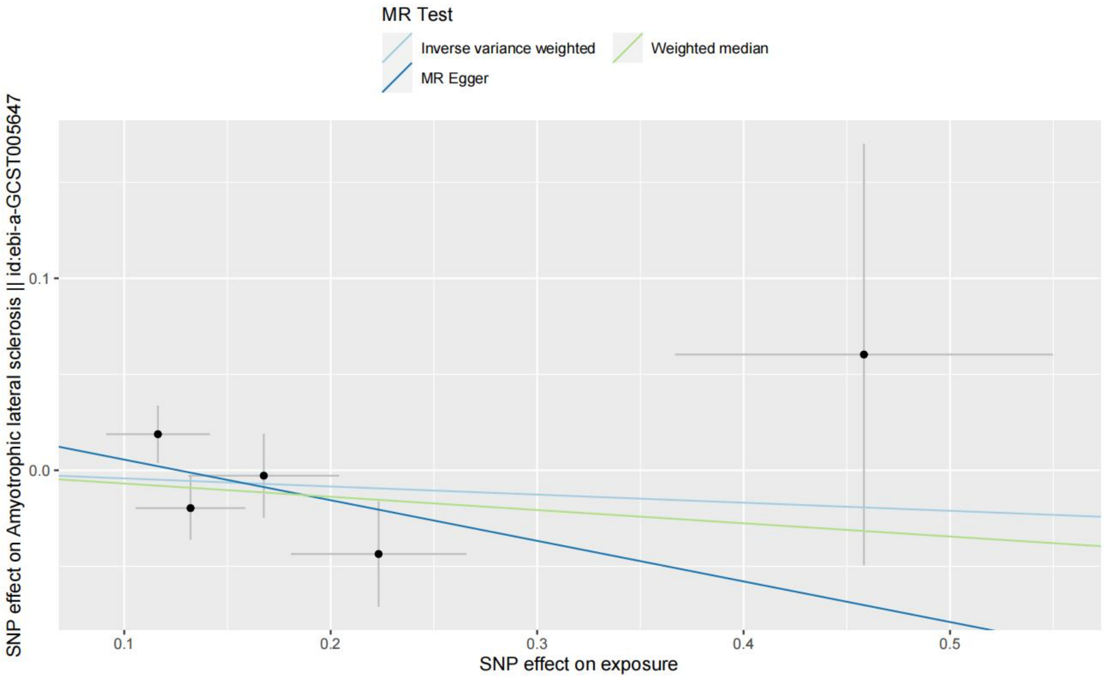

A. Scatter plot of MIF with risk of ALS

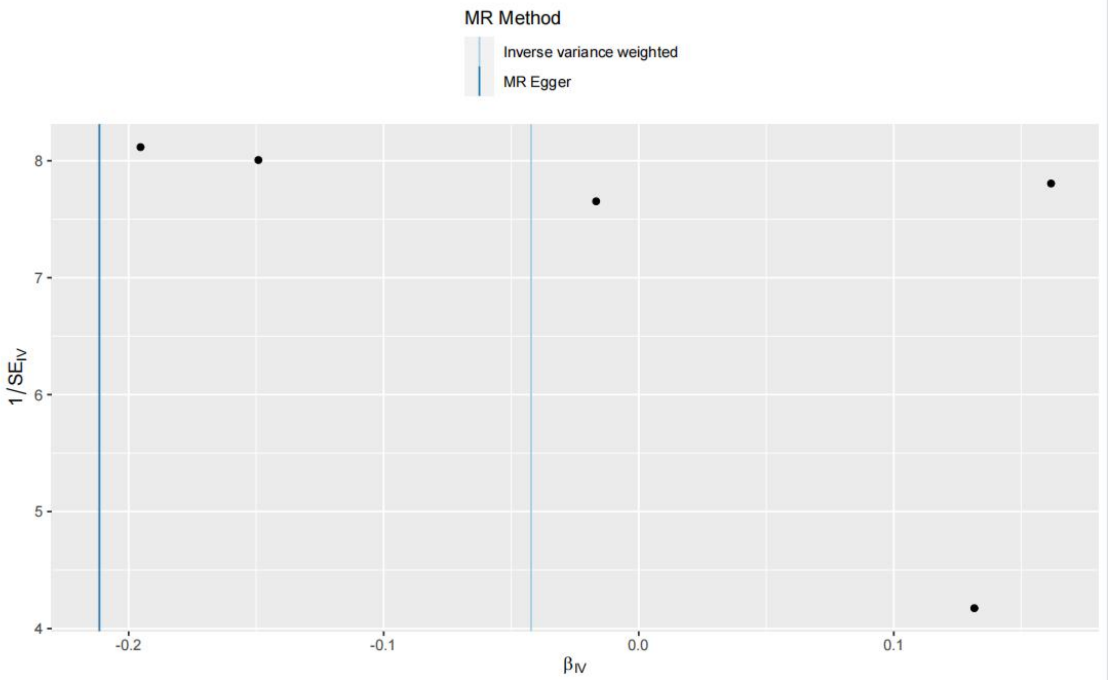

B. Funnel plot of MIF instruments strength on ALS

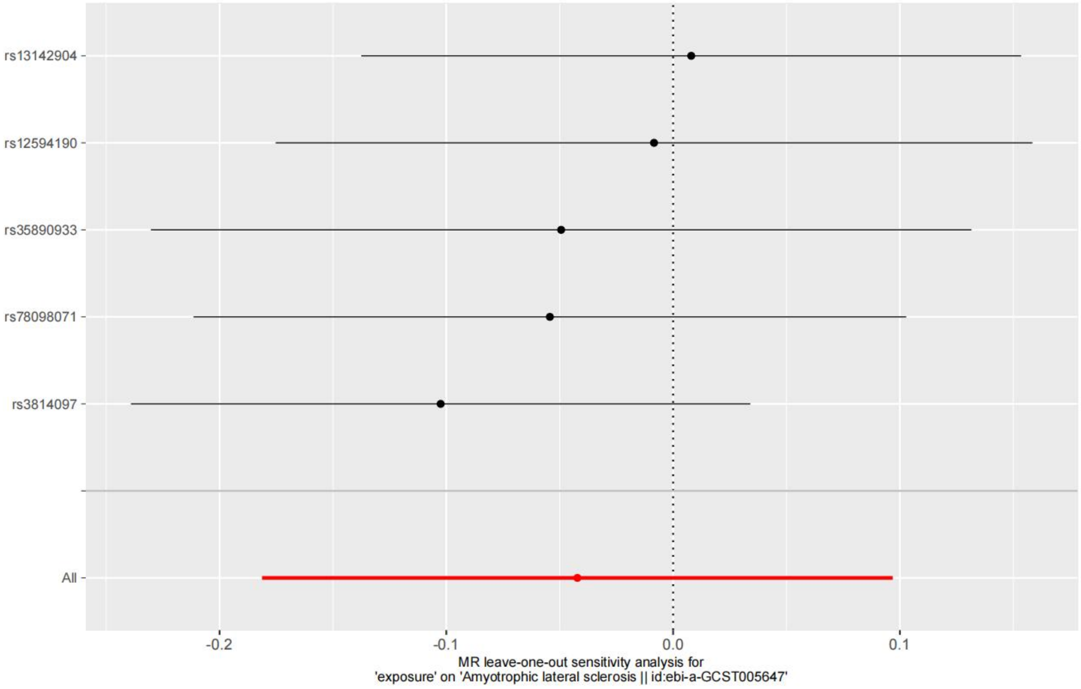

C. MR leave-one-out sensitivity analysis for MIF on ALS

eFigure 112. MIG-associated SNPs with risk of ALS

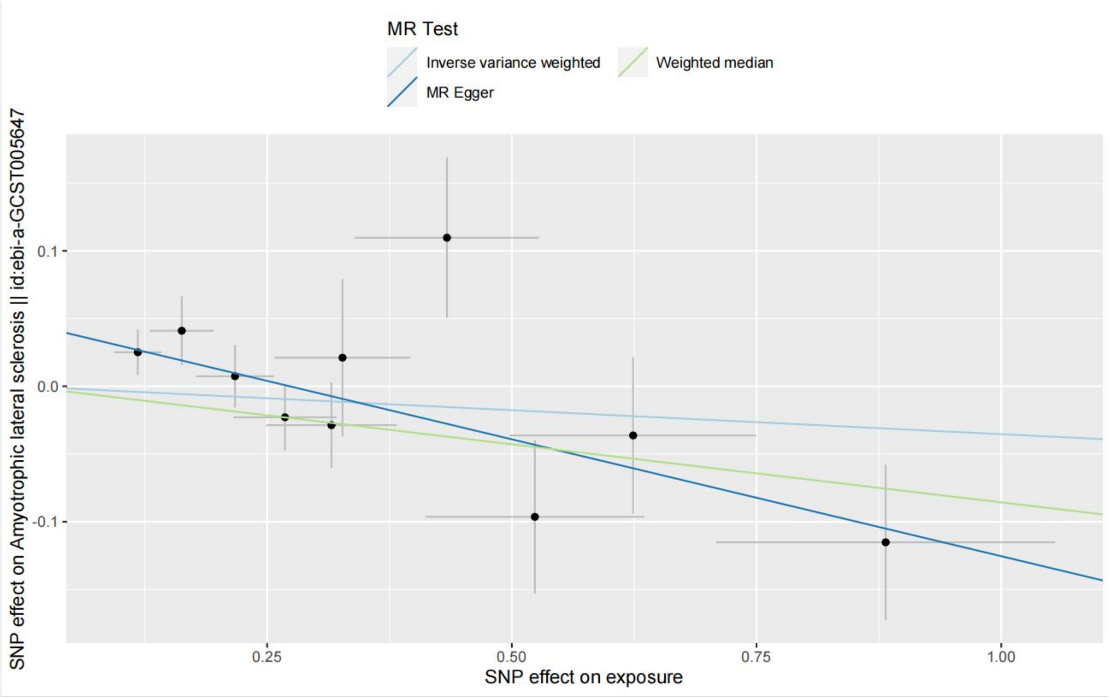

A. Scatter plot of MIG with risk of ALS

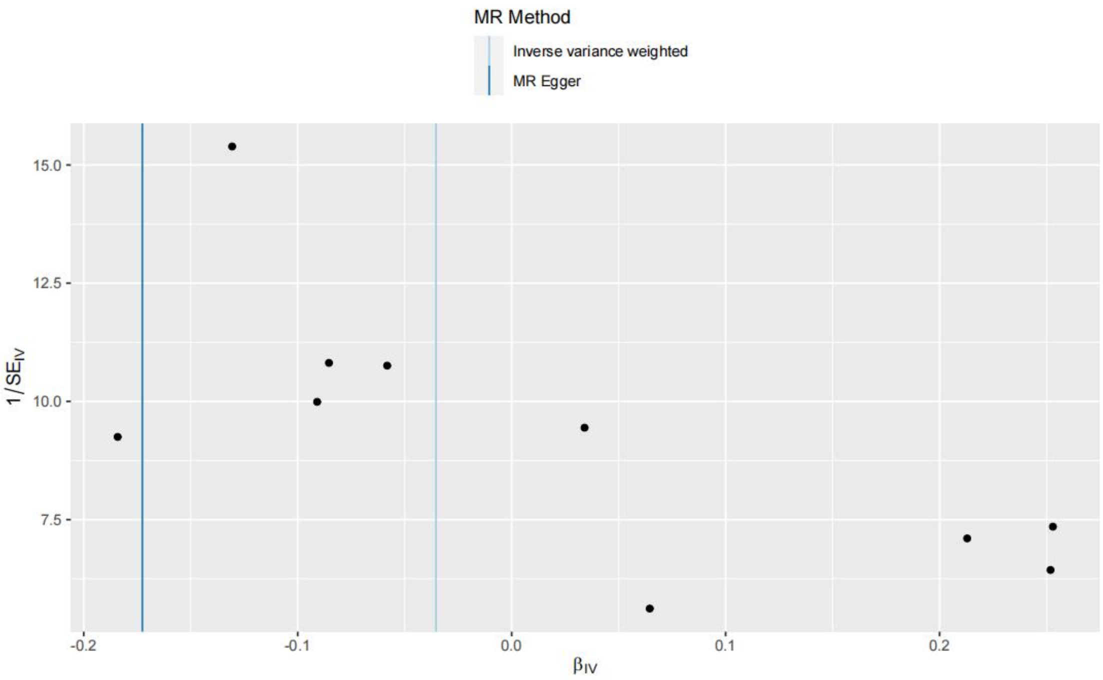

B. Funnel plot of MIG instruments strength on ALS

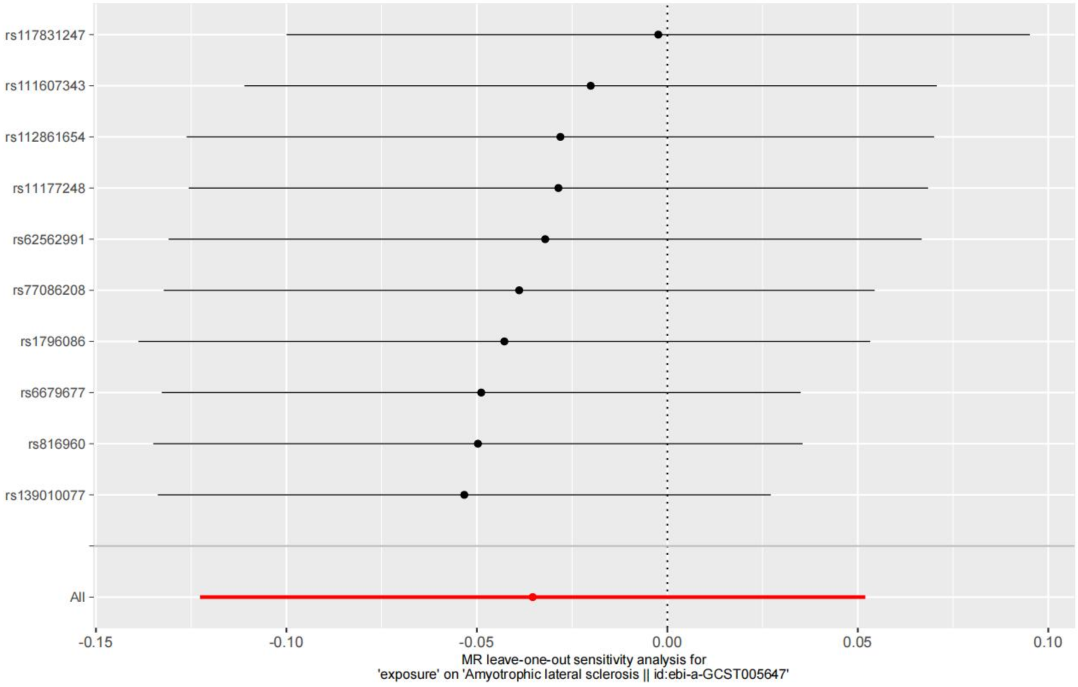

C. MR leave-one-out sensitivity analysis for MIG on ALS

eFigure 113. MIP-1A-associated SNPs with risk of ALS

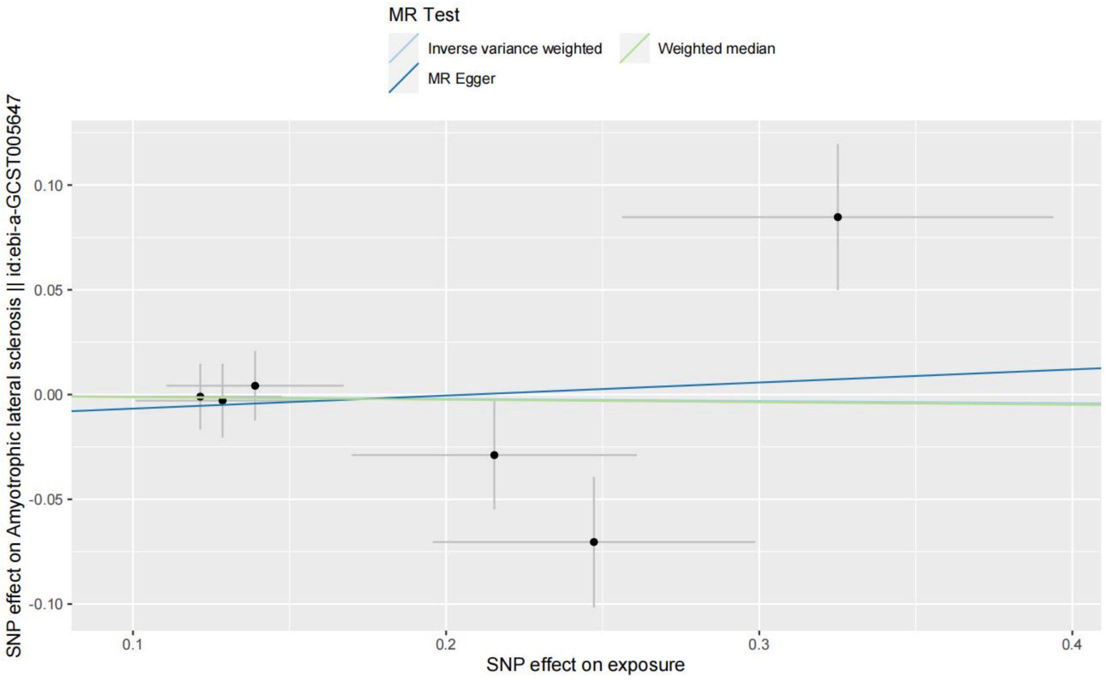

A. Scatter plot of MIP-1A with risk of ALS

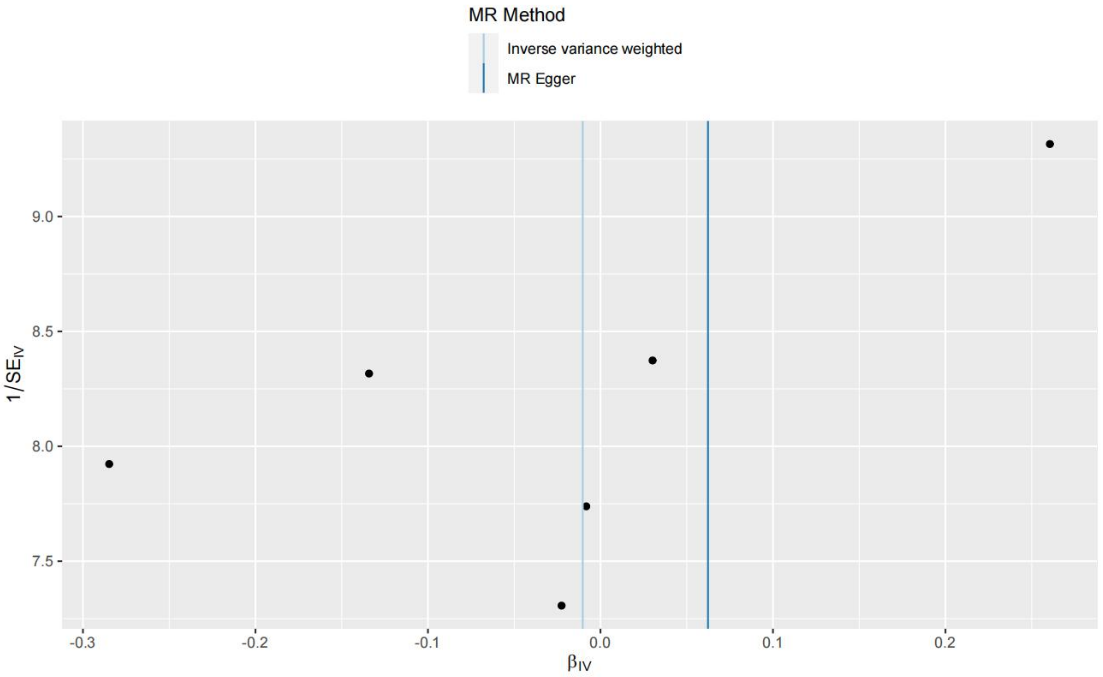

B. Funnel plot of MIP-1A instruments strength on ALS

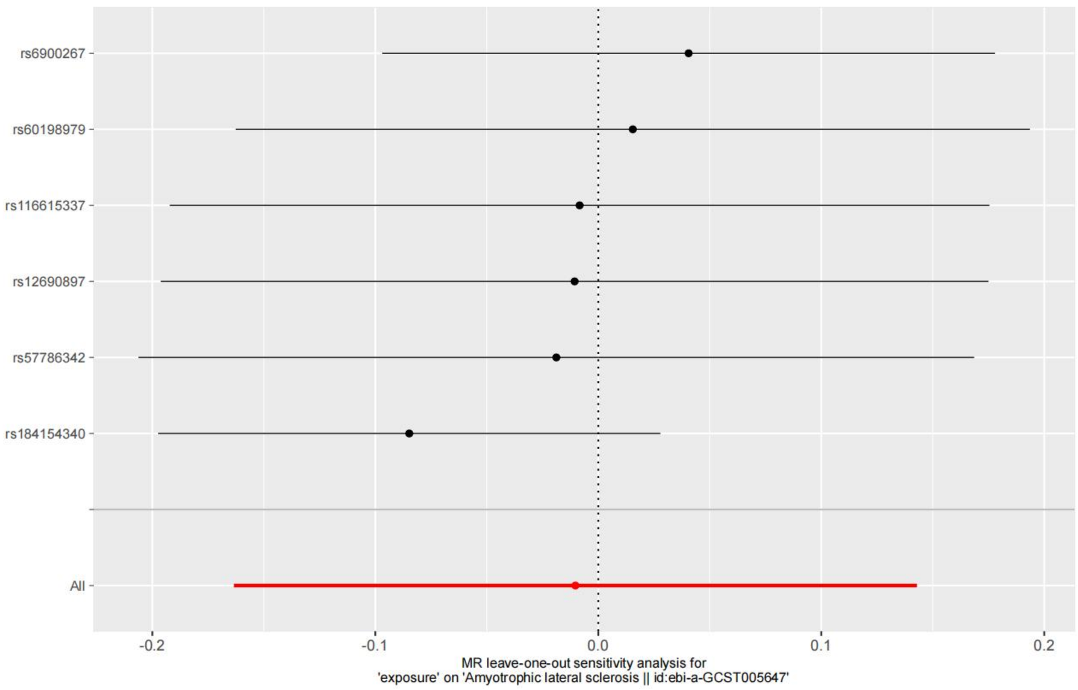

C. MR leave-one-out sensitivity analysis for MIP-1A on ALS

eFigure 114. MIP-1B-associated SNPs with risk of ALS

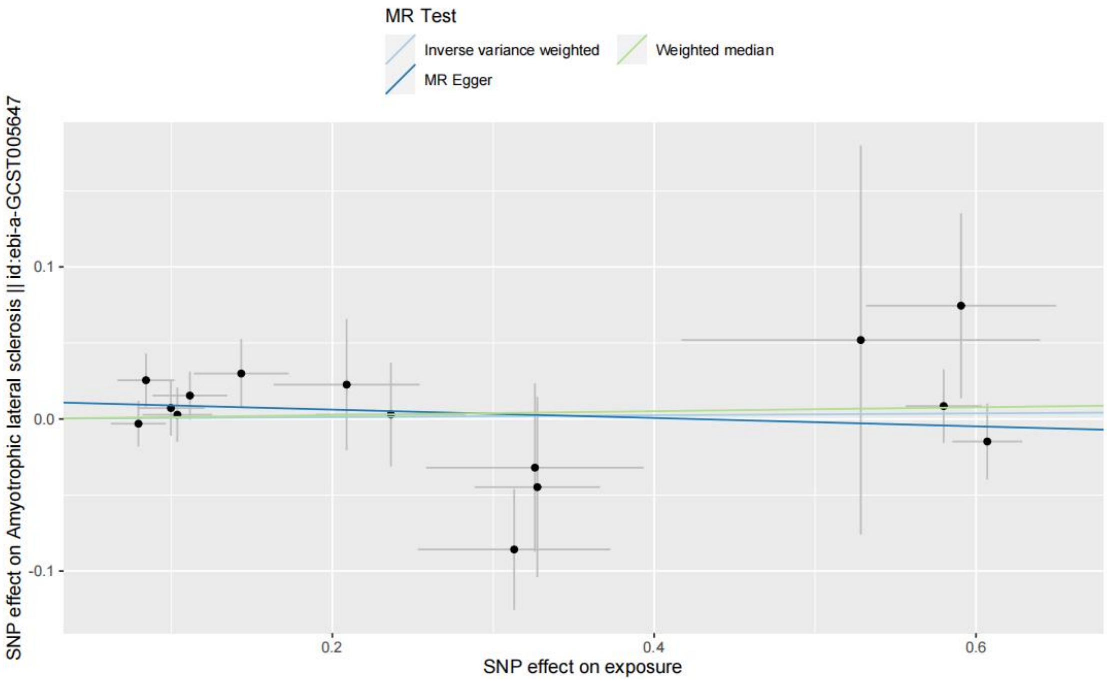

A. Scatter plot of MIP-1B with risk of ALS

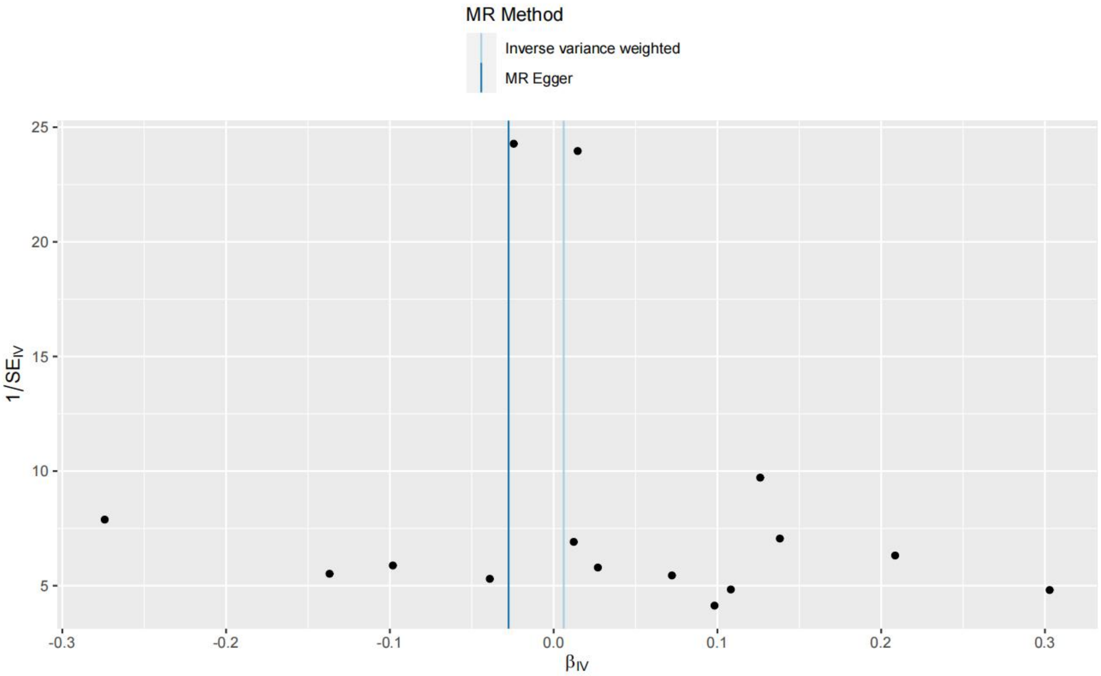

B. Funnel plot of MIP-1B instruments strength on ALS

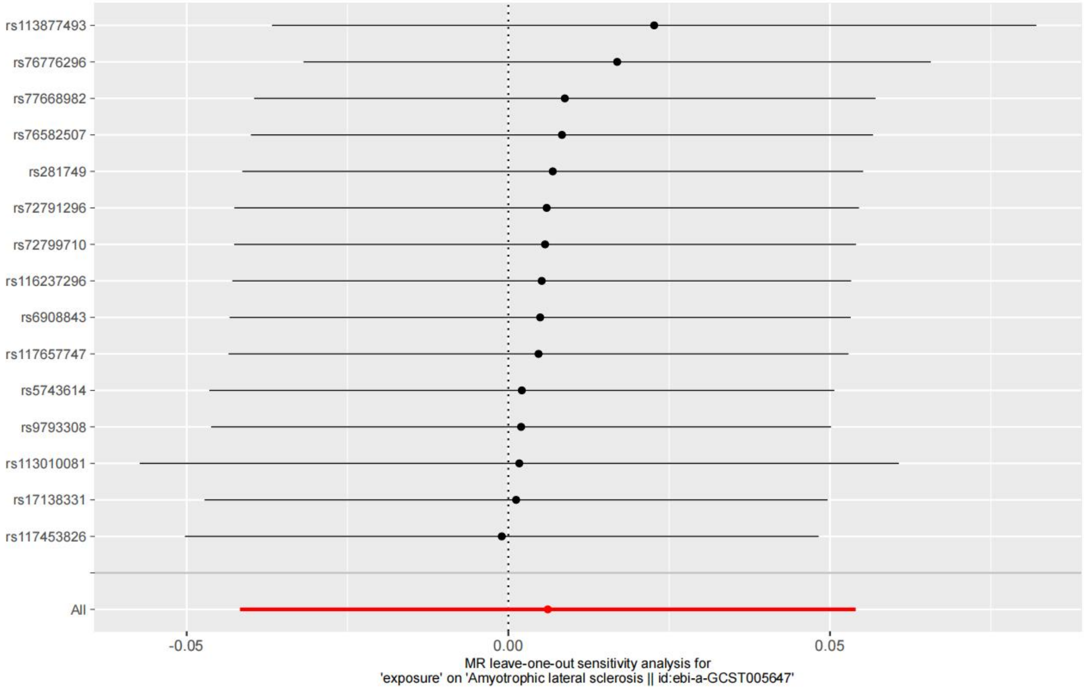

C. MR leave-one-out sensitivity analysis for MIP-1B on ALS

eFigure 115. PDGF-BB-associated SNPs with risk of ALS

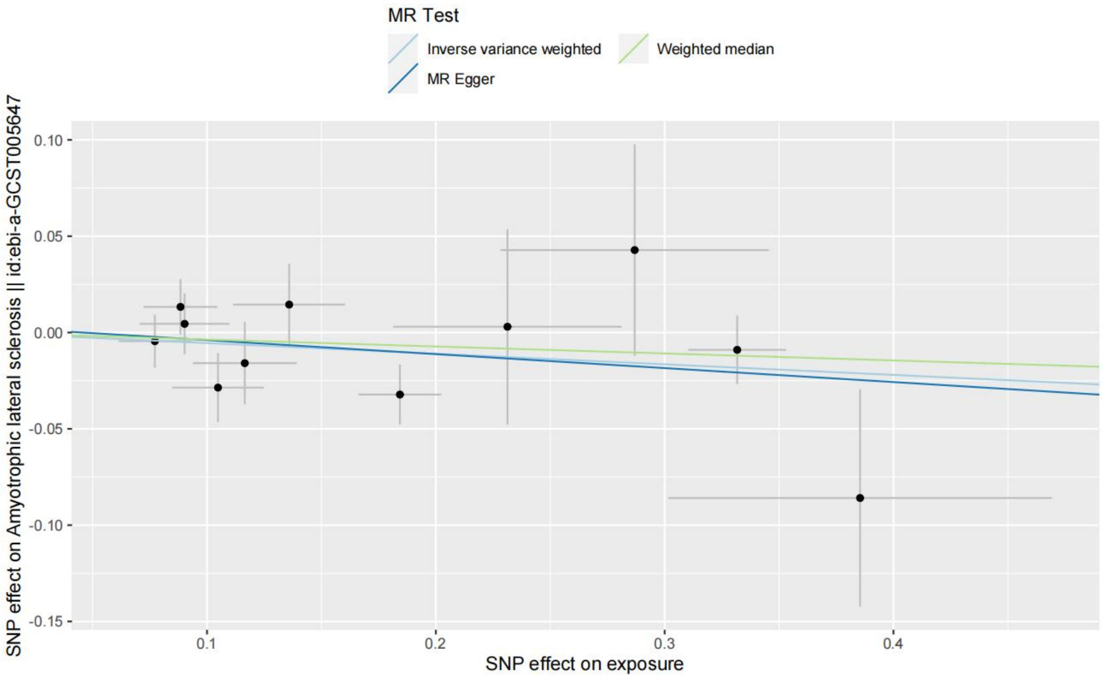

A. Scatter plot of PDGF-BB with risk of ALS

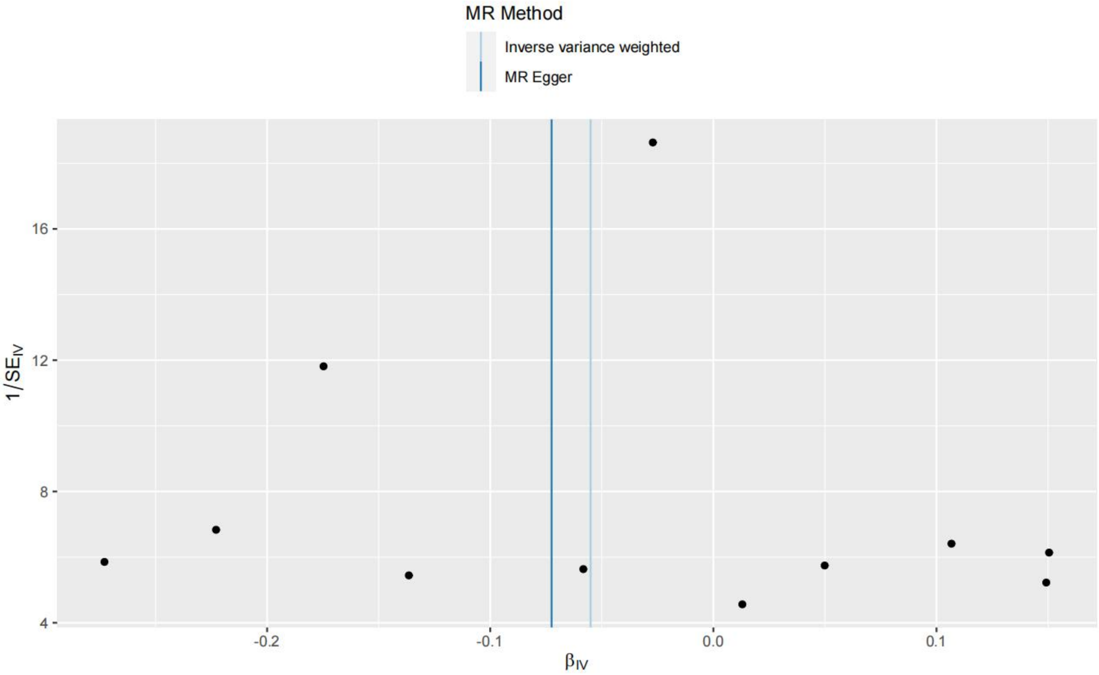

B. Funnel plot of PDGF-BB instruments strength on ALS

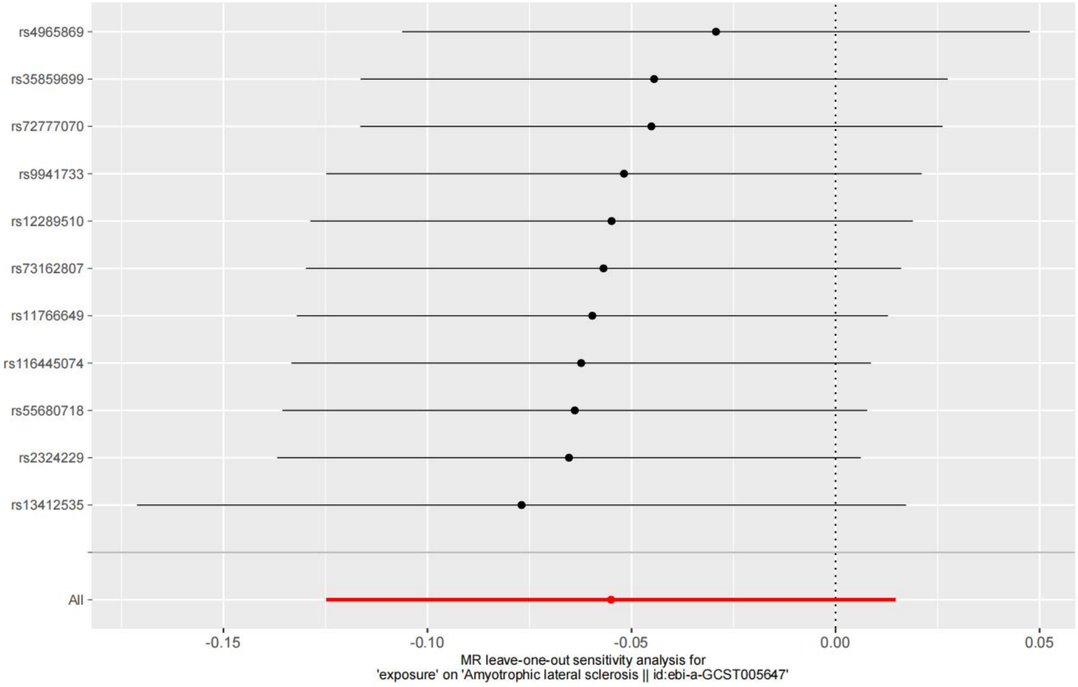

C. MR leave-one-out sensitivity analysis for PDGF-BB on ALS

eFigure 116. RANTES-associated SNPs with risk of ALS

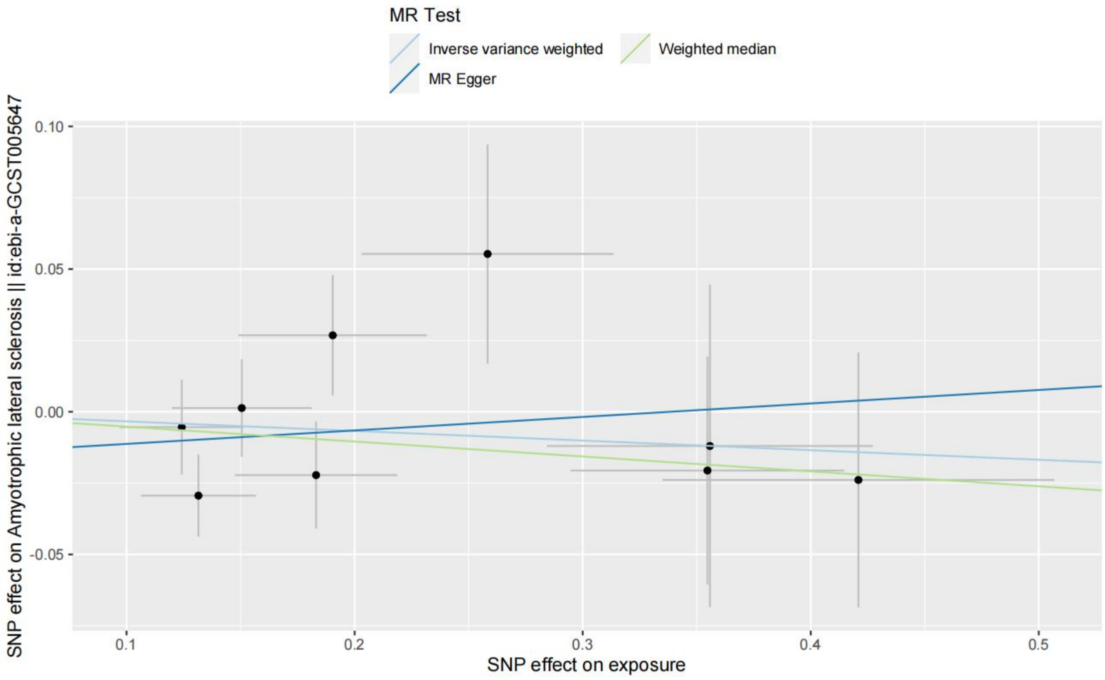

A. Scatter plot of RANTES with risk of ALS

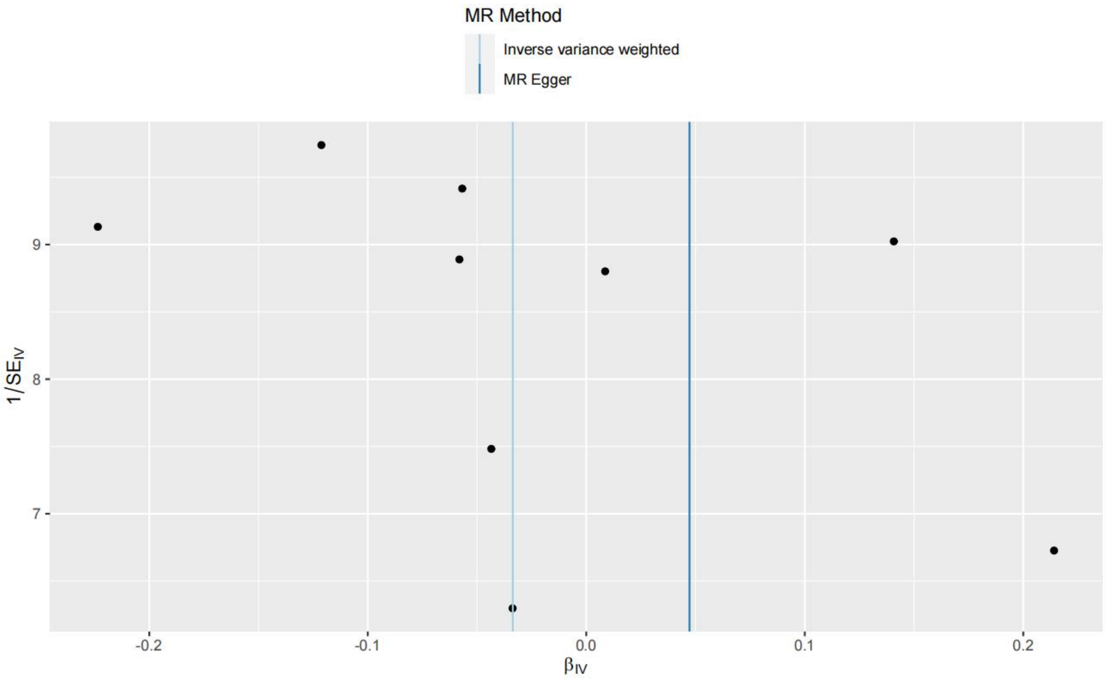

B. Funnel plot of RANTES instruments strength on ALS

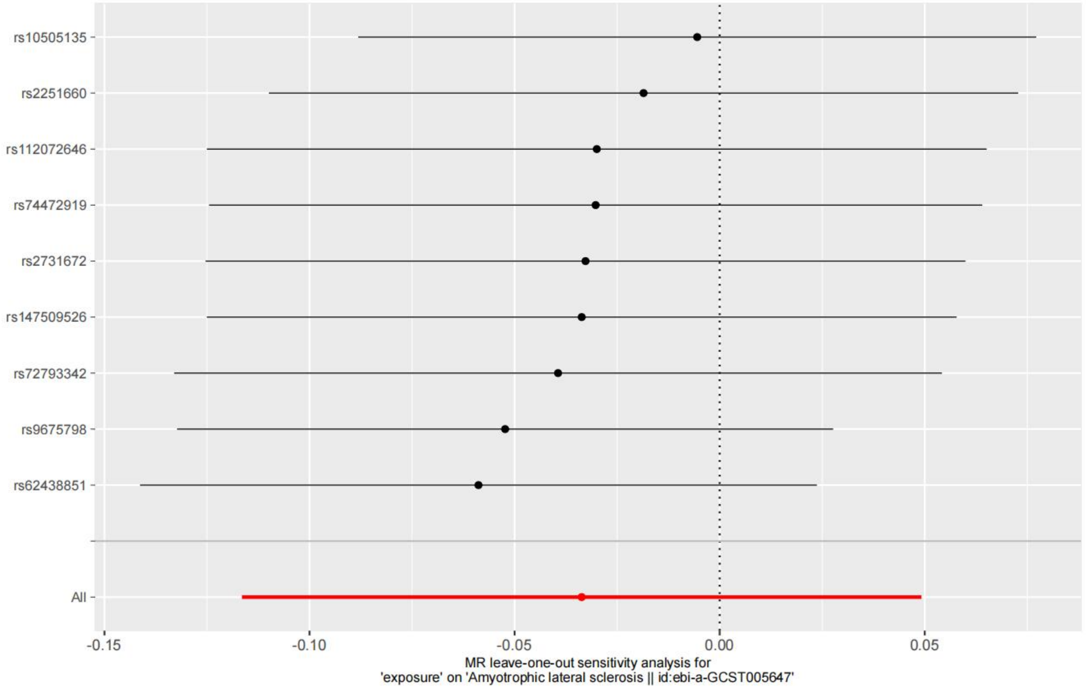

C. MR leave-one-out sensitivity analysis for RANTES on ALS

eFigure 117. SCF-associated SNPs with risk of ALS

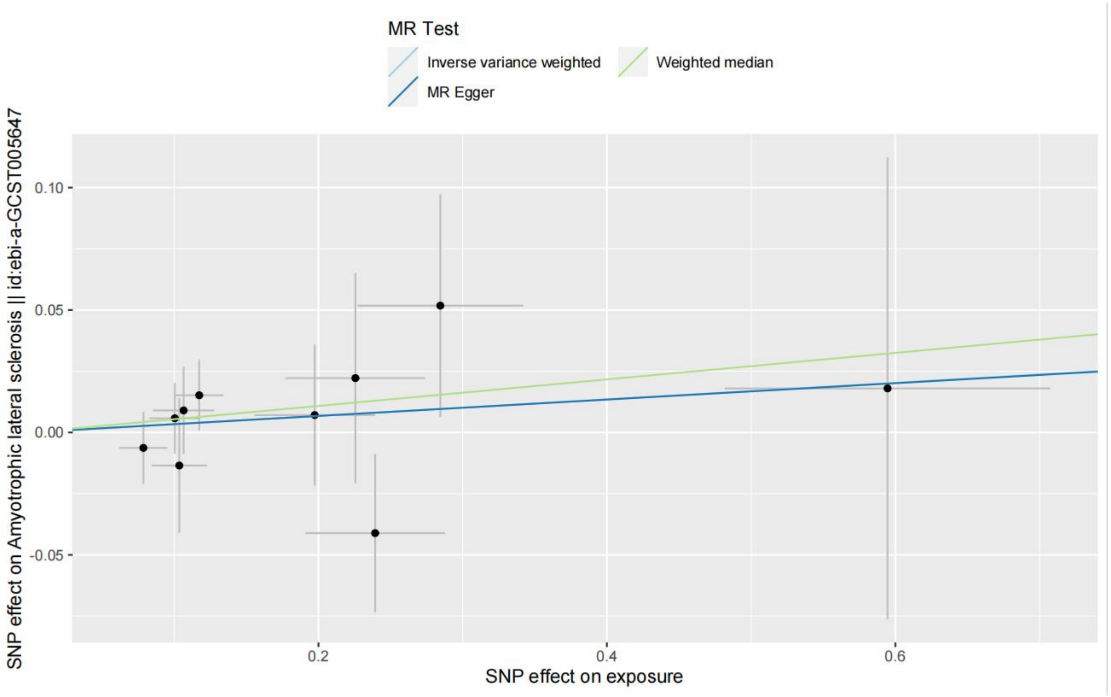

A. Scatter plot of SCF with risk of ALS

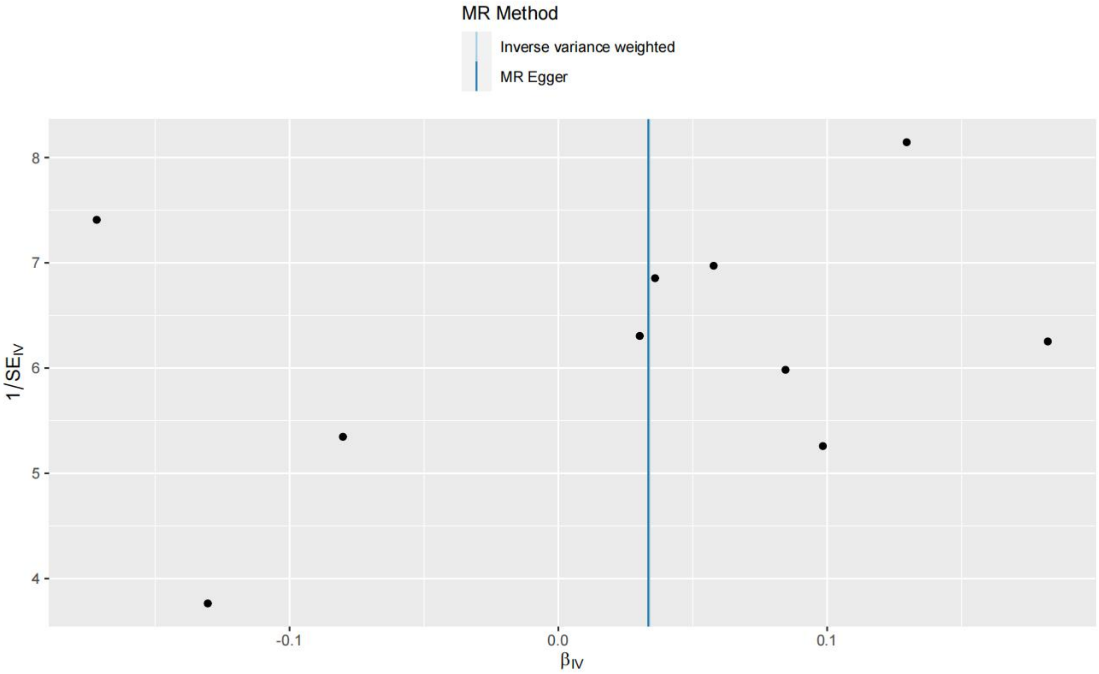

B. Funnel plot of SCF instruments strength on ALS

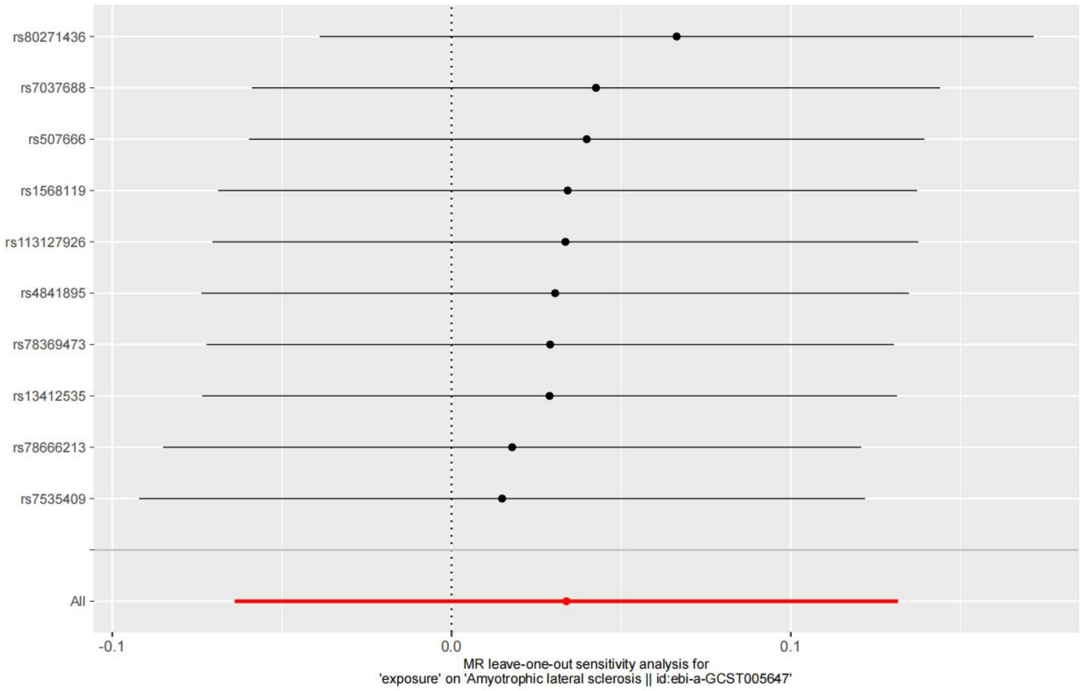

C. MR leave-one-out sensitivity analysis for SCF on ALS

eFigure 118. SCGFβ-associated SNPs with risk of ALS

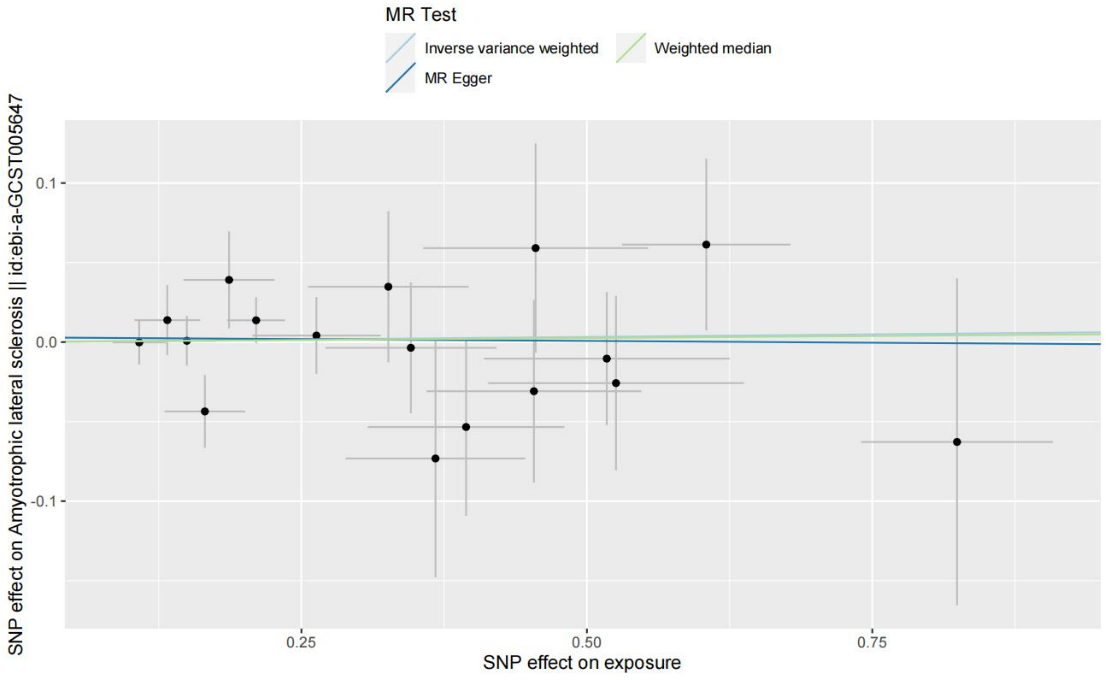

A. Scatter plot of SCGFβ with risk of ALS

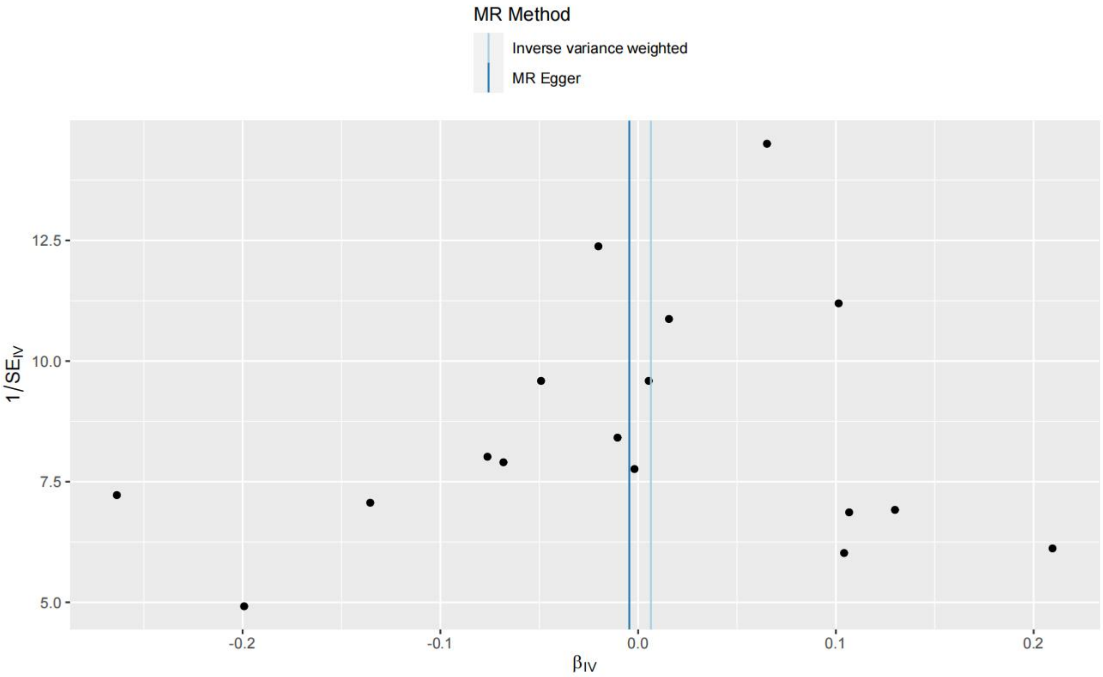

B. Funnel plot of SCGFβ instruments strength on ALS

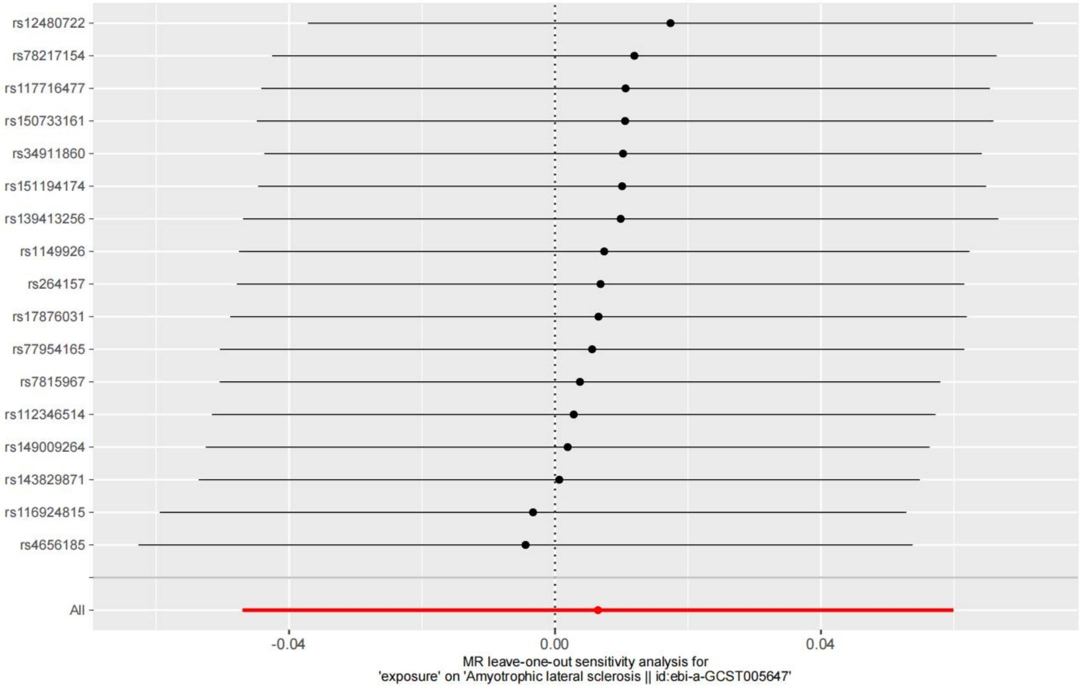

C. MR leave-one-out sensitivity analysis for SCGFβ on ALS

eFigure 119. SDF-1A-associated SNPs with risk of ALS

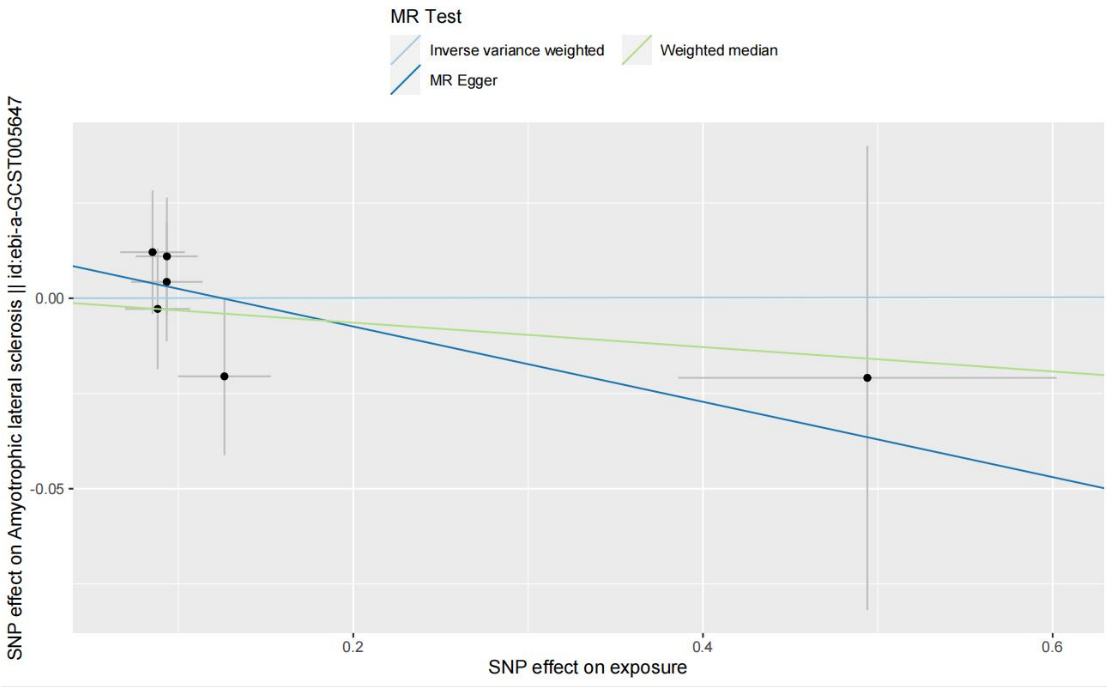

A. Scatter plot of SDF-1A with risk of ALS

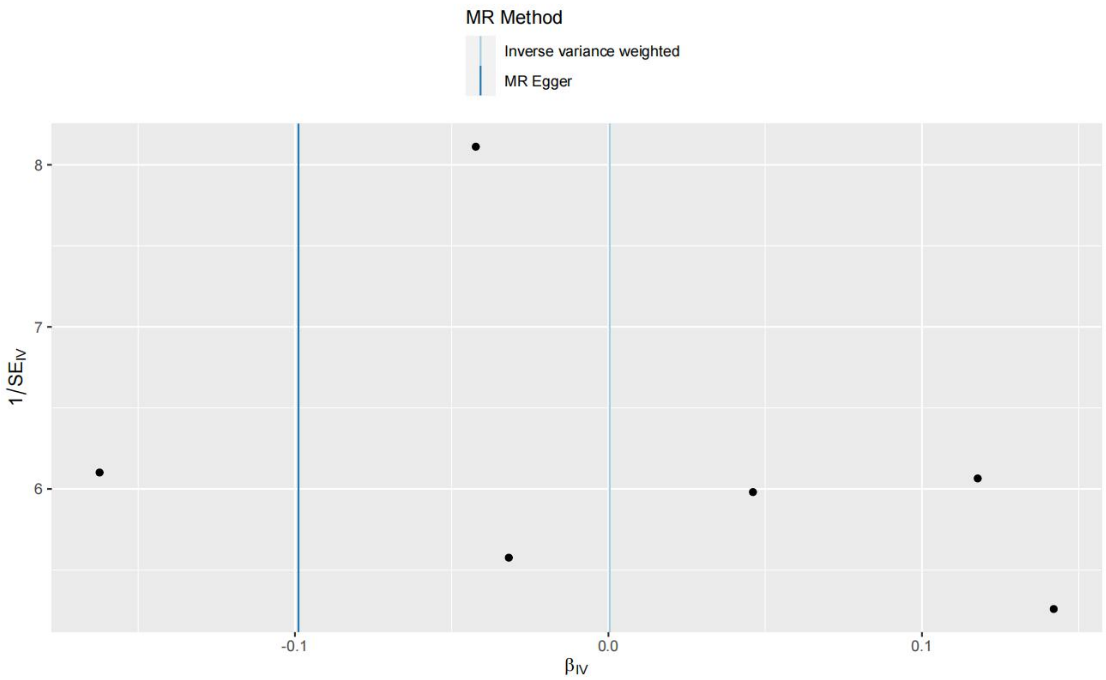

B. Funnel plot of SDF-1A instruments strength on ALS

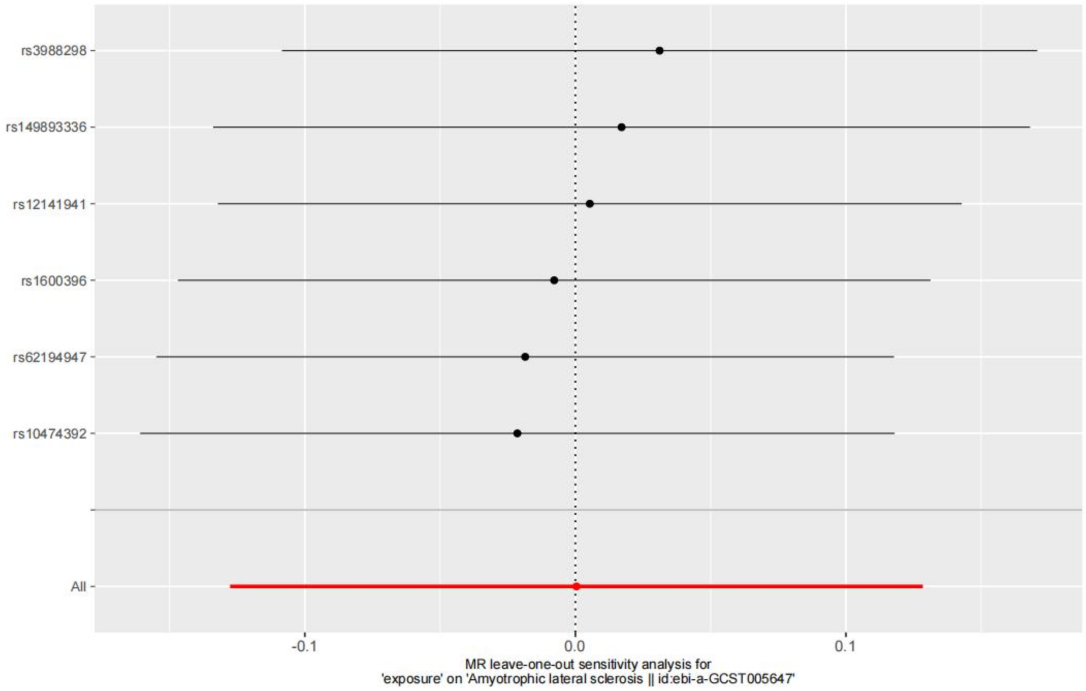

C. MR leave-one-out sensitivity analysis for SDF-1A on ALS

eFigure 120. TNF-A-associated SNPs with risk of ALS

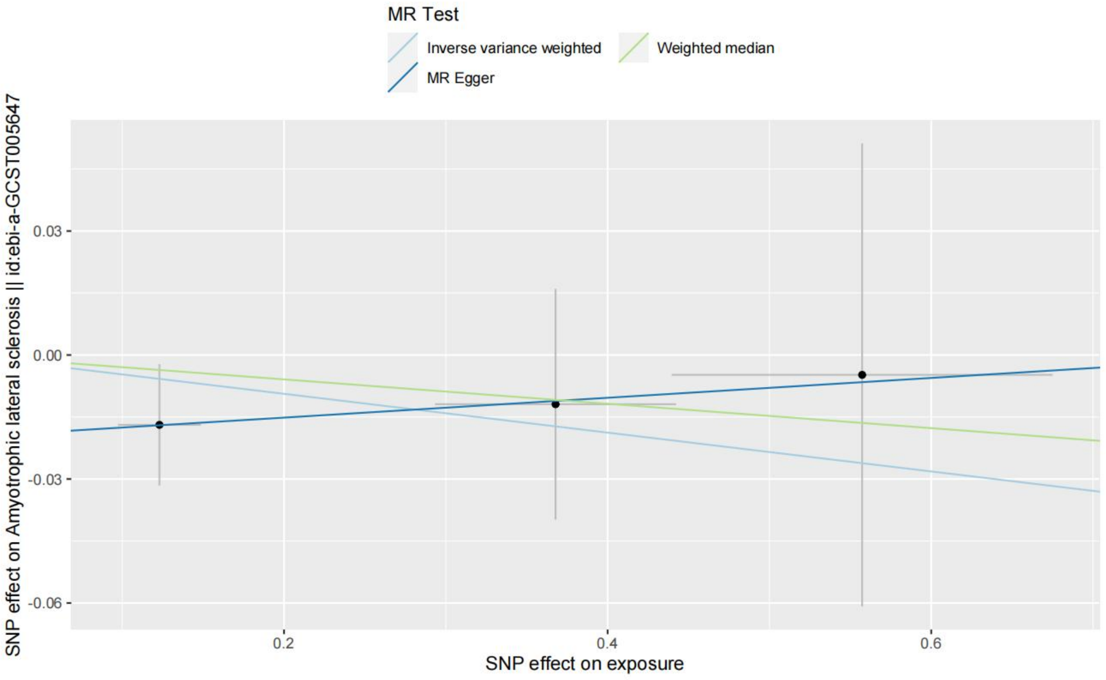

A. Scatter plot of TNF-A with risk of ALS

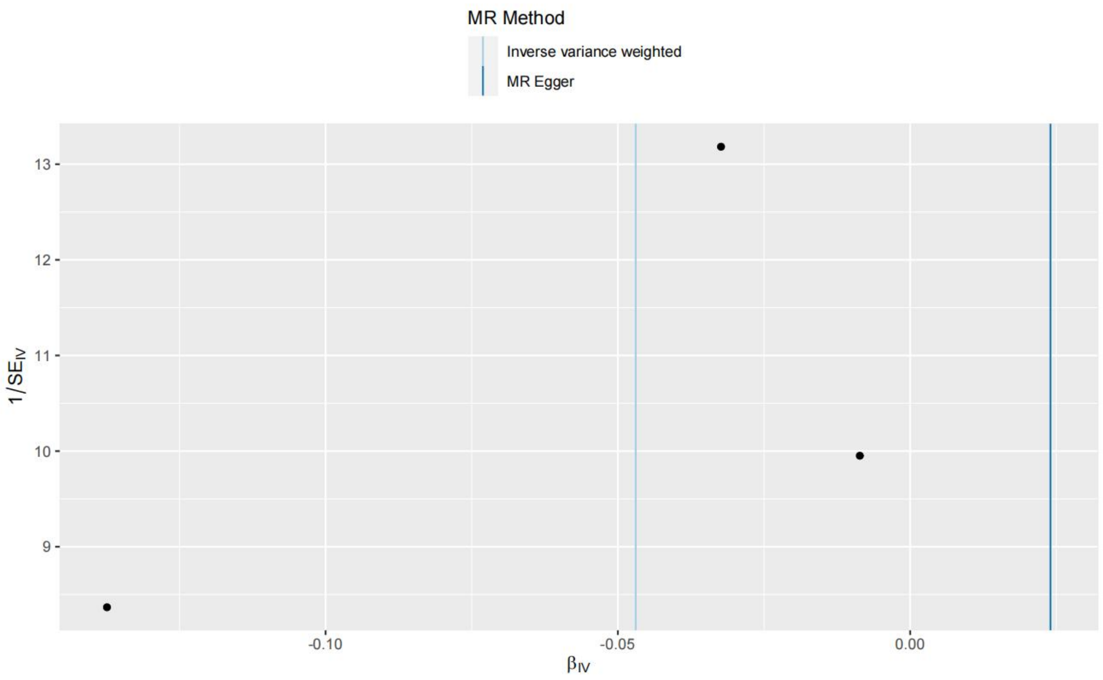

B. Funnel plot of TNF-A instruments strength on ALS

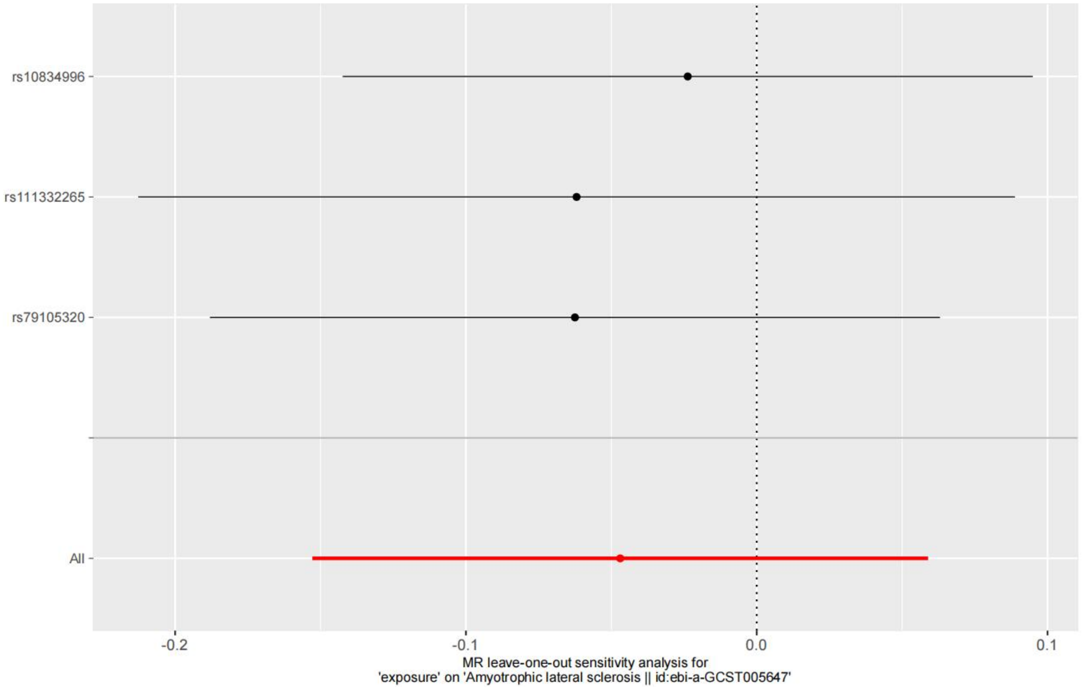

C. MR leave-one-out sensitivity analysis for TNF-A on ALS

eFigure 121. TNF-B-associated SNPs with risk of ALS

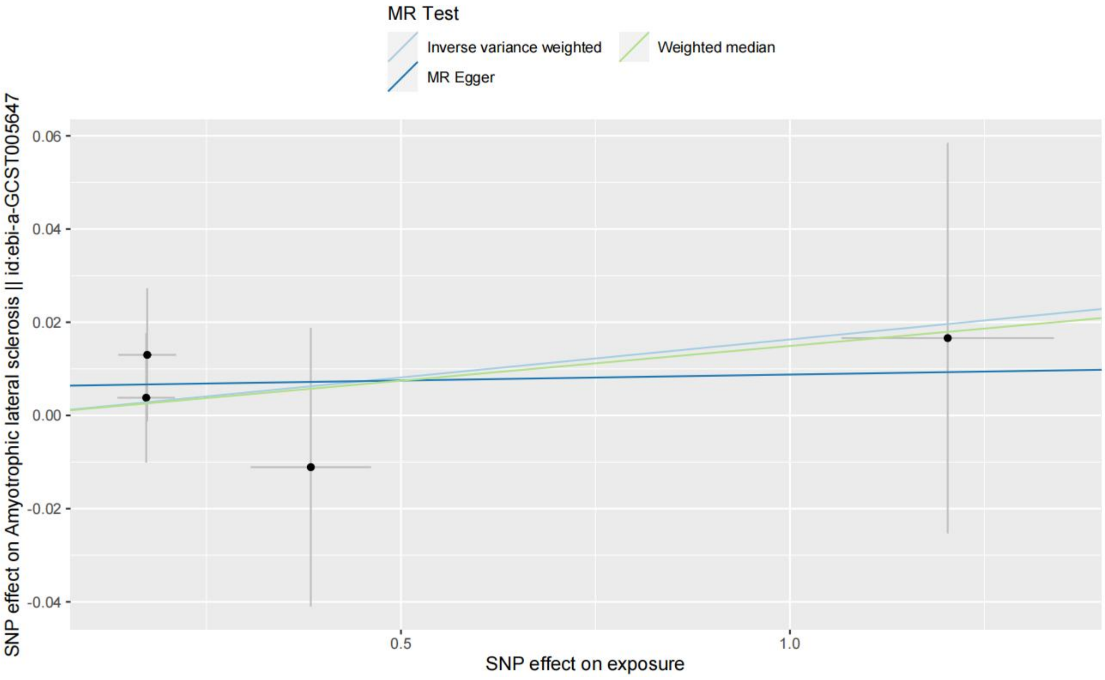

A. Scatter plot of TNF-B with risk of ALS

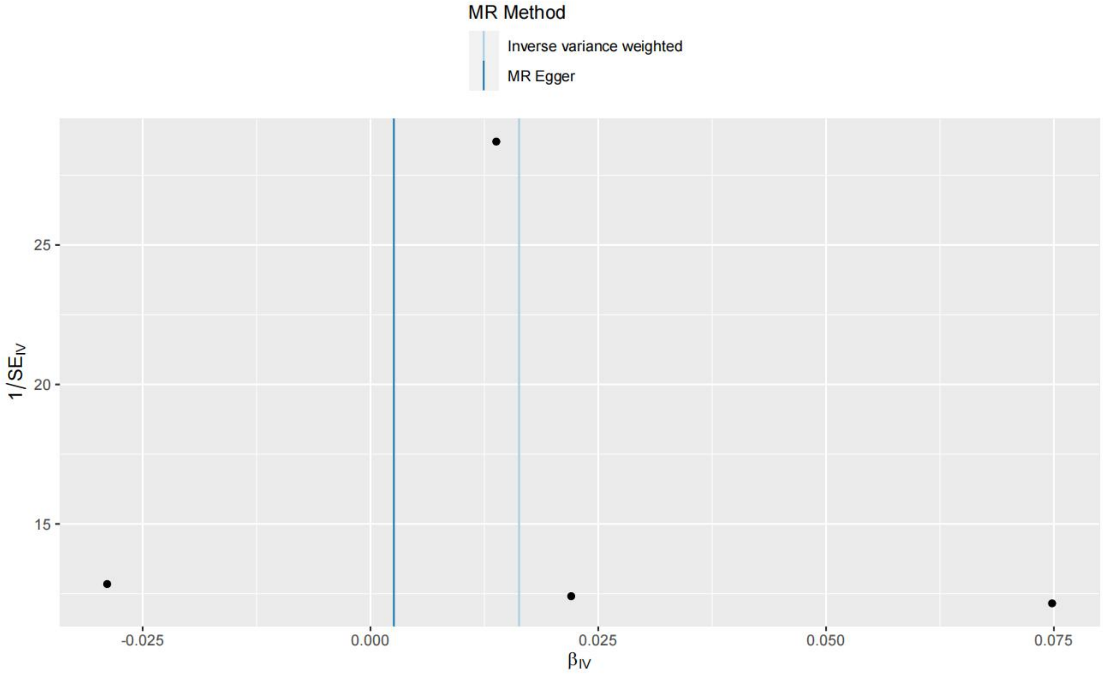

B. Funnel plot of TNF-B instruments strength on ALS

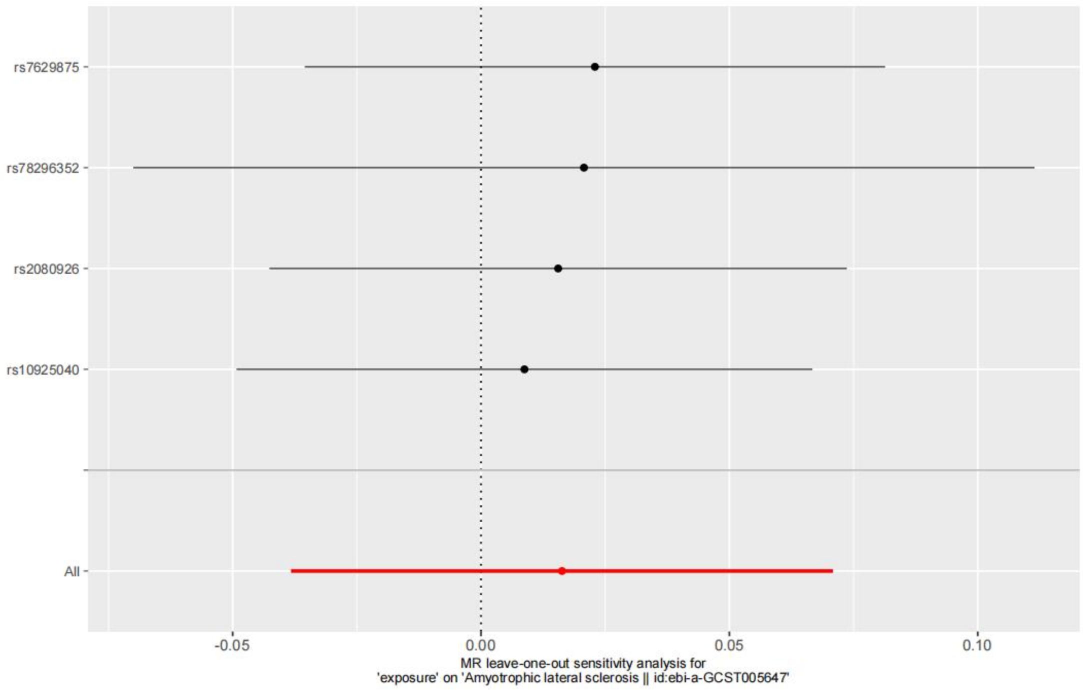

C. MR leave-one-out sensitivity analysis for TNF-B on ALS

eFigure 122. TRAIL-associated SNPs with risk of ALS

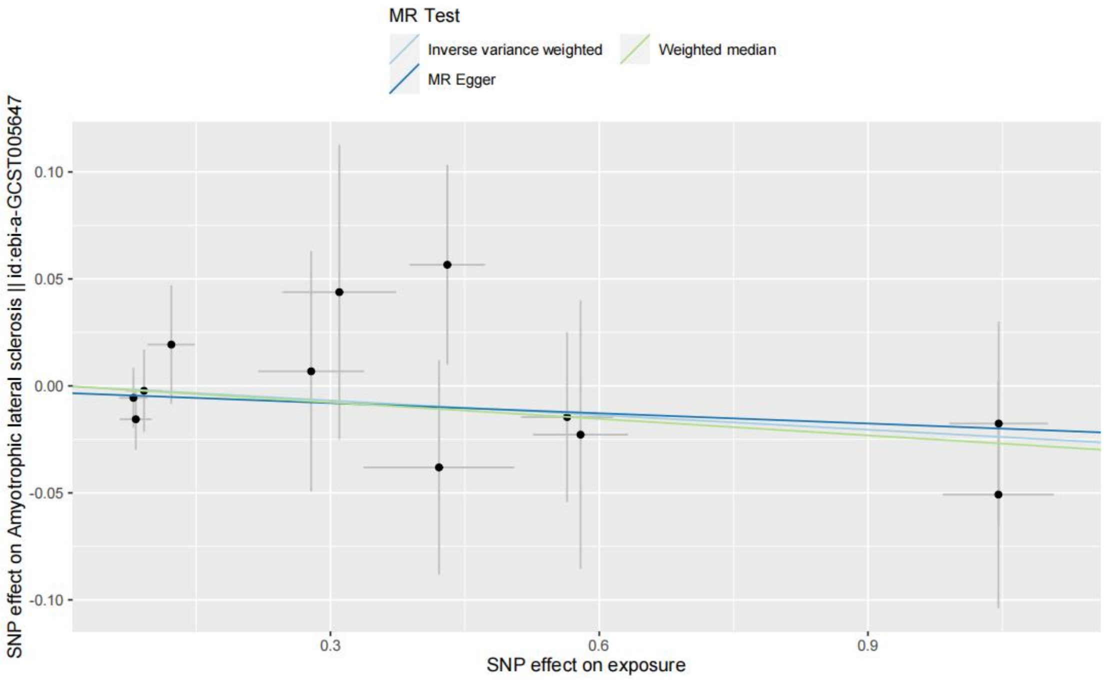

A. Scatter plot of TRAIL with risk of ALS

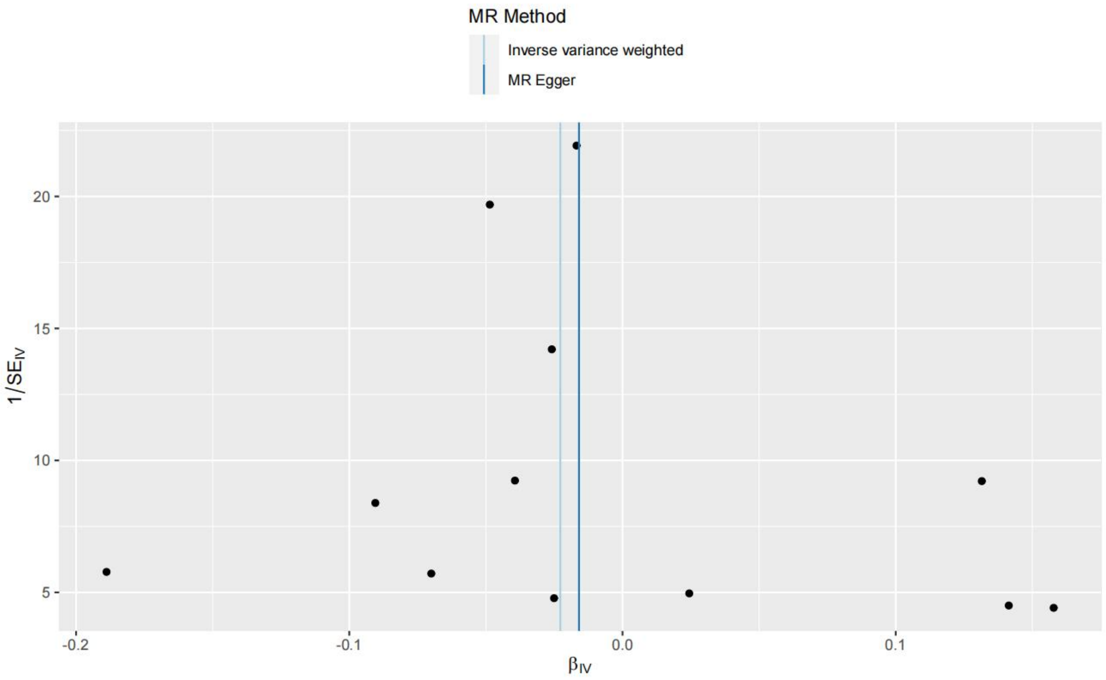

B. Funnel plot of TRAIL instruments strength on ALS

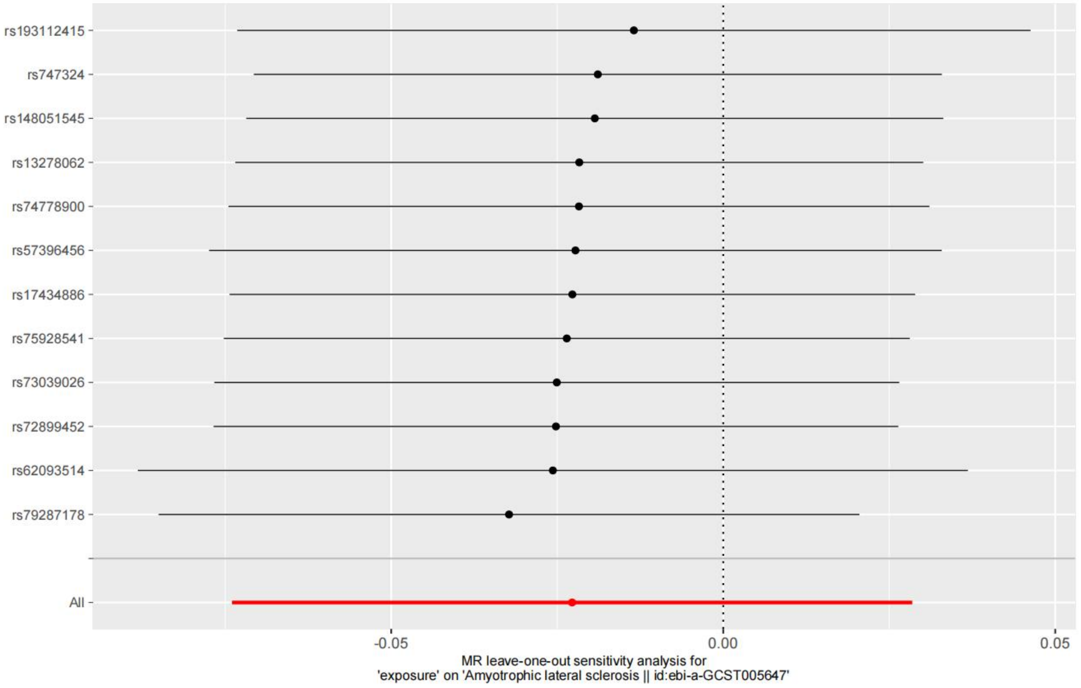

C. MR leave-one-out sensitivity analysis for TRAIL on ALS

eFigure 123. VEGF-associated SNPs with risk of ALS

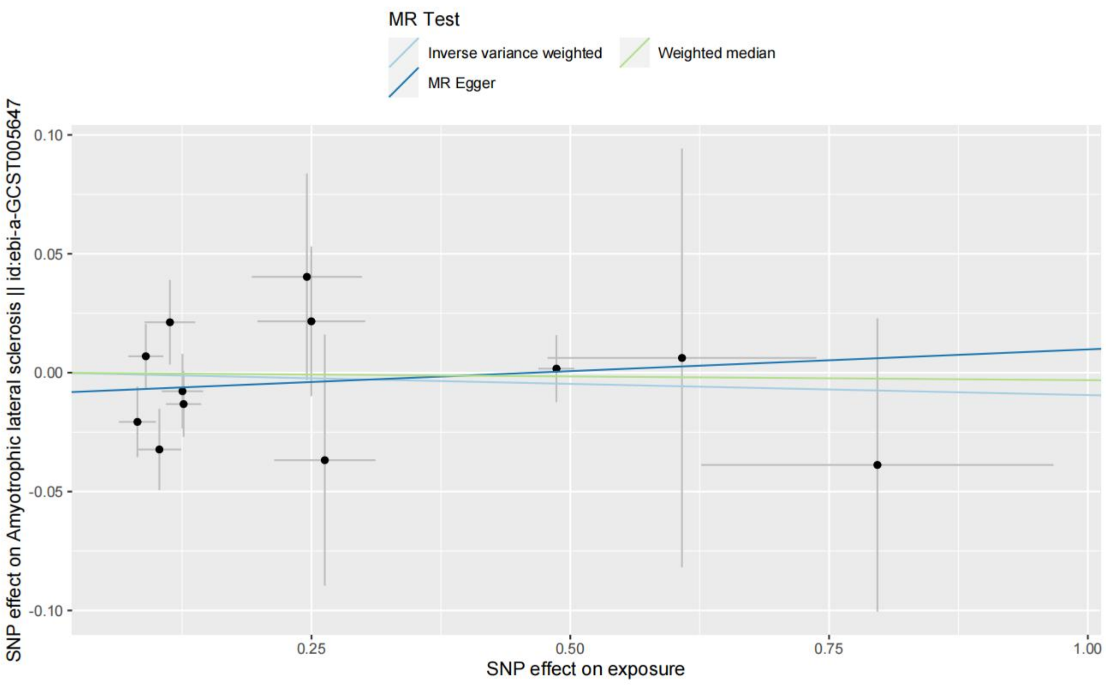

A. Scatter plot of VEGF with risk of ALS

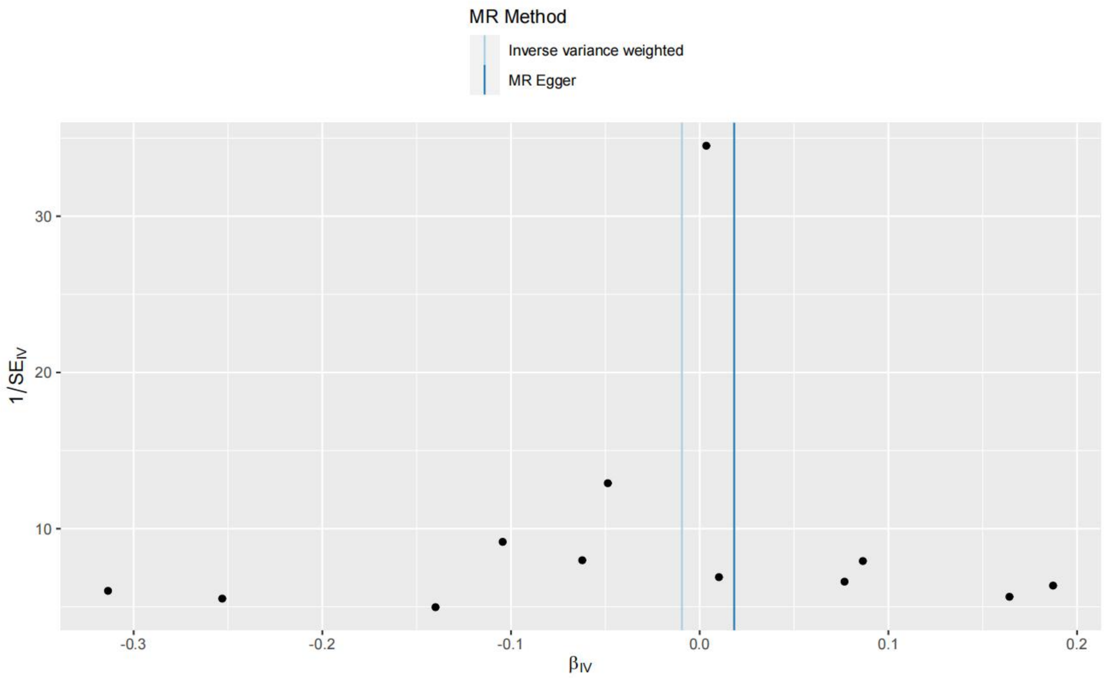

B. Funnel plot of VEGF instruments strength on ALS

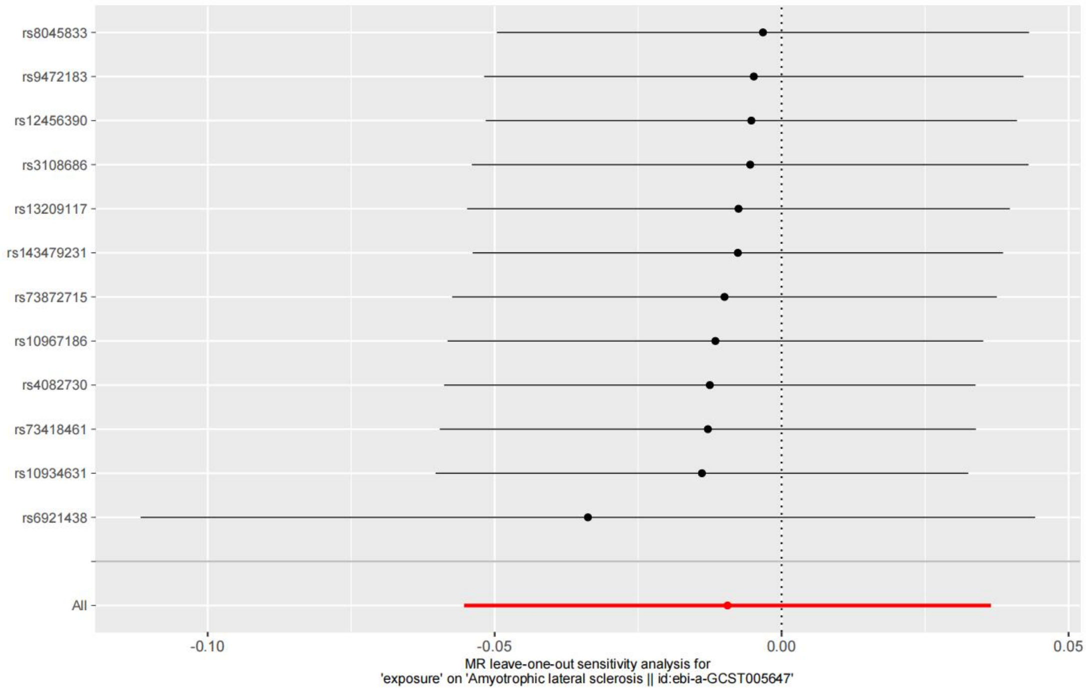

C. MR leave-one-out sensitivity analysis for VEGF on ALS

eFigure 124. AD-associated SNPs with BNGF

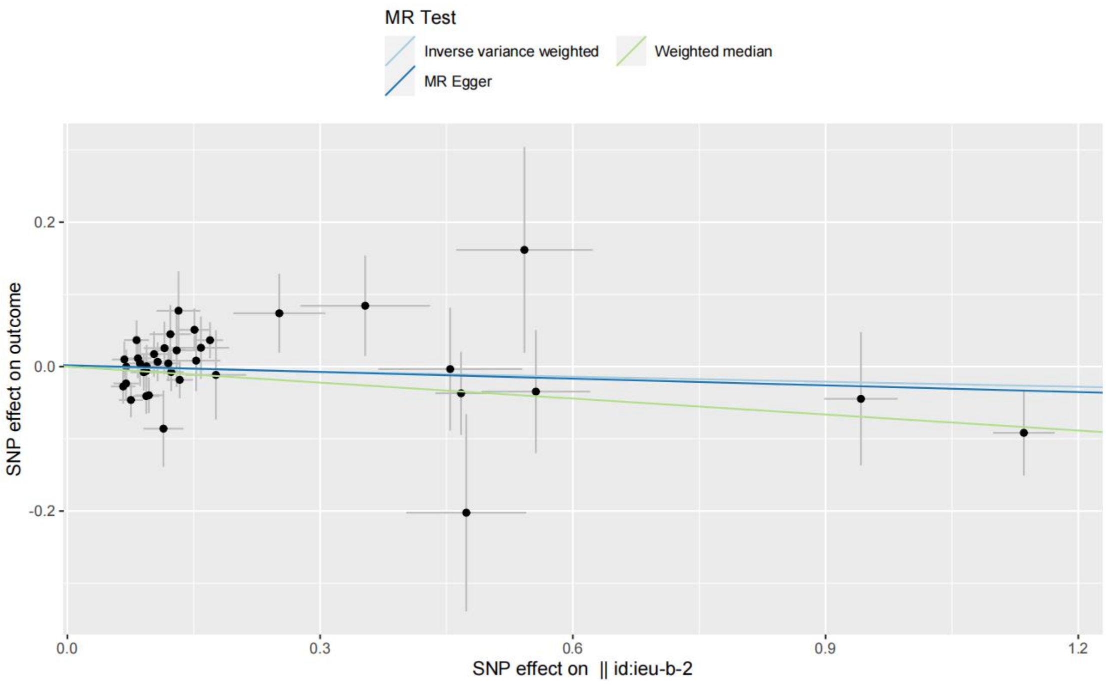

A. Scatter plot of AD on BNGF

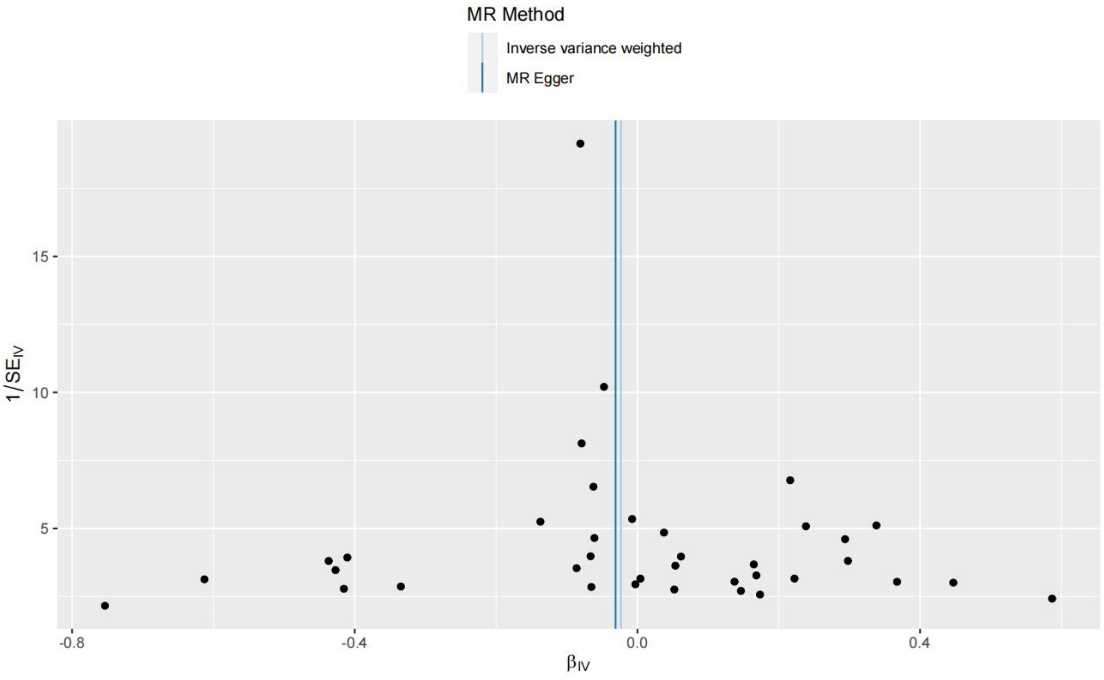

B. Funnel plot of AD on BNGF

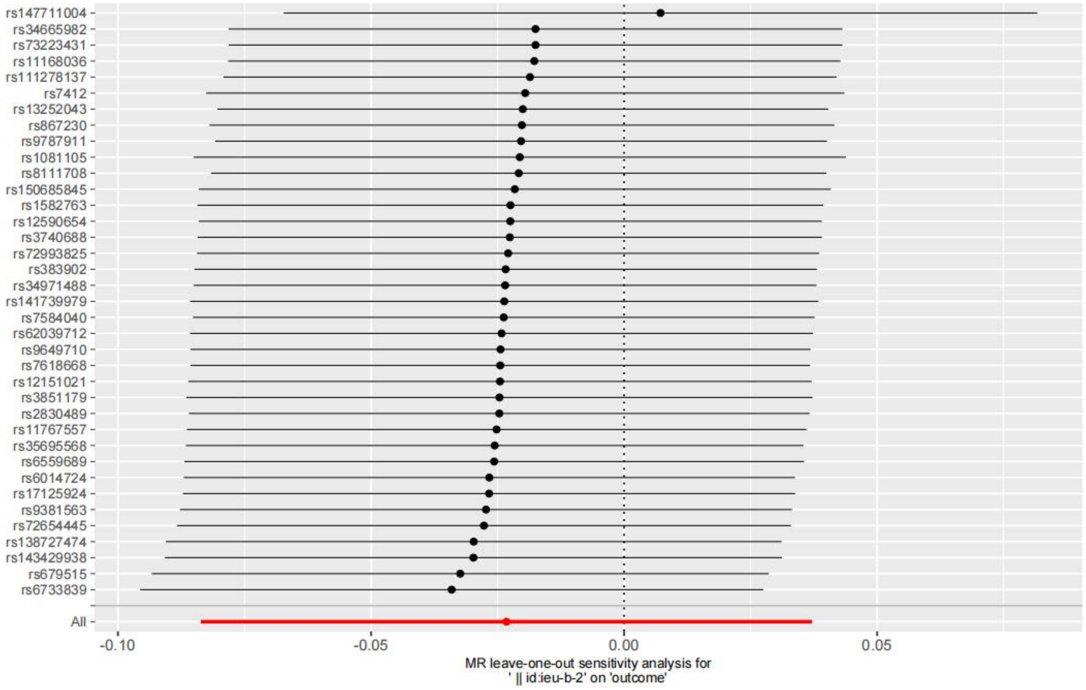

C. MR leave-one-out sensitivity analysis for AD on BNGF

eFigure 125. AD-associated SNPs with CTACK

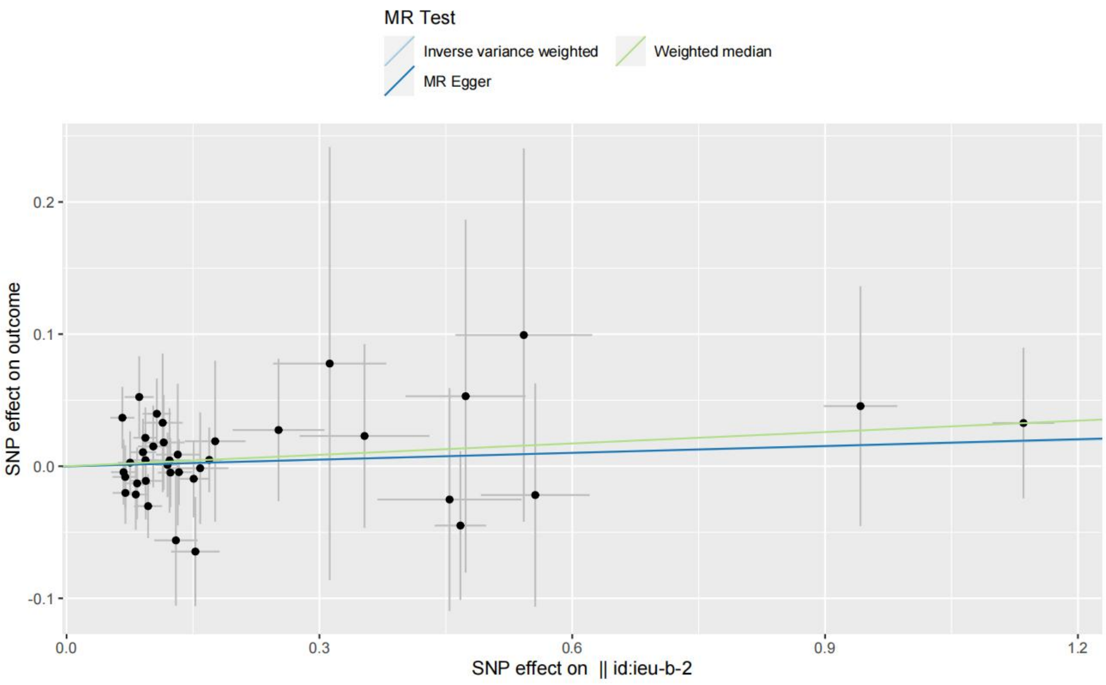

A. Scatter plot of AD on CTACK

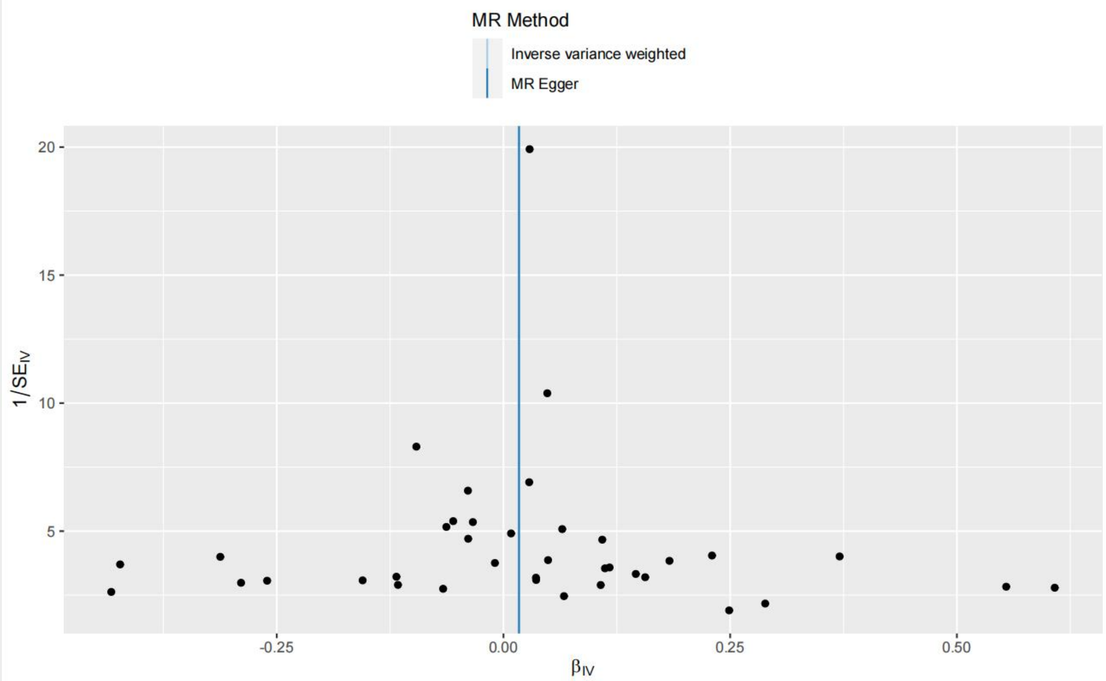

B. Funnel plot of AD on CTACK

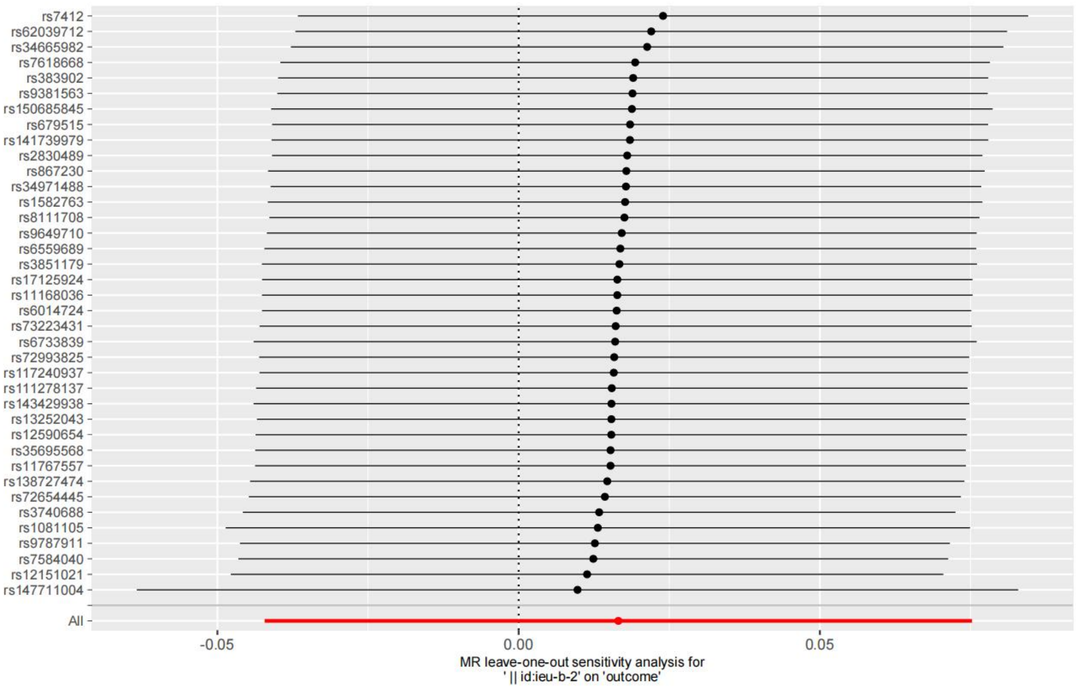

C. MR leave-one-out sensitivity analysis for AD on CTACK

eFigure 126. AD-associated SNPs with EOTAXIN

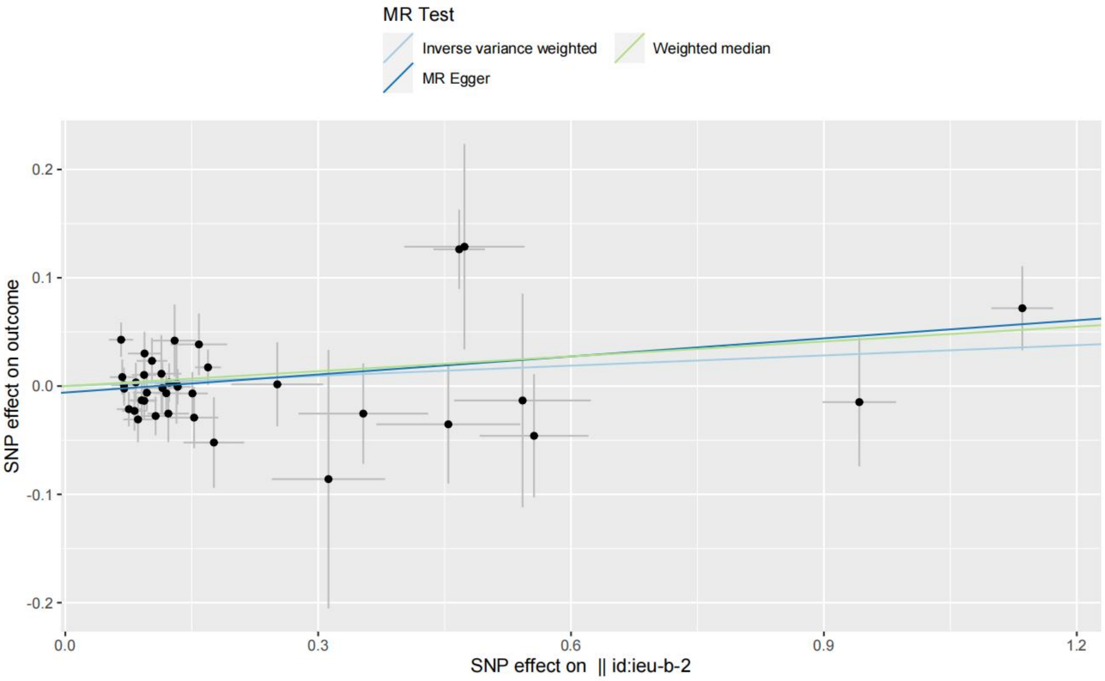

A. Scatter plot of AD on EOTAXIN

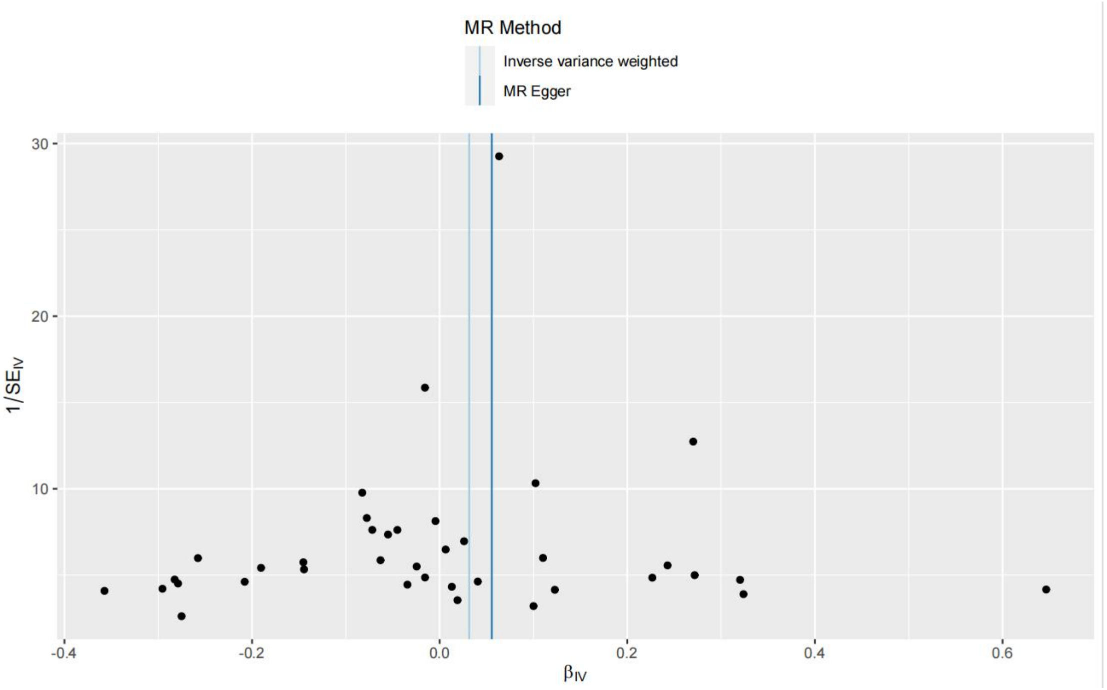

B. Funnel plot of AD on EOTAXIN

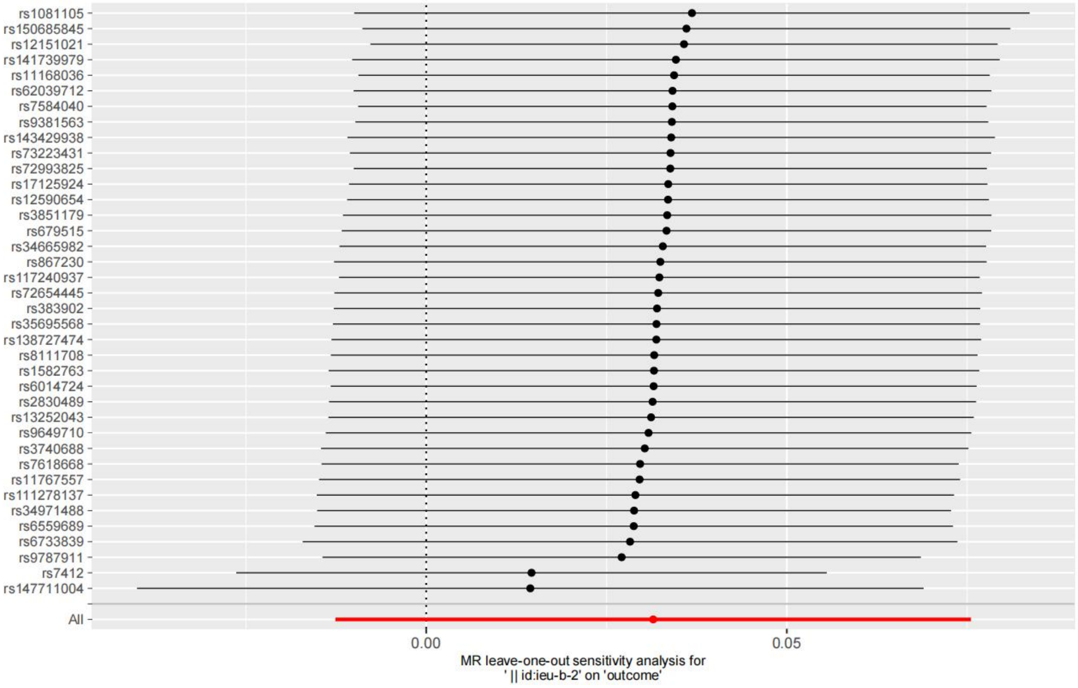

C. MR leave-one-out sensitivity analysis for AD on EOTAXIN

eFigure 127. AD-associated SNPs with bFGF

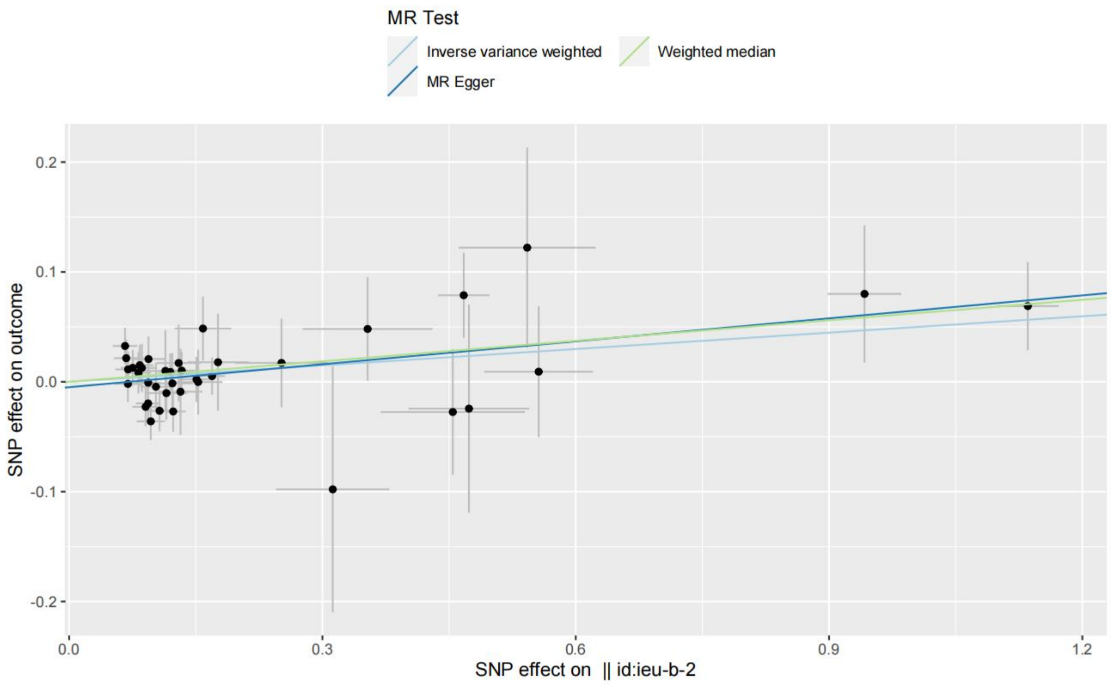

A. Scatter plot of AD on bFGF

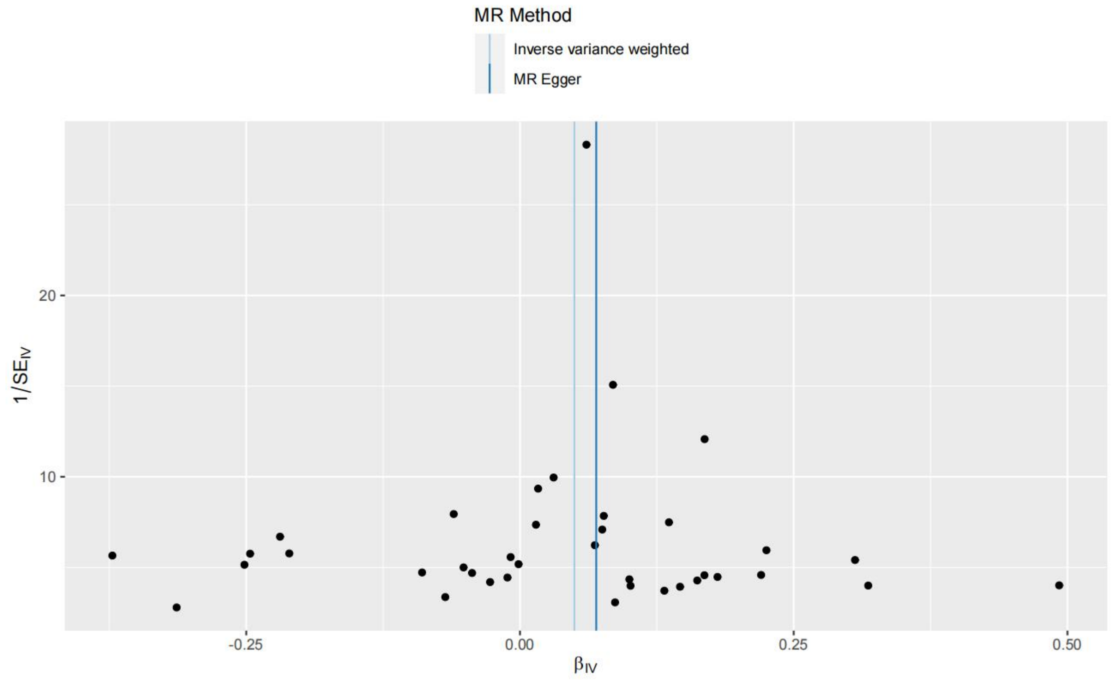

B. Funnel plot of AD on bFGF

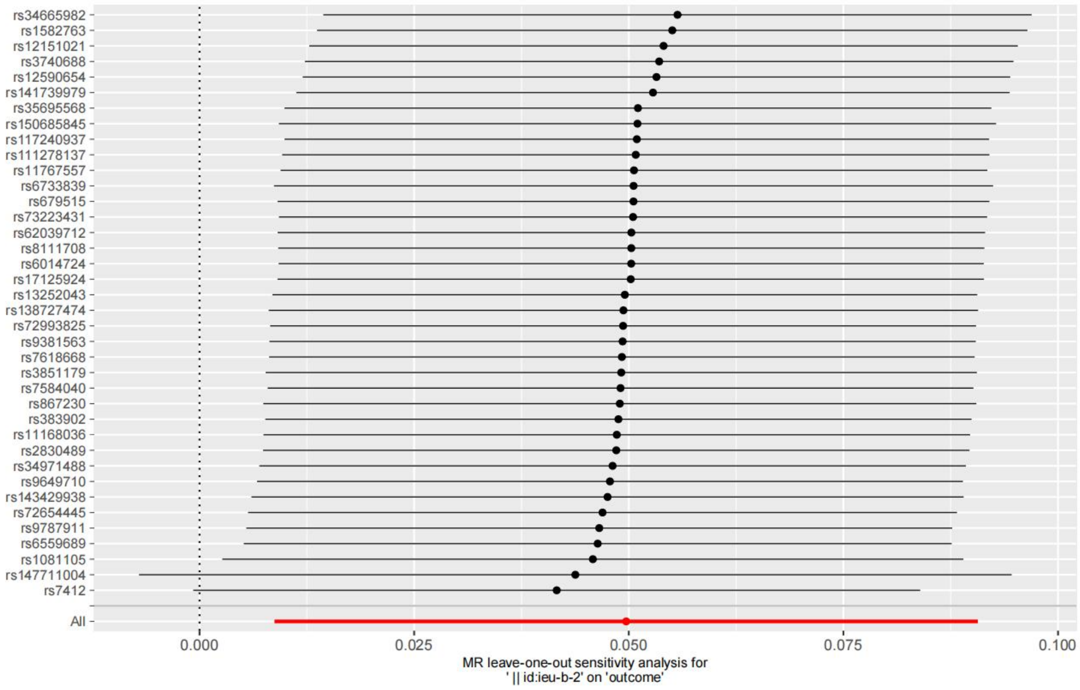

C. MR leave-one-out sensitivity analysis for AD on bFGF

eFigure 128. AD-associated SNPs with G-CSF

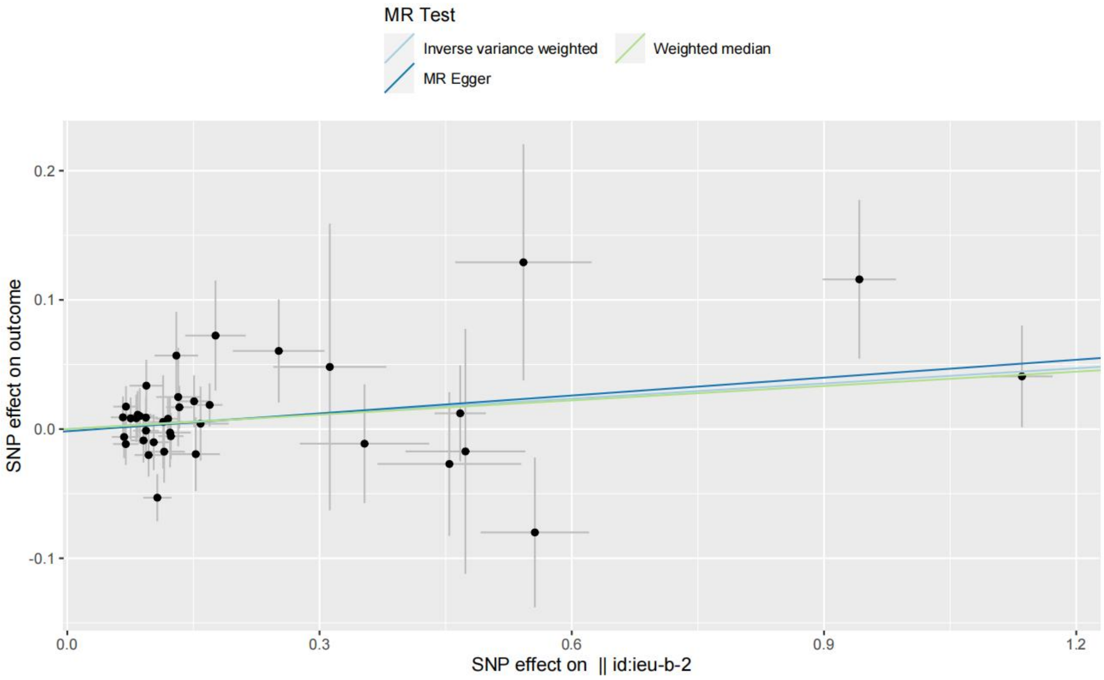

A. Scatter plot of AD on G-CSF

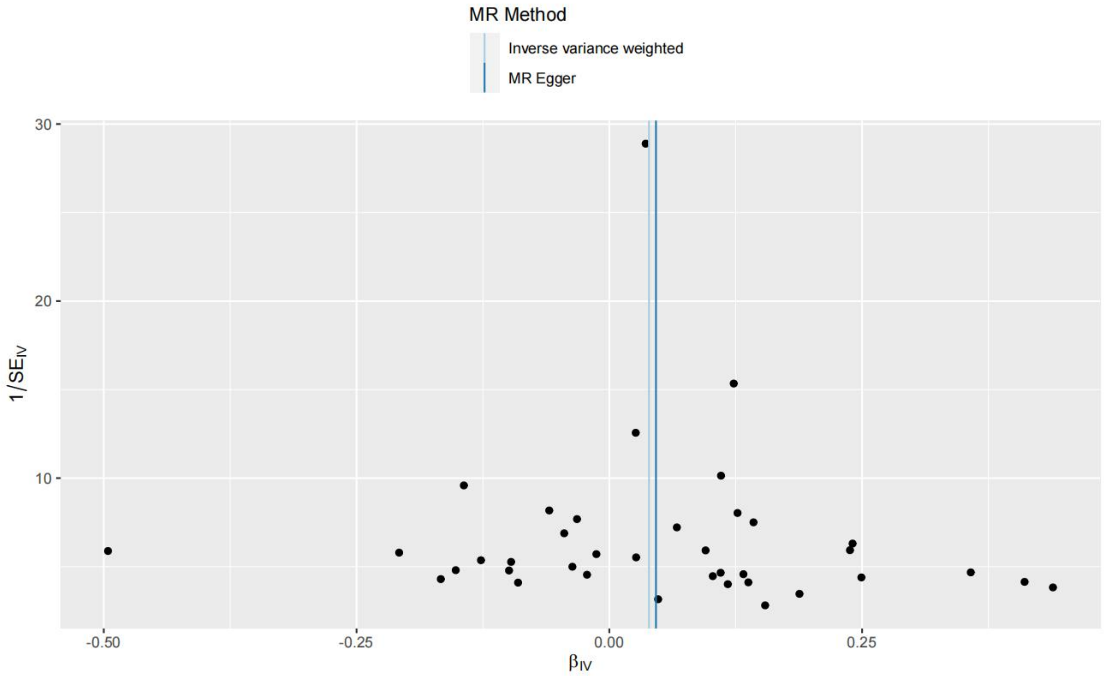

B. Funnel plot of AD on G-CSF

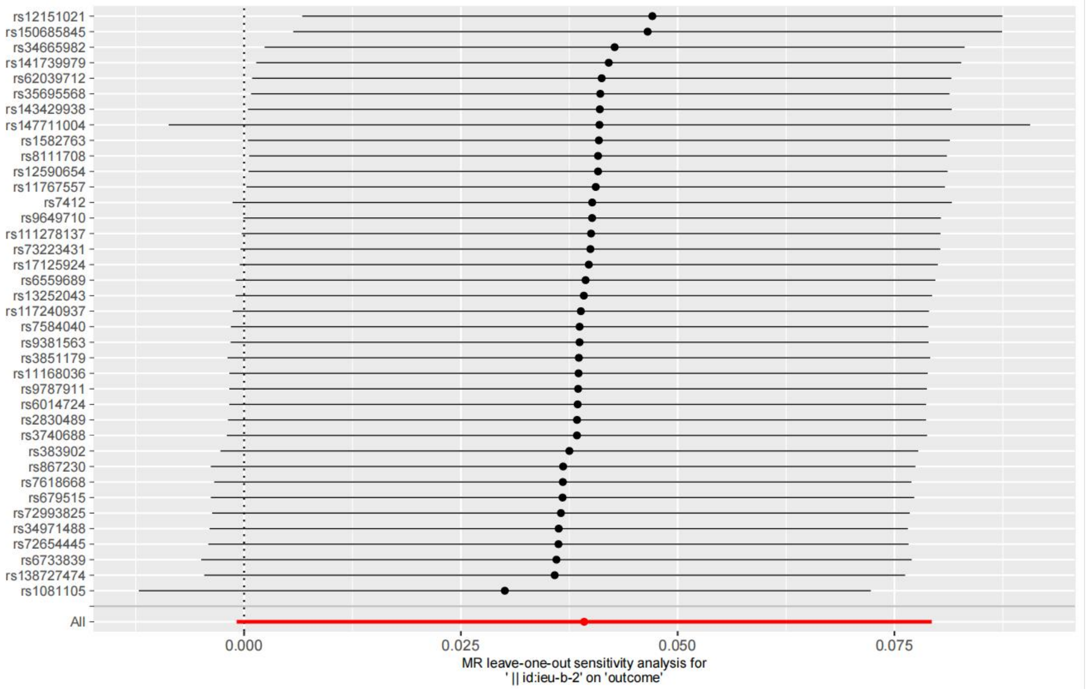

C. MR leave-one-out sensitivity analysis for AD on G-CSF

eFigure 129. AD-associated SNPs with GROA

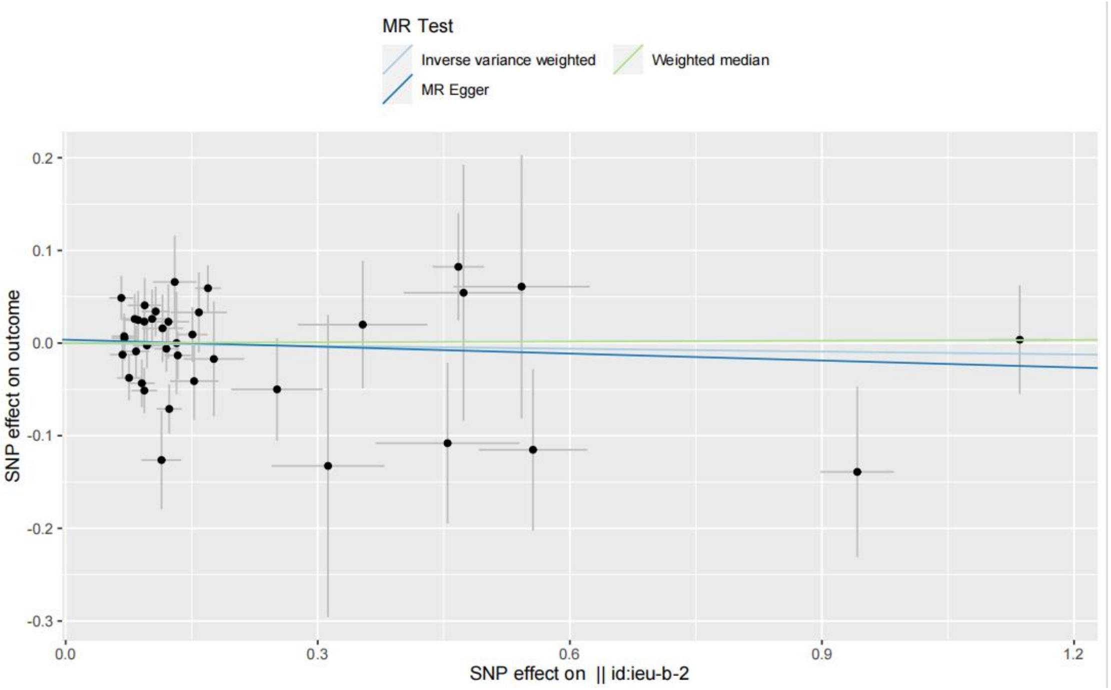

A. Scatter plot of AD on GROA

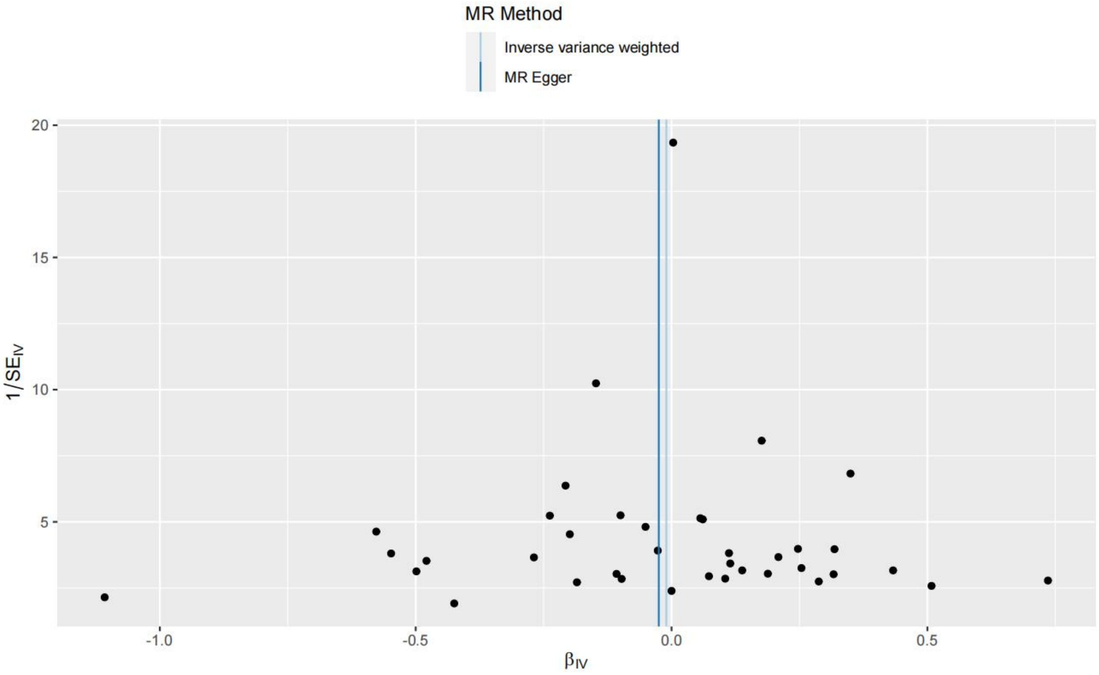

B. Funnel plot of AD on GROA

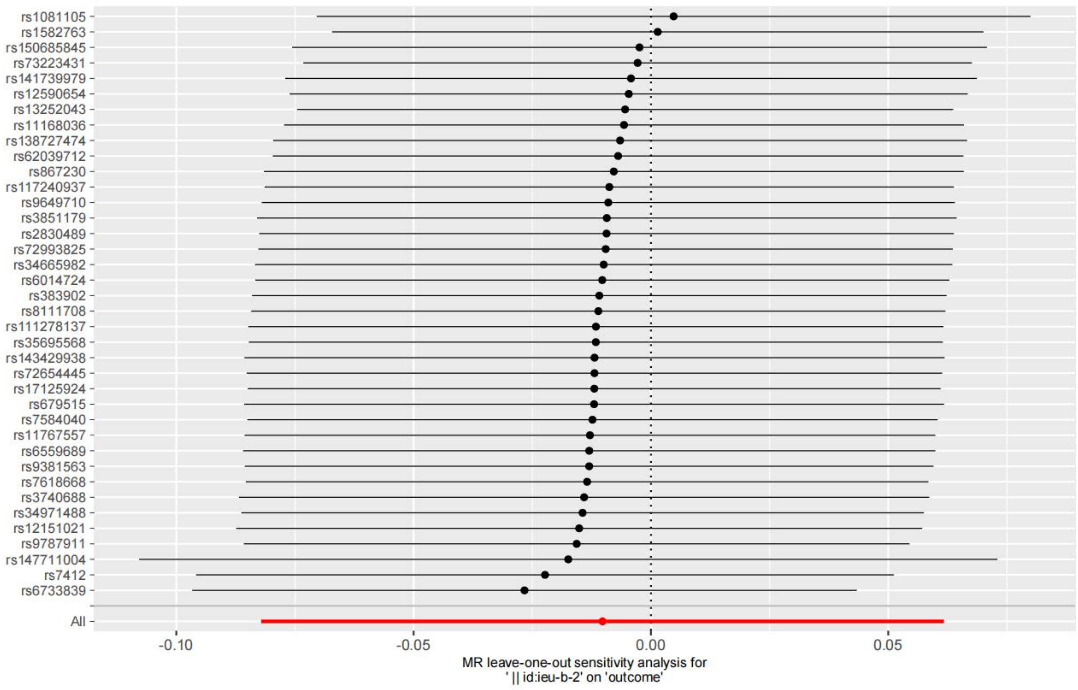

C. MR leave-one-out sensitivity analysis for AD on GROA

eFigure 130. AD-associated SNPs with HGF

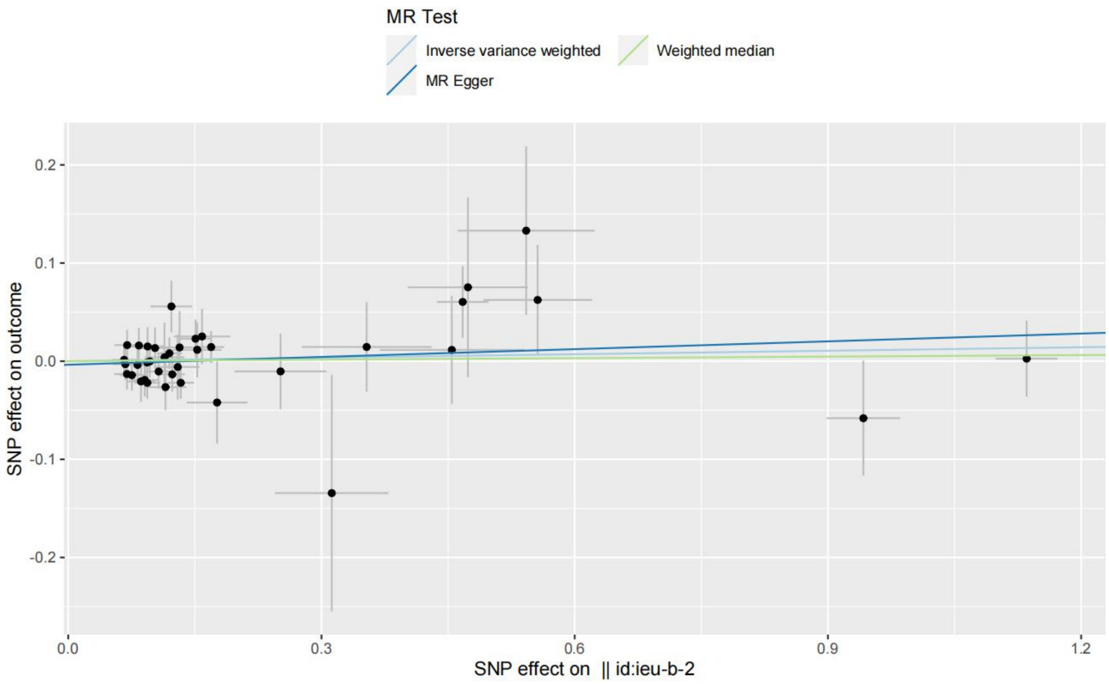

A. Scatter plot of AD on HGF

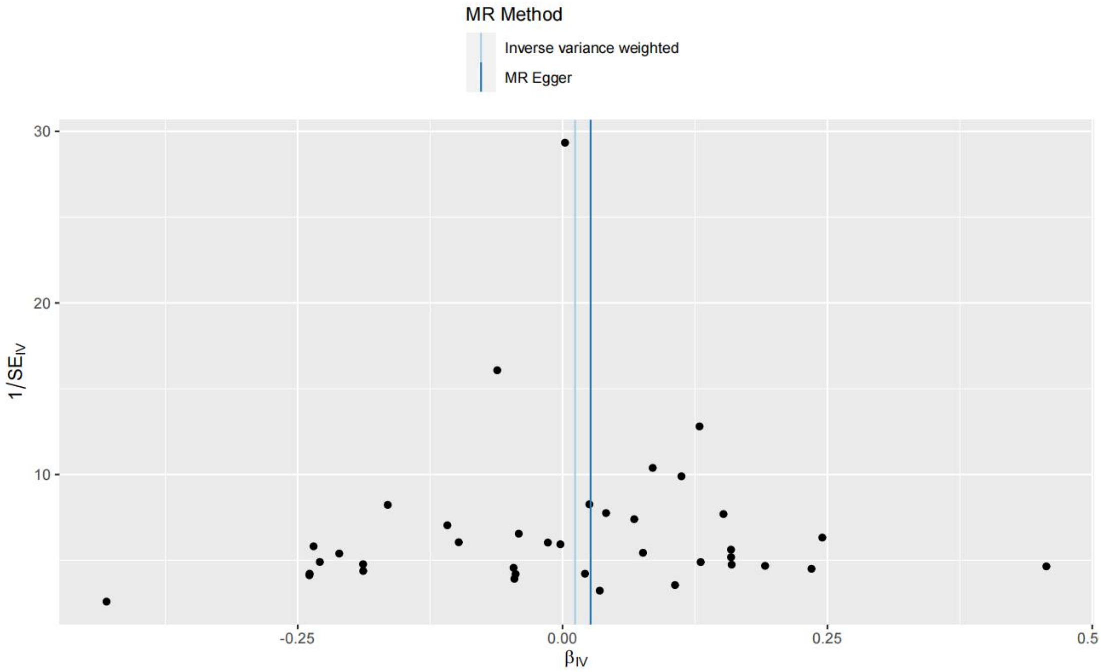

B. Funnel plot of AD on HGF

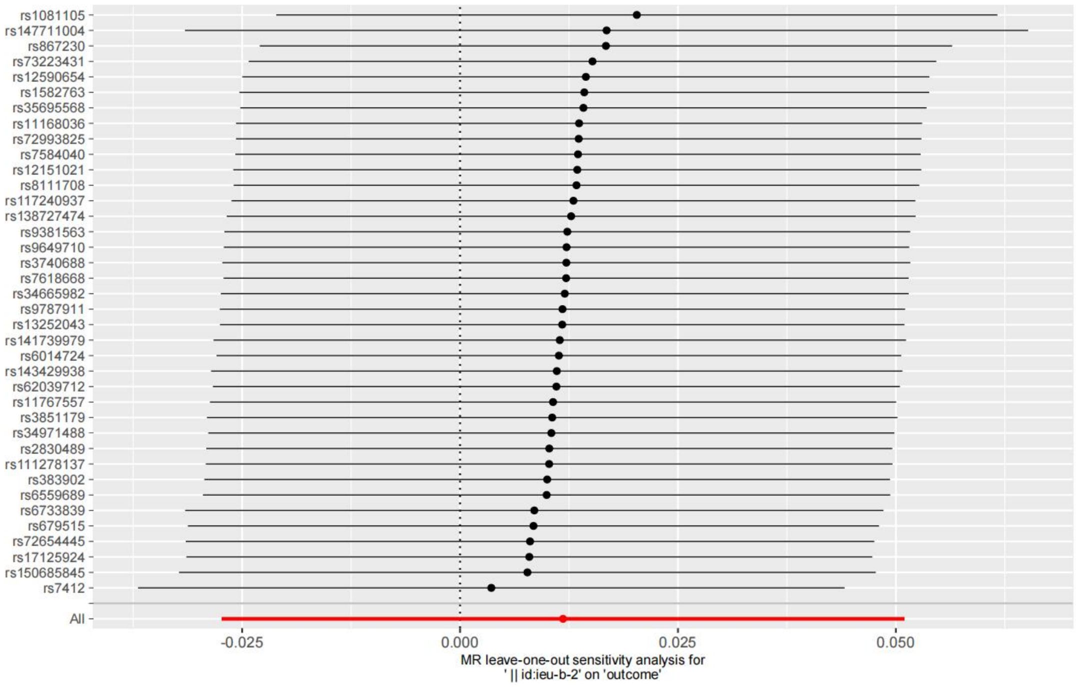

C. MR leave-one-out sensitivity analysis for AD on HGF

eFigure 131. AD-associated SNPs with IFN-G

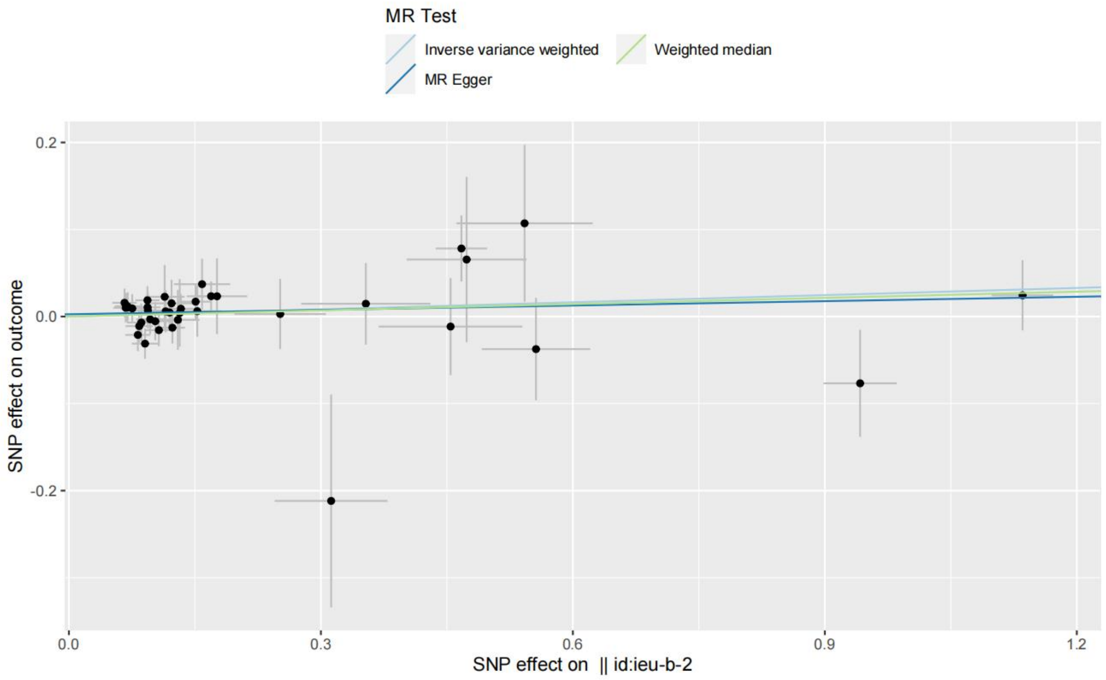

A. Scatter plot of AD on IFN-G

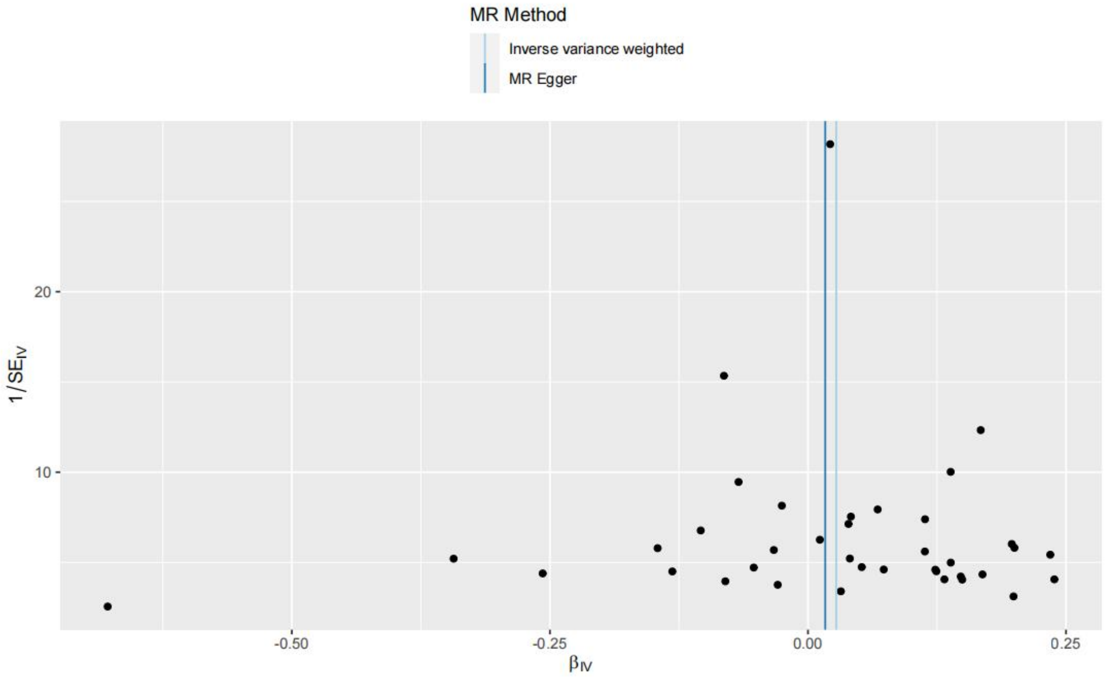

B. Funnel plot of AD on IFN-G

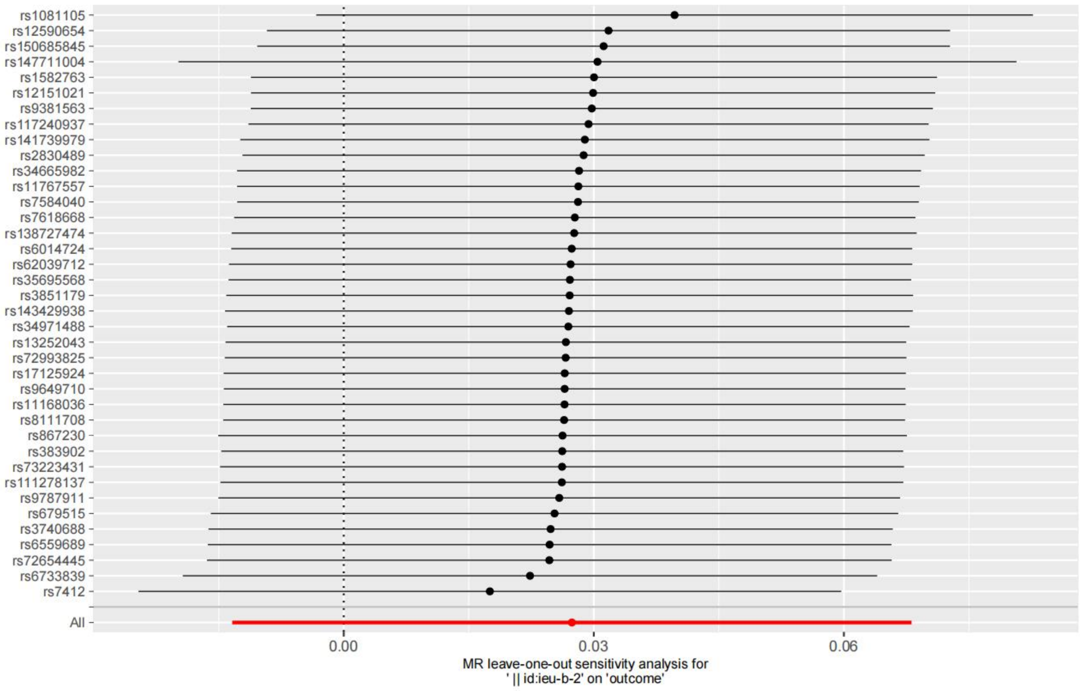

C. MR leave-one-out sensitivity analysis for AD on IFN-G

eFigure 132. AD-associated SNPs with IL-1B

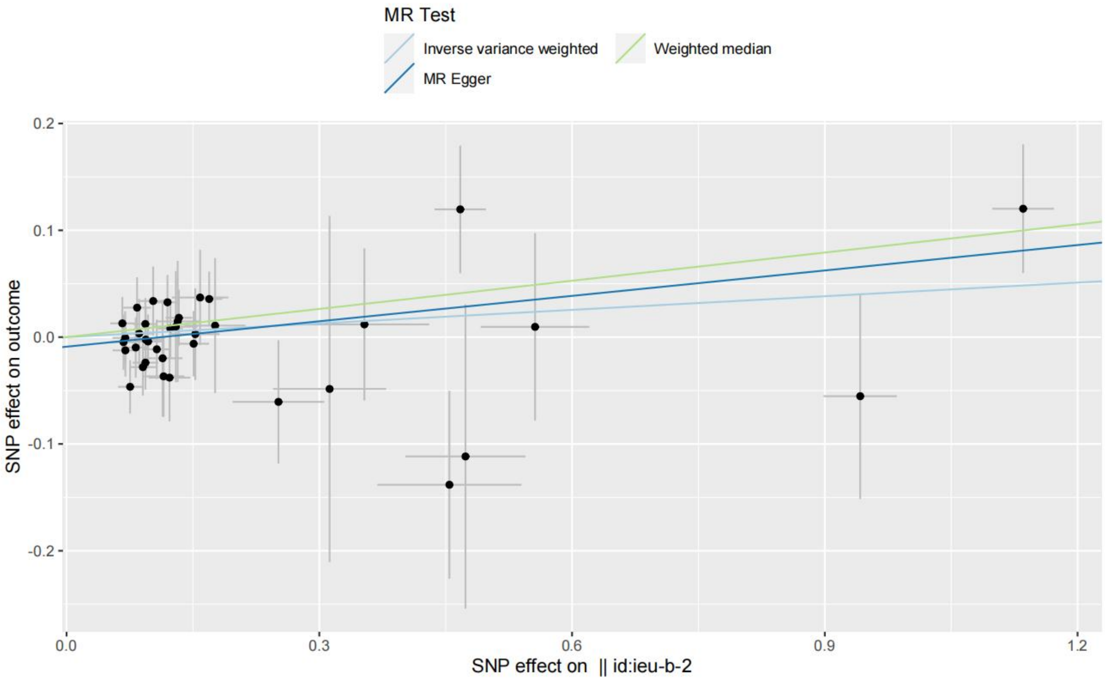

A. Scatter plot of AD on IL-1B

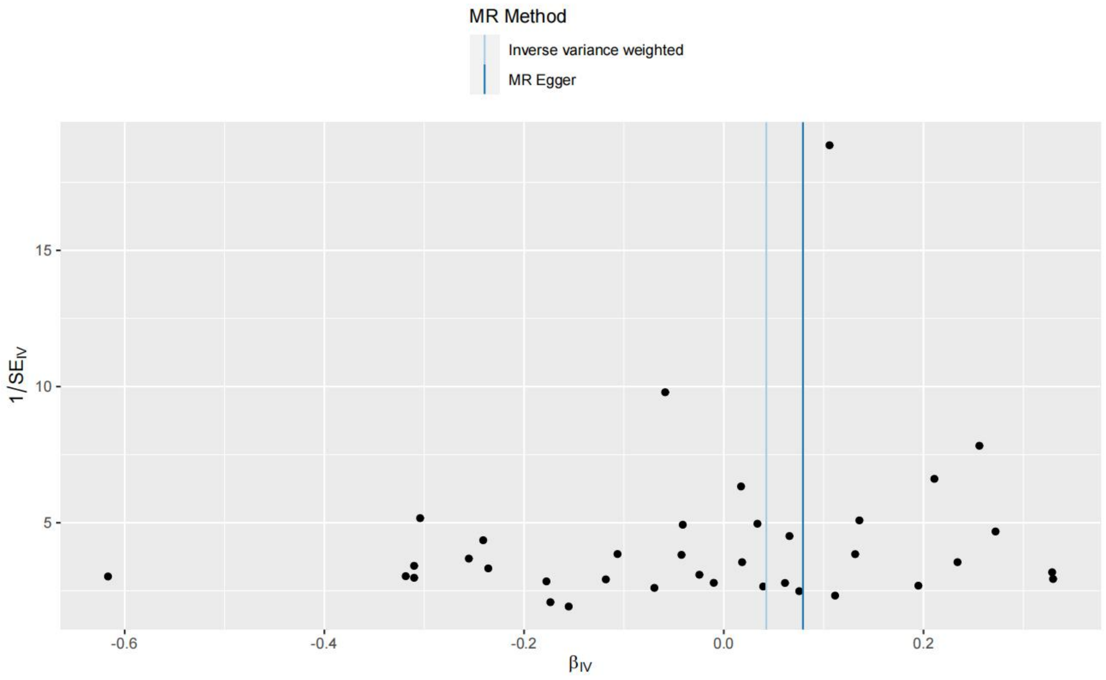

B. Funnel plot of AD on IL-1B

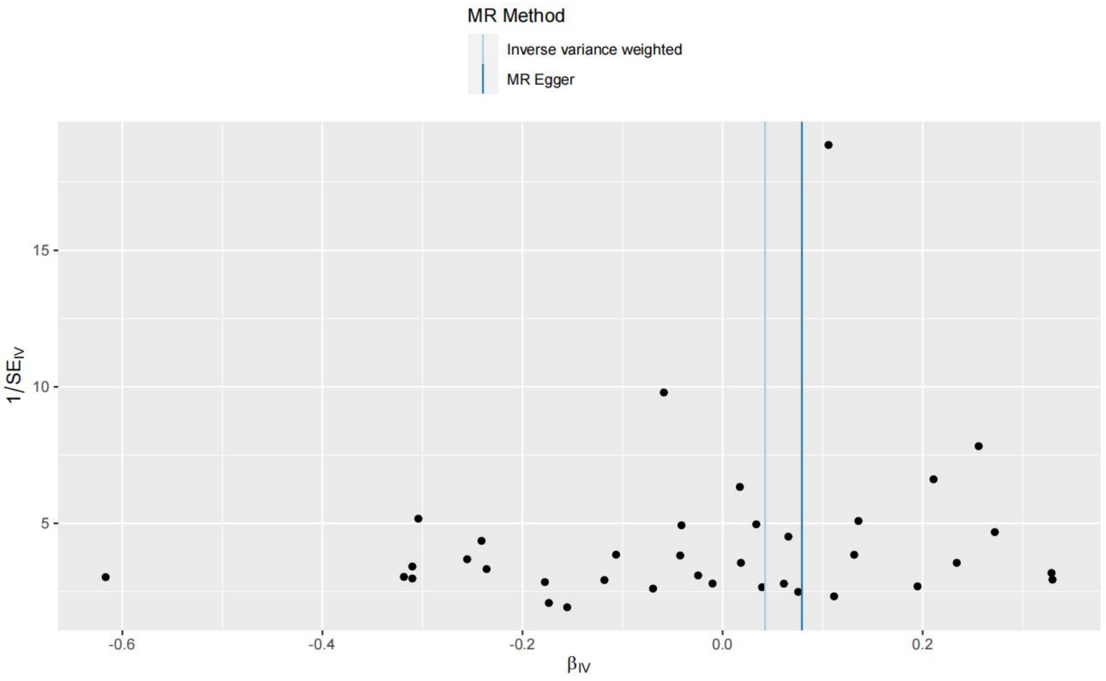

C. MR leave-one-out sensitivity analysis for AD on IL-1B

eFigure 133. AD-associated SNPs with IL-1RA

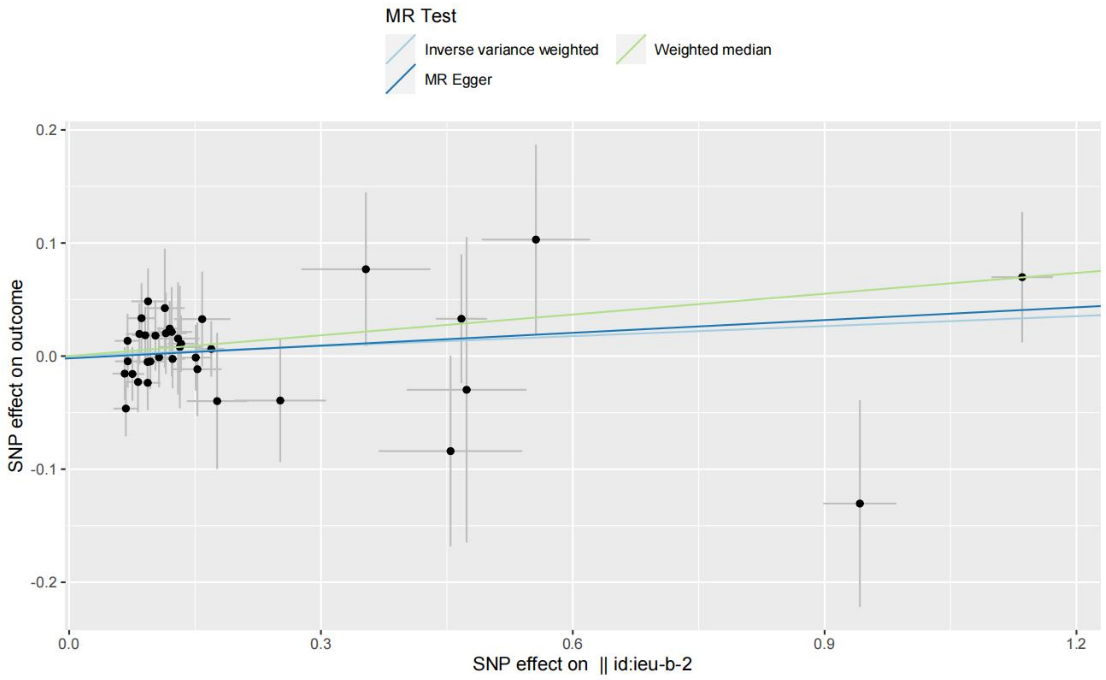

A. Scatter plot of AD on IL-1RA

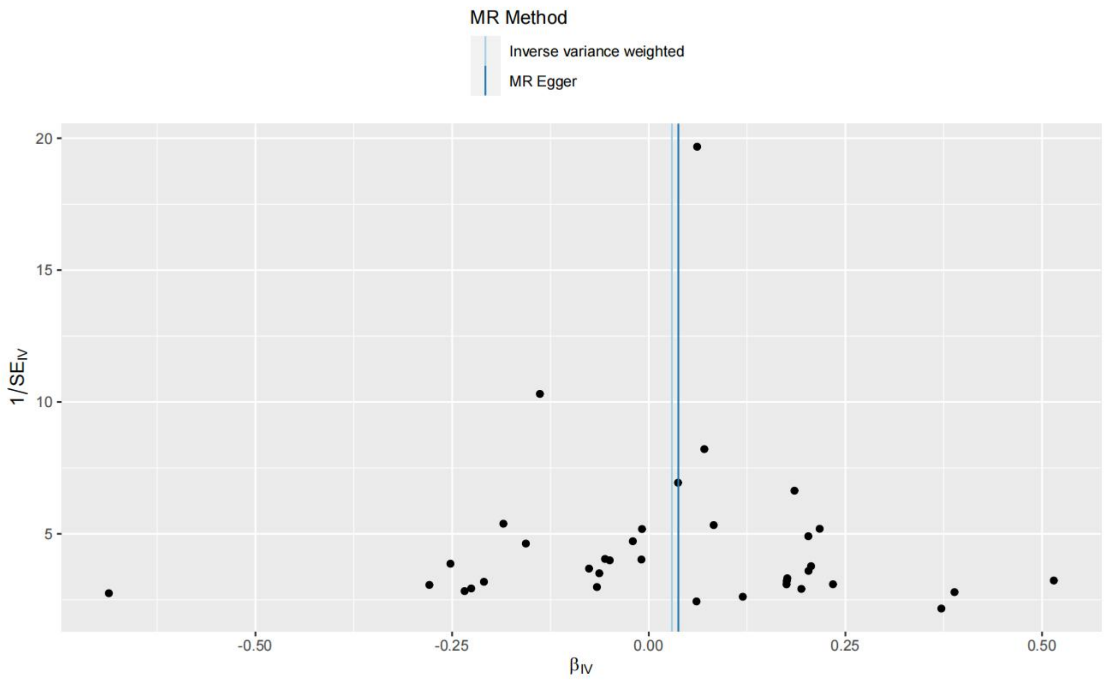

B. Funnel plot of AD on IL-1RA

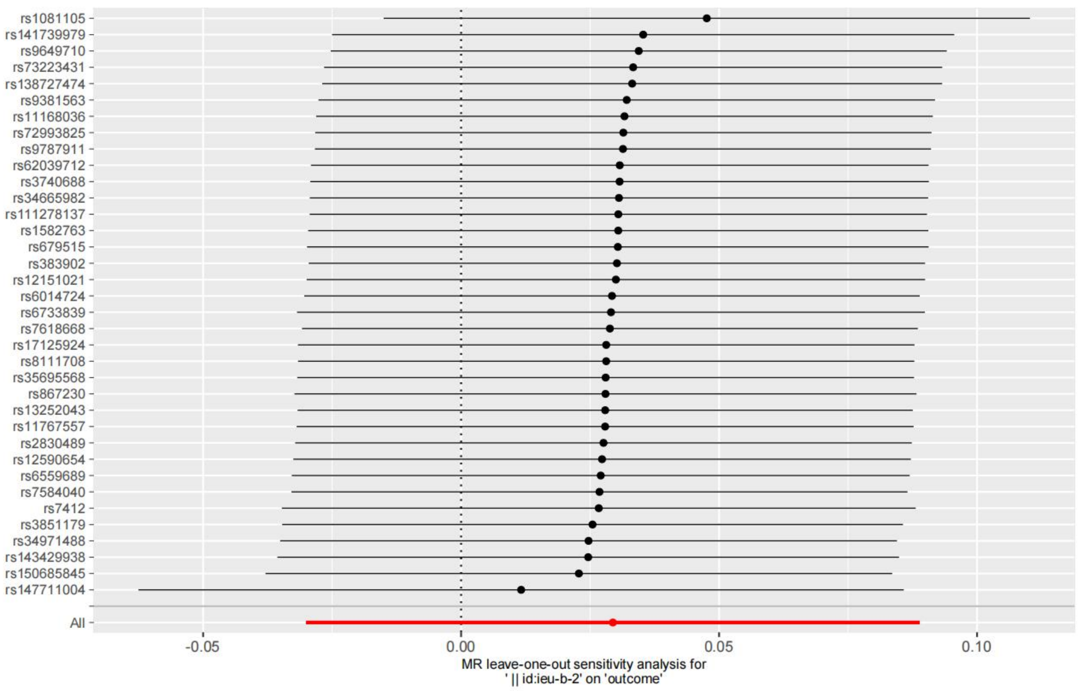

C. MR leave-one-out sensitivity analysis for AD on IL-1RA

eFigure 134. AD-associated SNPs with IL-2

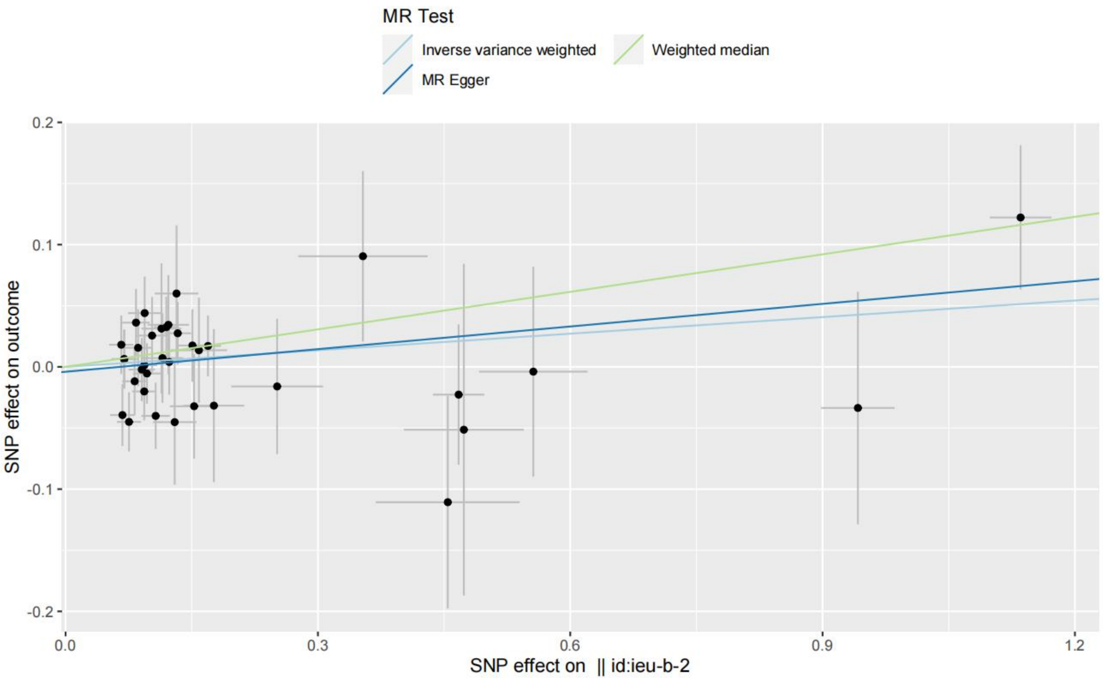

A. Scatter plot of AD on IL-2

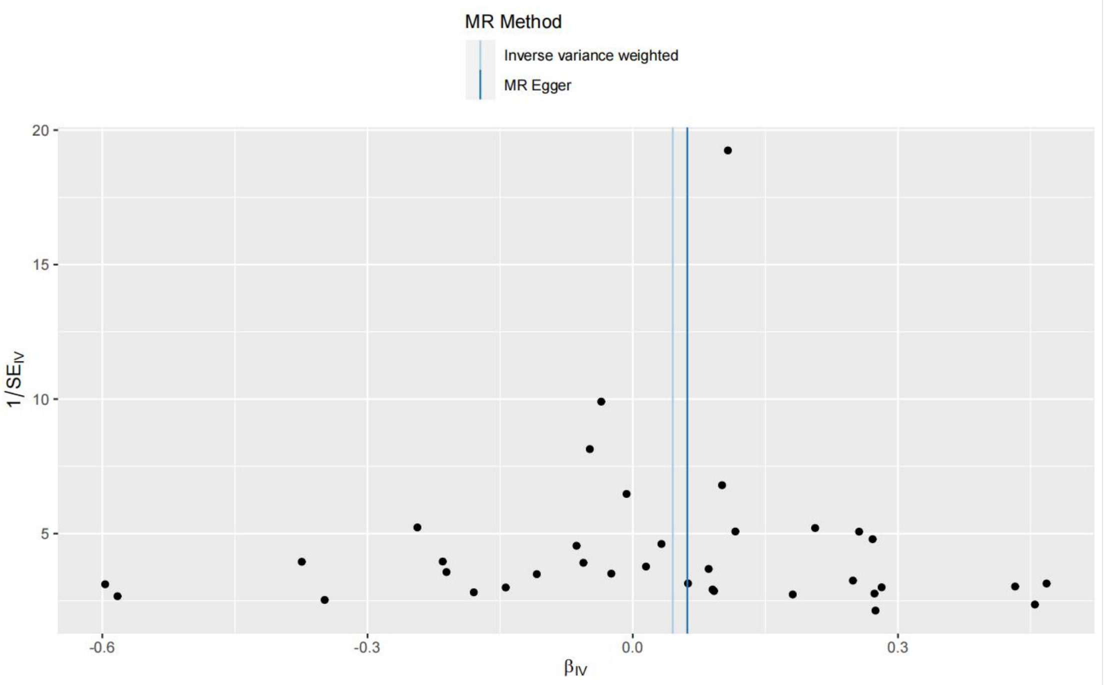

B. Funnel plot of AD on IL-2

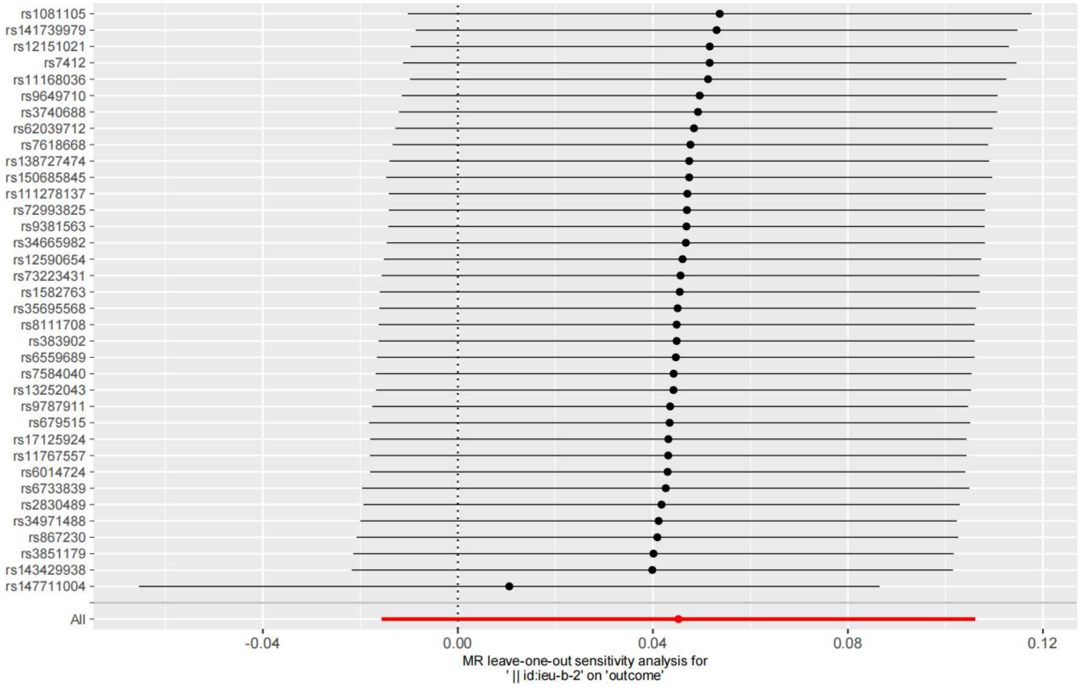

C. MR leave-one-out sensitivity analysis for AD on IL-2

eFigure 135. AD-associated SNPs with IL-2RA

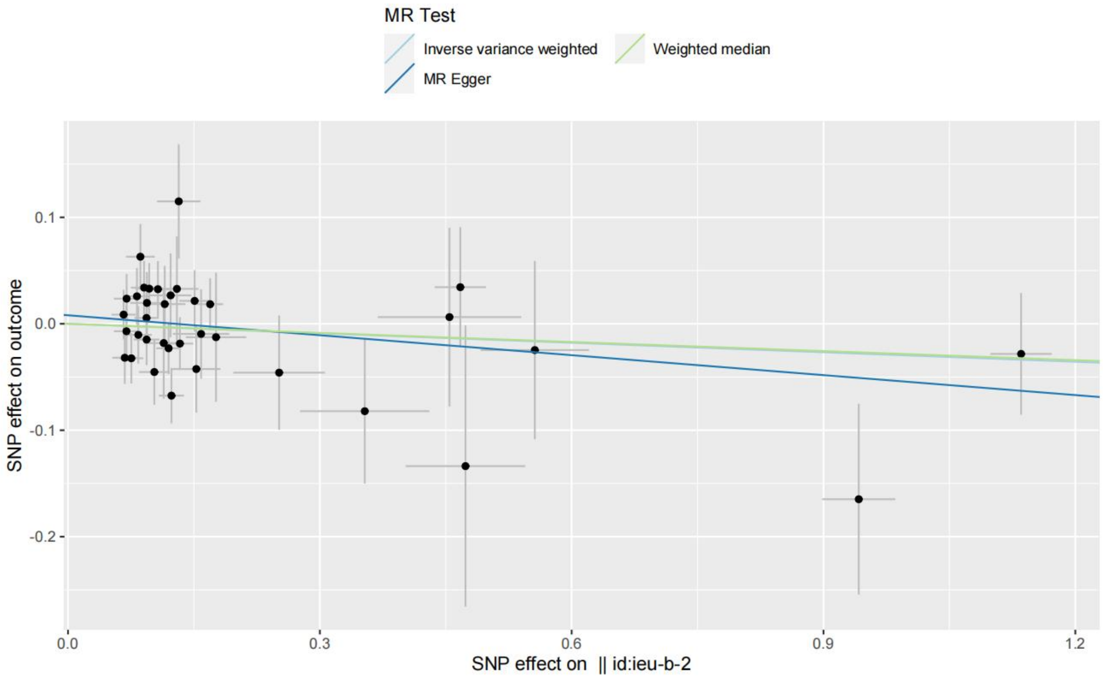

A. Scatter plot of AD on IL-2RA

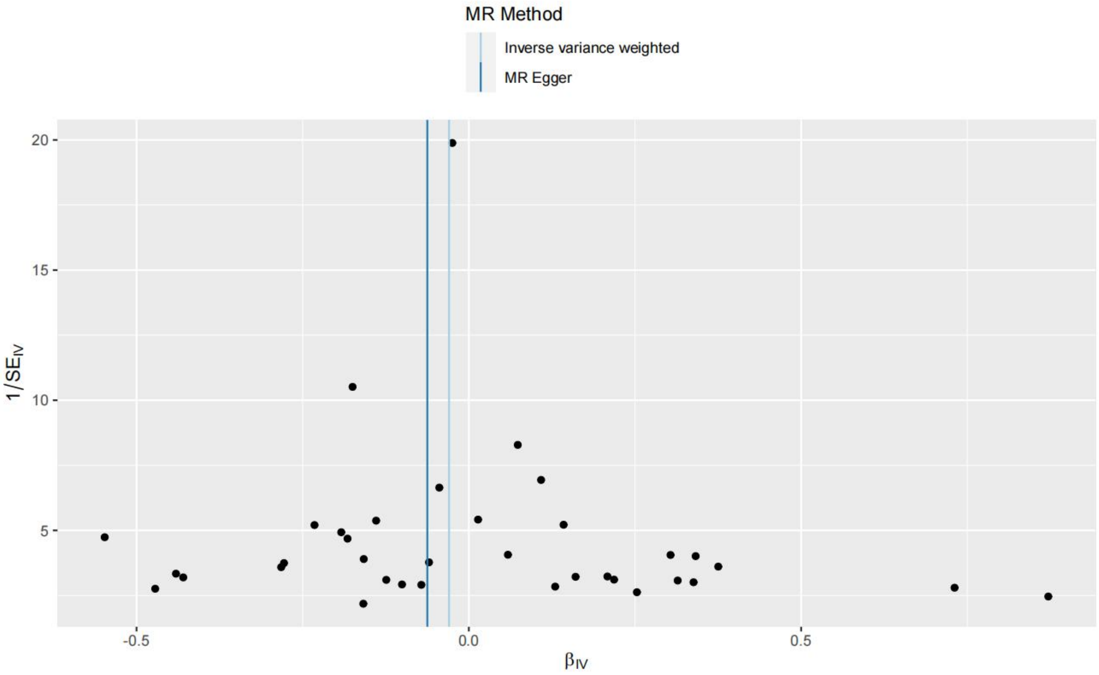

B. Funnel plot of AD on IL-2RA

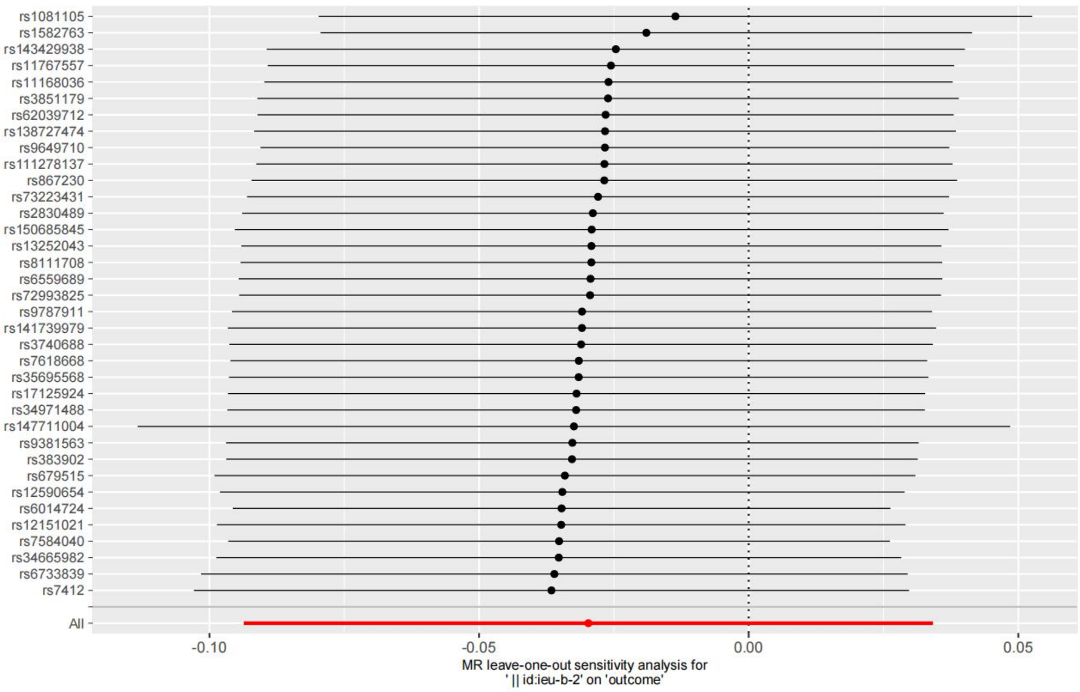

C. MR leave-one-out sensitivity analysis for AD on IL-2RA

eFigure 136. AD-associated SNPs with IL-4

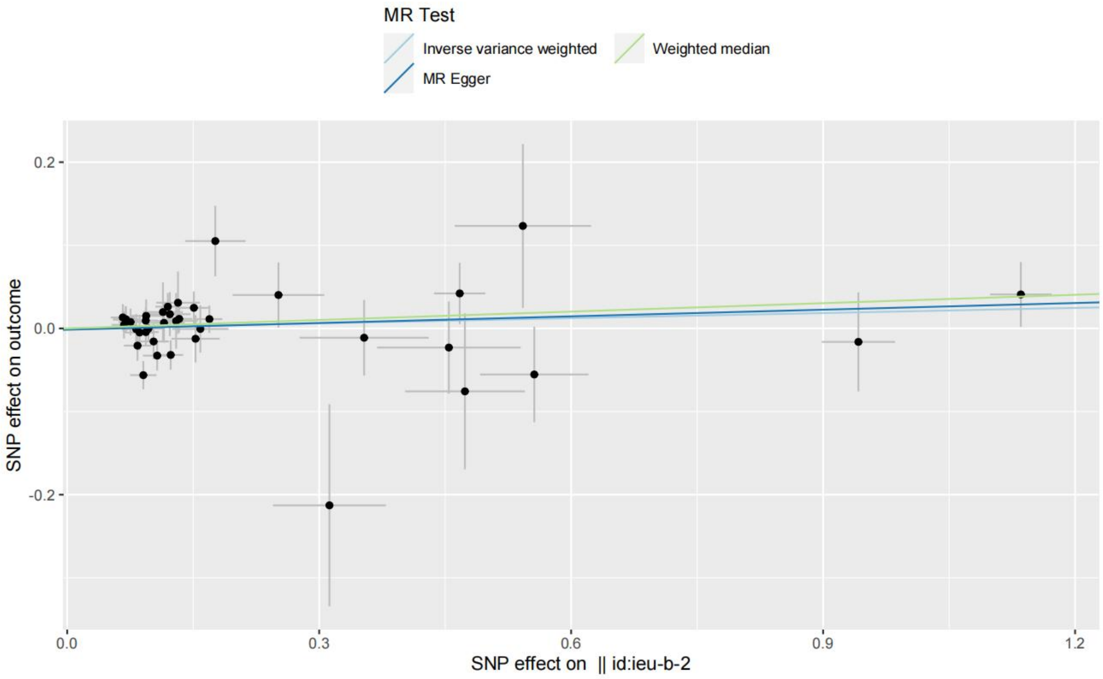

A. Scatter plot of AD on IL-4

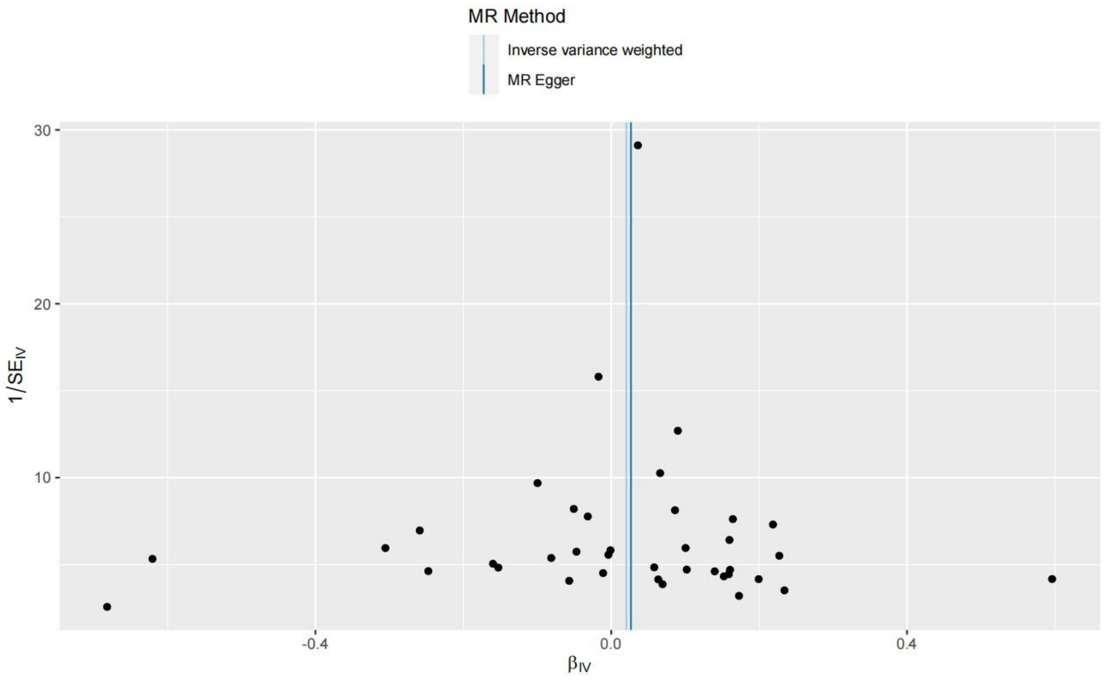

B. Funnel plot of AD on IL-4

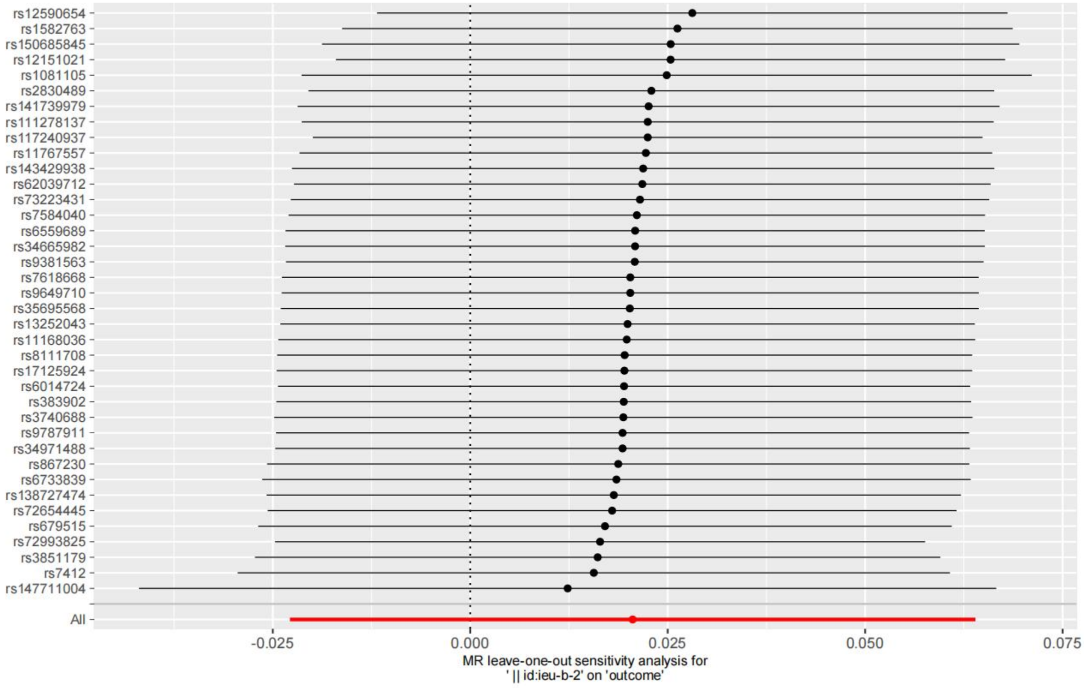

C. MR leave-one-out sensitivity analysis for AD on IL-4

eFigure 137. AD-associated SNPs with IL-5

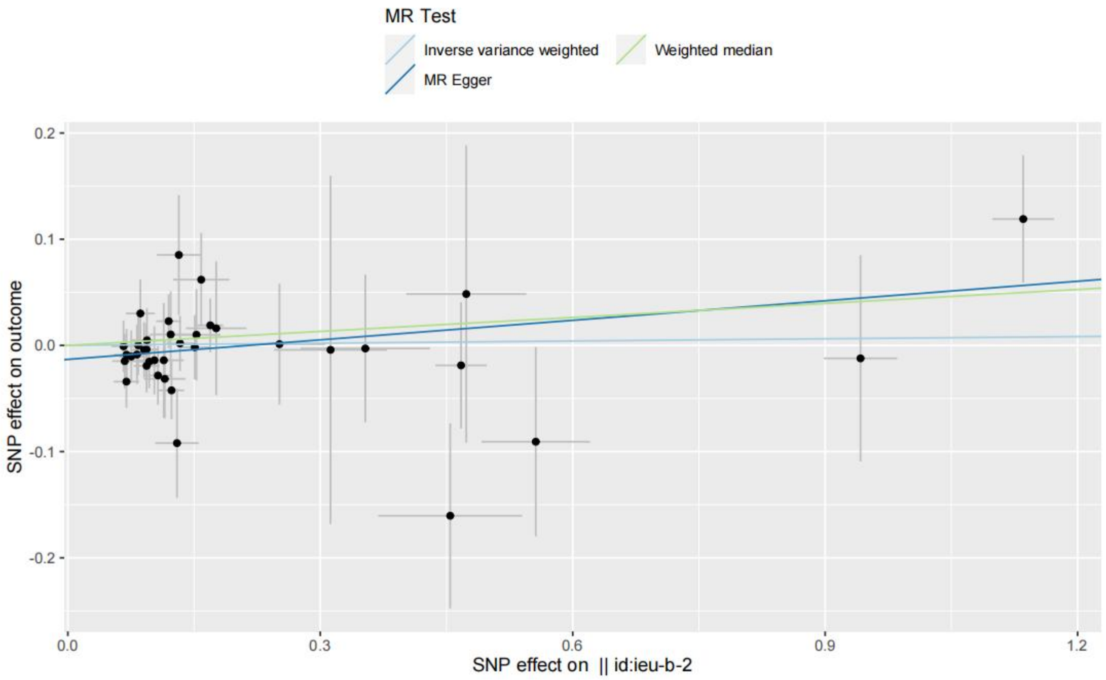

A. Scatter plot of AD on IL-5

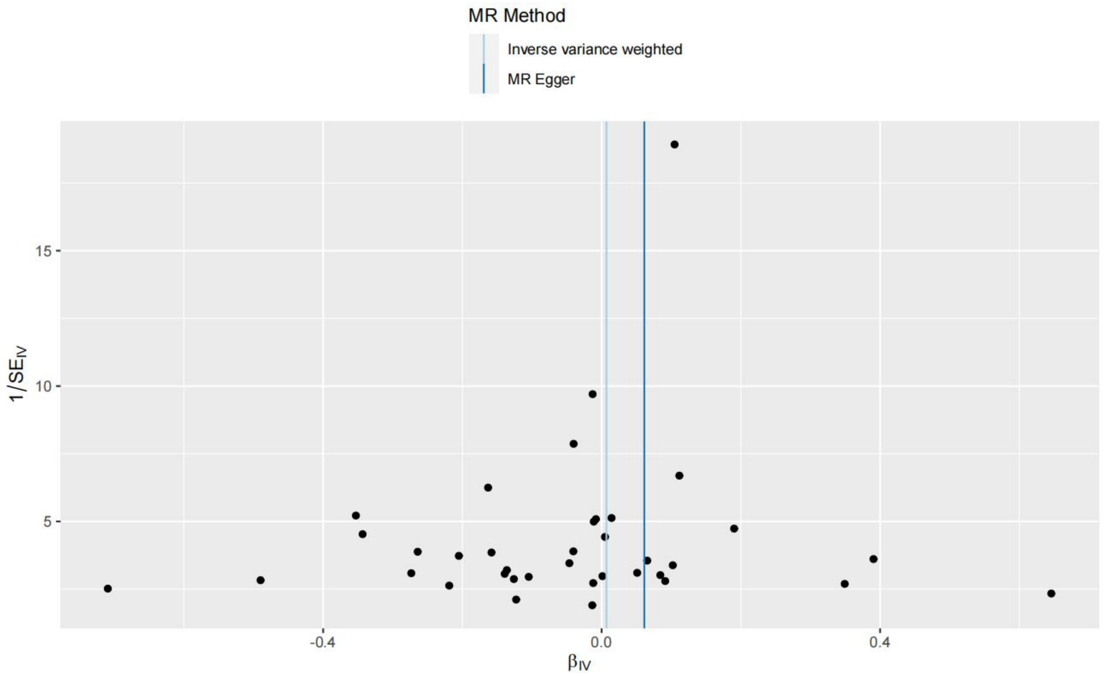

B. Funnel plot of AD on IL-5

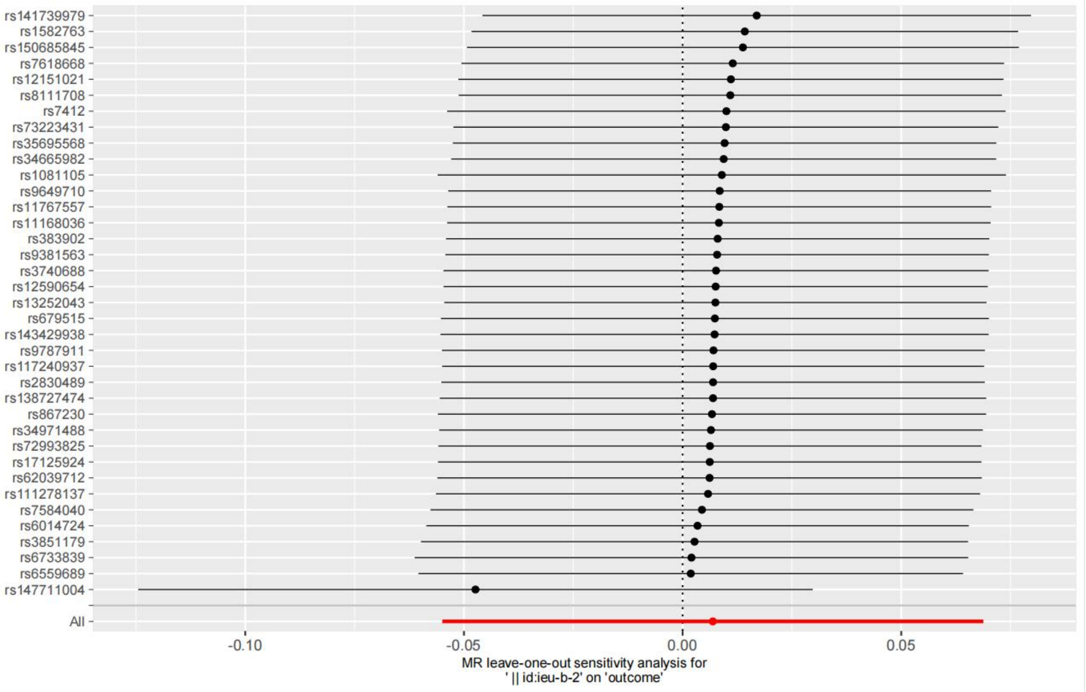

C. MR leave-one-out sensitivity analysis for AD on IL-5

eFigure 138. AD-associated SNPs with IL-6

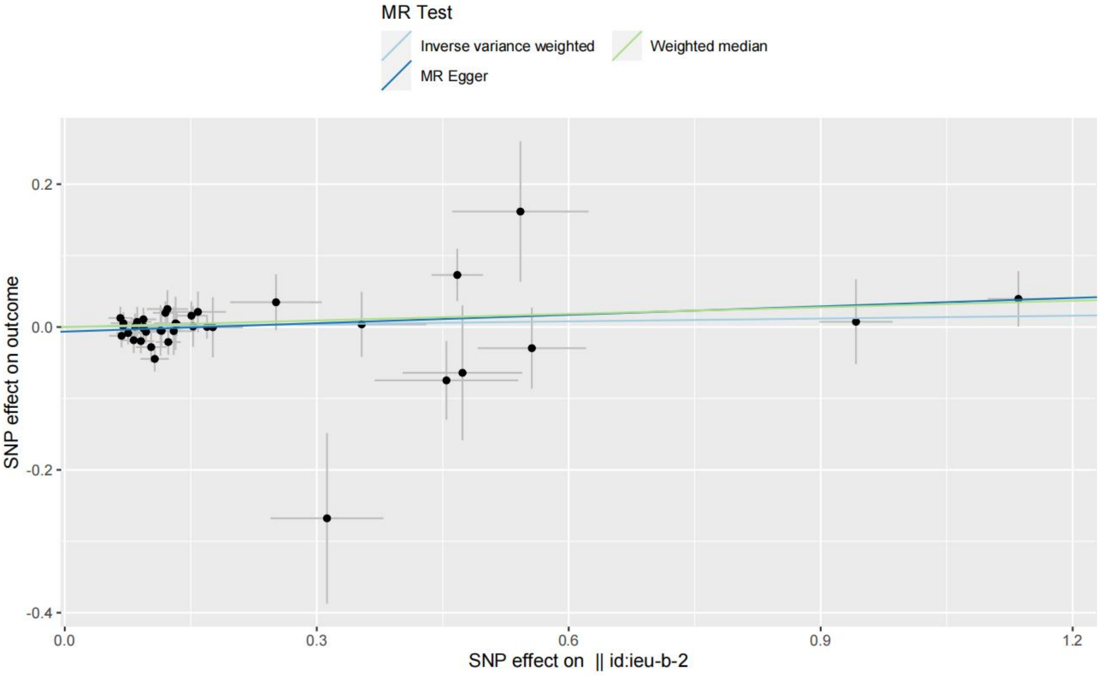

A. Scatter plot of AD on IL-6

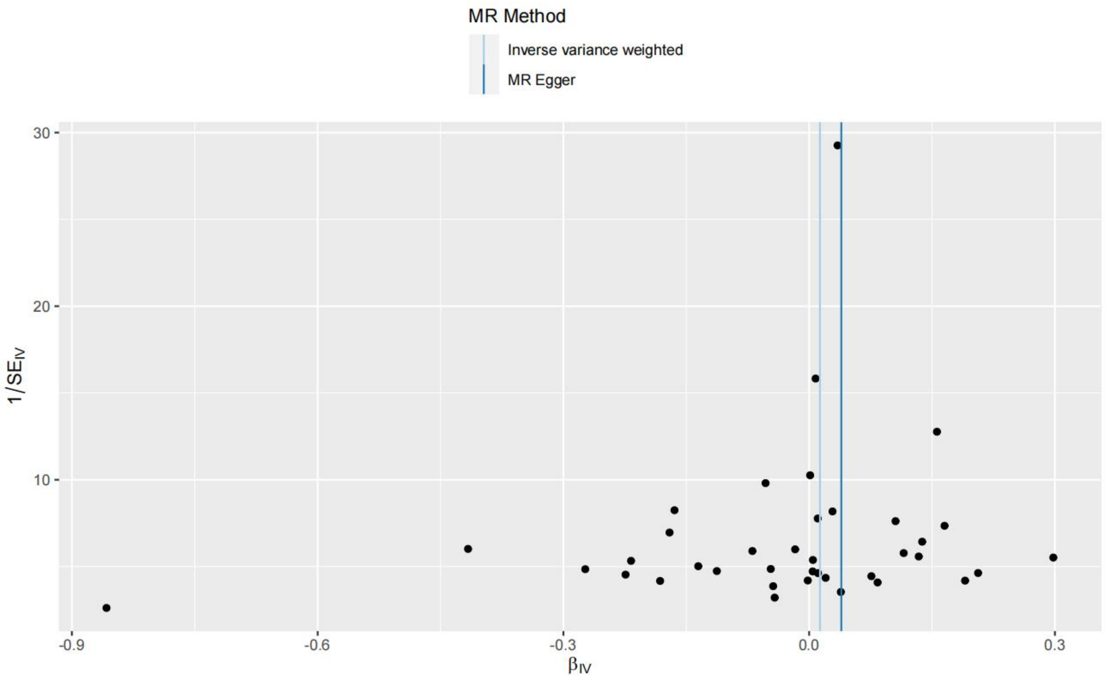

B. Funnel plot of AD on IL-6

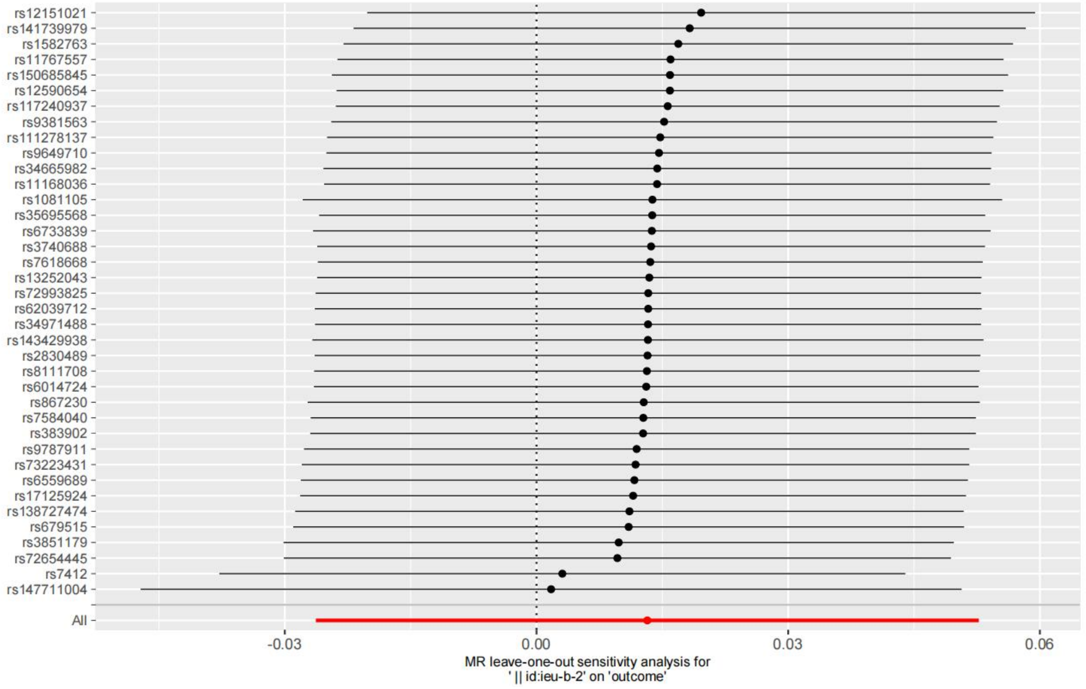

C. MR leave-one-out sensitivity analysis for AD on IL-6

eFigure 139. AD-associated SNPs with IL-7

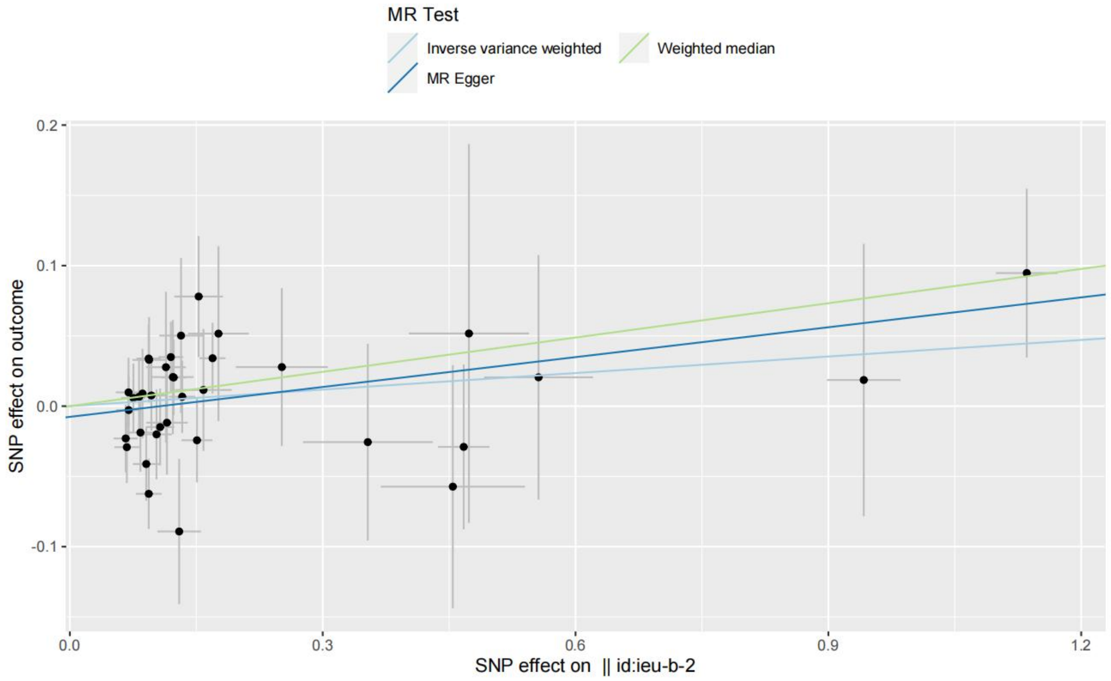

A. Scatter plot of AD on IL-7

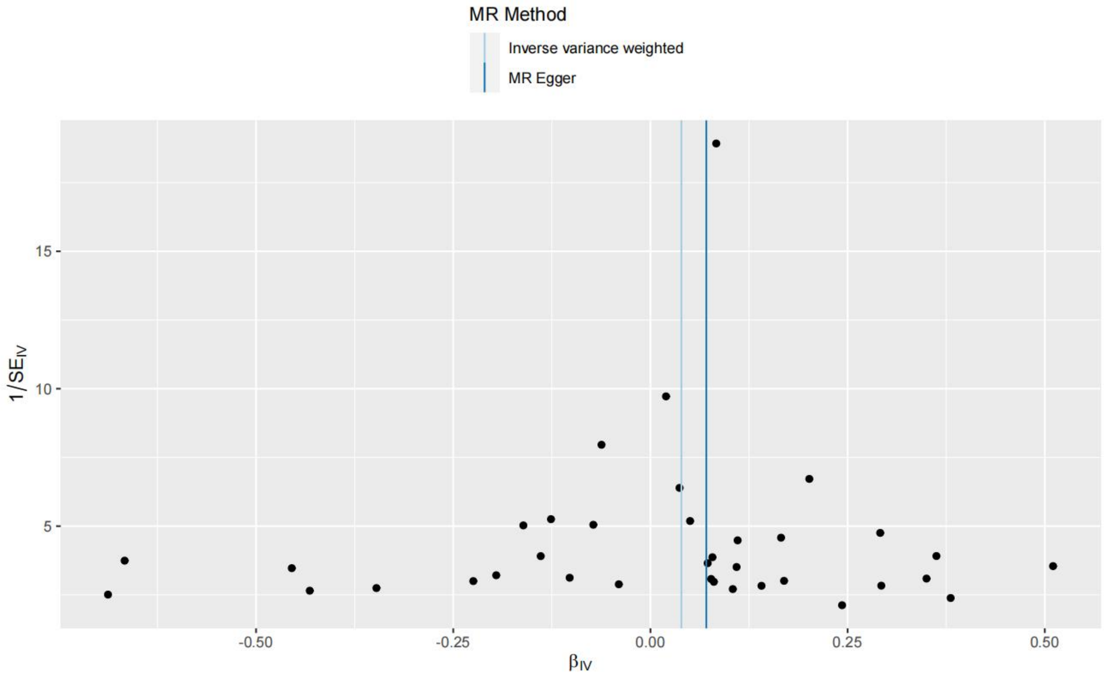

B. Funnel plot of AD on IL-7

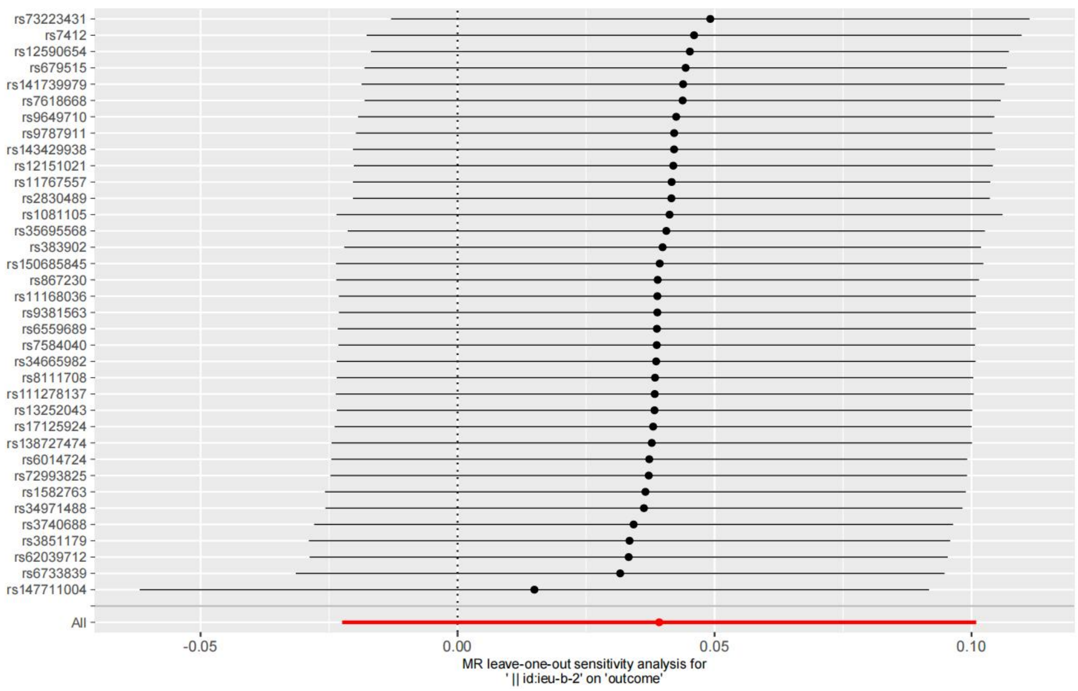

C. MR leave-one-out sensitivity analysis for AD on IL-7

eFigure 140. AD-associated SNPs with IL-8

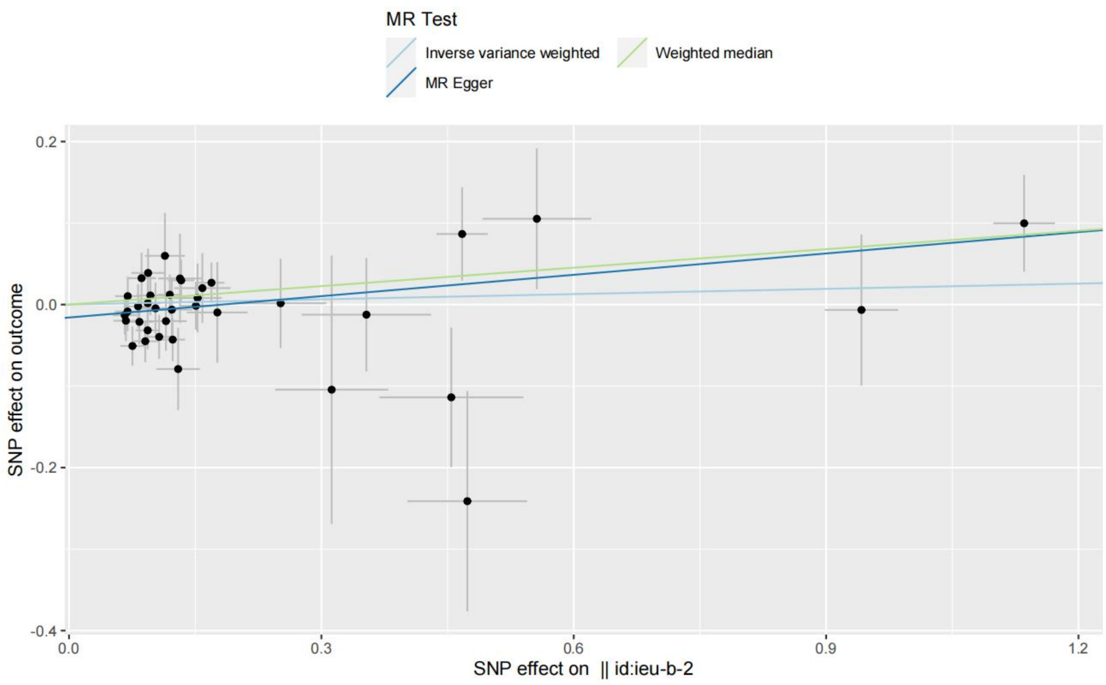

A. Scatter plot of AD on IL-8

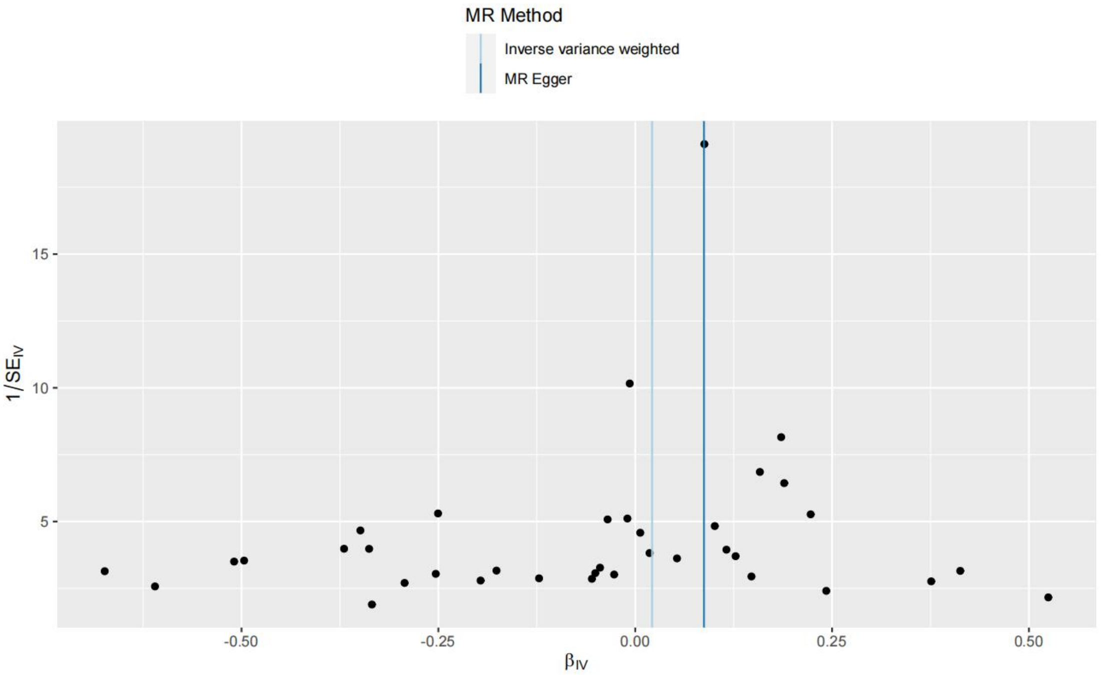

B. Funnel plot of AD on IL-8

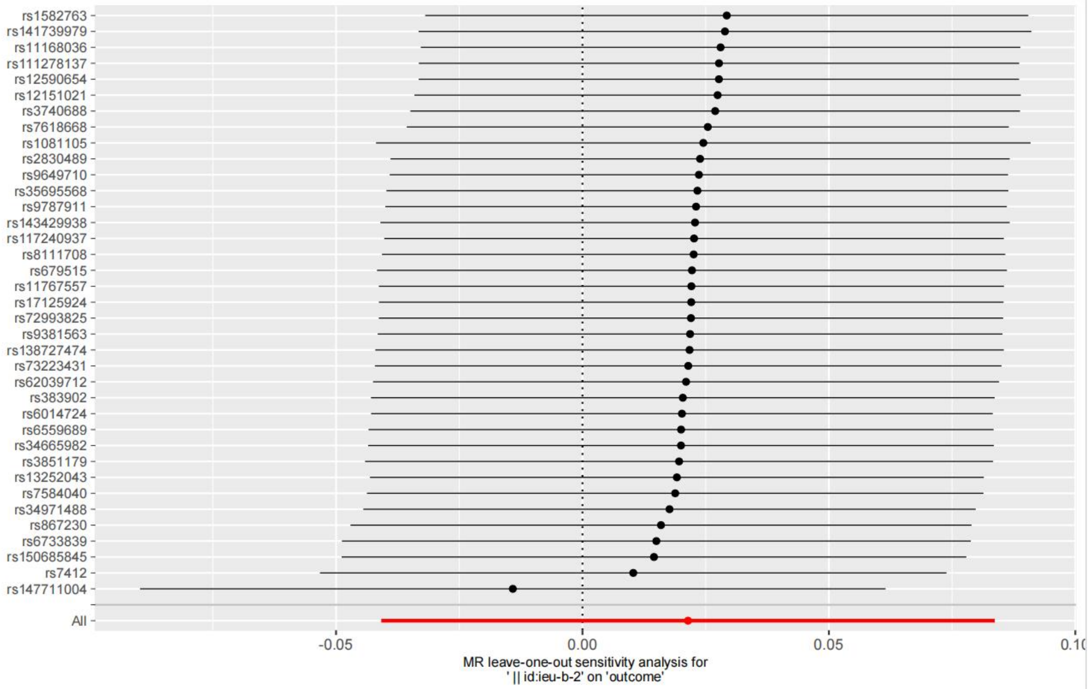

C. MR leave-one-out sensitivity analysis for AD on IL-8

eFigure 141. AD-associated SNPs with IL-9

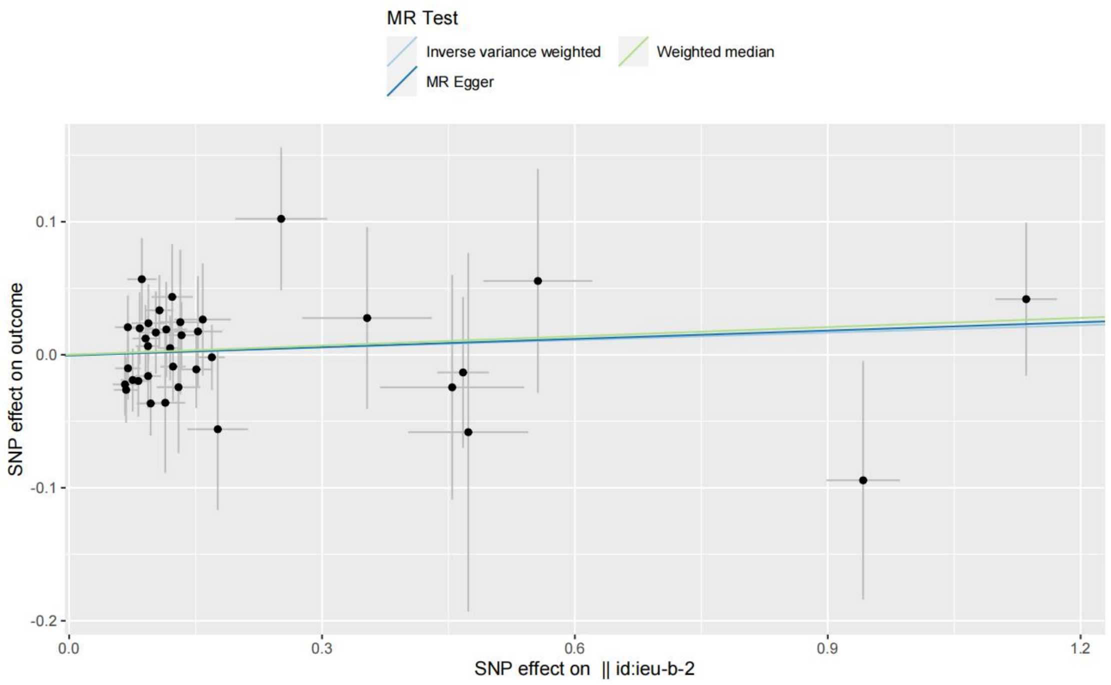

A. Scatter plot of AD on IL-9

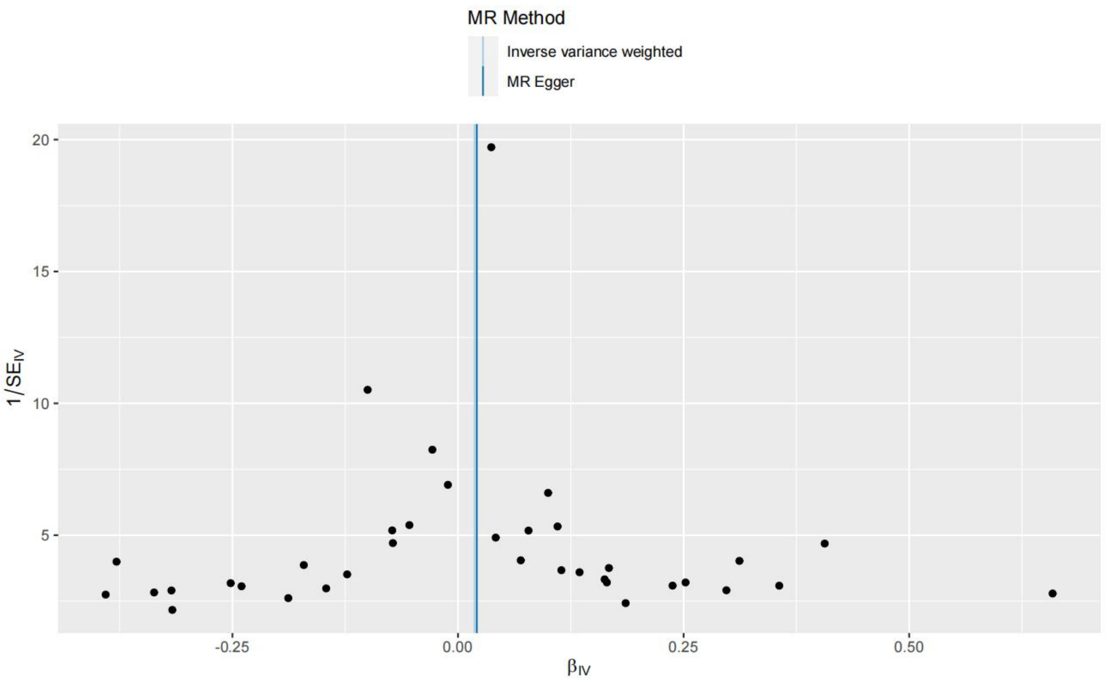

B. Funnel plot of AD on IL-9

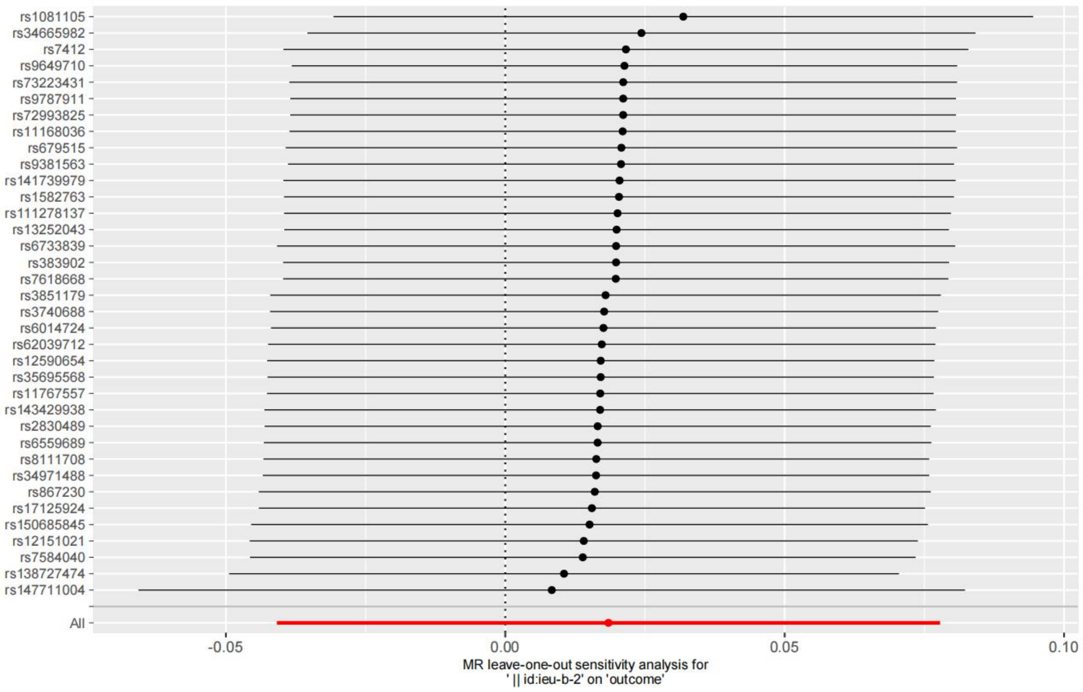

C. MR leave-one-out sensitivity analysis for AD on IL-9

eFigure 142. AD-associated SNPs with IL-10

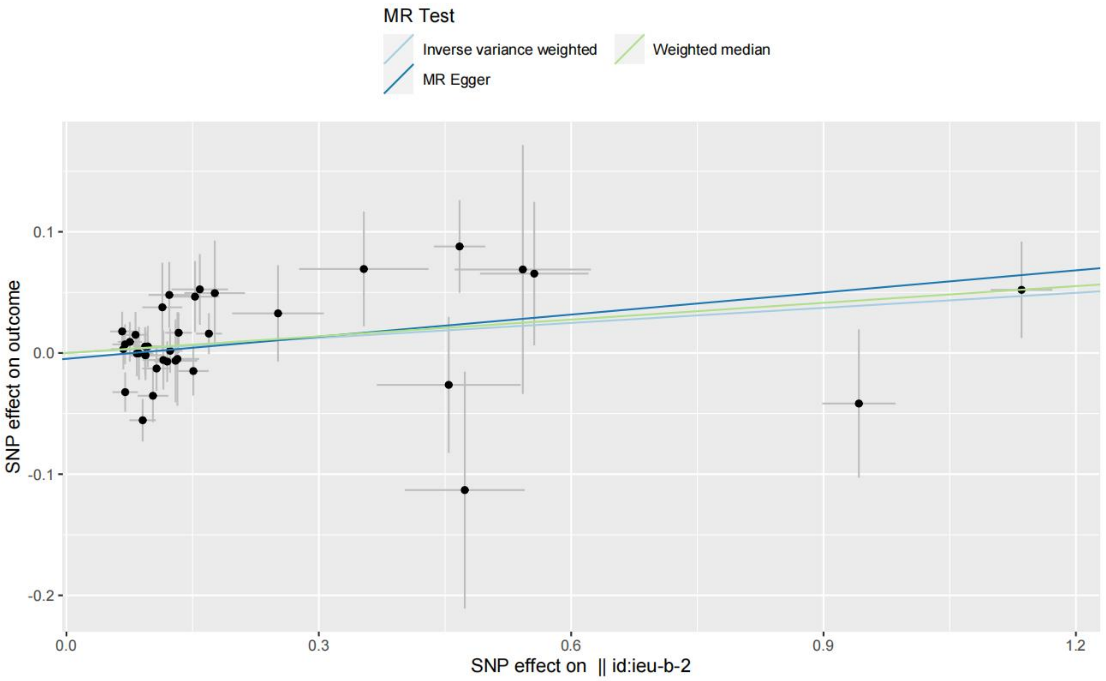

A. Scatter plot of AD on IL-10

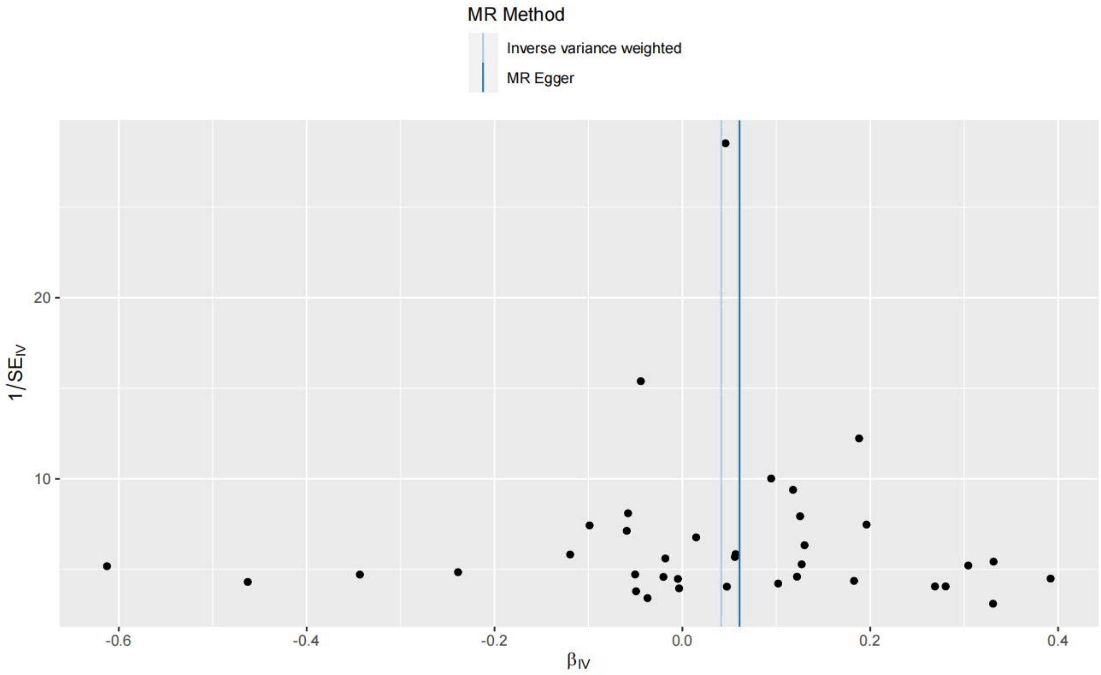

B. Funnel plot of AD on IL-10

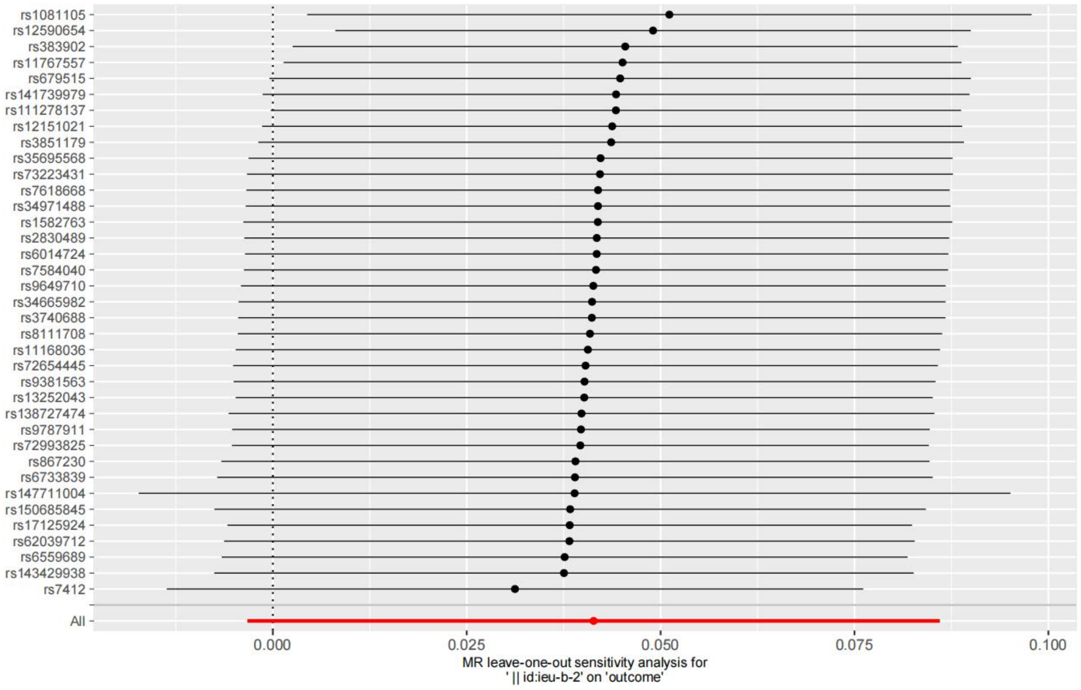

C. MR leave-one-out sensitivity analysis for AD on IL-10

eFigure 143. AD-associated SNPs with IL-12

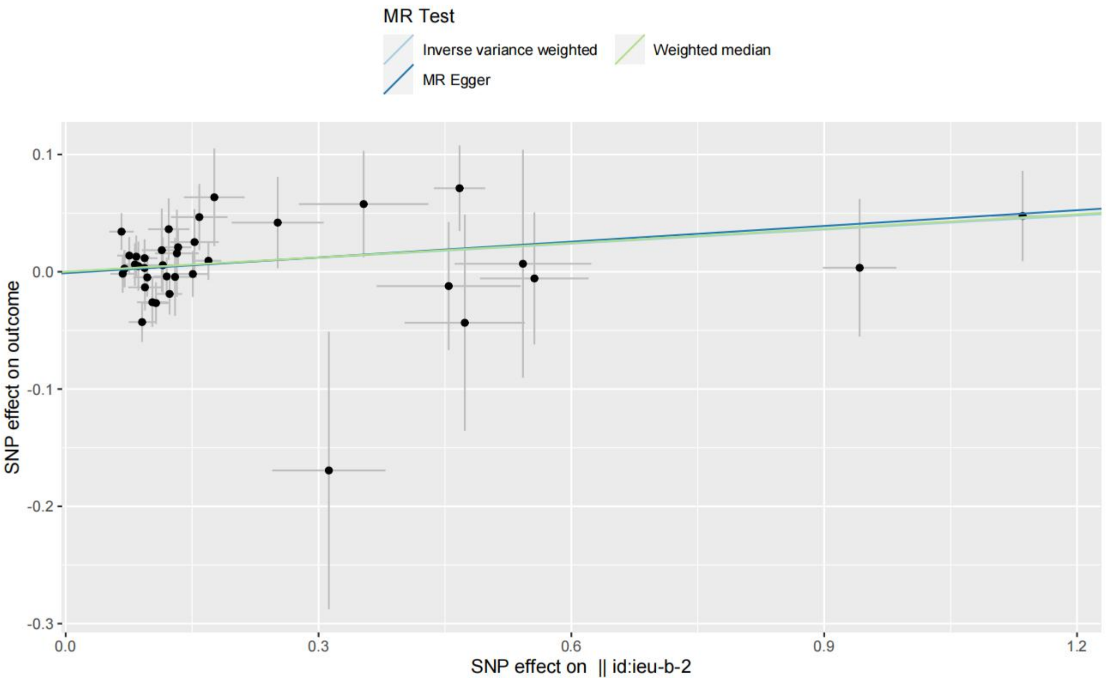

A. Scatter plot of AD on IL-12

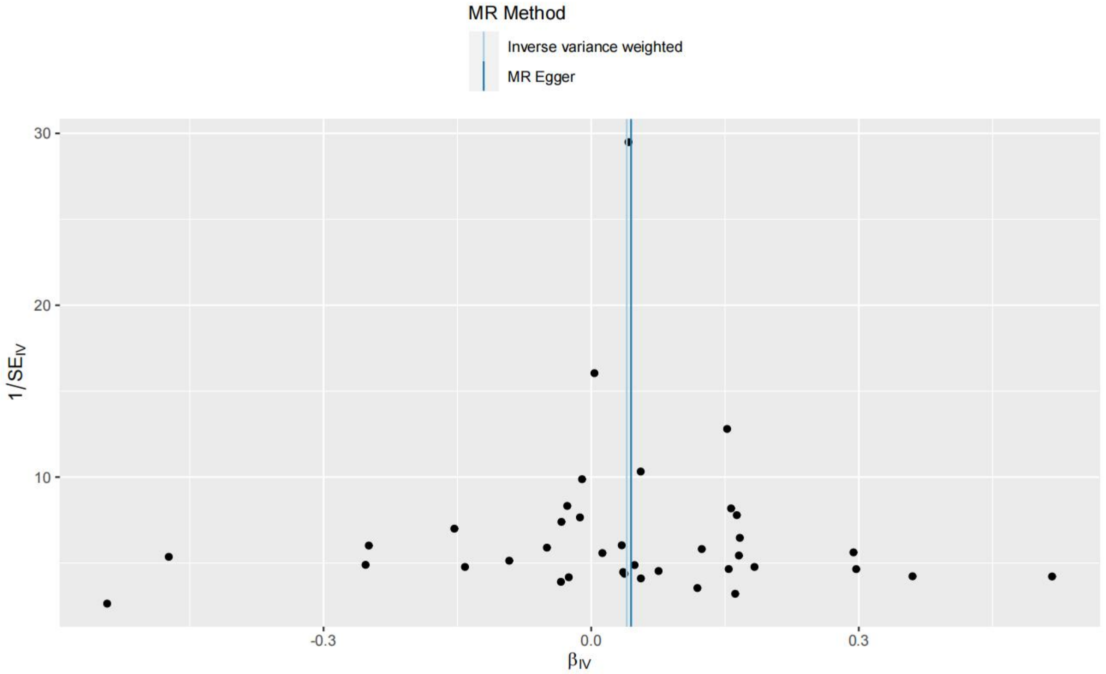

B. Funnel plot of AD on IL-12

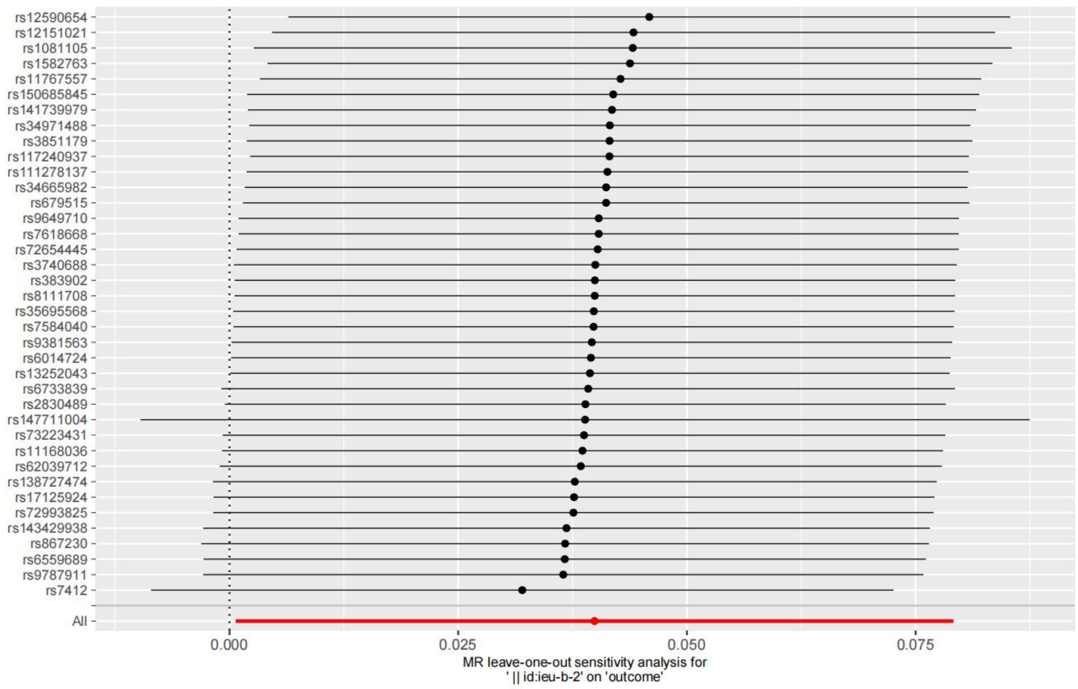

C. MR leave-one-out sensitivity analysis for AD on IL-12

eFigure 144. AD-associated SNPs with IL-13

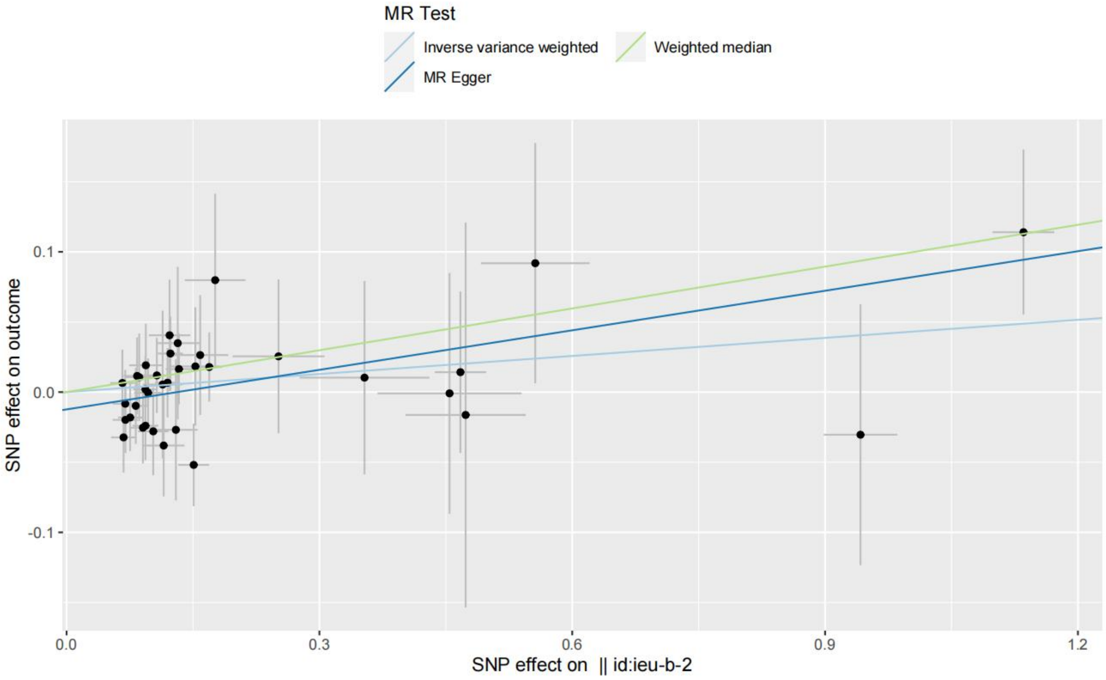

A. Scatter plot of AD on IL-13

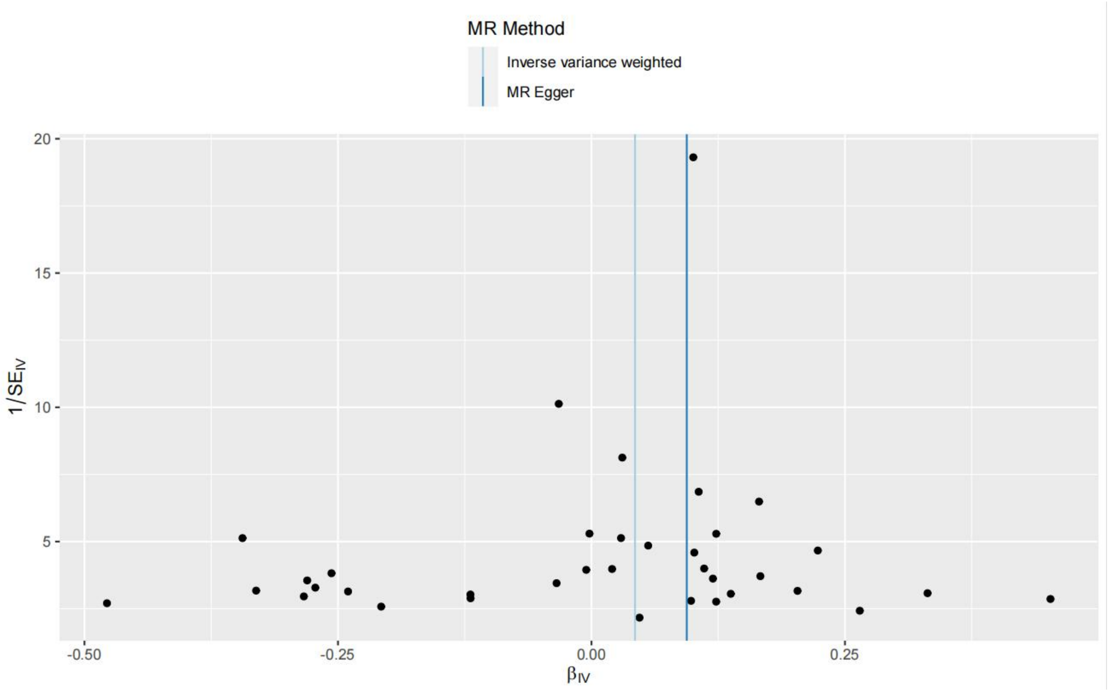

B. Funnel plot of AD on IL-13

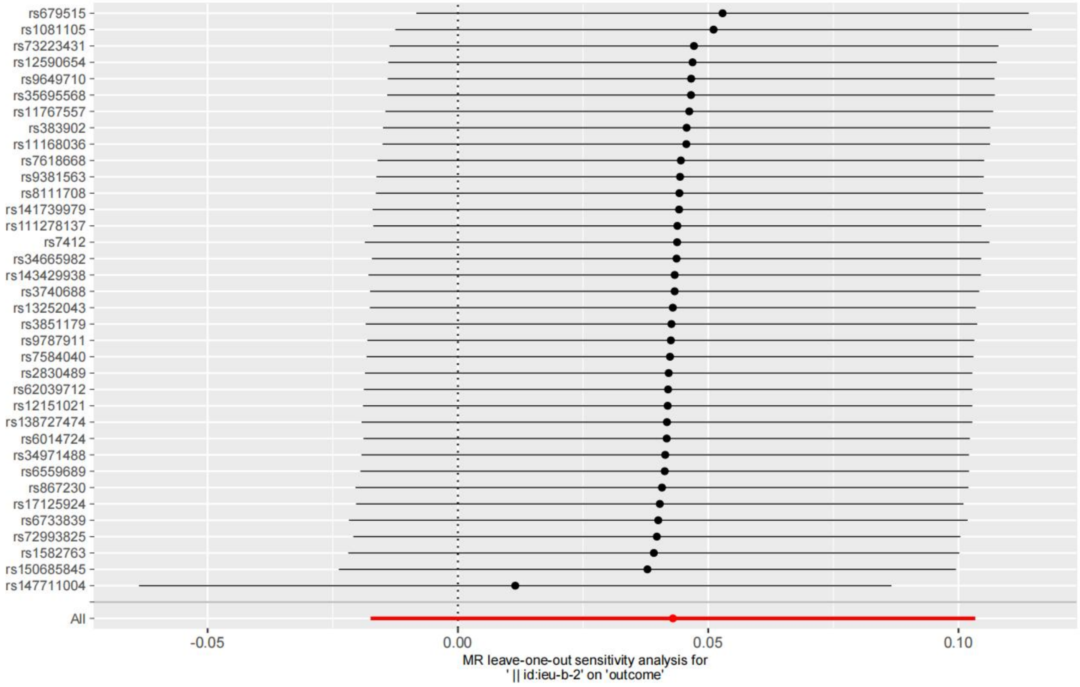

C. MR leave-one-out sensitivity analysis for AD on IL-13

eFigure 145. AD-associated SNPs with IL-16

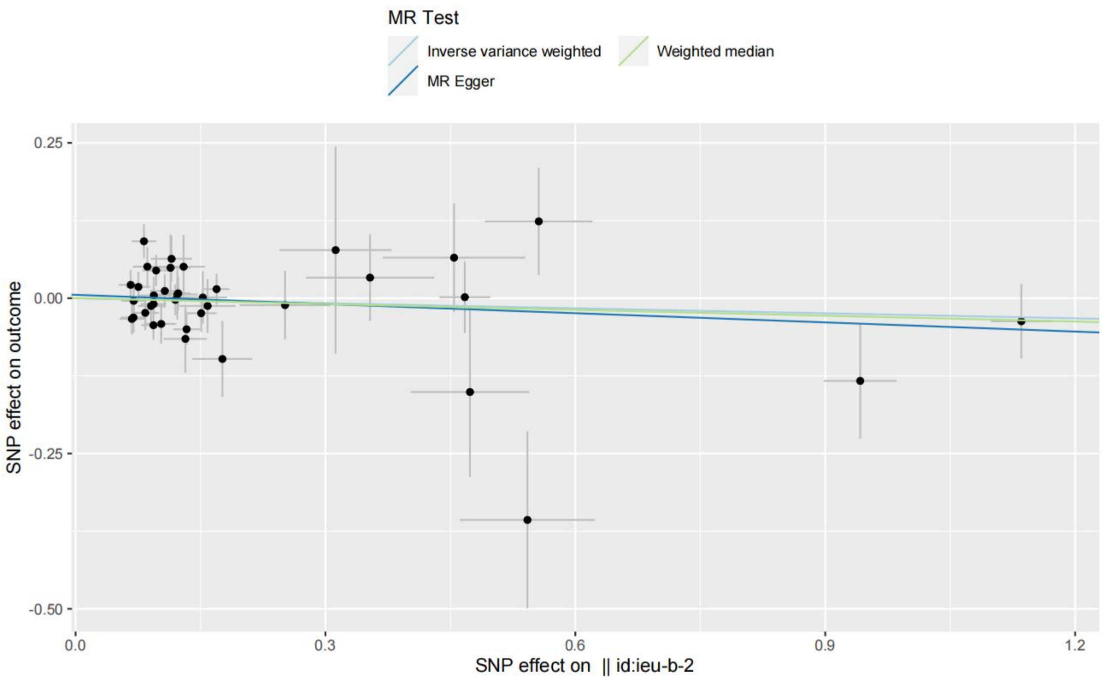

A. Scatter plot of AD on IL-16

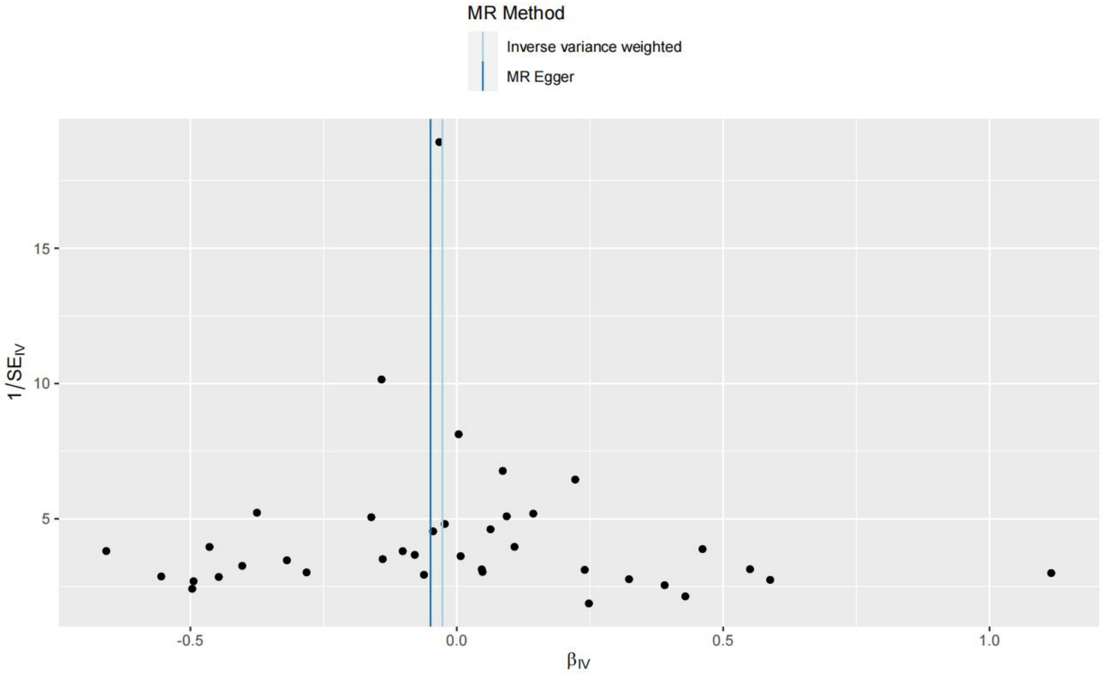

B. Funnel plot of AD on IL-16

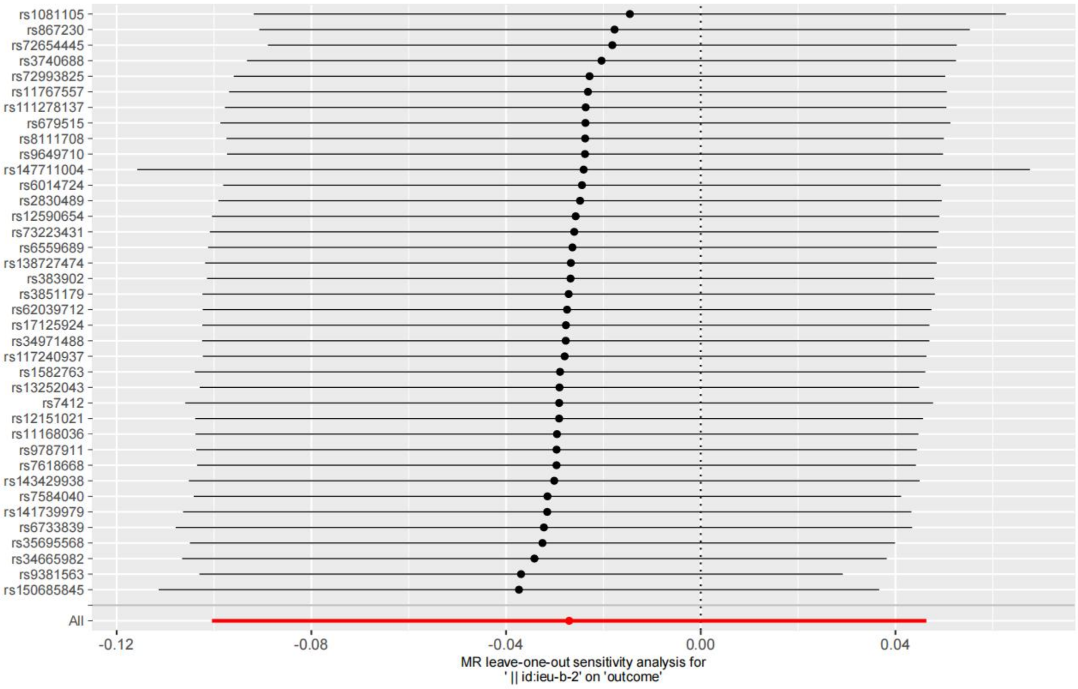

C. MR leave-one-out sensitivity analysis for AD on IL-16

eFigure 146. AD-associated SNPs with IL-17

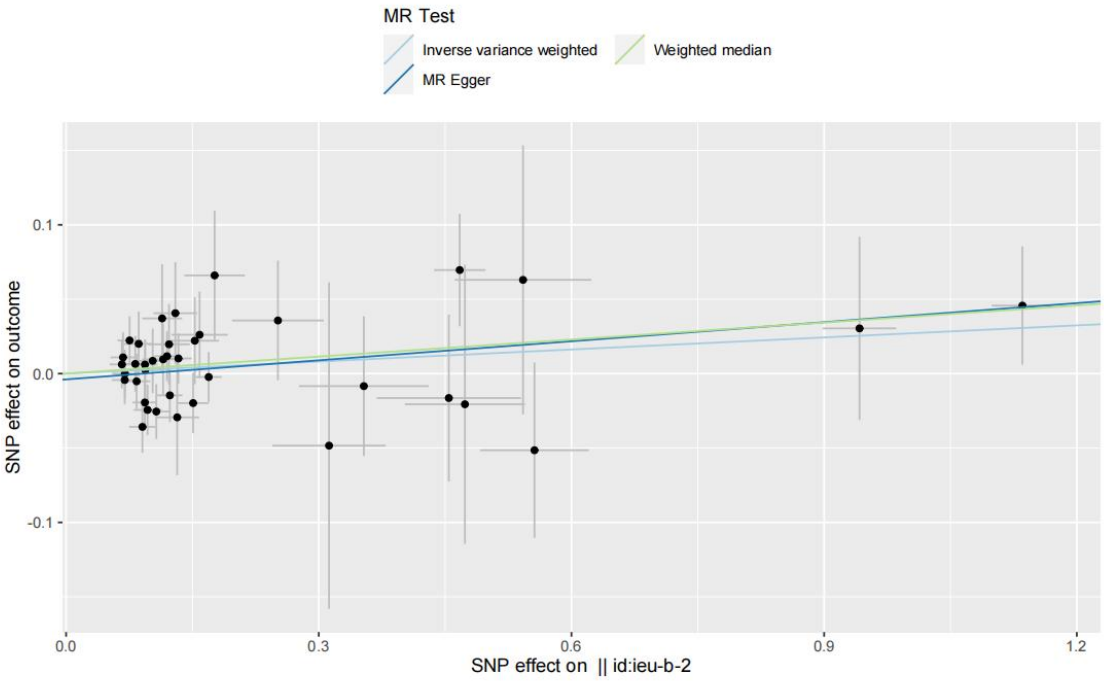

A. Scatter plot of AD on IL-17

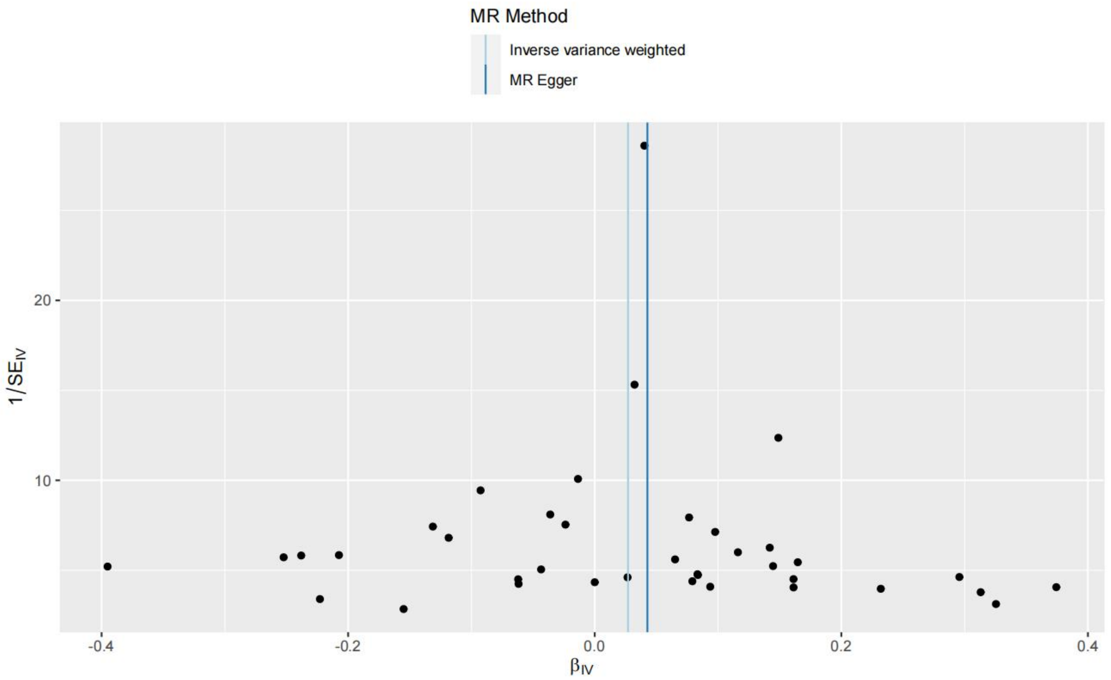

B. Funnel plot of AD on IL-17

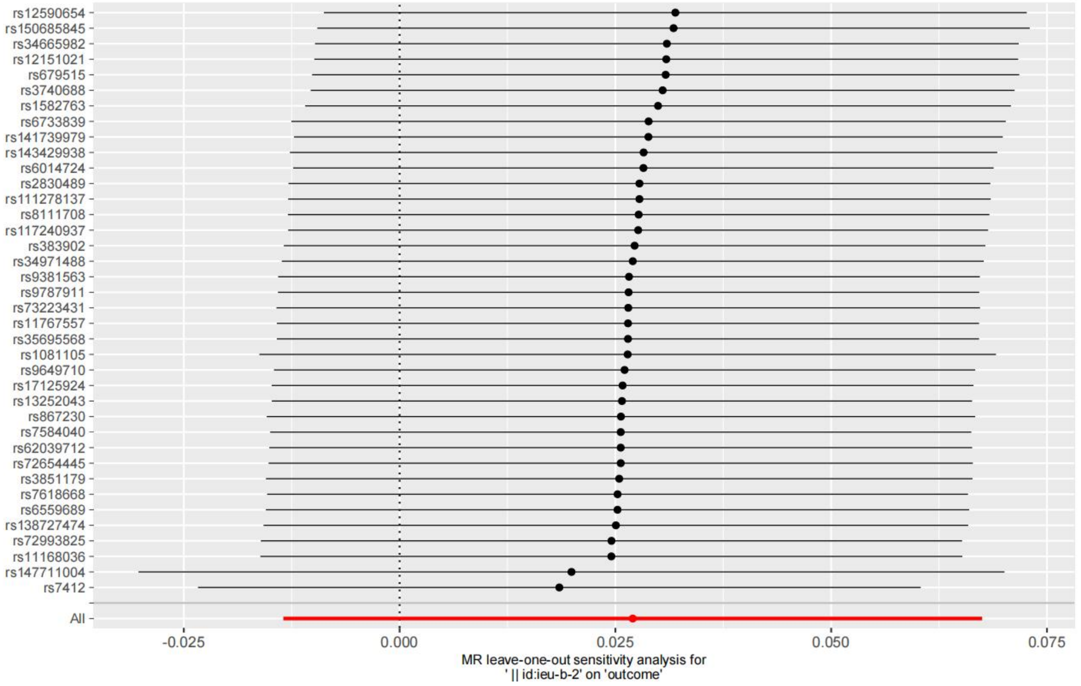

C. MR leave-one-out sensitivity analysis for AD on IL-17

eFigure 147. AD-associated SNPs with IL-18

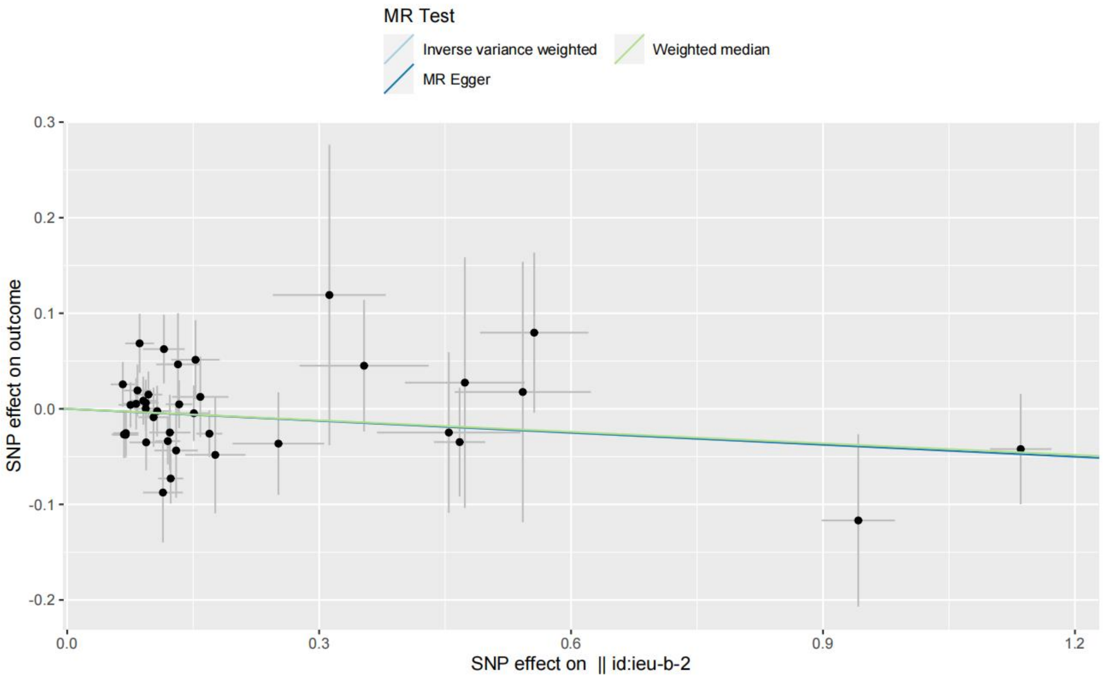

A. Scatter plot of AD on IL-18

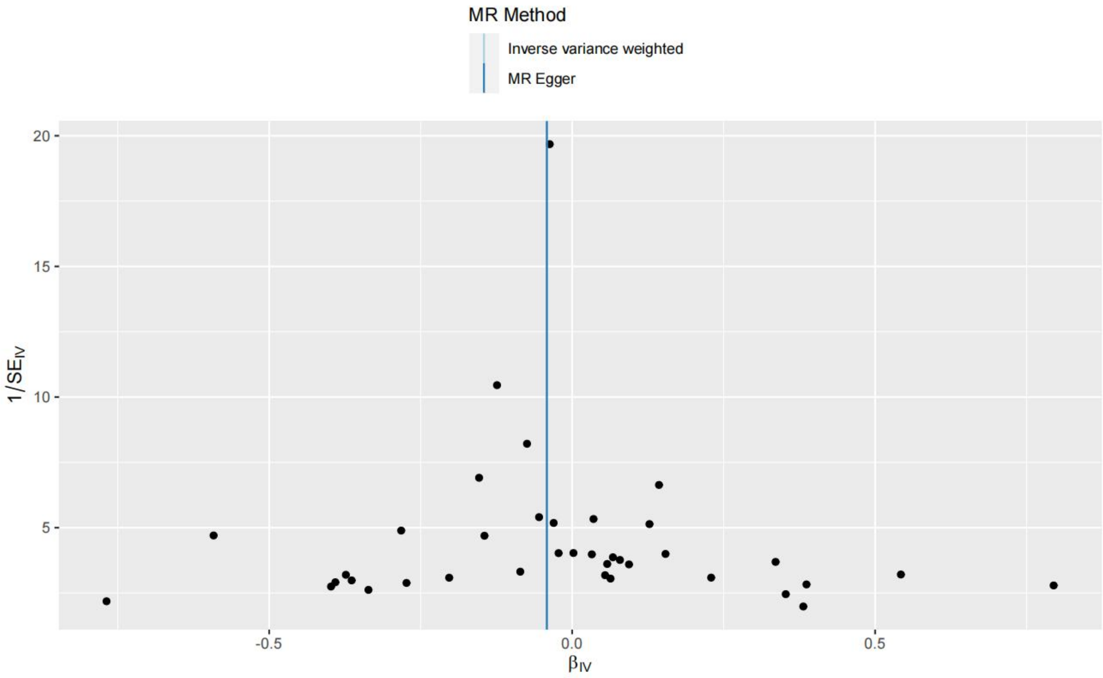

B. Funnel plot of AD on IL-18

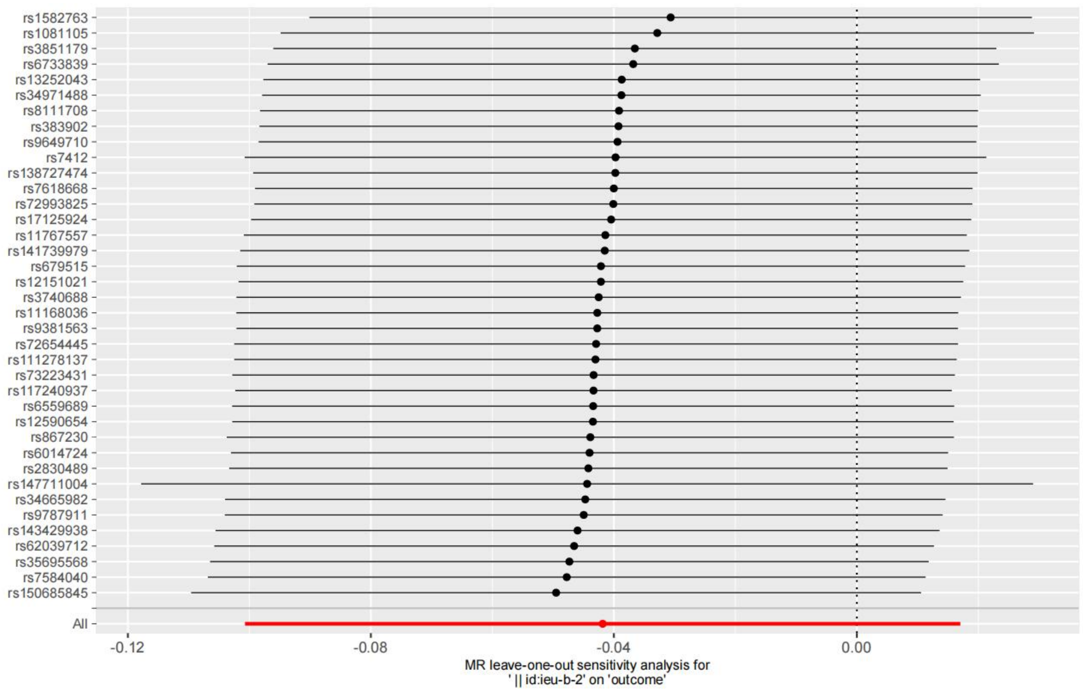

C. MR leave-one-out sensitivity analysis for AD on IL-18

eFigure 148. AD-associated SNPs with IP-10

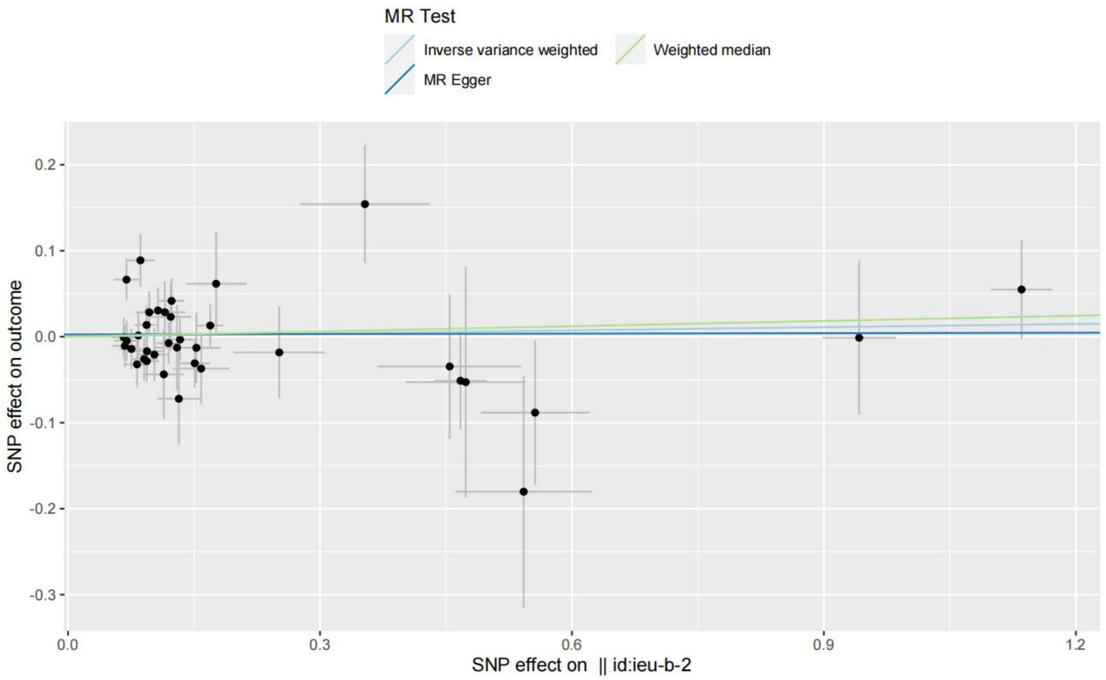

A. Scatter plot of AD on IP-10

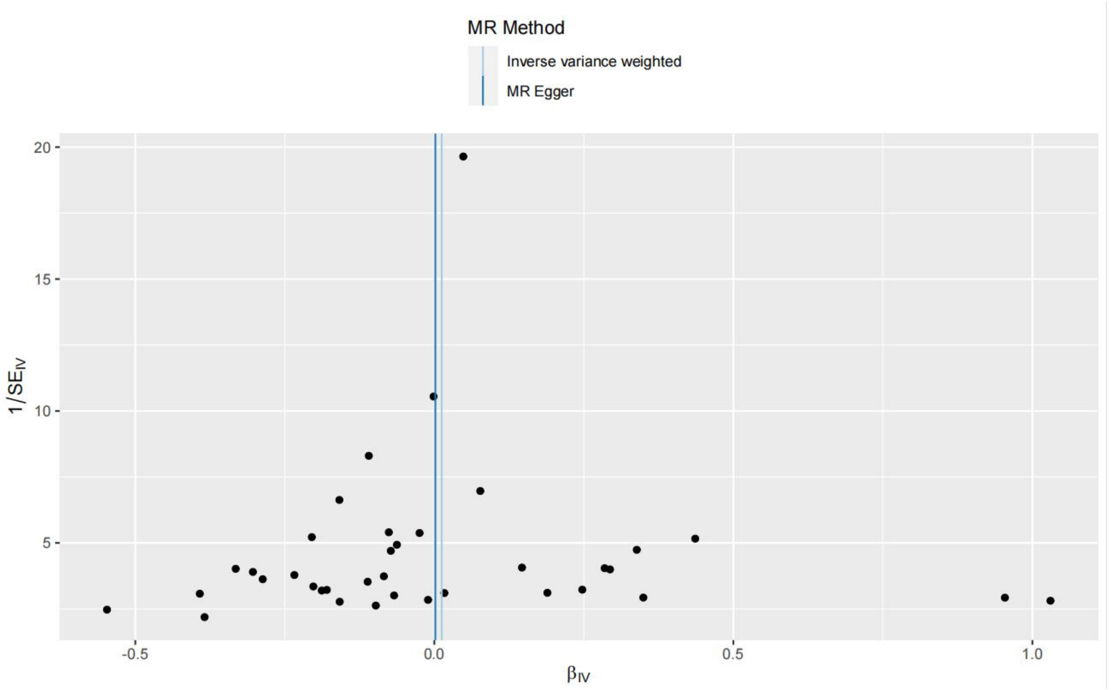

B. Funnel plot of AD on IP-10

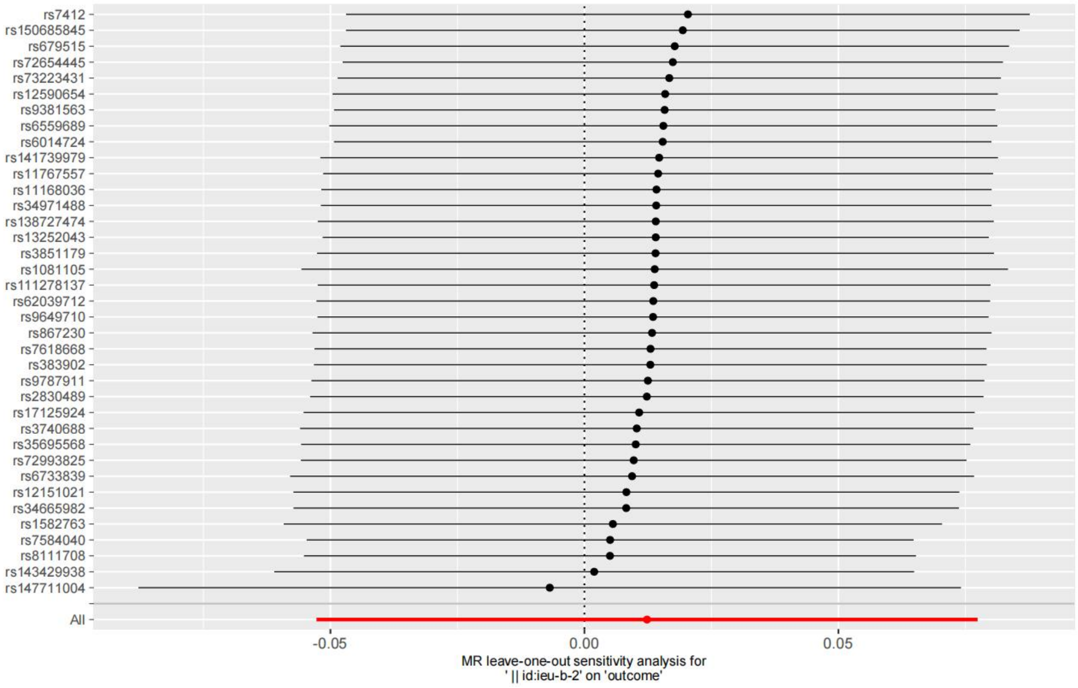

C. MR leave-one-out sensitivity analysis for AD on IP-10

eFigure 149. AD-associated SNPs with M-CSF

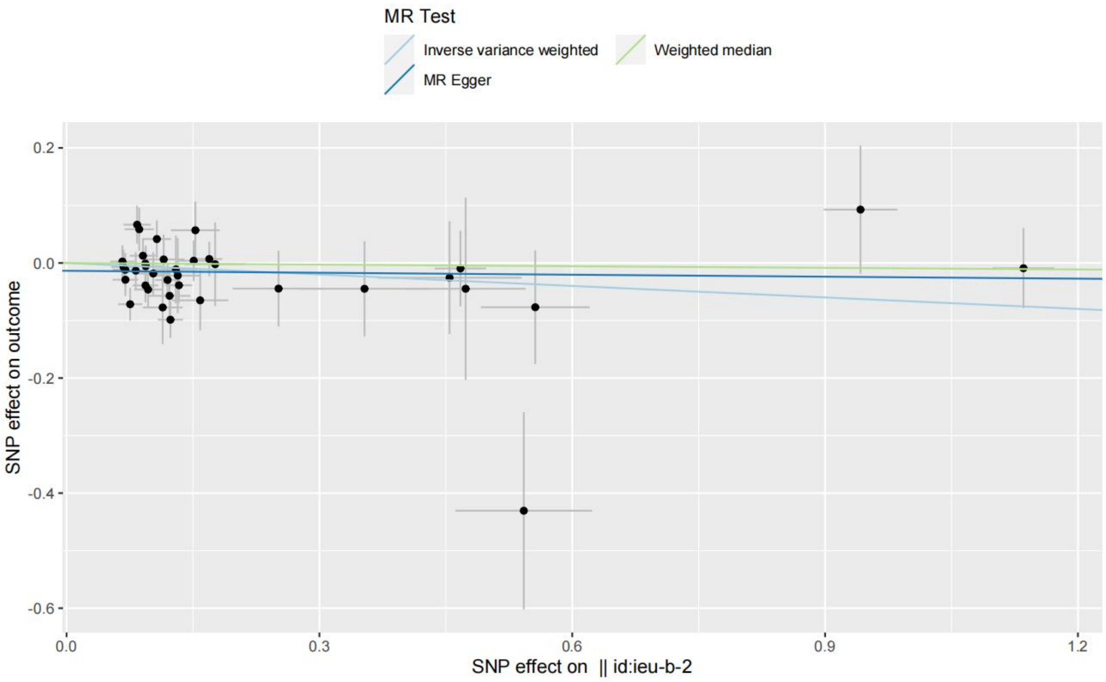

A. Scatter plot of AD on M-CSF

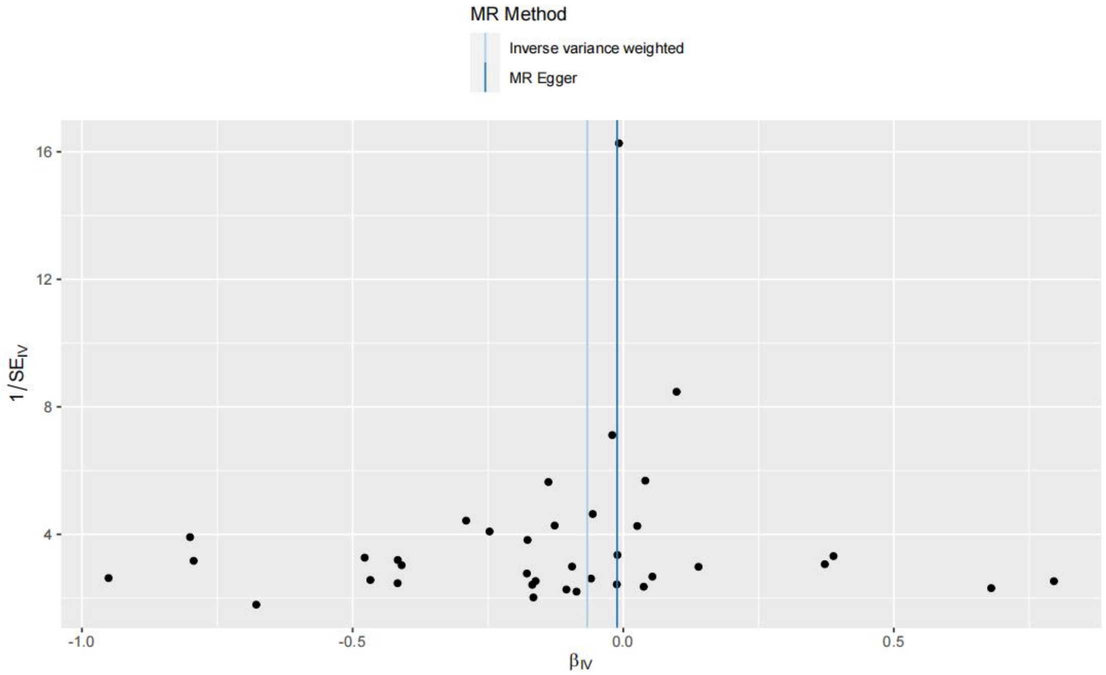

B. Funnel plot of AD on M-CSF

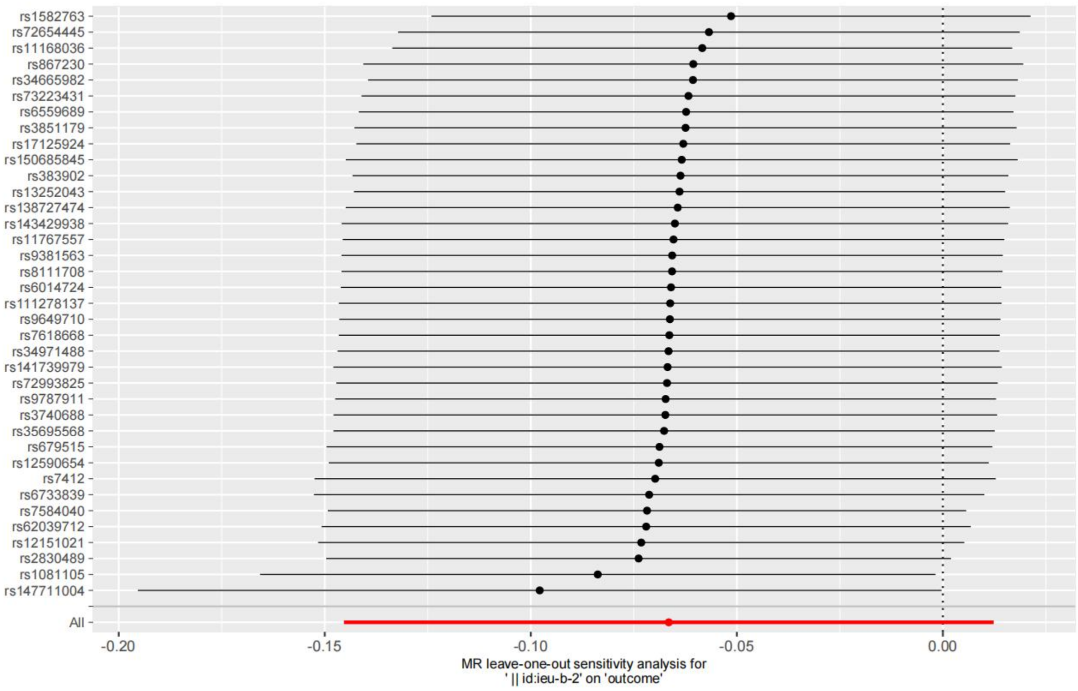

C. MR leave-one-out sensitivity analysis for AD on M-CSF

eFigure 150. AD-associated SNPs with MCP-1-MCAF

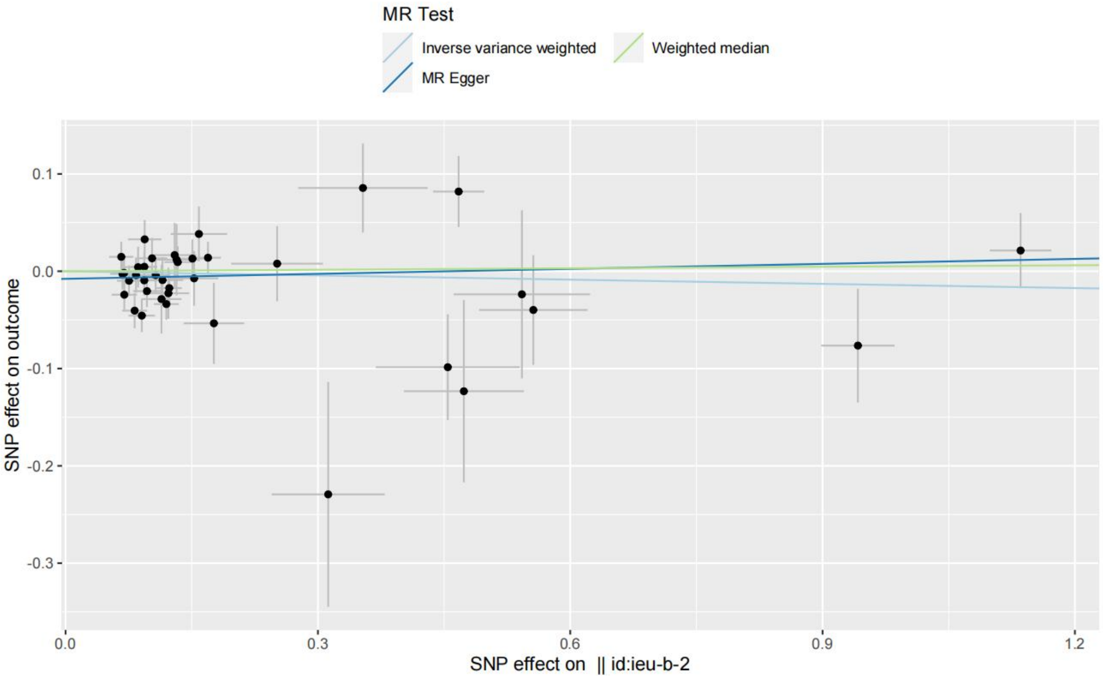

A. Scatter plot of AD on MCP-1-MCAF

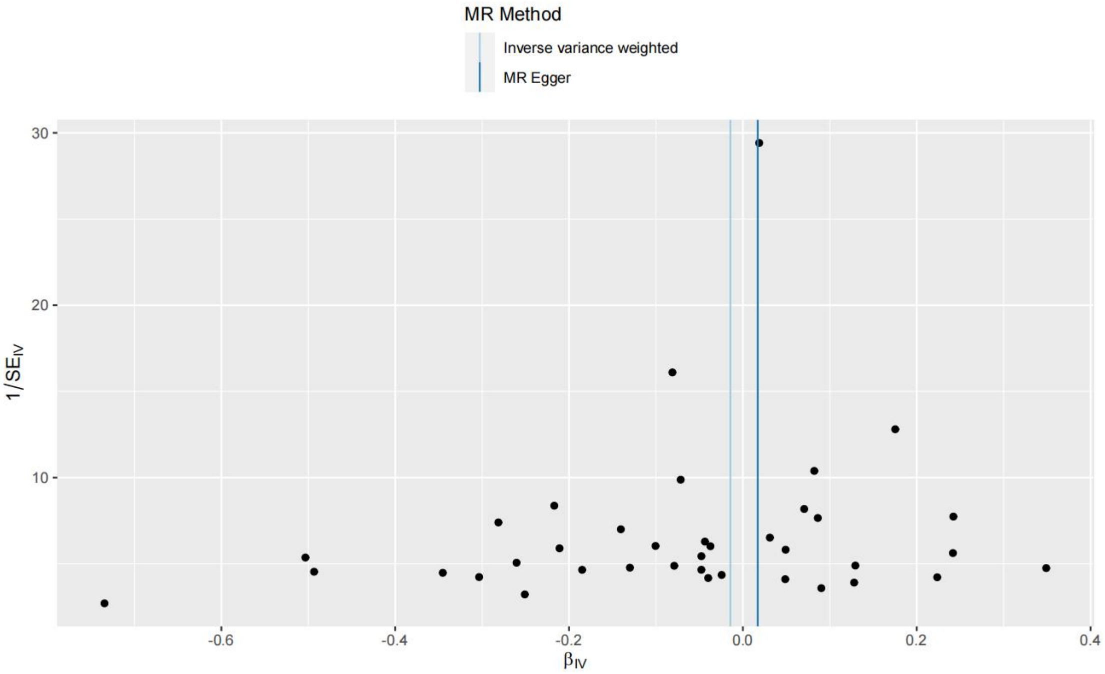

B. Funnel plot of AD on MCP-1-MCAF

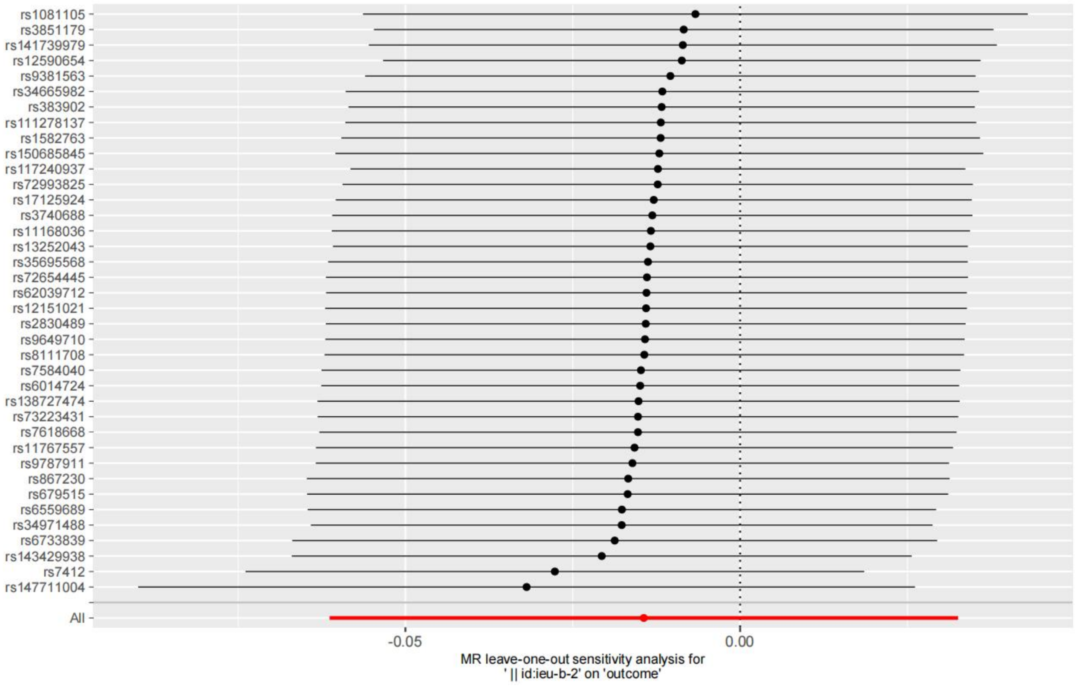

C. MR leave-one-out sensitivity analysis for AD on MCP-1-MCAF

eFigure 151. AD-associated SNPs with MCP-3

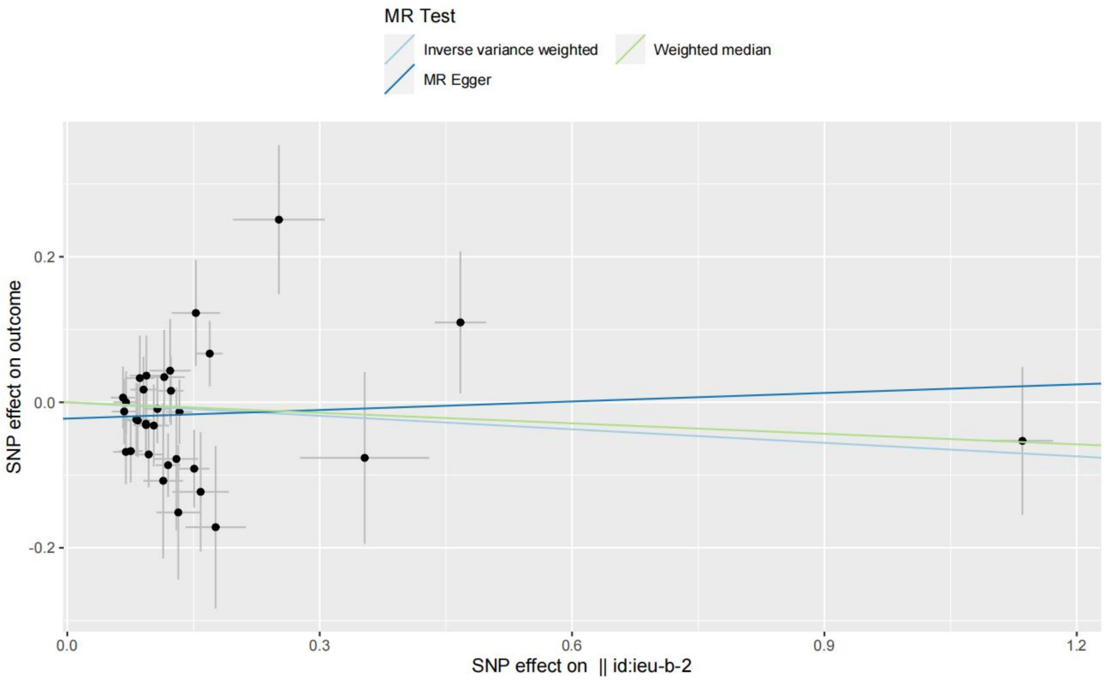

A. Scatter plot of AD on MCP-3

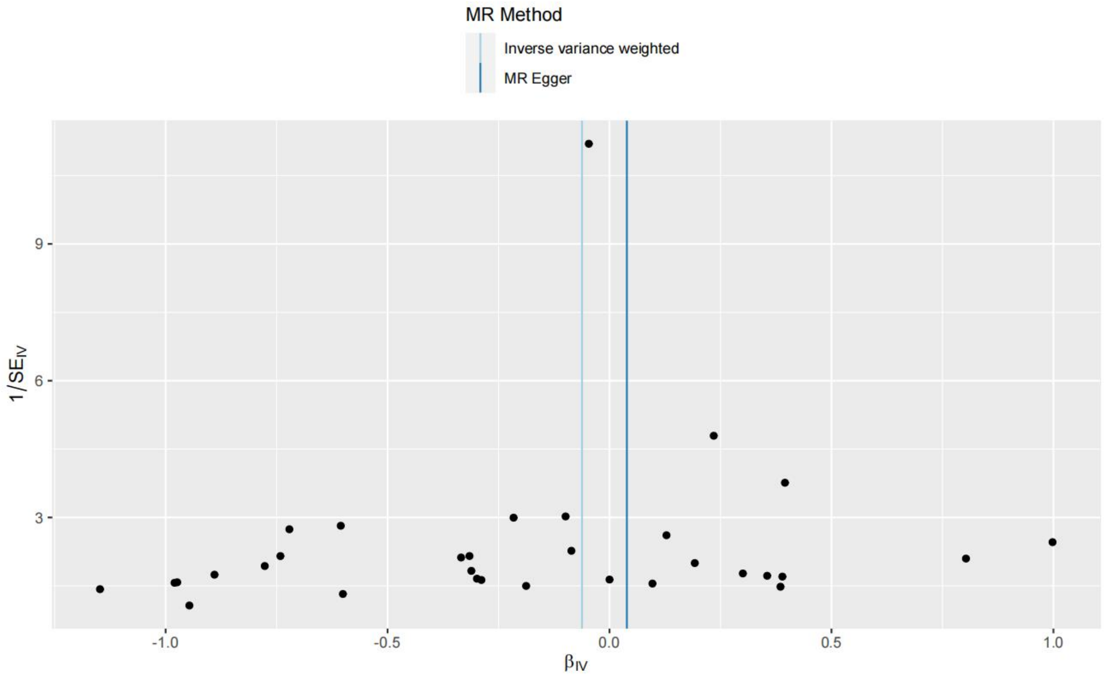

B. Funnel plot of AD on MCP-3

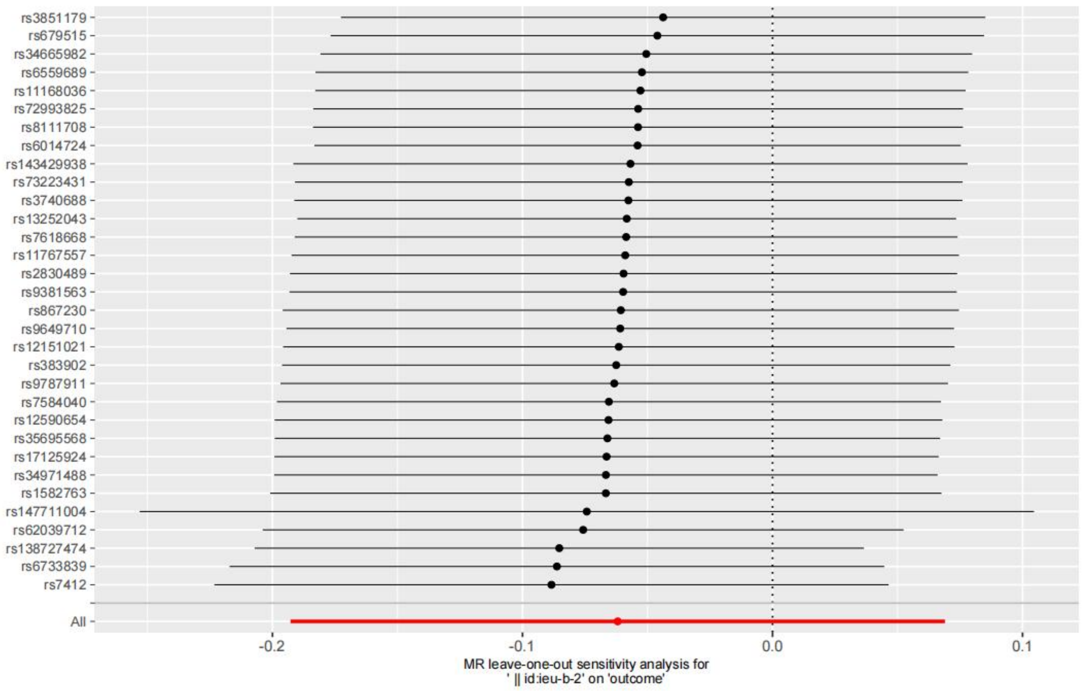

C. MR leave-one-out sensitivity analysis for AD on MCP-3

eFigure 152. AD-associated SNPs with MIF

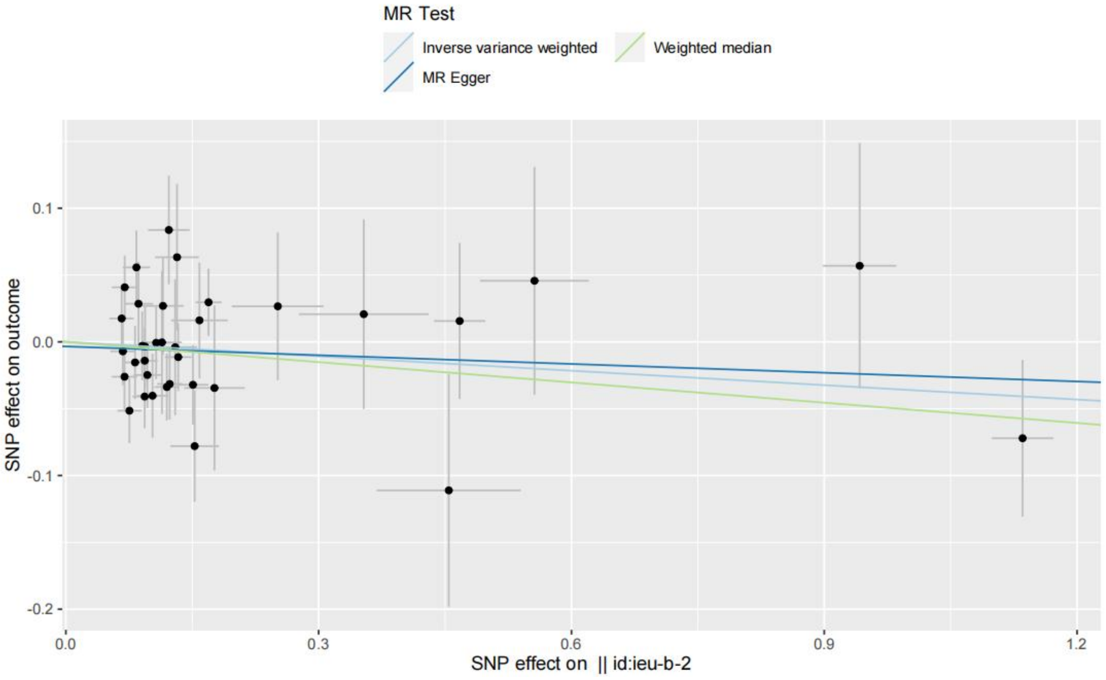

A. Scatter plot of AD on MIF

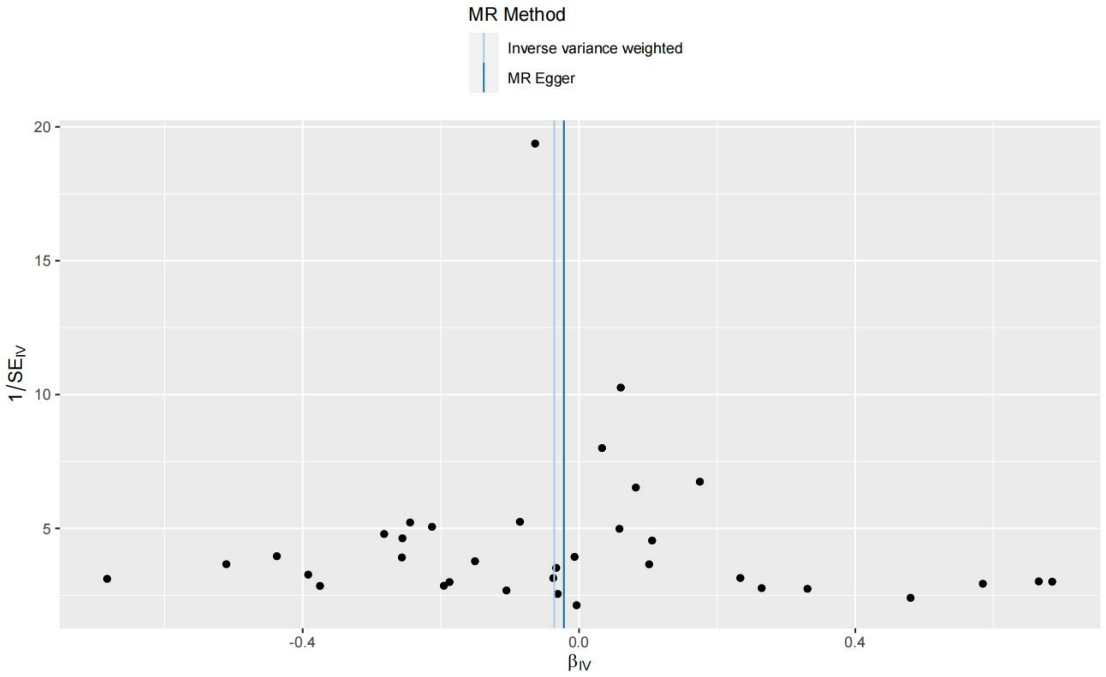

B. Funnel plot of AD on MIF

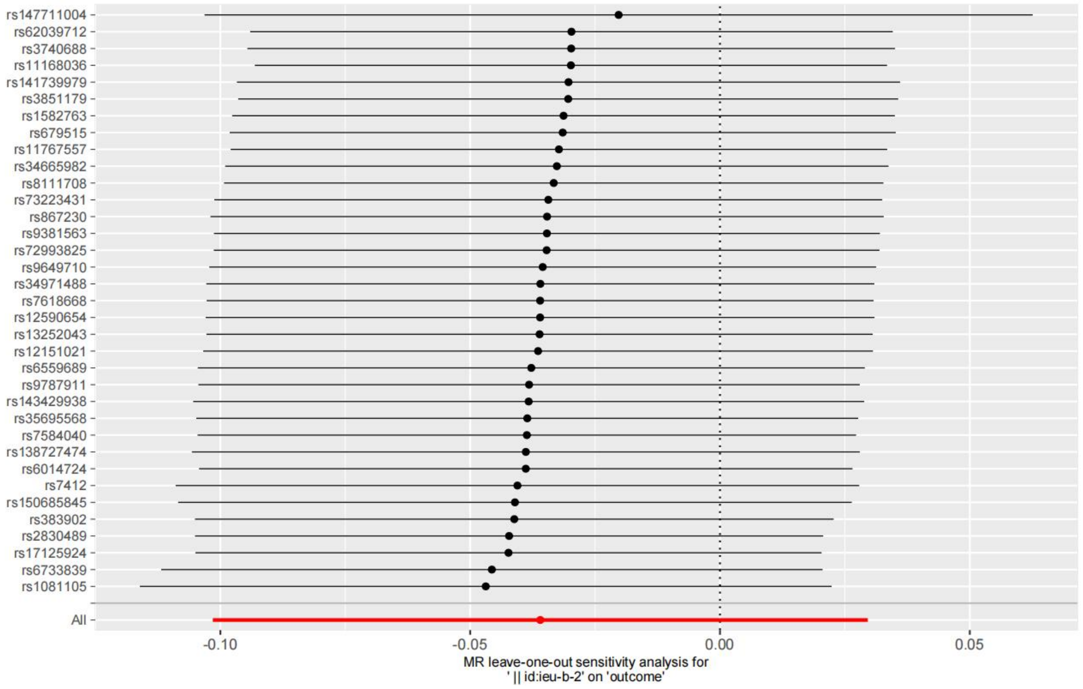

C. MR leave-one-out sensitivity analysis for AD on MIF

eFigure 153. AD-associated SNPs with MIG

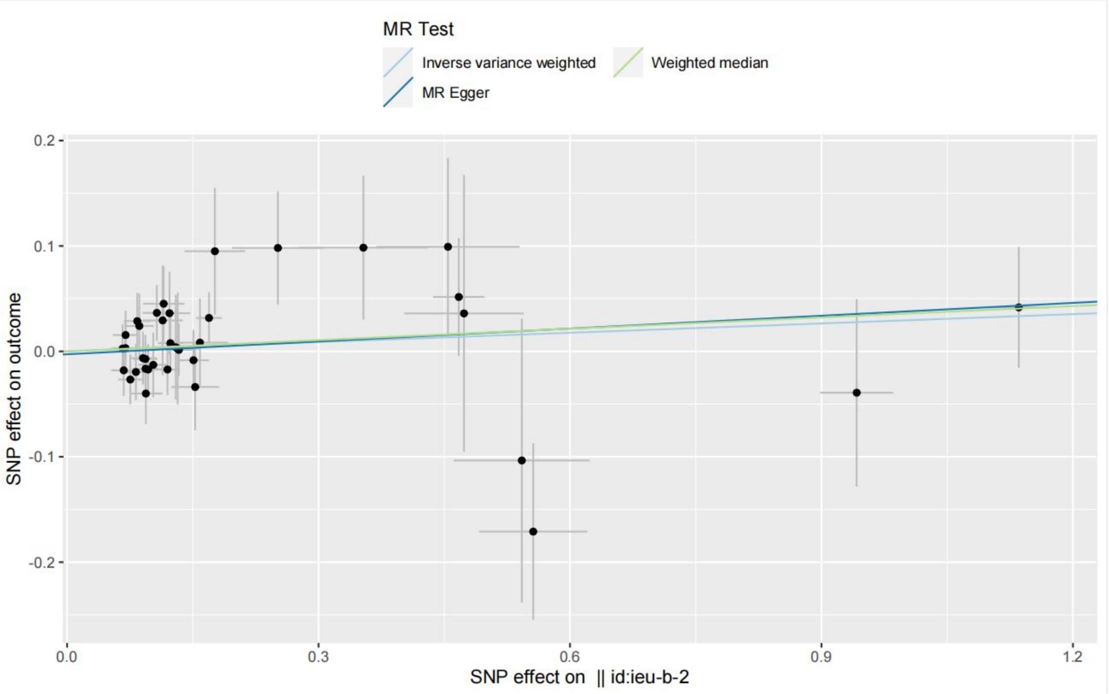

A. Scatter plot of AD on MIG

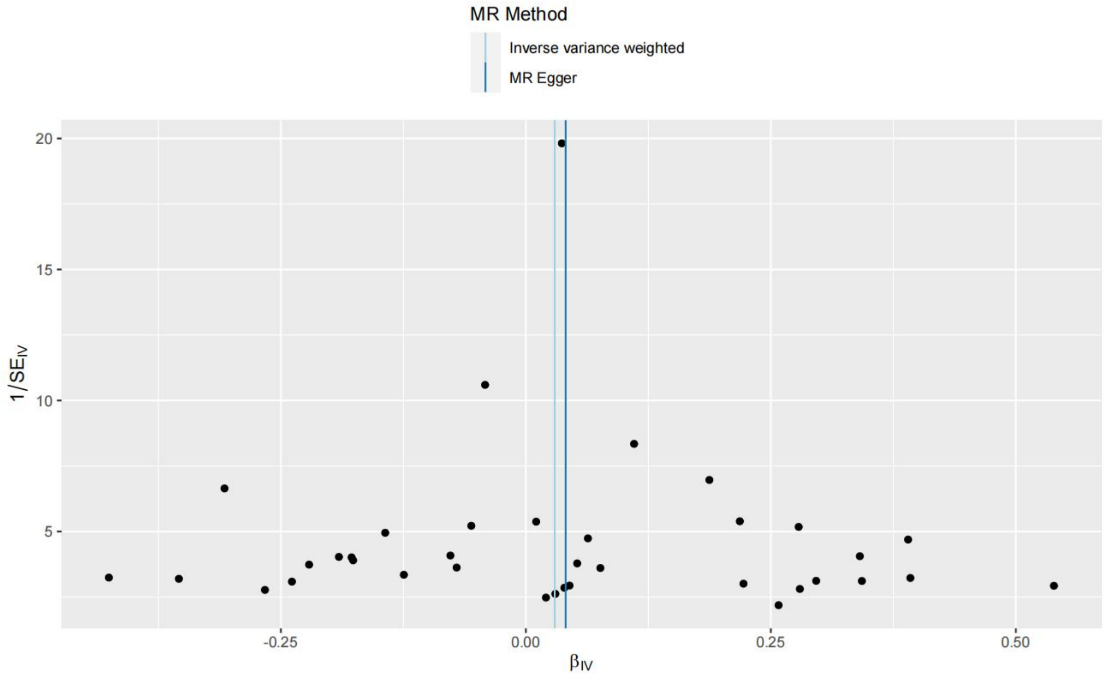

B. Funnel plot of AD on MIG

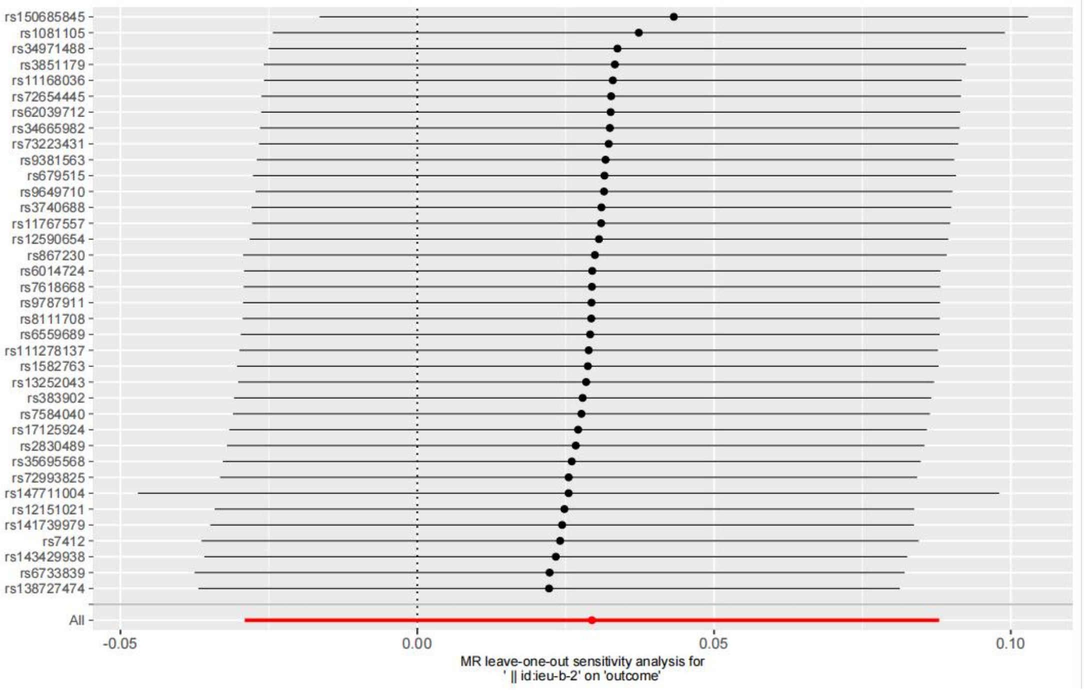

C. MR leave-one-out sensitivity analysis for AD on MIG

eFigure 154. AD-associated SNPs with MIP-1A

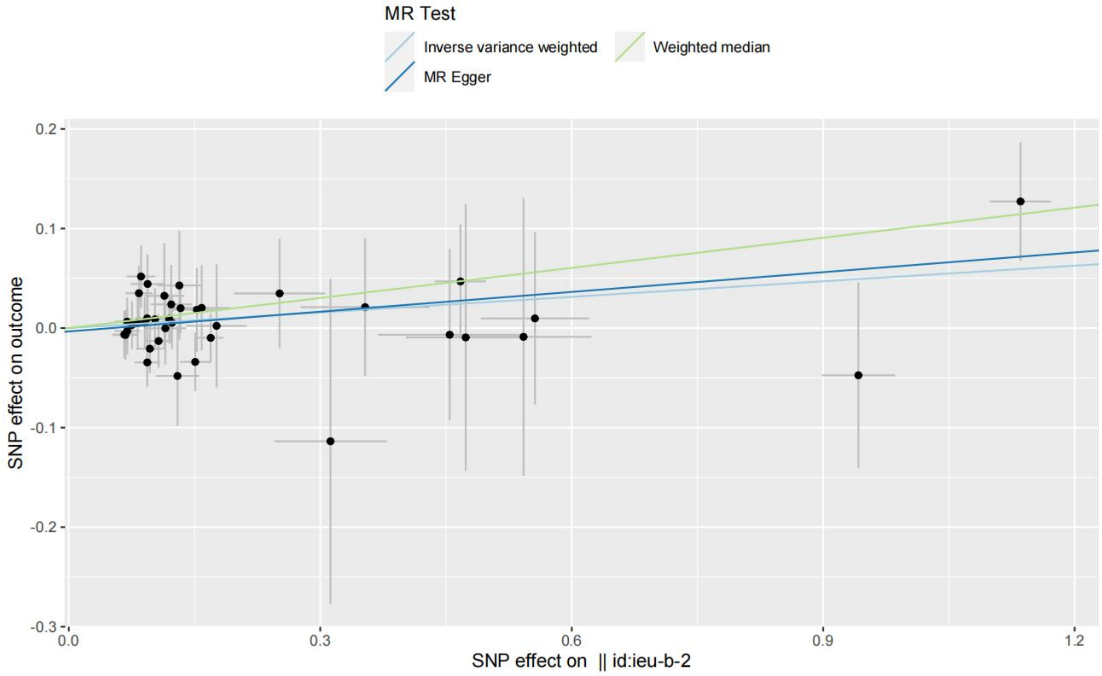

A. Scatter plot of AD on MIP-1A

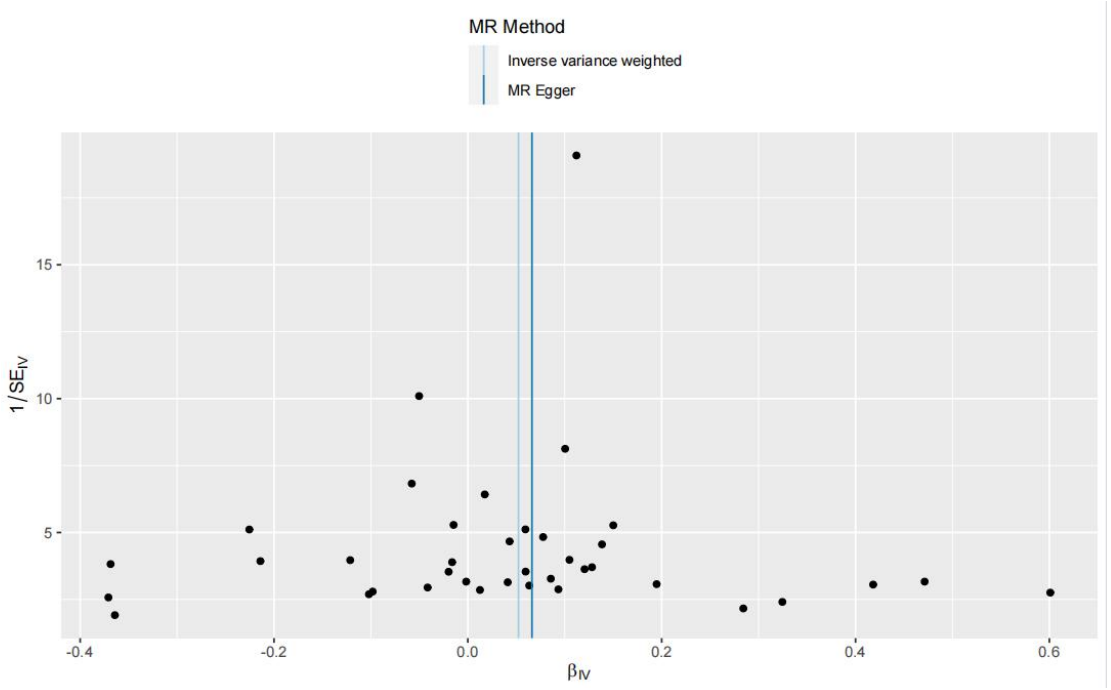

B. Funnel plot of AD on MIP-1A

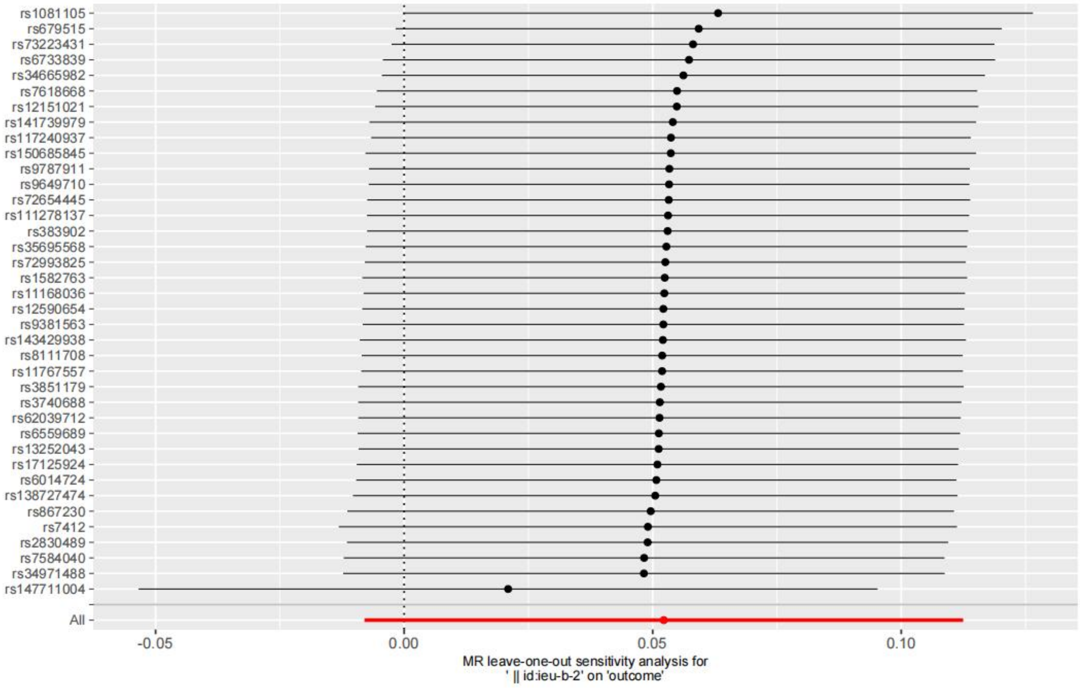

C. MR leave-one-out sensitivity analysis for AD on MIP-1A

eFigure 155. AD-associated SNPs with MIP-1B

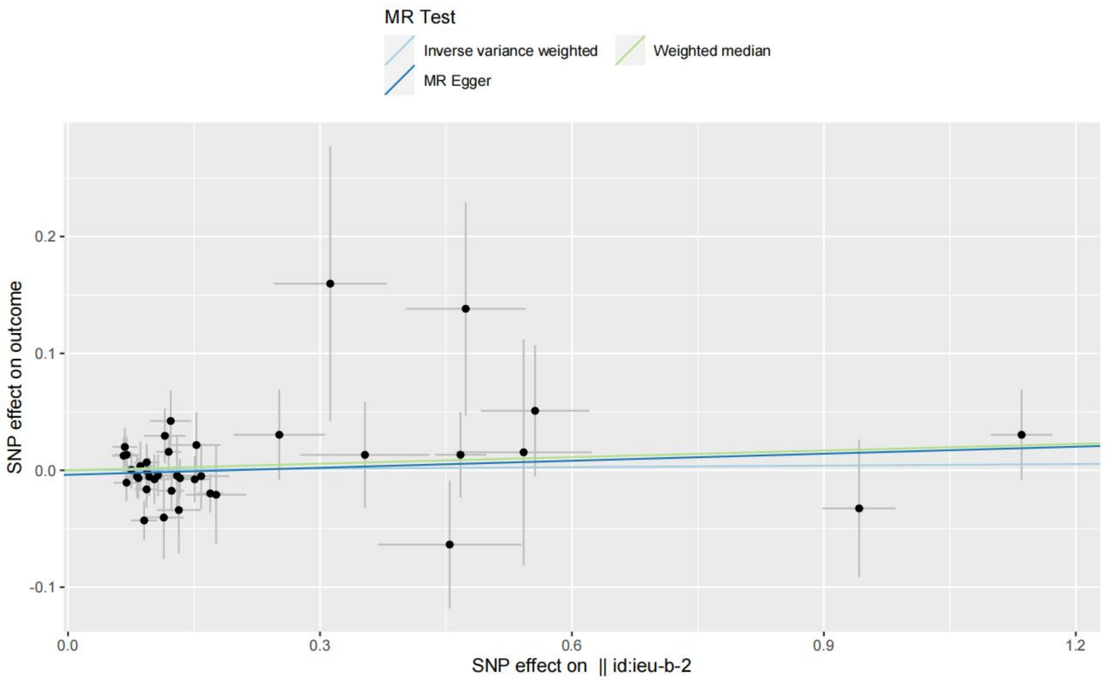

A. Scatter plot of AD on MIP-1B

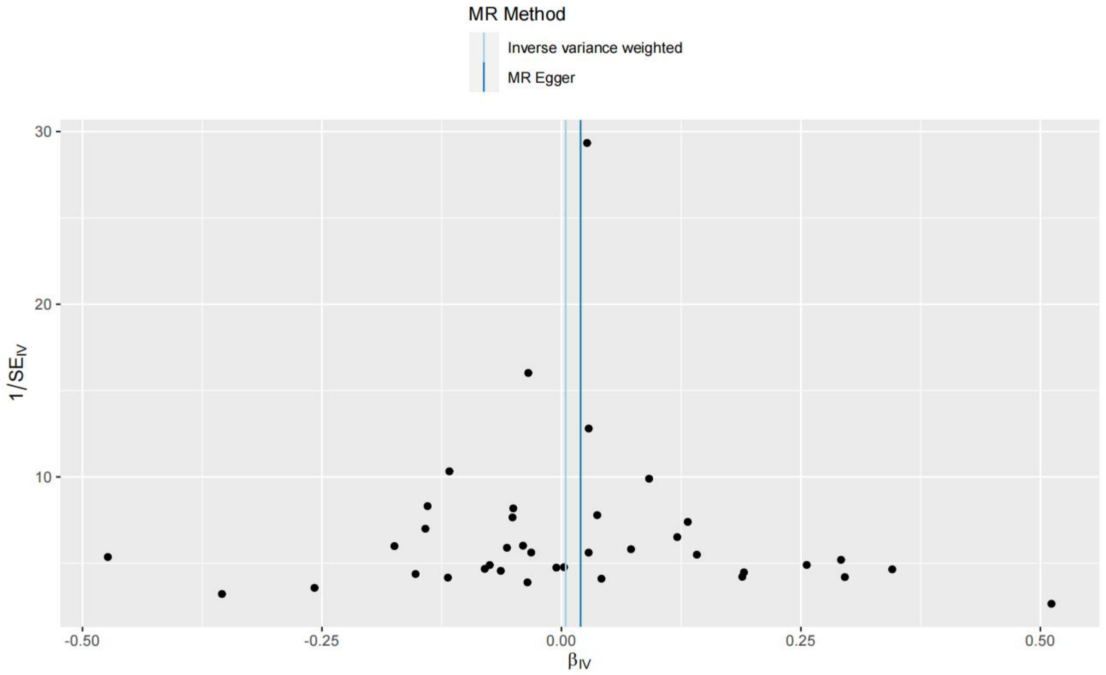

B. Funnel plot of AD on MIP-1B

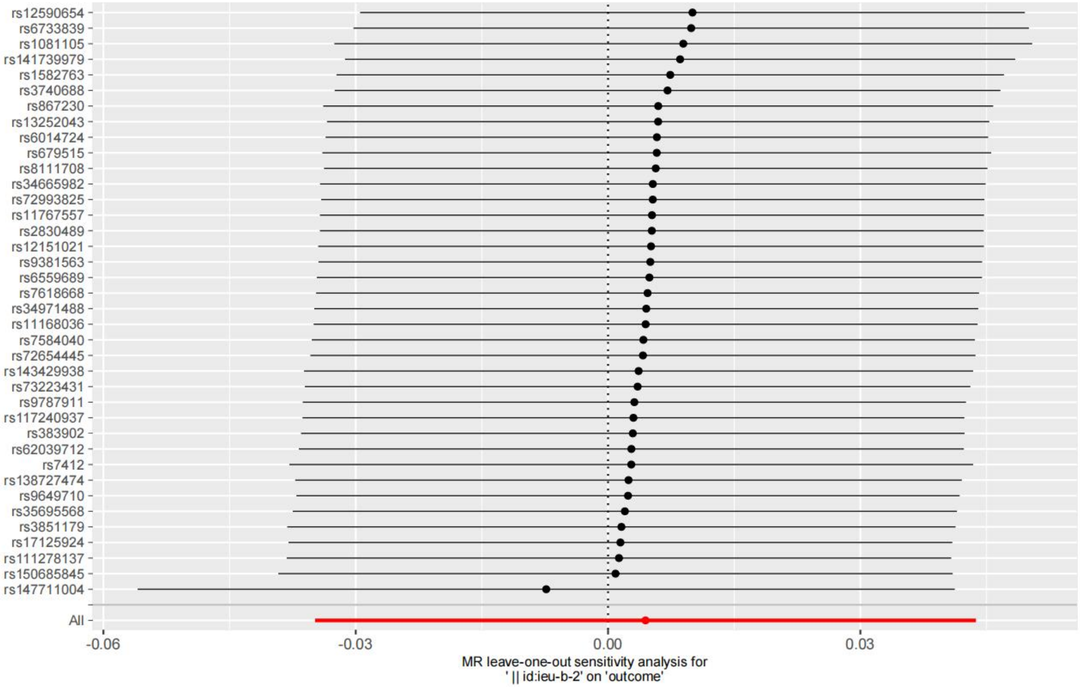

C. MR leave-one-out sensitivity analysis for AD on MIP-1B

eFigure 156. AD-associated SNPs with PDGF-BB

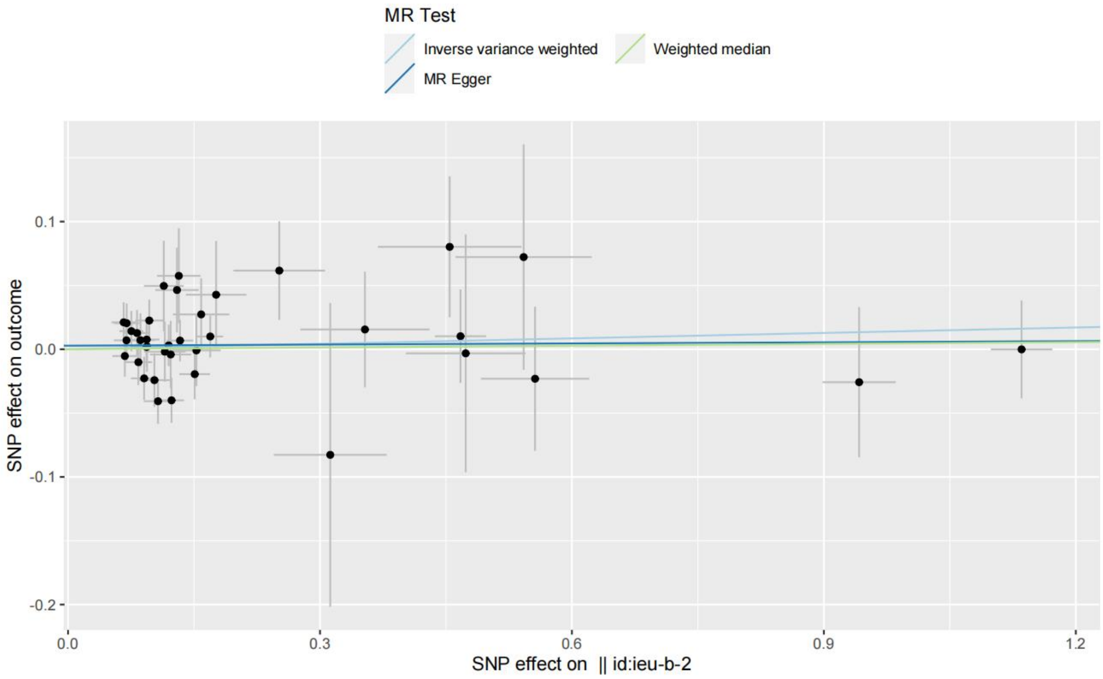

A. Scatter plot of AD on PDGF-BB

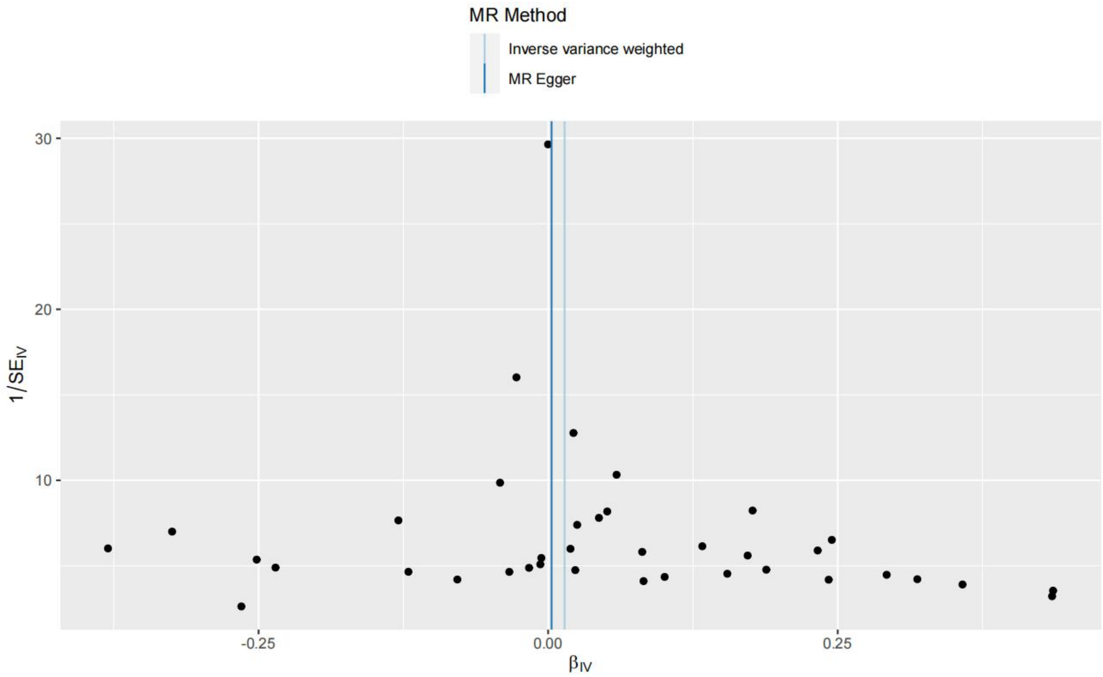

B. Funnel plot of AD on PDGF-BB

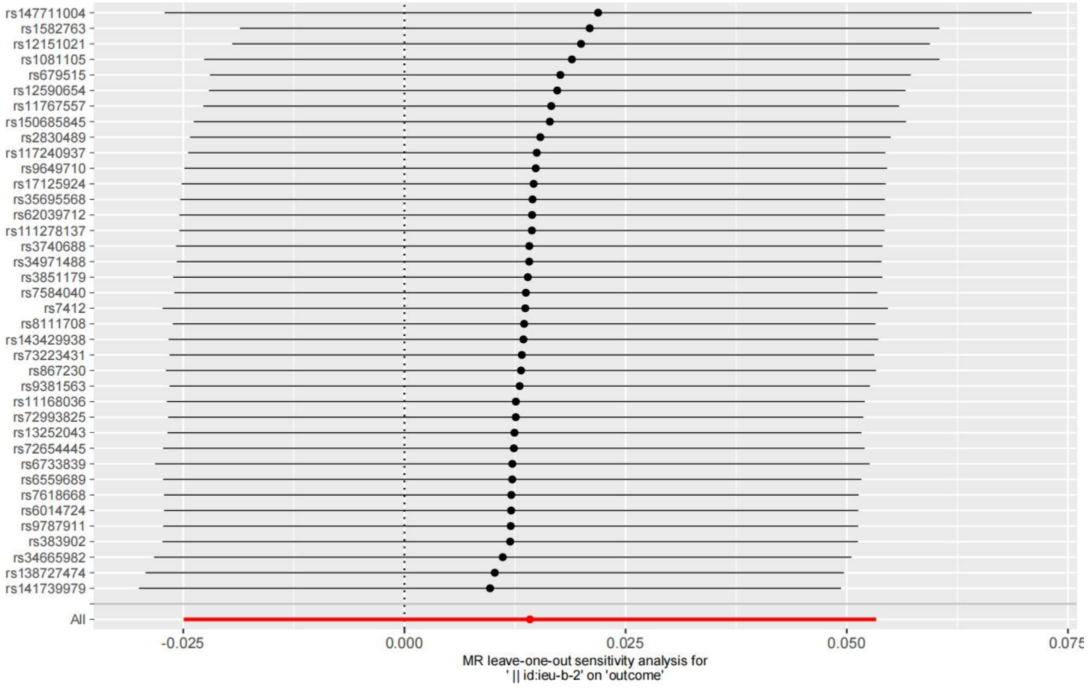

C. MR leave-one-out sensitivity analysis for AD on PDGF-BB

eFigure 157. AD-associated SNPs with RANTES

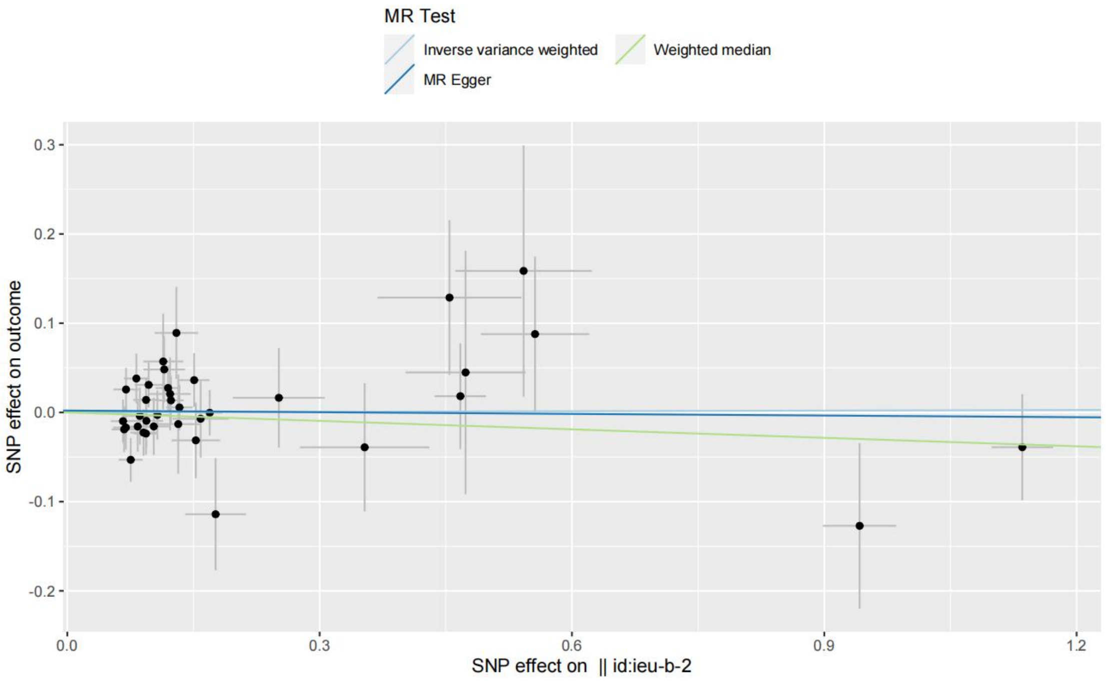

A. Scatter plot of AD on RANTES

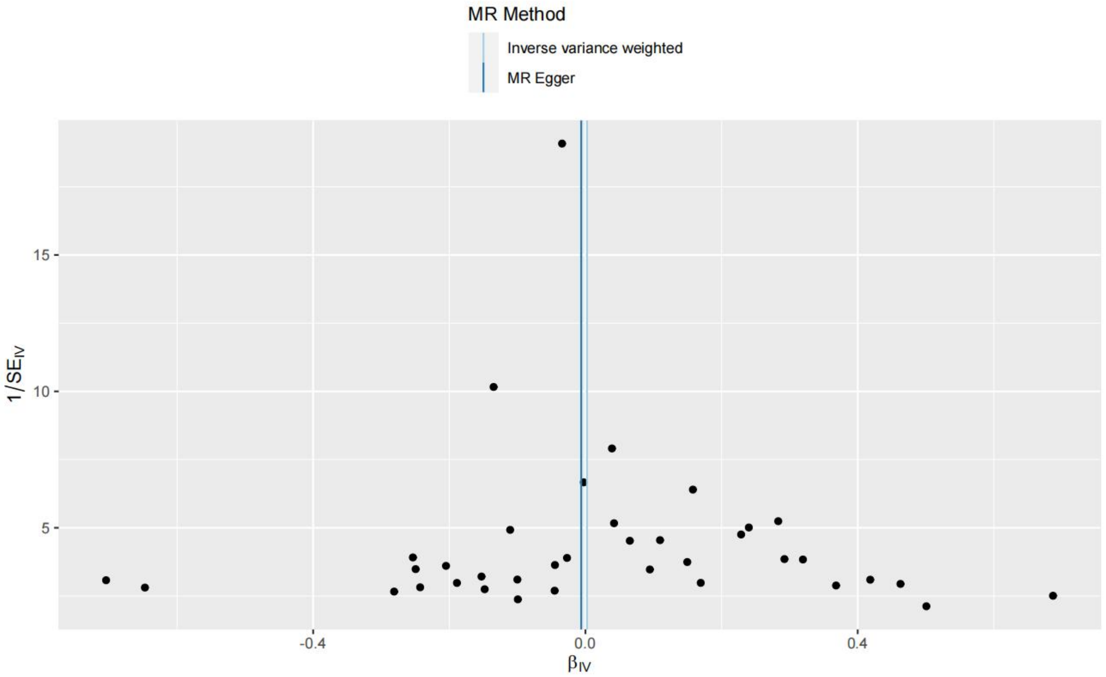

B. Funnel plot of AD on RANTES

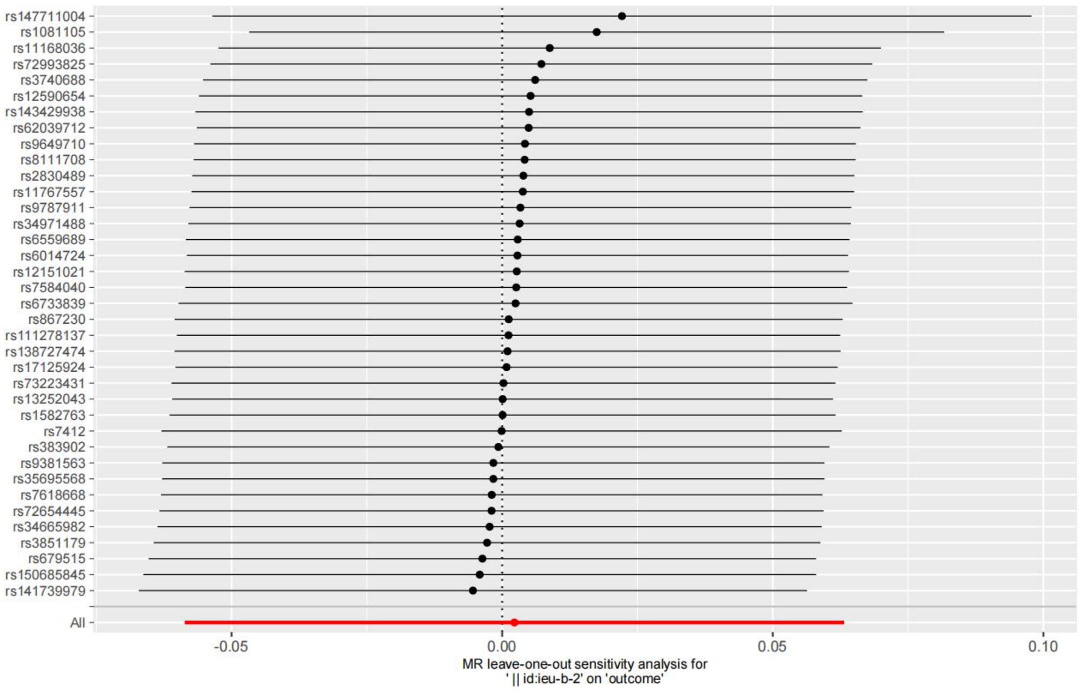

C. MR leave-one-out sensitivity analysis for AD on RANTES

eFigure 158. AD-associated SNPs with SCF

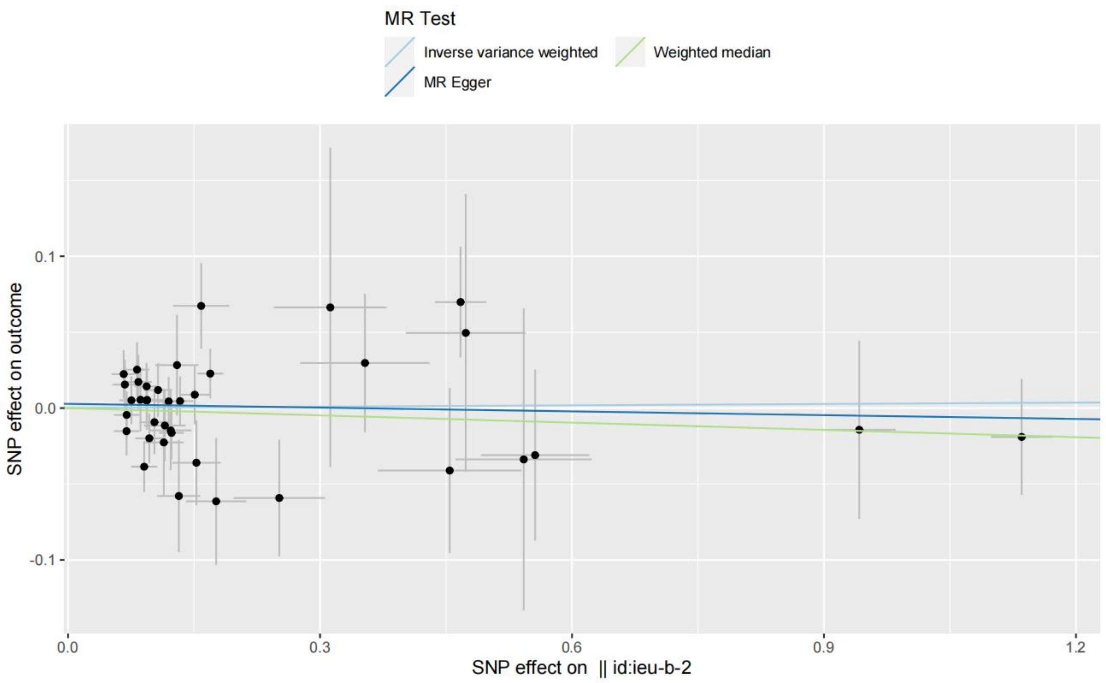

A. Scatter plot of AD on SCF

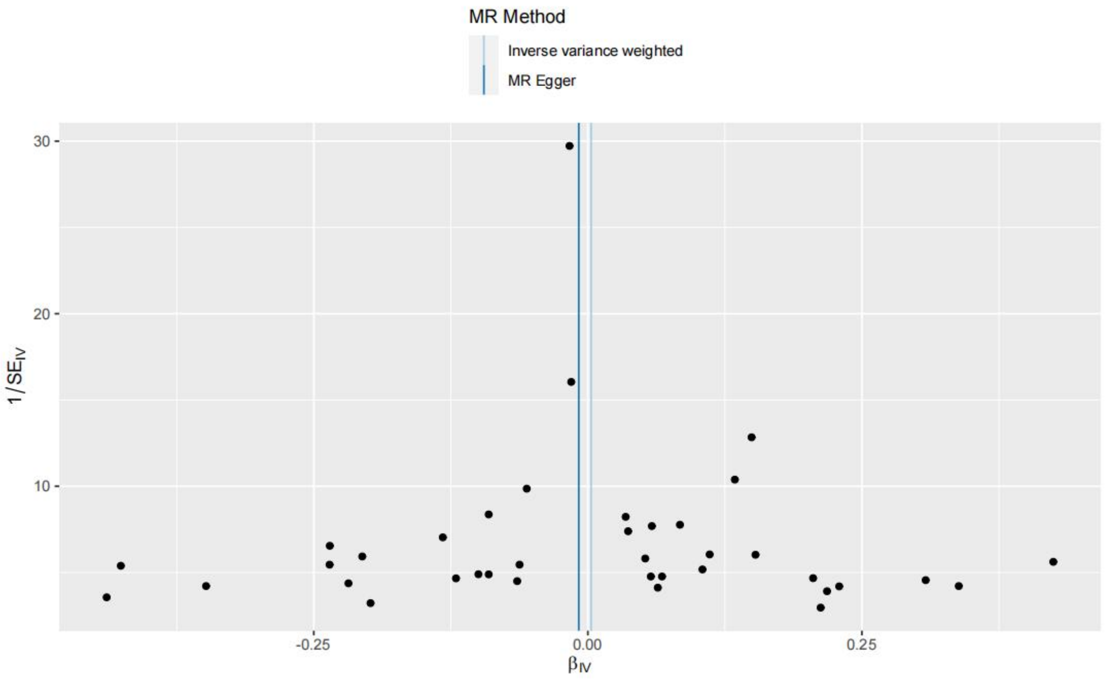

B. Funnel plot of AD on SCF

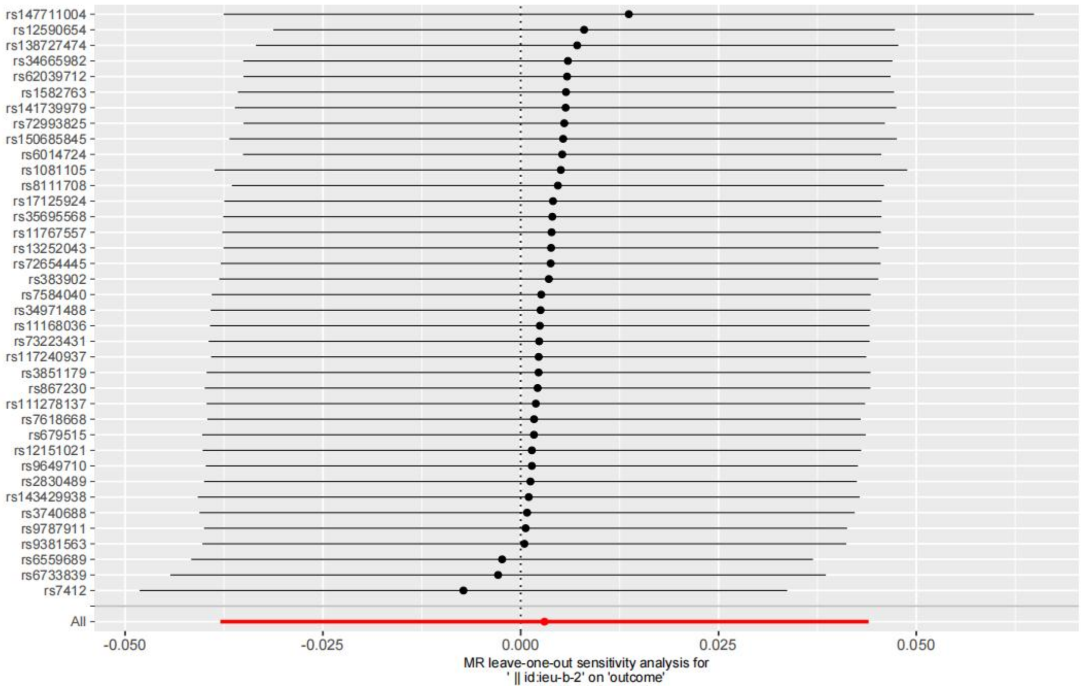

C. MR leave-one-out sensitivity analysis for AD on SCF

eFigure 159. AD-associated SNPs with SGCF

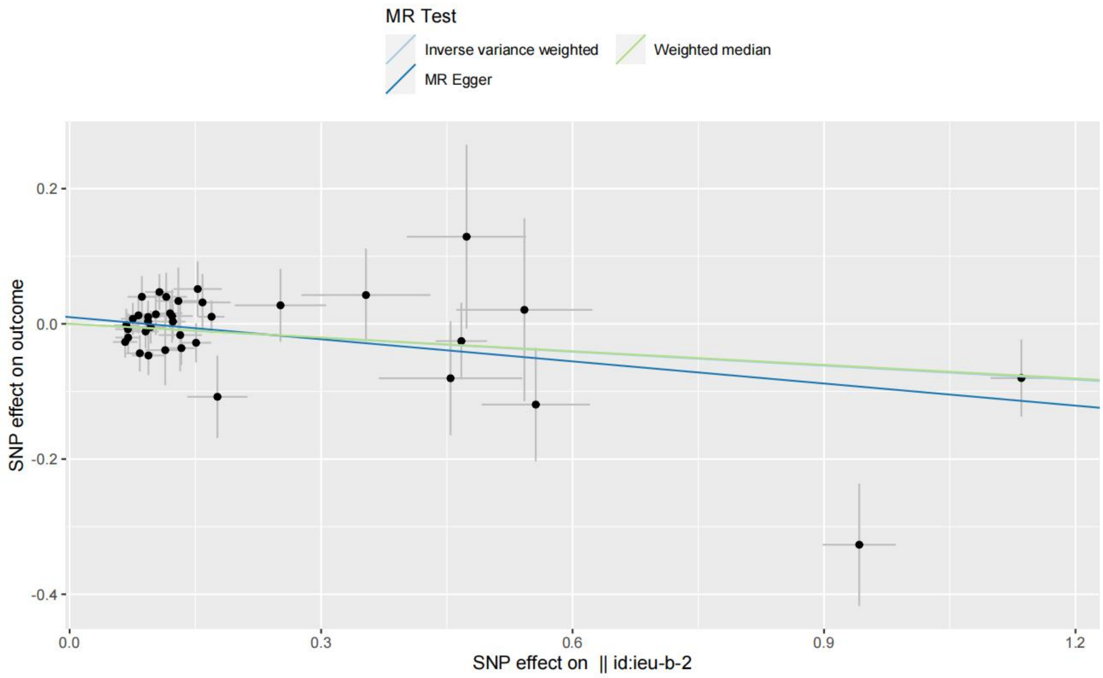

A. Scatter plot of AD on SGCF

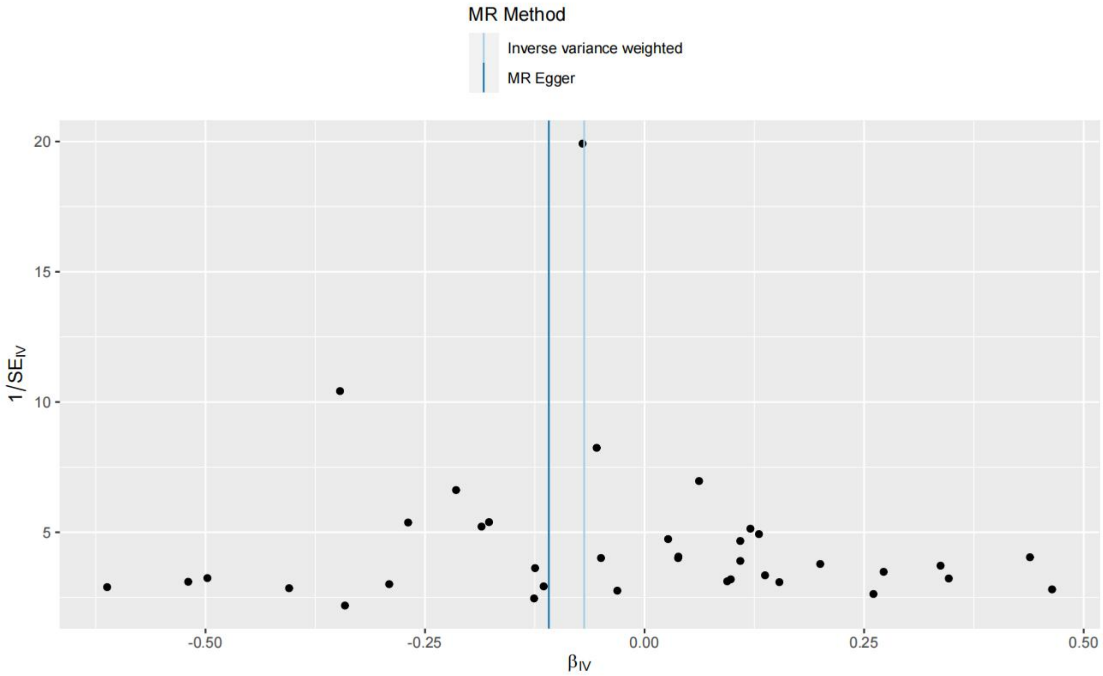

B. Funnel plot of AD on SGCF

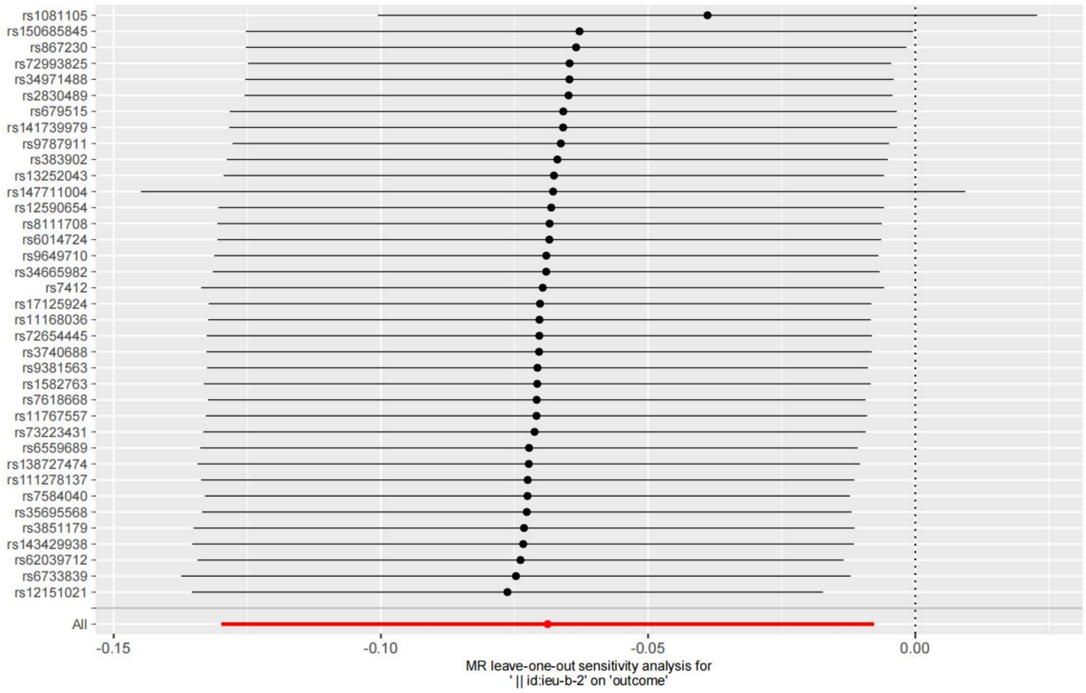

C. MR leave-one-out sensitivity analysis for AD on SGCF

eFigure 160. AD-associated SNPs with SDF-1A

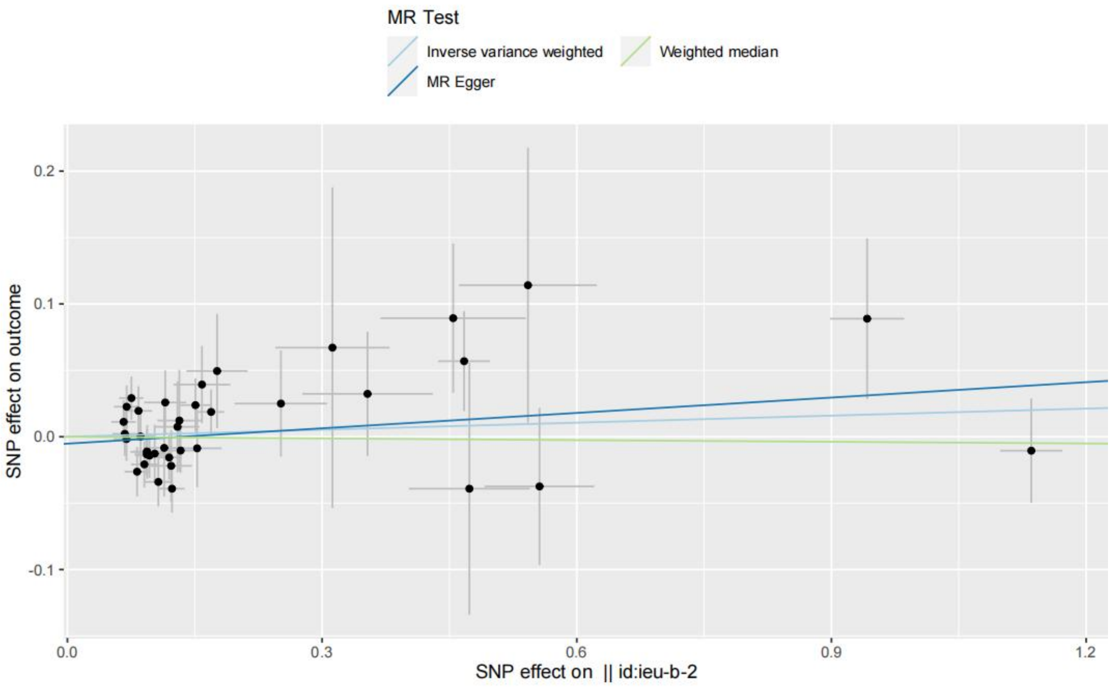

A. Scatter plot of AD on SDF-1A

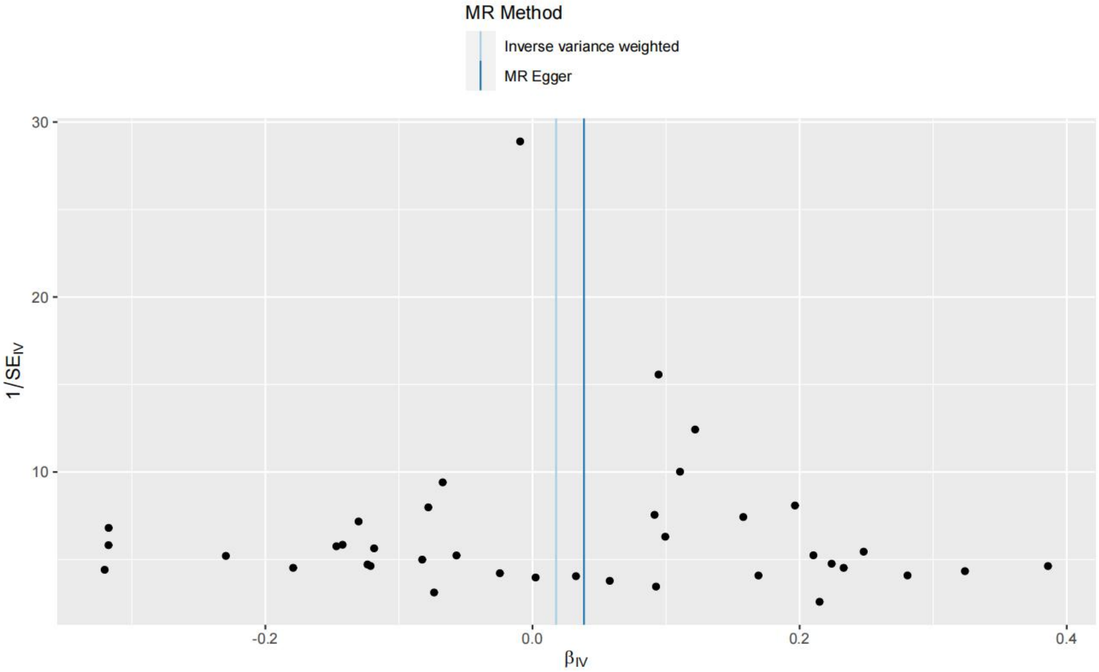

B. Funnel plot of AD on SDF-1A

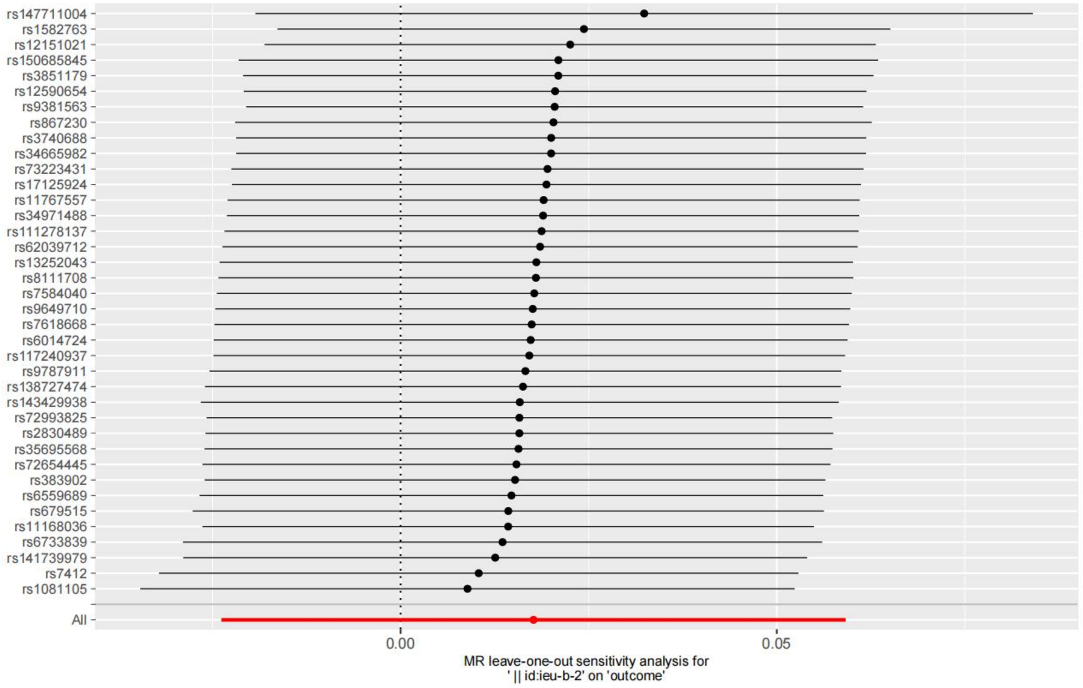

C. MR leave-one-out sensitivity analysis for AD on SDF-1A

eFigure 161. AD-associated SNPs with TNF-A

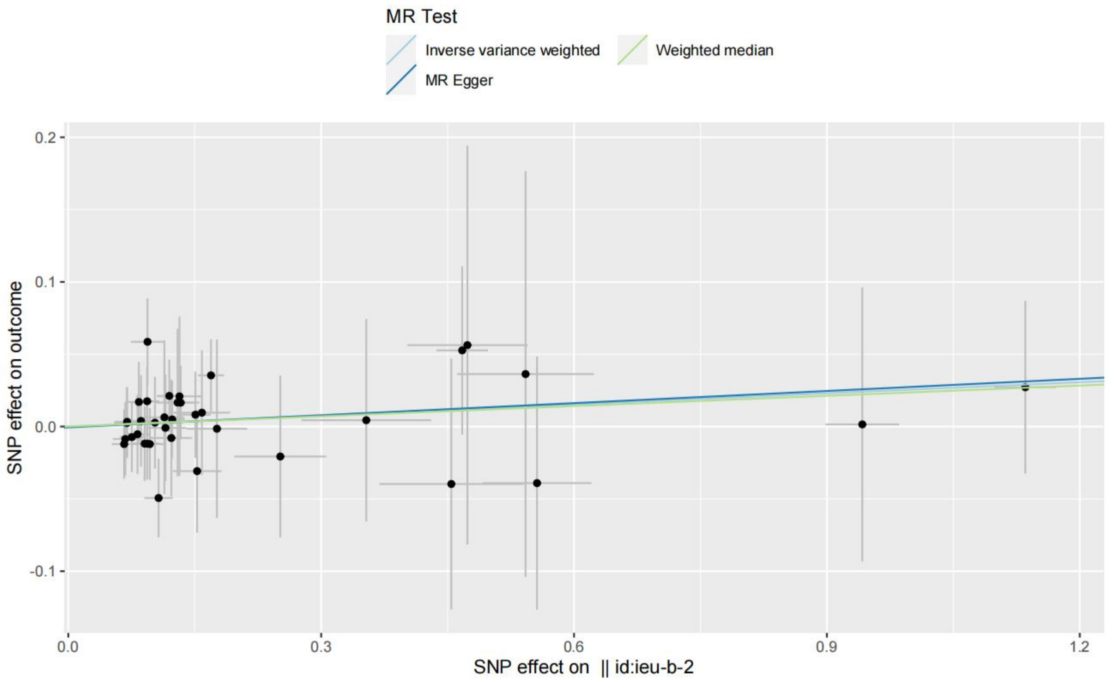

A. Scatter plot of AD on TNF-A

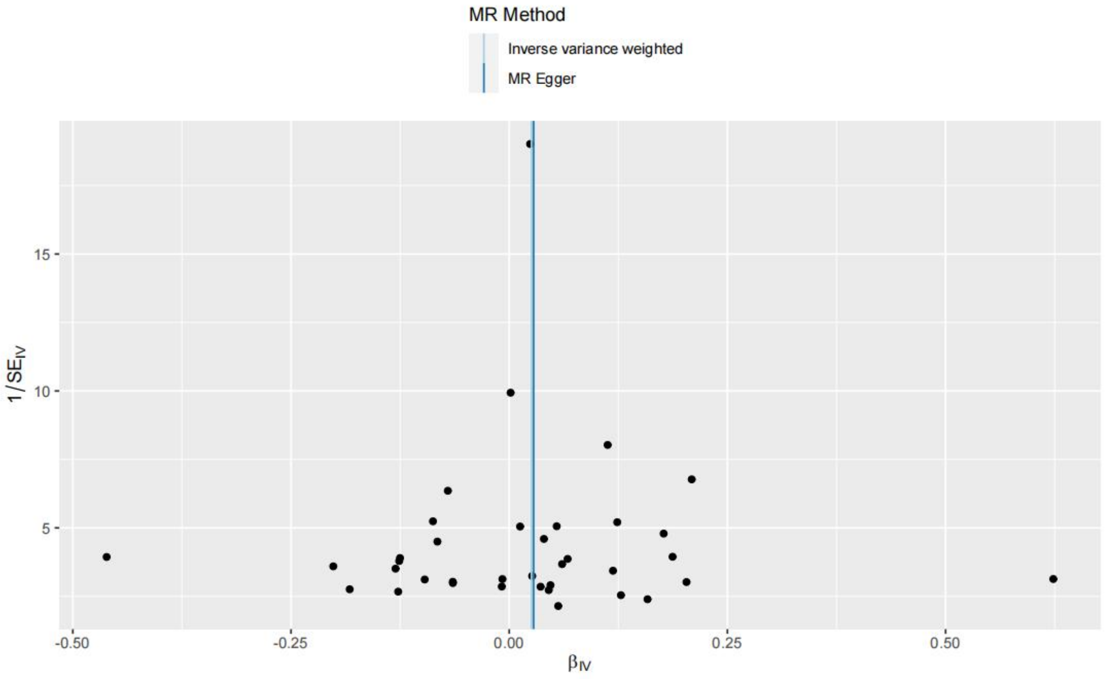

B. Funnel plot of AD on TNF-A

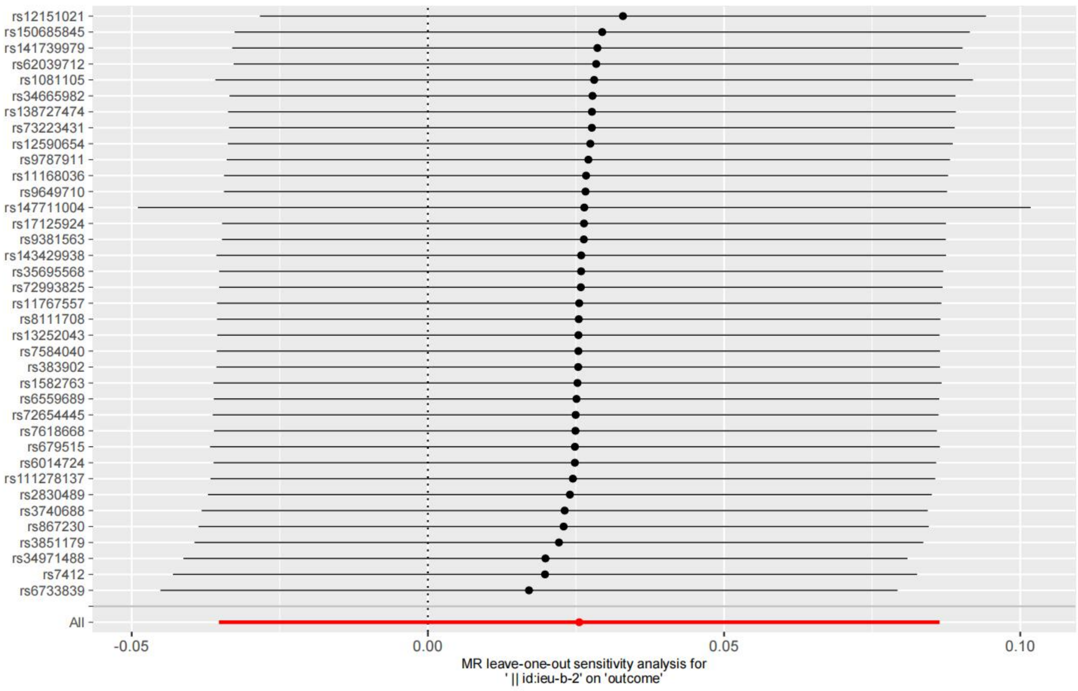

C. MR leave-one-out sensitivity analysis for AD on TNF-A

eFigure 162. AD-associated SNPs with TNF-B

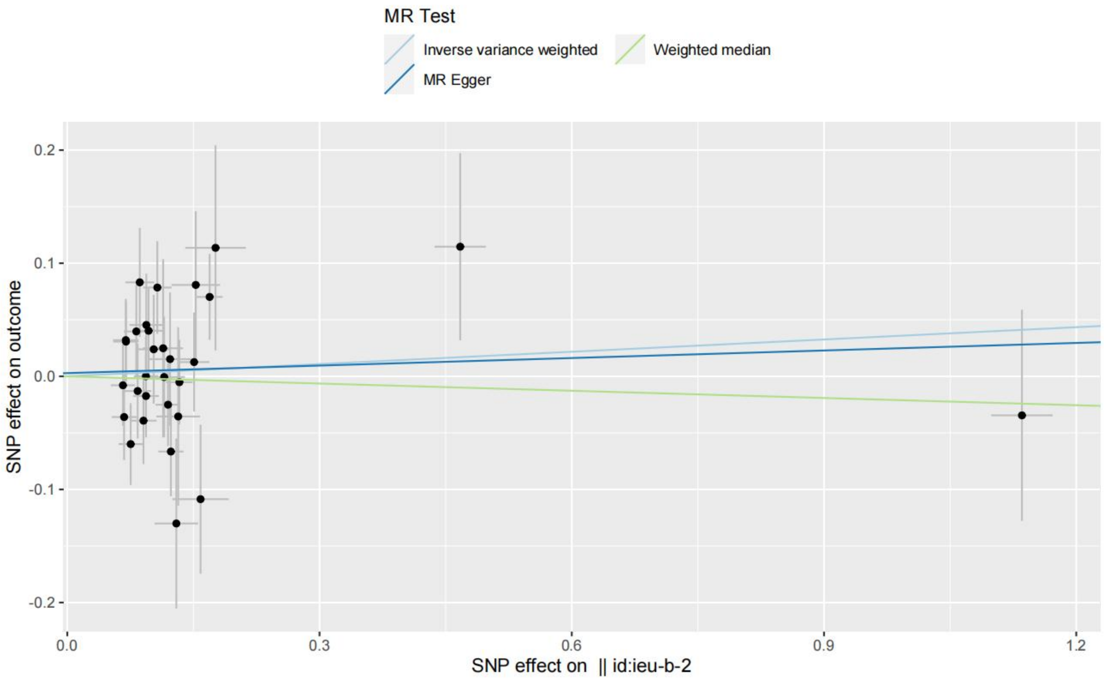

A. Scatter plot of AD on TNF-B

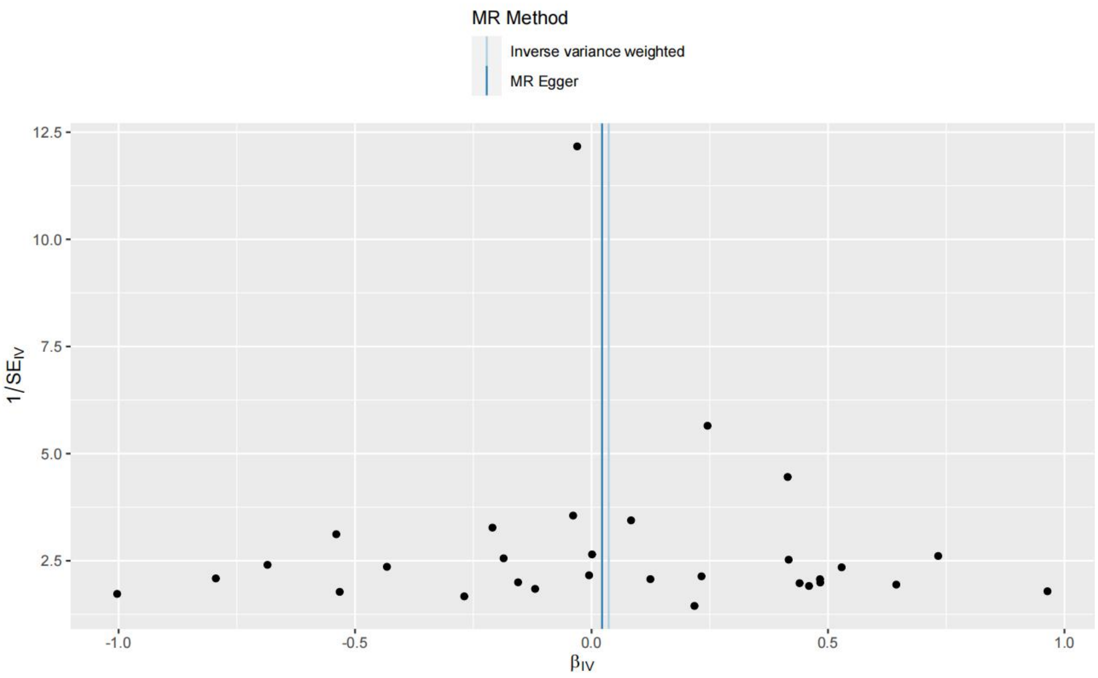

B. Funnel plot of AD on TNF-B

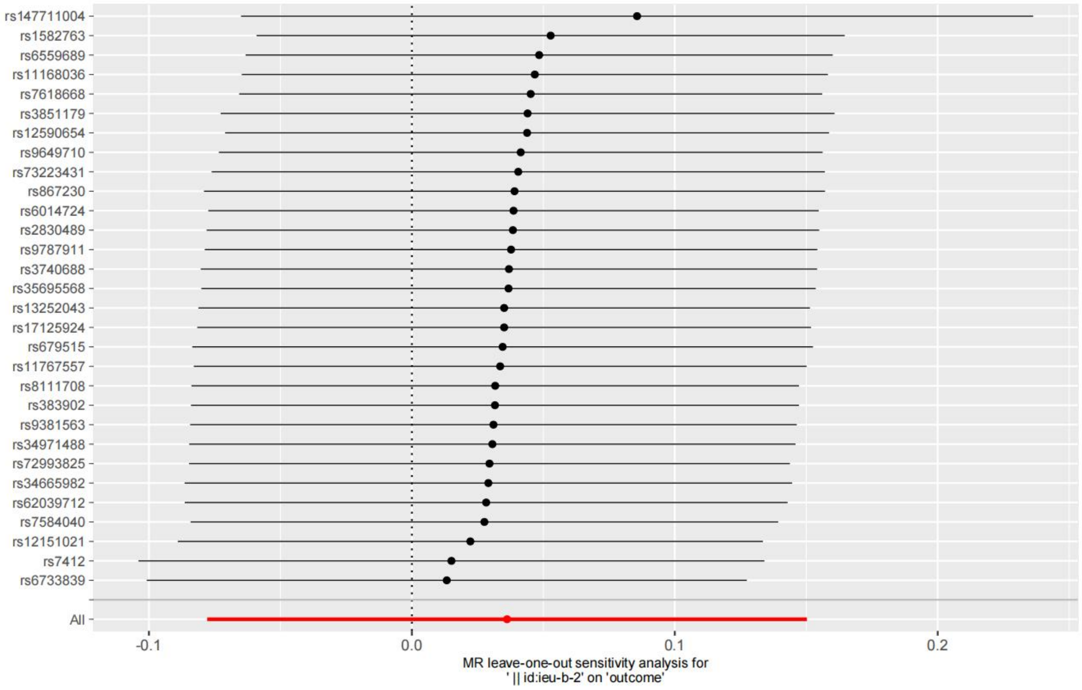

C. MR leave-one-out sensitivity analysis for AD on TNF-B

eFigure 163. AD-associated SNPs with TRAIL

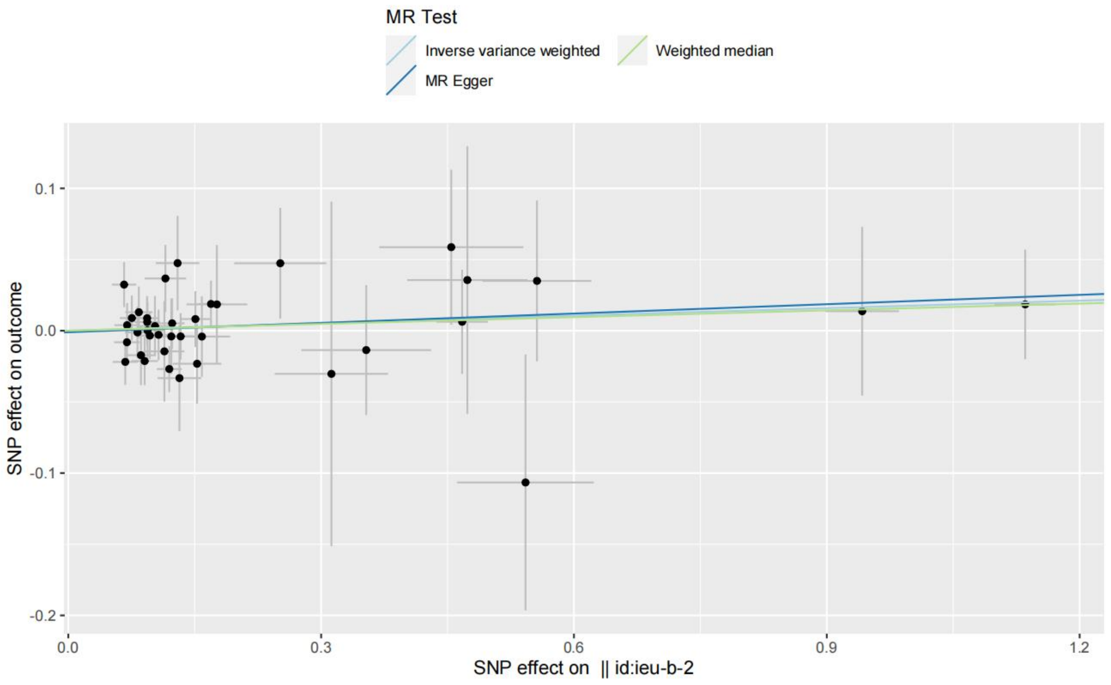

A. Scatter plot of AD on TRAIL

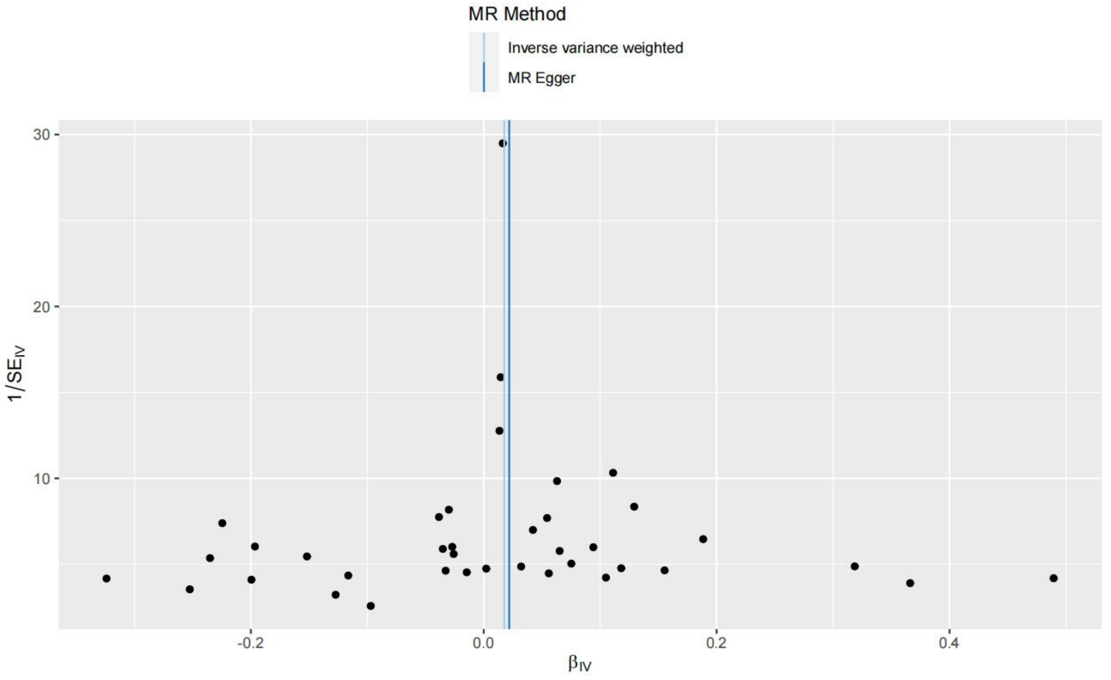

B. Funnel plot of AD on TRAIL

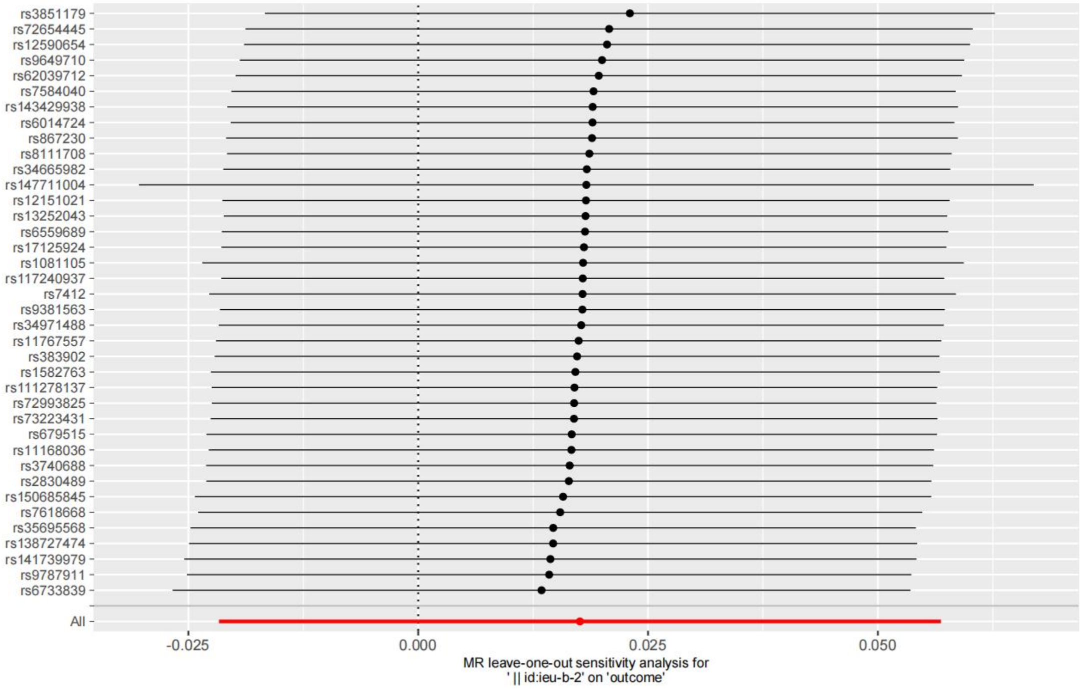

C. MR leave-one-out sensitivity analysis for AD on TRAIL

eFigure 164. AD-associated SNPs with VEGF

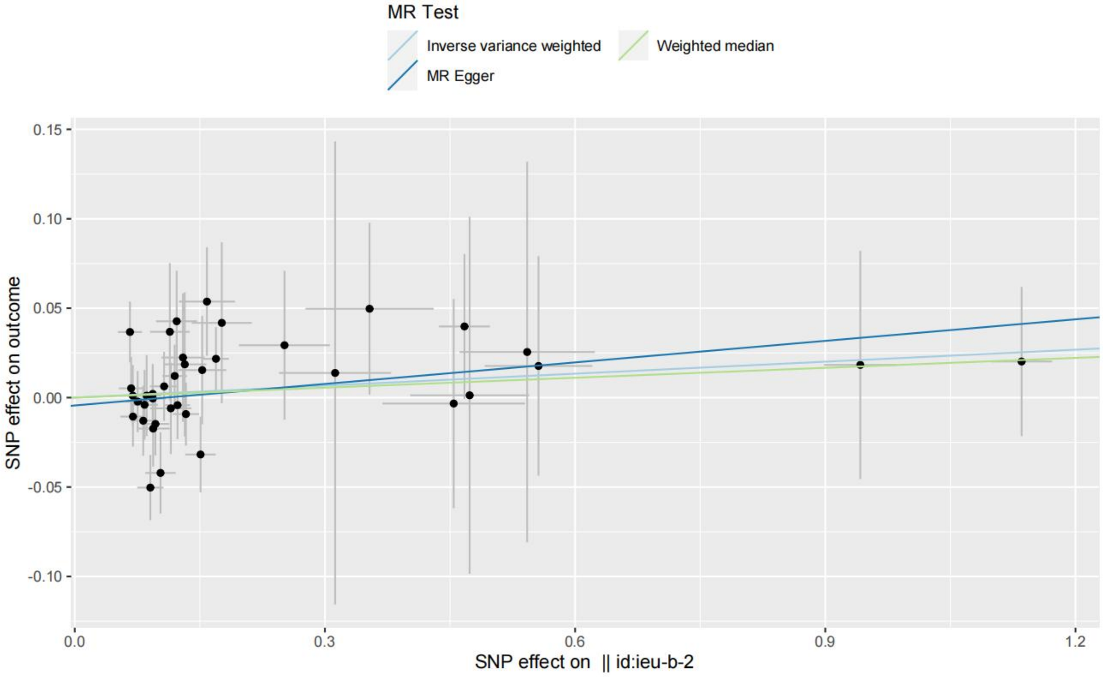

A. Scatter plot of AD on VEGF

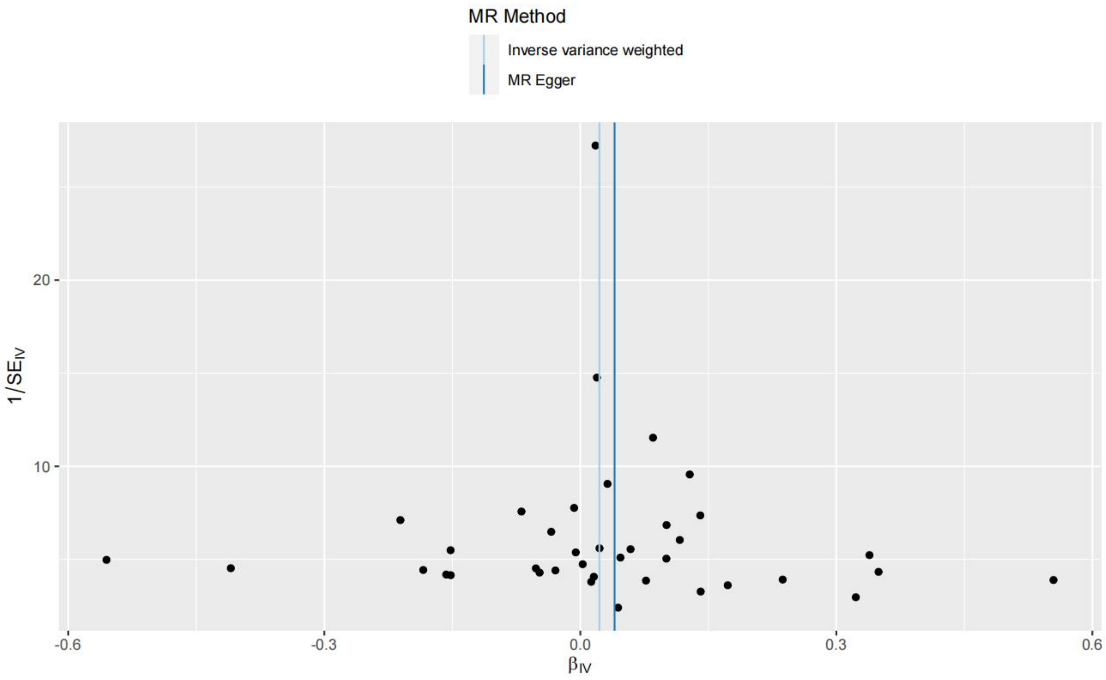

B. Funnel plot of AD on VEGF

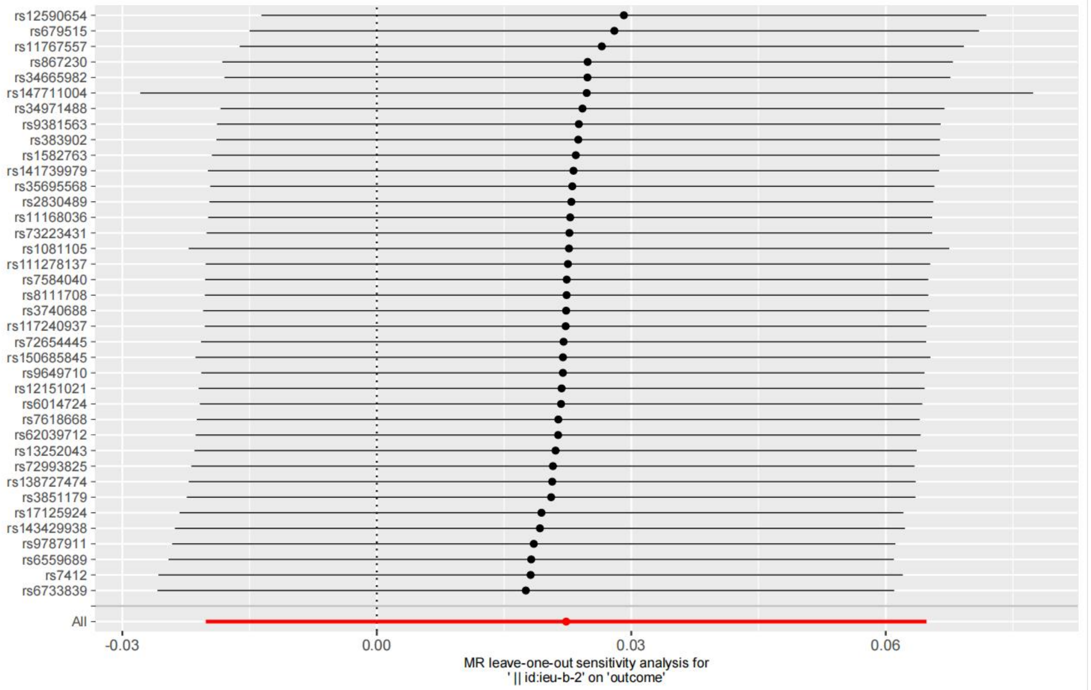

C. MR leave-one-out sensitivity analysis for AD on VEGF

eFigure 165. PD-associated SNPs with BNGF

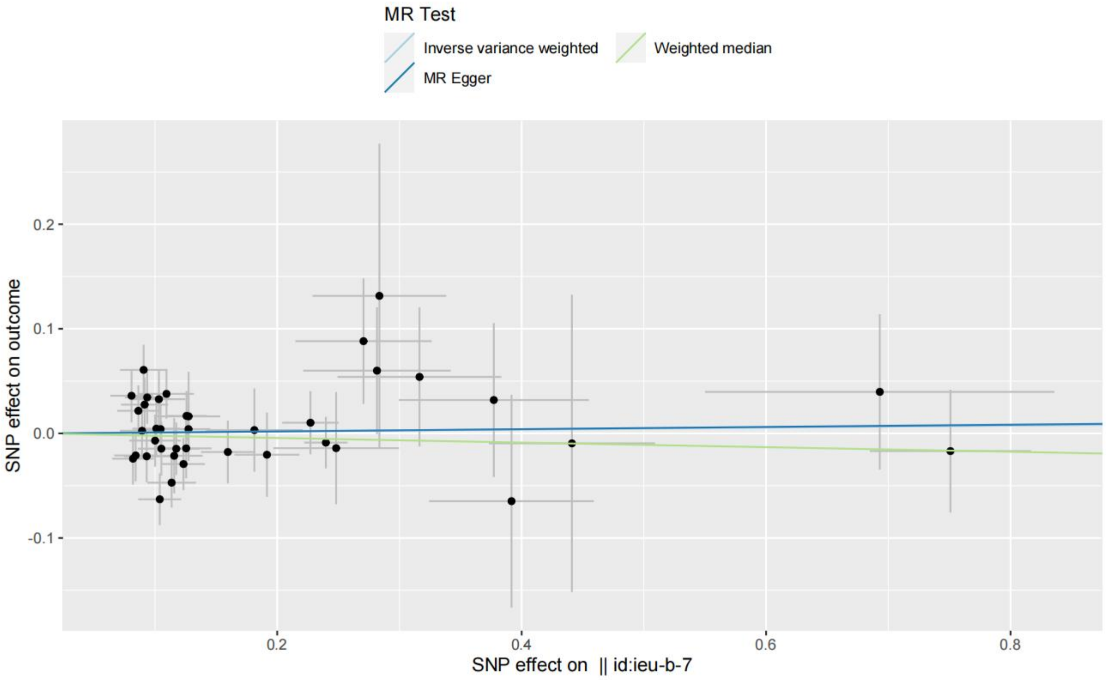

A. Scatter plot of PD on BNGF

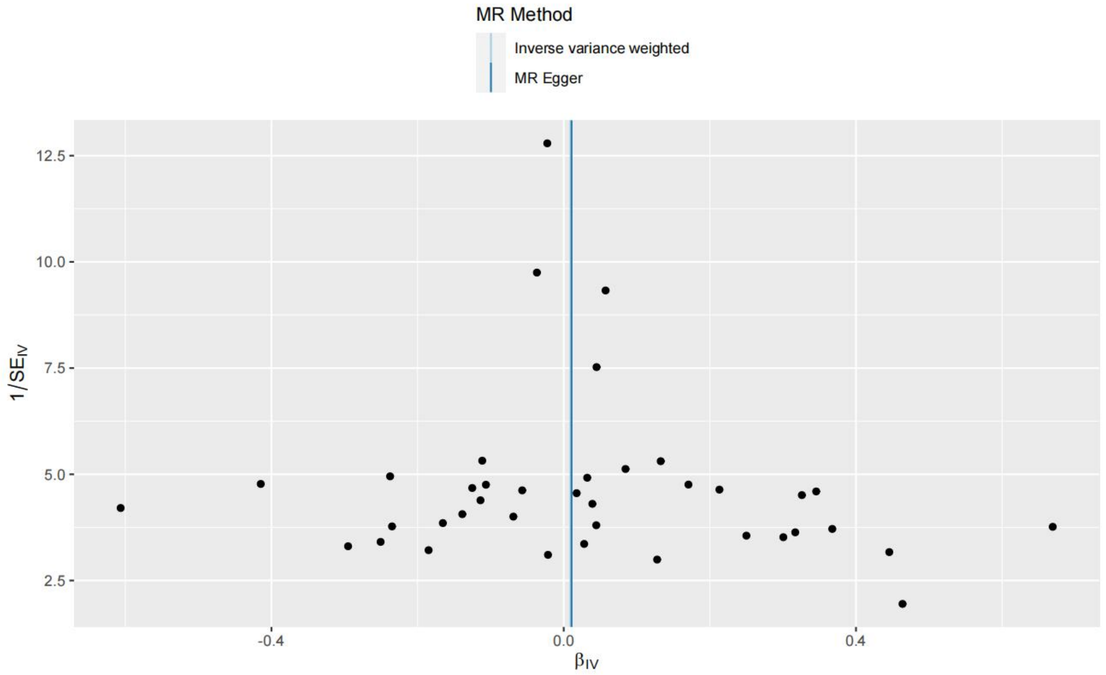

B. Funnel plot of PD on BNGF

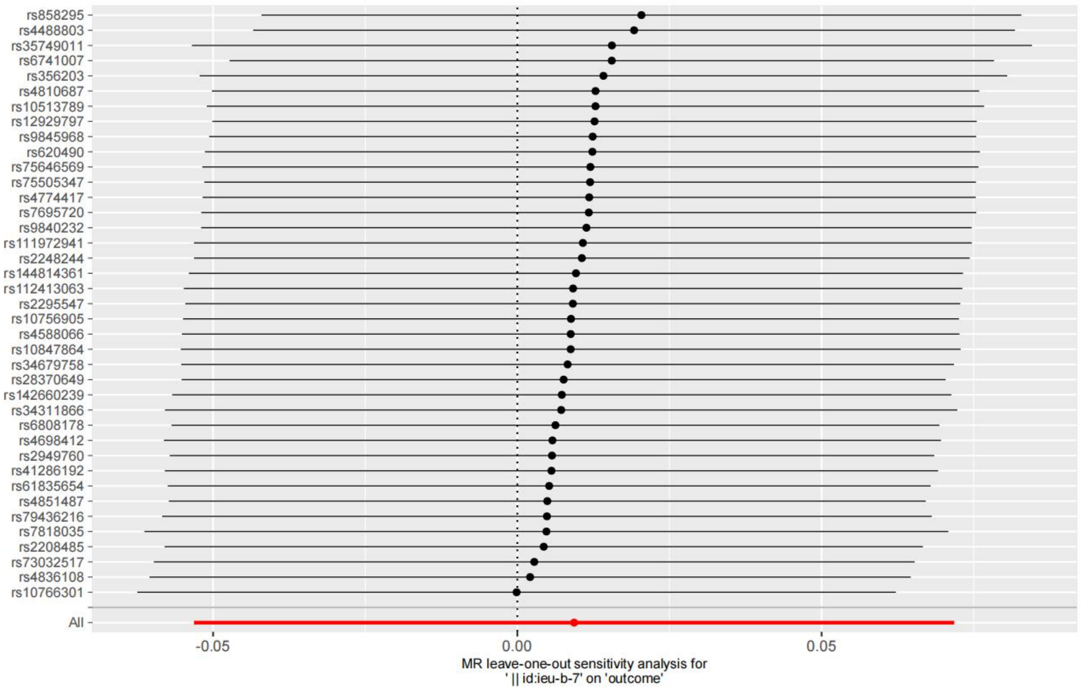

C. MR leave-one-out sensitivity analysis for PD on BNGF

eFigure 166. PD-associated SNPs with CTACK

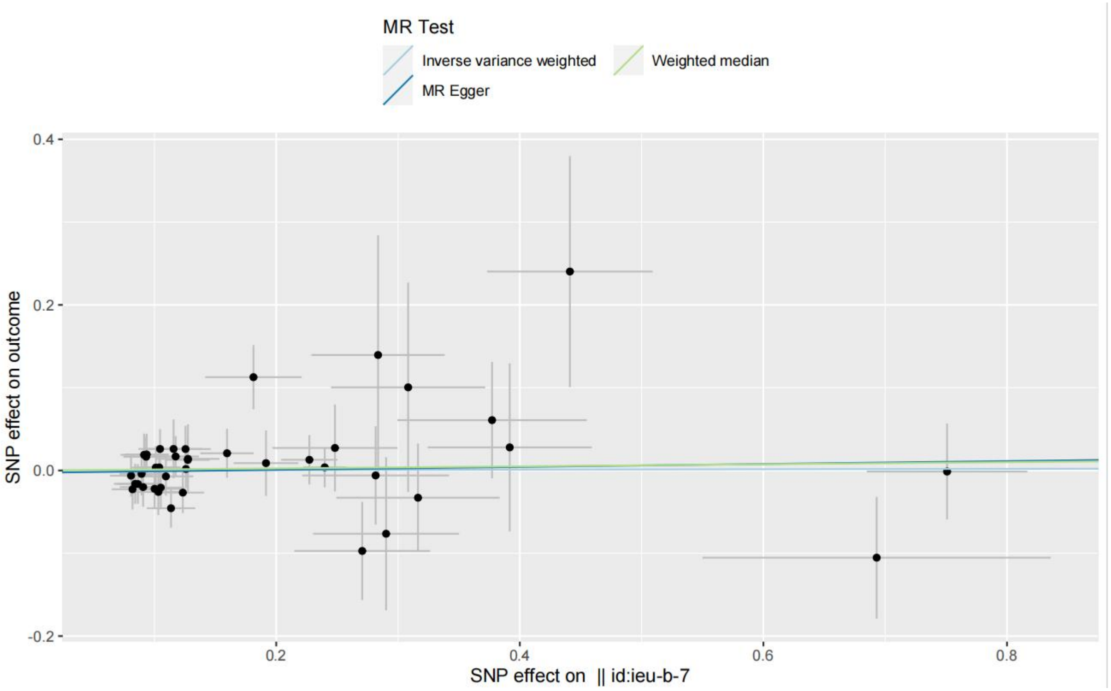

A. Scatter plot of PD on CTACK

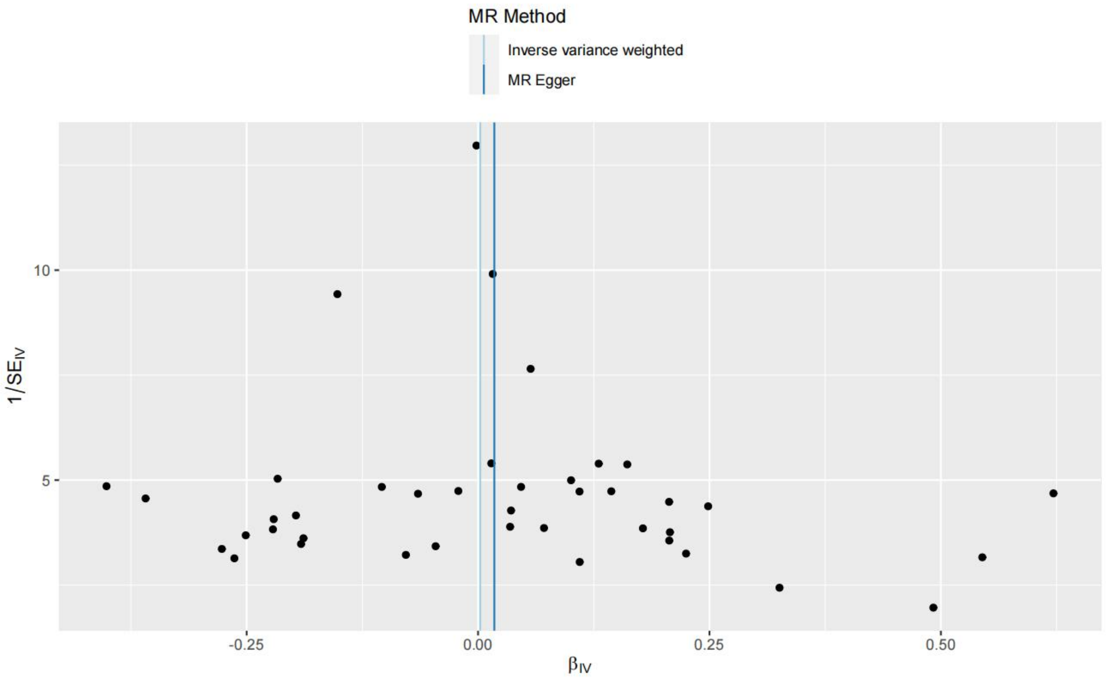

B. Funnel plot of PD on CTACK

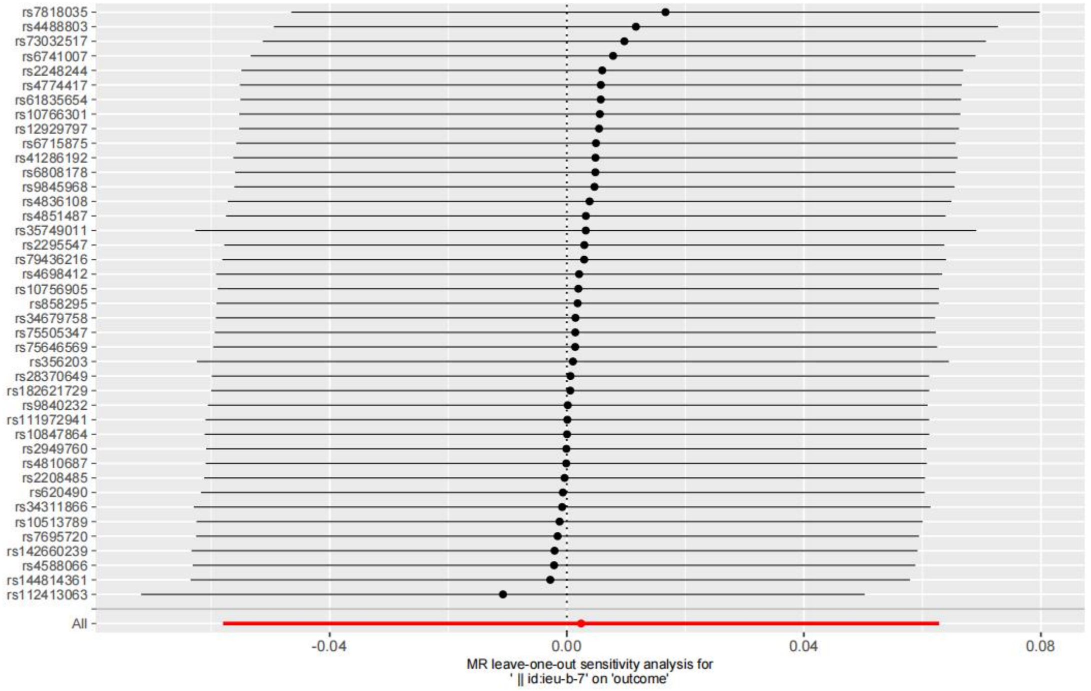

C. MR leave-one-out sensitivity analysis for PD on CTACK

eFigure 167. PD-associated SNPs with EOTAXIN

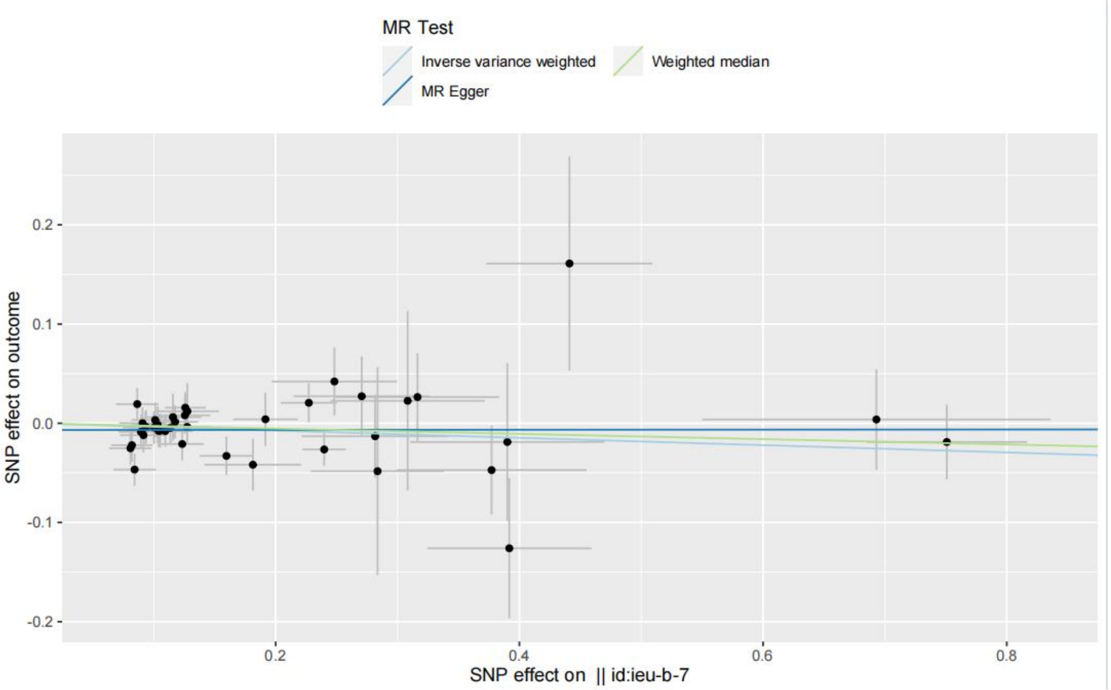

A. Scatter plot of PD on EOTAXIN

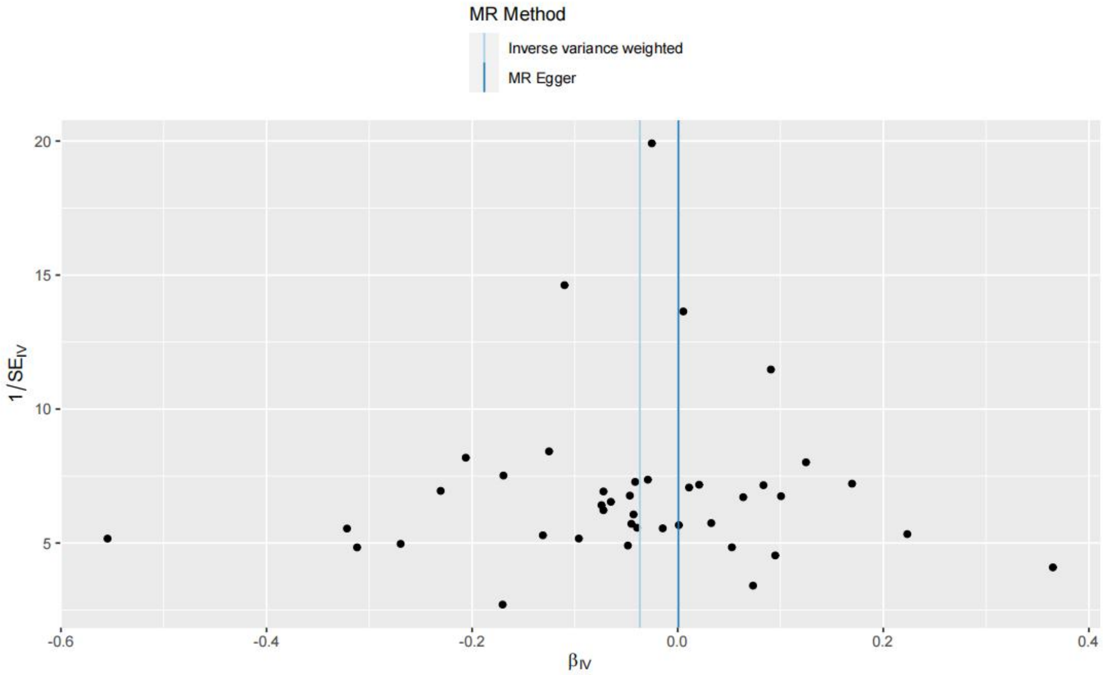

B. Funnel plot of PD on EOTAXIN

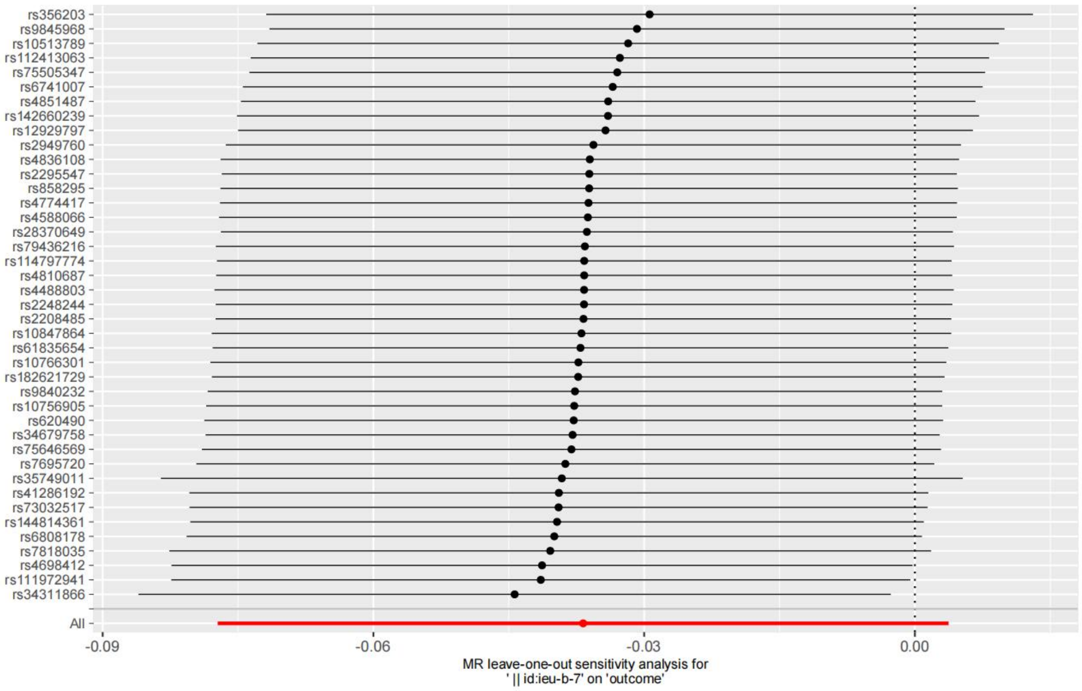

C. MR leave-one-out sensitivity analysis for PD on EOTAXIN

eFigure 168. PD-associated SNPs with bFGF

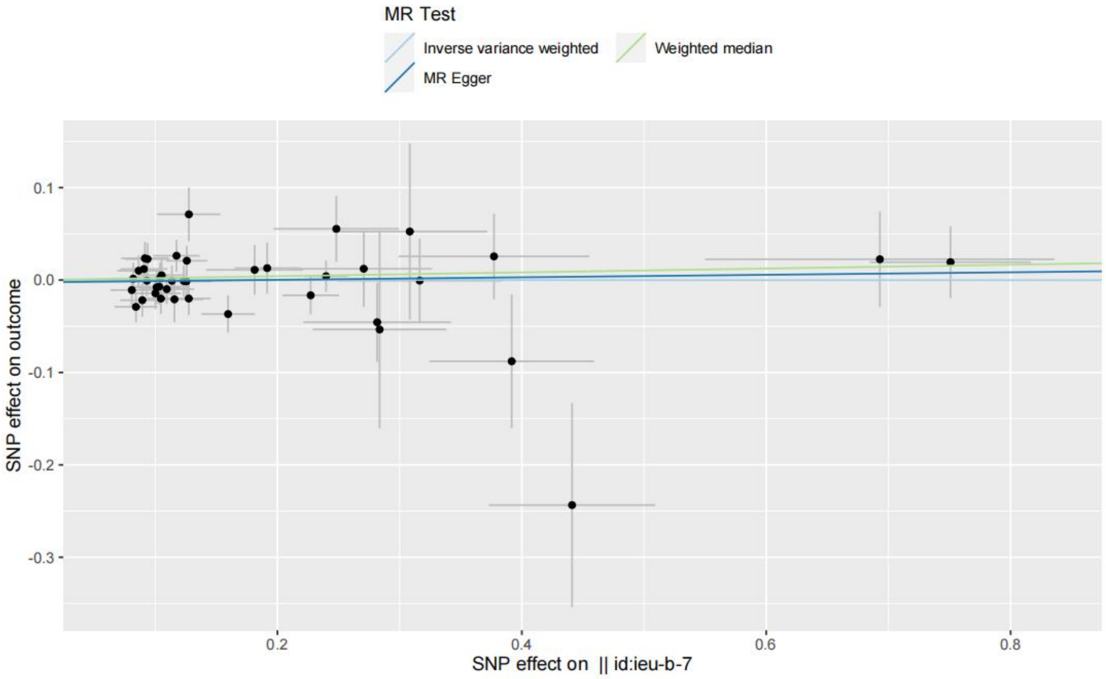

A. Scatter plot of PD on bFGF

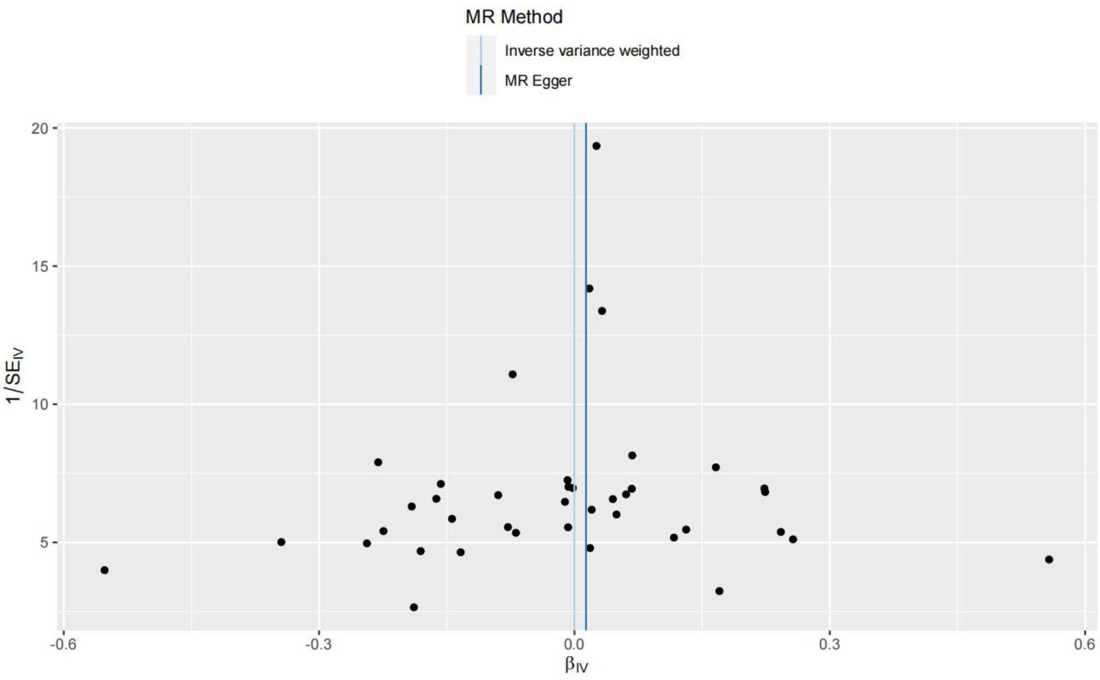

B. Funnel plot of PD on bFGF

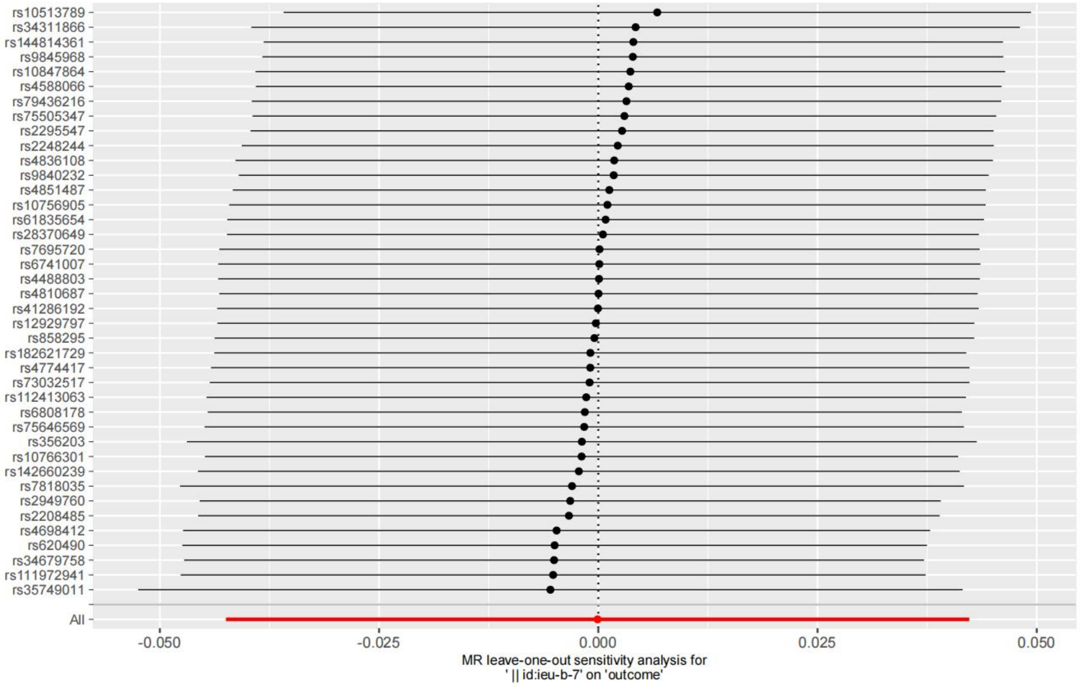

C. MR leave-one-out sensitivity analysis for PD on bFGF

eFigure 169. PD-associated SNPs with G-CSF

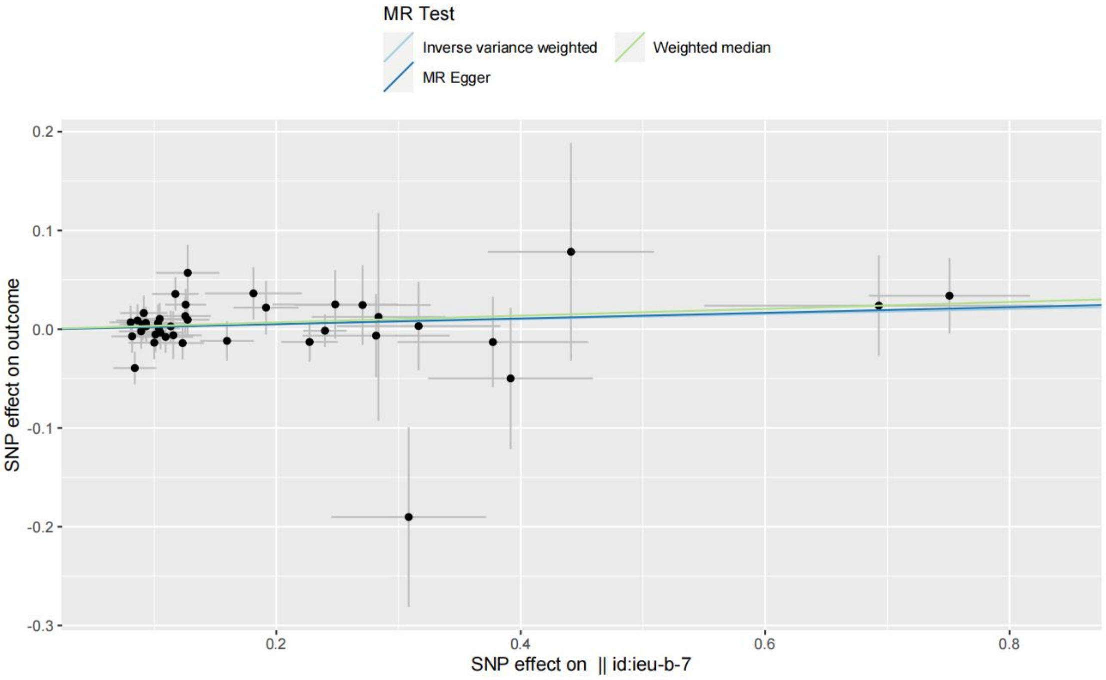

A. Scatter plot of PD on G-CSF

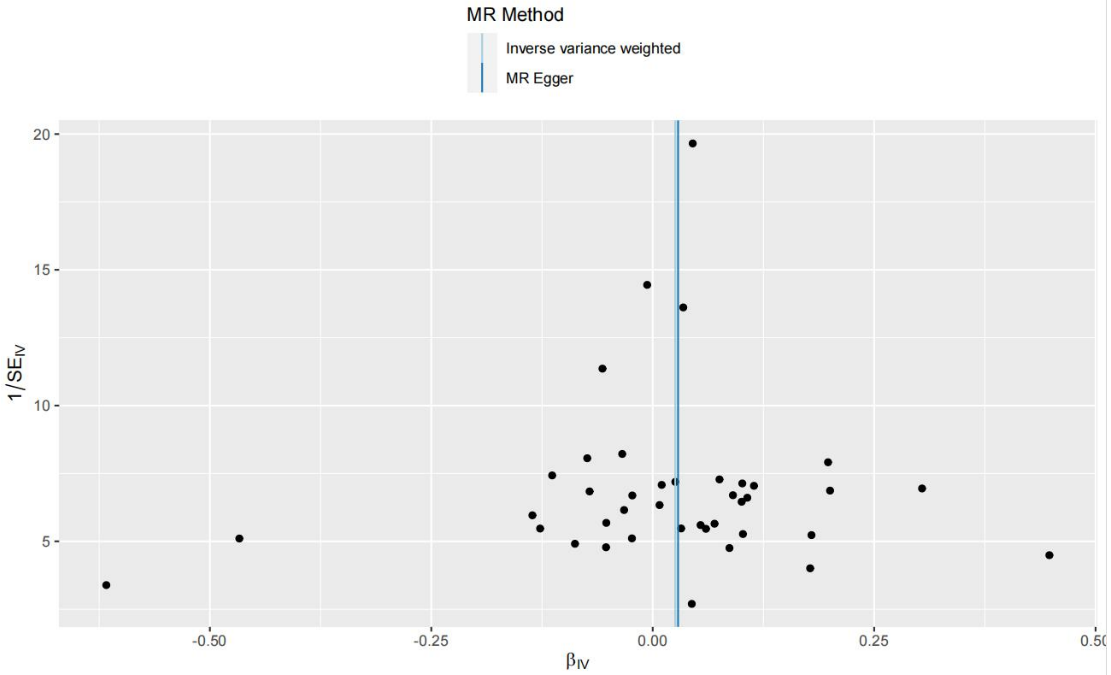

B. Funnel plot of PD on G-CSF

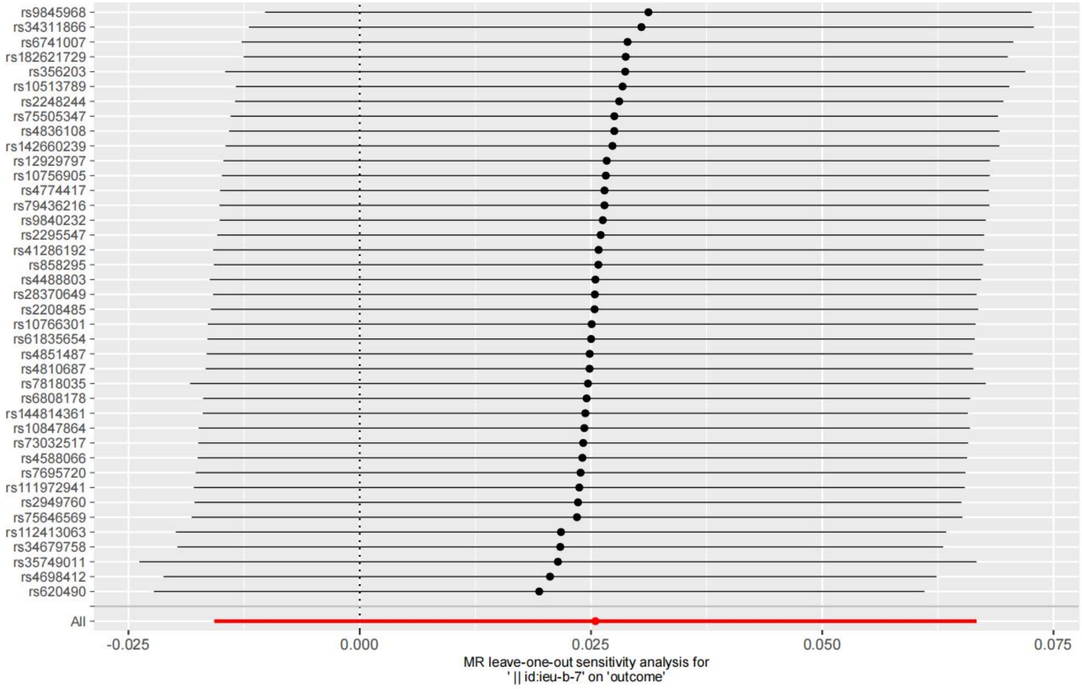

C. MR leave-one-out sensitivity analysis for PD on G-CSF

eFigure 181. PD-associated SNPs with IL-8

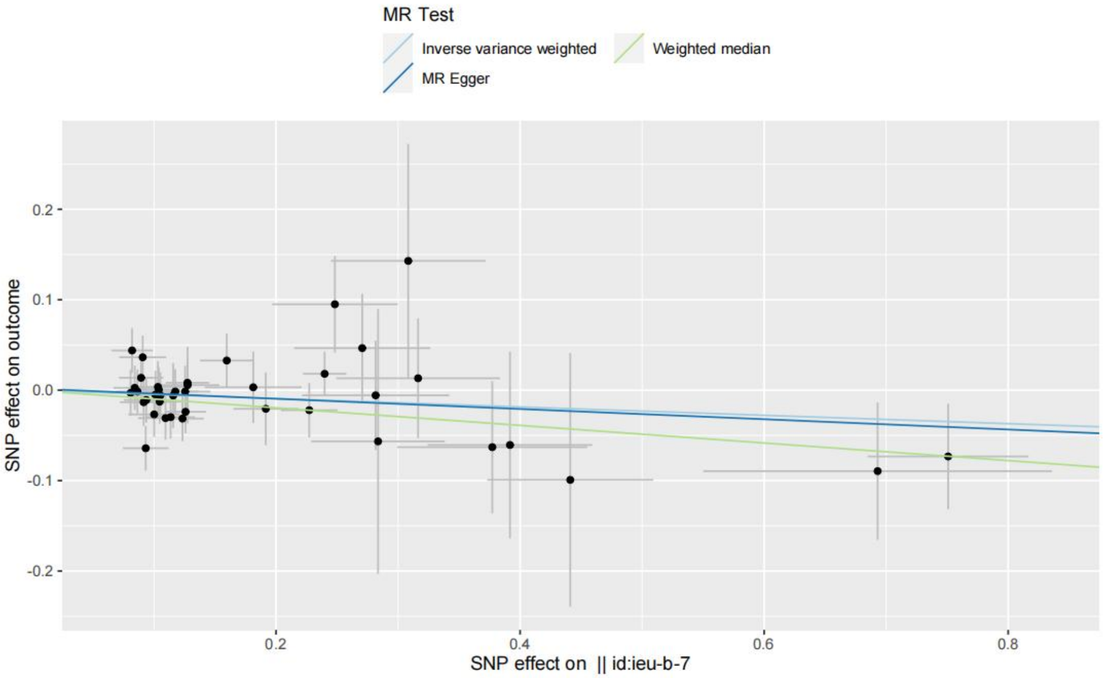

A. Scatter plot of PD on IL-8

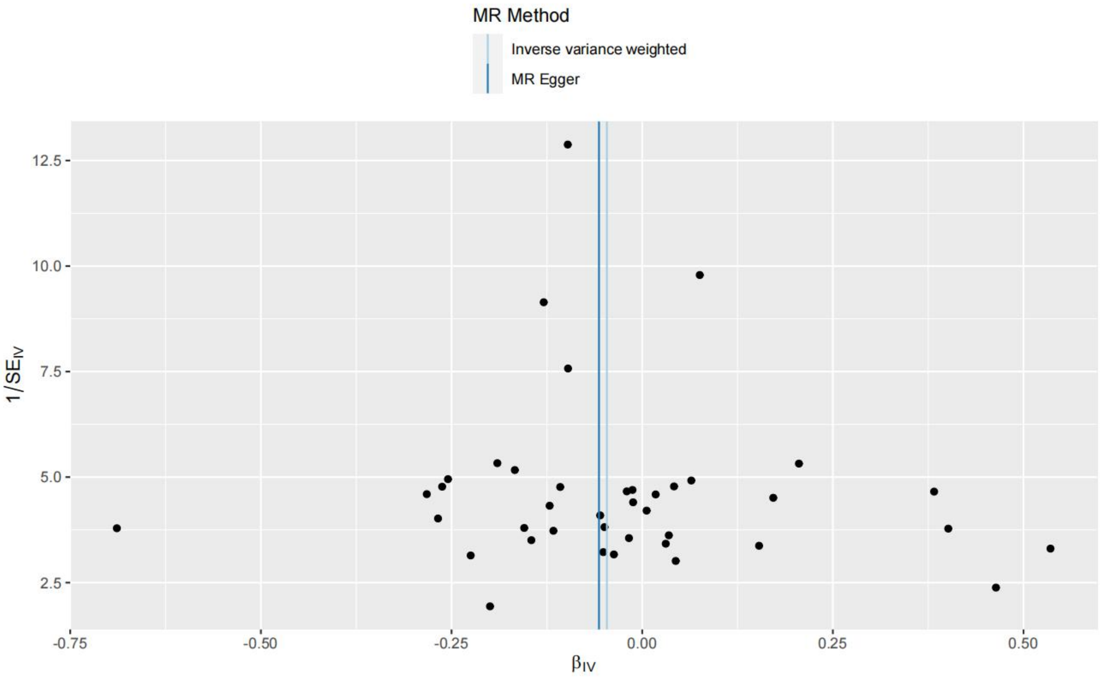

B. Funnel plot of PD on IL-8

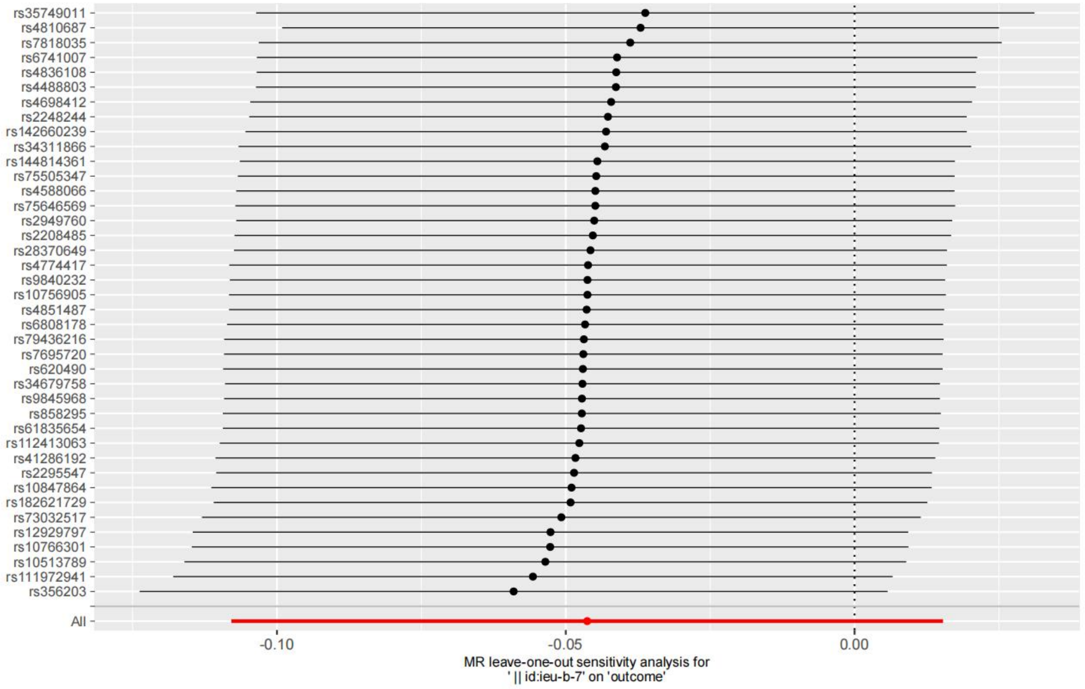

C. MR leave-one-out sensitivity analysis for PD on IL-8

eFigure 170. PD-associated SNPs with GROA

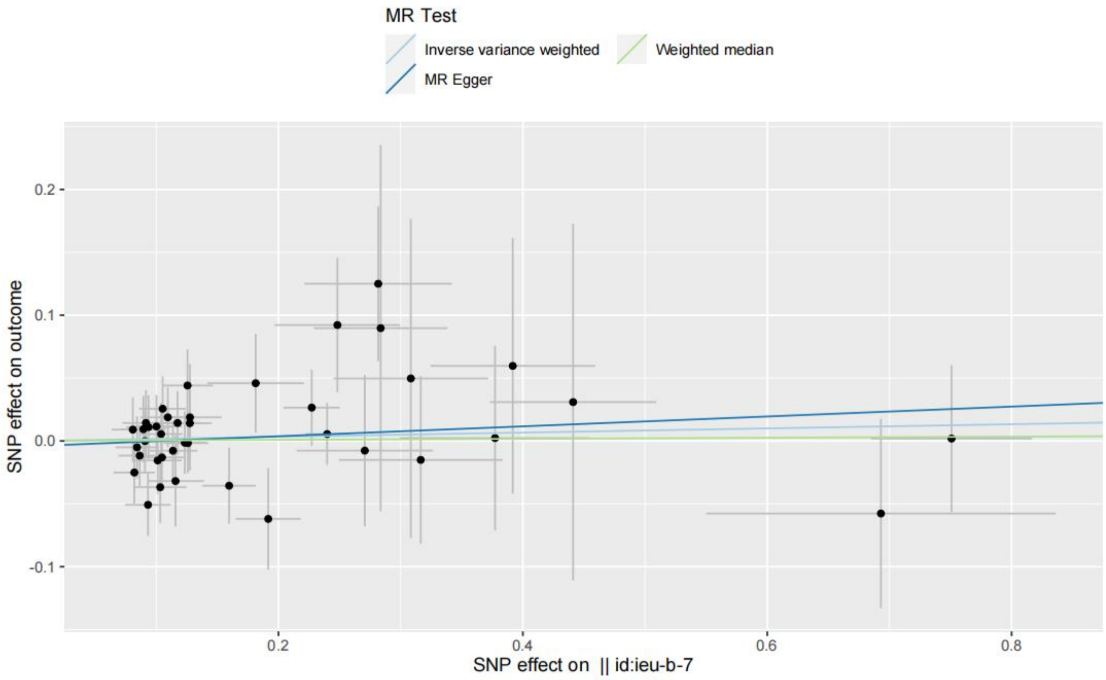

A. Scatter plot of PD on GROA

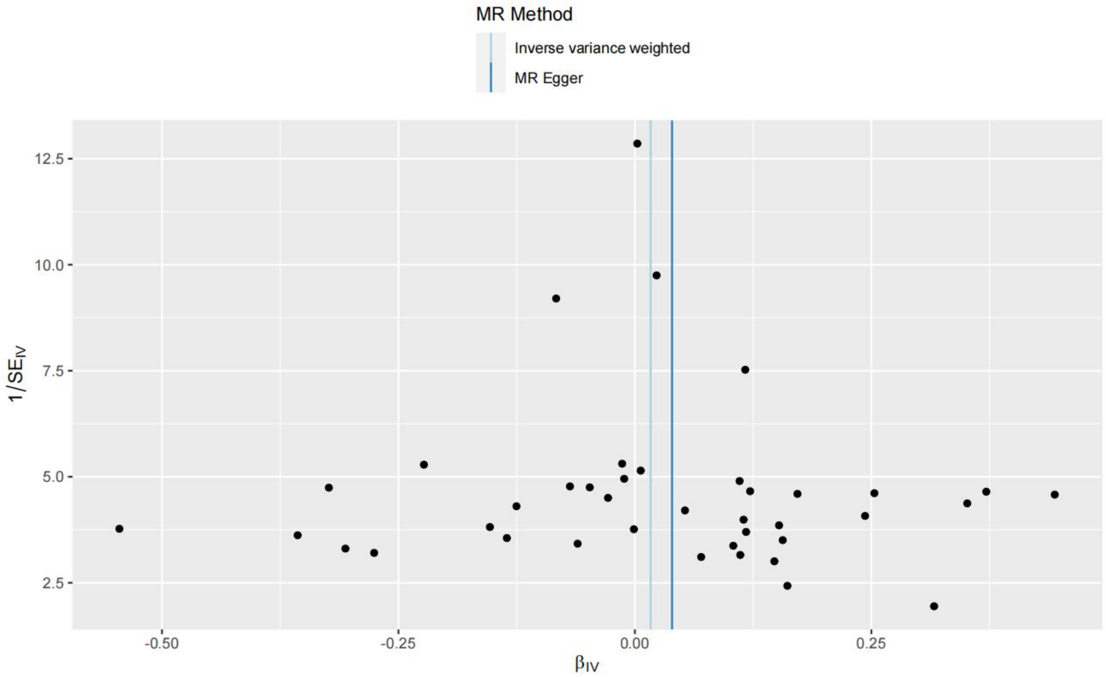

B. Funnel plot of PD on GROA

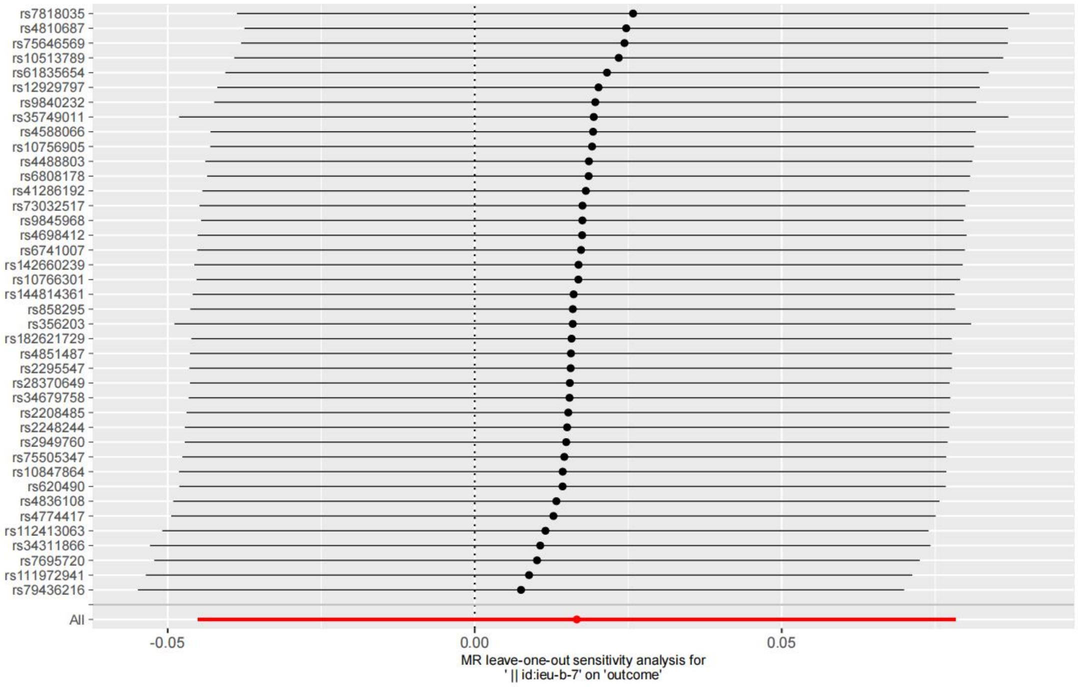

C. MR leave-one-out sensitivity analysis for PD on GROA

eFigure 171. PD-associated SNPs with HGF

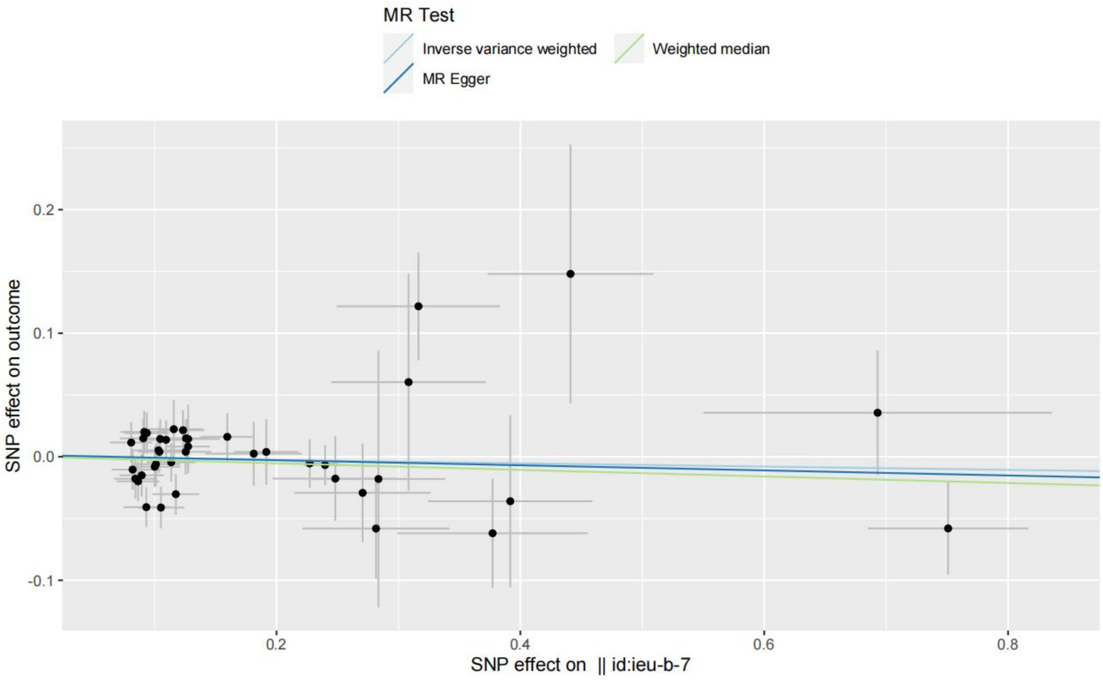

A. Scatter plot of PD on HGF

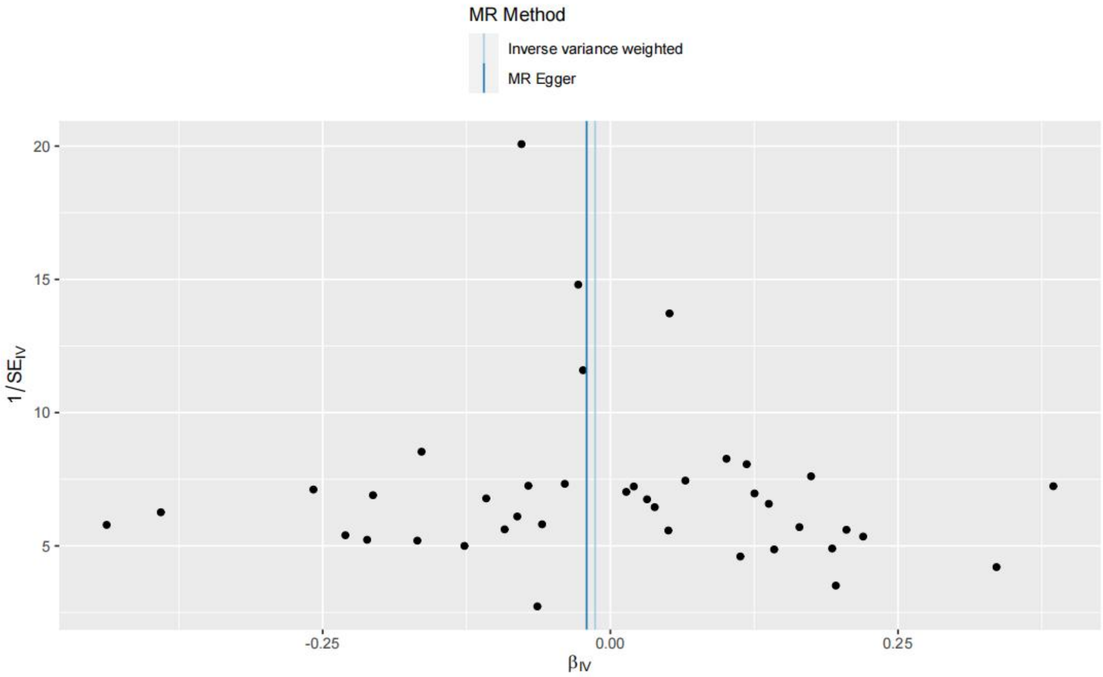

B. Funnel plot of PD on HGF

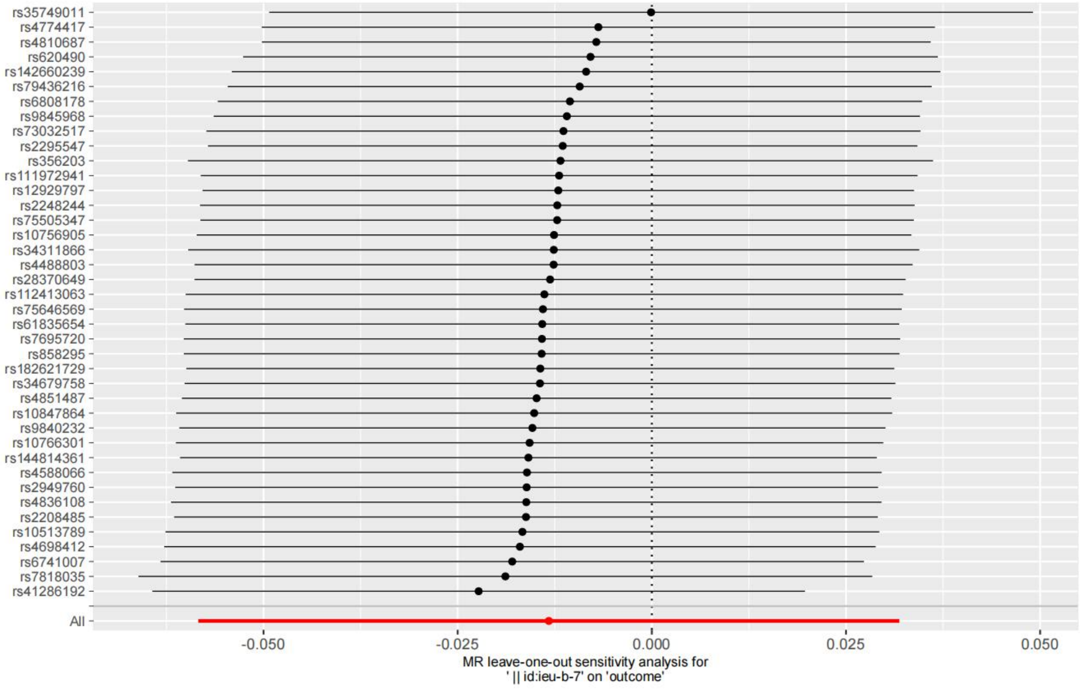

C. MR leave-one-out sensitivity analysis for PD on HGF

eFigure 172. PD-associated SNPs with IFN-G

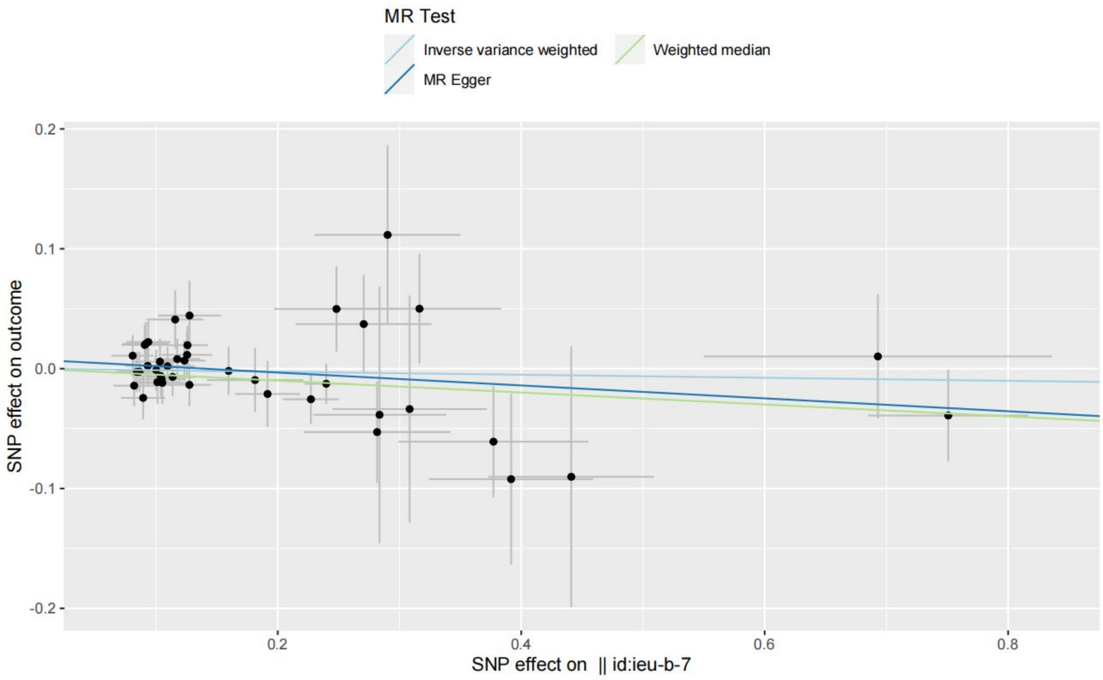

A. Scatter plot of PD on IFN-G

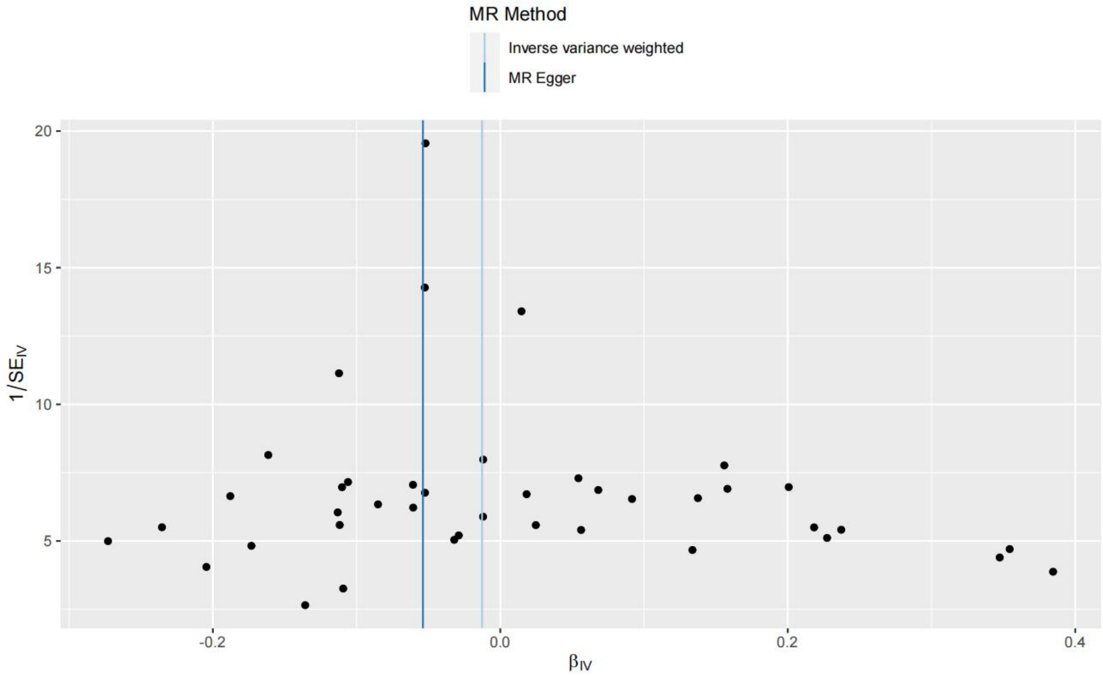

B. Funnel plot of PD on IFN-G

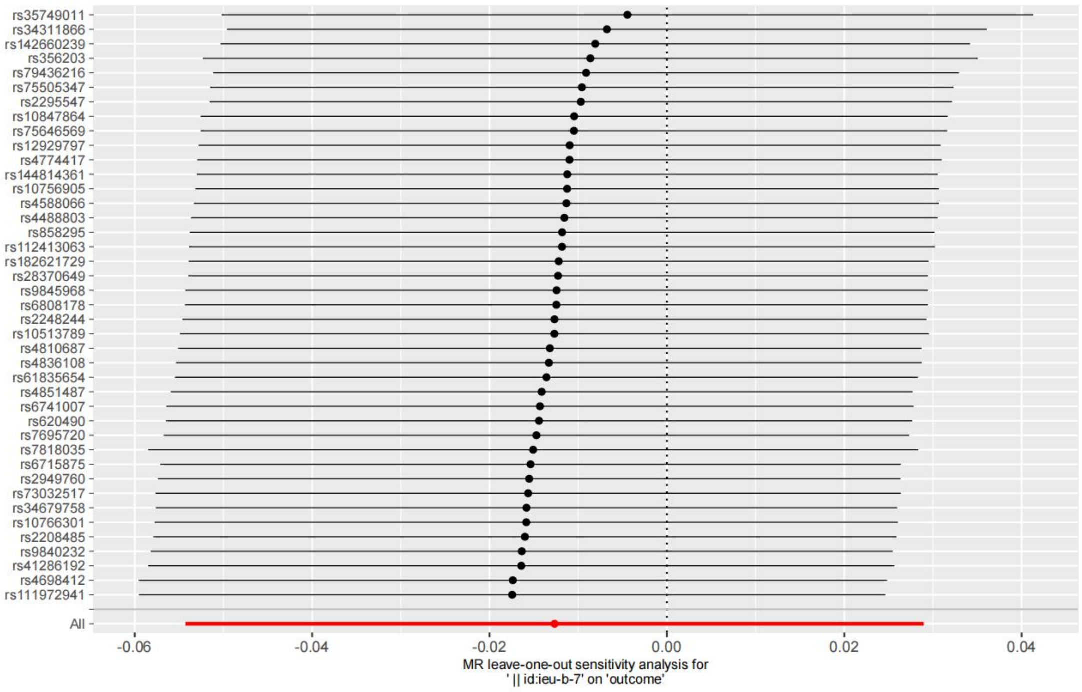

C. MR leave-one-out sensitivity analysis for PD on IFN-G

eFigure 173. PD-associated SNPs with IL-1B

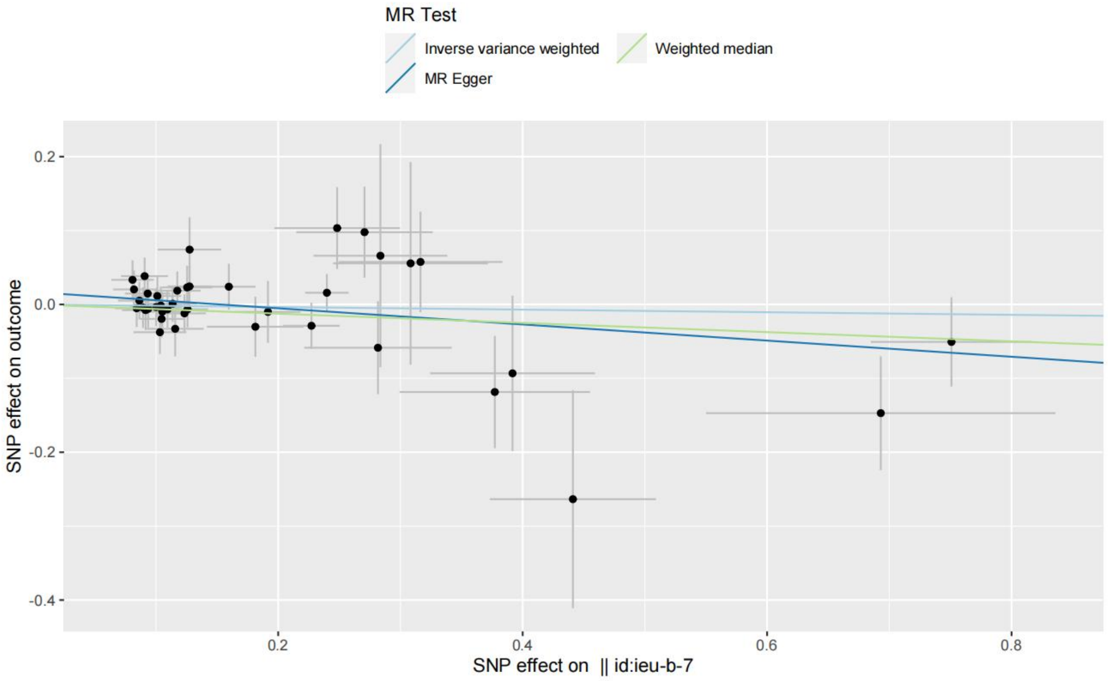

A. Scatter plot of PD on IL-1B

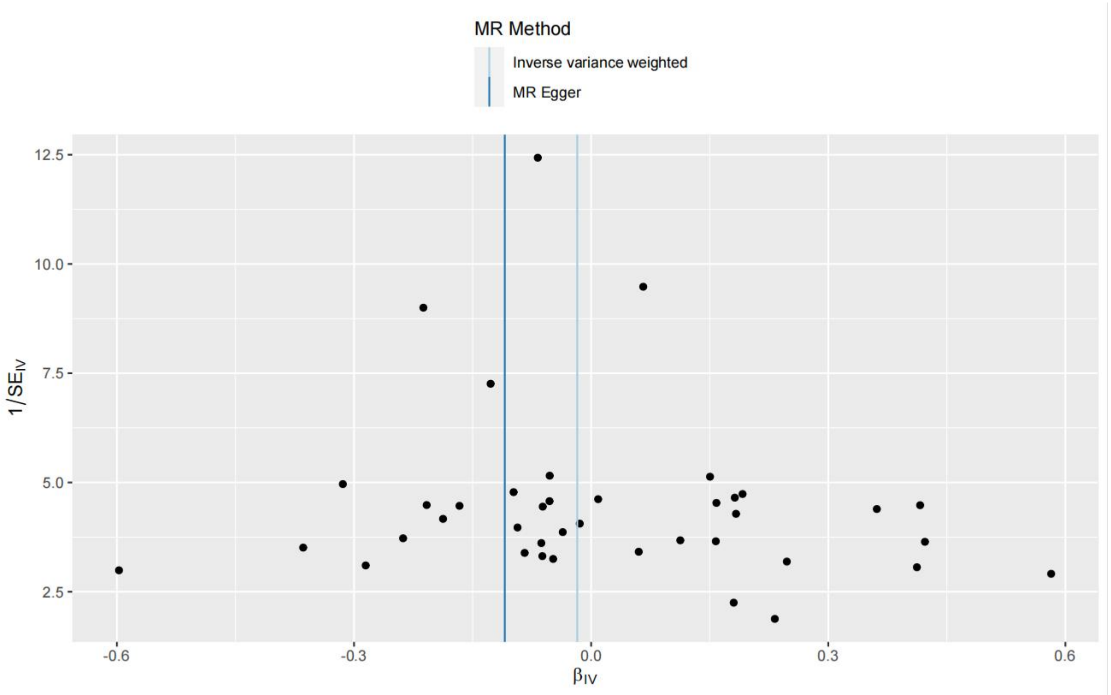

B. Funnel plot of PD on IL-1B

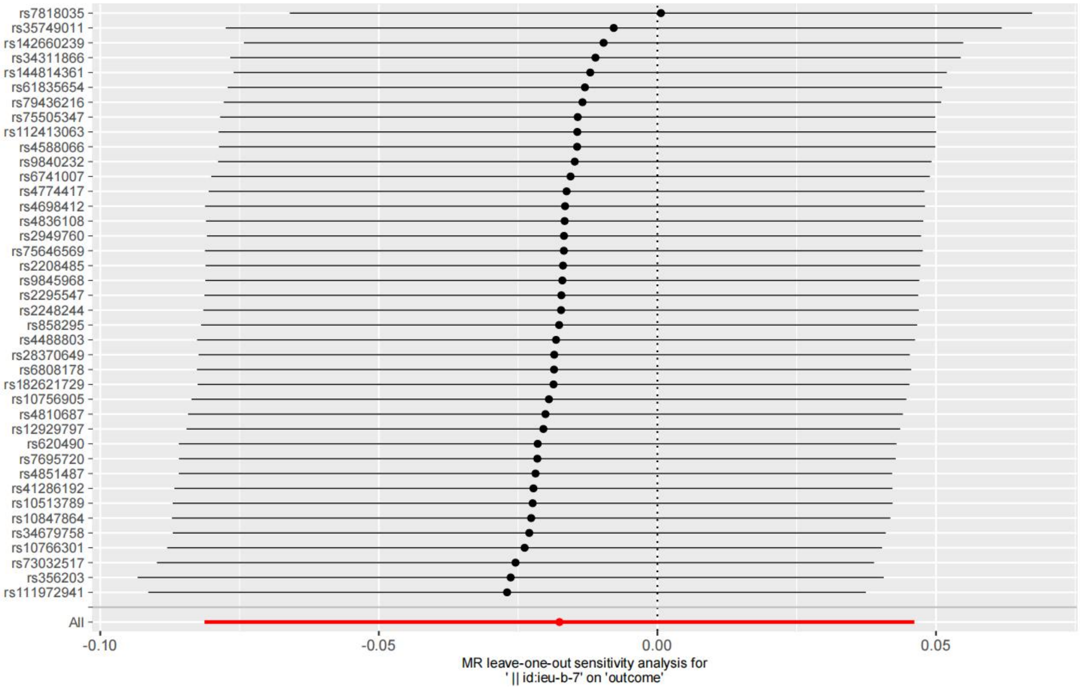

C. MR leave-one-out sensitivity analysis for PD on IL-1B

eFigure 174. PD-associated SNPs with IL-1RA

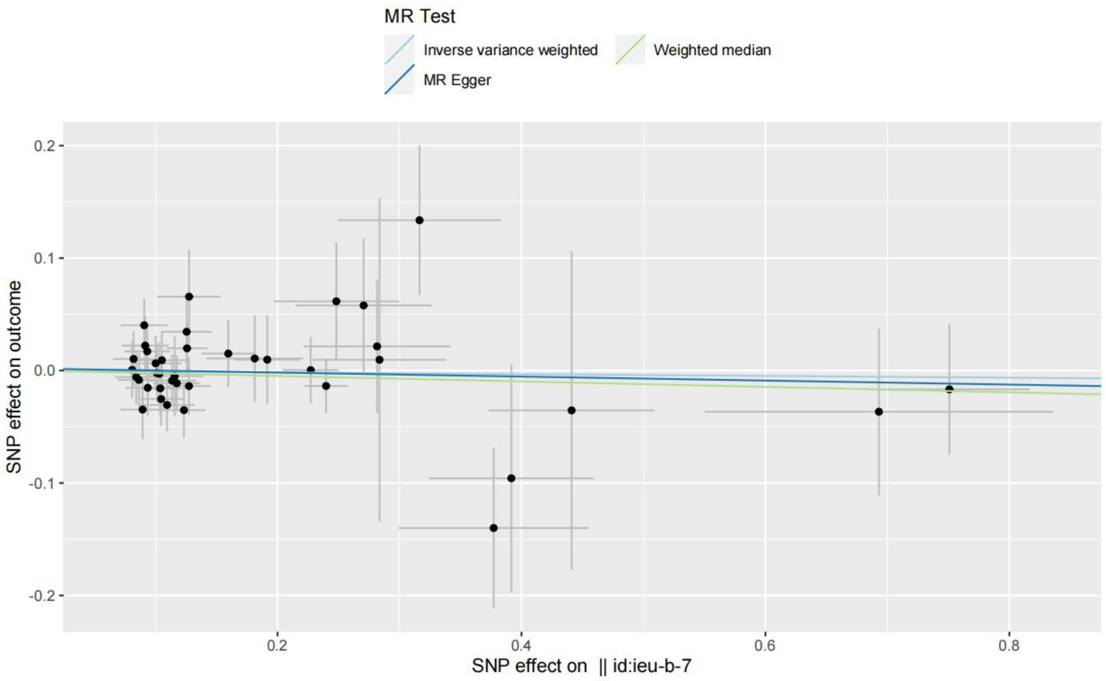

A. Scatter plot of PD on IL-1RA

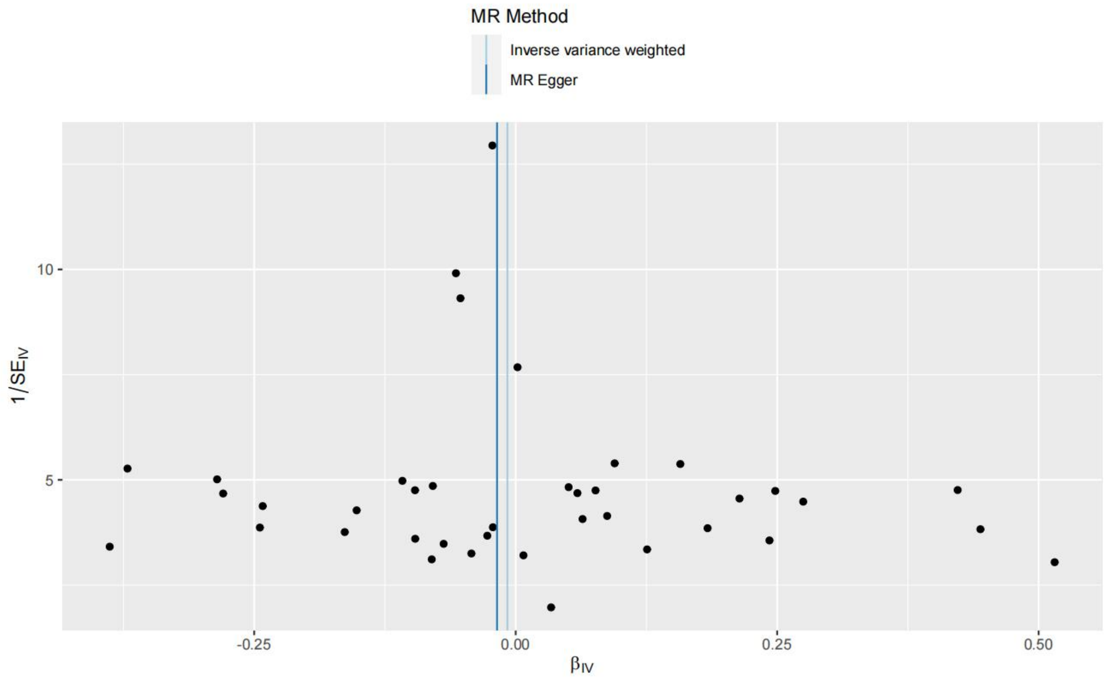

B. Funnel plot of PD on IL-1RA

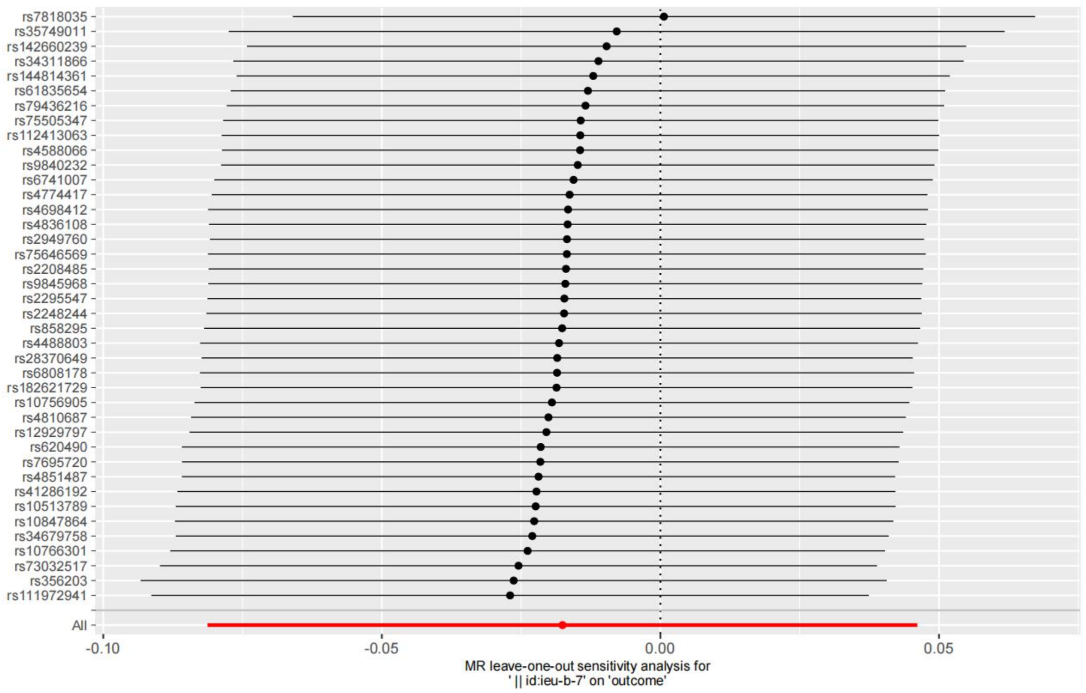

C. MR leave-one-out sensitivity analysis for PD on IL-1RA

eFigure 175. PD-associated SNPs with IL-2

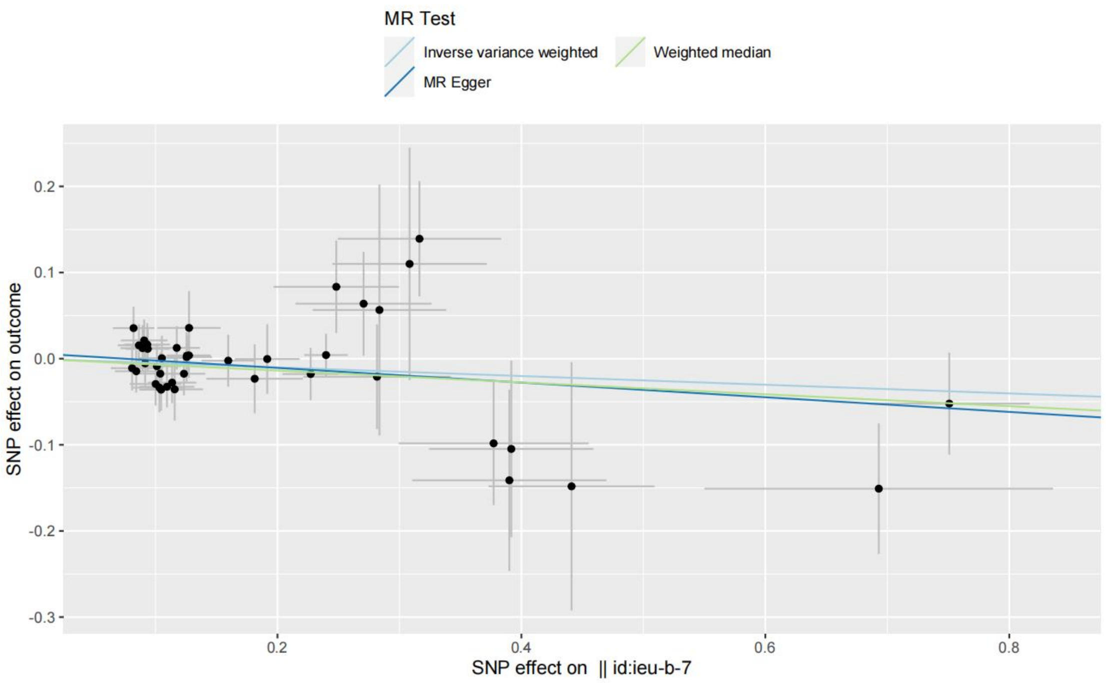

A. Scatter plot of PD on IL-2

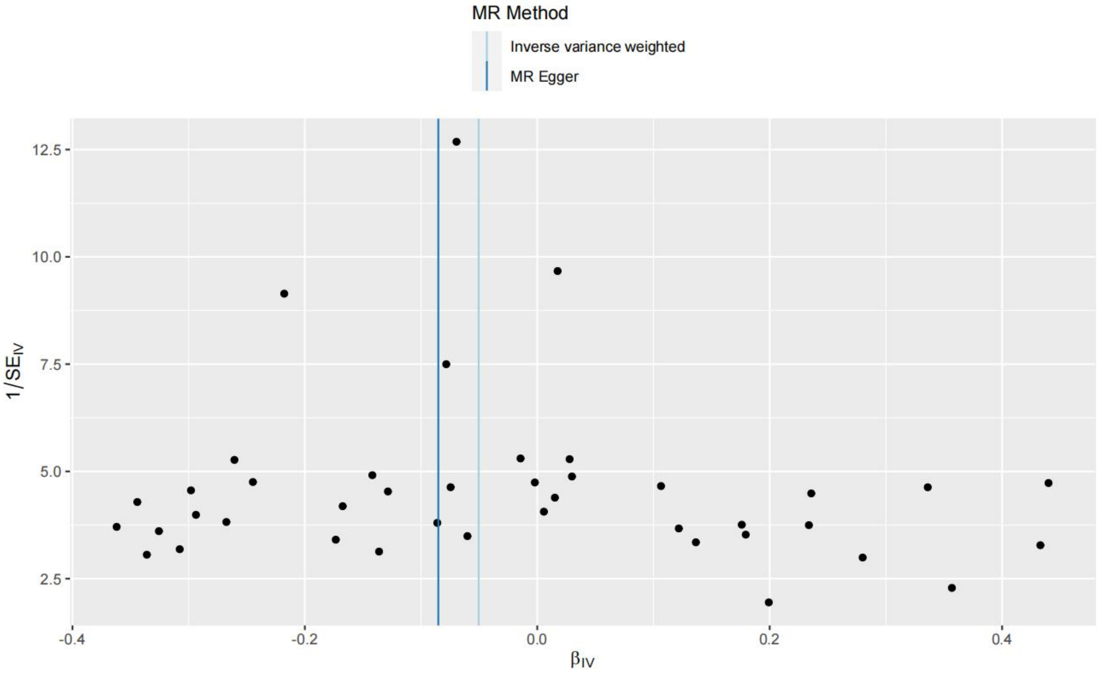

B. Funnel plot of PD on IL-2

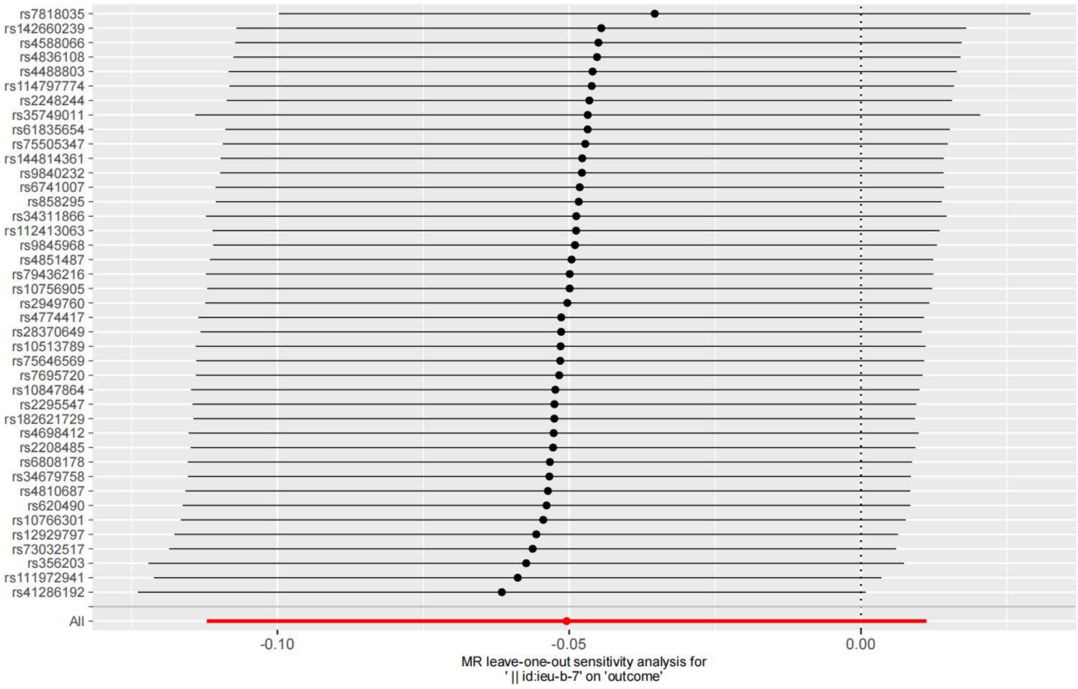

C. MR leave-one-out sensitivity analysis for PD on IL-2

eFigure 176. PD-associated SNPs with IL-2RA

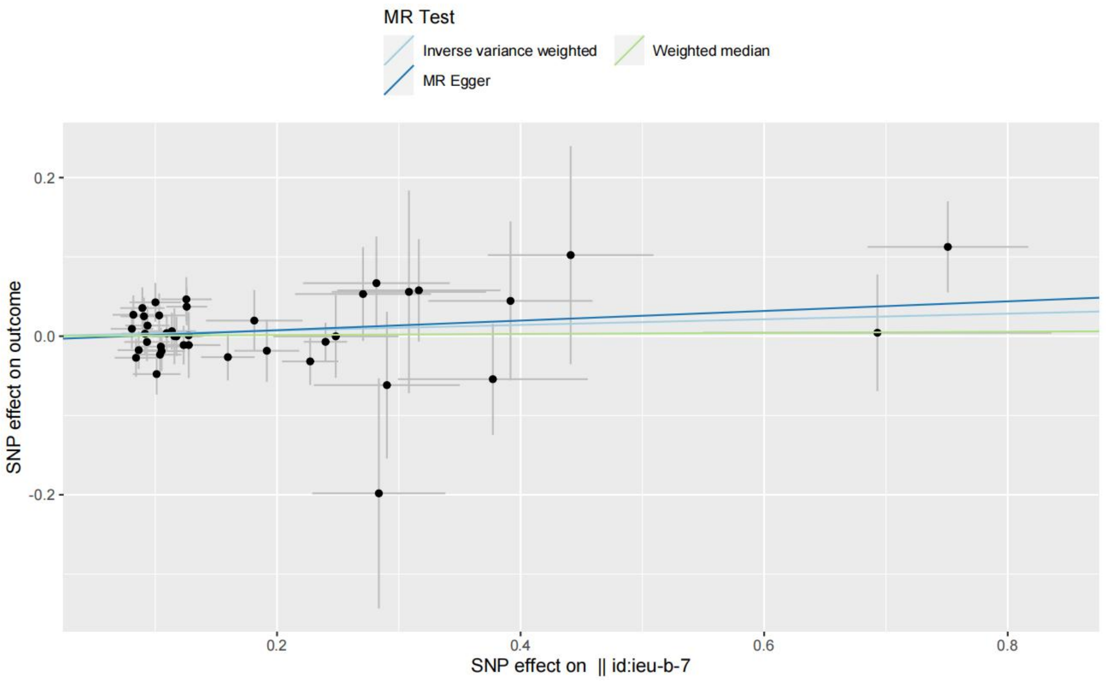

A. Scatter plot of PD on IL-2RA

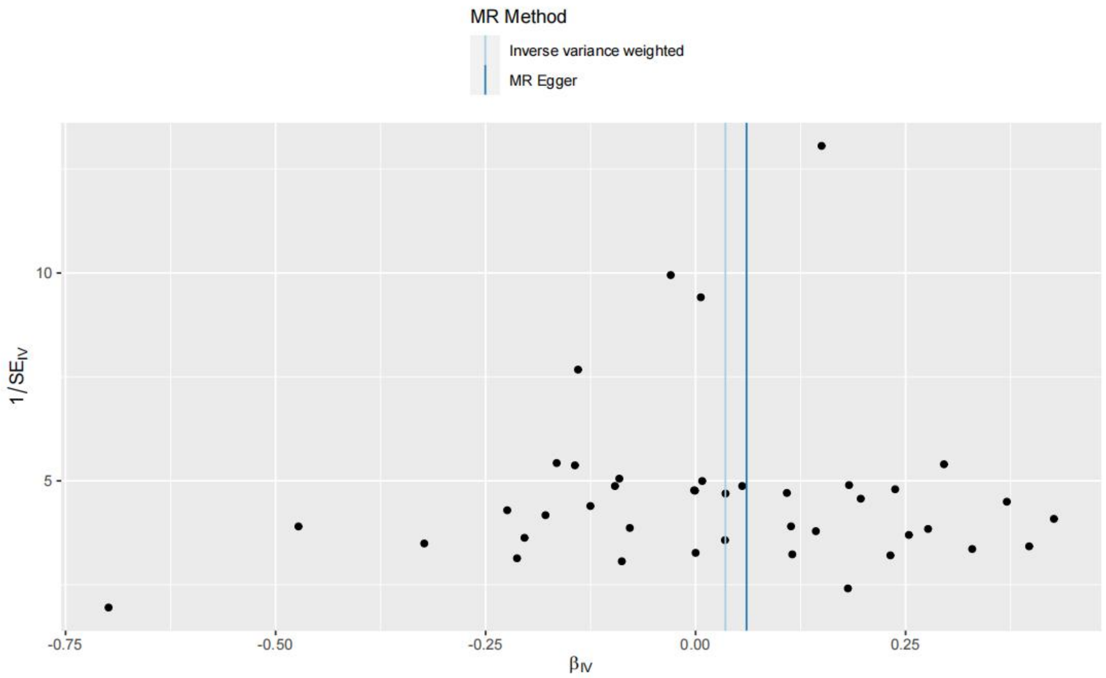

B. Funnel plot of PD on IL-2RA

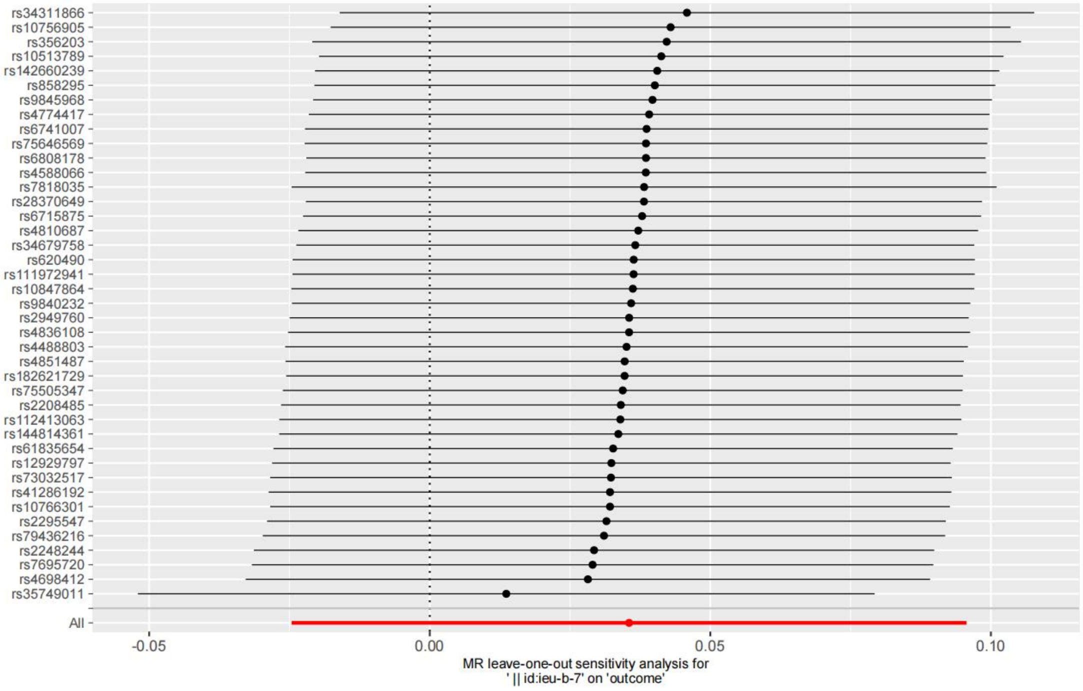

C. MR leave-one-out sensitivity analysis for PD on IL-2RA

eFigure 177. PD-associated SNPs with IL-4

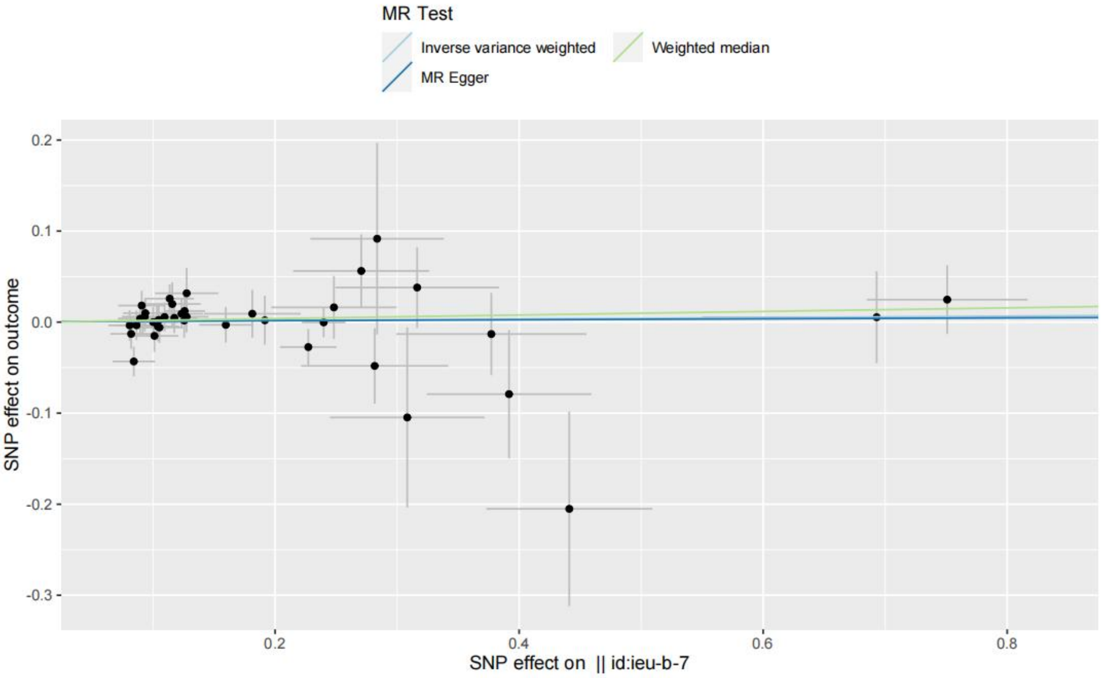

A. Scatter plot of PD on IL-4

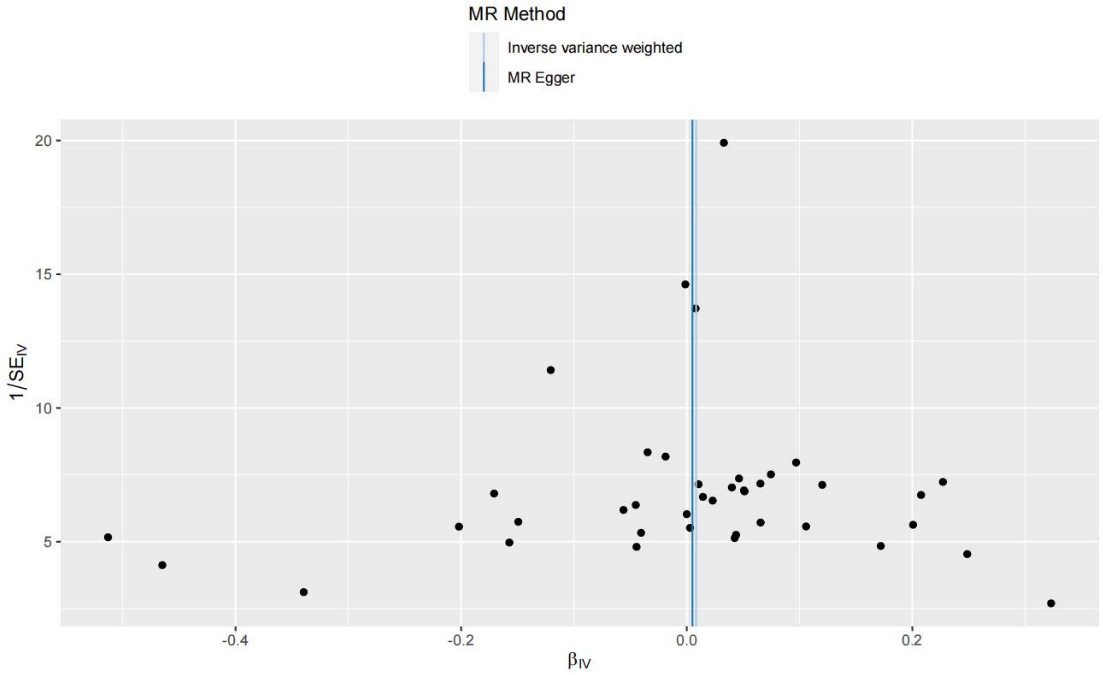

B. Funnel plot of PD on IL-4

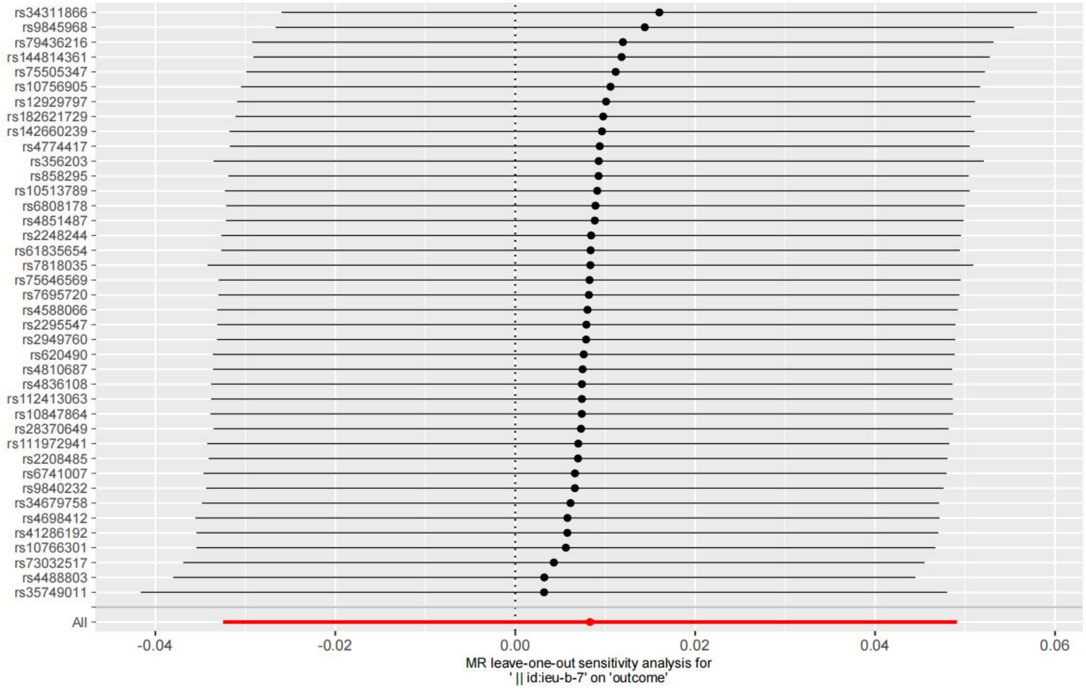

C. MR leave-one-out sensitivity analysis for PD on IL-4

eFigure 178. PD-associated SNPs with IL-5

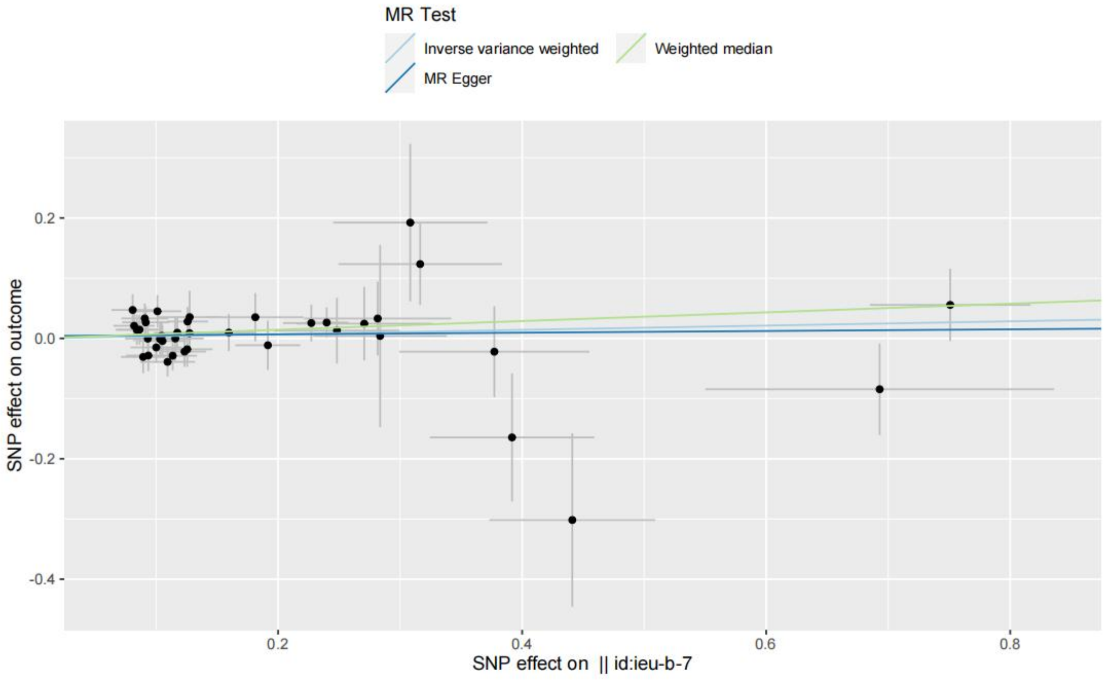

A. Scatter plot of PD on IL-5

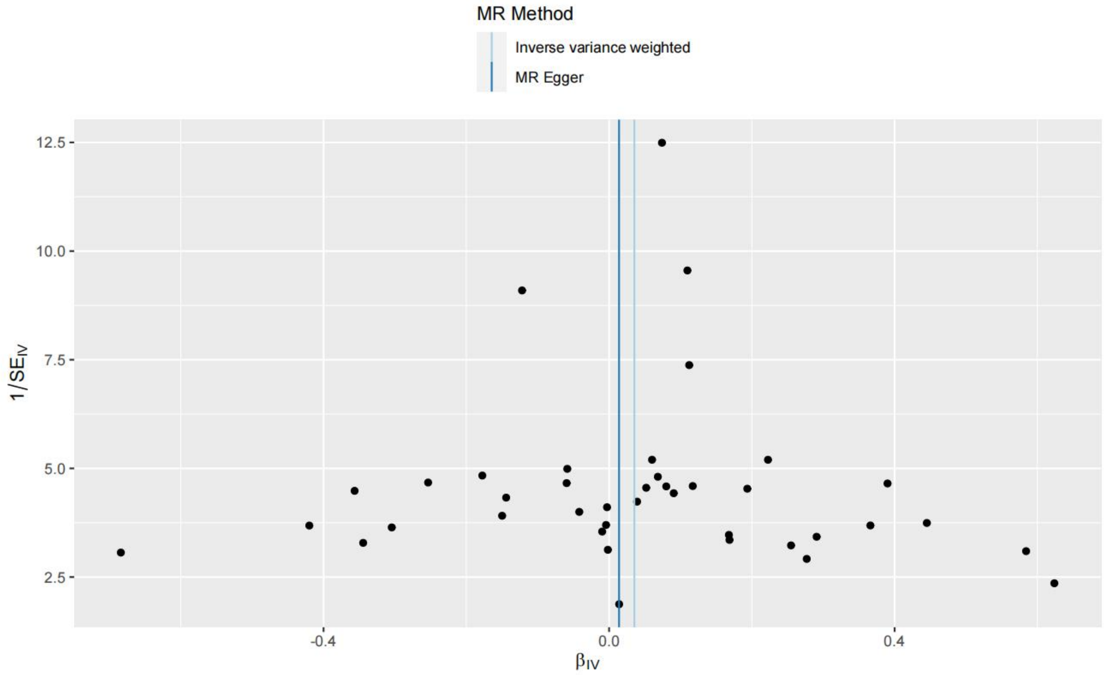

B. Funnel plot of PD on IL-5

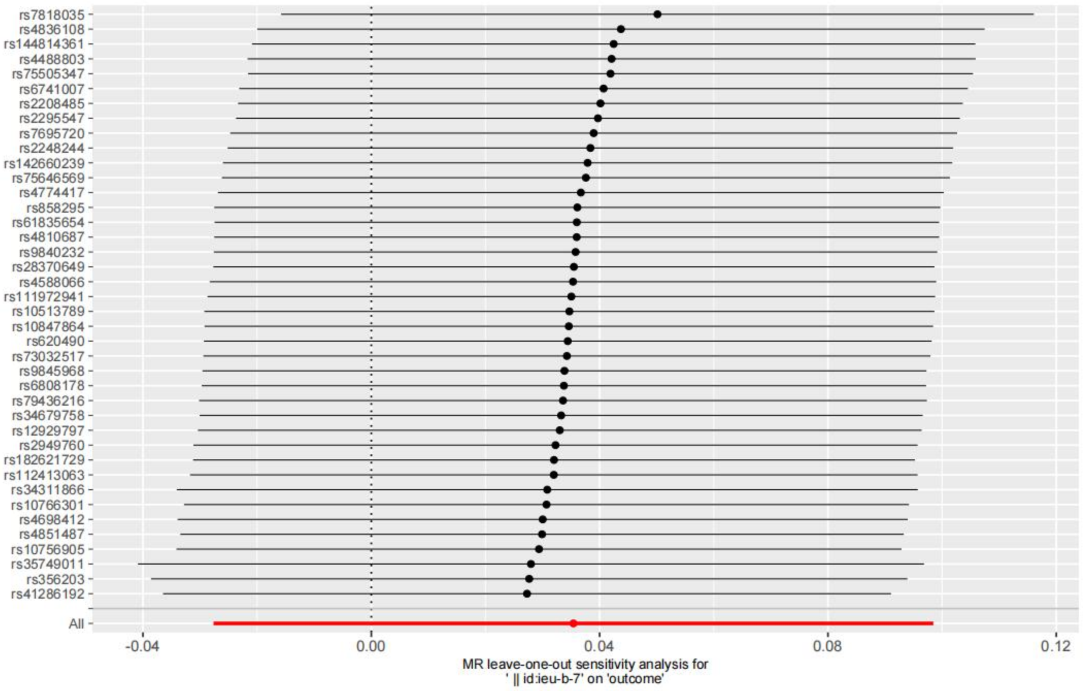

C. MR leave-one-out sensitivity analysis for PD on IL-5

eFigure 179. PD-associated SNPs with IL-6

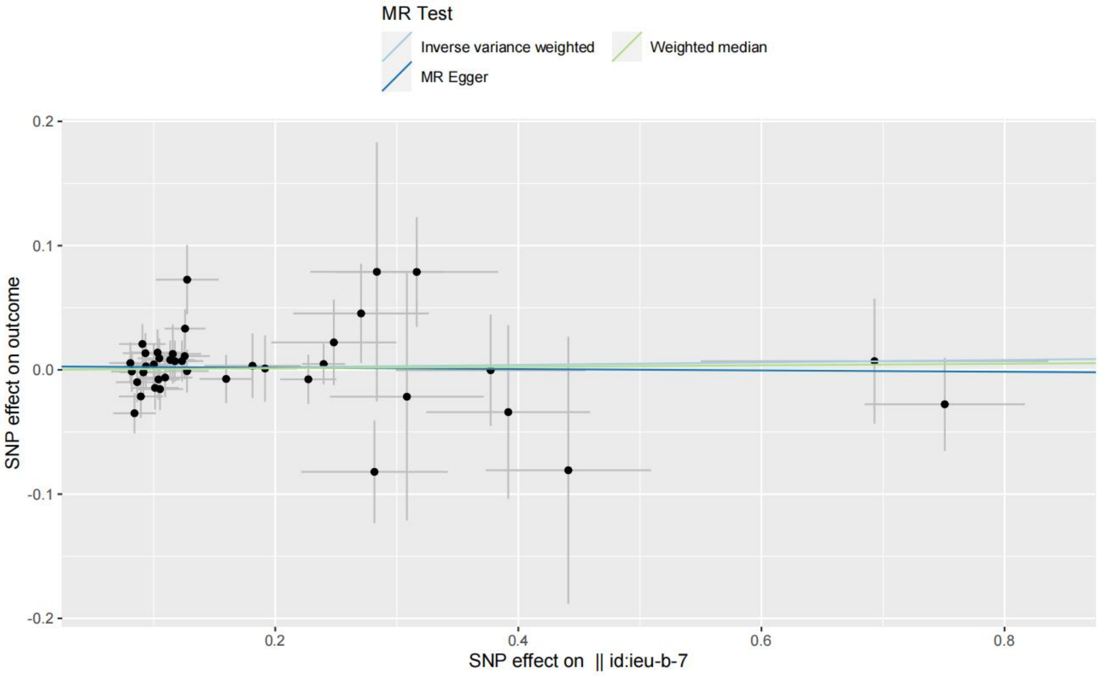

A. Scatter plot of PD on IL-6

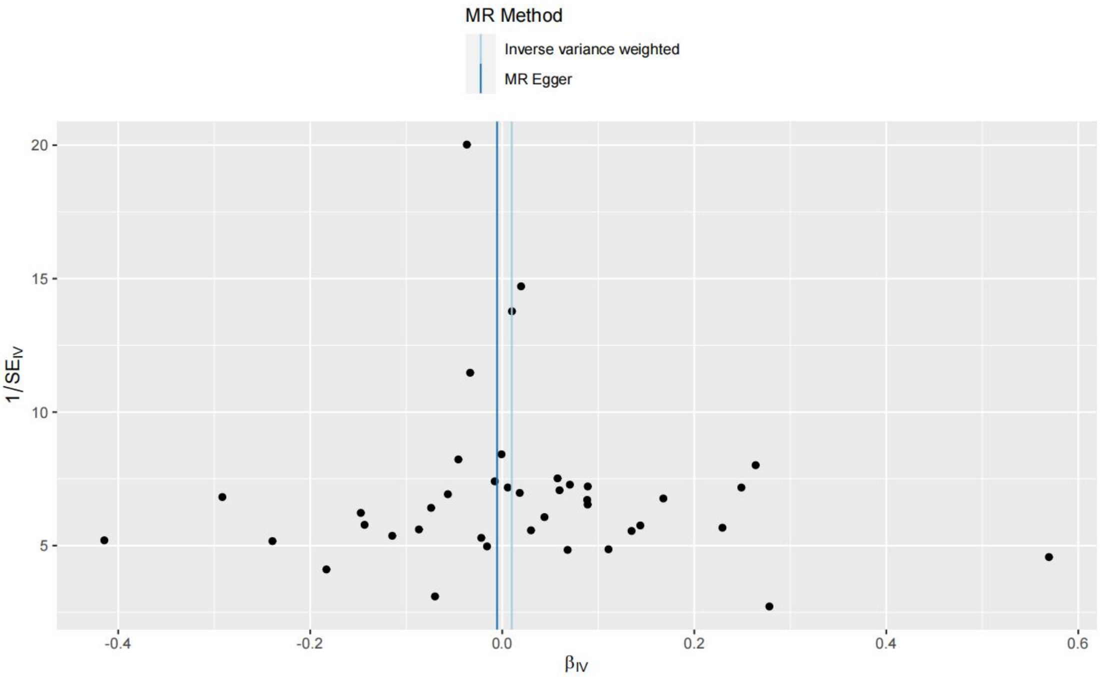

B. Funnel plot of PD on IL-6

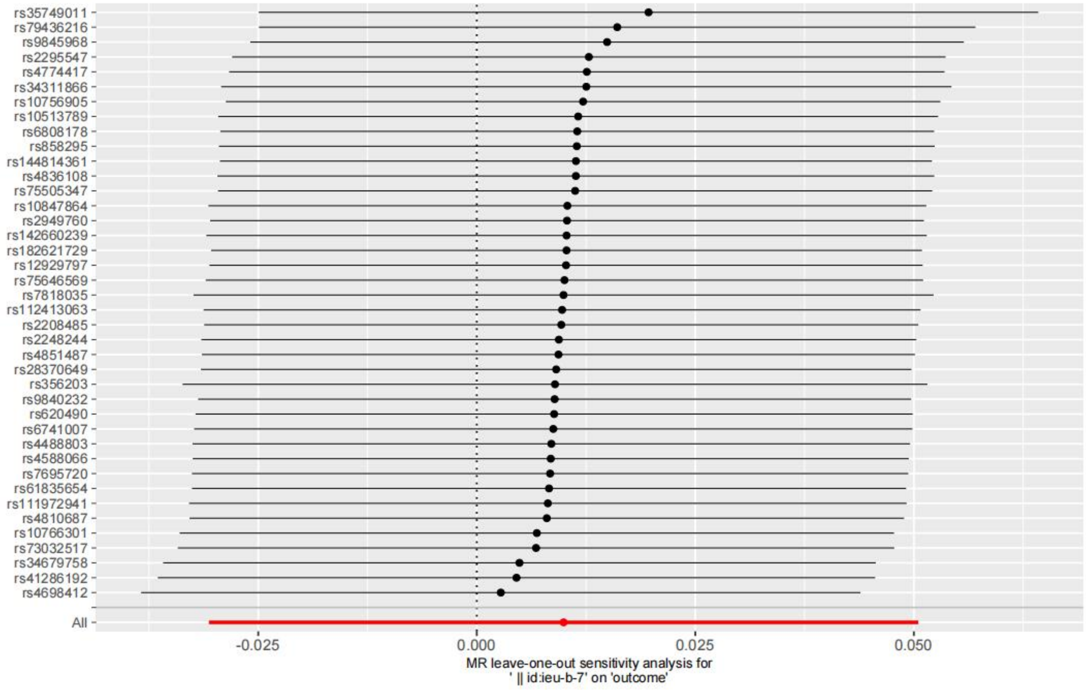

C. MR leave-one-out sensitivity analysis for PD on IL-6

eFigure 180. PD-associated SNPs with IL-7

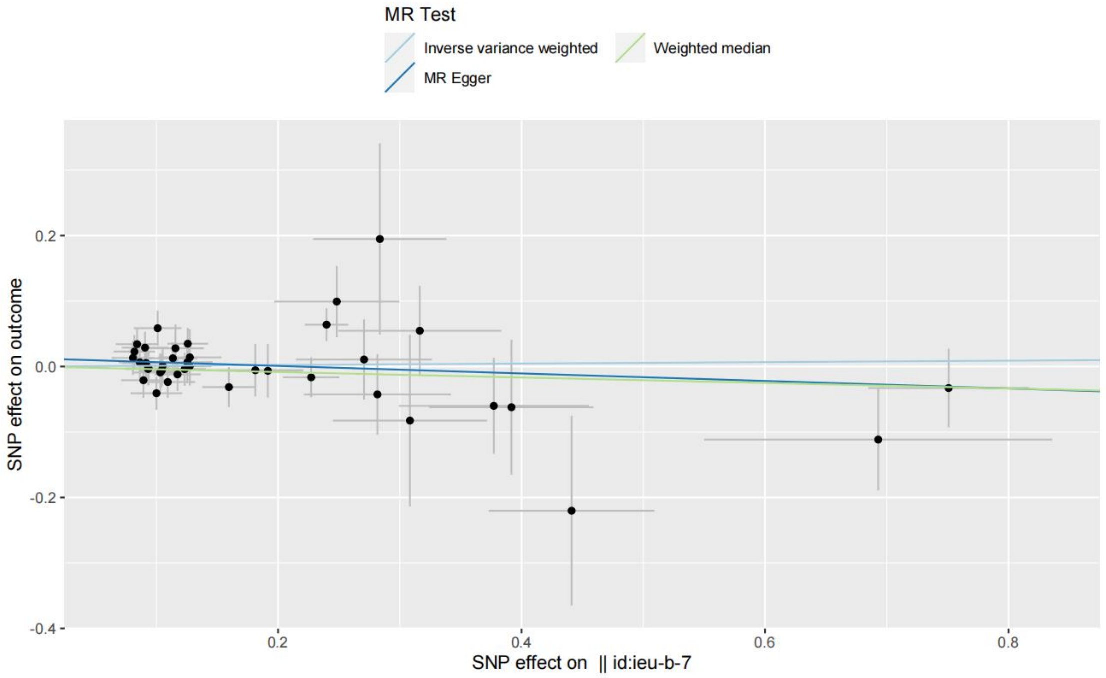

A. Scatter plot of PD on IL-7

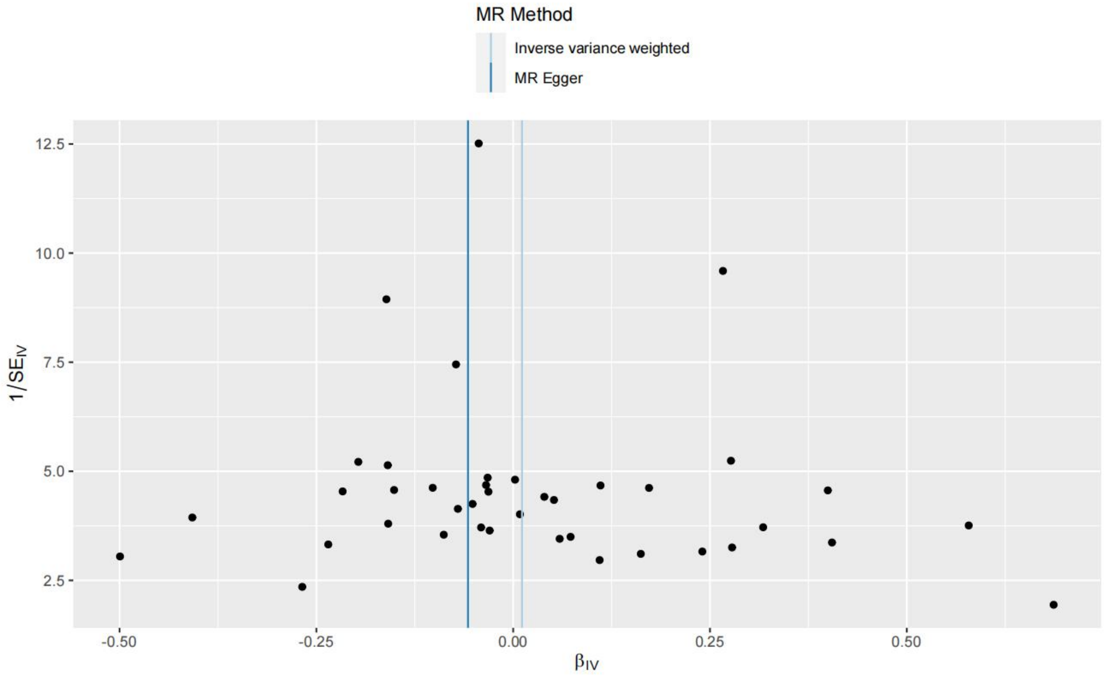

B. Funnel plot of PD on IL-7

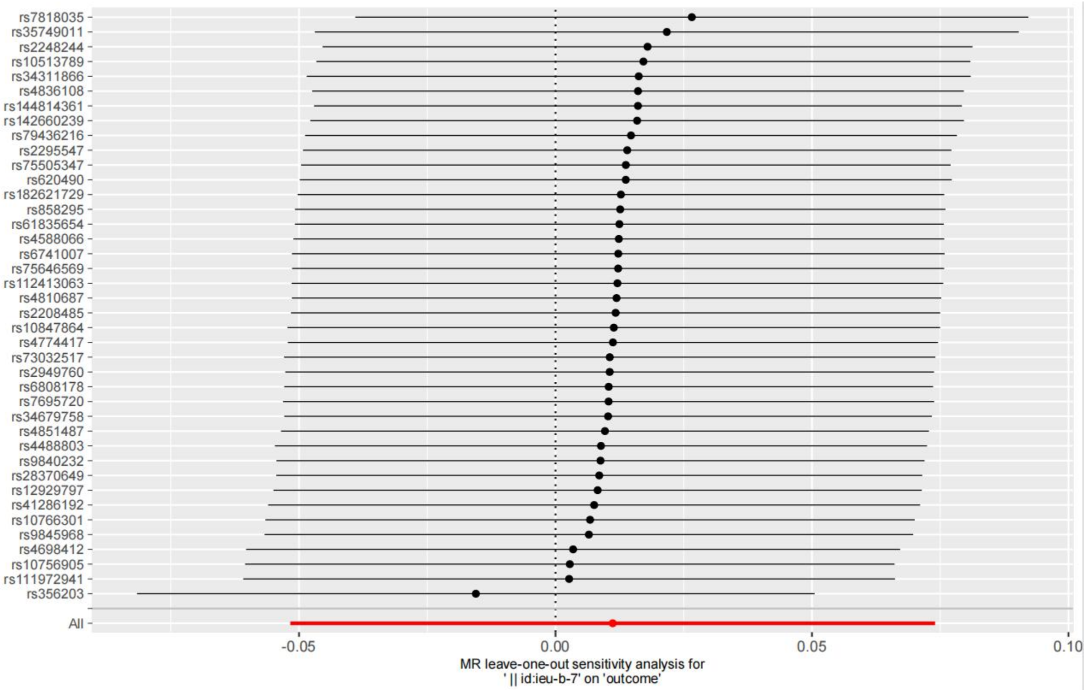

C. MR leave-one-out sensitivity analysis for PD on IL-7

eFigure 182. PD-associated SNPs with IL-9

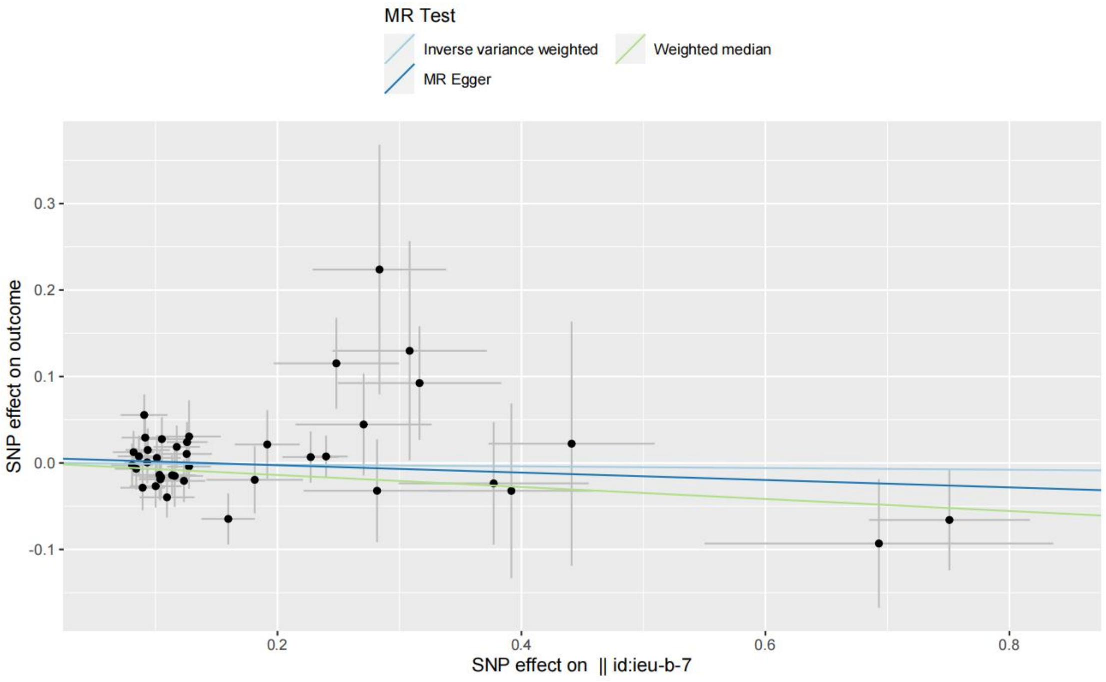

A. Scatter plot of PD on IL-9

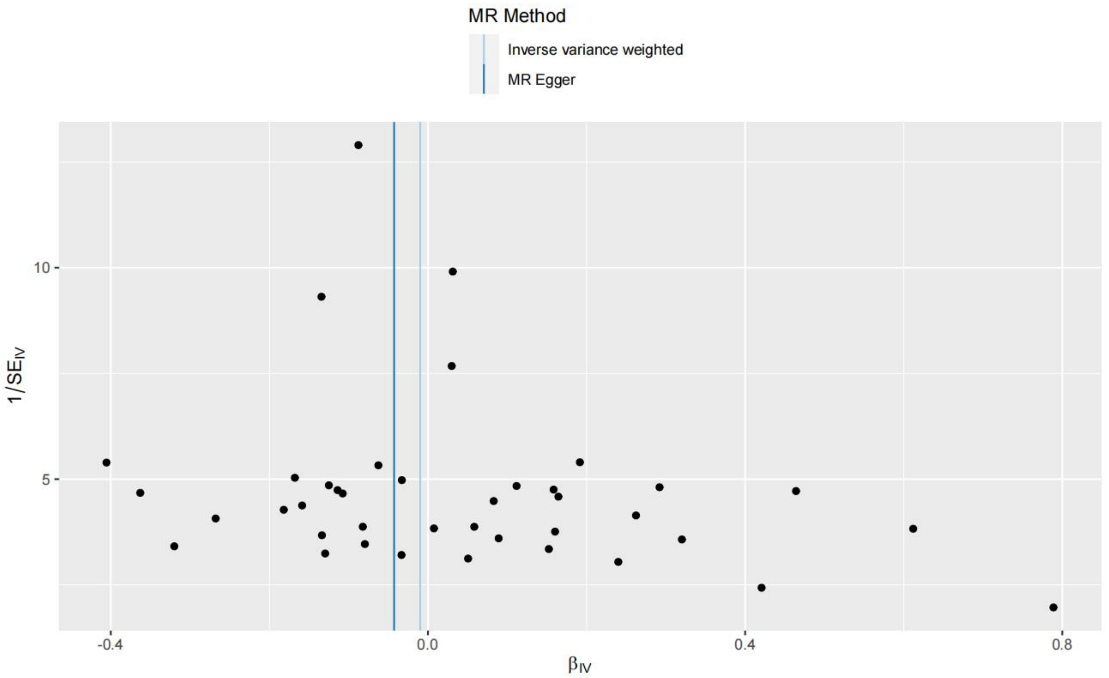

B. Funnel plot of PD on IL-9

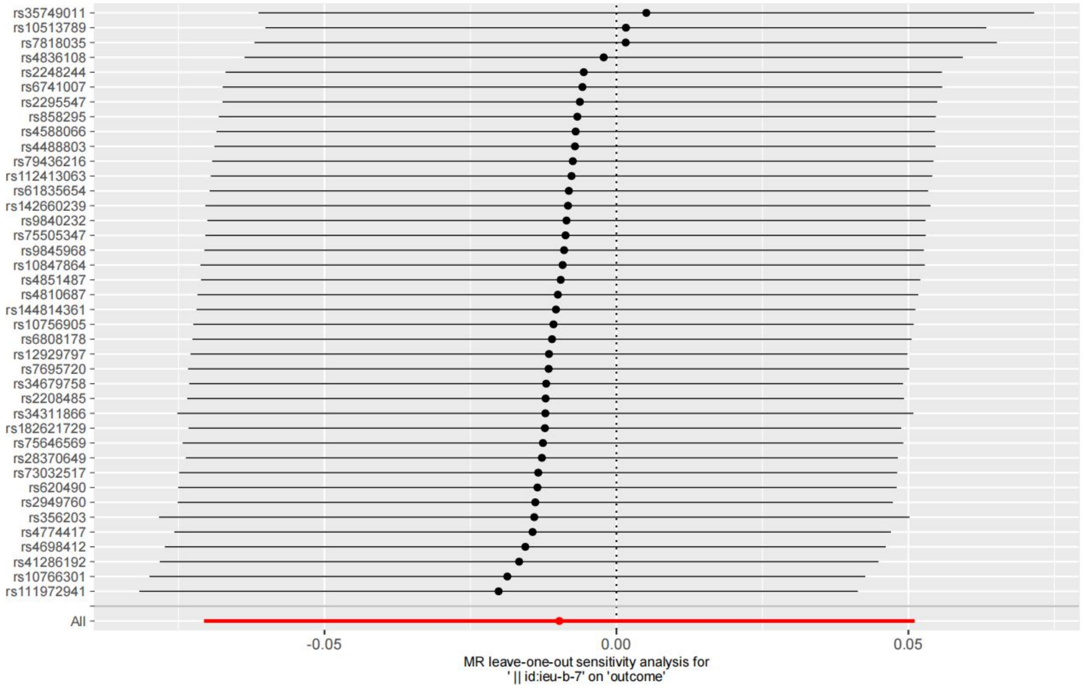

C. MR leave-one-out sensitivity analysis for PD on IL-9

eFigure 183. PD-associated SNPs with IL-10

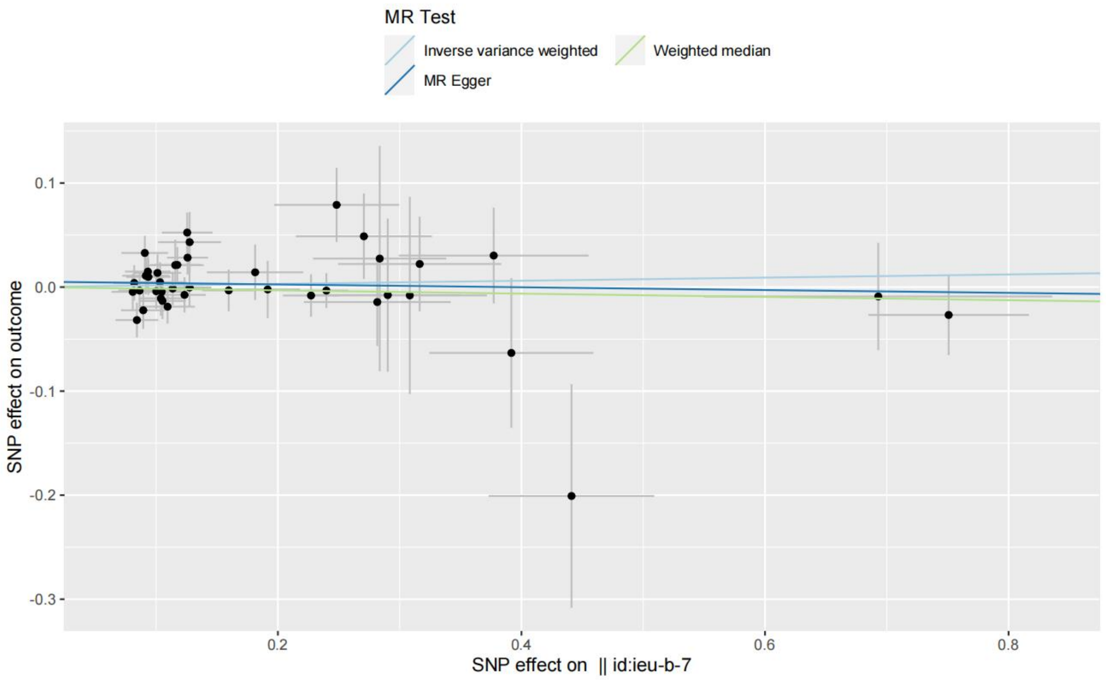

A. Scatter plot of PD on IL-10

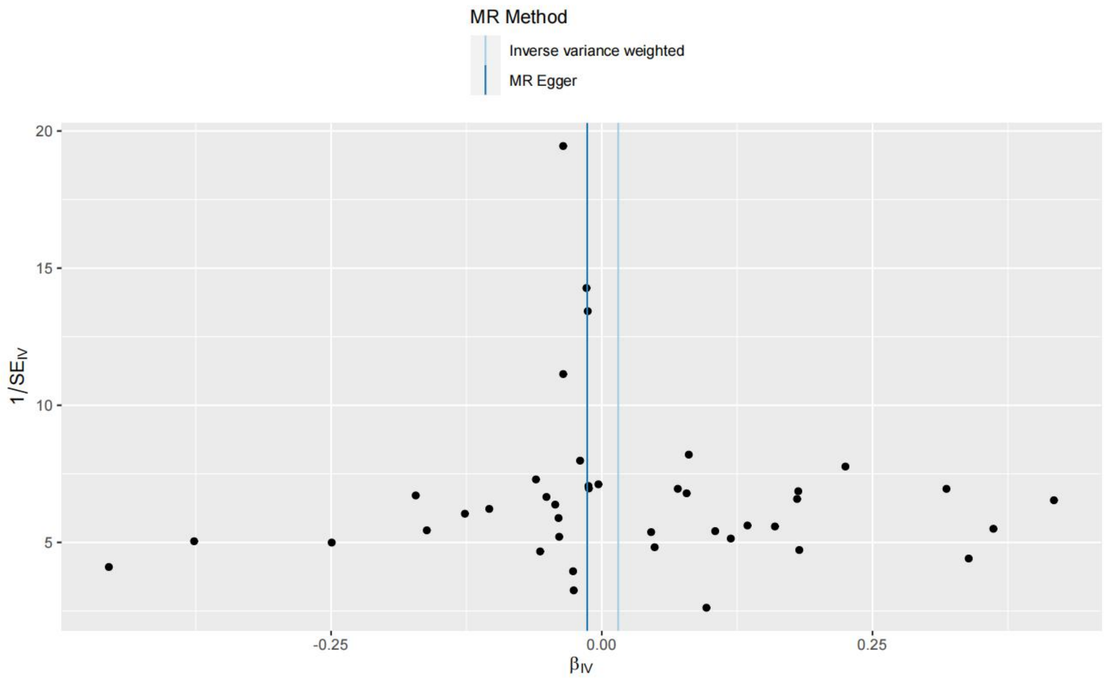

B. Funnel plot of PD on IL-10

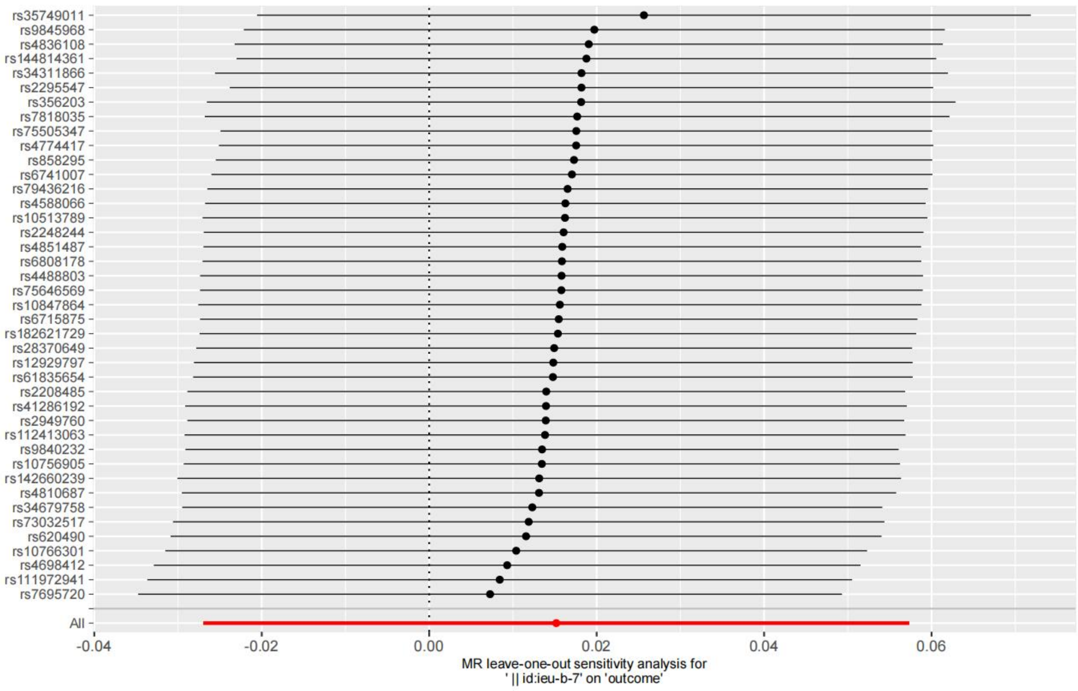

C. MR leave-one-out sensitivity analysis for PD on IL-10

eFigure 184. PD-associated SNPs with IL-12

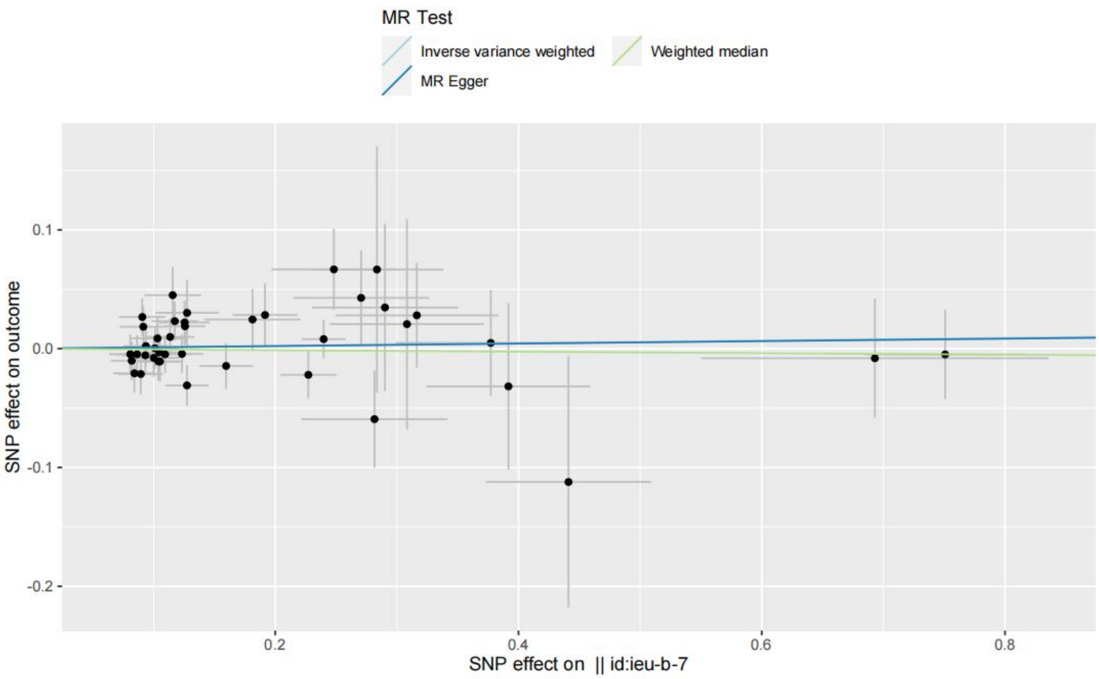

A. Scatter plot of PD on IL-12

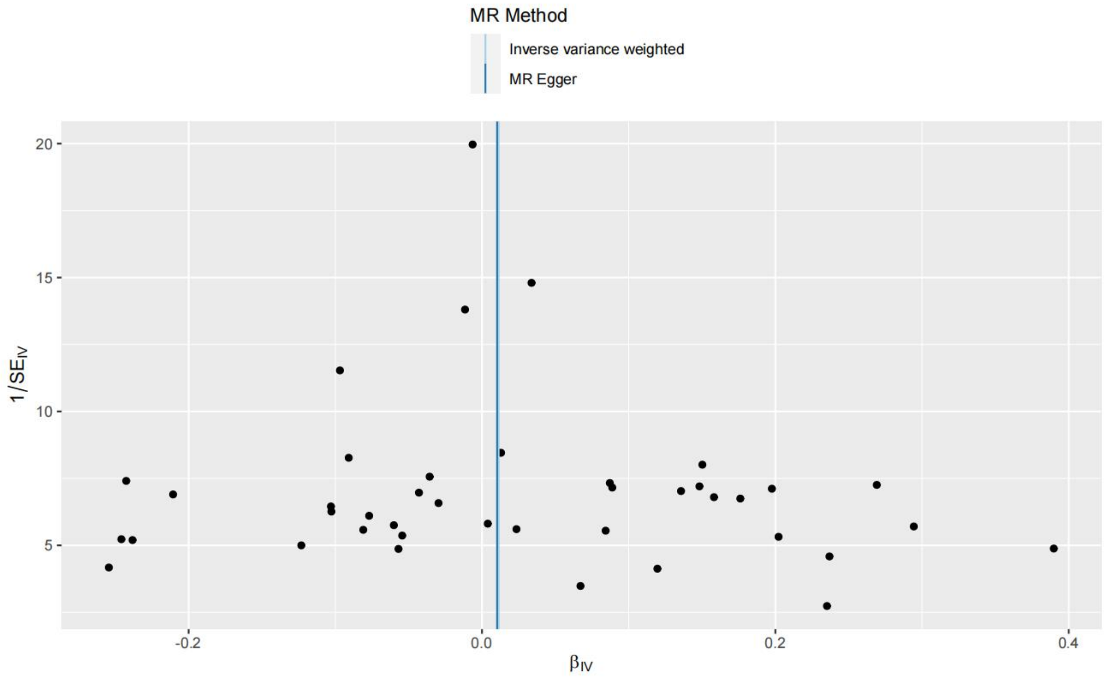

B. Funnel plot of PD on IL-12

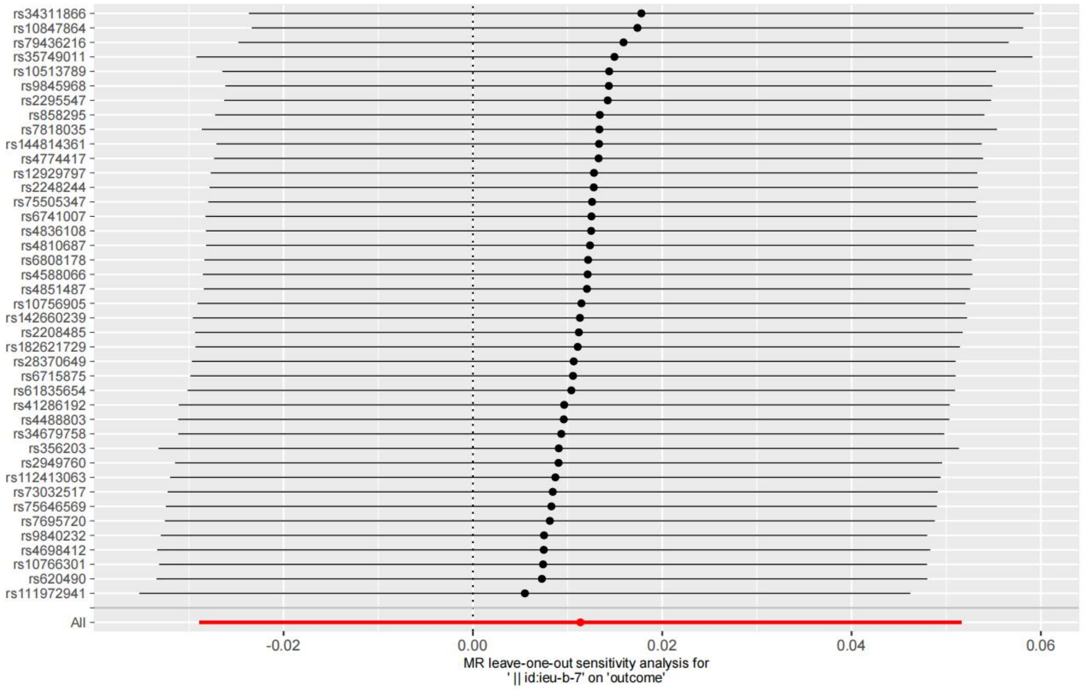

C. MR leave-one-out sensitivity analysis for PD on IL-12

eFigure 185. PD-associated SNPs with IL-13

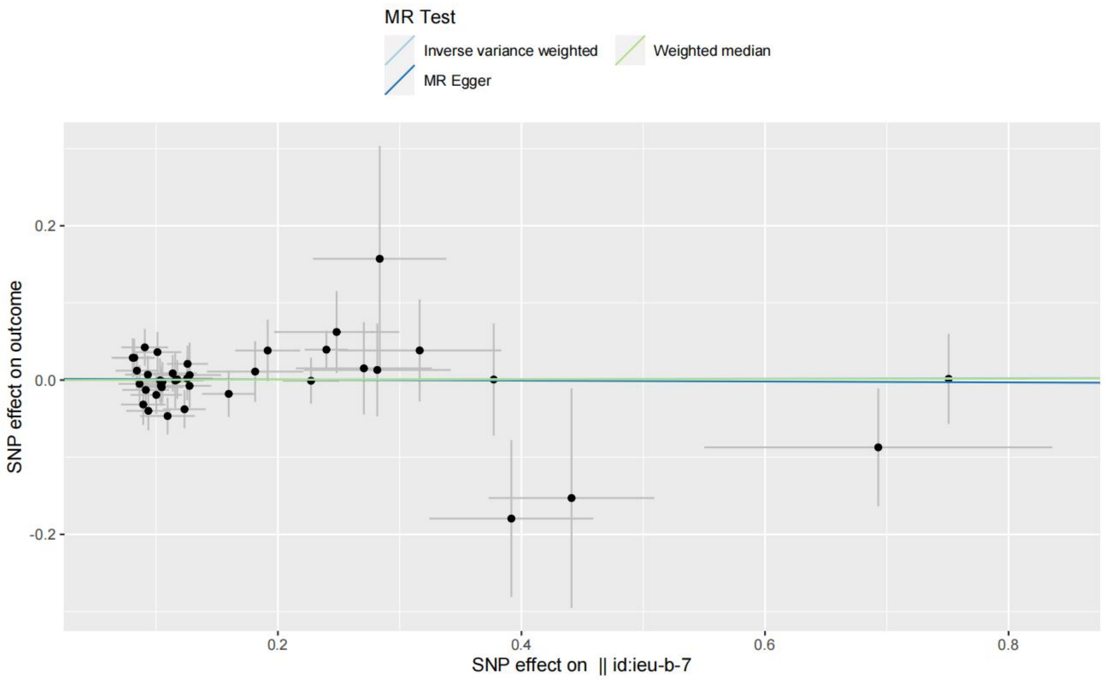

A. Scatter plot of PD on IL-13

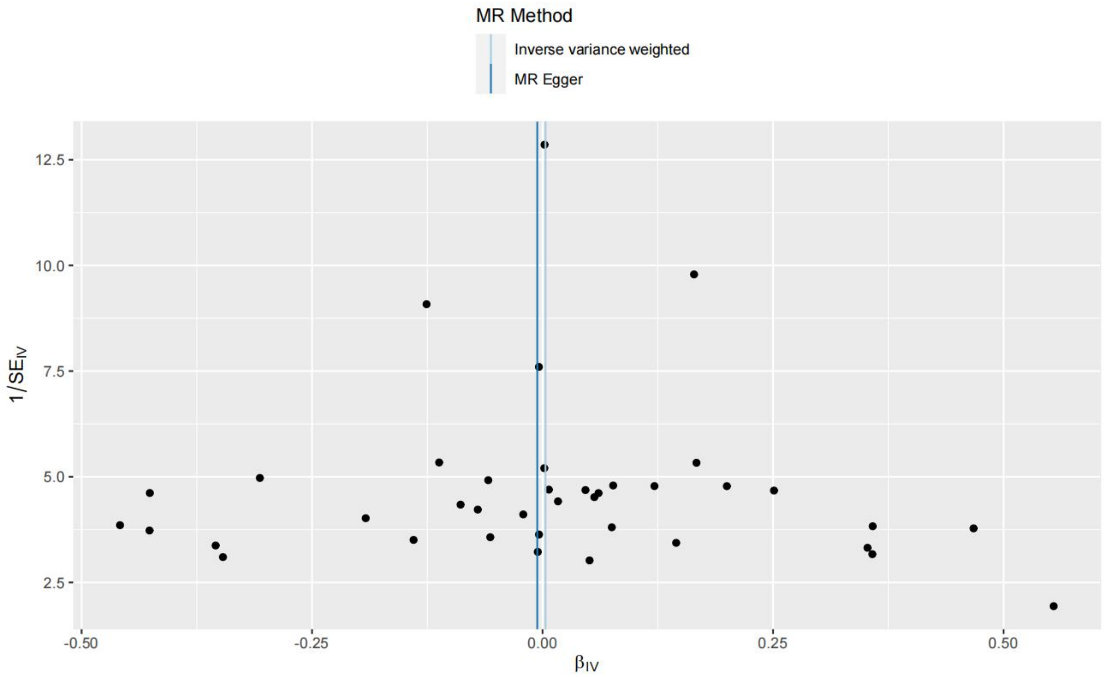

B. Funnel plot of PD on IL-13

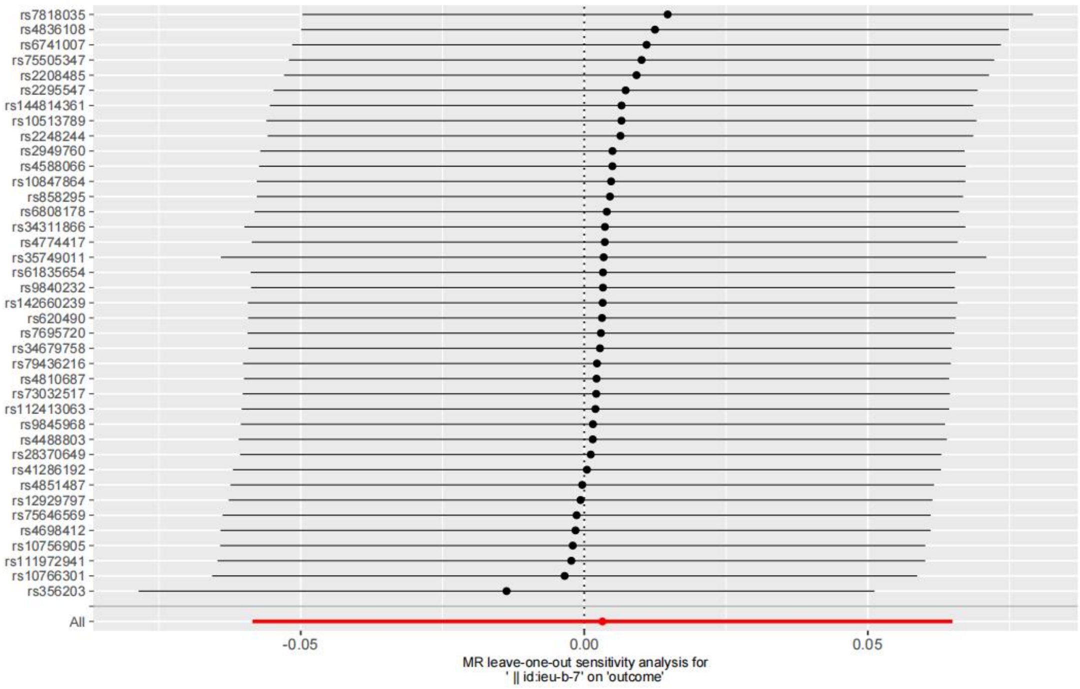

C. MR leave-one-out sensitivity analysis for PD on IL-13

eFigure 186. PD-associated SNPs with IL-16

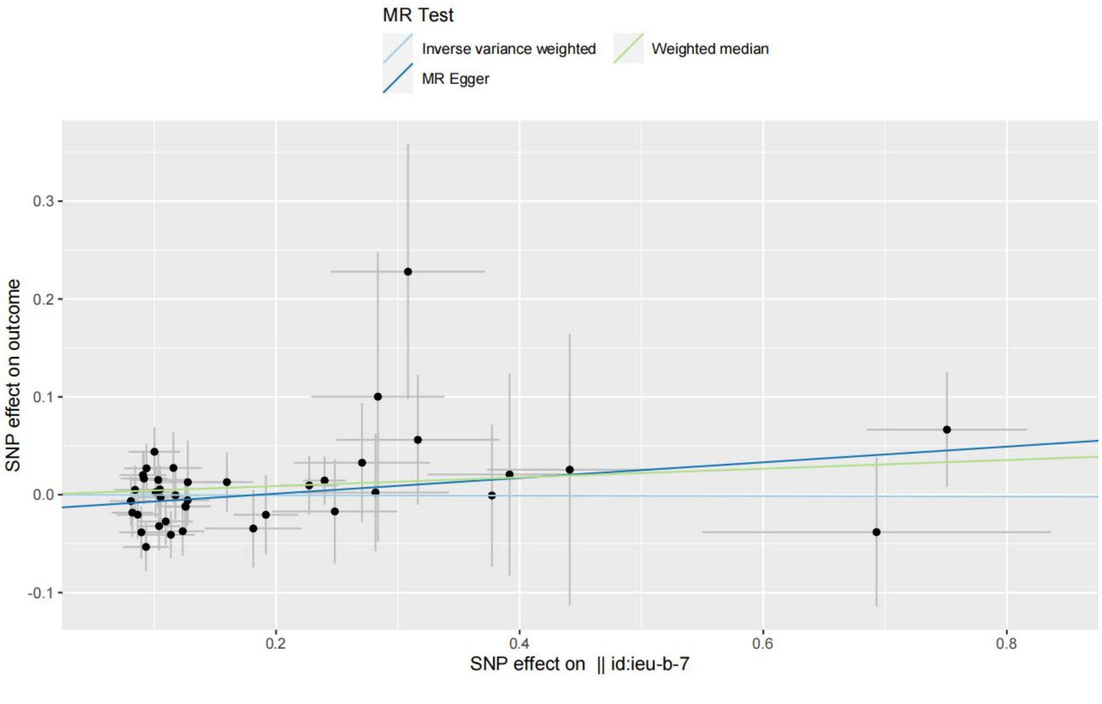

A. Scatter plot of PD on IL-16

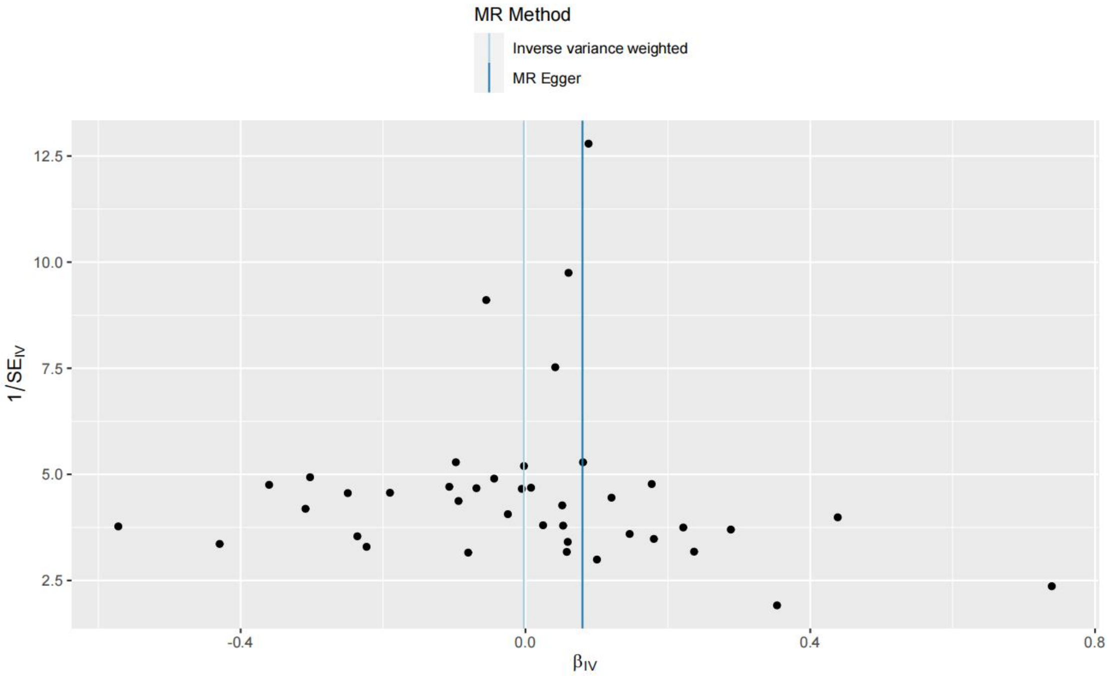

B. Funnel plot of PD on IL-16

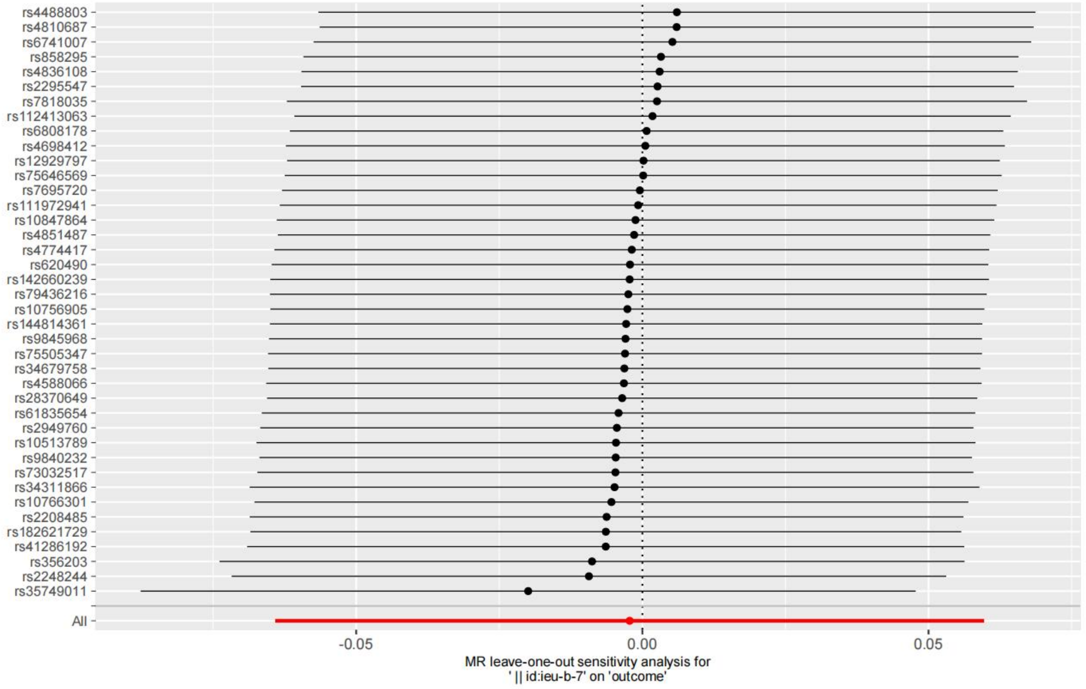

C. MR leave-one-out sensitivity analysis for PD on IL-16

eFigure 187. PD-associated SNPs with IL-17

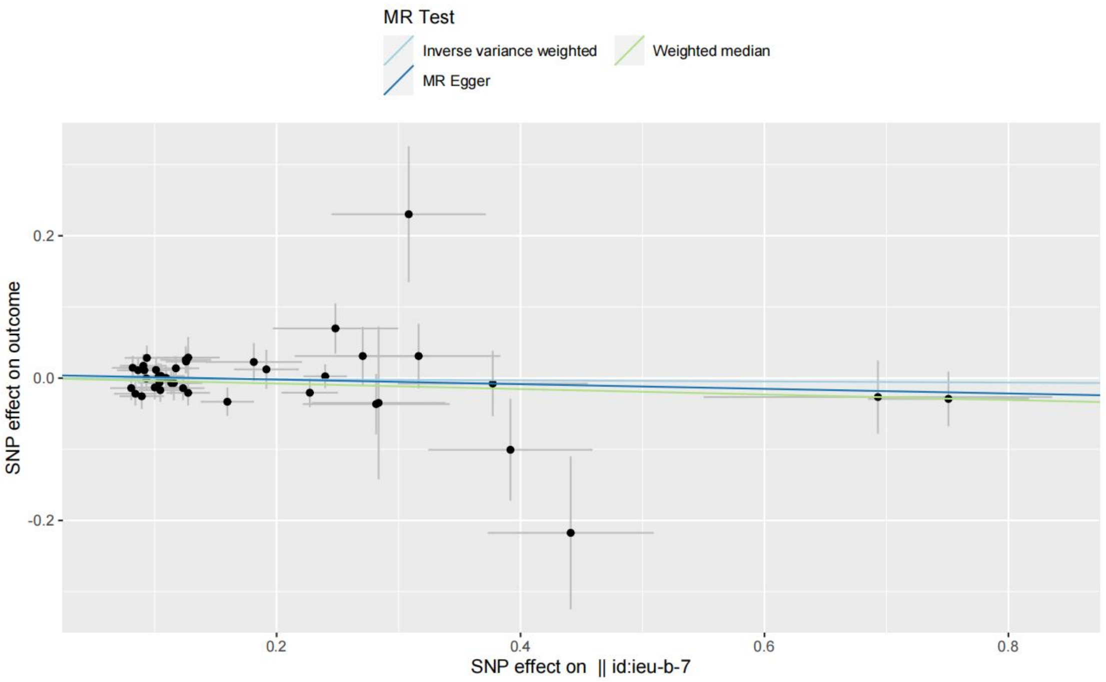

A. Scatter plot of PD on IL-17

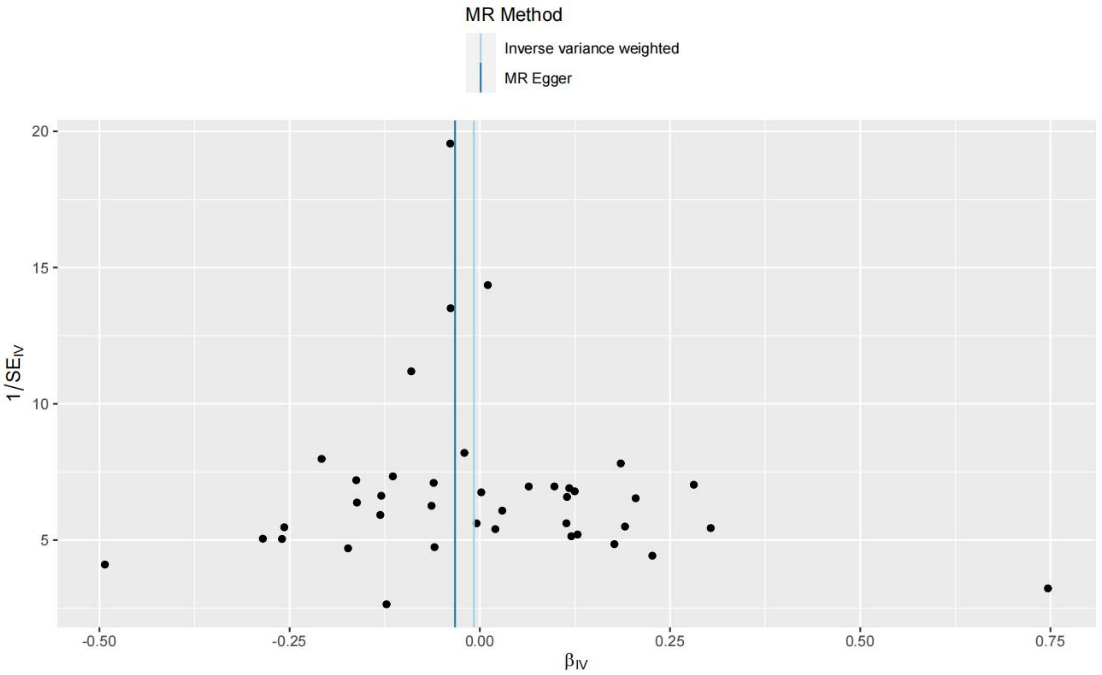

B. Funnel plot of PD on IL-17

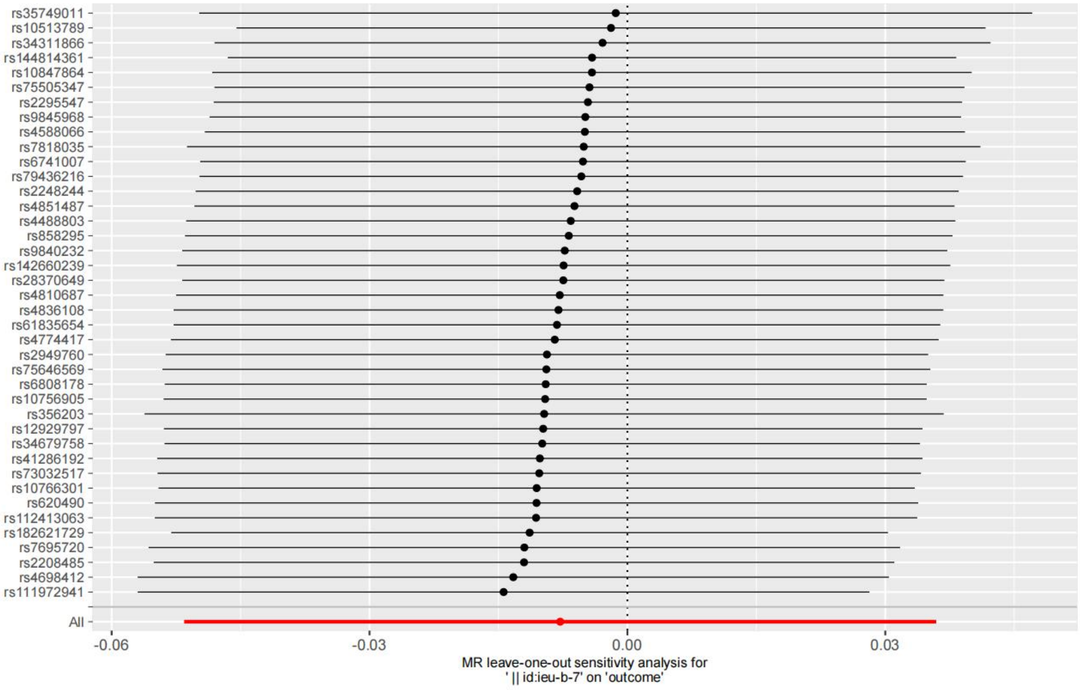

C. MR leave-one-out sensitivity analysis for PD on IL-17

eFigure 188. PD-associated SNPs with IL-18

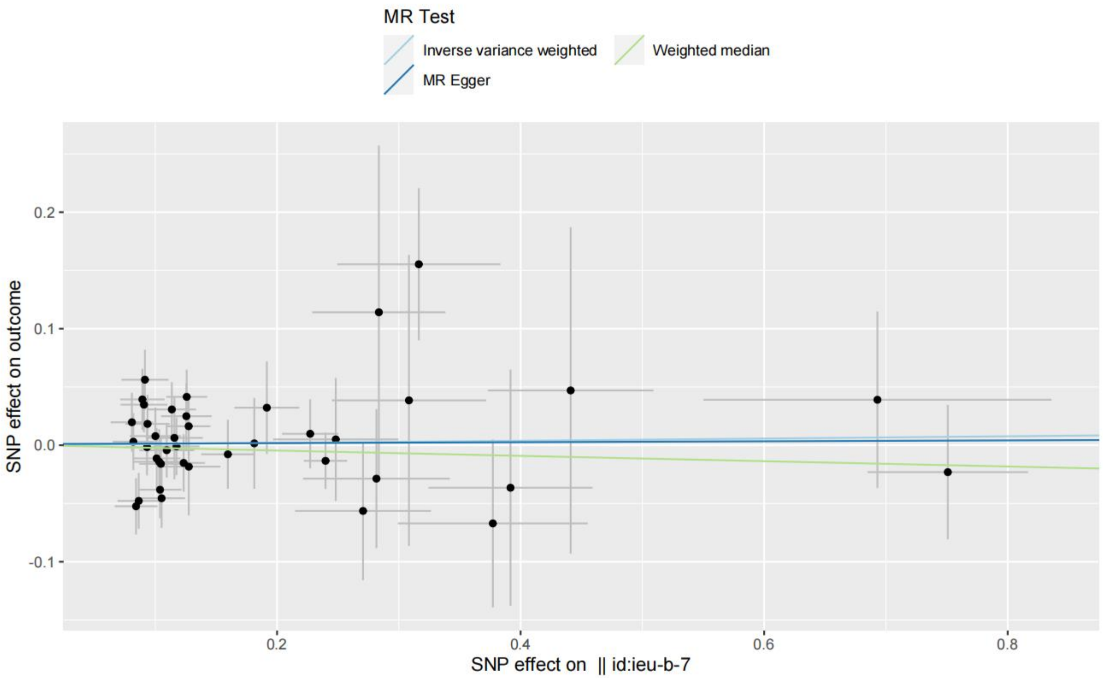

A. Scatter plot of PD on IL-18

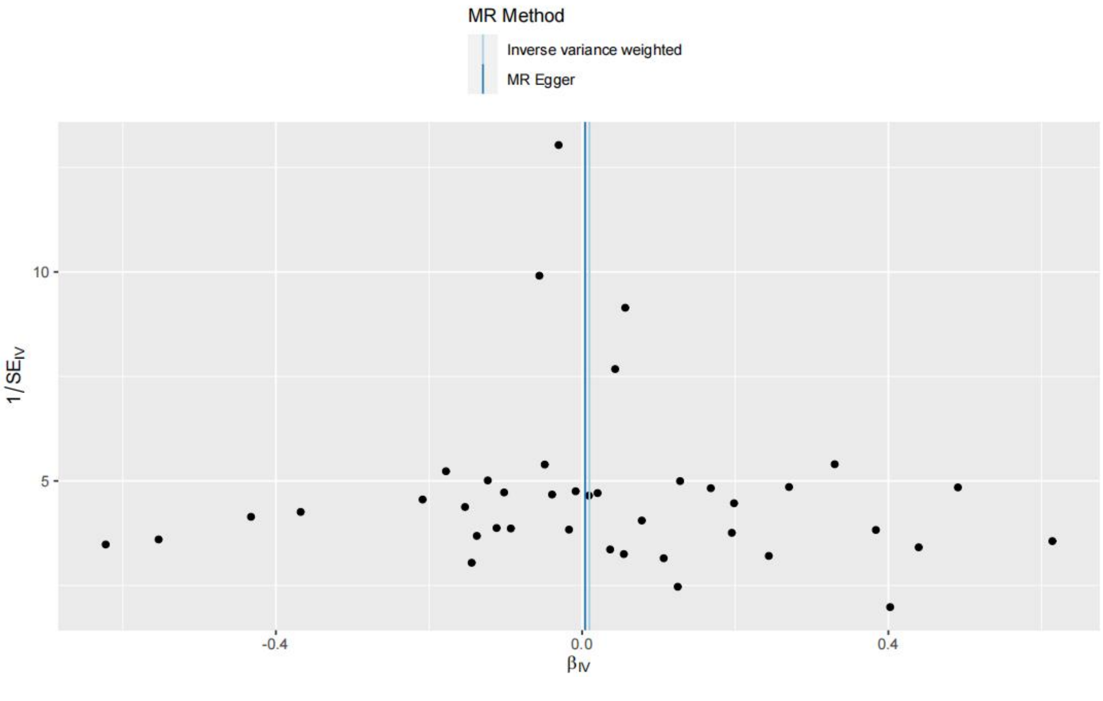

B. Funnel plot of PD on IL-18

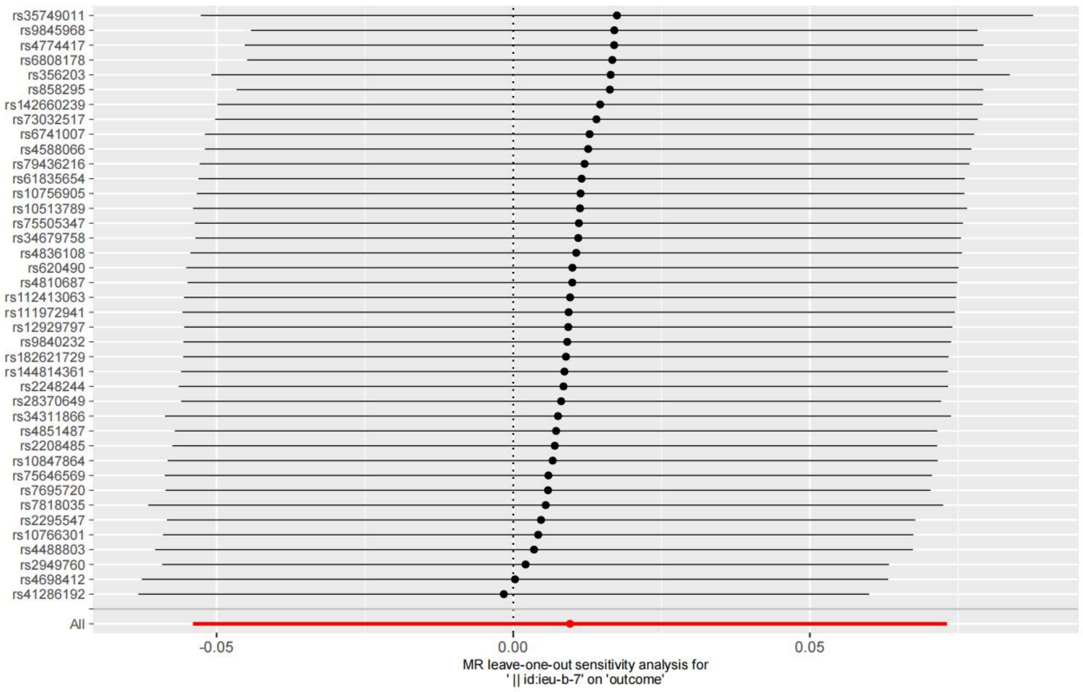

C. MR leave-one-out sensitivity analysis for PD on IL-18

eFigure 189. PD-associated SNPs with IP-10

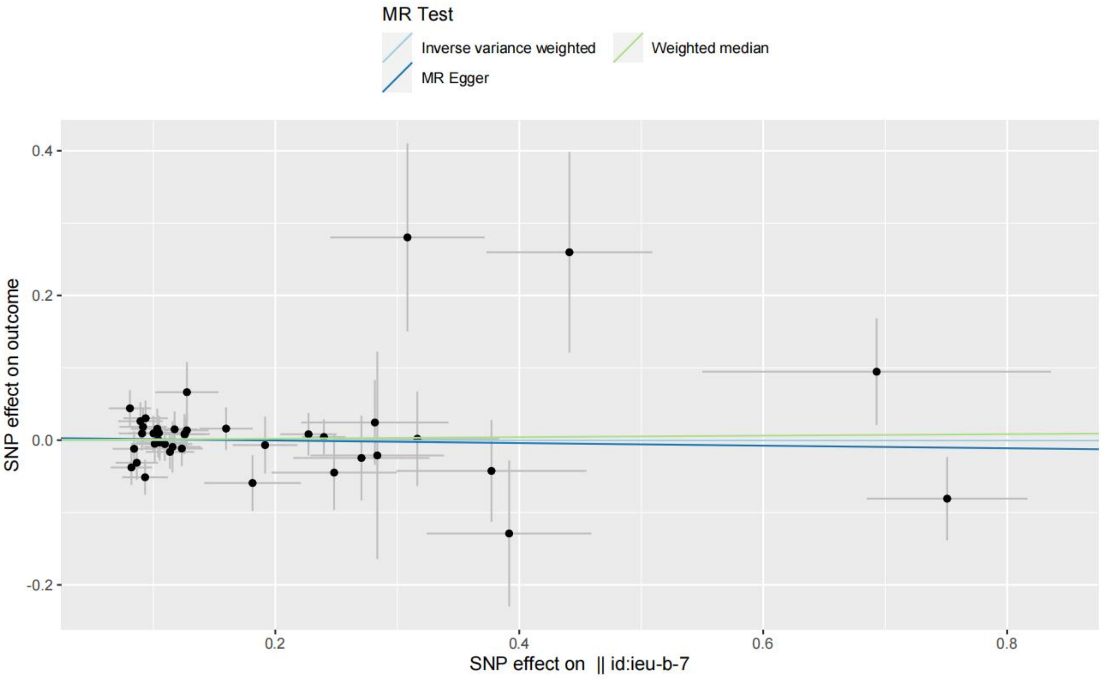

A. Scatter plot of PD on IP-10

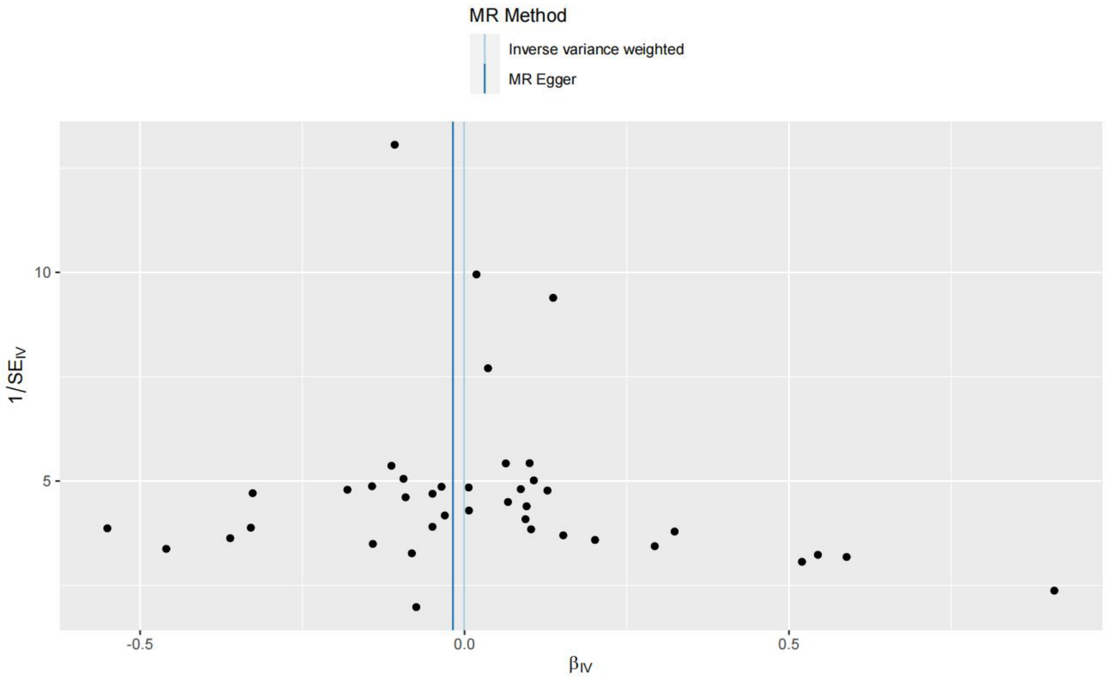

B. Funnel plot of PD on IP-10

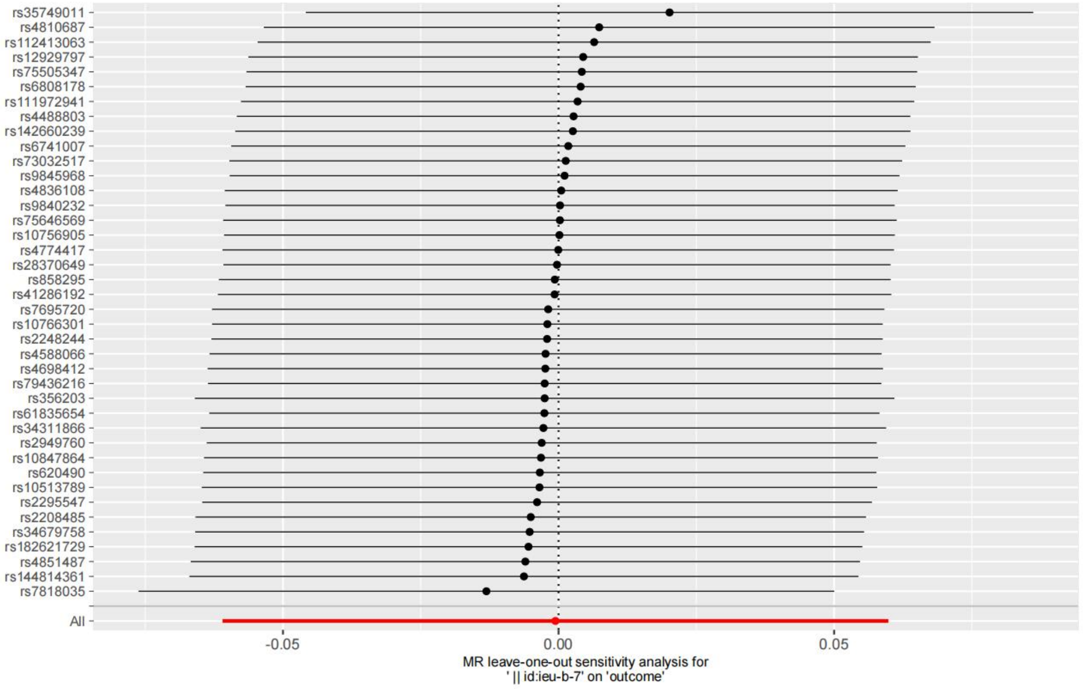

C. MR leave-one-out sensitivity analysis for PD on IP-10

eFigure 190. PD-associated SNPs with M-CSF

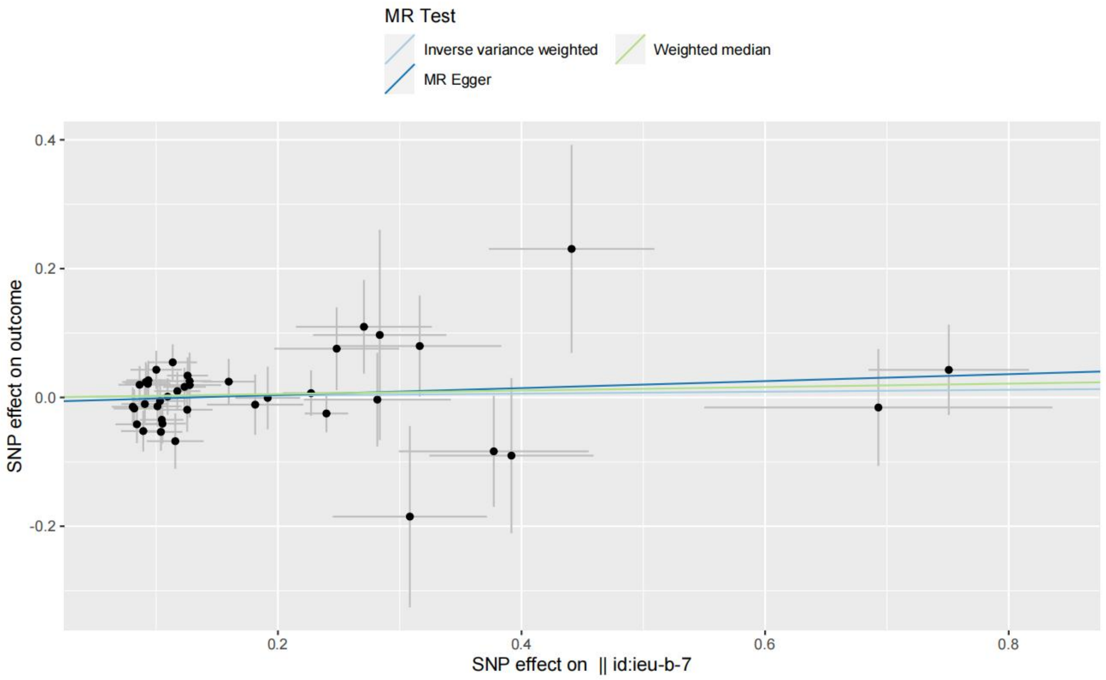

A. Scatter plot of PD on M-CSF

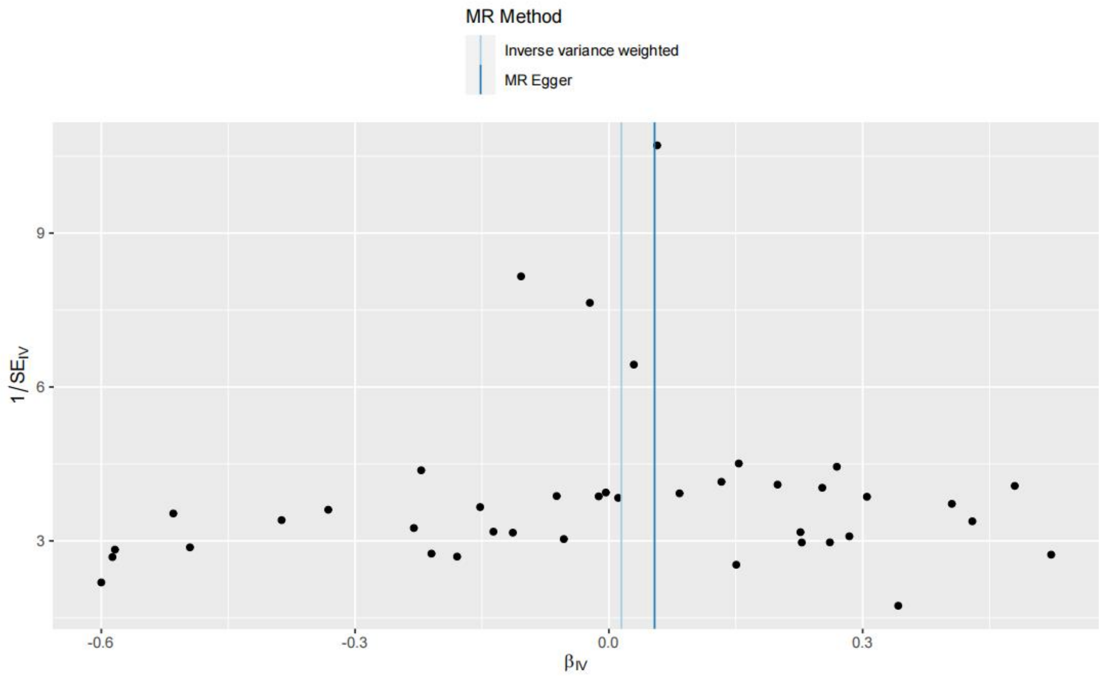

B. Funnel plot of PD on M-CSF

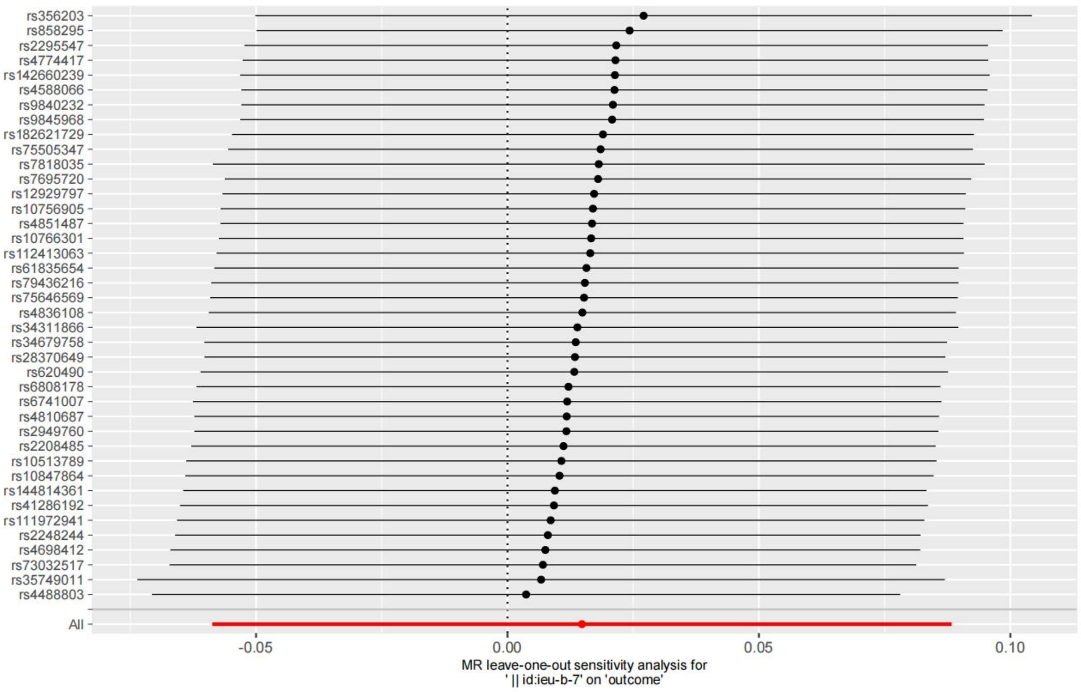

C. MR leave-one-out sensitivity analysis for PD on M-CSF

eFigure 191. PD-associated SNPs with MCP-1-MCAF

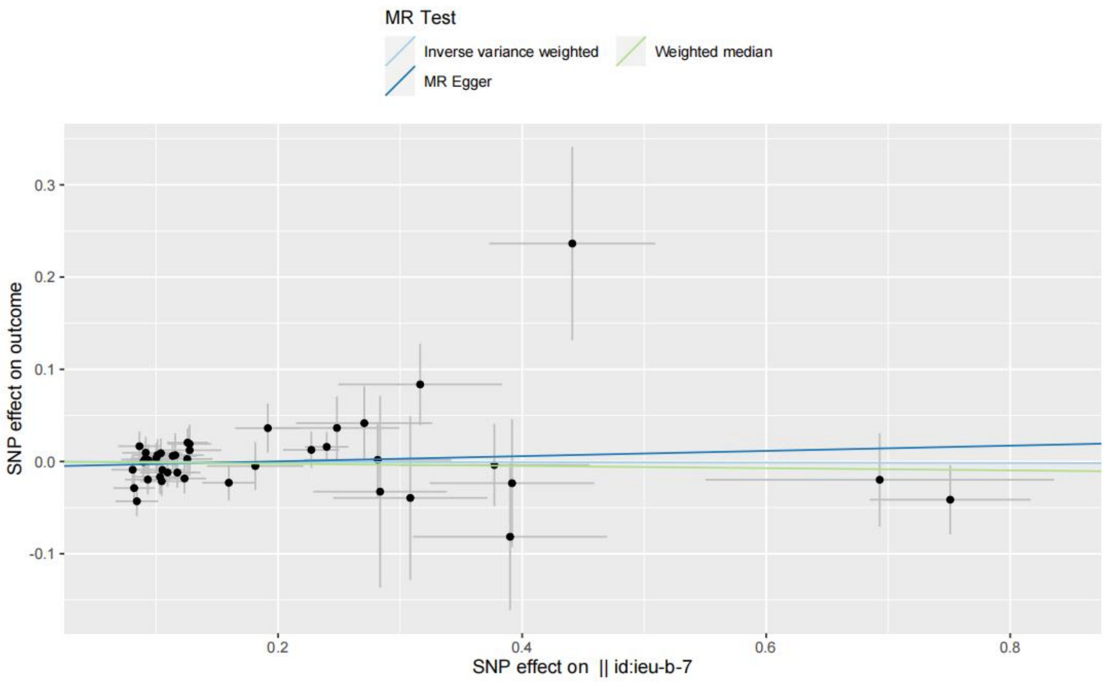

A. Scatter plot of PD on MCP-1-MCAF

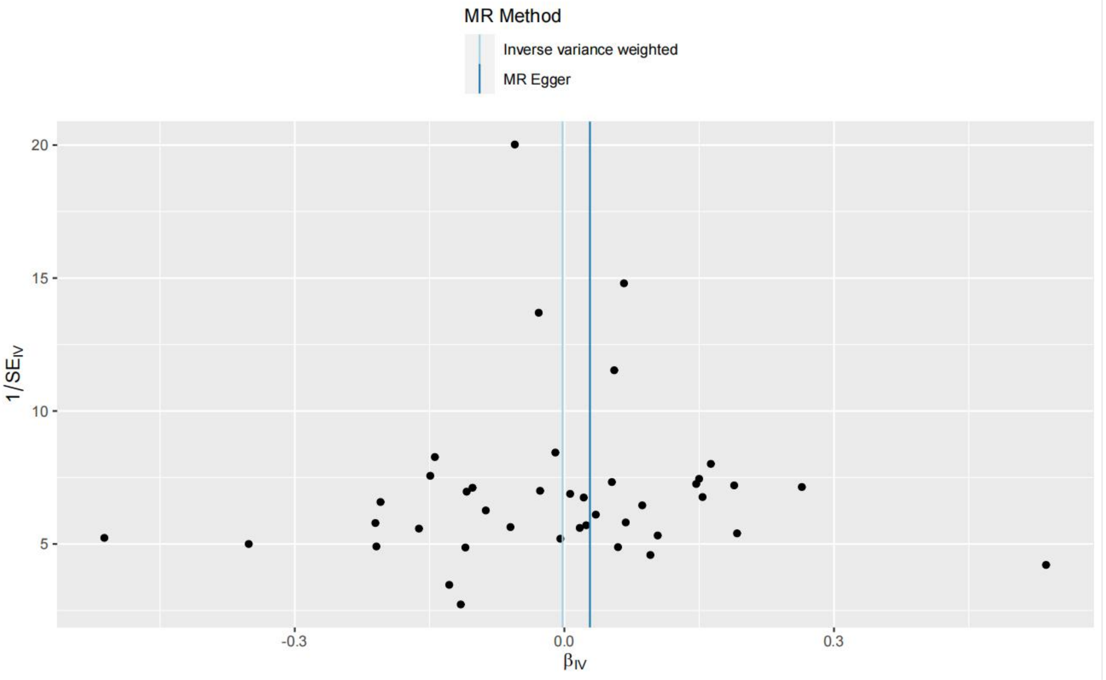

B. Funnel plot of PD on MCP-1-MCAF

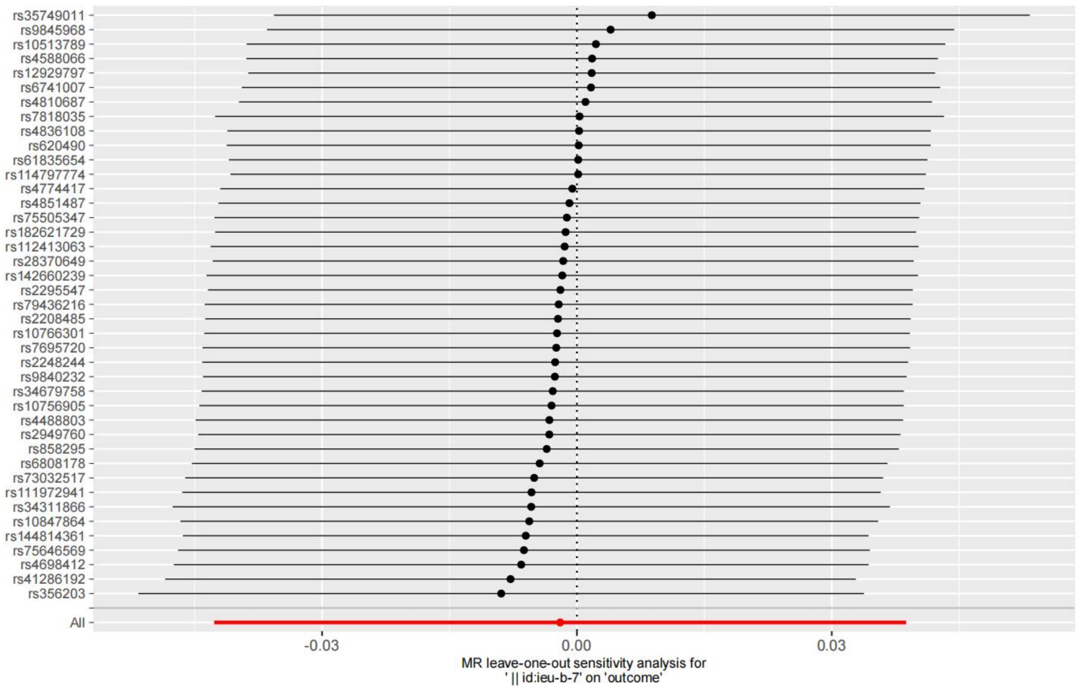

C. MR leave-one-out sensitivity analysis for PD on MCP-1-MCAF

eFigure 192. PD-associated SNPs with MCP-3

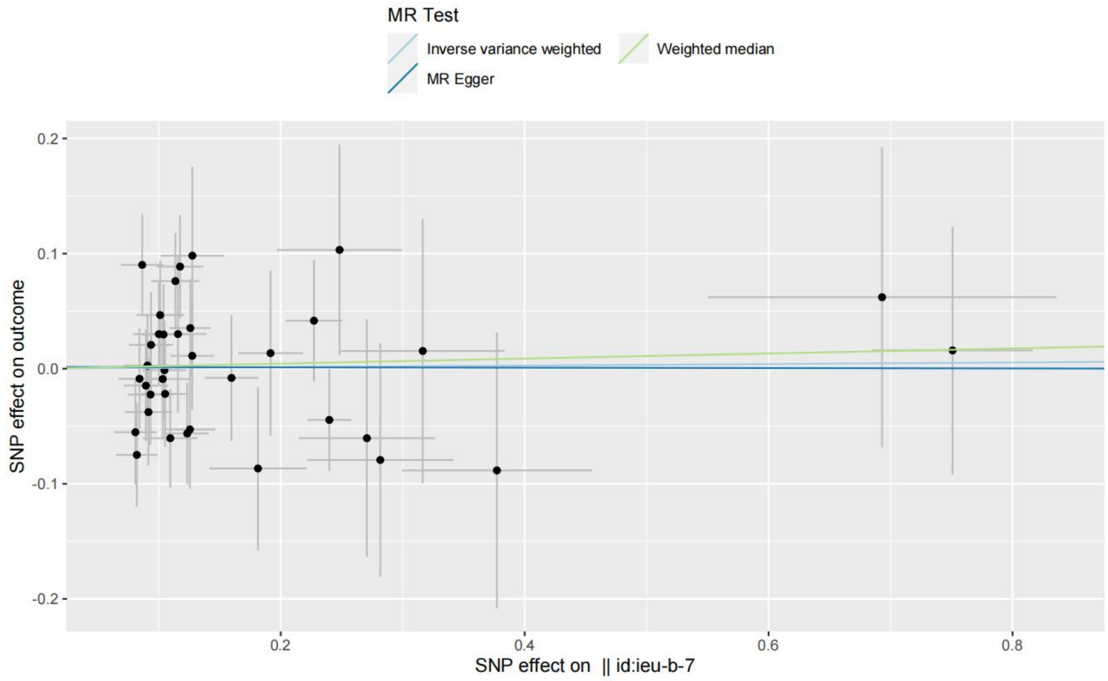

A. Scatter plot of PD on MCP-3

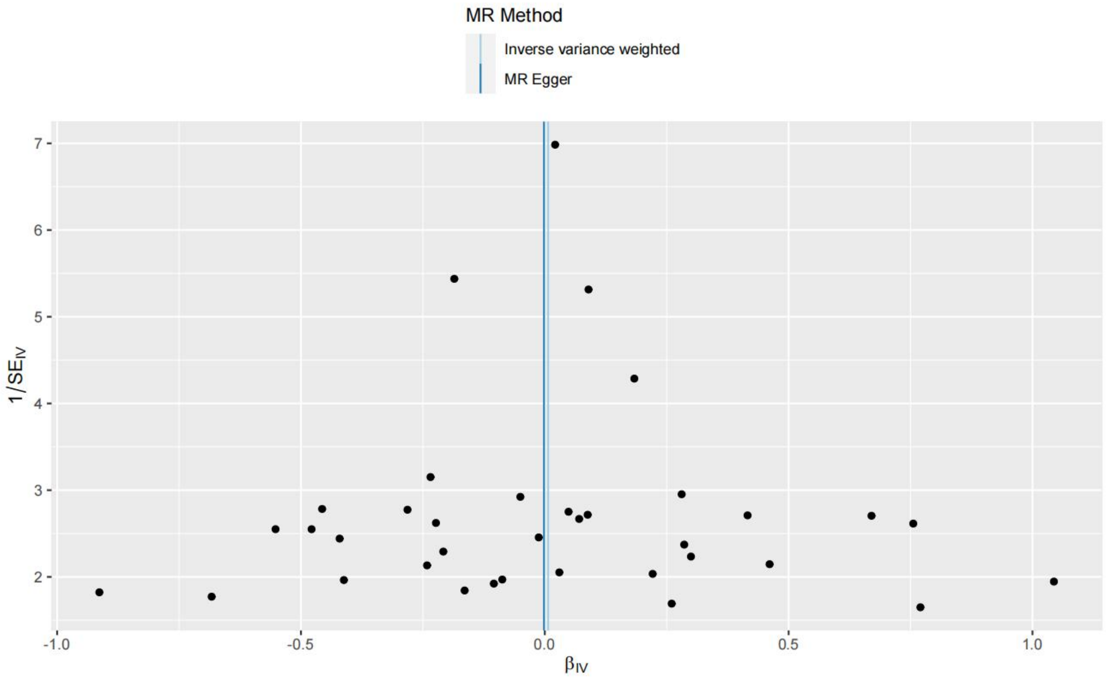

B. Funnel plot of PD on MCP-3

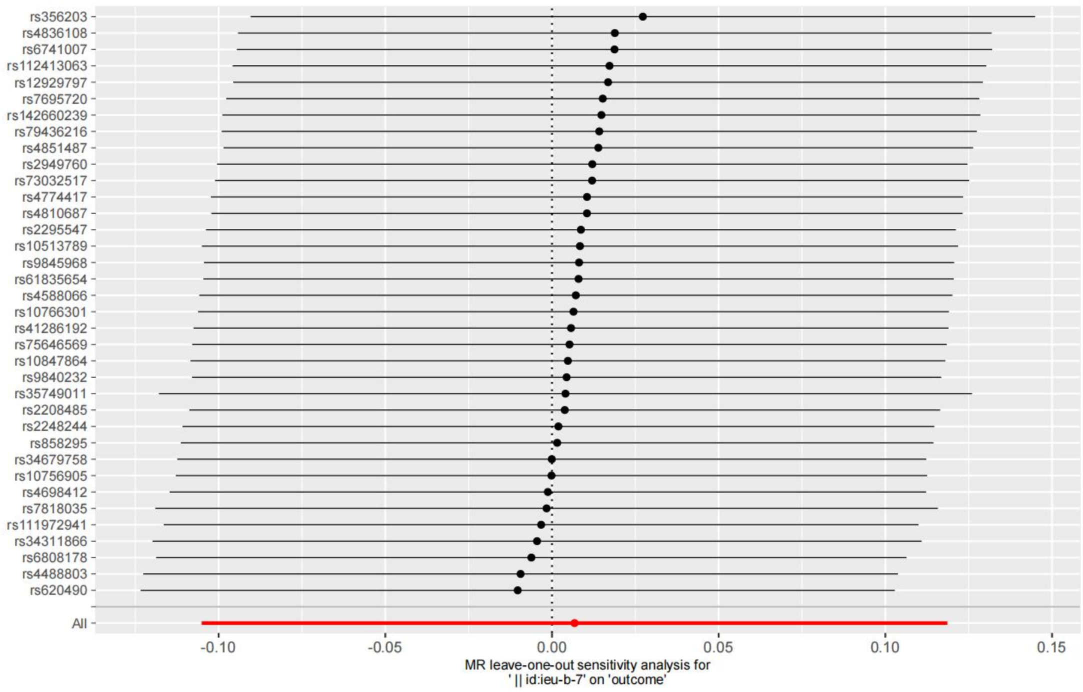

C. MR leave-one-out sensitivity analysis for PD on MCP-3

eFigure 193. PD-associated SNPs with MIF

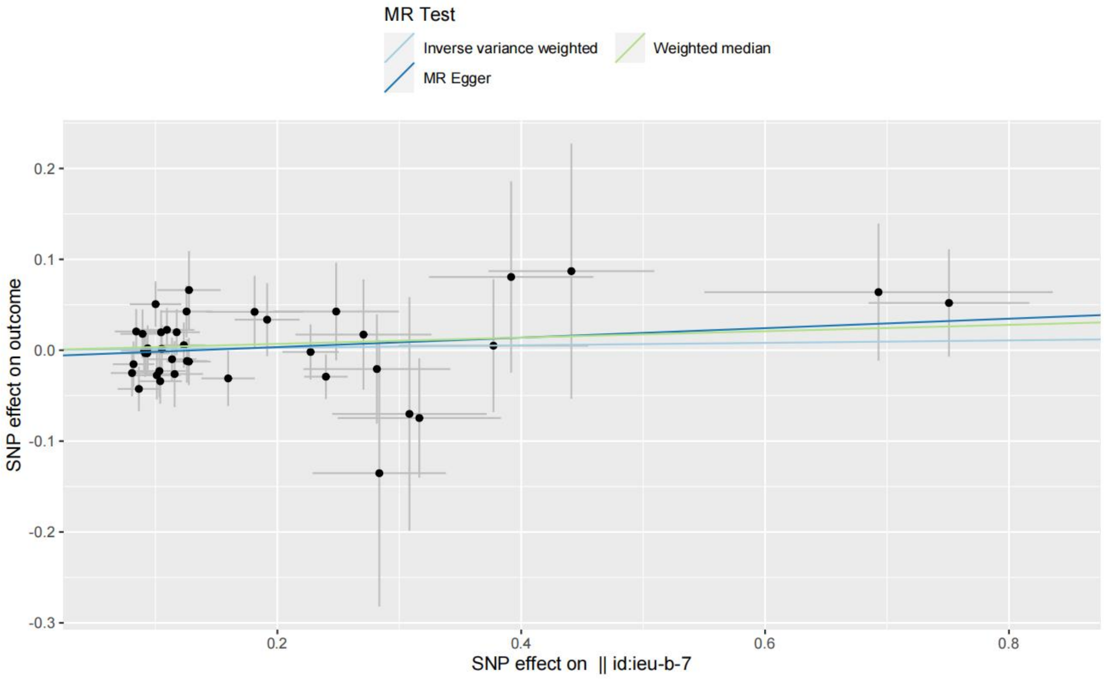

A. Scatter plot of PD on MIF

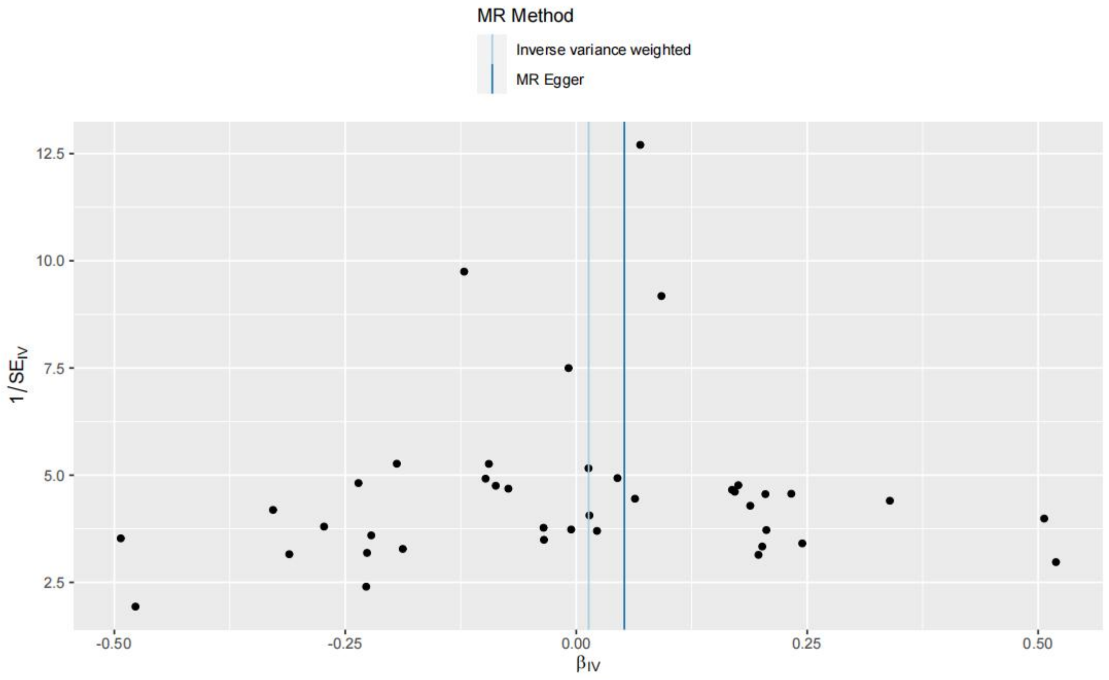

B. Funnel plot of PD on MIF

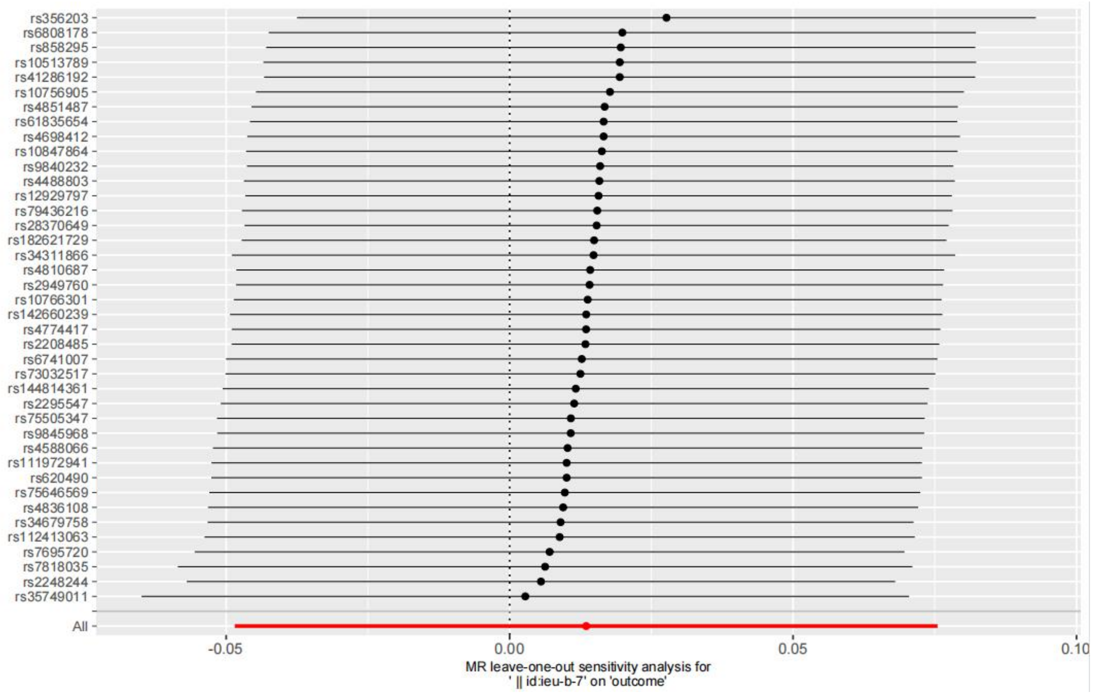

C. MR leave-one-out sensitivity analysis for PD on MIF

eFigure 194. PD-associated SNPs with MIG

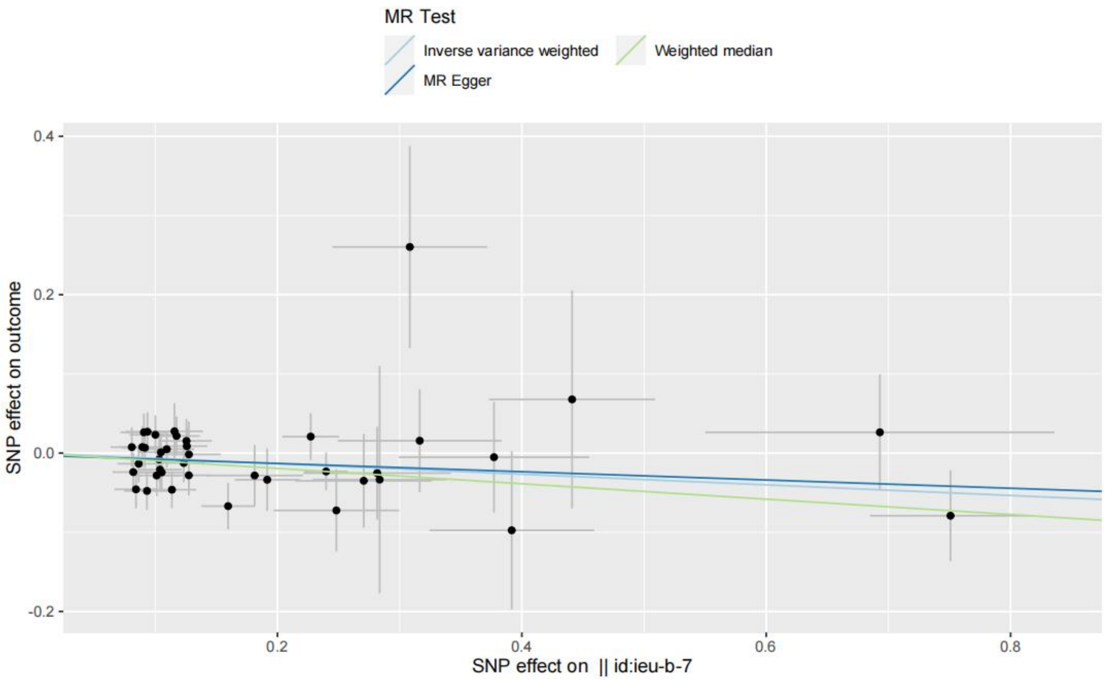

A. Scatter plot of PD on MIG

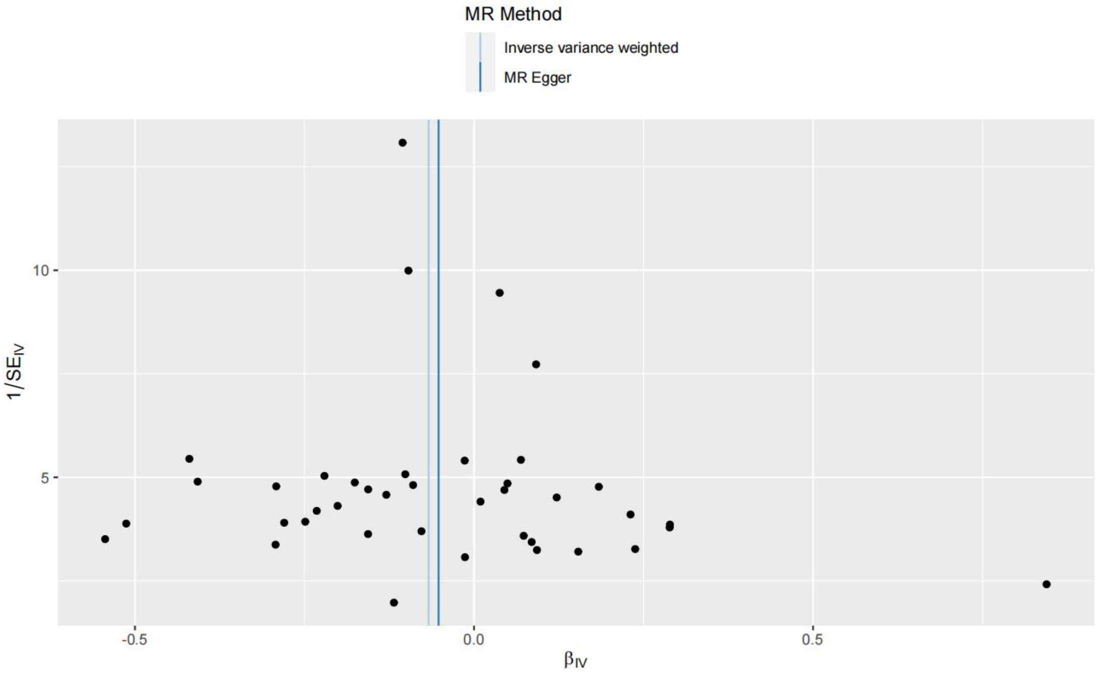

B. Funnel plot of PD on MIG

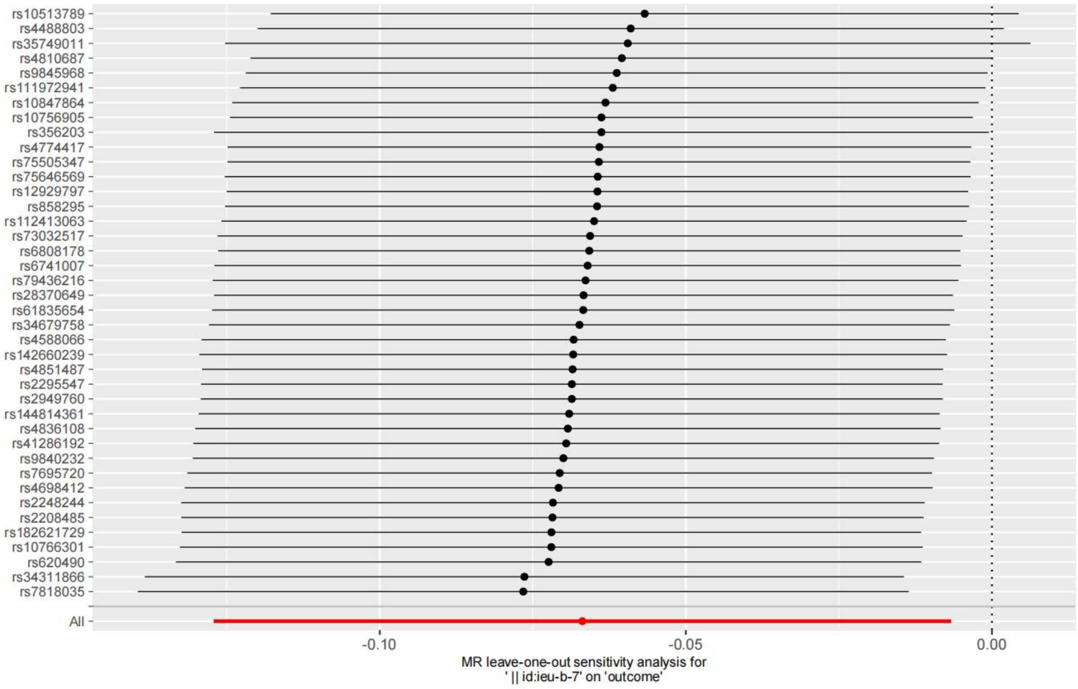

C. MR leave-one-out sensitivity analysis for PD on MIG

eFigure 195. PD-associated SNPs with MIP-1A

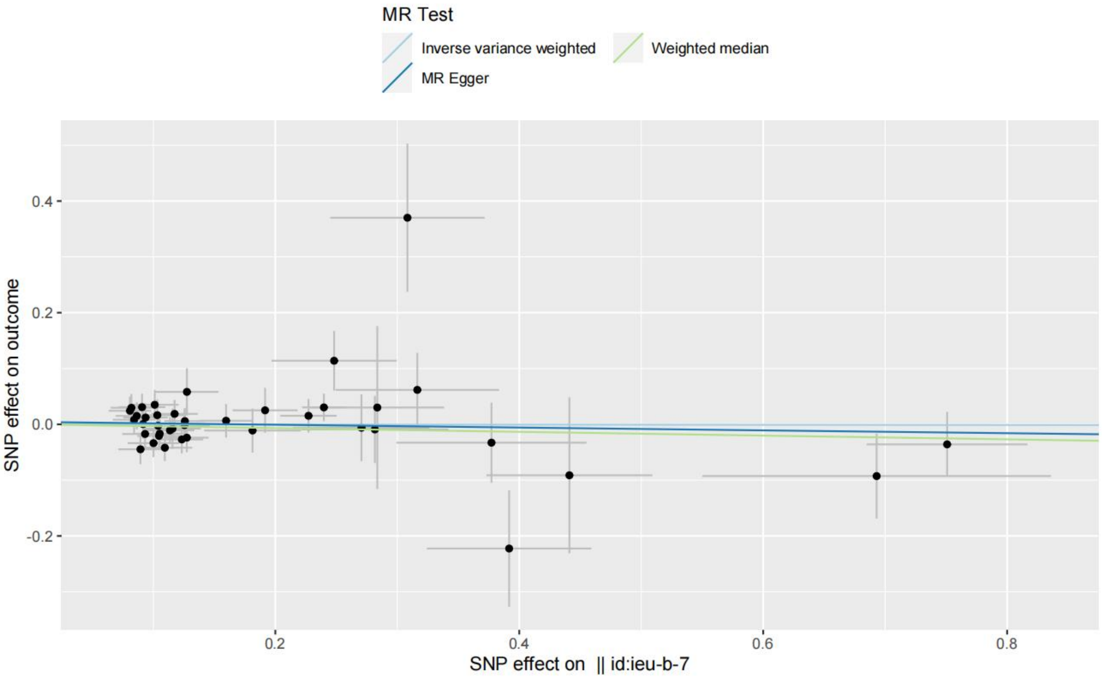

A. Scatter plot of PD on MIP-1A

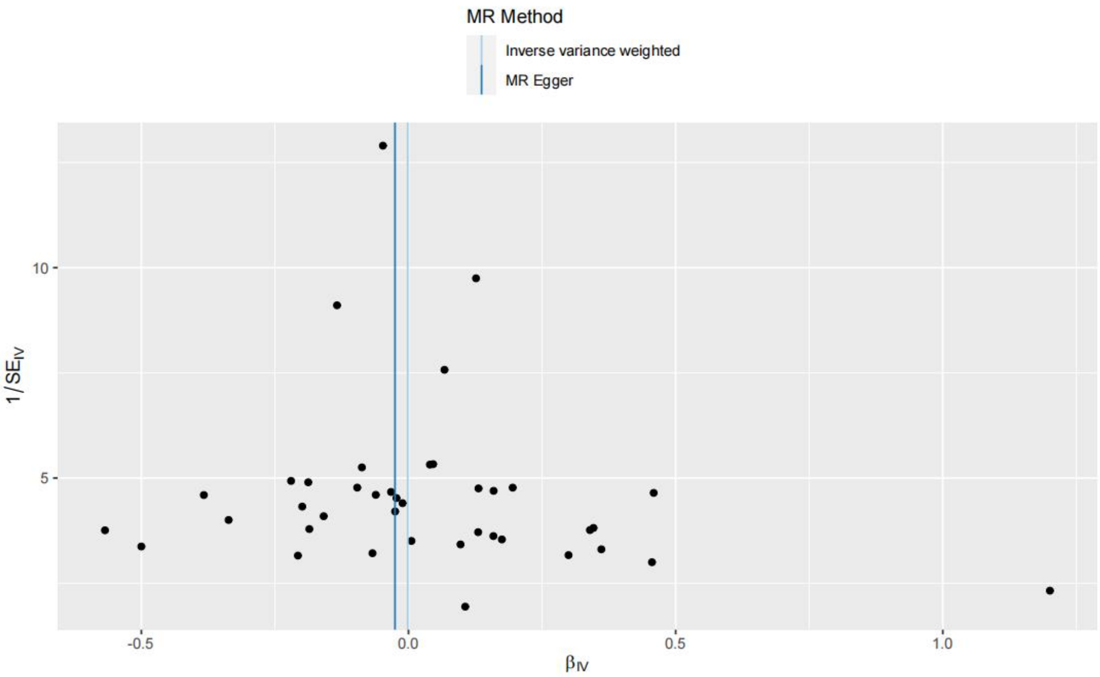

B. Funnel plot of PD on MIP-1A

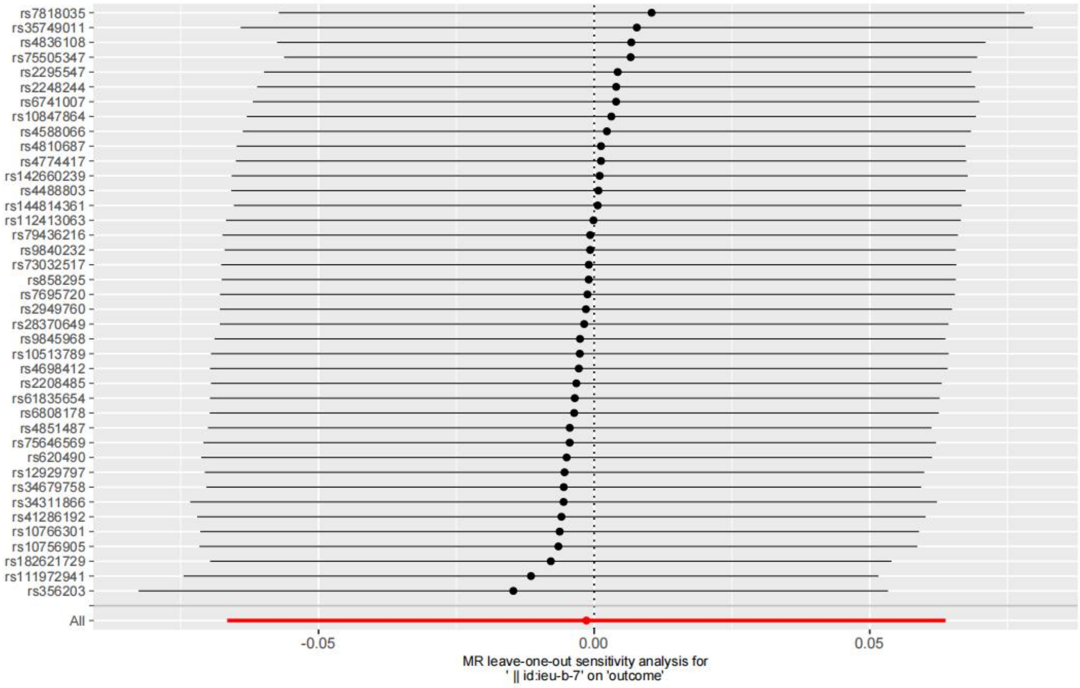

C. MR leave-one-out sensitivity analysis for PD on MIP-1A

eFigure 196. PD-associated SNPs with MIP-1B

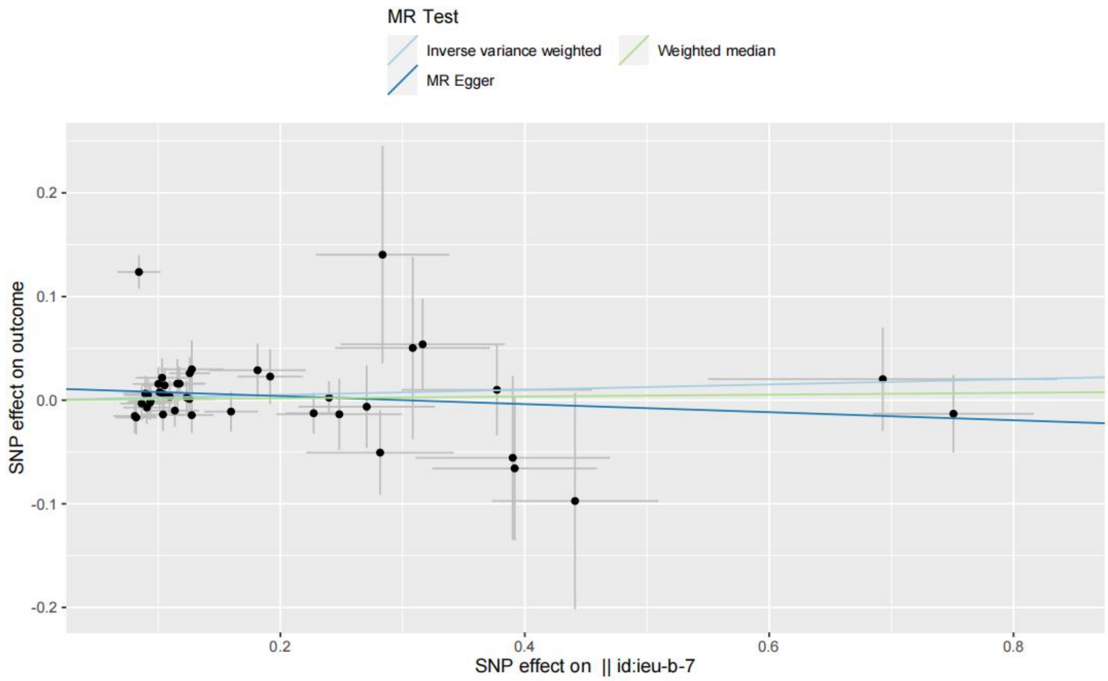

A. Scatter plot of PD on MIP-1B

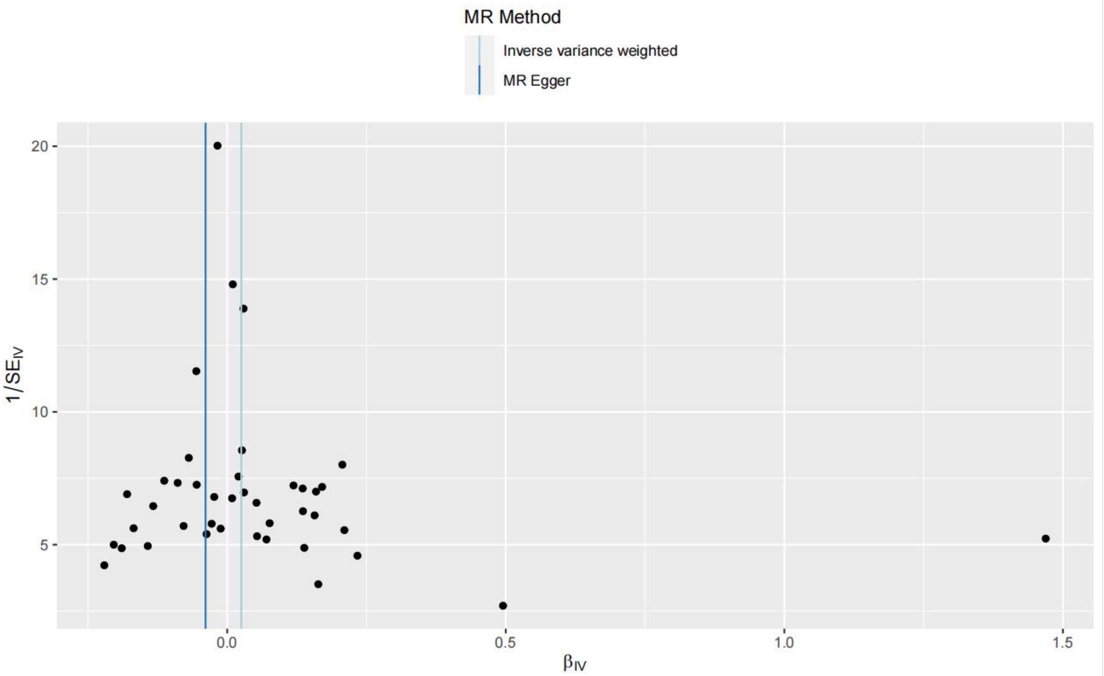

B. Funnel plot of PD on MIP-1B

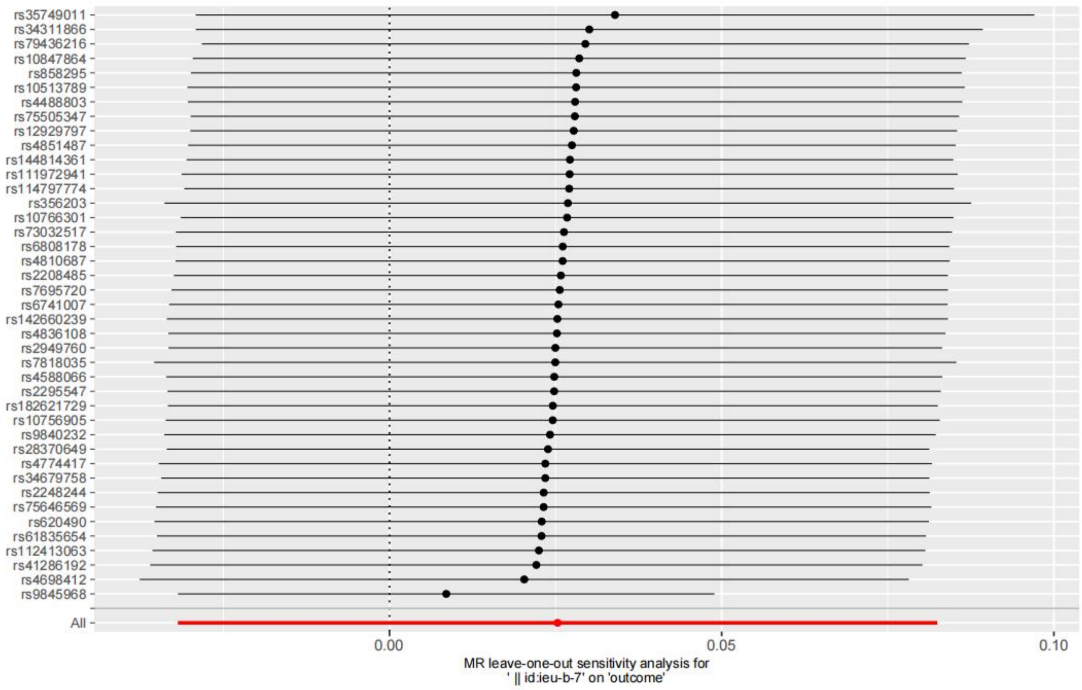

C. MR leave-one-out sensitivity analysis for PD on MIP-1B

eFigure 197. PD-associated SNPs with PDGF-BB

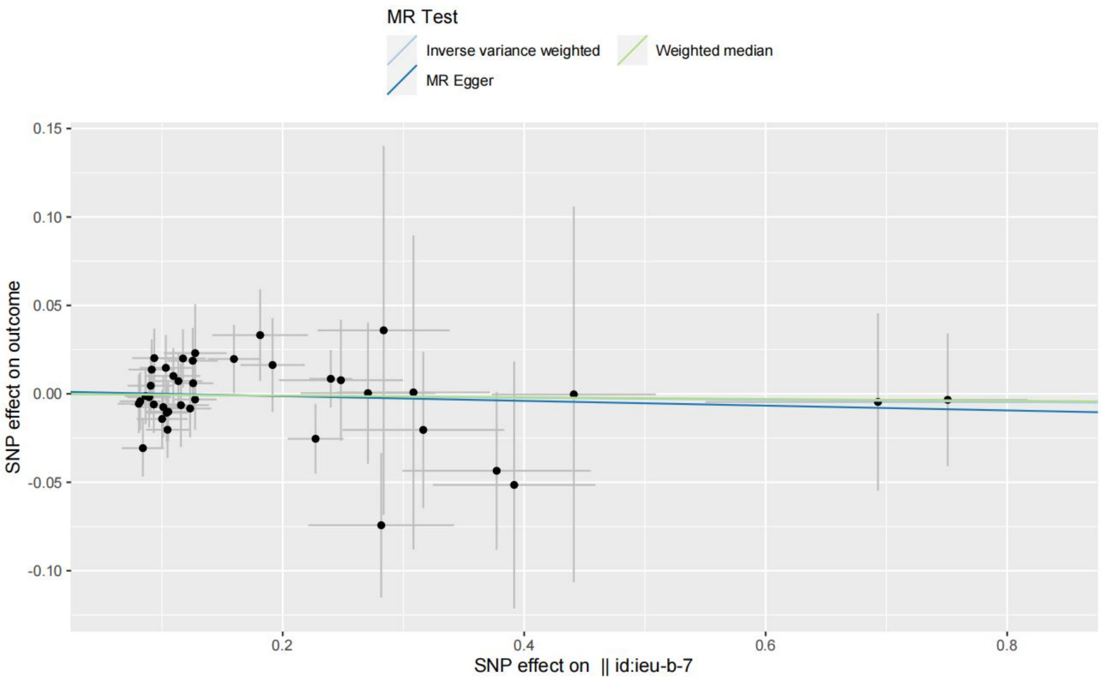

A. Scatter plot of PD on PDGF-BB

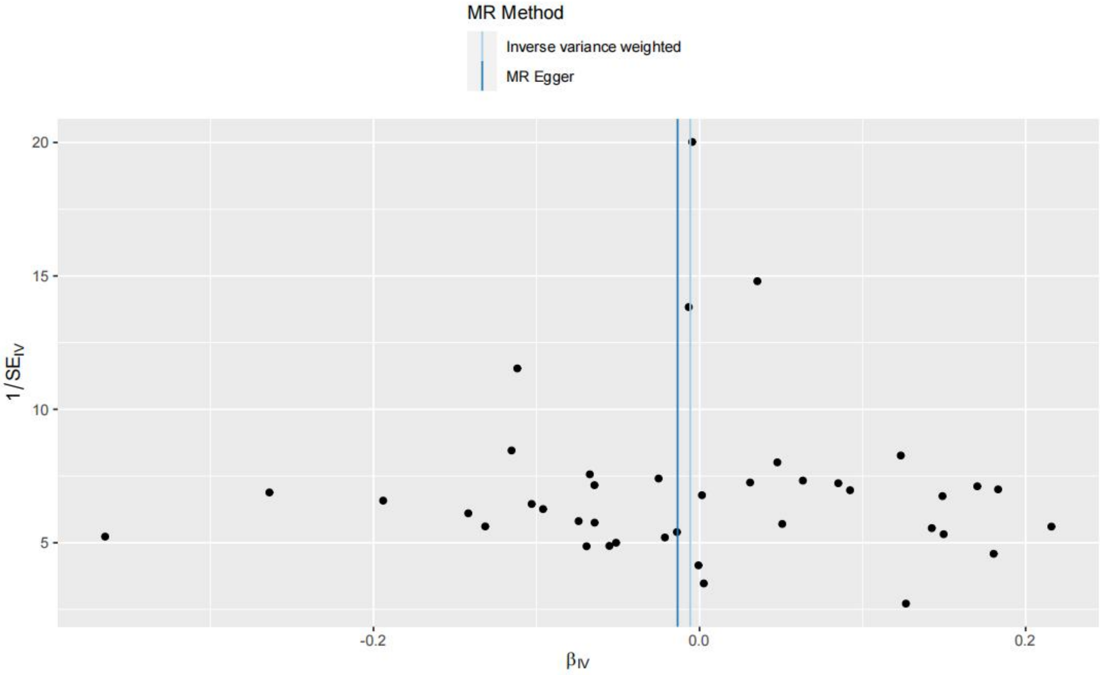

B. Funnel plot of PD on PDGF-BB

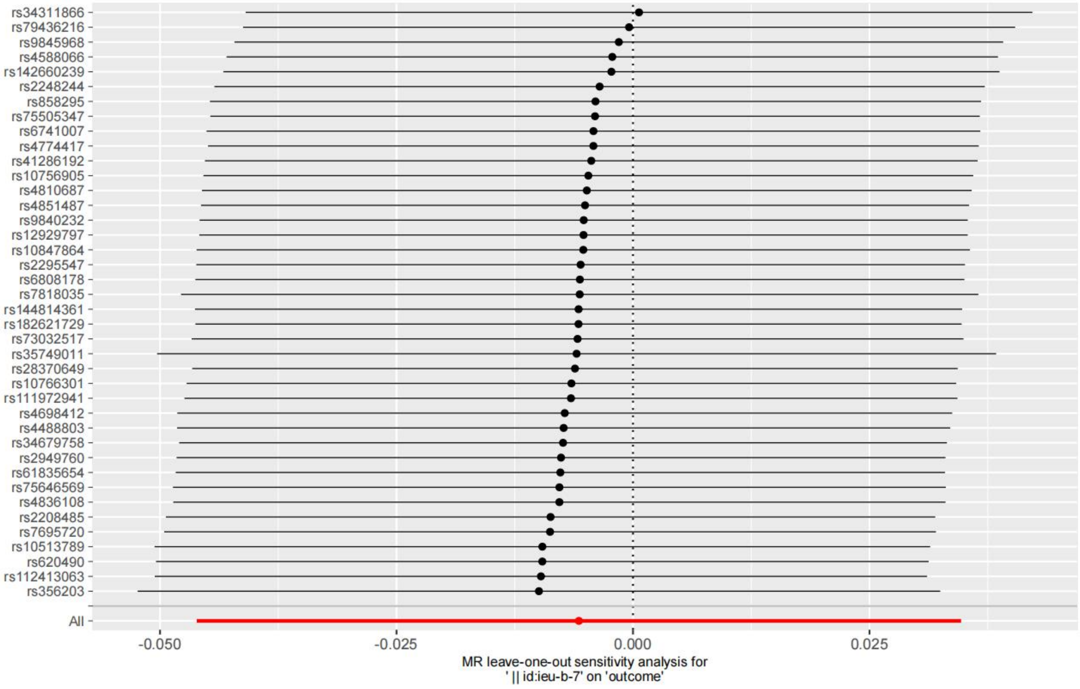

C. MR leave-one-out sensitivity analysis for PD on PDGF-BB

eFigure 198. PD-associated SNPs with RANTES

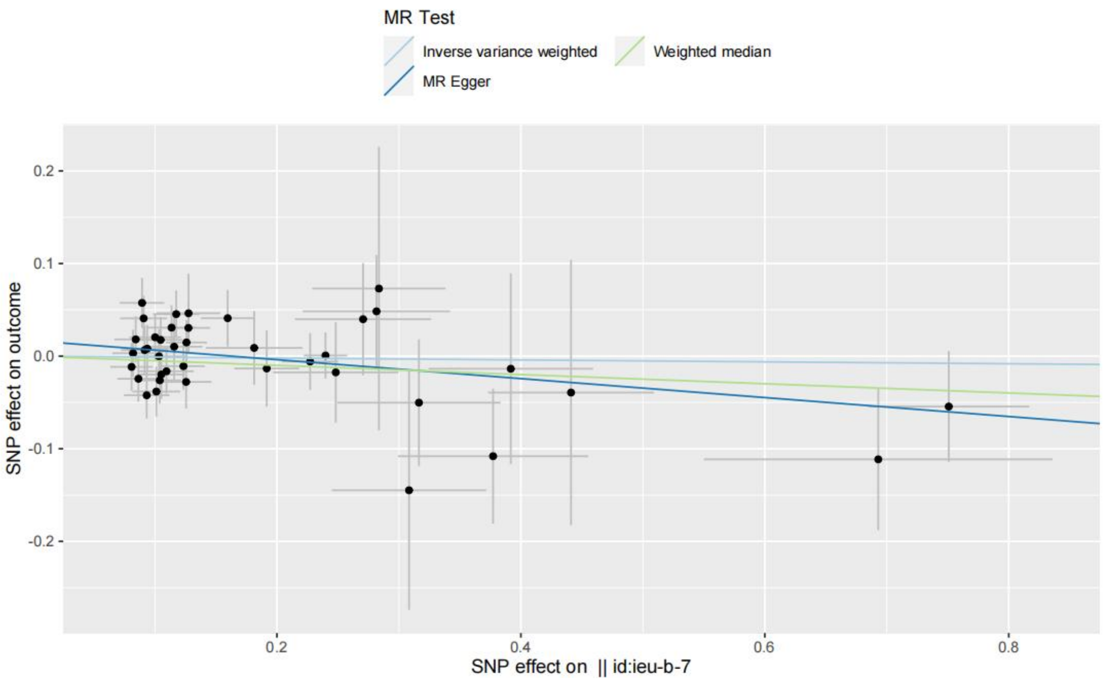

A. Scatter plot of PD on RANTES

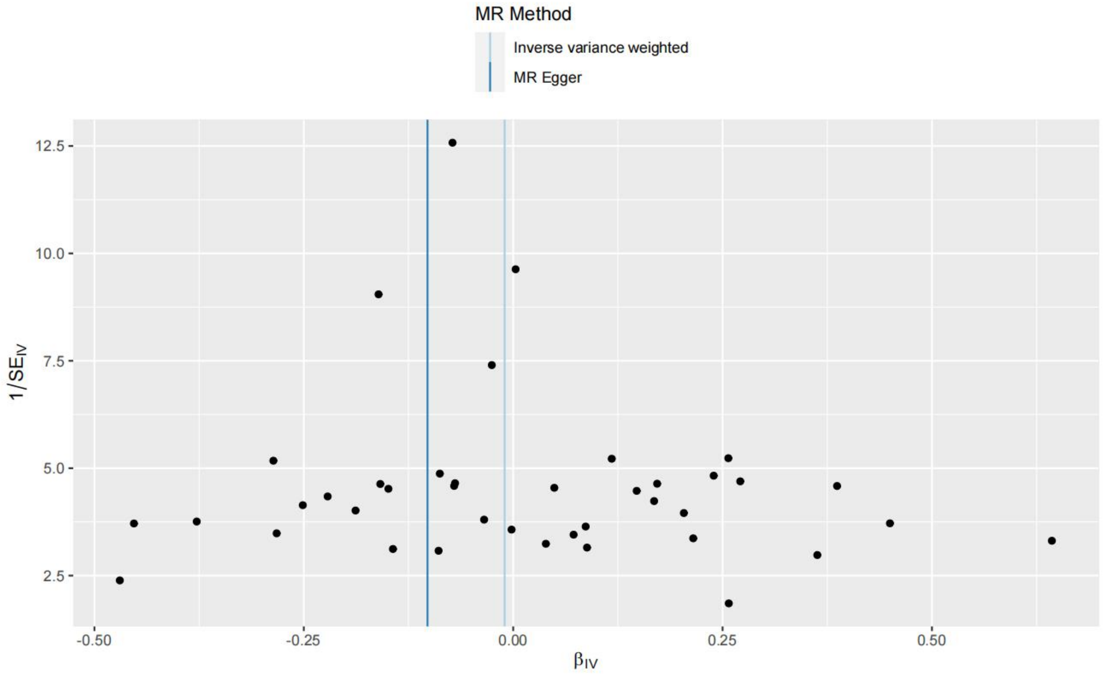

B. Funnel plot of PD on RANTES

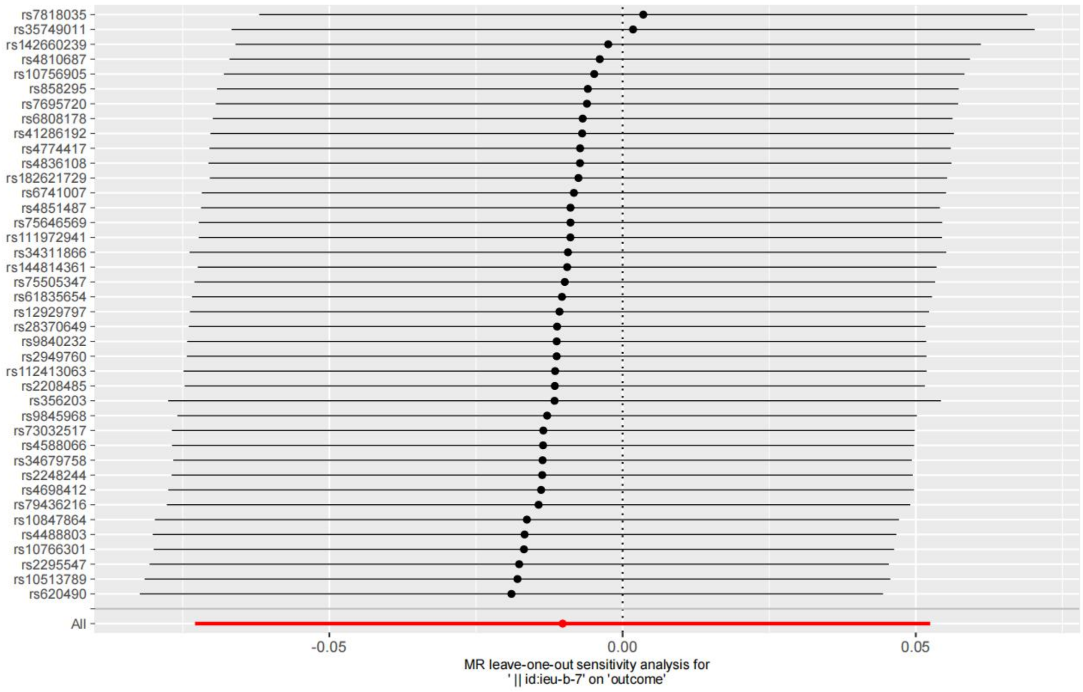

C. MR leave-one-out sensitivity analysis for PD on RANTES

eFigure 199. PD-associated SNPs with SCF

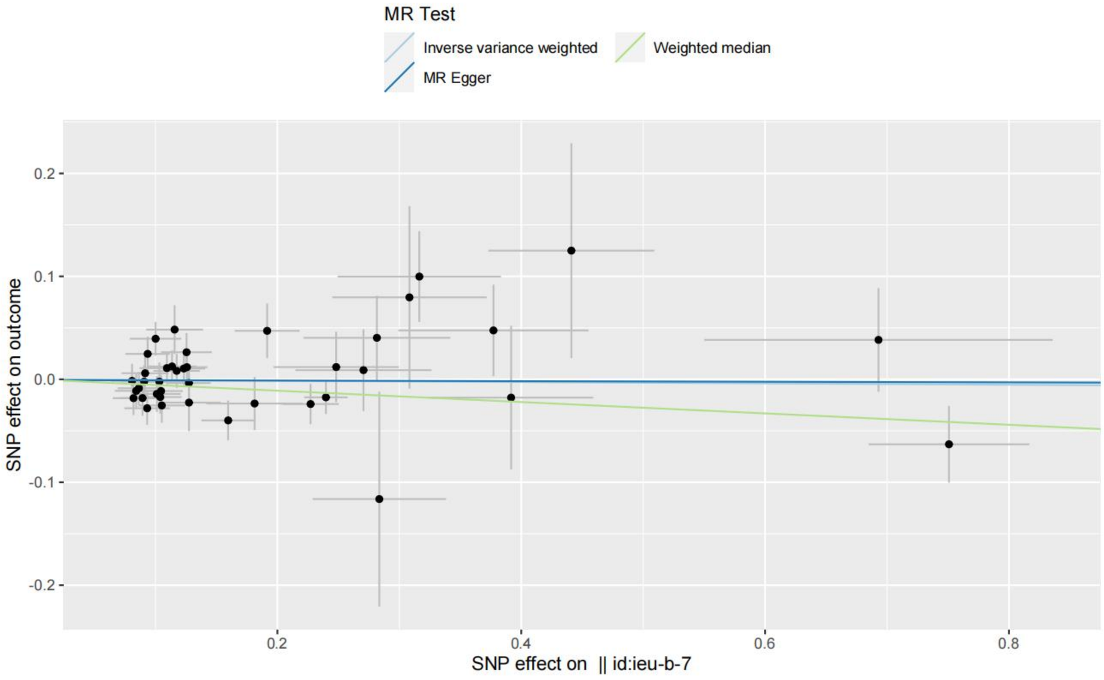

A. Scatter plot of PD on SCF

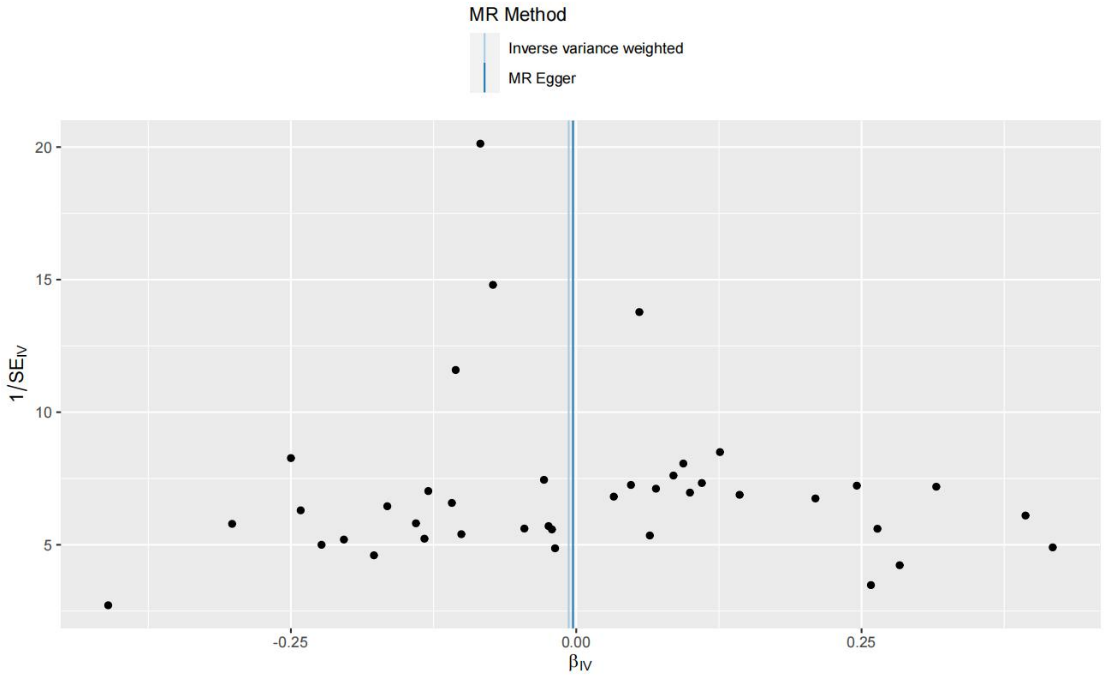

B. Funnel plot of PD on SCF

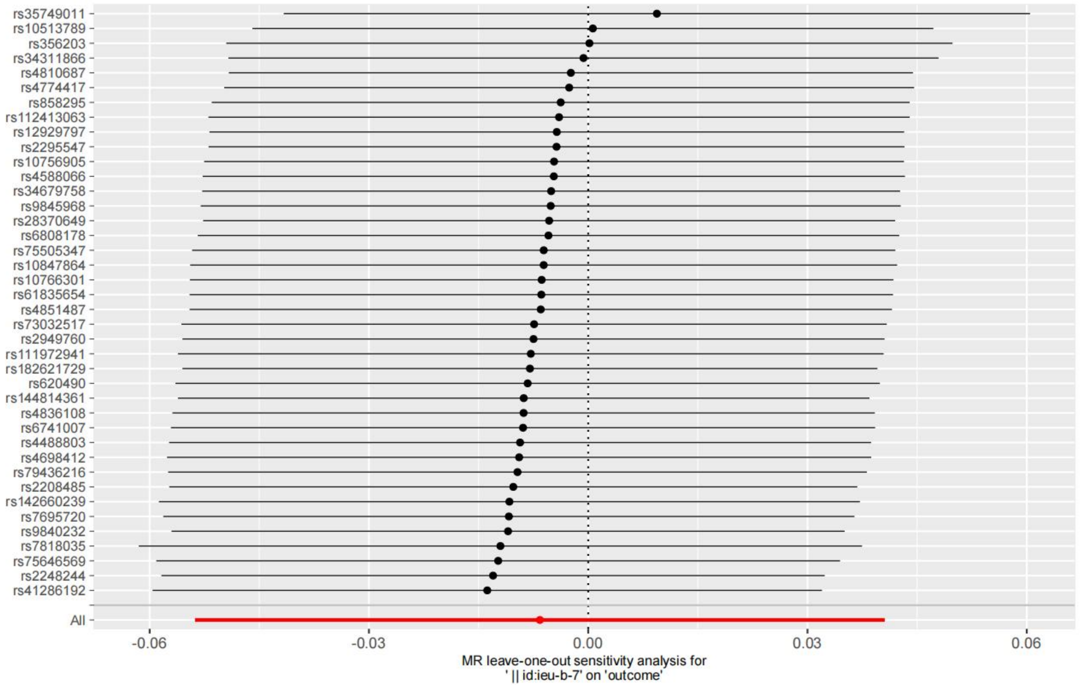

C. MR leave-one-out sensitivity analysis for PD on SCF

eFigure 200. PD-associated SNPs with SGCF

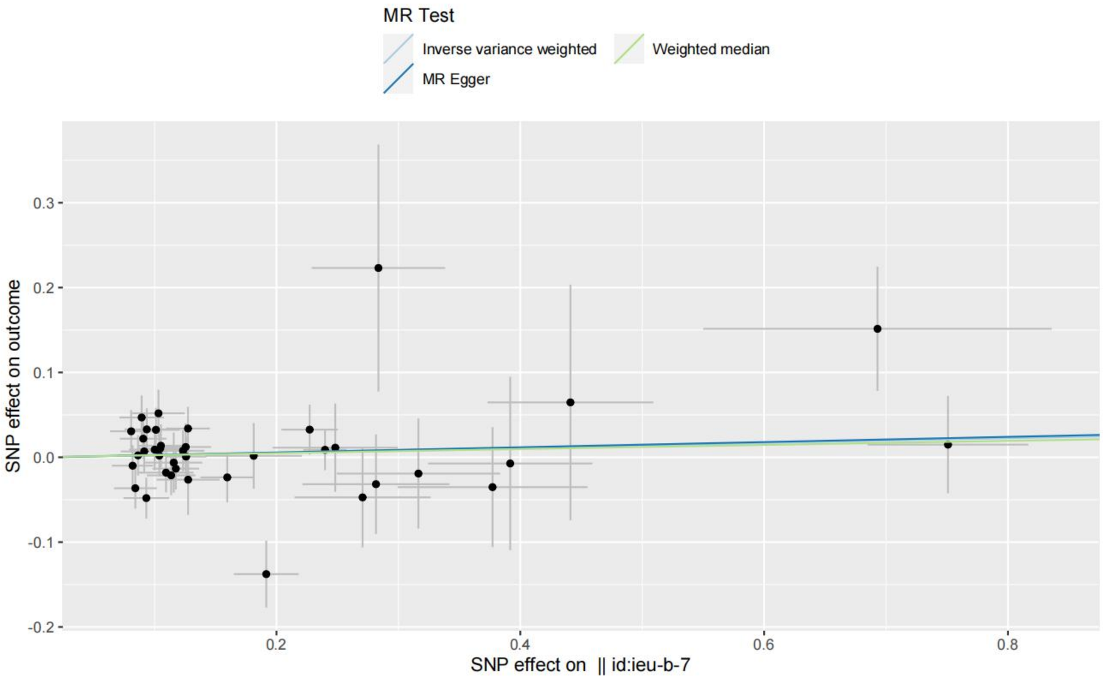

A. Scatter plot of PD on SGCF

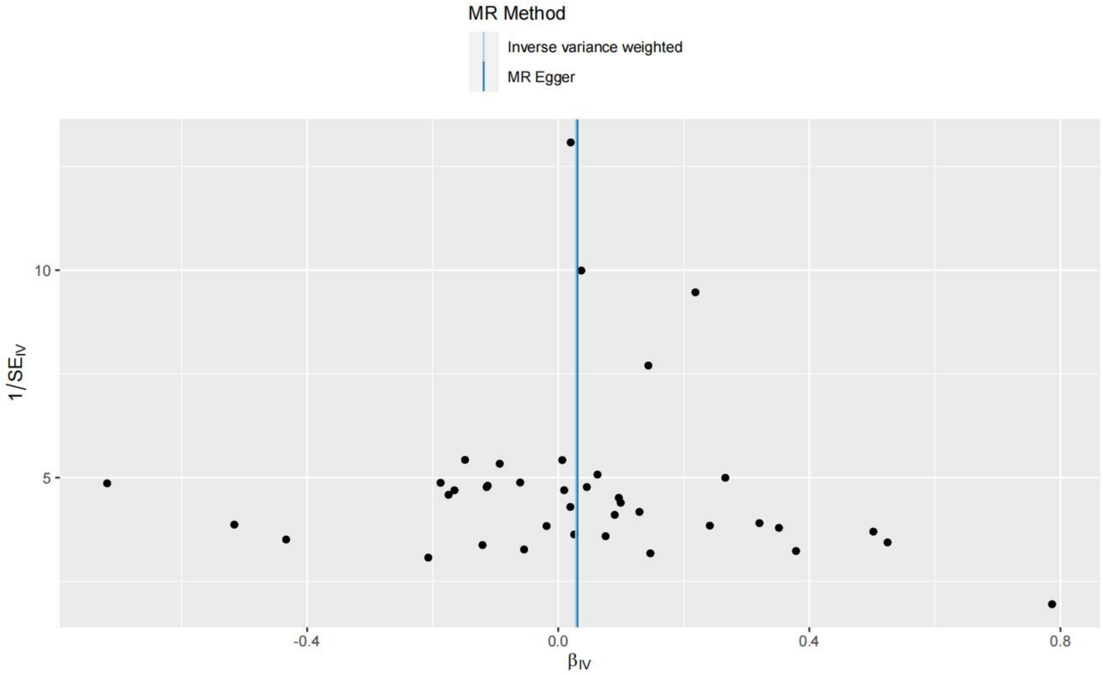

B. Funnel plot of PD on SGCF

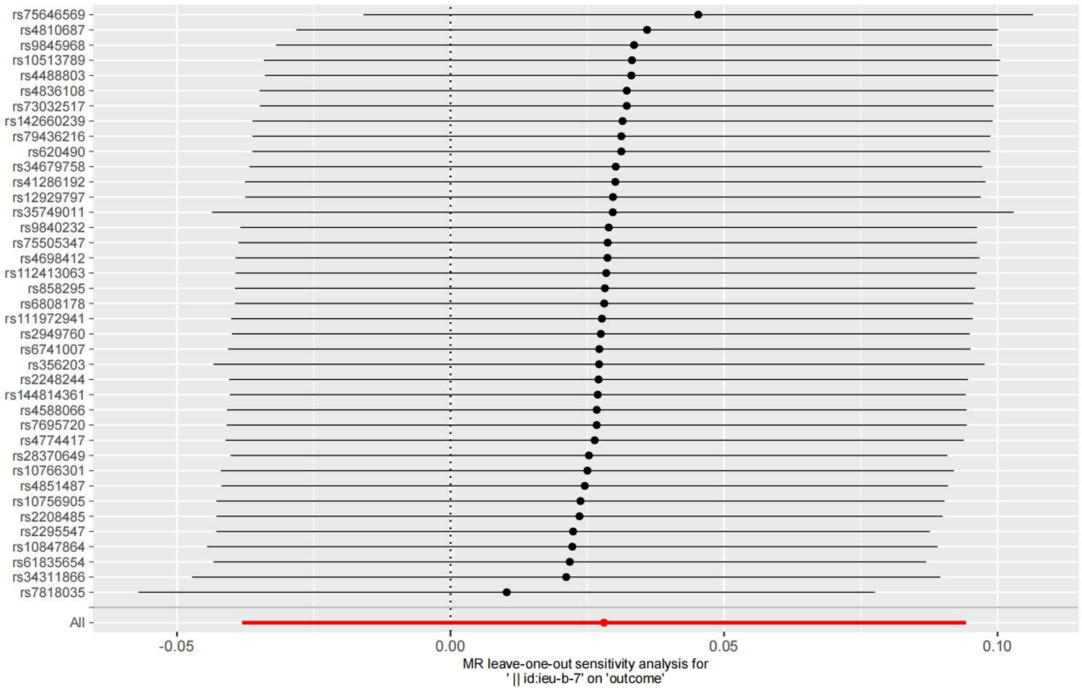

C. MR leave-one-out sensitivity analysis for PD on SGCF

eFigure 201. PD-associated SNPs with SDF-1A

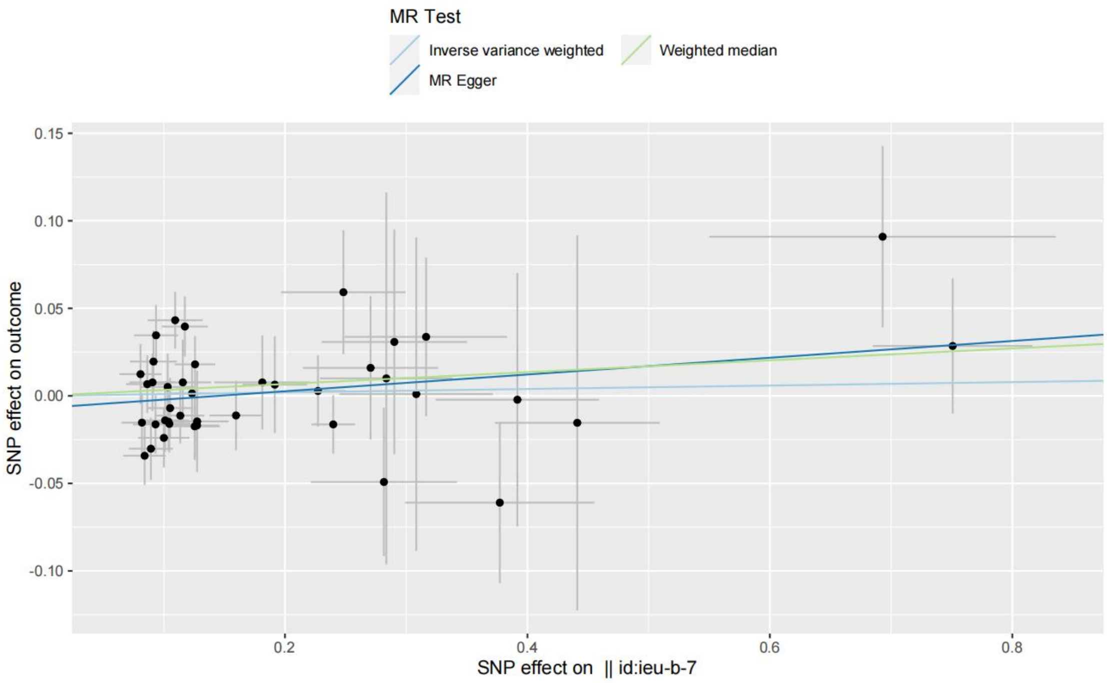

A. Scatter plot of PD on SDF-1A

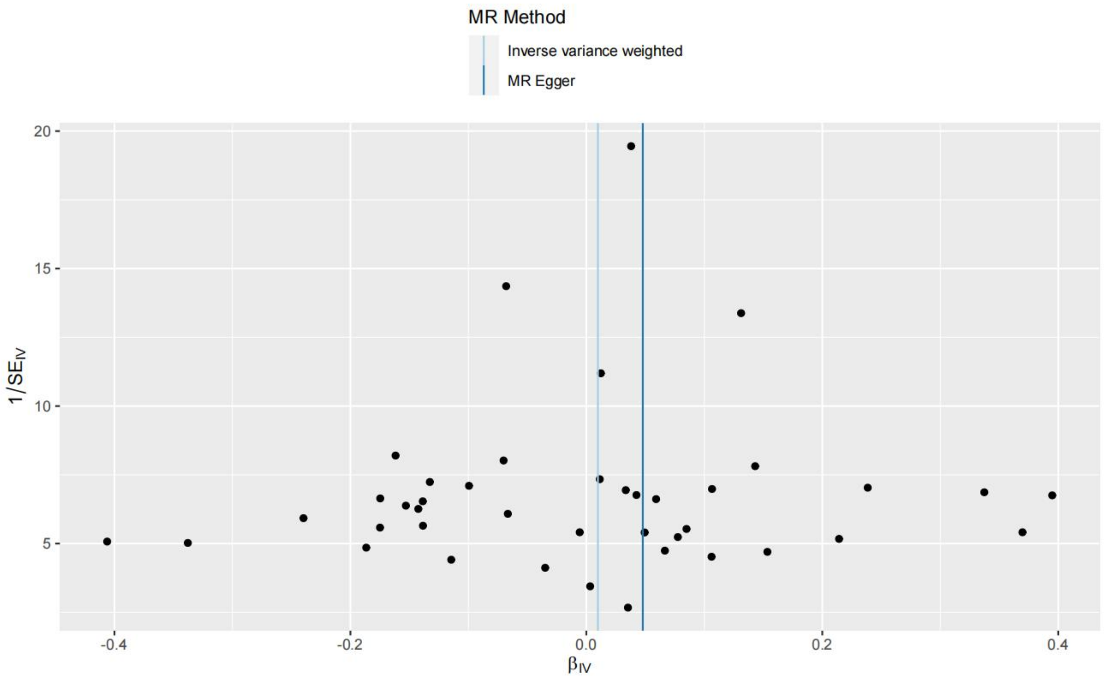

B. Funnel plot of PD on SDF-1A

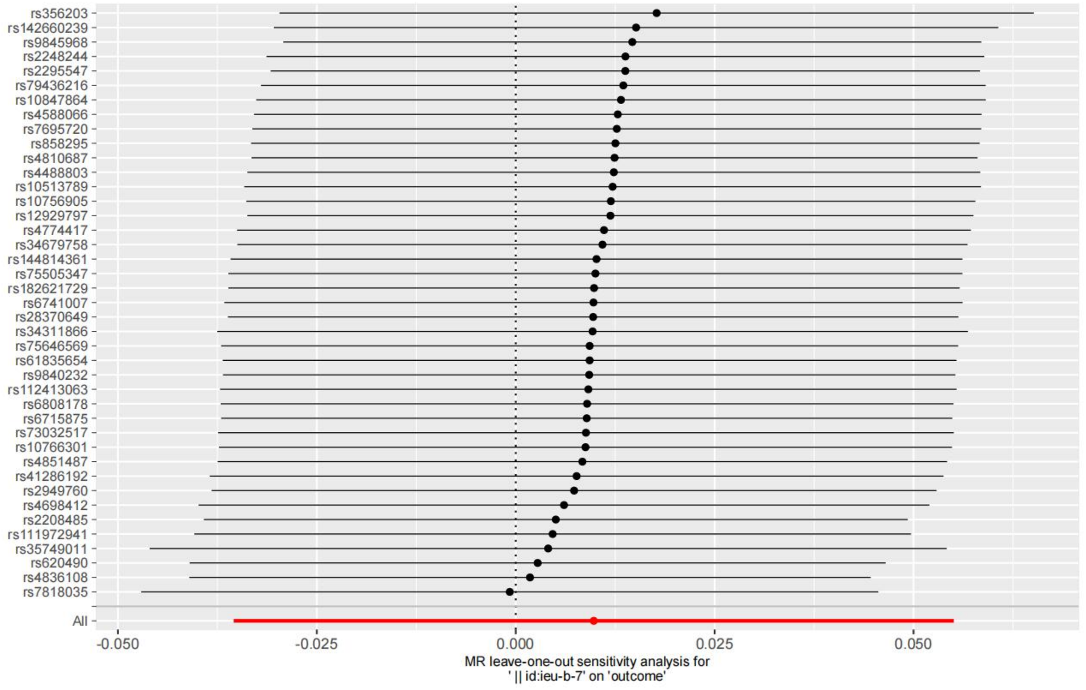

C. MR leave-one-out sensitivity analysis for PD on SDF-1A

eFigure 202. PD-associated SNPs with TNF-A

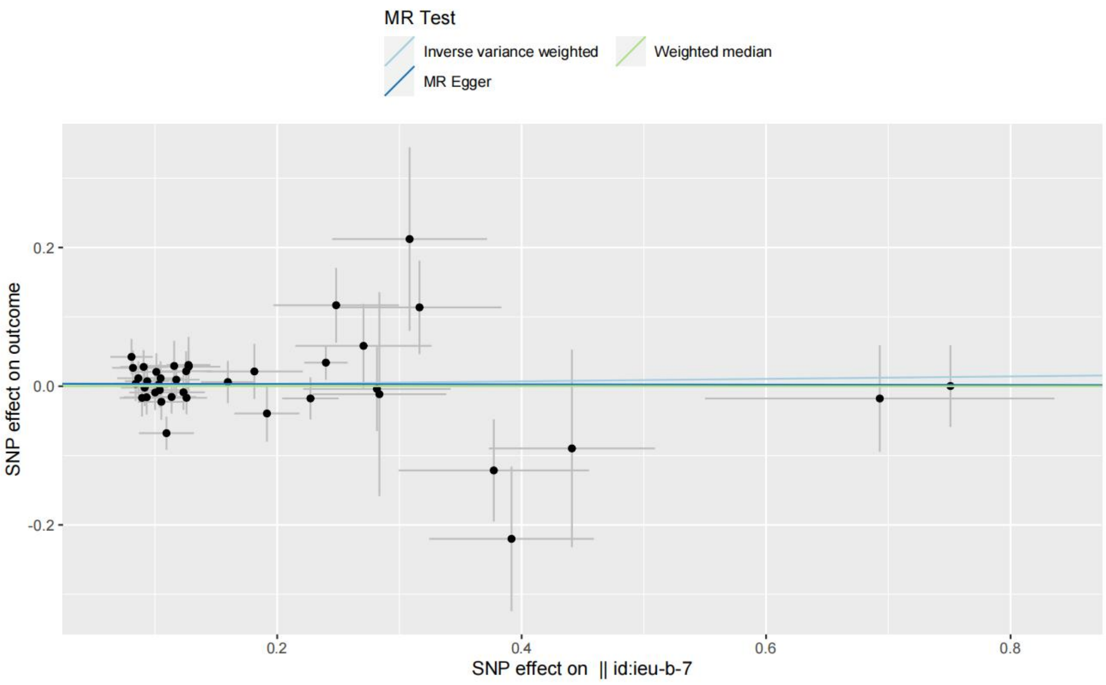

A. Scatter plot of PD on TNF-A

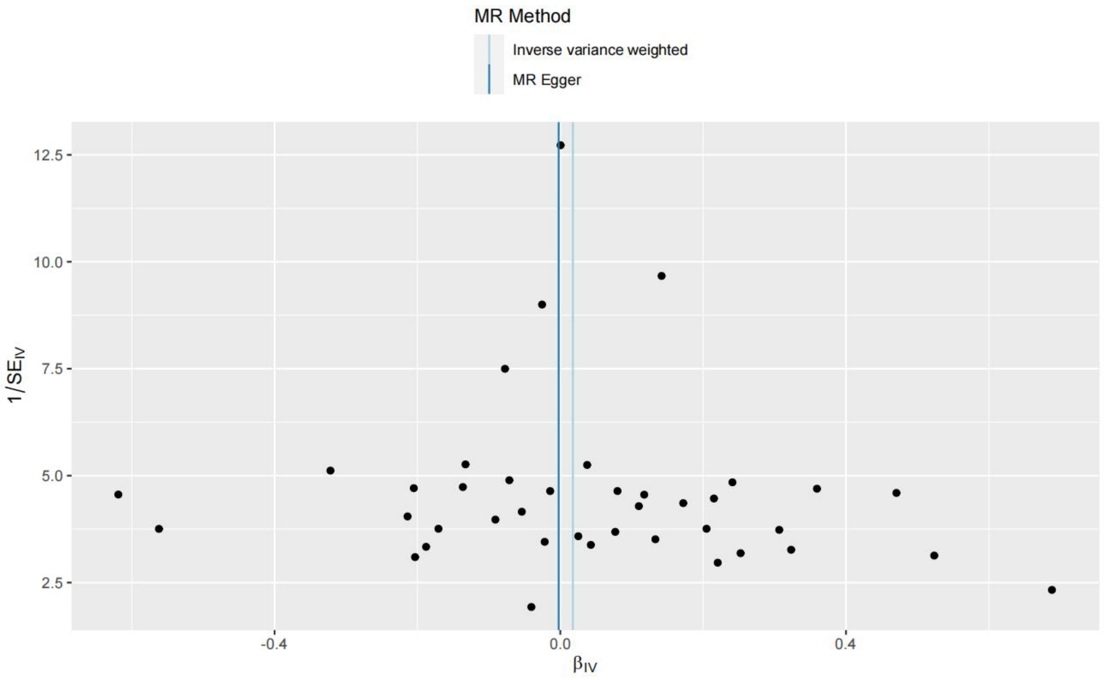

B. Funnel plot of PD on TNF-A

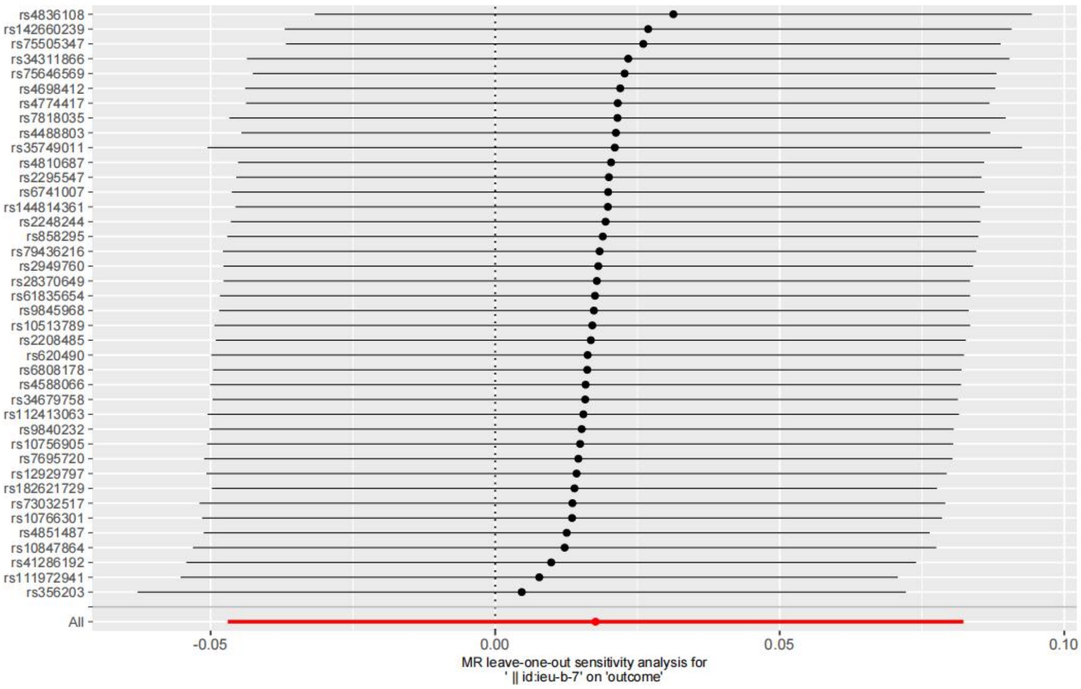

C. MR leave-one-out sensitivity analysis for PD on TNF-A

eFigure 203. PD-associated SNPs with TNF-B

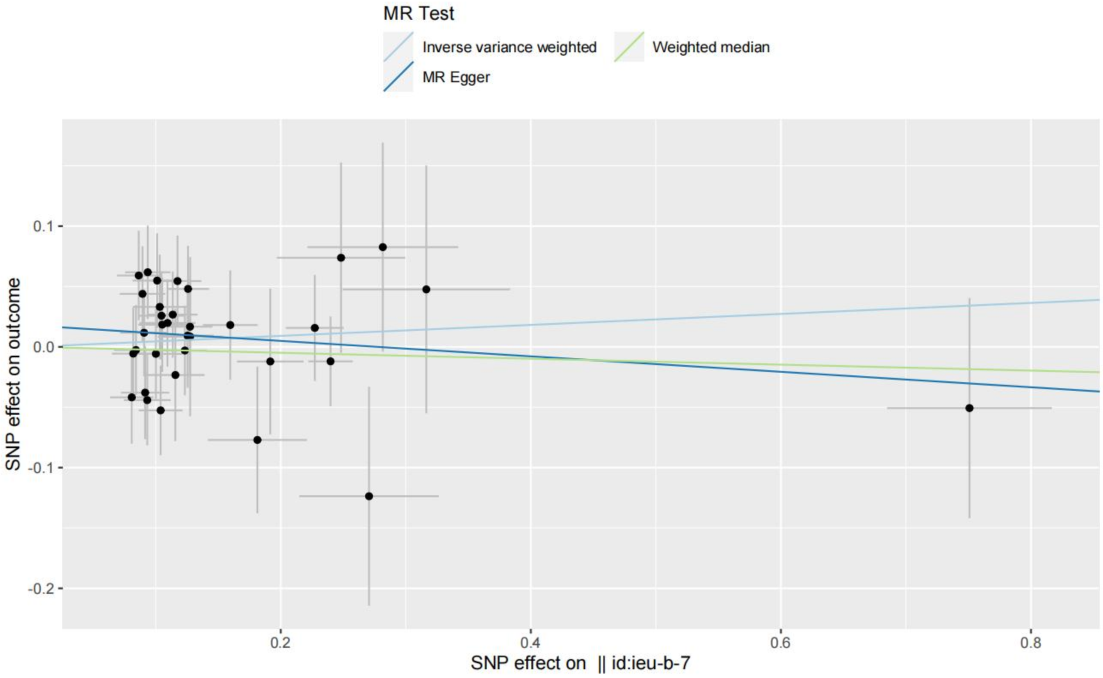

A. Scatter plot of PD on TNF-B

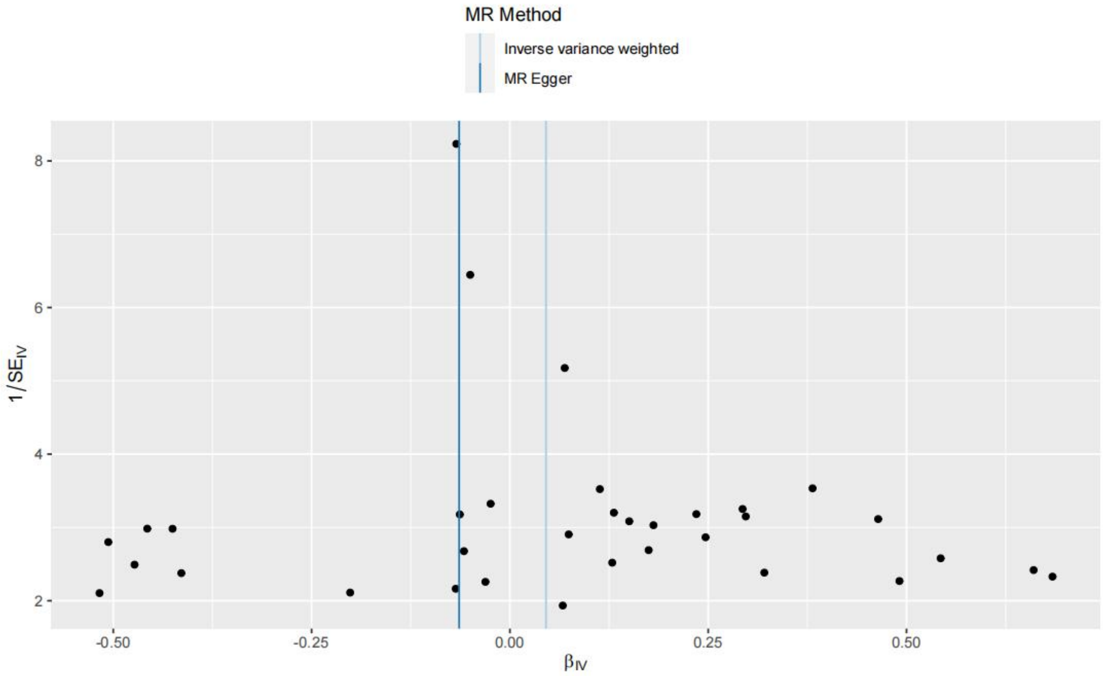

B. Funnel plot of PD on TNF-B

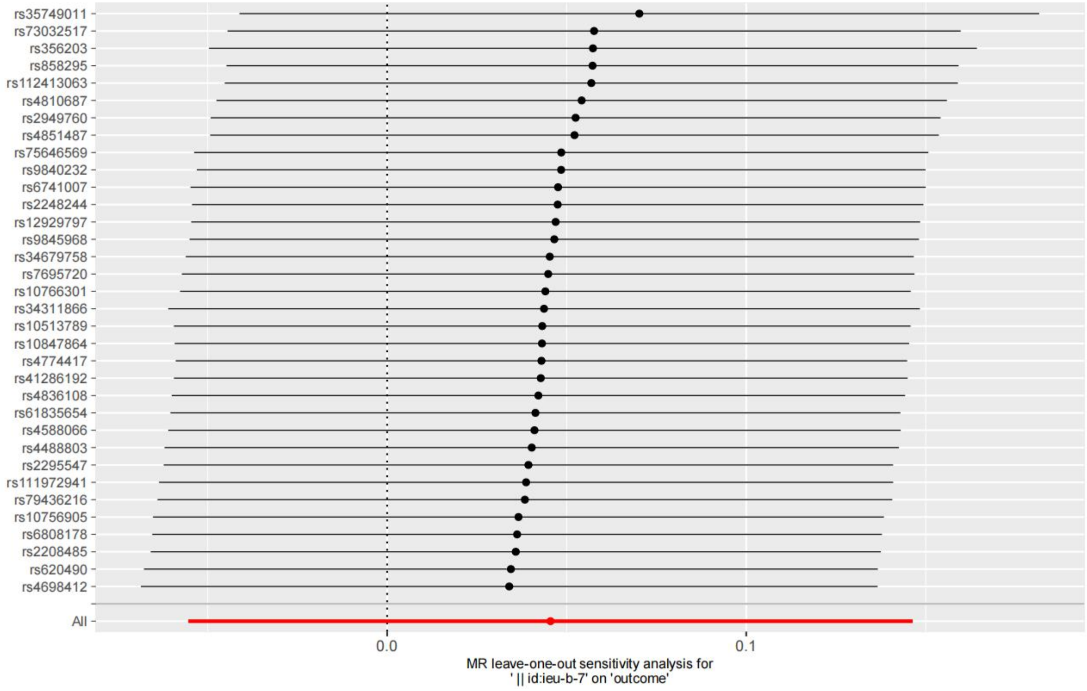

C. MR leave-one-out sensitivity analysis for PD on TNF-B

eFigure 204. PD-associated SNPs with TRAIL

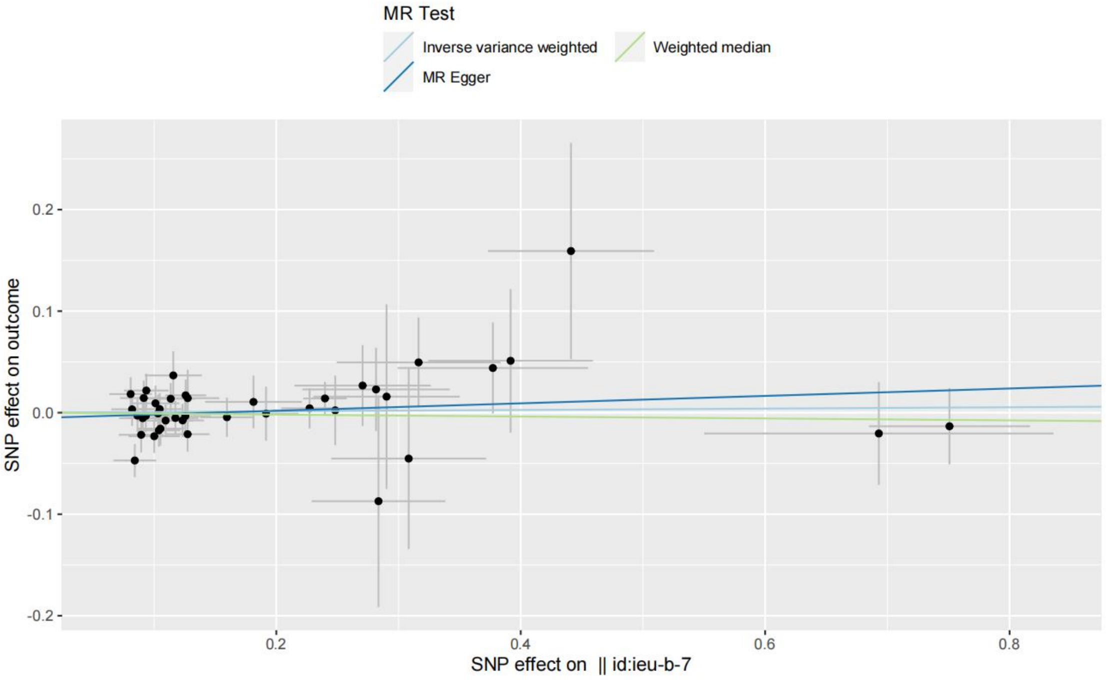

A. Scatter plot of PD on TRAIL

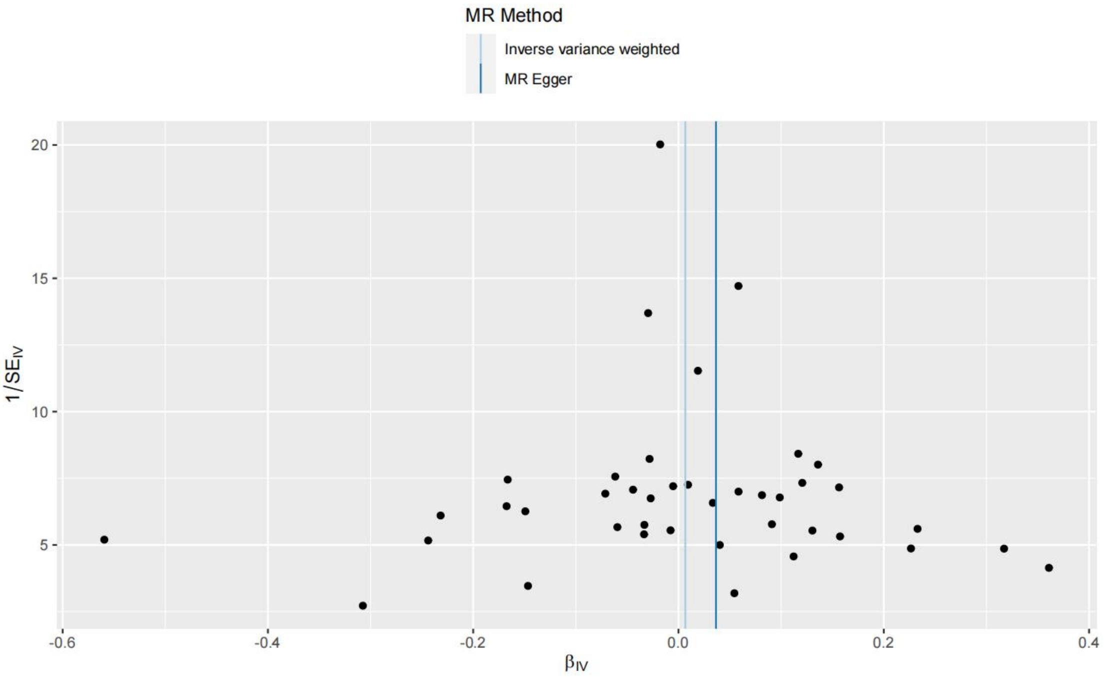

B. Funnel plot of PD on TRAIL

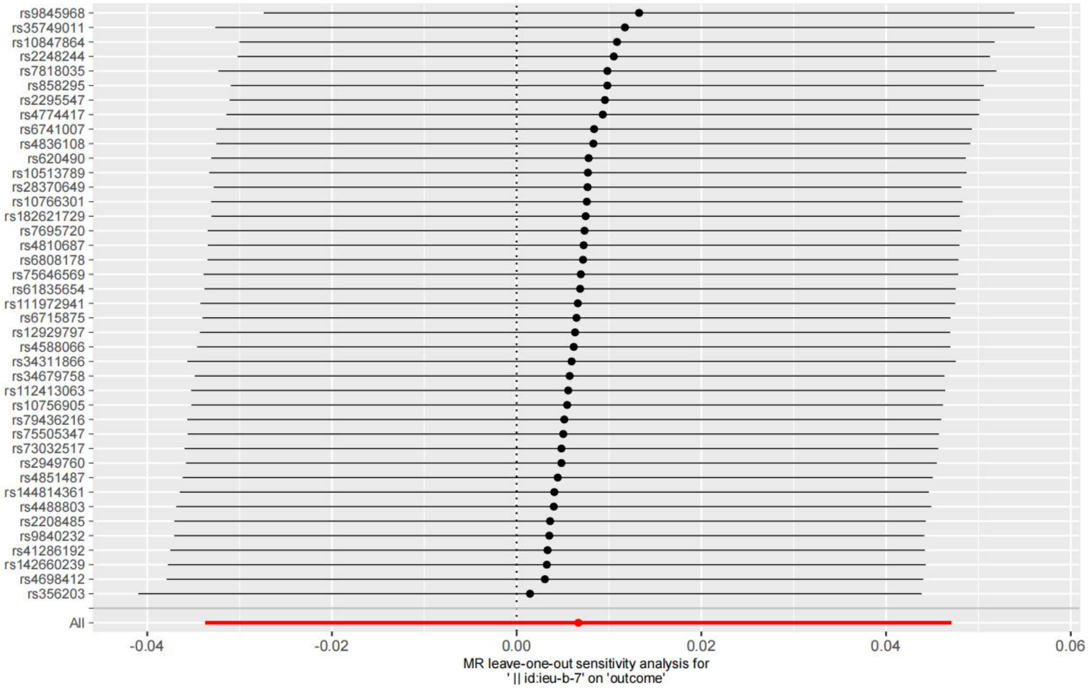

C. MR leave-one-out sensitivity analysis for PD on TRAIL

eFigure 205. PD-associated SNPs with VEGF

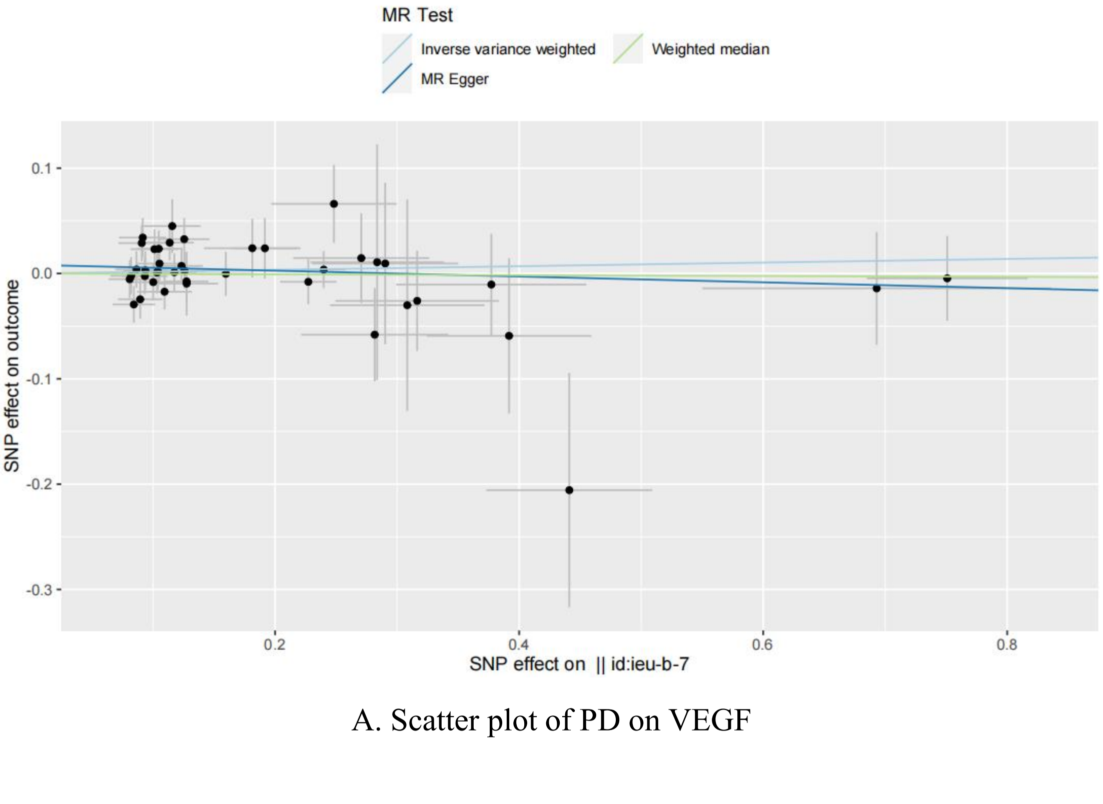

A. Scatter plot of PD on VEGF

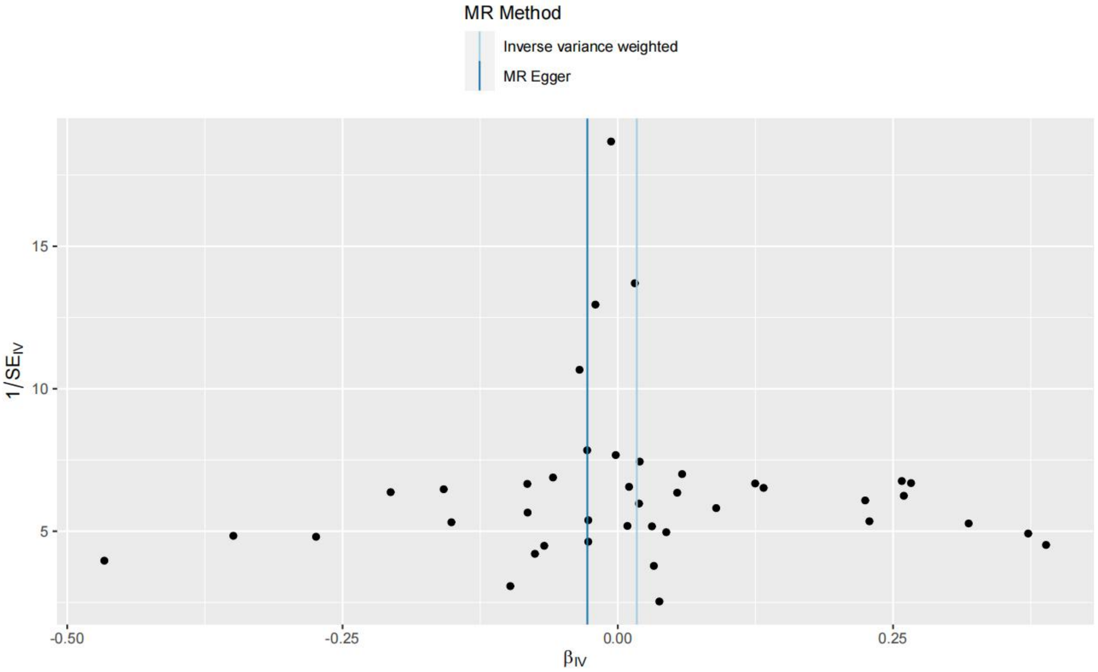

B. Funnel plot of PD on VEGF

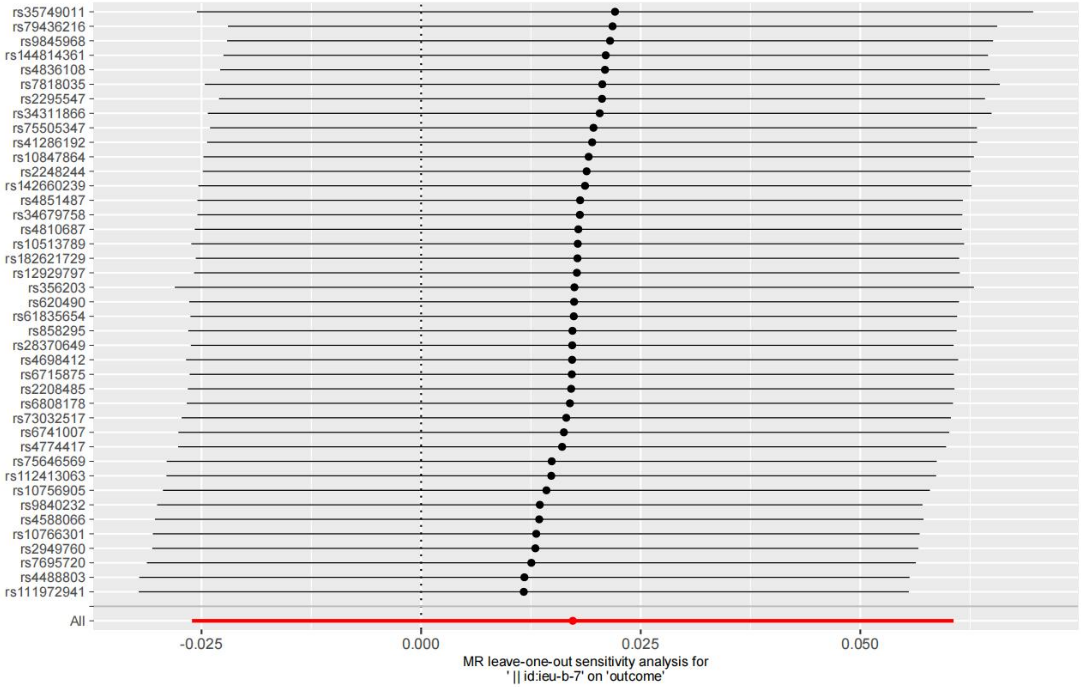

C. MR leave-one-out sensitivity analysis for PD on VEGF

eFigure 206. ALS-associated SNPs with BNGF

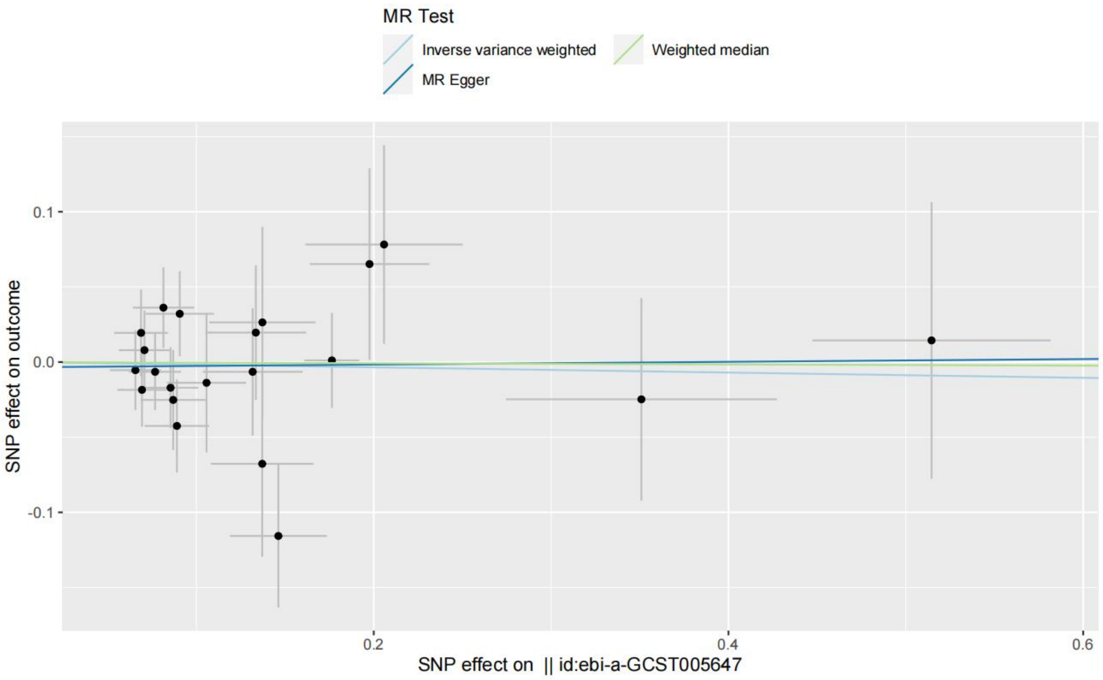

A. Scatter plot of ALS on BNGF

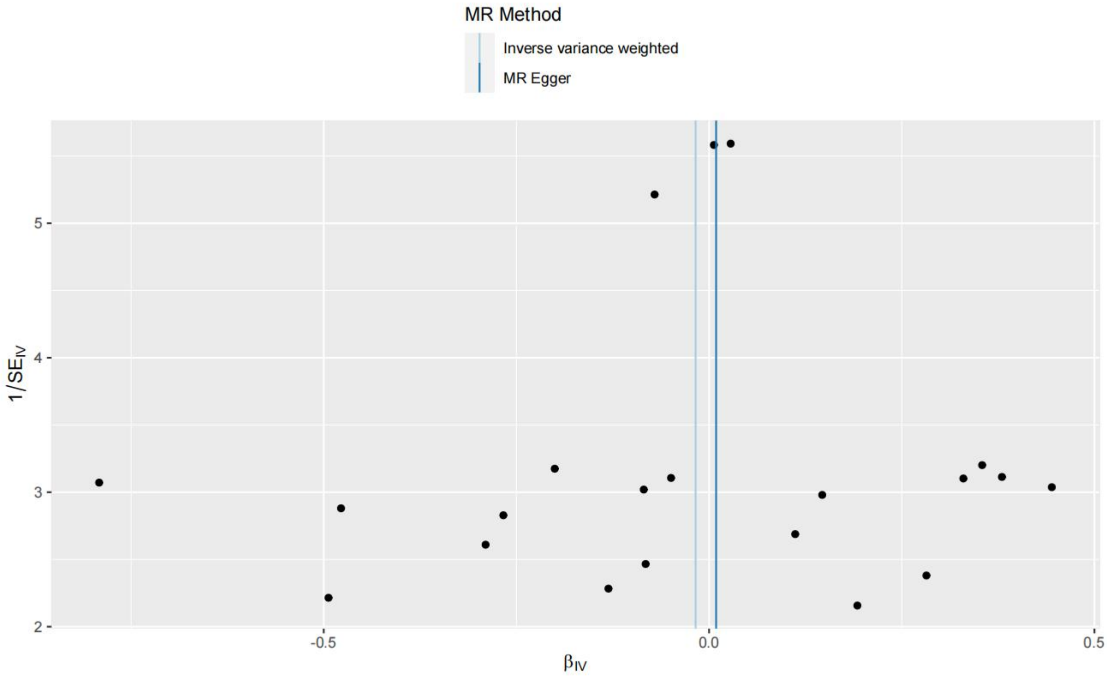

B. Funnel plot of ALS on BNGF

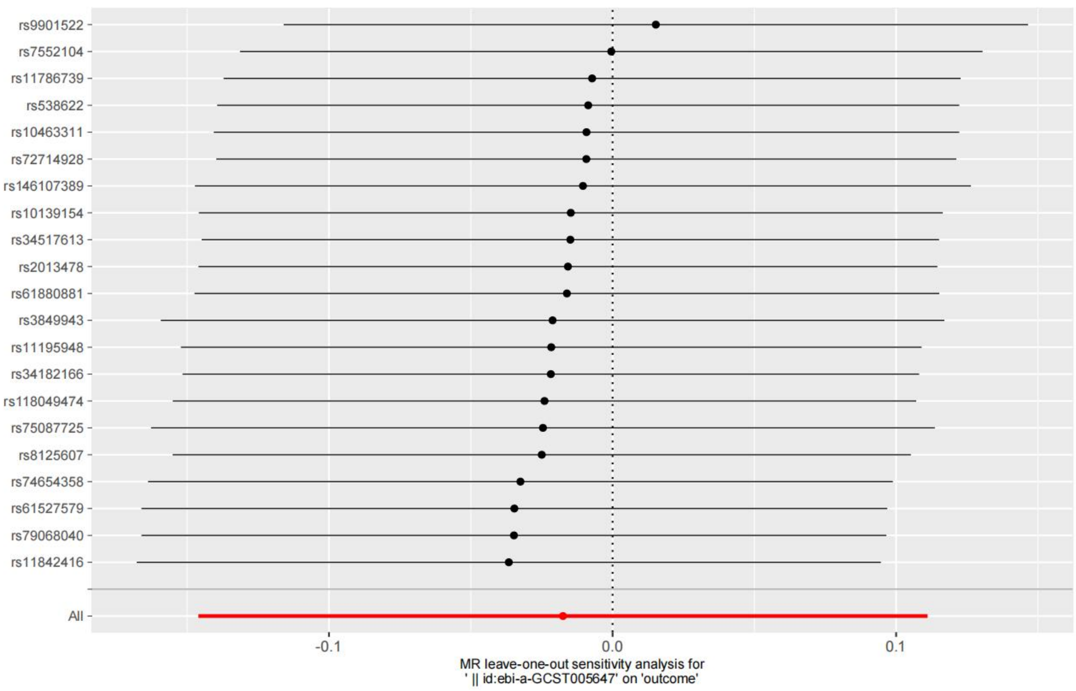

C. MR leave-one-out sensitivity analysis for ALS on BNGF

eFigure 207. ALS-associated SNPs with CTACK

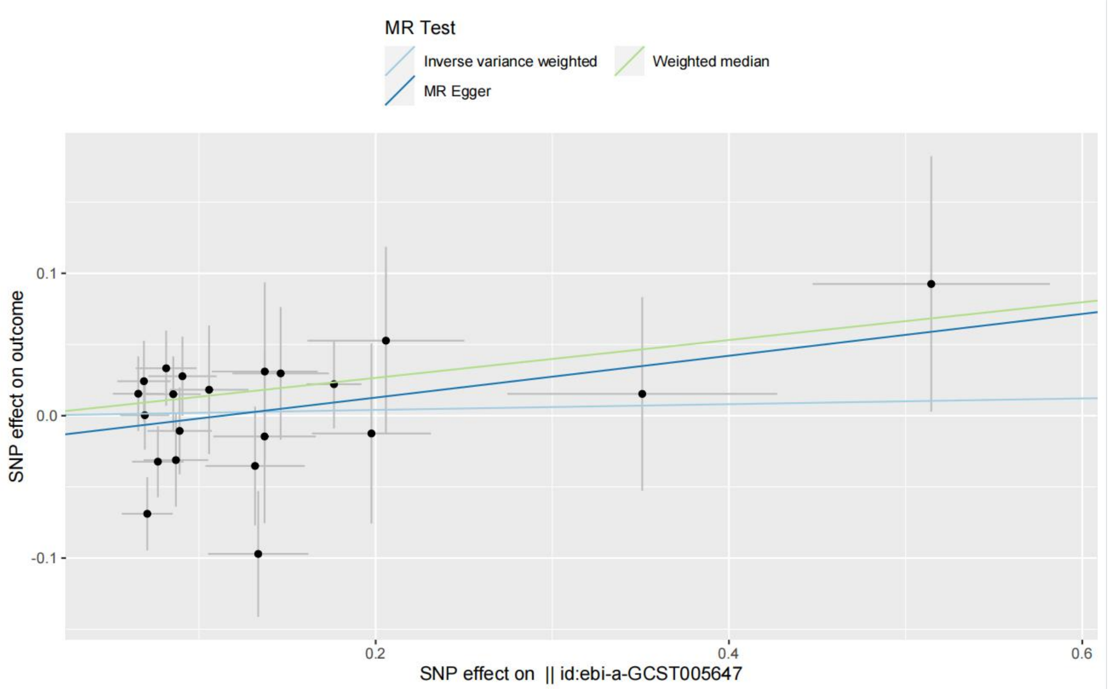

A. Scatter plot of ALS on CTACK

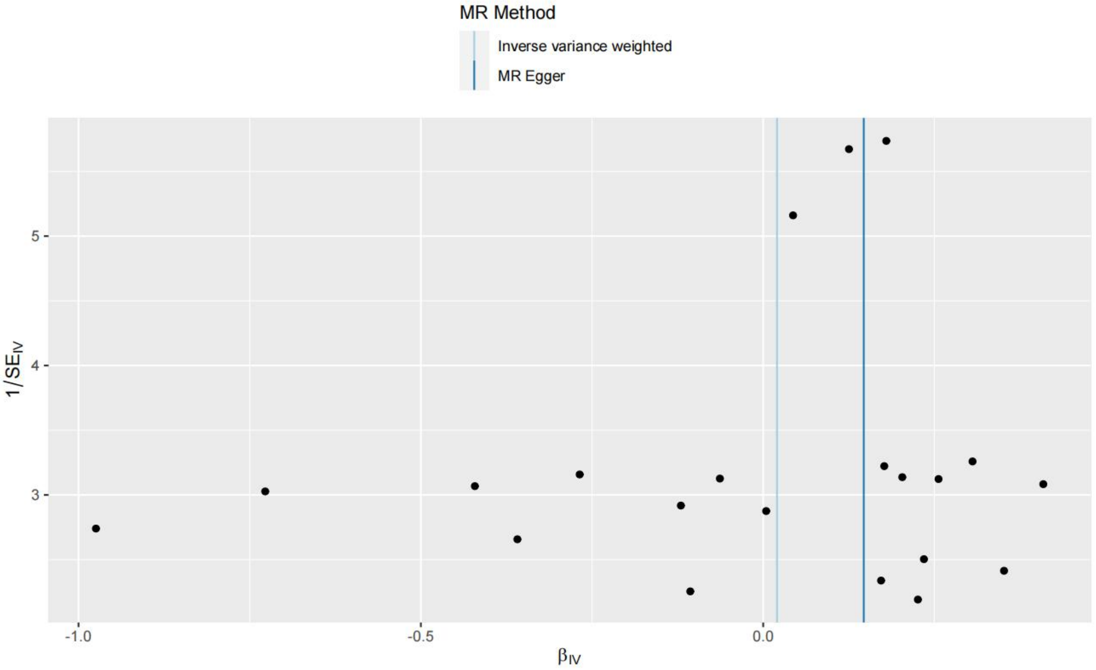

B. Funnel plot of ALS on CTACK

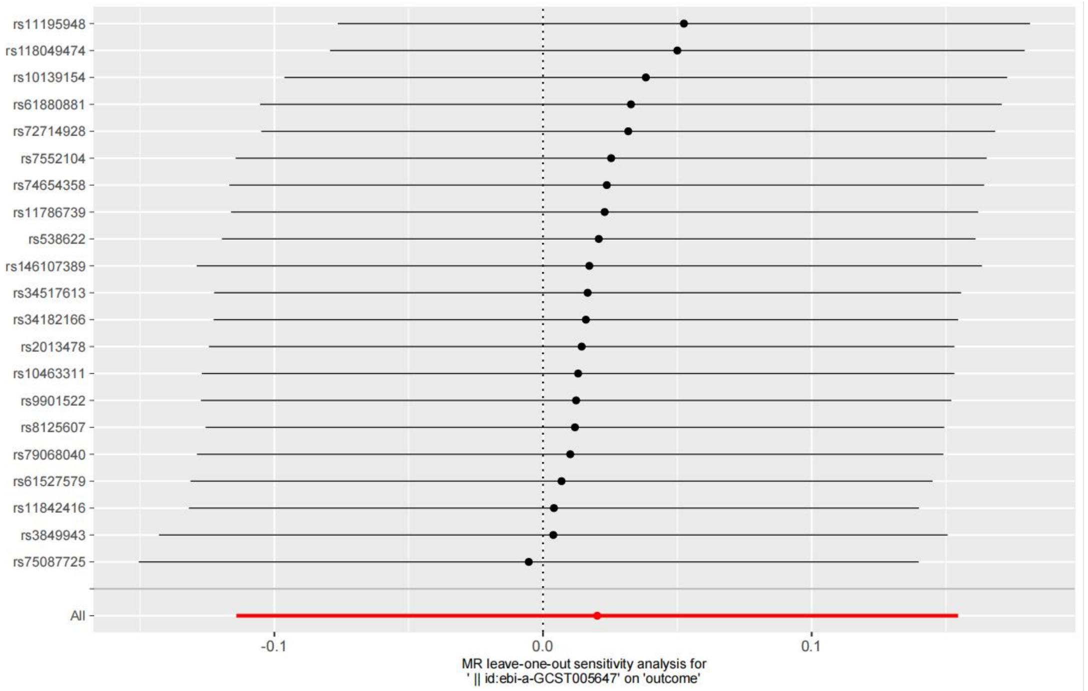

C. MR leave-one-out sensitivity analysis for ALS on CTACK

eFigure 208. ALS-associated SNPs with EOTAXIN

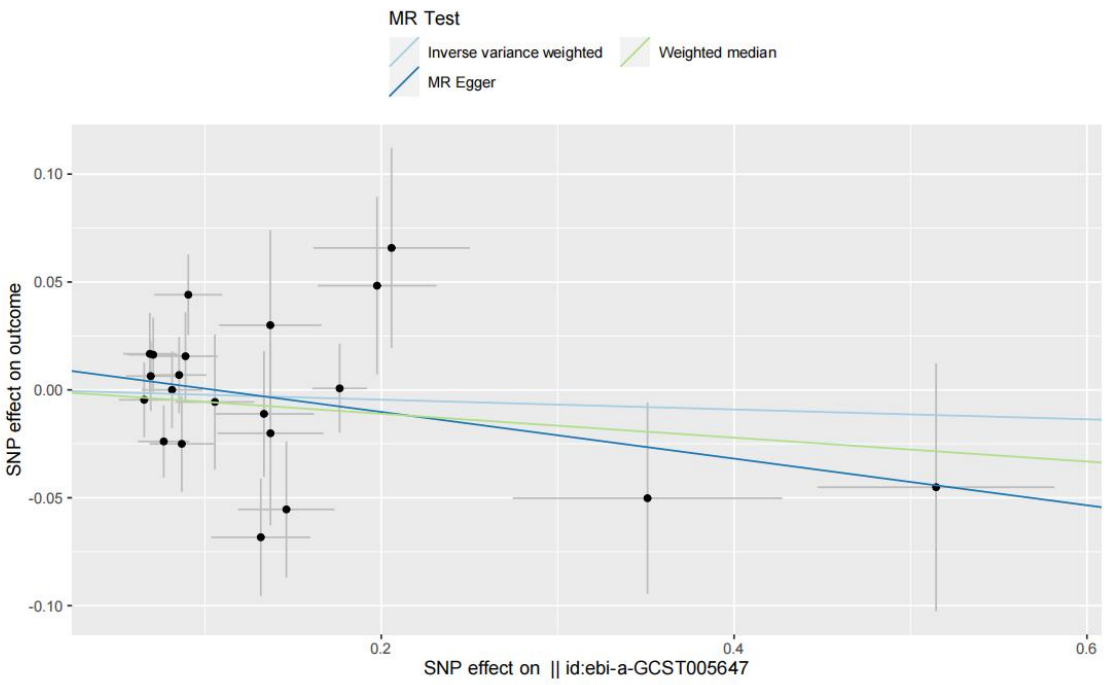

A. Scatter plot of ALS on EOTAXIN

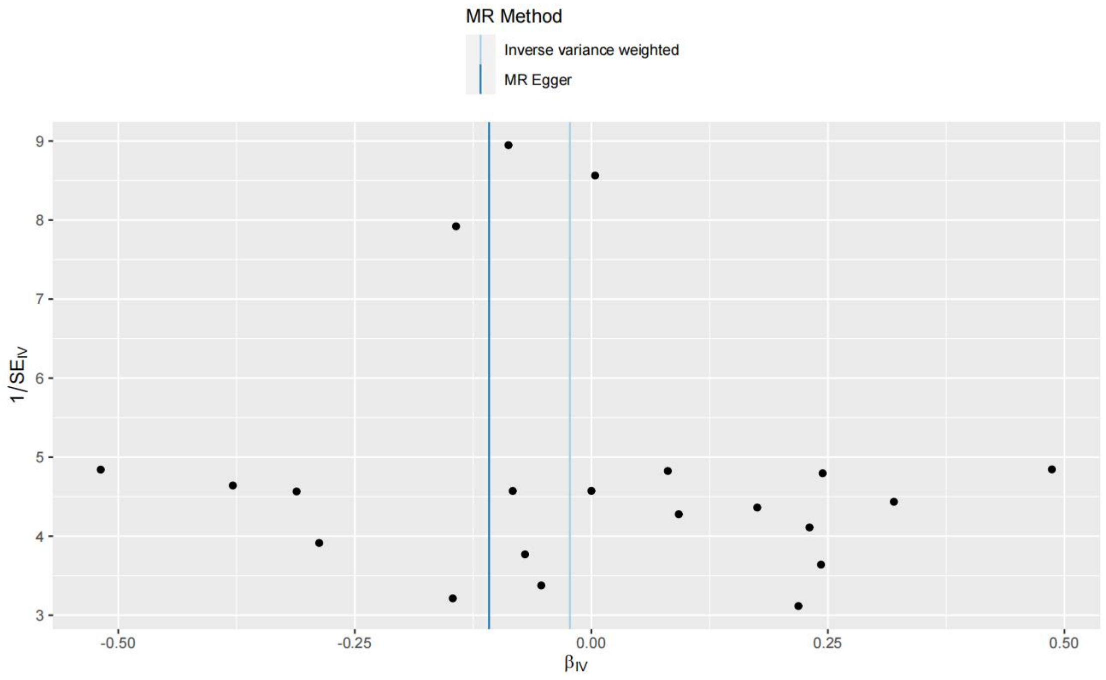

B. Funnel plot of ALS on EOTAXIN

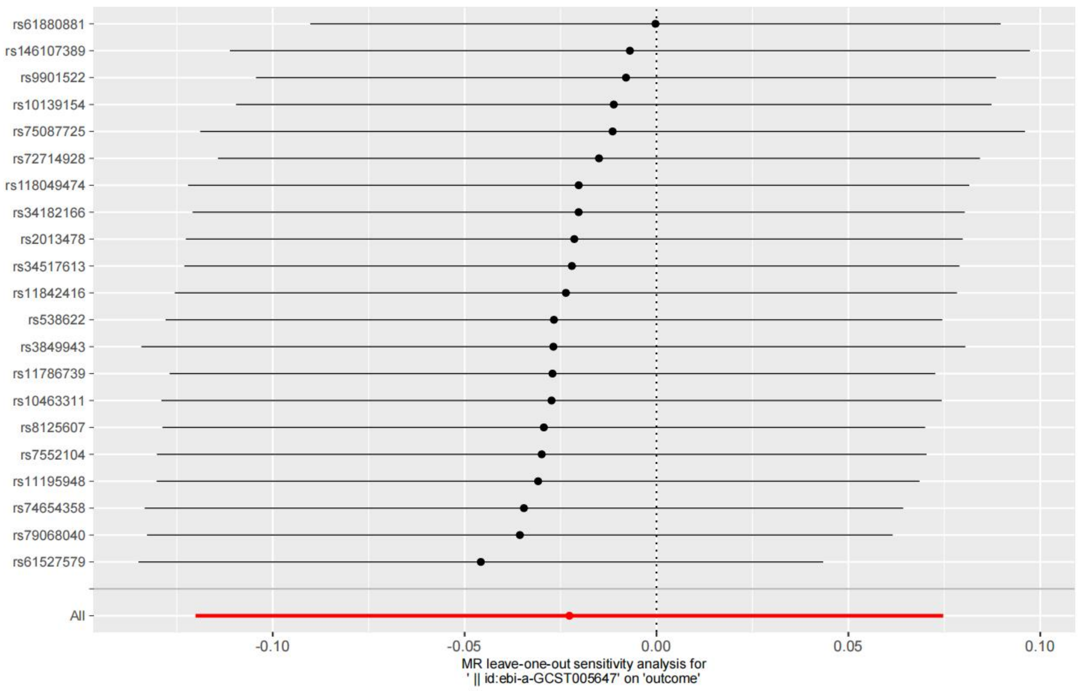

C. MR leave-one-out sensitivity analysis for ALS on EOTAXIN

eFigure 209. ALS-associated SNPs with bFGF

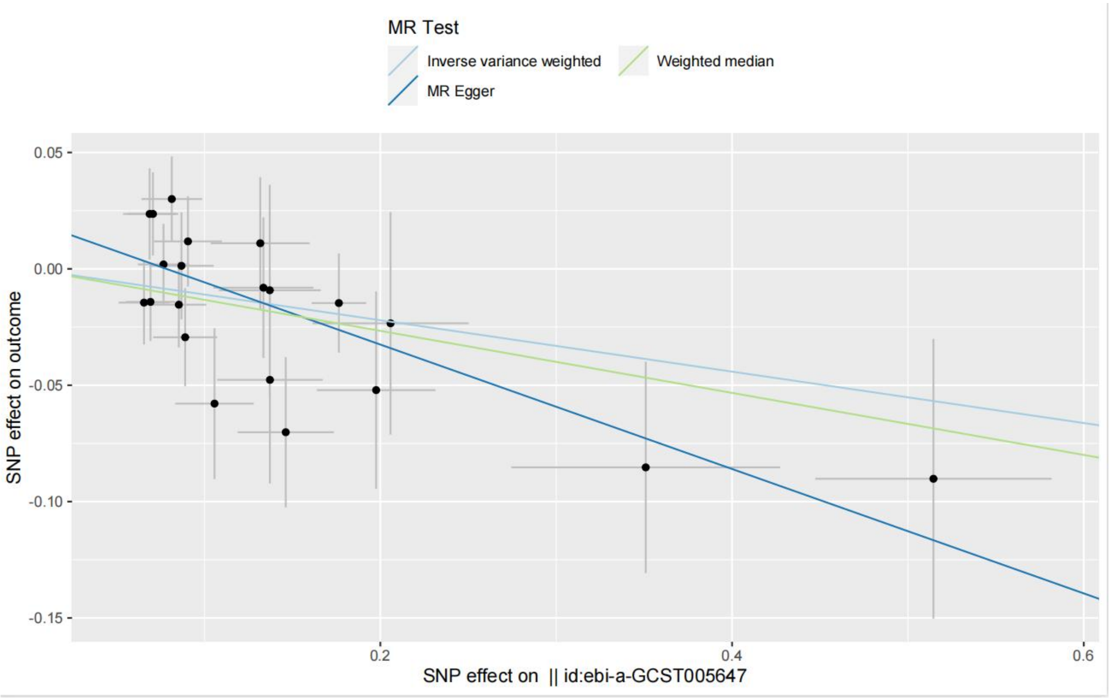

A. Scatter plot of ALS on bFGF

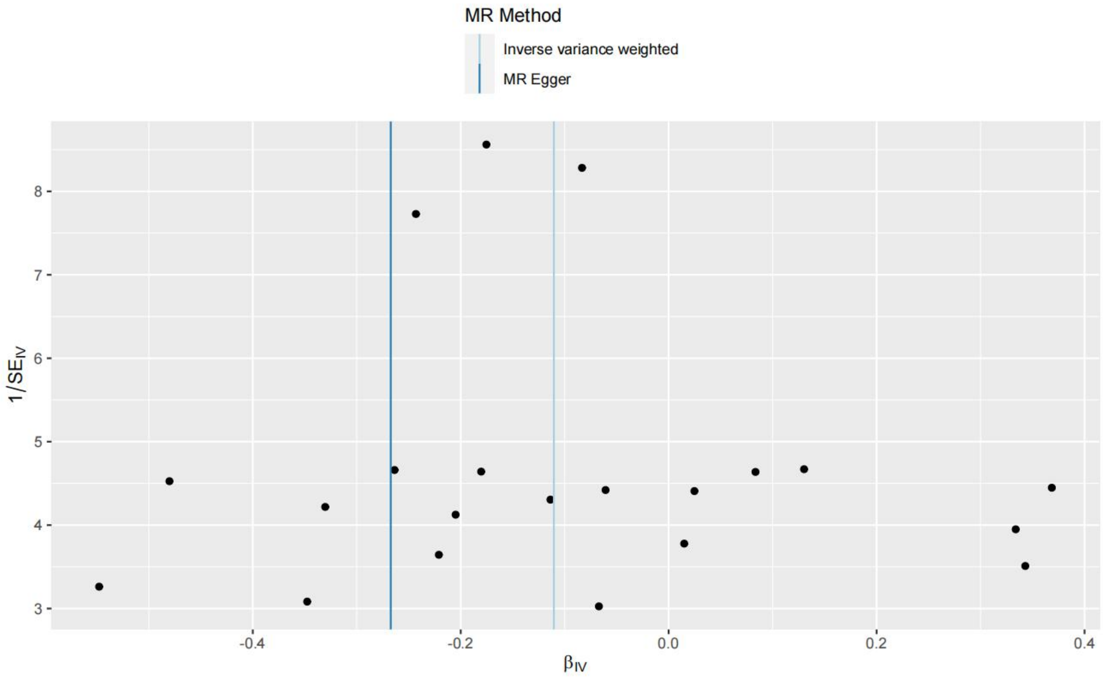

B. Funnel plot of ALS on bFGF

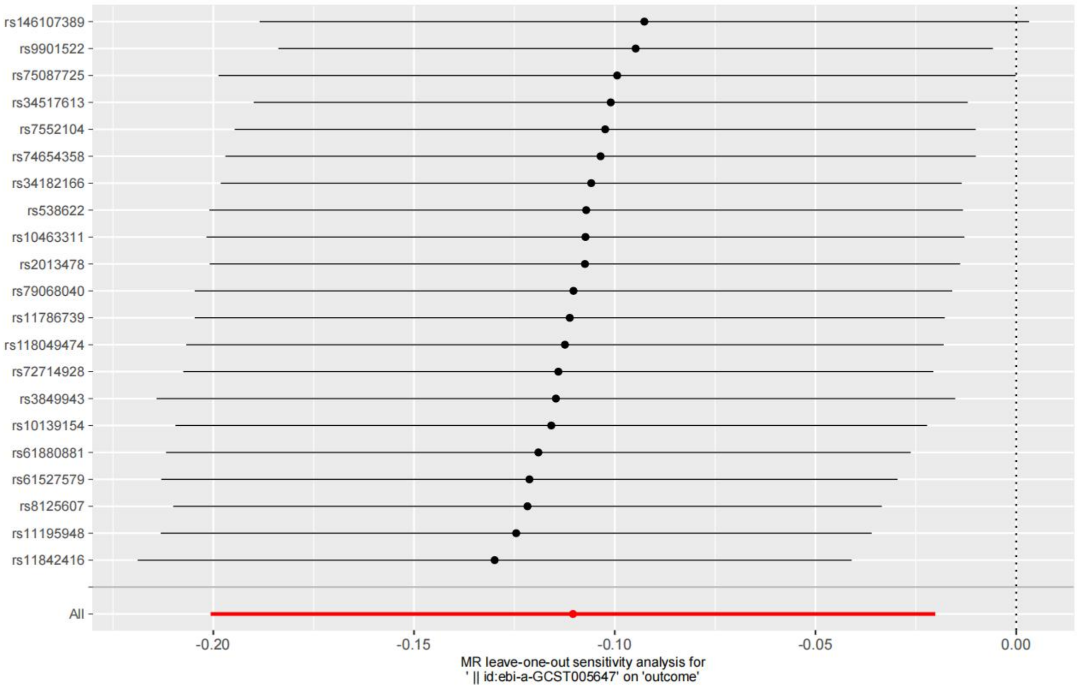

C. MR leave-one-out sensitivity analysis for ALS on bFGF

eFigure 210. ALS-associated SNPs with G-CSF

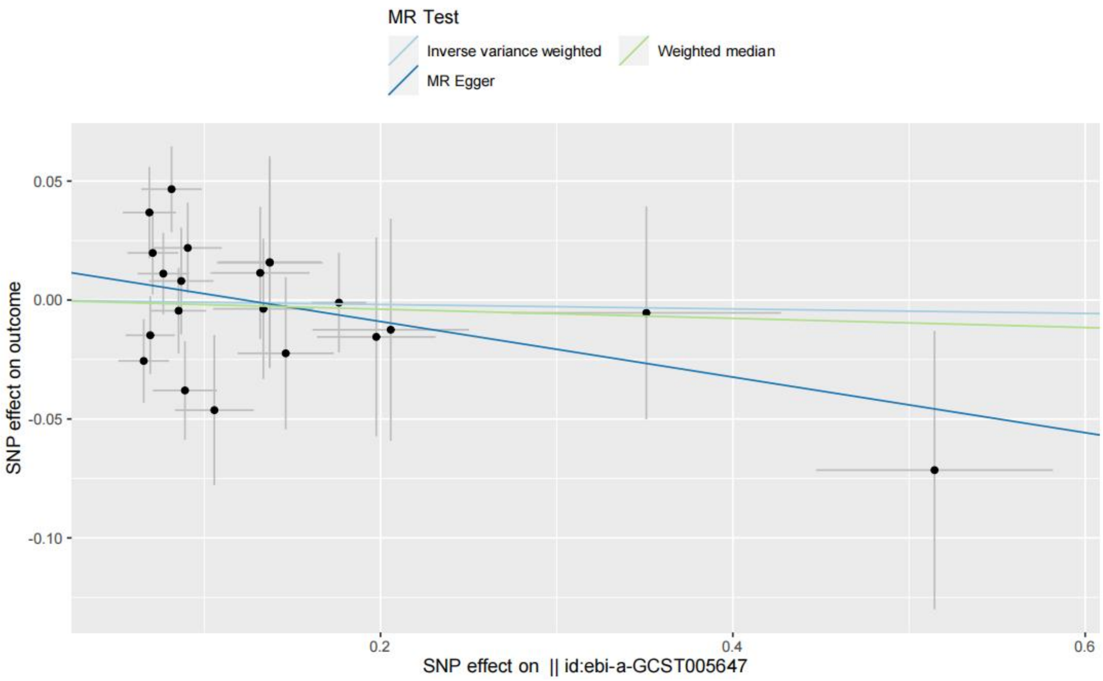

A. Scatter plot of ALS on G-CSF

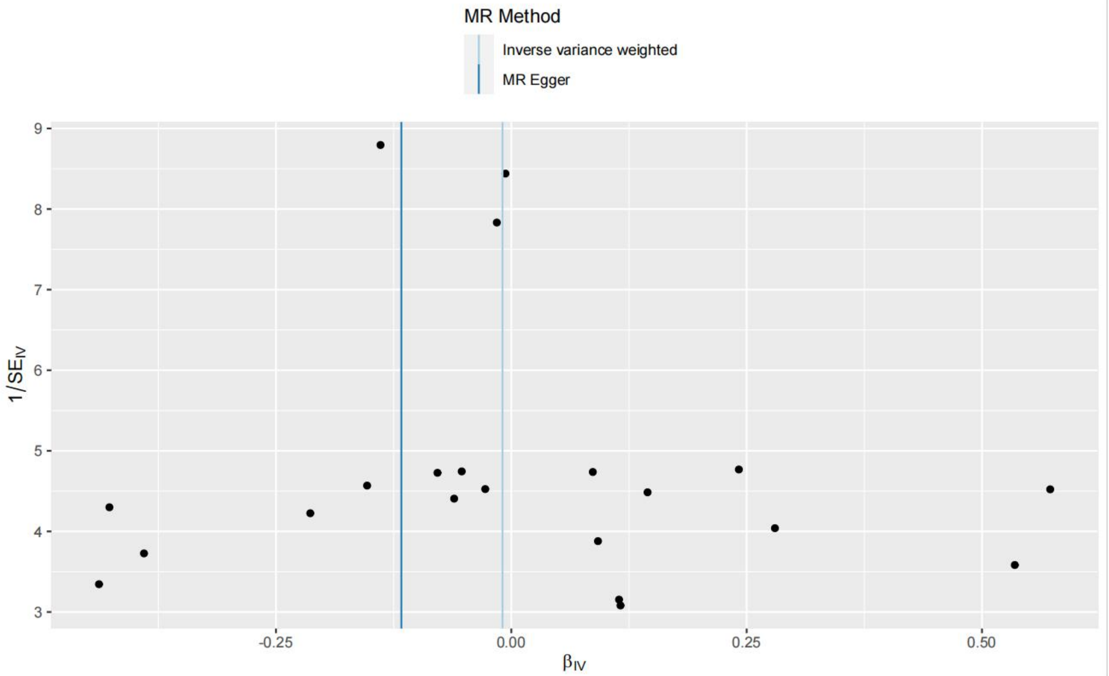

B. Funnel plot of ALS on G-CSF

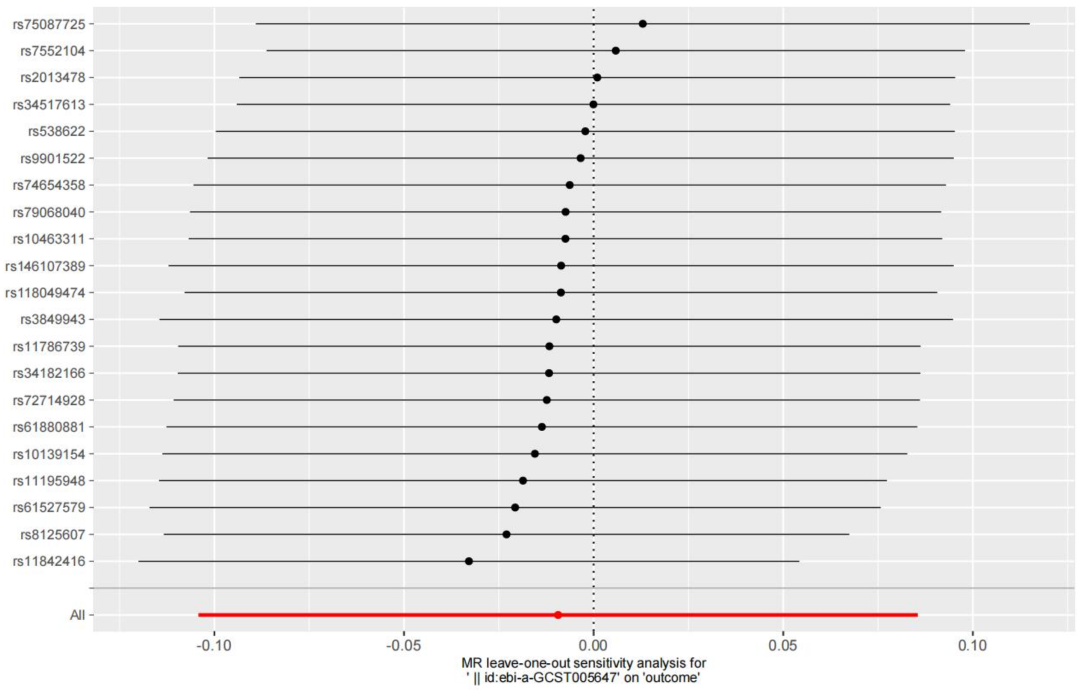

C. MR leave-one-out sensitivity analysis for ALS on G-CSF

eFigure 211. ALS-associated SNPs with GROA

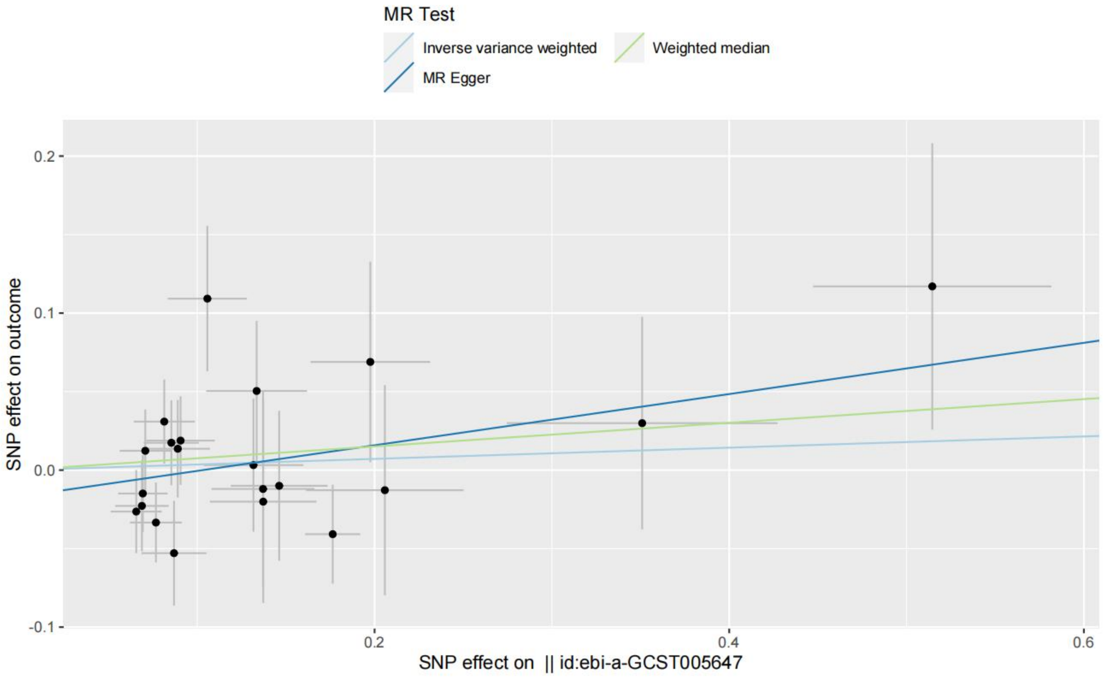

A. Scatter plot of ALS on GROA

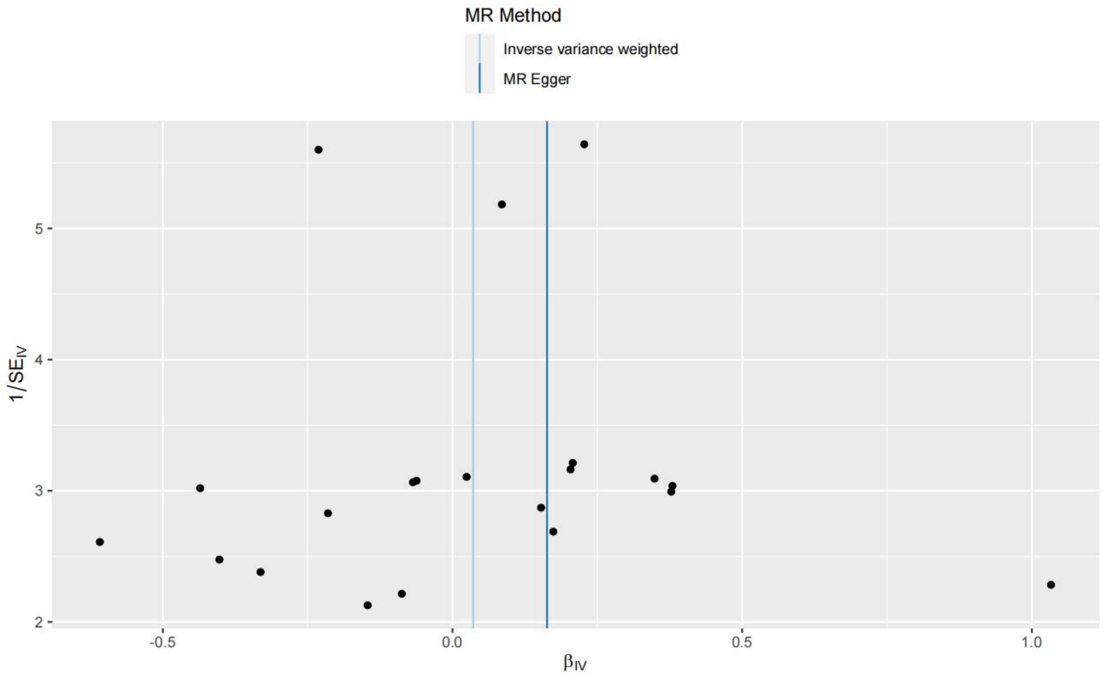

B. Funnel plot of ALS on GROA

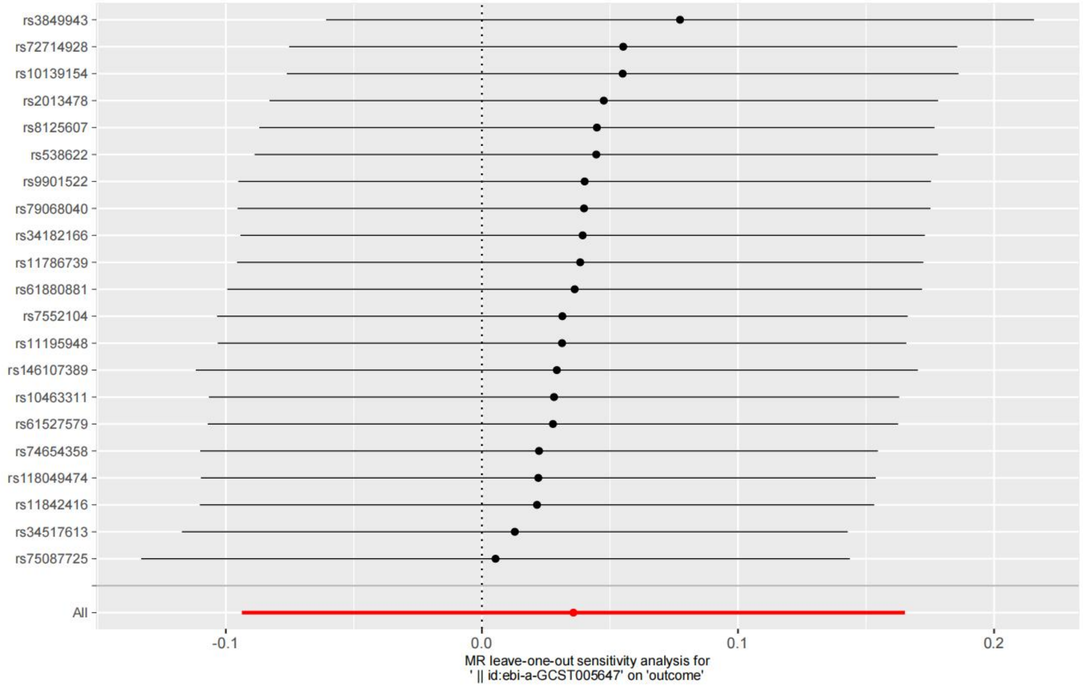

C. MR leave-one-out sensitivity analysis for ALS on GROA

eFigure 212. ALS-associated SNPs with HGF

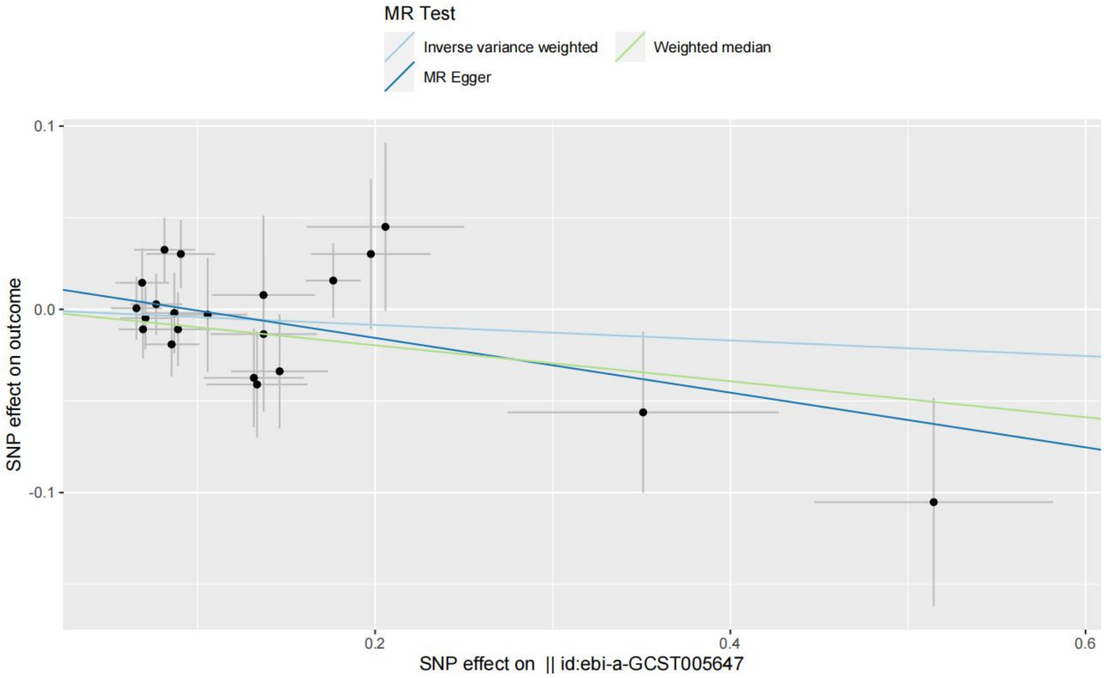

A. Scatter plot of ALS on HGF

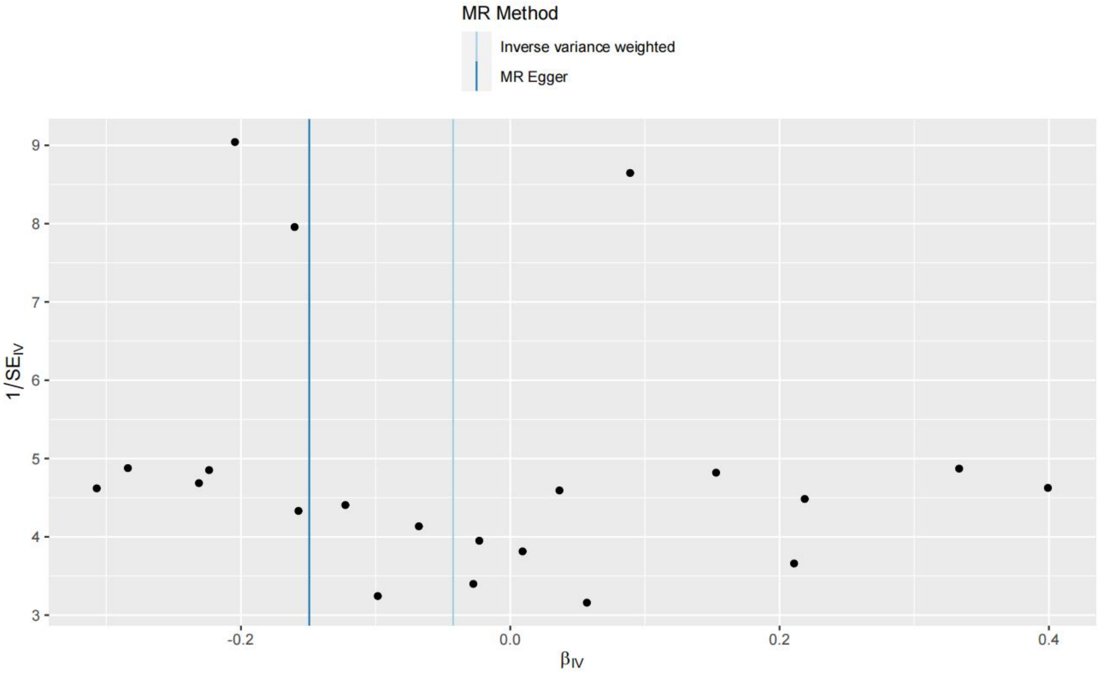

B. Funnel plot of ALS on HGF

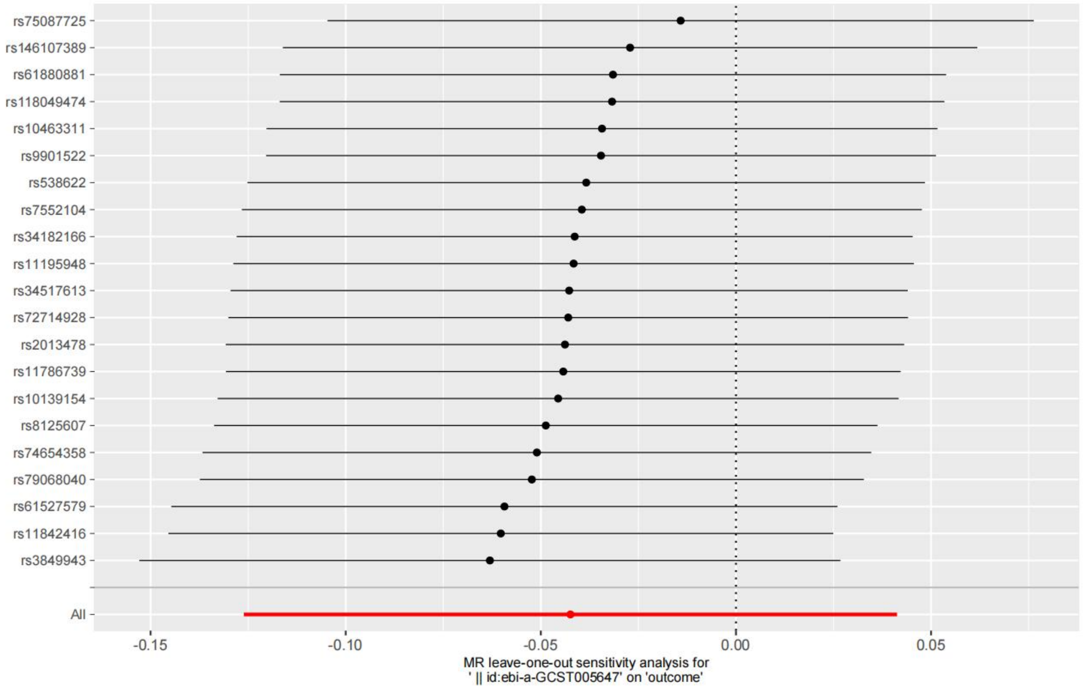

C. MR leave-one-out sensitivity analysis for ALS on HGF

eFigure 213. ALS-associated SNPs with IFN-G

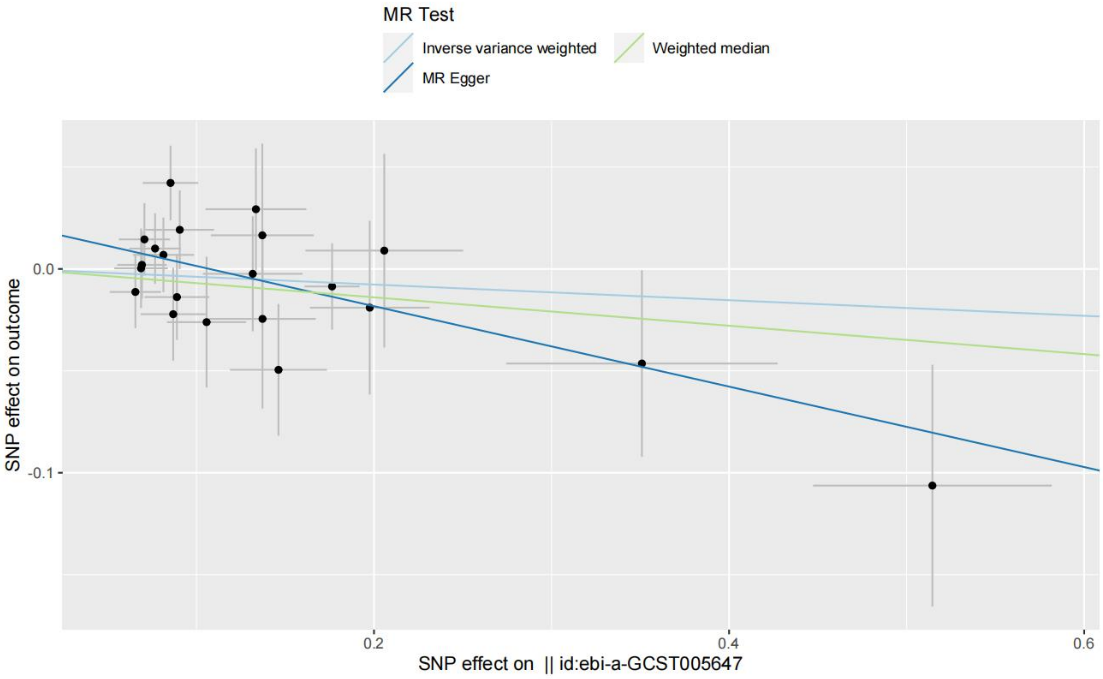

A. Scatter plot of ALS on IFN-G

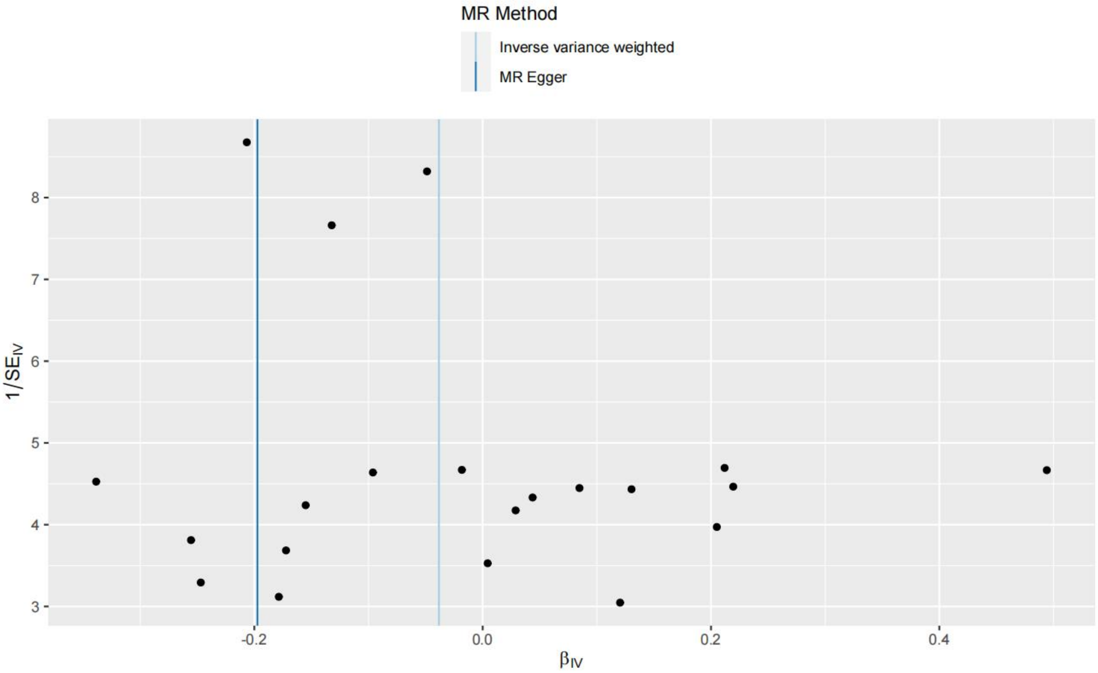

B. Funnel plot of ALS on IFN-G

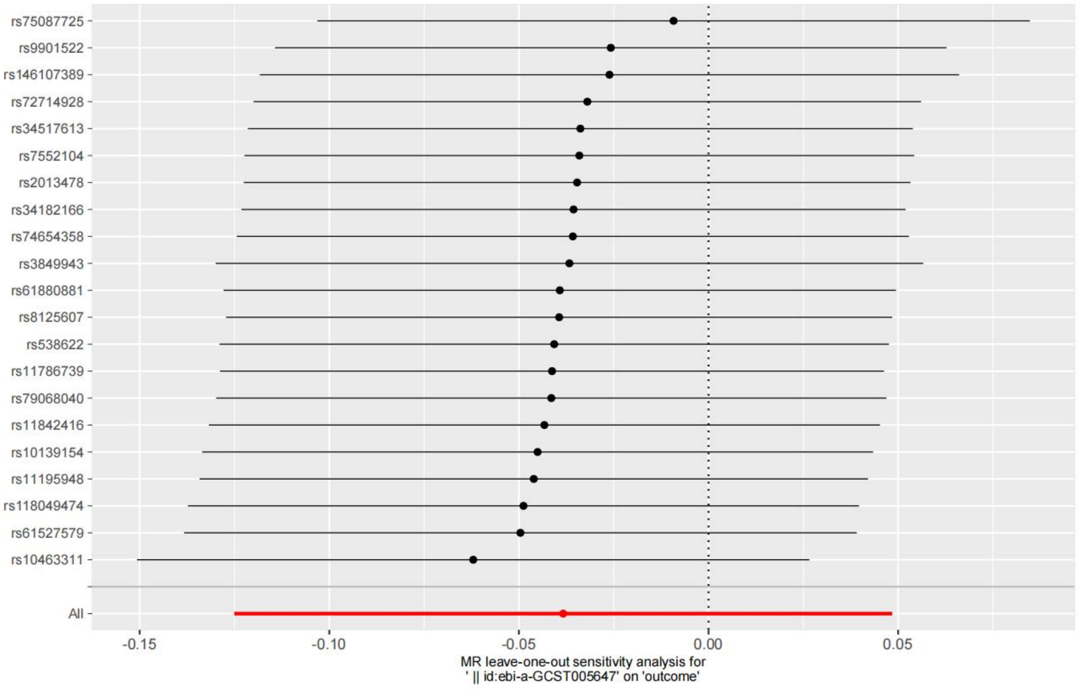

C. MR leave-one-out sensitivity analysis for ALS on IFN-G

eFigure 214. ALS-associated SNPs with IL-1B

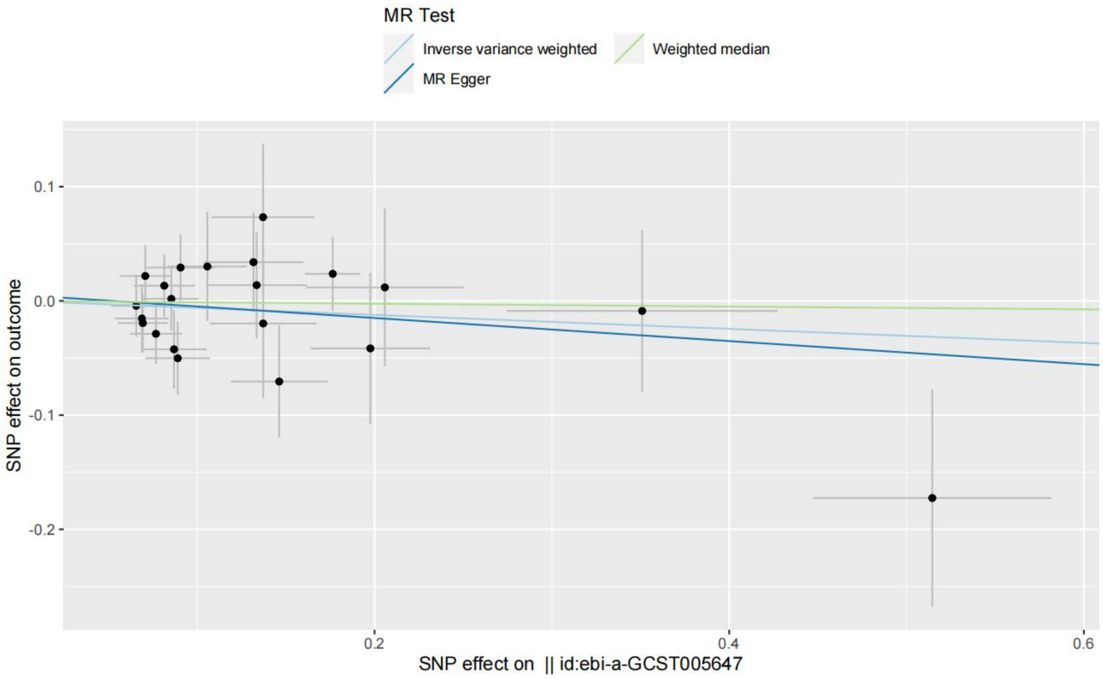

A. Scatter plot of ALS on IL-1B

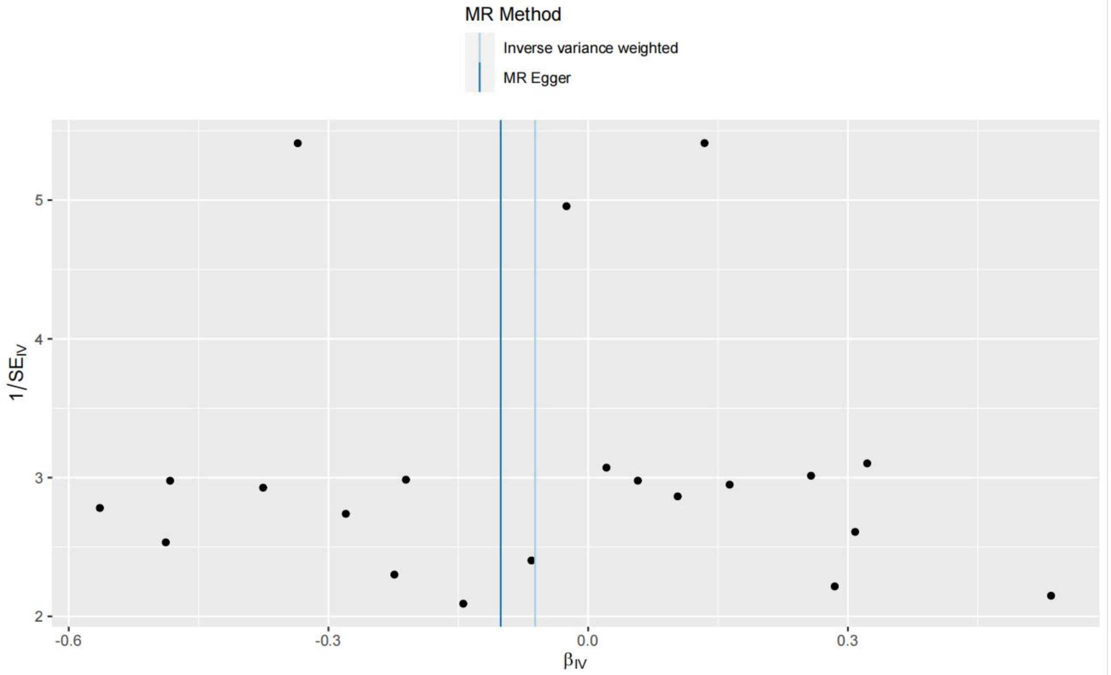

B. Funnel plot of ALS on IL-1B

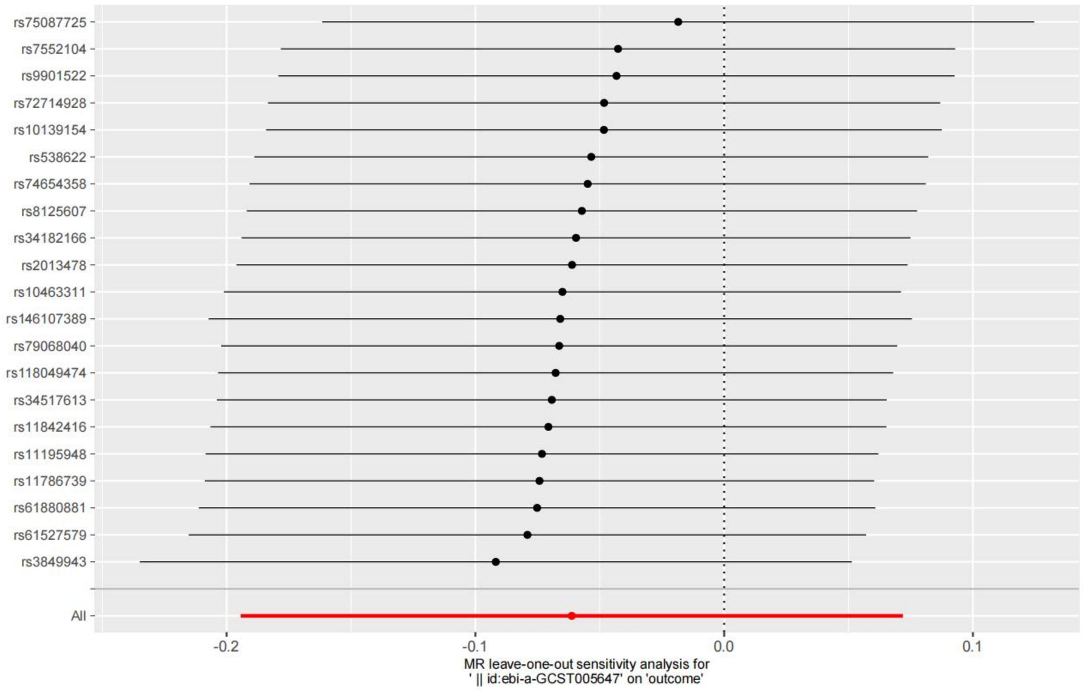

C. MR leave-one-out sensitivity analysis for ALS on IL-1B

eFigure 215. ALS-associated SNPs with IL-1RA

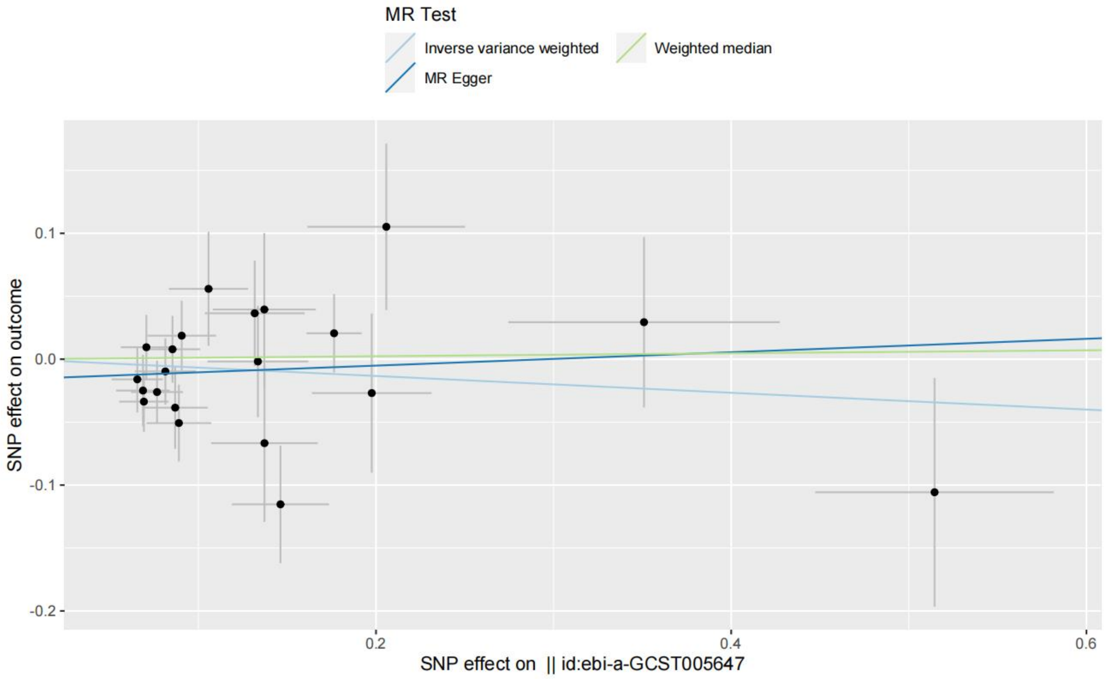

A. Scatter plot of ALS on IL-1RA

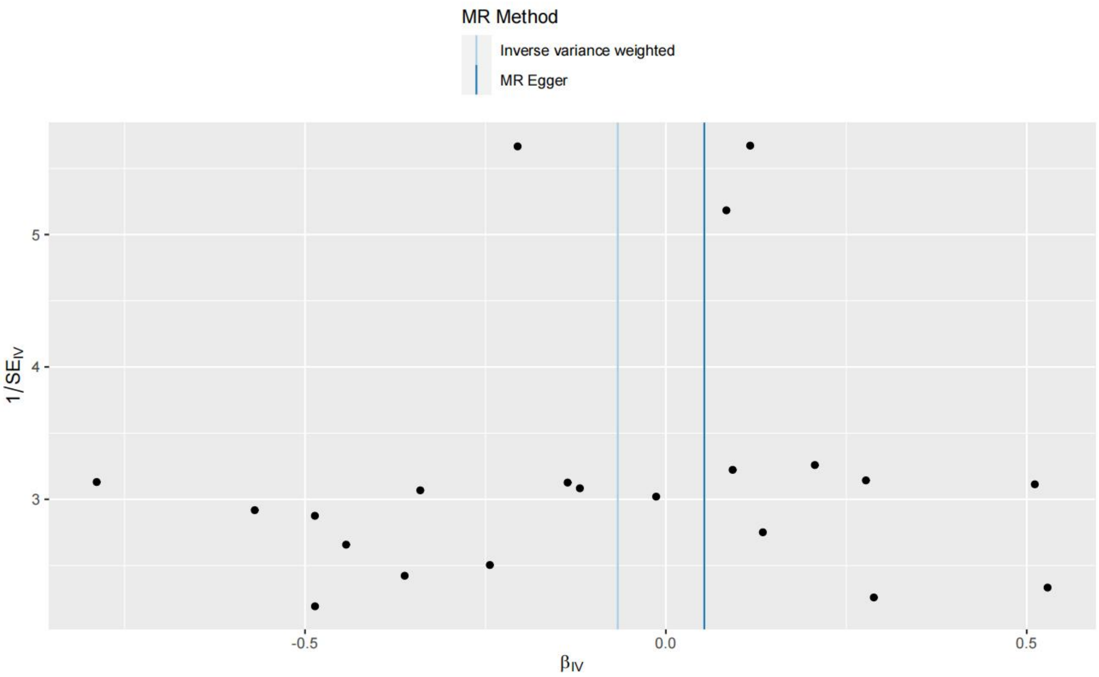

B. Funnel plot of ALS on IL-1RA

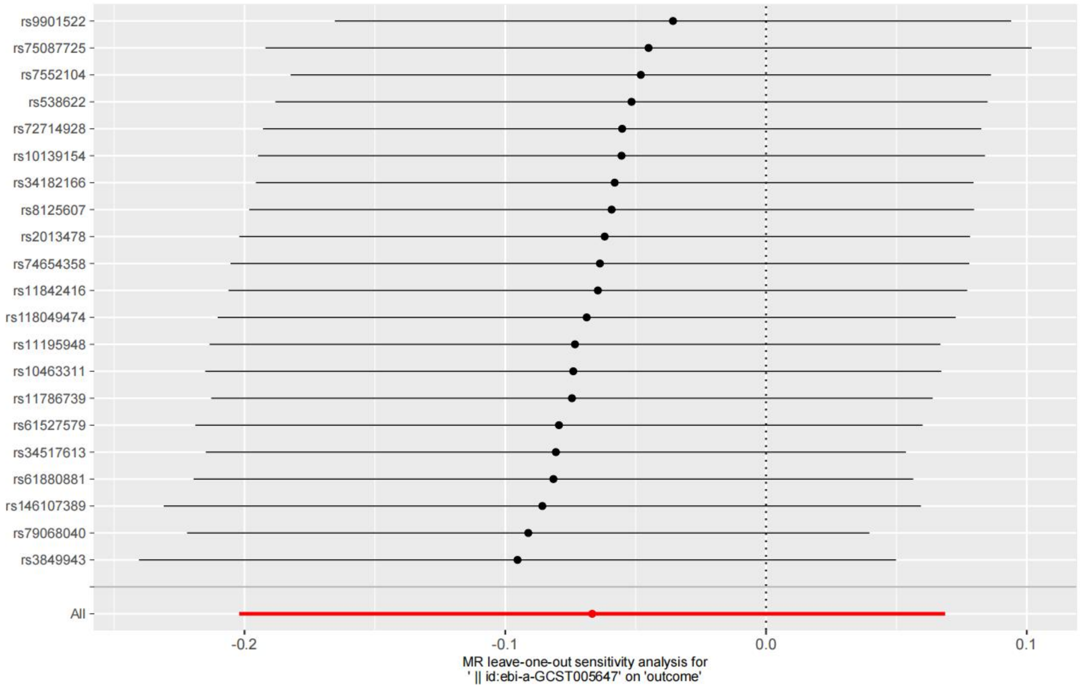

C. MR leave-one-out sensitivity analysis for ALS on IL-1RA

eFigure 216. ALS-associated SNPs with IL-2

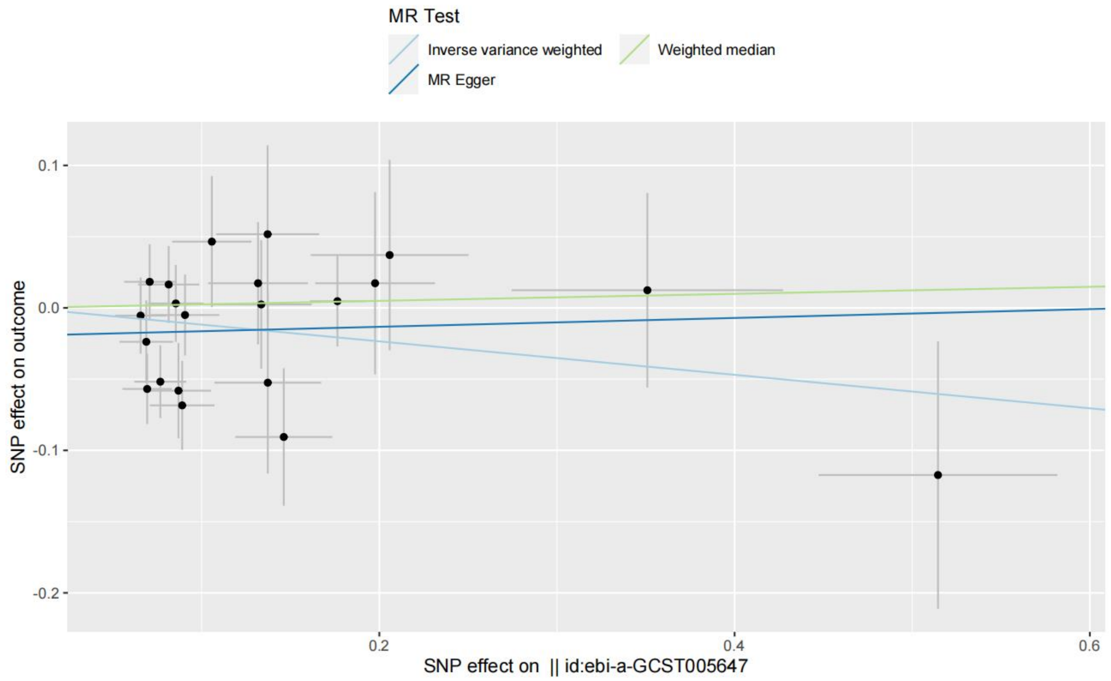

A. Scatter plot of ALS on IL-2

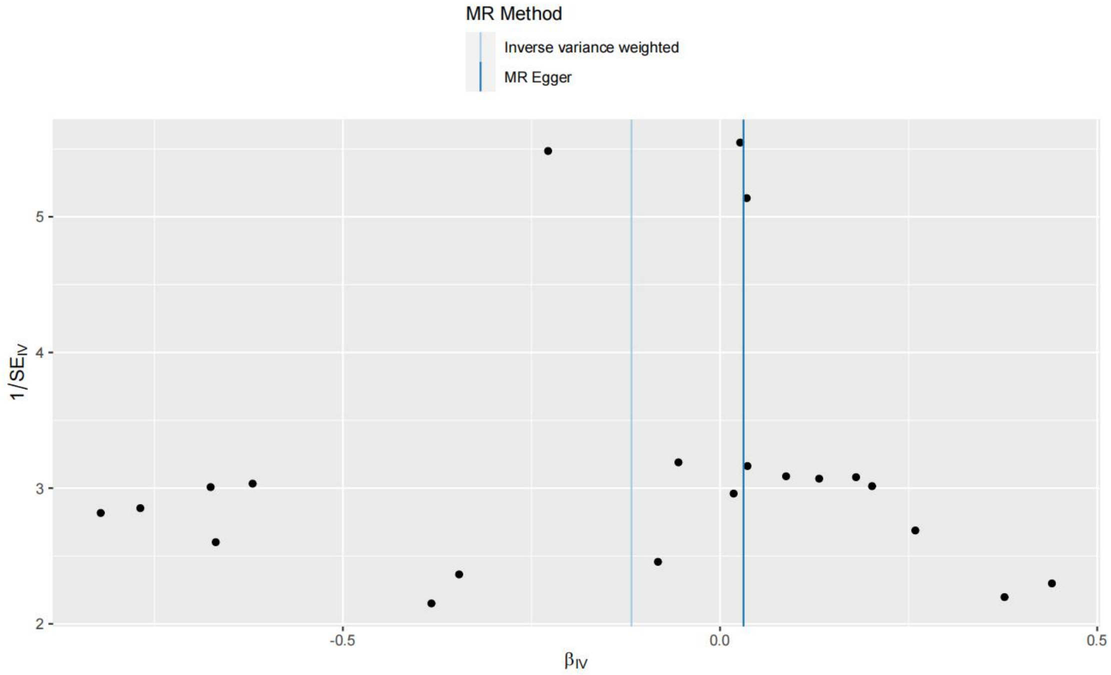

B. Funnel plot of ALS on IL-2

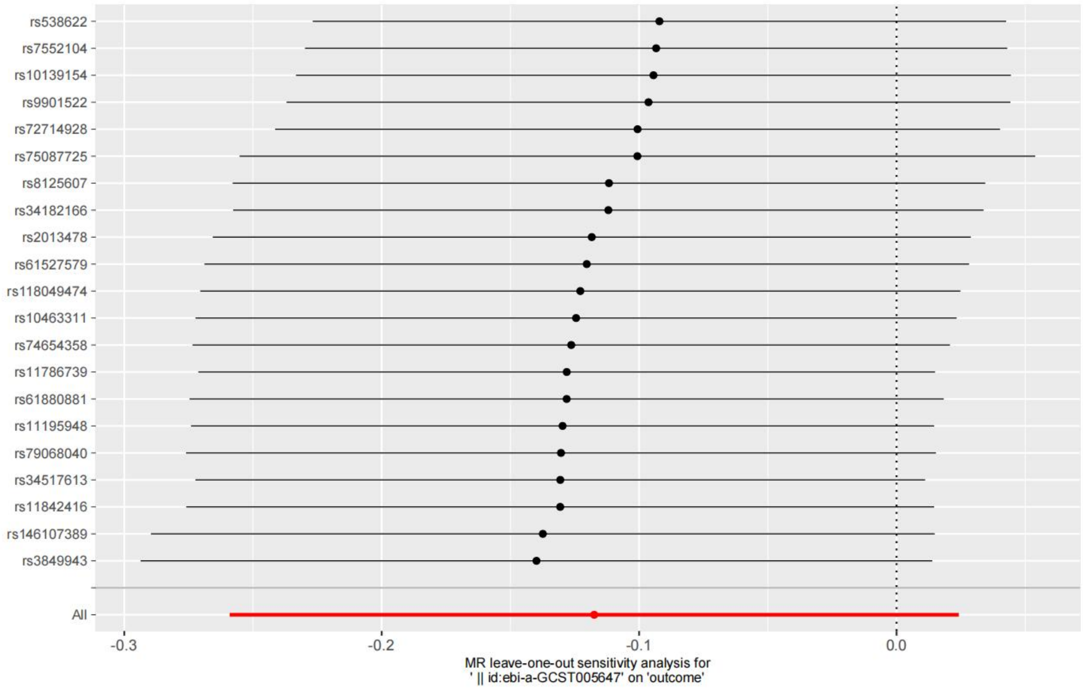

C. MR leave-one-out sensitivity analysis for ALS on IL-2

eFigure 217. ALS-associated SNPs with IL-2RA

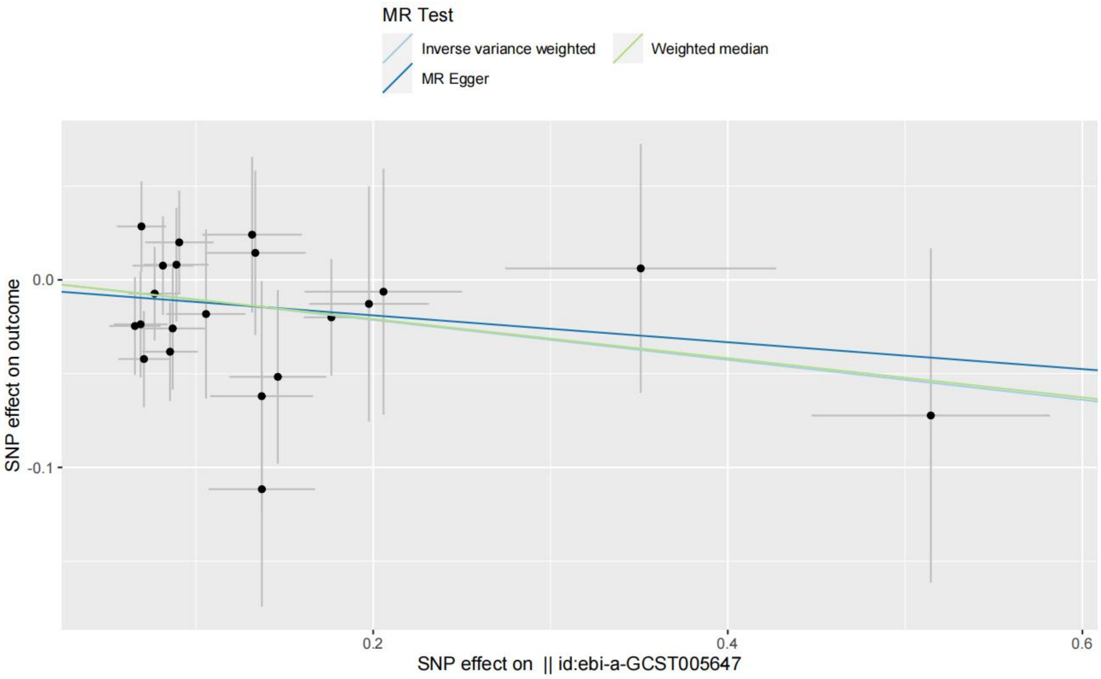

A. Scatter plot of ALS on IL-2RA

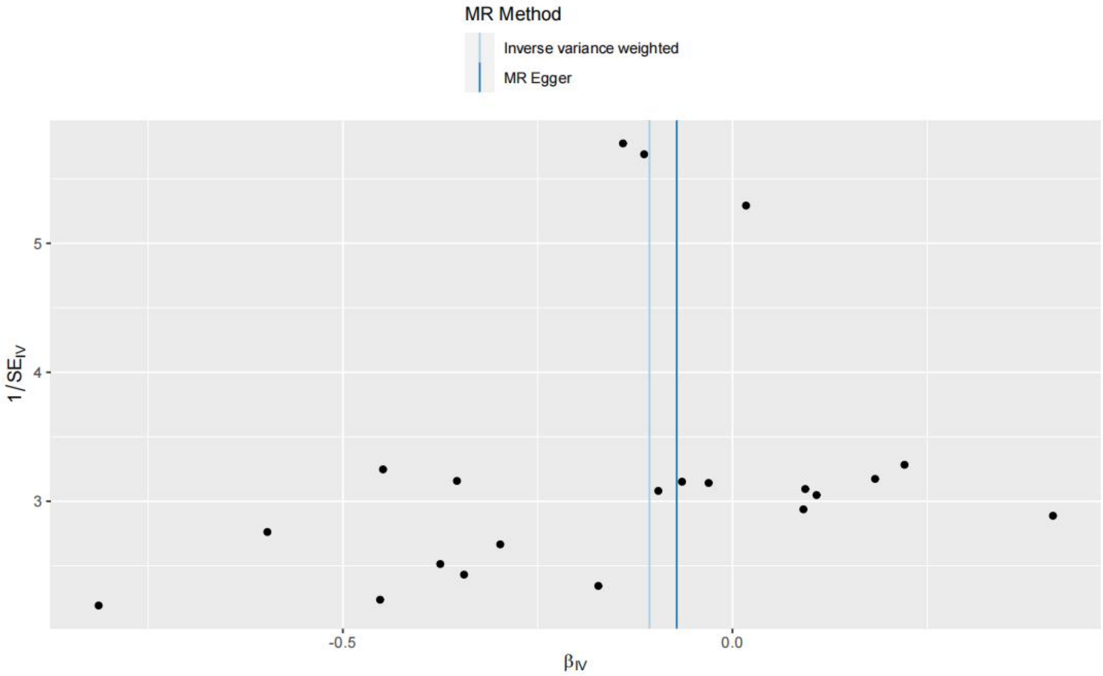

B. Funnel plot of ALS on IL-2RA

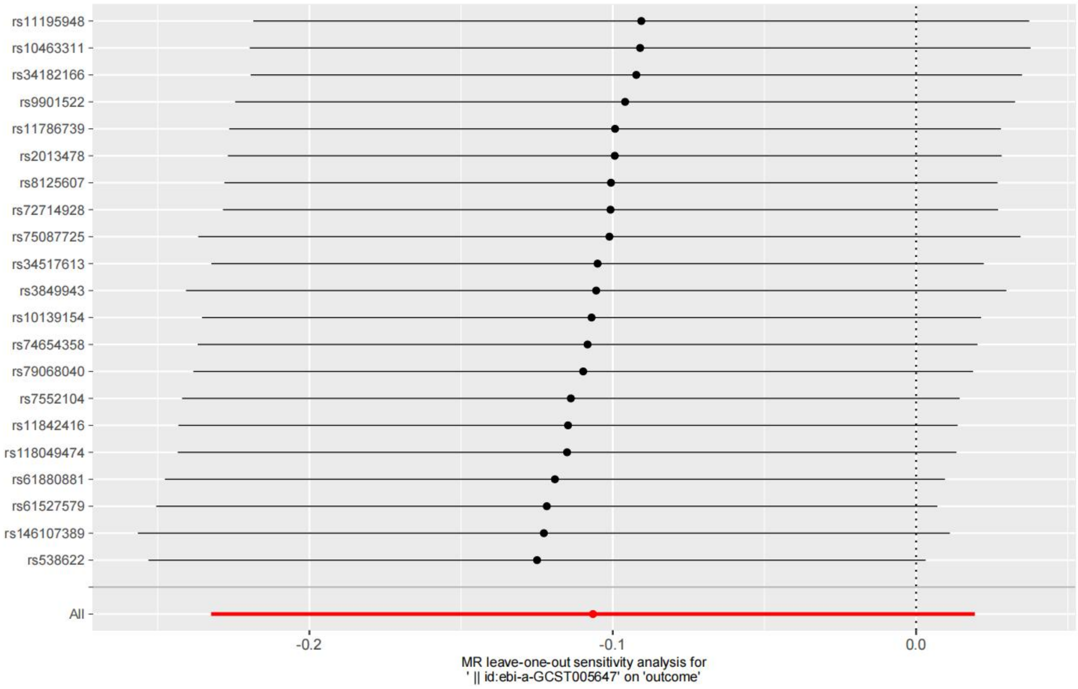

C. MR leave-one-out sensitivity analysis for ALS on IL-2RA

eFigure 218. ALS-associated SNPs with IL-4

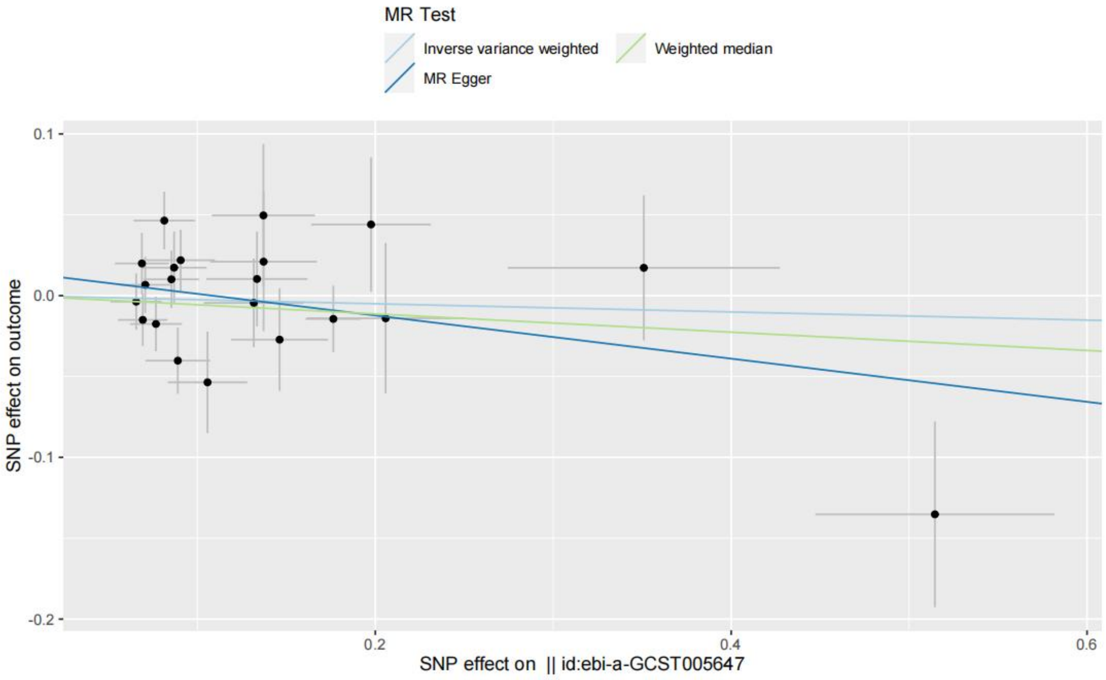

A. Scatter plot of ALS on IL-4

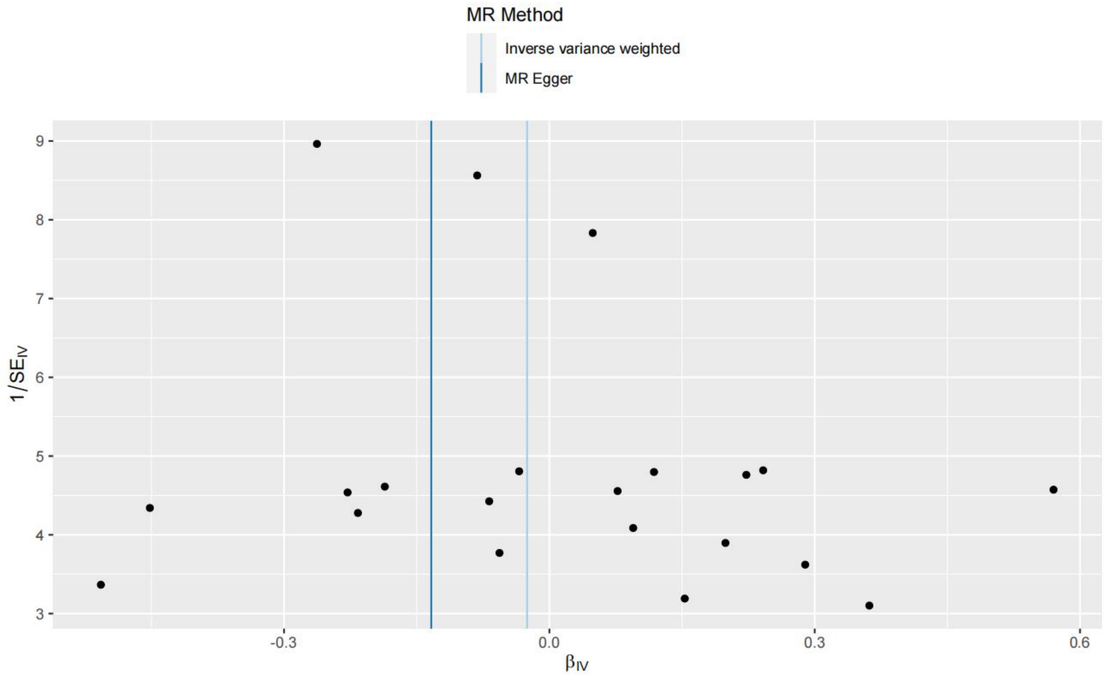

B. Funnel plot of ALS on IL-4

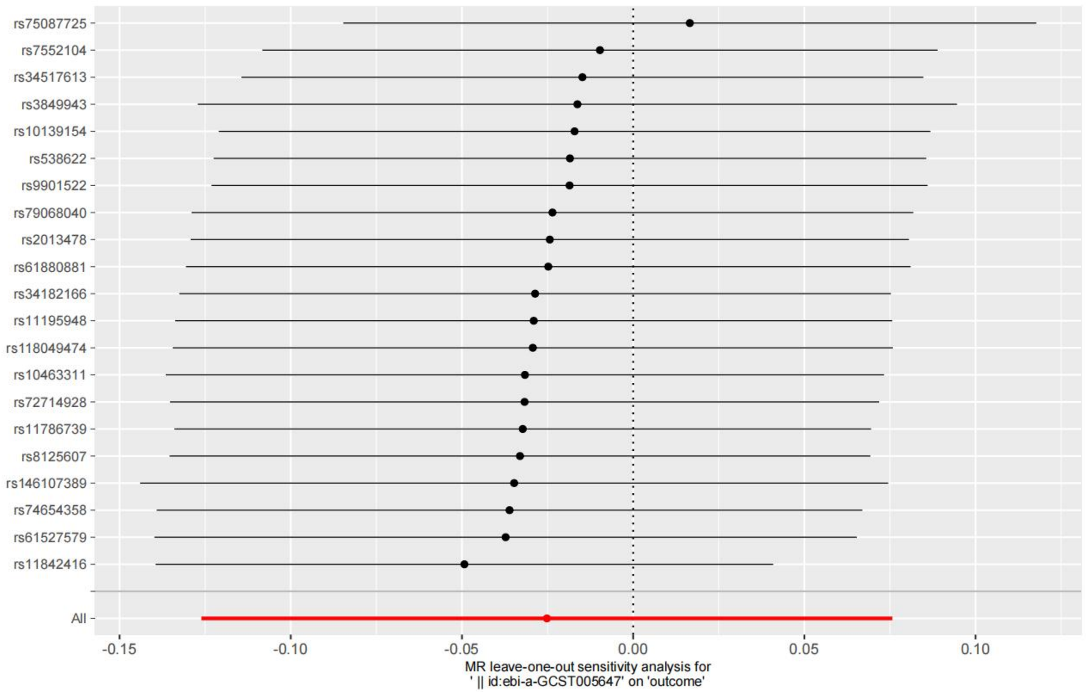

C. MR leave-one-out sensitivity analysis for ALS on IL-4

eFigure 219. ALS-associated SNPs with IL-5

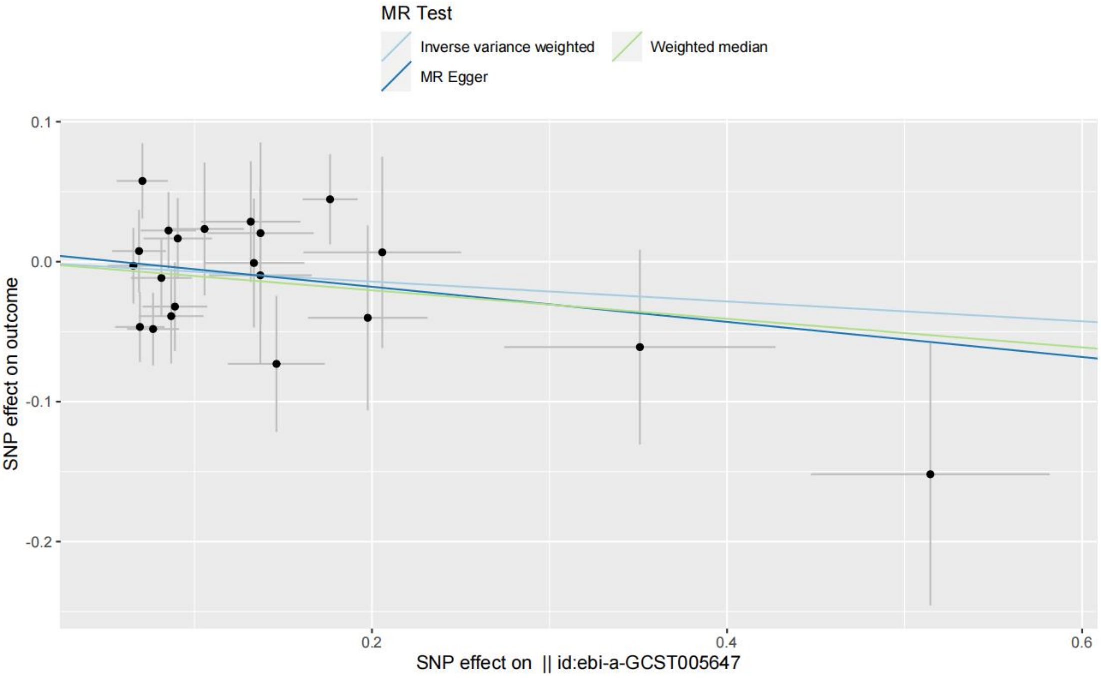

A. Scatter plot of ALS on IL-5

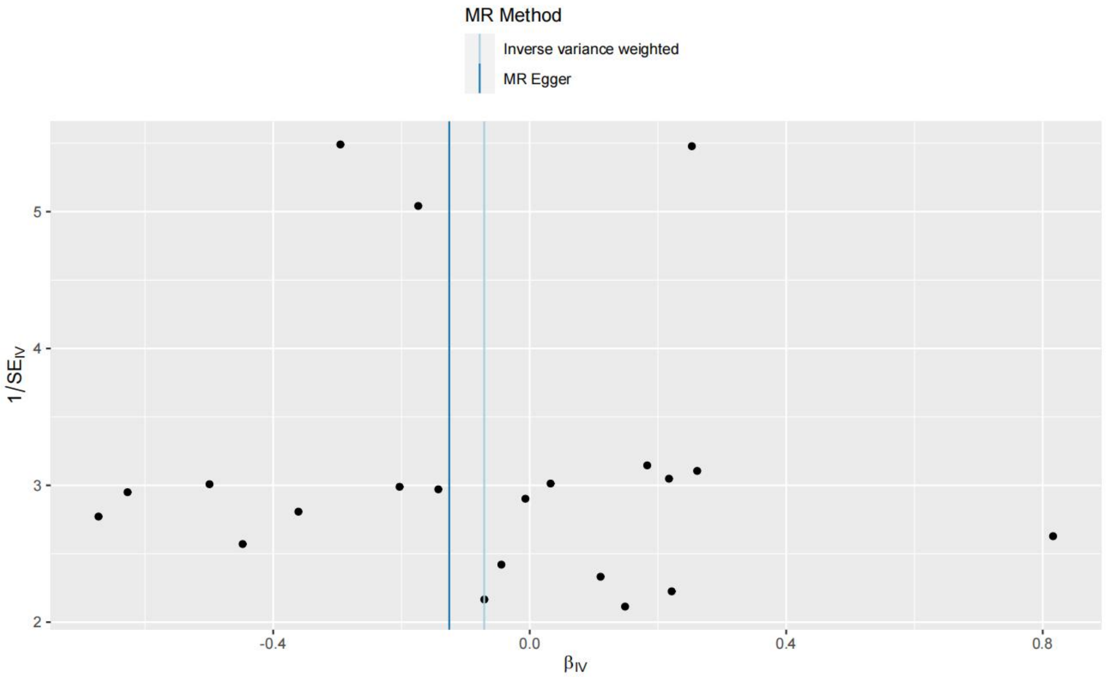

B. Funnel plot of ALS on IL-5

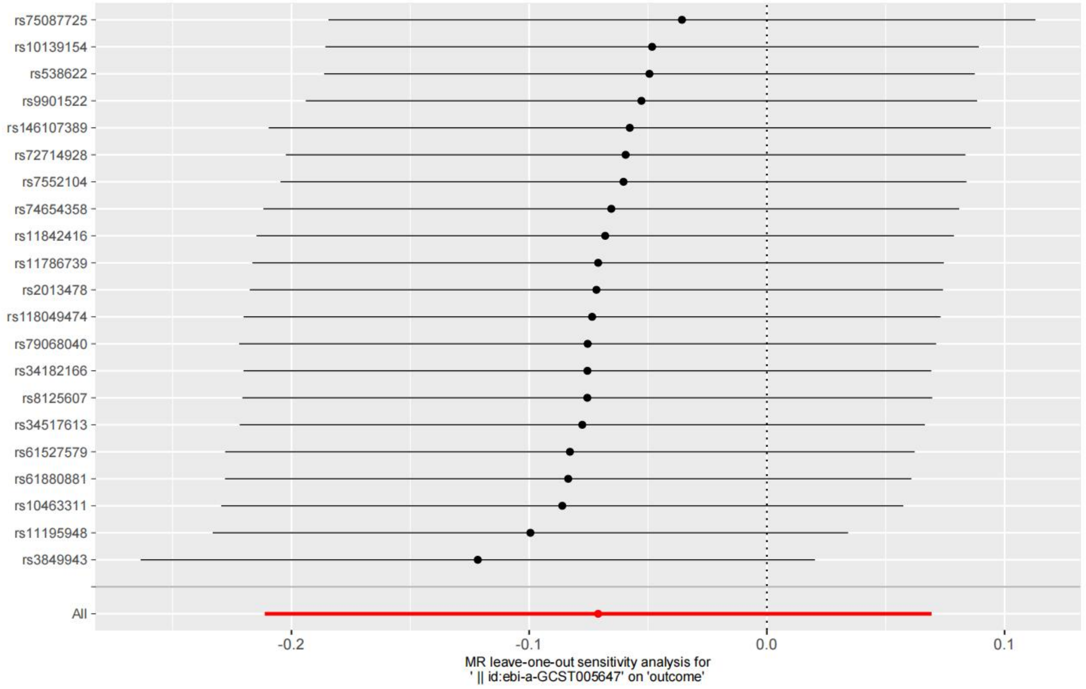

C. MR leave-one-out sensitivity analysis for ALS on IL-5

eFigure 220. ALS-associated SNPs with IL-6

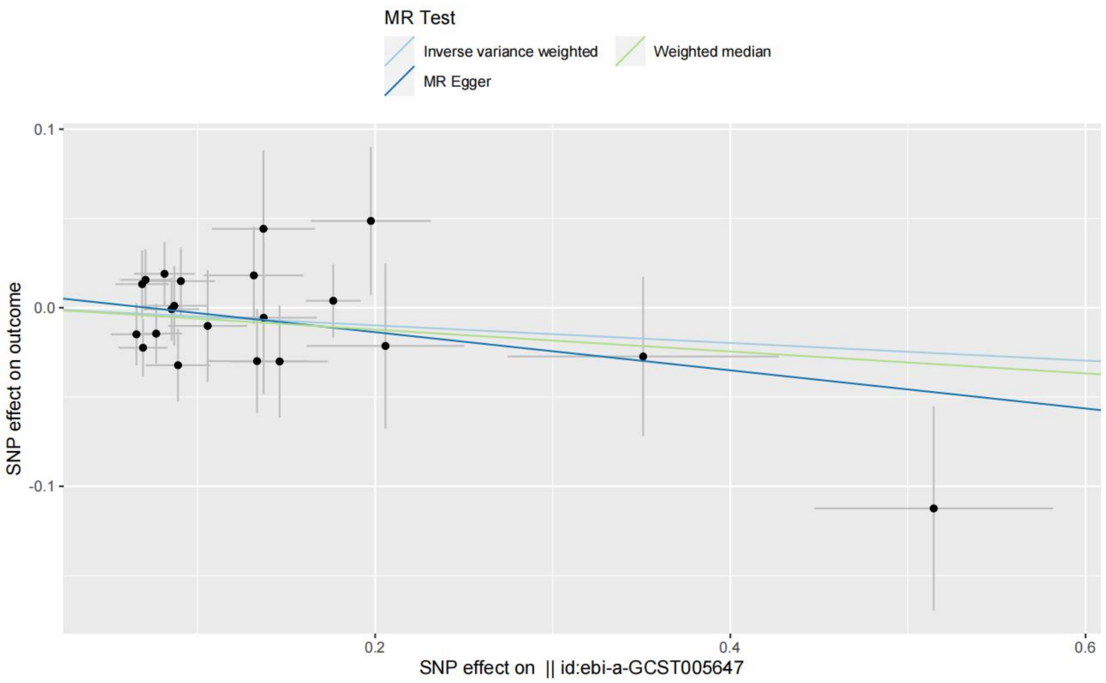

A. Scatter plot of ALS on IL-6

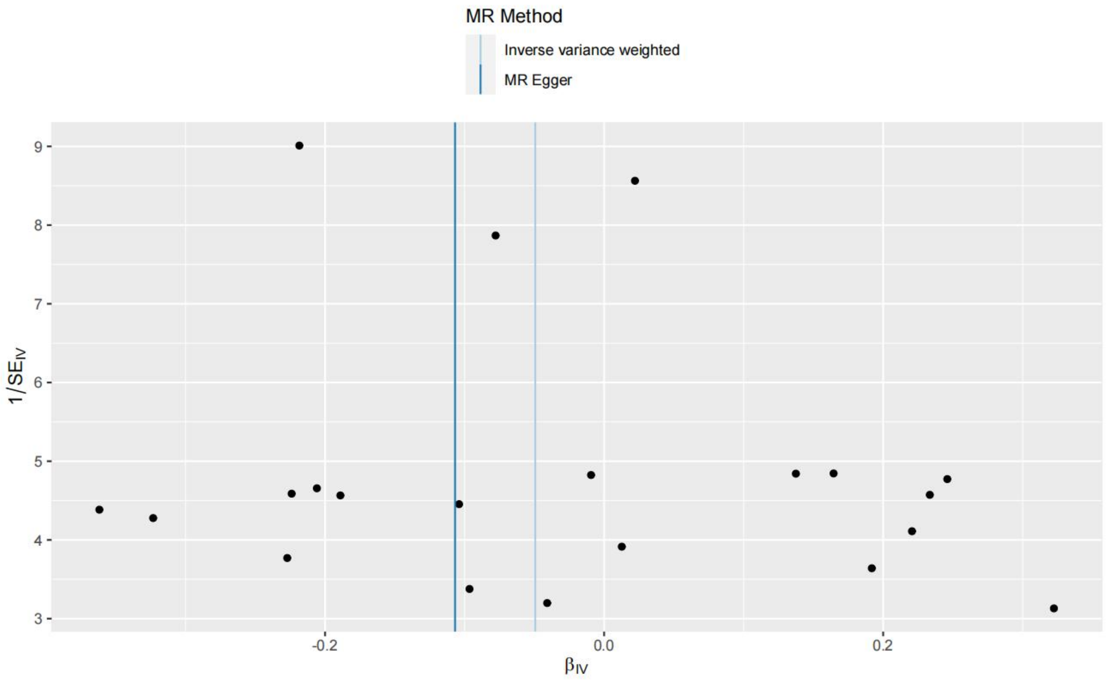

B. Funnel plot of ALS on IL-6

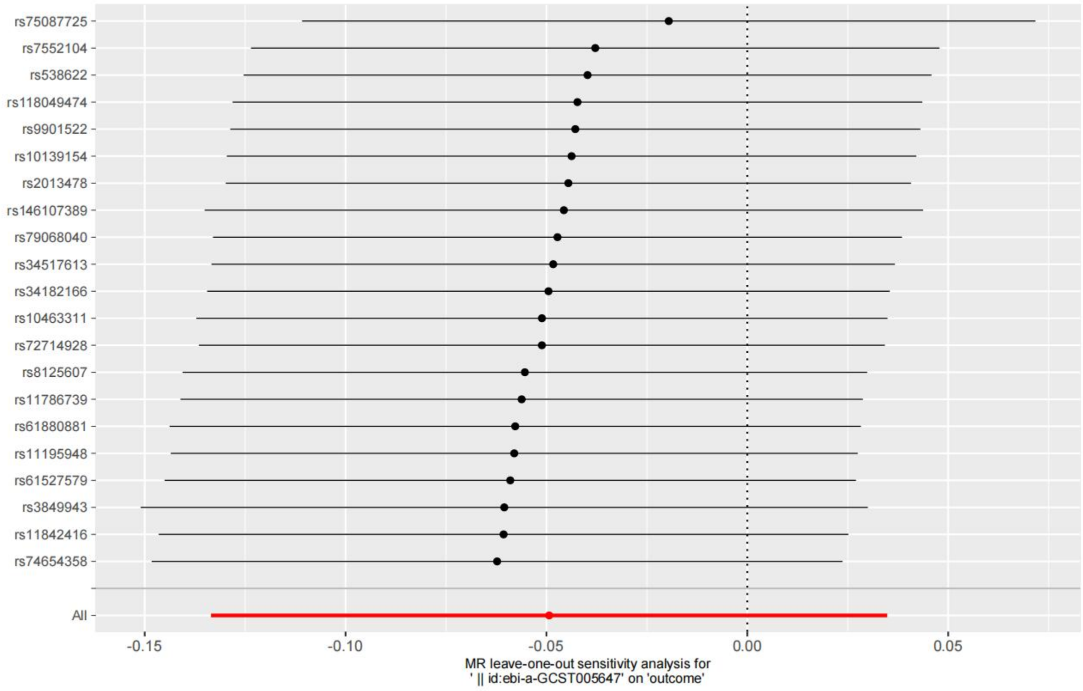

C. MR leave-one-out sensitivity analysis for ALS on IL-6

eFigure 221. ALS-associated SNPs with IL-7

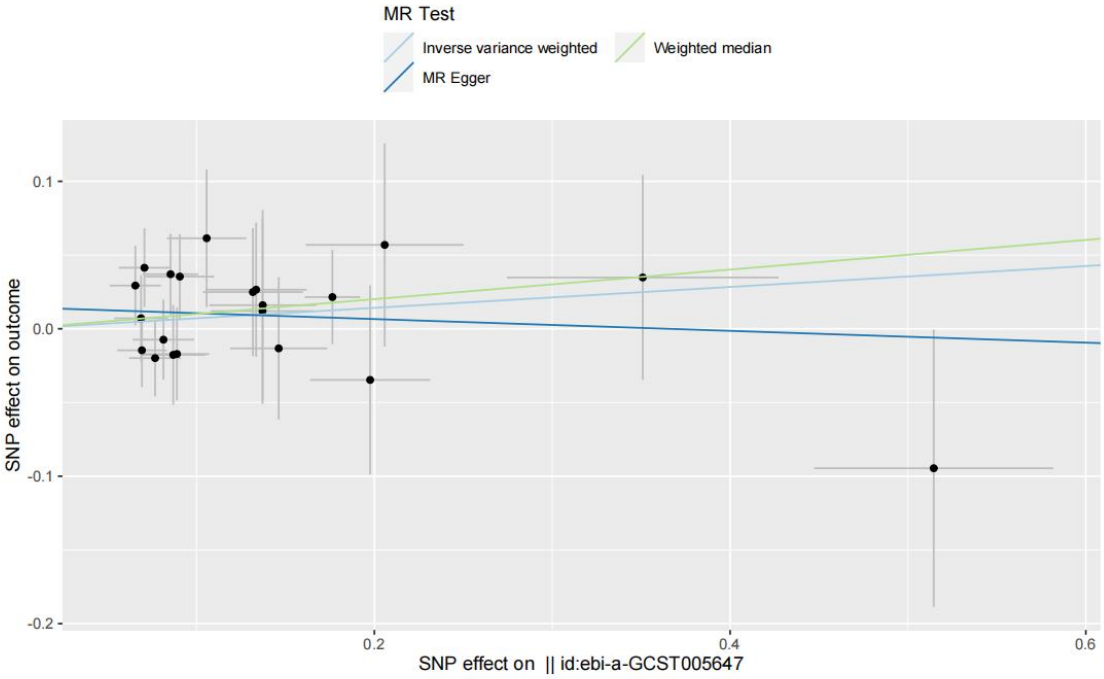

A. Scatter plot of ALS on IL-7

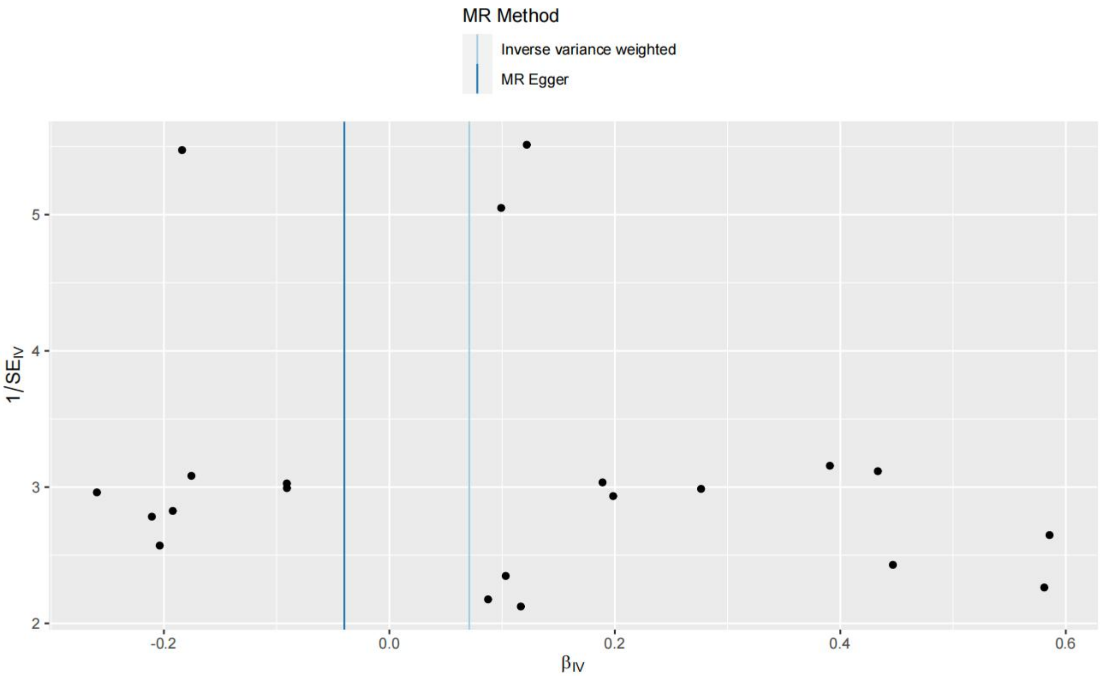

B. Funnel plot of ALS on IL-7

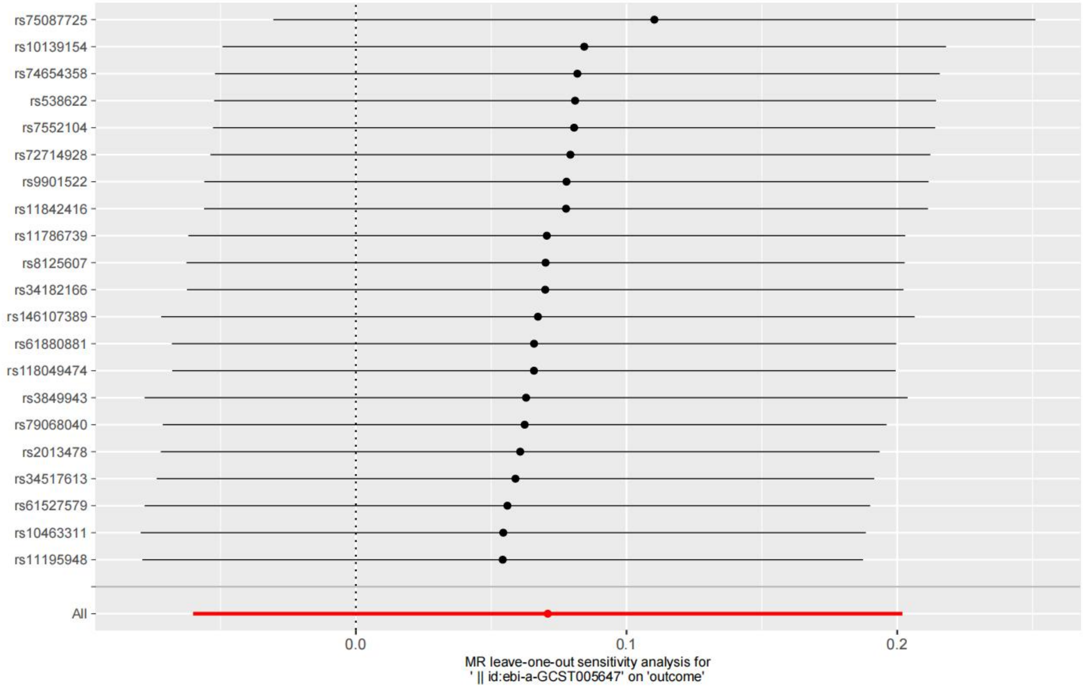

C. MR leave-one-out sensitivity analysis for ALS on IL-7

eFigure 222. ALS-associated SNPs with IL-8

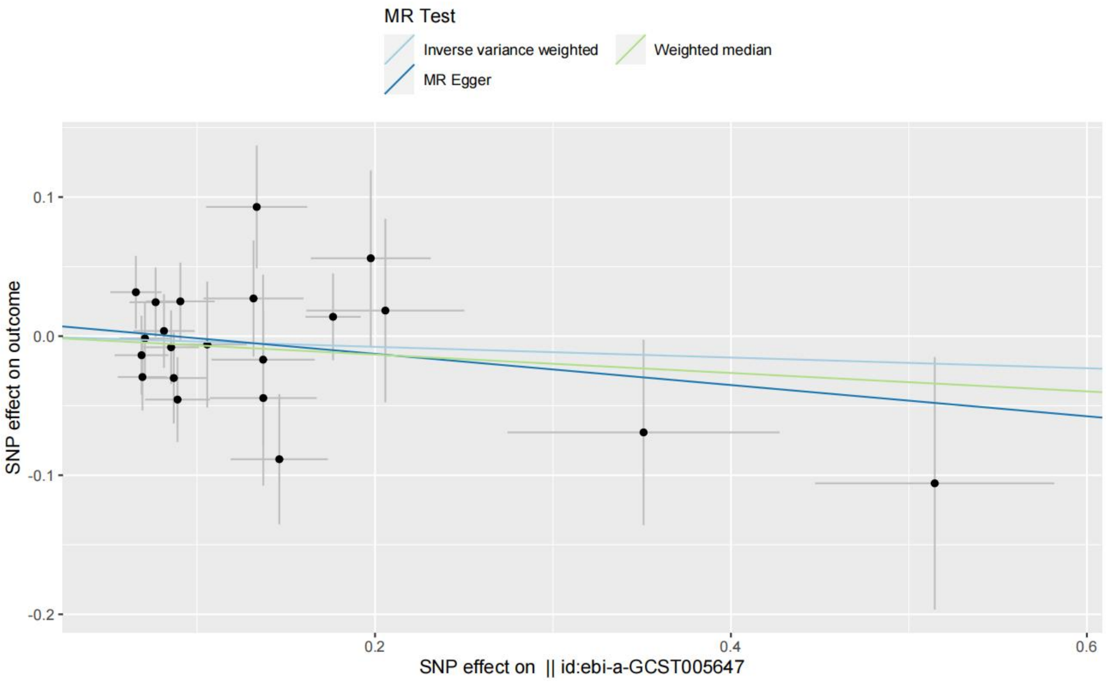

A. Scatter plot of ALS on IL-8

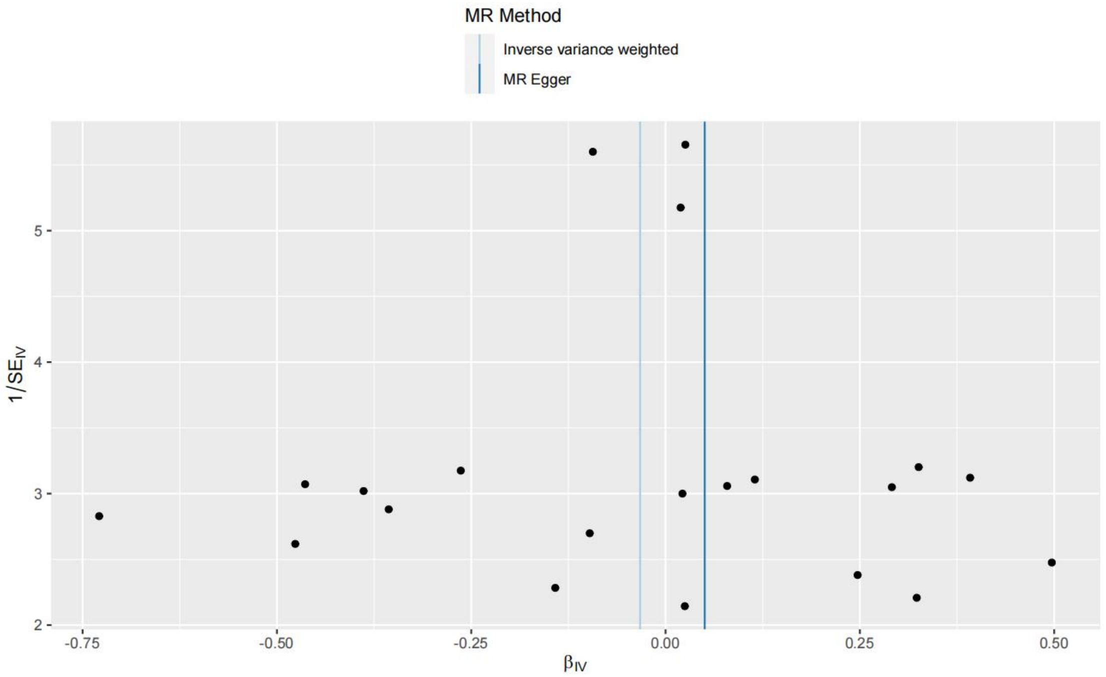

B. Funnel plot of ALS on IL-8

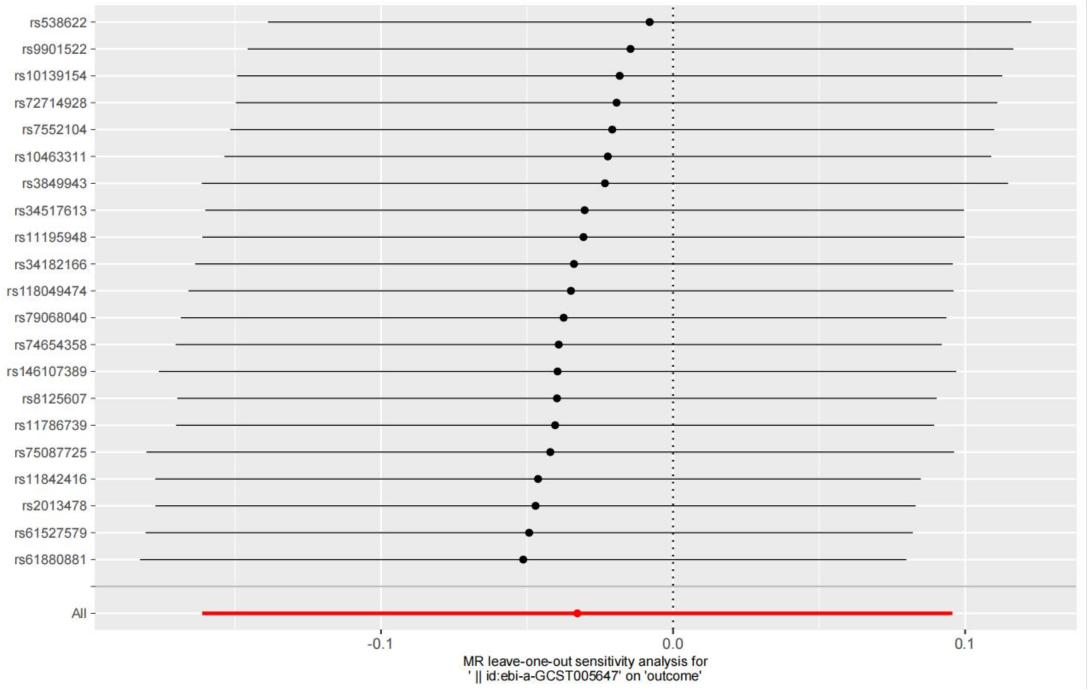

C. MR leave-one-out sensitivity analysis for ALS on IL-8

eFigure 223. ALS-associated SNPs with IL-9

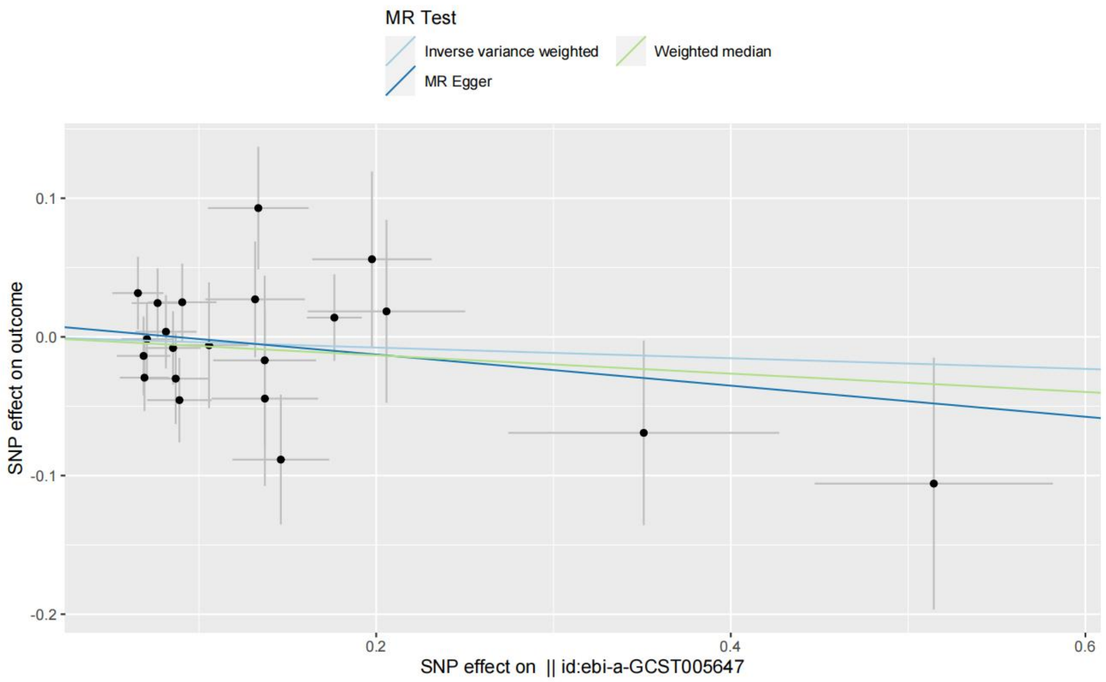

A. Scatter plot of ALS on IL-9

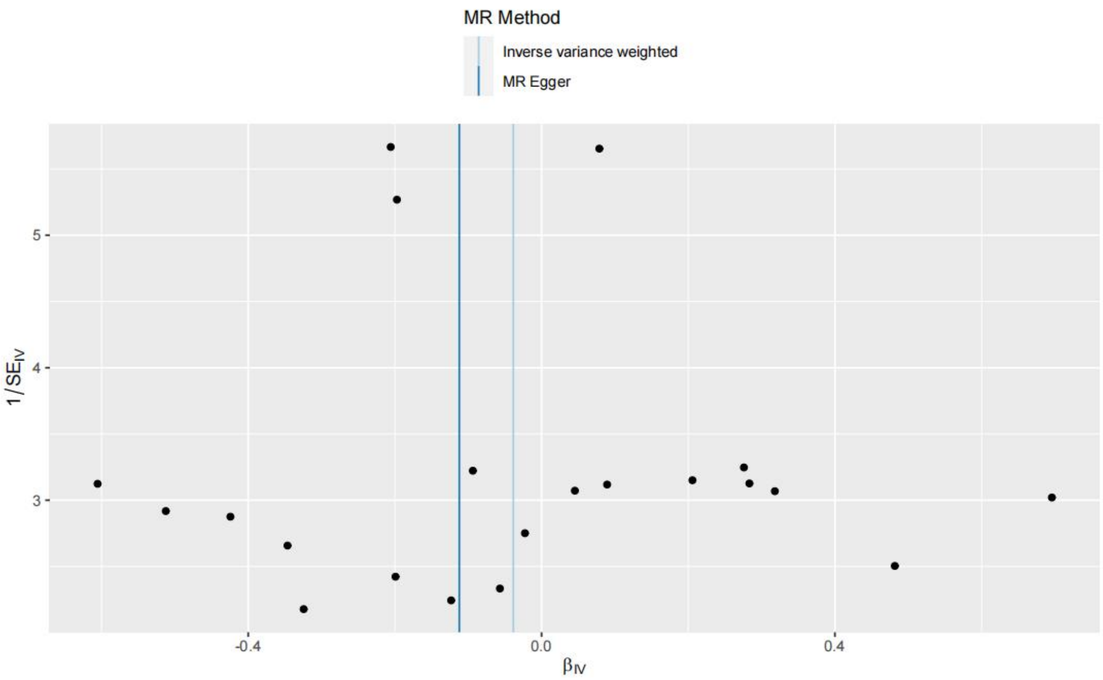

B. Funnel plot of ALS on IL-9

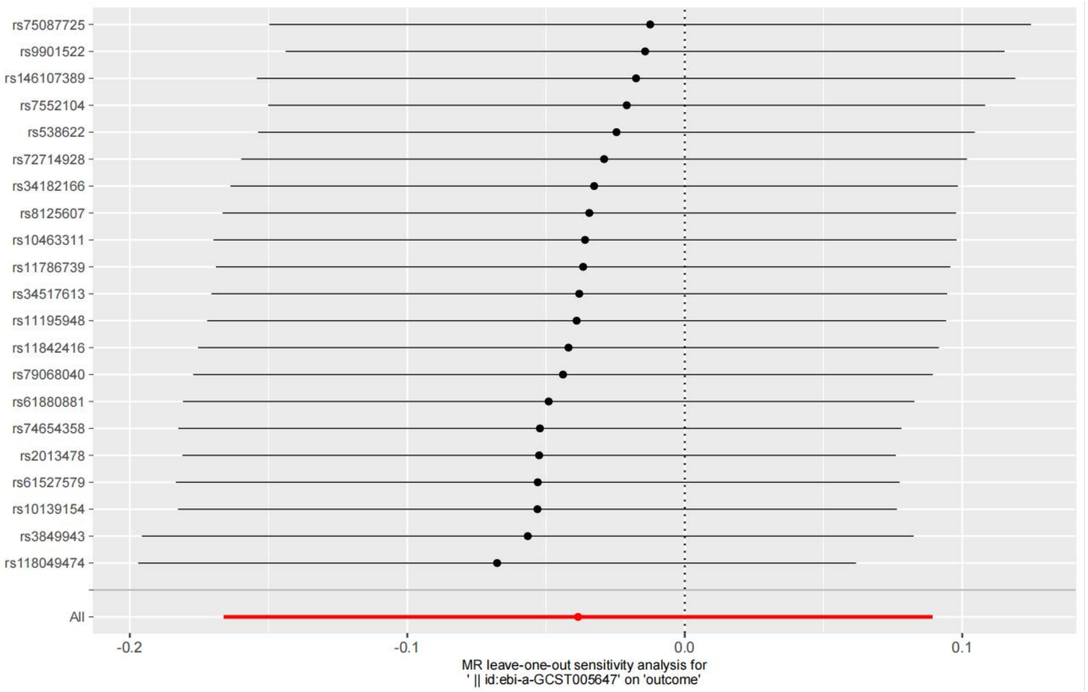

C. MR leave-one-out sensitivity analysis for ALS on IL-9

eFigure 224. ALS-associated SNPs with IL-10

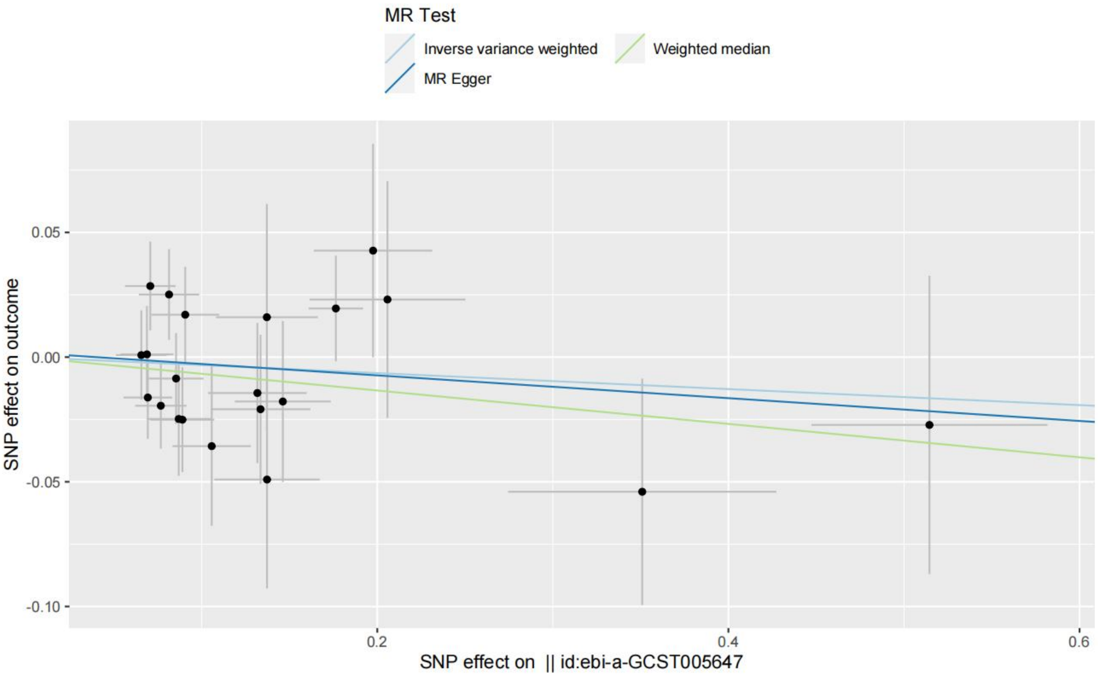

A. Scatter plot of ALS on IL-10

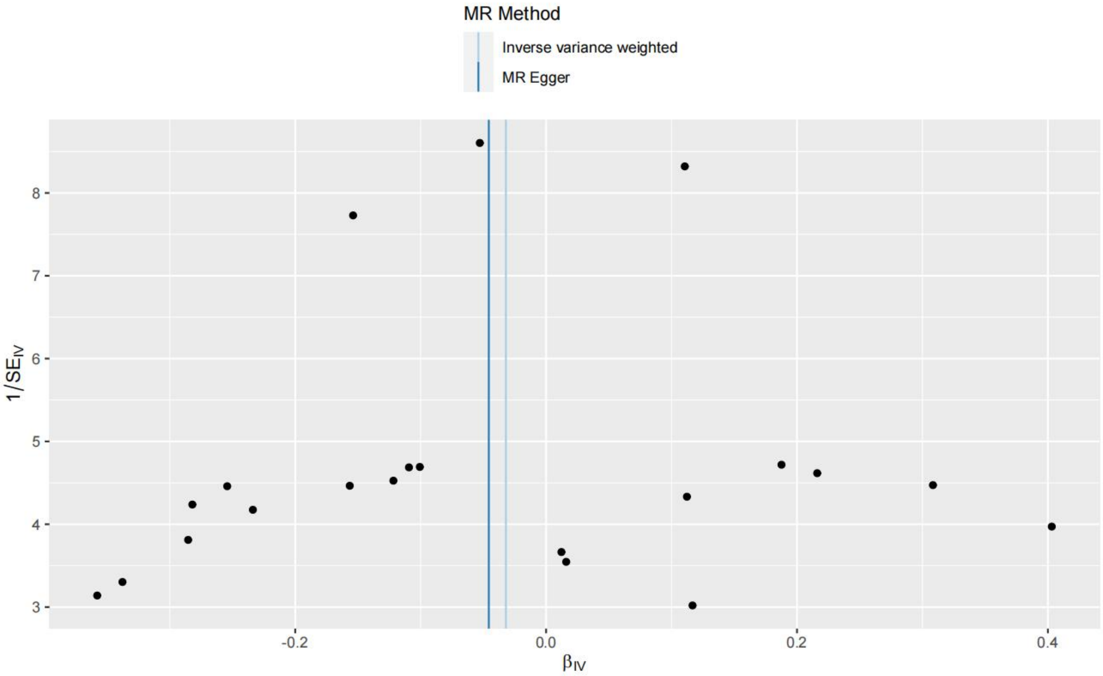

B. Funnel plot of ALS on IL-10

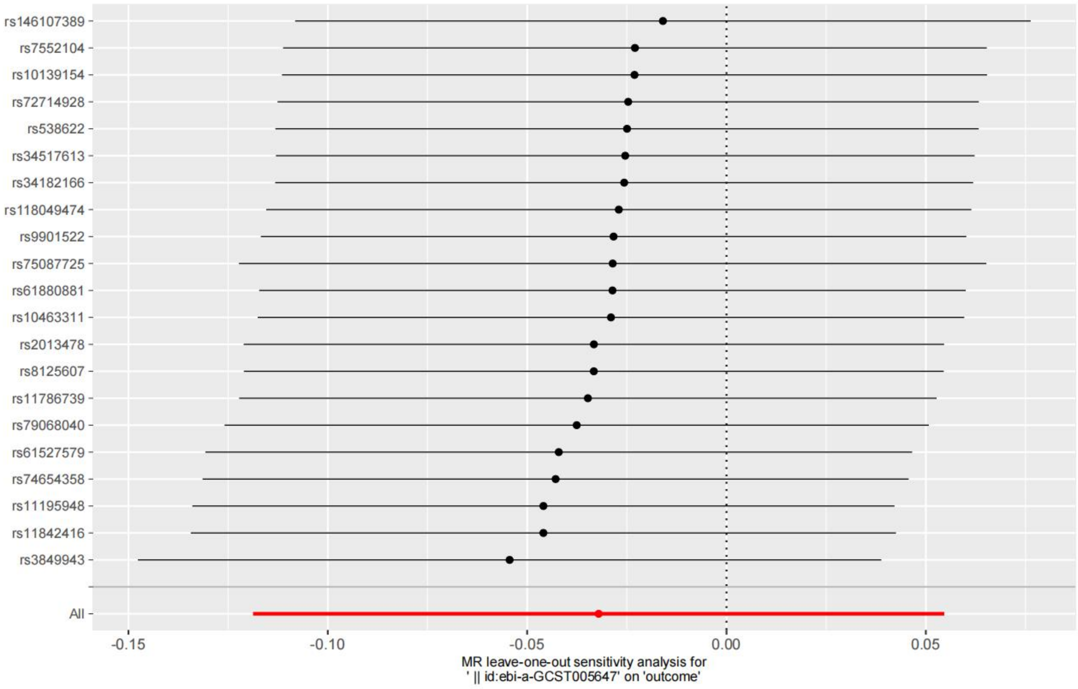

C. MR leave-one-out sensitivity analysis for ALS on IL-10

eFigure 225. ALS-associated SNPs with IL-12

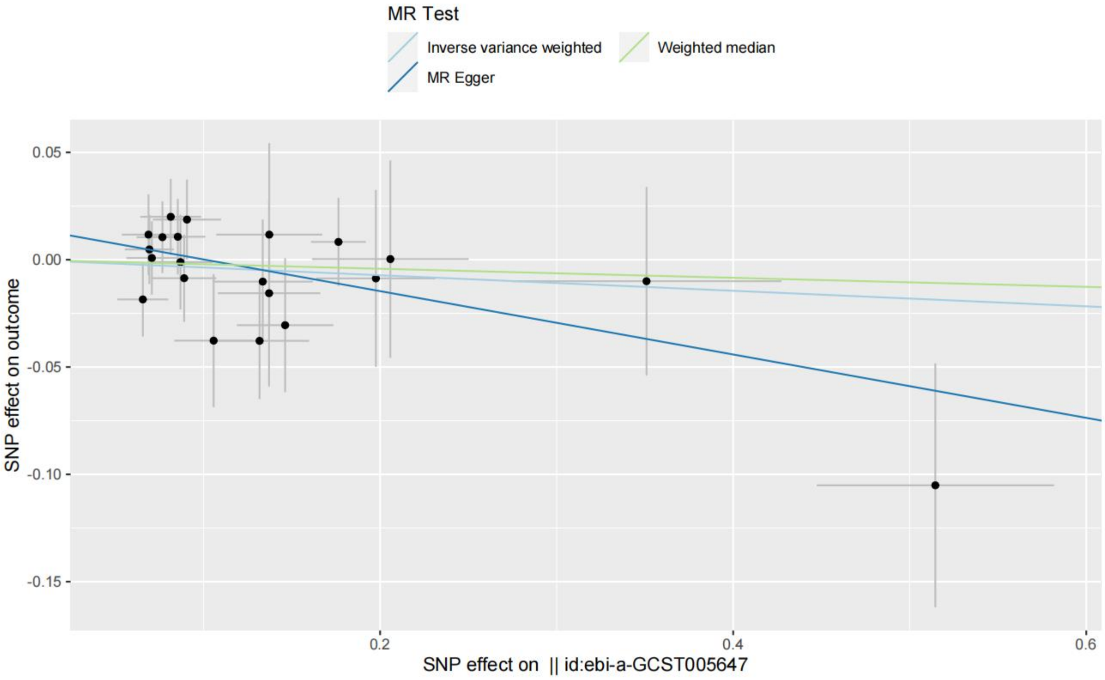

A. Scatter plot of ALS on IL-12

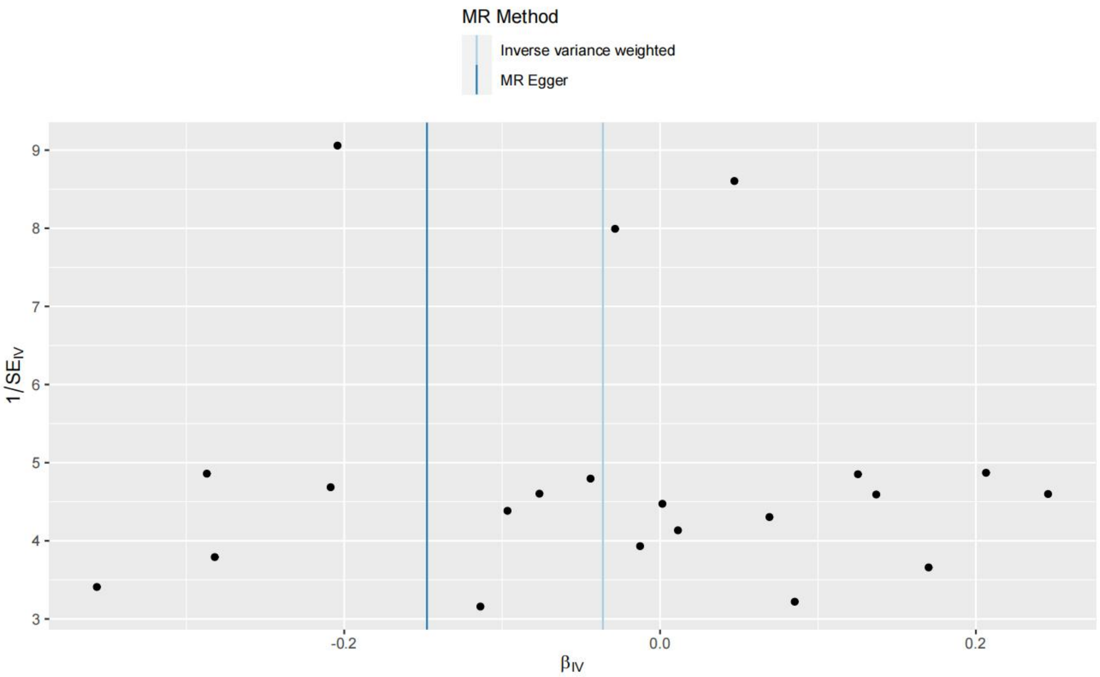

B. Funnel plot of ALS on IL-12

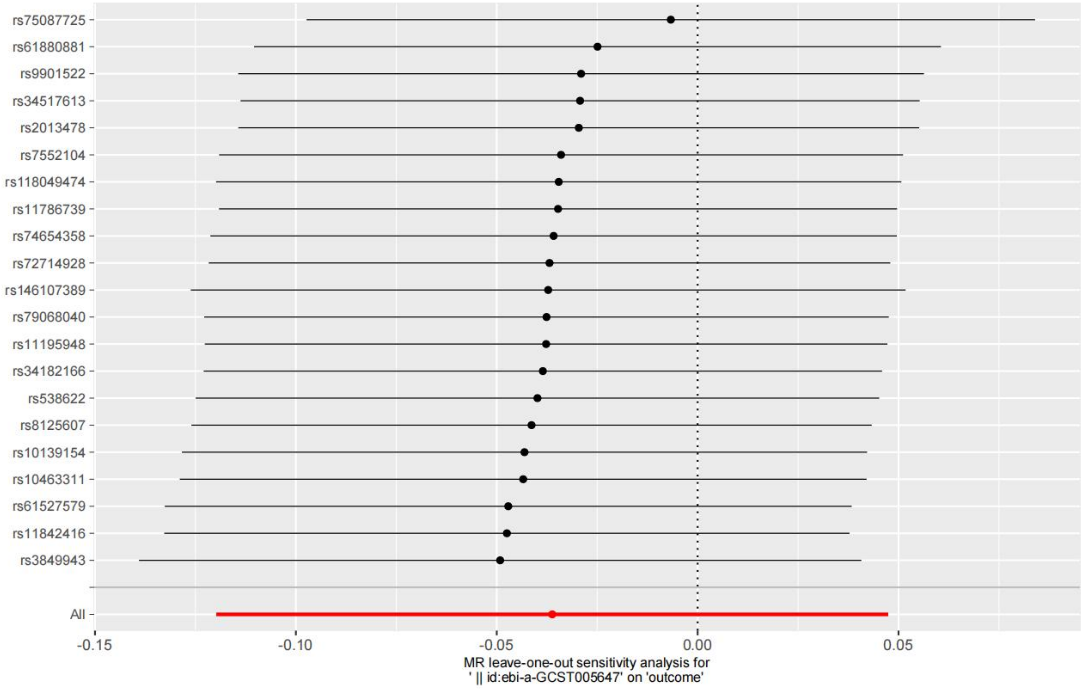

C. MR leave-one-out sensitivity analysis for ALS on IL-12

eFigure 226. ALS-associated SNPs with IL-13

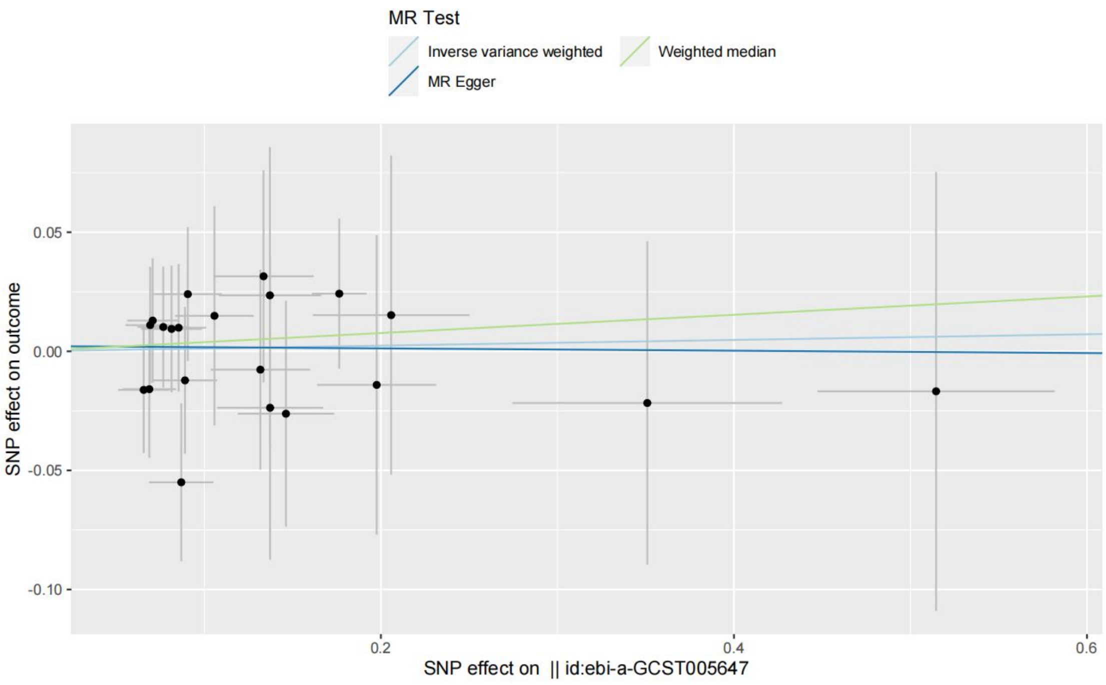

A. Scatter plot of ALS on IL-13

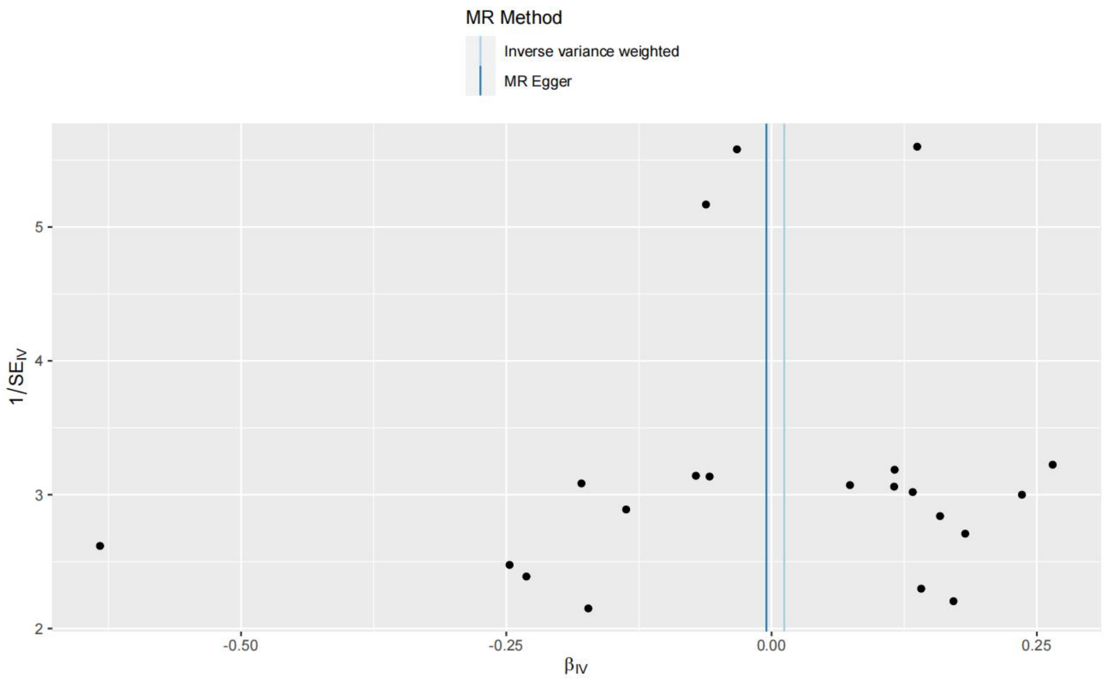

B. Funnel plot of ALS on IL-13

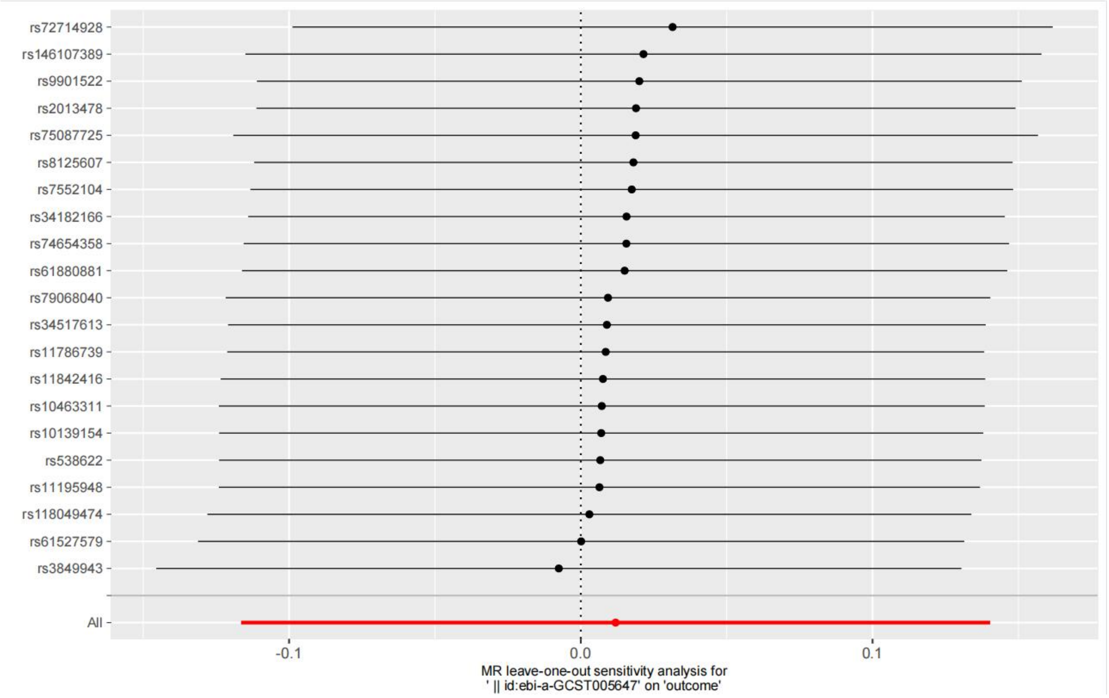

C. MR leave-one-out sensitivity analysis for ALS on IL-13

eFigure 227. ALS-associated SNPs with IL-16

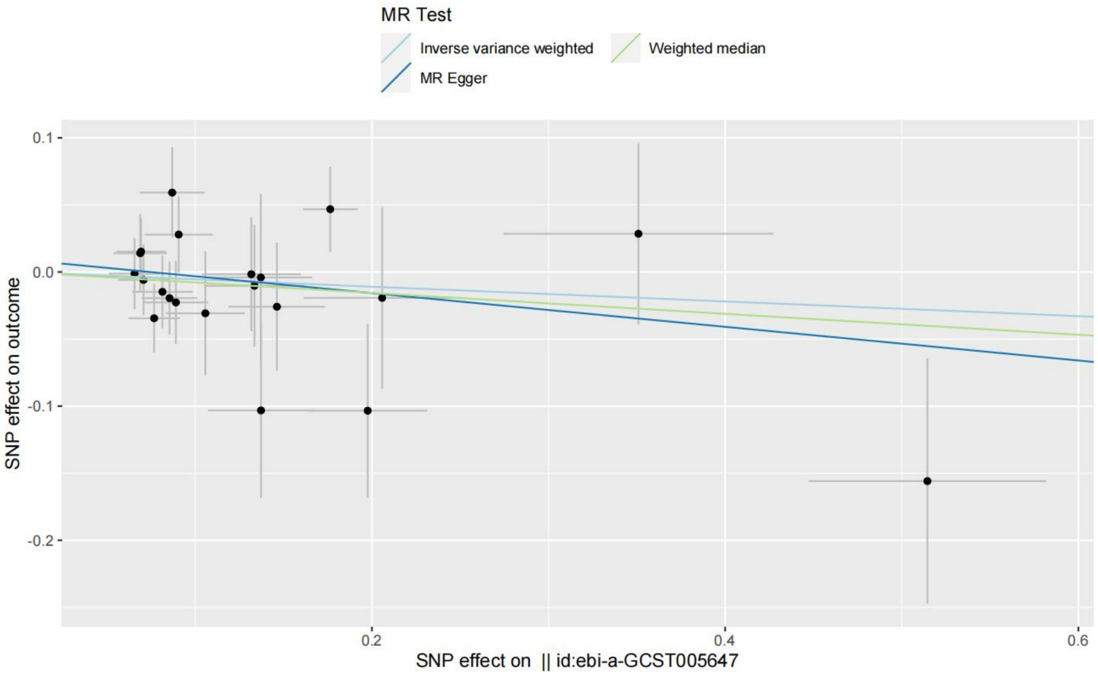

A. Scatter plot of ALS on IL-16

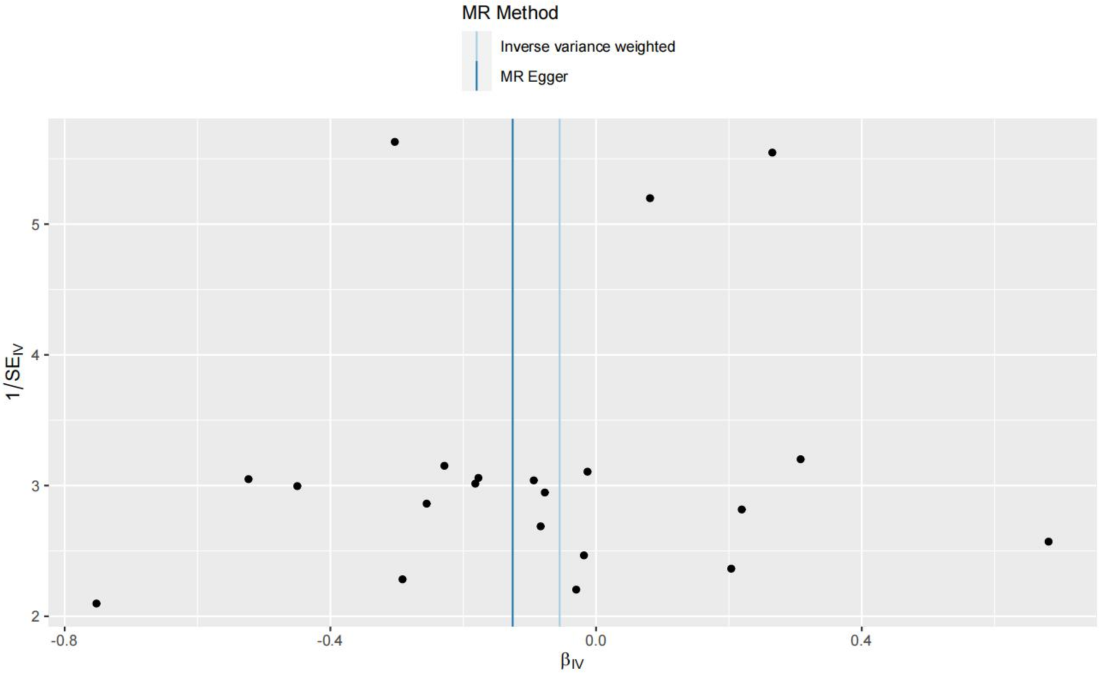

B. Funnel plot of ALS on IL-16

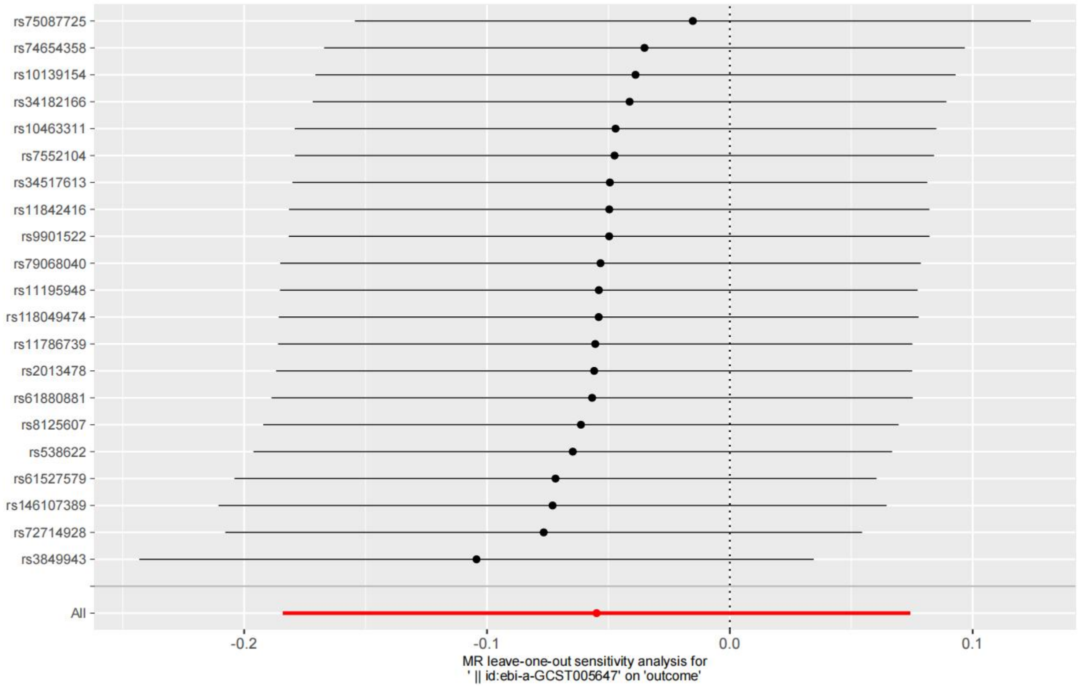

C. MR leave-one-out sensitivity analysis for ALS on IL-16

eFigure 228. ALS-associated SNPs with IL-17

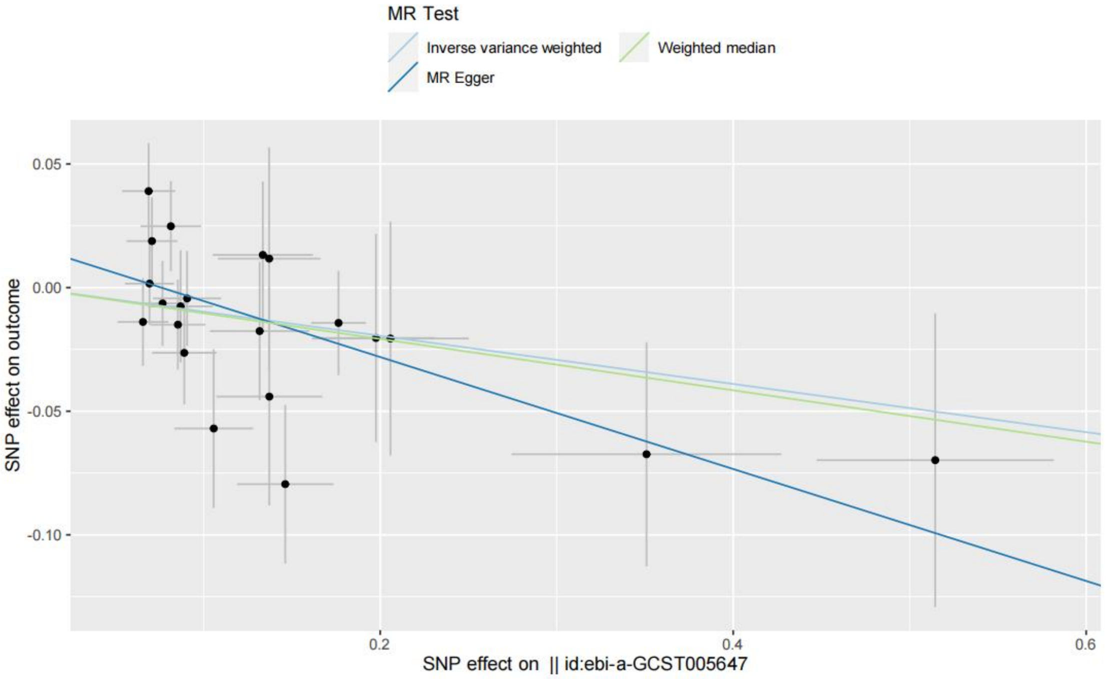

A. Scatter plot of ALS on IL-17

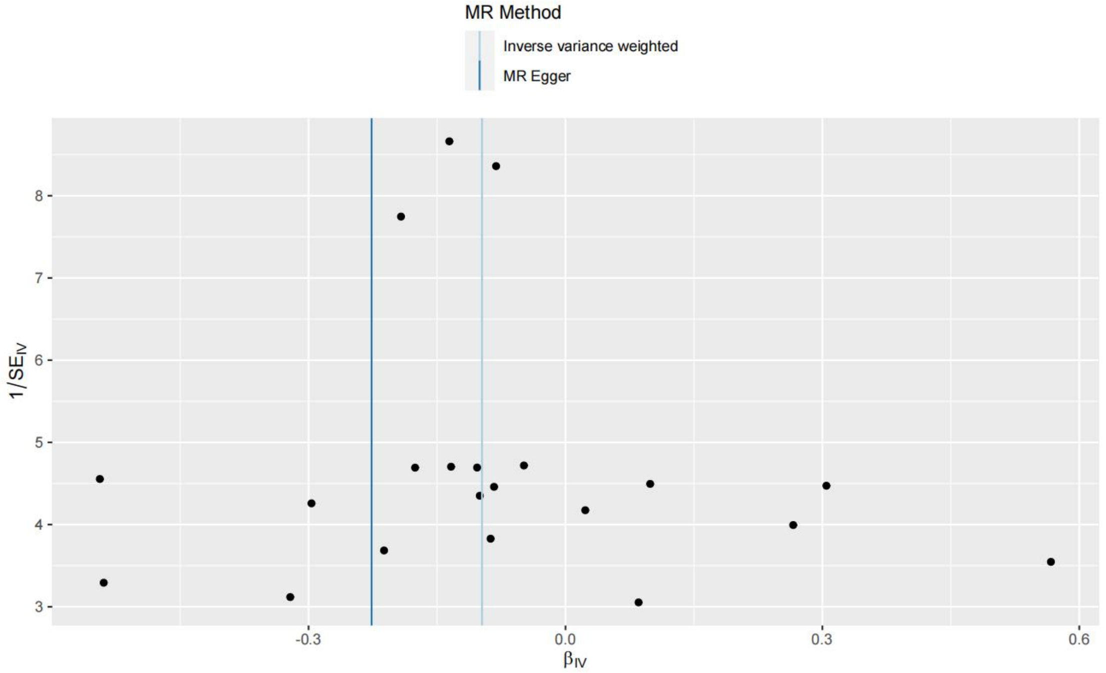

B. Funnel plot of ALS on IL-17

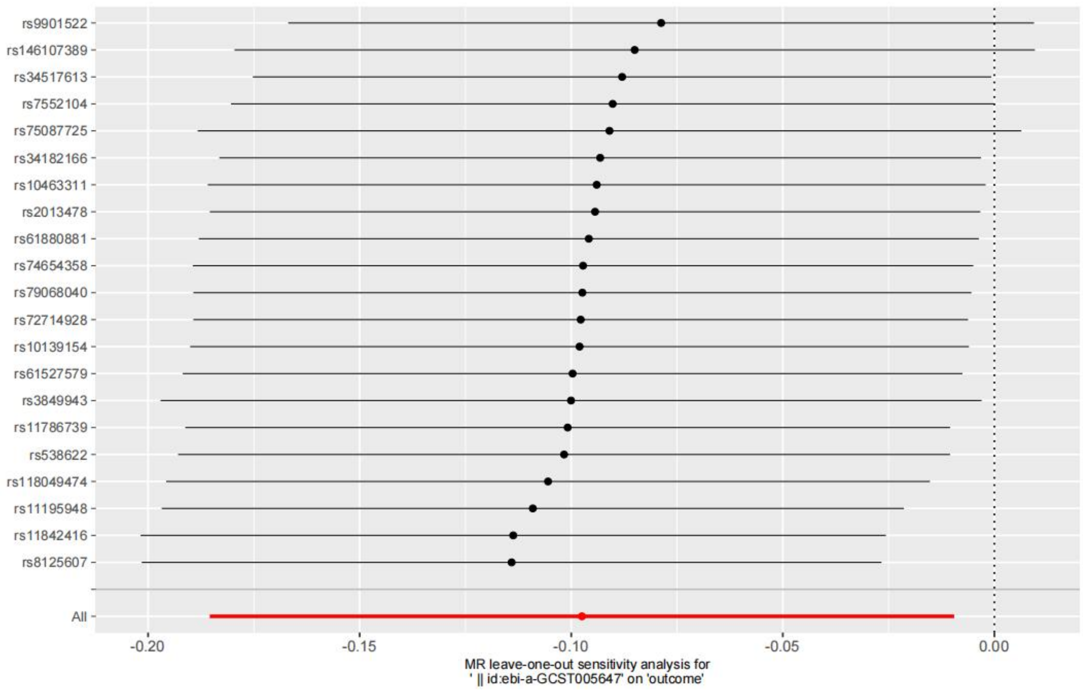

C. MR leave-one-out sensitivity analysis for ALS on IL-17

eFigure 229. ALS-associated SNPs with IL-18

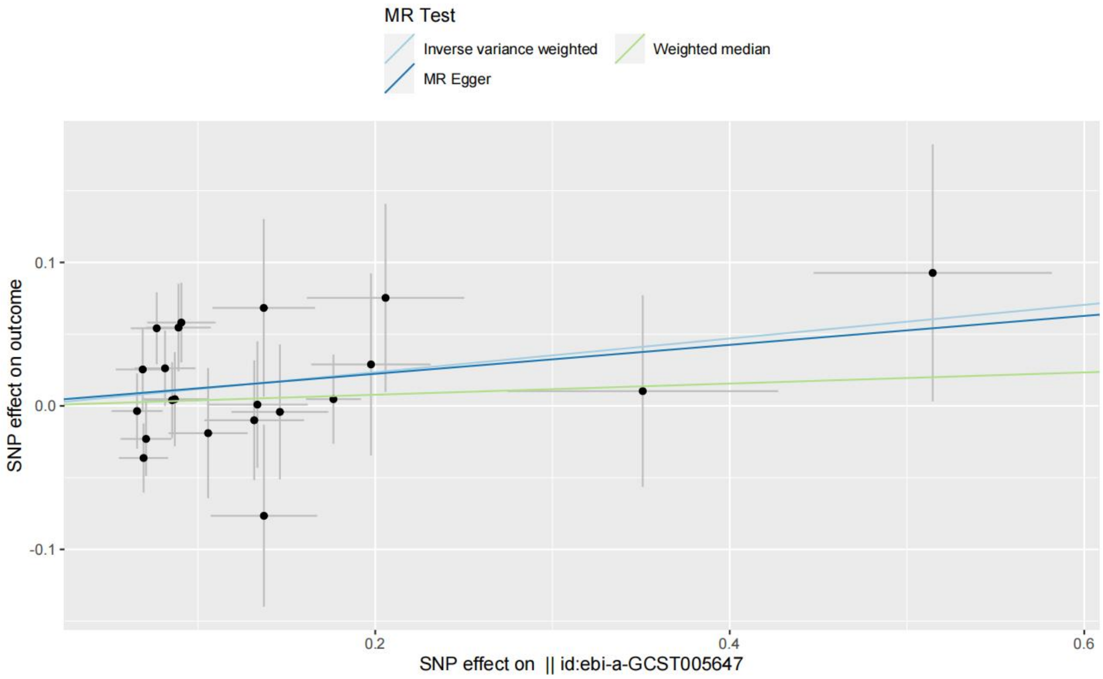

A. Scatter plot of ALS on IL-18

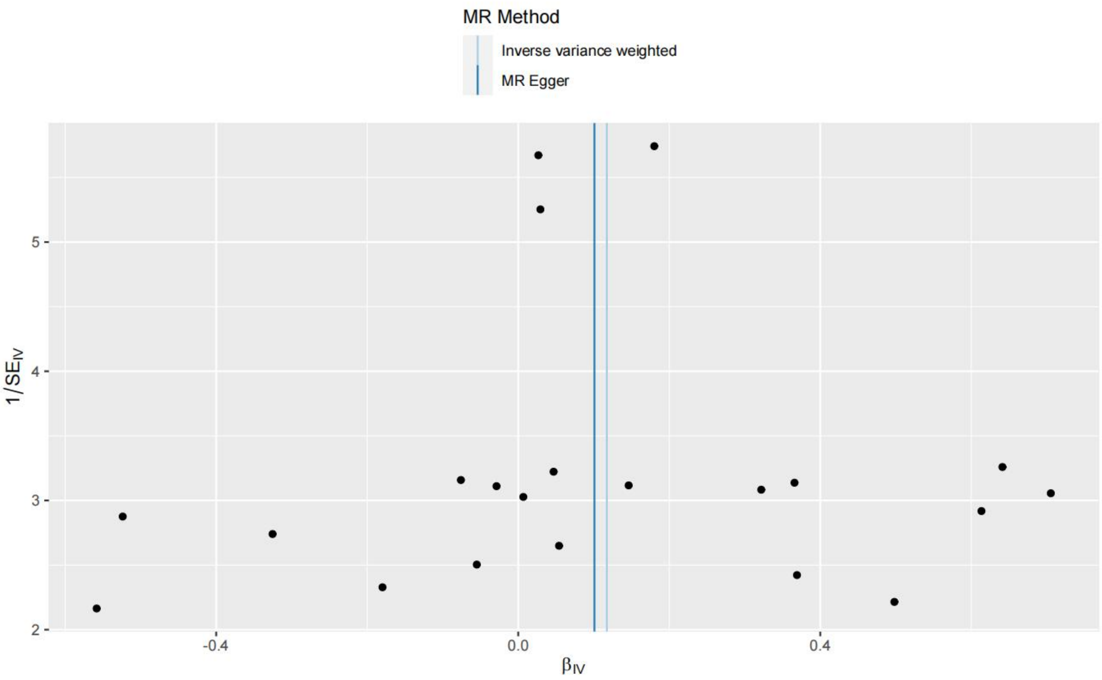

B. Funnel plot of ALS on IL-18

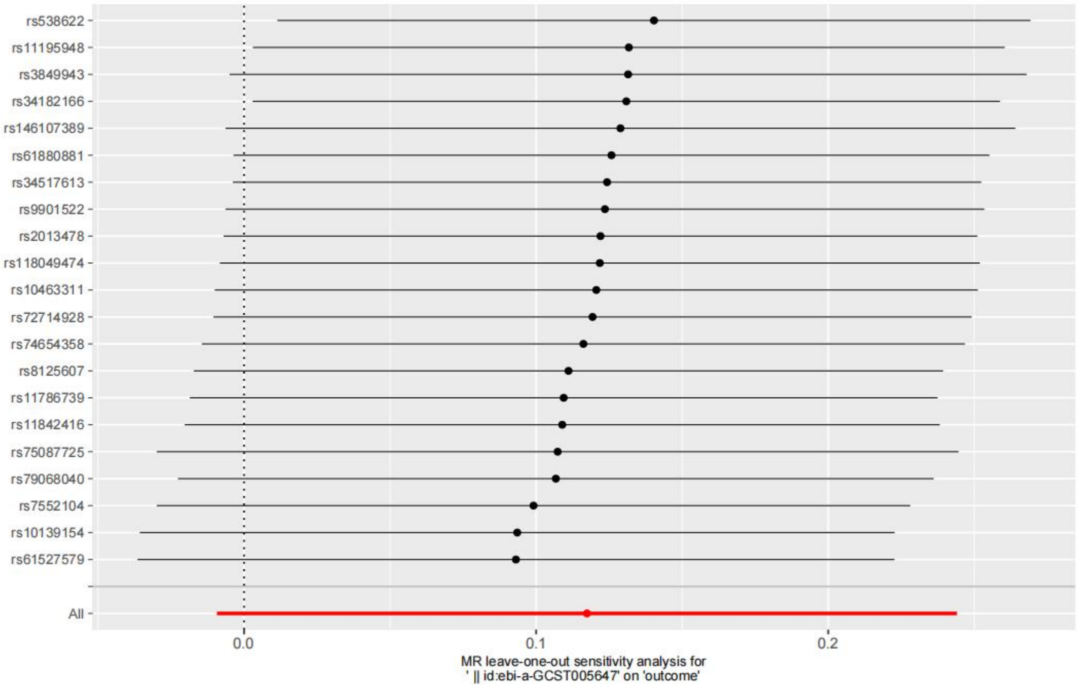

C. MR leave-one-out sensitivity analysis for ALS on IL-18

eFigure 230. ALS-associated SNPs with IP-10

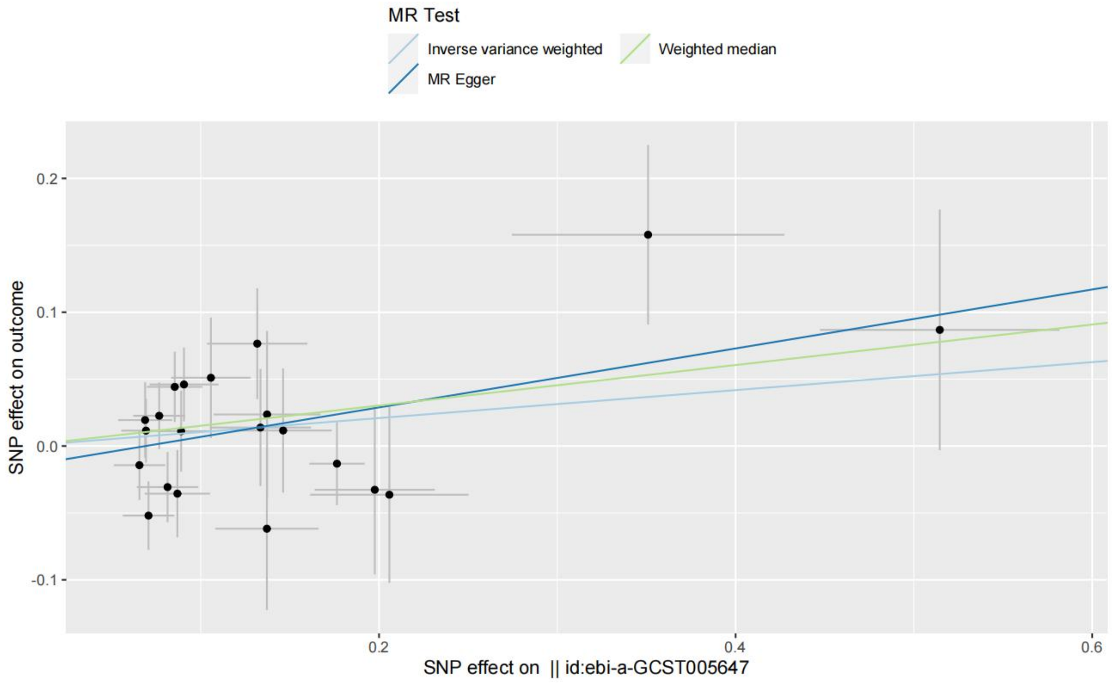

A. Scatter plot of ALS on IP-10

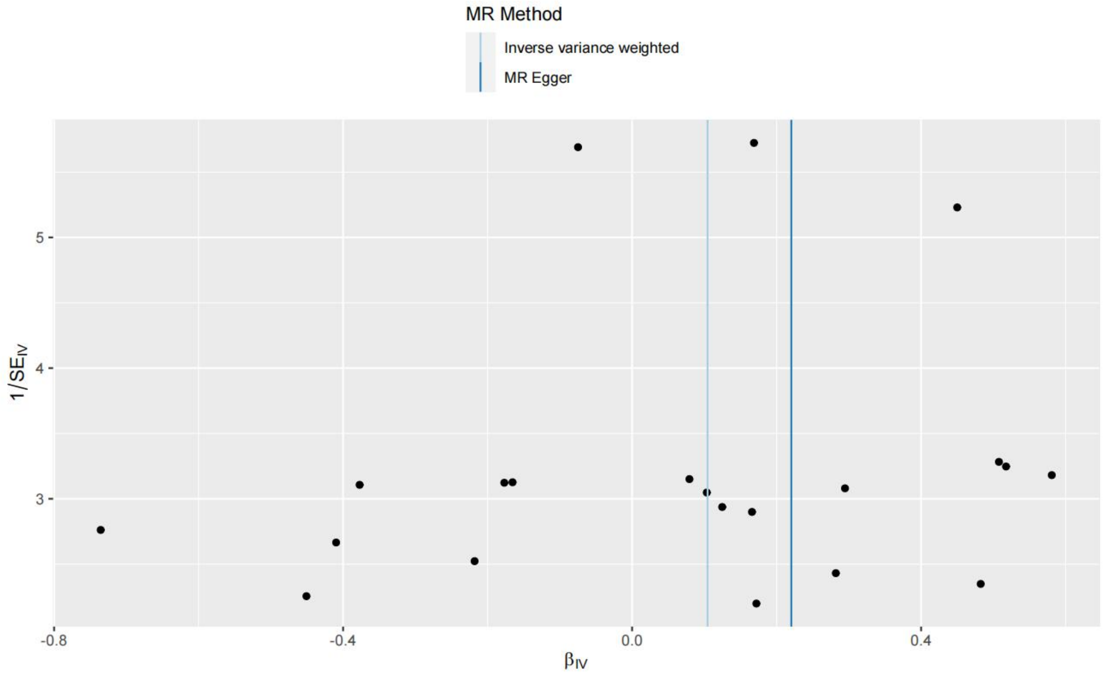

B. Funnel plot of ALS on IP-10

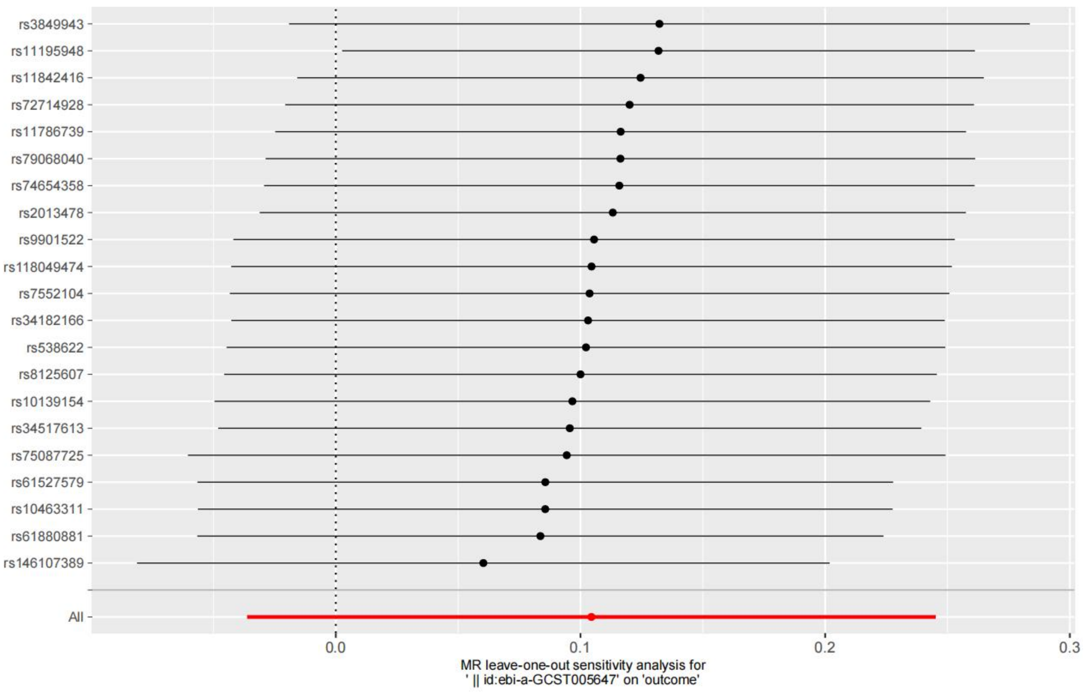

C. MR leave-one-out sensitivity analysis for ALS on IP-10

eFigure 231. ALS-associated SNPs with M-CSF

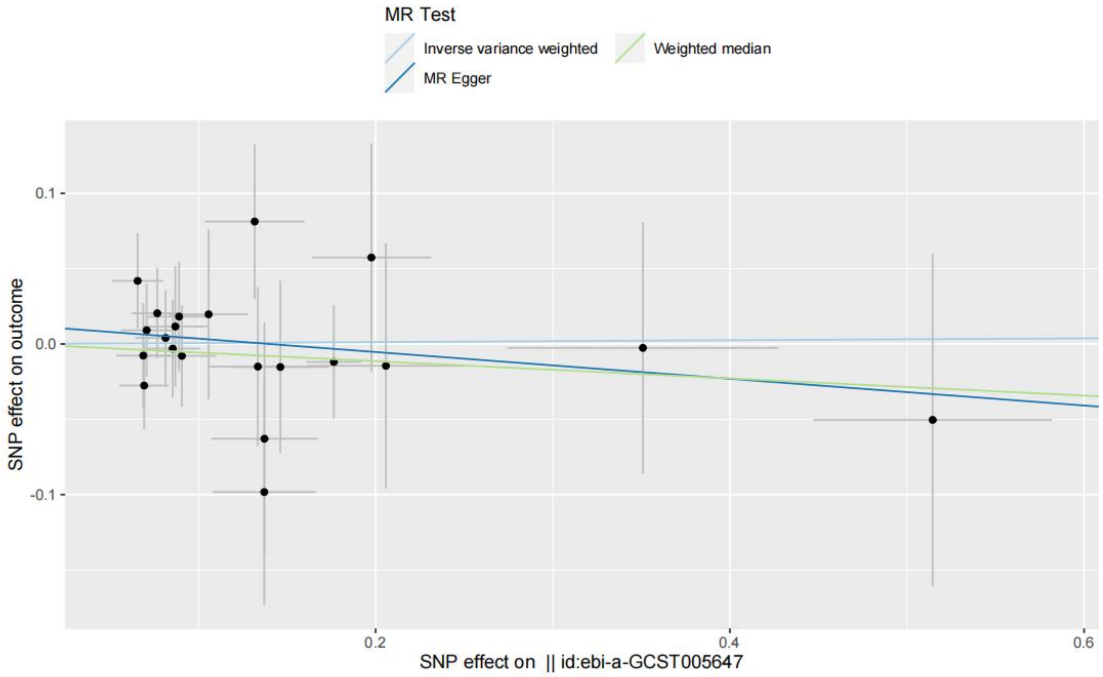

A. Scatter plot of ALS on M-CSF

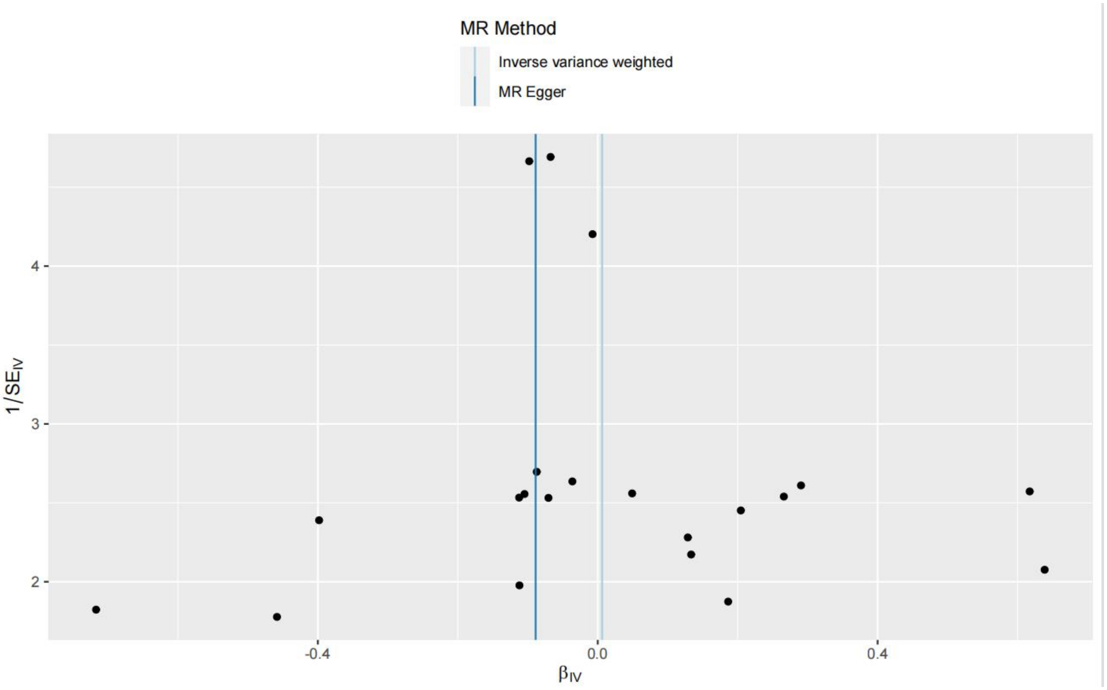

B. Funnel plot of ALS on M-CSF

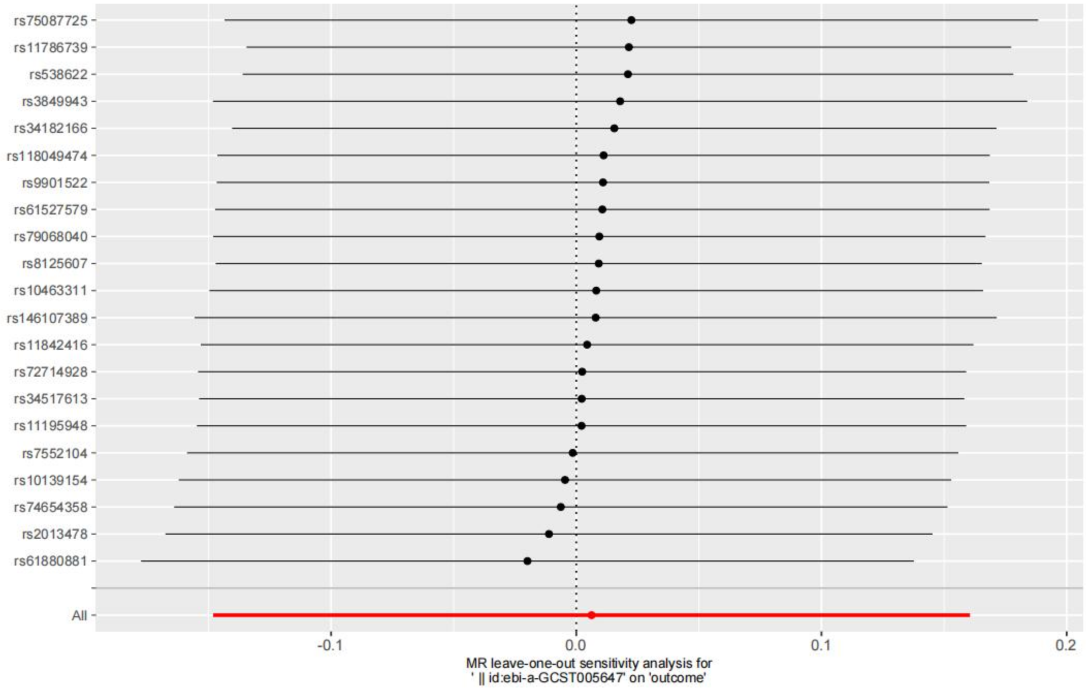

C. MR leave-one-out sensitivity analysis for ALS on M-CSF

eFigure 232. ALS-associated SNPs with MCP-1-MCAF

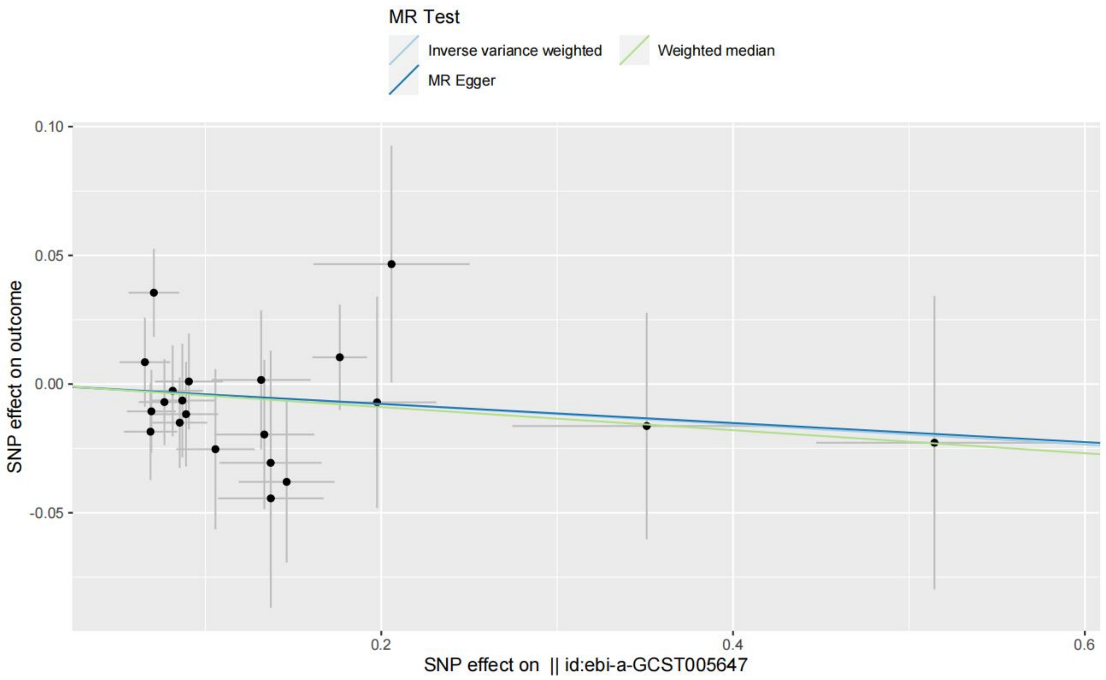

A. Scatter plot of ALS on MCP-1-MCAF

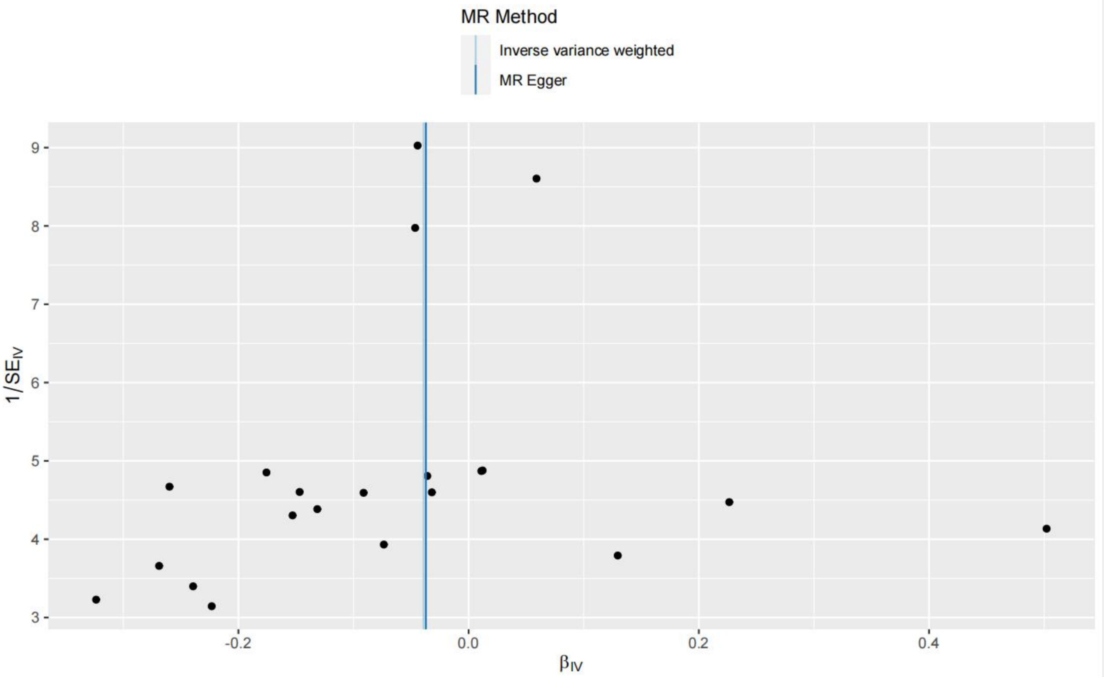

B. Funnel plot of ALS on MCP-1-MCAF

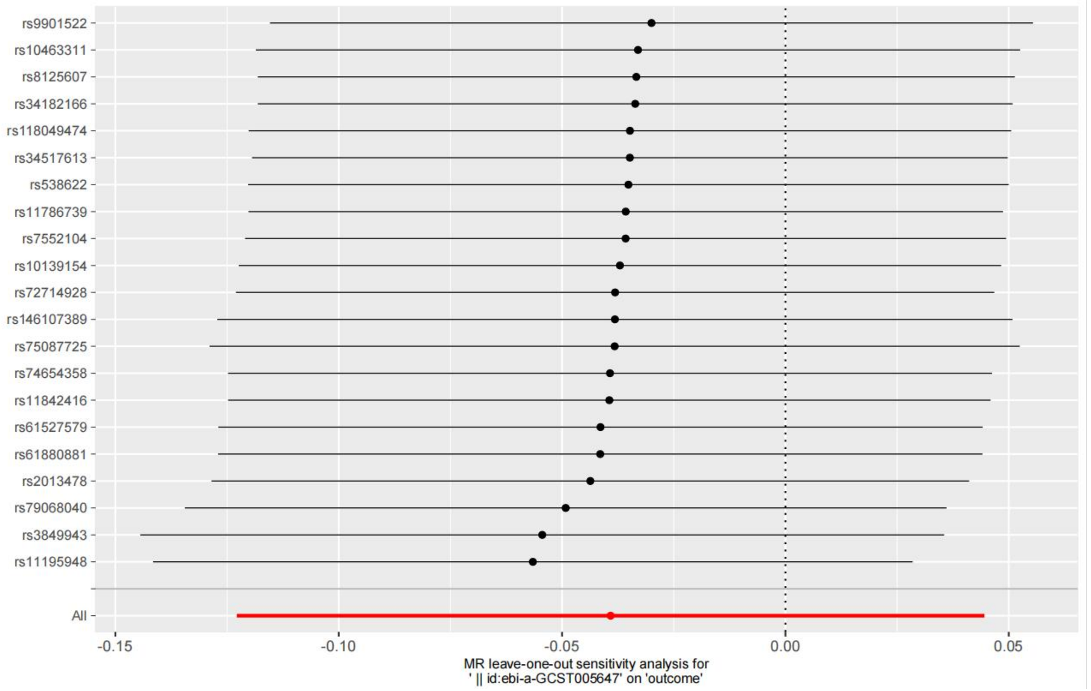

C. MR leave-one-out sensitivity analysis for ALS on MCP-1-MCAF

eFigure 233. ALS-associated SNPs with MCP-3

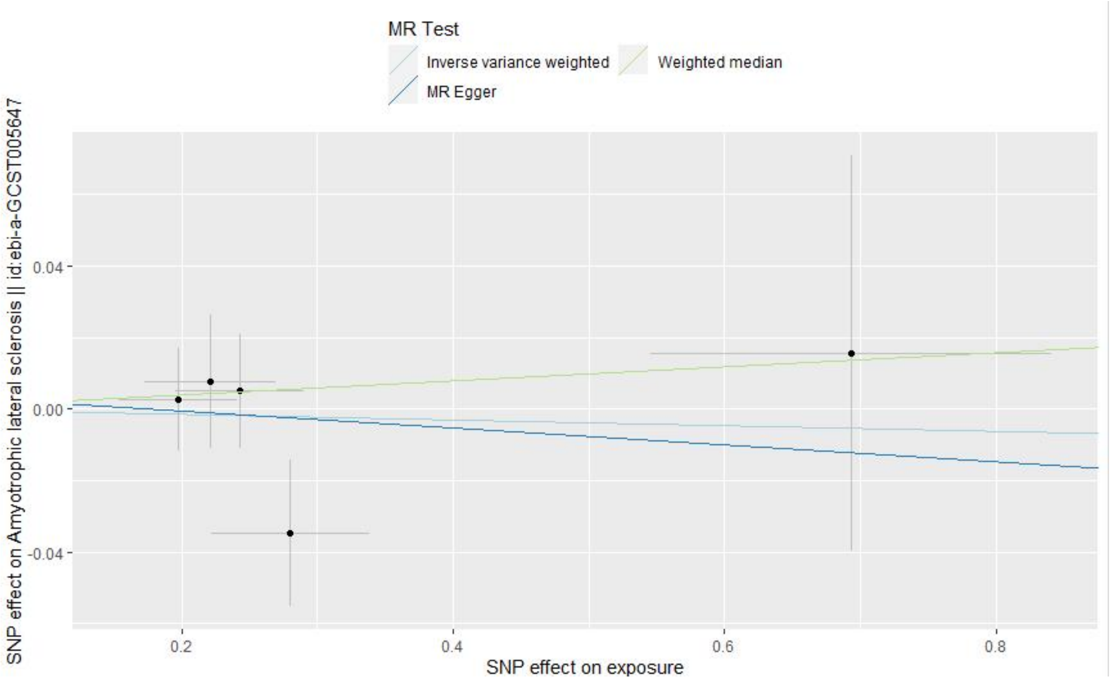

A. Scatter plot of ALS on MCP-3

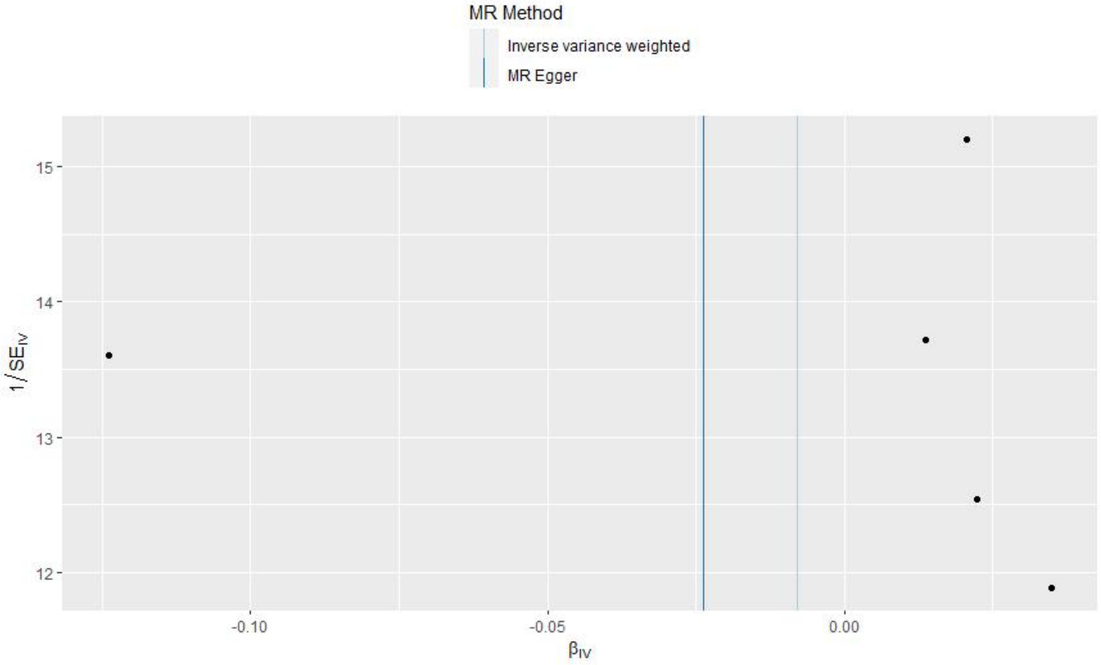

B. Funnel plot of ALS on MCP-3

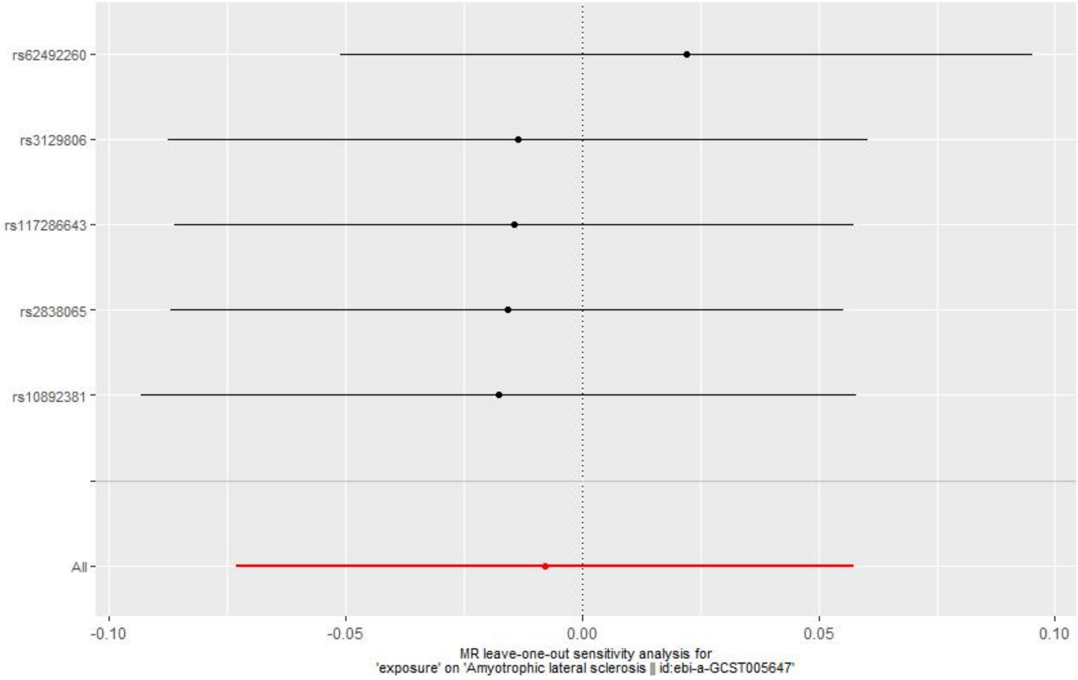

C. MR leave-one-out sensitivity analysis for ALS on MCP-3

eFigure 234. ALS-associated SNPs with MIF

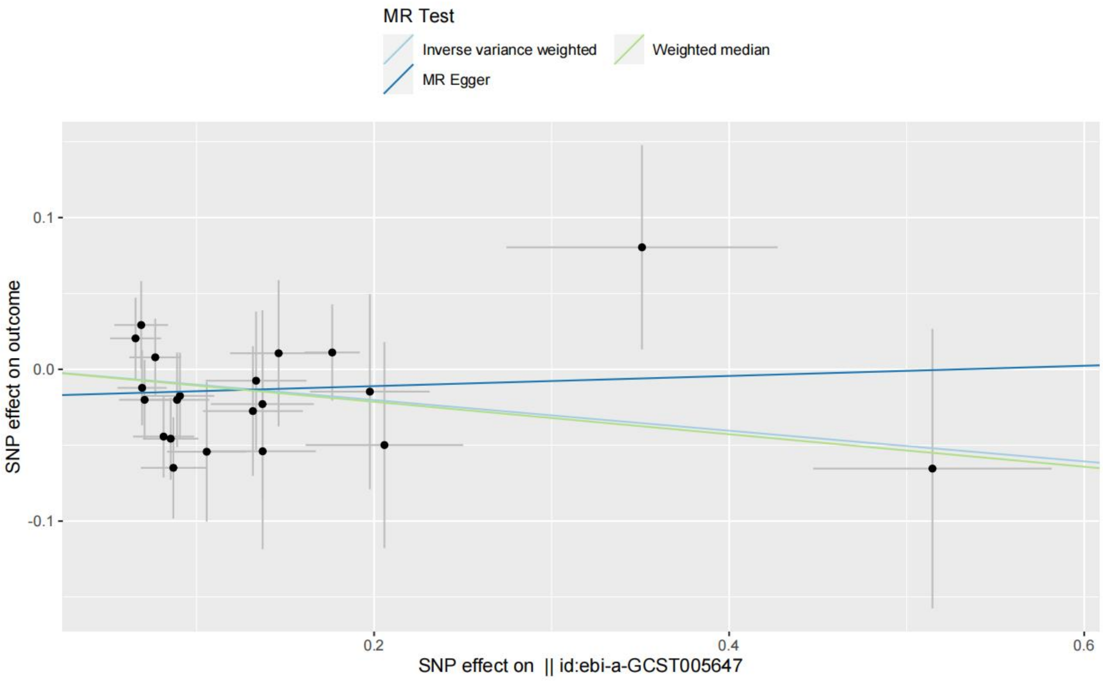

A. Scatter plot of ALS on MIF

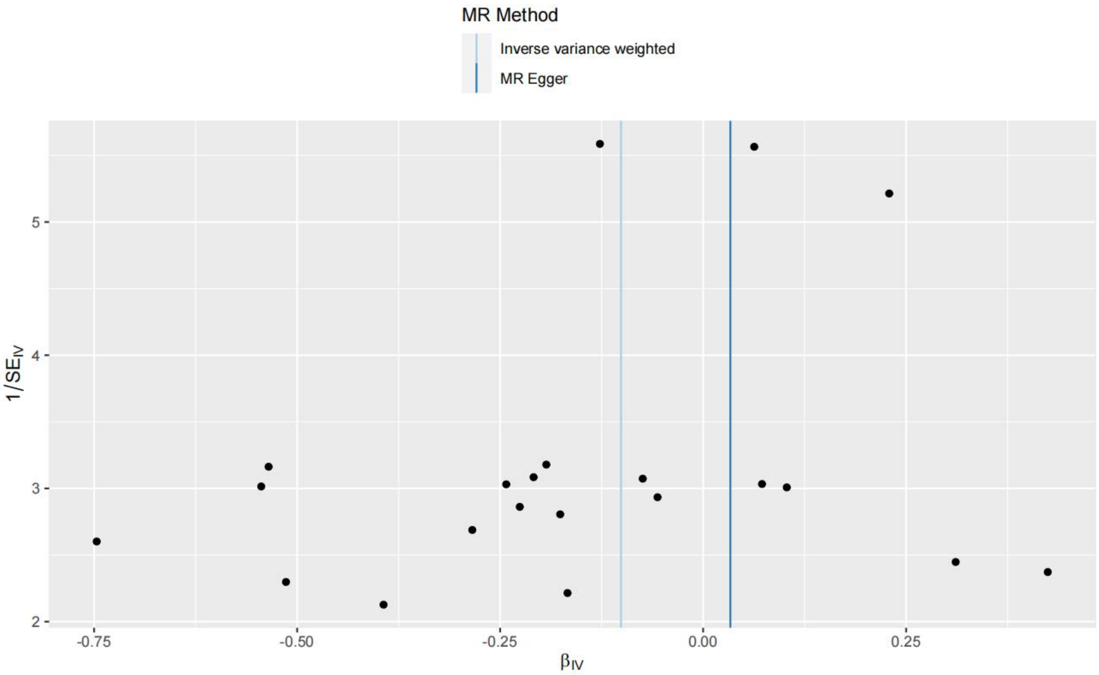

B. Funnel plot of ALS on MIF

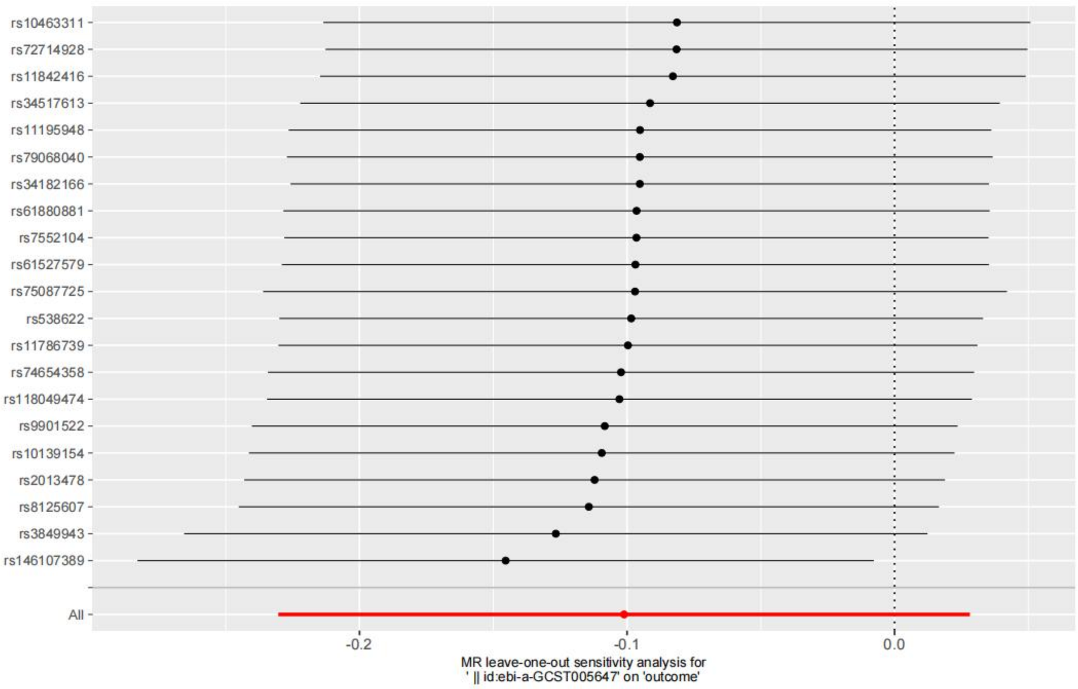

C. MR leave-one-out sensitivity analysis for ALS on MIF

eFigure 235. ALS-associated SNPs with MIG

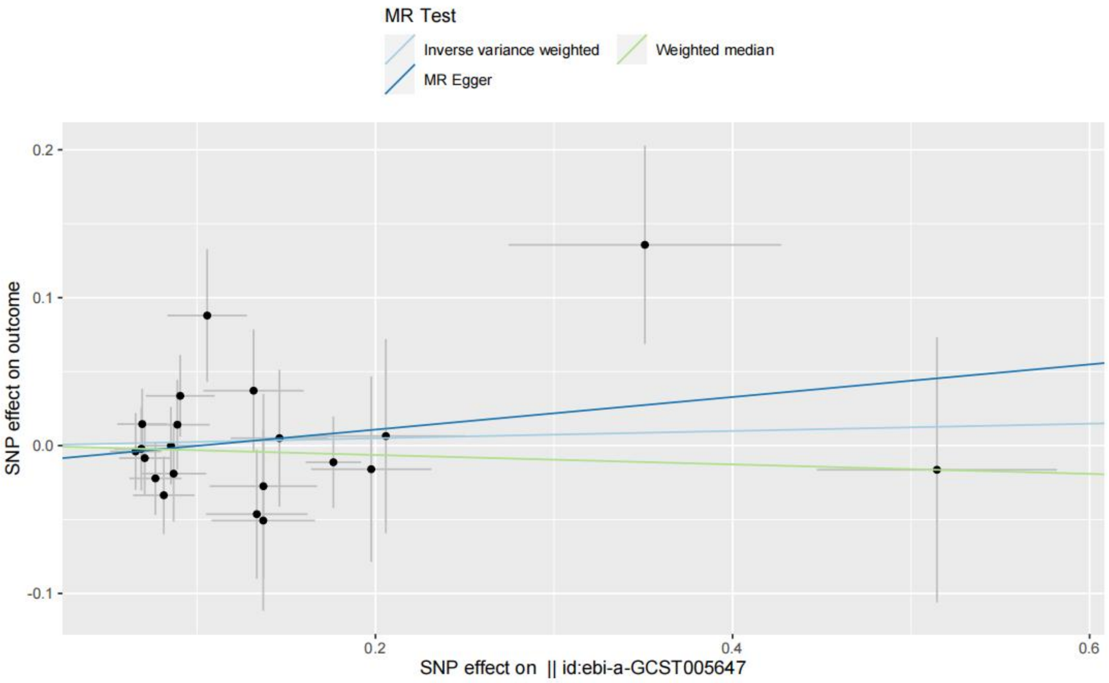

A. Scatter plot of ALS on MIG

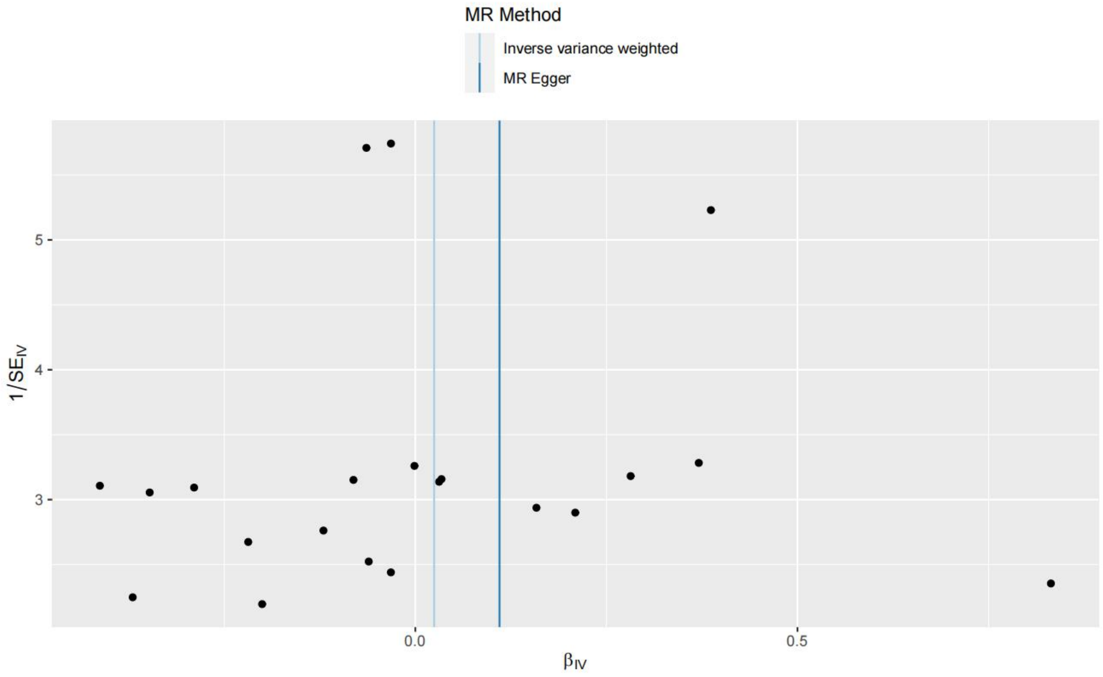

B. Funnel plot of ALS on MIG

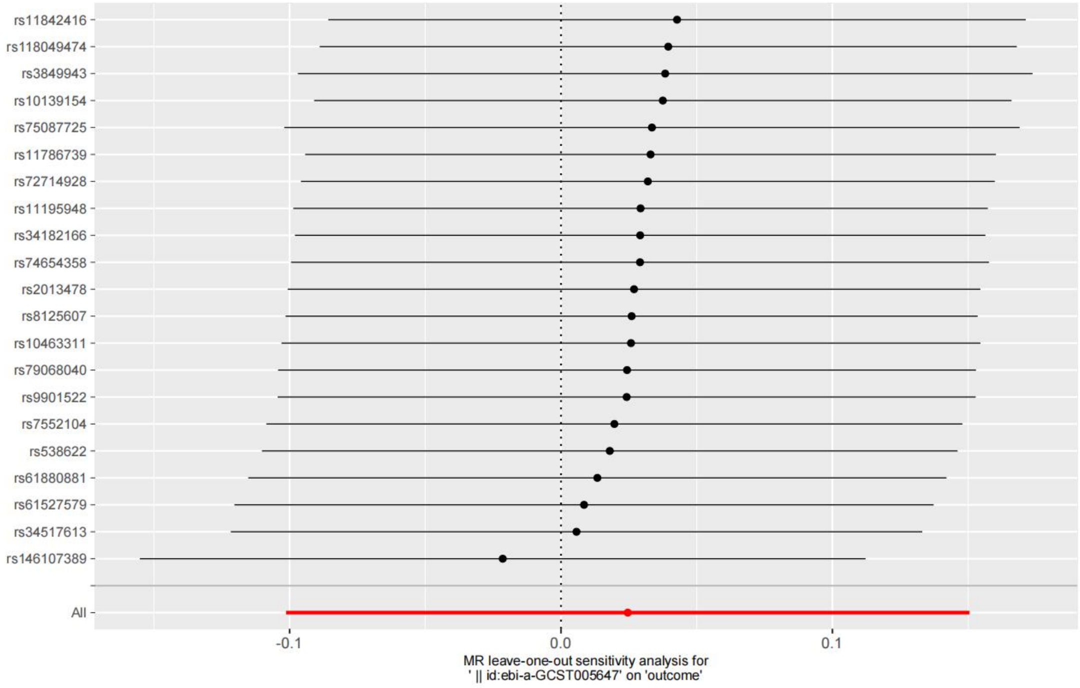

C. MR leave-one-out sensitivity analysis for ALS on MIG

eFigure 236. ALS-associated SNPs with MIP-1A

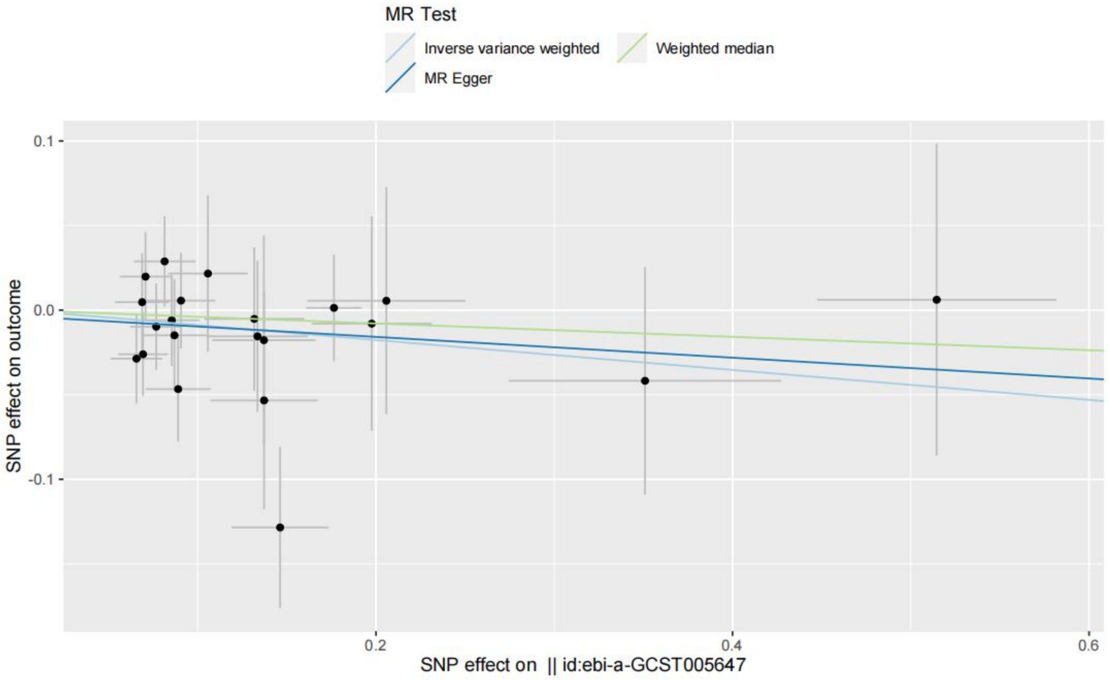

A. Scatter plot of ALS on MIP-1A

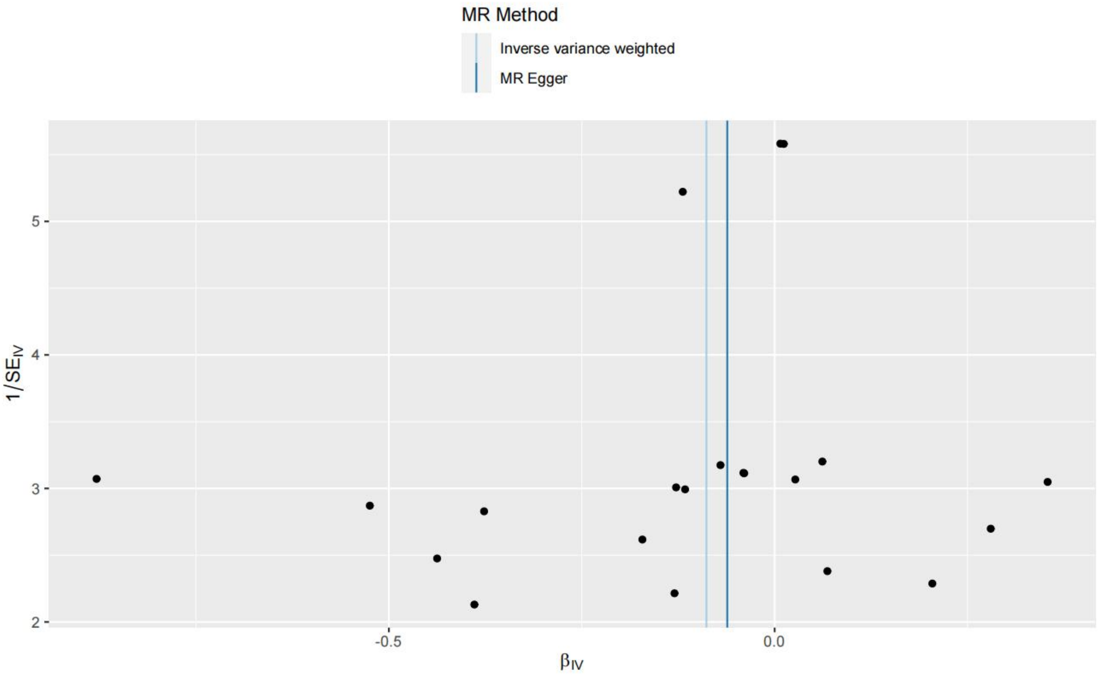

B. Funnel plot of ALS on MIP-1A

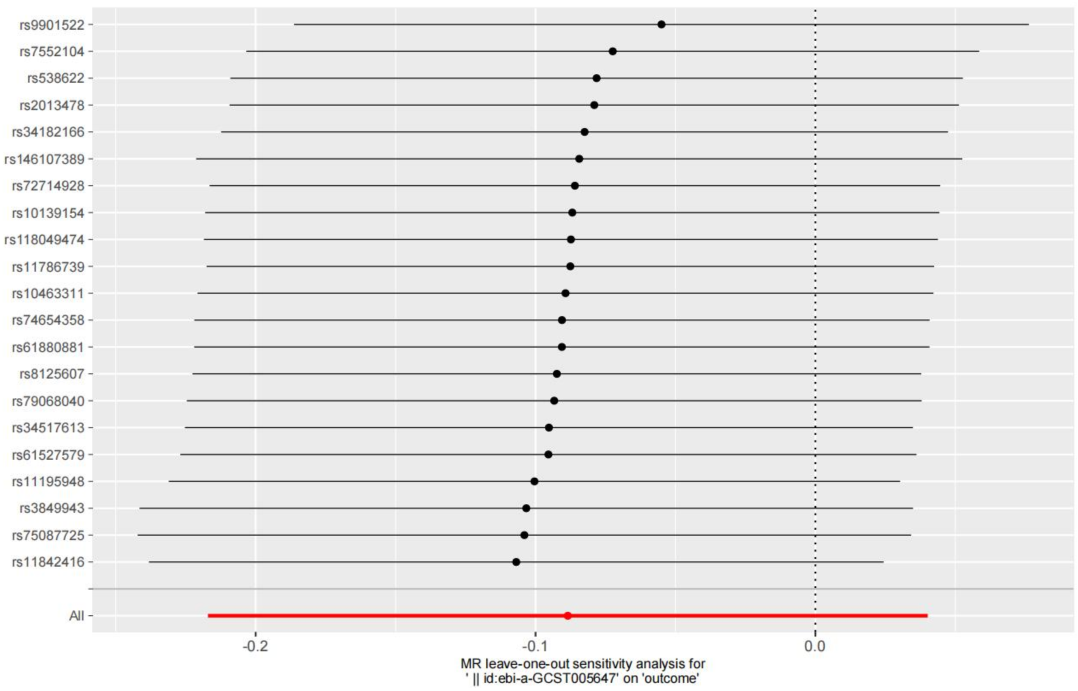

C. MR leave-one-out sensitivity analysis for ALS on MIP-1A

eFigure 237. ALS-associated SNPs with MIP-1B

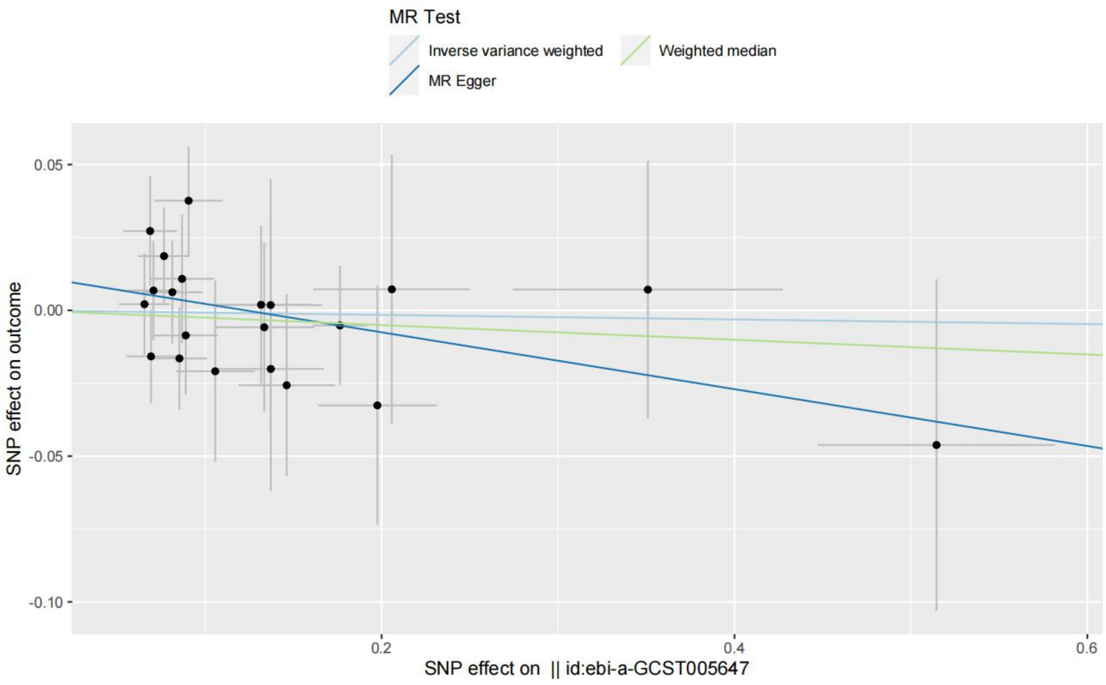

A. Scatter plot of ALS on MIP-1B

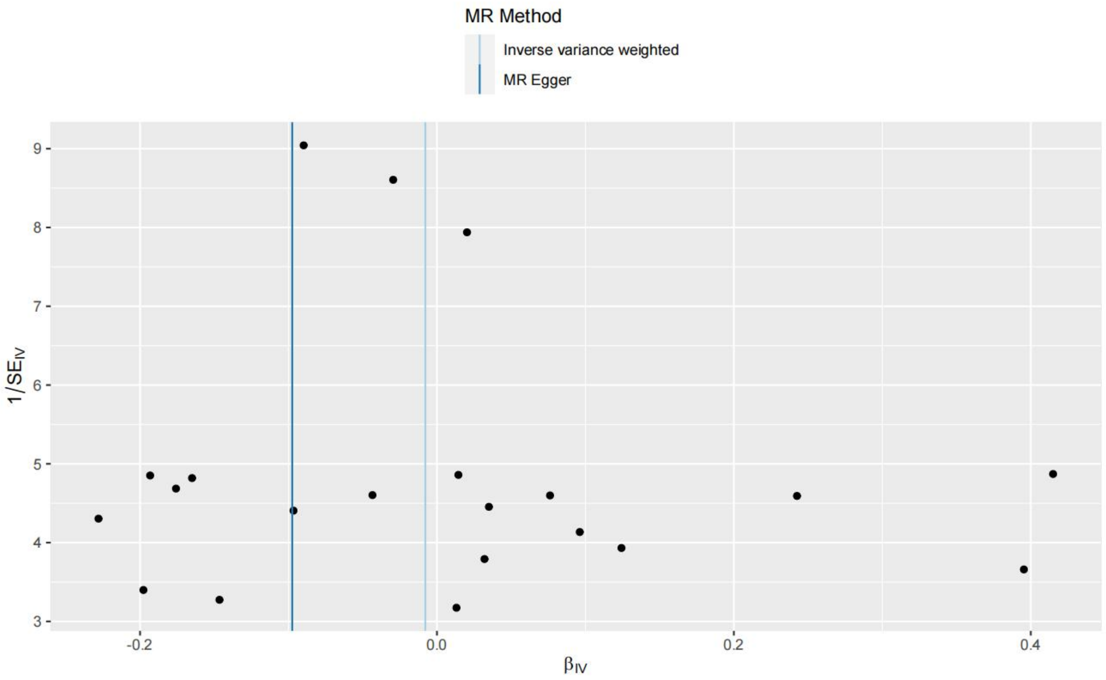

B. Funnel plot of ALS on MIP-1B

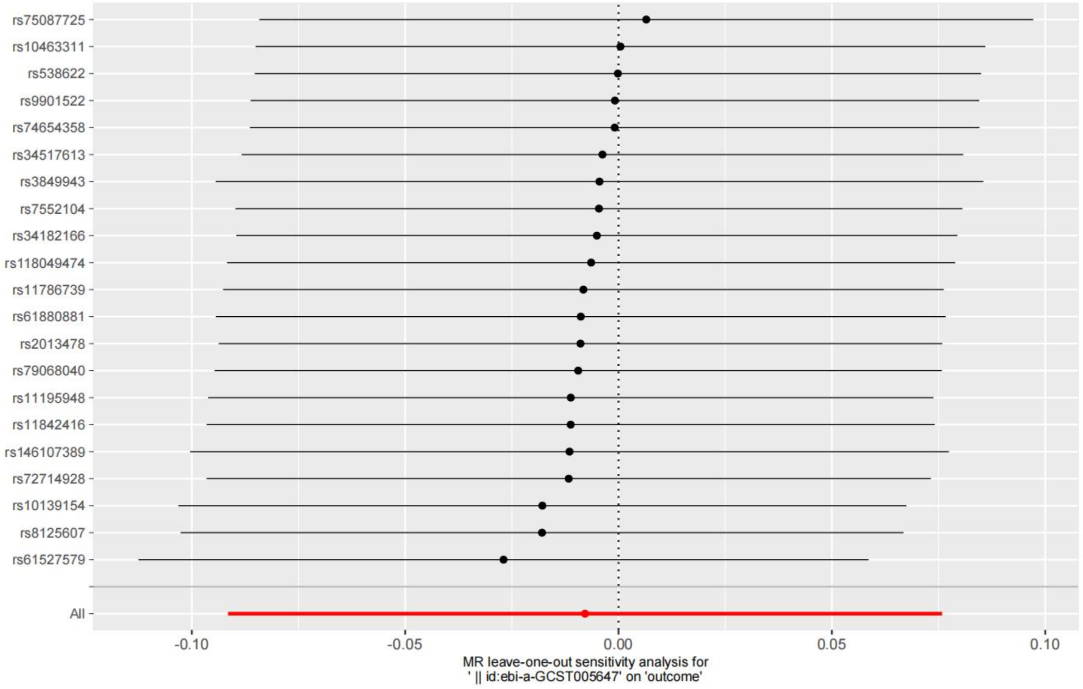

C. MR leave-one-out sensitivity analysis for ALS on MIP-1B

eFigure 238. ALS-associated SNPs with PDGF-BB

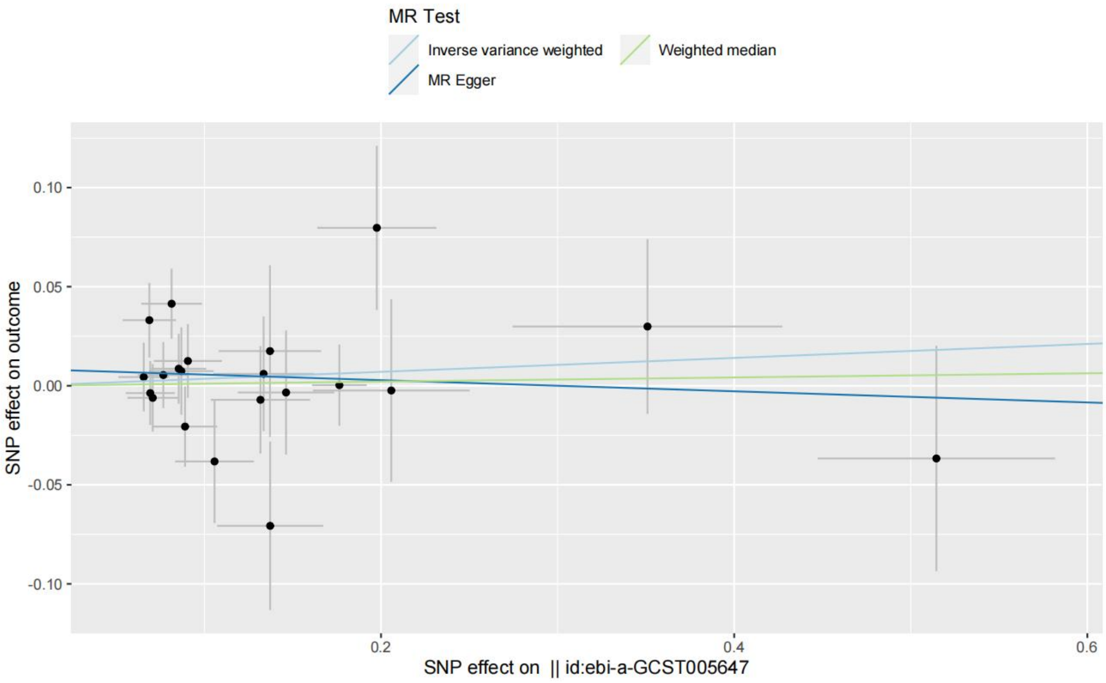

A. Scatter plot of ALS on PDGF-BB

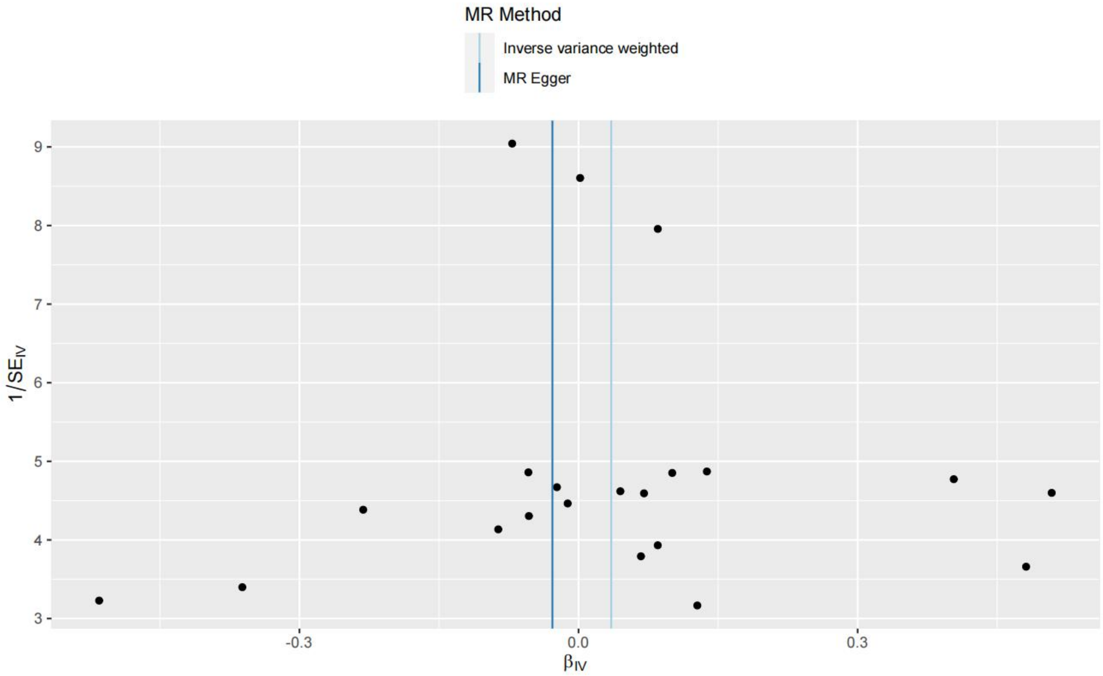

B. Funnel plot of ALS on PDGF-BB

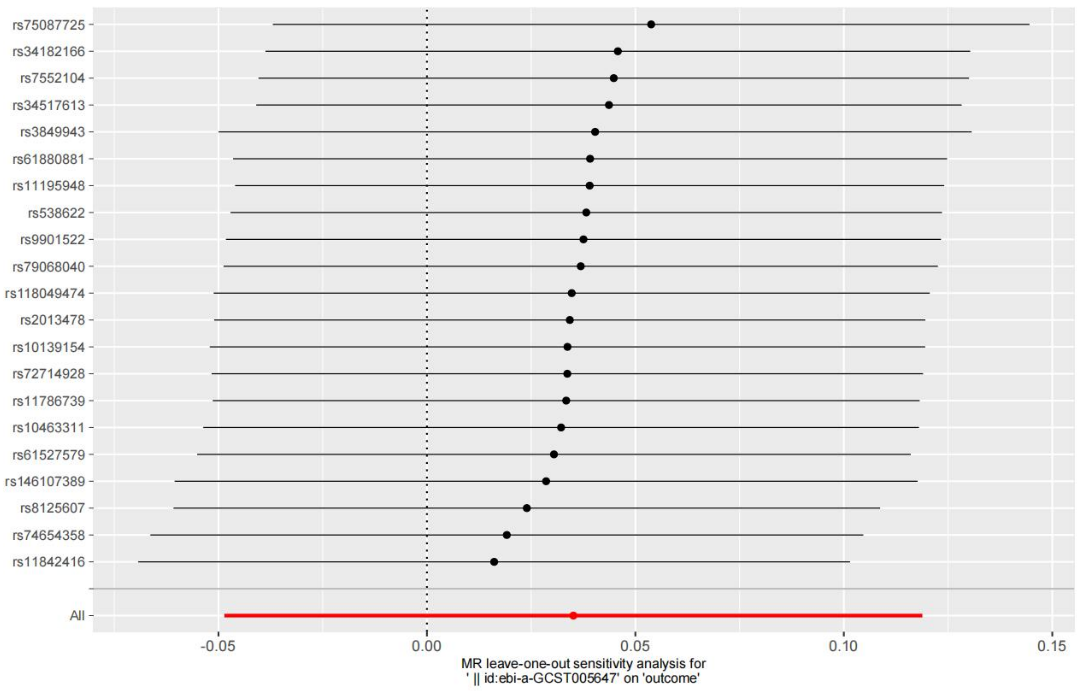

C. MR leave-one-out sensitivity analysis for ALS on PDGF-BB

eFigure 239. ALS-associated SNPs with RANTES

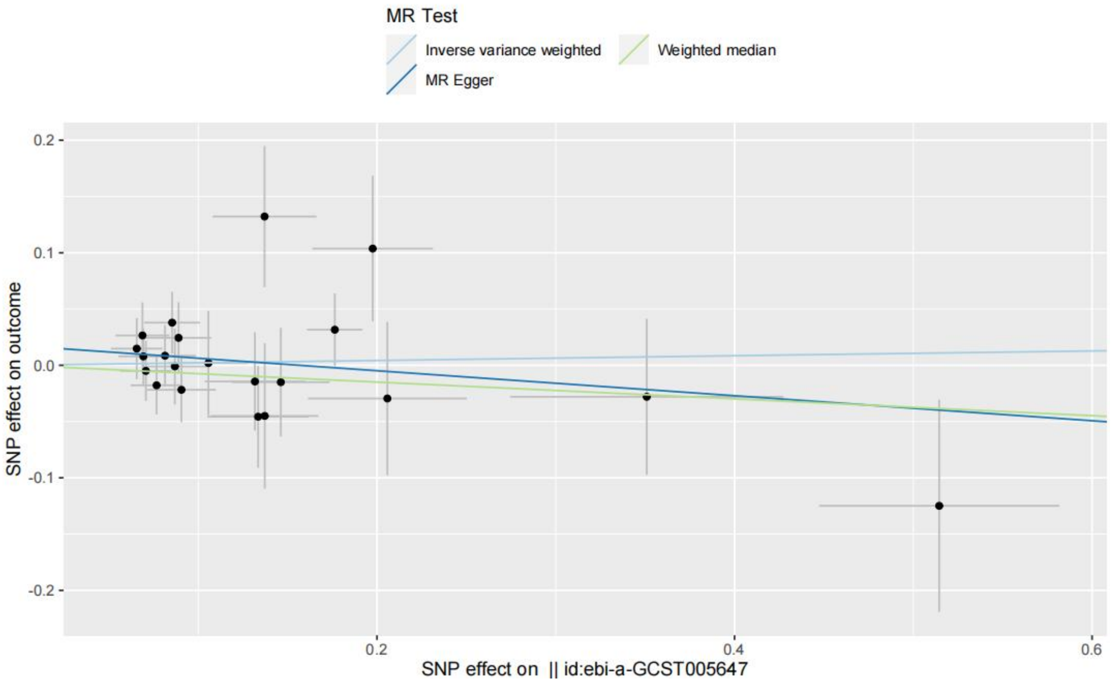

A. Scatter plot of ALS on RANTES

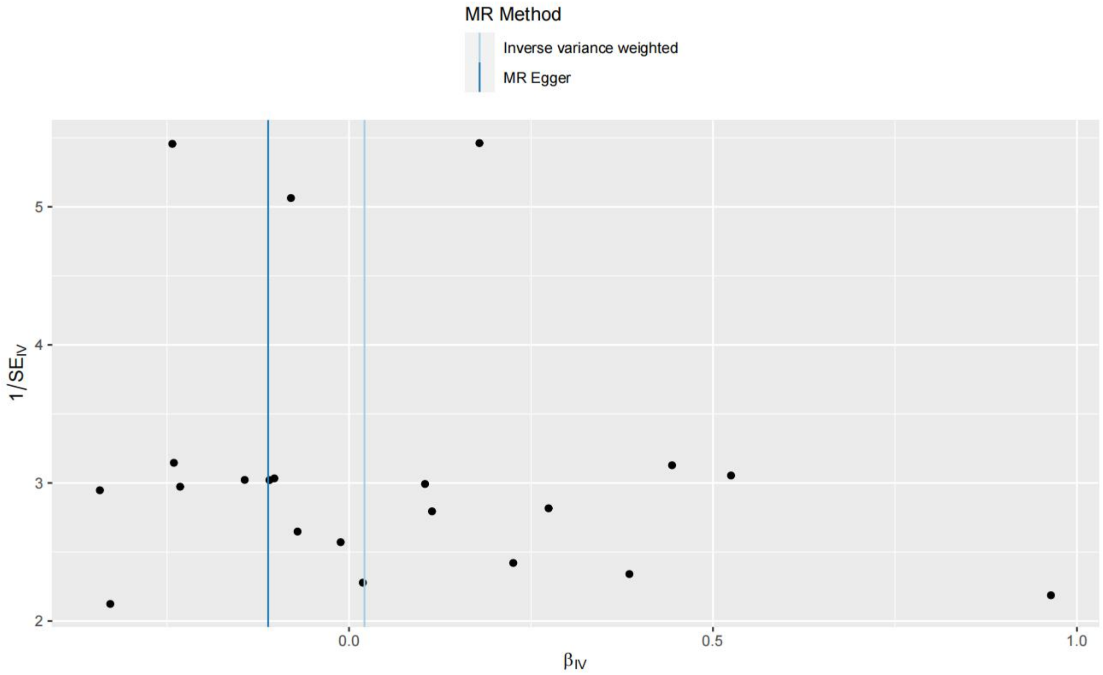

B. Funnel plot of ALS on RANTES

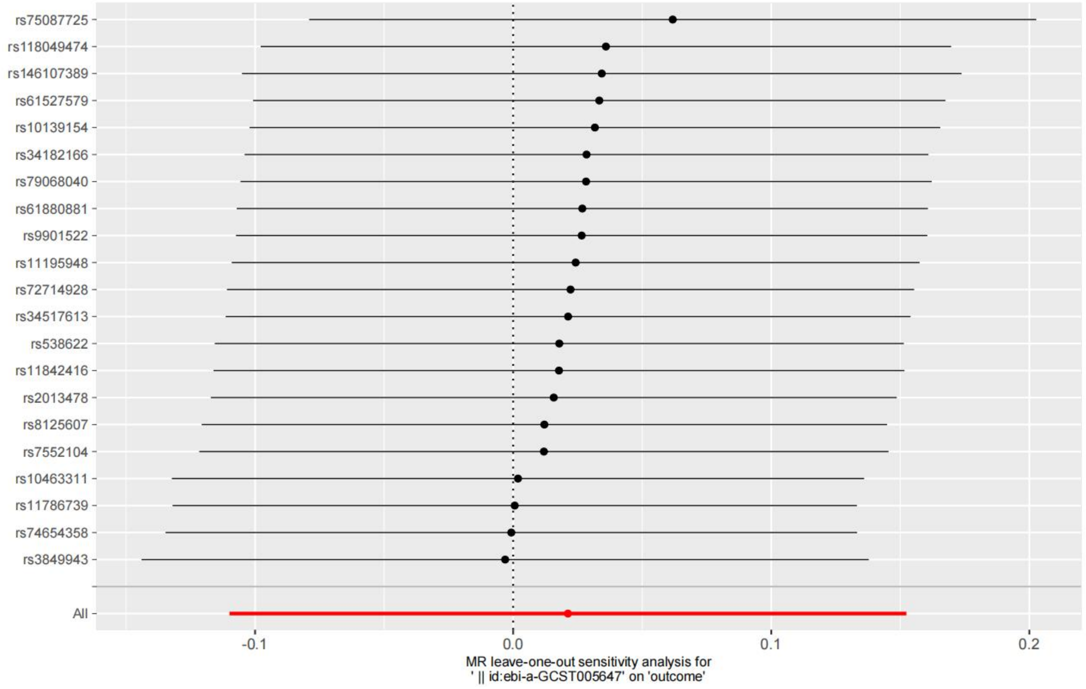

C. MR leave-one-out sensitivity analysis for ALS on RANTES

eFigure 240. ALS-associated SNPs with SCF

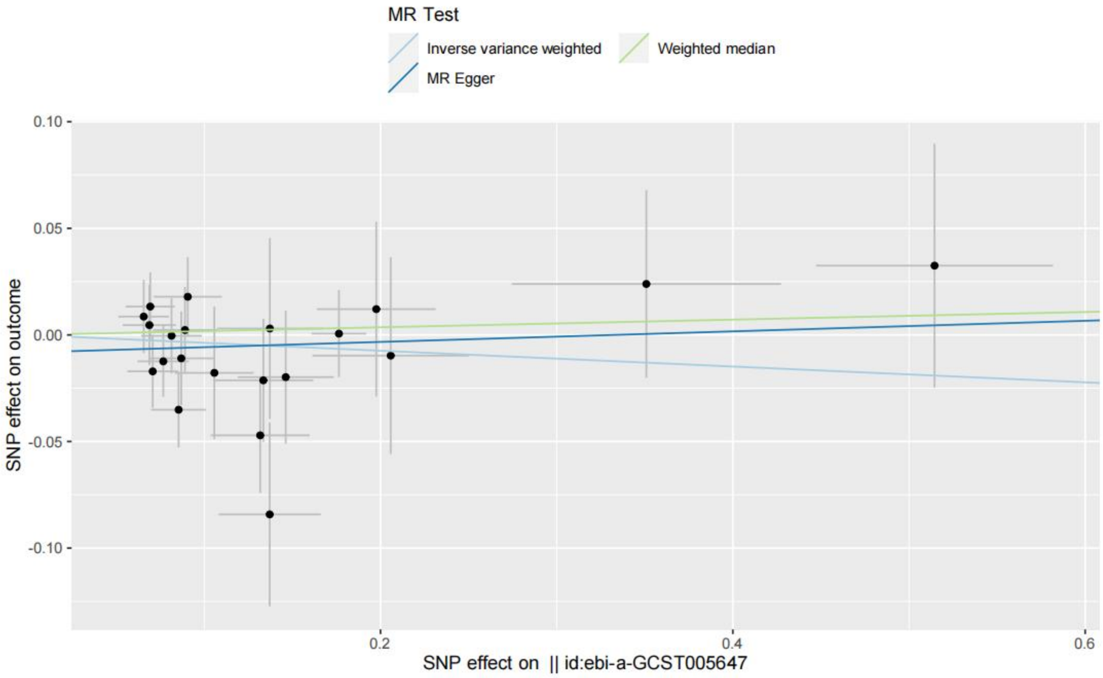

A. Scatter plot of ALS on SCF

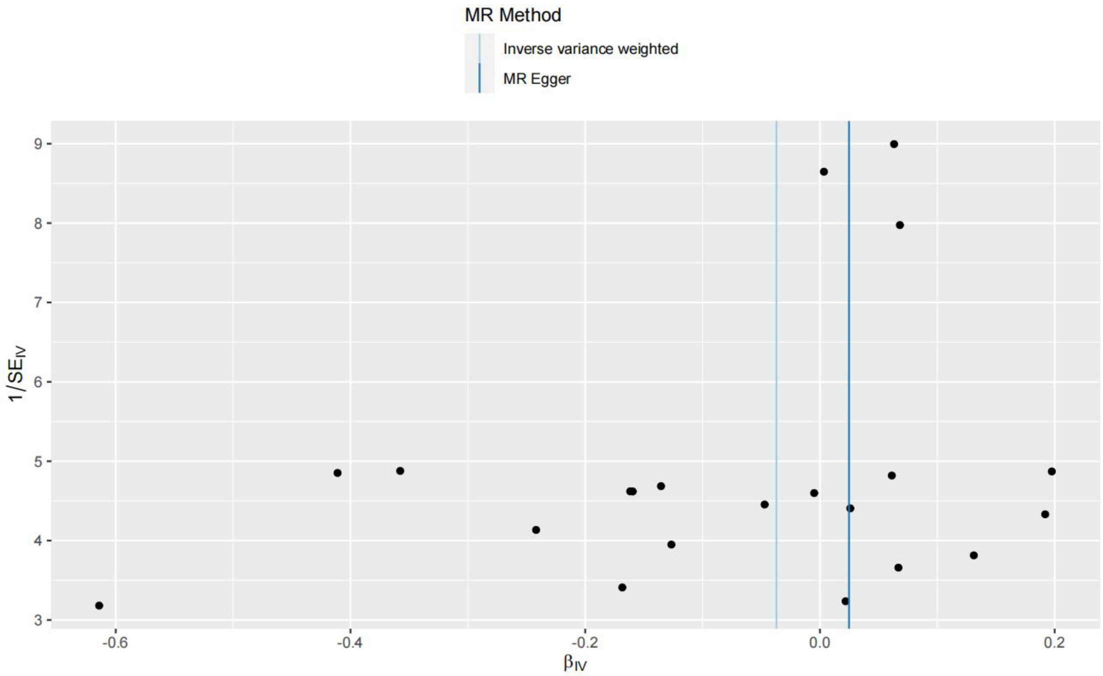

B. Funnel plot of ALS on SCF

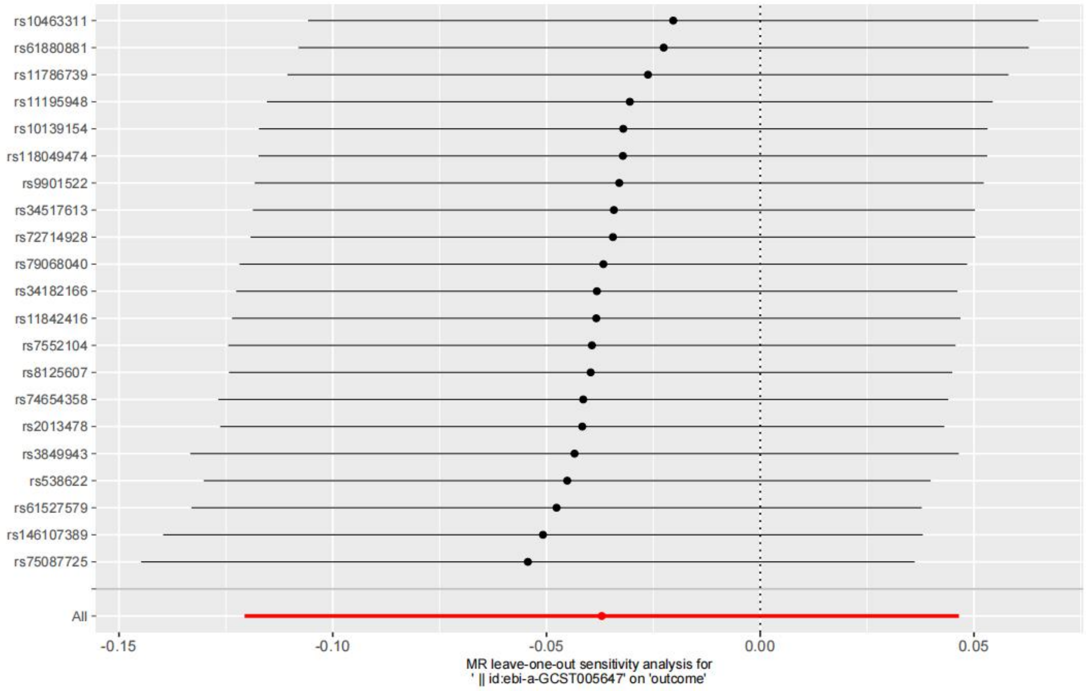

C. MR leave-one-out sensitivity analysis for ALS on SCF

eFigure 241. ALS-associated SNPs with SGCF

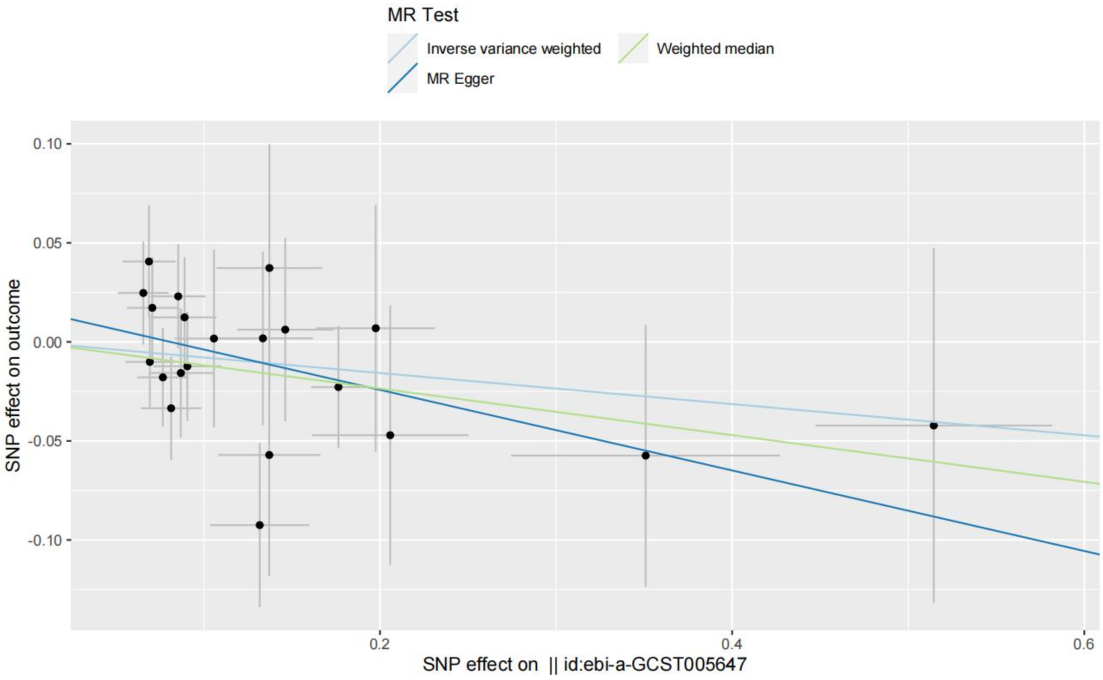

A. Scatter plot of ALS on SGCF

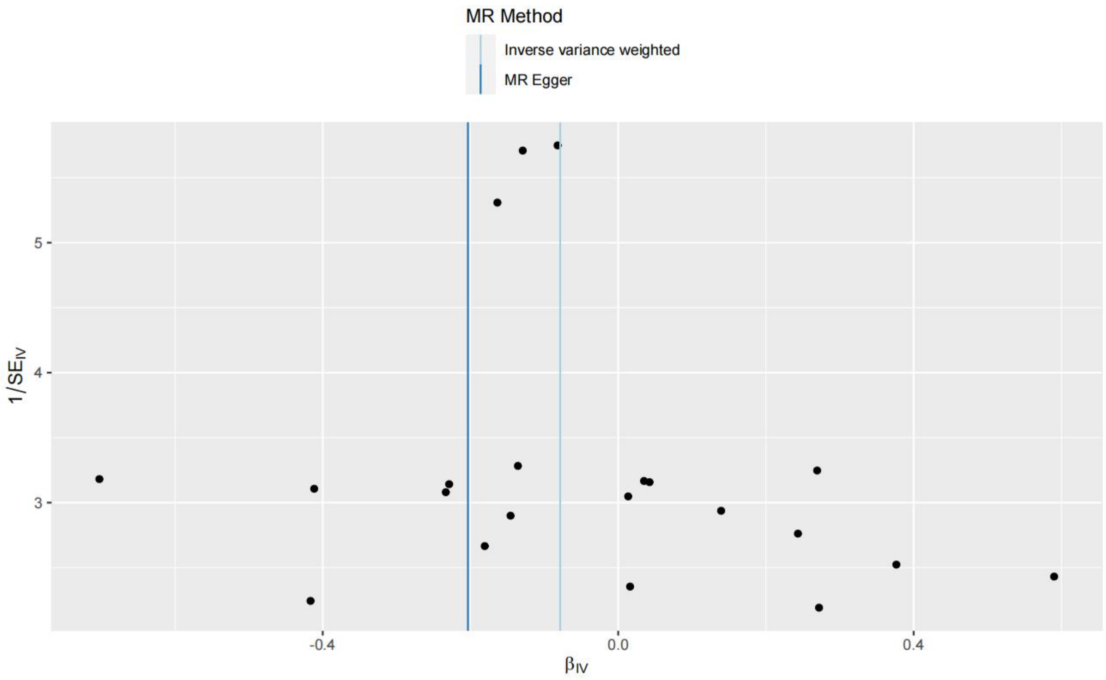

B. Funnel plot of ALS on SGCF

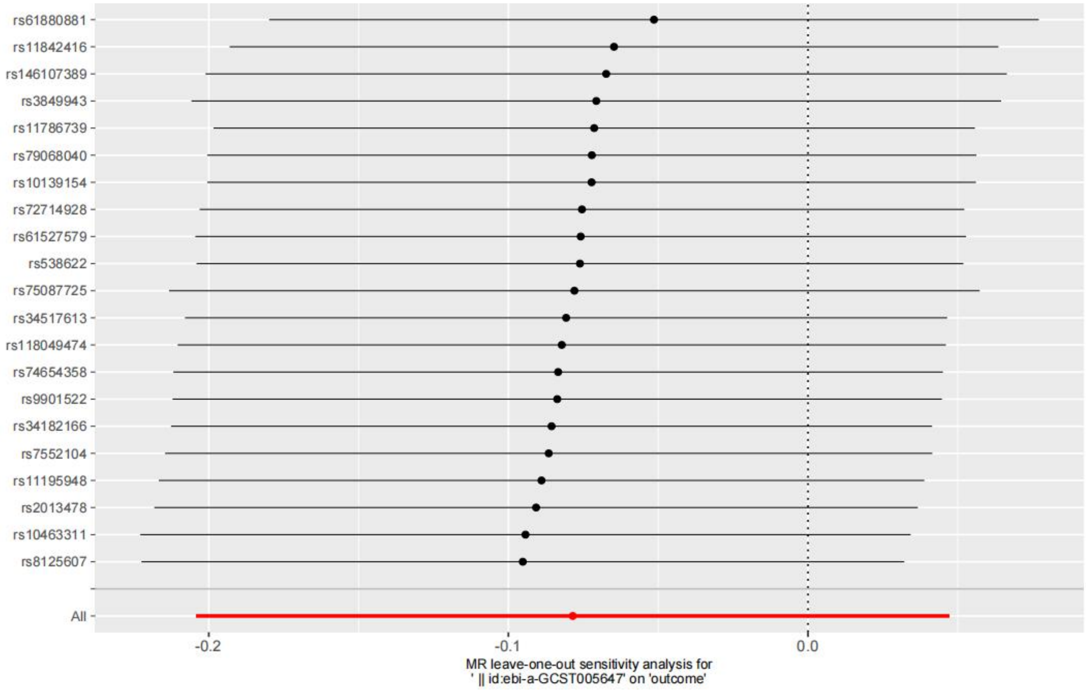

C. MR leave-one-out sensitivity analysis for ALS on SGCF

eFigure 242. ALS-associated SNPs with SDF-1A

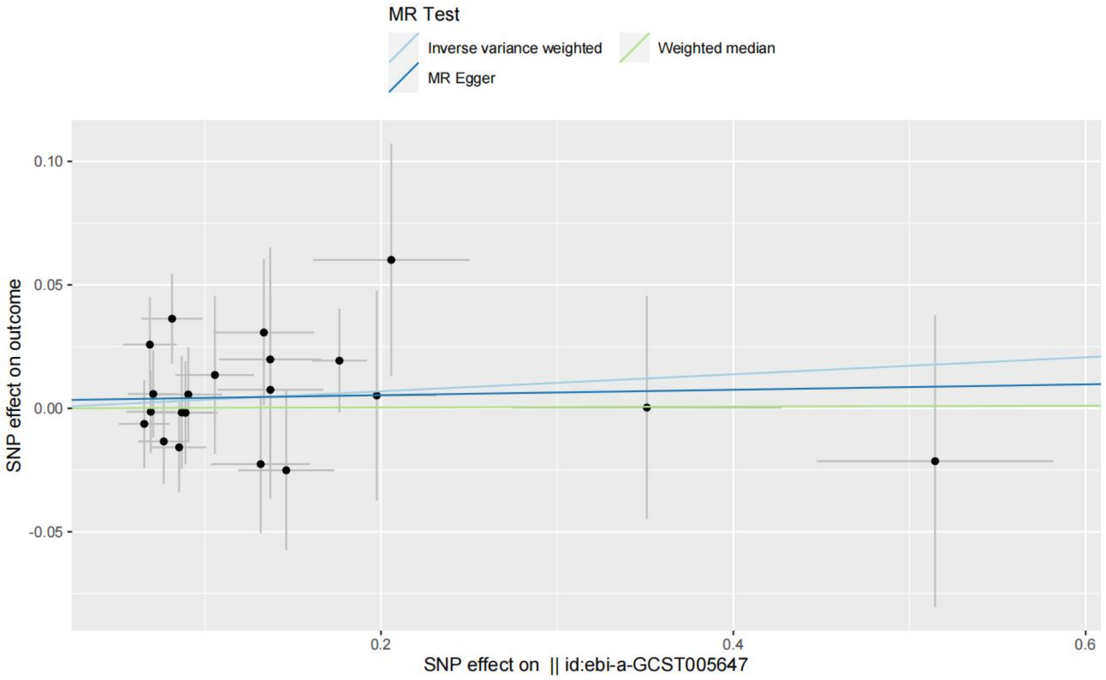

A. Scatter plot of ALS on SDF-1A

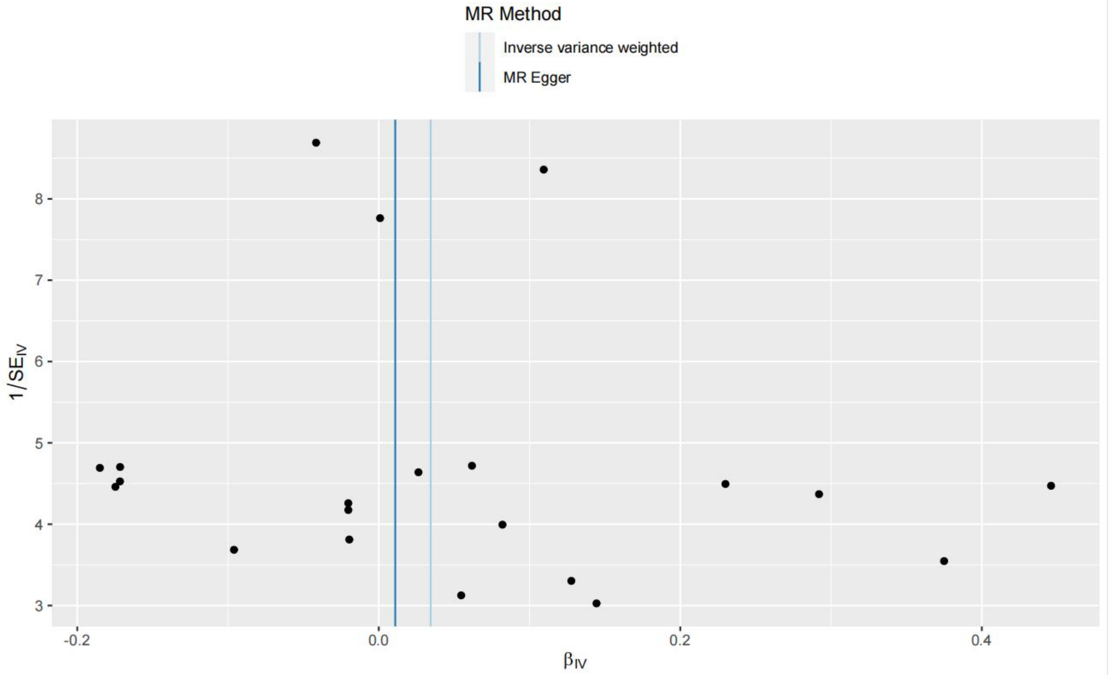

B. Funnel plot of ALS on SDF-1A

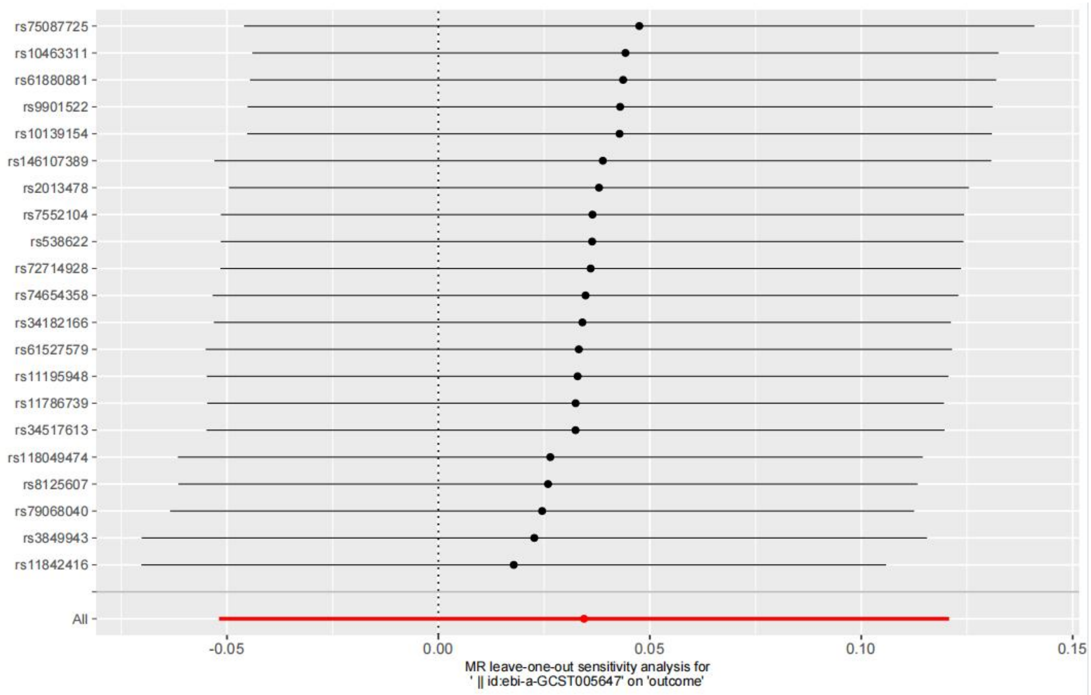

C. MR leave-one-out sensitivity analysis for ALS on SDF-1A

eFigure 243. ALS-associated SNPs with TNF-A

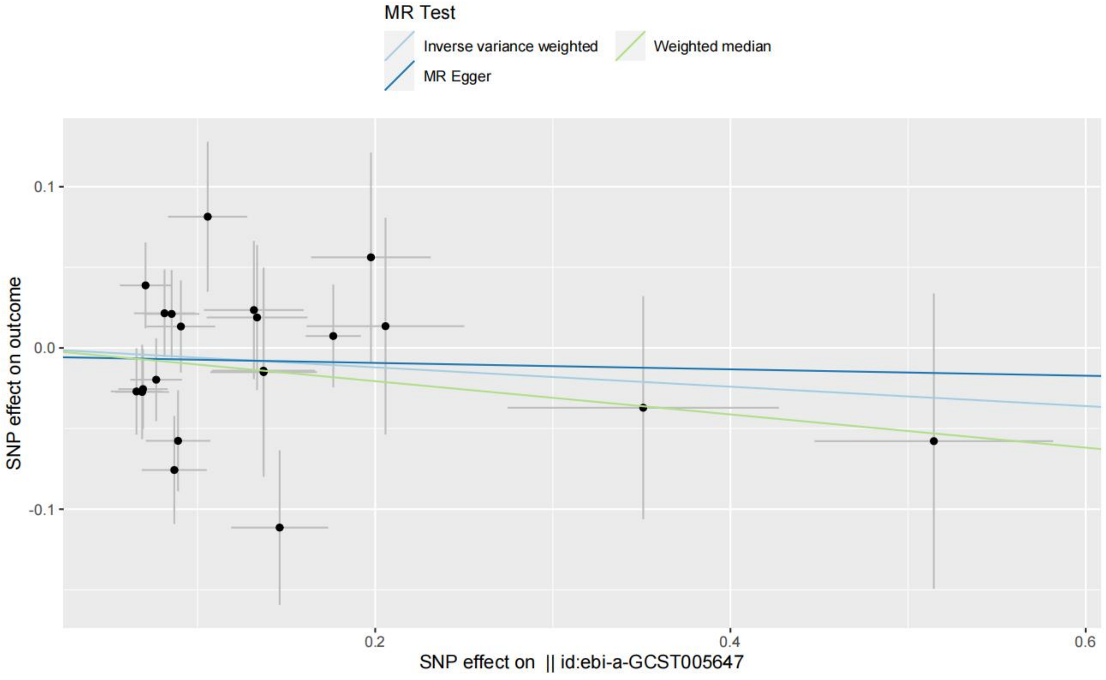

A. Scatter plot of ALS on TNF-A

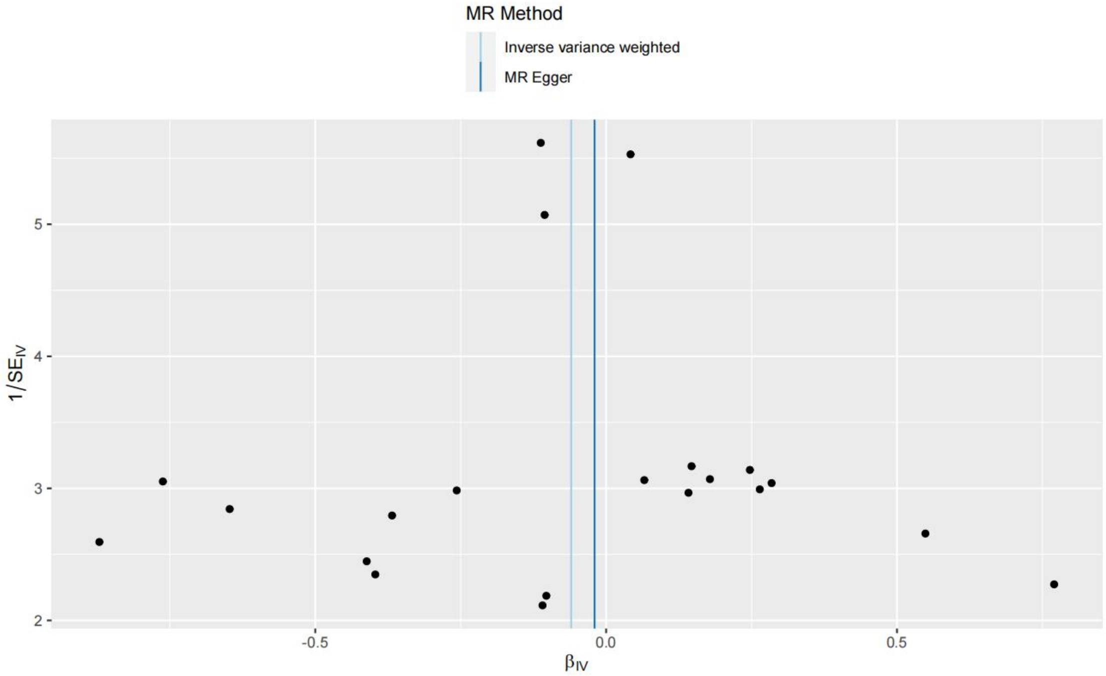

B. Funnel plot of ALS on TNF-A

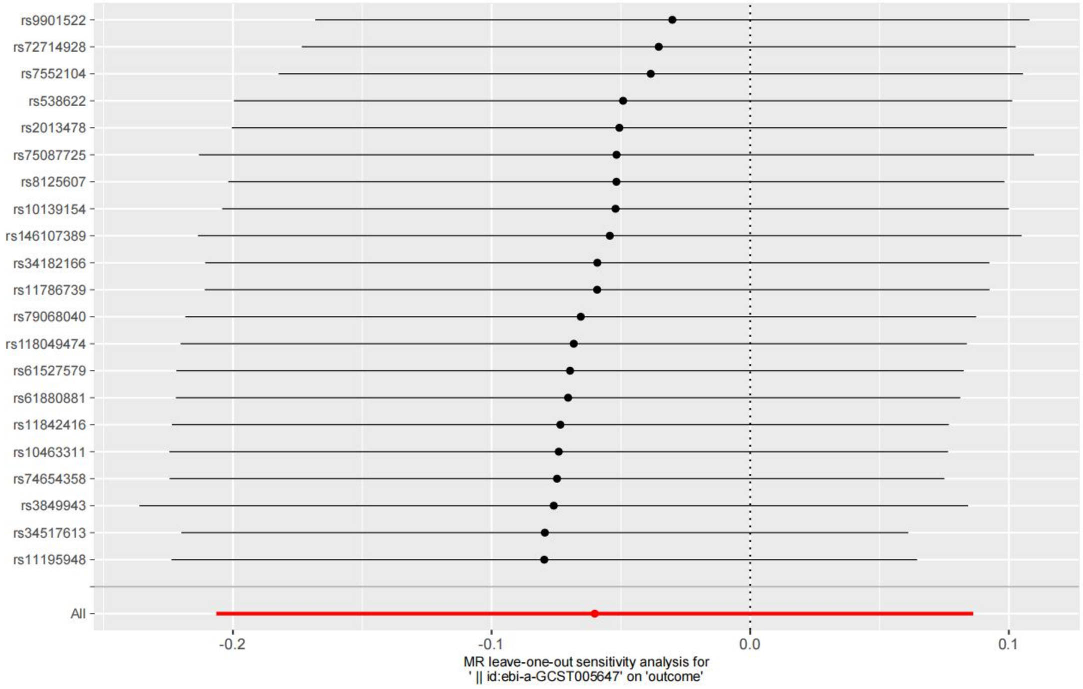

C. MR leave-one-out sensitivity analysis for ALS on TNF-A

eFigure 244. ALS-associated SNPs with TNF-B

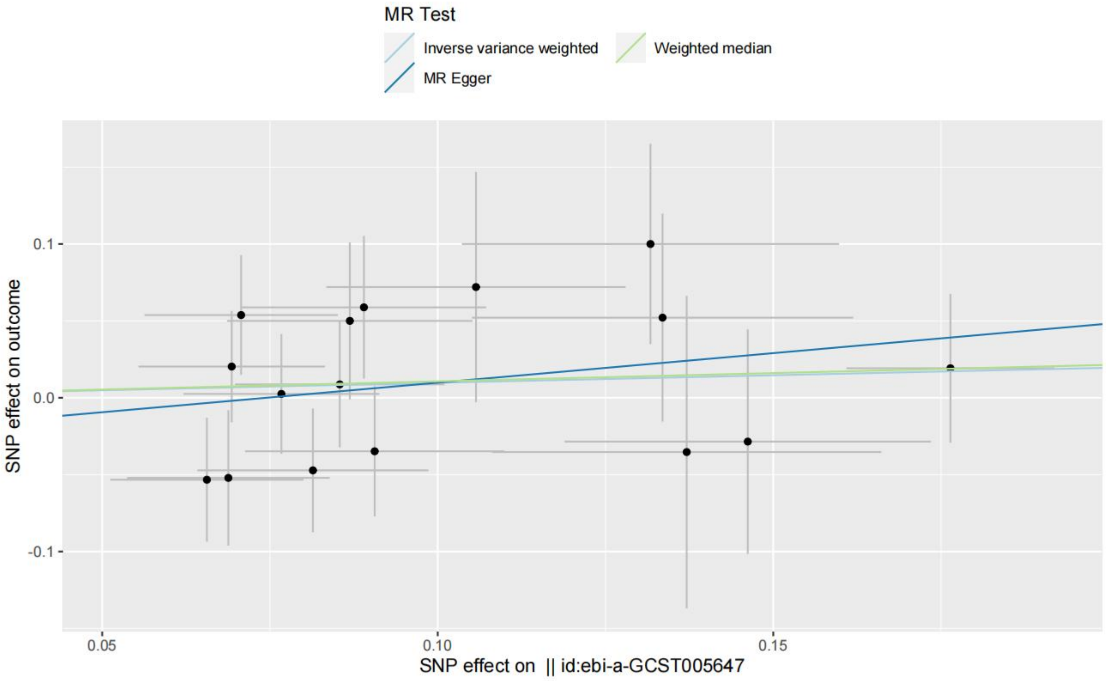

A. Scatter plot of ALS on TNF-B

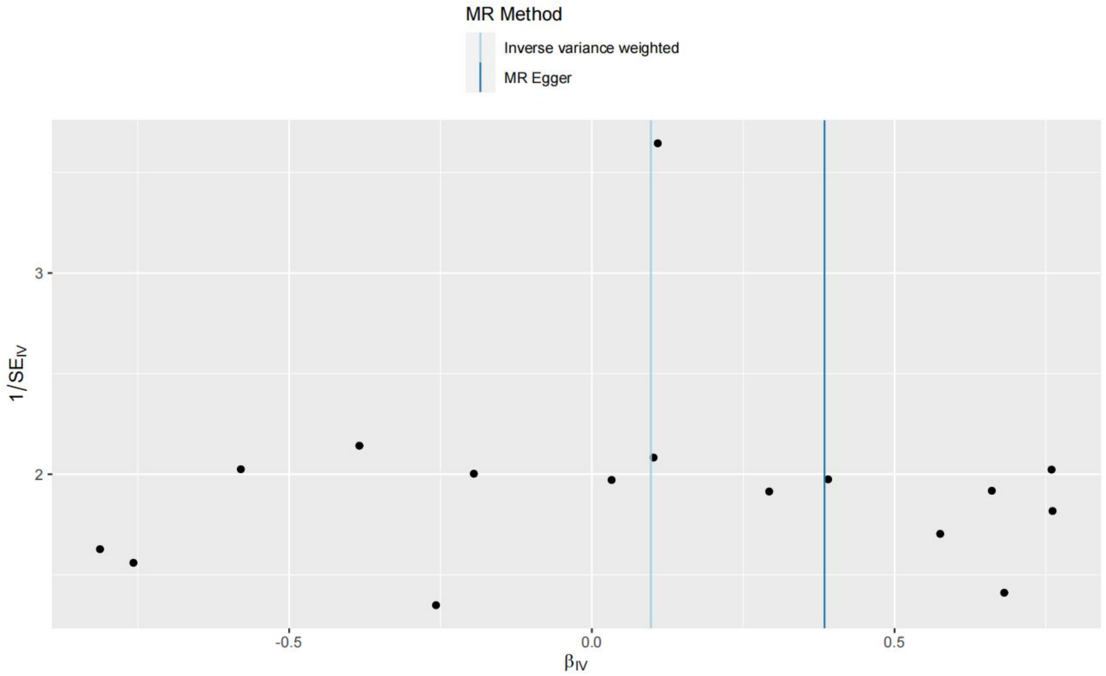

B. Funnel plot of ALS on TNF-B

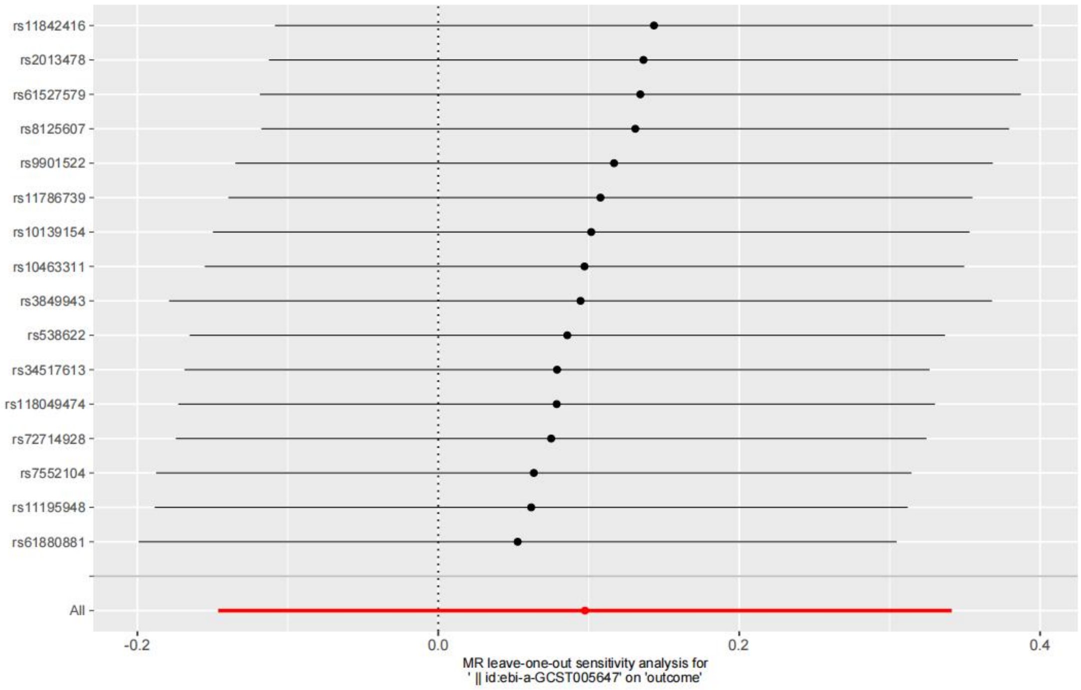

C. MR leave-one-out sensitivity analysis for ALS on TNF-B

eFigure 245. ALS-associated SNPs with TRAIL

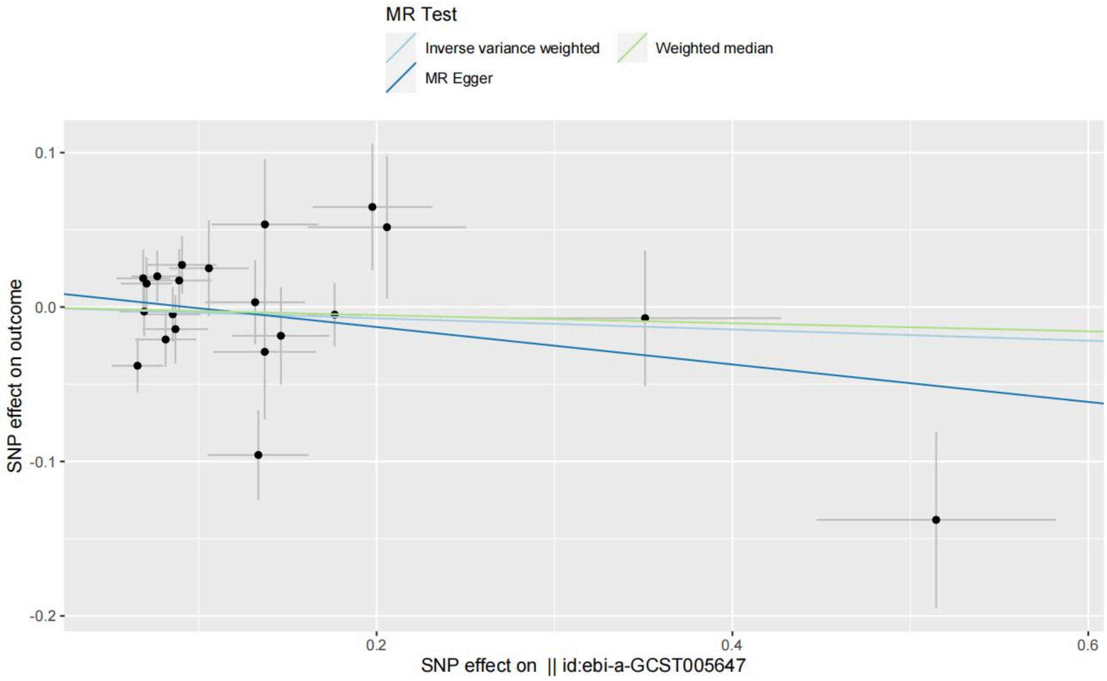

A. Scatter plot of ALS on TRAIL

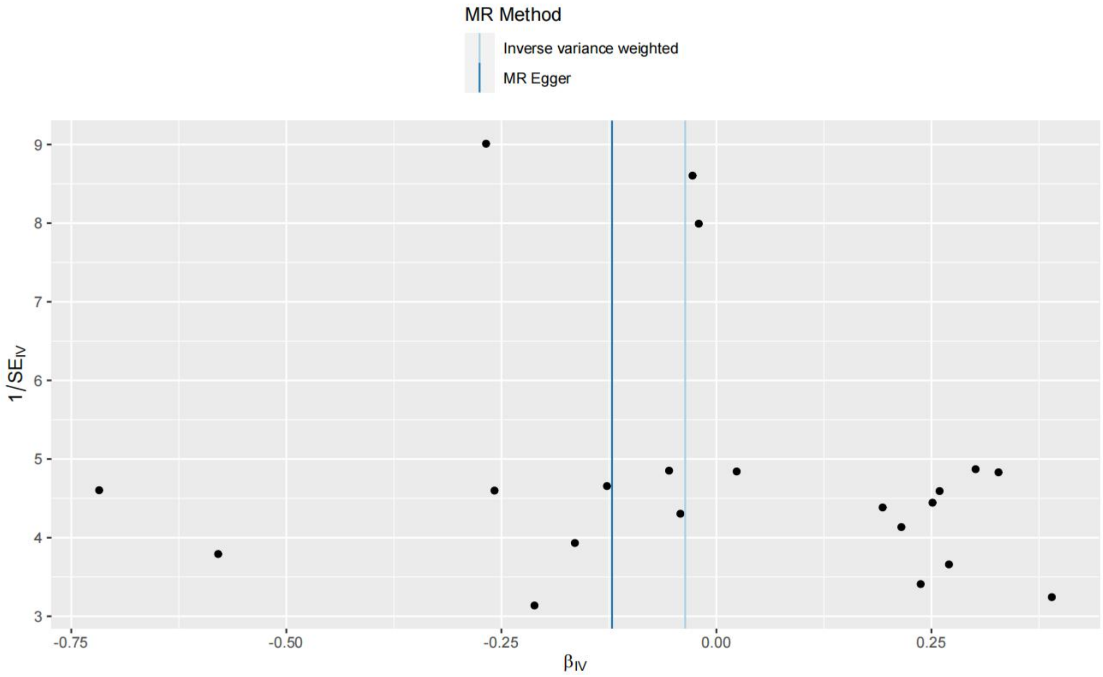

B. Funnel plot of ALS on TRAIL

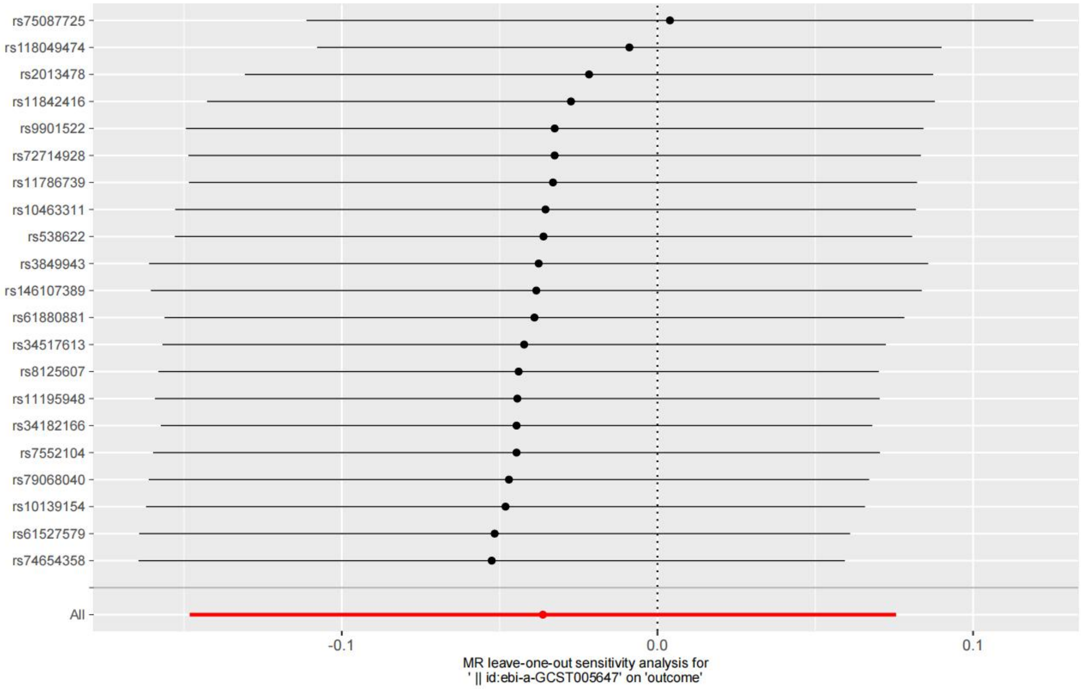

C. MR leave-one-out sensitivity analysis for ALS on TRAIL

eFigure 246. ALS-associated SNPs with VEGF

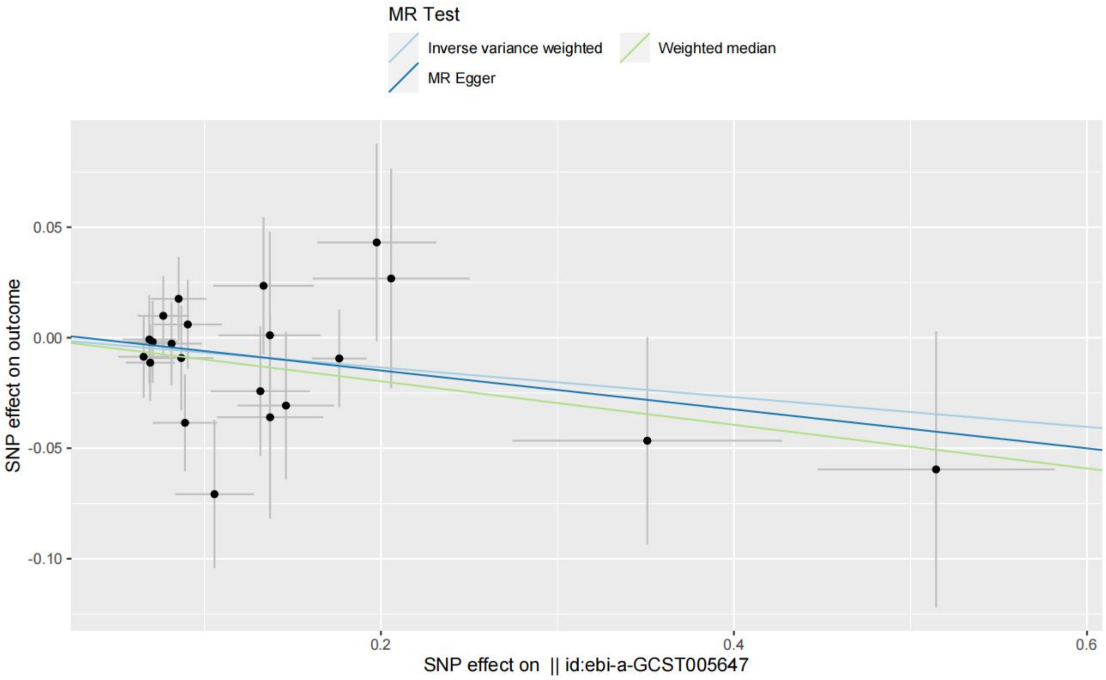

A. Scatter plot of ALS on VEGF

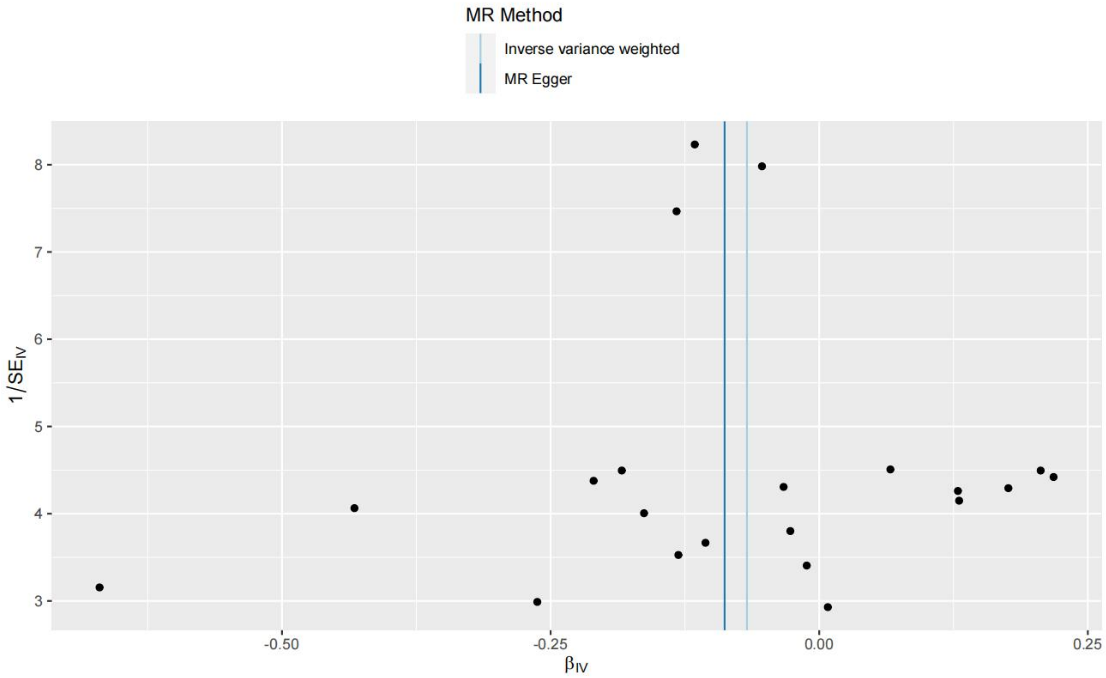

B. Funnel plot of ALS on VEGF

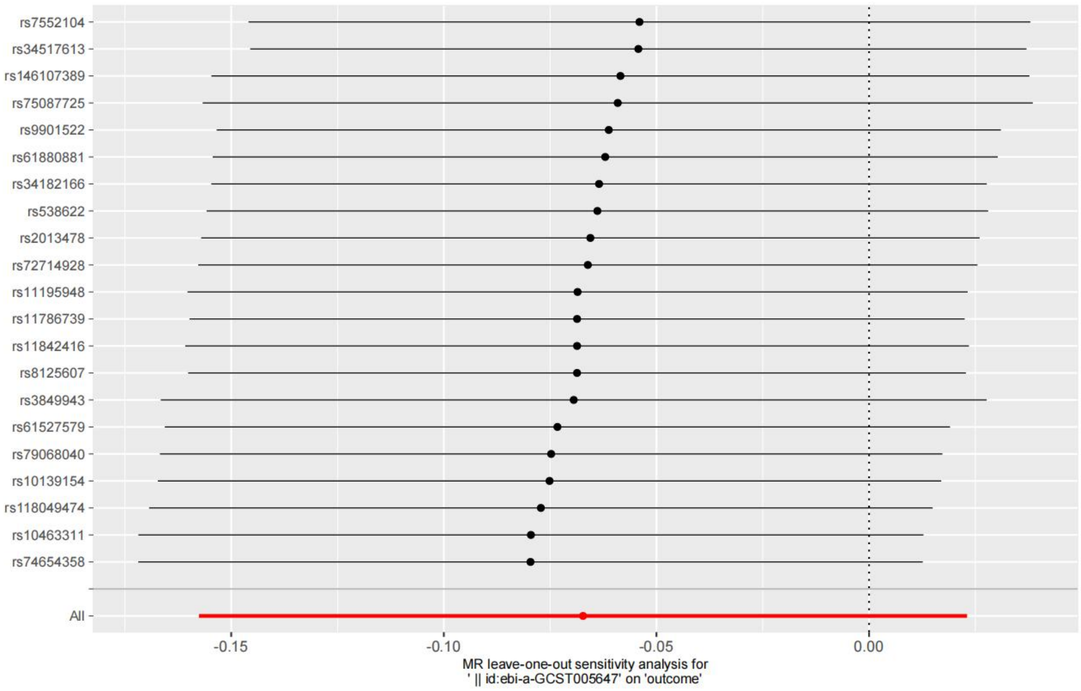

C. MR leave-one-out sensitivity analysis for ALS on VEGF
